# Identification by array comparative genomic hybridization of a new amplicon on chromosome 17q highly recurrent in *BRCA1* mutated triple negative breast cancer

**DOI:** 10.1186/s13058-014-0466-y

**Published:** 2014-11-22

**Authors:** Sébastien Toffoli, Isabelle Bar, Fadi Abdel-Sater, Paul Delrée, Pascale Hilbert, Frédéric Cavallin, Fabrice Moreau, Wim Van Criekinge, Magali Lacroix-Triki, Mario Campone, Anne-Laure Martin, Henri Roché, Jean-Pascal Machiels, Javier Carrasco, Jean-Luc Canon

**Affiliations:** 1 Laboratory of Translational Oncology, Institute of Pathology and Genetics/ Grand Hôpital de Charleroi, Avenue Georges Lemaître 25, Gosselies, 6041 Belgium; 2 Tumor Bank, Institute of Pathology and Genetics, Avenue Georges Lemaître 25, Gosselies, 6041 Belgium; 3 Department of Pathology, Institute of Pathology and Genetics, Avenue Georges Lemaître 25, Gosselies, 6041 Belgium; 4 Department of Molecular Biology, Institute of Pathology and Genetics, Avenue Georges Lemaître 25, Gosselies, 6041 Belgium; 5 MdxHealth Inc, 15279 Alton Parkway, Suite 100, Irvine, 92618 CA USA; 6 Département de Biologie et de Pathologie, Institut Claudius Regaud, 20-24, Rue Pont St Pierre, Toulouse, 31052 France; 7 Département d’Oncologie Médicale, Institut de Cancérologie de l’Ouest-René Gauducheau, Boulevard Jacques Monod, Saint-Herblain, Nantes, 44805 France; 8 R&D UNICANCER, UNICANCER, Rue de Tolbiac 101, Paris Cedex 13, 75654 France; 9 Département d’Oncologie Médicale, Institut Claudius Regaud, 20-24, Rue Pont St Pierre, Toulouse, 31300 France; 10 Department of Oncology, Cliniques Universitaires Saint-Luc, Avenue Hippocrate 10, Brussels, 1200 Belgium; 11 Service of Oncology-Hematology, Grand Hôpital de Charleroi, Grand’Rue, 3, Charleroi, 6000 Belgium

## Abstract

**Introduction:**

Triple Negative Breast Cancers (TNBC) represent about 12% to 20% of all breast cancers (BC) and have a worse outcome compared to other BC subtypes. TNBC often show a deficiency in DNA double-strand break repair mechanisms. This is generally related to the inactivation of a repair enzymatic complex involving BRCA1 caused either by genetic mutations, epigenetic modifications or by post-transcriptional regulations.

The identification of new molecular biomarkers that would allow the rapid identification of BC presenting a BRCA1 deficiency could be useful to select patients who could benefit from PARP inhibitors, alkylating agents or platinum-based chemotherapy.

**Methods:**

Genomic DNA from 131 formalin-fixed paraffin-embedded (FFPE) tumors (luminal A and B, HER2+ and triple negative BC) with known *BRCA1* mutation status or unscreened for *BRCA1* mutation were analysed by array Comparative Genomic Hybridization (array CGH). One highly significant and recurrent gain in the 17q25.3 genomic region was analysed by fluorescent in situ hybridization (FISH). Expression of the genes of the 17q25.3 amplicon was studied using customized Taqman low density arrays and single Taqman assays (Applied Biosystems).

**Results:**

We identified by array CGH and confirmed by FISH a gain in the 17q25.3 genomic region in 90% of the *BRCA1* mutated tumors. This chromosomal gain was present in only 28.6% of the *BRCA1* non-mutated TNBC, 26.7% of the unscreened TNBC, 13.6% of the luminal B, 19.0% of the HER2+ and 0% of the luminal A breast cancers. The 17q25.3 gain was also detected in 50% of the TNBC with *BRCA1* promoter methylation. Interestingly, *BRCA1* promoter methylation was never detected in *BRCA1* mutated BC. Gene expression analyses of the 17q25.3 sub-region showed a significant over-expression of 17 genes in *BRCA1* mutated TNBC (*n* = 15) as compared to the *BRCA1* non mutated TNBC (*n* = 13).

**Conclusions:**

In this study, we have identified by array CGH and confirmed by FISH a recurrent gain in 17q25.3 significantly associated to *BRCA1* mutated TNBC. Up-regulated genes in the 17q25.3 amplicon might represent potential therapeutic targets and warrant further investigation.

**Electronic supplementary material:**

The online version of this article (doi:10.1186/s13058-014-0466-y) contains supplementary material, which is available to authorized users.

## Introduction

Breast cancer (BC) is the most frequent female cancer, and is a complex and heterogeneous disease. Molecular analyses based on cDNA microarrays have revealed distinctive subtypes of BC, each characterized by a specific gene expression profile [1]-[3]. These subtypes include luminal A and B (positive for estrogen receptor (ER) and/or progesterone receptor (PR)), human epidermal growth factor 2-positive (HER2+) (high expression of the HER2 oncogene) and basal-like breast cancer (BLBC, expressing genes specifically of the basal cells of the normal breast) [4],[5]. The majority of BLBC are triple-negative (TN). TN breast cancer (TNBC) (that is, ER-negative, PR-negative, HER2-negative BC) accounts for about 12 to 20% of all BC [6]. BC subtypes are associated with different clinical outcomes, with the best prognosis for luminal A cancers and the worst for TN tumors. TNBC tumors are statistically larger in size, are of higher grade, and are biologically more aggressive compared to other cancer subtypes, with fewer than 30% of women with metastatic TNBC alive 5 years after diagnosis. These tumors constitute an important clinical challenge, as they do not respond to endocrine treatment or any other targeted therapies related to the absence of well-defined molecular targets.

One of the first molecular insights into TNBC came from the observation that BC from patients with *BRCA1* germline mutations, and from TNBC/BLBC patients, share a similar phenotype by immunohistochemistry or gene expression microarray [7]. Indeed, up to 90% of tumors with *BRCA1* mutation are triple-negative and about 10 to 20% of TNBC harbor a germline mutation in *BRCA1* [8],[9].

BRCA1 functions as a tumor suppressor protein that preserves genome integrity. Cells with homozygous *BRCA1* deficiency cannot repair DNA double-strand breaks, which results in a significant increase in genomic alterations and instability, finally leading to the development of tumors [10]. Several studies have shown that *BRCA1*-mutated tumors are sensitive to chemotherapy with DNA crosslinking agents such as platinum salts and alkylating agents, which lead to the formation of double-strand DNA breaks that cannot be repaired [11]-[13]. Another promising but still to be confirmed approach in the treatment of TNBC relies on the putative sensitivity of these tumors to poly ADP-ribose polymerase (PARP) inhibitors [14].

It is critical to identify tumors deficient in BRCA1 to select patients who could benefit from these selective treatments. The accumulation of gains and/or losses in the genome of *BRCA1*-mutated BC can generate a pattern of chromosomal aberrations that could constitute a molecular signature, and it has been shown that *BRCA1*-mutated tumors develop a particular comparative genomic hybridization (CGH) profile [13],[15]-[18]. In this study, we report the analysis by array CGH and fluorescent *in situ* hybridization (FISH) of 44 TNBC of known *BRCA1* status and well-defined histopathological features, and the identification of a recurrent region specifically gained in *BRCA1*-mutated TNBC. We have combined these results with gene expression data and highlighted the over-expression of genes that may prove to be important for TNBC development and progression.

## Methods

### Tumor samples

Formalin-fixed paraffin-embedded (FFPE) tumors were selected from the biobank of the Institute of Pathology and Genetics (IPG, Gosselies, Belgium). The *BRCA1* status was known for 53 patients screened in the context of a familial history of breast cancer or young age of diagnosis (23 with mutated TNBC, 9 with mutated non TNBC (7 with luminal A and 2 with HER2+ tumors) and 21 non-mutated TNBC). All the *BRCA1* wild-type tumors have a non-mutated *BRCA2* gene. We also analyzed 78 BC that were not screened for *BRCA1* mutation: 20 luminal A BC, 22 luminal B BC, 21 HER2+ BC and 15 TNBC.

In our study, luminal A was defined as ER+ or PR+, HER2−, and low Ki67 index (<14%); luminal B was defined as ER+ or PR+ and HER2− with high Ki67 index (≥30%); HER2+ was defined as ER+ or ER−, PR+ or PR− and HER2+ amplified. The TN group was defined as ER−, PR−, and HER2−. The cutoff for ER or PR to define a sample as negative was 3%.

These tumors were further characterized for cytokeratin (CK) 5/6, CK14, p63 and epidermal growth factor receptor (EGFR) expression by immunohistochemical (IHC) staining on tissue microarray (TMA). TMA were constructed as follows: H&E slides were reviewed by a pathologist to select representative infiltrating tumor area. Four tissue cores (0.6 μm diameter each, 3 tumoral and 1 normal control) were sampled from each block to account for tumor heterogeneity and inserted in a new receiver block. Staining IHC was performed in an Autostainer Plus (DAKO, Glostrup, Denmark) with the EnVisionFlex (DAKO, Glostrup, Denmark).

*BRCA1* and *BRCA2* status of patients was determined by geneticists with the patient agreement and according to the ethical rules in the context of a familial cancer investigation. All experiments involving human tissues were conducted with the permission of the ethics committee of the Grand Hôpital de Charleroi (GHdC, Charleroi, Belgium). Patients’ characteristics are summarized in Table 1.

**Table 1:** **Patient and tumor characteristics**

	*BRCA1*			*BRCA1*	*BRCA1*			
	Mutated			Non mutated	Unscreened			
Clinical characteristics	TN	Lum A	HER2+	TN	TN	Lum A	Lum B	HER2+
Tumors analyzed, n	23	7	2	21	15	20	22	21
Age, years, mean	44	44	48	50	59	63	59	59
(range)	(28 to 60)	(29 to 71)	(42 to 54)	(28 to 72)	(31 to 83)	(43 to 86)	(37 to 85)	(34 to 84)
**Histology, n (%)**								
Ductal	20	7	2	19	14	18	21	19
	(87%)	(100%)	(100%)	(90%)	(93%)	(90%)	(69%)	(90%)
Lobular	1	2	0	1	0	2	1	2
	(4%)	(29%)	(0%)	(5%)	(0%)	(10%)	(5%)	(10%)
Metaplastic	0	0	0	1	0	0	0	0
	(0%)	(0%)	(0%)	(5%)	(0%)	(0%)	(0%)	(0%)
Medullary	2	0	0	0	0	0	0	0
	(8.7%)	(0%)	(0%)	(0%)	(0%)	(0%)	(0%)	(0%)
Grade I	0	0	0	1	0	12	1	3
	(0%)	(0%)	(0%)	(5%)	(0%)	(60%)	(5%)	(14%)
Grade II	0	3	0	3	3	7	9	9
	(0%)	(43%)	(0%)	(14%)	(20%)	(35%)	(41%)	(43%)
Grade III	23	4	2	17	12	1	12	9
	(100%)	(57%)	(100%)	(81%)	(80%)	(5%)	(55%)	(43%)
**Immunohistochemistry, n (%)**								
ER-positive, n (%)	0	7	0	0	0	20	22	12
	(0%)	(100%)	(0%)	(0%)	(0%)	(100%)	(100%)	(57%)
PR-positive, n (%	0	5	0	0	0	20	22	12
	(0%)	(71%)	(0%)	(0%)	(0%)	(100%)	(100%)	(57%)
HER2-positive, n (%)	0	0	2	0	0	0	0	21
	(0%)	(0%)	(100%)	(0%)	(0%)	(0%)	(0%)	(100%)
KI67, mean, %	42	7	47	35	48	7	44	34
P63- or EGFR- or CK14- or CK5/6-positive, n (%)	11	1	1	7	9	0	1	3
	(48%)	(14%)	(50%)	(33%)	(60%)	(0%)	(5%)	(14%)
**Array CGH**								
Gain in 17q25.3, n (%)	20	7	2	6	4	0	3	4
	(87%)	(100%)	(100%)	(29%)	(27%)	(0%)	(14%)	(19%)

TN, triple-negative; Lum, luminal; HER2+, human epidermal growth factor 2-positive; ER, estrogen receptor; PR, progesterone receptor; EGFR, epidermal growth factor receptor; CK, cytokeratin; ACGH, array comparative genomic hybridization.

### Array comparative genomic hybridization (array CGH)

The invasive tumor component was selected by a pathologist on H&E sections of the FFPE blocks. Four to six 10-μm FFPE sections per tumor were used for CGH analyses and the infiltrating tumor region was macrodissected. Normal matched tissues were also analyzed as control (n = 4). DNA extraction, sample preparation and hybridization were carried out according to the manufacturer‘s protocols for FFPE samples (Number G4410-90020, Protocol version 3.3, August 2011, Agilent Technologies, Santa Clara, California, USA). A pool of seven different genomic DNA (gDNA) from seven healthy female donors, randomly chosen, was used as reference genome. CGH-array was performed using 8 × 60 K SurePrint G3 Human CGH Microarrays (Number G4450A, Agilent Technologies, Santa Clara, California, USA), which were scanned with an Agilent type C scanner (Agilent Technologies, Santa Clara, California, USA). Feature extraction software (version 10.7.3.1) with an hg19 annotated design file (021924_D_F_20111015) and a modified Agilent-provided extraction protocol (CGH_107_Sep09) (Agilent Technologies, Santa Clara, California, USA) were used to extract raw data from tiff files obtained after scanning. The array-based CGH data were analyzed with the Agilent Genomic Workbench 7.0 (Agilent Technologies, Santa Clara, California, USA). The *Z*-score algorithm was used with a threshold of 0.2 and a 2-Mb sliding window to detect significantly aberrant genomic regions (QQ-plots on the *P*-value from the enrichment analysis confirmed that the chosen threshold did not over-call some aberrations). Our copy number variation data were compared to the Database of Genomic Variants [19] to exclude genomic variants. Array CGH data are available through GEO [GSE54140].

### Enrichment analysis

Genomic intervals which have a significant overabundance of gains or losses in a given genomic region were identified with the hypergeometric tail algorithm available in Genomic Workbench 7.0 (Agilent Technologies, Santa Clara, California, USA). In Genomic Workbench 7.0, the genomic intervals are constructed from the data obtained with the aberration detection algorithm (*Z*-score algorithm) using a 2-Mb sliding window with a threshold at 0.2. The genomics intervals calculated by the *Z*-score algorithm are then used in the enrichment analysis. *P*-values of significant chromosomal regions (*P* ≤0.01) were –log-10 transformed and plotted according to the genomic position.

### Fluorescent *in situ*hybridization

FISH was performed using the Histology FISH Accessory kit (DAKO, Glostrup, Denmark) on 5-μm sections from FFPE samples, as recommended by the manufacturer. The probes consisted of a chromosome-17 centromeric probe (CEP17 Spectrum Green ™ probe, Vysis, Abbott Molecular, Des Plaines, Illinois, USA) and an in-house generated bacterial artificial chromosome (BAC) probe located in the amplified region of chromosome 17 (clone RP11-1055B8). The BAC probe was labeled with SpectrumOrange™ using a nick translation kit (Abbott Molecular, Des Plaines, Illinois, USA) and the specificity of the BAC probe was verified on leucocytes by metaphase FISH.

Images were collected with the BioView Duet™ System (BioView Ltd, Rehovot, Israel). Signals from both probes were scored in about 100 non-overlapping nuclei with malignant morphology in the infiltrating tumor zone (as selected by the pathologist). Adjacent fibroblasts or lymphocytes and residual normal breast tissues were used as internal control. For each tumor, the mean number of signals corresponding to the RP11-1055B8 probe was calculated. The ratio between the RP11-1055B8 probe and the centromeric probe was also evaluated for individual nuclei and the percentage of the nuclei with a ratio ≥3 was calculated for each TNBC. FISH slides were analyzed blinded to the CGH results.

### RNA isolation and cDNA synthesis

The invasive tumor zone, selected by the pathologist on H&E section, was macrodissected from unstained FFPE sections. Two to four 20-μm sections per tumor were used for the RNA extraction. Isolation of total ribonucleic acids was performed using the RecoverAll Total Nucleic Acid Isolation kit (Ambion, Life Technologies, Carlsbad, California, USA) according to the manufacturer’s protocol for FFPE samples. Extracted RNA was reverse transcribed into cDNA using the High Capacity cDNA Reverse Transcription Kit (Applied Biosystems, Life Technologies, Carlsbad, California, USA) according to the manufacturer’s recommendations.

### Taqman low-density arrays

Customised Taqman low-density arrays from Applied Biosystems (Life Technologies, Carlsbad, California, USA) were used to study the expression of 65 genes belonging to the 17q25.3 chromosomal region, as well as 18 putative housekeeping genes. For each tumor, one microgram of cDNA was loaded into two channels (500 ng cDNA/channel) of a microfluidic card, which was run on an Applied Biosystems 7900 HT Real-Time PCR System (Life Technologies, Carlsbad, California, USA) according to the manufacturer’s recommendation (Protocol number 4400263 Rev. B). Best housekeeping genes (*POLR2A, RPS11, GUSB, RPL37A, ACTB*) for data normalization were chosen according to their stability score calculated by the Expression Suite 1.0.1 software (Life Technologies, Carlsbad, California, USA). Transcript levels were expressed as 2^-ΔCt^, where ΔCt (cycle threshold) = CT_gene_ − CT_mean_ of five housekeeping genes.

### qPCR validation

Individual Taqman assays were performed in MicroAmp® Optical 96-Well Reaction Plates (Applied Biosystems, Life Technologies, Carlsbad, California, USA) according to the provided protocol (number 4333458 Rev. N 11/2010, Applied Biosystems, Life Technologies, Carlsbad, California, USA). Ten nanograms of cDNA were used in each qPCR reaction run on a 7900HT Real-Time PCR System (Applied Biosystems, Life Technologies, Carlsbad, California, USA). Based on the Taqman low-density array analyses previously carried out, *ACTB*, *GUSB*, *POLR2A*, *RPL37A* and *RPS11* were chosen as housekeeping genes. Transcript levels were expressed as 2^-ΔCt^, where ΔCt = CT_gene_ − CT_mean_ of five housekeeping genes.

### Methylation analysis

*BRCA1* promoter methylation was determined by real-time quantitative-methylation-specific PCR (QMSP) [20],[21]. The *BRCA1* amplicon used is significantly overlapping with the CpGs as previously reported by Veeck *et al*. [21]. Methylation ratios (× 1000) were determined relative to the *ACTB* reference gene. A standard curve of a serial dilution of a dual plasmid containing both *BRCA1* and *ACTB* was used for absolute quantification.

### Statistical analysis

FISH results were analyzed using the Mann-Whitney-Wilcoxon test. Statistical analysis of the Taqman low-density array data and the individual Taqman qPCR data was performed by Student’s *t*-test using Graphpad Prism software (GraphPad Software, La Jolla, California, USA) (*P <0.05; **P <0.01; ***P <0.001). The sensitivity was calculated as the proportion of *BRCA1*-mutated BC with a gain in 17q25.3 (true positives/true positives + false negative). The specificity was calculated as the proportion of BC without *BRCA1* mutation without a gain in 17q25.3 (true negative/true negative + false positive).

## Results

### Comparative analysis of *BRCA1*-mutated and non-mutated TNBC array CGH profiles

**Figure 1:**
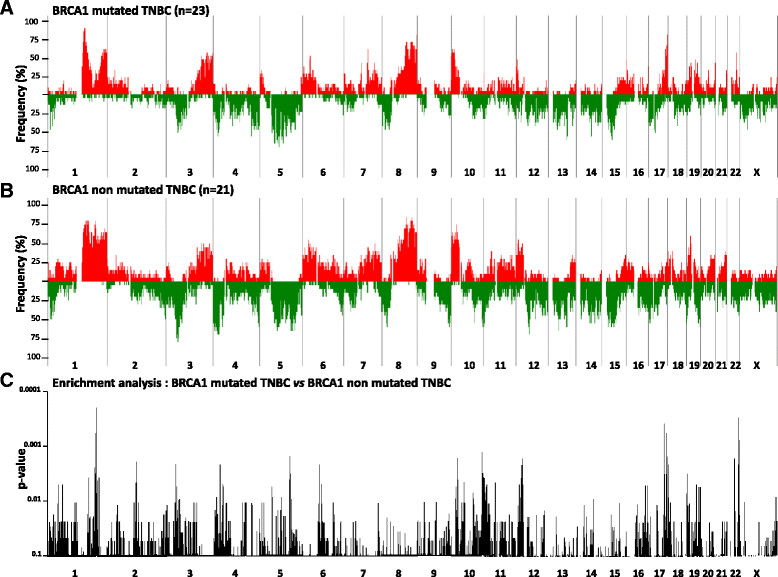
**Comparison of** ***BRCA1*** **-mutated and non-mutated triple-negative breast cancer (TNBC) array comparative genomic hybridization (CGH) aberration profiles.** Frequencies of copy number gains (red) and losses (green) are plotted as a function of genome location for 23 *BRCA1*-mutated **(A)** and 21 non-mutated **(B)** TNBC. The measure of the enrichment of the gains and losses in *BRCA1*-mutated TNBC in comparison to *BRCA1* non-mutated TNBC was calculated with the hypergeometric tail algorithm available in the Agilent software Genomic Workbench 7 (Agilent Technologies). *P*-values of significant chromosomal regions (*P* ≤0.1) are plotted according to the genomic position **(C)**.

As described in the literature, accumulation of gains and/or losses in the genome can generate a pattern of chromosomal aberrations, which could constitute a molecular signature [13],[15]-[18]. On the basis of these elements, we investigated whether such patterns of chromosomal aberrations could be observed in *BRCA1* mutated TNBC as compared to non mutated TNBC. Genomic DNA of 23 *BRCA1*-mutated and 21 non-mutated TNBC was studied by array CGH. For each tumor, all chromosome aberrations were plotted according to their position in the genome in order to generate an aberration profile. Individual profiles belonging to the same group of tumors (*BRCA1*-mutated or non-mutated) were summed to produce a global profile (Figure 1A and B). The general distribution of the genomic aberrations was relatively similar between the two tumor groups and showed a high proportion of gains and losses in both *BRCA1*-mutated and non-mutated TNBC (mutated TNBC: mean 102 aberrations per genome, range 18 to 211; non-mutated TNBC: mean 134 aberrations per genome, range 14 to 246). In order to pinpoint the significant differences between the two profiles, data were analyzed with the software Genomic Workbench 7.0 in combination with the hypergeometric tail algorithm (Figure 1C). We considered aberrations with a difference in frequency of at least 20% between the two groups, and with a *P*-value below 0.05.

The most frequent discriminating genomic alterations in the *BRCA1*-mutated group as compared to the non-mutated group were gains in 7p22.1, 8q24.3, 11p15.5, 15q22.31, 15q24.1-2, 16p24.3, 17q24.1, 17q24.3, 17q25.1, 17q25.3, 19q13.33, 22q12.3, 22q13.1, and losses in 5q14.3-21.2, 5q23.1, 11q24.3, 12q21.33, 18q12.1, 18q12.3, 19q13.2-3 (Table 2, and Table S1 in Additional file 1).

**Table 2:** **Main chromosomal aberrations detected in** ***BRCA1*** **-mutated TNBC**

		BRCA1-mutated		BRCA1-non-mutated	
**Chromosomes**	**Cytobandes**	**Gains, %**	**Losses, %**	**Gains, %**	**Losses, %**
chr5	q14.3 - q21.2	0.36	43.48	0.00	15.08
chr5	q23.1	0.00	39.13	0.00	14.29
chr7	p22.1	26.09	13.04	4.76	11.91
chr8	q24.3	82.61	4.35	57.14	9.52
chr11	p15.5	26.09	8.70	4.76	47.62
chr11	q24.3	4.35	26.09	19.05	4.76
chr12	p12.1	8.70	34.78	47.62	14.29
chr12	q21.33	0.00	39.13	4.76	9.52
chr15	q22.31	26.09	13.04	4.76	14.29
chr15	q24.1 - q24.2	23.92	16.67	2.38	33.33
chr16	q24.3	32.29	13.04	9.52	41.50
chr17	q24.1	39.13	4.35	14.29	4.76
chr17	q24.3	35.87	0.00	9.52	4.76
chr17	q25.1 - q25.3	58.95	3.59	25.12	21.63
chr18	q12.1	10.87	28.26	15.48	4.76
chr18	q12.3	0.00	42.03	0.00	14.29
chr19	q13.2	21.74	4.35	0.00	42.86
chr19	q13.33	21.74	8.70	0.00	42.86
chr19	q13.42	26.09	17.39	4.76	38.10
chr22	q12.3	49.28	11.59	11.91	30.16
chr22	q13.1	50.79	11.07	19.70	33.33

Significant aberrations (*P*-value ≤0.05) with a variation of gains or losses between *BRCA1*-mutated and non-mutated triple-negative breast cancer (TBNC) of at least 20% are indicated.

**Figure 2:**
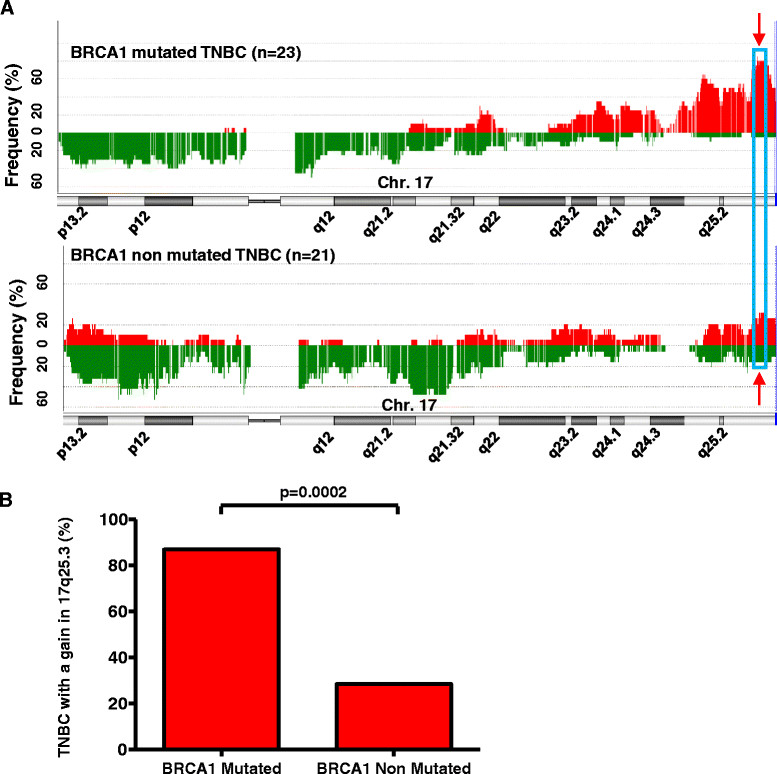
**Chromosomal aberrations along chromosome 17 in** ***BRCA1*** **-mutated and non-mutated triple-negative breast cancer (TNBC).** **(A)** Frequency of the chromosomal aberrations along chromosome 17 in *BRCA1*-mutated and non-mutated TNBC. Frequency of gains (red) and losses (green) on chromosome 17 are plotted according to their genome location for 23 *BRCA1*-mutated and 21 non-mutated TNBC. The most recurrent and significant aberration on chromosome 17 in *BRCA1*-mutated TNBC is surrounded by a blue frame in the q25.3 region. **(B)** Frequency of the 17q25.3 gain in *BRCA1-*mutated and non-mutated TNBC. The genome of 23 *BRCA1-*mutated and 21 non-mutated TNBC was analyzed by array comparative genomic hybridization (CGH). The presence of a chromosomal gain in the 17q25.3 region was determined for each tumor on the basis of the individual array CGH aberration profile analysis. Compared to the non-mutated TNBC, the gain in 17q25.3 was more frequent in *BRCA1*-mutated TNBC (*P* = 0.0002, two-tailed Fisher’s exact).

Several other significant gains and losses were detected in the non-mutated group, but these corresponded to regions that were preserved in *the BRCA1*-mutated tumors, like the chromosomal 1q31.3 region (Table S2 in Additional file 2). Amongst all the relevant aberrations, the most consistent gain in *BRCA1*-mutated TNBC was a highly significant (*P* = 0.0002) and recurrent gain of 2.4 Mb located between the position 78644018 and 81029941 on the chromosome 17 (17q25.3 (Human Genome built 37)). In the Database of Genomic Variant, most of the CNV in that region are losses, and we did not detect the 17q25.3 gain in normal tissue adjacent to positive tumor tissue, suggesting that this gain is not a copy number polymorphism. The gain in the 17q25.3 region was detected in 86.96% of *BRCA1*-mutated TNBC (20/23; sensitivity of 87%), and was only detected in 28.57% of non-mutated TNBC (6/21, specificity of 71%) (Figure 2A and B).

Other gains were also highly significant, but were less frequent in the mutated samples. For example, the 22q13.1 gain was only found in 13/23 (51%) of the TN mutated samples and was also found in 6/21 (20%) of the non-mutated TN samples.

Incorporating other significant regions (145 aberrations, Additional file 3: Table S3) into a classifier did not improve our sensitivity (21/23 cases detected, sensitivity of 91%), but did improve the specificity to 100% (see supplementary methods and results in Additional file 4). In this classifier, the chromosome 17 region 78260810-81029941 was also identified as the most important variable in discriminating between mutated and non-mutated samples. As the 17q25.3 gain was the most frequent gain in our *BRCA1-*mutated population, and has not yet been described in the context of *BRCA1* mutation, we have decided to characterize it further.

### Validation of the 17q25.3 gain by fluorescent *in situ*hybridization

**Figure 3:**
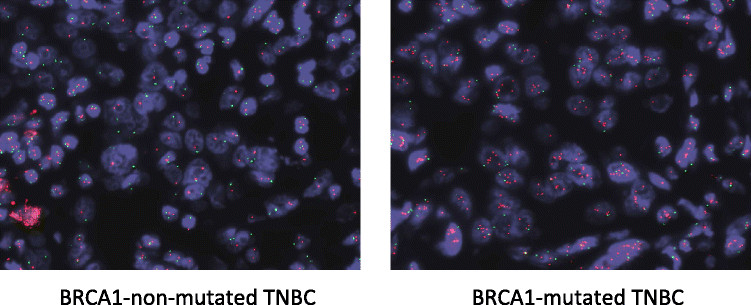
**Fluorescent** ***in situ*** **hybridization analysis of the 17q25.3 chromosomal region in** ***BRCA1*** **-mutated and non-mutated triple-negative breast cancer (TNBC).** Samples were hybridized with a chromosome 17 centromeric probe (green) and the 17q25.3-specific BAC probe (RP11-1055B8) (red). Copy number gain in the 17q25.3 region is clearly visible in this representative mutated case.

In order to validate the 17q25.3 gain detected by array CGH, FISH assays were carried out on all TNBC using a 17q25.3-specific BAC probe (BAC RP11-1055B8) together with a chromosome 17 centromeric control probe (cen 17 probe). In the FISH experiment illustrated in Figure 3, up to 10 copies of the 17q25.3 region were counted in the *BRCA1-*mutated tumor cells, whereas the signal was limited to two copies per nucleus in the non-mutated TNBC.

In all the cases where a gain in 17q25.3 was detected by CGH (20 *BRCA1-*mutated cases and 6 non-mutated cases), the gain was also detected by FISH and we observed an average of 3.5 copies of the 17q25.3 region and a maximum of 12 copies (median (IQR) 3.5 (2.7 to 4.2), n = 20 for the mutated samples, and 3.6 (2.9 to 4.6), n = 6 for the non-mutated cases). In the samples where a gain was not detected by CGH, the mean copy number of the 17q25.3 region was 2.0 (1.8 (1.8 to 2.7), n = 3) for the mutated samples and 2.1 ((1.7 to 2.6), n = 15 for the non mutated cases) (Table 3). The difference between the groups with or without a gain was highly significant (*P* <0.0001, Mann-Whitney test). These results confirm the data obtained by array CGH analysis.

**Table 3:** **Quantification of 17q25.3 gain in** ***BRCA1*** **-mutated and non-mutated TNBC by FISH**

*BRCA1*		aCGH		FISH: mean number of 17qter copies		Mann-Whitney test	FISH: % cells with ratio >3		Mann-Whitney test
		Gain in 17q25.3		Median (IQR)			Median (IQR)		
	Number	+	−	aCGH	aCGH	*P* -value	aCGH	aCGH	*P* -value
				Gain in 17q25.3	No gain in 17q25.3		Gain in 17q25.3	No gain in 17q25.3	
All	44	26	18	3.6 (2.9 to 4.2)	2.1 (1.7 to 2.6)	0.0001	20.6 (11.5 to 35.4)	0 (0 to 3.8)	0.0001
Mutated	23	20	3	3.5 (2.7 to 4.2)	1.8 (1.8 to 2.7)	0.0199	20.6 (9.9 to 34.2)	0 (0 to 10.6)	0.0253
Non-mutated	21	6	15	3.6 (2.9 to 4.6)	2.1 (1.7 to 2.6)	0.0009	24.0 (14.1 to 33.9)	0 (0 to 3.5)	0.0007

TNBC, triple-negative breast cancer; aCGH, array comparative genomic hybridization.

We also evaluated the 17q25.3/cen 17 ratio in individual nuclei of the *BRCA1-*mutated or non-mutated group. The percentage of nuclei with a 17q25.3/cen 17 ratio above 3% was higher in the CGH-positive cases (median (IQR) 20.6 (11.5 to 35.4), 20 mutated samples and 6 non-mutated samples), compared to CGH-negative cases (median (IQR) 0.0 to 3.8), 15 non-mutated samples and 3 mutated samples) (Table 3). In summary, the CGH results were confirmed by FISH and these analyses confirmed the significant recurrence of the 17q25.3 gain in *BRCA1-*mutated TNBC.

### Study of the 17q25.3 gain in different subgroups of breast cancers

**Figure 4:**
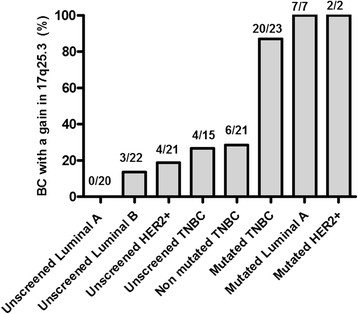
**Frequency of the 17q25.3 gain in different subgroups of breast cancers.** The genome of 20 luminal A (LumA), 22 luminal B, 21 human epidermal growth factor 2-positive (HER2+), 15 sporadic triple-negative breast cancer (TNBC), 9 *BRCA1-*mutated non-triple-negative (TN) breast cancers (7 LumA and 2 HER2+), as well as the genome of 23 *BRCA1-*mutated and 21 non-mutated TNBC was analyzed by array comparative genomic hybridization (array CGH). The presence of the 17q25.3 gain was assessed in these tumors on the basis of the individual array CGH aberration profile analysis.

According to our CGH analyses, the 17q25.3 gain is highly recurrent in TNBC with *BRCA1* mutation. We then investigated if the 17q25.3 gain could be detected in other subgroups of breast cancer. The presence of the 17q25.3 gain was assessed in 9 *BRCA1-*mutated non-TNBC (7 luminal A and 2 HER2+) and in a cohort of 78 BC unscreened for *BRCA1* mutation (20 luminal A, 22 luminal B, 21 HER2+ and 15 TNBC) (Table 1). The presence of the gain was determined on the basis of the individual array CGH profiles generated for each tumor. In the *BRCA1* unscreened population, the 17q25.3 gain was only detected in 3/22 luminal B (13.6%), 4/21 HER2+ (19%) and 4/15 TNBC (26.7%). No 17q25.3 gain was observed in the luminal A subgroup. The gain was detected in all the *BRCA1-*mutated non-TNBC (9/9) (100%) (Figure 4).

In summary, the prevalence of 17q25.3 gain did not exceed 17.2% in unscreened and non-mutated breast cancers (17/99), while the gain was observed in more than 90% of the *BRCA1-*mutated tumors (29/32). To further validate our results on an independent cohort, we analyzed the Breast Invasive Carcinoma cohort from TCGA [22] (Additional file 4). This cohort contains mutation data for 507 samples, copy number data for 778 samples, expression data for 526 samples and methylation data for 311 samples. BC cases (77 TN, 201 LumA, 101 LumB and 38 HER2+) were selected based on the classification data available in the TCGA cohort description (IHC and PAM50 classification). Fourteen cases have a mutation in *BRCA1*: 10 TNBC, 3 luminal A BC, 1 HER2+ BC (Table S5 in Additional file 4).

A gain in 17q25.3 was detected in 70% (7/10) of the *BRCA1-*mutated TNBC and in one of the mutated luminal A BC. No gain was detected in the mutated HER2+ BC, but the mutation in that sample is classified as low impact (p.D1344H) and might have no impact on the protein function. The gain was detected in 30% (20/67) of the TNBC without identified *BRCA1* mutation, in 36% (14/38) of the HER2+ BC cases, in 17% (35/201) of the luminal A cases and in 43% (43/101) of the luminal B cases. Thus, in the TCGA cohort, the gain in 17q25.3 was also more frequent in the *BRCA1-*mutated samples compared to BC without identified *BRCA1* mutation.

Interestingly, 26% (9 of the 35) of the LumA, 23% (10/43) of the LumB, 28% (4/14) of HER2+ and 45% (9/20) of the non-mutated TN with a 17q25.3 gain have a mutation, homozygous deletion or diminished expression in other genes involved in DNA repair (*ATM*, *BRCA2*, *CHECK2*, *FANCF*, *MLH1*, et cetera).

### Gene expression analysis of the 17q25.3 chromosomal region in *BRCA1-*mutated and non-mutated TNBC

**Figure 5:**
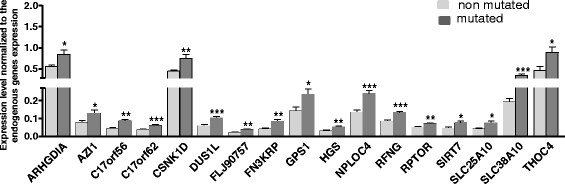
**Genes of the 17q25.3 chromosomal region differentially expressed between** ***BRCA1-*** **mutated (n = 15) and non-mutated triple-negative breast cancer (TNBC) (n = 13).** The analysis was carried out using customized Taqman low-density arrays. Statistical analysis: two-tailed Student’s *t*-test: *P <0.05; **P <0.01; ***P <0.001.

Several studies have shown that genome amplification can lead to the over-expression of genes located in the amplified regions (amplicon). This over-expression may contribute to tumor development and progression. According to our CGH analyses, the 17q25.3 subregion of interest spreads from the position 78644018 to 81029941 (NCBI built 37) and contains 65 protein coding genes. We investigated by low-density Taqman array the expression of the 65 genes of the 17q25.3 region in *BRCA1-*mutated and non-mutated TNBC previously analyzed by array CGH. Relative expression levels were compared to identify significant differently expressed genes in mutated tumors. Expression data analysis identified a significant over expression of 17 genes (*ARHGDIA*, *AZI1*, *C17orf56*, *C17orf62*, *CSNK1D*, *DUS1L*, *FLJ90757*, *FN3KRP*, *GPS1*, *HGS*, *NPLOC4*, *RFNG*, *RPTOR*, *SIRT7*, *SCL25A10*, *SCL38A10*, *THOC4*) in *BRCA1-*mutated TNBC (and CGH-positive for the gain in the 17q25.3 region) as compared to the non-mutated TNBC (and CGH-negative for the 17q25.3 region). Relative expression levels of these genes are presented in Figure 5 and in Additional file 4: Table S6).

**Figure 6:**
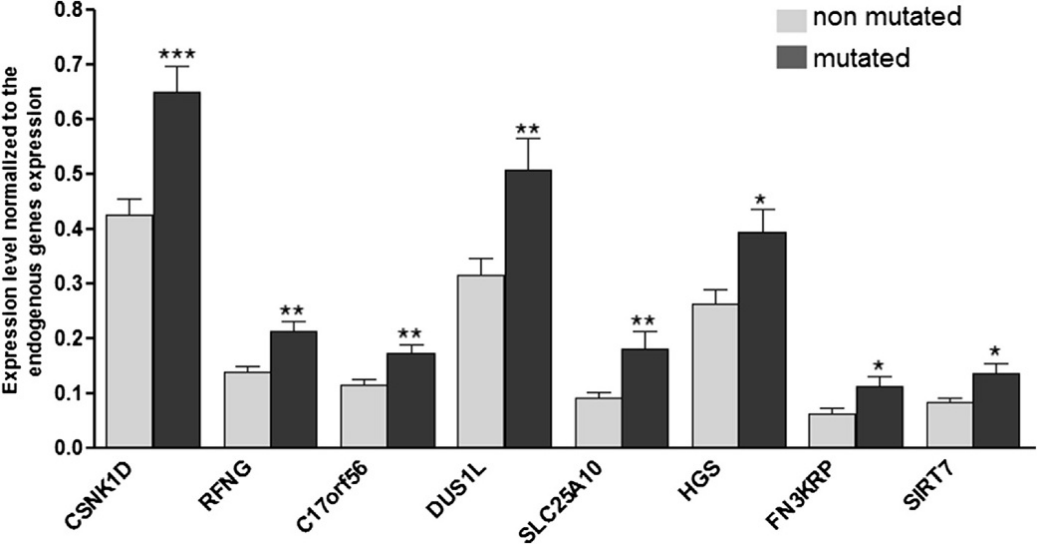
**Validation of Taqman low-density array analysis** **(TLDA)** **gene expression level.** The expression of eight genes previously studied by TLDA (*CSNK1D*, *RFNG*, *C17orf56*, *DUS1L*, *SLC25A10*, *HGS*, *FN3KRP*, *SIRT7*) was assessed in 15 *BRCA1-*mutated and 13 non-mutated triple-negative breast cancer (TNBC) using a standard qPCR method. Statistical analysis: two-tailed Student’s *t*-test: *P <0.05; **P <0.01; ***P <0.001.

In order to validate the expression levels measured with the Taqman low-density arrays, individual Taqman assays were performed on 15 *BRCA1-*mutated and 13 non-mutated TNBC. In this validation step, the relative expression of 8 out of the 17 genes was assessed (*C17orf56*, *CSNK1D*, *DUS1L*, *FN3KRP*, *HGS*, *SIRT7*, *SCL25A10*, *RFNG*). The results of this validation, presented in Figure 6 and Additional file 4: Table S7, confirm the over expression detected by Taqman low density arrays in *BRCA1-*mutated TNBC with a gain in 17q25.3. Compared to the non-mutated cases, expression levels are increased up to two times in the *BRCA1-*mutated group. As the RNA extracted from the tumor is probably contaminated by RNA extracted from normal cells, with TNBC often deeply infiltrated by numerous stromal and inflammatory diploid cells, the over expression in the tumor cells could be more important than what we have observed.

To validate our experimental results, the expression of several genes of the 17q25.3 region (*ARHGDIA*, *AZI1*, *C17orf56*, *DUS1L*, *FN3KRP*, *HGS*, *SIRT7*, *SCL25A10)* was compared in TNBC with or without a gain in the TCGA study (*Z*-scores values retrieved from [23]). In agreement with our own observations, tumors with copy number gains have an increased gene expression compared to cases without the 17q25.3 gain (Figure S2 in Additional file 4).

To gain insight into the importance of the genes in the 17q25.3 region, we analyzed publically available shRNA screen databases (COLT-cancer and genomeRNAi, Table S8 and S9 in Additional file 4) [24],[25]. In COLT-cancer, 9 of the 16 genes analyzed in our study (*AZI1*, *CSNK1D*, *DUS1L*, *FN3KRP*, *GPS1*, *RFNG*, *RPTOR*, *THOC4* and *HGS*) were found to be essential for survival in at least one cancer cell line. Some genes, like *THOC4*, are essential in almost all the cell lines tested. Interestingly, several of the genes in the 17q25.3 region were identified in shRNA screenings for genes involved in maintenance of genome stability or response to DNA damage (*ARGHDIA*, *GPS1*, *SLC38A10*, *THOC4*) [26]-[30].

### Methylation status of the *BRCA1*gene promoter in 17q25.3-amplified TNBC

The gain in the 17q25.3 region is highly recurrent in the *BRCA1-*mutated TNBC. However, this gain was also detected in non-mutated or unscreened samples. Epigenetic inactivation of tumor suppressor genes is a well-known mechanism. Methylation of the *BRCA1* promoter has been reported in about 10 to 30% of breast cancers [31]-[33] and *BRCA1-*methylated tumors have a *BRCA1*-like phenotype [34]. The presence of the 17q25.3 gain in the non-mutated TNBC could possibly be explained by this mechanism and hypermethylation of *BRCA1* promoter was assessed in the BC analyzed by array CGH.

**Figure 7:**
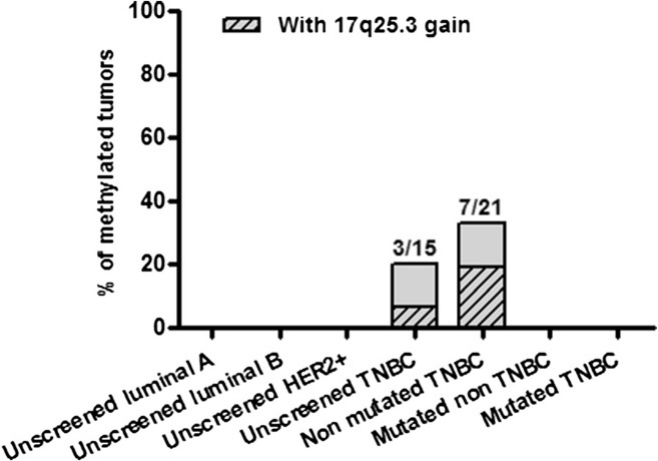
***BRCA1*** **promotor methylation in breast cancer (BC).** *BRCA1* promoter methylation was analyzed in 20 luminal A, 22 luminal B, 21 HER2+, 15 unscreened TN, 21 non-mutated TN, 23 *BRCA1-*mutated triple-negative (TN) and 9 mutated non-TN BC. Methylation of the *BRCA1* promoter was only detected in the unscreened (3/15, 20%) or non-mutated (7/21, 33%) TNBC.

Interestingly*, BRCA1* promoter methylation was not detected in the TNBC (n = 21) and non-TNBC (n = 9) carrying a *BRCA1* germline mutation. In the non-mutated TNBC, *BRCA1* promoter methylation was detected in seven TNBC (33%). Amongst these seven cases, four displayed a *BRCA1*-like profile, with a gain in 17q25.3. In the unscreened TNBC, *BRCA1* promoter methylation was observed in three tumors (20%) and the gain in 17q25.3 was detected in one TNBC (Figure 7). Altogether, the gain was detected in 50% (5/10) of the TNBC with *BRCA1* promoter methylation.

We did not detect *BRCA1* promoter methylation in the LumA, Lum B and HER2+ BC. To confirm our results, *BRCA1* promoter methylation data (HM27 beta value data, available for 311 samples) were retrieved for the TCGA study (samples with HM27 beta value >0.5 and *BRCA1* expression downregulation were considered as methylated). Methylation of the *BRCA1* promoter was not detected in the 14 *BRCA1* mutated samples from the TCGA study, but was detected in 16% of the TNBC without BRCA1 mutation (5/31 samples), in 0% of LumA (0/101) samples, in only 5% of LumB (3/64) samples and in 0% of HER2+ (0/31) samples (Figure S3 in Additional file 4). Amongst the sample with *BRCA1* promoter methylation, 50% (4/8) have a gain in 17q25.3, a result similar to our own observations.

## Discussion

We report the genomic profiles for *BRCA1-*mutated, non-mutated and unscreened TN breast tumors using DNA arrays composed of 60,000 probes with an average resolution of 40 kb. Some of the aberrations reported in this study have been described before, and confirm findings from other studies using techniques with lower resolution, including losses in 5q11-23 and 12q21 and gains in 8q24 that were previously shown to be associated with *BRCA1* tumors [15],[17],[18],[35].

We also describe the presence in *BRCA1-*mutated BC of a new recurrent and highly significant gain in the q25.3 region of chromosome 17. This 17q25.3 gain was detected in 90% of the *BRCA1-*mutated BC (86% of the mutated TNBC and 100% of the mutated non-TNBC) in our cohort and was also highly recurrent in the *BRCA1*-mutated breast cancer of the TCGA cohort. The gain was also present in 50% of the TNBC with *BRCA1* promoter methylation (TNBC unscreened for germline mutation or non-mutated TNBC).

Interestingly, in our study, *BRCA1* epigenetic silencing and *BRCA1* mutation are mutually exclusive events, as *BRCA1* promoter methylation was never detected in *BRCA1-*mutated TNBC and was detected in 33% of the non-mutated TNBC. Our observations confirm similar results reported in previous studies in ovarian and breast cancer [33],[36]-[38].

Amplification of the 17qter region has been described in several other cancer types, like prostate or ovarian cancers. A highly recurrent gain on chromosome 17q25.3 was reported in prostate cancer [39]. The region identified overlaps with the same region on chromosome 17q identified in our CGH assay. The significance of the presence of the 17q25.3 amplification in prostate cancer in relation to BRCA1 deficiency is not clear. In hereditary ovarian carcinoma, Zweemer *et al*. have observed an amplification of the 17q24-25 region in 19% of the cases (*BRCA1-* or *BRCA2-*mutated and familial but non-mutated) [40]. Leunen *et al*. also reported a gain in 17q25 in *BRCA1*-mutated ovarian tumors [41]. However, to our knowledge, the 17q25.3 gain has never before been associated with *BRCA1* mutation in breast cancer. The gain in 17q25.3 is highly recurrent in BRCA1 deficient TNBC, but was also detected in two non-mutated and unmethylated TNBC, and in two unscreened and unmethylated TNBC, in three LumB and in four HER2+ BC.

We cannot exclude a *BRCA1* mutation in the unscreened BC, but this is very unlikely considering the rarity of the *BRCA1* mutation in luminal and HER2+ BC. For ethical reasons, it has not been possible to sequence *BRCA1* in these samples. Several other mechanisms could be responsible for *BRCA1* downregulation or BRCA1 pathway dysfunction in the TNBC samples. One such mechanism could be the action of the DNA binding protein ID4. This protein is highly expressed in TNBC and it has been shown that ID4 can block *BRCA1* gene transcription *in vitro* and possibly downregulate *BRCA1* expression *in vivo* [42]. In the HER2+ BC, Chr17 usually displays very complex rearrangements [43]. This could be responsible for the 17q25.3 gain detected by CGH in these HER2+ amplified tumors.

In our work, some differences were observed in comparison to the CGH profiles that are available in the literature [13],[15],[17],[18],[35],[44]. Such differences may arise from bias in patient selection. One of the major caveats of such studies is the heterogeneity of the tumor groups analyzed. Indeed, *BRCA1-*mutated breast cancers, which are mainly TN tumors, are often compared to non-mutated tumors, is the majority of which are not TNBC, but luminal A, luminal B or HER2+ tumors. Similarly, some studies involved BLBC, which constitute a heterogeneous group of tumors composed of about 75% TNBC and 25% non-TNBC (luminal A, luminal B, HER2+ BC) [11],[45]. In this study, we have compared the genomic alterations linked to a defect in *BRCA1* within one breast cancer subtype, the TNBC. Another important methodological difference that must be considered is the type of CGH arrays used to perform the analyses. Most of the studies used BAC arrays that typically scan the genome at 1- or 2-Mb resolution, which can be considered low. The array CGH used in our study allow genome coverage at a mean resolution of about 40 kb. A better coverage of the genome allows for the identification of more numerous and smaller aberrations that cannot be detected with analytical tools of lower resolution. The coverage of the genome can also be different. For example, the BAC arrays used in the work of Joosse do not cover the specific subregion of the 17q25.3 cytoband that we detected in our study [17]. The homogeneity of the tumor groups as well as the 60 k CGH arrays used in our study could explain, at least in part, the fact that we were able to detect the 17q25.3 amplification in *BRCA1-*mutated tumors that was not found in the other studies.

It has been shown that genes that are consistently over-expressed when amplified may be required for the survival of cancer cells and may also have the potential to be therapeutic targets [46]-[48]. We validated eight genes of the 17q25.3 region that were significantly over-expressed in tumors harboring the 17q25.3 gain compared to negative tumors (*C17orf56*, *CSNK1D*, *DUS1L*, *FN3KRP*, *HGS*, *SIRT7*, *SCL25A10*, *RFNG*). Some of these over-expressed genes in the 17q25.3 region have been linked to cancer development and progression. The Casein kinase 1 delta (CSNK1D) has been implicated in colon, pancreatic and breast cancer and reduced CSNK1D activity impairs mammary tumorigenesis *in vivo* [49],[50]. HGS, the hepatocyte growth factor-regulated tyrosine kinase substrate, has been implicated in tumor growth and metastasis and over-expression of HGS prevents the degradation of the EGF receptor [51],[52].

It has recently been demonstrated that SIRT7 is a selective H3K18Ac deacetylase that mediates transcriptional repression and stabilizes the cancer cell phenotype [53]. Deletion of *SIRT7* in a xenograft model inhibits the growth of human cancer cells. In this context, it is tempting to make a link between the over-expression of SIRT7 and the sensitivity of some TNBC to deacetylase inhibitor [54].

Some of the genes of the 17q25.3 region have been linked to DNA repair in shRNA screens and their over-expression might affect the DNA repair capacity of the cell. Hence, upregulation of the DNA repair capacity of the cancer cell might represent a mechanism used to overcome the lack of a *BRCA1*-dependent repair mechanism.

Further studies will be carried out to address the functional significance and clinical relevance of the 17q25.3 gain and gene over-expression in *BRCA1-*mutated breast cancer, such as the prediction of the response to alkylating agent or platinum-based therapy.

## Conclusions

In this project, we have established an array CGH profile of *BRCA1* mutant TNBC. Particularly, we have identified a gain in the 17q25.3 region that is highly recurrent in *BRCA1-*mutated breast cancer. Several genes of this region are upregulated and might represent potential therapeutic targets.

## Additional files

## Electronic supplementary material

Additional file 1: Table S1.: Chromosomal aberrations detected in *BRCA1* mutated and non-mutated triple-negative breast cancer (TNBC) by array comparative genomic hybridization (CGH). (PDF 1006 KB)

Additional file 2: Table S2.: Gains and losses detected in BRCA1 non-mutated triple-negative breast cancer (TNBC) by array comparative genomic hybridization (CGH). (PDF 35 KB)

Additional file 3: Table S3.: List of the 145 most important segments. (PDF 370 KB)

Additional file 4: **Supplementary methods and results.** **Table S4.** classification results. **Table S5.** list of samples from the triple-negative breast cancer (TCGA) breast cancer used in this study. **Table S6.** Relative gene expression in mutated and non-mutated TNBC (Taqman low density arrays). **Table S7.** Relative gene expression in mutated and non-mutated TNBC (Taqman qPCR). Table S8 COLT-cancer database analysis. **Table S8.** GenomeRNAI database analysis. Figures S1 to S3. (PDF 904 KB)

Below are the links to the authors’ original submitted files for images.

Authors’ original file for figure 1

Authors’ original file for figure 2

Authors’ original file for figure 3

Authors’ original file for figure 4

Authors’ original file for figure 5

Authors’ original file for figure 6

Authors’ original file for figure 7

## Abbreviations

Array CGH: array comparative genomic hybridization
BAC: bacterial artificial chromosome
BC: breast cancer
BLBC: basal-like breast cancer
*BRCA1*: Breast cancer 1 gene
*BRCA2*: Breast cancer 2 gene
CK 14: cytokeratin 14
CK5/6: cytokeratin 5/6
Ct: cycle threshold
EGFR: epidermal growth factor receptor
ER: estrogen receptor
FFPE: formalin-fixed paraffin-embedded
FISH: fluorescent *in situ* hybridization
HE: hematoxylin and eosin stain
HER2: human epidermal growth factor receptor 2
IHC: immunohistochemical
PARP: poly ADP ribose polymerase
PR: progesterone receptor
TMA: tissue microarray
TNBC: triple negative breast cancer
