# Supplementary material for: Identification by array comparative genomic hybridization of a new amplicon on chromosome 17q highly recurrent in BRCA1 mutated triple negative breast cancer
Source: Breast Cancer Res. 2014 Nov 22;16:466. doi: 10.1186/s13058-014-0466-y (PMC4303204; doi:10.1186/s13058-014-0466-y)
Supplement: Supplementary file 1 — Additional file 1: Table S1.: Chromosomal aberrations detected in BRCA1 mutated and non-mutated triple-negative breast cancer (TNBC) by array comparative genomic hybridization (CGH). (PDF 1006 KB) [file 13058_2014_466_MOESM1_ESM.pdf]

Chr Name	Start	Stop	Size	No of Probes	Num Gains in Mutated	Num Losses in Mutated	-log10(PVal) Gain in Mutated	-log10(PVal) Loss in Mutated	Num Gains in Non Mutated	Num Losses in Non Mutated	-log10(PVal) Gain in Non Mutated	-log10(PVal) Loss in Non Mutated
chr1	759762	890945	131184	3	1	2	0.30102999	0.1575501	0	2	0	0.1575501
chr1	890945	891004	60	1	2	2	0.30102999	0.1575501	1	2	0.05404976	0.1575501
chr1	891004	1084501	193498	4	2	2	0.61140001	0.1575501	0	2	0	0.1575501
chr1	1084501	1110607	26107	2	2	2	0.61140001	0.0429175	0	4	0	0.47744371
chr1	1110607	1189360	78754	2	2	2	0.61140001	0.08289318	0	3	0	0.30102999
chr1	1189360	1249207	59848	3	2	2	0.61140001	0.0429175	0	4	0	0.47744371
chr1	1249207	1446819	197613	1	1	2	0.30102999	0.0429175	0	4	0	0.47744371
chr1	1446819	1786789	339971	7	1	2	0.30102999	0.02162467	0	5	0	0.68214471
chr1	1786789	1786848	60	1	2	2	0.30102999	0.02162467	1	5	0.05404976	0.68214471
chr1	1786848	1860942	74095	1	2	2	0.61140001	0.02162467	0	5	0	0.68214471
chr1	1860942	1861001	60	1	2	3	0.61140001	0.05670724	0	5	0	0.45545077
chr1	1861001	2013419	152419	3	1	2	0.30102999	0.02162467	0	5	0	0.68214471
chr1	2013419	2080309	66891	2	1	3	0.30102999	0.05670724	0	5	0	0.45545077
chr1	2080309	2186829	106521	3	1	3	0.30102999	0.03070643	0	6	0	0.63695542
chr1	2186829	2239438	52610	1	1	2	0.30102999	0.01053319	0	6	0	0.91219088
chr1	2239438	2281699	42262	2	2	3	0.61140001	0.03070643	0	6	0	0.63695542
chr1	2281699	2335155	53457	1	1	3	0.30102999	0.03070643	0	6	0	0.63695542
chr1	2335155	2455937	120783	2	1	2	0.30102999	0.01053319	0	6	0	0.91219088
chr1	2455937	2495194	39258	2	2	4	0.30102999	0.06713722	1	6	0.05404976	0.44141547
chr1	2495194	2723344	228151	2	2	3	0.61140001	0.03070643	0	6	0	0.63695542
chr1	2723344	2785042	61699	2	2	4	0.61140001	0.03812622	0	7	0	0.60763643
chr1	2785042	2850650	65609	2	2	5	0.1575501	0.07511598	2	7	0.1575501	0.43181735
chr1	2850650	2996775	146126	3	2	4	0.61140001	0.03812622	0	7	0	0.60763643
chr1	2996775	3138506	141732	2	1	4	0.30102999	0.03812622	0	7	0	0.60763643
chr1	3138506	3197921	59416	2	1	4	0.05404976	0.03812622	2	7	0.30102999	0.60763643
chr1	3197921	3197980	60	1	1	4	0.02438896	0.03812622	3	7	0.51676182	0.60763643
chr1	3197980	3314899	116920	2	1	3	0.02438896	0.01598258	3	7	0.51676182	0.84395715
chr1	3314899	3314958	60	1	2	3	0.08289318	0.01598258	3	7	0.30102999	0.84395715
chr1	3314958	3354713	39756	1	2	2	0.08289318	0.00493743	3	7	0.30102999	1.16581773
chr1	3354713	3354772	60	1	2	2	0.0429175	0.00493743	4	7	0.47744371	1.16581773
chr1	3354772	3472908	118137	3	1	2	0.01091641	0.00493743	4	7	0.76005302	1.16581773
chr1	3472908	3493063	20156	2	2	2	0.02162467	0.00493743	5	7	0.68214471	1.16581773
chr1	3493063	3531051	37989	1	1	2	0.01091641	0.00493743	4	7	0.76005302	1.16581773
chr1	3531051	3660051	129001	2	0	2	0	0.00493743	3	7	0.93173516	1.16581773
chr1	3660051	3753484	93434	3	0	3	0	0.01598258	4	7	1.26272838	0.84395715
chr1	3753484	3753543	60	1	2	3	0.0429175	0.01598258	4	7	0.47744371	0.84395715
chr1	3753543	3800088	46546	1	1	3	0.01091641	0.01598258	4	7	0.76005302	0.84395715
chr1	3800088	4028587	228500	1	0	3	0	0.03070643	1	6	0.30102999	0.63695542
chr1	4028587	4087443	58857	1	0	3	0	0.03070643	0	6	0	0.63695542
chr1	4087443	4271025	183583	2	0	2	0	0.01053319	0	6	0	0.91219088
chr1	4271025	4316908	45884	2	0	3	0	0.03070643	0	6	0	0.63695542
chr1	4316908	4401750	84843	2	1	3	0.30102999	0.03070643	0	6	0	0.63695542
chr1	4401750	4458182	56433	1	1	3	0.30102999	0.05670724	0	5	0	0.45545077
chr1	4458182	4629201	171020	2	0	2	0	0.0429175	0	4	0	0.47744371
chr1	4629201	4725355	96155	2	1	2	0.30102999	0.0429175	0	4	0	0.47744371
chr1	4725355	4958499	233145	4	0	2	0	0.0429175	0	4	0	0.47744371
chr1	4958499	4958558	60	1	1	3	0.30102999	0.10122019	0	4	0	0.30102999
chr1	4958558	5252926	294369	2	0	2	0	0.0429175	0	4	0	0.47744371
chr1	5252926	5491640	238715	2	1	2	0.30102999	0.02162467	0	5	0	0.68214471
chr1	5491640	5491699	60	1	1	3	0.30102999	0.05670724	0	5	0	0.45545077
chr1	5491699	5578149	86451	1	1	2	0.30102999	0.0429175	0	4	0	0.47744371
chr1	5578149	6130376	552228	7	1	0	0.30102999	0	0	4	0	1.26272838
chr1	6130376	6282504	152129	4	1	0	0.30102999	0	0	5	0	1.60515106
chr1	6282504	6411511	129008	3	1	1	0.30102999	0.00478973	0	5	0	1.02643191
chr1	6411511	6457775	46265	2	1	1	0.1218695	0.00478973	1	5	0.1218695	1.02643191
chr1	6457775	6524138	66364	1	1	0	0.30102999	0	0	5	0	1.60515106
chr1	6524138	6524197	60	1	1	1	0.30102999	0.00478973	0	5	0	1.02643191
chr1	6524197	6642305	118109	2	1	0	0.30102999	0	0	5	0	1.60515106
chr1	6642305	6823203	180899	4	1	1	0.30102999	0.00478973	0	5	0	1.02643191
chr1	6823203	6939147	115945	3	1	2	0.30102999	0.02162467	0	5	0	0.68214471
chr1	6939147	7156165	217019	5	1	3	0.30102999	0.05670724	0	5	0	0.45545077
chr1	7156165	7156224	60	1	1	4	0.30102999	0.11390336	0	5	0	0.30102999
chr1	7156224	7251615	95392	2	0	4	0	0.11390336	0	5	0	0.30102999
chr1	7251615	7350829	99215	2	0	3	0	0.05670724	0	5	0	0.45545077
chr1	7350829	7469863	119035	3	0	3	0	0.03070643	0	6	0	0.63695542
chr1	7469863	7527447	57585	1	0	2	0	0.01053319	0	6	0	0.91219088
chr1	7527447	7630342	102896	3	0	3	0	0.03070643	0	6	0	0.63695542
chr1	7630342	7630401	60	1	0	4	0	0.06713722	0	6	0	0.44141547
chr1	7630401	7836835	206435	4	0	3	0	0.03070643	0	6	0	0.63695542
chr1	7836835	7912091	75257	1	0	2	0	0.02162467	0	5	0	0.68214471
chr1	7912091	7995264	83174	1	0	2	0	0.0429175	0	4	0	0.47744371
chr1	7995264	8032149	36886	2	0	2	0	0.02162467	0	5	0	0.68214471
chr1	8032149	8181042	148894	2	0	1	0	0.01091641	0	4	0	0.76005302
chr1	8181042	8343754	162713	2	0	0	0	0	0	4	0	1.26272838
chr1	8343754	8343813	60	1	0	0	0	0	1	4	0.30102999	1.26272838
chr1	8343813	8380210	36398	1	0	0	0	0	1	3	0.30102999	0.93173516
chr1	8380210	8427574	47365	1	0	0	0	0	1	2	0.30102999	0.61140001
chr1	8427574	8427633	60	1	0	1	0	0.05404976	1	2	0.30102999	0.30102999
chr1	8427633	8518072	90440	2	0	1	0	0.1218695	1	1	0.30102999	0.1218695
chr1	8518072	8586452	68381	2	0	1	0	0.1218695	2	1	0.61140001	0.1218695
chr1	8586452	8653009	66558	1	0	1	0	0.1218695	1	1	0.30102999	0.1218695
chr1	8653009	8852383	199375	4	1	1	0.05404976	0.1218695	2	1	0.30102999	0.1218695
chr1	8852383	8971819	119437	4	1	1	0.01091641	0.1218695	4	1	0.76005302	0.1218695
chr1	8971819	9087907	116089	2	1	1	0.02438896	0.1218695	3	1	0.51676182	0.1218695
chr1	9087907	9389926	302020	8	1	1	0.01091641	0.1218695	4	1	0.76005302	0.1218695
chr1	9389926	9604625	214700	4	1	1	0.01091641	0.05404976	4	2	0.76005302	0.30102999
chr1	9604625	9816708	212084	5	1	1	0.01091641	0.02438896	4	3	0.76005302	0.51676182
chr1	9816708	9910349	93642	3	1	1	0.01091641	0.00478973	4	5	0.76005302	1.02643191
chr1	9910349	9910408	60	1	1	2	0.01091641	0.02162467	4	5	0.76005302	0.68214471
chr1	9910408	9953030	42623	1	1	2	0.01091641	0.0429175	4	4	0.76005302	0.47744371
chr1	9953030	9990357	37328	1	1	1	0.01091641	0.01091641	4	4	0.76005302	0.76005302
chr1	9990357	10059707	69351	2	1	1	0.01091641	0.00478973	4	5	0.76005302	1.02643191
chr1	10059707	10059761	55	1	1	2	0.01091641	0.01053319	4	6	0.76005302	0.91219088
chr1	10059761	10115173	55413	1	1	2	0.05404976	0.01053319	2	6	0.30102999	0.91219088
chr1	10115173	10162949	47777	2	1	2	0.05404976	0.00493743	2	7	0.30102999	1.16581773
chr1	10162949	10163008	60	1	1	2	0.02438896	0.00221948	3	8	0.51676182	1.44210395
chr1	10163008	10240877	77870	2	1	2	0.05404976	0.00221948	2	8	0.30102999	1.44210395
chr1	10240877	10282212	41336	1	0	2	0	0.00221948	1	8	0.30102999	1.44210395
chr1	10282212	10356926	74715	3	0	4	0	0.00247414	1	11	0.30102999	1.52371709
chr1	10356926	10356985	60	1	0	6	0	0.01518174	1	11	0.30102999	0.9449689
chr1	10356985	10441042	84058	2	0	6	0	0.02793176	1	10	0.30102999	0.74627054
chr1	10441042	10494770	53729	2	0	6	0	0.02793176	0	10	0	0.74627054
chr1	10494770	10510868	16099	1	0	6	0	0.04875589	0	9	0	0.5732208
chr1	10510868	10510927	60	1	0	8	0	0.08923391	0	10	0	0.4167287
chr1	10510927	10521730	10804	1	0	7	0	0.05232577	0	10	0	0.56314362
chr1	10521730	10596216	74487	5	0	7	0	0.03037338	0	11	0	0.73110763
chr1	10596216	10596275	60	1	0	8	0	0.05490675	1	11	0.30102999	0.55623409
chr1	10596275	10732710	136436	4	0	7	0	0.03037338	0	11	0	0.73110763
chr1	10732710	10753078	20369	2	0	8	0	0.05490675	0	11	0	0.55623409
chr1	10753078	10827771	74694	3	0	9	0	0.09154957	0	11	0	0.41444892
chr1	10827771	10955193	127423	2	0	8	0	0.05490675	0	11	0	0.55623409
chr1	10955193	11134268	179076	8	0	9	0	0.09154957	0	11	0	0.41444892
chr1	11134268	11167237	32970	3	0	9	0	0.0565833	0	12	0	0.55190077
chr1	11167237	11204969	37733	2	0	10	0	0.09290028	0	12	0	0.41314172
chr1	11204969	11270902	65934	4	0	11	0	0.14385241	0	12	0	0.30102999
chr1	11270902	11318613	47712	2	0	10	0	0.14303407	0	11	0	0.30102999
chr1	11318613	11318672	60	1	0	11	0	0.14385241	0	12	0	0.30102999
chr1	11318672	11343490	24819	1	0	10	0	0.09290028	0	12	0	0.41314172
chr1	11343490	11362834	19345	1	0	9	0	0.0565833	0	12	0	0.55190077
chr1	11362834	11362893	60	1	0	10	0	0.09290028	0	12	0	0.41314172
chr1	11362893	11512849	149957	1	0	8	0	0.05490675	0	11	0	0.55623409
chr1	11512849	11565652	52804	1	0	7	0	0.03037338	0	11	0	0.73110763

chr1	11565652	11688767	123116	1	0	6	0	0.01518174	0	11	0	0.9449689
chr1	11688767	11688826	60	1	0	6	0	0.00778066	0	12	0	1.17038931
chr1	11688826	11854429	165604	7	0	6	0	0.02793176	0	10	0	0.74627054
chr1	11854429	11865157	10729	2	0	6	0	0.01518174	0	11	0	0.9449689
chr1	11865157	11900997	35841	1	0	6	0	0.02793176	0	10	0	0.74627054
chr1	11900997	11917526	16530	2	0	6	0	0.04875589	0	9	0	0.5732208
chr1	11917526	11917585	60	1	0	6	0	0.02793176	0	10	0	0.74627054
chr1	11917585	11980367	62783	2	0	6	0	0.04875589	0	9	0	0.5732208
chr1	11980367	12080379	100013	5	0	6	0	0.02793176	0	10	0	0.74627054
chr1	12080379	12244030	163652	3	0	4	0	0.01077081	0	9	0	1.01542894
chr1	12244030	12677466	433437	7	0	3	0	0.00378107	0	9	0	1.33111237
chr1	12677466	12710136	32671	1	0	3	0	0.01598258	0	7	0	0.84395715
chr1	12710136	12710195	60	1	0	3	0	0.01598258	1	7	0.30102999	0.84395715
chr1	12710195	12743178	32984	1	0	3	0	0.03070643	1	6	0.30102999	0.63695542
chr1	12743178	12839470	96293	2	0	3	0	0.03070643	0	6	0	0.63695542
chr1	12839470	13804405	964936	1	0	1	0	0.00478973	0	5	0	1.02643191
chr1	13804405	13804464	60	1	0	2	0	0.01053319	0	6	0	0.91219088
chr1	13804464	13940920	136457	3	0	0	0	0	0	4	0	1.26272838
chr1	13940920	13940976	57	1	0	1	0	0.00478973	1	5	0.30102999	1.02643191
chr1	13940976	14112991	172016	3	0	1	0	0.00478973	0	5	0	1.02643191
chr1	14112991	14124977	11987	2	0	4	0	0.11390336	0	5	0	0.30102999
chr1	14124977	14303202	178226	3	0	1	0	0.00478973	0	5	0	1.02643191
chr1	14303202	14303261	60	1	0	3	0	0.0079614	1	8	0.30102999	1.07548421
chr1	14303261	14408766	105506	2	0	3	0	0.0079614	0	8	0	1.07548421
chr1	14408766	14408825	60	1	0	5	0	0.04407651	0	8	0	0.58747015
chr1	14408825	14466937	58113	1	0	2	0	0.01053319	0	6	0	0.91219088
chr1	14466937	14524312	57376	2	0	2	0	0.01053319	1	6	0.30102999	0.91219088
chr1	14524312	14524371	60	1	0	5	0	0.04407651	1	8	0.30102999	0.58747015
chr1	14524371	14609757	85387	1	0	4	0	0.02074938	1	8	0.30102999	0.79906872
chr1	14609757	14609816	60	1	0	5	0	0.04407651	1	8	0.30102999	0.58747015
chr1	14609816	14808786	198971	1	0	3	0	0.0079614	1	8	0.30102999	1.07548421
chr1	14808786	14808845	60	1	1	4	0.1218695	0.01077081	1	9	0.1218695	1.01542894
chr1	14808845	14933994	125150	1	1	3	0.1218695	0.0079614	1	8	0.1218695	1.07548421
chr1	14933994	14960403	26410	2	1	4	0.1218695	0.02074938	1	8	0.1218695	0.79906872
chr1	14960403	15017526	57124	2	1	4	0.1218695	0.01077081	1	9	0.1218695	1.01542894
chr1	15017526	15017585	60	1	1	5	0.1218695	0.01320236	1	10	0.1218695	0.97390707
chr1	15017585	15150914	133330	2	1	5	0.30102999	0.02473314	0	9	0	0.76806864
chr1	15150914	15150973	60	1	1	6	0.1218695	0.04875589	1	9	0.1218695	0.5732208
chr1	15150973	15388857	237885	4	0	6	0	0.04875589	0	9	0	0.5732208
chr1	15388857	15388916	60	1	0	6	0	0.04875589	2	9	0.61140001	0.5732208
chr1	15388916	15443521	54606	1	0	5	0	0.04407651	2	8	0.61140001	0.58747015
chr1	15443521	15536961	93441	4	0	5	0	0.02473314	2	9	0.61140001	0.76806864
chr1	15536961	15692335	155375	5	0	5	0	0.01320236	2	10	0.61140001	0.97390707
chr1	15692335	15794610	102276	4	0	5	0	0.01320236	1	10	0.30102999	0.97390707
chr1	15794610	15808532	13923	2	0	6	0	0.01518174	3	11	0.93173516	0.9449689
chr1	15808532	15849390	40859	3	0	5	0	0.00666883	1	11	0.30102999	1.20557689
chr1	15849390	15900109	50720	2	0	6	0	0.01518174	3	11	0.93173516	0.9449689
chr1	15900109	15955529	55421	1	0	5	0	0.01320236	2	10	0.61140001	0.97390707
chr1	15955529	15987184	31656	1	0	4	0	0.00530919	2	10	0.61140001	1.2568129
chr1	15987184	16023360	36177	2	0	4	0	0.00530919	3	10	0.93173516	1.2568129
chr1	16023360	16023419	60	1	1	4	0.02438896	0.00530919	3	10	0.51676182	1.2568129
chr1	16023419	16135436	112018	3	1	4	0.02438896	0.01077081	3	9	0.51676182	1.01542894
chr1	16135436	16331428	195993	4	1	4	0.05404976	0.01077081	2	9	0.30102999	1.01542894
chr1	16331428	16331487	60	1	1	5	0.02438896	0.02473314	3	9	0.51676182	0.76806864
chr1	16331487	16343658	12172	1	0	5	0	0.02473314	3	9	0.93173516	0.76806864
chr1	16343658	16392682	49025	5	0	5	0	0.02473314	2	9	0.61140001	0.76806864
chr1	16392682	16397917	5236	2	0	5	0	0.01320236	2	10	0.61140001	0.97390707
chr1	16397917	16479679	81763	5	1	5	0.05404976	0.01320236	2	10	0.30102999	0.97390707
chr1	16479679	16561578	81900	2	0	5	0	0.01320236	0	10	0	0.97390707
chr1	16561578	16620956	59379	2	0	5	0	0.02473314	0	9	0	0.76806864
chr1	16620956	16786493	165538	6	0	5	0	0.01320236	1	10	0.30102999	0.97390707
chr1	16786493	16840542	54050	1	0	5	0	0.02473314	1	9	0.30102999	0.76806864
chr1	16840542	16963573	123032	1	0	5	0	0.04407651	1	8	0.30102999	0.58747015
chr1	16963573	17048732	85160	2	0	5	0	0.02473314	1	9	0.30102999	0.76806864
chr1	17048732	17231758	183027	2	0	5	0	0.01320236	1	10	0.30102999	0.97390707
chr1	17231758	17303445	71688	2	0	6	0	0.01518174	1	11	0.30102999	0.9449689
chr1	17303445	17336192	32748	2	0	5	0	0.00666883	1	11	0.30102999	1.20557689
chr1	17336192	17336249	58	1	0	6	0	0.01518174	1	11	0.30102999	0.9449689
chr1	17336249	17359608	23360	2	0	4	0	0.00247414	0	11	0	1.52371709
chr1	17359608	17371292	11685	2	0	5	0	0.00666883	0	11	0	1.20557689
chr1	17371292	17393588	22297	2	0	4	0	0.00247414	0	11	0	1.52371709
chr1	17393588	17393647	60	1	0	5	0	0.00666883	0	11	0	1.20557689
chr1	17393647	17535628	141982	3	0	5	0	0.01320236	0	10	0	0.97390707
chr1	17535628	17584968	49341	3	0	6	0	0.02793176	0	10	0	0.74627054
chr1	17584968	17610746	25779	2	0	6	0	0.01518174	0	11	0	0.9449689
chr1	17610746	17721082	110337	3	0	6	0	0.04875589	0	9	0	0.5732208
chr1	17721082	17721141	60	1	0	6	0	0.02793176	0	10	0	0.74627054
chr1	17721141	17874116	152976	4	0	5	0	0.04407651	0	8	0	0.58747015
chr1	17874116	17874175	60	1	0	6	0	0.04875589	0	9	0	0.5732208
chr1	17874175	17904529	30355	1	0	5	0	0.02473314	0	9	0	0.76806864
chr1	17904529	18041667	137139	4	0	6	0	0.01518174	0	11	0	0.9449689
chr1	18041667	18083707	42041	2	0	6	0	0.00778066	0	12	0	1.17038931
chr1	18083707	18219116	135410	4	0	6	0	0.01518174	0	11	0	0.9449689
chr1	18219116	18307240	88125	1	0	6	0	0.02793176	0	10	0	0.74627054
chr1	18307240	18400121	92882	1	0	5	0	0.01320236	0	10	0	0.97390707
chr1	18400121	18528695	128575	3	0	3	0	0.00378107	0	9	0	1.33111237
chr1	18528695	18563494	34800	2	0	4	0	0.00530919	0	10	0	1.2568129
chr1	18563494	18563553	60	1	0	6	0	0.02793176	0	10	0	0.74627054
chr1	18563553	18632233	68681	1	0	3	0	0.00378107	0	9	0	1.33111237
chr1	18632233	18704902	72670	1	0	3	0	0.0079614	0	8	0	1.07548421
chr1	18704902	18704961	60	1	0	4	0	0.01077081	0	9	0	1.01542894
chr1	18704961	18900374	195414	3	0	3	0	0.00378107	0	9	0	1.33111237
chr1	18900374	18900433	60	1	0	4	0	0.01077081	0	9	0	1.01542894
chr1	18900433	18945458	45026	1	0	2	0	9.54E-04	0	9	0	1.74076927
chr1	18945458	18957624	12167	2	0	2	0	9.54E-04	1	9	0.30102999	1.74076927
chr1	18957624	18981595	23972	1	0	2	0	0.00221948	1	8	0.30102999	1.44210395
chr1	18981595	18981654	60	1	0	3	0	0.0079614	2	8	0.61140001	1.07548421
chr1	18981654	19086642	104989	4	0	3	0	0.01598258	2	7	0.61140001	0.84395715
chr1	19086642	19179851	93210	1	0	3	0	0.03070643	2	6	0.61140001	0.63695542
chr1	19179851	19400997	221147	5	0	3	0	0.01598258	2	7	0.61140001	0.84395715
chr1	19400997	19525383	124387	5	0	4	0	0.03812622	2	7	0.61140001	0.60763643
chr1	19525383	19635750	110368	5	0	3	0	0.01598258	2	7	0.61140001	0.84395715
chr1	19635750	19728321	92572	6	0	3	0	0.0079614	2	8	0.61140001	1.07548421
chr1	19728321	19825963	97643	2	0	3	0	0.01598258	2	7	0.61140001	0.84395715
chr1	19825963	19946430	120468	3	0	4	0	0.03812622	2	7	0.61140001	0.60763643
chr1	19946430	19969873	23444	2	0	5	0	0.04407651	2	8	0.61140001	0.58747015
chr1	19969873	20003656	33784	4	0	5	0	0.02473314	2	9	0.61140001	0.76806864
chr1	20003656	20118848	115193	2	0	4	0	0.02074938	2	8	0.61140001	0.79906872
chr1	20118848	20140840	21993	3	0	5	0	0.02473314	2	9	0.61140001	0.76806864
chr1	20140840	20249390	108551	2	0	4	0	0.01077081	2	9	0.61140001	1.01542894
chr1	20249390	20306316	56927	2	0	5	0	0.02473314	2	9	0.61140001	0.76806864
chr1	20306316	20306369	54	1	0	6	0	0.04875589	2	9	0.61140001	0.5732208
chr1	20306369	20366015	59647	1	0	4	0	0.01077081	2	9	0.61140001	1.01542894
chr1	20366015	20399606	33592	2	0	5	0	0.02473314	2	9	0.61140001	0.76806864
chr1	20399606	20469195	69590	3	0	6	0	0.04875589	2	9	0.61140001	0.5732208
chr1	20469195	20469249	55	1	0	7	0	0.08584816	2	9	0.61140001	0.42015402
chr1	20469249	20514667	45419	1	0	6	0	0.04875589	2	9	0.61140001	0.5732208
chr1	20514667	20514726	60	1	0	7	0	0.08584816	2	9	0.61140001	0.42015402
chr1	20514726	20572663	57938	2	0	7	0	0.13499366	2	8	0.61140001	0.30102999
chr1	20572663	20623226	50564	1	0	7	0	0.13499366	1	8	0.30102999	0.30102999
chr1	20623226	20623285	60	1	0	8	0	0.08923391	1	10	0.30102999	0.4167287
chr1	20623285	20675639	52355	2	0	7	0	0.05232577	0	10	0	0.56314362
chr1	20675639	20740964	65326	1	0	7	0	0.13499366	0	8	0	0.30102999
chr1	20740964	20810221	69258	3	0	7	0	0.13499366	1	8	0.30102999	0.30102999

chr1	20810221	20830940	20720	1	0	6	0	0.08122616	1	8	0.30102999	0.4250187
chr1	20830940	20865498	34559	2	0	6	0	0.04875589	1	9	0.30102999	0.5732208
chr1	20865498	20916440	50943	2	0	7	0	0.08584816	1	9	0.30102999	0.42015402
chr1	20916440	20934057	17618	3	0	7	0	0.05232577	1	10	0.30102999	0.56314362
chr1	20934057	20934116	60	1	0	8	0	0.08923391	2	10	0.61140001	0.4167287
chr1	20934116	20972425	38310	1	0	8	0	0.08923391	1	10	0.30102999	0.4167287
chr1	20972425	20984262	11838	1	0	7	0	0.05232577	1	10	0.30102999	0.56314362
chr1	20984262	21034428	50167	2	0	8	0	0.08923391	1	10	0.30102999	0.4167287
chr1	21034428	21103137	68710	3	0	10	0	0.21118145	1	10	0.30102999	0.21118145
chr1	21103137	21186986	83850	2	0	7	0	0.05232577	1	10	0.30102999	0.56314362
chr1	21186986	21268359	81374	2	0	5	0	0.01320236	1	10	0.30102999	0.97390707
chr1	21268359	21427403	159045	5	1	5	0.1218695	0.01320236	1	10	0.1218695	0.97390707
chr1	21427403	21449730	22328	1	1	5	0.1218695	0.02473314	1	9	0.1218695	0.76806864
chr1	21449730	21573743	124014	3	1	4	0.1218695	0.01077081	1	9	0.1218695	1.01542894
chr1	21573743	21598433	24691	2	1	5	0.1218695	0.02473314	1	9	0.1218695	0.76806864
chr1	21598433	21658422	59990	2	1	6	0.1218695	0.04875589	1	9	0.1218695	0.5732208
chr1	21658422	21938583	280162	7	1	5	0.1218695	0.02473314	1	9	0.1218695	0.76806864
chr1	21938583	22021600	83018	2	1	4	0.1218695	0.02074938	1	8	0.1218695	0.79906872
chr1	22021600	22050635	29036	1	0	3	0	0.01598258	1	7	0.30102999	0.84395715
chr1	22050635	22078993	28359	2	0	3	0	0.0079614	1	8	0.30102999	1.07548421
chr1	22078993	22100818	21826	2	0	3	0	0.00378107	1	9	0.30102999	1.33111237
chr1	22100818	22164082	63265	1	0	2	0	0.00493743	0	7	0	1.16581773
chr1	22164082	22223053	58972	2	0	3	0	0.01598258	0	7	0	0.84395715
chr1	22223053	22250468	27416	2	0	3	0	0.01598258	1	7	0.30102999	0.84395715
chr1	22250468	22308663	58196	1	0	2	0	0.00493743	1	7	0.30102999	1.16581773
chr1	22308663	22416798	108136	4	0	1	0	8.47E-04	1	7	0.30102999	1.62048027
chr1	22416798	22416857	60	1	0	4	0	0.03812622	1	7	0.30102999	0.60763643
chr1	22416857	22461773	44917	1	0	2	0	0.02162467	1	5	0.30102999	0.68214471
chr1	22461773	22580233	118461	1	0	0	0	0	1	5	0.30102999	1.60515106
chr1	22580233	22713848	133616	2	0	2	0	0.02162467	1	5	0.30102999	0.68214471
chr1	22713848	22713907	60	1	0	3	0	0.03070643	1	6	0.30102999	0.63695542
chr1	22713907	22828159	114253	2	0	2	0	0.02162467	1	5	0.30102999	0.68214471
chr1	22828159	22891616	63458	2	0	1	0	0.00478973	1	5	0.30102999	1.02643191
chr1	22891616	22891675	60	1	0	2	0	0.01053319	1	6	0.30102999	0.91219088
chr1	22891675	22964515	72841	1	0	2	0	0.02162467	1	5	0.30102999	0.68214471
chr1	22964515	22964564	50	1	0	2	0	0.02162467	2	5	0.61140001	0.68214471
chr1	22964564	23040846	76283	5	0	2	0	0.02162467	1	5	0.30102999	0.68214471
chr1	23040846	23040905	60	1	0	2	0	0.00493743	2	7	0.61140001	1.16581773
chr1	23040905	23104094	63190	1	0	2	0	0.01053319	2	6	0.61140001	0.91219088
chr1	23104094	23172454	68361	1	0	1	0	0.00204627	2	6	0.61140001	1.31360226
chr1	23172454	23199546	27093	1	0	1	0	0.00204627	1	6	0.30102999	1.31360226
chr1	23199546	23241030	41485	2	0	2	0	0.01053319	1	6	0.30102999	0.91219088
chr1	23241030	23241089	60	1	0	3	0	0.03070643	1	6	0.30102999	0.63695542
chr1	23241089	23356988	115900	2	0	1	0	0.00478973	1	5	0.30102999	1.02643191
chr1	23356988	23419852	62865	4	0	1	0	0.00204627	1	6	0.30102999	1.31360226
chr1	23419852	23455790	35939	2	0	1	0	0.00204627	2	6	0.61140001	1.31360226
chr1	23455790	23481411	25622	1	0	1	0	0.00204627	1	6	0.30102999	1.31360226
chr1	23481411	23481470	60	1	0	2	0	0.01053319	1	6	0.30102999	0.91219088
chr1	23481470	23519807	38338	1	0	1	0	0.00478973	1	5	0.30102999	1.02643191
chr1	23519807	23607991	88185	2	0	1	0	0.00478973	2	5	0.61140001	1.02643191
chr1	23607991	23660159	52169	4	0	1	0	0.00204627	2	6	0.61140001	1.31360226
chr1	23660159	23689659	29501	2	0	3	0	0.03070643	2	6	0.61140001	0.63695542
chr1	23689659	23689715	57	1	0	4	0	0.06713722	2	6	0.61140001	0.44141547
chr1	23689715	23852626	162912	4	0	4	0	0.06713722	1	6	0.30102999	0.44141547
chr1	23852626	23852685	60	1	0	4	0	0.06713722	2	6	0.61140001	0.44141547
chr1	23852685	23885141	32457	2	0	3	0	0.03070643	1	6	0.30102999	0.63695542
chr1	23885141	23886267	1127	2	0	3	0	0.03070643	2	6	0.61140001	0.63695542
chr1	23886267	23929208	42942	1	0	3	0	0.05670724	1	5	0.30102999	0.45545077
chr1	23929208	23966799	37592	1	0	3	0	0.10122019	1	4	0.30102999	0.30102999
chr1	23966799	23966858	60	1	0	3	0	0.10122019	2	4	0.61140001	0.30102999
chr1	23966858	24020118	53261	1	0	3	0	0.17593012	2	3	0.61140001	0.17593012
chr1	24020118	24082303	62186	4	0	3	0	0.17593012	3	3	0.93173516	0.17593012
chr1	24082303	24108598	26296	2	1	4	0.02438896	0.18734596	3	4	0.51676182	0.18734596
chr1	24108598	24233533	124936	3	1	3	0.02438896	0.10122019	3	4	0.51676182	0.30102999
chr1	24233533	24384595	151063	1	1	3	0.05404976	0.10122019	2	4	0.30102999	0.30102999
chr1	24384595	24411975	27381	3	1	3	0.02438896	0.05670724	3	5	0.51676182	0.45545077
chr1	24411975	24456460	44486	1	1	3	0.05404976	0.10122019	2	4	0.30102999	0.30102999
chr1	24456460	24509558	53099	3	1	3	0.05404976	0.05670724	2	5	0.30102999	0.45545077
chr1	24509558	24641638	132081	2	1	3	0.30102999	0.05670724	0	5	0	0.45545077
chr1	24641638	24641697	60	1	1	4	0.30102999	0.11390336	0	5	0	0.30102999
chr1	24641697	24684396	42700	2	1	3	0.30102999	0.10122019	0	4	0	0.30102999
chr1	24684396	24684451	56	1	1	4	0.1218695	0.18734596	1	4	0.1218695	0.18734596
chr1	24684451	24740328	55878	2	1	3	0.30102999	0.10122019	0	4	0	0.30102999
chr1	24740328	24771675	31348	1	1	2	0.30102999	0.0429175	0	4	0	0.47744371
chr1	24771675	24785922	14248	2	1	3	0.30102999	0.05670724	0	5	0	0.45545077
chr1	24785922	24840969	55048	2	1	4	0.1218695	0.06713722	1	6	0.1218695	0.44141547
chr1	24840969	24859795	18827	1	1	4	0.30102999	0.06713722	0	6	0	0.44141547
chr1	24859795	24936357	76563	1	1	3	0.30102999	0.05670724	0	5	0	0.45545077
chr1	24936357	24996383	60027	2	1	3	0.30102999	0.10122019	0	4	0	0.30102999
chr1	24996383	24996442	60	1	1	3	0.30102999	0.03070643	0	6	0	0.63695542
chr1	24996442	25119735	123294	2	1	1	0.30102999	0.00478973	0	5	0	1.02643191
chr1	25119735	25235732	115998	3	1	2	0.30102999	0.02162467	0	5	0	0.68214471
chr1	25235732	25235791	60	1	1	3	0.30102999	0.0079614	0	8	0	1.07548421
chr1	25235791	25557914	322124	4	1	3	0.30102999	0.01598258	0	7	0	0.84395715
chr1	25557914	26211420	653507	19	1	3	0.30102999	0.0079614	0	8	0	1.07548421
chr1	26211420	26211479	60	1	1	4	0.30102999	0.02074938	0	8	0	0.79906872
chr1	26211479	26226749	15271	1	1	3	0.30102999	0.0079614	0	8	0	1.07548421
chr1	26226749	26259826	33078	4	1	4	0.30102999	0.02074938	0	8	0	0.79906872
chr1	26259826	26259885	60	1	1	5	0.30102999	0.04407651	0	8	0	0.58747015
chr1	26259885	26358405	98521	2	1	4	0.30102999	0.03812622	0	7	0	0.60763643
chr1	26358405	26367040	8636	2	1	4	0.30102999	0.02074938	0	8	0	0.79906872
chr1	26367040	26656547	289508	10	1	5	0.30102999	0.04407651	0	8	0	0.58747015
chr1	26656547	26690641	34095	2	1	5	0.30102999	0.02473314	0	9	0	0.76806864
chr1	26690641	26750642	60002	1	1	5	0.30102999	0.04407651	0	8	0	0.58747015
chr1	26750642	26750701	60	1	1	5	0.30102999	0.02473314	0	9	0	0.76806864
chr1	26750701	26759521	8821	1	1	5	0.30102999	0.04407651	0	8	0	0.58747015
chr1	26759521	26796023	36503	2	1	5	0.30102999	0.02473314	0	9	0	0.76806864
chr1	26796023	26800716	4694	2	1	5	0.30102999	0.00666883	0	11	0	1.20557689
chr1	26800716	26941216	140501	4	1	5	0.30102999	0.01320236	0	10	0	0.97390707
chr1	26941216	27123177	181962	4	1	5	0.30102999	0.04407651	0	8	0	0.58747015
chr1	27123177	27190888	67712	3	1	5	0.30102999	0.02473314	0	9	0	0.76806864
chr1	27190888	27251731	60844	7	1	6	0.30102999	0.02793176	0	10	0	0.74627054
chr1	27251731	27336827	85097	2	1	5	0.30102999	0.01320236	0	10	0	0.97390707
chr1	27336827	27562742	225916	7	1	6	0.30102999	0.02793176	0	10	0	0.74627054
chr1	27562742	27623380	60639	2	1	6	0.30102999	0.08122616	0	8	0	0.4250187
chr1	27623380	27623432	53	1	1	6	0.30102999	0.02793176	0	10	0	0.74627054
chr1	27623432	27673275	49844	2	1	5	0.30102999	0.04407651	0	8	0	0.58747015
chr1	27673275	27685374	12100	2	1	6	0.30102999	0.02793176	0	10	0	0.74627054
chr1	27685374	27872811	187438	6	1	5	0.30102999	0.02473314	0	9	0	0.76806864
chr1	27872811	27913034	40224	2	1	5	0.1218695	0.02473314	1	9	0.1218695	0.76806864
chr1	27913034	27952963	39930	3	1	6	0.1218695	0.04875589	1	9	0.1218695	0.5732208
chr1	27952963	27997584	44622	2	1	6	0.1218695	0.08122616	1	8	0.1218695	0.4250187
chr1	27997584	28119663	122080	4	1	6	0.1218695	0.04875589	1	9	0.1218695	0.5732208
chr1	28119663	28119722	60	1	1	7	0.1218695	0.08584816	1	9	0.1218695	0.42015402
chr1	28119722	28142214	22493	1	1	6	0.1218695	0.04875589	1	9	0.1218695	0.5732208
chr1	28142214	28212975	70762	3	1	5	0.1218695	0.02473314	1	9	0.1218695	0.76806864
chr1	28212975	28233626	20652	2	1	5	0.30102999	0.02473314	0	9	0	0.76806864
chr1	28233626	28240509	6884	1	1	4	0.30102999	0.01077081	0	9	0	1.01542894
chr1	28240509	28374871	134363	6	1	4	0.1218695	0.01077081	1	9	0.1218695	1.01542894
chr1	28374871	28420867	45997	2	1	5	0.1218695	0.02473314	1	9	0.1218695	0.76806864
chr1	28420867	28476241	55375	2	1	6	0.1218695	0.04875589	1	9	0.1218695	0.5732208
chr1	28476241	28476296	56	1	1	7	0.1218695	0.08584816	1	9	0.1218695	0.42015402
chr1	28476296	28564583	88288	3	1	7	0.1218695	0.13499366	1	8	0.1218695	0.30102999
chr1	28564583	28597020	32438	1	1	6	0.1218695	0.08122616	1	8	0.1218695	0.4250187

chr1	28597020	28655666	58647	1	1	6	0.30102999	0.08122616	0	8	0	0.4250187
chr1	28655666	28662408	6743	2	1	7	0.30102999	0.05232577	0	10	0	0.56314362
chr1	28662408	28713293	50886	1	1	6	0.30102999	0.04875589	0	9	0	0.5732208
chr1	28713293	28815665	102373	2	1	5	0.30102999	0.02473314	0	9	0	0.76806864
chr1	28815665	28865136	49472	4	1	5	0.30102999	0.01320236	0	10	0	0.97390707
chr1	28865136	28947613	82478	4	1	4	0.30102999	0.00530919	0	10	0	1.2568129
chr1	28947613	28968884	21272	2	1	5	0.30102999	0.01320236	0	10	0	0.97390707
chr1	28968884	29089758	120875	4	1	5	0.30102999	0.02473314	0	9	0	0.76806864
chr1	29089758	29205448	115691	2	1	3	0.30102999	0.00378107	0	9	0	1.33111237
chr1	29205448	29205507	60	1	1	4	0.30102999	0.01077081	0	9	0	1.01542894
chr1	29205507	29277297	71791	2	1	3	0.30102999	0.00378107	0	9	0	1.33111237
chr1	29277297	29361225	83929	2	1	2	0.30102999	0.00221948	0	8	0	1.44210395
chr1	29361225	29386909	25685	1	1	1	0.30102999	3.39E-04	0	8	0	1.94674965
chr1	29386909	29386968	60	1	1	2	0.30102999	0.00221948	0	8	0	1.44210395
chr1	29386968	29443550	56583	2	1	2	0.30102999	0.00493743	0	7	0	1.16581773
chr1	29443550	29527822	84273	3	1	2	0.30102999	9.54E-04	0	9	0	1.74076927
chr1	29527822	29653134	125313	4	1	1	0.30102999	3.39E-04	0	8	0	1.94674965
chr1	29653134	29859635	206502	2	1	1	0.30102999	8.47E-04	0	7	0	1.62048027
chr1	29859635	30447087	587453	3	1	0	0.30102999	0	0	5	0	1.60515106
chr1	30447087	30630199	183113	2	1	1	0.30102999	0.00204627	0	6	0	1.31360226
chr1	30630199	30630258	60	1	1	1	0.30102999	8.47E-04	0	7	0	1.62048027
chr1	30630258	30717129	86872	1	1	1	0.30102999	0.00204627	0	6	0	1.31360226
chr1	30717129	31047671	330543	2	1	1	0.30102999	0.00478973	0	5	0	1.02643191
chr1	31047671	31184216	136546	2	1	1	0.30102999	0.00204627	0	6	0	1.31360226
chr1	31184216	31226876	42661	3	1	1	0.1218695	0.00204627	1	6	0.1218695	1.31360226
chr1	31226876	31315434	88559	2	1	1	0.05404976	8.47E-04	2	7	0.30102999	1.62048027
chr1	31315434	31350724	35291	2	1	1	0.05404976	3.39E-04	2	8	0.30102999	1.94674965
chr1	31350724	31467875	117152	2	1	1	0.1218695	3.39E-04	1	8	0.1218695	1.94674965
chr1	31467875	31660110	192236	6	1	1	0.1218695	1.30E-04	1	9	0.1218695	2.29264207
chr1	31660110	31710763	50654	1	1	1	0.1218695	3.39E-04	1	8	0.1218695	1.94674965
chr1	31710763	31710822	60	1	1	1	0.05404976	3.39E-04	2	8	0.30102999	1.94674965
chr1	31710822	31827548	116727	2	1	1	0.1218695	3.39E-04	1	8	0.1218695	1.94674965
chr1	31827548	31897250	69703	2	1	1	0.05404976	3.39E-04	2	8	0.30102999	1.94674965
chr1	31897250	31966338	69089	5	1	2	0.05404976	0.00221948	2	8	0.30102999	1.44210395
chr1	31966338	31966397	60	1	1	3	0.05404976	0.0079614	2	8	0.30102999	1.07548421
chr1	31966397	32200305	233909	7	1	2	0.05404976	0.00221948	2	8	0.30102999	1.44210395
chr1	32200305	32263127	62823	2	1	2	0.02438896	0.00221948	3	8	0.51676182	1.44210395
chr1	32263127	32263184	58	1	1	2	0.01091641	0.00221948	4	8	0.76005302	1.44210395
chr1	32263184	32278079	14896	1	1	2	0.05404976	0.00221948	2	8	0.30102999	1.44210395
chr1	32278079	32362921	84843	2	1	3	0.05404976	0.0079614	2	8	0.30102999	1.07548421
chr1	32362921	32489401	126481	5	1	4	0.02438896	0.02074938	3	8	0.51676182	0.79906872
chr1	32489401	32504134	14734	1	1	4	0.05404976	0.02074938	2	8	0.30102999	0.79906872
chr1	32504134	32697316	193183	14	1	4	0.02438896	0.02074938	3	8	0.51676182	0.79906872
chr1	32697316	32713551	16236	2	1	4	0.02438896	0.03812622	3	7	0.51676182	0.60763643
chr1	32713551	32936586	223036	10	1	4	0.02438896	0.02074938	3	8	0.51676182	0.79906872
chr1	32936586	32985991	49406	2	1	4	0.02438896	0.03812622	3	7	0.51676182	0.60763643
chr1	32985991	33065896	79906	4	1	4	0.02438896	0.02074938	3	8	0.51676182	0.79906872
chr1	33065896	33093081	27186	1	1	4	0.02438896	0.03812622	3	7	0.51676182	0.60763643
chr1	33093081	33146307	53227	4	1	4	0.01091641	0.03812622	4	7	0.76005302	0.60763643
chr1	33146307	33276552	130246	3	1	4	0.02438896	0.03812622	3	7	0.51676182	0.60763643
chr1	33276552	33364705	88154	6	1	3	0.02438896	0.01598258	3	7	0.51676182	0.84395715
chr1	33364705	33478678	113974	3	1	2	0.02438896	0.00493743	3	7	0.51676182	1.16581773
chr1	33478678	33499958	21281	1	1	2	0.05404976	0.00493743	2	7	0.30102999	1.16581773
chr1	33499958	33550093	50136	1	1	2	0.05404976	0.01053319	2	6	0.30102999	0.91219088
chr1	33550093	33550151	59	1	1	2	0.01091641	0.01053319	4	6	0.76005302	0.91219088
chr1	33550151	33576903	26753	1	1	2	0.02438896	0.01053319	3	6	0.51676182	0.91219088
chr1	33576903	33576962	60	1	1	2	0.02438896	0.00493743	3	7	0.51676182	1.16581773
chr1	33576962	33607663	30702	1	1	2	0.02438896	0.01053319	3	6	0.51676182	0.91219088
chr1	33607663	33637215	29553	1	1	2	0.05404976	0.01053319	2	6	0.30102999	0.91219088
chr1	33637215	33637274	60	1	1	2	0.05404976	0.00493743	2	7	0.30102999	1.16581773
chr1	33637274	34024160	386887	10	1	2	0.05404976	0.01053319	2	6	0.30102999	0.91219088
chr1	34024160	34227784	203625	5	1	2	0.05404976	0.02162467	2	5	0.30102999	0.68214471
chr1	34227784	34329841	102058	2	1	1	0.05404976	0.01091641	2	4	0.30102999	0.76005302
chr1	34329841	34330045	205	2	2	1	0.1575501	0.01091641	2	4	0.1575501	0.76005302
chr1	34330045	34381948	51904	1	2	1	0.1575501	0.02438896	2	3	0.1575501	0.51676182
chr1	34381948	34590598	208651	5	2	1	0.08289318	0.02438896	3	3	0.30102999	0.51676182
chr1	34590598	34635582	44985	2	2	1	0.1575501	0.02438896	2	3	0.1575501	0.51676182
chr1	34635582	34693419	57838	3	2	1	0.08289318	0.02438896	3	3	0.30102999	0.51676182
chr1	34693419	34833515	140097	1	1	1	0.02438896	0.02438896	3	3	0.51676182	0.51676182
chr1	34833515	34966903	133389	1	1	1	0.05404976	0.02438896	2	3	0.30102999	0.51676182
chr1	34966903	35055063	88161	2	2	1	0.1575501	0.02438896	2	3	0.1575501	0.51676182
chr1	35055063	35132942	77880	2	2	1	0.08289318	0.02438896	3	3	0.30102999	0.51676182
chr1	35132942	35133001	60	1	2	1	0.08289318	0.01091641	3	4	0.30102999	0.76005302
chr1	35133001	35191472	58472	1	2	0	0.08289318	0	3	4	0.30102999	1.26272838
chr1	35191472	35397728	206257	5	2	0	0.0429175	0	4	4	0.47744371	1.26272838
chr1	35397728	35397787	60	1	2	1	0.02162467	0.01091641	5	4	0.68214471	0.76005302
chr1	35397787	35496289	98503	3	2	1	0.0429175	0.01091641	4	4	0.47744371	0.76005302
chr1	35496289	35570289	74001	2	1	1	0.01091641	0.02438896	4	3	0.76005302	0.51676182
chr1	35570289	35582023	11735	2	2	1	0.02162467	0.01091641	5	4	0.68214471	0.76005302
chr1	35582023	35652402	70380	1	2	0	0.02162467	0	5	3	0.68214471	0.93173516
chr1	35652402	35741534	89133	3	2	0	0.02162467	0	5	4	0.68214471	1.26272838
chr1	35741534	35806757	65224	1	2	0	0.0429175	0	4	4	0.47744371	1.26272838
chr1	35806757	35836194	29438	2	2	0	0.0429175	0	4	5	0.47744371	1.60515106
chr1	35836194	35836253	60	1	2	0	0.02162467	0	5	5	0.68214471	1.60515106
chr1	35836253	35881147	44895	1	2	0	0.0429175	0	4	5	0.47744371	1.60515106
chr1	35881147	35925920	44774	2	2	1	0.0429175	0.00478973	4	5	0.47744371	1.02643191
chr1	35925920	35972436	46517	2	2	2	0.02162467	0.02162467	5	5	0.68214471	0.68214471
chr1	35972436	36067956	95521	6	2	2	0.01053319	0.02162467	6	5	0.91219088	0.68214471
chr1	36067956	36154746	86791	3	2	2	0.00493743	0.02162467	7	5	1.16581773	0.68214471
chr1	36154746	36205014	50269	3	2	2	0.01053319	0.02162467	6	5	0.91219088	0.68214471
chr1	36205014	36234382	29369	2	2	2	0.00493743	0.02162467	7	5	1.16581773	0.68214471
chr1	36234382	36375539	141158	4	2	2	0.02162467	0.02162467	5	5	0.68214471	0.68214471
chr1	36375539	36432527	56989	2	2	1	0.02162467	0.00478973	5	5	0.68214471	1.02643191
chr1	36432527	36432586	60	1	2	1	0.01053319	0.00478973	6	5	0.91219088	1.02643191
chr1	36432586	36602690	170105	6	2	1	0.02162467	0.00478973	5	5	0.68214471	1.02643191
chr1	36602690	36859105	256416	9	2	1	0.0429175	0.00478973	4	5	0.47744371	1.02643191
chr1	36859105	36909594	50490	2	1	1	0.01091641	0.00478973	4	5	0.76005302	1.02643191
chr1	36909594	36909648	55	1	2	1	0.02162467	0.00478973	5	5	0.68214471	1.02643191
chr1	36909648	36947946	38299	3	1	1	0.00478973	0.00478973	5	5	1.02643191	1.02643191
chr1	36947946	36948005	60	1	2	1	0.02162467	0.00478973	5	5	0.68214471	1.02643191
chr1	36948005	37075952	127948	1	2	1	0.0429175	0.00478973	4	5	0.47744371	1.02643191
chr1	37075952	37176842	100891	1	1	1	0.01091641	0.00478973	4	5	0.76005302	1.02643191
chr1	37176842	37270049	93208	2	1	1	0.02438896	0.00478973	3	5	0.51676182	1.02643191
chr1	37270049	37506451	236403	7	1	1	0.01091641	0.00478973	4	5	0.76005302	1.02643191
chr1	37506451	37606156	99706	1	1	1	0.01091641	0.01091641	4	4	0.76005302	0.76005302
chr1	37606156	37746925	140770	2	1	1	0.02438896	0.01091641	3	4	0.51676182	0.76005302
chr1	37746925	38261360	514436	15	1	1	0.05404976	0.02438896	2	3	0.30102999	0.51676182
chr1	38261360	38390953	129594	3	1	1	0.05404976	0.05404976	2	2	0.30102999	0.30102999
chr1	38390953	38591548	200596	7	1	1	0.02438896	0.05404976	3	2	0.51676182	0.30102999
chr1	38591548	38688609	97062	1	1	1	0.02438896	0.1218695	3	1	0.51676182	0.1218695
chr1	38688609	38688668	60	1	2	1	0.0429175	0.1218695	4	1	0.47744371	0.1218695
chr1	38688668	38831181	142514	1	2	1	0.0429175	0.30102999	4	0	0.47744371	0
chr1	38831181	39031717	200537	1	1	1	0.02438896	0.30102999	3	0	0.51676182	0
chr1	39031717	39179929	148213	1	1	1	0.05404976	0.30102999	2	0	0.30102999	0
chr1	39179929	39299969	120041	2	1	1	0.00478973	0.1218695	5	1	1.02643191	0.1218695
chr1	39299969	39304105	4137	2	2	1	0.02162467	0.1218695	5	1	0.68214471	0.1218695
chr1	39304105	39403410	99306	7	2	1	0.00493743	0.1218695	7	1	1.16581773	0.1218695
chr1	39403410	39460130	56721	1	2	1	0.02162467	0.1218695	5	1	0.68214471	0.1218695
chr1	39460130	39556236	96107	2	1	1	0.00478973	0.1218695	5	1	1.02643191	0.1218695
chr1	39556236	39604843	48608	2	1	1	0.00478973	0.02438896	5	3	1.02643191	0.51676182
chr1	39604843	39742171	137329	4	1	2	0.00204627	0.08289318	6	3	1.31360226	0.30102999
chr1	39742171	39742230	60	1	1	2	8.47E-04	0.0429175	7	4	1.62048027	0.47744371
chr1	39742230	39898728	156499	3	1	2	0.00204627	0.0429175	6	4	1.31360226	0.47744371
chr1	39898728	39898787	60	1	2	2	0.00493743	0.0429175	7	4	1.16581773	0.47744371

chr1	39898787	40033403	134617	2	2	2	0.01053319	0.0429175	6	4	0.91219088	0.47744371
chr1	40033403	40033462	60	1	2	2	0.00493743	0.0429175	7	4	1.16581773	0.47744371
chr1	40033462	40128786	95325	2	2	2	0.01053319	0.0429175	6	4	0.91219088	0.47744371
chr1	40128786	40224348	95563	5	2	2	0.00493743	0.0429175	7	4	1.16581773	0.47744371
chr1	40224348	40256963	32616	2	2	2	0.00493743	0.08289318	7	3	1.16581773	0.30102999
chr1	40256963	40313633	56671	2	2	1	0.00493743	0.02438896	7	3	1.16581773	0.51676182
chr1	40313633	40313692	60	1	2	2	0.00493743	0.08289318	7	3	1.16581773	0.30102999
chr1	40313692	40421743	108052	4	2	1	0.01053319	0.02438896	6	3	0.91219088	0.51676182
chr1	40421743	40509662	87920	3	2	1	0.01053319	0.01091641	6	4	0.91219088	0.76005302
chr1	40509662	40538616	28955	3	2	1	0.00221948	0.01091641	8	4	1.44210395	0.76005302
chr1	40538616	40831159	292544	7	2	1	0.01053319	0.01091641	6	4	0.91219088	0.76005302
chr1	40831159	41013737	182579	4	2	1	0.01053319	0.02438896	6	3	0.91219088	0.51676182
chr1	41013737	41013796	60	1	2	1	0.01053319	0.01091641	6	4	0.91219088	0.76005302
chr1	41013796	41088145	74350	2	2	1	0.01053319	0.02438896	6	3	0.91219088	0.51676182
chr1	41088145	41126424	38280	2	2	1	0.01053319	0.01091641	6	4	0.91219088	0.76005302
chr1	41126424	41234054	107631	4	2	1	0.01053319	0.02438896	6	3	0.91219088	0.51676182
chr1	41234054	41234113	60	1	2	2	0.01053319	0.08289318	6	3	0.91219088	0.30102999
chr1	41234113	41292272	58160	1	2	1	0.02162467	0.02438896	5	3	0.68214471	0.51676182
chr1	41292272	41292331	60	1	2	1	0.02162467	0.01091641	5	4	0.68214471	0.76005302
chr1	41292331	41326707	34377	1	2	1	0.02162467	0.02438896	5	3	0.68214471	0.51676182
chr1	41326707	41366489	39783	2	2	1	0.01053319	0.02438896	6	3	0.91219088	0.51676182
chr1	41366489	41484999	118511	4	2	0	0.01053319	0	6	3	0.91219088	0.93173516
chr1	41484999	41485058	60	1	2	1	0.01053319	0.02438896	6	3	0.91219088	0.51676182
chr1	41485058	41623469	138412	3	2	0	0.02162467	0	5	3	0.68214471	0.93173516
chr1	41623469	41706567	83099	3	2	0	0.00493743	0	7	3	1.16581773	0.93173516
chr1	41706567	41944420	237854	3	1	0	0.00204627	0	6	3	1.31360226	0.93173516
chr1	41944420	41975850	31431	3	1	0	8.47E-04	0	7	3	1.62048027	0.93173516
chr1	41975850	41975909	60	1	2	0	0.00221948	0	8	3	1.44210395	0.93173516
chr1	41975909	42007004	31096	1	2	0	0.00493743	0	7	3	1.16581773	0.93173516
chr1	42007004	42105619	98616	2	1	0	8.47E-04	0	7	3	1.62048027	0.93173516
chr1	42105619	42146021	40403	2	1	1	8.47E-04	0.02438896	7	3	1.62048027	0.51676182
chr1	42146021	42185100	39080	1	1	0	8.47E-04	0	7	2	1.62048027	0.61140001
chr1	42185100	42217798	32699	1	1	0	0.00478973	0	5	2	1.02643191	0.61140001
chr1	42217798	42629541	411744	8	1	1	0.00478973	0.05404976	5	2	1.02643191	0.30102999
chr1	42629541	42653193	23653	2	1	1	0.00478973	0.02438896	5	3	1.02643191	0.51676182
chr1	42653193	42744110	90918	2	1	1	0.01091641	0.05404976	4	2	0.76005302	0.30102999
chr1	42744110	42836786	92677	3	1	1	0.01091641	0.01091641	4	4	0.76005302	0.76005302
chr1	42836786	42865223	28438	2	1	2	0.01091641	0.0429175	4	4	0.76005302	0.47744371
chr1	42865223	42865275	53	1	1	2	0.00478973	0.0429175	5	4	1.02643191	0.47744371
chr1	42865275	42925635	60361	2	1	2	0.01091641	0.08289318	4	3	0.76005302	0.30102999
chr1	42925635	43200719	275085	9	1	2	8.47E-04	0.08289318	7	3	1.62048027	0.30102999
chr1	43200719	43295738	95020	4	1	1	0.00204627	0.02438896	6	3	1.31360226	0.51676182
chr1	43295738	43302859	7122	2	1	2	0.00204627	0.08289318	6	3	1.31360226	0.30102999
chr1	43302859	43530613	227755	3	1	1	0.00204627	0.02438896	6	3	1.31360226	0.51676182
chr1	43530613	43530672	60	1	1	1	0.00204627	0.01091641	6	4	1.31360226	0.76005302
chr1	43530672	43647378	116707	2	1	1	0.00478973	0.01091641	5	4	1.02643191	0.76005302
chr1	43647378	43708213	60836	1	1	1	0.00478973	0.02438896	5	3	1.02643191	0.51676182
chr1	43708213	43998723	290511	18	1	1	0.00204627	0.02438896	6	3	1.31360226	0.51676182
chr1	43998723	44201881	203159	5	1	1	0.01091641	0.02438896	4	3	0.76005302	0.51676182
chr1	44201881	44201940	60	1	1	1	0.00478973	0.02438896	5	3	1.02643191	0.51676182
chr1	44201940	44361728	159789	4	1	1	0.01091641	0.02438896	4	3	0.76005302	0.51676182
chr1	44361728	44615487	253760	9	1	1	0.01091641	0.01091641	4	4	0.76005302	0.76005302
chr1	44615487	44784377	168891	4	1	1	0.00478973	0.01091641	5	4	1.02643191	0.76005302
chr1	44784377	44784436	60	1	1	1	8.47E-04	0.01091641	7	4	1.62048027	0.76005302
chr1	44784436	44859759	75324	1	1	1	0.00204627	0.02438896	6	3	1.31360226	0.51676182
chr1	44859759	44859818	60	1	1	1	8.47E-04	0.02438896	7	3	1.62048027	0.51676182
chr1	44859818	44952254	92437	2	1	1	0.00204627	0.02438896	6	3	1.31360226	0.51676182
chr1	44952254	45050029	97776	3	1	2	0.00204627	0.08289318	6	3	1.31360226	0.30102999
chr1	45050029	45050088	60	1	1	2	8.47E-04	0.08289318	7	3	1.62048027	0.30102999
chr1	45050088	45179509	129422	4	1	2	0.00204627	0.08289318	6	3	1.31360226	0.30102999
chr1	45179509	45242144	62636	4	1	2	8.47E-04	0.08289318	7	3	1.62048027	0.30102999
chr1	45242144	45269860	27717	2	1	2	0.00204627	0.08289318	6	3	1.31360226	0.30102999
chr1	45269860	45297640	27781	2	1	2	8.47E-04	0.08289318	7	3	1.62048027	0.30102999
chr1	45297640	45303862	6223	2	2	2	0.00493743	0.08289318	7	3	1.16581773	0.30102999
chr1	45303862	45376102	72241	3	2	2	0.00493743	0.1575501	7	2	1.16581773	0.1575501
chr1	45376102	45376161	60	1	2	2	0.00493743	0.08289318	7	3	1.16581773	0.30102999
chr1	45376161	45444945	68785	1	2	2	0.00493743	0.1575501	7	2	1.16581773	0.1575501
chr1	45444945	45481223	36279	5	2	2	0.00221948	0.1575501	8	2	1.44210395	0.1575501
chr1	45481223	45481282	60	1	2	2	0.00221948	0.08289318	8	3	1.44210395	0.30102999
chr1	45481282	45562014	80733	2	2	2	0.00221948	0.1575501	8	2	1.44210395	0.1575501
chr1	45562014	45562073	60	1	2	2	9.54E-04	0.1575501	9	2	1.74076927	0.1575501
chr1	45562073	45793780	231708	3	1	2	1.30E-04	0.1575501	9	2	2.29264207	0.1575501
chr1	45793780	45923445	129666	8	1	2	1.30E-04	0.08289318	9	3	2.29264207	0.30102999
chr1	45923445	46017520	94076	4	2	2	9.54E-04	0.08289318	9	3	1.74076927	0.30102999
chr1	46017520	46106046	88527	2	2	2	0.00221948	0.08289318	8	3	1.44210395	0.30102999
chr1	46106046	46165590	59545	1	2	2	0.00493743	0.08289318	7	3	1.16581773	0.30102999
chr1	46165590	46165889	300	2	2	2	0.00493743	0.0429175	7	4	1.16581773	0.47744371
chr1	46165889	46322426	156538	4	1	2	8.47E-04	0.0429175	7	4	1.62048027	0.47744371
chr1	46322426	46650642	328217	8	1	2	8.47E-04	0.08289318	7	3	1.62048027	0.30102999
chr1	46650642	46671143	20502	3	1	2	8.47E-04	0.0429175	7	4	1.62048027	0.47744371
chr1	46671143	46764022	92880	5	1	2	8.47E-04	0.08289318	7	3	1.62048027	0.30102999
chr1	46764022	46811413	47392	2	1	1	8.47E-04	0.02438896	7	3	1.62048027	0.51676182
chr1	46811413	46864607	53195	2	1	2	8.47E-04	0.08289318	7	3	1.62048027	0.30102999
chr1	46864607	46910640	46034	1	1	1	8.47E-04	0.02438896	7	3	1.62048027	0.51676182
chr1	46910640	47119471	208832	9	1	0	8.47E-04	0	7	3	1.62048027	0.93173516
chr1	47119471	47143878	24408	2	1	1	8.47E-04	0.02438896	7	3	1.62048027	0.51676182
chr1	47143878	47224171	80294	2	1	1	0.00204627	0.02438896	6	3	1.31360226	0.51676182
chr1	47224171	47326538	102368	3	1	1	0.00478973	0.02438896	5	3	1.02643191	0.51676182
chr1	47326538	47630162	303625	6	1	1	0.01091641	0.02438896	4	3	0.76005302	0.51676182
chr1	47630162	47653590	23429	2	1	1	0.00478973	0.02438896	5	3	1.02643191	0.51676182
chr1	47653590	47728629	75040	4	1	0	0.01091641	0	4	3	0.76005302	0.93173516
chr1	47728629	47767909	39281	1	1	0	0.01091641	0	4	2	0.76005302	0.61140001
chr1	47767909	47767968	60	1	1	0	0.00478973	0	5	2	1.02643191	0.61140001
chr1	47767968	47937232	169265	4	1	0	0.01091641	0	4	2	0.76005302	0.61140001
chr1	47937232	47937291	60	1	1	0	8.47E-04	0	7	2	1.62048027	0.61140001
chr1	47937291	48016426	79136	2	1	0	0.00204627	0	6	2	1.31360226	0.61140001
chr1	48016426	48016485	60	1	1	0	8.47E-04	0	7	2	1.62048027	0.61140001
chr1	48016485	48263448	246964	2	1	0	0.00204627	0	6	2	1.31360226	0.61140001
chr1	48263448	48322470	59023	2	1	0	8.47E-04	0	7	2	1.62048027	0.61140001
chr1	48322470	48483977	161508	2	1	0	0.00204627	0	6	2	1.31360226	0.61140001
chr1	48483977	48484036	60	1	2	0	0.01053319	0	6	2	0.91219088	0.61140001
chr1	48484036	48559158	75123	1	1	0	0.00478973	0	5	2	1.02643191	0.61140001
chr1	48559158	48648062	88905	1	1	0	0.01091641	0	4	2	0.76005302	0.61140001
chr1	48648062	48648121	60	1	2	0	0.02162467	0	5	2	0.68214471	0.61140001
chr1	48648121	48709839	61719	1	1	0	0.02438896	0	3	2	0.51676182	0.61140001
chr1	48709839	48763881	54043	1	1	0	0.05404976	0	2	2	0.30102999	0.61140001
chr1	48763881	48825401	61521	2	1	0	0.02438896	0	3	2	0.51676182	0.61140001
chr1	48825401	48825453	53	1	1	0	0.02438896	0	3	3	0.51676182	0.93173516
chr1	48825453	48891864	66412	2	1	0	0.05404976	0	2	2	0.30102999	0.61140001
chr1	48891864	48918640	26777	2	2	0	0.1575501	0	2	2	0.1575501	0.61140001
chr1	48918640	48918698	59	1	3	0	0.17593012	0	3	3	0.17593012	0.93173516
chr1	48918698	49239706	321009	7	2	0	0.08289318	0	3	3	0.30102999	0.93173516
chr1	49239706	49360593	120888	3	1	0	0.02438896	0	3	3	0.51676182	0.93173516
chr1	49360593	49360652	60	1	1	0	0.01091641	0	4	3	0.76005302	0.93173516
chr1	49360652	49512100	151449	3	1	0	0.02438896	0	3	3	0.51676182	0.93173516
chr1	49512100	49631631	119532	3	1	1	0.02438896	0.02438896	3	3	0.51676182	0.51676182
chr1	49631631	49685337	53707	1	1	1	0.05404976	0.02438896	2	3	0.30102999	0.51676182
chr1	49685337	49759992	74656	1	1	0	0.05404976	0	2	3	0.30102999	0.93173516
chr1	49759992	49821553	61562	2	1	0	0.00478973	0	5	3	1.02643191	0.93173516
chr1	49821553	49903412	81860	2	1	0	0.00478973	0	5	2	1.02643191	0.61140001
chr1	49903412	49988076	84665	2	1	0	0.01091641	0	4	2	0.76005302	0.61140001
chr1	49988076	49988135	60	1	1	1	0.00478973	0.05404976	5	2	1.02643191	0.30102999
chr1	49988135	50087714	99580	2	1	1	0.01091641	0.05404976	4	2	0.76005302	0.30102999
chr1	50087714	50087773	60	1	1	1	0.00478973	0.05404976	5	2	1.02643191	0.30102999

chr1	50087773	50193502	105730	2	1	1	0.02438896	0.05404976	3	2	0.51676182	0.30102999
chr1	50193502	50251479	57978	1	1	0	0.05404976	0	2	1	0.30102999	0.30102999
chr1	50251479	50304991	53513	2	1	0	0.01091641	0	4	1	0.76005302	0.30102999
chr1	50304991	50305050	60	1	1	0	0.00478973	0	5	1	1.02643191	0.30102999
chr1	50305050	50419450	114401	2	1	0	0.02438896	0	3	1	0.51676182	0.30102999
chr1	50419450	50484358	64909	2	1	0	0.01091641	0	4	1	0.76005302	0.30102999
chr1	50484358	50607503	123146	4	1	0	0.00478973	0	5	1	1.02643191	0.30102999
chr1	50607503	50610664	3162	2	1	0	0.00204627	0	6	1	1.31360226	0.30102999
chr1	50610664	50666998	56335	1	1	0	0.00478973	0	5	1	1.02643191	0.30102999
chr1	50666998	50732062	65065	1	1	0	0.00478973	0	5	0	1.02643191	0
chr1	50732062	50817322	85261	2	1	0	0.00204627	0	6	0	1.31360226	0
chr1	50817322	51073201	255880	6	1	0	0.00204627	0	6	1	1.31360226	0.30102999
chr1	51073201	51134787	61587	1	1	0	0.00478973	0	5	1	1.02643191	0.30102999
chr1	51134787	51204584	69798	1	1	0	0.00478973	0	5	0	1.02643191	0
chr1	51204584	51267338	62755	2	1	0	0.00204627	0	6	0	1.31360226	0
chr1	51267338	51295690	28353	1	1	0	0.00478973	0	5	0	1.02643191	0
chr1	51295690	51295749	60	1	1	0	0.00204627	0	6	0	1.31360226	0
chr1	51295749	51368914	73166	1	1	0	0.01091641	0	4	0	0.76005302	0
chr1	51368914	51421960	53047	2	1	0	0.00478973	0	5	0	1.02643191	0
chr1	51421960	51437689	15730	3	1	0	8.47E-04	0	7	0	1.62048027	0
chr1	51437689	51639403	201715	3	1	0	0.00204627	0	6	0	1.31360226	0
chr1	51639403	51703676	64274	2	1	0	0.00204627	0	6	2	1.31360226	0.61140001
chr1	51703676	51826867	123192	2	1	0	0.00204627	0	6	1	1.31360226	0.30102999
chr1	51826867	51972508	145642	5	1	0	0.00204627	0	6	2	1.31360226	0.61140001
chr1	51972508	52135114	162607	4	1	1	0.00204627	0.05404976	6	2	1.31360226	0.30102999
chr1	52135114	52135169	56	1	2	1	0.01053319	0.05404976	6	2	0.91219088	0.30102999
chr1	52135169	52179686	44518	1	1	1	0.00204627	0.05404976	6	2	1.31360226	0.30102999
chr1	52179686	52254215	74530	2	1	1	8.47E-04	0.05404976	7	2	1.62048027	0.30102999
chr1	52254215	52385262	131048	5	2	1	0.00493743	0.05404976	7	2	1.16581773	0.30102999
chr1	52385262	52385321	60	1	3	1	0.01598258	0.05404976	7	2	0.84395715	0.30102999
chr1	52385321	52467725	82405	2	2	1	0.00493743	0.1218695	7	1	1.16581773	0.1218695
chr1	52467725	52564775	97051	3	1	1	8.47E-04	0.1218695	7	1	1.62048027	0.1218695
chr1	52564775	52564834	60	1	2	1	0.00493743	0.1218695	7	1	1.16581773	0.1218695
chr1	52564834	52632041	67208	1	2	1	0.01053319	0.1218695	6	1	0.91219088	0.1218695
chr1	52632041	52682108	50068	2	2	1	0.01053319	0.05404976	6	2	0.91219088	0.30102999
chr1	52682108	52703591	21484	2	2	1	0.00493743	0.05404976	7	2	1.16581773	0.30102999
chr1	52703591	53104025	400435	8	2	1	0.01053319	0.05404976	6	2	0.91219088	0.30102999
chr1	53104025	53162174	58150	3	3	1	0.03070643	0.05404976	6	2	0.63695542	0.30102999
chr1	53162174	53162233	60	1	3	1	0.01598258	0.05404976	7	2	0.84395715	0.30102999
chr1	53162233	53246226	83994	2	2	1	0.01053319	0.05404976	6	2	0.91219088	0.30102999
chr1	53246226	53246285	60	1	3	1	0.01598258	0.05404976	7	2	0.84395715	0.30102999
chr1	53246285	53293068	46784	1	1	1	0.00204627	0.05404976	6	2	1.31360226	0.30102999
chr1	53293068	53366252	73185	2	1	2	0.00204627	0.1575501	6	2	1.31360226	0.1575501
chr1	53366252	53371432	5181	3	2	2	0.00493743	0.1575501	7	2	1.16581773	0.1575501
chr1	53371432	53395707	24276	1	1	2	8.47E-04	0.1575501	7	2	1.62048027	0.1575501
chr1	53395707	53427174	31468	2	2	2	0.00493743	0.1575501	7	2	1.16581773	0.1575501
chr1	53427174	53427232	59	1	3	2	0.01598258	0.1575501	7	2	0.84395715	0.1575501
chr1	53427232	53493769	66538	1	1	2	8.47E-04	0.1575501	7	2	1.62048027	0.1575501
chr1	53493769	53643189	149421	6	1	2	0.00204627	0.1575501	6	2	1.31360226	0.1575501
chr1	53643189	53701905	58717	5	2	2	0.01053319	0.1575501	6	2	0.91219088	0.1575501
chr1	53701905	53763468	61564	2	1	2	0.00204627	0.1575501	6	2	1.31360226	0.1575501
chr1	53763468	53897468	134001	3	1	2	0.00204627	0.08289318	6	3	1.31360226	0.30102999
chr1	53897468	54108906	211439	7	1	3	0.00204627	0.17593012	6	3	1.31360226	0.17593012
chr1	54108906	54199792	90887	2	1	3	0.00478973	0.17593012	5	3	1.02643191	0.17593012
chr1	54199792	54199845	54	1	1	3	0.00204627	0.17593012	6	3	1.31360226	0.17593012
chr1	54199845	54252893	53049	3	1	3	0.00478973	0.17593012	5	3	1.02643191	0.17593012
chr1	54252893	54324951	72059	3	1	3	0.00478973	0.10122019	5	4	1.02643191	0.30102999
chr1	54324951	54376677	51727	3	1	3	0.00204627	0.10122019	6	4	1.31360226	0.30102999
chr1	54376677	54421881	45205	4	1	3	0.00204627	0.05670724	6	5	1.31360226	0.45545077
chr1	54421881	54505441	83561	2	1	3	0.00478973	0.05670724	5	5	1.02643191	0.45545077
chr1	54505441	54512973	7533	2	1	3	0.00204627	0.05670724	6	5	1.31360226	0.45545077
chr1	54512973	54534566	21594	1	1	3	0.00478973	0.05670724	5	5	1.02643191	0.45545077
chr1	54534566	54577664	43099	1	1	2	0.01091641	0.02162467	4	5	0.76005302	0.68214471
chr1	54577664	54577723	60	1	1	2	0.00478973	0.02162467	5	5	1.02643191	0.68214471
chr1	54577723	54673152	95430	2	1	2	0.00478973	0.0429175	5	4	1.02643191	0.47744371
chr1	54673152	54748969	75818	1	1	1	0.00478973	0.01091641	5	4	1.02643191	0.76005302
chr1	54748969	54749028	60	1	1	1	0.00204627	0.01091641	6	4	1.31360226	0.76005302
chr1	54749028	54832143	83116	2	1	1	0.00478973	0.01091641	5	4	1.02643191	0.76005302
chr1	54832143	54832202	60	1	1	2	0.00478973	0.0429175	5	4	1.02643191	0.47744371
chr1	54832202	54869282	37081	1	1	1	0.00478973	0.01091641	5	4	1.02643191	0.76005302
chr1	54869282	55134060	264779	6	1	1	0.01091641	0.01091641	4	4	0.76005302	0.76005302
chr1	55134060	55134119	60	1	1	1	0.01091641	0.00478973	4	5	0.76005302	1.02643191
chr1	55134119	55183468	49350	1	1	1	0.01091641	0.01091641	4	4	0.76005302	0.76005302
chr1	55183468	55252418	68951	5	1	1	0.00478973	0.01091641	5	4	1.02643191	0.76005302
chr1	55252418	55338790	86373	3	1	1	0.01091641	0.01091641	4	4	0.76005302	0.76005302
chr1	55338790	55465890	127101	3	0	1	0	0.01091641	4	4	1.26272838	0.76005302
chr1	55465890	55636899	171010	4	0	1	0	0.05404976	4	2	1.26272838	0.30102999
chr1	55636899	55709778	72880	3	0	1	0	0.02438896	4	3	1.26272838	0.51676182
chr1	55709778	55848004	138227	1	0	1	0	0.02438896	3	3	0.93173516	0.51676182
chr1	55848004	56001939	153936	2	0	1	0	0.05404976	3	2	0.93173516	0.30102999
chr1	56001939	56001998	60	1	0	1	0	0.02438896	3	3	0.93173516	0.51676182
chr1	56001998	56084805	82808	1	0	1	0	0.05404976	3	2	0.93173516	0.30102999
chr1	56084805	56137547	52743	2	0	1	0	0.05404976	4	2	1.26272838	0.30102999
chr1	56137547	56193319	55773	2	1	1	0.01091641	0.05404976	4	2	0.76005302	0.30102999
chr1	56193319	56299437	106119	1	1	1	0.01091641	0.1218695	4	1	0.76005302	0.1218695
chr1	56299437	56429654	130218	1	1	1	0.05404976	0.1218695	2	1	0.30102999	0.1218695
chr1	56429654	56591530	161877	1	0	1	0	0.1218695	2	1	0.61140001	0.1218695
chr1	56591530	56591589	60	1	0	1	0	0.05404976	3	2	0.93173516	0.30102999
chr1	56591589	56911268	319680	2	0	1	0	0.1218695	3	1	0.93173516	0.1218695
chr1	56911268	56911327	60	1	0	2	0	0.1575501	3	2	0.93173516	0.1575501
chr1	56911327	56960896	49570	1	0	2	0	0.1575501	2	2	0.61140001	0.1575501
chr1	56960896	57006286	45391	2	1	2	0.05404976	0.1575501	2	2	0.30102999	0.1575501
chr1	57006286	57119876	113591	5	1	2	0.05404976	0.08289318	2	3	0.30102999	0.30102999
chr1	57119876	57383514	263639	6	1	2	0.02438896	0.08289318	3	3	0.51676182	0.30102999
chr1	57383514	57499255	115742	4	1	2	0.01091641	0.08289318	4	3	0.76005302	0.30102999
chr1	57499255	57865962	366708	7	1	2	0.01091641	0.1575501	4	2	0.76005302	0.1575501
chr1	57865962	57911493	45532	2	1	2	0.00478973	0.1575501	5	2	1.02643191	0.1575501
chr1	57911493	58043100	131608	2	1	2	0.01091641	0.1575501	4	2	0.76005302	0.1575501
chr1	58043100	58130775	87676	3	1	2	0.00478973	0.1575501	5	2	1.02643191	0.1575501
chr1	58130775	58229711	98937	2	1	2	0.01091641	0.1575501	4	2	0.76005302	0.1575501
chr1	58229711	58229770	60	1	1	2	0.00478973	0.1575501	5	2	1.02643191	0.1575501
chr1	58229770	58616209	386440	8	1	2	0.00478973	0.30102999	5	1	1.02643191	0.05404976
chr1	58616209	58616262	54	1	1	2	0.00204627	0.30102999	6	1	1.31360226	0.05404976
chr1	58616262	58733477	117216	2	1	2	0.00478973	0.30102999	5	1	1.02643191	0.05404976
chr1	58733477	58938470	204994	5	1	2	0.01091641	0.30102999	4	1	0.76005302	0.05404976
chr1	58938470	59011048	72579	2	1	2	0.01091641	0.61140001	4	0	0.76005302	0
chr1	59011048	59011107	60	1	1	2	0.01091641	0.30102999	4	1	0.76005302	0.05404976
chr1	59011107	59117685	106579	2	1	2	0.01091641	0.61140001	4	0	0.76005302	0
chr1	59117685	59117744	60	1	1	2	0.01091641	0.30102999	4	1	0.76005302	0.05404976
chr1	59117744	59249622	131879	5	1	2	0.01091641	0.61140001	4	0	0.76005302	0
chr1	59249622	59406914	157293	2	1	1	0.01091641	0.30102999	4	0	0.76005302	0
chr1	59406914	59617938	211025	3	1	1	0.00478973	0.30102999	5	0	1.02643191	0
chr1	59617938	59617997	60	1	1	1	0.00204627	0.30102999	6	0	1.31360226	0
chr1	59617997	59704456	86460	1	1	1	0.00478973	0.30102999	5	0	1.02643191	0
chr1	59704456	59787180	82725	1	1	1	0.01091641	0.30102999	4	0	0.76005302	0
chr1	59787180	59848246	61067	2	1	1	0.00478973	0.30102999	5	0	1.02643191	0
chr1	59848246	59922750	74505	3	1	1	0.00204627	0.30102999	6	0	1.31360226	0
chr1	59922750	59983599	60850	1	0	1	0	0.30102999	4	0	1.26272838	0
chr1	59983599	60012563	28965	1	0	1	0	0.30102999	3	0	0.93173516	0
chr1	60012563	60073564	61002	2	0	1	0	0.30102999	4	0	1.26272838	0
chr1	60073564	60103940	30377	2	1	1	0.01091641	0.30102999	4	0	0.76005302	0
chr1	60103940	60168207	64268	1	1	1	0.02438896	0.30102999	3	0	0.51676182	0
chr1	60168207	60235003	66797	1	1	1	0.05404976	0.30102999	2	0	0.30102999	0
chr1	60235003	60235062	60	1	1	2	0.05404976	0.61140001	2	0	0.30102999	0

chr1	60235062	60568797	333736	8	1	2	0.1218695	0.61140001	1	0	0.1218695	0
chr1	60568797	60719746	150950	2	1	3	0.05404976	0.93173516	2	0	0.30102999	0
chr1	60719746	60719805	60	1	1	3	0.01091641	0.30102999	4	2	0.76005302	0.08289318
chr1	60719805	60907458	187654	1	1	2	0.01091641	0.61140001	4	0	0.76005302	0
chr1	60907458	60997806	90349	2	1	2	0.00204627	0.61140001	6	0	1.31360226	0
chr1	60997806	61162108	164303	2	1	2	0.00478973	0.61140001	5	0	1.02643191	0
chr1	61162108	61280688	118581	3	1	2	0.00204627	0.61140001	6	0	1.31360226	0
chr1	61280688	61280747	60	1	1	2	0.00204627	0.1575501	6	2	1.31360226	0.1575501
chr1	61280747	61353663	72917	1	1	2	0.00204627	0.30102999	6	1	1.31360226	0.05404976
chr1	61353663	61417247	63585	1	1	2	0.00478973	0.30102999	5	1	1.02643191	0.05404976
chr1	61417247	61485099	67853	1	1	2	0.00478973	0.61140001	5	0	1.02643191	0
chr1	61485099	61485158	60	1	1	2	0.00204627	0.61140001	6	0	1.31360226	0
chr1	61485158	61557457	72300	2	1	1	0.00204627	0.30102999	6	0	1.31360226	0
chr1	61557457	62518529	961073	20	2	1	0.01053319	0.30102999	6	0	0.91219088	0
chr1	62518529	62580114	61586	2	2	1	0.01053319	0.1218695	6	1	0.91219088	0.1218695
chr1	62580114	62863202	283089	4	1	1	0.00204627	0.30102999	6	0	1.31360226	0
chr1	62863202	62910630	47429	1	1	0	0.00204627	0	6	0	1.31360226	0
chr1	62910630	63159347	248718	7	1	0	0.00204627	0	6	1	1.31360226	0.30102999
chr1	63159347	63250454	91108	2	2	0	0.01053319	0	6	1	0.91219088	0.30102999
chr1	63250454	63307081	56628	2	2	0	0.02162467	0	5	1	0.68214471	0.30102999
chr1	63307081	63307140	60	1	2	0	0.01053319	0	6	1	0.91219088	0.30102999
chr1	63307140	63353105	45966	2	1	0	0.00204627	0	6	0	1.31360226	0
chr1	63353105	63353164	60	1	1	0	8.47E-04	0	7	1	1.62048027	0.30102999
chr1	63353164	63654978	301815	2	1	0	0.00204627	0	6	1	1.31360226	0.30102999
chr1	63654978	63684743	29766	2	1	2	0.00204627	0.1575501	6	2	1.31360226	0.1575501
chr1	63684743	63791203	106461	3	1	3	0.00204627	0.30102999	6	2	1.31360226	0.08289318
chr1	63791203	63836529	45327	1	1	3	0.00478973	0.30102999	5	2	1.02643191	0.08289318
chr1	63836529	63894700	58172	1	1	0	0.00478973	0	5	2	1.02643191	0.61140001
chr1	63894700	63945809	51110	2	1	0	0.00204627	0	6	2	1.31360226	0.61140001
chr1	63945809	63945868	60	1	1	2	0.00204627	0.1575501	6	2	1.31360226	0.1575501
chr1	63945868	63986935	41068	1	1	2	0.00478973	0.1575501	5	2	1.02643191	0.1575501
chr1	63986935	64011651	24717	1	1	1	0.00478973	0.1218695	5	1	1.02643191	0.1218695
chr1	64011651	64020995	9345	1	1	1	0.00478973	0.30102999	5	0	1.02643191	0
chr1	64020995	64021054	60	1	1	2	0.00478973	0.30102999	5	1	1.02643191	0.05404976
chr1	64021054	64125397	104344	2	1	2	0.00478973	0.61140001	5	0	1.02643191	0
chr1	64125397	64156739	31343	2	1	3	0.00478973	0.30102999	5	2	1.02643191	0.08289318
chr1	64156739	64249068	92330	1	1	2	0.00478973	0.1575501	5	2	1.02643191	0.1575501
chr1	64249068	64360282	111215	2	1	2	0.00478973	0.30102999	5	1	1.02643191	0.05404976
chr1	64360282	64474992	114711	3	1	2	0.00478973	0.1575501	5	2	1.02643191	0.1575501
chr1	64474992	64901143	426152	9	2	2	0.01053319	0.1575501	6	2	0.91219088	0.1575501
chr1	64901143	64948870	47728	2	2	4	0.01053319	0.47744371	6	2	0.91219088	0.0429175
chr1	64948870	65016263	67394	1	2	4	0.02162467	0.47744371	5	2	0.68214471	0.0429175
chr1	65016263	65016322	60	1	2	4	0.02162467	0.30102999	5	3	0.68214471	0.10122019
chr1	65016322	65068470	52149	1	1	4	0.00478973	0.47744371	5	2	1.02643191	0.0429175
chr1	65068470	65098061	29592	2	1	4	0.00204627	0.47744371	6	2	1.31360226	0.0429175
chr1	65098061	65098120	60	1	2	4	0.01053319	0.47744371	6	2	0.91219088	0.0429175
chr1	65098120	65218480	120361	2	1	3	0.00478973	0.30102999	5	2	1.02643191	0.08289318
chr1	65218480	65218539	60	1	2	3	0.01053319	0.30102999	6	2	0.91219088	0.08289318
chr1	65218539	65255117	36579	1	2	3	0.02162467	0.30102999	5	2	0.68214471	0.08289318
chr1	65255117	65281966	26850	2	2	4	0.02162467	0.30102999	5	3	0.68214471	0.10122019
chr1	65281966	65349103	67138	1	1	4	0.00478973	0.30102999	5	3	1.02643191	0.10122019
chr1	65349103	65461918	112816	4	1	4	0.00478973	0.18734596	5	4	1.02643191	0.18734596
chr1	65461918	65626868	164951	4	1	2	0.00478973	0.1575501	5	2	1.02643191	0.1575501
chr1	65626868	65709538	82671	3	2	2	0.02162467	0.1575501	5	2	0.68214471	0.1575501
chr1	65709538	65764893	55356	2	1	2	0.00478973	0.1575501	5	2	1.02643191	0.1575501
chr1	65764893	65854982	90090	2	1	2	0.00478973	0.30102999	5	1	1.02643191	0.05404976
chr1	65854982	65887445	32464	2	2	2	0.02162467	0.1575501	5	2	0.68214471	0.1575501
chr1	65887445	66036463	149019	3	1	2	0.00478973	0.1575501	5	2	1.02643191	0.1575501
chr1	66036463	66099329	62867	1	1	1	0.01091641	0.05404976	4	2	0.76005302	0.30102999
chr1	66099329	66099388	60	1	3	1	0.10122019	0.05404976	4	2	0.30102999	0.30102999
chr1	66099388	66172315	72928	1	3	0	0.10122019	0	4	2	0.30102999	0.61140001
chr1	66172315	66221653	49339	2	3	0	0.03070643	0	6	2	0.63695542	0.61140001
chr1	66221653	66221712	60	1	3	1	0.03070643	0.05404976	6	2	0.63695542	0.30102999
chr1	66221712	66333375	111664	2	1	0	0.00478973	0	5	2	1.02643191	0.61140001
chr1	66333375	66379014	45640	2	3	0	0.05670724	0	5	2	0.45545077	0.61140001
chr1	66379014	66497377	118364	2	2	0	0.02162467	0	5	2	0.68214471	0.61140001
chr1	66497377	66549455	52079	1	2	0	0.0429175	0	4	2	0.47744371	0.61140001
chr1	66549455	66619477	70023	2	2	1	0.0429175	0.05404976	4	2	0.47744371	0.30102999
chr1	66619477	66685095	65619	1	2	1	0.08289318	0.05404976	3	2	0.30102999	0.30102999
chr1	66685095	66685154	60	1	3	1	0.10122019	0.05404976	4	2	0.30102999	0.30102999
chr1	66685154	66716728	31575	1	3	1	0.17593012	0.05404976	3	2	0.17593012	0.30102999
chr1	66716728	66716787	60	1	4	1	0.18734596	0.05404976	4	2	0.18734596	0.30102999
chr1	66716787	66773257	56471	1	3	0	0.10122019	0	4	2	0.30102999	0.61140001
chr1	66773257	66806578	33322	2	3	0	0.05670724	0	5	2	0.45545077	0.61140001
chr1	66806578	66840093	33516	3	3	1	0.05670724	0.05404976	5	2	0.45545077	0.30102999
chr1	66840093	66840152	60	1	4	1	0.11390336	0.05404976	5	2	0.30102999	0.30102999
chr1	66840152	66943592	103441	1	3	1	0.05670724	0.05404976	5	2	0.45545077	0.30102999
chr1	66943592	66943651	60	1	4	1	0.11390336	0.05404976	5	2	0.30102999	0.30102999
chr1	66943651	67148594	204944	4	4	1	0.18734596	0.05404976	4	2	0.18734596	0.30102999
chr1	67148594	67263482	114889	3	3	1	0.10122019	0.05404976	4	2	0.30102999	0.30102999
chr1	67263482	67263541	60	1	3	2	0.10122019	0.1575501	4	2	0.30102999	0.1575501
chr1	67263541	67291225	27685	1	3	1	0.10122019	0.05404976	4	2	0.30102999	0.30102999
chr1	67291225	67299300	8076	2	3	1	0.05670724	0.05404976	5	2	0.45545077	0.30102999
chr1	67299300	67423725	124426	3	3	1	0.10122019	0.05404976	4	2	0.30102999	0.30102999
chr1	67423725	67423784	60	1	3	1	0.05670724	0.05404976	5	2	0.45545077	0.30102999
chr1	67423784	67510466	86683	2	3	1	0.10122019	0.05404976	4	2	0.30102999	0.30102999
chr1	67510466	67559899	49434	2	2	1	0.0429175	0.05404976	4	2	0.47744371	0.30102999
chr1	67559899	67559958	60	1	3	2	0.10122019	0.1575501	4	2	0.30102999	0.1575501
chr1	67559958	67635084	75127	1	2	2	0.0429175	0.1575501	4	2	0.47744371	0.1575501
chr1	67635084	67721572	86489	3	2	4	0.0429175	0.30102999	4	3	0.47744371	0.10122019
chr1	67721572	67721631	60	1	2	4	0.02162467	0.30102999	5	3	0.68214471	0.10122019
chr1	67721631	67845644	124014	4	2	4	0.0429175	0.30102999	4	3	0.47744371	0.10122019
chr1	67845644	67845701	58	1	2	4	0.0429175	0.18734596	4	4	0.47744371	0.18734596
chr1	67845701	67892445	46745	2	2	4	0.0429175	0.30102999	4	3	0.47744371	0.10122019
chr1	67892445	68010825	118381	1	2	3	0.08289318	0.17593012	3	3	0.30102999	0.17593012
chr1	68010825	68010884	60	1	3	3	0.17593012	0.17593012	3	3	0.17593012	0.17593012
chr1	68010884	68211951	201068	7	2	3	0.08289318	0.17593012	3	3	0.30102999	0.17593012
chr1	68211951	68283988	72038	2	1	3	0.02438896	0.17593012	3	3	0.51676182	0.17593012
chr1	68283988	68361872	77885	1	0	3	0	0.17593012	3	3	0.93173516	0.17593012
chr1	68361872	68429823	67952	1	0	1	0	0.02438896	3	3	0.93173516	0.51676182
chr1	68429823	68772057	342235	9	0	1	0	0.01091641	3	4	0.93173516	0.76005302
chr1	68772057	68895332	123276	2	0	0	0	0	3	3	0.93173516	0.93173516
chr1	68895332	68942862	47531	3	0	1	0	0.02438896	3	3	0.93173516	0.51676182
chr1	68942862	69004328	61467	4	0	2	0	0.08289318	3	3	0.93173516	0.30102999
chr1	69004328	69372414	368087	2	0	0	0	0	3	2	0.93173516	0.61140001
chr1	69372414	69445415	73002	2	0	2	0	0.08289318	3	3	0.93173516	0.30102999
chr1	69445415	69906026	460612	2	0	0	0	0	3	3	0.93173516	0.93173516
chr1	69906026	70049486	143461	2	0	3	0	0.17593012	3	3	0.93173516	0.17593012
chr1	70049486	70398314	348829	9	0	3	0	0.10122019	3	4	0.93173516	0.30102999
chr1	70398314	70508127	109814	2	0	3	0	0.17593012	3	3	0.93173516	0.17593012
chr1	70508127	70508186	60	1	0	3	0	0.10122019	3	4	0.93173516	0.30102999
chr1	70508186	70588342	80157	2	0	3	0	0.17593012	3	3	0.93173516	0.17593012
chr1	70588342	70625097	36756	3	0	3	0	0.10122019	3	4	0.93173516	0.30102999
chr1	70625097	70694095	68999	2	0	3	0	0.17593012	3	3	0.93173516	0.17593012
chr1	70694095	70890028	195934	7	0	3	0	0.10122019	3	4	0.93173516	0.30102999
chr1	70890028	70909788	19761	3	0	4	0	0.06713722	3	6	0.93173516	0.44141547
chr1	70909788	71185224	275437	3	0	4	0	0.18734596	3	4	0.93173516	0.18734596
chr1	71185224	71318416	133193	1	0	3	0	0.10122019	3	4	0.93173516	0.30102999
chr1	71318416	71477958	159543	6	0	3	0	0.05670724	3	5	0.93173516	0.45545077
chr1	71477958	71569394	91437	3	0	3	0	0.10122019	3	4	0.93173516	0.30102999
chr1	71569394	71677606	108213	1	0	3	0	0.17593012	3	3	0.93173516	0.17593012
chr1	71677606	71869199	191594	2	0	3	0	0.30102999	3	2	0.93173516	0.08289318
chr1	71869199	71869258	60	1	0	3	0	0.17593012	3	3	0.93173516	0.17593012
chr1	71869258	71996503	127246	3	0	3	0	0.30102999	3	2	0.93173516	0.08289318

chr1	71996503	71996562	60	1	0	3	0	0.17593012	3	3	0.93173516	0.17593012
chr1	71996562	72207033	210472	4	0	3	0	0.30102999	3	2	0.93173516	0.08289318
chr1	72207033	72292088	85056	3	0	3	0	0.17593012	3	3	0.93173516	0.17593012
chr1	72292088	72380292	88205	2	0	3	0	0.30102999	3	2	0.93173516	0.08289318
chr1	72380292	72523905	143614	3	0	3	0	0.17593012	3	3	0.93173516	0.17593012
chr1	72523905	72667496	143592	3	0	2	0	0.1575501	3	2	0.93173516	0.1575501
chr1	72667496	72667555	60	1	0	2	0	0.08289318	3	3	0.93173516	0.30102999
chr1	72667555	72992971	325417	3	0	2	0	0.1575501	3	2	0.93173516	0.1575501
chr1	72992971	72993030	60	1	0	3	0	0.30102999	3	2	0.93173516	0.08289318
chr1	72993030	73309668	316639	2	0	1	0	0.30102999	3	0	0.93173516	0
chr1	73309668	73309727	60	1	0	1	0	0.1218695	3	1	0.93173516	0.1218695
chr1	73309727	73628814	319088	2	0	1	0	0.30102999	2	0	0.61140001	0
chr1	73628814	73628873	60	1	1	1	0.05404976	0.1218695	2	1	0.30102999	0.1218695
chr1	73628873	73739318	110446	1	0	1	0	0.1218695	2	1	0.61140001	0.1218695
chr1	73739318	73908968	169651	2	0	2	0	0.1575501	3	2	0.93173516	0.1575501
chr1	73908968	73909027	60	1	0	4	0	0.30102999	3	3	0.93173516	0.10122019
chr1	73909027	74298462	389436	3	0	2	0	0.08289318	3	3	0.93173516	0.30102999
chr1	74298462	74298521	60	1	1	2	0.02438896	0.08289318	3	3	0.51676182	0.30102999
chr1	74298521	74425862	127342	1	0	2	0	0.08289318	3	3	0.93173516	0.30102999
chr1	74425862	74606861	181000	3	0	2	0	0.1575501	3	2	0.93173516	0.1575501
chr1	74606861	74654147	47287	1	0	2	0	0.30102999	3	1	0.93173516	0.05404976
chr1	74654147	74672597	18451	2	0	2	0	0.1575501	3	2	0.93173516	0.1575501
chr1	74672597	74763993	91397	3	0	2	0	0.08289318	3	3	0.93173516	0.30102999
chr1	74763993	75060461	296469	6	0	2	0	0.1575501	3	2	0.93173516	0.1575501
chr1	75060461	75122210	61750	1	0	2	0	0.1575501	2	2	0.61140001	0.1575501
chr1	75122210	75172840	50631	3	0	2	0	0.0429175	2	4	0.61140001	0.47744371
chr1	75172840	75230948	58109	1	0	2	0	0.08289318	2	3	0.61140001	0.30102999
chr1	75230948	75438153	207206	1	0	2	0	0.1575501	2	2	0.61140001	0.1575501
chr1	75438153	75438212	60	1	0	2	0	0.08289318	2	3	0.61140001	0.30102999
chr1	75438212	75617998	179787	1	0	2	0	0.1575501	2	2	0.61140001	0.1575501
chr1	75617998	75618057	60	1	1	2	0.05404976	0.08289318	2	3	0.30102999	0.30102999
chr1	75618057	75672223	54167	1	1	2	0.05404976	0.1575501	2	2	0.30102999	0.1575501
chr1	75672223	76076715	404493	8	0	2	0	0.1575501	2	2	0.61140001	0.1575501
chr1	76076715	76198509	121795	3	0	2	0	0.1575501	3	2	0.93173516	0.1575501
chr1	76198509	76227026	28518	3	0	2	0	0.08289318	3	3	0.93173516	0.30102999
chr1	76227026	76333172	106147	2	0	2	0	0.1575501	2	2	0.61140001	0.1575501
chr1	76333172	76393109	59938	3	0	2	0	0.08289318	3	3	0.93173516	0.30102999
chr1	76393109	76607428	214320	3	0	2	0	0.1575501	3	2	0.93173516	0.1575501
chr1	76607428	76607487	60	1	0	2	0	0.0429175	3	4	0.93173516	0.47744371
chr1	76607487	76671617	64131	1	0	2	0	0.1575501	3	2	0.93173516	0.1575501
chr1	76671617	76779517	107901	3	0	2	0	0.08289318	3	3	0.93173516	0.30102999
chr1	76779517	76779569	53	1	0	2	0	0.02162467	3	5	0.93173516	0.68214471
chr1	76779569	76813869	34301	1	0	2	0	0.0429175	3	4	0.93173516	0.47744371
chr1	76813869	76877932	64064	1	0	2	0	0.08289318	3	3	0.93173516	0.30102999
chr1	76877932	76999406	121475	2	0	2	0	0.1575501	2	2	0.61140001	0.1575501
chr1	76999406	77043603	44198	2	0	2	0	0.0429175	2	4	0.61140001	0.47744371
chr1	77043603	77099919	56317	3	0	2	0	0.0429175	3	4	0.93173516	0.47744371
chr1	77099919	77296310	196392	2	0	2	0	0.08289318	3	3	0.93173516	0.30102999
chr1	77296310	77333185	36876	2	0	2	0	0.0429175	3	4	0.93173516	0.47744371
chr1	77333185	77398143	64959	1	0	2	0	0.08289318	3	3	0.93173516	0.30102999
chr1	77398143	77398202	60	1	0	2	0	0.08289318	4	3	1.26272838	0.30102999
chr1	77398202	77487296	89095	2	0	1	0	0.02438896	4	3	1.26272838	0.51676182
chr1	77487296	77672306	185011	6	0	2	0	0.08289318	4	3	1.26272838	0.30102999
chr1	77672306	77672365	60	1	0	2	0	0.08289318	5	3	1.60515106	0.30102999
chr1	77672365	77747296	74932	1	0	1	0	0.02438896	5	3	1.60515106	0.51676182
chr1	77747296	77883406	136111	2	0	1	0	0.05404976	5	2	1.60515106	0.30102999
chr1	77883406	78002243	118838	2	0	1	0	0.05404976	4	2	1.26272838	0.30102999
chr1	78002243	78030194	27952	2	1	1	0.00478973	0.05404976	5	2	1.02643191	0.30102999
chr1	78030194	78069124	38931	3	1	1	0.01091641	0.05404976	4	2	0.76005302	0.30102999
chr1	78069124	78069183	60	1	1	1	0.00204627	0.05404976	6	2	1.31360226	0.30102999
chr1	78069183	78131099	61917	2	1	1	0.01091641	0.05404976	4	2	0.76005302	0.30102999
chr1	78131099	78161649	30551	2	1	1	0.00478973	0.05404976	5	2	1.02643191	0.30102999
chr1	78161649	78208270	46622	2	1	1	0.02438896	0.05404976	3	2	0.51676182	0.30102999
chr1	78208270	78286423	78154	3	1	1	0.00478973	0.05404976	5	2	1.02643191	0.30102999
chr1	78286423	78470596	184174	6	1	1	0.01091641	0.05404976	4	2	0.76005302	0.30102999
chr1	78470596	78560709	90114	4	1	2	0.01091641	0.1575501	4	2	0.76005302	0.1575501
chr1	78560709	78601492	40784	1	1	1	0.01091641	0.05404976	4	2	0.76005302	0.30102999
chr1	78601492	78688407	86916	1	1	1	0.02438896	0.05404976	3	2	0.51676182	0.30102999
chr1	78688407	78688466	60	1	1	1	0.01091641	0.05404976	4	2	0.76005302	0.30102999
chr1	78688466	78756739	68274	1	1	1	0.02438896	0.05404976	3	2	0.51676182	0.30102999
chr1	78756739	78789110	32372	2	1	1	0.01091641	0.05404976	4	2	0.76005302	0.30102999
chr1	78789110	78789169	60	1	1	1	0.00478973	0.05404976	5	2	1.02643191	0.30102999
chr1	78789169	78894164	104996	2	1	1	0.01091641	0.05404976	4	2	0.76005302	0.30102999
chr1	78894164	78993287	99124	4	1	1	0.00478973	0.05404976	5	2	1.02643191	0.30102999
chr1	78993287	79334253	340967	5	1	1	0.02438896	0.05404976	3	2	0.51676182	0.30102999
chr1	79334253	79383374	49122	2	1	0	0.02438896	0	3	1	0.51676182	0.30102999
chr1	79383374	79438635	55262	1	1	0	0.05404976	0	2	1	0.30102999	0.30102999
chr1	79438635	79438694	60	1	1	1	0.05404976	0.1218695	2	1	0.30102999	0.1218695
chr1	79438694	79476571	37878	1	1	0	0.05404976	0	2	1	0.30102999	0.30102999
chr1	79476571	79476630	60	1	1	1	0.05404976	0.1218695	2	1	0.30102999	0.1218695
chr1	79476630	79541419	64790	1	0	1	0	0.1218695	2	1	0.61140001	0.1218695
chr1	79541419	79777884	236466	1	0	0	0	0	2	1	0.61140001	0.30102999
chr1	79777884	79777943	60	1	1	0	0.05404976	0	2	1	0.30102999	0.30102999
chr1	79777943	79964480	186538	1	0	0	0	0	2	1	0.61140001	0.30102999
chr1	79964480	80057279	92800	2	0	1	0	0.1218695	2	1	0.61140001	0.1218695
chr1	80057279	80057338	60	1	0	1	0	0.05404976	2	2	0.61140001	0.30102999
chr1	80057338	80505598	448261	2	0	1	0	0.1218695	1	1	0.30102999	0.1218695
chr1	80505598	80656615	151018	2	0	1	0	0.1218695	2	1	0.61140001	0.1218695
chr1	80656615	80928119	271505	2	0	1	0	0.1218695	0	1	0	0.1218695
chr1	80928119	80961418	33300	2	0	1	0	0.1218695	2	1	0.61140001	0.1218695
chr1	80961418	81352882	391465	2	0	1	0	0.1218695	1	1	0.30102999	0.1218695
chr1	81352882	81352941	60	1	0	2	0	0.1575501	2	2	0.61140001	0.1575501
chr1	81352941	81696782	343842	3	0	1	0	0.05404976	2	2	0.61140001	0.30102999
chr1	81696782	81902679	205898	4	0	2	0	0.1575501	2	2	0.61140001	0.1575501
chr1	81902679	81951341	48663	1	0	1	0	0.05404976	2	2	0.61140001	0.30102999
chr1	81951341	82117214	165874	3	0	1	0	0.1218695	2	1	0.61140001	0.1218695
chr1	82117214	82302640	185427	5	0	2	0	0.30102999	2	1	0.61140001	0.05404976
chr1	82302640	82302699	60	1	0	2	0	0.1575501	4	2	1.26272838	0.1575501
chr1	82302699	82409276	106578	2	0	1	0	0.1218695	2	1	0.61140001	0.1218695
chr1	82409276	82455389	46114	3	0	2	0	0.1575501	3	2	0.93173516	0.1575501
chr1	82455389	82911924	456536	3	0	1	0	0.1218695	3	1	0.93173516	0.1218695
chr1	82911924	83288711	376788	2	0	0	0	0	2	1	0.61140001	0.30102999
chr1	83288711	83965176	676466	4	0	0	0	0	2	0	0.61140001	0
chr1	83965176	84080513	115338	2	0	0	0	0	2	1	0.61140001	0.30102999
chr1	84080513	84204375	123863	2	0	1	0	0.02438896	2	3	0.61140001	0.51676182
chr1	84204375	84204434	60	1	0	1	0	0.00478973	2	5	0.61140001	1.02643191
chr1	84204434	84334389	129956	2	0	1	0	0.01091641	2	4	0.61140001	0.76005302
chr1	84334389	84372188	37800	3	1	2	0.05404976	0.0429175	2	4	0.30102999	0.47744371
chr1	84372188	84459892	87705	2	1	1	0.05404976	0.01091641	2	4	0.30102999	0.76005302
chr1	84459892	84512203	52312	1	1	1	0.05404976	0.02438896	2	3	0.30102999	0.51676182
chr1	84512203	84554078	41876	2	1	2	0.05404976	0.0429175	2	4	0.30102999	0.47744371
chr1	84554078	84610179	56102	1	1	1	0.05404976	0.01091641	2	4	0.30102999	0.76005302
chr1	84610179	84767606	157428	5	1	1	0.05404976	0.00478973	2	5	0.30102999	1.02643191
chr1	84767606	84767665	60	1	1	3	0.05404976	0.05670724	2	5	0.30102999	0.45545077
chr1	84767665	84955332	187668	3	1	2	0.05404976	0.0429175	2	4	0.30102999	0.47744371
chr1	84955332	84963276	7945	2	1	2	0.02438896	0.0429175	3	4	0.51676182	0.47744371
chr1	84963276	84988114	24839	3	1	2	0.05404976	0.08289318	2	3	0.30102999	0.30102999
chr1	84988114	85255353	267240	6	1	2	0.02438896	0.0429175	3	4	0.51676182	0.47744371
chr1	85255353	85308029	52677	3	1	3	0.02438896	0.10122019	3	4	0.51676182	0.30102999
chr1	85308029	85407264	99236	2	1	3	0.02438896	0.17593012	3	3	0.51676182	0.17593012
chr1	85407264	85484587	77324	2	1	3	0.02438896	0.10122019	3	4	0.51676182	0.30102999
chr1	85484587	85510853	26267	3	1	3	0.01091641	0.10122019	4	4	0.76005302	0.30102999
chr1	85510853	85640378	129526	3	1	1	0.01091641	0.01091641	4	4	0.76005302	0.76005302
chr1	85640378	85738699	98322	6	1	2	0.01091641	0.0429175	4	4	0.76005302	0.47744371

chr1	85738699	85786373	47675	1	0	1	0	0.01091641	2	4	0.61140001	0.76005302
chr1	85786373	85817170	30798	1	0	1	0	0.02438896	2	3	0.61140001	0.51676182
chr1	85817170	85890856	73687	3	0	1	0	0.02438896	3	3	0.93173516	0.51676182
chr1	85890856	85920127	29272	2	0	2	0	0.0429175	3	4	0.93173516	0.47744371
chr1	85920127	86246693	326567	7	0	1	0	0.02438896	3	3	0.93173516	0.51676182
chr1	86246693	86246752	60	1	0	2	0	0.0429175	3	4	0.93173516	0.47744371
chr1	86246752	86369841	123090	2	0	1	0	0.01091641	3	4	0.93173516	0.76005302
chr1	86369841	86369900	60	1	0	2	0	0.0429175	3	4	0.93173516	0.47744371
chr1	86369900	86480595	110696	2	0	2	0	0.08289318	2	3	0.61140001	0.30102999
chr1	86480595	86480654	60	1	0	2	0	0.02162467	3	5	0.93173516	0.68214471
chr1	86480654	86524822	44169	1	0	2	0	0.08289318	3	3	0.93173516	0.30102999
chr1	86524822	86524881	60	1	0	2	0	0.0429175	4	4	1.26272838	0.47744371
chr1	86524881	86622160	97280	2	0	1	0	0.01091641	3	4	0.93173516	0.76005302
chr1	86622160	86658426	36267	2	0	1	0	0.01091641	4	4	1.26272838	0.76005302
chr1	86658426	86792408	133983	1	0	1	0	0.01091641	3	4	0.93173516	0.76005302
chr1	86792408	87026053	233646	6	0	2	0	0.01053319	3	6	0.93173516	0.91219088
chr1	87026053	87101691	75639	1	0	2	0	0.01053319	2	6	0.61140001	0.91219088
chr1	87101691	87329131	227441	4	0	2	0	0.02162467	2	5	0.61140001	0.68214471
chr1	87329131	87382584	53454	3	0	2	0	0.01053319	2	6	0.61140001	0.91219088
chr1	87382584	87382643	60	1	0	2	0	0.00493743	2	7	0.61140001	1.16581773
chr1	87382643	87464692	82050	2	0	2	0	0.02162467	2	5	0.61140001	0.68214471
chr1	87464692	87464751	60	1	0	2	0	0.01053319	2	6	0.61140001	0.91219088
chr1	87464751	87634241	169491	5	0	2	0	0.0429175	2	4	0.61140001	0.47744371
chr1	87634241	87674452	40212	2	0	2	0	0.01053319	2	6	0.61140001	0.91219088
chr1	87674452	87674511	60	1	0	2	0	0.01053319	3	6	0.93173516	0.91219088
chr1	87674511	87797897	123387	1	0	1	0	0.00478973	3	5	0.93173516	1.02643191
chr1	87797897	87810977	13081	3	0	1	0	0.00204627	3	6	0.93173516	1.31360226
chr1	87810977	87873063	62087	1	0	1	0	0.00478973	2	5	0.61140001	1.02643191
chr1	87873063	88079298	206236	2	0	0	0	0	2	5	0.61140001	1.60515106
chr1	88079298	88079357	60	1	0	1	0	0.00478973	2	5	0.61140001	1.02643191
chr1	88079357	88415379	336023	2	0	0	0	0	2	1	0.61140001	0.30102999
chr1	88415379	88415438	60	1	0	1	0	0.1218695	2	1	0.61140001	0.1218695
chr1	88415438	88673048	257611	2	0	0	0	0	2	1	0.61140001	0.30102999
chr1	88673048	88767982	94935	2	0	1	0	0.1218695	2	1	0.61140001	0.1218695
chr1	88767982	88768041	60	1	0	1	0	0.05404976	2	2	0.61140001	0.30102999
chr1	88768041	89074748	306708	3	0	0	0	0	2	1	0.61140001	0.30102999
chr1	89074748	89137658	62911	2	0	0	0	0	3	2	0.93173516	0.61140001
chr1	89137658	89456653	318996	8	1	0	0.02438896	0	3	2	0.51676182	0.61140001
chr1	89456653	89531764	75112	3	1	1	0.02438896	0.05404976	3	2	0.51676182	0.30102999
chr1	89531764	89729598	197835	5	1	1	0.01091641	0.05404976	4	2	0.76005302	0.30102999
chr1	89729598	90033591	303994	7	1	1	0.00478973	0.05404976	5	2	1.02643191	0.30102999
chr1	90033591	90133991	100401	5	2	1	0.02162467	0.05404976	5	2	0.68214471	0.30102999
chr1	90133991	90209527	75537	2	1	1	0.00478973	0.05404976	5	2	1.02643191	0.30102999
chr1	90209527	90287674	78148	2	2	2	0.02162467	0.1575501	5	2	0.68214471	0.1575501
chr1	90287674	90461398	173725	3	2	1	0.02162467	0.05404976	5	2	0.68214471	0.30102999
chr1	90461398	90570894	109497	4	2	2	0.02162467	0.1575501	5	2	0.68214471	0.1575501
chr1	90570894	90706502	135609	1	0	1	0	0.05404976	4	2	1.26272838	0.30102999
chr1	90706502	90826352	119851	2	0	1	0	0.02438896	4	3	1.26272838	0.51676182
chr1	90826352	90826411	60	1	1	2	0.01091641	0.08289318	4	3	0.76005302	0.30102999
chr1	90826411	91006280	179870	1	0	2	0	0.1575501	4	2	1.26272838	0.1575501
chr1	91006280	91193502	187223	2	0	2	0	0.30102999	4	1	1.26272838	0.05404976
chr1	91193502	91404764	211263	4	0	2	0	0.1575501	4	2	1.26272838	0.1575501
chr1	91404764	91447869	43106	1	0	2	0	0.30102999	4	1	1.26272838	0.05404976
chr1	91447869	91447928	60	1	0	2	0	0.30102999	5	1	1.60515106	0.05404976
chr1	91447928	91518883	70956	2	0	1	0	0.30102999	5	0	1.60515106	0
chr1	91518883	91603849	84967	1	0	0	0	0	5	0	1.60515106	0
chr1	91603849	91656401	52553	2	0	1	0	0.30102999	6	0	1.95986592	0
chr1	91656401	91656460	60	1	0	2	0	0.61140001	6	0	1.95986592	0
chr1	91656460	91783820	127361	2	0	1	0	0.30102999	6	0	1.95986592	0
chr1	91783820	91841825	58006	2	0	2	0	0.61140001	6	0	1.95986592	0
chr1	91841825	91841884	60	1	0	2	0	0.30102999	6	1	1.95986592	0.05404976
chr1	91841884	91915483	73600	1	0	2	0	0.30102999	5	1	1.60515106	0.05404976
chr1	91915483	91968170	52688	1	0	2	0	0.30102999	4	1	1.26272838	0.05404976
chr1	91968170	92047477	79308	2	0	2	0	0.30102999	3	1	0.93173516	0.05404976
chr1	92047477	92148550	101074	3	0	2	0	0.30102999	4	1	1.26272838	0.05404976
chr1	92148550	92292559	144010	4	0	2	0	0.30102999	3	1	0.93173516	0.05404976
chr1	92292559	92292618	60	1	0	2	0	0.08289318	4	3	1.26272838	0.30102999
chr1	92292618	92365223	72606	2	0	2	0	0.08289318	2	3	0.61140001	0.30102999
chr1	92365223	92394807	29585	2	0	2	0	0.08289318	3	3	0.93173516	0.30102999
chr1	92394807	92446314	51508	3	0	2	0	0.1575501	2	2	0.61140001	0.1575501
chr1	92446314	92446373	60	1	0	2	0	0.1575501	3	2	0.93173516	0.1575501
chr1	92446373	92636078	189706	5	0	2	0	0.1575501	2	2	0.61140001	0.1575501
chr1	92636078	92668850	32773	2	0	2	0	0.08289318	3	3	0.93173516	0.30102999
chr1	92668850	92730220	61371	2	0	2	0	0.08289318	4	3	1.26272838	0.30102999
chr1	92730220	92789083	58864	2	0	2	0	0.08289318	3	3	0.93173516	0.30102999
chr1	92789083	93045810	256728	7	0	2	0	0.08289318	4	3	1.26272838	0.30102999
chr1	93045810	93160922	115113	2	0	2	0	0.08289318	3	3	0.93173516	0.30102999
chr1	93160922	93160981	60	1	0	2	0	0.08289318	4	3	1.26272838	0.30102999
chr1	93160981	93202086	41106	1	0	2	0	0.1575501	4	2	1.26272838	0.1575501
chr1	93202086	93257895	55810	1	0	2	0	0.1575501	3	2	0.93173516	0.1575501
chr1	93257895	93257948	54	1	0	2	0	0.08289318	3	3	0.93173516	0.30102999
chr1	93257948	93422917	164970	5	0	1	0	0.02438896	3	3	0.93173516	0.51676182
chr1	93422917	93545991	123075	2	0	1	0	0.05404976	3	2	0.93173516	0.30102999
chr1	93545991	93546050	60	1	0	1	0	0.02438896	3	3	0.93173516	0.51676182
chr1	93546050	93599711	53662	1	0	1	0	0.05404976	3	2	0.93173516	0.30102999
chr1	93599711	93645136	45426	3	0	1	0	0.02438896	3	3	0.93173516	0.51676182
chr1	93645136	93736157	91022	2	0	1	0	0.05404976	3	2	0.93173516	0.30102999
chr1	93736157	93825274	89118	5	0	1	0	0.02438896	3	3	0.93173516	0.51676182
chr1	93825274	93919246	93973	1	0	0	0	0	3	2	0.93173516	0.61140001
chr1	93919246	93966723	47478	2	1	0	0.02438896	0	3	2	0.51676182	0.61140001
chr1	93966723	93998556	31834	2	2	0	0.08289318	0	3	2	0.30102999	0.61140001
chr1	93998556	94078420	79865	3	2	0	0.0429175	0	4	2	0.47744371	0.61140001
chr1	94078420	94107847	29428	1	0	0	0	0	4	2	1.26272838	0.61140001
chr1	94107847	94140514	32668	2	0	0	0	0	5	2	1.60515106	0.61140001
chr1	94140514	94217172	76659	1	0	0	0	0	4	0	1.26272838	0
chr1	94217172	94276328	59157	2	2	0	0.0429175	0	4	0	0.47744371	0
chr1	94276328	94276387	60	1	2	0	0.01053319	0	6	0	0.91219088	0
chr1	94276387	94354475	78089	4	2	0	0.02162467	0	5	0	0.68214471	0
chr1	94354475	94375551	21077	1	2	0	0.0429175	0	4	0	0.47744371	0
chr1	94375551	94480073	104523	3	1	0	0.01091641	0	4	0	0.76005302	0
chr1	94480073	94517215	37143	2	1	0	0.00478973	0	5	0	1.02643191	0
chr1	94517215	94588230	71016	1	1	0	0.01091641	0	4	0	0.76005302	0
chr1	94588230	94638562	50333	2	2	0	0.0429175	0	4	0	0.47744371	0
chr1	94638562	94667859	29298	3	2	0	0.02162467	0	5	0	0.68214471	0
chr1	94667859	94743938	76080	2	1	0	0.01091641	0	4	0	0.76005302	0
chr1	94743938	94743997	60	1	2	0	0.01053319	0	6	0	0.91219088	0
chr1	94743997	94827539	83543	1	2	0	0.0429175	0	4	0	0.47744371	0
chr1	94827539	94897653	70115	2	2	0	0.02162467	0	5	0	0.68214471	0
chr1	94897653	95387398	489746	12	1	0	0.00478973	0	5	0	1.02643191	0
chr1	95387398	95710242	322845	8	0	0	0	0	5	0	1.60515106	0
chr1	95710242	95873833	163592	2	0	0	0	0	4	0	1.26272838	0
chr1	95873833	96011540	137708	2	0	0	0	0	5	0	1.60515106	0
chr1	96011540	96149407	137868	2	0	0	0	0	6	0	1.95986592	0
chr1	96149407	96308950	159544	2	0	0	0	0	6	1	1.95986592	0.30102999
chr1	96308950	96420239	111290	1	0	0	0	0	4	1	1.26272838	0.30102999
chr1	96420239	96420298	60	1	0	1	0	0.1218695	4	1	1.26272838	0.1218695
chr1	96420298	96501616	81319	1	0	0	0	0	3	1	0.93173516	0.30102999
chr1	96501616	96632596	130981	1	0	0	0	0	3	0	0.93173516	0
chr1	96632596	96632655	60	1	0	0	0	0	4	0	1.26272838	0
chr1	96632655	96808651	175997	2	0	0	0	0	3	0	0.93173516	0
chr1	96808651	96808710	60	1	0	0	0	0	4	0	1.26272838	0
chr1	96808710	97103753	295044	4	0	0	0	0	2	0	0.61140001	0
chr1	97103753	97154602	50850	2	0	0	0	0	3	0	0.93173516	0
chr1	97154602	97268310	113709	4	0	1	0	0.30102999	3	0	0.93173516	0

chr1	97268310	97479666	211357	2	0	1	0	0.30102999	2	0	0.61140001	0
chr1	97479666	97547908	68243	2	0	1	0	0.30102999	3	0	0.93173516	0
chr1	97547908	97547967	60	1	0	2	0	0.30102999	4	1	1.26272838	0.05404976
chr1	97547967	97665736	117770	2	0	2	0	0.30102999	3	1	0.93173516	0.05404976
chr1	97665736	97665795	60	1	1	2	0.01091641	0.30102999	4	1	0.76005302	0.05404976
chr1	97665795	97790476	124682	2	0	2	0	0.30102999	3	1	0.93173516	0.05404976
chr1	97790476	97790535	60	1	1	2	0.01091641	0.30102999	4	1	0.76005302	0.05404976
chr1	97790535	97839220	48686	1	0	2	0	0.30102999	4	1	1.26272838	0.05404976
chr1	97839220	98186019	346800	6	0	2	0	0.30102999	3	1	0.93173516	0.05404976
chr1	98186019	98187099	1081	3	1	2	0.01091641	0.30102999	4	1	0.76005302	0.05404976
chr1	98187099	98299938	112840	2	1	1	0.02438896	0.1218695	3	1	0.51676182	0.1218695
chr1	98299938	98348814	48877	2	1	2	0.02438896	0.30102999	3	1	0.51676182	0.05404976
chr1	98348814	98348873	60	1	1	2	0.02438896	0.1575501	3	2	0.51676182	0.1575501
chr1	98348873	98431803	82931	1	1	2	0.05404976	0.1575501	2	2	0.30102999	0.1575501
chr1	98431803	98746393	314591	5	1	1	0.05404976	0.1218695	2	1	0.30102999	0.1218695
chr1	98746393	98825244	78852	2	1	1	0.05404976	0.02438896	2	3	0.30102999	0.51676182
chr1	98825244	98942876	117633	1	1	1	0.05404976	0.05404976	2	2	0.30102999	0.30102999
chr1	98942876	99126777	183902	1	1	1	0.05404976	0.1218695	2	1	0.30102999	0.1218695
chr1	99126777	99176062	49286	2	2	1	0.1575501	0.1218695	2	1	0.1575501	0.1218695
chr1	99176062	99176121	60	1	2	1	0.1575501	0.05404976	2	2	0.1575501	0.30102999
chr1	99176121	99258146	82026	1	1	1	0.05404976	0.1218695	2	1	0.30102999	0.1218695
chr1	99258146	99530526	272381	4	0	1	0	0.1218695	2	1	0.61140001	0.1218695
chr1	99530526	99530585	60	1	1	1	0.02438896	0.1218695	3	1	0.51676182	0.1218695
chr1	99530585	99764554	233970	4	0	1	0	0.1218695	3	1	0.93173516	0.1218695
chr1	99764554	99810130	45577	2	1	2	0.01091641	0.30102999	4	1	0.76005302	0.05404976
chr1	99810130	99940579	130450	1	0	2	0	0.30102999	3	1	0.93173516	0.05404976
chr1	99940579	100031527	90949	1	0	2	0	0.30102999	2	1	0.61140001	0.05404976
chr1	100031527	100111307	79781	2	0	2	0	0.1575501	2	2	0.61140001	0.1575501
chr1	100111307	100111354	48	1	1	2	0.02438896	0.1575501	3	2	0.51676182	0.1575501
chr1	100111354	100389498	278145	6	0	2	0	0.1575501	3	2	0.93173516	0.1575501
chr1	100389498	100457204	67707	2	0	2	0	0.1575501	4	2	1.26272838	0.1575501
chr1	100457204	100489195	31992	2	0	2	0	0.08289318	4	3	1.26272838	0.30102999
chr1	100489195	100489249	55	1	0	3	0	0.17593012	5	3	1.60515106	0.17593012
chr1	100489249	100733749	244501	10	0	2	0	0.08289318	5	3	1.60515106	0.30102999
chr1	100733749	100978349	244601	6	0	2	0	0.08289318	4	3	1.26272838	0.30102999
chr1	100978349	101029348	51000	2	0	2	0	0.08289318	5	3	1.60515106	0.30102999
chr1	101029348	101128282	98935	2	0	2	0	0.1575501	4	2	1.26272838	0.1575501
chr1	101128282	101188756	60475	2	0	2	0	0.08289318	4	3	1.26272838	0.30102999
chr1	101188756	101271526	82771	3	0	3	0	0.17593012	4	3	1.26272838	0.17593012
chr1	101271526	101399663	128138	4	0	2	0	0.1575501	4	2	1.26272838	0.1575501
chr1	101399663	101456958	57296	1	0	2	0	0.1575501	3	2	0.93173516	0.1575501
chr1	101456958	101457017	60	1	0	2	0	0.08289318	3	3	0.93173516	0.30102999
chr1	101457017	101474231	17215	1	0	2	0	0.1575501	3	2	0.93173516	0.1575501
chr1	101474231	101503582	29352	3	0	2	0	0.1575501	4	2	1.26272838	0.1575501
chr1	101503582	101706477	202896	4	0	2	0	0.1575501	2	2	0.61140001	0.1575501
chr1	101706477	101952121	245645	2	0	1	0	0.1218695	2	1	0.61140001	0.1218695
chr1	101952121	102421982	469862	5	0	0	0	0	2	0	0.61140001	0
chr1	102421982	102422041	60	1	0	1	0	0.30102999	2	0	0.61140001	0
chr1	102422041	102491885	69845	1	0	0	0	0	2	0	0.61140001	0
chr1	102491885	102491944	60	1	0	0	0	0	2	1	0.61140001	0.30102999
chr1	102491944	102687379	195436	1	0	0	0	0	1	1	0.30102999	0.30102999
chr1	102687379	102687438	60	1	0	0	0	0	1	2	0.30102999	0.61140001
chr1	102687438	102893767	206330	2	0	0	0	0	1	0	0.30102999	0
chr1	102893767	103165390	271624	3	0	0	0	0	1	2	0.30102999	0.61140001
chr1	103165390	103165449	60	1	0	1	0	0.05404976	1	2	0.30102999	0.30102999
chr1	103165449	103282129	116681	1	0	1	0	0.1218695	1	1	0.30102999	0.1218695
chr1	103282129	103343285	61157	1	0	0	0	0	1	0	0.30102999	0
chr1	103343285	103363149	19865	2	0	0	0	0	2	0	0.61140001	0
chr1	103363149	103455085	91937	2	0	0	0	0	1	0	0.30102999	0
chr1	103455085	103455144	60	1	0	1	0	0.1218695	2	1	0.61140001	0.1218695
chr1	103455144	103573915	118772	3	0	1	0	0.1218695	1	1	0.30102999	0.1218695
chr1	103573915	104067184	493270	2	0	0	0	0	1	1	0.30102999	0.30102999
chr1	104067184	104124994	57811	4	0	1	0	0.1218695	1	1	0.30102999	0.1218695
chr1	104124994	104307708	182715	1	0	0	0	0	1	1	0.30102999	0.30102999
chr1	104852325	105300334	448010	4	0	0	0	0	1	0	0.30102999	0
chr1	106497293	107293664	796372	7	0	0	0	0	1	0	0.30102999	0
chr1	107293664	107343818	50155	2	1	1	0.1218695	0.30102999	1	0	0.1218695	0
chr1	107343818	107783310	439493	8	1	1	0.05404976	0.30102999	2	0	0.30102999	0
chr1	107783310	107901351	118042	3	0	1	0	0.30102999	2	0	0.61140001	0
chr1	107901351	107901410	60	1	0	1	0	0.1218695	2	1	0.61140001	0.1218695
chr1	107901410	108188693	287284	4	0	1	0	0.30102999	2	0	0.61140001	0
chr1	108188693	108505036	316344	7	0	1	0	0.1218695	2	1	0.61140001	0.1218695
chr1	108505036	108584295	79260	1	0	1	0	0.30102999	2	0	0.61140001	0
chr1	108584295	108584354	60	1	0	2	0	0.30102999	2	1	0.61140001	0.05404976
chr1	108584354	108703829	119476	2	0	1	0	0.1218695	2	1	0.61140001	0.1218695
chr1	108703829	108724614	20786	2	0	2	0	0.1575501	2	2	0.61140001	0.1575501
chr1	108724614	108811085	86472	1	0	2	0	0.30102999	2	1	0.61140001	0.05404976
chr1	108811085	108879235	68151	1	0	1	0	0.1218695	2	1	0.61140001	0.1218695
chr1	108879235	109023411	144177	3	0	1	0	0.05404976	2	2	0.61140001	0.30102999
chr1	109023411	109146143	122733	3	0	3	0	0.30102999	2	2	0.61140001	0.08289318
chr1	109146143	109290716	144574	5	1	3	0.05404976	0.30102999	2	2	0.30102999	0.08289318
chr1	109290716	109472445	181730	7	1	3	0.1218695	0.30102999	1	2	0.1218695	0.08289318
chr1	109472445	109479276	6832	2	1	3	0.05404976	0.30102999	2	2	0.30102999	0.08289318
chr1	109479276	109563696	84421	2	1	3	0.05404976	0.51676182	2	1	0.30102999	0.02438896
chr1	109563696	109666050	102355	3	1	3	0.02438896	0.51676182	3	1	0.51676182	0.02438896
chr1	109666050	109779638	113589	6	1	3	0.05404976	0.51676182	2	1	0.30102999	0.02438896
chr1	109779638	109779697	60	1	1	3	0.02438896	0.17593012	3	3	0.51676182	0.17593012
chr1	109779697	109856331	76635	3	1	2	0.02438896	0.1575501	3	2	0.51676182	0.1575501
chr1	109856331	109878897	22567	2	1	2	0.02438896	0.08289318	3	3	0.51676182	0.30102999
chr1	109878897	109922325	43429	1	1	2	0.02438896	0.1575501	3	2	0.51676182	0.1575501
chr1	109922325	109922384	60	1	2	2	0.08289318	0.1575501	3	2	0.30102999	0.1575501
chr1	109922384	110023272	100889	2	1	2	0.02438896	0.1575501	3	2	0.51676182	0.1575501
chr1	110023272	110023330	59	1	2	3	0.08289318	0.30102999	3	2	0.30102999	0.08289318
chr1	110023330	110083989	60660	2	1	3	0.02438896	0.30102999	3	2	0.51676182	0.08289318
chr1	110083989	110084048	60	1	2	3	0.08289318	0.30102999	3	2	0.30102999	0.08289318
chr1	110084048	110148918	64871	2	2	2	0.08289318	0.1575501	3	2	0.30102999	0.1575501
chr1	110148918	110200584	51667	3	2	2	0.0429175	0.1575501	4	2	0.47744371	0.1575501
chr1	110200584	110464566	263983	11	2	2	0.08289318	0.1575501	3	2	0.30102999	0.1575501
chr1	110464566	110562564	97999	2	1	2	0.05404976	0.1575501	2	2	0.30102999	0.1575501
chr1	110562564	110562623	60	1	1	2	0.02438896	0.1575501	3	2	0.51676182	0.1575501
chr1	110562623	110686853	124231	3	1	2	0.05404976	0.1575501	2	2	0.30102999	0.1575501
chr1	110686853	110742010	55158	2	1	2	0.02438896	0.1575501	3	2	0.51676182	0.1575501
chr1	110742010	110775874	33865	1	1	2	0.05404976	0.30102999	2	1	0.30102999	0.05404976
chr1	110775874	110845020	69147	1	1	1	0.05404976	0.1218695	2	1	0.30102999	0.1218695
chr1	110845020	110888005	42986	2	1	1	0.05404976	0.05404976	2	2	0.30102999	0.30102999
chr1	110888005	110906455	18451	2	1	1	0.05404976	0.02438896	2	3	0.30102999	0.51676182
chr1	110906455	110906514	60	1	1	1	0.05404976	0.01091641	2	4	0.30102999	0.76005302
chr1	110906514	110994884	88371	2	0	1	0	0.02438896	2	3	0.61140001	0.51676182
chr1	110994884	111059875	64992	1	0	1	0	0.05404976	2	2	0.61140001	0.30102999
chr1	111059875	111170598	110724	4	0	2	0	0.1575501	2	2	0.61140001	0.1575501
chr1	111170598	111310390	139793	4	0	3	0	0.30102999	2	2	0.61140001	0.08289318
chr1	111310390	111440417	130028	2	0	3	0	0.51676182	2	1	0.61140001	0.02438896
chr1	111440417	111440471	55	1	0	3	0	0.30102999	2	2	0.61140001	0.08289318
chr1	111440471	111495549	55079	1	0	3	0	0.51676182	2	1	0.61140001	0.02438896
chr1	111495549	111557895	62347	3	0	3	0	0.30102999	2	2	0.61140001	0.08289318
chr1	111557895	111557954	60	1	0	3	0	0.17593012	2	3	0.61140001	0.17593012
chr1	111557954	111782671	224718	10	0	3	0	0.30102999	2	2	0.61140001	0.08289318
chr1	111782671	111891163	108493	3	0	3	0	0.51676182	2	1	0.61140001	0.02438896
chr1	111891163	111891222	60	1	0	3	0	0.30102999	2	2	0.61140001	0.08289318
chr1	111891222	112021063	129842	5	0	3	0	0.51676182	2	1	0.61140001	0.02438896
chr1	112021063	112279145	258083	9	0	3	0	0.30102999	2	2	0.61140001	0.08289318
chr1	112279145	112468708	189564	6	0	3	0	0.17593012	2	3	0.61140001	0.17593012
chr1	112468708	112633589	164882	3	0	2	0	0.1575501	2	2	0.61140001	0.1575501
chr1	112633589	112633648	60	1	0	2	0	0.08289318	2	3	0.61140001	0.30102999

chr1	112633648	112845790	212143	2	0	2	0	0.1575501	2	2	0.61140001	0.1575501
chr1	112845790	113005492	159703	3	0	2	0	0.1575501	1	2	0.30102999	0.1575501
chr1	113005492	113005539	48	1	1	2	0.1218695	0.1575501	1	2	0.1218695	0.1575501
chr1	113005539	113066745	61207	3	0	2	0	0.1575501	1	2	0.30102999	0.1575501
chr1	113066745	113153434	86690	3	0	2	0	0.30102999	1	1	0.30102999	0.05404976
chr1	113153434	113177892	24459	2	0	2	0	0.30102999	2	1	0.61140001	0.05404976
chr1	113177892	113709879	531988	16	0	2	0	0.1575501	2	2	0.61140001	0.1575501
chr1	113709879	114012621	302743	4	0	3	0	0.30102999	2	2	0.61140001	0.08289318
chr1	114012621	114012680	60	1	0	3	0	0.30102999	3	2	0.93173516	0.08289318
chr1	114012680	114092245	79566	2	0	3	0	0.30102999	2	2	0.61140001	0.08289318
chr1	114092245	114185035	92791	2	0	2	0	0.1575501	2	2	0.61140001	0.1575501
chr1	114185035	114280830	95796	4	0	3	0	0.17593012	2	3	0.61140001	0.17593012
chr1	114280830	114348386	67557	2	0	3	0	0.30102999	2	2	0.61140001	0.08289318
chr1	114348386	114348445	60	1	0	3	0	0.17593012	2	3	0.61140001	0.17593012
chr1	114348445	114412437	63993	2	0	3	0	0.30102999	2	2	0.61140001	0.08289318
chr1	114412437	114412496	60	1	0	3	0	0.17593012	3	3	0.93173516	0.17593012
chr1	114412496	114523716	111221	7	0	3	0	0.30102999	3	2	0.93173516	0.08289318
chr1	114523716	114633644	109929	1	0	3	0	0.30102999	2	2	0.61140001	0.08289318
chr1	114633644	114633703	60	1	0	3	0	0.17593012	2	3	0.61140001	0.17593012
chr1	114633703	114687535	53833	1	0	3	0	0.17593012	1	3	0.30102999	0.17593012
chr1	114687535	114831001	143467	1	0	3	0	0.30102999	1	2	0.30102999	0.08289318
chr1	114831001	114942064	111064	2	0	3	0	0.17593012	2	3	0.61140001	0.17593012
chr1	114942064	115121131	179068	6	0	3	0	0.05670724	2	5	0.61140001	0.45545077
chr1	115121131	115148610	27480	1	0	3	0	0.10122019	2	4	0.61140001	0.30102999
chr1	115148610	115398406	249797	9	0	3	0	0.10122019	3	4	0.93173516	0.30102999
chr1	115398406	115489021	90616	4	0	3	0	0.05670724	3	5	0.93173516	0.45545077
chr1	115489021	115621914	132894	4	0	3	0	0.10122019	3	4	0.93173516	0.30102999
chr1	115621914	115874785	252872	5	0	2	0	0.0429175	3	4	0.93173516	0.47744371
chr1	115874785	116235824	361040	7	0	3	0	0.10122019	3	4	0.93173516	0.30102999
chr1	116235824	116270823	35000	3	1	3	0.02438896	0.10122019	3	4	0.51676182	0.30102999
chr1	116270823	116677525	406703	9	1	3	0.02438896	0.05670724	3	5	0.51676182	0.45545077
chr1	116677525	117147182	469658	11	1	3	0.02438896	0.10122019	3	4	0.51676182	0.30102999
chr1	117147182	117207200	60019	1	1	3	0.05404976	0.10122019	2	4	0.30102999	0.30102999
chr1	117207200	117269845	62646	3	1	3	0.05404976	0.05670724	2	5	0.30102999	0.45545077
chr1	117269845	117315364	45520	2	1	3	0.05404976	0.10122019	2	4	0.30102999	0.30102999
chr1	117315364	117624672	309309	6	1	3	0.05404976	0.05670724	2	5	0.30102999	0.45545077
chr1	117624672	117775388	150717	5	1	3	0.02438896	0.05670724	3	5	0.51676182	0.45545077
chr1	117775388	118003260	227873	4	1	3	0.02438896	0.10122019	3	4	0.51676182	0.30102999
chr1	118003260	118168804	165545	6	1	3	0.02438896	0.17593012	3	3	0.51676182	0.17593012
chr1	118168804	118344509	175706	3	0	3	0	0.17593012	3	3	0.93173516	0.17593012
chr1	118344509	118344568	60	1	0	3	0	0.10122019	3	4	0.93173516	0.30102999
chr1	118344568	118420090	75523	2	0	2	0	0.08289318	3	3	0.93173516	0.30102999
chr1	118420090	118420149	60	1	0	2	0	0.0429175	3	4	0.93173516	0.47744371
chr1	118420149	118502389	82241	4	0	1	0	0.02438896	3	3	0.93173516	0.51676182
chr1	118502389	118571048	68660	1	0	1	0	0.05404976	3	2	0.93173516	0.30102999
chr1	118571048	118630796	59749	1	0	1	0	0.05404976	2	2	0.61140001	0.30102999
chr1	118630796	118733871	103076	3	0	1	0	0.02438896	2	3	0.61140001	0.51676182
chr1	118733871	118789436	55566	2	0	1	0	0.01091641	2	4	0.61140001	0.76005302
chr1	118789436	118789495	60	1	1	1	0.05404976	0.01091641	2	4	0.30102999	0.76005302
chr1	118789495	118983652	194158	2	0	1	0	0.02438896	2	3	0.61140001	0.51676182
chr1	118983652	119148222	164571	1	0	1	0	0.05404976	2	2	0.61140001	0.30102999
chr1	119148222	119148281	60	1	0	1	0	0.05404976	3	2	0.93173516	0.30102999
chr1	119148281	119261982	113702	1	0	1	0	0.05404976	2	2	0.61140001	0.30102999
chr1	119261982	119367354	105373	2	0	1	0	0.02438896	2	3	0.61140001	0.51676182
chr1	119367354	119419142	51789	1	0	1	0	0.05404976	2	2	0.61140001	0.30102999
chr1	119419142	119491487	72346	2	1	1	0.05404976	0.05404976	2	2	0.30102999	0.30102999
chr1	119491487	119688977	197491	5	1	1	0.05404976	0.02438896	2	3	0.30102999	0.51676182
chr1	119688977	119689036	60	1	1	1	0.01091641	0.02438896	4	3	0.76005302	0.51676182
chr1	119689036	119800039	111004	2	1	0	0.05404976	0	2	3	0.30102999	0.93173516
chr1	119800039	119873476	73438	2	1	0	0.02438896	0	3	3	0.51676182	0.93173516
chr1	119873476	120164940	291465	7	1	0	0.05404976	0	2	3	0.30102999	0.93173516
chr1	120164940	120270940	106001	2	1	0	0.05404976	0	2	2	0.30102999	0.61140001
chr1	120270940	120307096	36157	3	1	0	0.02438896	0	3	3	0.51676182	0.93173516
chr1	120307096	120351499	44404	4	1	1	0.02438896	0.02438896	3	3	0.51676182	0.51676182
chr1	120351499	120414981	63483	1	1	0	0.02438896	0	3	2	0.51676182	0.61140001
chr1	120414981	120585364	170384	5	1	0	0.05404976	0	2	2	0.30102999	0.61140001
chr1	120585364	120585423	60	1	2	0	0.1575501	0	2	2	0.1575501	0.61140001
chr1	120585423	121184327	598905	1	1	0	0.05404976	0	2	0	0.30102999	0
chr1	121184327	144927778	23743452	2	0	0	0	0	1	0	0.30102999	0
chr1	144927778	145291770	363993	9	9	0	0.76806864	0	5	0	0.02473314	0
chr1	145291770	145663189	371420	16	8	0	0.58747015	0	5	0	0.04407651	0
chr1	145663189	145663248	60	1	9	0	0.5732208	0	6	0	0.04875589	0
chr1	145663248	145715602	52355	2	8	0	0.58747015	0	5	0	0.04407651	0
chr1	145715602	145740598	24997	2	8	0	0.4250187	0	6	0	0.08122616	0
chr1	145740598	145747269	6672	2	9	0	0.5732208	0	6	0	0.04875589	0
chr1	145747269	146324068	576800	1	9	0	0.76806864	0	5	0	0.02473314	0
chr1	146324068	146347155	23088	2	13	0	0.30102999	0	12	0	0.14385241	0
chr1	146347155	146564802	217648	1	12	0	0.41314172	0	10	0	0.09290028	0
chr1	146564802	146646378	81577	2	10	0	0.21118145	0	10	0	0.21118145	0
chr1	146646378	146696665	50288	2	6	0	0.04875589	0	9	0	0.5732208	0
chr1	146696665	146696722	58	1	7	0	0.08584816	0	9	0	0.42015402	0
chr1	146696722	146738716	41995	1	6	0	0.04875589	0	9	0	0.5732208	0
chr1	146738716	146738775	60	1	7	0	0.08584816	0	9	0	0.42015402	0
chr1	146738775	146807433	68659	4	6	0	0.08122616	0	8	0	0.4250187	0
chr1	146807433	147356575	549143	13	8	0	0.13872638	0	9	0	0.30102999	0
chr1	147356575	147745551	388977	2	9	0	0.20975986	0	9	0	0.20975986	0
chr1	147745551	147786647	41097	2	9	0	0.14135546	0	10	0	0.30102999	0
chr1	147786647	147786706	60	1	10	0	0.14303407	0	11	0	0.30102999	0
chr1	147786706	149223984	1437279	4	3	0	0.17593012	0	3	0	0.17593012	0
chr1	149223984	149815145	591162	2	5	0	0.19510895	0	5	0	0.19510895	0
chr1	149815145	149973911	158767	12	16	1	0.91308053	0.30102999	11	0	0.01767679	0
chr1	149973911	150125741	151831	5	17	1	1.14735594	0.30102999	11	0	0.00859896	0
chr1	150125741	150192197	66457	2	17	1	0.92532268	0.30102999	12	0	0.01667721	0
chr1	150192197	150224224	32028	4	17	1	0.56314362	0.30102999	14	0	0.05232577	0
chr1	150224224	150238254	14031	2	17	1	0.73110763	0.30102999	13	0	0.03037338	0
chr1	150238254	150386632	148379	8	17	1	0.56314362	0.30102999	14	0	0.05232577	0
chr1	150386632	150529388	142757	7	17	1	0.42015402	0.30102999	15	0	0.08584816	0
chr1	150529388	150600916	71529	3	17	1	0.30102999	0.30102999	16	0	0.13499366	0
chr1	150600916	150771641	170726	4	17	1	0.42015402	0.30102999	15	0	0.08584816	0
chr1	150771641	150799043	27403	2	18	1	0.5732208	0.30102999	15	0	0.04875589	0
chr1	150799043	150808990	9948	2	18	1	0.4250187	0.30102999	16	0	0.08122616	0
chr1	150808990	150846585	37596	2	18	1	0.5732208	0.30102999	15	0	0.04875589	0
chr1	150846585	150846644	60	1	18	1	0.4250187	0.30102999	16	0	0.08122616	0
chr1	150846644	151023409	176766	10	18	1	0.5732208	0.30102999	15	0	0.04875589	0
chr1	151023409	151239239	215831	14	18	1	0.4250187	0.30102999	16	0	0.08122616	0
chr1	151239239	151298195	58957	2	18	1	0.5732208	0.30102999	15	0	0.04875589	0
chr1	151298195	151298254	60	1	18	1	0.4250187	0.30102999	16	0	0.08122616	0
chr1	151298254	151467919	169666	7	18	0	0.4250187	0	16	0	0.08122616	0
chr1	151467919	151669579	201661	7	18	0	0.74627054	0	14	0	0.02793176	0
chr1	151669579	151678270	8692	2	18	0	0.5732208	0	15	0	0.04875589	0
chr1	151678270	151680659	2390	2	18	0	0.4250187	0	16	0	0.08122616	0
chr1	151680659	151780354	99696	5	18	0	0.5732208	0	15	0	0.04875589	0
chr1	151780354	152069453	289100	9	18	0	0.4250187	0	16	0	0.08122616	0
chr1	152069453	152069512	60	1	18	0	0.30102999	0	17	0	0.129913	0
chr1	152069512	152127131	57620	2	18	0	0.4250187	0	16	0	0.08122616	0
chr1	152127131	152127608	478	2	18	0	0.30102999	0	17	0	0.129913	0
chr1	152127608	152200889	73282	1	18	0	0.4250187	0	16	0	0.08122616	0
chr1	152200889	152330164	129276	2	18	0	0.5732208	0	15	0	0.04875589	0
chr1	152330164	152330223	60	1	19	0	0.76806864	0	15	0	0.02473314	0
chr1	152330223	152484439	154217	2	18	0	0.5732208	0	15	0	0.04875589	0
chr1	152484439	152487632	3194	2	20	0	1.01542894	0	15	0	0.01077081	0
chr1	152487632	152595156	107525	4	19	0	0.97390707	0	14	0	0.01320236	0
chr1	152595156	152748629	153474	7	18	0	0.74627054	0	14	0	0.02793176	0
chr1	152748629	152778094	29466	3	19	0	0.97390707	0	14	0	0.01320236	0

chr1	152778094	152822767	44674	3	18	0	0.74627054	0	14	0	0.02793176	0
chr1	152822767	152882381	59615	3	19	0	0.97390707	0	14	0	0.01320236	0
chr1	152882381	152957573	75193	2	17	0	0.56314362	0	14	0	0.05232577	0
chr1	152957573	153113473	155901	6	18	0	0.74627054	0	14	0	0.02793176	0
chr1	153113473	153281469	167997	3	17	0	0.56314362	0	14	0	0.05232577	0
chr1	153281469	153390393	108925	7	17	0	0.42015402	0	15	0	0.08584816	0
chr1	153390393	153431653	41261	2	18	0	0.5732208	0	15	0	0.04875589	0
chr1	153431653	153587847	156195	2	17	0	0.42015402	0	15	0	0.08584816	0
chr1	153587847	153613593	25747	2	17	0	0.56314362	0	14	0	0.05232577	0
chr1	153613593	153653756	40164	6	18	0	0.5732208	0	15	0	0.04875589	0
chr1	153653756	153751465	97710	2	16	0	0.30102999	0	15	0	0.13872638	0
chr1	153751465	153780441	28977	2	17	0	0.42015402	0	15	0	0.08584816	0
chr1	153780441	153780500	60	1	18	0	0.5732208	0	15	0	0.04875589	0
chr1	153780500	153911364	130865	2	17	0	0.42015402	0	15	0	0.08584816	0
chr1	153911364	154244969	333606	18	18	0	0.5732208	0	15	0	0.04875589	0
chr1	154244969	154556368	311400	12	19	0	0.76806864	0	15	0	0.02473314	0
chr1	154556368	154668883	112516	2	18	0	0.5732208	0	15	0	0.04875589	0
chr1	154668883	154768680	99798	3	19	0	0.76806864	0	15	0	0.02473314	0
chr1	154768680	154841535	72856	1	18	0	0.5732208	0	15	0	0.04875589	0
chr1	154841535	154841591	57	1	18	1	0.5732208	0.30102999	15	0	0.04875589	0
chr1	154841591	154874822	33232	1	17	1	0.42015402	0.30102999	15	0	0.08584816	0
chr1	154874822	154905221	30400	2	18	1	0.5732208	0.30102999	15	0	0.04875589	0
chr1	154905221	154950080	44860	8	19	1	0.76806864	0.30102999	15	0	0.02473314	0
chr1	154950080	154956259	6180	3	20	1	1.01542894	0.30102999	15	0	0.01077081	0
chr1	154956259	154966445	10187	2	17	0	0.42015402	0	15	0	0.08584816	0
chr1	154966445	154966497	53	1	20	0	1.01542894	0	15	0	0.01077081	0
chr1	154966497	155002715	36219	2	19	0	0.76806864	0	15	0	0.02473314	0
chr1	155002715	155041934	39220	3	18	0	0.5732208	0	15	0	0.04875589	0
chr1	155041934	155102460	60527	4	20	1	1.01542894	0.30102999	15	0	0.01077081	0
chr1	155102460	155105978	3519	3	20	1	0.79906872	0.30102999	16	0	0.02074938	0
chr1	155105978	155150156	44179	2	19	1	0.58747015	0.30102999	16	0	0.04407651	0
chr1	155150156	155160541	10386	2	20	1	0.79906872	0.30102999	16	0	0.02074938	0
chr1	155160541	155179763	19223	2	20	0	0.79906872	0	16	0	0.02074938	0
chr1	155179763	155223256	43494	2	20	1	0.79906872	0.30102999	16	0	0.02074938	0
chr1	155223256	155296964	73709	8	21	1	1.07548421	0.30102999	16	0	0.0079614	0
chr1	155296964	155301141	4178	2	21	1	0.84395715	0.30102999	17	0	0.01598258	0
chr1	155301141	155451995	150855	4	21	1	1.07548421	0.30102999	16	0	0.0079614	0
chr1	155451995	155490934	38940	2	21	1	0.84395715	0.30102999	17	0	0.01598258	0
chr1	155490934	155583172	92239	2	21	1	1.07548421	0.30102999	16	0	0.0079614	0
chr1	155583172	155618638	35467	2	21	1	0.84395715	0.30102999	17	0	0.01598258	0
chr1	155618638	155676280	57643	2	21	1	0.63695542	0.30102999	18	0	0.03070643	0
chr1	155676280	155725479	49200	1	21	1	0.84395715	0.30102999	17	0	0.01598258	0
chr1	155725479	155823553	98075	3	20	1	0.60763643	0.30102999	17	0	0.03812622	0
chr1	155823553	155834366	10814	2	20	1	0.44141547	0.30102999	18	0	0.06713722	0
chr1	155834366	155854920	20555	2	21	1	0.63695542	0.30102999	18	0	0.03070643	0
chr1	155854920	155878883	23964	1	21	1	0.84395715	0.30102999	17	0	0.01598258	0
chr1	155878883	155891663	12781	1	20	1	0.60763643	0.30102999	17	0	0.03812622	0
chr1	155891663	155912396	20734	3	20	1	0.44141547	0.30102999	18	0	0.06713722	0
chr1	155912396	156035336	122941	6	20	1	0.60763643	0.30102999	17	0	0.03812622	0
chr1	156035336	156087914	52579	2	19	1	0.43181735	0.30102999	17	0	0.07511598	0
chr1	156087914	156102366	14453	2	20	1	0.60763643	0.30102999	17	0	0.03812622	0
chr1	156102366	156226345	123980	6	19	1	0.43181735	0.30102999	17	0	0.07511598	0
chr1	156226345	156345455	119111	7	18	1	0.30102999	0.30102999	17	0	0.129913	0
chr1	156345455	156495512	150058	5	19	1	0.43181735	0.30102999	17	0	0.07511598	0
chr1	156495512	156590310	94799	4	19	0	0.43181735	0	17	0	0.07511598	0
chr1	156590310	156643483	53174	4	19	0	0.30102999	0	18	0	0.12309572	0
chr1	156643483	156718114	74632	5	19	0	0.43181735	0	17	0	0.07511598	0
chr1	156718114	156764562	46449	1	18	0	0.30102999	0	17	0	0.129913	0
chr1	156764562	156764620	59	1	18	0	0.20064824	0	18	0	0.20064824	0
chr1	156764620	156786539	21920	2	18	0	0.30102999	0	17	0	0.129913	0
chr1	156786539	156826196	39658	2	19	0	0.43181735	0	17	0	0.07511598	0
chr1	156826196	156831829	5634	2	19	0	0.30102999	0	18	0	0.12309572	0
chr1	156831829	156902854	71026	4	19	0	0.43181735	0	17	0	0.07511598	0
chr1	156902854	156931500	28647	2	19	0	0.30102999	0	18	0	0.12309572	0
chr1	156931500	157014484	82985	3	18	0	0.20064824	0	18	0	0.20064824	0
chr1	157014484	157073703	59220	1	17	0	0.30102999	0	16	0	0.13499366	0
chr1	157073703	157136656	62954	4	16	0	0.20764654	0	16	0	0.20764654	0
chr1	157136656	157522226	385571	5	15	0	0.13872638	0	16	0	0.30102999	0
chr1	157522226	157588107	65882	5	14	0	0.08923391	0	16	0	0.4167287	0
chr1	157588107	157805710	217604	5	14	0	0.14135546	0	15	0	0.30102999	0
chr1	157805710	158027224	221515	4	13	0	0.09154957	0	15	0	0.41444892	0
chr1	158027224	158027283	60	1	14	0	0.14135546	0	15	0	0.30102999	0
chr1	158027283	158056876	29594	1	14	0	0.21118145	0	14	0	0.21118145	0
chr1	158056876	158122085	65210	1	14	0	0.30102999	0	13	0	0.14303407	0
chr1	158122085	158224393	102309	2	13	0	0.30102999	0	12	0	0.14385241	0
chr1	158224393	158390898	166506	6	14	0	0.30102999	0	13	0	0.14303407	0
chr1	158390898	158488089	97192	2	14	0	0.41314172	0	12	0	0.09290028	0
chr1	158488089	158488148	60	1	14	0	0.30102999	0	13	0	0.14303407	0
chr1	158488148	158538605	50458	1	14	0	0.41314172	0	12	0	0.09290028	0
chr1	158538605	158720740	182136	5	14	0	0.5498098	0	11	0	0.0574087	0
chr1	158720740	158720799	60	1	14	0	0.30102999	0	13	0	0.14303407	0
chr1	158720799	158801272	80474	2	14	0	0.5498098	0	11	0	0.0574087	0
chr1	158801272	158871944	70673	2	14	0	0.41314172	0	12	0	0.09290028	0
chr1	158871944	158969905	97962	3	14	0	0.21118145	0	14	0	0.21118145	0
chr1	158969905	159033336	63432	4	14	1	0.14135546	0.30102999	15	0	0.30102999	0
chr1	159033336	159033395	60	1	15	1	0.13872638	0.30102999	16	0	0.30102999	0
chr1	159033395	159109758	76364	1	15	1	0.20975986	0.30102999	15	0	0.20975986	0
chr1	159109758	159109817	60	1	15	1	0.13872638	0.30102999	16	0	0.30102999	0
chr1	159109817	159153841	44025	1	14	1	0.08923391	0.30102999	16	0	0.4167287	0
chr1	159153841	159175344	21504	3	15	1	0.08584816	0.30102999	17	0	0.42015402	0
chr1	159175344	159245116	69773	1	14	1	0.05232577	0.30102999	17	0	0.56314362	0
chr1	159245116	159272127	27012	1	13	1	0.05490675	0.30102999	16	0	0.55623409	0
chr1	159272127	159338089	65963	1	13	1	0.09154957	0.30102999	15	0	0.41444892	0
chr1	159338089	159374865	36777	2	15	1	0.13872638	0.30102999	16	0	0.30102999	0
chr1	159374865	159410021	35157	2	15	1	0.08584816	0.30102999	17	0	0.42015402	0
chr1	159410021	159449736	39716	1	15	1	0.13872638	0.30102999	16	0	0.30102999	0
chr1	159449736	159552255	102520	2	14	1	0.08923391	0.30102999	16	0	0.4167287	0
chr1	159552255	159611998	59744	2	14	1	0.05232577	0.30102999	17	0	0.56314362	0
chr1	159611998	159612057	60	1	15	1	0.08584816	0.30102999	17	0	0.42015402	0
chr1	159612057	159752080	140024	5	15	1	0.13872638	0.30102999	16	0	0.30102999	0
chr1	159752080	159797580	45501	2	15	1	0.04875589	0.30102999	18	0	0.5732208	0
chr1	159797580	159922706	125127	5	15	1	0.08584816	0.30102999	17	0	0.42015402	0
chr1	159922706	159924051	1346	2	15	1	0.04875589	0.30102999	18	0	0.5732208	0
chr1	159924051	160152793	228743	8	15	1	0.08584816	0.30102999	17	0	0.42015402	0
chr1	160152793	160152851	59	1	15	1	0.04875589	0.30102999	18	0	0.5732208	0
chr1	160152851	160214263	61413	3	15	1	0.08584816	0.30102999	17	0	0.42015402	0
chr1	160214263	160231172	16910	2	15	1	0.04875589	0.30102999	18	0	0.5732208	0
chr1	160231172	160295340	64169	3	15	1	0.08584816	0.30102999	17	0	0.42015402	0
chr1	160295340	160580343	285004	11	15	1	0.04875589	0.30102999	18	0	0.5732208	0
chr1	160580343	160580401	59	1	15	1	0.02473314	0.30102999	19	0	0.76806864	0
chr1	160580401	160653787	73387	2	14	1	0.01320236	0.30102999	19	0	0.97390707	0
chr1	160653787	160676627	22841	2	15	1	0.02473314	0.30102999	19	0	0.76806864	0
chr1	160676627	160719255	42629	1	14	1	0.01320236	0.30102999	19	0	0.97390707	0
chr1	160719255	160751516	32262	1	14	1	0.02793176	0.30102999	18	0	0.74627054	0
chr1	160751516	160751575	60	1	15	1	0.04875589	0.30102999	18	0	0.5732208	0
chr1	160751575	160825968	74394	3	13	1	0.03037338	0.30102999	17	0	0.73110763	0
chr1	160825968	160826027	60	1	14	1	0.01320236	0.30102999	19	0	0.97390707	0
chr1	160826027	160848510	22484	1	13	1	0.03037338	0.30102999	17	0	0.73110763	0
chr1	160848510	160917695	69186	2	14	1	0.05232577	0.30102999	17	0	0.56314362	0
chr1	160917695	160966739	49045	3	14	1	0.02793176	0.30102999	18	0	0.74627054	0
chr1	160966739	161201012	234274	18	13	1	0.01518174	0.30102999	18	0	0.9449689	0
chr1	161201012	161253825	52814	1	13	0	0.01518174	0	18	0	0.9449689	0
chr1	161253825	161291124	37300	3	14	0	0.02793176	0	18	0	0.74627054	0
chr1	161291124	161331897	40774	1	14	0	0.08923391	0	16	0	0.4167287	0

chr1	161331897	161479451	147555	4	15	0	0.13872638	0	16	0	0.30102999	0
chr1	161479451	161618904	139454	1	14	0	0.14135546	0	15	0	0.30102999	0
chr1	161618904	161722268	103365	4	14	0	0.21118145	0	14	0	0.21118145	0
chr1	161722268	161816271	94004	2	13	0	0.14303407	0	14	0	0.30102999	0
chr1	161816271	161882105	65835	1	13	0	0.21200206	0	13	0	0.21200206	0
chr1	161882105	162053488	171384	4	13	0	0.14303407	0	14	0	0.30102999	0
chr1	162053488	162175443	121956	4	14	0	0.14135546	0	15	0	0.30102999	0
chr1	162175443	162175502	60	1	15	0	0.20975986	0	15	0	0.20975986	0
chr1	162175502	162257127	81626	2	12	0	0.09290028	0	14	0	0.41314172	0
chr1	162257127	162492953	235827	9	14	0	0.21118145	0	14	0	0.21118145	0
chr1	162492953	162681021	188069	6	15	0	0.20975986	0	15	0	0.20975986	0
chr1	162681021	162703368	22348	2	15	0	0.13872638	0	16	0	0.30102999	0
chr1	162703368	162703427	60	1	16	0	0.20764654	0	16	0	0.20764654	0
chr1	162703427	162743293	39867	1	15	0	0.13872638	0	16	0	0.30102999	0
chr1	162743293	162832502	89210	2	14	0	0.08923391	0	16	0	0.4167287	0
chr1	162832502	163257049	424548	6	14	0	0.14135546	0	15	0	0.30102999	0
chr1	163257049	163298084	41036	2	13	0	0.14303407	0	14	0	0.30102999	0
chr1	163298084	163379594	81511	2	12	0	0.30102999	0	11	0	0.14385241	0
chr1	163379594	163379653	60	1	12	0	0.21227046	0	12	0	0.21227046	0
chr1	163379653	163574086	194434	3	11	0	0.14385241	0	12	0	0.30102999	0
chr1	163574086	163574145	60	1	13	0	0.14303407	0	14	0	0.30102999	0
chr1	163574145	163692170	118026	1	13	0	0.21200206	0	13	0	0.21200206	0
chr1	163692170	163692229	60	1	14	0	0.14135546	0	15	0	0.30102999	0
chr1	163692229	163811431	119203	2	13	0	0.14303407	0	14	0	0.30102999	0
chr1	163811431	163811490	60	1	14	0	0.14135546	0	15	0	0.30102999	0
chr1	163811490	163887005	75516	1	13	0	0.14303407	0	14	0	0.30102999	0
chr1	163887005	163917093	30089	1	13	0	0.21200206	0	13	0	0.21200206	0
chr1	163917093	163917152	60	1	15	0	0.30102999	0	14	0	0.14135546	0
chr1	163917152	164005836	88685	1	15	0	0.41444892	0	13	0	0.09154957	0
chr1	164005836	164005895	60	1	15	0	0.20975986	0	15	0	0.20975986	0
chr1	164005895	164102192	96298	1	15	0	0.30102999	0	14	0	0.14135546	0
chr1	164102192	164152825	50634	1	13	0	0.14303407	0	14	0	0.30102999	0
chr1	164152825	164204881	52057	1	13	0	0.30102999	0	12	0	0.14385241	0
chr1	164204881	164279155	74275	1	12	0	0.21227046	0	12	0	0.21227046	0
chr1	164279155	164330973	51819	2	13	0	0.30102999	0	12	0	0.14385241	0
chr1	164330973	164394122	63150	2	13	0	0.21200206	0	13	0	0.21200206	0
chr1	164394122	164532450	138329	2	12	0	0.14385241	0	13	0	0.30102999	0
chr1	164532450	164532509	60	1	13	0	0.14303407	0	14	0	0.30102999	0
chr1	164532509	164584717	52209	1	11	0	0.0574087	0	14	0	0.5498098	0
chr1	164584717	164584776	60	1	11	0	0.0331093	0	15	0	0.71538971	0
chr1	164584776	164611232	26457	1	11	0	0.0574087	0	14	0	0.5498098	0
chr1	164611232	164643942	32711	1	10	0	0.03344703	0	14	0	0.71353304	0
chr1	164643942	164643995	54	1	11	0	0.0331093	0	15	0	0.71538971	0
chr1	164643995	164697258	53264	1	11	0	0.09334429	0	13	0	0.41271556	0
chr1	164697258	164761881	64624	1	11	0	0.14385241	0	12	0	0.30102999	0
chr1	164761881	164904489	142609	3	11	0	0.09334429	0	13	0	0.41271556	0
chr1	164904489	164982393	77905	2	13	0	0.21200206	0	13	0	0.21200206	0
chr1	164982393	165280442	298050	5	13	0	0.30102999	0	12	0	0.14385241	0
chr1	165280442	165319618	39177	2	13	0	0.21200206	0	13	0	0.21200206	0
chr1	165319618	165739928	420311	12	13	0	0.41271556	0	11	0	0.09334429	0
chr1	165739928	165801395	61468	1	12	0	0.30102999	0	11	0	0.14385241	0
chr1	165801395	165823211	21817	1	12	0	0.41314172	0	10	0	0.09290028	0
chr1	165823211	165904815	81605	2	12	0	0.30102999	0	11	0	0.14385241	0
chr1	165904815	166270637	365823	7	12	0	0.21227046	0	12	0	0.21227046	0
chr1	166270637	166482444	211808	2	11	0	0.14385241	0	12	0	0.30102999	0
chr1	166482444	166482503	60	1	12	0	0.21227046	0	12	0	0.21227046	0
chr1	166482503	166664028	181526	4	11	0	0.14385241	0	12	0	0.30102999	0
chr1	166664028	166732069	68042	1	10	0	0.09290028	0	12	0	0.41314172	0
chr1	166732069	166967808	235740	7	11	0	0.14385241	0	12	0	0.30102999	0
chr1	166967808	167047588	79781	3	11	0	0.09334429	0	13	0	0.41271556	0
chr1	167047588	167047647	60	1	12	0	0.03209037	0	16	0	0.72109894	0
chr1	167047647	167101001	53355	2	11	0	0.0574087	0	14	0	0.5498098	0
chr1	167101001	167385239	284239	8	11	1	0.0331093	0.30102999	15	0	0.71538971	0
chr1	167385239	167385298	60	1	12	1	0.03209037	0.30102999	16	0	0.72109894	0
chr1	167385298	167461685	76388	2	11	1	0.01767679	0.30102999	16	0	0.91308053	0
chr1	167461685	167461744	60	1	12	1	0.03209037	0.30102999	16	0	0.72109894	0
chr1	167461744	167562534	100791	2	11	1	0.01767679	0.30102999	16	0	0.91308053	0
chr1	167562534	167562593	60	1	11	1	0.00141024	0.30102999	19	0	1.7506523	0
chr1	167562593	167614818	52226	1	11	1	0.00373523	0.30102999	18	0	1.42397267	0
chr1	167614818	167614877	60	1	13	1	0.01518174	0.30102999	18	0	0.9449689	0
chr1	167614877	167759326	144450	4	12	1	0.01667721	0.30102999	17	0	0.92532268	0
chr1	167759326	167759385	60	1	13	1	0.03037338	0.30102999	17	0	0.73110763	0
chr1	167759385	167792234	32850	1	12	1	0.01667721	0.30102999	17	0	0.92532268	0
chr1	167792234	167825627	33394	1	12	1	0.03209037	0.30102999	16	0	0.72109894	0
chr1	167825627	167870982	45356	3	13	1	0.05490675	0.30102999	16	0	0.55623409	0
chr1	167870982	167901229	30248	1	12	1	0.0565833	0.30102999	15	0	0.55190077	0
chr1	167901229	167921120	19892	2	12	1	0.03209037	0.30102999	16	0	0.72109894	0
chr1	167921120	167957816	36697	1	11	1	0.01767679	0.30102999	16	0	0.91308053	0
chr1	167957816	168020100	62285	2	10	1	0.01817691	0.30102999	15	0	0.90719478	0
chr1	168020100	168020159	60	1	11	1	0.0331093	0.30102999	15	0	0.71538971	0
chr1	168020159	168068778	48620	2	10	1	0.03344703	0.30102999	14	0	0.71353304	0
chr1	168068778	168094753	25976	2	10	1	0.00909834	0.30102999	16	0	1.13428373	0
chr1	168094753	168153189	58437	2	11	1	0.00373523	0.30102999	18	0	1.42397267	0
chr1	168153189	168171284	18096	1	10	1	0.00413023	0.30102999	17	0	1.39918918	0
chr1	168171284	168240910	69627	2	10	1	0.00909834	0.30102999	16	0	1.13428373	0
chr1	168240910	168334254	93345	2	10	1	0.01817691	0.30102999	15	0	0.90719478	0
chr1	168334254	168334313	60	1	12	1	0.01667721	0.30102999	17	0	0.92532268	0
chr1	168334313	168368728	34416	1	11	1	0.00859896	0.30102999	17	0	1.14735594	0
chr1	168368728	168368787	60	1	12	1	0.00778066	0.30102999	18	0	1.17038931	0
chr1	168368787	168439535	70749	1	11	1	0.00373523	0.30102999	18	0	1.42397267	0
chr1	168439535	168439594	60	1	12	1	0.00778066	0.30102999	18	0	1.17038931	0
chr1	168439594	168505498	65905	1	11	1	0.00373523	0.30102999	18	0	1.42397267	0
chr1	168505498	168547630	42133	1	11	1	0.01767679	0.30102999	16	0	0.91308053	0
chr1	168547630	168547689	60	1	12	1	0.03209037	0.30102999	16	0	0.72109894	0
chr1	168547689	168642532	94844	1	11	1	0.01767679	0.30102999	16	0	0.91308053	0
chr1	168642532	168642591	60	1	11	1	0.00859896	0.30102999	17	0	1.14735594	0
chr1	168642591	168665785	23195	1	11	1	0.01767679	0.30102999	16	0	0.91308053	0
chr1	168665785	168697417	31633	2	11	1	0.00859896	0.30102999	17	0	1.14735594	0
chr1	168697417	168888764	191348	4	12	1	0.01667721	0.30102999	17	0	0.92532268	0
chr1	168888764	168888823	60	1	13	1	0.03037338	0.30102999	17	0	0.73110763	0
chr1	168888823	168952588	63766	1	12	1	0.03209037	0.30102999	16	0	0.72109894	0
chr1	168952588	169046928	94341	2	13	1	0.03037338	0.30102999	17	0	0.73110763	0
chr1	169046928	169134292	87365	2	12	1	0.01667721	0.30102999	17	0	0.92532268	0
chr1	169134292	169256516	122225	2	11	1	0.00859896	0.30102999	17	0	1.14735594	0
chr1	169256516	169256575	60	1	12	1	0.01667721	0.30102999	17	0	0.92532268	0
chr1	169256575	169333135	76561	2	11	1	0.00859896	0.30102999	17	0	1.14735594	0
chr1	169333135	169333194	60	1	12	1	0.01667721	0.30102999	17	0	0.92532268	0
chr1	169333194	169483612	150419	7	11	1	0.00859896	0.30102999	17	0	1.14735594	0
chr1	169483612	169541537	57926	2	11	1	0.01767679	0.30102999	16	0	0.91308053	0
chr1	169541537	170071816	530280	19	12	1	0.01667721	0.30102999	17	0	0.92532268	0
chr1	170071816	170151383	79568	3	12	1	0.00778066	0.30102999	18	0	1.17038931	0
chr1	170151383	170208606	57224	1	12	1	0.01667721	0.30102999	17	0	0.92532268	0
chr1	170208606	170624938	416333	6	11	1	0.00859896	0.30102999	17	0	1.14735594	0
chr1	170624938	170917318	292381	6	11	1	0.00373523	0.30102999	18	0	1.42397267	0
chr1	170917318	170917377	60	1	12	1	0.00778066	0.30102999	18	0	1.17038931	0
chr1	170917377	171009175	91799	2	11	1	0.00373523	0.30102999	18	0	1.42397267	0
chr1	171009175	171235919	226745	6	10	1	0.00166733	0.30102999	18	0	1.70763027	0
chr1	171235919	171354454	118536	6	10	1	5.83E-04	0.30102999	19	0	2.06733314	0
chr1	171354454	171459452	104999	2	10	1	0.00166733	0.30102999	18	0	1.70763027	0
chr1	171459452	171459511	60	1	10	1	5.83E-04	0.30102999	19	0	2.06733314	0
chr1	171459511	171481310	21800	1	9	1	2.22E-04	0.30102999	19	0	2.41659593	0
chr1	171481310	171519306	37997	1	9	1	6.86E-04	0.30102999	18	0	2.02387299	0
chr1	171519306	171604596	85291	2	9	1	0.00183109	0.30102999	17	0	1.68336308	0
chr1	171604596	171622017	17422	2	9	1	2.22E-04	0.30102999	19	0	2.41659593	0

chr1	171622017	171754834	132818	2	9	1	0.00433294	0.30102999	16	0	1.3873209	0
chr1	171754834	171758230	3397	2	9	1	0.00183109	0.30102999	17	0	1.68336308	0
chr1	171758230	171922237	164008	3	9	1	0.00433294	0.30102999	16	0	1.3873209	0
chr1	171922237	171961411	39175	2	9	1	0.00183109	0.30102999	17	0	1.68336308	0
chr1	171961411	172011180	49770	1	9	1	0.00433294	0.30102999	16	0	1.3873209	0
chr1	172011180	172076969	65790	1	9	1	0.00926608	0.30102999	15	0	1.13004284	0
chr1	172076969	172096461	19493	2	9	1	0.00433294	0.30102999	16	0	1.3873209	0
chr1	172096461	172130663	34203	2	9	1	0.00183109	0.30102999	17	0	1.68336308	0
chr1	172130663	172412433	281771	7	9	1	0.00433294	0.30102999	16	0	1.3873209	0
chr1	172412433	172501817	89385	2	9	1	0.01817691	0.30102999	14	0	0.90719478	0
chr1	172501817	172501871	55	1	9	1	0.00433294	0.30102999	16	0	1.3873209	0
chr1	172501871	172526843	24973	1	9	1	0.00926608	0.30102999	15	0	1.13004284	0
chr1	172526843	172579985	53143	2	8	1	0.00433294	0.30102999	15	0	1.3873209	0
chr1	172579985	172634529	54545	2	8	1	0.00909834	0.30102999	14	0	1.13428373	0
chr1	172634529	172728910	94382	4	8	1	0.00433294	0.30102999	15	0	1.3873209	0
chr1	172728910	172887687	158778	3	8	2	0.00188722	0.61140001	16	0	1.67551277	0
chr1	172887687	173013018	125332	2	8	2	0.00433294	0.61140001	15	0	1.3873209	0
chr1	173013018	173154984	141967	4	8	2	0.00188722	0.61140001	16	0	1.67551277	0
chr1	173154984	173195574	40591	3	8	2	0.00433294	0.61140001	15	0	1.3873209	0
chr1	173195574	173272080	76507	2	8	2	0.00188722	0.61140001	16	0	1.67551277	0
chr1	173272080	173331115	59036	1	8	2	0.00433294	0.61140001	15	0	1.3873209	0
chr1	173331115	173452803	121689	2	8	2	0.00909834	0.61140001	14	0	1.13428373	0
chr1	173452803	173490449	37647	2	8	2	0.00433294	0.61140001	15	0	1.3873209	0
chr1	173490449	173490508	60	1	8	2	0.00188722	0.61140001	16	0	1.67551277	0
chr1	173490508	173545877	55370	2	8	2	0.00433294	0.61140001	15	0	1.3873209	0
chr1	173545877	173884316	338440	12	8	2	0.00909834	0.61140001	14	0	1.13428373	0
chr1	173884316	174002163	117848	2	8	2	0.01767679	0.61140001	13	0	0.91308053	0
chr1	174002163	174124974	122812	3	8	3	0.01767679	0.93173516	13	0	0.91308053	0
chr1	174124974	174265632	140659	2	6	3	0.00778066	0.93173516	12	0	1.17038931	0
chr1	174265632	174265691	60	1	7	3	0.01667721	0.93173516	12	0	0.92532268	0
chr1	174265691	174340225	74535	1	6	3	0.00778066	0.93173516	12	0	1.17038931	0
chr1	174340225	174417647	77423	1	6	3	0.01518174	0.93173516	11	0	0.9449689	0
chr1	174417647	174468168	50522	2	6	3	0.02793176	0.93173516	10	0	0.74627054	0
chr1	174468168	174507537	39370	2	6	3	0.01518174	0.93173516	11	0	0.9449689	0
chr1	174507537	174507596	60	1	7	3	0.03037338	0.93173516	11	0	0.73110763	0
chr1	174507596	174553313	45718	1	6	3	0.01518174	0.93173516	11	0	0.9449689	0
chr1	174553313	174606489	53177	1	6	2	0.01518174	0.61140001	11	0	0.9449689	0
chr1	174606489	174709489	103001	3	7	2	0.03037338	0.61140001	11	0	0.73110763	0
chr1	174709489	174709546	58	1	7	2	0.01667721	0.61140001	12	0	0.92532268	0
chr1	174709546	174862913	153368	4	6	2	0.01518174	0.61140001	11	0	0.9449689	0
chr1	174862913	174926542	63630	3	6	2	0.00778066	0.61140001	12	0	1.17038931	0
chr1	174926542	174961849	35308	1	6	2	0.01518174	0.61140001	11	0	0.9449689	0
chr1	174961849	174987608	25760	3	7	2	0.03037338	0.61140001	11	0	0.73110763	0
chr1	174987608	175134107	146500	3	6	2	0.01518174	0.61140001	11	0	0.9449689	0
chr1	175134107	175176432	42326	3	6	2	0.00778066	0.61140001	12	0	1.17038931	0
chr1	175176432	175310373	133942	2	6	2	0.01518174	0.61140001	11	0	0.9449689	0
chr1	175310373	175332874	22502	2	6	2	0.00778066	0.61140001	12	0	1.17038931	0
chr1	175332874	175497460	164587	3	6	2	0.01518174	0.61140001	11	0	0.9449689	0
chr1	175497460	175535442	37983	2	6	2	0.02793176	0.61140001	10	0	0.74627054	0
chr1	175535442	175581791	46350	2	6	2	0.01518174	0.61140001	11	0	0.9449689	0
chr1	175581791	175958529	376739	7	7	2	0.03037338	0.61140001	11	0	0.73110763	0
chr1	175958529	176178810	220282	6	6	2	0.01518174	0.61140001	11	0	0.9449689	0
chr1	176178810	176178869	60	1	6	2	0.00778066	0.61140001	12	0	1.17038931	0
chr1	176178869	176432507	253639	4	6	2	0.01518174	0.61140001	11	0	0.9449689	0
chr1	176432507	176630878	198372	5	6	2	0.00778066	0.61140001	12	0	1.17038931	0
chr1	176630878	176716297	85420	2	6	2	0.01518174	0.61140001	11	0	0.9449689	0
chr1	176716297	176811726	95430	2	6	2	0.02793176	0.61140001	10	0	0.74627054	0
chr1	176811726	176926623	114898	2	5	2	0.01320236	0.61140001	10	0	0.97390707	0
chr1	176926623	177057284	130662	4	6	2	0.02793176	0.61140001	10	0	0.74627054	0
chr1	177057284	177456183	398900	9	6	2	0.00778066	0.61140001	12	0	1.17038931	0
chr1	177456183	177456242	60	1	6	2	0.00166733	0.61140001	14	0	1.70763027	0
chr1	177456242	177800358	344117	2	6	2	0.00778066	0.61140001	12	0	1.17038931	0
chr1	177800358	177800417	60	1	7	2	0.00859896	0.61140001	13	0	1.14735594	0
chr1	177800417	177898011	97595	1	6	2	0.02793176	0.61140001	10	0	0.74627054	0
chr1	177898011	177974860	76850	5	7	2	0.05232577	0.61140001	10	0	0.56314362	0
chr1	177974860	177974919	60	1	8	2	0.05490675	0.61140001	11	0	0.55623409	0
chr1	177974919	178065328	90410	2	7	1	0.05232577	0.30102999	10	0	0.56314362	0
chr1	178065328	178065387	60	1	8	2	0.05490675	0.61140001	11	0	0.55623409	0
chr1	178065387	178196998	131612	2	7	2	0.03037338	0.61140001	11	0	0.73110763	0
chr1	178196998	178248438	51441	1	7	2	0.08584816	0.61140001	9	0	0.42015402	0
chr1	178248438	178315977	67540	2	8	2	0.13872638	0.61140001	9	0	0.30102999	0
chr1	178315977	178443113	127137	2	7	2	0.08584816	0.61140001	9	0	0.42015402	0
chr1	178443113	178490969	47857	2	7	2	0.13499366	0.61140001	8	0	0.30102999	0
chr1	178490969	178517686	26718	2	7	2	0.08584816	0.61140001	9	0	0.42015402	0
chr1	178517686	178520811	3126	2	8	2	0.13872638	0.61140001	9	0	0.30102999	0
chr1	178520811	178671697	150887	1	8	1	0.20764654	0.30102999	8	0	0.20764654	0
chr1	178671697	178708604	36908	2	9	1	0.09154957	0.30102999	11	0	0.41444892	0
chr1	178708604	178777213	68610	2	9	2	0.09154957	0.61140001	11	0	0.41444892	0
chr1	178777213	178777272	60	1	9	2	0.0565833	0.61140001	12	0	0.55190077	0
chr1	178777272	178855220	77949	4	9	2	0.09154957	0.61140001	11	0	0.41444892	0
chr1	178855220	178855279	60	1	10	2	0.14303407	0.61140001	11	0	0.30102999	0
chr1	178855279	178885805	30527	1	9	2	0.09154957	0.61140001	11	0	0.41444892	0
chr1	178885805	179063269	177465	4	8	2	0.08923391	0.61140001	10	0	0.4167287	0
chr1	179063269	179111998	48730	2	8	2	0.13872638	0.61140001	9	0	0.30102999	0
chr1	179111998	179185186	73189	4	8	2	0.08923391	0.61140001	10	0	0.4167287	0
chr1	179185186	179256760	71575	2	8	2	0.13872638	0.61140001	9	0	0.30102999	0
chr1	179256760	179256819	60	1	8	2	0.05490675	0.61140001	11	0	0.55623409	0
chr1	179256819	179337729	80911	2	8	2	0.08923391	0.61140001	10	0	0.4167287	0
chr1	179337729	179452353	114625	5	8	2	0.05490675	0.61140001	11	0	0.55623409	0
chr1	179452353	179523750	71398	3	8	2	0.08923391	0.61140001	10	0	0.4167287	0
chr1	179523750	179562879	39130	3	9	2	0.09154957	0.61140001	11	0	0.41444892	0
chr1	179562879	179864197	301319	10	9	1	0.09154957	0.30102999	11	0	0.41444892	0
chr1	179864197	179955613	91417	2	9	0	0.09154957	0	11	0	0.41444892	0
chr1	179955613	180010831	55219	1	8	0	0.05490675	0	11	0	0.55623409	0
chr1	180010831	180091262	80432	2	8	0	0.08923391	0	10	0	0.4167287	0
chr1	180091262	180142148	50887	2	8	0	0.05490675	0	11	0	0.55623409	0
chr1	180142148	180244610	102463	3	9	0	0.09154957	0	11	0	0.41444892	0
chr1	180244610	180399392	154783	3	7	0	0.03037338	0	11	0	0.73110763	0
chr1	180399392	180521573	122182	2	6	0	0.01518174	0	11	0	0.9449689	0
chr1	180521573	180521632	60	1	7	0	0.03037338	0	11	0	0.73110763	0
chr1	180521632	180598712	77081	1	6	0	0.01518174	0	11	0	0.9449689	0
chr1	180598712	180598771	60	1	7	0	0.03037338	0	11	0	0.73110763	0
chr1	180598771	180640058	41288	1	6	0	0.01518174	0	11	0	0.9449689	0
chr1	180640058	180640117	60	1	7	0	0.03037338	0	11	0	0.73110763	0
chr1	180640117	180702559	62443	1	6	0	0.02793176	0	10	0	0.74627054	0
chr1	180702559	180756865	54307	1	5	0	0.01320236	0	10	0	0.97390707	0
chr1	180756865	180756924	60	1	6	0	0.02793176	0	10	0	0.74627054	0
chr1	180756924	180794025	37102	1	5	0	0.02473314	0	9	0	0.76806864	0
chr1	180794025	180794084	60	1	5	0	0.01320236	0	10	0	0.97390707	0
chr1	180794084	180889688	95605	2	5	0	0.02473314	0	9	0	0.76806864	0
chr1	180889688	180889747	60	1	5	0	0.00666883	0	11	0	1.20557689	0
chr1	180889747	181069851	180105	7	5	0	0.01320236	0	10	0	0.97390707	0
chr1	181069851	181069910	60	1	7	0	0.05232577	0	10	0	0.56314362	0
chr1	181069910	181213027	143118	1	6	0	0.04875589	0	9	0	0.5732208	0
chr1	181213027	181269653	56627	1	4	0	0.01077081	0	9	0	1.01542894	0
chr1	181269653	181400912	131260	2	5	0	0.01320236	0	10	0	0.97390707	0
chr1	181400912	181400971	60	1	6	0	0.02793176	0	10	0	0.74627054	0
chr1	181400971	181473972	73002	2	6	0	0.04875589	0	9	0	0.5732208	0
chr1	181473972	181548312	74341	2	6	0	0.02793176	0	10	0	0.74627054	0
chr1	181548312	181684509	136198	3	8	0	0.05490675	0	11	0	0.55623409	0
chr1	181684509	181746968	62460	3	8	0	0.13872638	0	9	0	0.30102999	0
chr1	181746968	181774393	27426	1	7	0	0.13499366	0	8	0	0.30102999	0
chr1	181774393	182067459	293067	2	6	0	0.08122616	0	8	0	0.4250187	0
chr1	182067459	182355914	288456	5	9	0	0.09154957	0	11	0	0.41444892	0

chr1	182355914	182393883	37970	4	9	0	0.0565833	0	12	0	0.55190077	0
chr1	182393883	182429176	35294	2	9	0	0.0331093	0	13	0	0.71538971	0
chr1	182429176	182555461	126286	6	9	0	0.09154957	0	11	0	0.41444892	0
chr1	182555461	182664454	108994	4	9	0	0.0565833	0	12	0	0.55190077	0
chr1	182664454	182664513	60	1	9	0	0.0331093	0	13	0	0.71538971	0
chr1	182664513	182782726	118214	2	9	0	0.09154957	0	11	0	0.41444892	0
chr1	182782726	182811709	28984	2	9	0	0.0331093	0	13	0	0.71538971	0
chr1	182811709	182888856	77148	2	9	0	0.0565833	0	12	0	0.55190077	0
chr1	182888856	182888915	60	1	9	0	0.0331093	0	13	0	0.71538971	0
chr1	182888915	182920485	31571	1	9	0	0.0565833	0	12	0	0.55190077	0
chr1	182920485	182920544	60	1	9	0	0.0331093	0	13	0	0.71538971	0
chr1	182920544	182988513	67970	2	9	0	0.0565833	0	12	0	0.55190077	0
chr1	182988513	183044082	55570	2	8	0	0.03209037	0	12	0	0.72109894	0
chr1	183044082	183213332	169251	6	8	0	0.01767679	0	13	0	0.91308053	0
chr1	183213332	183299871	86540	2	8	0	0.03209037	0	12	0	0.72109894	0
chr1	183299871	183485066	185196	6	8	0	0.01767679	0	13	0	0.91308053	0
chr1	183485066	183525229	40164	3	8	0	0.00909834	0	14	0	1.13428373	0
chr1	183525229	183623881	98653	2	8	0	0.01767679	0	13	0	0.91308053	0
chr1	183623881	183680966	57086	2	9	0	0.0331093	0	13	0	0.71538971	0
chr1	183680966	183681025	60	1	9	0	0.01817691	0	14	0	0.90719478	0
chr1	183681025	183775552	94528	2	9	0	0.0331093	0	13	0	0.71538971	0
chr1	183775552	183775602	51	1	9	0	0.00926608	0	15	0	1.13004284	0
chr1	183775602	183896269	120668	3	9	0	0.01817691	0	14	0	0.90719478	0
chr1	183896269	183923887	27619	1	9	0	0.0331093	0	13	0	0.71538971	0
chr1	183923887	183947592	23706	2	9	0	0.01817691	0	14	0	0.90719478	0
chr1	183947592	183947651	60	1	9	0	0.00926608	0	15	0	1.13004284	0
chr1	183947651	184021511	73861	2	9	0	0.01817691	0	14	0	0.90719478	0
chr1	184021511	184091213	69703	2	8	0	0.03209037	0	12	0	0.72109894	0
chr1	184091213	184208950	117738	2	8	0	0.00909834	0	14	0	1.13428373	0
chr1	184208950	184319011	110062	2	8	0	0.00433294	0	15	0	1.3873209	0
chr1	184319011	184319070	60	1	8	1	0.00188722	0.30102999	16	0	1.67551277	0
chr1	184319070	184431762	112693	2	8	1	0.00433294	0.30102999	15	0	1.3873209	0
chr1	184431762	184476696	44935	2	9	1	0.00926608	0.30102999	15	0	1.13004284	0
chr1	184476696	184476752	57	1	9	1	0.00433294	0.30102999	16	0	1.3873209	0
chr1	184476752	184512910	36159	1	9	1	0.00926608	0.30102999	15	0	1.13004284	0
chr1	184512910	184662134	149225	4	8	1	0.00433294	0.30102999	15	0	1.3873209	0
chr1	184662134	184723111	60978	2	8	1	0.01767679	0.30102999	13	0	0.91308053	0
chr1	184723111	184832585	109475	4	8	1	0.00909834	0.30102999	14	0	1.13428373	0
chr1	184832585	184919870	87286	2	8	1	0.01767679	0.30102999	13	0	0.91308053	0
chr1	184919870	184983650	63781	3	8	1	0.00909834	0.30102999	14	0	1.13428373	0
chr1	184983650	185029069	45420	1	8	1	0.01767679	0.30102999	13	0	0.91308053	0
chr1	185029069	185106572	77504	2	8	0	0.01767679	0	13	0	0.91308053	0
chr1	185106572	185125974	19403	2	8	1	0.01767679	0.30102999	13	0	0.91308053	0
chr1	185125974	185149558	23585	2	8	1	0.00909834	0.30102999	14	0	1.13428373	0
chr1	185149558	185240518	90961	3	8	0	0.01767679	0	13	0	0.91308053	0
chr1	185240518	185267168	26651	2	7	0	0.03037338	0	11	0	0.73110763	0
chr1	185267168	185276218	9051	2	8	0	0.05490675	0	11	0	0.55623409	0
chr1	185276218	185278266	2049	2	8	0	0.01767679	0	13	0	0.91308053	0
chr1	185278266	185453551	175286	3	8	0	0.00909834	0	14	0	1.13428373	0
chr1	185453551	185576474	122924	2	8	1	0.00909834	0.30102999	14	0	1.13428373	0
chr1	185576474	185926188	349715	6	8	1	0.01767679	0.30102999	13	0	0.91308053	0
chr1	185926188	185926247	60	1	8	1	0.00909834	0.30102999	14	0	1.13428373	0
chr1	185926247	185962462	36216	2	8	0	0.00909834	0	14	0	1.13428373	0
chr1	185962462	186060881	98420	3	8	1	0.00909834	0.30102999	14	0	1.13428373	0
chr1	186060881	186113517	52637	1	8	1	0.01767679	0.30102999	13	0	0.91308053	0
chr1	186113517	186123104	9588	2	8	1	0.00909834	0.30102999	14	0	1.13428373	0
chr1	186123104	186173009	49906	1	7	1	0.00859896	0.30102999	13	0	1.14735594	0
chr1	186173009	186257228	84220	1	6	1	0.00373523	0.30102999	13	0	1.42397267	0
chr1	186257228	186257287	60	1	6	2	0.00373523	0.61140001	13	0	1.42397267	0
chr1	186257287	186273308	16022	1	6	0	0.00373523	0	13	0	1.42397267	0
chr1	186273308	186278235	4928	3	7	0	0.00859896	0	13	0	1.14735594	0
chr1	186278235	186278294	60	1	7	1	0.00859896	0.30102999	13	0	1.14735594	0
chr1	186278294	186296518	18225	1	6	0	0.00373523	0	13	0	1.42397267	0
chr1	186296518	186421943	125426	8	6	0	0.00166733	0	14	0	1.70763027	0
chr1	186421943	186549764	127822	1	6	0	0.00778066	0	12	0	1.17038931	0
chr1	186549764	186785078	235315	7	7	0	0.01667721	0	12	0	0.92532268	0
chr1	186785078	186785137	60	1	7	0	0.00859896	0	13	0	1.14735594	0
chr1	186785137	186863228	78092	2	7	0	0.01667721	0	12	0	0.92532268	0
chr1	186863228	186863287	60	1	7	0	0.00859896	0	13	0	1.14735594	0
chr1	186863287	186934611	71325	2	6	0	0.00778066	0	12	0	1.17038931	0
chr1	186934611	187074744	140134	2	6	0	0.01518174	0	11	0	0.9449689	0
chr1	187074744	187218418	143675	1	6	0	0.02793176	0	10	0	0.74627054	0
chr1	187218418	187218477	60	1	6	1	0.02793176	0.30102999	10	0	0.74627054	0
chr1	187218477	187290553	72077	1	6	0	0.02793176	0	10	0	0.74627054	0
chr1	187290553	187540051	249499	3	6	0	0.01518174	0	11	0	0.9449689	0
chr1	187540051	187615965	75915	1	3	0	0.00170589	0	10	0	1.61091002	0
chr1	187615965	187813722	197758	2	3	0	0.01598258	0	7	0	0.84395715	0
chr1	187813722	187956699	142978	1	3	0	0.03070643	0	6	0	0.63695542	0
chr1	187956699	188206008	249310	2	3	0	0.05670724	0	5	0	0.45545077	0
chr1	188206008	188391393	185386	1	3	0	0.10122019	0	4	0	0.30102999	0
chr1	188391393	188574502	183110	2	3	0	0.05670724	0	5	0	0.45545077	0
chr1	188574502	188659081	84580	1	1	0	0.02438896	0	3	0	0.51676182	0
chr1	188659081	188786712	127632	2	1	0	0.01091641	0	4	0	0.76005302	0
chr1	188786712	189101185	314474	2	1	0	0.1218695	0	1	0	0.1218695	0
chr1	189101185	189101244	60	1	1	0	0.01091641	0	4	0	0.76005302	0
chr1	189101244	189366715	265472	3	1	0	0.02438896	0	3	0	0.51676182	0
chr1	189366715	189366774	60	1	2	0	0.02162467	0	5	0	0.68214471	0
chr1	189366774	189574701	207928	1	2	0	0.0429175	0	4	0	0.47744371	0
chr1	189574701	189574760	60	1	3	0	0.10122019	0	4	0	0.30102999	0
chr1	189574760	189770699	195940	1	1	0	0.01091641	0	4	0	0.76005302	0
chr1	189770699	190005334	234636	3	1	0	0.00478973	0	5	0	1.02643191	0
chr1	190005334	190086193	80860	2	2	0	0.01053319	0	6	0	0.91219088	0
chr1	190086193	190218321	132129	2	1	0	0.00204627	0	6	0	1.31360226	0
chr1	190218321	190218380	60	1	5	0	0.07511598	0	7	0	0.43181735	0
chr1	190218380	190319553	101174	2	2	0	0.01053319	0	6	0	0.91219088	0
chr1	190319553	190394529	74977	1	1	0	0.00478973	0	5	0	1.02643191	0
chr1	190394529	190624423	229895	3	2	0	0.02162467	0	5	0	0.68214471	0
chr1	190624423	190705646	81224	2	3	0	0.0079614	0	8	0	1.07548421	0
chr1	190705646	191102159	396514	4	2	0	0.00221948	0	8	0	1.44210395	0
chr1	191102159	191102218	60	1	3	0	0.00378107	0	9	0	1.33111237	0
chr1	191102218	191164061	61844	1	3	0	0.01598258	0	7	0	0.84395715	0
chr1	191164061	191164120	60	1	4	0	0.03812622	0	7	0	0.60763643	0
chr1	191164120	191388560	224441	1	4	0	0.06713722	0	6	0	0.44141547	0
chr1	191388560	191388619	60	1	5	0	0.07511598	0	7	0	0.43181735	0
chr1	191388619	191568354	179736	1	4	0	0.03812622	0	7	0	0.60763643	0
chr1	191568354	191759606	191253	1	3	0	0.01598258	0	7	0	0.84395715	0
chr1	191759606	191759665	60	1	4	0	0.02074938	0	8	0	0.79906872	0
chr1	191759665	191888324	128660	1	3	0	0.0079614	0	8	0	1.07548421	0
chr1	191888324	192092423	204100	3	4	0	0.02074938	0	8	0	0.79906872	0
chr1	192092423	192092482	60	1	4	0	0.00108487	0	12	0	1.81706455	0
chr1	192092482	192150485	58004	1	3	0	2.93E-04	0	12	0	2.24559516	0
chr1	192150485	192227887	77403	3	3	0	1.10E-04	0	13	0	2.60294723	0
chr1	192227887	192422867	194981	4	4	0	1.70E-04	0	14	0	2.48919369	0
chr1	192422867	192422926	60	1	5	0	5.83E-04	0	14	0	2.06733314	0
chr1	192422926	192548394	125469	3	4	0	1.70E-04	0	14	0	2.48919369	0
chr1	192548394	192624083	75690	2	4	0	0.00108487	0	12	0	1.81706455	0
chr1	192624083	192697218	73136	2	4	0	4.45E-04	0	13	0	2.13824703	0
chr1	192697218	192780297	83080	1	4	0	0.00108487	0	12	0	1.81706455	0
chr1	192780297	192780356	60	1	4	0	4.45E-04	0	13	0	2.13824703	0
chr1	192780356	192944023	163668	1	4	0	0.00108487	0	12	0	1.81706455	0
chr1	192944023	192944082	60	1	4	0	1.70E-04	0	14	0	2.48919369	0
chr1	192944082	193091411	147330	7	4	0	4.45E-04	0	13	0	2.13824703	0
chr1	193091411	193119463	28053	1	4	0	0.00108487	0	12	0	1.81706455	0
chr1	193119463	193202105	82643	2	4	0	0.00530919	0	10	0	1.2568129	0

chr1	193202105	193277676	75572	3	4	0	4.45E-04	0	13	0	2.13824703	0
chr1	193277676	193489523	211848	1	2	0	0.00493743	0	7	0	1.16581773	0
chr1	193489523	193725938	236416	2	1	0	0.00478973	0	5	0	1.02643191	0
chr1	193725938	193725997	60	1	2	0	0.00221948	0	8	0	1.44210395	0
chr1	193725997	193878906	152910	1	2	0	0.01053319	0	6	0	0.91219088	0
chr1	193878906	194198125	319220	2	1	0	0.00478973	0	5	0	1.02643191	0
chr1	194198125	194583280	385156	3	0	0	0	0	2	0	0.61140001	0
chr1	194583280	195051375	468096	4	0	0	0	0	3	0	0.93173516	0
chr1	195051375	195223664	172290	1	0	0	0	0	2	0	0.61140001	0
chr1	195223664	195433626	209963	2	1	0	0.01091641	0	4	0	0.76005302	0
chr1	195433626	195433685	60	1	2	0	0.02162467	0	5	0	0.68214471	0
chr1	195433685	195687659	253975	1	1	0	0.00478973	0	5	0	1.02643191	0
chr1	195687659	195687718	60	1	1	0	0.00204627	0	6	0	1.31360226	0
chr1	195687718	195914114	226397	1	1	0	0.00478973	0	5	0	1.02643191	0
chr1	195914114	196102198	188085	2	1	0	0.00204627	0	6	0	1.31360226	0
chr1	196102198	196204178	101981	2	2	0	0.00493743	0	7	0	1.16581773	0
chr1	196204178	196244549	40372	2	2	0	0.00221948	0	8	0	1.44210395	0
chr1	196244549	196244608	60	1	3	0	0.00170589	0	10	0	1.61091002	0
chr1	196244608	196341182	96575	2	2	0	3.90E-04	0	10	0	2.0620585	0
chr1	196341182	196341241	60	1	3	1	0.00170589	0.30102999	10	0	1.61091002	0
chr1	196341241	196419359	78119	1	3	0	0.00170589	0	10	0	1.61091002	0
chr1	196419359	196573625	154267	3	2	0	9.54E-04	0	9	0	1.74076927	0
chr1	196573625	196573684	60	1	4	1	0.00247414	0.30102999	11	0	1.52371709	0
chr1	196573684	196657666	83983	2	4	1	0.01077081	0.30102999	9	0	1.01542894	0
chr1	196657666	196711090	53425	3	5	1	0.01320236	0.30102999	10	0	0.97390707	0
chr1	196711090	196780309	69220	2	5	1	0.00666883	0.30102999	11	0	1.20557689	0
chr1	196780309	196883398	103090	3	5	1	0.00141024	0.30102999	13	0	1.7506523	0
chr1	196883398	197111575	228178	9	5	1	0.00666883	0.30102999	11	0	1.20557689	0
chr1	197111575	197111634	60	1	5	1	0.00317045	0.30102999	12	0	1.46403142	0
chr1	197111634	197237527	125894	4	5	0	0.00317045	0	12	0	1.46403142	0
chr1	197237527	197272011	34485	2	5	0	5.83E-04	0	14	0	2.06733314	0
chr1	197272011	197313461	41451	2	5	0	2.22E-04	0	15	0	2.41659593	0
chr1	197313461	197356976	43516	1	5	0	5.83E-04	0	14	0	2.06733314	0
chr1	197356976	197403924	46949	1	5	0	0.00141024	0	13	0	1.7506523	0
chr1	197403924	197498692	94769	2	4	0	4.45E-04	0	13	0	2.13824703	0
chr1	197498692	197546906	48215	3	5	1	2.41E-05	0.30102999	17	0	3.22720381	0
chr1	197546906	197641307	94402	2	5	1	5.83E-04	0.30102999	14	0	2.06733314	0
chr1	197641307	197686321	45015	1	5	1	0.00141024	0.30102999	13	0	1.7506523	0
chr1	197686321	197686380	60	1	5	1	5.83E-04	0.30102999	14	0	2.06733314	0
chr1	197686380	197743792	57413	1	5	0	5.83E-04	0	14	0	2.06733314	0
chr1	197743792	197745923	2132	2	5	1	5.83E-04	0.30102999	14	0	2.06733314	0
chr1	197745923	197886549	140627	1	5	0	5.83E-04	0	14	0	2.06733314	0
chr1	197886549	197959837	73289	3	5	0	2.41E-05	0	17	0	3.22720381	0
chr1	197959837	197959896	60	1	5	0	6.64E-06	0	18	0	3.69875624	0
chr1	197959896	198172608	212713	2	4	0	0.00247414	0	11	0	1.52371709	0
chr1	198172608	198222269	49662	3	4	0	0.00108487	0	12	0	1.81706455	0
chr1	198222269	198319505	97237	2	4	0	0.00247414	0	11	0	1.52371709	0
chr1	198319505	198365433	45929	2	6	0	0.00778066	0	12	0	1.17038931	0
chr1	198365433	198465127	99695	2	5	0	0.00666883	0	11	0	1.20557689	0
chr1	198465127	198498814	33688	3	6	0	0.00778066	0	12	0	1.17038931	0
chr1	198498814	198577644	78831	1	5	0	0.00666883	0	11	0	1.20557689	0
chr1	198577644	198652996	75353	4	5	0	0.00317045	0	12	0	1.46403142	0
chr1	198652996	198653055	60	1	5	0	0.00141024	0	13	0	1.7506523	0
chr1	198653055	198756025	102971	3	4	0	0.00247414	0	11	0	1.52371709	0
chr1	198756025	198838996	82972	3	4	0	0.00108487	0	12	0	1.81706455	0
chr1	198838996	199118773	279778	3	4	0	0.00247414	0	11	0	1.52371709	0
chr1	199118773	199229665	110893	1	4	0	0.00530919	0	10	0	1.2568129	0
chr1	199229665	199369446	139782	2	4	0	0.01077081	0	9	0	1.01542894	0
chr1	199369446	199369505	60	1	4	0	0.00247414	0	11	0	1.52371709	0
chr1	199369505	199615787	246283	2	4	0	0.00530919	0	10	0	1.2568129	0
chr1	199615787	199779407	163621	2	4	0	0.00108487	0	12	0	1.81706455	0
chr1	199779407	199779466	60	1	4	0	4.45E-04	0	13	0	2.13824703	0
chr1	199779466	199868196	88731	1	4	0	0.00108487	0	12	0	1.81706455	0
chr1	199868196	200098404	230209	5	4	0	4.45E-04	0	13	0	2.13824703	0
chr1	200098404	200209628	111225	5	5	0	0.00141024	0	13	0	1.7506523	0
chr1	200209628	200209687	60	1	7	0	0.00859896	0	13	0	1.14735594	0
chr1	200209687	200321425	111739	2	5	0	0.00141024	0	13	0	1.7506523	0
chr1	200321425	200635455	314031	8	7	0	0.00859896	0	13	0	1.14735594	0
chr1	200635455	200728080	92626	2	7	0	0.00413023	0	14	0	1.39918918	0
chr1	200728080	200940617	212538	7	7	0	0.00183109	0	15	0	1.68336308	0
chr1	200940617	201251334	310718	10	8	0	0.00433294	0	15	0	1.3873209	0
chr1	201251334	201366803	115470	3	7	0	0.00183109	0	15	0	1.68336308	0
chr1	201366803	201613737	246935	8	8	0	0.00188722	0	16	0	1.67551277	0
chr1	201613737	201613796	60	1	8	0	7.41E-04	0	17	0	2.00316618	0
chr1	201613796	201680121	66326	2	8	0	0.00188722	0	16	0	1.67551277	0
chr1	201680121	201907113	226993	9	9	0	0.00433294	0	16	0	1.3873209	0
chr1	201907113	202017037	109925	2	7	0	7.41E-04	0	16	0	2.00316618	0
chr1	202017037	202017096	60	1	8	0	0.00188722	0	16	0	1.67551277	0
chr1	202017096	202216046	198951	7	7	0	7.41E-04	0	16	0	2.00316618	0
chr1	202216046	202275394	59349	2	7	0	2.70E-04	0	17	0	2.36290411	0
chr1	202275394	202334231	58838	2	7	0	7.41E-04	0	16	0	2.00316618	0
chr1	202334231	202403843	69613	1	7	0	0.00413023	0	14	0	1.39918918	0
chr1	202403843	202554802	150960	2	6	0	0.00166733	0	14	0	1.70763027	0
chr1	202554802	202598287	43486	2	7	0	0.00183109	0	15	0	1.68336308	0
chr1	202598287	202642917	44631	2	6	0	0.00166733	0	14	0	1.70763027	0
chr1	202642917	202675951	33035	2	7	0	0.00183109	0	15	0	1.68336308	0
chr1	202675951	202698829	22879	2	6	0	0.00166733	0	14	0	1.70763027	0
chr1	202698829	202735711	36883	1	6	0	0.00373523	0	13	0	1.42397267	0
chr1	202735711	202735767	57	1	7	0	0.00413023	0	14	0	1.39918918	0
chr1	202735767	202821177	85411	2	6	0	0.00373523	0	13	0	1.42397267	0
chr1	202821177	202821236	60	1	7	0	0.00413023	0	14	0	1.39918918	0
chr1	202821236	202878048	56813	4	6	0	0.00166733	0	14	0	1.70763027	0
chr1	202878048	203120599	242552	8	7	0	0.00413023	0	14	0	1.39918918	0
chr1	203120599	203120658	60	1	8	0	0.00909834	0	14	0	1.13428373	0
chr1	203120658	203190295	69638	4	7	0	0.01667721	0	12	0	0.92532268	0
chr1	203190295	203391054	200760	8	8	0	0.03209037	0	12	0	0.72109894	0
chr1	203391054	203539647	148594	2	8	0	0.05490675	0	11	0	0.55623409	0
chr1	203539647	203650551	110905	4	8	0	0.00909834	0	14	0	1.13428373	0
chr1	203650551	203734574	84024	5	8	0	0.01767679	0	13	0	0.91308053	0
chr1	203734574	203820364	85791	4	7	0	0.01667721	0	12	0	0.92532268	0
chr1	203820364	203820423	60	1	8	0	0.01767679	0	13	0	0.91308053	0
chr1	203820423	204081031	260609	7	7	0	0.01667721	0	12	0	0.92532268	0
chr1	204081031	204224751	143721	9	6	0	0.00778066	0	12	0	1.17038931	0
chr1	204224751	204330100	105350	3	6	0	0.00373523	0	13	0	1.42397267	0
chr1	204330100	204375288	45189	2	6	1	0.00373523	0.30102999	13	0	1.42397267	0
chr1	204375288	204425914	50627	2	5	1	0.00317045	0.30102999	12	0	1.46403142	0
chr1	204425914	204425973	60	1	6	1	0.00373523	0.30102999	13	0	1.42397267	0
chr1	204425973	204507394	81422	2	5	1	0.00317045	0.30102999	12	0	1.46403142	0
chr1	204507394	204535418	28025	2	6	1	0.00778066	0.30102999	12	0	1.17038931	0
chr1	204535418	204578875	43458	1	6	0	0.00778066	0	12	0	1.17038931	0
chr1	204578875	204597892	19018	2	7	0	0.01667721	0	12	0	0.92532268	0
chr1	204597892	204808306	210415	4	7	0	0.00859896	0	13	0	1.14735594	0
chr1	204808306	204972361	164056	3	7	0	0.01667721	0	12	0	0.92532268	0
chr1	204972361	204972420	60	1	7	0	0.00859896	0	13	0	1.14735594	0
chr1	204972420	205159212	186793	4	7	0	0.01667721	0	12	0	0.92532268	0
chr1	205159212	205159271	60	1	7	0	0.00859896	0	13	0	1.14735594	0
chr1	205159271	205313910	154640	4	7	0	0.03037338	0	11	0	0.73110763	0
chr1	205313910	205391783	77874	2	6	0	0.01518174	0	11	0	0.9449689	0
chr1	205391783	205391842	60	1	7	0	0.03037338	0	11	0	0.73110763	0
chr1	205391842	205422001	30160	1	6	0	0.01518174	0	11	0	0.9449689	0
chr1	205422001	205692921	270921	10	6	0	0.02793176	0	10	0	0.74627054	0
chr1	205692921	205715494	22574	2	8	0	0.08923391	0	10	0	0.4167287	0
chr1	205715494	205739190	23697	1	7	0	0.05232577	0	10	0	0.56314362	0
chr1	205739190	205902280	163091	5	5	0	0.01320236	0	10	0	0.97390707	0

chr1	205902280	205914384	12105	2	6	0	0.02793176	0	10	0	0.74627054	0
chr1	205914384	206243724	329341	1	5	0	0.02473314	0	9	0	0.76806864	0
chr1	206243724	206305147	61424	2	6	0	0.04875589	0	9	0	0.5732208	0
chr1	206305147	206305206	60	1	6	0	0.01518174	0	11	0	0.9449689	0
chr1	206305206	206593620	288415	1	4	0	0.01077081	0	9	0	1.01542894	0
chr1	206593620	206822426	228807	6	5	0	0.02473314	0	9	0	0.76806864	0
chr1	206822426	207107507	285082	13	5	0	0.04407651	0	8	0	0.58747015	0
chr1	207107507	207893459	785953	23	5	0	0.02473314	0	9	0	0.76806864	0
chr1	207893459	207983222	89764	2	5	0	0.04407651	0	8	0	0.58747015	0
chr1	207983222	207983281	60	1	5	0	0.02473314	0	9	0	0.76806864	0
chr1	207983281	208196373	213093	4	5	0	0.04407651	0	8	0	0.58747015	0
chr1	208196373	208236865	40493	3	5	0	0.02473314	0	9	0	0.76806864	0
chr1	208236865	208339422	102558	2	5	0	0.04407651	0	8	0	0.58747015	0
chr1	208339422	208427927	88506	3	5	0	0.02473314	0	9	0	0.76806864	0
chr1	208427927	208821364	393438	2	5	0	0.04407651	0	8	0	0.58747015	0
chr1	208821364	208821423	60	1	6	0	0.04875589	0	9	0	0.5732208	0
chr1	208821423	209645905	824483	9	5	0	0.02473314	0	9	0	0.76806864	0
chr1	209645905	209760272	114368	2	5	0	0.01320236	0	10	0	0.97390707	0
chr1	209760272	210273811	513540	15	5	0	0.00666883	0	11	0	1.20557689	0
chr1	210273811	210414985	141175	2	5	0	0.02473314	0	9	0	0.76806864	0
chr1	210414985	210465054	50070	2	5	0	0.01320236	0	10	0	0.97390707	0
chr1	210465054	210572164	107111	4	5	0	0.00666883	0	11	0	1.20557689	0
chr1	210572164	210686471	114308	2	5	0	0.01320236	0	10	0	0.97390707	0
chr1	210686471	210774632	88162	3	5	0	0.00317045	0	12	0	1.46403142	0
chr1	210774632	210889791	115160	2	5	0	0.01320236	0	10	0	0.97390707	0
chr1	210889791	210940349	50559	2	5	0	0.00666883	0	11	0	1.20557689	0
chr1	210940349	211004039	63691	2	5	0	0.00317045	0	12	0	1.46403142	0
chr1	211004039	211081199	77161	2	5	0	5.83E-04	0	14	0	2.06733314	0
chr1	211081199	211109547	28349	2	6	0	0.00166733	0	14	0	1.70763027	0
chr1	211109547	211226701	117155	4	6	0	0.00373523	0	13	0	1.42397267	0
chr1	211226701	211276850	50150	1	5	0	0.00141024	0	13	0	1.7506523	0
chr1	211276850	211433483	156634	4	5	0	5.83E-04	0	14	0	2.06733314	0
chr1	211433483	211520137	86655	4	5	0	2.22E-04	0	15	0	2.41659593	0
chr1	211520137	211605847	85711	2	5	0	5.83E-04	0	14	0	2.06733314	0
chr1	211605847	211840221	234375	4	5	0	0.00141024	0	13	0	1.7506523	0
chr1	211840221	211840476	256	3	5	0	5.83E-04	0	14	0	2.06733314	0
chr1	211840476	211949537	109062	2	5	0	0.00317045	0	12	0	1.46403142	0
chr1	211949537	211949596	60	1	5	0	5.83E-04	0	14	0	2.06733314	0
chr1	211949596	212021610	72015	2	5	0	0.00141024	0	13	0	1.7506523	0
chr1	212021610	212180023	158414	5	5	0	5.83E-04	0	14	0	2.06733314	0
chr1	212180023	212180082	60	1	6	0	6.86E-04	0	15	0	2.02387299	0
chr1	212180082	212277763	97682	4	6	0	0.00166733	0	14	0	1.70763027	0
chr1	212277763	212466999	189237	2	6	0	0.00778066	0	12	0	1.17038931	0
chr1	212466999	212502622	35624	2	6	0	0.00373523	0	13	0	1.42397267	0
chr1	212502622	212629086	126465	4	6	0	0.01518174	0	11	0	0.9449689	0
chr1	212629086	212792244	163159	2	6	0	0.02793176	0	10	0	0.74627054	0
chr1	212792244	212800129	7886	4	6	0	0.00778066	0	12	0	1.17038931	0
chr1	212800129	213136512	336384	7	6	0	0.01518174	0	11	0	0.9449689	0
chr1	213136512	213186303	49792	2	6	0	0.02793176	0	10	0	0.74627054	0
chr1	213186303	213186362	60	1	7	0	0.03037338	0	11	0	0.73110763	0
chr1	213186362	213596479	410118	7	6	0	0.02793176	0	10	0	0.74627054	0
chr1	213596479	213724183	127705	2	7	0	0.05232577	0	10	0	0.56314362	0
chr1	213724183	213724242	60	1	8	0	0.05490675	0	11	0	0.55623409	0
chr1	213724242	214037496	313255	3	8	0	0.08923391	0	10	0	0.4167287	0
chr1	214037496	214161654	124159	3	8	0	0.05490675	0	11	0	0.55623409	0
chr1	214161654	214161704	51	1	8	0	0.03209037	0	12	0	0.72109894	0
chr1	214161704	214209193	47490	2	8	0	0.05490675	0	11	0	0.55623409	0
chr1	214209193	215160664	951472	18	8	0	0.03209037	0	12	0	0.72109894	0
chr1	215160664	215287388	126725	4	8	0	0.05490675	0	11	0	0.55623409	0
chr1	215287388	215287447	60	1	8	0	0.03209037	0	12	0	0.72109894	0
chr1	215287447	215546016	258570	4	8	0	0.05490675	0	11	0	0.55623409	0
chr1	215546016	215795099	249084	6	8	0	0.01767679	0	13	0	0.91308053	0
chr1	215795099	215982915	187817	3	8	0	0.05490675	0	11	0	0.55623409	0
chr1	215982915	216144115	161201	3	7	0	0.03037338	0	11	0	0.73110763	0
chr1	216144115	216191211	47097	2	8	0	0.05490675	0	11	0	0.55623409	0
chr1	216191211	216373227	182017	4	9	0	0.09154957	0	11	0	0.41444892	0
chr1	216373227	216596984	223758	6	9	1	0.09154957	0.30102999	11	0	0.41444892	0
chr1	216596984	216677938	80955	2	8	1	0.05490675	0.30102999	11	0	0.55623409	0
chr1	216677938	216896675	218738	4	8	0	0.05490675	0	11	0	0.55623409	0
chr1	216896675	217229922	333248	7	8	0	0.03209037	0	12	0	0.72109894	0
chr1	217229922	217229981	60	1	8	0	0.01767679	0	13	0	0.91308053	0
chr1	217229981	217510116	280136	3	8	0	0.03209037	0	12	0	0.72109894	0
chr1	217510116	217665042	154927	3	8	0	0.01767679	0	13	0	0.91308053	0
chr1	217665042	218006521	341480	9	8	0	0.03209037	0	12	0	0.72109894	0
chr1	218006521	218055815	49295	2	8	0	0.01767679	0	13	0	0.91308053	0
chr1	218055815	218223567	167753	1	8	0	0.05490675	0	11	0	0.55623409	0
chr1	218223567	218480900	257334	3	8	0	0.13872638	0	9	0	0.30102999	0
chr1	218480900	218693490	212591	4	6	0	0.04875589	0	9	0	0.5732208	0
chr1	218693490	218693549	60	1	7	0	0.08584816	0	9	0	0.42015402	0
chr1	218693549	218841086	147538	1	6	0	0.04875589	0	9	0	0.5732208	0
chr1	218841086	219134907	293822	2	4	0	0.02074938	0	8	0	0.79906872	0
chr1	219134907	219249705	114799	2	5	0	0.04407651	0	8	0	0.58747015	0
chr1	219249705	219317337	67633	2	6	0	0.04875589	0	9	0	0.5732208	0
chr1	219317337	219383878	66542	3	7	0	0.08584816	0	9	0	0.42015402	0
chr1	219383878	219453775	69898	2	8	0	0.13872638	0	9	0	0.30102999	0
chr1	219453775	219722368	268594	2	7	0	0.13499366	0	8	0	0.30102999	0
chr1	219722368	219722427	60	1	8	0	0.13872638	0	9	0	0.30102999	0
chr1	219722427	219926238	203812	2	7	0	0.08584816	0	9	0	0.42015402	0
chr1	219926238	219926297	60	1	9	0	0.09154957	0	11	0	0.41444892	0
chr1	219926297	219963762	37466	1	8	0	0.05490675	0	11	0	0.55623409	0
chr1	219963762	220071313	107552	2	9	0	0.09154957	0	11	0	0.41444892	0
chr1	220071313	220321821	250509	10	10	0	0.09290028	0	12	0	0.41314172	0
chr1	220321821	220386251	64431	2	10	0	0.14303407	0	11	0	0.30102999	0
chr1	220386251	220437972	51722	3	10	0	0.09290028	0	12	0	0.41314172	0
chr1	220437972	220677645	239674	3	10	0	0.14303407	0	11	0	0.30102999	0
chr1	220677645	220720576	42932	2	10	0	0.09290028	0	12	0	0.41314172	0
chr1	220720576	220948405	227830	7	10	0	0.14303407	0	11	0	0.30102999	0
chr1	220948405	221044440	96036	4	10	0	0.0574087	0	13	0	0.5498098	0
chr1	221044440	221329166	284727	2	10	0	0.14303407	0	11	0	0.30102999	0
chr1	221329166	221654393	325228	2	9	0	0.09154957	0	11	0	0.41444892	0
chr1	221654393	221817860	163468	1	8	0	0.08923391	0	10	0	0.4167287	0
chr1	221817860	221947976	130117	4	8	0	0.03209037	0	12	0	0.72109894	0
chr1	221947976	222058519	110544	1	7	0	0.08584816	0	9	0	0.42015402	0
chr1	222058519	222058578	60	1	7	0	0.05232577	0	10	0	0.56314362	0
chr1	222058578	222177351	118774	1	6	0	0.02793176	0	10	0	0.74627054	0
chr1	222177351	222367840	190490	3	6	0	0.00778066	0	12	0	1.17038931	0
chr1	222367840	222762100	394261	6	9	0	0.0331093	0	13	0	0.71538971	0
chr1	222762100	222762159	60	1	10	0	0.03344703	0	14	0	0.71353304	0
chr1	222762159	222850184	88026	2	9	0	0.01817691	0	14	0	0.90719478	0
chr1	222850184	222850243	60	1	10	0	0.03344703	0	14	0	0.71353304	0
chr1	222850243	222959713	109471	2	9	0	0.0331093	0	13	0	0.71538971	0
chr1	222959713	223079424	119712	1	8	0	0.01767679	0	13	0	0.91308053	0
chr1	223079424	223172590	93167	3	9	0	0.01817691	0	14	0	0.90719478	0
chr1	223172590	223356789	184200	2	9	0	0.0331093	0	13	0	0.71538971	0
chr1	223356789	223356848	60	1	10	0	0.03344703	0	14	0	0.71353304	0
chr1	223356848	223444986	88139	1	10	0	0.0574087	0	13	0	0.5498098	0
chr1	223444986	223497808	52823	2	11	0	0.0331093	0	15	0	0.71538971	0
chr1	223497808	223719893	222086	2	11	0	0.0574087	0	14	0	0.5498098	0
chr1	223719893	223844898	125006	2	11	0	0.0331093	0	15	0	0.71538971	0
chr1	223844898	223939747	94850	2	11	0	0.09334429	0	13	0	0.41271556	0
chr1	223939747	224034322	94576	5	11	0	0.0331093	0	15	0	0.71538971	0
chr1	224034322	224304311	269990	2	10	0	0.03344703	0	14	0	0.71353304	0
chr1	224304311	224304370	60	1	10	0	0.01817691	0	15	0	0.90719478	0
chr1	224304370	224371825	67456	2	10	0	0.03344703	0	14	0	0.71353304	0

chr1	224371825	224424250	52426	4	11	0	0.0574087	0	14	0	0.5498098	0
chr1	224424250	224702757	278508	6	10	0	0.03344703	0	14	0	0.71353304	0
chr1	224702757	224752562	49806	2	10	0	0.01817691	0	15	0	0.90719478	0
chr1	224752562	224902680	150119	4	10	0	0.03344703	0	14	0	0.71353304	0
chr1	224902680	224902739	60	1	11	0	0.0574087	0	14	0	0.5498098	0
chr1	224902739	224928253	25515	1	10	0	0.03344703	0	14	0	0.71353304	0
chr1	224928253	224952932	24680	1	10	0	0.0574087	0	13	0	0.5498098	0
chr1	224952932	225090272	137341	2	11	0	0.09334429	0	13	0	0.41271556	0
chr1	225090272	225339772	249501	5	11	0	0.0574087	0	14	0	0.5498098	0
chr1	225339772	225394432	54661	1	10	0	0.03344703	0	14	0	0.71353304	0
chr1	225394432	225446243	51812	2	10	0	0.01817691	0	15	0	0.90719478	0
chr1	225446243	225601204	154962	4	11	0	0.0331093	0	15	0	0.71538971	0
chr1	225601204	225616476	15273	2	11	0	0.0574087	0	14	0	0.5498098	0
chr1	225616476	225691534	75059	2	11	0	0.0331093	0	15	0	0.71538971	0
chr1	225691534	225718291	26758	2	12	0	0.03209037	0	16	0	0.72109894	0
chr1	225718291	225837855	119565	2	12	0	0.0565833	0	15	0	0.55190077	0
chr1	225837855	226541451	703597	15	11	0	0.0574087	0	14	0	0.5498098	0
chr1	226541451	226584688	43238	4	12	0	0.09290028	0	14	0	0.41314172	0
chr1	226584688	226871602	286915	5	11	0	0.0574087	0	14	0	0.5498098	0
chr1	226871602	227009610	138009	3	12	0	0.09290028	0	14	0	0.41314172	0
chr1	227009610	227071152	61543	3	13	0	0.14303407	0	14	0	0.30102999	0
chr1	227071152	227180677	109526	3	12	0	0.14385241	0	13	0	0.30102999	0
chr1	227180677	227239910	59234	2	13	0	0.21200206	0	13	0	0.21200206	0
chr1	227239910	227330649	90740	2	13	0	0.30102999	0	12	0	0.14385241	0
chr1	227330649	227330708	60	1	13	0	0.14303407	0	14	0	0.30102999	0
chr1	227330708	227504757	174050	3	13	0	0.21200206	0	13	0	0.21200206	0
chr1	227504757	227669534	164778	2	12	0	0.14385241	0	13	0	0.30102999	0
chr1	227669534	227721097	51564	2	12	0	0.14385241	0	13	1	0.30102999	0.30102999
chr1	227721097	227810271	89175	2	12	0	0.30102999	0	11	1	0.14385241	0.30102999
chr1	227810271	227849204	38934	4	12	0	0.21227046	0	12	1	0.21227046	0.30102999
chr1	227849204	227894486	45283	1	11	0	0.14385241	0	12	1	0.30102999	0.30102999
chr1	227894486	227922416	27931	1	11	0	0.21200206	0	11	1	0.21200206	0.30102999
chr1	227922416	227926333	3918	2	12	0	0.30102999	0	11	1	0.14385241	0.30102999
chr1	227926333	227964074	37742	3	12	0	0.21227046	0	12	1	0.21227046	0.30102999
chr1	227964074	228026364	62291	1	11	0	0.14385241	0	12	1	0.30102999	0.30102999
chr1	228026364	228123496	97133	1	11	0	0.14385241	0	12	0	0.30102999	0
chr1	228123496	228510600	387105	16	12	0	0.21227046	0	12	0	0.21227046	0
chr1	228510600	228787740	277141	8	11	0	0.14385241	0	12	0	0.30102999	0
chr1	228787740	228825776	38037	2	11	0	0.09334429	0	13	0	0.41271556	0
chr1	228825776	228880117	54342	3	11	0	0.0574087	0	14	0	0.5498098	0
chr1	228880117	228997888	117772	1	11	0	0.09334429	0	13	0	0.41271556	0
chr1	228997888	229212426	214539	2	10	0	0.0574087	0	13	0	0.5498098	0
chr1	229212426	229424529	212104	2	10	0	0.09290028	0	12	0	0.41314172	0
chr1	229424529	229477313	52785	3	10	0	0.0574087	0	13	0	0.5498098	0
chr1	229477313	229477372	60	1	10	0	0.03344703	0	14	0	0.71353304	0
chr1	229477372	229600390	123019	4	10	0	0.0574087	0	13	0	0.5498098	0
chr1	229600390	229619882	19493	2	10	0	0.03344703	0	14	0	0.71353304	0
chr1	229619882	229795290	175409	6	10	0	0.0574087	0	13	0	0.5498098	0
chr1	229795290	230141642	346353	4	10	0	0.03344703	0	14	0	0.71353304	0
chr1	230141642	230332201	190560	6	11	0	0.0574087	0	14	0	0.5498098	0
chr1	230332201	230332260	60	1	12	0	0.09290028	0	14	0	0.41314172	0
chr1	230332260	230405964	73705	2	11	0	0.0574087	0	14	0	0.5498098	0
chr1	230405964	230450225	44262	2	12	0	0.09290028	0	14	0	0.41314172	0
chr1	230450225	230450284	60	1	13	0	0.14303407	0	14	0	0.30102999	0
chr1	230450284	230477751	27468	1	12	0	0.09290028	0	14	0	0.41314172	0
chr1	230477751	230557199	79449	3	13	0	0.14303407	0	14	0	0.30102999	0
chr1	230557199	230824675	267477	4	12	0	0.09290028	0	14	0	0.41314172	0
chr1	230824675	231042452	217778	6	11	0	0.0574087	0	14	0	0.5498098	0
chr1	231042452	231119461	77010	3	11	1	0.0574087	0.30102999	14	0	0.5498098	0
chr1	231119461	231320451	200991	7	12	1	0.09290028	0.30102999	14	0	0.41314172	0
chr1	231320451	231376250	55800	2	12	0	0.09290028	0	14	0	0.41314172	0
chr1	231376250	231449141	72892	3	13	0	0.14303407	0	14	0	0.30102999	0
chr1	231449141	231469697	20557	2	13	1	0.14303407	0.30102999	14	0	0.30102999	0
chr1	231469697	231474669	4973	1	13	1	0.21200206	0.30102999	13	0	0.21200206	0
chr1	231474669	231523497	48829	1	13	0	0.21200206	0	13	0	0.21200206	0
chr1	231523497	231523556	60	1	13	0	0.14303407	0	14	0	0.30102999	0
chr1	231523556	231559907	36352	1	12	0	0.09290028	0	14	0	0.41314172	0
chr1	231559907	231559966	60	1	12	0	0.0565833	0	15	0	0.55190077	0
chr1	231559966	231685406	125441	2	12	0	0.09290028	0	14	0	0.41314172	0
chr1	231685406	231737926	52521	2	13	0	0.09154957	0	15	0	0.41444892	0
chr1	231737926	231737985	60	1	13	1	0.09154957	0.30102999	15	0	0.41444892	0
chr1	231737985	231788366	50382	1	13	1	0.14303407	0.30102999	14	0	0.30102999	0
chr1	231788366	231859742	71377	2	12	1	0.09290028	0.30102999	14	0	0.41314172	0
chr1	231859742	231935770	76029	1	10	1	0.03344703	0.30102999	14	0	0.71353304	0
chr1	231935770	232002490	66721	1	9	1	0.0331093	0.30102999	13	0	0.71538971	0
chr1	232002490	232072932	70443	3	10	1	0.0574087	0.30102999	13	0	0.5498098	0
chr1	232072932	232191881	118950	3	10	1	0.03344703	0.30102999	14	0	0.71353304	0
chr1	232191881	232308868	116988	1	10	0	0.03344703	0	14	0	0.71353304	0
chr1	232308868	232383904	75037	1	10	0	0.0574087	0	13	0	0.5498098	0
chr1	232383904	232540308	156405	2	10	1	0.0574087	0.30102999	13	0	0.5498098	0
chr1	232540308	232605805	65498	2	10	1	0.03344703	0.30102999	14	0	0.71353304	0
chr1	232605805	232643611	37807	1	10	1	0.0574087	0.30102999	13	0	0.5498098	0
chr1	232643611	232825288	181678	3	9	1	0.0331093	0.30102999	13	0	0.71538971	0
chr1	232825288	232825347	60	1	9	1	0.0331093	0.1218695	13	1	0.71538971	0.1218695
chr1	232825347	232942284	116938	1	9	1	0.0331093	0.30102999	13	0	0.71538971	0
chr1	232942284	232942343	60	1	10	1	0.03344703	0.30102999	14	0	0.71353304	0
chr1	232942343	232981590	39248	2	10	1	0.0574087	0.30102999	13	0	0.5498098	0
chr1	232981590	233091303	109714	2	10	1	0.09290028	0.30102999	12	0	0.41314172	0
chr1	233091303	233152905	61603	3	10	1	0.03344703	0.30102999	14	0	0.71353304	0
chr1	233152905	233193009	40105	1	10	1	0.0574087	0.30102999	13	0	0.5498098	0
chr1	233193009	233284641	91633	2	10	0	0.0574087	0	13	0	0.5498098	0
chr1	233284641	233284700	60	1	11	0	0.09334429	0	13	0	0.41271556	0
chr1	233284700	233422815	138116	4	10	0	0.0574087	0	13	0	0.5498098	0
chr1	233422815	233511622	88808	5	10	1	0.03344703	0.30102999	14	0	0.71353304	0
chr1	233511622	233649171	137550	3	11	1	0.0574087	0.30102999	14	0	0.5498098	0
chr1	233649171	233768132	118962	1	11	0	0.0574087	0	14	0	0.5498098	0
chr1	233768132	233998258	230127	6	12	0	0.09290028	0	14	0	0.41314172	0
chr1	233998258	234070031	71774	2	12	0	0.0565833	0	15	0	0.55190077	0
chr1	234070031	234070090	60	1	12	0	0.03209037	0	16	0	0.72109894	0
chr1	234070090	234273831	203742	4	12	0	0.0565833	0	15	0	0.55190077	0
chr1	234273831	234536914	263084	5	11	0	0.0574087	0	14	0	0.5498098	0
chr1	234536914	234665501	128588	5	11	0	0.0331093	0	15	0	0.71538971	0
chr1	234665501	234774725	109225	4	12	0	0.0565833	0	15	0	0.55190077	0
chr1	234774725	234867432	92708	2	13	0	0.09154957	0	15	0	0.41444892	0
chr1	234867432	234973514	106083	2	14	0	0.14135546	0	15	0	0.30102999	0
chr1	234973514	234973573	60	1	14	1	0.14135546	0.30102999	15	0	0.30102999	0
chr1	234973573	235046384	72812	1	14	0	0.14135546	0	15	0	0.30102999	0
chr1	235046384	235046443	60	1	14	0	0.08923391	0	16	0	0.4167287	0
chr1	235046443	235155872	109430	1	14	0	0.14135546	0	15	0	0.30102999	0
chr1	235155872	235267544	111673	1	14	0	0.21118145	0	14	0	0.21118145	0
chr1	235267544	235267603	60	1	15	1	0.13872638	0.30102999	16	0	0.30102999	0
chr1	235267603	235345183	77581	2	15	1	0.41444892	0.30102999	13	0	0.09154957	0
chr1	235345183	235383222	38040	2	15	1	0.30102999	0.30102999	14	0	0.14135546	0
chr1	235383222	235420451	37230	1	15	0	0.30102999	0	14	0	0.14135546	0
chr1	235420451	235420510	60	1	15	0	0.20975986	0	15	0	0.20975986	0
chr1	235420510	235551307	130798	3	15	0	0.30102999	0	14	0	0.14135546	0
chr1	235551307	235681301	129995	5	15	0	0.20975986	0	15	0	0.20975986	0
chr1	235681301	235681360	60	1	15	1	0.20975986	0.30102999	15	0	0.20975986	0
chr1	235681360	235715011	33652	1	14	0	0.14135546	0	15	0	0.30102999	0
chr1	235715011	235759126	44116	1	13	0	0.14303407	0	14	0	0.30102999	0
chr1	235759126	235759185	60	1	13	1	0.14303407	0.30102999	14	0	0.30102999	0
chr1	235759185	235875499	116315	3	12	1	0.09290028	0.30102999	14	0	0.41314172	0
chr1	235875499	236139440	263942	6	12	1	0.14385241	0.30102999	13	0	0.30102999	0
chr1	236139440	236180573	41134	3	12	2	0.14385241	0.61140001	13	0	0.30102999	0

chr1	236180573	236286038	105466	3	12	1	0.14385241	0.30102999	13	0	0.30102999	0
chr1	236286038	236332057	46020	1	12	1	0.21227046	0.30102999	12	0	0.21227046	0
chr1	236332057	236389691	57635	2	12	1	0.30102999	0.30102999	11	0	0.14385241	0
chr1	236389691	236442263	52573	2	12	1	0.21227046	0.30102999	12	0	0.21227046	0
chr1	236442263	236442322	60	1	13	1	0.30102999	0.30102999	12	0	0.14385241	0
chr1	236442322	236554819	112498	1	13	1	0.41271556	0.30102999	11	0	0.09334429	0
chr1	236554819	236572538	17720	2	14	1	0.41314172	0.30102999	12	0	0.09290028	0
chr1	236572538	236631570	59033	3	14	1	0.30102999	0.30102999	13	0	0.14303407	0
chr1	236631570	236748164	116595	5	14	0	0.30102999	0	13	0	0.14303407	0
chr1	236748164	236852256	104093	1	12	0	0.21227046	0	12	0	0.21227046	0
chr1	236852256	236889896	37641	2	12	1	0.21227046	0.30102999	12	0	0.21227046	0
chr1	236889896	236961331	71436	3	13	1	0.21200206	0.30102999	13	0	0.21200206	0
chr1	236961331	236999014	37684	3	13	1	0.14303407	0.30102999	14	0	0.30102999	0
chr1	236999014	237060949	61936	2	12	0	0.14385241	0	13	0	0.30102999	0
chr1	237060949	237127282	66334	2	12	0	0.09290028	0	14	0	0.41314172	0
chr1	237127282	237127341	60	1	14	0	0.21118145	0	14	0	0.21118145	0
chr1	237127341	237205104	77764	1	13	0	0.21200206	0	13	0	0.21200206	0
chr1	237205104	237604669	399566	8	12	0	0.14385241	0	13	0	0.30102999	0
chr1	237604669	237728098	123430	3	13	0	0.21200206	0	13	0	0.21200206	0
chr1	237728098	237728157	60	1	13	0	0.14303407	0	14	0	0.30102999	0
chr1	237728157	237817664	89508	2	13	0	0.30102999	0	12	0	0.14385241	0
chr1	237817664	238362806	545143	9	13	0	0.14303407	0	14	0	0.30102999	0
chr1	238362806	238650957	288152	3	11	0	0.0574087	0	14	0	0.5498098	0
chr1	238650957	238888870	237914	2	11	0	0.14385241	0	12	0	0.30102999	0
chr1	238888870	239011061	122192	2	11	0	0.0574087	0	14	0	0.5498098	0
chr1	239011061	239459749	448689	3	11	0	0.14385241	0	12	0	0.30102999	0
chr1	239459749	239721730	261982	3	12	0	0.09290028	0	14	0	0.41314172	0
chr1	239721730	239927943	206214	5	12	1	0.09290028	0.30102999	14	0	0.41314172	0
chr1	239927943	239928002	60	1	14	1	0.21118145	0.30102999	14	0	0.21118145	0
chr1	239928002	240193465	265464	6	13	1	0.14303407	0.30102999	14	0	0.30102999	0
chr1	240193465	240313763	120299	3	14	1	0.21118145	0.30102999	14	0	0.21118145	0
chr1	240313763	240681465	367703	9	13	1	0.14303407	0.30102999	14	0	0.30102999	0
chr1	240681465	240773332	91868	4	13	1	0.09154957	0.30102999	15	0	0.41444892	0
chr1	240773332	240773391	60	1	14	1	0.14135546	0.30102999	15	0	0.30102999	0
chr1	240773391	241031936	258546	5	13	1	0.09154957	0.30102999	15	0	0.41444892	0
chr1	241031936	241031995	60	1	14	1	0.14135546	0.30102999	15	0	0.30102999	0
chr1	241031995	241178061	146067	2	14	1	0.21118145	0.30102999	14	0	0.21118145	0
chr1	241178061	241225681	47621	2	14	1	0.14135546	0.30102999	15	0	0.30102999	0
chr1	241225681	241337270	111590	3	14	1	0.08923391	0.30102999	16	0	0.4167287	0
chr1	241337270	241337329	60	1	14	1	0.05232577	0.30102999	17	0	0.56314362	0
chr1	241337329	241380807	43479	1	14	1	0.14135546	0.30102999	15	0	0.30102999	0
chr1	241380807	241380866	60	1	15	1	0.20975986	0.30102999	15	0	0.20975986	0
chr1	241380866	241439921	59056	1	15	0	0.20975986	0	15	0	0.20975986	0
chr1	241439921	241439980	60	1	15	0	0.08584816	0	17	0	0.42015402	0
chr1	241439980	241522090	82111	2	15	0	0.13872638	0	16	0	0.30102999	0
chr1	241522090	241680610	158521	2	14	0	0.08923391	0	16	0	0.4167287	0
chr1	241680610	241704844	24235	1	14	0	0.14135546	0	15	0	0.30102999	0
chr1	241704844	241757184	52341	2	13	0	0.09154957	0	15	0	0.41444892	0
chr1	241757184	241799086	41903	2	14	0	0.14135546	0	15	0	0.30102999	0
chr1	241799086	241799145	60	1	15	0	0.20975986	0	15	0	0.20975986	0
chr1	241799145	241857097	57953	1	13	0	0.09154957	0	15	0	0.41444892	0
chr1	241857097	241913757	56661	1	12	0	0.0565833	0	15	0	0.55190077	0
chr1	241913757	241951077	37321	3	13	0	0.09154957	0	15	0	0.41444892	0
chr1	241951077	242023977	72901	4	14	0	0.14135546	0	15	0	0.30102999	0
chr1	242023977	242151589	127613	3	13	0	0.09154957	0	15	0	0.41444892	0
chr1	242151589	242223280	71692	2	14	0	0.14135546	0	15	0	0.30102999	0
chr1	242223280	242287764	64485	2	12	0	0.0565833	0	15	0	0.55190077	0
chr1	242287764	242287823	60	1	13	0	0.09154957	0	15	0	0.41444892	0
chr1	242287823	242357208	69386	1	11	0	0.0331093	0	15	0	0.71538971	0
chr1	242357208	242404099	46892	2	12	0	0.0565833	0	15	0	0.55190077	0
chr1	242404099	242404158	60	1	12	0	0.03209037	0	16	0	0.72109894	0
chr1	242404158	242442098	37941	1	12	0	0.0565833	0	15	0	0.55190077	0
chr1	242442098	242442157	60	1	13	0	0.09154957	0	15	0	0.41444892	0
chr1	242442157	242512907	70751	1	12	0	0.0565833	0	15	0	0.55190077	0
chr1	242512907	242573871	60965	2	12	0	0.03209037	0	16	0	0.72109894	0
chr1	242573871	242607168	33298	2	13	0	0.05490675	0	16	0	0.55623409	0
chr1	242607168	242656519	49352	2	14	0	0.08923391	0	16	0	0.4167287	0
chr1	242656519	242772314	115796	1	13	0	0.05490675	0	16	0	0.55623409	0
chr1	242772314	242987737	215424	2	13	0	0.09154957	0	15	0	0.41444892	0
chr1	242987737	242987796	60	1	13	0	0.05490675	0	16	0	0.55623409	0
chr1	242987796	243309051	321256	1	13	0	0.09154957	0	15	0	0.41444892	0
chr1	243309051	243348977	39927	2	14	0	0.14135546	0	15	0	0.30102999	0
chr1	243348977	243456375	107399	2	11	0	0.0331093	0	15	0	0.71538971	0
chr1	243456375	243456434	60	1	14	0	0.14135546	0	15	0	0.30102999	0
chr1	243456434	243542020	85587	2	13	0	0.14303407	0	14	0	0.30102999	0
chr1	243542020	243542079	60	1	14	0	0.21118145	0	14	0	0.21118145	0
chr1	243542079	243736246	194168	5	13	0	0.14303407	0	14	0	0.30102999	0
chr1	243736246	243736301	56	1	13	0	0.09154957	0	15	0	0.41444892	0
chr1	243736301	243809193	72893	1	12	0	0.09290028	0	14	0	0.41314172	0
chr1	243809193	243809252	60	1	13	0	0.09154957	0	15	0	0.41444892	0
chr1	243809252	243880099	70848	1	12	0	0.0565833	0	15	0	0.55190077	0
chr1	243880099	243880158	60	1	12	0	0.03209037	0	16	0	0.72109894	0
chr1	243880158	243949931	69774	2	12	0	0.09290028	0	14	0	0.41314172	0
chr1	243949931	244033798	83868	3	13	0	0.14303407	0	14	0	0.30102999	0
chr1	244033798	244305407	271610	5	14	0	0.14135546	0	15	0	0.30102999	0
chr1	244305407	244572034	266628	5	14	0	0.08923391	0	16	0	0.4167287	0
chr1	244572034	245019894	447861	10	14	0	0.14135546	0	15	0	0.30102999	0
chr1	245019894	245617025	597132	16	15	0	0.20975986	0	15	0	0.20975986	0
chr1	245617025	246116583	499559	11	15	0	0.13872638	0	16	0	0.30102999	0
chr1	246116583	246201500	84918	2	14	0	0.08923391	0	16	0	0.4167287	0
chr1	246201500	246550260	348761	7	14	0	0.14135546	0	15	0	0.30102999	0
chr1	246550260	246704463	154204	2	12	0	0.0565833	0	15	0	0.55190077	0
chr1	246704463	246731512	27050	2	13	0	0.09154957	0	15	0	0.41444892	0
chr1	246731512	246731571	60	1	14	0	0.14135546	0	15	0	0.30102999	0
chr1	246731571	246831012	99442	3	13	0	0.09154957	0	15	0	0.41444892	0
chr1	246831012	246890227	59216	3	14	0	0.14135546	0	15	1	0.30102999	0.30102999
chr1	246890227	246931110	40884	1	13	0	0.09154957	0	15	1	0.41444892	0.30102999
chr1	246931110	247009584	78475	2	12	0	0.0565833	0	15	1	0.55190077	0.30102999
chr1	247009584	247074401	64818	2	13	0	0.09154957	0	15	1	0.41444892	0.30102999
chr1	247074401	247074460	60	1	13	0	0.05490675	0	16	1	0.55623409	0.30102999
chr1	247074460	247203067	128608	5	13	0	0.09154957	0	15	1	0.41444892	0.30102999
chr1	247203067	247320972	117906	3	12	0	0.0565833	0	15	1	0.55190077	0.30102999
chr1	247320972	247348787	27816	2	13	0	0.09154957	0	15	1	0.41444892	0.30102999
chr1	247348787	247348846	60	1	13	0	0.05490675	0	16	1	0.55623409	0.30102999
chr1	247348846	247419744	70899	1	12	0	0.03209037	0	16	1	0.72109894	0.30102999
chr1	247419744	247473765	54022	1	11	0	0.01767679	0	16	1	0.91308053	0.30102999
chr1	247473765	247489684	15920	1	10	0	0.00909834	0	16	1	1.13428373	0.30102999
chr1	247489684	247581464	91781	1	10	0	0.01817691	0	15	1	0.90719478	0.30102999
chr1	247581464	247605064	23601	2	12	0	0.0565833	0	15	1	0.55190077	0.30102999
chr1	247605064	247712522	107459	3	11	0	0.0331093	0	15	1	0.71538971	0.30102999
chr1	247712522	247879926	167405	3	11	0	0.0574087	0	14	1	0.5498098	0.30102999
chr1	247879926	247927234	47309	2	11	0	0.0331093	0	15	2	0.71538971	0.61140001
chr1	247927234	247927293	60	1	11	1	0.0331093	0.05404976	15	2	0.71538971	0.30102999
chr1	247927293	248004027	76735	1	11	1	0.0574087	0.05404976	14	2	0.5498098	0.30102999
chr1	248004027	248021883	17857	1	11	1	0.0574087	0.1218695	14	1	0.5498098	0.1218695
chr1	248021883	248060419	38537	3	11	1	0.0331093	0.1218695	15	1	0.71538971	0.1218695
chr1	248060419	248131353	70935	1	11	0	0.0574087	0	14	1	0.5498098	0.30102999
chr1	248131353	248131412	60	1	11	0	0.0574087	0	14	2	0.5498098	0.61140001
chr1	248131412	248179055	47644	1	11	0	0.0574087	0	14	1	0.5498098	0.30102999
chr1	248179055	248203263	24209	2	11	0	0.0331093	0	15	1	0.71538971	0.30102999
chr1	248203263	248262713	59451	2	11	1	0.0331093	0.1218695	15	1	0.71538971	0.1218695
chr1	248262713	248360905	98193	3	11	0	0.0331093	0	15	1	0.71538971	0.30102999
chr1	248360905	248438406	77502	2	11	1	0.0331093	0.1218695	15	1	0.71538971	0.1218695
chr1	248438406	248438465	60	1	13	1	0.09154957	0.1218695	15	1	0.41444892	0.1218695

chr1	248438465	248684850	246386	5	11	0	0.0331093	0	15	1	0.71538971	0.30102999
chr1	248684850	248684909	60	1	11	1	0.0331093	0.1218695	15	1	0.71538971	0.1218695
chr1	248684909	248745802	60894	1	11	1	0.0331093	0.30102999	15	0	0.71538971	0
chr1	248745802	248786842	41041	2	12	1	0.0565833	0.30102999	15	0	0.55190077	0
chr1	248786842	248904492	117651	2	11	1	0.0574087	0.30102999	14	0	0.5498098	0
chr1	248904492	249212668	308177	6	11	1	0.30102999	0.30102999	10	0	0.14303407	0
chr2	42444	84669	42226	2	2	2	0.1575501	0.61140001	2	0	0.1575501	0
chr2	84669	84728	60	1	2	2	0.00493743	0.1575501	7	2	1.16581773	0.1575501
chr2	84728	272602	187875	2	2	2	0.01053319	0.61140001	6	0	0.91219088	0
chr2	272602	272661	60	1	2	2	0.01053319	0.1575501	6	2	0.91219088	0.1575501
chr2	272661	386460	113800	2	2	2	0.02162467	0.61140001	5	0	0.68214471	0
chr2	386460	386519	60	1	3	2	0.01598258	0.61140001	7	0	0.84395715	0
chr2	386519	456961	70443	1	3	2	0.03070643	0.61140001	6	0	0.63695542	0
chr2	456961	457020	60	1	3	2	0.03070643	0.30102999	6	1	0.63695542	0.05404976
chr2	457020	506983	49964	1	3	2	0.03070643	0.61140001	6	0	0.63695542	0
chr2	506983	642724	135742	3	3	2	0.01598258	0.1575501	7	2	0.84395715	0.1575501
chr2	642724	768496	125773	3	3	2	0.01598258	0.08289318	7	3	0.84395715	0.30102999
chr2	768496	883836	115341	3	3	3	0.01598258	0.17593012	7	3	0.84395715	0.17593012
chr2	883836	978269	94434	3	4	3	0.03812622	0.17593012	7	3	0.60763643	0.17593012
chr2	978269	1151213	172945	5	4	3	0.03812622	0.10122019	7	4	0.60763643	0.30102999
chr2	1151213	1286051	134839	3	3	3	0.01598258	0.10122019	7	4	0.84395715	0.30102999
chr2	1286051	1286110	60	1	3	3	0.0079614	0.10122019	8	4	1.07548421	0.30102999
chr2	1286110	1318927	32818	1	3	3	0.01598258	0.10122019	7	4	0.84395715	0.30102999
chr2	1318927	1467170	148244	4	4	3	0.03812622	0.10122019	7	4	0.60763643	0.30102999
chr2	1467170	1587525	120356	2	4	3	0.06713722	0.10122019	6	4	0.44141547	0.30102999
chr2	1587525	1635643	48119	1	3	3	0.03070643	0.10122019	6	4	0.63695542	0.30102999
chr2	1635643	1635702	60	1	3	3	0.01598258	0.10122019	7	4	0.84395715	0.30102999
chr2	1635702	1842130	206429	4	3	3	0.03070643	0.10122019	6	4	0.63695542	0.30102999
chr2	1842130	1973115	130986	2	3	3	0.03070643	0.17593012	6	3	0.63695542	0.17593012
chr2	1973115	2063821	90707	3	3	3	0.01598258	0.17593012	7	3	0.84395715	0.17593012
chr2	2063821	2063880	60	1	3	3	0.01598258	0.10122019	7	4	0.84395715	0.30102999
chr2	2063880	2144860	80981	1	3	3	0.03070643	0.10122019	6	4	0.63695542	0.30102999
chr2	2144860	2198678	53819	1	3	3	0.05670724	0.10122019	5	4	0.45545077	0.30102999
chr2	2198678	2198737	60	1	4	3	0.11390336	0.10122019	5	4	0.30102999	0.30102999
chr2	2198737	2550621	351885	5	3	3	0.05670724	0.10122019	5	4	0.45545077	0.30102999
chr2	2550621	2606073	55453	1	3	3	0.10122019	0.17593012	4	3	0.30102999	0.17593012
chr2	2606073	2688584	82512	1	3	3	0.17593012	0.17593012	3	3	0.17593012	0.17593012
chr2	2688584	2859209	170626	3	3	3	0.10122019	0.17593012	4	3	0.30102999	0.17593012
chr2	2859209	3501952	642744	9	3	2	0.10122019	0.08289318	4	3	0.30102999	0.30102999
chr2	3501952	3584739	82788	2	4	2	0.18734596	0.08289318	4	3	0.18734596	0.30102999
chr2	3584739	3627380	42642	2	5	2	0.30102999	0.08289318	4	3	0.11390336	0.30102999
chr2	3627380	3680384	53005	2	5	2	0.12309572	0.08289318	6	3	0.30102999	0.30102999
chr2	3680384	3729182	48799	1	5	2	0.19510895	0.08289318	5	3	0.19510895	0.30102999
chr2	3729182	3729241	60	1	5	2	0.12309572	0.08289318	6	3	0.30102999	0.30102999
chr2	3729241	3792291	63051	1	5	2	0.12309572	0.1575501	6	2	0.30102999	0.1575501
chr2	3792291	3897248	104958	3	5	2	0.07511598	0.1575501	7	2	0.43181735	0.1575501
chr2	3897248	4120428	223181	3	4	2	0.03812622	0.1575501	7	2	0.60763643	0.1575501
chr2	4120428	4283717	163290	3	3	2	0.01598258	0.1575501	7	2	0.84395715	0.1575501
chr2	4283717	4514232	230516	3	2	2	0.00493743	0.1575501	7	2	1.16581773	0.1575501
chr2	4514232	4647940	133709	2	2	1	0.02162467	0.1218695	5	1	0.68214471	0.1218695
chr2	4647940	4745093	97154	2	2	2	0.02162467	0.30102999	5	1	0.68214471	0.05404976
chr2	4745093	4745152	60	1	2	2	0.01053319	0.30102999	6	1	0.91219088	0.05404976
chr2	4745152	4981582	236431	3	1	1	0.00478973	0.1218695	5	1	1.02643191	0.1218695
chr2	4981582	5309018	327437	1	1	1	0.01091641	0.30102999	4	0	0.76005302	0
chr2	5309018	5494126	185109	2	1	2	0.01091641	0.61140001	4	0	0.76005302	0
chr2	5494126	5727968	233843	1	1	2	0.02438896	0.61140001	3	0	0.51676182	0
chr2	5727968	5838952	110985	1	1	1	0.02438896	0.30102999	3	0	0.51676182	0
chr2	5838952	6016525	177574	1	1	1	0.05404976	0.30102999	2	0	0.30102999	0
chr2	6016525	6135104	118580	1	1	0	0.05404976	0	2	0	0.30102999	0
chr2	6135104	6135163	60	1	1	1	0.00478973	0.1218695	5	1	1.02643191	0.1218695
chr2	6135163	6559225	424063	2	0	1	0	0.1218695	4	1	1.26272838	0.1218695
chr2	6559225	6559284	60	1	1	2	0.01091641	0.1575501	4	2	0.76005302	0.1575501
chr2	6559284	6631393	72110	1	1	2	0.02438896	0.1575501	3	2	0.51676182	0.1575501
chr2	6631393	6772153	140761	1	1	1	0.05404976	0.05404976	2	2	0.30102999	0.30102999
chr2	6772153	6772212	60	1	1	2	0.02438896	0.1575501	3	2	0.51676182	0.1575501
chr2	6772212	6872681	100470	1	1	1	0.02438896	0.05404976	3	2	0.51676182	0.30102999
chr2	6872681	6872740	60	1	2	1	0.08289318	0.05404976	3	2	0.30102999	0.30102999
chr2	6872740	7182324	309585	6	2	1	0.08289318	0.1218695	3	1	0.30102999	0.1218695
chr2	7182324	7304259	121936	2	3	1	0.10122019	0.05404976	4	2	0.30102999	0.30102999
chr2	7304259	7669507	365249	3	2	1	0.0429175	0.1218695	4	1	0.47744371	0.1218695
chr2	7669507	7669566	60	1	2	2	0.0429175	0.1575501	4	2	0.47744371	0.1575501
chr2	7669566	7760558	90993	1	2	1	0.0429175	0.05404976	4	2	0.47744371	0.30102999
chr2	7760558	8046583	286026	3	1	1	0.01091641	0.05404976	4	2	0.76005302	0.30102999
chr2	8046583	8113345	66763	1	0	1	0	0.05404976	4	2	1.26272838	0.30102999
chr2	8113345	8113404	60	1	1	1	0.01091641	0.05404976	4	2	0.76005302	0.30102999
chr2	8113404	8260791	147388	2	1	0	0.01091641	0	4	2	0.76005302	0.61140001
chr2	8260791	8260850	60	1	2	0	0.0429175	0	4	2	0.47744371	0.61140001
chr2	8260850	8809143	548294	4	2	0	0.0429175	0	4	1	0.47744371	0.30102999
chr2	8809143	9217329	408187	8	2	2	0.0429175	0.1575501	4	2	0.47744371	0.1575501
chr2	9217329	9364692	147364	2	2	1	0.0429175	0.1218695	4	1	0.47744371	0.1218695
chr2	9364692	9453436	88745	3	2	1	0.0429175	0.05404976	4	2	0.47744371	0.30102999
chr2	9453436	9549012	95577	2	2	1	0.0429175	0.1218695	4	1	0.47744371	0.1218695
chr2	9549012	9633089	84078	3	2	1	0.0429175	0.05404976	4	2	0.47744371	0.30102999
chr2	9633089	9633148	60	1	2	2	0.0429175	0.08289318	4	3	0.47744371	0.30102999
chr2	9633148	9766950	133803	4	2	1	0.0429175	0.02438896	4	3	0.47744371	0.51676182
chr2	9766950	9767009	60	1	3	1	0.10122019	0.02438896	4	3	0.30102999	0.51676182
chr2	9767009	9955630	188622	2	3	1	0.10122019	0.05404976	4	2	0.30102999	0.30102999
chr2	9955630	10348261	392632	10	3	2	0.10122019	0.08289318	4	3	0.30102999	0.30102999
chr2	10348261	10454342	106082	2	3	2	0.10122019	0.1575501	4	2	0.30102999	0.1575501
chr2	10454342	10454401	60	1	4	2	0.18734596	0.08289318	4	3	0.18734596	0.30102999
chr2	10454401	10490106	35706	1	4	2	0.18734596	0.1575501	4	2	0.18734596	0.1575501
chr2	10490106	10526561	36456	2	5	2	0.30102999	0.1575501	4	2	0.11390336	0.1575501
chr2	10526561	10711719	185159	5	4	2	0.18734596	0.1575501	4	2	0.18734596	0.1575501
chr2	10711719	10711778	60	1	5	2	0.30102999	0.1575501	4	2	0.11390336	0.1575501
chr2	10711778	10797965	86188	2	5	1	0.30102999	0.05404976	4	2	0.11390336	0.30102999
chr2	10797965	10924176	126212	3	5	0	0.30102999	0	4	2	0.11390336	0.61140001
chr2	10924176	10942708	18533	2	5	1	0.30102999	0.02438896	4	3	0.11390336	0.51676182
chr2	10942708	10988915	46208	1	5	0	0.30102999	0	4	2	0.11390336	0.61140001
chr2	10988915	11593842	604928	11	5	0	0.30102999	0	4	1	0.11390336	0.30102999
chr2	11593842	11678622	84781	3	5	0	0.19510895	0	5	1	0.19510895	0.30102999
chr2	11678622	11731941	53320	2	5	0	0.12309572	0	6	1	0.30102999	0.30102999
chr2	11731941	11907955	176015	4	5	0	0.19510895	0	5	1	0.19510895	0.30102999
chr2	11907955	12039338	131384	3	5	0	0.30102999	0	4	1	0.11390336	0.30102999
chr2	12039338	12166951	127614	1	4	0	0.18734596	0	4	1	0.18734596	0.30102999
chr2	12166951	12354092	187142	3	4	0	0.06713722	0	6	1	0.44141547	0.30102999
chr2	12354092	12575128	221037	3	3	0	0.05670724	0	5	1	0.45545077	0.30102999
chr2	12575128	12668284	93157	1	2	0	0.02162467	0	5	1	0.68214471	0.30102999
chr2	12668284	12720801	52518	2	2	0	0.01053319	0	6	1	0.91219088	0.30102999
chr2	12720801	13046700	325900	4	2	0	0.01053319	0	6	0	0.91219088	0
chr2	13046700	13153313	106614	1	2	0	0.02162467	0	5	0	0.68214471	0
chr2	13153313	13301606	148294	1	2	0	0.0429175	0	4	0	0.47744371	0
chr2	13301606	13462537	160932	1	1	0	0.01091641	0	4	0	0.76005302	0
chr2	13462537	13664684	202148	2	1	0	0.01091641	0	4	1	0.76005302	0.30102999
chr2	13664684	13664743	60	1	1	0	0.01091641	0	4	2	0.76005302	0.61140001
chr2	13664743	13838120	173378	1	1	0	0.01091641	0	4	1	0.76005302	0.30102999
chr2	13838120	13910712	72593	2	1	1	0.01091641	0.1218695	4	1	0.76005302	0.1218695
chr2	13910712	14415306	504595	6	1	1	0.00478973	0.1218695	5	1	1.02643191	0.1218695
chr2	14415306	14820798	405493	7	1	1	0.00478973	0.05404976	5	2	1.02643191	0.30102999
chr2	14820798	15169762	348965	2	1	1	0.01091641	0.05404976	4	2	0.76005302	0.30102999
chr2	15169762	15629112	459351	9	1	1	0.00478973	0.05404976	5	2	1.02643191	0.30102999
chr2	15629112	15744556	115445	3	2	1	0.02162467	0.05404976	5	2	0.68214471	0.30102999
chr2	15744556	15823818	79263	3	2	2	0.02162467	0.1575501	5	2	0.68214471	0.1575501
chr2	15823818	16185278	361461	7	2	1	0.02162467	0.05404976	5	2	0.68214471	0.30102999
chr2	16185278	16185337	60	1	2	2	0.02162467	0.1575501	5	2	0.68214471	0.1575501

chr2	16185337	16409634	224298	1	1	2	0.00478973	0.1575501	5	2	1.02643191	0.1575501
chr2	16409634	16409693	60	1	2	2	0.02162467	0.1575501	5	2	0.68214471	0.1575501
chr2	16409693	16632311	222619	1	1	2	0.00478973	0.30102999	5	1	1.02643191	0.05404976
chr2	16632311	16733900	101590	1	1	1	0.00478973	0.30102999	5	0	1.02643191	0
chr2	16733900	16733959	60	1	2	1	0.02162467	0.30102999	5	0	0.68214471	0
chr2	16733959	16769329	35371	1	2	0	0.02162467	0	5	0	0.68214471	0
chr2	16769329	16805169	35841	2	3	0	0.05670724	0	5	0	0.45545077	0
chr2	16805169	16842257	37089	1	2	0	0.02162467	0	5	0	0.68214471	0
chr2	16842257	17005054	162798	1	2	0	0.0429175	0	4	0	0.47744371	0
chr2	17005054	17046079	41026	2	3	1	0.05670724	0.05404976	5	2	0.45545077	0.30102999
chr2	17046079	17046138	60	1	3	1	0.03070643	0.05404976	6	2	0.63695542	0.30102999
chr2	17046138	17113351	67214	1	2	0	0.01053319	0	6	0	0.91219088	0
chr2	17113351	17371225	257875	2	1	0	0.00204627	0	6	0	1.31360226	0
chr2	17371225	17371284	60	1	2	0	0.01053319	0	6	1	0.91219088	0.30102999
chr2	17371284	17447798	76515	1	2	0	0.01053319	0	6	0	0.91219088	0
chr2	17447798	17657171	209374	4	3	0	0.03070643	0	6	0	0.63695542	0
chr2	17657171	17750698	93528	1	2	0	0.01053319	0	6	0	0.91219088	0
chr2	17750698	17837399	86702	2	1	0	0.00478973	0	5	0	1.02643191	0
chr2	17837399	17846623	9225	2	1	0	0.00204627	0	6	0	1.31360226	0
chr2	17846623	18031185	184563	6	1	1	0.00204627	0.1218695	6	1	1.31360226	0.1218695
chr2	18031185	18359851	328667	5	1	0	0.00478973	0	5	1	1.02643191	0.30102999
chr2	18359851	18359910	60	1	1	1	0.00478973	0.05404976	5	2	1.02643191	0.30102999
chr2	18359910	18447369	87460	1	1	0	0.00478973	0	5	2	1.02643191	0.61140001
chr2	18447369	18447428	60	1	2	0	0.02162467	0	5	2	0.68214471	0.61140001
chr2	18447428	18736359	288932	2	1	0	0.00478973	0	5	2	1.02643191	0.61140001
chr2	18736359	18770751	34393	3	2	0	0.02162467	0	5	2	0.68214471	0.61140001
chr2	18770751	18829052	58302	1	2	0	0.0429175	0	4	2	0.47744371	0.61140001
chr2	18829052	18829111	60	1	2	0	0.0429175	0	4	3	0.47744371	0.93173516
chr2	18829111	18951650	122540	1	2	0	0.0429175	0	4	1	0.47744371	0.30102999
chr2	18951650	18951709	60	1	2	0	0.02162467	0	5	1	0.68214471	0.30102999
chr2	18951709	19250159	298451	2	2	0	0.0429175	0	4	0	0.47744371	0
chr2	19250159	19427196	177038	3	2	1	0.0429175	0.30102999	4	0	0.47744371	0
chr2	19427196	19427255	60	1	4	2	0.18734596	0.61140001	4	0	0.18734596	0
chr2	19427255	19553993	126739	1	2	2	0.0429175	0.61140001	4	0	0.47744371	0
chr2	19553993	19679049	125057	2	2	2	0.02162467	0.30102999	5	1	0.68214471	0.05404976
chr2	19679049	19679108	60	1	3	2	0.05670724	0.30102999	5	1	0.45545077	0.05404976
chr2	19679108	19934222	255115	1	2	1	0.02162467	0.30102999	5	0	0.68214471	0
chr2	19934222	20073352	139131	2	2	1	0.01053319	0.30102999	6	0	0.91219088	0
chr2	20073352	20196868	123517	4	3	1	0.03070643	0.30102999	6	0	0.63695542	0
chr2	20196868	20205537	8670	3	3	2	0.03070643	0.61140001	6	0	0.63695542	0
chr2	20205537	20247845	42309	1	2	2	0.01053319	0.61140001	6	0	0.91219088	0
chr2	20247845	20273355	25511	1	1	2	0.00204627	0.61140001	6	0	1.31360226	0
chr2	20273355	20401961	128607	2	1	1	0.00204627	0.30102999	6	0	1.31360226	0
chr2	20401961	20405187	3227	2	1	2	0.00204627	0.61140001	6	0	1.31360226	0
chr2	20405187	20990162	584976	11	1	2	0.00478973	0.61140001	5	0	1.02643191	0
chr2	20990162	21247202	257041	3	0	2	0	0.61140001	5	0	1.60515106	0
chr2	21247202	21634471	387270	5	0	2	0	0.61140001	4	0	1.26272838	0
chr2	21634471	21914332	279862	2	0	2	0	0.61140001	3	0	0.93173516	0
chr2	21914332	22117705	203374	3	0	2	0	0.61140001	4	0	1.26272838	0
chr2	22117705	22117764	60	1	1	3	0.01091641	0.51676182	4	1	0.76005302	0.02438896
chr2	22117764	22363942	246179	2	0	3	0	0.93173516	3	0	0.93173516	0
chr2	22363942	22364001	60	1	0	3	0	0.51676182	3	1	0.93173516	0.02438896
chr2	22364001	22599910	235910	4	0	1	0	0.30102999	3	0	0.93173516	0
chr2	22599910	22599969	60	1	1	3	0.02438896	0.51676182	3	1	0.51676182	0.02438896
chr2	22599969	22756131	156163	1	1	1	0.02438896	0.1218695	3	1	0.51676182	0.1218695
chr2	22756131	22891684	135554	1	1	0	0.02438896	0	3	1	0.51676182	0.30102999
chr2	22891684	23050393	158710	2	1	2	0.02438896	0.30102999	3	1	0.51676182	0.05404976
chr2	23050393	23364285	313893	4	1	2	0.02438896	0.1575501	3	2	0.51676182	0.1575501
chr2	23364285	23565295	201011	2	1	2	0.01091641	0.1575501	4	2	0.76005302	0.1575501
chr2	23565295	23719143	153849	3	2	2	0.02162467	0.1575501	5	2	0.68214471	0.1575501
chr2	23719143	24480925	761783	18	2	1	0.02162467	0.05404976	5	2	0.68214471	0.30102999
chr2	24480925	24572616	91692	2	2	1	0.0429175	0.05404976	4	2	0.47744371	0.30102999
chr2	24572616	24929887	357272	6	2	1	0.02162467	0.05404976	5	2	0.68214471	0.30102999
chr2	24929887	24929946	60	1	2	1	0.01053319	0.05404976	6	2	0.91219088	0.30102999
chr2	24929946	25294614	364669	10	2	1	0.02162467	0.05404976	5	2	0.68214471	0.30102999
chr2	25294614	25456738	162125	3	2	1	0.02162467	0.1218695	5	1	0.68214471	0.1218695
chr2	25456738	25888918	432181	10	2	1	0.02162467	0.30102999	5	0	0.68214471	0
chr2	25888918	25964522	75605	4	4	1	0.11390336	0.30102999	5	0	0.30102999	0
chr2	25964522	25964581	60	1	5	1	0.19510895	0.30102999	5	0	0.19510895	0
chr2	25964581	26029091	64511	1	2	1	0.02162467	0.30102999	5	0	0.68214471	0
chr2	26029091	26068375	39285	2	2	1	0.02162467	0.1218695	5	1	0.68214471	0.1218695
chr2	26068375	26068434	60	1	3	1	0.05670724	0.1218695	5	1	0.45545077	0.1218695
chr2	26068434	26085264	16831	1	3	1	0.05670724	0.30102999	5	0	0.45545077	0
chr2	26085264	26149727	64464	2	4	1	0.11390336	0.30102999	5	0	0.30102999	0
chr2	26149727	26149786	60	1	4	1	0.11390336	0.1218695	5	1	0.30102999	0.1218695
chr2	26149786	26199908	50123	1	2	1	0.02162467	0.1218695	5	1	0.68214471	0.1218695
chr2	26199908	26257541	57634	2	2	2	0.02162467	0.30102999	5	1	0.68214471	0.05404976
chr2	26257541	26257596	56	1	3	2	0.05670724	0.30102999	5	1	0.45545077	0.05404976
chr2	26257596	26321308	63713	2	2	2	0.02162467	0.30102999	5	1	0.68214471	0.05404976
chr2	26321308	26321367	60	1	4	2	0.11390336	0.30102999	5	1	0.30102999	0.05404976
chr2	26321367	26437398	116032	3	3	2	0.05670724	0.30102999	5	1	0.45545077	0.05404976
chr2	26437398	26477168	39771	2	4	2	0.11390336	0.30102999	5	1	0.30102999	0.05404976
chr2	26477168	26533191	56024	1	3	2	0.05670724	0.30102999	5	1	0.45545077	0.05404976
chr2	26533191	26533250	60	1	3	2	0.03070643	0.30102999	6	1	0.63695542	0.05404976
chr2	26533250	26584974	51725	1	2	2	0.01053319	0.30102999	6	1	0.91219088	0.05404976
chr2	26584974	26750699	165726	4	2	2	0.02162467	0.30102999	5	1	0.68214471	0.05404976
chr2	26750699	26750754	56	1	3	2	0.05670724	0.30102999	5	1	0.45545077	0.05404976
chr2	26750754	26823339	72586	2	2	2	0.02162467	0.30102999	5	1	0.68214471	0.05404976
chr2	26823339	26823398	60	1	3	2	0.05670724	0.30102999	5	1	0.45545077	0.05404976
chr2	26823398	26895639	72242	2	2	2	0.02162467	0.30102999	5	1	0.68214471	0.05404976
chr2	26895639	26895698	60	1	3	2	0.05670724	0.30102999	5	1	0.45545077	0.05404976
chr2	26895698	27000109	104412	3	2	2	0.02162467	0.30102999	5	1	0.68214471	0.05404976
chr2	27000109	27000168	60	1	3	2	0.05670724	0.30102999	5	1	0.45545077	0.05404976
chr2	27000168	27088538	88371	5	2	2	0.02162467	0.30102999	5	1	0.68214471	0.05404976
chr2	27088538	27172128	83591	4	2	2	0.01053319	0.30102999	6	1	0.91219088	0.05404976
chr2	27172128	27230992	58865	2	2	2	0.02162467	0.30102999	5	1	0.68214471	0.05404976
chr2	27230992	27231051	60	1	2	2	0.01053319	0.30102999	6	1	0.91219088	0.05404976
chr2	27231051	27278229	47179	2	2	2	0.02162467	0.30102999	5	1	0.68214471	0.05404976
chr2	27278229	27283092	4864	2	2	2	0.01053319	0.30102999	6	1	0.91219088	0.05404976
chr2	27283092	27324127	41036	2	2	2	0.02162467	0.30102999	5	1	0.68214471	0.05404976
chr2	27324127	27324184	58	1	2	2	0.01053319	0.30102999	6	1	0.91219088	0.05404976
chr2	27324184	27353620	29437	2	2	2	0.02162467	0.30102999	5	1	0.68214471	0.05404976
chr2	27353620	27361473	7854	3	2	2	0.01053319	0.30102999	6	1	0.91219088	0.05404976
chr2	27361473	27422953	61481	1	2	2	0.02162467	0.30102999	5	1	0.68214471	0.05404976
chr2	27422953	27442757	19805	3	3	2	0.05670724	0.30102999	5	1	0.45545077	0.05404976
chr2	27442757	27566308	123552	7	3	2	0.03070643	0.30102999	6	1	0.63695542	0.05404976
chr2	27566308	27608773	42466	6	2	2	0.01053319	0.30102999	6	1	0.91219088	0.05404976
chr2	27608773	27656262	47490	2	2	2	0.02162467	0.30102999	5	1	0.68214471	0.05404976
chr2	27656262	27656313	52	1	2	2	0.01053319	0.30102999	6	1	0.91219088	0.05404976
chr2	27656313	27802539	146227	4	2	2	0.02162467	0.30102999	5	1	0.68214471	0.05404976
chr2	27802539	27806272	3734	2	2	2	0.01053319	0.30102999	6	1	0.91219088	0.05404976
chr2	27806272	27869784	63513	3	1	2	0.00478973	0.30102999	5	1	1.02643191	0.05404976
chr2	27869784	27918681	48898	4	2	2	0.02162467	0.30102999	5	1	0.68214471	0.05404976
chr2	27918681	28000550	81870	2	3	2	0.05670724	0.30102999	5	1	0.45545077	0.05404976
chr2	28000550	28039094	38545	1	3	1	0.05670724	0.1218695	5	1	0.45545077	0.1218695
chr2	28039094	28108890	69797	2	1	1	0.00478973	0.1218695	5	1	1.02643191	0.1218695
chr2	28108890	28108949	60	1	2	1	0.02162467	0.1218695	5	1	0.68214471	0.1218695
chr2	28108949	28319485	210537	4	1	1	0.00478973	0.1218695	5	1	1.02643191	0.1218695
chr2	28319485	28386831	67347	1	1	0	0.00478973	0	5	1	1.02643191	0.30102999
chr2	28386831	28421764	34934	2	2	0	0.02162467	0	5	1	0.68214471	0.30102999
chr2	28421764	28522209	100446	2	1	0	0.01091641	0	4	1	0.76005302	0.30102999
chr2	28522209	28522268	60	1	2	0	0.01053319	0	6	1	0.91219088	0.30102999
chr2	28522268	28623124	100857	2	2	0	0.02162467	0	5	1	0.68214471	0.30102999
chr2	28623124	28637284	14161	2	3	0	0.03070643	0	6	1	0.63695542	0.30102999

chr2	28637284	28659295	22012	1	3	0	0.05670724	0	5	1	0.45545077	0.30102999
chr2	28659295	28791353	132059	2	2	0	0.02162467	0	5	1	0.68214471	0.30102999
chr2	28791353	28863297	71945	3	3	0	0.05670724	0	5	1	0.45545077	0.30102999
chr2	28863297	28863356	60	1	4	0	0.11390336	0	5	1	0.30102999	0.30102999
chr2	28863356	28984459	121104	2	3	0	0.10122019	0	4	1	0.30102999	0.30102999
chr2	28984459	29058634	74176	3	4	0	0.18734596	0	4	1	0.18734596	0.30102999
chr2	29058634	29058693	60	1	5	0	0.30102999	0	4	1	0.11390336	0.30102999
chr2	29058693	29146504	87812	3	2	0	0.0429175	0	4	0	0.47744371	0
chr2	29146504	29213106	66603	3	3	0	0.10122019	0	4	0	0.30102999	0
chr2	29213106	29266508	53403	1	2	0	0.0429175	0	4	0	0.47744371	0
chr2	29266508	29266567	60	1	2	0	0.0429175	0	4	1	0.47744371	0.30102999
chr2	29266567	29449922	183356	5	1	0	0.01091641	0	4	1	0.76005302	0.30102999
chr2	29449922	29604618	154697	3	0	0	0	0	4	1	1.26272838	0.30102999
chr2	29604618	29604677	60	1	1	0	0.01091641	0	4	1	0.76005302	0.30102999
chr2	29604677	29851069	246393	6	0	0	0	0	4	1	1.26272838	0.30102999
chr2	29851069	30049401	198333	5	1	0	0.01091641	0	4	1	0.76005302	0.30102999
chr2	30049401	30049460	60	1	2	0	0.0429175	0	4	1	0.47744371	0.30102999
chr2	30049460	30259646	210187	2	1	0	0.01091641	0	4	1	0.76005302	0.30102999
chr2	30259646	30301261	41616	2	2	0	0.0429175	0	4	1	0.47744371	0.30102999
chr2	30301261	30466094	164834	3	1	0	0.01091641	0	4	1	0.76005302	0.30102999
chr2	30466094	30559170	93077	2	1	0	0.02438896	0	3	1	0.51676182	0.30102999
chr2	30559170	30671022	111853	2	1	0	0.01091641	0	4	1	0.76005302	0.30102999
chr2	30671022	30671081	60	1	1	0	0.00478973	0	5	1	1.02643191	0.30102999
chr2	30671081	30790983	119903	2	1	0	0.01091641	0	4	1	0.76005302	0.30102999
chr2	30790983	30832151	41169	4	1	0	0.00478973	0	5	1	1.02643191	0.30102999
chr2	30832151	31048002	215852	5	1	0	0.01091641	0	4	1	0.76005302	0.30102999
chr2	31048002	31091267	43266	1	1	0	0.02438896	0	3	1	0.51676182	0.30102999
chr2	31091267	31190168	98902	2	1	0	0.05404976	0	2	1	0.30102999	0.30102999
chr2	31190168	31249841	59674	2	1	0	0.02438896	0	3	1	0.51676182	0.30102999
chr2	31249841	31348131	98291	3	2	0	0.08289318	0	3	1	0.30102999	0.30102999
chr2	31348131	31348190	60	1	2	0	0.0429175	0	4	1	0.47744371	0.30102999
chr2	31348190	31637483	289294	6	2	0	0.0429175	0	4	0	0.47744371	0
chr2	31637483	31637539	57	1	2	1	0.0429175	0.30102999	4	0	0.47744371	0
chr2	31637539	31870146	232608	4	2	0	0.0429175	0	4	0	0.47744371	0
chr2	31870146	31962996	92851	2	3	0	0.10122019	0	4	0	0.30102999	0
chr2	31962996	31963055	60	1	4	0	0.18734596	0	4	0	0.18734596	0
chr2	31963055	32060696	97642	1	3	0	0.10122019	0	4	0	0.30102999	0
chr2	32060696	32113726	53031	2	4	0	0.18734596	0	4	0	0.18734596	0
chr2	32113726	32113785	60	1	6	0	0.30102999	0	5	0	0.12309572	0
chr2	32113785	32210207	96423	2	5	0	0.19510895	0	5	0	0.19510895	0
chr2	32210207	32257547	47341	2	6	0	0.20064824	0	6	0	0.20064824	0
chr2	32257547	32257606	60	1	8	0	0.4250187	0	6	0	0.08122616	0
chr2	32257606	32357069	99464	2	8	0	0.58747015	0	5	0	0.04407651	0
chr2	32357069	32357128	60	1	9	0	0.76806864	0	5	0	0.02473314	0
chr2	32357128	32418978	61851	2	7	0	0.43181735	0	5	0	0.07511598	0
chr2	32418978	32419036	59	1	8	0	0.58747015	0	5	0	0.04407651	0
chr2	32419036	32490104	71069	2	7	0	0.43181735	0	5	0	0.07511598	0
chr2	32490104	32526494	36391	3	8	0	0.4250187	0	6	0	0.08122616	0
chr2	32526494	32606111	79618	2	7	0	0.43181735	0	5	0	0.07511598	0
chr2	32606111	32606170	60	1	8	0	0.58747015	0	5	0	0.04407651	0
chr2	32606170	32747742	141573	3	7	0	0.43181735	0	5	0	0.07511598	0
chr2	32747742	32747801	60	1	9	0	0.76806864	0	5	0	0.02473314	0
chr2	32747801	32858980	111180	4	8	0	0.58747015	0	5	0	0.04407651	0
chr2	32858980	32958900	99921	3	7	0	0.43181735	0	5	0	0.07511598	0
chr2	32958900	33090028	131129	4	7	0	0.30102999	0	6	0	0.129913	0
chr2	33090028	33121645	31618	1	6	0	0.30102999	0	5	0	0.12309572	0
chr2	33121645	33216675	95031	2	5	0	0.19510895	0	5	0	0.19510895	0
chr2	33216675	33249136	32462	2	5	1	0.12309572	0.30102999	6	0	0.30102999	0
chr2	33249136	33294782	45647	1	3	1	0.03070643	0.30102999	6	0	0.63695542	0
chr2	33294782	33370251	75470	1	3	1	0.05670724	0.30102999	5	0	0.45545077	0
chr2	33370251	33505157	134907	2	2	0	0.02162467	0	5	0	0.68214471	0
chr2	33505157	33562398	57242	1	2	0	0.0429175	0	4	0	0.47744371	0
chr2	33562398	33562457	60	1	2	0	0.02162467	0	5	0	0.68214471	0
chr2	33562457	33590377	27921	1	2	0	0.0429175	0	4	0	0.47744371	0
chr2	33590377	33655068	64692	3	2	0	0.02162467	0	5	0	0.68214471	0
chr2	33655068	33992197	337130	9	3	0	0.05670724	0	5	0	0.45545077	0
chr2	33992197	34090362	98166	3	3	0	0.03070643	0	6	0	0.63695542	0
chr2	34090362	34533415	443054	7	2	0	0.01053319	0	6	0	0.91219088	0
chr2	34533415	34533474	60	1	3	0	0.03070643	0	6	0	0.63695542	0
chr2	34533474	34627053	93580	2	3	0	0.05670724	0	5	0	0.45545077	0
chr2	34627053	34727088	100036	3	3	0	0.03070643	0	6	0	0.63695542	0
chr2	34727088	34773844	46757	1	1	0	0.00204627	0	6	0	1.31360226	0
chr2	34773844	34773903	60	1	1	0	8.47E-04	0	7	0	1.62048027	0
chr2	34773903	34881322	107420	3	1	0	0.00204627	0	6	0	1.31360226	0
chr2	34881322	34881381	60	1	2	0	0.01053319	0	6	0	0.91219088	0
chr2	34881381	34915000	33620	1	1	0	0.00204627	0	6	0	1.31360226	0
chr2	34915000	34956442	41443	2	1	0	8.47E-04	0	7	0	1.62048027	0
chr2	34956442	34993128	36687	1	1	0	0.00204627	0	6	0	1.31360226	0
chr2	34993128	35047843	54716	2	2	0	0.01053319	0	6	0	0.91219088	0
chr2	35047843	35200938	153096	3	1	0	0.00204627	0	6	0	1.31360226	0
chr2	35200938	35359594	158657	4	2	0	0.01053319	0	6	0	0.91219088	0
chr2	35359594	35507967	148374	5	3	0	0.03070643	0	6	0	0.63695542	0
chr2	35507967	35508026	60	1	4	0	0.03812622	0	7	0	0.60763643	0
chr2	35508026	36305915	797890	17	3	0	0.03070643	0	6	0	0.63695542	0
chr2	36305915	36490157	184243	3	1	0	0.00204627	0	6	0	1.31360226	0
chr2	36490157	36623837	133681	4	2	0	0.01053319	0	6	0	0.91219088	0
chr2	36623837	36704029	80193	1	2	0	0.02162467	0	5	0	0.68214471	0
chr2	36704029	36704088	60	1	2	0	0.00493743	0	7	0	1.16581773	0
chr2	36704088	36732225	28138	1	2	0	0.01053319	0	6	0	0.91219088	0
chr2	36732225	36956588	224364	6	2	0	0.00493743	0	7	0	1.16581773	0
chr2	36956588	37255890	299303	9	2	0	9.54E-04	0	9	0	1.74076927	0
chr2	37255890	37315544	59655	2	3	0	0.00378107	0	9	0	1.33111237	0
chr2	37315544	37363800	48257	3	3	1	0.00378107	0.30102999	9	0	1.33111237	0
chr2	37363800	37373828	10029	2	4	1	0.01077081	0.30102999	9	0	1.01542894	0
chr2	37373828	37573211	199384	9	4	1	0.00530919	0.30102999	10	0	1.2568129	0
chr2	37573211	37754609	181399	6	3	1	0.00170589	0.30102999	10	0	1.61091002	0
chr2	37754609	37754667	59	1	5	1	0.01320236	0.30102999	10	0	0.97390707	0
chr2	37754667	37886565	131899	2	4	1	0.00530919	0.30102999	10	0	1.2568129	0
chr2	37886565	37886624	60	1	5	1	0.00666883	0.30102999	11	0	1.20557689	0
chr2	37886624	37936435	49812	3	4	1	0.00247414	0.30102999	11	0	1.52371709	0
chr2	37936435	37936494	60	1	5	1	0.00666883	0.30102999	11	0	1.20557689	0
chr2	37936494	38085399	148906	3	4	1	0.00247414	0.30102999	11	0	1.52371709	0
chr2	38085399	38085458	60	1	5	1	0.00666883	0.30102999	11	0	1.20557689	0
chr2	38085458	38295146	209689	5	3	1	7.28E-04	0.30102999	11	0	1.91540774	0
chr2	38295146	38427784	132639	4	3	1	0.00378107	0.30102999	9	0	1.33111237	0
chr2	38427784	38481308	53525	1	3	1	0.0079614	0.30102999	8	0	1.07548421	0
chr2	38481308	38570591	89284	3	3	1	0.01598258	0.30102999	7	0	0.84395715	0
chr2	38570591	38570650	60	1	3	1	0.0079614	0.30102999	8	0	1.07548421	0
chr2	38570650	38667186	96537	2	3	1	0.01598258	0.30102999	7	0	0.84395715	0
chr2	38667186	38747947	80762	1	3	1	0.03070643	0.30102999	6	0	0.63695542	0
chr2	38747947	38917013	169067	5	3	1	0.01598258	0.30102999	7	0	0.84395715	0
chr2	38917013	38976101	59089	1	3	1	0.05670724	0.30102999	5	0	0.45545077	0
chr2	38976101	38976160	60	1	3	1	0.03070643	0.30102999	6	0	0.63695542	0
chr2	38976160	39024897	48738	2	3	1	0.05670724	0.30102999	5	0	0.45545077	0
chr2	39024897	39024956	60	1	3	1	0.03070643	0.30102999	6	0	0.63695542	0
chr2	39024956	39086869	61914	2	3	1	0.05670724	0.30102999	5	0	0.45545077	0
chr2	39086869	39143793	56925	4	3	1	0.03070643	0.30102999	6	0	0.63695542	0
chr2	39143793	39323484	179692	4	3	1	0.05670724	0.30102999	5	0	0.45545077	0
chr2	39323484	39373159	49676	3	4	1	0.11390336	0.30102999	5	0	0.30102999	0
chr2	39373159	39476602	103444	2	3	1	0.05670724	0.30102999	5	0	0.45545077	0
chr2	39476602	39892499	415898	9	2	1	0.02162467	0.30102999	5	0	0.68214471	0
chr2	39892499	39944722	52224	2	2	1	0.0429175	0.30102999	4	0	0.47744371	0
chr2	39944722	39990166	45445	3	2	1	0.02162467	0.30102999	5	0	0.68214471	0
chr2	39990166	40272159	281994	6	1	1	0.00478973	0.30102999	5	0	1.02643191	0

chr2	40272159	40487057	214899	7	2	1	0.02162467	0.30102999	5	0	0.68214471	0
chr2	40487057	40511988	24932	2	2	2	0.02162467	0.61140001	5	0	0.68214471	0
chr2	40511988	40619683	107696	2	2	1	0.02162467	0.30102999	5	0	0.68214471	0
chr2	40619683	40619742	60	1	2	2	0.02162467	0.61140001	5	0	0.68214471	0
chr2	40619742	40805135	185394	4	2	1	0.02162467	0.30102999	5	0	0.68214471	0
chr2	40805135	41112480	307346	7	2	2	0.02162467	0.61140001	5	0	0.68214471	0
chr2	41112480	41290191	177712	5	2	2	0.01053319	0.61140001	6	0	0.91219088	0
chr2	41290191	41352334	62144	1	2	2	0.02162467	0.61140001	5	0	0.68214471	0
chr2	41352334	41445536	93203	1	1	1	0.00478973	0.30102999	5	0	1.02643191	0
chr2	41445536	41731170	285635	1	1	0	0.00478973	0	5	0	1.02643191	0
chr2	41731170	41731229	60	1	2	0	0.01053319	0	6	0	0.91219088	0
chr2	41731229	42108746	377518	4	1	0	0.00478973	0	5	0	1.02643191	0
chr2	42108746	42738358	629613	14	2	0	0.02162467	0	5	0	0.68214471	0
chr2	42738358	42797410	59053	1	2	0	0.0429175	0	4	0	0.47744371	0
chr2	42797410	42862369	64960	2	2	0	0.02162467	0	5	0	0.68214471	0
chr2	42862369	42995542	133174	4	2	0	0.01053319	0	6	0	0.91219088	0
chr2	42995542	42995597	56	1	2	1	0.01053319	0.30102999	6	0	0.91219088	0
chr2	42995597	43142825	147229	3	2	0	0.02162467	0	5	0	0.68214471	0
chr2	43142825	43230099	87275	2	3	0	0.05670724	0	5	0	0.45545077	0
chr2	43230099	43267660	37562	2	3	0	0.03070643	0	6	0	0.63695542	0
chr2	43267660	43625164	357505	6	3	0	0.05670724	0	5	0	0.45545077	0
chr2	43625164	43793885	168722	5	3	0	0.03070643	0	6	0	0.63695542	0
chr2	43793885	44071113	277229	8	3	1	0.03070643	0.30102999	6	0	0.63695542	0
chr2	44071113	44418154	347042	8	3	0	0.05670724	0	5	0	0.45545077	0
chr2	44418154	44507974	89821	4	3	0	0.03070643	0	6	0	0.63695542	0
chr2	44507974	44625405	117432	3	2	0	0.01053319	0	6	0	0.91219088	0
chr2	44625405	44657296	31892	2	3	0	0.03070643	0	6	0	0.63695542	0
chr2	44657296	44657355	60	1	3	0	0.01598258	0	7	0	0.84395715	0
chr2	44657355	44689098	31744	2	3	0	0.03070643	0	6	0	0.63695542	0
chr2	44689098	44934622	245525	6	3	0	0.01598258	0	7	0	0.84395715	0
chr2	44934622	44993698	59077	1	2	0	0.00493743	0	7	0	1.16581773	0
chr2	44993698	45394752	401055	4	2	0	0.01053319	0	6	0	0.91219088	0
chr2	45394752	45394811	60	1	2	0	0.00221948	0	8	0	1.44210395	0
chr2	45394811	45616537	221727	2	1	0	8.47E-04	0	7	0	1.62048027	0
chr2	45616537	45645486	28950	2	3	0	0.01598258	0	7	0	0.84395715	0
chr2	45645486	45686868	41383	2	4	0	0.03812622	0	7	0	0.60763643	0
chr2	45686868	45822610	135743	3	3	0	0.01598258	0	7	0	0.84395715	0
chr2	45822610	45909120	86511	2	2	0	0.00493743	0	7	0	1.16581773	0
chr2	45909120	45961523	52404	2	3	0	0.01598258	0	7	0	0.84395715	0
chr2	45961523	45961582	60	1	4	0	0.03812622	0	7	0	0.60763643	0
chr2	45961582	46070182	108601	2	3	0	0.01598258	0	7	0	0.84395715	0
chr2	46070182	46156868	86687	2	2	0	0.00493743	0	7	0	1.16581773	0
chr2	46156868	46234572	77705	3	3	0	0.01598258	0	7	0	0.84395715	0
chr2	46234572	46413569	178998	6	4	0	0.03812622	0	7	0	0.60763643	0
chr2	46413569	46549532	135964	2	3	0	0.01598258	0	7	0	0.84395715	0
chr2	46549532	46670180	120649	2	2	0	0.00493743	0	7	0	1.16581773	0
chr2	46670180	46739090	68911	2	3	0	0.01598258	0	7	0	0.84395715	0
chr2	46739090	46847224	108135	7	3	1	0.01598258	0.30102999	7	0	0.84395715	0
chr2	46847224	46852188	4965	2	4	1	0.03812622	0.30102999	7	0	0.60763643	0
chr2	46852188	46959918	107731	3	1	1	8.47E-04	0.30102999	7	0	1.62048027	0
chr2	46959918	46959977	60	1	3	1	0.01598258	0.30102999	7	0	0.84395715	0
chr2	46959977	47129729	169753	2	1	1	8.47E-04	0.30102999	7	0	1.62048027	0
chr2	47129729	47771969	642241	17	2	1	0.00493743	0.30102999	7	0	1.16581773	0
chr2	47771969	47772028	60	1	3	1	0.0079614	0.30102999	8	0	1.07548421	0
chr2	47772028	47938187	166160	3	2	1	0.00221948	0.30102999	8	0	1.44210395	0
chr2	47938187	48012926	74740	1	2	0	0.00221948	0	8	0	1.44210395	0
chr2	48012926	48032122	19197	2	2	0	0.00493743	0	7	0	1.16581773	0
chr2	48032122	48085000	52879	2	2	0	0.00221948	0	8	0	1.44210395	0
chr2	48085000	48116010	31011	2	2	1	0.00221948	0.30102999	8	0	1.44210395	0
chr2	48116010	48155501	39492	1	2	0	0.00493743	0	7	0	1.16581773	0
chr2	48155501	48244418	88918	2	3	0	0.01598258	0	7	0	0.84395715	0
chr2	48244418	48366589	122172	3	3	0	0.0079614	0	8	0	1.07548421	0
chr2	48366589	48592466	225878	4	2	0	0.00221948	0	8	0	1.44210395	0
chr2	48592466	48592525	60	1	3	0	0.0079614	0	8	0	1.07548421	0
chr2	48592525	48737116	144592	5	2	0	0.00221948	0	8	0	1.44210395	0
chr2	48737116	49051274	314159	8	2	0	0.00493743	0	7	0	1.16581773	0
chr2	49051274	49208081	156808	3	2	0	0.01053319	0	6	0	0.91219088	0
chr2	49208081	49402636	194556	5	2	0	0.00221948	0	8	0	1.44210395	0
chr2	49402636	49484686	82051	2	3	0	0.0079614	0	8	0	1.07548421	0
chr2	49484686	49484745	60	1	5	0	0.04407651	0	8	0	0.58747015	0
chr2	49484745	49804254	319510	2	4	0	0.02074938	0	8	0	0.79906872	0
chr2	49804254	49804313	60	1	5	0	0.04407651	0	8	0	0.58747015	0
chr2	49804313	50148852	344540	3	5	0	0.07511598	0	7	0	0.43181735	0
chr2	50148852	50240188	91337	2	4	0	0.03812622	0	7	0	0.60763643	0
chr2	50240188	50240247	60	1	4	1	0.03812622	0.30102999	7	0	0.60763643	0
chr2	50240247	50393957	153711	3	4	0	0.03812622	0	7	0	0.60763643	0
chr2	50393957	51446814	1052858	22	4	1	0.03812622	0.30102999	7	0	0.60763643	0
chr2	51446814	51702007	255194	5	4	1	0.03812622	0.1218695	7	1	0.60763643	0.1218695
chr2	51702007	51739098	37092	1	4	1	0.06713722	0.30102999	6	0	0.44141547	0
chr2	51739098	51846950	107853	2	4	0	0.06713722	0	6	0	0.44141547	0
chr2	51846950	51847009	60	1	4	1	0.06713722	0.30102999	6	0	0.44141547	0
chr2	51847009	52185474	338466	5	4	0	0.06713722	0	6	0	0.44141547	0
chr2	52185474	52434693	249220	3	4	0	0.11390336	0	5	0	0.30102999	0
chr2	52434693	53031053	596361	7	3	0	0.05670724	0	5	0	0.45545077	0
chr2	53031053	53107069	76017	2	3	0	0.03070643	0	6	0	0.63695542	0
chr2	53107069	53747584	640516	6	2	0	0.01053319	0	6	0	0.91219088	0
chr2	53747584	53800287	52704	1	2	0	0.02162467	0	5	0	0.68214471	0
chr2	53800287	53869591	69305	2	3	0	0.05670724	0	5	0	0.45545077	0
chr2	53869591	53869650	60	1	3	0	0.03070643	0	6	1	0.63695542	0.30102999
chr2	53869650	54360764	491115	13	3	0	0.03070643	0	6	0	0.63695542	0
chr2	54360764	54360823	60	1	4	0	0.06713722	0	6	0	0.44141547	0
chr2	54360823	54453210	92388	2	4	0	0.11390336	0	5	0	0.30102999	0
chr2	54453210	54453269	60	1	4	0	0.06713722	0	6	1	0.44141547	0.30102999
chr2	54453269	54531800	78532	1	4	0	0.06713722	0	6	0	0.44141547	0
chr2	54531800	54595080	63281	3	4	0	0.03812622	0	7	0	0.60763643	0
chr2	54595080	54595139	60	1	4	0	0.02074938	0	8	0	0.79906872	0
chr2	54595139	54755314	160176	3	4	0	0.03812622	0	7	0	0.60763643	0
chr2	54755314	54755951	638	2	4	0	0.02074938	0	8	0	0.79906872	0
chr2	54755951	54786608	30658	1	4	0	0.03812622	0	7	0	0.60763643	0
chr2	54786608	54893075	106468	2	3	0	0.01598258	0	7	0	0.84395715	0
chr2	54893075	54954387	61313	2	4	0	0.02074938	0	8	0	0.79906872	0
chr2	54954387	55022901	68515	2	4	0	0.01077081	0	9	0	1.01542894	0
chr2	55022901	55109485	86585	2	3	0	0.00378107	0	9	0	1.33111237	0
chr2	55109485	55233894	124410	3	3	0	0.0079614	0	8	0	1.07548421	0
chr2	55233894	55272924	39031	2	4	0	0.02074938	0	8	0	0.79906872	0
chr2	55272924	55550066	277143	8	5	0	0.04407651	0	8	0	0.58747015	0
chr2	55550066	55585157	35092	2	5	0	0.02473314	0	9	0	0.76806864	0
chr2	55585157	55679812	94656	2	5	0	0.04407651	0	8	0	0.58747015	0
chr2	55679812	55797714	117903	3	5	0	0.07511598	0	7	0	0.43181735	0
chr2	55797714	55830683	32970	2	5	0	0.04407651	0	8	0	0.58747015	0
chr2	55830683	55868847	38165	2	5	0	0.02473314	0	9	0	0.76806864	0
chr2	55868847	55913466	44620	1	5	0	0.07511598	0	7	0	0.43181735	0
chr2	55913466	56097899	184434	4	5	1	0.07511598	0.30102999	7	0	0.43181735	0
chr2	56097899	56188491	90593	3	4	0	0.06713722	0	6	0	0.44141547	0
chr2	56188491	56188550	60	1	5	0	0.12309572	0	6	0	0.30102999	0
chr2	56188550	56265162	76613	3	4	0	0.06713722	0	6	0	0.44141547	0
chr2	56265162	56345448	80287	1	2	0	0.01053319	0	6	0	0.91219088	0
chr2	56345448	56345507	60	1	2	1	0.01053319	0.30102999	6	0	0.91219088	0
chr2	56345507	56440589	95083	2	2	0	0.01053319	0	6	0	0.91219088	0
chr2	56440589	56440648	60	1	4	1	0.06713722	0.30102999	6	0	0.44141547	0
chr2	56440648	56587953	147306	3	3	0	0.03070643	0	6	0	0.63695542	0
chr2	56587953	57436098	848146	5	2	0	0.01053319	0	6	0	0.91219088	0
chr2	57436098	57771681	335584	4	2	1	0.01053319	0.30102999	6	0	0.91219088	0
chr2	57771681	57906726	135046	1	2	0	0.01053319	0	6	0	0.91219088	0

chr2	57906726	58275967	369242	5	2	0	0.02162467	0	5	0	0.68214471	0
chr2	58275967	58468777	192811	6	3	1	0.03070643	0.30102999	6	0	0.63695542	0
chr2	58468777	58566616	97840	1	3	1	0.05670724	0.30102999	5	0	0.45545077	0
chr2	58566616	58669495	102880	2	4	1	0.11390336	0.30102999	5	0	0.30102999	0
chr2	58669495	58669554	60	1	5	1	0.12309572	0.30102999	6	0	0.30102999	0
chr2	58669554	58803133	133580	2	5	1	0.19510895	0.30102999	5	0	0.19510895	0
chr2	58803133	58858910	55778	1	5	0	0.19510895	0	5	0	0.19510895	0
chr2	58858910	58858969	60	1	6	0	0.129913	0	7	0	0.30102999	0
chr2	58858969	58957602	98634	1	5	0	0.07511598	0	7	0	0.43181735	0
chr2	58957602	59178774	221173	2	5	0	0.12309572	0	6	0	0.30102999	0
chr2	59178774	59274583	95810	2	5	0	0.07511598	0	7	0	0.43181735	0
chr2	59274583	59274642	60	1	5	0	0.04407651	0	8	0	0.58747015	0
chr2	59274642	59497510	222869	3	4	0	0.18734596	0	4	0	0.18734596	0
chr2	59497510	59749446	251937	2	3	0	0.17593012	0	3	0	0.17593012	0
chr2	59749446	60013464	264019	4	4	0	0.18734596	0	4	0	0.18734596	0
chr2	60013464	60185767	172304	2	4	0	0.11390336	0	5	0	0.30102999	0
chr2	60185767	60294104	108338	2	4	0	0.06713722	0	6	0	0.44141547	0
chr2	60294104	60294163	60	1	4	1	0.06713722	0.30102999	6	0	0.44141547	0
chr2	60294163	60381835	87673	1	4	1	0.11390336	0.30102999	5	0	0.30102999	0
chr2	60381835	60488034	106200	2	4	1	0.06713722	0.30102999	6	0	0.44141547	0
chr2	60488034	60488093	60	1	5	1	0.07511598	0.30102999	7	0	0.43181735	0
chr2	60488093	60676037	187945	2	4	1	0.06713722	0.30102999	6	0	0.44141547	0
chr2	60676037	60676096	60	1	4	1	0.03812622	0.30102999	7	0	0.60763643	0
chr2	60676096	61149560	473465	12	4	1	0.06713722	0.30102999	6	0	0.44141547	0
chr2	61149560	61618758	469199	13	4	1	0.03812622	0.30102999	7	0	0.60763643	0
chr2	61618758	61684571	65814	1	4	1	0.06713722	0.30102999	6	0	0.44141547	0
chr2	61684571	61742573	58003	2	4	0	0.06713722	0	6	0	0.44141547	0
chr2	61742573	61742632	60	1	5	1	0.12309572	0.30102999	6	0	0.30102999	0
chr2	61742632	61848845	106214	2	4	1	0.06713722	0.30102999	6	0	0.44141547	0
chr2	61848845	61952880	104036	1	3	0	0.03070643	0	6	0	0.63695542	0
chr2	61952880	62405706	452827	10	3	0	0.01598258	0	7	0	0.84395715	0
chr2	62405706	62481591	75886	3	3	0	0.03070643	0	6	0	0.63695542	0
chr2	62481591	62605999	124409	2	3	1	0.03070643	0.30102999	6	0	0.63695542	0
chr2	62605999	62727947	121949	2	3	1	0.01598258	0.30102999	7	0	0.84395715	0
chr2	62727947	62775202	47256	2	3	1	0.03070643	0.30102999	6	0	0.63695542	0
chr2	62775202	63140968	365767	8	3	1	0.01598258	0.30102999	7	0	0.84395715	0
chr2	63140968	63380102	239135	7	3	1	0.00378107	0.30102999	9	0	1.33111237	0
chr2	63380102	63448572	68471	1	3	0	0.0079614	0	8	0	1.07548421	0
chr2	63448572	63448631	60	1	3	0	0.00378107	0	9	0	1.33111237	0
chr2	63448631	63646819	198189	5	3	0	0.0079614	0	8	0	1.07548421	0
chr2	63646819	63646878	60	1	3	0	0.00378107	0	9	0	1.33111237	0
chr2	63646878	63742573	95696	2	3	0	0.0079614	0	8	0	1.07548421	0
chr2	63742573	63816969	74397	2	3	0	0.00378107	0	9	0	1.33111237	0
chr2	63816969	63885258	68290	2	4	0	0.01077081	0	9	0	1.01542894	0
chr2	63885258	63921668	36411	2	5	1	0.02473314	0.30102999	9	0	0.76806864	0
chr2	63921668	64102682	181015	2	5	0	0.04407651	0	8	0	0.58747015	0
chr2	64102682	64141004	38323	3	5	0	0.02473314	0	9	0	0.76806864	0
chr2	64141004	64245660	104657	2	4	0	0.02074938	0	8	0	0.79906872	0
chr2	64245660	64245716	57	1	4	0	0.01077081	0	9	0	1.01542894	0
chr2	64245716	64323624	77909	1	4	0	0.02074938	0	8	0	0.79906872	0
chr2	64323624	64633979	310356	4	4	0	0.03812622	0	7	0	0.60763643	0
chr2	64633979	64683562	49584	2	4	0	0.01077081	0	9	0	1.01542894	0
chr2	64683562	65142743	459182	7	4	0	0.02074938	0	8	0	0.79906872	0
chr2	65142743	65348811	206069	5	2	0	0.00221948	0	8	0	1.44210395	0
chr2	65348811	65348870	60	1	3	0	0.0079614	0	8	0	1.07548421	0
chr2	65348870	65523986	175117	5	3	0	0.01598258	0	7	0	0.84395715	0
chr2	65523986	65688972	164987	4	2	0	0.01053319	0	6	0	0.91219088	0
chr2	65688972	65731798	42827	2	2	0	0.00493743	0	7	0	1.16581773	0
chr2	65731798	66056391	324594	6	2	0	0.01053319	0	6	0	0.91219088	0
chr2	66056391	66171444	115054	1	2	0	0.02162467	0	5	0	0.68214471	0
chr2	66171444	66258676	87233	2	2	0	0.01053319	0	6	0	0.91219088	0
chr2	66258676	66258735	60	1	3	0	0.03070643	0	6	0	0.63695542	0
chr2	66258735	66353450	94716	1	1	0	0.00204627	0	6	0	1.31360226	0
chr2	66353450	66484262	130813	1	1	0	0.00478973	0	5	0	1.02643191	0
chr2	66484262	66484321	60	1	2	0	0.02162467	0	5	0	0.68214471	0
chr2	66484321	66972843	488523	6	1	0	0.00478973	0	5	0	1.02643191	0
chr2	66972843	67473825	500983	4	1	0	0.01091641	0	4	0	0.76005302	0
chr2	67473825	67566920	93096	2	1	0	0.00478973	0	5	0	1.02643191	0
chr2	67566920	67566979	60	1	1	0	0.00204627	0	6	0	1.31360226	0
chr2	67566979	67828842	261864	4	1	0	0.01091641	0	4	0	0.76005302	0
chr2	67828842	68385638	556797	9	1	0	0.00478973	0	5	0	1.02643191	0
chr2	68385638	68412899	27262	1	1	0	0.01091641	0	4	0	0.76005302	0
chr2	68412899	68464760	51862	2	1	0	0.00478973	0	5	0	1.02643191	0
chr2	68464760	68701748	236989	6	1	0	0.00204627	0	6	0	1.31360226	0
chr2	68701748	68701807	60	1	2	0	0.01053319	0	6	0	0.91219088	0
chr2	68701807	68758519	56713	2	1	0	0.00204627	0	6	0	1.31360226	0
chr2	68758519	68813947	55429	2	2	0	0.01053319	0	6	0	0.91219088	0
chr2	68813947	69029193	215247	6	3	0	0.03070643	0	6	0	0.63695542	0
chr2	69029193	69095331	66139	1	3	0	0.05670724	0	5	0	0.45545077	0
chr2	69095331	69342450	247120	6	3	1	0.05670724	0.30102999	5	0	0.45545077	0
chr2	69342450	69399604	57155	1	2	1	0.0429175	0.30102999	4	0	0.47744371	0
chr2	69399604	69447536	47933	1	2	0	0.0429175	0	4	0	0.47744371	0
chr2	69447536	69473533	25998	2	3	0	0.10122019	0	4	0	0.30102999	0
chr2	69473533	69642021	168489	3	2	0	0.0429175	0	4	0	0.47744371	0
chr2	69642021	69642080	60	1	3	0	0.05670724	0	5	0	0.45545077	0
chr2	69642080	69732684	90605	2	2	0	0.02162467	0	5	0	0.68214471	0
chr2	69732684	69901895	169212	5	2	2	0.02162467	0.61140001	5	0	0.68214471	0
chr2	69901895	69998713	96819	2	3	2	0.05670724	0.61140001	5	0	0.45545077	0
chr2	69998713	70053063	54351	3	4	2	0.11390336	0.61140001	5	0	0.30102999	0
chr2	70053063	70053122	60	1	4	2	0.06713722	0.61140001	6	0	0.44141547	0
chr2	70053122	70187221	134100	7	4	1	0.11390336	0.30102999	5	0	0.30102999	0
chr2	70187221	70188253	1033	2	4	2	0.11390336	0.61140001	5	0	0.30102999	0
chr2	70188253	70298339	110087	1	4	1	0.11390336	0.30102999	5	0	0.30102999	0
chr2	70298339	70409868	111530	3	4	1	0.06713722	0.30102999	6	0	0.44141547	0
chr2	70409868	70409927	60	1	4	2	0.06713722	0.61140001	6	0	0.44141547	0
chr2	70409927	70451740	41814	2	3	2	0.03070643	0.61140001	6	0	0.63695542	0
chr2	70451740	70604585	152846	5	3	2	0.05670724	0.61140001	5	0	0.45545077	0
chr2	70604585	70604644	60	1	4	2	0.06713722	0.61140001	6	0	0.44141547	0
chr2	70604644	70704310	99667	2	4	1	0.06713722	0.30102999	6	0	0.44141547	0
chr2	70704310	70984721	280412	6	3	1	0.03070643	0.30102999	6	0	0.63695542	0
chr2	70984721	70984780	60	1	3	2	0.03070643	0.61140001	6	0	0.63695542	0
chr2	70984780	71046534	61755	1	3	1	0.03070643	0.30102999	6	0	0.63695542	0
chr2	71046534	71181720	135187	3	3	0	0.03070643	0	6	0	0.63695542	0
chr2	71181720	71377046	195327	6	3	0	0.05670724	0	5	0	0.45545077	0
chr2	71377046	71427147	50102	1	3	0	0.17593012	0	3	0	0.17593012	0
chr2	71427147	71524888	97742	2	3	0	0.17593012	0	3	1	0.17593012	0.30102999
chr2	71524888	71524947	60	1	3	0	0.10122019	0	4	2	0.30102999	0.61140001
chr2	71524947	71636946	112000	4	3	0	0.17593012	0	3	1	0.17593012	0.30102999
chr2	71636946	71662017	25072	2	3	0	0.17593012	0	3	2	0.17593012	0.61140001
chr2	71662017	71762389	100373	2	2	0	0.08289318	0	3	1	0.30102999	0.30102999
chr2	71762389	71875276	112888	3	3	0	0.10122019	0	4	1	0.30102999	0.30102999
chr2	71875276	71875335	60	1	4	0	0.18734596	0	4	1	0.18734596	0.30102999
chr2	71875335	71970872	95538	3	3	0	0.10122019	0	4	1	0.30102999	0.30102999
chr2	71970872	72069218	98347	2	4	0	0.18734596	0	4	1	0.18734596	0.30102999
chr2	72069218	72159119	89902	1	3	0	0.10122019	0	4	1	0.30102999	0.30102999
chr2	72159119	72442548	283430	3	3	0	0.17593012	0	3	1	0.17593012	0.30102999
chr2	72442548	72498842	56295	2	4	0	0.18734596	0	4	1	0.18734596	0.30102999
chr2	72498842	72544514	45673	1	3	0	0.10122019	0	4	1	0.30102999	0.30102999
chr2	72544514	72620622	76109	2	3	0	0.17593012	0	3	1	0.17593012	0.30102999
chr2	72620622	72740531	119910	2	2	0	0.08289318	0	3	1	0.30102999	0.30102999
chr2	72740531	72821730	81200	3	3	0	0.17593012	0	3	1	0.17593012	0.30102999
chr2	72821730	72962362	140633	4	4	0	0.30102999	0	3	1	0.10122019	0.30102999
chr2	72962362	73007153	44792	1	3	0	0.17593012	0	3	1	0.17593012	0.30102999
chr2	73007153	73118147	110995	2	3	0	0.17593012	0	3	0	0.17593012	0

chr2	73118147	73118206	60	1	3	0	0.17593012	0	3	1	0.17593012	0.30102999
chr2	73118206	73153830	35625	1	3	0	0.17593012	0	3	0	0.17593012	0
chr2	73153830	73153889	60	1	4	0	0.18734596	0	4	0	0.18734596	0
chr2	73153889	73211161	57273	1	3	0	0.10122019	0	4	0	0.30102999	0
chr2	73211161	73265071	53911	2	4	0	0.18734596	0	4	0	0.18734596	0
chr2	73265071	73316307	51237	3	4	0	0.18734596	0	4	1	0.18734596	0.30102999
chr2	73316307	73336374	20068	1	3	0	0.17593012	0	3	1	0.17593012	0.30102999
chr2	73336374	73400870	64497	1	2	0	0.08289318	0	3	1	0.30102999	0.30102999
chr2	73400870	73400929	60	1	2	0	0.0429175	0	4	1	0.47744371	0.30102999
chr2	73400929	73459121	58193	1	2	0	0.08289318	0	3	1	0.30102999	0.30102999
chr2	73459121	73518150	59030	3	4	0	0.30102999	0	3	1	0.10122019	0.30102999
chr2	73518150	73602944	84795	1	3	0	0.17593012	0	3	0	0.17593012	0
chr2	73602944	73642073	39130	2	4	0	0.30102999	0	3	0	0.10122019	0
chr2	73642073	73675234	33162	1	3	0	0.17593012	0	3	0	0.17593012	0
chr2	73675234	73675293	60	1	3	0	0.17593012	0	3	1	0.17593012	0.30102999
chr2	73675293	73718381	43089	1	3	0	0.17593012	0	3	0	0.17593012	0
chr2	73718381	73718440	60	1	5	0	0.45545077	0	3	0	0.05670724	0
chr2	73718440	73879011	160572	4	5	0	0.68214471	0	2	0	0.02162467	0
chr2	73879011	73957150	78140	2	6	0	0.63695542	0	3	0	0.03070643	0
chr2	73957150	73989419	32270	1	5	0	0.45545077	0	3	0	0.05670724	0
chr2	73989419	74089750	100332	3	4	0	0.47744371	0	2	0	0.0429175	0
chr2	74089750	74089809	60	1	5	0	0.68214471	0	2	0	0.02162467	0
chr2	74089809	74182101	92293	3	4	0	0.47744371	0	2	0	0.0429175	0
chr2	74182101	74182160	60	1	5	0	0.68214471	0	2	0	0.02162467	0
chr2	74182160	74320231	138072	2	4	0	0.47744371	0	2	0	0.0429175	0
chr2	74320231	74362495	42265	3	4	1	0.47744371	0.30102999	2	0	0.0429175	0
chr2	74362495	74834374	471880	27	3	1	0.30102999	0.30102999	2	0	0.08289318	0
chr2	74834374	74998815	164442	2	2	1	0.1575501	0.30102999	2	0	0.1575501	0
chr2	74998815	74998874	60	1	3	1	0.30102999	0.30102999	2	0	0.08289318	0
chr2	74998874	75347744	348871	8	2	1	0.1575501	0.30102999	2	0	0.1575501	0
chr2	75347744	75582299	234556	3	2	0	0.1575501	0	2	0	0.1575501	0
chr2	75582299	75648767	66469	2	2	1	0.1575501	0.30102999	2	0	0.1575501	0
chr2	75648767	75729632	80866	1	2	0	0.1575501	0	2	0	0.1575501	0
chr2	75729632	75847471	117840	2	2	0	0.30102999	0	1	0	0.05404976	0
chr2	75847471	76069645	222175	5	2	1	0.30102999	0.30102999	1	0	0.05404976	0
chr2	76069645	76339564	269920	1	2	0	0.30102999	0	1	0	0.05404976	0
chr2	76339564	76510135	170572	2	2	0	0.1575501	0	2	0	0.1575501	0
chr2	76510135	76841649	331515	3	3	0	0.30102999	0	2	0	0.08289318	0
chr2	76841649	76996090	154442	2	2	0	0.1575501	0	2	0	0.1575501	0
chr2	76996090	77708163	712074	16	3	1	0.30102999	0.30102999	2	0	0.08289318	0
chr2	77708163	77788862	80700	5	2	1	0.1575501	0.30102999	2	0	0.1575501	0
chr2	77788862	77788921	60	1	4	1	0.47744371	0.30102999	2	0	0.0429175	0
chr2	77788921	77883284	94364	1	3	1	0.30102999	0.30102999	2	0	0.08289318	0
chr2	77883284	77946599	63316	1	2	1	0.1575501	0.30102999	2	0	0.1575501	0
chr2	77946599	77946658	60	1	2	2	0.1575501	0.61140001	2	0	0.1575501	0
chr2	77946658	78261806	315149	2	2	1	0.1575501	0.30102999	2	0	0.1575501	0
chr2	78261806	78261865	60	1	2	2	0.1575501	0.61140001	2	0	0.1575501	0
chr2	78261865	78495737	233873	3	2	1	0.1575501	0.30102999	2	0	0.1575501	0
chr2	78495737	78680879	185143	2	1	1	0.05404976	0.30102999	2	0	0.30102999	0
chr2	78680879	78680938	60	1	1	2	0.05404976	0.61140001	2	0	0.30102999	0
chr2	78680938	78914816	233879	3	1	1	0.05404976	0.30102999	2	0	0.30102999	0
chr2	78914816	79059451	144636	2	2	1	0.1575501	0.30102999	2	0	0.1575501	0
chr2	79059451	79268615	209165	3	3	1	0.30102999	0.30102999	2	0	0.08289318	0
chr2	79268615	79312949	44335	1	3	1	0.51676182	0.30102999	1	0	0.02438896	0
chr2	79312949	79313008	60	1	3	1	0.30102999	0.30102999	2	0	0.08289318	0
chr2	79313008	79314588	1581	1	3	0	0.30102999	0	2	0	0.08289318	0
chr2	79314588	79363571	48984	1	3	0	0.51676182	0	1	0	0.02438896	0
chr2	79363571	79817793	454223	9	3	1	0.51676182	0.30102999	1	0	0.02438896	0
chr2	79817793	80376767	558975	14	3	1	0.51676182	0.1218695	1	1	0.02438896	0.1218695
chr2	80376767	80540787	164021	6	3	1	0.51676182	0.30102999	1	0	0.02438896	0
chr2	80540787	80662558	121772	2	2	1	0.30102999	0.30102999	1	0	0.05404976	0
chr2	80662558	80752010	89453	3	3	1	0.51676182	0.30102999	1	0	0.02438896	0
chr2	80752010	80811045	59036	1	3	0	0.51676182	0	1	0	0.02438896	0
chr2	80811045	81119400	308356	3	2	0	0.30102999	0	1	0	0.05404976	0
chr2	81119400	81509933	390534	2	0	0	0	0	1	0	0.30102999	0
chr2	81509933	81683592	173660	2	2	0	0.30102999	0	1	0	0.05404976	0
chr2	81683592	81756950	73359	1	2	0	0.61140001	0	0	0	0	0
chr2	81756950	82361952	605003	5	1	0	0.30102999	0	0	0	0	0
chr2	82510808	82790634	279827	4	1	0	0.30102999	0	0	0	0	0
chr2	82790634	83090093	299460	4	1	1	0.30102999	0.30102999	0	0	0	0
chr2	83090093	83452304	362212	2	0	1	0	0.30102999	0	0	0	0
chr2	83613013	83895822	282810	3	0	0	0	0	1	0	0.30102999	0
chr2	83895822	84184014	288193	2	2	0	0.30102999	0	1	0	0.05404976	0
chr2	84184014	84424093	240080	2	3	0	0.51676182	0	1	0	0.02438896	0
chr2	84424093	84609453	185361	2	3	0	0.30102999	0	2	0	0.08289318	0
chr2	84609453	84668205	58753	3	3	0	0.17593012	0	3	0	0.17593012	0
chr2	84668205	85091044	422840	11	3	0	0.30102999	0	2	0	0.08289318	0
chr2	85091044	85420434	329391	7	3	0	0.17593012	0	3	0	0.17593012	0
chr2	85420434	85420493	60	1	4	0	0.18734596	0	4	0	0.18734596	0
chr2	85420493	85474356	53864	1	4	0	0.30102999	0	3	0	0.10122019	0
chr2	85474356	85598458	124103	7	6	0	0.63695542	0	3	0	0.03070643	0
chr2	85598458	85598506	49	1	6	0	0.44141547	0	4	0	0.06713722	0
chr2	85598506	85678499	79994	6	6	0	0.63695542	0	3	0	0.03070643	0
chr2	85678499	85769559	91061	1	4	0	0.30102999	0	3	0	0.10122019	0
chr2	85769559	85787915	18357	3	4	0	0.18734596	0	4	0	0.18734596	0
chr2	85787915	85924807	136893	5	4	0	0.47744371	0	2	0	0.0429175	0
chr2	85924807	86002237	77431	2	3	0	0.30102999	0	2	0	0.08289318	0
chr2	86002237	86002296	60	1	4	0	0.47744371	0	2	0	0.0429175	0
chr2	86002296	86294476	292181	5	2	0	0.1575501	0	2	0	0.1575501	0
chr2	86294476	86419467	124992	6	2	0	0.08289318	0	3	0	0.30102999	0
chr2	86419467	86436834	17368	1	2	0	0.1575501	0	2	0	0.1575501	0
chr2	86436834	86469217	32384	2	3	0	0.30102999	0	2	0	0.08289318	0
chr2	86469217	86789240	320024	6	2	0	0.1575501	0	2	0	0.1575501	0
chr2	86789240	87776206	986967	11	1	0	0.05404976	0	2	0	0.30102999	0
chr2	87776206	87940883	164678	2	2	0	0.1575501	0	2	0	0.1575501	0
chr2	87940883	88234876	293994	1	2	0	0.30102999	0	1	0	0.05404976	0
chr2	88234876	88536245	301370	9	1	0	0.1218695	0	1	0	0.1218695	0
chr2	88536245	88998788	462544	10	2	0	0.1575501	0	2	0	0.1575501	0
chr2	88998788	89203402	204615	5	2	1	0.1575501	0.30102999	2	0	0.1575501	0
chr2	89203402	89508067	304666	4	2	1	0.30102999	0.30102999	1	0	0.05404976	0
chr2	89508067	89606481	98415	1	1	1	0.1218695	0.30102999	1	0	0.1218695	0
chr2	89606481	89606540	60	1	1	1	0.1218695	0.1218695	1	1	0.1218695	0.1218695
chr2	89606540	89914552	308013	1	0	0	0	0	1	1	0.30102999	0.30102999
chr2	89914552	90105896	191345	3	0	0	0	0	0	1	0	0.30102999
chr2	95529039	96074013	544975	12	2	0	0.30102999	0	1	0	0.05404976	0
chr2	96074013	96198927	124915	3	2	0	0.1575501	0	2	0	0.1575501	0
chr2	96198927	96236712	37786	2	2	0	0.1575501	0	2	1	0.1575501	0.30102999
chr2	96236712	96779631	542920	1	1	0	0.05404976	0	2	0	0.30102999	0
chr2	96779631	96780180	550	2	3	0	0.30102999	0	2	3	0.08289318	0.93173516
chr2	96780180	96857538	77359	2	2	0	0.1575501	0	2	3	0.1575501	0.93173516
chr2	96857538	96944738	87201	3	3	0	0.30102999	0	2	4	0.08289318	1.26272838
chr2	96944738	97030312	85575	2	3	0	0.30102999	0	2	3	0.08289318	0.93173516
chr2	97030312	97117792	87481	2	2	0	0.1575501	0	2	2	0.1575501	0.61140001
chr2	97117792	97190200	72409	3	2	1	0.1575501	0.02438896	2	3	0.1575501	0.51676182
chr2	97190200	97190259	60	1	2	1	0.1575501	0.01091641	2	4	0.1575501	0.76005302
chr2	97190259	97400234	209976	6	2	1	0.1575501	0.02438896	2	3	0.1575501	0.51676182
chr2	97400234	97400287	54	1	2	1	0.1575501	0.01091641	2	4	0.1575501	0.76005302
chr2	97400287	97583196	182910	11	1	1	0.05404976	0.01091641	2	4	0.30102999	0.76005302
chr2	97583196	97820513	237318	5	2	1	0.1575501	0.01091641	2	4	0.1575501	0.76005302
chr2	97820513	98021592	201080	2	2	1	0.08289318	0.01091641	3	4	0.30102999	0.76005302
chr2	98021592	98263438	241847	1	1	1	0.02438896	0.02438896	3	3	0.51676182	0.51676182
chr2	98263438	98264438	1001	2	1	1	0.02438896	0.01091641	3	4	0.51676182	0.76005302
chr2	98264438	98337586	73149	2	1	1	0.02438896	0.02438896	3	3	0.51676182	0.51676182
chr2	98337586	98467419	129834	3	1	1	0.02438896	0.05404976	3	2	0.51676182	0.30102999

chr2	98467419	98467478	60	1	1	1	0.02438896	0.02438896	3	3	0.51676182	0.51676182
chr2	98467478	98526012	58535	1	1	1	0.02438896	0.05404976	3	2	0.51676182	0.30102999
chr2	98526012	98526071	60	1	1	1	0.01091641	0.05404976	4	2	0.76005302	0.30102999
chr2	98526071	98562260	36190	1	1	1	0.02438896	0.05404976	3	2	0.51676182	0.30102999
chr2	98562260	98709495	147236	3	1	2	0.02438896	0.1575501	3	2	0.51676182	0.1575501
chr2	98709495	98809387	99893	3	1	2	0.02438896	0.08289318	3	3	0.51676182	0.30102999
chr2	98809387	98809445	59	1	1	2	0.01091641	0.08289318	4	3	0.76005302	0.30102999
chr2	98809445	99069899	260455	7	1	2	0.02438896	0.08289318	3	3	0.51676182	0.30102999
chr2	99069899	99116256	46358	2	1	3	0.02438896	0.17593012	3	3	0.51676182	0.17593012
chr2	99116256	99256623	140368	6	1	3	0.01091641	0.17593012	4	3	0.76005302	0.17593012
chr2	99256623	99342875	86253	2	1	2	0.01091641	0.08289318	4	3	0.76005302	0.30102999
chr2	99342875	99440874	98000	2	1	2	0.02438896	0.08289318	3	3	0.51676182	0.30102999
chr2	99440874	99440933	60	1	1	2	0.00478973	0.0429175	5	4	1.02643191	0.47744371
chr2	99440933	99487756	46824	1	1	2	0.01091641	0.0429175	4	4	0.76005302	0.47744371
chr2	99487756	99554074	66319	2	1	2	0.01091641	0.08289318	4	3	0.76005302	0.30102999
chr2	99554074	99614488	60415	1	1	2	0.02438896	0.08289318	3	3	0.51676182	0.30102999
chr2	99614488	99635037	20550	2	1	2	0.02438896	0.0429175	3	4	0.51676182	0.47744371
chr2	99635037	99743468	108432	2	1	2	0.02438896	0.08289318	3	3	0.51676182	0.30102999
chr2	99743468	99801244	57777	6	1	3	0.02438896	0.17593012	3	3	0.51676182	0.17593012
chr2	99801244	99909815	108572	2	1	2	0.02438896	0.08289318	3	3	0.51676182	0.30102999
chr2	99909815	99954049	44235	3	1	3	0.02438896	0.10122019	3	4	0.51676182	0.30102999
chr2	99954049	100017576	63528	2	1	3	0.02438896	0.17593012	3	3	0.51676182	0.17593012
chr2	100017576	100017635	60	1	1	3	0.02438896	0.10122019	3	4	0.51676182	0.30102999
chr2	100017635	100103999	86365	2	1	3	0.02438896	0.17593012	3	3	0.51676182	0.17593012
chr2	100103999	100104058	60	1	1	3	0.02438896	0.10122019	3	4	0.51676182	0.30102999
chr2	100104058	100182106	78049	1	1	3	0.05404976	0.10122019	2	4	0.30102999	0.30102999
chr2	100182106	100260926	78821	1	1	3	0.05404976	0.30102999	2	2	0.30102999	0.08289318
chr2	100260926	100288221	27296	2	1	3	0.02438896	0.30102999	3	2	0.51676182	0.08289318
chr2	100288221	100625292	337072	8	1	3	0.05404976	0.30102999	2	2	0.30102999	0.08289318
chr2	100625292	100667897	42606	3	1	3	0.01091641	0.17593012	4	3	0.76005302	0.17593012
chr2	100667897	100749773	81877	3	1	3	0.01091641	0.10122019	4	4	0.76005302	0.30102999
chr2	100749773	100848899	99127	2	1	3	0.01091641	0.01598258	4	7	0.76005302	0.84395715
chr2	100848899	100921820	72922	2	1	3	0.05404976	0.01598258	2	7	0.30102999	0.84395715
chr2	100921820	100921879	60	1	1	3	0.02438896	0.01598258	3	7	0.51676182	0.84395715
chr2	100921879	101031590	109712	2	1	3	0.05404976	0.01598258	2	7	0.30102999	0.84395715
chr2	101031590	101190300	158711	2	1	3	0.1218695	0.03070643	1	6	0.1218695	0.63695542
chr2	101190300	101223568	33269	2	1	3	0.05404976	0.03070643	2	6	0.30102999	0.63695542
chr2	101223568	101480160	256593	3	1	3	0.05404976	0.05670724	2	5	0.30102999	0.45545077
chr2	101480160	101611527	131368	3	1	3	0.1218695	0.05670724	1	5	0.1218695	0.45545077
chr2	101611527	101627972	16446	3	1	3	0.05404976	0.05670724	2	5	0.30102999	0.45545077
chr2	101627972	101628026	55	1	1	3	0.05404976	0.03070643	2	6	0.30102999	0.63695542
chr2	101628026	101809623	181598	4	1	3	0.05404976	0.05670724	2	5	0.30102999	0.45545077
chr2	101809623	101883289	73667	1	1	3	0.1218695	0.05670724	1	5	0.1218695	0.45545077
chr2	101883289	101918729	35441	3	1	3	0.1218695	0.03070643	1	6	0.1218695	0.63695542
chr2	101918729	101918788	60	1	1	3	0.05404976	0.03070643	2	6	0.30102999	0.63695542
chr2	101918788	102288554	369767	8	1	3	0.05404976	0.05670724	2	5	0.30102999	0.45545077
chr2	102288554	102490195	201642	6	1	3	0.05404976	0.03070643	2	6	0.30102999	0.63695542
chr2	102490195	102546955	56761	3	1	3	0.1218695	0.03070643	1	6	0.1218695	0.63695542
chr2	102546955	102685545	138591	2	1	3	0.1218695	0.05670724	1	5	0.1218695	0.45545077
chr2	102685545	102818143	132599	3	1	3	0.1218695	0.10122019	1	4	0.1218695	0.30102999
chr2	102818143	102855769	37627	2	1	4	0.1218695	0.18734596	1	4	0.1218695	0.18734596
chr2	102855769	103013191	157423	5	1	2	0.1218695	0.0429175	1	4	0.1218695	0.47744371
chr2	103013191	103057812	44622	2	1	3	0.1218695	0.10122019	1	4	0.1218695	0.30102999
chr2	103057812	103148474	90663	2	1	2	0.1218695	0.0429175	1	4	0.1218695	0.47744371
chr2	103148474	103148533	60	1	1	3	0.1218695	0.10122019	1	4	0.1218695	0.30102999
chr2	103148533	103311486	162954	3	1	1	0.1218695	0.05404976	1	2	0.1218695	0.30102999
chr2	103311486	103311545	60	1	1	2	0.1218695	0.1575501	1	2	0.1218695	0.1575501
chr2	103311545	103432491	120947	3	1	2	0.1218695	0.30102999	1	1	0.1218695	0.05404976
chr2	103432491	103504768	72278	2	1	2	0.1218695	0.1575501	1	2	0.1218695	0.1575501
chr2	103504768	103603707	98940	2	1	3	0.1218695	0.17593012	1	3	0.1218695	0.17593012
chr2	103603707	103603766	60	1	1	3	0.1218695	0.10122019	1	4	0.1218695	0.30102999
chr2	103603766	103879269	275504	1	1	1	0.30102999	0.1218695	0	1	0	0.1218695
chr2	103879269	104099844	220576	1	0	0	0	0	0	1	0	0.30102999
chr2	104099844	104099903	60	1	0	1	0	0.05404976	0	2	0	0.30102999
chr2	104356251	104356310	60	1	0	1	0	0.30102999	0	0	0	0
chr2	104514353	104731573	217221	2	0	0	0	0	0	1	0	0.30102999
chr2	104731573	104731632	60	1	0	1	0	0.01091641	0	4	0	0.76005302
chr2	104731632	104922046	190415	1	0	1	0	0.02438896	0	3	0	0.51676182
chr2	104922046	104922105	60	1	1	1	0.1218695	0.02438896	1	3	0.1218695	0.51676182
chr2	104922105	105450733	528629	6	1	0	0.1218695	0	1	1	0.1218695	0.30102999
chr2	105450733	105653729	202997	3	1	0	0.1218695	0	1	2	0.1218695	0.61140001
chr2	105653729	105756825	103097	3	1	1	0.1218695	0.05404976	1	2	0.1218695	0.30102999
chr2	105756825	105756884	60	1	1	1	0.1218695	0.02438896	1	3	0.1218695	0.51676182
chr2	105756884	106005707	248824	7	1	1	0.1218695	0.05404976	1	2	0.1218695	0.30102999
chr2	106005707	106506684	500978	6	1	1	0.1218695	0.1218695	1	1	0.1218695	0.1218695
chr2	106506684	106710185	203502	4	1	0	0.1218695	0	1	1	0.1218695	0.30102999
chr2	106710185	106797328	87144	3	1	0	0.1218695	0	1	2	0.1218695	0.61140001
chr2	106797328	106817917	20590	2	1	1	0.1218695	0.05404976	1	2	0.1218695	0.30102999
chr2	106817917	106929257	111341	1	1	0	0.1218695	0	1	1	0.1218695	0.30102999
chr2	106929257	106929316	60	1	1	0	0.1218695	0	1	3	0.1218695	0.93173516
chr2	106929316	107429866	500551	4	1	0	0.1218695	0	1	2	0.1218695	0.61140001
chr2	107429866	107496805	66940	3	1	2	0.1218695	0.08289318	1	3	0.1218695	0.30102999
chr2	107496805	107682262	185458	1	1	1	0.1218695	0.02438896	1	3	0.1218695	0.51676182
chr2	107682262	107949894	267633	2	0	0	0	0	1	2	0.30102999	0.61140001
chr2	107949894	107949953	60	1	0	0	0	0	1	3	0.30102999	0.93173516
chr2	107949953	108083897	133945	1	0	0	0	0	1	2	0.30102999	0.61140001
chr2	108083897	108272055	188159	2	1	0	0.1218695	0	1	2	0.1218695	0.61140001
chr2	108272055	108272114	60	1	1	0	0.1218695	0	1	3	0.1218695	0.93173516
chr2	108272114	108403193	131080	1	0	0	0	0	1	3	0.30102999	0.93173516
chr2	108403193	108403252	60	1	0	1	0	0.02438896	1	3	0.30102999	0.51676182
chr2	108403252	108575844	172593	1	0	1	0	0.05404976	1	2	0.30102999	0.30102999
chr2	108575844	108925066	349223	8	1	1	0.05404976	0.05404976	2	2	0.30102999	0.30102999
chr2	108925066	108925125	60	1	1	2	0.05404976	0.1575501	2	2	0.30102999	0.1575501
chr2	108925125	108998296	73172	1	1	1	0.05404976	0.05404976	2	2	0.30102999	0.30102999
chr2	108998296	109258450	260155	6	1	1	0.02438896	0.05404976	3	2	0.51676182	0.30102999
chr2	109258450	109380702	122253	2	1	1	0.02438896	0.1218695	3	1	0.51676182	0.1218695
chr2	109380702	109443529	62828	2	1	0	0.02438896	0	3	1	0.51676182	0.30102999
chr2	109443529	109549543	106015	4	1	1	0.02438896	0.05404976	3	2	0.51676182	0.30102999
chr2	109549543	109602555	53013	1	1	0	0.02438896	0	3	1	0.51676182	0.30102999
chr2	109602555	109707313	104759	1	1	0	0.05404976	0	2	1	0.30102999	0.30102999
chr2	109707313	109755769	48457	2	1	1	0.05404976	0.1218695	2	1	0.30102999	0.1218695
chr2	109755769	109960540	204772	4	1	0	0.05404976	0	2	1	0.30102999	0.30102999
chr2	109960540	109960589	50	1	1	1	0.02438896	0.1218695	3	1	0.51676182	0.1218695
chr2	109960589	110029307	68719	1	1	1	0.1218695	0.1218695	1	1	0.1218695	0.1218695
chr2	110029307	110074098	44792	2	1	2	0.1218695	0.30102999	1	1	0.1218695	0.05404976
chr2	110074098	110074155	58	1	1	2	0.02438896	0.30102999	3	1	0.51676182	0.05404976
chr2	110074155	110260776	186622	1	1	1	0.02438896	0.1218695	3	1	0.51676182	0.1218695
chr2	110260776	110343675	82900	2	1	1	0.05404976	0.1218695	2	1	0.30102999	0.1218695
chr2	110343675	110427195	83521	1	1	1	0.1218695	0.1218695	1	1	0.1218695	0.1218695
chr2	110427195	110427254	60	1	1	2	0.05404976	0.1575501	2	2	0.30102999	0.1575501
chr2	110427254	110841715	414462	1	1	0	0.05404976	0	2	1	0.30102999	0.30102999
chr2	110841715	110901452	59738	3	1	0	0.01091641	0	4	2	0.76005302	0.61140001
chr2	110901452	110970221	68770	1	1	0	0.02438896	0	3	2	0.51676182	0.61140001
chr2	110970221	110980401	10181	2	1	1	0.02438896	0.05404976	3	2	0.51676182	0.30102999
chr2	110980401	111399243	418843	2	1	0	0.02438896	0	3	2	0.51676182	0.61140001
chr2	111399243	111512856	113614	5	1	1	0.02438896	0.05404976	3	2	0.51676182	0.30102999
chr2	111512856	111596965	84110	4	1	1	0.02438896	0.02438896	3	3	0.51676182	0.51676182
chr2	111596965	111679828	82864	2	1	1	0.05404976	0.02438896	2	3	0.30102999	0.51676182
chr2	111679828	111897091	217264	6	1	1	0.01091641	0.02438896	4	3	0.76005302	0.51676182
chr2	111897091	112102800	205710	3	1	1	0.02438896	0.02438896	3	3	0.51676182	0.51676182
chr2	112102800	112102859	60	1	1	1	0.02438896	0.01091641	3	4	0.51676182	0.76005302
chr2	112102859	112278478	175620	1	1	1	0.02438896	0.02438896	3	3	0.51676182	0.51676182
chr2	112278478	112474868	196391	1	1	0	0.02438896	0	3	3	0.51676182	0.93173516
chr2	112474868	112592087	117220	1	1	0	0.05404976	0	2	3	0.30102999	0.93173516

chr2	112592087	112702624	110538	4	1	1	0.05404976	0.01091641	2	4	0.30102999	0.76005302
chr2	112702624	112776973	74350	2	1	1	0.1218695	0.01091641	1	4	0.1218695	0.76005302
chr2	112776973	112818801	41829	2	1	1	0.05404976	0.01091641	2	4	0.30102999	0.76005302
chr2	112818801	113631898	813098	21	1	1	0.1218695	0.01091641	1	4	0.1218695	0.76005302
chr2	113631898	113671257	39360	2	1	1	0.05404976	0.01091641	2	4	0.30102999	0.76005302
chr2	113671257	113671316	60	1	1	1	0.05404976	0.00478973	2	5	0.30102999	1.02643191
chr2	113671316	113742578	71263	1	1	1	0.1218695	0.00478973	1	5	0.1218695	1.02643191
chr2	113742578	113780063	37486	3	1	1	0.1218695	0.00204627	1	6	0.1218695	1.31360226
chr2	113780063	113948613	168551	6	1	1	0.1218695	0.00478973	1	5	0.1218695	1.02643191
chr2	113948613	114007431	58819	2	1	1	0.1218695	0.01091641	1	4	0.1218695	0.76005302
chr2	114007431	114699960	692530	12	1	1	0.1218695	0.00478973	1	5	0.1218695	1.02643191
chr2	114699960	114844660	144701	2	1	0	0.1218695	0	1	5	0.1218695	1.60515106
chr2	114844660	115003555	158896	1	1	0	0.1218695	0	1	4	0.1218695	1.26272838
chr2	115003555	115200111	196557	2	1	0	0.1218695	0	1	3	0.1218695	0.93173516
chr2	115200111	115738271	538161	10	1	0	0.1218695	0	1	5	0.1218695	1.60515106
chr2	115738271	116026016	287746	6	1	0	0.1218695	0	1	6	0.1218695	1.95986592
chr2	116026016	116094610	68595	3	1	0	0.1218695	0	1	5	0.1218695	1.60515106
chr2	116094610	116195266	100657	3	1	0	0.1218695	0	1	6	0.1218695	1.95986592
chr2	116195266	116374560	179295	4	1	0	0.1218695	0	1	5	0.1218695	1.60515106
chr2	116374560	116418952	44393	2	1	0	0.1218695	0	1	6	0.1218695	1.95986592
chr2	116418952	116464046	45095	2	1	1	0.1218695	0.00204627	1	6	0.1218695	1.31360226
chr2	116464046	116735678	271633	4	1	0	0.1218695	0	1	5	0.1218695	1.60515106
chr2	116735678	116735737	60	1	1	1	0.1218695	0.00204627	1	6	0.1218695	1.31360226
chr2	116735737	116895746	160010	1	1	0	0.1218695	0	1	5	0.1218695	1.60515106
chr2	116895746	117074480	178735	1	0	0	0	0	1	5	0.30102999	1.60515106
chr2	117074480	117074539	60	1	0	0	0	0	2	5	0.61140001	1.60515106
chr2	117074539	117353027	278489	1	0	0	0	0	1	3	0.30102999	0.93173516
chr2	117353027	117679959	326933	2	0	0	0	0	0	2	0	0.61140001
chr2	117679959	117680018	60	1	1	0	0.05404976	0	2	3	0.30102999	0.93173516
chr2	117680018	117830337	150320	1	0	0	0	0	2	3	0.61140001	0.93173516
chr2	117830337	117959656	129320	1	0	0	0	0	2	2	0.61140001	0.61140001
chr2	117959656	118485046	525391	5	1	0	0.05404976	0	2	2	0.30102999	0.61140001
chr2	118485046	118647245	162200	4	1	0	0.05404976	0	2	3	0.30102999	0.93173516
chr2	118647245	118735793	88549	3	1	1	0.05404976	0.01091641	2	4	0.30102999	0.76005302
chr2	118735793	119071856	336064	5	1	0	0.05404976	0	2	3	0.30102999	0.93173516
chr2	119071856	119071915	60	1	1	0	0.05404976	0	2	4	0.30102999	1.26272838
chr2	119071915	119336704	264790	1	1	0	0.05404976	0	2	3	0.30102999	0.93173516
chr2	119336704	119541107	204404	2	1	1	0.05404976	0.02438896	2	3	0.30102999	0.51676182
chr2	119541107	119541166	60	1	1	1	0.05404976	0.01091641	2	4	0.30102999	0.76005302
chr2	119541166	119833027	291862	5	1	1	0.1218695	0.02438896	1	3	0.1218695	0.51676182
chr2	119833027	119913861	80835	2	1	1	0.05404976	0.02438896	2	3	0.30102999	0.51676182
chr2	119913861	119951896	38036	1	1	1	0.1218695	0.02438896	1	3	0.1218695	0.51676182
chr2	119951896	119990907	39012	2	1	1	0.05404976	0.01091641	2	4	0.30102999	0.76005302
chr2	119990907	120059513	68607	2	1	1	0.05404976	0.02438896	2	3	0.30102999	0.51676182
chr2	120059513	120194944	135432	4	1	1	0.05404976	0.01091641	2	4	0.30102999	0.76005302
chr2	120194944	120361587	166644	4	1	1	0.1218695	0.01091641	1	4	0.1218695	0.76005302
chr2	120361587	120480623	119037	4	1	1	0.1218695	0.02438896	1	3	0.1218695	0.51676182
chr2	120480623	120520224	39602	1	1	1	0.1218695	0.05404976	1	2	0.1218695	0.30102999
chr2	120520224	120567392	47169	2	1	1	0.1218695	0.02438896	1	3	0.1218695	0.51676182
chr2	120567392	120628484	61093	2	1	1	0.1218695	0.01091641	1	4	0.1218695	0.76005302
chr2	120628484	120900646	272163	7	1	1	0.1218695	0.00478973	1	5	0.1218695	1.02643191
chr2	120900646	121012182	111537	2	1	1	0.1218695	0.01091641	1	4	0.1218695	0.76005302
chr2	121012182	121043478	31297	2	1	1	0.1218695	0.00478973	1	5	0.1218695	1.02643191
chr2	121043478	121050848	7371	2	1	1	0.1218695	0.00204627	1	6	0.1218695	1.31360226
chr2	121050848	121106344	55497	1	1	1	0.1218695	0.00478973	1	5	0.1218695	1.02643191
chr2	121106344	121394787	288444	2	1	1	0.1218695	0.01091641	1	4	0.1218695	0.76005302
chr2	121394787	121486947	92161	2	1	1	0.1218695	0.00478973	1	5	0.1218695	1.02643191
chr2	121486947	121487006	60	1	1	1	0.1218695	0.00204627	1	6	0.1218695	1.31360226
chr2	121487006	121684726	197721	5	1	1	0.1218695	0.00478973	1	5	0.1218695	1.02643191
chr2	121684726	121684785	60	1	1	1	0.1218695	8.47E-04	1	7	0.1218695	1.62048027
chr2	121684785	121786284	101500	2	1	0	0.1218695	0	1	7	0.1218695	2.3278427
chr2	121786284	121786343	60	1	1	1	0.1218695	8.47E-04	1	7	0.1218695	1.62048027
chr2	121786343	122003852	217510	3	0	0	0	0	1	6	0.30102999	1.95986592
chr2	122003852	122095336	91485	1	0	0	0	0	1	5	0.30102999	1.60515106
chr2	122095336	122363764	268429	6	0	0	0	0	1	6	0.30102999	1.95986592
chr2	122363764	122484636	120873	3	0	0	0	0	1	5	0.30102999	1.60515106
chr2	122484636	122552993	68358	3	0	0	0	0	1	4	0.30102999	1.26272838
chr2	122552993	122662279	109287	1	0	0	0	0	1	3	0.30102999	0.93173516
chr2	122662279	122662338	60	1	0	1	0	0.02438896	1	3	0.30102999	0.51676182
chr2	122662338	123086730	424393	3	0	0	0	0	1	2	0.30102999	0.61140001
chr2	123086730	123086789	60	1	0	1	0	0.05404976	1	2	0.30102999	0.30102999
chr2	123086789	123161667	74879	1	0	1	0	0.1218695	0	1	0	0.1218695
chr2	123161667	123396233	234567	1	0	0	0	0	0	1	0	0.30102999
chr2	123823807	123973518	149712	2	0	0	0	0	0	1	0	0.30102999
chr2	123973518	124398034	424517	3	0	0	0	0	0	2	0	0.61140001
chr2	124398034	124398093	60	1	1	0	0.1218695	0	1	4	0.1218695	1.26272838
chr2	124398093	124574590	176498	1	1	0	0.1218695	0	1	3	0.1218695	0.93173516
chr2	124574590	124770883	196294	2	1	0	0.1218695	0	1	5	0.1218695	1.60515106
chr2	124770883	124816328	45446	2	1	1	0.1218695	0.00204627	1	6	0.1218695	1.31360226
chr2	124816328	124816387	60	1	1	2	0.1218695	0.00493743	1	7	0.1218695	1.16581773
chr2	124816387	125261841	445455	8	1	1	0.1218695	0.00204627	1	6	0.1218695	1.31360226
chr2	125261841	125611168	349328	9	1	2	0.1218695	0.01053319	1	6	0.1218695	0.91219088
chr2	125611168	125672677	61510	2	1	2	0.1218695	0.00493743	1	7	0.1218695	1.16581773
chr2	125672677	125805899	133223	2	1	1	0.1218695	8.47E-04	1	7	0.1218695	1.62048027
chr2	125805899	126218634	412736	2	1	1	0.1218695	0.00204627	1	6	0.1218695	1.31360226
chr2	126218634	126218693	60	1	1	2	0.1218695	0.01053319	1	6	0.1218695	0.91219088
chr2	126218693	126482320	263628	1	0	0	0	0	1	5	0.30102999	1.60515106
chr2	126482320	126916189	433870	2	0	0	0	0	0	2	0	0.61140001
chr2	126916189	127083044	166856	2	0	1	0	8.47E-04	0	7	0	1.62048027
chr2	127083044	127184889	101846	2	0	1	0	3.39E-04	0	8	0	1.94674965
chr2	127184889	127184948	60	1	1	1	0.30102999	3.39E-04	0	8	0	1.94674965
chr2	127184948	127433973	249026	2	0	1	0	3.39E-04	0	8	0	1.94674965
chr2	127433973	127449298	15326	2	1	1	0.30102999	3.39E-04	0	8	0	1.94674965
chr2	127449298	127524507	75210	1	1	1	0.30102999	8.47E-04	0	7	0	1.62048027
chr2	127524507	127805976	281470	2	1	0	0.30102999	0	0	7	0	2.3278427
chr2	127805976	127914480	108505	3	1	0	0.30102999	0	0	8	0	2.71017764
chr2	127914480	127914539	60	1	1	1	0.1218695	3.39E-04	1	8	0.1218695	1.94674965
chr2	127914539	128042157	127619	3	1	0	0.1218695	0	1	8	0.1218695	2.71017764
chr2	128042157	128185319	143163	6	1	1	0.1218695	3.39E-04	1	8	0.1218695	1.94674965
chr2	128185319	128238602	53284	2	1	1	0.1218695	1.30E-04	1	9	0.1218695	2.29264207
chr2	128238602	128284305	45704	1	1	1	0.1218695	3.39E-04	1	8	0.1218695	1.94674965
chr2	128284305	128405943	121639	3	1	0	0.1218695	0	1	7	0.1218695	2.3278427
chr2	128405943	128428229	22287	2	1	0	0.1218695	0	1	6	0.1218695	1.95986592
chr2	128428229	128452004	23776	2	1	0	0.1218695	0	1	7	0.1218695	2.3278427
chr2	128452004	128462866	10863	3	1	1	0.1218695	8.47E-04	1	7	0.1218695	1.62048027
chr2	128462866	128641717	178852	3	1	1	0.30102999	0.00204627	0	6	0	1.31360226
chr2	128641717	128641776	60	1	1	1	0.1218695	8.47E-04	1	7	0.1218695	1.62048027
chr2	128641776	128772484	130709	3	1	1	0.1218695	0.00478973	1	5	0.1218695	1.02643191
chr2	128772484	128934513	162030	4	1	1	0.30102999	0.00478973	0	5	0	1.02643191
chr2	128934513	128934572	60	1	1	1	0.1218695	0.00478973	1	5	0.1218695	1.02643191
chr2	128934572	129168767	234196	3	1	0	0.30102999	0	0	4	0	1.26272838
chr2	129168767	129168826	60	1	1	0	0.1218695	0	1	4	0.1218695	1.26272838
chr2	129168826	129333431	164606	2	1	0	0.30102999	0	0	3	0	0.93173516
chr2	129333431	129333490	60	1	1	0	0.1218695	0	1	3	0.1218695	0.93173516
chr2	129333490	129635169	301680	2	1	0	0.30102999	0	0	3	0	0.93173516
chr2	129635169	129970478	335310	2	1	0	0.30102999	0	0	2	0	0.61140001
chr2	129970478	129970537	60	1	1	0	0.30102999	0	0	3	0	0.93173516
chr2	129970537	130036197	65661	1	0	0	0	0	0	3	0	0.93173516
chr2	130036197	130113483	77287	2	0	0	0	0	0	4	0	1.26272838
chr2	130113483	130185237	71755	2	1	0	0.30102999	0	0	4	0	1.26272838
chr2	130185237	130378446	193210	1	1	0	0.30102999	0	0	3	0	0.93173516
chr2	130378446	130617148	238703	2	0	0	0	0	0	2	0	0.61140001
chr2	130617148	130742410	125263	2	1	0	0.30102999	0	0	3	0	0.93173516
chr2	130742410	130786789	44380	1	1	0	0.30102999	0	0	2	0	0.61140001

chr2	130786789	130964454	177666	2	1	0	0.30102999	0	0	3	0	0.93173516
chr2	130964454	130964513	60	1	1	0	0.30102999	0	0	4	0	1.26272838
chr2	130964513	131095306	130794	3	1	0	0.30102999	0	0	3	0	0.93173516
chr2	131095306	131317614	222309	5	1	0	0.30102999	0	0	2	0	0.61140001
chr2	131317614	131317673	60	1	1	0	0.05404976	0	2	2	0.30102999	0.61140001
chr2	131317673	131758279	440607	6	1	0	0.1218695	0	1	2	0.1218695	0.61140001
chr2	131758279	131905082	146804	6	1	0	0.05404976	0	2	2	0.30102999	0.61140001
chr2	131905082	132098109	193028	1	1	0	0.1218695	0	1	1	0.1218695	0.30102999
chr2	132098109	132190346	92238	2	1	0	0.1218695	0	1	0	0.1218695	0
chr2	132190346	132493872	303527	3	1	0	0.1218695	0	1	1	0.1218695	0.30102999
chr2	132493872	132493931	60	1	1	1	0.05404976	0.02438896	2	3	0.30102999	0.51676182
chr2	132493931	133036817	542887	2	0	1	0	0.05404976	2	2	0.61140001	0.30102999
chr2	133036817	133147448	110632	1	0	1	0	0.1218695	1	1	0.30102999	0.1218695
chr2	133147448	133217677	70230	3	0	1	0	0.02438896	1	3	0.30102999	0.51676182
chr2	133217677	133253506	35830	2	0	1	0	0.01091641	1	4	0.30102999	0.76005302
chr2	133253506	133306434	52929	1	0	1	0	0.02438896	1	3	0.30102999	0.51676182
chr2	133306434	133347724	41291	2	0	1	0	0.02438896	2	3	0.61140001	0.51676182
chr2	133347724	133402552	54829	3	0	1	0	0.00478973	2	5	0.61140001	1.02643191
chr2	133402552	133458454	55903	4	0	1	0	0.00204627	2	6	0.61140001	1.31360226
chr2	133458454	134055460	597007	11	0	1	0	0.00478973	2	5	0.61140001	1.02643191
chr2	134055460	134055519	60	1	0	1	0	0.00204627	2	6	0.61140001	1.31360226
chr2	134055519	134095816	40298	1	0	1	0	0.00478973	2	5	0.61140001	1.02643191
chr2	134095816	134208785	112970	2	0	1	0	0.02438896	2	3	0.61140001	0.51676182
chr2	134208785	134344602	135818	3	0	1	0	0.05404976	2	2	0.61140001	0.30102999
chr2	134344602	134400111	55510	1	0	1	0	0.1218695	2	1	0.61140001	0.1218695
chr2	134400111	134400170	60	1	0	1	0	0.05404976	2	2	0.61140001	0.30102999
chr2	134400170	134665458	265289	1	0	1	0	0.1218695	1	1	0.30102999	0.1218695
chr2	134665458	134665517	60	1	1	1	0.1218695	0.1218695	1	1	0.1218695	0.1218695
chr2	134665517	134953062	287546	1	1	0	0.1218695	0	1	0	0.1218695	0
chr2	134953062	134953121	60	1	1	0	0.1218695	0	1	1	0.1218695	0.30102999
chr2	134953121	135011015	57895	1	1	0	0.30102999	0	0	1	0	0.30102999
chr2	135011015	135062506	51492	2	1	1	0.30102999	0.02438896	0	3	0	0.51676182
chr2	135062506	135062565	60	1	1	1	0.1218695	0.02438896	1	3	0.1218695	0.51676182
chr2	135062565	135275483	212919	5	1	0	0.1218695	0	1	3	0.1218695	0.93173516
chr2	135275483	135301967	26485	2	1	0	0.1218695	0	1	4	0.1218695	1.26272838
chr2	135301967	135362917	60951	1	1	0	0.1218695	0	1	3	0.1218695	0.93173516
chr2	135362917	135406655	43739	2	1	0	0.1218695	0	1	4	0.1218695	1.26272838
chr2	135406655	135596235	189581	3	1	0	0.1218695	0	1	3	0.1218695	0.93173516
chr2	135596235	135619518	23284	1	1	0	0.1218695	0	1	2	0.1218695	0.61140001
chr2	135619518	135619565	48	1	1	0	0.1218695	0	1	6	0.1218695	1.95986592
chr2	135619565	135737566	118002	2	1	0	0.1218695	0	1	4	0.1218695	1.26272838
chr2	135737566	135805998	68433	4	1	0	0.05404976	0	2	5	0.30102999	1.60515106
chr2	135805998	135910526	104529	3	1	0	0.05404976	0	2	6	0.30102999	1.95986592
chr2	135910526	135941282	30757	2	1	2	0.05404976	0.01053319	2	6	0.30102999	0.91219088
chr2	135941282	136033293	92012	3	1	1	0.05404976	0.00204627	2	6	0.30102999	1.31360226
chr2	136033293	136071347	38055	1	1	0	0.05404976	0	2	5	0.30102999	1.60515106
chr2	136071347	136197033	125687	3	1	0	0.05404976	0	2	4	0.30102999	1.26272838
chr2	136197033	136700985	503953	16	1	0	0.05404976	0	2	5	0.30102999	1.60515106
chr2	136700985	136803071	102087	4	1	0	0.02438896	0	3	5	0.51676182	1.60515106
chr2	136803071	136803130	60	1	1	2	0.02438896	0.02162467	3	5	0.51676182	0.68214471
chr2	136803130	136871993	68864	1	1	2	0.05404976	0.02162467	2	5	0.30102999	0.68214471
chr2	136871993	136888233	16241	2	1	3	0.05404976	0.05670724	2	5	0.30102999	0.45545077
chr2	136888233	137082556	194324	1	1	2	0.1218695	0.02162467	1	5	0.1218695	0.68214471
chr2	137082556	137381168	298613	4	1	3	0.1218695	0.05670724	1	5	0.1218695	0.45545077
chr2	137381168	137419643	38476	2	1	3	0.05404976	0.05670724	2	5	0.30102999	0.45545077
chr2	137419643	137668681	249039	3	1	2	0.05404976	0.02162467	2	5	0.30102999	0.68214471
chr2	137668681	137785796	117116	2	1	2	0.1218695	0.02162467	1	5	0.1218695	0.68214471
chr2	137785796	137785855	60	1	1	2	0.05404976	0.02162467	2	5	0.30102999	0.68214471
chr2	137785855	138227914	442060	10	1	2	0.1218695	0.02162467	1	5	0.1218695	0.68214471
chr2	138227914	138485012	257099	4	0	2	0	0.0429175	1	4	0.30102999	0.47744371
chr2	138485012	138581222	96211	2	0	2	0	0.02162467	1	5	0.30102999	0.68214471
chr2	138581222	138581281	60	1	0	2	0	0.02162467	2	5	0.61140001	0.68214471
chr2	138581281	138771527	190247	3	0	2	0	0.0429175	1	4	0.30102999	0.47744371
chr2	138771527	139261374	489848	4	0	2	0	0.08289318	1	3	0.30102999	0.30102999
chr2	139261374	139295544	34171	2	0	2	0	0.0429175	1	4	0.30102999	0.47744371
chr2	139295544	139543947	248404	4	0	1	0	0.02438896	1	3	0.30102999	0.51676182
chr2	139543947	139632425	88479	1	0	0	0	0	1	1	0.30102999	0.30102999
chr2	139632425	140151339	518915	2	0	0	0	0	0	1	0	0.30102999
chr2	140151339	140151398	60	1	0	2	0	0.1575501	1	2	0.30102999	0.1575501
chr2	140151398	140625834	474437	4	0	1	0	0.1218695	1	1	0.30102999	0.1218695
chr2	140625834	140930169	304336	3	0	2	0	0.08289318	1	3	0.30102999	0.30102999
chr2	140930169	141015240	85072	2	0	2	0	0.02162467	1	5	0.30102999	0.68214471
chr2	141015240	141094413	79174	2	0	2	0	0.0429175	1	4	0.30102999	0.47744371
chr2	141094413	141169679	75267	2	0	2	0	0.02162467	1	5	0.30102999	0.68214471
chr2	141169679	141232845	63167	1	0	2	0	0.0429175	1	4	0.30102999	0.47744371
chr2	141232845	141333794	100950	2	0	2	0	0.08289318	1	3	0.30102999	0.30102999
chr2	141333794	141445879	112086	3	0	2	0	0.0429175	2	4	0.61140001	0.47744371
chr2	141445879	141533612	87734	2	0	1	0	0.01091641	2	4	0.61140001	0.76005302
chr2	141533612	141533671	60	1	0	2	0	0.02162467	2	5	0.61140001	0.68214471
chr2	141533671	141625347	91677	2	0	2	0	0.0429175	2	4	0.61140001	0.47744371
chr2	141625347	141660774	35428	2	0	2	0	0.0429175	3	4	0.93173516	0.47744371
chr2	141660774	141660833	60	1	0	2	0	0.00493743	3	7	0.93173516	1.16581773
chr2	141660833	141769326	108494	2	0	2	0	0.01053319	3	6	0.93173516	0.91219088
chr2	141769326	142167696	398371	9	0	2	0	0.00493743	3	7	0.93173516	1.16581773
chr2	142167696	142287243	119548	2	0	2	0	0.01053319	3	6	0.93173516	0.91219088
chr2	142287243	142319133	31891	2	1	2	0.02438896	0.01053319	3	6	0.51676182	0.91219088
chr2	142319133	142480686	161554	3	1	1	0.02438896	0.01091641	3	4	0.51676182	0.76005302
chr2	142480686	142567937	87252	2	0	1	0	0.01091641	3	4	0.93173516	0.76005302
chr2	142567937	142645376	77440	3	1	1	0.02438896	0.01091641	3	4	0.51676182	0.76005302
chr2	142645376	142805609	160234	4	0	1	0	0.01091641	3	4	0.93173516	0.76005302
chr2	142805609	142899918	94310	2	0	1	0	0.05404976	3	2	0.93173516	0.30102999
chr2	142899918	142899977	60	1	0	1	0	0.02438896	3	3	0.93173516	0.51676182
chr2	142899977	142995636	95660	1	0	1	0	0.05404976	2	2	0.61140001	0.30102999
chr2	142995636	143174405	178770	2	1	1	0.05404976	0.05404976	2	2	0.30102999	0.30102999
chr2	143174405	143174464	60	1	2	1	0.1575501	0.05404976	2	2	0.1575501	0.30102999
chr2	143174464	143475770	301307	1	1	1	0.1218695	0.05404976	1	2	0.1218695	0.30102999
chr2	143475770	143475829	60	1	1	2	0.05404976	0.1575501	2	2	0.30102999	0.1575501
chr2	143475829	143685258	209430	2	1	2	0.1218695	0.1575501	1	2	0.1218695	0.1575501
chr2	143685258	143747062	61805	3	2	2	0.30102999	0.1575501	1	2	0.05404976	0.1575501
chr2	143747062	143820269	73208	2	1	2	0.1218695	0.1575501	1	2	0.1218695	0.1575501
chr2	143820269	143820328	60	1	2	2	0.30102999	0.1575501	1	2	0.05404976	0.1575501
chr2	143820328	143931445	111118	2	2	2	0.30102999	0.30102999	1	1	0.05404976	0.05404976
chr2	143931445	144051648	120204	3	2	2	0.30102999	0.1575501	1	2	0.05404976	0.1575501
chr2	144051648	144122740	71093	2	2	2	0.1575501	0.1575501	2	2	0.1575501	0.1575501
chr2	144122740	144220283	97544	5	3	2	0.30102999	0.08289318	2	3	0.08289318	0.30102999
chr2	144220283	144533339	313057	7	3	1	0.30102999	0.02438896	2	3	0.08289318	0.51676182
chr2	144533339	144533398	60	1	3	1	0.30102999	0.01091641	2	4	0.08289318	0.76005302
chr2	144533398	144714856	181459	2	3	1	0.30102999	0.05404976	2	2	0.08289318	0.30102999
chr2	144714856	144714911	56	1	3	1	0.30102999	0.02438896	2	3	0.08289318	0.51676182
chr2	144714911	144754766	39856	1	3	1	0.51676182	0.02438896	1	3	0.02438896	0.51676182
chr2	144754766	144909193	154428	5	3	1	0.51676182	0.01091641	1	4	0.02438896	0.76005302
chr2	144909193	144909252	60	1	3	1	0.30102999	0.01091641	2	4	0.08289318	0.76005302
chr2	144909252	144932907	23656	1	3	1	0.30102999	0.02438896	2	3	0.08289318	0.51676182
chr2	144932907	145075561	142655	4	3	1	0.51676182	0.02438896	1	3	0.02438896	0.51676182
chr2	145075561	145182456	106896	3	3	1	0.51676182	0.01091641	1	4	0.02438896	0.76005302
chr2	145182456	145356459	174004	4	2	1	0.30102999	0.01091641	1	4	0.05404976	0.76005302
chr2	145356459	145515183	158725	1	2	1	0.30102999	0.02438896	1	3	0.05404976	0.51676182
chr2	145515183	145719103	203921	4	1	1	0.1218695	0.02438896	1	3	0.1218695	0.51676182
chr2	145719103	145827808	108706	1	1	0	0.1218695	0	1	3	0.1218695	0.93173516
chr2	145827808	146048545	220738	2	1	0	0.30102999	0	0	2	0	0.61140001
chr2	146048545	146205401	156857	1	0	0	0	0	0	2	0	0.61140001
chr2	146205401	147735637	1530237	9	0	0	0	0	0	1	0	0.30102999
chr2	147735637	147934943	199307	2	1	0	0.30102999	0	0	2	0	0.61140001
chr2	147934943	147935002	60	1	2	0	0.61140001	0	0	2	0	0.61140001

chr2	147935002	148186210	251209	2	0	0	0	0	0	2	0	0.61140001
chr2	148186210	148372337	186128	3	2	0	0.30102999	0	1	2	0.05404976	0.61140001
chr2	148372337	148602463	230127	3	2	0	0.30102999	0	1	4	0.05404976	1.26272838
chr2	148602463	148657041	54579	2	2	0	0.30102999	0	1	3	0.05404976	0.93173516
chr2	148657041	148879681	222641	6	2	0	0.30102999	0	1	4	0.05404976	1.26272838
chr2	148879681	149002435	122755	2	2	0	0.30102999	0	1	3	0.05404976	0.93173516
chr2	149002435	149002494	60	1	2	0	0.30102999	0	1	5	0.05404976	1.60515106
chr2	149002494	149228395	225902	6	2	0	0.30102999	0	1	4	0.05404976	1.26272838
chr2	149228395	149263045	34651	1	1	0	0.1218695	0	1	4	0.1218695	1.26272838
chr2	149263045	149263104	60	1	1	0	0.1218695	0	1	5	0.1218695	1.60515106
chr2	149263104	149378946	115843	1	1	0	0.1218695	0	1	4	0.1218695	1.26272838
chr2	149378946	149447756	68811	3	2	0	0.30102999	0	1	4	0.05404976	1.26272838
chr2	149447756	149674914	227159	5	2	0	0.30102999	0	1	5	0.05404976	1.60515106
chr2	149674914	149957905	282992	6	1	0	0.1218695	0	1	4	0.1218695	1.26272838
chr2	149957905	149987423	29519	2	2	0	0.30102999	0	1	4	0.05404976	1.26272838
chr2	149987423	150147521	160099	3	1	0	0.1218695	0	1	4	0.1218695	1.26272838
chr2	150147521	150147580	60	1	2	0	0.30102999	0	1	4	0.05404976	1.26272838
chr2	150147580	150305610	158031	4	1	0	0.1218695	0	1	4	0.1218695	1.26272838
chr2	150305610	150344859	39250	1	1	0	0.1218695	0	1	3	0.1218695	0.93173516
chr2	150344859	150443172	98314	2	1	0	0.1218695	0	1	4	0.1218695	1.26272838
chr2	150443172	150443231	60	1	1	0	0.1218695	0	1	5	0.1218695	1.60515106
chr2	150443231	150493583	50353	1	1	0	0.1218695	0	1	3	0.1218695	0.93173516
chr2	150493583	150493642	60	1	1	0	0.1218695	0	1	5	0.1218695	1.60515106
chr2	150493642	150763297	269656	3	0	0	0	0	0	3	0	0.93173516
chr2	150763297	151128706	365410	2	0	0	0	0	0	2	0	0.61140001
chr2	151792931	151902664	109734	2	1	0	0.30102999	0	0	0	0	0
chr2	151902664	152084100	181437	3	1	0	0.30102999	0	0	1	0	0.30102999
chr2	152084100	152226633	142534	2	1	0	0.30102999	0	0	0	0	0
chr2	152226633	152278253	51621	2	1	0	0.1218695	0	1	1	0.1218695	0.30102999
chr2	152278253	152326676	48424	2	1	0	0.1218695	0	1	2	0.1218695	0.61140001
chr2	152326676	152546197	219522	6	1	0	0.1218695	0	1	0	0.1218695	0
chr2	152546197	152578867	32671	2	1	0	0.1218695	0	1	1	0.1218695	0.30102999
chr2	152578867	152641430	62564	2	1	0	0.05404976	0	2	1	0.30102999	0.30102999
chr2	152641430	152727396	85967	4	2	0	0.1575501	0	2	1	0.1575501	0.30102999
chr2	152727396	153029854	302459	6	1	0	0.05404976	0	2	1	0.30102999	0.30102999
chr2	153029854	153243343	213490	3	1	0	0.1218695	0	1	1	0.1218695	0.30102999
chr2	153243343	153374551	131209	3	1	0	0.05404976	0	2	1	0.30102999	0.30102999
chr2	153374551	153374610	60	1	2	0	0.1575501	0	2	2	0.1575501	0.61140001
chr2	153374610	153503455	128846	2	2	0	0.1575501	0	2	1	0.1575501	0.30102999
chr2	153503455	153577066	73612	3	1	0	0.1218695	0	1	1	0.1218695	0.30102999
chr2	153577066	153633955	56890	2	2	0	0.30102999	0	1	1	0.05404976	0.30102999
chr2	153633955	153634014	60	1	2	0	0.1575501	0	2	1	0.1575501	0.30102999
chr2	153634014	153741336	107323	1	2	0	0.30102999	0	1	1	0.05404976	0.30102999
chr2	153741336	154013041	271706	2	2	0	0.61140001	0	0	1	0	0.30102999
chr2	154013041	154013100	60	1	2	0	0.30102999	0	1	2	0.05404976	0.61140001
chr2	154013100	154164613	151514	1	2	0	0.61140001	0	0	1	0	0.30102999
chr2	154164613	154333871	169259	4	2	0	0.61140001	0	0	2	0	0.61140001
chr2	154333871	154338522	4652	3	2	0	0.30102999	0	1	2	0.05404976	0.61140001
chr2	154338522	154538832	200311	2	2	0	0.61140001	0	0	2	0	0.61140001
chr2	154538832	154538891	60	1	2	1	0.61140001	0.05404976	0	2	0	0.30102999
chr2	154538891	154747912	209022	1	2	0	0.61140001	0	0	2	0	0.61140001
chr2	154747912	154938385	190474	5	3	0	0.93173516	0	0	2	0	0.61140001
chr2	154938385	154938444	60	1	3	0	0.93173516	0	0	3	0	0.93173516
chr2	154938444	155138630	200187	4	3	0	0.93173516	0	0	2	0	0.61140001
chr2	155138630	155258410	119781	3	3	1	0.93173516	0.05404976	0	2	0	0.30102999
chr2	155258410	155398098	139689	2	3	0	0.93173516	0	0	2	0	0.61140001
chr2	155398098	155398157	60	1	4	1	1.26272838	0.05404976	0	2	0	0.30102999
chr2	155398157	155711313	313157	6	3	1	0.93173516	0.05404976	0	2	0	0.30102999
chr2	155711313	155859143	147831	2	3	1	0.93173516	0.1218695	0	1	0	0.1218695
chr2	155859143	155859202	60	1	3	1	0.93173516	0.05404976	0	2	0	0.30102999
chr2	155859202	156109766	250565	3	2	0	0.61140001	0	0	1	0	0.30102999
chr2	156109766	156109825	60	1	3	0	0.93173516	0	0	1	0	0.30102999
chr2	156109825	156338297	228473	3	2	0	0.61140001	0	0	1	0	0.30102999
chr2	156338297	156522754	184458	1	2	0	0.61140001	0	0	0	0	0
chr2	156522754	156522813	60	1	3	1	0.51676182	0.1218695	1	1	0.02438896	0.1218695
chr2	156522813	156761458	238646	1	2	1	0.30102999	0.30102999	1	0	0.05404976	0
chr2	156761458	156882590	121133	1	2	0	0.30102999	0	1	0	0.05404976	0
chr2	156882590	156882649	60	1	2	0	0.30102999	0	1	1	0.05404976	0.30102999
chr2	156882649	156989242	106594	1	2	0	0.30102999	0	1	0	0.05404976	0
chr2	156989242	157181872	192631	2	2	0	0.61140001	0	0	0	0	0
chr2	157181872	157214974	33103	3	2	0	0.30102999	0	1	0	0.05404976	0
chr2	157214974	157353001	138028	2	2	0	0.61140001	0	0	0	0	0
chr2	157353001	157353060	60	1	2	0	0.61140001	0	0	1	0	0.30102999
chr2	157353060	157511681	158622	3	2	0	0.61140001	0	0	0	0	0
chr2	157511681	157511740	60	1	2	0	0.30102999	0	1	1	0.05404976	0.30102999
chr2	157511740	157792260	280521	2	2	0	0.61140001	0	0	1	0	0.30102999
chr2	157792260	157792319	60	1	2	0	0.30102999	0	1	1	0.05404976	0.30102999
chr2	157792319	158179678	387360	5	2	0	0.61140001	0	0	1	0	0.30102999
chr2	158179678	158179737	60	1	2	0	0.30102999	0	1	2	0.05404976	0.61140001
chr2	158179737	158385820	206084	2	1	0	0.30102999	0	0	1	0	0.30102999
chr2	158385820	158476521	90702	4	1	0	0.30102999	0	0	2	0	0.61140001
chr2	158476521	158622675	146155	2	1	0	0.30102999	0	0	1	0	0.30102999
chr2	158622675	159150071	527397	13	2	0	0.30102999	0	1	1	0.05404976	0.30102999
chr2	159150071	159150130	60	1	2	0	0.30102999	0	1	2	0.05404976	0.61140001
chr2	159150130	159246300	96171	2	2	0	0.30102999	0	1	1	0.05404976	0.30102999
chr2	159246300	159246359	60	1	2	0	0.30102999	0	1	3	0.05404976	0.93173516
chr2	159246359	159344553	98195	2	1	0	0.1218695	0	1	2	0.1218695	0.61140001
chr2	159344553	159421330	76778	2	2	0	0.30102999	0	1	2	0.05404976	0.61140001
chr2	159421330	159655906	234577	7	2	0	0.30102999	0	1	3	0.05404976	0.93173516
chr2	159655906	159844069	188164	2	2	0	0.30102999	0	1	2	0.05404976	0.61140001
chr2	159844069	159920859	76791	3	2	0	0.1575501	0	2	2	0.1575501	0.61140001
chr2	159920859	159920918	60	1	3	1	0.30102999	0.05404976	2	2	0.08289318	0.30102999
chr2	159920918	160004580	83663	2	3	1	0.30102999	0.1218695	2	1	0.08289318	0.1218695
chr2	160004580	160004639	60	1	3	1	0.30102999	0.05404976	2	2	0.08289318	0.30102999
chr2	160004639	160175647	171009	4	3	1	0.51676182	0.05404976	1	2	0.02438896	0.30102999
chr2	160175647	160175706	60	1	3	1	0.30102999	0.05404976	2	2	0.08289318	0.30102999
chr2	160175706	160473407	297702	8	3	0	0.51676182	0	1	2	0.02438896	0.61140001
chr2	160473407	160537598	64192	2	3	0	0.51676182	0	1	3	0.02438896	0.93173516
chr2	160537598	160573862	36265	1	2	0	0.30102999	0	1	3	0.05404976	0.93173516
chr2	160573862	160573921	60	1	2	1	0.30102999	0.02438896	1	3	0.05404976	0.51676182
chr2	160573921	160659984	86064	6	2	0	0.30102999	0	1	3	0.05404976	0.93173516
chr2	160659984	160688288	28305	2	2	1	0.30102999	0.02438896	1	3	0.05404976	0.51676182
chr2	160688288	160766746	78459	2	2	0	0.30102999	0	1	2	0.05404976	0.61140001
chr2	160766746	160766805	60	1	2	1	0.30102999	0.05404976	1	2	0.05404976	0.30102999
chr2	160766805	160916723	149919	3	2	0	0.30102999	0	1	2	0.05404976	0.61140001
chr2	160916723	161192063	275341	6	1	0	0.1218695	0	1	2	0.1218695	0.61140001
chr2	161192063	161542384	350322	7	2	0	0.30102999	0	1	2	0.05404976	0.61140001
chr2	161542384	161715746	173363	1	1	0	0.1218695	0	1	1	0.1218695	0.30102999
chr2	161715746	162047499	331754	4	1	0	0.1218695	0	1	2	0.1218695	0.61140001
chr2	162047499	162047558	60	1	1	0	0.1218695	0	1	3	0.1218695	0.93173516
chr2	162047558	162177878	130321	3	1	0	0.1218695	0	1	2	0.1218695	0.61140001
chr2	162177878	162281027	103150	4	1	0	0.1218695	0	1	3	0.1218695	0.93173516
chr2	162281027	162281086	60	1	2	0	0.30102999	0	1	3	0.05404976	0.93173516
chr2	162281086	162350876	69791	1	1	0	0.1218695	0	1	3	0.1218695	0.93173516
chr2	162350876	162411008	60133	2	2	0	0.30102999	0	1	3	0.05404976	0.93173516
chr2	162411008	162524214	113207	3	2	0	0.30102999	0	1	2	0.05404976	0.61140001
chr2	162524214	163136480	612267	13	3	0	0.51676182	0	1	3	0.02438896	0.93173516
chr2	163136480	163169454	32975	2	3	1	0.51676182	0.01091641	1	4	0.02438896	0.76005302
chr2	163169454	163201360	31907	1	3	0	0.51676182	0	1	4	0.02438896	1.26272838
chr2	163201360	163228010	26651	1	2	0	0.30102999	0	1	3	0.05404976	0.93173516
chr2	163228010	163228069	60	1	2	1	0.30102999	0.02438896	1	3	0.05404976	0.51676182
chr2	163228069	163360987	132919	3	2	1	0.30102999	0.05404976	1	2	0.05404976	0.30102999
chr2	163360987	163361046	60	1	2	1	0.30102999	0.02438896	1	3	0.05404976	0.51676182
chr2	163361046	163483074	122029	2	2	1	0.30102999	0.05404976	1	2	0.05404976	0.30102999

chr2	163483074	163562260	79187	3	2	1	0.30102999	0.02438896	1	3	0.05404976	0.51676182
chr2	163562260	163744864	182605	3	2	0	0.30102999	0	1	3	0.05404976	0.93173516
chr2	163744864	163875903	131040	1	1	0	0.1218695	0	1	2	0.1218695	0.61140001
chr2	163875903	163875962	60	1	1	2	0.1218695	0.1575501	1	2	0.1218695	0.1575501
chr2	163875962	164405692	529731	3	0	1	0	0.05404976	0	2	0	0.30102999
chr2	164405692	164405751	60	1	1	1	0.30102999	0.05404976	0	2	0	0.30102999
chr2	164405751	164640805	235055	5	0	1	0	0.05404976	0	2	0	0.30102999
chr2	164640805	164640864	60	1	0	2	0	0.1575501	0	2	0	0.1575501
chr2	164640864	165053775	412912	3	0	1	0	0.05404976	0	2	0	0.30102999
chr2	165053775	165173679	119905	2	1	1	0.1218695	0.05404976	1	2	0.1218695	0.30102999
chr2	165173679	165429580	255902	5	1	1	0.30102999	0.05404976	0	2	0	0.30102999
chr2	165429580	165755330	325751	7	0	1	0	0.05404976	0	2	0	0.30102999
chr2	165755330	165943988	188659	4	1	1	0.30102999	0.05404976	0	2	0	0.30102999
chr2	165943988	166019786	75799	4	3	1	0.93173516	0.05404976	0	2	0	0.30102999
chr2	166019786	166198780	178995	5	3	1	0.51676182	0.05404976	1	2	0.02438896	0.30102999
chr2	166198780	166246100	47321	3	3	2	0.51676182	0.1575501	1	2	0.02438896	0.1575501
chr2	166246100	166429058	182959	3	1	2	0.30102999	0.1575501	0	2	0	0.1575501
chr2	166429058	166478707	49650	1	1	1	0.30102999	0.05404976	0	2	0	0.30102999
chr2	166478707	166519701	40995	2	3	1	0.93173516	0.05404976	0	2	0	0.30102999
chr2	166519701	166802060	282360	5	3	0	0.93173516	0	0	2	0	0.61140001
chr2	166802060	166993501	191442	6	3	1	0.93173516	0.05404976	0	2	0	0.30102999
chr2	166993501	166993560	60	1	3	2	0.93173516	0.08289318	0	3	0	0.30102999
chr2	166993560	167099556	105997	2	1	0	0.30102999	0	0	2	0	0.61140001
chr2	167099556	167099615	60	1	1	1	0.30102999	0.02438896	0	3	0	0.51676182
chr2	167099615	167138284	38670	1	1	0	0.30102999	0	0	3	0	0.93173516
chr2	167138284	167182965	44682	2	2	0	0.61140001	0	0	3	0	0.93173516
chr2	167182965	167224787	41823	2	3	0	0.93173516	0	0	3	0	0.93173516
chr2	167224787	167322057	97271	3	2	0	0.61140001	0	0	3	0	0.93173516
chr2	167322057	167373565	51509	2	2	0	0.61140001	0	0	2	0	0.61140001
chr2	167373565	167373624	60	1	2	0	0.61140001	0	0	3	0	0.93173516
chr2	167373624	167602904	229281	1	1	0	0.30102999	0	0	3	0	0.93173516
chr2	167602904	167602963	60	1	2	0	0.61140001	0	0	3	0	0.93173516
chr2	167602963	167823075	220113	3	1	0	0.30102999	0	0	3	0	0.93173516
chr2	167823075	167823134	60	1	3	0	0.93173516	0	0	3	0	0.93173516
chr2	167823134	168220382	397249	8	2	0	0.61140001	0	0	3	0	0.93173516
chr2	168220382	168481627	261246	2	2	0	0.61140001	0	0	2	0	0.61140001
chr2	168481627	168610662	129036	2	2	0	0.30102999	0	1	2	0.05404976	0.61140001
chr2	168610662	168689947	79286	1	1	0	0.30102999	0	0	2	0	0.61140001
chr2	168689947	168811070	121124	3	1	0	0.30102999	0	0	3	0	0.93173516
chr2	168811070	168821181	10112	2	2	0	0.30102999	0	1	3	0.05404976	0.93173516
chr2	168821181	168920871	99691	2	1	0	0.1218695	0	1	3	0.1218695	0.93173516
chr2	168920871	168986197	65327	1	1	0	0.1218695	0	1	2	0.1218695	0.61140001
chr2	168986197	169038537	52341	2	1	1	0.1218695	0.05404976	1	2	0.1218695	0.30102999
chr2	169038537	169225746	187210	2	1	1	0.30102999	0.05404976	0	2	0	0.30102999
chr2	169225746	169315509	89764	2	1	1	0.30102999	0.01091641	0	4	0	0.76005302
chr2	169315509	169370853	55345	1	1	0	0.30102999	0	0	2	0	0.61140001
chr2	169370853	169370912	60	1	1	0	0.30102999	0	0	3	0	0.93173516
chr2	169370912	169418459	47548	1	1	0	0.30102999	0	0	2	0	0.61140001
chr2	169418459	169510157	91699	3	1	1	0.1218695	0.05404976	1	2	0.1218695	0.30102999
chr2	169510157	169565350	55194	2	1	1	0.1218695	0.02438896	1	3	0.1218695	0.51676182
chr2	169565350	169565409	60	1	1	2	0.1218695	0.08289318	1	3	0.1218695	0.30102999
chr2	169565409	169625853	60445	1	1	1	0.1218695	0.05404976	1	2	0.1218695	0.30102999
chr2	169625853	169668104	42252	2	1	1	0.1218695	0.1218695	1	1	0.1218695	0.1218695
chr2	169668104	169668161	58	1	1	1	0.1218695	0.05404976	1	2	0.1218695	0.30102999
chr2	169668161	169801430	133270	6	1	0	0.1218695	0	1	2	0.1218695	0.61140001
chr2	169801430	169833157	31728	2	1	1	0.1218695	0.05404976	1	2	0.1218695	0.30102999
chr2	169833157	169869812	36656	2	1	1	0.1218695	0.02438896	1	3	0.1218695	0.51676182
chr2	169869812	169926184	56373	1	1	1	0.1218695	0.05404976	1	2	0.1218695	0.30102999
chr2	169926184	169952036	25853	2	1	2	0.1218695	0.1575501	1	2	0.1218695	0.1575501
chr2	169952036	169952095	60	1	1	2	0.05404976	0.1575501	2	2	0.30102999	0.1575501
chr2	169952095	170042047	89953	2	1	2	0.1218695	0.1575501	1	2	0.1218695	0.1575501
chr2	170042047	170175251	133205	3	1	3	0.1218695	0.30102999	1	2	0.1218695	0.08289318
chr2	170175251	170175302	52	1	1	4	0.1218695	0.47744371	1	2	0.1218695	0.0429175
chr2	170175302	170235905	60604	1	1	4	0.1218695	0.76005302	1	1	0.1218695	0.01091641
chr2	170235905	170339643	103739	1	1	3	0.1218695	0.51676182	1	1	0.1218695	0.02438896
chr2	170339643	170367406	27764	3	1	3	0.1218695	0.17593012	1	3	0.1218695	0.17593012
chr2	170367406	170377468	10063	2	1	3	0.1218695	0.30102999	1	2	0.1218695	0.08289318
chr2	170377468	170394745	17278	3	1	3	0.1218695	0.17593012	1	3	0.1218695	0.17593012
chr2	170394745	170480267	85523	2	1	2	0.1218695	0.08289318	1	3	0.1218695	0.30102999
chr2	170480267	170551718	71452	2	1	2	0.1218695	0.1575501	1	2	0.1218695	0.1575501
chr2	170551718	170656741	105024	3	1	2	0.1218695	0.08289318	1	3	0.1218695	0.30102999
chr2	170656741	170763134	106394	4	1	1	0.1218695	0.05404976	1	2	0.1218695	0.30102999
chr2	170763134	171055858	292725	9	1	1	0.1218695	0.02438896	1	3	0.1218695	0.51676182
chr2	171055858	171172665	116808	2	1	0	0.1218695	0	1	3	0.1218695	0.93173516
chr2	171172665	171376097	203433	4	1	0	0.1218695	0	1	2	0.1218695	0.61140001
chr2	171376097	171376156	60	1	1	1	0.1218695	0.05404976	1	2	0.1218695	0.30102999
chr2	171376156	171509654	133499	2	1	0	0.1218695	0	1	2	0.1218695	0.61140001
chr2	171509654	171559139	49486	2	1	0	0.30102999	0	0	2	0	0.61140001
chr2	171559139	171686360	127222	3	1	0	0.1218695	0	1	2	0.1218695	0.61140001
chr2	171686360	171843871	157512	4	1	1	0.1218695	0.05404976	1	2	0.1218695	0.30102999
chr2	171843871	171949550	105680	3	1	0	0.1218695	0	1	2	0.1218695	0.61140001
chr2	171949550	171949609	60	1	2	1	0.30102999	0.02438896	1	3	0.05404976	0.51676182
chr2	171949609	171975803	26195	1	2	0	0.30102999	0	1	3	0.05404976	0.93173516
chr2	171975803	171975862	60	1	2	0	0.30102999	0	1	4	0.05404976	1.26272838
chr2	171975862	172083377	107516	2	2	0	0.30102999	0	1	2	0.05404976	0.61140001
chr2	172083377	172083436	60	1	2	0	0.30102999	0	1	3	0.05404976	0.93173516
chr2	172083436	172285743	202308	4	2	0	0.30102999	0	1	2	0.05404976	0.61140001
chr2	172285743	172285802	60	1	2	0	0.30102999	0	1	3	0.05404976	0.93173516
chr2	172285802	172386933	101132	3	2	0	0.30102999	0	1	1	0.05404976	0.30102999
chr2	172386933	172512183	125251	3	2	0	0.30102999	0	1	2	0.05404976	0.61140001
chr2	172512183	172666705	154523	4	2	0	0.30102999	0	1	3	0.05404976	0.93173516
chr2	172666705	172666764	60	1	2	0	0.30102999	0	1	4	0.05404976	1.26272838
chr2	172666764	172750667	83904	2	2	0	0.30102999	0	1	2	0.05404976	0.61140001
chr2	172750667	172782089	31423	2	2	0	0.30102999	0	1	3	0.05404976	0.93173516
chr2	172782089	173295419	513331	10	2	0	0.30102999	0	1	2	0.05404976	0.61140001
chr2	173295419	173352660	57242	2	2	0	0.30102999	0	1	3	0.05404976	0.93173516
chr2	173352660	173352719	60	1	2	0	0.30102999	0	1	4	0.05404976	1.26272838
chr2	173352719	173602276	249558	7	2	0	0.30102999	0	1	3	0.05404976	0.93173516
chr2	173602276	173602335	60	1	2	0	0.30102999	0	1	4	0.05404976	1.26272838
chr2	173602335	173659874	57540	1	2	0	0.30102999	0	1	2	0.05404976	0.61140001
chr2	173659874	174356532	696659	14	2	0	0.30102999	0	1	1	0.05404976	0.30102999
chr2	174356532	174356591	60	1	2	0	0.30102999	0	1	2	0.05404976	0.61140001
chr2	174356591	174506070	149480	1	2	0	0.30102999	0	1	0	0.05404976	0
chr2	174506070	174757314	251245	4	2	1	0.30102999	0.30102999	1	0	0.05404976	0
chr2	174757314	175045780	288467	7	2	1	0.30102999	0.1218695	1	1	0.05404976	0.1218695
chr2	175045780	175258350	212571	5	2	1	0.1575501	0.1218695	2	1	0.1575501	0.1218695
chr2	175258350	175268847	10498	2	2	1	0.08289318	0.1218695	3	1	0.30102999	0.1218695
chr2	175268847	175386335	117489	4	2	1	0.1575501	0.1218695	2	1	0.1575501	0.1218695
chr2	175386335	175548502	162168	3	2	1	0.30102999	0.1218695	1	1	0.05404976	0.1218695
chr2	175548502	175742652	194151	5	2	1	0.1575501	0.1218695	2	1	0.1575501	0.1218695
chr2	175742652	175810499	67848	1	2	1	0.30102999	0.1218695	1	1	0.05404976	0.1218695
chr2	175810499	175904189	93691	3	2	1	0.30102999	0.05404976	1	2	0.05404976	0.30102999
chr2	175904189	176002885	98697	4	2	1	0.1575501	0.05404976	2	2	0.1575501	0.30102999
chr2	176002885	176597468	594584	7	2	1	0.30102999	0.05404976	1	2	0.05404976	0.30102999
chr2	176597468	176738092	140625	2	2	1	0.30102999	0.01091641	1	4	0.05404976	0.76005302
chr2	176738092	176974288	236197	8	2	1	0.30102999	0.00478973	1	5	0.05404976	1.02643191
chr2	176974288	176996612	22325	2	2	0	0.30102999	0	1	5	0.05404976	1.60515106
chr2	176996612	176996671	60	1	2	1	0.30102999	0.00478973	1	5	0.05404976	1.02643191
chr2	176996671	177017907	21237	2	2	0	0.30102999	0	1	4	0.05404976	1.26272838
chr2	177017907	177037460	19554	2	2	0	0.30102999	0	1	5	0.05404976	1.60515106
chr2	177037460	177135154	97695	2	2	0	0.30102999	0	1	2	0.05404976	0.61140001
chr2	177135154	177202281	67128	2	2	0	0.61140001	0	0	2	0	0.61140001
chr2	177202281	177466584	264304	4	2	0	0.61140001	0	0	3	0	0.93173516
chr2	177466584	177905315	438732	5	2	0	0.61140001	0	0	2	0	0.61140001

chr2	177905315	177905374	60	1	2	0	0.61140001	0	0	3	0	0.93173516
chr2	177905374	178081522	176149	3	2	0	0.61140001	0	0	2	0	0.61140001
chr2	178081522	178362416	280895	10	2	0	0.30102999	0	1	2	0.05404976	0.61140001
chr2	178362416	178588622	226207	7	2	0	0.30102999	0	1	3	0.05404976	0.93173516
chr2	178588622	178684998	96377	3	2	1	0.30102999	0.02438896	1	3	0.05404976	0.51676182
chr2	178684998	178936530	251533	6	2	0	0.30102999	0	1	3	0.05404976	0.93173516
chr2	178936530	178970269	33740	2	2	1	0.30102999	0.01091641	1	4	0.05404976	0.76005302
chr2	178970269	178978247	7979	1	2	1	0.30102999	0.02438896	1	3	0.05404976	0.51676182
chr2	178978247	179032111	53865	2	2	0	0.30102999	0	1	3	0.05404976	0.93173516
chr2	179032111	179032170	60	1	2	0	0.1575501	0	2	3	0.1575501	0.93173516
chr2	179032170	179090097	57928	1	2	0	0.30102999	0	1	3	0.05404976	0.93173516
chr2	179090097	179090156	60	1	2	1	0.30102999	0.02438896	1	3	0.05404976	0.51676182
chr2	179090156	179170825	80670	1	2	0	0.30102999	0	1	3	0.05404976	0.93173516
chr2	179170825	179170884	60	1	2	0	0.1575501	0	2	4	0.1575501	1.26272838
chr2	179170884	179369665	198782	6	2	0	0.30102999	0	1	4	0.05404976	1.26272838
chr2	179369665	179499968	130304	4	2	0	0.30102999	0	1	5	0.05404976	1.60515106
chr2	179499968	179536799	36832	2	2	1	0.30102999	0.00478973	1	5	0.05404976	1.02643191
chr2	179536799	179606242	69444	2	2	1	0.30102999	0.01091641	1	4	0.05404976	0.76005302
chr2	179606242	179753424	147183	6	2	1	0.30102999	0.00478973	1	5	0.05404976	1.02643191
chr2	179753424	179753483	60	1	2	1	0.30102999	0.00204627	1	6	0.05404976	1.31360226
chr2	179753483	179794147	40665	1	2	1	0.30102999	0.00478973	1	5	0.05404976	1.02643191
chr2	179794147	179893928	99782	2	2	1	0.1575501	0.00478973	2	5	0.1575501	1.02643191
chr2	179893928	180093318	199391	4	2	1	0.30102999	0.01091641	1	4	0.05404976	0.76005302
chr2	180093318	180117349	24032	2	3	1	0.51676182	0.01091641	1	4	0.02438896	0.76005302
chr2	180117349	180306799	189451	2	2	1	0.30102999	0.01091641	1	4	0.05404976	0.76005302
chr2	180306799	180306858	60	1	3	1	0.51676182	0.01091641	1	4	0.02438896	0.76005302
chr2	180306858	180485698	178841	6	3	1	0.93173516	0.01091641	0	4	0	0.76005302
chr2	180485698	180558211	72514	2	2	1	0.61140001	0.01091641	0	4	0	0.76005302
chr2	180558211	180759301	201091	5	2	1	0.61140001	0.02438896	0	3	0	0.51676182
chr2	180759301	180879667	120367	3	2	1	0.61140001	0.01091641	0	4	0	0.76005302
chr2	180879667	180982972	103306	1	1	1	0.30102999	0.01091641	0	4	0	0.76005302
chr2	180982972	181233021	250050	1	0	0	0	0	0	3	0	0.93173516
chr2	181233021	181400740	167720	2	1	0	0.30102999	0	0	3	0	0.93173516
chr2	181400740	181570071	169332	1	0	0	0	0	0	2	0	0.61140001
chr2	181570071	181725015	154945	1	0	0	0	0	0	1	0	0.30102999
chr2	181725015	181725074	60	1	1	0	0.30102999	0	0	2	0	0.61140001
chr2	181725074	181968837	243764	4	0	0	0	0	0	2	0	0.61140001
chr2	181968837	181968896	60	1	0	0	0	0	0	3	0	0.93173516
chr2	181968896	182201330	232435	3	0	0	0	0	0	2	0	0.61140001
chr2	182201330	182714011	512682	11	0	0	0	0	0	3	0	0.93173516
chr2	182714011	182912528	198518	7	1	1	0.30102999	0.02438896	0	3	0	0.51676182
chr2	182912528	183011861	99334	3	1	1	0.1218695	0.02438896	1	3	0.1218695	0.51676182
chr2	183011861	183387206	375346	8	1	0	0.1218695	0	1	3	0.1218695	0.93173516
chr2	183387206	183643261	256056	5	1	0	0.30102999	0	0	3	0	0.93173516
chr2	183643261	183699541	56281	3	2	0	0.61140001	0	0	3	0	0.93173516
chr2	183699541	183726554	27014	1	1	0	0.30102999	0	0	3	0	0.93173516
chr2	183726554	183894766	168213	3	1	0	0.30102999	0	0	2	0	0.61140001
chr2	183894766	184016273	121508	3	1	0	0.30102999	0	0	3	0	0.93173516
chr2	184016273	184140036	123764	2	1	0	0.30102999	0	0	2	0	0.61140001
chr2	184140036	184330449	190414	2	0	0	0	0	0	2	0	0.61140001
chr2	184330449	184420806	90358	2	1	0	0.30102999	0	0	2	0	0.61140001
chr2	184420806	184420865	60	1	1	0	0.30102999	0	0	3	0	0.93173516
chr2	184420865	184558208	137344	1	1	0	0.30102999	0	0	2	0	0.61140001
chr2	184558208	184737955	179748	1	0	0	0	0	0	2	0	0.61140001
chr2	184737955	184738014	60	1	1	0	0.30102999	0	0	2	0	0.61140001
chr2	184738014	184965883	227870	2	0	0	0	0	0	2	0	0.61140001
chr2	184965883	184965942	60	1	0	0	0	0	0	4	0	1.26272838
chr2	184965942	185234224	268283	2	0	0	0	0	0	2	0	0.61140001
chr2	185234224	185337884	103661	2	0	0	0	0	0	3	0	0.93173516
chr2	185337884	185618130	280247	7	0	0	0	0	0	2	0	0.61140001
chr2	185618130	186094549	476420	6	0	0	0	0	0	3	0	0.93173516
chr2	186094549	186217556	123008	2	1	0	0.30102999	0	0	3	0	0.93173516
chr2	186217556	186474070	256515	2	0	0	0	0	0	3	0	0.93173516
chr2	186474070	186590113	116044	3	1	0	0.30102999	0	0	3	0	0.93173516
chr2	186590113	186612135	22023	2	2	0	0.61140001	0	0	3	0	0.93173516
chr2	186612135	186659631	47497	2	1	0	0.30102999	0	0	3	0	0.93173516
chr2	186659631	186659690	60	1	2	0	0.61140001	0	0	3	0	0.93173516
chr2	186659690	186763813	104124	1	1	0	0.30102999	0	0	3	0	0.93173516
chr2	186763813	186763872	60	1	2	0	0.30102999	0	1	3	0.05404976	0.93173516
chr2	186763872	186989575	225704	2	2	0	0.30102999	0	1	2	0.05404976	0.61140001
chr2	186989575	187303408	313834	3	3	0	0.51676182	0	1	2	0.02438896	0.61140001
chr2	187303408	187367117	63710	2	4	0	0.76005302	0	1	2	0.01091641	0.61140001
chr2	187367117	187406659	39543	4	4	0	0.76005302	0	1	3	0.01091641	0.93173516
chr2	187406659	187477526	70868	2	4	0	0.76005302	0	1	2	0.01091641	0.61140001
chr2	187477526	187559898	82373	2	4	0	0.76005302	0	1	3	0.01091641	0.93173516
chr2	187559898	188103179	543282	8	3	0	0.51676182	0	1	3	0.02438896	0.93173516
chr2	188103179	188210149	106971	2	3	0	0.51676182	0	1	4	0.02438896	1.26272838
chr2	188210149	188296166	86018	4	4	0	0.76005302	0	1	4	0.01091641	1.26272838
chr2	188296166	188361705	65540	2	3	0	0.51676182	0	1	4	0.02438896	1.26272838
chr2	188361705	188361764	60	1	4	0	0.76005302	0	1	4	0.01091641	1.26272838
chr2	188361764	188546067	184304	3	3	0	0.51676182	0	1	4	0.02438896	1.26272838
chr2	188546067	188842449	296383	2	2	0	0.61140001	0	0	4	0	1.26272838
chr2	188842449	188960064	117616	2	2	1	0.61140001	0.01091641	0	4	0	0.76005302
chr2	188960064	189200464	240401	6	3	1	0.51676182	0.00478973	1	5	0.02438896	1.02643191
chr2	189200464	189314202	113739	3	2	1	0.30102999	0.00478973	1	5	0.05404976	1.02643191
chr2	189314202	189354436	40235	1	2	1	0.30102999	0.01091641	1	4	0.05404976	0.76005302
chr2	189354436	189354495	60	1	3	1	0.51676182	0.01091641	1	4	0.02438896	0.76005302
chr2	189354495	189654645	300151	6	3	1	0.51676182	0.02438896	1	3	0.02438896	0.51676182
chr2	189654645	189654704	60	1	3	1	0.51676182	0.01091641	1	4	0.02438896	0.76005302
chr2	189654704	189735237	80534	1	2	1	0.30102999	0.01091641	1	4	0.05404976	0.76005302
chr2	189735237	189822690	87454	2	2	1	0.30102999	0.00478973	1	5	0.05404976	1.02643191
chr2	189822690	189839204	16515	2	2	1	0.30102999	0.00204627	1	6	0.05404976	1.31360226
chr2	189839204	189925490	86287	4	2	1	0.30102999	0.00478973	1	5	0.05404976	1.02643191
chr2	189925490	189975131	49642	1	2	1	0.30102999	0.01091641	1	4	0.05404976	0.76005302
chr2	189975131	190044294	69164	3	2	1	0.30102999	0.00478973	1	5	0.05404976	1.02643191
chr2	190044294	190258780	214487	1	2	1	0.30102999	0.02438896	1	3	0.05404976	0.51676182
chr2	190258780	190340163	81384	2	2	1	0.1575501	0.02438896	2	3	0.1575501	0.51676182
chr2	190340163	190532680	192518	3	2	1	0.1575501	0.01091641	2	4	0.1575501	0.76005302
chr2	190532680	190655560	122881	4	2	1	0.1575501	0.02438896	2	3	0.1575501	0.51676182
chr2	190655560	190682804	27245	3	2	1	0.1575501	0.01091641	2	4	0.1575501	0.76005302
chr2	190682804	190742094	59291	3	2	1	0.1575501	0.02438896	2	3	0.1575501	0.51676182
chr2	190742094	190811987	69894	1	2	1	0.30102999	0.02438896	1	3	0.05404976	0.51676182
chr2	190811987	190921273	109287	1	2	0	0.30102999	0	1	3	0.05404976	0.93173516
chr2	190921273	190970894	49622	3	3	0	0.51676182	0	1	3	0.02438896	0.93173516
chr2	190970894	190970953	60	1	3	0	0.51676182	0	1	4	0.02438896	1.26272838
chr2	190970953	191158964	188012	5	2	0	0.30102999	0	1	3	0.05404976	0.93173516
chr2	191158964	191355622	196659	6	2	0	0.30102999	0	1	4	0.05404976	1.26272838
chr2	191355622	191451638	96017	2	2	0	0.30102999	0	1	3	0.05404976	0.93173516
chr2	191451638	191556500	104863	3	2	0	0.30102999	0	1	4	0.05404976	1.26272838
chr2	191556500	191704308	147809	1	2	0	0.30102999	0	1	3	0.05404976	0.93173516
chr2	191704308	192391121	686814	15	2	0	0.30102999	0	1	2	0.05404976	0.61140001
chr2	192391121	192814902	423782	7	2	0	0.61140001	0	0	2	0	0.61140001
chr2	192814902	192814961	60	1	2	0	0.30102999	0	1	2	0.05404976	0.61140001
chr2	192814961	192969462	154502	3	2	0	0.61140001	0	0	2	0	0.61140001
chr2	192969462	193773574	804113	7	0	0	0	0	0	2	0	0.61140001
chr2	193773574	193773633	60	1	1	0	0.30102999	0	0	3	0	0.93173516
chr2	194682359	194682418	60	1	1	0	0.30102999	0	0	0	0	0
chr2	195588003	195799327	211325	2	0	0	0	0	0	1	0	0.30102999
chr2	195799327	195909737	110411	2	0	0	0	0	0	2	0	0.61140001
chr2	195909737	196101908	192172	2	0	0	0	0	0	3	0	0.93173516
chr2	196101908	196101967	60	1	2	0	0.30102999	0	1	4	0.05404976	1.26272838
chr2	196101967	196351251	249285	1	2	0	0.30102999	0	1	3	0.05404976	0.93173516
chr2	196351251	196499005	147755	2	1	0	0.1218695	0	1	3	0.1218695	0.93173516
chr2	196499005	196837193	338189	9	1	0	0.1218695	0	1	4	0.1218695	1.26272838

chr2	196837193	197081786	244594	5	1	0	0.1218695	0	1	3	0.1218695	0.93173516
chr2	197081786	197081845	60	1	1	0	0.1218695	0	1	4	0.1218695	1.26272838
chr2	197081845	197399574	317730	6	1	0	0.1218695	0	1	3	0.1218695	0.93173516
chr2	197399574	197451480	51907	2	1	0	0.1218695	0	1	4	0.1218695	1.26272838
chr2	197451480	197584153	132674	4	1	1	0.1218695	0.01091641	1	4	0.1218695	0.76005302
chr2	197584153	197597450	13298	2	1	2	0.1218695	0.02162467	1	5	0.1218695	0.68214471
chr2	197597450	197597509	60	1	1	2	0.1218695	0.01053319	1	6	0.1218695	0.91219088
chr2	197597509	197645140	47632	2	1	2	0.1218695	0.0429175	1	4	0.1218695	0.47744371
chr2	197645140	197645199	60	1	1	2	0.1218695	0.02162467	1	5	0.1218695	0.68214471
chr2	197645199	197712380	67182	2	1	2	0.1218695	0.0429175	1	4	0.1218695	0.47744371
chr2	197712380	197737191	24812	1	1	1	0.1218695	0.01091641	1	4	0.1218695	0.76005302
chr2	197737191	197767292	30102	2	1	1	0.1218695	0.00478973	1	5	0.1218695	1.02643191
chr2	197767292	197767351	60	1	1	1	0.05404976	0.00478973	2	5	0.30102999	1.02643191
chr2	197767351	197815812	48462	1	1	1	0.05404976	0.01091641	2	4	0.30102999	0.76005302
chr2	197815812	197878082	62271	1	1	1	0.05404976	0.02438896	2	3	0.30102999	0.51676182
chr2	197878082	198153006	274925	9	1	1	0.05404976	0.01091641	2	4	0.30102999	0.76005302
chr2	198153006	198274696	121691	2	1	1	0.1218695	0.01091641	1	4	0.1218695	0.76005302
chr2	198274696	198382204	107509	7	1	2	0.05404976	0.0429175	2	4	0.30102999	0.47744371
chr2	198382204	198436548	54345	2	1	1	0.05404976	0.01091641	2	4	0.30102999	0.76005302
chr2	198436548	198436607	60	1	1	2	0.05404976	0.0429175	2	4	0.30102999	0.47744371
chr2	198436607	198495889	59283	2	1	1	0.1218695	0.01091641	1	4	0.1218695	0.76005302
chr2	198495889	198495948	60	1	1	2	0.1218695	0.0429175	1	4	0.1218695	0.47744371
chr2	198495948	198572328	76381	2	1	1	0.1218695	0.01091641	1	4	0.1218695	0.76005302
chr2	198572328	198572387	60	1	1	1	0.05404976	0.01091641	2	4	0.30102999	0.76005302
chr2	198572387	198636604	64218	2	1	0	0.1218695	0	1	4	0.1218695	1.26272838
chr2	198636604	198684739	48136	2	1	0	0.1218695	0	1	5	0.1218695	1.60515106
chr2	198684739	198717116	32378	1	1	0	0.1218695	0	1	4	0.1218695	1.26272838
chr2	198717116	198926296	209181	5	1	0	0.30102999	0	0	4	0	1.26272838
chr2	198926296	199011771	85476	2	1	0	0.30102999	0	0	2	0	0.61140001
chr2	199011771	199112754	100984	3	1	0	0.30102999	0	0	4	0	1.26272838
chr2	199112754	199245600	132847	1	0	0	0	0	0	4	0	1.26272838
chr2	199245600	199410661	165062	1	0	0	0	0	0	2	0	0.61140001
chr2	199410661	199818779	408119	3	0	0	0	0	0	1	0	0.30102999
chr2	199818779	200188500	369722	5	0	0	0	0	0	3	0	0.93173516
chr2	200188500	200436597	248098	8	0	1	0	0.02438896	0	3	0	0.51676182
chr2	200436597	200527692	91096	1	0	1	0	0.05404976	0	2	0	0.30102999
chr2	200527692	200623899	96208	2	0	2	0	0.1575501	0	2	0	0.1575501
chr2	200623899	200676482	52584	2	0	2	0	0.0429175	0	4	0	0.47744371
chr2	200676482	200734985	58504	2	1	2	0.30102999	0.0429175	0	4	0	0.47744371
chr2	200734985	200803807	68823	4	1	2	0.30102999	0.02162467	0	5	0	0.68214471
chr2	200803807	200946982	143176	3	1	1	0.30102999	0.01091641	0	4	0	0.76005302
chr2	200946982	200947041	60	1	1	3	0.1218695	0.05670724	1	5	0.1218695	0.45545077
chr2	200947041	201033024	85984	1	1	3	0.1218695	0.10122019	1	4	0.1218695	0.30102999
chr2	201033024	201119231	86208	1	1	3	0.1218695	0.17593012	1	3	0.1218695	0.17593012
chr2	201119231	201169195	49965	2	1	3	0.1218695	0.10122019	1	4	0.1218695	0.30102999
chr2	201169195	201204544	35350	1	1	3	0.1218695	0.17593012	1	3	0.1218695	0.17593012
chr2	201204544	201204603	60	1	1	3	0.1218695	0.10122019	1	4	0.1218695	0.30102999
chr2	201204603	201229684	25082	1	1	3	0.30102999	0.17593012	0	3	0	0.17593012
chr2	201229684	201229743	60	1	1	3	0.30102999	0.10122019	0	4	0	0.30102999
chr2	201229743	201373685	143943	4	1	2	0.30102999	0.0429175	0	4	0	0.47744371
chr2	201373685	201434635	60951	3	1	3	0.30102999	0.05670724	0	5	0	0.45545077
chr2	201434635	201434694	60	1	1	3	0.1218695	0.05670724	1	5	0.1218695	0.45545077
chr2	201434694	201448172	13479	2	1	2	0.30102999	0.02162467	0	5	0	0.68214471
chr2	201448172	201673479	225308	6	1	3	0.30102999	0.05670724	0	5	0	0.45545077
chr2	201673479	201766681	93203	6	1	4	0.30102999	0.11390336	0	5	0	0.30102999
chr2	201766681	202221948	455268	15	1	3	0.30102999	0.05670724	0	5	0	0.45545077
chr2	202221948	202222007	60	1	1	3	0.1218695	0.05670724	1	5	0.1218695	0.45545077
chr2	202222007	202272223	50217	2	1	2	0.30102999	0.02162467	0	5	0	0.68214471
chr2	202272223	202313327	41105	3	1	3	0.1218695	0.05670724	1	5	0.1218695	0.45545077
chr2	202313327	202376776	63450	3	1	2	0.1218695	0.02162467	1	5	0.1218695	0.68214471
chr2	202376776	202446821	70046	2	1	0	0.1218695	0	1	4	0.1218695	1.26272838
chr2	202446821	202625782	178962	6	1	1	0.1218695	0.01091641	1	4	0.1218695	0.76005302
chr2	202625782	202625839	58	1	1	1	0.02438896	0.01091641	3	4	0.51676182	0.76005302
chr2	202625839	202714071	88233	2	1	1	0.1218695	0.01091641	1	4	0.1218695	0.76005302
chr2	202714071	202779782	65712	2	1	1	0.30102999	0.01091641	0	4	0	0.76005302
chr2	202779782	202847186	67405	2	1	2	0.30102999	0.0429175	0	4	0	0.47744371
chr2	202847186	202902999	55814	2	1	2	0.1218695	0.0429175	1	4	0.1218695	0.47744371
chr2	202902999	202998521	95523	2	1	2	0.30102999	0.0429175	0	4	0	0.47744371
chr2	202998521	203149074	150554	3	1	1	0.30102999	0.01091641	0	4	0	0.76005302
chr2	203149074	203218504	69431	3	1	2	0.1218695	0.0429175	1	4	0.1218695	0.47744371
chr2	203218504	203218563	60	1	1	2	0.05404976	0.0429175	2	4	0.30102999	0.47744371
chr2	203218563	203289542	70980	1	1	2	0.1218695	0.0429175	1	4	0.1218695	0.47744371
chr2	203289542	203379728	90187	3	1	3	0.05404976	0.10122019	2	4	0.30102999	0.30102999
chr2	203379728	203456771	77044	2	1	3	0.1218695	0.10122019	1	4	0.1218695	0.30102999
chr2	203456771	203507328	50558	2	1	3	0.05404976	0.10122019	2	4	0.30102999	0.30102999
chr2	203507328	203631801	124474	4	1	2	0.05404976	0.0429175	2	4	0.30102999	0.47744371
chr2	203631801	203661667	29867	2	1	2	0.02438896	0.0429175	3	4	0.51676182	0.47744371
chr2	203661667	203686021	24355	1	1	2	0.1218695	0.0429175	1	4	0.1218695	0.47744371
chr2	203686021	203686080	60	1	1	3	0.1218695	0.10122019	1	4	0.1218695	0.30102999
chr2	203686080	203735358	49279	1	1	2	0.1218695	0.08289318	1	3	0.1218695	0.30102999
chr2	203735358	203760902	25545	1	1	2	0.1218695	0.1575501	1	2	0.1218695	0.1575501
chr2	203760902	203776773	15872	2	1	3	0.1218695	0.17593012	1	3	0.1218695	0.17593012
chr2	203776773	203776829	57	1	1	3	0.1218695	0.10122019	1	4	0.1218695	0.30102999
chr2	203776829	203798971	22143	1	1	2	0.1218695	0.0429175	1	4	0.1218695	0.47744371
chr2	203798971	204031010	232040	6	1	2	0.1218695	0.02162467	1	5	0.1218695	0.68214471
chr2	204031010	204137415	106406	2	1	1	0.1218695	0.00478973	1	5	0.1218695	1.02643191
chr2	204137415	204137474	60	1	1	2	0.1218695	0.02162467	1	5	0.1218695	0.68214471
chr2	204137474	204324671	187198	8	0	2	0	0.0429175	1	4	0.30102999	0.47744371
chr2	204324671	204394564	69894	2	0	2	0	0.1575501	1	2	0.30102999	0.1575501
chr2	204394564	204493161	98598	2	0	2	0	0.08289318	1	3	0.30102999	0.30102999
chr2	204493161	204581362	88202	1	0	2	0	0.1575501	1	2	0.30102999	0.1575501
chr2	204581362	204594462	13101	2	0	2	0	0.0429175	1	4	0.30102999	0.47744371
chr2	204594462	204631833	37372	2	0	2	0	0.02162467	1	5	0.30102999	0.68214471
chr2	204631833	204735723	103891	2	0	2	0	0.1575501	1	2	0.30102999	0.1575501
chr2	204735723	204738033	2311	2	0	2	0	0.08289318	1	3	0.30102999	0.30102999
chr2	204738033	205354258	616226	4	0	0	0	0	1	1	0.30102999	0.30102999
chr2	205354258	205354317	60	1	0	0	0	0	1	3	0.30102999	0.93173516
chr2	205354317	205550889	196573	3	0	0	0	0	1	1	0.30102999	0.30102999
chr2	205550889	205580256	29368	2	0	0	0	0	1	2	0.30102999	0.61140001
chr2	205580256	205580315	60	1	1	0	0.05404976	0	2	4	0.30102999	1.26272838
chr2	205580315	205732083	151769	2	1	0	0.1218695	0	1	4	0.1218695	1.26272838
chr2	205732083	205794583	62501	1	0	0	0	0	0	4	0	1.26272838
chr2	205794583	205851012	56430	2	0	0	0	0	0	2	0	0.61140001
chr2	205851012	205851071	60	1	0	0	0	0	0	3	0	0.93173516
chr2	205851071	206106328	255258	4	0	0	0	0	0	2	0	0.61140001
chr2	206106328	206249500	143173	4	0	0	0	0	1	2	0.30102999	0.61140001
chr2	206249500	206376848	127349	4	0	0	0	0	1	3	0.30102999	0.93173516
chr2	206376848	206376907	60	1	0	1	0	0.02438896	1	3	0.30102999	0.51676182
chr2	206376907	206420219	43313	1	0	1	0	0.05404976	1	2	0.30102999	0.30102999
chr2	206420219	206461698	41480	1	0	1	0	0.05404976	0	2	0	0.30102999
chr2	206461698	206574546	112849	4	0	1	0	0.02438896	0	3	0	0.51676182
chr2	206574546	206574605	60	1	0	1	0	0.02438896	1	3	0.30102999	0.51676182
chr2	206574605	206631540	56936	2	0	1	0	0.02438896	0	3	0	0.51676182
chr2	206631540	206659450	27911	1	0	1	0	0.05404976	0	2	0	0.30102999
chr2	206659450	206659509	60	1	0	1	0	0.02438896	1	3	0.30102999	0.51676182
chr2	206659509	206832454	172946	2	0	1	0	0.02438896	0	3	0	0.51676182
chr2	206832454	206882391	49938	2	0	1	0	0.00478973	0	5	0	1.02643191
chr2	206882391	206882448	58	1	1	1	0.05404976	0.00478973	2	5	0.30102999	1.02643191
chr2	206882448	207162749	280302	7	1	1	0.30102999	0.00478973	0	5	0	1.02643191
chr2	207162749	207235292	72544	2	1	1	0.1218695	0.00478973	1	5	0.1218695	1.02643191
chr2	207235292	207544157	308866	6	1	1	0.30102999	0.00478973	0	5	0	1.02643191
chr2	207544157	207648864	104708	2	1	1	0.30102999	0.01091641	0	4	0	0.76005302
chr2	207648864	207833054	184191	6	1	1	0.30102999	0.00478973	0	5	0	1.02643191
chr2	207833054	207972973	139920	3	1	1	0.30102999	0.01091641	0	4	0	0.76005302

chr2	207972973	207973032	60	1	1	1	0.30102999	0.00478973	0	5	0	1.02643191
chr2	207973032	208313163	340132	6	1	1	0.30102999	0.01091641	0	4	0	0.76005302
chr2	208313163	208404527	91365	2	1	1	0.30102999	0.00478973	0	5	0	1.02643191
chr2	208404527	208541742	137216	7	1	1	0.1218695	0.00478973	1	5	0.1218695	1.02643191
chr2	208541742	208631364	89623	3	1	1	0.1218695	0.01091641	1	4	0.1218695	0.76005302
chr2	208631364	208710304	78941	1	1	1	0.30102999	0.01091641	0	4	0	0.76005302
chr2	208710304	208741236	30933	2	1	1	0.30102999	0.00478973	0	5	0	1.02643191
chr2	208741236	208857221	115986	2	1	1	0.30102999	0.01091641	0	4	0	0.76005302
chr2	208857221	208857280	60	1	1	1	0.30102999	0.00478973	0	5	0	1.02643191
chr2	208857280	208993534	136255	5	1	1	0.30102999	0.01091641	0	4	0	0.76005302
chr2	208993534	209026391	32858	2	1	1	0.30102999	0.02438896	0	3	0	0.51676182
chr2	209026391	209026450	60	1	1	1	0.30102999	0.01091641	0	4	0	0.76005302
chr2	209026450	209309442	282993	9	1	1	0.30102999	0.02438896	0	3	0	0.51676182
chr2	209309442	209359004	49563	2	1	1	0.30102999	0.01091641	0	4	0	0.76005302
chr2	209359004	209491032	132029	2	1	1	0.30102999	0.02438896	0	3	0	0.51676182
chr2	209491032	209568496	77465	2	1	1	0.30102999	0.01091641	0	4	0	0.76005302
chr2	209568496	209745339	176844	2	0	1	0	0.05404976	0	2	0	0.30102999
chr2	209745339	209836320	90982	2	0	1	0	0.02438896	0	3	0	0.51676182
chr2	209836320	210021463	185144	2	0	2	0	0.08289318	0	3	0	0.30102999
chr2	210021463	210341410	319948	4	0	3	0	0.10122019	1	4	0.30102999	0.30102999
chr2	210341410	210408298	66889	2	0	2	0	0.0429175	1	4	0.30102999	0.47744371
chr2	210408298	210408357	60	1	0	3	0	0.10122019	1	4	0.30102999	0.30102999
chr2	210408357	210489802	81446	2	0	2	0	0.0429175	0	4	0	0.47744371
chr2	210489802	210489861	60	1	0	4	0	0.18734596	0	4	0	0.18734596
chr2	210489861	210561692	71832	1	0	3	0	0.10122019	0	4	0	0.30102999
chr2	210561692	210561751	60	1	0	3	0	0.05670724	0	5	0	0.45545077
chr2	210561751	210682866	121116	3	0	3	0	0.10122019	0	4	0	0.30102999
chr2	210682866	210821769	138904	4	0	2	0	0.0429175	0	4	0	0.47744371
chr2	210821769	210999516	177748	6	0	1	0	0.01091641	0	4	0	0.76005302
chr2	210999516	211241961	242446	5	0	1	0	0.02438896	0	3	0	0.51676182
chr2	211241961	211242020	60	1	0	3	0	0.05670724	0	5	0	0.45545077
chr2	211242020	211296666	54647	1	0	2	0	0.0429175	0	4	0	0.47744371
chr2	211296666	211305491	8826	2	0	3	0	0.10122019	0	4	0	0.30102999
chr2	211305491	211376495	71005	2	0	1	0	0.01091641	0	4	0	0.76005302
chr2	211376495	211458283	81789	3	0	3	0	0.10122019	0	4	0	0.30102999
chr2	211458283	211483655	25373	2	0	3	0	0.05670724	0	5	0	0.45545077
chr2	211483655	211542926	59272	3	0	2	0	0.02162467	0	5	0	0.68214471
chr2	211542926	211687462	144537	1	0	1	0	0.01091641	0	4	0	0.76005302
chr2	211687462	211687521	60	1	0	1	0	0.01091641	1	4	0.30102999	0.76005302
chr2	211687521	212207827	520307	2	0	1	0	0.02438896	0	3	0	0.51676182
chr2	212207827	212360311	152485	4	0	2	0	0.08289318	0	3	0	0.30102999
chr2	212360311	212435598	75288	1	0	2	0	0.1575501	0	2	0	0.1575501
chr2	212435598	212488620	53023	2	1	2	0.30102999	0.1575501	0	2	0	0.1575501
chr2	212488620	212488679	60	1	1	2	0.1218695	0.08289318	1	3	0.1218695	0.30102999
chr2	212488679	212626304	137626	3	1	2	0.30102999	0.08289318	0	3	0	0.30102999
chr2	212626304	212885502	259199	5	0	2	0	0.1575501	0	2	0	0.1575501
chr2	212885502	212945192	59691	2	0	2	0	0.1575501	1	2	0.30102999	0.1575501
chr2	212945192	213010278	65087	2	0	2	0	0.08289318	2	3	0.61140001	0.30102999
chr2	213010278	213010337	60	1	0	2	0	0.0429175	2	4	0.61140001	0.47744371
chr2	213010337	213146361	136025	3	0	2	0	0.1575501	2	2	0.61140001	0.1575501
chr2	213146361	213211210	64850	2	0	2	0	0.0429175	2	4	0.61140001	0.47744371
chr2	213211210	213278502	67293	2	0	2	0	0.08289318	1	3	0.30102999	0.30102999
chr2	213278502	213292156	13655	2	0	3	0	0.10122019	2	4	0.61140001	0.30102999
chr2	213292156	213425995	133840	3	0	2	0	0.08289318	1	3	0.30102999	0.30102999
chr2	213425995	213638894	212900	3	0	3	0	0.10122019	2	4	0.61140001	0.30102999
chr2	213638894	213877289	238396	2	0	3	0	0.10122019	1	4	0.30102999	0.30102999
chr2	213877289	213935829	58541	4	0	3	0	0.10122019	2	4	0.61140001	0.30102999
chr2	213935829	213980390	44562	2	1	3	0.05404976	0.10122019	2	4	0.30102999	0.30102999
chr2	213980390	213980449	60	1	1	3	0.05404976	0.05670724	2	5	0.30102999	0.45545077
chr2	213980449	213999327	18879	1	0	3	0	0.05670724	1	5	0.30102999	0.45545077
chr2	213999327	214215255	215929	4	0	3	0	0.10122019	0	4	0	0.30102999
chr2	214215255	214229493	14239	2	0	3	0	0.05670724	1	5	0.30102999	0.45545077
chr2	214229493	214229552	60	1	0	4	0	0.11390336	1	5	0.30102999	0.30102999
chr2	214229552	214354752	125201	2	0	3	0	0.05670724	1	5	0.30102999	0.45545077
chr2	214354752	214548902	194151	4	0	2	0	0.0429175	1	4	0.30102999	0.47744371
chr2	214548902	214683886	134985	3	0	3	0	0.10122019	1	4	0.30102999	0.30102999
chr2	214683886	214683945	60	1	0	5	0	0.19510895	1	5	0.30102999	0.19510895
chr2	214683945	214858265	174321	3	0	4	0	0.11390336	1	5	0.30102999	0.30102999
chr2	214858265	214919902	61638	2	0	3	0	0.05670724	1	5	0.30102999	0.45545077
chr2	214919902	214919961	60	1	0	4	0	0.11390336	1	5	0.30102999	0.30102999
chr2	214919961	214992026	72066	2	0	4	0	0.18734596	1	4	0.30102999	0.18734596
chr2	214992026	215050998	58973	1	0	3	0	0.10122019	1	4	0.30102999	0.30102999
chr2	215050998	215051057	60	1	0	3	0	0.05670724	1	5	0.30102999	0.45545077
chr2	215051057	215190468	139412	2	0	3	0	0.10122019	1	4	0.30102999	0.30102999
chr2	215190468	215378625	188158	6	0	4	0	0.11390336	1	5	0.30102999	0.30102999
chr2	215378625	215420448	41824	1	0	3	0	0.05670724	1	5	0.30102999	0.45545077
chr2	215420448	215944012	523565	11	0	2	0	0.02162467	1	5	0.30102999	0.68214471
chr2	215944012	215976407	32396	2	0	2	0	0.01053319	1	6	0.30102999	0.91219088
chr2	215976407	216226309	249903	6	0	2	0	0.02162467	1	5	0.30102999	0.68214471
chr2	216226309	216299499	73191	3	0	2	0	0.01053319	1	6	0.30102999	0.91219088
chr2	216299499	216299557	59	1	0	3	0	0.03070643	1	6	0.30102999	0.63695542
chr2	216299557	216351479	51923	1	0	2	0	0.02162467	1	5	0.30102999	0.68214471
chr2	216351479	216435542	84064	2	0	3	0	0.05670724	1	5	0.30102999	0.45545077
chr2	216435542	216435601	60	1	0	4	0	0.06713722	1	6	0.30102999	0.44141547
chr2	216435601	216512368	76768	1	0	3	0	0.03070643	1	6	0.30102999	0.63695542
chr2	216512368	216733008	220641	3	0	2	0	0.02162467	1	5	0.30102999	0.68214471
chr2	216733008	216810439	77432	2	0	2	0	0.01053319	1	6	0.30102999	0.91219088
chr2	216810439	216850184	39746	1	0	1	0	0.00204627	1	6	0.30102999	1.31360226
chr2	216850184	217277777	427594	9	0	1	0	0.00478973	1	5	0.30102999	1.02643191
chr2	217277777	217279429	1653	2	0	1	0	0.00204627	1	6	0.30102999	1.31360226
chr2	217279429	217428847	149419	4	0	1	0	0.00478973	1	5	0.30102999	1.02643191
chr2	217428847	217509737	80891	2	0	1	0	0.01091641	1	4	0.30102999	0.76005302
chr2	217509737	217525718	15982	1	0	1	0	0.02438896	1	3	0.30102999	0.51676182
chr2	217525718	217592468	66751	5	0	2	0	0.0429175	1	4	0.30102999	0.47744371
chr2	217592468	217592527	60	1	0	2	0	0.02162467	1	5	0.30102999	0.68214471
chr2	217592527	217724168	131642	2	0	2	0	0.08289318	1	3	0.30102999	0.30102999
chr2	217724168	217724527	360	2	0	2	0	0.01053319	1	6	0.30102999	0.91219088
chr2	217724527	217821395	96869	1	0	2	0	0.08289318	1	3	0.30102999	0.30102999
chr2	217821395	217955845	134451	1	0	2	0	0.08289318	0	3	0	0.30102999
chr2	217955845	218195736	239892	1	0	2	0	0.1575501	0	2	0	0.1575501
chr2	218195736	218195795	60	1	0	2	0	0.1575501	1	2	0.30102999	0.1575501
chr2	218195795	218293587	97793	1	0	2	0	0.30102999	1	1	0.30102999	0.05404976
chr2	218293587	218293646	60	1	1	2	0.1218695	0.30102999	1	1	0.1218695	0.05404976
chr2	218293646	218511372	217727	2	0	1	0	0.1218695	1	1	0.30102999	0.1218695
chr2	218511372	218604084	92713	2	1	2	0.1218695	0.1575501	1	2	0.1218695	0.1575501
chr2	218604084	218678618	74535	2	1	2	0.1218695	0.08289318	1	3	0.1218695	0.30102999
chr2	218678618	218723943	45326	2	1	2	0.1218695	0.0429175	1	4	0.1218695	0.47744371
chr2	218723943	218747064	23122	2	1	2	0.1218695	0.02162467	1	5	0.1218695	0.68214471
chr2	218747064	218747123	60	1	1	2	0.1218695	0.01053319	1	6	0.1218695	0.91219088
chr2	218747123	218864922	117800	3	1	2	0.1218695	0.02162467	1	5	0.1218695	0.68214471
chr2	218864922	218864981	60	1	1	2	0.1218695	0.01053319	1	6	0.1218695	0.91219088
chr2	218864981	218903828	38848	1	1	2	0.1218695	0.02162467	1	5	0.1218695	0.68214471
chr2	218903828	218946576	42749	2	1	2	0.05404976	0.02162467	2	5	0.30102999	0.68214471
chr2	218946576	218946635	60	1	2	2	0.1575501	0.02162467	2	5	0.1575501	0.68214471
chr2	218946635	218990464	43830	1	2	2	0.1575501	0.0429175	2	4	0.1575501	0.47744371
chr2	218990464	218990513	50	1	2	2	0.1575501	0.02162467	2	5	0.1575501	0.68214471
chr2	218990513	219083500	92988	2	2	2	0.30102999	0.02162467	1	5	0.05404976	0.68214471
chr2	219083500	219093517	10018	2	2	2	0.1575501	0.02162467	2	5	0.1575501	0.68214471
chr2	219093517	219114285	20769	1	2	2	0.30102999	0.0429175	1	4	0.05404976	0.47744371
chr2	219114285	219128867	14583	2	3	2	0.51676182	0.01053319	1	6	0.02438896	0.91219088
chr2	219128867	219189272	60406	6	3	2	0.51676182	0.00493743	1	7	0.02438896	1.16581773
chr2	219189272	219189331	60	1	3	2	0.30102999	0.00493743	2	7	0.08289318	1.16581773
chr2	219189331	219314175	124845	4	3	2	0.51676182	0.00493743	1	7	0.02438896	1.16581773
chr2	219314175	219394722	80548	3	3	2	0.51676182	0.01053319	1	6	0.02438896	0.91219088

chr2	219394722	219427401	32680	2	3	2	0.51676182	0.00493743	1	7	0.02438896	1.16581773
chr2	219427401	219457387	29987	2	3	3	0.51676182	0.01598258	1	7	0.02438896	0.84395715
chr2	219457387	219457446	60	1	3	4	0.51676182	0.02074938	1	8	0.02438896	0.79906872
chr2	219457446	219746429	288984	13	3	4	0.51676182	0.03812622	1	7	0.02438896	0.60763643
chr2	219746429	220047798	301370	14	2	4	0.30102999	0.03812622	1	7	0.05404976	0.60763643
chr2	220047798	220134029	86232	8	2	3	0.30102999	0.01598258	1	7	0.05404976	0.84395715
chr2	220134029	220149280	15252	2	2	4	0.30102999	0.03812622	1	7	0.05404976	0.60763643
chr2	220149280	220471933	322654	14	2	3	0.61140001	0.01598258	0	7	0	0.84395715
chr2	220471933	220474444	2512	2	2	3	0.61140001	0.0079614	0	8	0	1.07548421
chr2	220474444	220561475	87032	3	2	3	0.61140001	0.01598258	0	7	0	0.84395715
chr2	220561475	220561534	60	1	2	3	0.61140001	0.0079614	0	8	0	1.07548421
chr2	220561534	220697256	135723	1	1	3	0.30102999	0.03070643	0	6	0	0.63695542
chr2	220697256	220813776	116521	1	1	3	0.30102999	0.05670724	0	5	0	0.45545077
chr2	220813776	220988286	174511	1	0	2	0	0.1575501	0	2	0	0.1575501
chr2	220988286	221059983	71698	2	0	2	0	0.08289318	0	3	0	0.30102999
chr2	221059983	221060042	60	1	0	3	0	0.17593012	0	3	0	0.17593012
chr2	221060042	221148418	88377	1	0	2	0	0.1575501	0	2	0	0.1575501
chr2	221148418	221542658	394241	4	0	2	0	0.30102999	0	1	0	0.05404976
chr2	221542658	221867870	325213	3	0	2	0	0.1575501	0	2	0	0.1575501
chr2	221867870	221975079	107210	2	0	3	0	0.17593012	0	3	0	0.17593012
chr2	221975079	222252339	277261	2	0	2	0	0.08289318	0	3	0	0.30102999
chr2	222252339	222308259	55921	3	0	2	0	0.0429175	0	4	0	0.47744371
chr2	222308259	222347103	38845	1	0	2	0	0.08289318	0	3	0	0.30102999
chr2	222347103	222429081	81979	2	0	2	0	0.1575501	0	2	0	0.1575501
chr2	222429081	223308421	879341	15	0	2	0	0.08289318	0	3	0	0.30102999
chr2	223308421	223389260	80840	2	0	2	0	0.1575501	0	2	0	0.1575501
chr2	223389260	223574560	185301	6	0	2	0	0.08289318	0	3	0	0.30102999
chr2	223574560	223746522	171963	2	0	2	0	0.1575501	0	2	0	0.1575501
chr2	223746522	223746581	60	1	0	2	0	0.08289318	1	3	0.30102999	0.30102999
chr2	223746581	223864544	117964	4	0	2	0	0.08289318	0	3	0	0.30102999
chr2	223864544	224312511	447968	8	0	3	0	0.17593012	0	3	0	0.17593012
chr2	224312511	224406198	93688	2	0	3	0	0.10122019	0	4	0	0.30102999
chr2	224406198	224462205	56008	2	0	3	0	0.05670724	0	5	0	0.45545077
chr2	224462205	224642532	180328	3	0	3	0	0.10122019	0	4	0	0.30102999
chr2	224642532	224666777	24246	2	0	3	0	0.05670724	0	5	0	0.45545077
chr2	224666777	224706849	40073	2	0	3	0	0.03070643	0	6	0	0.63695542
chr2	224706849	224741506	34658	1	0	3	0	0.10122019	0	4	0	0.30102999
chr2	224741506	224741565	60	1	0	3	0	0.05670724	0	5	0	0.45545077
chr2	224741565	224787064	45500	2	0	3	0	0.10122019	0	4	0	0.30102999
chr2	224787064	224787123	60	1	0	3	0	0.05670724	0	5	0	0.45545077
chr2	224787123	224830160	43038	1	0	3	0	0.10122019	0	4	0	0.30102999
chr2	224830160	224864014	33855	3	0	3	0	0.03070643	0	6	0	0.63695542
chr2	224864014	225053772	189759	5	0	2	0	0.0429175	0	4	0	0.47744371
chr2	225053772	225107864	54093	2	0	2	0	0.01053319	0	6	0	0.91219088
chr2	225107864	225243447	135584	2	0	2	0	0.00493743	0	7	0	1.16581773
chr2	225243447	225244553	1107	2	0	3	0	0.01598258	0	7	0	0.84395715
chr2	225244553	225336951	92399	2	0	2	0	0.00493743	0	7	0	1.16581773
chr2	225336951	225422472	85522	2	0	2	0	0.01053319	0	6	0	0.91219088
chr2	225422472	225470188	47717	3	0	2	0	0.00493743	0	7	0	1.16581773
chr2	225470188	225470247	60	1	0	3	0	0.01598258	0	7	0	0.84395715
chr2	225470247	225630226	159980	1	0	2	0	0.00493743	0	7	0	1.16581773
chr2	225630226	225707064	76839	4	0	2	0	0.00221948	0	8	0	1.44210395
chr2	225707064	225837099	130036	2	0	2	0	0.01053319	0	6	0	0.91219088
chr2	225837099	225945697	108599	2	0	2	0	0.02162467	0	5	0	0.68214471
chr2	225945697	226092485	146789	1	0	2	0	0.08289318	0	3	0	0.30102999
chr2	226092485	226427293	334809	5	0	1	0	0.05404976	0	2	0	0.30102999
chr2	226427293	227025192	597900	6	0	1	0	0.1218695	0	1	0	0.1218695
chr2	227025192	227332936	307745	3	0	2	0	0.1575501	0	2	0	0.1575501
chr2	227332936	227332995	60	1	0	2	0	0.0429175	0	4	0	0.47744371
chr2	227332995	227677913	344919	5	0	2	0	0.08289318	0	3	0	0.30102999
chr2	227677913	227723111	45199	1	0	2	0	0.1575501	0	2	0	0.1575501
chr2	227723111	227771631	48521	2	0	2	0	0.08289318	0	3	0	0.30102999
chr2	227771631	227771686	56	1	0	2	0	0.0429175	0	4	0	0.47744371
chr2	227771686	228032209	260524	9	0	1	0	0.02438896	0	3	0	0.51676182
chr2	228032209	228142296	110088	4	0	1	0	0.01091641	0	4	0	0.76005302
chr2	228142296	228419217	276922	7	0	1	0	0.02438896	0	3	0	0.51676182
chr2	228419217	228419276	60	1	0	1	0	0.00204627	0	6	0	1.31360226
chr2	228419276	228579881	160606	3	0	1	0	0.02438896	0	3	0	0.51676182
chr2	228579881	228767785	187905	4	0	1	0	0.00478973	0	5	0	1.02643191
chr2	228767785	228789218	21434	3	0	2	0	0.01053319	0	6	0	0.91219088
chr2	228789218	228856979	67762	1	0	2	0	0.02162467	0	5	0	0.68214471
chr2	228856979	229030046	173068	3	0	2	0	0.0429175	0	4	0	0.47744371
chr2	229030046	229284538	254493	2	0	2	0	0.1575501	0	2	0	0.1575501
chr2	229284538	229284597	60	1	0	2	0	0.0429175	0	4	0	0.47744371
chr2	229284597	229409257	124661	1	0	2	0	0.08289318	0	3	0	0.30102999
chr2	229409257	229542588	133332	1	0	2	0	0.1575501	0	2	0	0.1575501
chr2	229542588	229644717	102130	2	0	3	0	0.17593012	0	3	0	0.17593012
chr2	229644717	229755076	110360	2	0	4	0	0.30102999	0	3	0	0.10122019
chr2	229755076	229755135	60	1	0	4	0	0.18734596	0	4	0	0.18734596
chr2	229755135	229849823	94689	1	0	3	0	0.30102999	0	2	0	0.08289318
chr2	229849823	229930751	80929	2	0	3	0	0.17593012	0	3	0	0.17593012
chr2	229930751	230102420	171670	4	0	3	0	0.30102999	0	2	0	0.08289318
chr2	230102420	230102479	60	1	0	4	0	0.11390336	0	5	0	0.30102999
chr2	230102479	230161616	59138	1	0	4	0	0.30102999	0	3	0	0.10122019
chr2	230161616	230272278	110663	4	0	4	0	0.18734596	0	4	0	0.18734596
chr2	230272278	230294609	22332	1	0	4	0	0.30102999	0	3	0	0.10122019
chr2	230294609	230294668	60	1	0	5	0	0.30102999	0	4	0	0.11390336
chr2	230294668	230357931	63264	1	0	3	0	0.10122019	0	4	0	0.30102999
chr2	230357931	230411764	53834	2	0	3	0	0.05670724	0	5	0	0.45545077
chr2	230411764	230575419	163656	5	0	4	0	0.11390336	0	5	0	0.30102999
chr2	230575419	230724038	148620	3	0	2	0	0.0429175	0	4	0	0.47744371
chr2	230724038	230994184	270147	7	0	2	0	0.01053319	0	6	0	0.91219088
chr2	230994184	231079796	85613	3	0	2	0	0.00493743	0	7	0	1.16581773
chr2	231079796	231079855	60	1	0	3	0	0.01598258	0	7	0	0.84395715
chr2	231079855	231132544	52690	1	0	3	0	0.03070643	0	6	0	0.63695542
chr2	231132544	231159229	26686	2	0	3	0	0.01598258	0	7	0	0.84395715
chr2	231159229	231159288	60	1	0	3	0	0.0079614	0	8	0	1.07548421
chr2	231159288	231267663	108376	2	0	2	0	0.00493743	0	7	0	1.16581773
chr2	231267663	231314338	46676	1	0	2	0	0.01053319	0	6	0	0.91219088
chr2	231314338	231338227	23890	2	0	2	0	0.00493743	0	7	0	1.16581773
chr2	231338227	231444802	106576	4	0	2	0	0.00221948	0	8	0	1.44210395
chr2	231444802	231820231	375430	9	0	2	0	0.00493743	0	7	0	1.16581773
chr2	231820231	232123769	303539	10	0	2	0	0.00221948	0	8	0	1.44210395
chr2	232123769	232199699	75931	2	0	2	0	0.00493743	0	7	0	1.16581773
chr2	232199699	232261932	62234	3	0	2	0	0.00221948	0	8	0	1.44210395
chr2	232261932	232291533	29602	2	0	2	0	9.54E-04	0	9	0	1.74076927
chr2	232291533	232458023	166491	4	0	2	0	0.00221948	0	8	0	1.44210395
chr2	232458023	232597102	139080	6	1	2	0.30102999	9.54E-04	0	9	0	1.74076927
chr2	232597102	232627921	30820	1	0	2	0	9.54E-04	0	9	0	1.74076927
chr2	232627921	232627980	60	1	0	3	0	0.00378107	0	9	0	1.33111237
chr2	232627980	232675889	47910	2	0	2	0	9.54E-04	0	9	0	1.74076927
chr2	232675889	232756751	80863	2	0	2	0	0.00221948	0	8	0	1.44210395
chr2	232756751	232801404	44654	2	0	2	0	9.54E-04	0	9	0	1.74076927
chr2	232801404	233001154	199751	6	0	3	0	0.00378107	0	9	0	1.33111237
chr2	233001154	233055165	54012	3	1	4	0.30102999	0.01077081	0	9	0	1.01542894
chr2	233055165	233083943	28779	2	1	4	0.30102999	0.00530919	0	10	0	1.2568129
chr2	233083943	233344536	260594	5	1	3	0.30102999	0.0079614	0	8	0	1.07548421
chr2	233344536	233346962	2427	2	1	4	0.30102999	0.01077081	0	9	0	1.01542894
chr2	233346962	233474072	127111	6	1	3	0.30102999	0.0079614	0	8	0	1.07548421
chr2	233474072	233474131	60	1	1	3	0.30102999	0.00378107	0	9	0	1.33111237
chr2	233474131	233527432	53302	2	1	3	0.30102999	0.01598258	0	7	0	0.84395715
chr2	233527432	233586348	58917	3	1	3	0.30102999	0.0079614	0	8	0	1.07548421
chr2	233586348	233638238	51891	3	1	4	0.30102999	0.02074938	0	8	0	0.79906872
chr2	233638238	233638297	60	1	1	5	0.30102999	0.04407651	0	8	0	0.58747015

chr2	233638297	233641208	2912	1	1	5	0.30102999	0.07511598	0	7	0	0.43181735
chr2	233641208	233677097	35890	1	1	4	0.30102999	0.03812622	0	7	0	0.60763643
chr2	233677097	233710442	33346	2	1	4	0.30102999	0.02074938	0	8	0	0.79906872
chr2	233710442	233785208	74767	3	1	5	0.30102999	0.04407651	0	8	0	0.58747015
chr2	233785208	233785260	53	1	1	5	0.1218695	0.04407651	1	8	0.1218695	0.58747015
chr2	233785260	233839404	54145	2	1	5	0.30102999	0.04407651	0	8	0	0.58747015
chr2	233839404	233839461	58	1	1	5	0.1218695	0.04407651	1	8	0.1218695	0.58747015
chr2	233839461	233897931	58471	2	1	5	0.30102999	0.04407651	0	8	0	0.58747015
chr2	233897931	233944109	46179	2	1	5	0.1218695	0.02473314	1	9	0.1218695	0.76806864
chr2	233944109	234087008	142900	2	1	4	0.30102999	0.02074938	0	8	0	0.79906872
chr2	234087008	234296830	209823	5	1	4	0.30102999	0.01077081	0	9	0	1.01542894
chr2	234296830	234380754	83925	2	1	4	0.30102999	0.02074938	0	8	0	0.79906872
chr2	234380754	234473239	92486	2	1	4	0.30102999	0.06713722	0	6	0	0.44141547
chr2	234473239	234546256	73018	2	1	3	0.30102999	0.03070643	0	6	0	0.63695542
chr2	234546256	234602276	56021	2	0	3	0	0.03070643	0	6	0	0.63695542
chr2	234602276	234705903	103628	4	1	3	0.30102999	0.03070643	0	6	0	0.63695542
chr2	234705903	234761518	55616	3	1	3	0.30102999	0.05670724	0	5	0	0.45545077
chr2	234761518	235178141	416624	6	1	3	0.30102999	0.10122019	0	4	0	0.30102999
chr2	235178141	235178200	60	1	1	3	0.30102999	0.05670724	0	5	0	0.45545077
chr2	235178200	235313304	135105	1	1	2	0.30102999	0.0429175	0	4	0	0.47744371
chr2	235313304	235499809	186506	3	1	1	0.30102999	0.02438896	0	3	0	0.51676182
chr2	235499809	235499868	60	1	1	2	0.30102999	0.0429175	0	4	0	0.47744371
chr2	235499868	235670827	170960	1	0	2	0	0.0429175	0	4	0	0.47744371
chr2	235670827	235670886	60	1	0	3	0	0.05670724	0	5	0	0.45545077
chr2	235670886	235875361	204476	2	0	2	0	0.1575501	0	2	0	0.1575501
chr2	235875361	235904817	29457	1	0	1	0	0.05404976	0	2	0	0.30102999
chr2	235904817	235950587	45771	1	0	1	0	0.1218695	0	1	0	0.1218695
chr2	235950587	236008523	57937	2	0	2	0	0.30102999	0	1	0	0.05404976
chr2	236008523	236114456	105934	2	0	2	0	0.08289318	0	3	0	0.30102999
chr2	236114456	236114515	60	1	1	2	0.30102999	0.0429175	0	4	0	0.47744371
chr2	236114515	236247753	133239	1	0	2	0	0.0429175	0	4	0	0.47744371
chr2	236247753	236337127	89375	2	0	2	0	0.00493743	0	7	0	1.16581773
chr2	236337127	236337186	60	1	0	2	0	9.54E-04	0	9	0	1.74076927
chr2	236337186	236429985	92800	1	0	2	0	0.00493743	0	7	0	1.16581773
chr2	236429985	236470407	40423	2	0	3	0	0.01598258	0	7	0	0.84395715
chr2	236470407	236532351	61945	1	0	3	0	0.03070643	0	6	0	0.63695542
chr2	236532351	236640344	107994	3	0	3	0	0.01598258	0	7	0	0.84395715
chr2	236640344	236795731	155388	3	0	3	0	0.03070643	0	6	0	0.63695542
chr2	236795731	236795790	60	1	0	3	0	0.0079614	0	8	0	1.07548421
chr2	236795790	236905033	109244	2	0	3	0	0.05670724	0	5	0	0.45545077
chr2	236905033	236933820	28788	2	0	3	0	0.01598258	0	7	0	0.84395715
chr2	236933820	237133520	199701	3	0	3	0	0.05670724	0	5	0	0.45545077
chr2	237133520	237165845	32326	2	0	3	0	0.01598258	0	7	0	0.84395715
chr2	237165845	237232835	66991	1	0	3	0	0.03070643	0	6	0	0.63695542
chr2	237232835	237276063	43229	2	0	3	0	0.05670724	0	5	0	0.45545077
chr2	237276063	237422530	146468	5	0	3	0	0.03070643	0	6	0	0.63695542
chr2	237422530	237422589	60	1	0	4	0	0.03812622	0	7	0	0.60763643
chr2	237422589	237490861	68273	1	0	4	0	0.06713722	0	6	0	0.44141547
chr2	237490861	237795183	304323	4	0	4	0	0.03812622	0	7	0	0.60763643
chr2	237795183	237795242	60	1	0	4	0	0.01077081	1	9	0.30102999	1.01542894
chr2	237795242	237875465	80224	1	0	4	0	0.02074938	1	8	0.30102999	0.79906872
chr2	237875465	238001694	126230	2	0	3	0	0.0079614	1	8	0.30102999	1.07548421
chr2	238001694	238006797	5104	2	0	4	0	0.01077081	1	9	0.30102999	1.01542894
chr2	238006797	238216063	209267	1	0	3	0	0.00378107	1	9	0.30102999	1.33111237
chr2	238216063	238216122	60	1	0	4	0	0.01077081	1	9	0.30102999	1.01542894
chr2	238216122	238287801	71680	2	0	3	0	0.00378107	1	9	0.30102999	1.33111237
chr2	238287801	238369689	81889	2	0	3	0	0.0079614	1	8	0.30102999	1.07548421
chr2	238369689	238440095	70407	2	0	3	0	0.01598258	1	7	0.30102999	0.84395715
chr2	238440095	238482937	42843	4	0	3	0	0.0079614	1	8	0.30102999	1.07548421
chr2	238482937	238525319	42383	3	0	3	0	0.00378107	1	9	0.30102999	1.33111237
chr2	238525319	238619349	94031	2	0	3	0	0.0079614	1	8	0.30102999	1.07548421
chr2	238619349	238950601	331253	10	1	3	0.1218695	0.00378107	1	9	0.1218695	1.33111237
chr2	238950601	239185794	235194	9	1	4	0.1218695	0.01077081	1	9	0.1218695	1.01542894
chr2	239185794	239237421	51628	2	1	4	0.1218695	0.00247414	1	11	0.1218695	1.52371709
chr2	239237421	239237480	60	1	1	4	0.1218695	0.00108487	1	12	0.1218695	1.81706455
chr2	239237480	239341717	104238	3	1	4	0.1218695	0.00247414	1	11	0.1218695	1.52371709
chr2	239341717	239360005	18289	2	1	4	0.1218695	0.00108487	1	12	0.1218695	1.81706455
chr2	239360005	239665010	305006	4	0	4	0	0.00108487	1	12	0.30102999	1.81706455
chr2	239665010	239860877	195868	3	1	4	0.1218695	0.00108487	1	12	0.1218695	1.81706455
chr2	239860877	239860936	60	1	1	5	0.1218695	0.00317045	1	12	0.1218695	1.46403142
chr2	239860936	239914182	53247	1	1	4	0.1218695	0.00108487	1	12	0.1218695	1.81706455
chr2	239914182	240007003	92822	3	0	4	0	0.00108487	1	12	0.30102999	1.81706455
chr2	240007003	240007062	60	1	1	5	0.1218695	0.00317045	1	12	0.1218695	1.46403142
chr2	240007062	240105793	98732	2	0	5	0	0.00317045	1	12	0.30102999	1.46403142
chr2	240105793	240404483	298691	8	1	5	0.1218695	0.00317045	1	12	0.1218695	1.46403142
chr2	240404483	240548405	143923	3	1	4	0.1218695	0.00108487	1	12	0.1218695	1.81706455
chr2	240548405	240640049	91645	2	1	5	0.1218695	0.00317045	1	12	0.1218695	1.46403142
chr2	240640049	240880626	240578	4	1	4	0.1218695	0.00108487	1	12	0.1218695	1.81706455
chr2	240880626	241070294	189669	8	1	4	0.1218695	0.00247414	1	11	0.1218695	1.52371709
chr2	241070294	241446627	376334	10	1	4	0.1218695	0.00108487	1	12	0.1218695	1.81706455
chr2	241446627	241588655	142029	3	1	4	0.1218695	0.00247414	1	11	0.1218695	1.52371709
chr2	241588655	241739081	150427	4	1	4	0.1218695	0.00530919	1	10	0.1218695	1.2568129
chr2	241739081	241811504	72424	2	1	4	0.1218695	0.01077081	1	9	0.1218695	1.01542894
chr2	241811504	241826014	14511	2	1	4	0.1218695	0.00530919	1	10	0.1218695	1.2568129
chr2	241826014	241830215	4202	2	1	4	0.1218695	0.00247414	1	11	0.1218695	1.52371709
chr2	241830215	241909646	79432	1	1	4	0.1218695	0.01077081	1	9	0.1218695	1.01542894
chr2	241909646	241959632	49987	1	1	4	0.1218695	0.02074938	1	8	0.1218695	0.79906872
chr2	241959632	242038917	79286	2	1	4	0.1218695	0.00530919	1	10	0.1218695	1.2568129
chr2	242038917	242066091	27175	3	1	4	0.1218695	0.00108487	1	12	0.1218695	1.81706455
chr2	242066091	242260435	194345	5	1	4	0.1218695	0.00247414	1	11	0.1218695	1.52371709
chr2	242260435	242405269	144835	4	1	4	0.1218695	0.00530919	1	10	0.1218695	1.2568129
chr2	242405269	242440010	34742	2	1	4	0.1218695	0.00247414	1	11	0.1218695	1.52371709
chr2	242440010	242440066	57	1	1	4	0.1218695	0.00108487	1	12	0.1218695	1.81706455
chr2	242440066	242520838	80773	4	1	4	0.1218695	0.00530919	1	10	0.1218695	1.2568129
chr2	242520838	242710466	189629	1	0	3	0	0.00378107	0	9	0	1.33111237
chr2	242710466	242800391	89926	2	0	2	0	0.00221948	0	8	0	1.44210395
chr2	242800391	242886386	85996	3	0	3	0	0.00378107	0	9	0	1.33111237
chr2	242886386	242886445	60	1	0	3	0	7.28E-04	0	11	0	1.91540774
chr2	242886445	243007300	120856	4	0	3	0	0.00170589	0	10	0	1.61091002
chr2	243007300	243007359	60	1	0	3	0	7.28E-04	0	11	0	1.91540774
chr2	243007359	243041364	34006	1	0	3	0	0.00170589	0	10	0	1.61091002
chr3	93949	270649	176701	4	0	2	0	0.30102999	5	1	1.60515106	0.05404976
chr3	270649	270708	60	1	1	2	0.00478973	0.30102999	5	1	1.02643191	0.05404976
chr3	270708	451933	181226	4	1	0	0.00478973	0	5	1	1.02643191	0.30102999
chr3	451933	451992	60	1	1	3	0.00478973	0.30102999	5	2	1.02643191	0.08289318
chr3	451992	633449	181458	2	1	3	0.00478973	0.51676182	5	1	1.02643191	0.02438896
chr3	633449	726645	93197	2	1	3	0.00478973	0.30102999	5	2	1.02643191	0.08289318
chr3	726645	726704	60	1	2	3	0.02162467	0.17593012	5	3	0.68214471	0.17593012
chr3	726704	1125700	398997	5	1	3	0.00478973	0.51676182	5	1	1.02643191	0.02438896
chr3	1125700	1197682	71983	2	2	3	0.02162467	0.30102999	5	2	0.68214471	0.08289318
chr3	1197682	1255089	57408	1	2	3	0.02162467	0.51676182	5	1	0.68214471	0.02438896
chr3	1255089	1255148	60	1	2	3	0.02162467	0.30102999	5	2	0.68214471	0.08289318
chr3	1255148	1301859	46712	1	1	3	0.00478973	0.30102999	5	2	1.02643191	0.08289318
chr3	1301859	1337273	35415	1	1	3	0.01091641	0.30102999	4	2	0.76005302	0.08289318
chr3	1337273	1337332	60	1	1	3	0.01091641	0.10122019	4	4	0.76005302	0.30102999
chr3	1337332	1558505	221174	4	1	3	0.01091641	0.30102999	4	2	0.76005302	0.08289318
chr3	1558505	1822272	263768	5	1	3	0.01091641	0.17593012	4	3	0.76005302	0.17593012
chr3	1822272	1822331	60	1	1	3	0.01091641	0.10122019	4	4	0.76005302	0.30102999
chr3	1822331	1922459	100129	1	1	3	0.01091641	0.17593012	4	3	0.76005302	0.17593012
chr3	1922459	1922518	60	1	1	3	0.01091641	0.05670724	4	5	0.76005302	0.45545077
chr3	1922518	2262633	340116	5	1	3	0.01091641	0.10122019	4	4	0.76005302	0.30102999
chr3	2262633	2262692	60	1	1	4	0.01091641	0.11390336	4	5	0.76005302	0.30102999
chr3	2262692	2327753	65062	1	1	4	0.01091641	0.18734596	4	4	0.76005302	0.18734596
chr3	2327753	2358583	30831	2	1	4	0.01091641	0.11390336	4	5	0.76005302	0.30102999

chr3	2358583	2417896	59314	2	1	4	0.01091641	0.06713722	4	6	0.76005302	0.44141547
chr3	2417896	2449418	31523	1	1	4	0.01091641	0.11390336	4	5	0.76005302	0.30102999
chr3	2449418	2539073	89656	2	1	3	0.01091641	0.05670724	4	5	0.76005302	0.45545077
chr3	2539073	2685713	146641	3	1	3	0.01091641	0.10122019	4	4	0.76005302	0.30102999
chr3	2685713	2784019	98307	3	1	3	0.01091641	0.05670724	4	5	0.76005302	0.45545077
chr3	2784019	2784078	60	1	1	3	0.01091641	0.01598258	4	7	0.76005302	0.84395715
chr3	2784078	2962570	178493	3	1	3	0.01091641	0.03070643	4	6	0.76005302	0.63695542
chr3	2962570	3212936	250367	5	1	3	0.01091641	0.01598258	4	7	0.76005302	0.84395715
chr3	3212936	3260882	47947	1	1	3	0.01091641	0.03070643	4	6	0.76005302	0.63695542
chr3	3260882	3260941	60	1	1	4	0.01091641	0.06713722	4	6	0.76005302	0.44141547
chr3	3260941	3316827	55887	1	1	2	0.01091641	0.0429175	4	4	0.76005302	0.47744371
chr3	3316827	3316886	60	1	2	2	0.0429175	0.02162467	4	5	0.47744371	0.68214471
chr3	3316886	3490879	173994	2	2	2	0.0429175	0.0429175	4	4	0.47744371	0.47744371
chr3	3490879	3490938	60	1	2	2	0.02162467	0.02162467	5	5	0.68214471	0.68214471
chr3	3490938	3560835	69898	1	1	2	0.01091641	0.02162467	4	5	0.76005302	0.68214471
chr3	3560835	3653346	92512	2	1	2	0.01091641	0.01053319	4	6	0.76005302	0.91219088
chr3	3653346	3653405	60	1	2	2	0.02162467	0.01053319	5	6	0.68214471	0.91219088
chr3	3653405	3754545	101141	1	1	2	0.01091641	0.08289318	4	3	0.76005302	0.30102999
chr3	3754545	3837892	83348	2	1	2	0.00478973	0.08289318	5	3	1.02643191	0.30102999
chr3	3837892	3887161	49270	2	2	3	0.02162467	0.10122019	5	4	0.68214471	0.30102999
chr3	3887161	3969008	81848	2	2	2	0.02162467	0.0429175	5	4	0.68214471	0.47744371
chr3	3969008	4027733	58726	2	2	3	0.02162467	0.10122019	5	4	0.68214471	0.30102999
chr3	4027733	4273103	245371	5	2	2	0.02162467	0.0429175	5	4	0.68214471	0.47744371
chr3	4273103	4273162	60	1	2	2	0.01053319	0.0429175	6	4	0.91219088	0.47744371
chr3	4273162	4356005	82844	2	2	1	0.02162467	0.02438896	5	3	0.68214471	0.51676182
chr3	4356005	4356052	48	1	2	2	0.02162467	0.0429175	5	4	0.68214471	0.47744371
chr3	4356052	4475351	119300	2	2	1	0.02162467	0.05404976	5	2	0.68214471	0.30102999
chr3	4475351	4475410	60	1	2	1	0.02162467	0.01091641	5	4	0.68214471	0.76005302
chr3	4475410	4562627	87218	2	2	1	0.02162467	0.05404976	5	2	0.68214471	0.30102999
chr3	4562627	5179888	617262	12	2	1	0.01053319	0.05404976	6	2	0.91219088	0.30102999
chr3	5179888	5179947	60	1	3	1	0.03070643	0.05404976	6	2	0.63695542	0.30102999
chr3	5179947	5218766	38820	1	1	1	0.00204627	0.05404976	6	2	1.31360226	0.30102999
chr3	5218766	5285429	66664	1	1	0	0.00204627	0	6	2	1.31360226	0.61140001
chr3	5285429	5427855	142427	1	1	0	0.00204627	0	6	1	1.31360226	0.30102999
chr3	5427855	5695954	268100	1	1	0	0.00478973	0	5	1	1.02643191	0.30102999
chr3	5695954	6063048	367095	2	1	0	0.00478973	0	5	0	1.02643191	0
chr3	6063048	6063107	60	1	3	0	0.05670724	0	5	1	0.45545077	0.30102999
chr3	6063107	6274495	211389	1	1	0	0.00478973	0	5	1	1.02643191	0.30102999
chr3	6274495	6517841	243347	2	1	0	0.00478973	0	5	2	1.02643191	0.61140001
chr3	6517841	6517900	60	1	1	2	0.00478973	0.1575501	5	2	1.02643191	0.1575501
chr3	6517900	6665884	147985	2	1	2	0.01091641	0.1575501	4	2	0.76005302	0.1575501
chr3	6665884	6665943	60	1	1	2	0.00478973	0.08289318	5	3	1.02643191	0.30102999
chr3	6665943	6719025	53083	1	1	2	0.01091641	0.08289318	4	3	0.76005302	0.30102999
chr3	6719025	6858404	139380	2	1	2	0.01091641	0.02162467	4	5	0.76005302	0.68214471
chr3	6858404	6858463	60	1	2	2	0.01053319	0.01053319	6	6	0.91219088	0.91219088
chr3	6858463	6969680	111218	2	1	2	0.00478973	0.02162467	5	5	1.02643191	0.68214471
chr3	6969680	7028183	58504	2	2	2	0.01053319	0.00493743	6	7	0.91219088	1.16581773
chr3	7028183	7189083	160901	3	1	2	0.00478973	0.01053319	5	6	1.02643191	0.91219088
chr3	7189083	7222618	33536	2	3	2	0.03070643	0.01053319	6	6	0.63695542	0.91219088
chr3	7222618	7281688	59071	1	3	2	0.05670724	0.01053319	5	6	0.45545077	0.91219088
chr3	7281688	7281747	60	1	3	3	0.05670724	0.01598258	5	7	0.45545077	0.84395715
chr3	7281747	7403750	122004	3	2	3	0.02162467	0.01598258	5	7	0.68214471	0.84395715
chr3	7403750	7442823	39074	1	2	2	0.02162467	0.00493743	5	7	0.68214471	1.16581773
chr3	7442823	7484425	41603	2	3	2	0.03070643	0.00493743	6	7	0.63695542	1.16581773
chr3	7484425	7484484	60	1	3	3	0.03070643	0.01598258	6	7	0.63695542	0.84395715
chr3	7484484	7591304	106821	2	3	2	0.03070643	0.01053319	6	6	0.63695542	0.91219088
chr3	7591304	7674343	83040	3	3	2	0.03070643	0.00493743	6	7	0.63695542	1.16581773
chr3	7674343	7747898	73556	2	3	2	0.05670724	0.00493743	5	7	0.45545077	1.16581773
chr3	7747898	7845117	97220	2	3	2	0.05670724	0.01053319	5	6	0.45545077	0.91219088
chr3	7845117	7960638	115522	2	3	2	0.03070643	0.00493743	6	7	0.63695542	1.16581773
chr3	7960638	8141335	180698	1	3	2	0.03070643	0.01053319	6	6	0.63695542	0.91219088
chr3	8141335	8141394	60	1	4	2	0.06713722	0.01053319	6	6	0.44141547	0.91219088
chr3	8141394	8325115	183722	2	4	2	0.11390336	0.0429175	5	4	0.30102999	0.47744371
chr3	8325115	8325174	60	1	4	2	0.06713722	0.0429175	6	4	0.44141547	0.47744371
chr3	8325174	8466006	140833	2	4	2	0.06713722	0.08289318	6	3	0.44141547	0.30102999
chr3	8466006	8527127	61122	1	4	2	0.06713722	0.1575501	6	2	0.44141547	0.1575501
chr3	8527127	8615225	88099	3	4	2	0.06713722	0.08289318	6	3	0.44141547	0.30102999
chr3	8615225	8681446	66222	1	3	1	0.03070643	0.02438896	6	3	0.63695542	0.51676182
chr3	8681446	8749407	67962	1	3	1	0.03070643	0.05404976	6	2	0.63695542	0.30102999
chr3	8749407	8881364	131958	2	3	1	0.05670724	0.05404976	5	2	0.45545077	0.30102999
chr3	8881364	8945209	63846	2	3	1	0.03070643	0.05404976	6	2	0.63695542	0.30102999
chr3	8945209	9211364	266156	5	3	1	0.05670724	0.05404976	5	2	0.45545077	0.30102999
chr3	9211364	9273789	62426	2	3	1	0.05670724	0.02438896	5	3	0.45545077	0.51676182
chr3	9273789	9273848	60	1	4	1	0.03812622	0.02438896	7	3	0.60763643	0.51676182
chr3	9273848	9422487	148640	2	3	1	0.03070643	0.02438896	6	3	0.63695542	0.51676182
chr3	9422487	9475984	53498	2	3	1	0.03070643	0.01091641	6	4	0.63695542	0.76005302
chr3	9475984	9532384	56401	1	3	1	0.05670724	0.02438896	5	3	0.45545077	0.51676182
chr3	9532384	9532443	60	1	3	1	0.05670724	0.01091641	5	4	0.45545077	0.76005302
chr3	9532443	9576330	43888	1	3	1	0.05670724	0.02438896	5	3	0.45545077	0.51676182
chr3	9576330	9616619	40290	2	4	1	0.06713722	0.02438896	6	3	0.44141547	0.51676182
chr3	9616619	9695981	79363	2	4	1	0.06713722	0.01091641	6	4	0.44141547	0.76005302
chr3	9695981	9845627	149647	5	4	1	0.06713722	0.00478973	6	5	0.44141547	1.02643191
chr3	9845627	10185707	340081	9	3	0	0.03070643	0	6	5	0.63695542	1.60515106
chr3	10185707	10185766	60	1	3	1	0.03070643	0.00478973	6	5	0.63695542	1.02643191
chr3	10185766	10236010	50245	1	3	1	0.03070643	0.01091641	6	4	0.63695542	0.76005302
chr3	10236010	10315736	79727	2	3	1	0.03070643	0.02438896	6	3	0.63695542	0.51676182
chr3	10315736	10331281	15546	3	3	1	0.01598258	0.02438896	7	3	0.84395715	0.51676182
chr3	10331281	10562542	231262	7	2	1	0.01053319	0.02438896	6	3	0.91219088	0.51676182
chr3	10562542	10725151	162610	3	2	1	0.02162467	0.02438896	5	3	0.68214471	0.51676182
chr3	10725151	10725210	60	1	2	1	0.00493743	0.02438896	7	3	1.16581773	0.51676182
chr3	10725210	10823718	98509	1	2	1	0.01053319	0.02438896	6	3	0.91219088	0.51676182
chr3	10823718	11207076	383359	7	2	1	0.02162467	0.02438896	5	3	0.68214471	0.51676182
chr3	11207076	11245825	38750	2	2	1	0.02162467	0.01091641	5	4	0.68214471	0.76005302
chr3	11245825	11349112	103288	4	2	1	0.0429175	0.01091641	4	4	0.47744371	0.76005302
chr3	11349112	11421431	72320	2	2	1	0.0429175	0.02438896	4	3	0.47744371	0.51676182
chr3	11421431	11569918	148488	4	2	1	0.0429175	0.00478973	4	5	0.47744371	1.02643191
chr3	11569918	11633378	63461	4	2	2	0.0429175	0.02162467	4	5	0.47744371	0.68214471
chr3	11633378	11633437	60	1	2	2	0.02162467	0.01053319	5	6	0.68214471	0.91219088
chr3	11633437	11765281	131845	2	2	2	0.02162467	0.0429175	5	4	0.68214471	0.47744371
chr3	11765281	11848995	83715	2	2	2	0.02162467	0.01053319	5	6	0.68214471	0.91219088
chr3	11848995	12200026	351032	10	2	3	0.02162467	0.03070643	5	6	0.68214471	0.63695542
chr3	12200026	12306916	106891	2	2	2	0.02162467	0.01053319	5	6	0.68214471	0.91219088
chr3	12306916	12393173	86258	3	2	3	0.02162467	0.03070643	5	6	0.68214471	0.63695542
chr3	12393173	12620571	227399	6	2	2	0.02162467	0.02162467	5	5	0.68214471	0.68214471
chr3	12620571	12684054	63484	2	2	2	0.0429175	0.02162467	4	5	0.47744371	0.68214471
chr3	12684054	12775771	91718	3	2	2	0.02162467	0.02162467	5	5	0.68214471	0.68214471
chr3	12775771	12851413	75643	1	1	2	0.00478973	0.0429175	5	4	1.02643191	0.47744371
chr3	12851413	12942015	90603	1	1	2	0.01091641	0.0429175	4	4	0.76005302	0.47744371
chr3	12942015	13038766	96752	3	1	2	0.01091641	0.02162467	4	5	0.76005302	0.68214471
chr3	13038766	13278630	239865	4	1	2	0.00478973	0.02162467	5	5	1.02643191	0.68214471
chr3	13278630	13278689	60	1	1	4	0.00478973	0.06713722	5	6	1.02643191	0.44141547
chr3	13278689	13358885	80197	1	1	3	0.00478973	0.03070643	5	6	1.02643191	0.63695542
chr3	13358885	13421481	62597	2	1	2	0.00478973	0.01053319	5	6	1.02643191	0.91219088
chr3	13421481	13557154	135674	4	1	3	0.00478973	0.03070643	5	6	1.02643191	0.63695542
chr3	13557154	13595977	38824	2	1	3	0.00478973	0.01598258	5	7	1.02643191	0.84395715
chr3	13595977	13632853	36877	1	1	3	0.01091641	0.01598258	4	7	0.76005302	0.84395715
chr3	13632853	13757726	124874	3	1	4	0.01091641	0.03812622	4	7	0.76005302	0.60763643
chr3	13757726	13859662	101937	2	1	4	0.01091641	0.02074938	4	8	0.76005302	0.79906872
chr3	13859662	13860301	640	2	1	4	0.01091641	0.01077081	4	9	0.76005302	1.01542894
chr3	13860301	14021597	161297	4	1	4	0.01091641	0.03812622	4	7	0.76005302	0.60763643
chr3	14021597	14021656	60	1	1	4	0.00478973	0.02074938	5	8	1.02643191	0.79906872
chr3	14021656	14107503	85848	2	1	4	0.01091641	0.02074938	4	8	0.76005302	0.79906872
chr3	14107503	14163080	55578	1	1	4	0.01091641	0.03812622	4	7	0.76005302	0.60763643
chr3	14163080	14220645	57566	7	1	4	0.00478973	0.03812622	5	7	1.02643191	0.60763643

chr3	14220645	14236174	15530	2	1	4	0.00478973	0.02074938	5	8	1.02643191	0.79906872
chr3	14236174	14756827	520654	12	1	4	0.00478973	0.03812622	5	7	1.02643191	0.60763643
chr3	14756827	14833510	76684	2	1	4	0.01091641	0.03812622	4	7	0.76005302	0.60763643
chr3	14833510	14938686	105177	3	1	4	0.00478973	0.03812622	5	7	1.02643191	0.60763643
chr3	14938686	15007880	69195	3	2	4	0.02162467	0.03812622	5	7	0.68214471	0.60763643
chr3	15007880	15113408	105529	3	2	4	0.0429175	0.03812622	4	7	0.47744371	0.60763643
chr3	15113408	15126540	13133	2	2	4	0.02162467	0.03812622	5	7	0.68214471	0.60763643
chr3	15126540	15160058	33519	1	2	4	0.0429175	0.03812622	4	7	0.47744371	0.60763643
chr3	15160058	15218245	58188	1	2	4	0.0429175	0.06713722	4	6	0.47744371	0.44141547
chr3	15218245	15218304	60	1	2	4	0.02162467	0.06713722	5	6	0.68214471	0.44141547
chr3	15218304	15292837	74534	2	2	4	0.0429175	0.06713722	4	6	0.47744371	0.44141547
chr3	15292837	15410270	117434	5	2	4	0.02162467	0.06713722	5	6	0.68214471	0.44141547
chr3	15410270	15410329	60	1	2	4	0.02162467	0.03812622	5	7	0.68214471	0.60763643
chr3	15410329	15541870	131542	4	2	4	0.02162467	0.11390336	5	5	0.68214471	0.30102999
chr3	15541870	15577177	35308	1	2	4	0.02162467	0.18734596	5	4	0.68214471	0.18734596
chr3	15577177	15610035	32859	2	2	4	0.01053319	0.18734596	6	4	0.91219088	0.18734596
chr3	15610035	15643605	33571	1	2	4	0.02162467	0.18734596	5	4	0.68214471	0.18734596
chr3	15643605	15683479	39875	3	2	4	0.02162467	0.11390336	5	5	0.68214471	0.30102999
chr3	15683479	15806193	122715	2	2	4	0.02162467	0.18734596	5	4	0.68214471	0.18734596
chr3	15806193	15930426	124234	4	2	3	0.02162467	0.17593012	5	3	0.68214471	0.17593012
chr3	15930426	15989846	59421	2	2	3	0.02162467	0.05670724	5	5	0.68214471	0.45545077
chr3	15989846	15989905	60	1	2	4	0.02162467	0.11390336	5	5	0.68214471	0.30102999
chr3	15989905	16046188	56284	1	2	4	0.02162467	0.18734596	5	4	0.68214471	0.18734596
chr3	16046188	16136229	90042	1	2	4	0.0429175	0.18734596	4	4	0.47744371	0.18734596
chr3	16136229	16206839	70611	2	2	4	0.0429175	0.11390336	4	5	0.47744371	0.30102999
chr3	16206839	16206898	60	1	2	4	0.0429175	0.06713722	4	6	0.47744371	0.44141547
chr3	16206898	16322409	115512	3	2	4	0.0429175	0.11390336	4	5	0.47744371	0.30102999
chr3	16322409	16336399	13991	2	2	4	0.0429175	0.03812622	4	7	0.47744371	0.60763643
chr3	16336399	16372299	35901	1	2	3	0.08289318	0.01598258	3	7	0.30102999	0.84395715
chr3	16372299	16447117	74819	2	2	3	0.1575501	0.01598258	2	7	0.1575501	0.84395715
chr3	16447117	16447176	60	1	2	4	0.08289318	0.03812622	3	7	0.30102999	0.60763643
chr3	16447176	16508412	61237	1	2	3	0.08289318	0.01598258	3	7	0.30102999	0.84395715
chr3	16508412	16567573	59162	1	2	3	0.1575501	0.01598258	2	7	0.1575501	0.84395715
chr3	16567573	16633512	65940	2	2	4	0.1575501	0.03812622	2	7	0.1575501	0.60763643
chr3	16633512	16874601	241090	4	2	4	0.08289318	0.03812622	3	7	0.30102999	0.60763643
chr3	16874601	16874660	60	1	2	4	0.0429175	0.03812622	4	7	0.47744371	0.60763643
chr3	16874660	16955606	80947	2	2	4	0.08289318	0.06713722	3	6	0.30102999	0.44141547
chr3	16955606	16955665	60	1	2	4	0.08289318	0.03812622	3	7	0.30102999	0.60763643
chr3	16955665	17052125	96461	2	2	4	0.08289318	0.06713722	3	6	0.30102999	0.44141547
chr3	17052125	17122660	70536	1	2	4	0.08289318	0.11390336	3	5	0.30102999	0.30102999
chr3	17122660	17188340	65681	3	2	4	0.08289318	0.06713722	3	6	0.30102999	0.44141547
chr3	17188340	17300010	111671	2	2	4	0.08289318	0.11390336	3	5	0.30102999	0.30102999
chr3	17300010	17300069	60	1	2	4	0.08289318	0.06713722	3	6	0.30102999	0.44141547
chr3	17300069	17338295	38227	1	2	4	0.08289318	0.11390336	3	5	0.30102999	0.30102999
chr3	17338295	17372773	34479	1	2	4	0.1575501	0.11390336	2	5	0.1575501	0.30102999
chr3	17372773	17372832	60	1	2	4	0.1575501	0.06713722	2	6	0.1575501	0.44141547
chr3	17372832	17425472	52641	1	1	3	0.1218695	0.03070643	1	6	0.1218695	0.63695542
chr3	17425472	17425531	60	1	1	3	0.05404976	0.03070643	2	6	0.30102999	0.63695542
chr3	17425531	17550024	124494	2	1	3	0.1218695	0.03070643	1	6	0.1218695	0.63695542
chr3	17550024	17780990	230967	5	1	3	0.1218695	0.01598258	1	7	0.1218695	0.84395715
chr3	17780990	17981748	200759	2	1	3	0.30102999	0.01598258	0	7	0	0.84395715
chr3	17981748	17981807	60	1	1	3	0.1218695	0.01598258	1	7	0.1218695	0.84395715
chr3	17981807	18119539	137733	1	1	3	0.30102999	0.05670724	0	5	0	0.45545077
chr3	18119539	18181903	62365	1	1	2	0.30102999	0.0429175	0	4	0	0.47744371
chr3	18181903	18402001	220099	2	0	2	0	0.08289318	0	3	0	0.30102999
chr3	18402001	18608409	206409	3	0	4	0	0.18734596	0	4	0	0.18734596
chr3	18608409	18805698	197290	1	0	2	0	0.1575501	0	2	0	0.1575501
chr3	18805698	18922247	116550	1	0	2	0	0.30102999	0	1	0	0.05404976
chr3	18922247	19056029	133783	3	0	2	0	0.1575501	0	2	0	0.1575501
chr3	19056029	19190174	134146	2	0	3	0	0.01598258	1	7	0.30102999	0.84395715
chr3	19190174	19219352	29179	2	0	4	0	0.02074938	1	8	0.30102999	0.79906872
chr3	19219352	19219411	60	1	1	5	0.1218695	0.04407651	1	8	0.1218695	0.58747015
chr3	19219411	19295386	75976	1	1	4	0.1218695	0.02074938	1	8	0.1218695	0.79906872
chr3	19295386	19431982	136597	3	0	4	0	0.02074938	1	8	0.30102999	0.79906872
chr3	19431982	19432041	60	1	1	4	0.1218695	0.02074938	1	8	0.1218695	0.79906872
chr3	19432041	19556803	124763	2	1	4	0.30102999	0.02074938	0	8	0	0.79906872
chr3	19556803	19556862	60	1	1	4	0.1218695	0.02074938	1	8	0.1218695	0.79906872
chr3	19556862	19940924	384063	4	1	3	0.1218695	0.03070643	1	6	0.1218695	0.63695542
chr3	19940924	19992271	51348	2	1	3	0.1218695	0.01598258	1	7	0.1218695	0.84395715
chr3	19992271	20214132	221862	9	1	4	0.1218695	0.03812622	1	7	0.1218695	0.60763643
chr3	20214132	20321478	107347	2	1	2	0.30102999	0.01053319	0	6	0	0.91219088
chr3	20321478	20452995	131518	1	0	2	0	0.02162467	0	5	0	0.68214471
chr3	20452995	20595889	142895	1	0	1	0	0.01091641	0	4	0	0.76005302
chr3	20595889	20742252	146364	2	1	1	0.1218695	0.00478973	1	5	0.1218695	1.02643191
chr3	20742252	20972974	230723	3	1	3	0.1218695	0.03070643	1	6	0.1218695	0.63695542
chr3	20972974	20973033	60	1	1	4	0.1218695	0.06713722	1	6	0.1218695	0.44141547
chr3	20973033	21105079	132047	1	1	3	0.1218695	0.17593012	1	3	0.1218695	0.17593012
chr3	21105079	21105138	60	1	1	3	0.05404976	0.17593012	2	3	0.30102999	0.17593012
chr3	21105138	21176822	71685	1	0	3	0	0.17593012	1	3	0.30102999	0.17593012
chr3	21176822	21462875	286054	2	0	3	0	0.30102999	0	2	0	0.08289318
chr3	21462875	21462934	60	1	0	3	0	0.17593012	1	3	0.30102999	0.17593012
chr3	21462934	21505542	42609	1	0	3	0	0.17593012	0	3	0	0.17593012
chr3	21505542	21552397	46856	2	1	3	0.30102999	0.17593012	0	3	0	0.17593012
chr3	21552397	21552452	56	1	2	3	0.1575501	0.10122019	2	4	0.1575501	0.30102999
chr3	21552452	21974264	421813	8	2	3	0.30102999	0.10122019	1	4	0.05404976	0.30102999
chr3	21974264	22035741	61478	1	1	3	0.1218695	0.10122019	1	4	0.1218695	0.30102999
chr3	22035741	22035800	60	1	1	4	0.1218695	0.18734596	1	4	0.1218695	0.18734596
chr3	22035800	22197035	161236	2	1	3	0.1218695	0.10122019	1	4	0.1218695	0.30102999
chr3	22197035	22409736	212702	3	1	4	0.1218695	0.18734596	1	4	0.1218695	0.18734596
chr3	22409736	22466137	56402	2	1	4	0.05404976	0.18734596	2	4	0.30102999	0.18734596
chr3	22466137	22466196	60	1	2	4	0.08289318	0.18734596	3	4	0.30102999	0.18734596
chr3	22466196	22550728	84533	2	2	4	0.1575501	0.18734596	2	4	0.1575501	0.18734596
chr3	22550728	22550787	60	1	2	4	0.1575501	0.11390336	2	5	0.1575501	0.30102999
chr3	22550787	22583813	33027	1	2	4	0.1575501	0.18734596	2	4	0.1575501	0.18734596
chr3	22583813	22646787	62975	1	1	4	0.05404976	0.30102999	2	3	0.30102999	0.10122019
chr3	22646787	22769245	122459	1	1	3	0.05404976	0.17593012	2	3	0.30102999	0.17593012
chr3	22769245	22769304	60	1	1	3	0.05404976	0.10122019	2	4	0.30102999	0.30102999
chr3	22769304	23035194	265891	2	1	3	0.05404976	0.17593012	2	3	0.30102999	0.17593012
chr3	23035194	23035253	60	1	1	4	0.02438896	0.30102999	3	3	0.51676182	0.10122019
chr3	23035253	23250273	215021	3	1	2	0.05404976	0.1575501	2	2	0.30102999	0.1575501
chr3	23250273	23250327	55	1	1	4	0.05404976	0.30102999	2	3	0.30102999	0.10122019
chr3	23250327	23336511	86185	2	1	4	0.05404976	0.47744371	2	2	0.30102999	0.0429175
chr3	23336511	23416017	79507	2	1	4	0.1218695	0.47744371	1	2	0.1218695	0.0429175
chr3	23416017	23631581	215565	6	1	4	0.05404976	0.47744371	2	2	0.30102999	0.0429175
chr3	23631581	23883684	252104	4	1	3	0.05404976	0.30102999	2	2	0.30102999	0.08289318
chr3	23883684	23961532	77849	2	1	2	0.05404976	0.1575501	2	2	0.30102999	0.1575501
chr3	23961532	24232078	270547	5	1	4	0.05404976	0.47744371	2	2	0.30102999	0.0429175
chr3	24232078	24270511	38434	1	1	3	0.05404976	0.30102999	2	2	0.30102999	0.08289318
chr3	24270511	24336498	65988	1	0	3	0	0.30102999	1	2	0.30102999	0.08289318
chr3	24336498	24378779	42282	2	0	4	0	0.47744371	1	2	0.30102999	0.0429175
chr3	24378779	24378831	53	1	0	4	0	0.30102999	1	3	0.30102999	0.10122019
chr3	24378831	24438932	60102	1	0	4	0	0.47744371	1	2	0.30102999	0.0429175
chr3	24438932	24488613	49682	1	0	3	0	0.30102999	1	2	0.30102999	0.08289318
chr3	24488613	24589672	101060	2	1	3	0.1218695	0.30102999	1	2	0.1218695	0.08289318
chr3	24589672	24746895	157224	2	1	3	0.1218695	0.17593012	1	3	0.1218695	0.17593012
chr3	24746895	24901071	154177	2	1	4	0.1218695	0.11390336	1	5	0.1218695	0.30102999
chr3	24901071	24955349	54279	2	1	4	0.1218695	0.06713722	1	6	0.1218695	0.44141547
chr3	24955349	24955408	60	1	2	4	0.30102999	0.06713722	1	6	0.05404976	0.44141547
chr3	24955408	25107049	151642	2	2	4	0.30102999	0.11390336	1	5	0.05404976	0.30102999
chr3	25107049	25219853	112805	3	2	4	0.30102999	0.06713722	1	6	0.05404976	0.44141547
chr3	25219853	25335203	115351	4	2	5	0.30102999	0.12309572	1	6	0.05404976	0.30102999
chr3	25335203	25760735	425533	11	2	4	0.30102999	0.11390336	1	5	0.05404976	0.30102999
chr3	25760735	25824254	63520	2	2	4	0.30102999	0.06713722	1	6	0.05404976	0.44141547
chr3	25824254	25835866	11613	2	2	5	0.30102999	0.12309572	1	6	0.05404976	0.30102999

chr3	25835866	26146266	310401	2	2	5	0.30102999	0.19510895	1	5	0.05404976	0.19510895
chr3	26146266	26146325	60	1	2	5	0.30102999	0.12309572	1	6	0.05404976	0.30102999
chr3	26146325	26396273	249949	1	1	4	0.1218695	0.06713722	1	6	0.1218695	0.44141547
chr3	26396273	26396332	60	1	1	5	0.1218695	0.12309572	1	6	0.1218695	0.30102999
chr3	26396332	26623695	227364	1	1	5	0.1218695	0.19510895	1	5	0.1218695	0.19510895
chr3	26623695	26807485	183791	3	1	2	0.1218695	0.08289318	1	3	0.1218695	0.30102999
chr3	26807485	26957381	149897	2	1	3	0.1218695	0.17593012	1	3	0.1218695	0.17593012
chr3	26957381	26957440	60	1	1	3	0.1218695	0.05670724	1	5	0.1218695	0.45545077
chr3	26957440	27104159	146720	1	1	2	0.1218695	0.02162467	1	5	0.1218695	0.68214471
chr3	27104159	27269941	165783	4	1	2	0.1218695	0.1575501	1	2	0.1218695	0.1575501
chr3	27269941	27326179	56239	3	1	2	0.1218695	0.0429175	1	4	0.1218695	0.47744371
chr3	27326179	27416026	89848	2	1	1	0.1218695	0.01091641	1	4	0.1218695	0.76005302
chr3	27416026	27478855	62830	3	1	1	0.1218695	0.00478973	1	5	0.1218695	1.02643191
chr3	27478855	27478914	60	1	1	1	0.1218695	8.47E-04	1	7	0.1218695	1.62048027
chr3	27478914	27639046	160133	2	1	1	0.1218695	0.00478973	1	5	0.1218695	1.02643191
chr3	27639046	27851048	212003	3	1	1	0.1218695	0.01091641	1	4	0.1218695	0.76005302
chr3	27851048	28025781	174734	2	1	1	0.1218695	0.00478973	1	5	0.1218695	1.02643191
chr3	28025781	28025840	60	1	1	1	0.1218695	8.47E-04	1	7	0.1218695	1.62048027
chr3	28025840	28252753	226914	1	1	1	0.1218695	0.00478973	1	5	0.1218695	1.02643191
chr3	28252753	28252812	60	1	2	1	0.30102999	0.00478973	1	5	0.05404976	1.02643191
chr3	28252812	28368072	115261	2	2	1	0.30102999	0.02438896	1	3	0.05404976	0.51676182
chr3	28368072	28499551	131480	5	2	1	0.61140001	0.02438896	0	3	0	0.51676182
chr3	28499551	28499610	60	1	2	1	0.61140001	0.01091641	0	4	0	0.76005302
chr3	28499610	28616503	116894	2	2	0	0.61140001	0	0	3	0	0.93173516
chr3	28616503	28616562	60	1	2	0	0.30102999	0	1	4	0.05404976	1.26272838
chr3	28616562	28700624	84063	1	2	0	0.30102999	0	1	3	0.05404976	0.93173516
chr3	28700624	28700683	60	1	3	1	0.51676182	0.02438896	1	3	0.02438896	0.51676182
chr3	28700683	28815545	114863	1	3	1	0.51676182	0.05404976	1	2	0.02438896	0.30102999
chr3	28815545	28952820	137276	2	3	1	0.51676182	0.01091641	1	4	0.02438896	0.76005302
chr3	28952820	29046584	93765	2	3	1	0.51676182	0.00204627	1	6	0.02438896	1.31360226
chr3	29046584	29114567	67984	2	3	1	0.51676182	8.47E-04	1	7	0.02438896	1.62048027
chr3	29114567	29206087	91521	2	3	3	0.51676182	0.01598258	1	7	0.02438896	0.84395715
chr3	29206087	29323063	116977	2	3	2	0.51676182	0.01053319	1	6	0.02438896	0.91219088
chr3	29323063	29405895	82833	2	3	1	0.51676182	0.00204627	1	6	0.02438896	1.31360226
chr3	29405895	29405954	60	1	3	2	0.51676182	0.01053319	1	6	0.02438896	0.91219088
chr3	29405954	29565057	159104	4	3	1	0.51676182	0.00204627	1	6	0.02438896	1.31360226
chr3	29565057	29596768	31712	2	3	3	0.51676182	0.03070643	1	6	0.02438896	0.63695542
chr3	29596768	29643160	46393	1	3	1	0.51676182	0.00204627	1	6	0.02438896	1.31360226
chr3	29643160	29692849	49690	1	2	1	0.30102999	0.00204627	1	6	0.05404976	1.31360226
chr3	29692849	29804444	111596	3	2	3	0.30102999	0.03070643	1	6	0.05404976	0.63695542
chr3	29804444	29997537	193094	4	3	3	0.30102999	0.03070643	2	6	0.08289318	0.63695542
chr3	29997537	30032709	35173	2	3	3	0.30102999	0.01598258	2	7	0.08289318	0.84395715
chr3	30032709	30226234	193526	2	3	3	0.30102999	0.03070643	2	6	0.08289318	0.63695542
chr3	30226234	30226293	60	1	3	3	0.30102999	0.0079614	2	8	0.08289318	1.07548421
chr3	30226293	30651199	424907	4	2	3	0.1575501	0.01598258	2	7	0.1575501	0.84395715
chr3	30651199	30664495	13297	2	2	3	0.1575501	0.0079614	2	8	0.1575501	1.07548421
chr3	30664495	30664793	299	2	2	3	0.1575501	0.00378107	2	9	0.1575501	1.33111237
chr3	30664793	30759894	95102	4	1	3	0.05404976	0.0079614	2	8	0.30102999	1.07548421
chr3	30759894	30795662	35769	2	1	4	0.05404976	0.02074938	2	8	0.30102999	0.79906872
chr3	30795662	30856625	60964	1	1	3	0.05404976	0.0079614	2	8	0.30102999	1.07548421
chr3	30856625	30995629	139005	3	1	3	0.05404976	0.00378107	2	9	0.30102999	1.33111237
chr3	30995629	31120841	125213	1	1	3	0.1218695	0.00378107	1	9	0.1218695	1.33111237
chr3	31120841	31120900	60	1	1	3	0.05404976	0.00378107	2	9	0.30102999	1.33111237
chr3	31120900	31656667	535768	5	1	3	0.1218695	0.00378107	1	9	0.1218695	1.33111237
chr3	31656667	31676611	19945	1	1	3	0.1218695	0.0079614	1	8	0.1218695	1.07548421
chr3	31676611	31805724	129114	2	1	3	0.1218695	0.01598258	1	7	0.1218695	0.84395715
chr3	31805724	31921241	115518	3	1	3	0.1218695	0.0079614	1	8	0.1218695	1.07548421
chr3	31921241	31921288	48	1	1	5	0.1218695	0.04407651	1	8	0.1218695	0.58747015
chr3	31921288	31966445	45158	1	1	4	0.1218695	0.02074938	1	8	0.1218695	0.79906872
chr3	31966445	32000976	34532	2	1	4	0.1218695	0.01077081	1	9	0.1218695	1.01542894
chr3	32000976	32256459	255484	5	1	3	0.1218695	0.0079614	1	8	0.1218695	1.07548421
chr3	32256459	32343447	86989	2	1	3	0.1218695	0.01598258	1	7	0.1218695	0.84395715
chr3	32343447	32398947	55501	2	1	3	0.1218695	0.0079614	1	8	0.1218695	1.07548421
chr3	32398947	32399005	59	1	1	3	0.1218695	0.00378107	1	9	0.1218695	1.33111237
chr3	32399005	32509966	110962	3	1	3	0.1218695	0.01598258	1	7	0.1218695	0.84395715
chr3	32509966	32541875	31910	2	1	4	0.1218695	0.02074938	1	8	0.1218695	0.79906872
chr3	32541875	32544431	2557	1	1	4	0.1218695	0.03812622	1	7	0.1218695	0.60763643
chr3	32544431	32544483	53	1	1	4	0.1218695	0.01077081	1	9	0.1218695	1.01542894
chr3	32544483	32576054	31572	1	1	4	0.1218695	0.02074938	1	8	0.1218695	0.79906872
chr3	32576054	32653663	77610	1	1	3	0.1218695	0.0079614	1	8	0.1218695	1.07548421
chr3	32653663	32653722	60	1	1	3	0.1218695	0.00378107	1	9	0.1218695	1.33111237
chr3	32653722	32785668	131947	2	1	3	0.1218695	0.01598258	1	7	0.1218695	0.84395715
chr3	32785668	32892815	107148	3	1	3	0.05404976	0.01598258	2	7	0.30102999	0.84395715
chr3	32892815	32995580	102766	2	1	3	0.1218695	0.01598258	1	7	0.1218695	0.84395715
chr3	32995580	32995633	54	1	1	3	0.05404976	0.01598258	2	7	0.30102999	0.84395715
chr3	32995633	33133877	138245	3	1	3	0.1218695	0.01598258	1	7	0.1218695	0.84395715
chr3	33133877	33171478	37602	2	2	3	0.1575501	0.01598258	2	7	0.1575501	0.84395715
chr3	33171478	33229045	57568	1	1	2	0.05404976	0.01053319	2	6	0.30102999	0.91219088
chr3	33229045	33382941	153897	4	1	2	0.05404976	0.02162467	2	5	0.30102999	0.68214471
chr3	33382941	33383000	60	1	1	2	0.05404976	0.00493743	2	7	0.30102999	1.16581773
chr3	33383000	33467282	84283	3	1	2	0.1218695	0.01053319	1	6	0.1218695	0.91219088
chr3	33467282	33544145	76864	1	1	2	0.1218695	0.0429175	1	4	0.1218695	0.47744371
chr3	33544145	33597849	53705	1	1	1	0.1218695	0.02438896	1	3	0.1218695	0.51676182
chr3	33597849	33639827	41979	2	1	1	0.05404976	0.01091641	2	4	0.30102999	0.76005302
chr3	33639827	33639886	60	1	1	2	0.05404976	0.0429175	2	4	0.30102999	0.47744371
chr3	33639886	33855094	215209	4	1	1	0.05404976	0.01091641	2	4	0.30102999	0.76005302
chr3	33855094	33909834	54741	2	1	1	0.05404976	0.02438896	2	3	0.30102999	0.51676182
chr3	33909834	34087138	177305	1	1	0	0.05404976	0	2	3	0.30102999	0.93173516
chr3	34087138	34316258	229121	1	1	0	0.1218695	0	1	3	0.1218695	0.93173516
chr3	34316258	34316317	60	1	1	0	0.1218695	0	1	5	0.1218695	1.60515106
chr3	34316317	34444559	128243	1	1	0	0.1218695	0	1	4	0.1218695	1.26272838
chr3	34444559	34635274	190716	2	1	0	0.1218695	0	1	5	0.1218695	1.60515106
chr3	34635274	34761306	126033	2	1	0	0.1218695	0	1	6	0.1218695	1.95986592
chr3	34761306	34761365	60	1	1	1	0.1218695	0.00204627	1	6	0.1218695	1.31360226
chr3	34761365	35028022	266658	3	1	0	0.30102999	0	0	4	0	1.26272838
chr3	35028022	35028081	60	1	1	0	0.1218695	0	1	6	0.1218695	1.95986592
chr3	35028081	35388245	360165	5	1	0	0.30102999	0	0	5	0	1.60515106
chr3	35388245	35565301	177057	1	1	0	0.30102999	0	0	4	0	1.26272838
chr3	35565301	35565360	60	1	1	0	0.1218695	0	1	5	0.1218695	1.60515106
chr3	35565360	35724826	159467	2	1	0	0.1218695	0	1	4	0.1218695	1.26272838
chr3	35724826	35756989	32164	2	1	1	0.1218695	0.00478973	1	5	0.1218695	1.02643191
chr3	35756989	35798853	41865	2	1	1	0.1218695	0.02438896	1	3	0.1218695	0.51676182
chr3	35798853	35835586	36734	2	1	2	0.1218695	0.02162467	1	5	0.1218695	0.68214471
chr3	35835586	36271361	435776	2	1	2	0.30102999	0.0429175	0	4	0	0.47744371
chr3	36271361	36271420	60	1	1	2	0.1218695	0.01053319	1	6	0.1218695	0.91219088
chr3	36271420	36545886	274467	4	1	2	0.1218695	0.02162467	1	5	0.1218695	0.68214471
chr3	36545886	36589006	43121	2	1	3	0.1218695	0.01598258	1	7	0.1218695	0.84395715
chr3	36589006	36689646	100641	1	1	2	0.1218695	0.00493743	1	7	0.1218695	1.16581773
chr3	36689646	36780626	90981	1	1	2	0.30102999	0.01053319	0	6	0	0.91219088
chr3	36780626	36836085	55460	2	1	3	0.30102999	0.03070643	0	6	0	0.63695542
chr3	36836085	36955434	119350	3	1	3	0.1218695	0.0079614	1	8	0.1218695	1.07548421
chr3	36955434	37055990	100557	3	1	3	0.1218695	0.01598258	1	7	0.1218695	0.84395715
chr3	37055990	37056049	60	1	1	3	0.1218695	0.0079614	1	8	0.1218695	1.07548421
chr3	37056049	37189508	133460	4	1	3	0.1218695	0.01598258	1	7	0.1218695	0.84395715
chr3	37189508	37221473	31966	2	1	3	0.05404976	0.01598258	2	7	0.30102999	0.84395715
chr3	37221473	37409646	188174	4	1	3	0.05404976	0.0079614	2	8	0.30102999	1.07548421
chr3	37409646	37433604	23959	2	1	3	0.05404976	0.00170589	2	10	0.30102999	1.61091002
chr3	37433604	37479563	45960	2	1	3	0.05404976	0.0079614	2	8	0.30102999	1.07548421
chr3	37479563	37479622	60	1	1	3	0.05404976	0.00170589	2	10	0.30102999	1.61091002
chr3	37479622	37665417	185796	5	1	3	0.05404976	0.00378107	2	9	0.30102999	1.33111237
chr3	37665417	38316625	651209	23	1	3	0.05404976	0.00170589	2	10	0.30102999	1.61091002
chr3	38316625	38418384	101760	4	1	4	0.05404976	0.00530919	2	10	0.30102999	1.2568129
chr3	38418384	38418443	60	1	1	4	0.05404976	0.00247414	2	11	0.30102999	1.52371709
chr3	38418443	38522873	104431	3	1	4	0.05404976	0.00530919	2	10	0.30102999	1.2568129

chr3	38522873	38615248	92376	2	1	3	0.05404976	0.00170589	2	10	0.30102999	1.61091002
chr3	38615248	38698412	83165	2	1	3	0.05404976	0.00378107	2	9	0.30102999	1.33111237
chr3	38698412	38830481	132070	3	1	2	0.05404976	9.54E-04	2	9	0.30102999	1.74076927
chr3	38830481	38830540	60	1	1	3	0.05404976	0.00378107	2	9	0.30102999	1.33111237
chr3	38830540	38891858	61319	1	1	2	0.05404976	9.54E-04	2	9	0.30102999	1.74076927
chr3	38891858	38938342	46485	2	1	2	0.05404976	3.90E-04	2	10	0.30102999	2.0620585
chr3	38938342	38938401	60	1	1	3	0.05404976	0.00170589	2	10	0.30102999	1.61091002
chr3	38938401	38995553	57153	1	1	2	0.05404976	3.90E-04	2	10	0.30102999	2.0620585
chr3	38995553	39115065	119513	2	1	1	0.05404976	4.80E-05	2	10	0.30102999	2.6588298
chr3	39115065	39374803	259739	8	1	2	0.05404976	3.90E-04	2	10	0.30102999	2.0620585
chr3	39374803	39532603	157801	3	1	1	0.05404976	0.00204627	2	6	0.30102999	1.31360226
chr3	39532603	39532662	60	1	1	2	0.05404976	0.01053319	2	6	0.30102999	0.91219088
chr3	39532662	39681962	149301	2	1	1	0.05404976	0.00204627	2	6	0.30102999	1.31360226
chr3	39681962	39682021	60	1	1	1	0.05404976	8.47E-04	2	7	0.30102999	1.62048027
chr3	39682021	39873559	191539	2	1	1	0.1218695	0.00204627	1	6	0.1218695	1.31360226
chr3	39873559	40162426	288868	6	1	0	0.1218695	0	1	4	0.1218695	1.26272838
chr3	40162426	40255918	93493	2	1	0	0.1218695	0	1	3	0.1218695	0.93173516
chr3	40255918	40255977	60	1	2	0	0.30102999	0	1	3	0.05404976	0.93173516
chr3	40255977	40352356	96380	2	1	0	0.1218695	0	1	3	0.1218695	0.93173516
chr3	40352356	40352412	57	1	1	0	0.1218695	0	1	4	0.1218695	1.26272838
chr3	40352412	40529789	177378	4	1	0	0.1218695	0	1	3	0.1218695	0.93173516
chr3	40529789	40570473	40685	3	1	0	0.1218695	0	1	4	0.1218695	1.26272838
chr3	40570473	40630279	59807	2	1	0	0.1218695	0	1	5	0.1218695	1.60515106
chr3	40630279	40759986	129708	2	1	1	0.1218695	0.00478973	1	5	0.1218695	1.02643191
chr3	40759986	41177103	417118	3	1	2	0.05404976	0.00493743	2	7	0.30102999	1.16581773
chr3	41177103	41177162	60	1	1	3	0.05404976	0.01598258	2	7	0.30102999	0.84395715
chr3	41177162	41457495	280334	6	1	1	0.1218695	0.00204627	1	6	0.1218695	1.31360226
chr3	41457495	41457554	60	1	1	3	0.1218695	0.01598258	1	7	0.1218695	0.84395715
chr3	41457554	41570251	112698	2	1	2	0.1218695	0.01053319	1	6	0.1218695	0.91219088
chr3	41570251	41614450	44200	1	1	1	0.1218695	0.00204627	1	6	0.1218695	1.31360226
chr3	41614450	41667185	52736	2	1	2	0.1218695	0.01053319	1	6	0.1218695	0.91219088
chr3	41667185	41715768	48584	2	1	3	0.1218695	0.03070643	1	6	0.1218695	0.63695542
chr3	41715768	41831015	115248	2	1	2	0.1218695	0.01053319	1	6	0.1218695	0.91219088
chr3	41831015	41831074	60	1	1	3	0.1218695	0.0079614	1	8	0.1218695	1.07548421
chr3	41831074	41927881	96808	2	1	2	0.1218695	0.01053319	1	6	0.1218695	0.91219088
chr3	41927881	41965241	37361	2	1	2	0.1218695	0.00493743	1	7	0.1218695	1.16581773
chr3	41965241	41996795	31555	2	1	4	0.1218695	0.02074938	1	8	0.1218695	0.79906872
chr3	41996795	41996854	60	1	1	4	0.05404976	0.00247414	2	11	0.30102999	1.52371709
chr3	41996854	42167061	170208	3	1	4	0.05404976	0.01077081	2	9	0.30102999	1.01542894
chr3	42167061	42167119	59	1	1	5	0.05404976	0.00317045	2	12	0.30102999	1.46403142
chr3	42167119	42226820	59702	1	1	5	0.05404976	0.00666883	2	11	0.30102999	1.20557689
chr3	42226820	42305446	78627	1	1	5	0.05404976	0.02473314	2	9	0.30102999	0.76806864
chr3	42305446	42390341	84896	1	1	4	0.05404976	0.01077081	2	9	0.30102999	1.01542894
chr3	42390341	42452015	61675	3	1	4	0.05404976	0.00530919	2	10	0.30102999	1.2568129
chr3	42452015	42452072	58	1	1	5	0.05404976	0.00666883	2	11	0.30102999	1.20557689
chr3	42452072	42533372	81301	2	1	4	0.05404976	0.00530919	2	10	0.30102999	1.2568129
chr3	42533372	42635823	102452	3	1	5	0.05404976	0.01320236	2	10	0.30102999	0.97390707
chr3	42635823	42750902	115080	4	1	5	0.05404976	0.00666883	2	11	0.30102999	1.20557689
chr3	42750902	42750961	60	1	1	6	0.05404976	0.01518174	2	11	0.30102999	0.9449689
chr3	42750961	43129680	378720	13	1	5	0.05404976	0.00666883	2	11	0.30102999	1.20557689
chr3	43129680	43249503	119824	2	1	3	0.1218695	0.00170589	1	10	0.1218695	1.61091002
chr3	43249503	43249562	60	1	1	3	0.1218695	7.28E-04	1	11	0.1218695	1.91540774
chr3	43249562	43330446	80885	1	1	3	0.1218695	0.00170589	1	10	0.1218695	1.61091002
chr3	43330446	43403101	72656	5	1	3	0.05404976	0.00170589	2	10	0.30102999	1.61091002
chr3	43403101	43448201	45101	2	1	3	0.05404976	7.28E-04	2	11	0.30102999	1.91540774
chr3	43448201	43474154	25954	2	1	3	0.05404976	2.93E-04	2	12	0.30102999	2.24559516
chr3	43474154	43474210	57	1	1	4	0.05404976	0.00108487	2	12	0.30102999	1.81706455
chr3	43474210	43552272	78063	2	1	4	0.05404976	0.00247414	2	11	0.30102999	1.52371709
chr3	43552272	43743683	191412	5	1	4	0.05404976	0.00108487	2	12	0.30102999	1.81706455
chr3	43743683	43743742	60	1	1	6	0.05404976	0.00778066	2	12	0.30102999	1.17038931
chr3	43743742	43865299	121558	2	1	5	0.05404976	0.00666883	2	11	0.30102999	1.20557689
chr3	43865299	43865358	60	1	1	7	0.05404976	0.01667721	2	12	0.30102999	0.92532268
chr3	43865358	44362626	497269	5	1	6	0.05404976	0.01518174	2	11	0.30102999	0.9449689
chr3	44362626	44426012	63387	3	1	7	0.05404976	0.03037338	2	11	0.30102999	0.73110763
chr3	44426012	44681785	255774	7	1	6	0.05404976	0.02793176	2	10	0.30102999	0.74627054
chr3	44681785	44681844	60	1	1	6	0.05404976	0.01518174	2	11	0.30102999	0.9449689
chr3	44681844	44694207	12364	1	1	5	0.05404976	0.00666883	2	11	0.30102999	1.20557689
chr3	44694207	44777967	83761	3	1	5	0.05404976	0.02473314	2	9	0.30102999	0.76806864
chr3	44777967	44904900	126934	6	1	5	0.05404976	0.01320236	2	10	0.30102999	0.97390707
chr3	44904900	44938171	33272	3	1	6	0.05404976	0.02793176	2	10	0.30102999	0.74627054
chr3	44938171	45044866	106696	4	1	7	0.05404976	0.05232577	2	10	0.30102999	0.56314362
chr3	45044866	45052868	8003	2	1	8	0.05404976	0.08923391	2	10	0.30102999	0.4167287
chr3	45052868	45102264	49397	2	1	6	0.05404976	0.02793176	2	10	0.30102999	0.74627054
chr3	45102264	45139372	37109	2	1	7	0.05404976	0.05232577	2	10	0.30102999	0.56314362
chr3	45139372	45541987	402616	8	1	6	0.05404976	0.02793176	2	10	0.30102999	0.74627054
chr3	45541987	45542040	54	1	1	6	0.05404976	0.01518174	2	11	0.30102999	0.9449689
chr3	45542040	45661623	119584	2	1	6	0.05404976	0.02793176	2	10	0.30102999	0.74627054
chr3	45661623	45688578	26956	2	1	7	0.05404976	0.05232577	2	10	0.30102999	0.56314362
chr3	45688578	45688637	60	1	1	8	0.05404976	0.05490675	2	11	0.30102999	0.55623409
chr3	45688637	45773620	84984	2	1	7	0.05404976	0.03037338	2	11	0.30102999	0.73110763
chr3	45773620	45799276	25657	1	1	6	0.05404976	0.01518174	2	11	0.30102999	0.9449689
chr3	45799276	45867117	67842	2	1	6	0.05404976	0.02793176	2	10	0.30102999	0.74627054
chr3	45867117	45879470	12354	2	1	6	0.05404976	0.01518174	2	11	0.30102999	0.9449689
chr3	45879470	45879529	60	1	1	8	0.05404976	0.03209037	2	12	0.30102999	0.72109894
chr3	45879529	45932245	52717	2	1	7	0.05404976	0.03037338	2	11	0.30102999	0.73110763
chr3	45932245	45932304	60	1	1	8	0.05404976	0.05490675	2	11	0.30102999	0.55623409
chr3	45932304	45986647	54344	2	1	7	0.05404976	0.03037338	2	11	0.30102999	0.73110763
chr3	45986647	45989683	3037	2	1	8	0.05404976	0.03209037	2	12	0.30102999	0.72109894
chr3	45989683	46033995	44313	1	1	8	0.05404976	0.05490675	2	11	0.30102999	0.55623409
chr3	46033995	46062944	28950	1	1	7	0.05404976	0.05232577	2	10	0.30102999	0.56314362
chr3	46062944	46186921	123978	1	1	7	0.1218695	0.05232577	1	10	0.1218695	0.56314362
chr3	46186921	46247594	60674	3	1	7	0.1218695	0.03037338	1	11	0.1218695	0.73110763
chr3	46247594	46303826	56233	1	1	7	0.1218695	0.05232577	1	10	0.1218695	0.56314362
chr3	46303826	46505662	201837	1	0	7	0	0.05232577	0	10	0	0.56314362
chr3	46505662	46541671	36010	2	0	7	0	0.00859896	0	13	0	1.14735594
chr3	46541671	46601487	59817	3	0	7	0	0.00859896	1	13	0.30102999	1.14735594
chr3	46601487	46684954	83468	3	0	6	0	0.00373523	1	13	0.30102999	1.42397267
chr3	46684954	46685013	60	1	0	8	0	0.00909834	1	14	0.30102999	1.13428373
chr3	46685013	46747141	62129	2	0	8	0	0.01767679	1	13	0.30102999	0.91308053
chr3	46747141	46852723	105583	3	0	8	0	0.00909834	1	14	0.30102999	1.13428373
chr3	46852723	46873458	20736	2	0	8	0	0.00188722	1	16	0.30102999	1.67551277
chr3	46873458	46873517	60	1	0	8	0	7.41E-04	1	17	0.30102999	2.00316618
chr3	46873517	46904528	31012	1	0	7	0	0.00183109	1	15	0.30102999	1.68336308
chr3	46904528	46967059	62532	1	0	7	0	0.00413023	1	14	0.30102999	1.39918918
chr3	46967059	46967115	57	1	0	8	0	0.00433294	2	15	0.61140001	1.3873209
chr3	46967115	47003272	36158	1	0	8	0	0.00909834	1	14	0.30102999	1.13428373
chr3	47003272	47003331	60	1	0	8	0	0.00433294	1	15	0.30102999	1.3873209
chr3	47003331	47084157	80827	2	0	7	0	0.00183109	1	15	0.30102999	1.68336308
chr3	47084157	47084216	60	1	0	8	0	0.00188722	1	16	0.30102999	1.67551277
chr3	47084216	47127929	43714	1	0	7	0	7.41E-04	1	16	0.30102999	2.00316618
chr3	47127929	47203633	75705	2	0	7	0	0.00183109	1	15	0.30102999	1.68336308
chr3	47203633	47282412	78780	1	0	7	0	0.00413023	1	14	0.30102999	1.39918918
chr3	47282412	47328649	46238	2	0	7	0	0.00413023	2	14	0.61140001	1.39918918
chr3	47328649	47454667	126019	6	0	9	0	0.01817691	2	14	0.61140001	0.90719478
chr3	47454667	47537275	82609	3	0	10	0	0.03344703	2	14	0.61140001	0.71353304
chr3	47537275	47578981	41707	2	0	11	0	0.0574087	2	14	0.61140001	0.5498098
chr3	47578981	47677519	98539	3	0	11	0	0.09334429	2	13	0.61140001	0.41271556
chr3	47677519	47677578	60	1	0	11	0	0.0331093	2	15	0.61140001	0.71538971
chr3	47677578	47718219	40642	2	0	10	0	0.01817691	2	15	0.61140001	0.90719478
chr3	47718219	47777534	59316	1	0	10	0	0.03344703	1	14	0.30102999	0.71353304
chr3	47777534	47803572	26039	2	0	10	0	0.01817691	1	15	0.30102999	0.90719478
chr3	47803572	47891694	88123	4	1	10	0.1218695	0.01817691	1	15	0.1218695	0.90719478
chr3	47891694	47911231	19538	1	0	10	0	0.01817691	1	15	0.30102999	0.90719478
chr3	47911231	47963287	52057	2	0	11	0	0.0331093	1	15	0.30102999	0.71538971

chr3	47963287	48002013	38727	1	0	11	0	0.0574087	1	14	0.30102999	0.5498098
chr3	48002013	48094781	92769	2	0	10	0	0.03344703	1	14	0.30102999	0.71353304
chr3	48094781	48128176	33396	2	0	10	0	0.00909834	1	16	0.30102999	1.13428373
chr3	48128176	48160737	32562	2	0	11	0	0.01767679	1	16	0.30102999	0.91308053
chr3	48160737	48200083	39347	1	0	10	0	0.01817691	1	15	0.30102999	0.90719478
chr3	48200083	48219728	19646	1	0	9	0	0.00926608	1	15	0.30102999	1.13004284
chr3	48219728	48228287	8560	2	1	9	0.1218695	0.00926608	1	15	0.1218695	1.13004284
chr3	48228287	48265559	37273	1	1	9	0.1218695	0.01817691	1	14	0.1218695	0.90719478
chr3	48265559	48335484	69926	3	1	8	0.1218695	0.01767679	1	13	0.1218695	0.91308053
chr3	48335484	48371553	36070	3	1	8	0.1218695	0.00909834	1	14	0.1218695	1.13428373
chr3	48371553	48421714	50162	1	1	8	0.1218695	0.03209037	1	12	0.1218695	0.72109894
chr3	48421714	48421773	60	1	1	8	0.1218695	0.01767679	1	13	0.1218695	0.91308053
chr3	48421773	48600890	179118	7	1	8	0.1218695	0.03209037	1	12	0.1218695	0.72109894
chr3	48600890	48603090	2201	2	1	8	0.1218695	0.01767679	1	13	0.1218695	0.91308053
chr3	48603090	48630602	27513	1	1	8	0.1218695	0.03209037	1	12	0.1218695	0.72109894
chr3	48630602	48630648	47	1	1	8	0.1218695	0.01767679	1	13	0.1218695	0.91308053
chr3	48630648	48674554	43907	4	0	8	0	0.01767679	1	13	0.30102999	0.91308053
chr3	48674554	48717333	42780	3	1	8	0.1218695	0.00909834	1	14	0.1218695	1.13428373
chr3	48717333	48725407	8075	2	1	8	0.1218695	0.01767679	1	13	0.1218695	0.91308053
chr3	48725407	48730993	5587	4	1	8	0.1218695	0.00909834	1	14	0.1218695	1.13428373
chr3	48730993	48860144	129152	3	1	8	0.1218695	0.01767679	1	13	0.1218695	0.91308053
chr3	48860144	48900155	40012	1	1	8	0.1218695	0.03209037	1	12	0.1218695	0.72109894
chr3	48900155	48933052	32898	2	1	8	0.1218695	0.01767679	1	13	0.1218695	0.91308053
chr3	48933052	48933111	60	1	1	8	0.1218695	0.00909834	1	14	0.1218695	1.13428373
chr3	48933111	48974688	41578	1	1	7	0.1218695	0.00413023	1	14	0.1218695	1.39918918
chr3	48974688	48974747	60	1	1	8	0.1218695	0.00909834	1	14	0.1218695	1.13428373
chr3	48974747	49011215	36469	1	1	7	0.1218695	0.00859896	1	13	0.1218695	1.14735594
chr3	49011215	49047095	35881	3	1	7	0.1218695	0.00183109	1	15	0.1218695	1.68336308
chr3	49047095	49092591	45497	5	1	7	0.1218695	7.41E-04	1	16	0.1218695	2.00316618
chr3	49092591	49092650	60	1	1	8	0.1218695	0.00188722	1	16	0.1218695	1.67551277
chr3	49092650	49169538	76889	4	1	7	0.1218695	7.41E-04	1	16	0.1218695	2.00316618
chr3	49169538	49169587	50	1	1	8	0.1218695	0.00188722	1	16	0.1218695	1.67551277
chr3	49169587	49199320	29734	1	1	7	0.1218695	7.41E-04	1	16	0.1218695	2.00316618
chr3	49199320	49199373	54	1	1	8	0.1218695	0.00188722	1	16	0.1218695	1.67551277
chr3	49199373	49249270	49898	3	1	7	0.1218695	7.41E-04	1	16	0.1218695	2.00316618
chr3	49249270	49274002	24733	1	1	7	0.1218695	0.00183109	1	15	0.1218695	1.68336308
chr3	49274002	49315808	41807	2	2	7	0.30102999	0.00183109	1	15	0.05404976	1.68336308
chr3	49315808	49395230	79423	5	2	7	0.30102999	7.41E-04	1	16	0.05404976	2.00316618
chr3	49395230	49421743	26514	4	2	9	0.30102999	0.00183109	1	17	0.05404976	1.68336308
chr3	49421743	49457011	35269	2	2	8	0.30102999	7.41E-04	1	17	0.05404976	2.00316618
chr3	49457011	49462789	5779	2	2	9	0.30102999	0.00183109	1	17	0.05404976	1.68336308
chr3	49462789	49513093	50305	1	2	8	0.30102999	7.41E-04	1	17	0.05404976	2.00316618
chr3	49513093	49637222	124130	3	1	8	0.1218695	7.41E-04	1	17	0.1218695	2.00316618
chr3	49637222	49673422	36201	2	1	9	0.1218695	6.86E-04	1	18	0.1218695	2.02387299
chr3	49673422	49673481	60	1	1	11	0.1218695	0.00373523	1	18	0.1218695	1.42397267
chr3	49673481	49720947	47467	1	1	10	0.1218695	0.00166733	1	18	0.1218695	1.70763027
chr3	49720947	49811521	90575	5	0	9	0	6.86E-04	1	18	0.30102999	2.02387299
chr3	49811521	49898264	86744	4	0	9	0	0.00183109	1	17	0.30102999	1.68336308
chr3	49898264	49924607	26344	2	0	8	0	7.41E-04	1	17	0.30102999	2.00316618
chr3	49924607	49932774	8168	3	0	9	0	0.00183109	1	17	0.30102999	1.68336308
chr3	49932774	49977269	44496	3	0	8	0	7.41E-04	1	17	0.30102999	2.00316618
chr3	49977269	50005791	28523	2	0	9	0	0.00183109	1	17	0.30102999	1.68336308
chr3	50005791	50085653	79863	2	0	8	0	7.41E-04	1	17	0.30102999	2.00316618
chr3	50085653	50085707	55	1	0	10	0	0.00413023	1	17	0.30102999	1.39918918
chr3	50085707	50311912	226206	7	0	8	0	0.00188722	1	16	0.30102999	1.67551277
chr3	50311912	50323584	11673	2	0	9	0	0.00433294	1	16	0.30102999	1.3873209
chr3	50323584	50339735	16152	2	0	10	0	0.00909834	1	16	0.30102999	1.13428373
chr3	50339735	50342224	2490	3	0	10	0	0.00413023	1	17	0.30102999	1.39918918
chr3	50342224	50367646	25423	4	0	9	0	0.00183109	1	17	0.30102999	1.68336308
chr3	50367646	50390376	22731	3	0	8	0	0.00433294	1	15	0.30102999	1.3873209
chr3	50390376	50408802	18427	3	0	8	0	0.00188722	1	16	0.30102999	1.67551277
chr3	50408802	50440246	31445	2	0	10	0	0.00413023	1	17	0.30102999	1.39918918
chr3	50440246	50440305	60	1	0	12	0	0.01667721	1	17	0.30102999	0.92532268
chr3	50440305	50539207	98903	2	0	11	0	0.00859896	1	17	0.30102999	1.14735594
chr3	50539207	50607825	68619	3	0	11	0	0.00373523	1	18	0.30102999	1.42397267
chr3	50607825	50665961	58137	3	0	11	0	0.00859896	1	17	0.30102999	1.14735594
chr3	50665961	50666020	60	1	0	11	0	0.00373523	1	18	0.30102999	1.42397267
chr3	50666020	50679116	13097	1	0	11	0	0.00859896	1	17	0.30102999	1.14735594
chr3	50679116	50713799	34684	2	0	11	0	0.00373523	1	18	0.30102999	1.42397267
chr3	50713799	50787294	73496	2	0	11	0	0.00859896	1	17	0.30102999	1.14735594
chr3	50787294	50824511	37218	2	0	11	0	0.00373523	1	18	0.30102999	1.42397267
chr3	50824511	50824570	60	1	0	11	0	0.00141024	1	19	0.30102999	1.7506523
chr3	50824570	50913458	88889	2	0	11	0	0.00859896	1	17	0.30102999	1.14735594
chr3	50913458	51183809	270352	5	0	11	0	0.01767679	1	16	0.30102999	0.91308053
chr3	51183809	51378155	194347	4	0	11	0	0.01767679	0	16	0	0.91308053
chr3	51378155	51378214	60	1	0	13	0	0.03037338	0	17	0	0.73110763
chr3	51378214	51413402	35189	1	0	11	0	0.00859896	0	17	0	1.14735594
chr3	51413402	51436181	22780	5	0	11	0	0.00373523	0	18	0	1.42397267
chr3	51436181	51486485	50305	2	0	11	0	0.01767679	0	16	0	0.91308053
chr3	51486485	51486544	60	1	0	11	0	0.00373523	0	18	0	1.42397267
chr3	51486544	51520629	34086	1	0	11	0	0.00859896	0	17	0	1.14735594
chr3	51520629	51613907	93279	3	0	11	0	0.01767679	0	16	0	0.91308053
chr3	51613907	51613966	60	1	0	11	0	0.00859896	0	17	0	1.14735594
chr3	51613966	51662236	48271	1	0	11	0	0.01767679	0	16	0	0.91308053
chr3	51662236	51672205	9970	1	0	11	0	0.0574087	0	14	0	0.5498098
chr3	51672205	51672253	49	1	0	12	0	0.09290028	0	14	0	0.41314172
chr3	51672253	51721479	49227	1	0	11	0	0.09334429	0	13	0	0.41271556
chr3	51721479	51743590	22112	2	0	11	0	0.0574087	0	14	0	0.5498098
chr3	51743590	51743641	52	1	0	12	0	0.0565833	0	15	0	0.55190077
chr3	51743641	51896472	152832	2	0	11	0	0.0574087	0	14	0	0.5498098
chr3	51896472	51929623	33152	2	0	12	0	0.09290028	0	14	0	0.41314172
chr3	51929623	51929673	51	1	0	12	0	0.03209037	0	16	0	0.72109894
chr3	51929673	51973337	43665	2	0	11	0	0.0331093	0	15	0	0.71538971
chr3	51973337	51978495	5159	2	0	11	0	0.01767679	0	16	0	0.91308053
chr3	51978495	52028981	50487	6	0	11	0	0.0331093	0	15	0	0.71538971
chr3	52028981	52029029	49	1	0	12	0	0.0565833	0	15	0	0.55190077
chr3	52029029	52133519	104491	2	0	12	0	0.09290028	0	14	0	0.41314172
chr3	52133519	52208487	74969	1	0	11	0	0.0574087	0	14	0	0.5498098
chr3	52208487	52235425	26939	2	0	11	0	0.01767679	0	16	0	0.91308053
chr3	52235425	52246329	10905	2	0	11	0	0.00859896	0	17	0	1.14735594
chr3	52246329	52267593	21265	3	0	11	0	0.01767679	0	16	0	0.91308053
chr3	52267593	52412144	144552	8	0	11	0	0.00373523	0	18	0	1.42397267
chr3	52412144	52443268	31125	2	0	11	0	0.01767679	0	16	0	0.91308053
chr3	52443268	52452536	9269	1	0	11	0	0.0331093	0	15	0	0.71538971
chr3	52452536	52457616	5081	2	0	11	0	0.01767679	0	16	0	0.91308053
chr3	52457616	52472974	15359	2	0	11	0	0.00859896	0	17	0	1.14735594
chr3	52472974	52702636	229663	9	0	11	0	0.0331093	0	15	0	0.71538971
chr3	52702636	52723028	20393	2	0	11	0	0.0574087	0	14	0	0.5498098
chr3	52723028	52820348	97321	6	0	11	0	0.0331093	0	15	0	0.71538971
chr3	52820348	52852319	31972	2	0	11	0	0.0574087	0	14	0	0.5498098
chr3	52852319	52938949	86631	2	0	10	0	0.03344703	0	14	0	0.71353304
chr3	52938949	52965015	26067	2	0	11	0	0.01767679	0	16	0	0.91308053
chr3	52965015	53022005	56991	1	0	10	0	0.01817691	0	15	0	0.90719478
chr3	53022005	53022064	60	1	0	10	0	0.00909834	0	16	0	1.13428373
chr3	53022064	53123438	101375	2	0	10	0	0.03344703	0	14	0	0.71353304
chr3	53123438	53133469	10032	2	0	10	0	0.01817691	0	15	0	0.90719478
chr3	53133469	53207082	73614	2	0	10	0	0.00909834	0	16	0	1.13428373
chr3	53207082	53207141	60	1	0	10	0	0.00413023	0	17	0	1.39918918
chr3	53207141	53226523	19383	2	0	10	0	0.00909834	0	16	0	1.13428373
chr3	53226523	53276971	50449	2	0	9	0	0.01817691	0	14	0	0.90719478
chr3	53276971	53288546	11576	2	0	9	0	0.00926608	0	15	0	1.13004284
chr3	53288546	53408660	120115	3	0	9	0	0.01817691	0	14	0	0.90719478
chr3	53408660	53492645	83986	1	0	9	0	0.0565833	0	12	0	0.55190077
chr3	53492645	53492704	60	1	0	10	0	0.09290028	0	12	0	0.41314172

chr3	53492704	53579702	86999	2	0	8	0	0.03209037	0	12	0	0.72109894
chr3	53579702	53632989	53288	2	0	9	0	0.0331093	0	13	0	0.71538971
chr3	53632989	53699768	66780	1	0	8	0	0.01767679	0	13	0	0.91308053
chr3	53699768	53707078	7311	1	0	8	0	0.03209037	0	12	0	0.72109894
chr3	53707078	53851470	144393	3	0	8	0	0.05490675	0	11	0	0.55623409
chr3	53851470	54205015	353546	12	0	8	0	0.01767679	0	13	0	0.91308053
chr3	54205015	54264485	59471	1	0	7	0	0.00859896	0	13	0	1.14735594
chr3	54264485	54301899	37415	1	0	7	0	0.01667721	0	12	0	0.92532268
chr3	54301899	54420758	118860	2	0	7	0	0.08584816	0	9	0	0.42015402
chr3	54420758	54511105	90348	3	0	7	0	0.05232577	0	10	0	0.56314362
chr3	54511105	54511164	60	1	0	7	0	0.01667721	0	12	0	0.92532268
chr3	54511164	54596850	85687	2	0	7	0	0.05232577	0	10	0	0.56314362
chr3	54596850	54596909	60	1	0	7	0	0.00859896	0	13	0	1.14735594
chr3	54596909	54659843	62935	1	0	7	0	0.01667721	0	12	0	0.92532268
chr3	54659843	54731990	72148	2	0	7	0	0.00859896	0	13	0	1.14735594
chr3	54731990	54833351	101362	3	0	7	0	0.00413023	0	14	0	1.39918918
chr3	54833351	54867690	34340	2	0	8	0	0.00909834	0	14	0	1.13428373
chr3	54867690	54867749	60	1	0	9	0	0.01817691	0	14	0	0.90719478
chr3	54867749	54959033	91285	2	0	7	0	0.01667721	0	12	0	0.92532268
chr3	54959033	55000866	41834	2	0	7	0	0.00859896	0	13	0	1.14735594
chr3	55000866	55092576	91711	3	0	7	0	0.00413023	0	14	0	1.39918918
chr3	55092576	55180765	88190	1	0	7	0	0.00859896	0	13	0	1.14735594
chr3	55180765	55375793	195029	1	0	7	0	0.05232577	0	10	0	0.56314362
chr3	55375793	55503453	127661	2	0	7	0	0.03037338	0	11	0	0.73110763
chr3	55503453	55510704	7252	2	0	7	0	0.00413023	0	14	0	1.39918918
chr3	55510704	55542756	32053	2	0	7	0	0.00859896	0	13	0	1.14735594
chr3	55542756	55620468	77713	2	0	7	0	0.03037338	0	11	0	0.73110763
chr3	55620468	55691364	70897	2	0	7	0	0.00859896	0	13	0	1.14735594
chr3	55691364	55692063	700	2	0	8	0	0.01767679	0	13	0	0.91308053
chr3	55692063	55859685	167623	3	0	6	0	0.00373523	0	13	0	1.42397267
chr3	55859685	55859744	60	1	0	7	0	0.00859896	0	13	0	1.14735594
chr3	55859744	55948782	89039	2	0	6	0	0.00373523	0	13	0	1.42397267
chr3	55948782	56150493	201712	5	0	7	0	0.00859896	0	13	0	1.14735594
chr3	56150493	56469070	318578	7	0	6	0	0.00373523	0	13	0	1.42397267
chr3	56469070	56665459	196390	3	0	6	0	0.00778066	0	12	0	1.17038931
chr3	56665459	56738024	72566	2	0	6	0	0.01518174	0	11	0	0.9449689
chr3	56738024	56738083	60	1	0	6	0	0.00373523	0	13	0	1.42397267
chr3	56738083	56846587	108505	2	0	5	0	0.00666883	0	11	0	1.20557689
chr3	56846587	56890501	43915	2	0	6	0	0.01518174	0	11	0	0.9449689
chr3	56890501	56890560	60	1	0	6	0	0.00778066	0	12	0	1.17038931
chr3	56890560	56974312	83753	2	0	4	0	0.00530919	0	10	0	1.2568129
chr3	56974312	57004574	30263	2	0	5	0	0.01320236	0	10	0	0.97390707
chr3	57004574	57059396	54823	1	0	3	0	0.00378107	0	9	0	1.33111237
chr3	57059396	57104377	44982	1	0	3	0	0.0079614	0	8	0	1.07548421
chr3	57104377	57106457	2081	2	0	4	0	0.01077081	0	9	0	1.01542894
chr3	57106457	57156030	49574	1	0	3	0	0.0079614	0	8	0	1.07548421
chr3	57156030	57156089	60	1	0	4	0	0.01077081	0	9	0	1.01542894
chr3	57156089	57199594	43506	1	0	4	0	0.02074938	0	8	0	0.79906872
chr3	57199594	57280132	80539	3	0	5	0	0.04407651	0	8	0	0.58747015
chr3	57280132	57307137	27006	2	0	5	0	0.02473314	0	9	0	0.76806864
chr3	57307137	57312440	5304	2	0	5	0	0.01320236	0	10	0	0.97390707
chr3	57312440	57368535	56096	1	0	5	0	0.02473314	0	9	0	0.76806864
chr3	57368535	57447339	78805	3	0	6	0	0.04875589	0	9	0	0.5732208
chr3	57447339	57493398	46060	2	0	7	0	0.01667721	0	12	0	0.92532268
chr3	57493398	57578459	85062	5	0	7	0	0.00859896	0	13	0	1.14735594
chr3	57578459	57578518	60	1	0	7	0	0.00413023	0	14	0	1.39918918
chr3	57578518	57716972	138455	4	0	7	0	0.00859896	0	13	0	1.14735594
chr3	57716972	57808492	91521	2	0	7	0	0.01667721	0	12	0	0.92532268
chr3	57808492	57868145	59654	2	0	7	0	0.00859896	0	13	0	1.14735594
chr3	57868145	57908594	40450	3	0	8	0	0.00909834	0	14	0	1.13428373
chr3	57908594	58003431	94838	3	0	7	0	0.00413023	0	14	0	1.39918918
chr3	58003431	58063965	60535	2	0	7	0	0.03037338	0	11	0	0.73110763
chr3	58063965	58098048	34084	2	0	7	0	0.01667721	0	12	0	0.92532268
chr3	58098048	58225417	127370	3	0	6	0	0.01518174	0	11	0	0.9449689
chr3	58225417	58225476	60	1	0	8	0	0.03209037	0	12	0	0.72109894
chr3	58225476	58271119	45644	1	0	7	0	0.01667721	0	12	0	0.92532268
chr3	58271119	58356175	85057	2	0	6	0	0.01518174	0	11	0	0.9449689
chr3	58356175	58356233	59	1	0	6	0	0.00778066	0	12	0	1.17038931
chr3	58356233	58413496	57264	2	0	6	0	0.02793176	0	10	0	0.74627054
chr3	58413496	58480226	66731	2	0	6	0	0.04875589	0	9	0	0.5732208
chr3	58480226	58482816	2591	2	0	6	0	0.02793176	0	10	0	0.74627054
chr3	58482816	58484351	1536	2	0	6	0	0.04875589	0	9	0	0.5732208
chr3	58484351	58552334	67984	3	0	6	0	0.02793176	0	10	0	0.74627054
chr3	58552334	58552380	47	1	0	6	0	0.01518174	0	11	0	0.9449689
chr3	58552380	58598577	46198	1	0	6	0	0.02793176	0	10	0	0.74627054
chr3	58598577	58598636	60	1	0	7	0	0.01667721	0	12	0	0.92532268
chr3	58598636	58729348	130713	2	0	6	0	0.02793176	0	10	0	0.74627054
chr3	58729348	58963022	233675	5	0	7	0	0.05232577	0	10	0	0.56314362
chr3	58963022	59233839	270818	4	0	9	0	0.0331093	0	13	0	0.71538971
chr3	59233839	59233898	60	1	0	10	0	0.01817691	0	15	0	0.90719478
chr3	59233898	59704251	470354	2	0	7	0	0.03037338	0	11	0	0.73110763
chr3	59704251	59802366	98116	2	0	6	0	0.02793176	0	10	0	0.74627054
chr3	59802366	59836057	33692	2	0	6	0	0.01518174	0	11	0	0.9449689
chr3	59836057	59891439	55383	1	0	5	0	0.00666883	0	11	0	1.20557689
chr3	59891439	59926153	34715	2	0	7	0	0.03037338	0	11	0	0.73110763
chr3	59926153	59926212	60	1	1	9	0.30102999	0.0565833	0	12	0	0.55190077
chr3	59926212	60011102	84891	4	1	8	0.30102999	0.03209037	0	12	0	0.72109894
chr3	60011102	60088412	77311	3	1	9	0.30102999	0.0565833	0	12	0	0.55190077
chr3	60088412	60142586	54175	1	1	7	0.30102999	0.01667721	0	12	0	0.92532268
chr3	60142586	60292281	149696	3	1	6	0.30102999	0.01518174	0	11	0	0.9449689
chr3	60292281	60331268	38988	1	0	6	0	0.01518174	0	11	0	0.9449689
chr3	60331268	60472555	141288	4	0	7	0	0.03037338	0	11	0	0.73110763
chr3	60472555	60522686	50132	1	0	6	0	0.01518174	0	11	0	0.9449689
chr3	60522686	60597480	74795	1	0	5	0	0.01320236	0	10	0	0.97390707
chr3	60597480	60684223	86744	2	0	5	0	0.02473314	0	9	0	0.76806864
chr3	60684223	60684282	60	1	0	5	0	0.01320236	0	10	0	0.97390707
chr3	60684282	60728525	44244	1	0	5	0	0.02473314	0	9	0	0.76806864
chr3	60728525	60768201	39677	2	0	6	0	0.04875589	0	9	0	0.5732208
chr3	60768201	60807716	39516	2	0	6	0	0.02793176	0	10	0	0.74627054
chr3	60807716	60877562	69847	2	0	6	0	0.04875589	0	9	0	0.5732208
chr3	60877562	60877621	60	1	0	6	0	0.02793176	0	10	0	0.74627054
chr3	60877621	60941904	64284	1	0	6	0	0.04875589	0	9	0	0.5732208
chr3	60941904	60993082	51179	1	0	5	0	0.04407651	0	8	0	0.58747015
chr3	60993082	61067181	74100	1	0	5	0	0.12309572	0	6	0	0.30102999
chr3	61067181	61155881	88701	2	0	5	0	0.45545077	0	3	0	0.05670724
chr3	61155881	61330047	174167	3	0	5	0	0.12309572	0	6	0	0.30102999
chr3	61330047	61330106	60	1	0	5	0	0.07511598	0	7	0	0.43181735
chr3	61330106	61552488	222383	1	0	3	0	0.05670724	0	5	0	0.45545077
chr3	61552488	61673552	121065	3	0	3	0	0.03070643	0	6	0	0.63695542
chr3	61673552	61726654	53103	2	0	4	0	0.03812622	0	7	0	0.60763643
chr3	61726654	61782025	55372	3	0	5	0	0.07511598	0	7	0	0.43181735
chr3	61782025	61895427	113403	3	0	5	0	0.07511598	1	7	0.30102999	0.43181735
chr3	61895427	61895486	60	1	0	6	0	0.08122616	1	8	0.30102999	0.4250187
chr3	61895486	61956580	61095	1	0	5	0	0.12309572	1	6	0.30102999	0.30102999
chr3	61956580	62015386	58807	1	0	4	0	0.06713722	1	6	0.30102999	0.44141547
chr3	62015386	62063875	48490	2	0	4	0	0.03812622	1	7	0.30102999	0.60763643
chr3	62063875	62063932	58	1	0	5	0	0.02473314	1	9	0.30102999	0.76806864
chr3	62063932	62212491	148560	2	0	5	0	0.04407651	1	8	0.30102999	0.58747015
chr3	62212491	62304456	91966	4	0	7	0	0.08584816	1	9	0.30102999	0.42015402
chr3	62304456	62452003	147548	4	0	7	0	0.13499366	1	8	0.30102999	0.30102999
chr3	62452003	62506522	54520	2	0	8	0	0.20764654	1	8	0.30102999	0.20764654
chr3	62506522	62555365	48844	2	0	8	0	0.13872638	1	9	0.30102999	0.30102999
chr3	62555365	62604904	49540	2	0	9	0	0.14135546	1	10	0.30102999	0.30102999
chr3	62604904	62604963	60	1	0	9	0	0.09154957	1	11	0.30102999	0.41444892

chr3	62604963	62636769	31807	1	0	8	0	0.08923391	1	10	0.30102999	0.4167287
chr3	62636769	62718641	81873	1	0	8	0	0.13872638	1	9	0.30102999	0.30102999
chr3	62718641	62749547	30907	2	0	9	0	0.14135546	1	10	0.30102999	0.30102999
chr3	62749547	62829648	80102	3	0	9	0	0.0565833	1	12	0.30102999	0.55190077
chr3	62829648	62829707	60	1	0	10	0	0.09290028	1	12	0.30102999	0.41314172
chr3	62829707	62982510	152804	2	0	9	0	0.0565833	1	12	0.30102999	0.55190077
chr3	62982510	63021905	39396	2	0	10	0	0.09290028	1	12	0.30102999	0.41314172
chr3	63021905	63021964	60	1	0	11	0	0.14385241	1	12	0.30102999	0.30102999
chr3	63021964	63270300	248337	2	0	10	0	0.09290028	1	12	0.30102999	0.41314172
chr3	63270300	63429139	158840	4	0	9	0	0.0565833	1	12	0.30102999	0.55190077
chr3	63429139	63534706	105568	3	0	8	0	0.05490675	1	11	0.30102999	0.55623409
chr3	63534706	63601696	66991	2	0	9	0	0.09154957	1	11	0.30102999	0.41444892
chr3	63601696	63602558	863	2	0	11	0	0.14385241	1	12	0.30102999	0.30102999
chr3	63602558	63749238	146681	2	0	10	0	0.21118145	1	10	0.30102999	0.21118145
chr3	63749238	63749297	60	1	0	11	0	0.14385241	1	12	0.30102999	0.30102999
chr3	63749297	63850382	101086	3	0	8	0	0.08923391	1	10	0.30102999	0.4167287
chr3	63850382	64005029	154648	5	0	9	0	0.09154957	1	11	0.30102999	0.41444892
chr3	64005029	64162112	157084	3	0	8	0	0.05490675	1	11	0.30102999	0.55623409
chr3	64162112	64210840	48729	1	0	7	0	0.05232577	1	10	0.30102999	0.56314362
chr3	64210840	64210899	60	1	0	8	0	0.08923391	1	10	0.30102999	0.4167287
chr3	64210899	64274077	63179	1	0	6	0	0.04875589	1	9	0.30102999	0.5732208
chr3	64274077	64376621	102545	1	0	6	0	0.08122616	0	8	0	0.4250187
chr3	64376621	64436127	59507	2	0	6	0	0.02793176	0	10	0	0.74627054
chr3	64436127	64436186	60	1	0	6	0	0.00778066	0	12	0	1.17038931
chr3	64436186	64527211	91026	1	0	5	0	0.00666883	0	11	0	1.20557689
chr3	64527211	64527270	60	1	0	6	0	0.01518174	0	11	0	0.9449689
chr3	64527270	64600977	73708	2	0	6	0	0.04875589	0	9	0	0.5732208
chr3	64600977	64666994	66018	2	0	5	0	0.02473314	0	9	0	0.76806864
chr3	64666994	64782183	115190	2	0	5	0	0.04407651	0	8	0	0.58747015
chr3	64782183	64859476	77294	1	0	3	0	0.01598258	0	7	0	0.84395715
chr3	64859476	64962685	103210	1	0	3	0	0.03070643	0	6	0	0.63695542
chr3	64962685	64962744	60	1	0	4	0	0.02074938	0	8	0	0.79906872
chr3	64962744	65063442	100699	1	0	1	0	0.02438896	0	3	0	0.51676182
chr3	65063442	65343024	279583	2	0	1	0	0.05404976	0	2	0	0.30102999
chr3	65343024	65463087	120064	4	0	1	0	0.02438896	0	3	0	0.51676182
chr3	65463087	65522030	58944	2	0	1	0	0.00478973	0	5	0	1.02643191
chr3	65522030	65522089	60	1	0	1	0	8.47E-04	0	7	0	1.62048027
chr3	65522089	65552195	30107	1	0	1	0	0.01091641	0	4	0	0.76005302
chr3	65552195	65609289	57095	1	0	1	0	0.02438896	0	3	0	0.51676182
chr3	65609289	65657597	48309	2	0	1	0	0.01091641	0	4	0	0.76005302
chr3	65657597	65728429	70833	2	0	1	0	0.00478973	0	5	0	1.02643191
chr3	65728429	66046077	317649	6	0	1	0	0.05404976	0	2	0	0.30102999
chr3	66046077	66312513	266437	2	0	1	0	0.02438896	0	3	0	0.51676182
chr3	66312513	66429429	116917	4	0	5	0	0.45545077	0	3	0	0.05670724
chr3	66429429	66499834	70406	2	0	4	0	0.30102999	0	3	0	0.10122019
chr3	66499834	66560836	61003	2	0	5	0	0.45545077	0	3	0	0.05670724
chr3	66560836	66656437	95602	2	0	5	0	0.12309572	0	6	0	0.30102999
chr3	66656437	66656496	60	1	0	5	0	0.07511598	0	7	0	0.43181735
chr3	66656496	66789762	133267	1	0	5	0	0.19510895	0	5	0	0.19510895
chr3	66789762	66789821	60	1	0	5	0	0.12309572	0	6	0	0.30102999
chr3	66789821	66870601	80781	1	0	5	0	0.19510895	0	5	0	0.19510895
chr3	66870601	66870660	60	1	0	5	0	0.12309572	0	6	0	0.30102999
chr3	66870660	66930519	59860	1	0	4	0	0.11390336	0	5	0	0.30102999
chr3	66930519	67013525	83007	2	0	5	0	0.19510895	0	5	0	0.19510895
chr3	67013525	67054101	40577	2	0	7	0	0.30102999	0	6	0	0.129913
chr3	67054101	67060463	6363	3	0	8	0	0.20764654	0	8	0	0.20764654
chr3	67060463	67161871	101409	1	0	7	0	0.30102999	0	6	0	0.129913
chr3	67161871	67229219	67349	2	0	7	0	0.20469099	0	7	0	0.20469099
chr3	67229219	67310639	81421	2	0	7	0	0.08584816	0	9	0	0.42015402
chr3	67310639	67310698	60	1	0	7	0	0.03037338	0	11	0	0.73110763
chr3	67310698	67426529	115832	2	0	7	0	0.05232577	0	10	0	0.56314362
chr3	67426529	67506803	80275	2	0	7	0	0.08584816	0	9	0	0.42015402
chr3	67506803	67560723	53921	2	0	7	0	0.05232577	0	10	0	0.56314362
chr3	67560723	67560782	60	1	0	7	0	0.03037338	0	11	0	0.73110763
chr3	67560782	67891660	330879	6	0	6	0	0.01518174	0	11	0	0.9449689
chr3	67891660	67891719	60	1	0	7	0	0.00859896	0	13	0	1.14735594
chr3	67891719	68077107	185389	1	0	6	0	0.00373523	0	13	0	1.42397267
chr3	68077107	68192539	115433	3	0	7	0	0.00183109	0	15	0	1.68336308
chr3	68192539	68192598	60	1	0	10	0	0.01817691	0	15	0	0.90719478
chr3	68192598	68333063	140466	3	0	8	0	0.00909834	0	14	0	1.13428373
chr3	68333063	68457396	124334	2	0	7	0	0.00413023	0	14	0	1.39918918
chr3	68457396	68457455	60	1	0	8	0	0.00909834	0	14	0	1.13428373
chr3	68457455	68595380	137926	3	0	8	0	0.01767679	0	13	0	0.91308053
chr3	68595380	68688219	92840	1	0	7	0	0.01667721	0	12	0	0.92532268
chr3	68688219	68688278	60	1	0	8	0	0.03209037	0	12	0	0.72109894
chr3	68688278	68780973	92696	1	0	7	0	0.01667721	0	12	0	0.92532268
chr3	68780973	68874986	94014	3	0	7	0	0.00859896	0	13	0	1.14735594
chr3	68874986	68875045	60	1	0	9	0	0.0331093	0	13	0	0.71538971
chr3	68875045	68934354	59310	2	0	8	0	0.03209037	0	12	0	0.72109894
chr3	68934354	68953606	19253	2	0	9	0	0.0331093	0	13	0	0.71538971
chr3	68953606	69036632	83027	2	0	8	0	0.03209037	0	12	0	0.72109894
chr3	69036632	69154209	117578	4	0	7	0	0.03037338	0	11	0	0.73110763
chr3	69154209	69243229	89021	3	0	6	0	0.01518174	0	11	0	0.9449689
chr3	69243229	69317896	74668	1	0	6	0	0.02793176	0	10	0	0.74627054
chr3	69317896	69436836	118941	3	0	5	0	0.02473314	0	9	0	0.76806864
chr3	69436836	69436895	60	1	0	5	0	0.01320236	0	10	0	0.97390707
chr3	69436895	69554645	117751	2	0	4	0	0.06713722	0	6	0	0.44141547
chr3	69554645	69554704	60	1	0	5	0	0.12309572	0	6	0	0.30102999
chr3	69554704	69756504	201801	1	0	4	0	0.06713722	0	6	0	0.44141547
chr3	69756504	69756563	60	1	0	5	0	0.07511598	0	7	0	0.43181735
chr3	69756563	69912462	155900	3	0	4	0	0.03812622	0	7	0	0.60763643
chr3	69912462	70161708	249247	3	0	3	0	0.10122019	0	4	0	0.30102999
chr3	70161708	70161767	60	1	0	3	0	0.10122019	1	4	0.30102999	0.30102999
chr3	70161767	70295249	133483	1	0	3	0	0.17593012	1	3	0.30102999	0.17593012
chr3	70295249	70295308	60	1	0	3	0	0.10122019	1	4	0.30102999	0.30102999
chr3	70295308	70515573	220266	1	0	2	0	0.08289318	1	3	0.30102999	0.30102999
chr3	70515573	70659936	144364	2	0	3	0	0.05670724	1	5	0.30102999	0.45545077
chr3	70659936	70765449	105514	2	0	3	0	0.01598258	1	7	0.30102999	0.84395715
chr3	70765449	71041607	276159	4	0	3	0	0.03070643	1	6	0.30102999	0.63695542
chr3	71041607	71073590	31984	2	0	4	0	0.02074938	1	8	0.30102999	0.79906872
chr3	71073590	71199917	126328	3	0	4	0	0.06713722	1	6	0.30102999	0.44141547
chr3	71199917	71319916	120000	3	0	4	0	0.03812622	1	7	0.30102999	0.60763643
chr3	71319916	71448410	128495	5	0	5	0	0.01320236	1	10	0.30102999	0.97390707
chr3	71448410	71573888	125479	4	0	5	0	0.00317045	1	12	0.30102999	1.46403142
chr3	71573888	71712411	138524	3	0	4	0	0.00530919	1	10	0.30102999	1.2568129
chr3	71712411	71733309	20899	2	0	4	0	0.00247414	1	11	0.30102999	1.52371709
chr3	71733309	71829248	95940	2	0	4	0	0.00530919	1	10	0.30102999	1.2568129
chr3	71829248	71829307	60	1	0	5	0	0.01320236	1	10	0.30102999	0.97390707
chr3	71829307	71900135	70829	1	0	5	0	0.04407651	1	8	0.30102999	0.58747015
chr3	71900135	71900194	60	1	0	5	0	0.04407651	2	8	0.61140001	0.58747015
chr3	71900194	72176769	276576	3	0	3	0	0.05670724	2	5	0.61140001	0.45545077
chr3	72176769	72176828	60	1	0	4	0	0.11390336	2	5	0.61140001	0.30102999
chr3	72176828	72312493	135666	2	0	3	0	0.05670724	2	5	0.61140001	0.45545077
chr3	72312493	72494685	182193	3	0	3	0	0.05670724	1	5	0.30102999	0.45545077
chr3	72494685	72494744	60	1	0	4	0	0.11390336	1	5	0.30102999	0.30102999
chr3	72494744	72915275	420532	6	0	2	0	0.08289318	1	3	0.30102999	0.30102999
chr3	72915275	73248335	333061	10	1	2	0.1218695	0.08289318	1	3	0.1218695	0.30102999
chr3	73248335	73344292	95958	1	1	1	0.1218695	0.05404976	1	2	0.1218695	0.30102999
chr3	73344292	73509597	165306	3	0	1	0	0.05404976	1	2	0.30102999	0.30102999
chr3	73509597	73587796	78200	3	1	1	0.1218695	0.02438896	1	3	0.1218695	0.51676182
chr3	73587796	73631758	43963	2	1	1	0.1218695	0.01091641	1	4	0.1218695	0.76005302
chr3	73631758	73893412	261655	2	1	1	0.1218695	0.02438896	1	3	0.1218695	0.51676182
chr3	73893412	74028091	134680	2	0	1	0	0.02438896	1	3	0.30102999	0.51676182
chr3	74028091	74132044	103954	2	0	2	0	0.08289318	1	3	0.30102999	0.30102999

chr3	74132044	74184593	52550	1	0	1	0	0.02438896	1	3	0.30102999	0.51676182
chr3	74184593	74281126	96534	2	0	1	0	0.01091641	1	4	0.30102999	0.76005302
chr3	74281126	74413474	132349	4	0	1	0	0.00478973	1	5	0.30102999	1.02643191
chr3	74413474	74560348	146875	3	0	1	0	0.01091641	1	4	0.30102999	0.76005302
chr3	74560348	74560407	60	1	0	3	0	0.03070643	2	6	0.61140001	0.63695542
chr3	74560407	74676232	115826	1	0	2	0	0.0429175	2	4	0.61140001	0.47744371
chr3	74676232	74880834	204603	1	0	1	0	0.01091641	2	4	0.61140001	0.76005302
chr3	74880834	74964351	83518	1	0	1	0	0.01091641	0	4	0	0.76005302
chr3	74964351	74964410	60	1	0	3	0	0.03070643	0	6	0	0.63695542
chr3	74964410	75047236	82827	1	0	2	0	0.02162467	0	5	0	0.68214471
chr3	75047236	75047295	60	1	0	2	0	0.01053319	0	6	0	0.91219088
chr3	75047295	75767533	720239	3	0	2	0	0.02162467	0	5	0	0.68214471
chr3	75767533	75936605	169073	2	0	4	0	0.06713722	1	6	0.30102999	0.44141547
chr3	75936605	75936664	60	1	0	4	0	0.06713722	2	6	0.61140001	0.44141547
chr3	75936664	76026268	89605	1	0	4	0	0.06713722	1	6	0.30102999	0.44141547
chr3	76026268	76084202	57935	2	0	5	0	0.12309572	1	6	0.30102999	0.30102999
chr3	76084202	76156912	72711	2	0	3	0	0.03070643	1	6	0.30102999	0.63695542
chr3	76156912	76454834	297923	1	0	2	0	0.01053319	1	6	0.30102999	0.91219088
chr3	76454834	76498893	44060	2	0	4	0	0.03812622	1	7	0.30102999	0.60763643
chr3	76498893	76498952	60	1	0	6	0	0.04875589	1	9	0.30102999	0.5732208
chr3	76498952	76594483	95532	2	0	5	0	0.02473314	1	9	0.30102999	0.76806864
chr3	76594483	76594542	60	1	0	7	0	0.08584816	1	9	0.30102999	0.42015402
chr3	76594542	76724667	130126	3	0	6	0	0.04875589	1	9	0.30102999	0.5732208
chr3	76724667	76764322	39656	2	0	8	0	0.13872638	1	9	0.30102999	0.30102999
chr3	76764322	76874648	110327	2	0	7	0	0.13499366	1	8	0.30102999	0.30102999
chr3	76874648	76918117	43470	2	0	8	0	0.13872638	1	9	0.30102999	0.30102999
chr3	76918117	77002113	83997	2	0	7	0	0.08584816	1	9	0.30102999	0.42015402
chr3	77002113	77097318	95206	2	0	5	0	0.02473314	1	9	0.30102999	0.76806864
chr3	77097318	77470069	372752	9	0	6	0	0.04875589	1	9	0.30102999	0.5732208
chr3	77470069	77496712	26644	2	0	7	0	0.08584816	1	9	0.30102999	0.42015402
chr3	77496712	77575075	78364	2	0	6	0	0.04875589	1	9	0.30102999	0.5732208
chr3	77575075	77575134	60	1	0	7	0	0.08584816	1	9	0.30102999	0.42015402
chr3	77575134	77607540	32407	1	0	6	0	0.08122616	1	8	0.30102999	0.4250187
chr3	77607540	77820055	212516	2	0	4	0	0.03812622	1	7	0.30102999	0.60763643
chr3	77820055	77820114	60	1	0	5	0	0.04407651	1	8	0.30102999	0.58747015
chr3	77820114	77923996	103883	1	0	5	0	0.07511598	1	7	0.30102999	0.43181735
chr3	77923996	77924055	60	1	0	6	0	0.08122616	1	8	0.30102999	0.4250187
chr3	77924055	78074793	150739	1	0	5	0	0.04407651	1	8	0.30102999	0.58747015
chr3	78074793	78074852	60	1	0	5	0	0.02473314	2	9	0.61140001	0.76806864
chr3	78074852	78154423	79572	1	0	5	0	0.04407651	1	8	0.30102999	0.58747015
chr3	78154423	78239521	85099	3	0	7	0	0.08584816	1	9	0.30102999	0.42015402
chr3	78239521	78378162	138642	1	0	6	0	0.08122616	1	8	0.30102999	0.4250187
chr3	78378162	78647198	269037	2	0	4	0	0.02074938	1	8	0.30102999	0.79906872
chr3	78647198	78689449	42252	2	0	5	0	0.04407651	2	8	0.61140001	0.58747015
chr3	78689449	78797837	108389	2	0	4	0	0.02074938	2	8	0.61140001	0.79906872
chr3	78797837	78797896	60	1	0	5	0	0.04407651	2	8	0.61140001	0.58747015
chr3	78797896	79203398	405503	7	0	4	0	0.02074938	1	8	0.30102999	0.79906872
chr3	79203398	79203457	60	1	0	4	0	0.02074938	2	8	0.61140001	0.79906872
chr3	79203457	79318928	115472	2	0	4	0	0.02074938	1	8	0.30102999	0.79906872
chr3	79318928	79347798	28871	2	0	4	0	0.02074938	2	8	0.61140001	0.79906872
chr3	79347798	79467905	120108	2	0	3	0	0.0079614	2	8	0.61140001	1.07548421
chr3	79467905	79639015	171111	4	0	4	0	0.02074938	2	8	0.61140001	0.79906872
chr3	79639015	79639072	58	1	0	4	0	0.01077081	2	9	0.61140001	1.01542894
chr3	79639072	79699930	60859	1	0	3	0	0.0079614	1	8	0.30102999	1.07548421
chr3	79699930	79759929	60000	1	0	3	0	0.01598258	1	7	0.30102999	0.84395715
chr3	79759929	79940164	180236	2	0	4	0	0.03812622	1	7	0.30102999	0.60763643
chr3	79940164	79940223	60	1	0	4	0	0.01077081	1	9	0.30102999	1.01542894
chr3	79940223	80312583	372361	2	0	1	0	8.47E-04	1	7	0.30102999	1.62048027
chr3	80312583	80312642	60	1	0	4	0	0.03812622	1	7	0.30102999	0.60763643
chr3	80312642	80461965	149324	1	0	4	0	0.06713722	0	6	0	0.44141547
chr3	80461965	80616078	154114	1	0	3	0	0.03070643	0	6	0	0.63695542
chr3	80616078	80616137	60	1	0	4	0	0.06713722	0	6	0	0.44141547
chr3	80616137	80991324	375188	4	0	3	0	0.03070643	0	6	0	0.63695542
chr3	80991324	81201515	210192	1	0	3	0	0.05670724	0	5	0	0.45545077
chr3	81201515	81201574	60	1	0	5	0	0.19510895	0	5	0	0.19510895
chr3	81201574	81478254	276681	1	0	3	0	0.05670724	0	5	0	0.45545077
chr3	81478254	81586100	107847	3	0	3	0	0.05670724	1	5	0.30102999	0.45545077
chr3	81586100	81586159	60	1	0	4	0	0.06713722	1	6	0.30102999	0.44141547
chr3	81586159	81698088	111930	2	0	3	0	0.05670724	1	5	0.30102999	0.45545077
chr3	81698088	81754679	56592	2	1	3	0.1218695	0.05670724	1	5	0.1218695	0.45545077
chr3	81754679	81791748	37070	2	1	4	0.1218695	0.06713722	1	6	0.1218695	0.44141547
chr3	81791748	82045726	253979	2	1	3	0.30102999	0.05670724	0	5	0	0.45545077
chr3	82045726	82165617	119892	2	1	3	0.30102999	0.03070643	0	6	0	0.63695542
chr3	82165617	82469723	304107	4	1	3	0.30102999	0.10122019	0	4	0	0.30102999
chr3	82469723	82719327	249605	3	1	2	0.30102999	0.0429175	0	4	0	0.47744371
chr3	82719327	82777977	58651	1	1	2	0.30102999	0.08289318	0	3	0	0.30102999
chr3	82777977	83049742	271766	2	0	2	0	0.08289318	0	3	0	0.30102999
chr3	83049742	83189102	139361	1	0	1	0	0.02438896	0	3	0	0.51676182
chr3	83189102	83938767	749666	4	0	0	0	0	0	2	0	0.61140001
chr3	83938767	84168509	229743	2	0	1	0	0.05404976	0	2	0	0.30102999
chr3	84168509	84331847	163339	2	0	3	0	0.10122019	0	4	0	0.30102999
chr3	84331847	84331906	60	1	0	5	0	0.12309572	0	6	0	0.30102999
chr3	84331906	84654831	322926	2	0	3	0	0.03070643	0	6	0	0.63695542
chr3	84654831	84777412	122582	2	0	4	0	0.06713722	1	6	0.30102999	0.44141547
chr3	84777412	84854143	76732	2	0	4	0	0.03812622	1	7	0.30102999	0.60763643
chr3	84854143	84854202	60	1	0	4	0	0.01077081	2	9	0.61140001	1.01542894
chr3	84854202	84959321	105120	1	0	4	0	0.01077081	1	9	0.30102999	1.01542894
chr3	84959321	85017897	58577	2	0	5	0	0.01320236	1	10	0.30102999	0.97390707
chr3	85017897	85119132	101236	3	0	6	0	0.01518174	1	11	0.30102999	0.9449689
chr3	85119132	85119191	60	1	0	8	0	0.05490675	2	11	0.61140001	0.55623409
chr3	85119191	85246245	127055	3	0	7	0	0.03037338	2	11	0.61140001	0.73110763
chr3	85246245	85362677	116433	2	0	7	0	0.05232577	2	10	0.61140001	0.56314362
chr3	85362677	85362736	60	1	0	8	0	0.08923391	3	10	0.93173516	0.4167287
chr3	85362736	85489796	127061	3	0	6	0	0.04875589	3	9	0.93173516	0.5732208
chr3	85489796	85615627	125832	2	0	6	0	0.04875589	2	9	0.61140001	0.5732208
chr3	85615627	85690196	74570	1	0	6	0	0.08122616	2	8	0.61140001	0.4250187
chr3	85690196	85896326	206131	6	0	7	0	0.13499366	3	8	0.93173516	0.30102999
chr3	85896326	85935371	39046	2	0	8	0	0.20764654	3	8	0.93173516	0.20764654
chr3	85935371	85984988	49618	1	0	8	0	0.20764654	2	8	0.61140001	0.20764654
chr3	85984988	86014922	29935	1	0	6	0	0.129913	2	7	0.61140001	0.30102999
chr3	86014922	86014981	60	1	0	6	0	0.129913	3	7	0.93173516	0.30102999
chr3	86014981	86155424	140444	4	0	5	0	0.07511598	3	7	0.93173516	0.43181735
chr3	86155424	86155483	60	1	0	6	0	0.129913	3	7	0.93173516	0.30102999
chr3	86155483	86395801	240319	2	0	5	0	0.12309572	1	6	0.30102999	0.30102999
chr3	86395801	86395860	60	1	0	5	0	0.12309572	2	6	0.61140001	0.30102999
chr3	86395860	86499242	103383	1	0	5	0	0.12309572	1	6	0.30102999	0.30102999
chr3	86499242	86499301	60	1	0	6	0	0.20064824	1	6	0.30102999	0.20064824
chr3	86499301	86661018	161718	1	0	6	0	0.20064824	0	6	0	0.20064824
chr3	86661018	86661077	60	1	0	7	0	0.30102999	0	6	0	0.129913
chr3	86661077	86789415	128339	1	0	3	0	0.05670724	0	5	0	0.45545077
chr3	86789415	86917318	127904	1	0	2	0	0.02162467	0	5	0	0.68214471
chr3	86917318	86917377	60	1	0	3	0	0.05670724	0	5	0	0.45545077
chr3	93580736	94130567	549832	11	0	0	0	0	0	2	0	0.61140001
chr3	94130567	94259056	128490	2	0	0	0	0	1	3	0.30102999	0.93173516
chr3	94259056	94728345	469290	3	0	0	0	0	0	3	0	0.93173516
chr3	94728345	94798716	70372	2	0	0	0	0	0	4	0	1.26272838
chr3	94798716	94798775	60	1	0	0	0	0	0	5	0	1.60515106
chr3	94798775	95417572	618798	4	0	0	0	0	0	2	0	0.61140001
chr3	95417572	95417631	60	1	0	1	0	0.02438896	1	3	0.30102999	0.51676182
chr3	95417631	95699765	282135	1	0	0	0	0	1	2	0.30102999	0.61140001
chr3	95699765	95699824	60	1	0	0	0	0	2	3	0.61140001	0.93173516
chr3	95699824	95857330	157507	1	0	0	0	0	2	2	0.61140001	0.61140001
chr3	95857330	96127024	269695	3	0	1	0	0.05404976	2	2	0.61140001	0.30102999
chr3	96127024	96514417	387394	6	0	1	0	0.02438896	2	3	0.61140001	0.51676182

chr3	96514417	96561197	46781	2	0	2	0	0.08289318	2	3	0.61140001	0.30102999
chr3	96561197	96635896	74700	1	0	1	0	0.02438896	2	3	0.61140001	0.51676182
chr3	96635896	96674586	38691	2	0	1	0	0.01091641	2	4	0.61140001	0.76005302
chr3	96674586	96674645	60	1	0	1	0	0.01091641	3	4	0.93173516	0.76005302
chr3	96674645	96718305	43661	1	0	1	0	0.02438896	3	3	0.93173516	0.51676182
chr3	96718305	96803210	84906	3	0	2	0	0.08289318	3	3	0.93173516	0.30102999
chr3	96803210	96851351	48142	2	0	2	0	0.0429175	3	4	0.93173516	0.47744371
chr3	96851351	96890667	39317	1	0	2	0	0.08289318	3	3	0.93173516	0.30102999
chr3	96890667	97027202	136536	3	0	2	0	0.0429175	3	4	0.93173516	0.47744371
chr3	97027202	97027261	60	1	1	2	0.01091641	0.0429175	4	4	0.76005302	0.47744371
chr3	97027261	97065926	38666	1	0	2	0	0.0429175	3	4	0.93173516	0.47744371
chr3	97065926	97198151	132226	3	0	1	0	0.01091641	3	4	0.93173516	0.76005302
chr3	97198151	97198208	58	1	0	1	0	0.01091641	4	4	1.26272838	0.76005302
chr3	97198208	97251356	53149	1	0	1	0	0.01091641	3	4	0.93173516	0.76005302
chr3	97251356	97387375	136020	4	1	2	0.02438896	0.0429175	3	4	0.51676182	0.47744371
chr3	97387375	97387434	60	1	1	3	0.02438896	0.10122019	3	4	0.51676182	0.30102999
chr3	97387434	97510587	123154	2	0	3	0	0.10122019	3	4	0.93173516	0.30102999
chr3	97510587	97583413	72827	2	0	3	0	0.05670724	3	5	0.93173516	0.45545077
chr3	97583413	97634376	50964	2	0	3	0	0.03070643	4	6	1.26272838	0.63695542
chr3	97634376	98107832	473457	14	0	4	0	0.06713722	4	6	1.26272838	0.44141547
chr3	98107832	98217752	109921	2	0	2	0	0.02162467	4	5	1.26272838	0.68214471
chr3	98217752	98237805	20054	2	0	3	0	0.05670724	4	5	1.26272838	0.45545077
chr3	98237805	98300538	62734	2	0	2	0	0.0429175	4	4	1.26272838	0.47744371
chr3	98300538	98376112	75575	2	0	2	0	0.02162467	4	5	1.26272838	0.68214471
chr3	98376112	98425882	49771	2	0	2	0	0.02162467	5	5	1.60515106	0.68214471
chr3	98425882	98519608	93727	5	0	3	0	0.05670724	5	5	1.60515106	0.45545077
chr3	98519608	98600450	80843	3	0	3	0	0.10122019	5	4	1.60515106	0.30102999
chr3	98600450	98707794	107345	1	0	2	0	0.0429175	5	4	1.60515106	0.47744371
chr3	98707794	98923250	215457	1	0	1	0	0.01091641	4	4	1.26272838	0.76005302
chr3	98923250	98923309	60	1	0	2	0	0.02162467	4	5	1.26272838	0.68214471
chr3	98923309	99092014	168706	1	0	1	0	0.01091641	4	4	1.26272838	0.76005302
chr3	99092014	99361512	269499	3	0	1	0	0.01091641	5	4	1.60515106	0.76005302
chr3	99361512	99394565	33054	2	0	2	0	0.0429175	5	4	1.60515106	0.47744371
chr3	99394565	99449158	54594	1	0	1	0	0.01091641	5	4	1.60515106	0.76005302
chr3	99449158	99449217	60	1	1	1	0.00478973	0.01091641	5	4	1.02643191	0.76005302
chr3	99449217	99800234	351018	7	1	1	0.01091641	0.02438896	4	3	0.76005302	0.51676182
chr3	99800234	100058793	258560	8	1	1	0.01091641	0.01091641	4	4	0.76005302	0.76005302
chr3	100058793	100179891	121099	2	1	1	0.02438896	0.01091641	3	4	0.51676182	0.76005302
chr3	100179891	100287758	107868	5	1	2	0.02438896	0.0429175	3	4	0.51676182	0.47744371
chr3	100287758	100487464	199707	6	1	2	0.01091641	0.0429175	4	4	0.76005302	0.47744371
chr3	100487464	101023092	535629	9	1	2	0.02438896	0.0429175	3	4	0.51676182	0.47744371
chr3	101023092	101140101	117010	3	1	2	0.02438896	0.08289318	3	3	0.51676182	0.30102999
chr3	101140101	101369031	228931	6	1	2	0.02438896	0.0429175	3	4	0.51676182	0.47744371
chr3	101369031	101401286	32256	1	1	2	0.02438896	0.08289318	3	3	0.51676182	0.30102999
chr3	101401286	101481600	80315	2	0	1	0	0.05404976	3	2	0.93173516	0.30102999
chr3	101481600	101535734	54135	3	0	2	0	0.08289318	3	3	0.93173516	0.30102999
chr3	101535734	101535793	60	1	0	2	0	0.0429175	3	4	0.93173516	0.47744371
chr3	101535793	101576165	40373	3	0	2	0	0.1575501	3	2	0.93173516	0.1575501
chr3	101576165	101987827	411663	6	0	1	0	0.05404976	3	2	0.93173516	0.30102999
chr3	101987827	102052187	64361	1	0	0	0	0	3	2	0.93173516	0.61140001
chr3	102052187	102058218	6032	2	1	0	0.02438896	0	3	2	0.51676182	0.61140001
chr3	102058218	102219027	160810	3	1	0	0.02438896	0	3	1	0.51676182	0.30102999
chr3	102219027	102452797	233771	2	0	0	0	0	3	1	0.93173516	0.30102999
chr3	102452797	102546125	93329	1	0	0	0	0	2	1	0.61140001	0.30102999
chr3	102546125	102949572	403448	3	0	0	0	0	2	0	0.61140001	0
chr3	102949572	103013579	64008	2	0	0	0	0	2	1	0.61140001	0.30102999
chr3	103013579	103567529	553951	3	0	0	0	0	1	1	0.30102999	0.30102999
chr3	103567529	103982720	415192	4	0	0	0	0	1	0	0.30102999	0
chr3	103982720	104147170	164451	2	0	0	0	0	2	0	0.61140001	0
chr3	104147170	104264937	117768	2	0	0	0	0	3	0	0.93173516	0
chr3	104264937	104429016	164080	2	1	0	0.02438896	0	3	0	0.51676182	0
chr3	104429016	104429075	60	1	2	0	0.0429175	0	4	0	0.47744371	0
chr3	104429075	104522243	93169	1	1	0	0.01091641	0	4	0	0.76005302	0
chr3	104522243	104964233	441991	4	1	0	0.01091641	0	4	1	0.76005302	0.30102999
chr3	104964233	104964292	60	1	1	1	0.01091641	0.05404976	4	2	0.76005302	0.30102999
chr3	104964292	105115687	151396	2	1	1	0.01091641	0.1218695	4	1	0.76005302	0.1218695
chr3	105115687	105294238	178552	6	1	1	0.01091641	0.30102999	4	0	0.76005302	0
chr3	105294238	105378304	84067	1	1	0	0.01091641	0	4	0	0.76005302	0
chr3	105378304	105404293	25990	2	1	0	0.00478973	0	5	1	1.02643191	0.30102999
chr3	105404293	105464779	60487	1	1	0	0.01091641	0	4	0	0.76005302	0
chr3	105464779	105496033	31255	2	1	0	0.00478973	0	5	0	1.02643191	0
chr3	105496033	105572391	76359	1	1	0	0.01091641	0	4	0	0.76005302	0
chr3	105572391	105602953	30563	2	1	0	0.01091641	0	4	1	0.76005302	0.30102999
chr3	105602953	105840810	237858	3	1	0	0.01091641	0	4	2	0.76005302	0.61140001
chr3	105840810	105840869	60	1	1	1	0.00478973	0.05404976	5	2	1.02643191	0.30102999
chr3	105840869	106228514	387646	3	1	0	0.00478973	0	5	0	1.02643191	0
chr3	106228514	106228573	60	1	1	0	0.00204627	0	6	0	1.31360226	0
chr3	106228573	106269524	40952	1	1	0	0.00478973	0	5	0	1.02643191	0
chr3	106269524	106309142	39619	1	1	0	0.01091641	0	4	0	0.76005302	0
chr3	106309142	106579618	270477	3	1	1	0.01091641	0.30102999	4	0	0.76005302	0
chr3	106579618	106579677	60	1	3	1	0.10122019	0.30102999	4	0	0.30102999	0
chr3	106579677	106840784	261108	3	2	1	0.0429175	0.30102999	4	0	0.47744371	0
chr3	106840784	106942747	101964	2	3	1	0.10122019	0.30102999	4	0	0.30102999	0
chr3	106942747	107016343	73597	2	3	1	0.05670724	0.30102999	5	0	0.45545077	0
chr3	107016343	107016402	60	1	3	1	0.03070643	0.30102999	6	0	0.63695542	0
chr3	107016402	107096646	80245	1	3	1	0.05670724	0.30102999	5	0	0.45545077	0
chr3	107096646	107204000	107355	2	3	1	0.03070643	0.30102999	6	0	0.63695542	0
chr3	107204000	107379608	175609	5	3	1	0.01598258	0.30102999	7	0	0.84395715	0
chr3	107379608	107379667	60	1	4	1	0.03812622	0.30102999	7	0	0.60763643	0
chr3	107379667	107447665	67999	1	4	1	0.06713722	0.30102999	6	0	0.44141547	0
chr3	107447665	107599677	152013	3	4	1	0.11390336	0.30102999	5	0	0.30102999	0
chr3	107599677	107599736	60	1	4	1	0.03812622	0.30102999	7	0	0.60763643	0
chr3	107599736	107676327	76592	1	3	1	0.01598258	0.30102999	7	0	0.84395715	0
chr3	107676327	107894872	218546	8	3	1	0.01598258	0.1218695	7	1	0.84395715	0.1218695
chr3	107894872	107894931	60	1	3	1	0.01598258	0.05404976	7	2	0.84395715	0.30102999
chr3	107894931	107974670	79740	2	3	1	0.10122019	0.1218695	4	1	0.30102999	0.1218695
chr3	107974670	108029553	54884	2	3	1	0.10122019	0.05404976	4	2	0.30102999	0.30102999
chr3	108029553	108029612	60	1	3	1	0.10122019	0.02438896	4	3	0.30102999	0.51676182
chr3	108029612	108072261	42650	1	2	1	0.08289318	0.02438896	3	3	0.30102999	0.51676182
chr3	108072261	108366967	294707	8	2	1	0.08289318	0.01091641	3	4	0.30102999	0.76005302
chr3	108366967	108367026	60	1	2	1	0.08289318	0.00478973	3	5	0.30102999	1.02643191
chr3	108367026	108549571	182546	3	2	1	0.08289318	0.01091641	3	4	0.30102999	0.76005302
chr3	108549571	108914802	365232	9	2	1	0.08289318	0.00478973	3	5	0.30102999	1.02643191
chr3	108914802	109056864	142063	4	1	1	0.02438896	0.00478973	3	5	0.51676182	1.02643191
chr3	109056864	109134338	77475	1	1	1	0.02438896	0.02438896	3	3	0.51676182	0.51676182
chr3	109134338	109237152	102815	1	1	1	0.05404976	0.02438896	2	3	0.30102999	0.51676182
chr3	109237152	109546841	309690	2	1	1	0.1218695	0.02438896	1	3	0.1218695	0.51676182
chr3	109546841	109748375	201535	1	0	1	0	0.05404976	1	2	0.30102999	0.30102999
chr3	109748375	109955692	207318	1	0	1	0	0.1218695	1	1	0.30102999	0.1218695
chr3	109955692	110091806	136115	1	0	0	0	0	1	1	0.30102999	0.30102999
chr3	110091806	110091865	60	1	0	0	0	0	2	1	0.61140001	0.30102999
chr3	110091865	110330579	238715	2	0	0	0	0	1	0	0.30102999	0
chr3	110330579	110619739	289161	4	0	0	0	0	3	1	0.93173516	0.30102999
chr3	110619739	110750728	130990	1	0	0	0	0	3	0	0.93173516	0
chr3	110750728	110796048	45321	2	0	0	0	0	3	1	0.93173516	0.30102999
chr3	110796048	110796107	60	1	0	0	0	0	3	2	0.93173516	0.61140001
chr3	110796107	110840981	44875	2	0	0	0	0	3	1	0.93173516	0.30102999
chr3	110840981	110853458	12478	2	1	0	0.02438896	0	3	2	0.51676182	0.61140001
chr3	110853458	110884292	30835	2	1	0	0.02438896	0	3	3	0.51676182	0.93173516
chr3	110884292	110991115	106824	2	1	0	0.02438896	0	3	2	0.51676182	0.61140001
chr3	110991115	110991174	60	1	1	0	0.02438896	0	3	4	0.51676182	1.26272838
chr3	110991174	111368682	377509	6	1	0	0.02438896	0	3	3	0.51676182	0.93173516
chr3	111368682	111398543	29862	2	1	0	0.01091641	0	4	3	0.76005302	0.93173516
chr3	111398543	111542889	144347	3	1	0	0.01091641	0	4	2	0.76005302	0.61140001

chr3	111542889	111685401	142513	5	2	0	0.0429175	0	4	2	0.47744371	0.61140001
chr3	111685401	111711404	26004	3	2	0	0.0429175	0	4	3	0.47744371	0.93173516
chr3	111711404	111729387	17984	3	2	0	0.0429175	0	4	4	0.47744371	1.26272838
chr3	111729387	111782247	52861	1	2	0	0.0429175	0	4	3	0.47744371	0.93173516
chr3	111782247	111812291	30045	2	2	0	0.0429175	0	4	4	0.47744371	1.26272838
chr3	111812291	111812349	59	1	2	0	0.0429175	0	4	5	0.47744371	1.60515106
chr3	111812349	111849865	37517	4	2	0	0.0429175	0	4	4	0.47744371	1.26272838
chr3	111849865	111887735	37871	1	2	0	0.0429175	0	4	3	0.47744371	0.93173516
chr3	111887735	111958710	70976	2	2	0	0.0429175	0	4	2	0.47744371	0.61140001
chr3	111958710	112051487	92778	3	2	0	0.0429175	0	4	3	0.47744371	0.93173516
chr3	112051487	112152600	101114	4	2	0	0.0429175	0	4	4	0.47744371	1.26272838
chr3	112152600	112329039	176440	5	2	0	0.02162467	0	5	4	0.68214471	1.26272838
chr3	112329039	112429568	100530	4	2	0	0.01053319	0	6	6	0.91219088	1.95986592
chr3	112429568	112576244	146677	2	2	0	0.02162467	0	5	6	0.68214471	1.95986592
chr3	112576244	112686563	110320	3	2	0	0.0429175	0	4	4	0.47744371	1.26272838
chr3	112686563	112711385	24823	2	2	0	0.0429175	0	4	5	0.47744371	1.60515106
chr3	112711385	112723889	12505	2	2	0	0.0429175	0	4	6	0.47744371	1.95986592
chr3	112723889	112872231	148343	2	2	0	0.0429175	0	4	5	0.47744371	1.60515106
chr3	112872231	112872290	60	1	2	0	0.02162467	0	5	5	0.68214471	1.60515106
chr3	112872290	113162786	290497	7	2	0	0.0429175	0	4	5	0.47744371	1.60515106
chr3	113162786	113225337	62552	2	3	0	0.05670724	0	5	5	0.45545077	1.60515106
chr3	113225337	113225396	60	1	3	0	0.03070643	0	6	5	0.63695542	1.60515106
chr3	113225396	113253681	28286	1	3	0	0.05670724	0	5	4	0.45545077	1.26272838
chr3	113253681	113309157	55477	2	3	0	0.05670724	0	5	3	0.45545077	0.93173516
chr3	113309157	113348129	38973	1	2	0	0.02162467	0	5	3	0.68214471	0.93173516
chr3	113348129	113410537	62409	2	2	0	0.01053319	0	6	3	0.91219088	0.93173516
chr3	113410537	113410596	60	1	2	0	0.01053319	0	6	5	0.91219088	1.60515106
chr3	113410596	113524332	113737	2	2	0	0.02162467	0	5	5	0.68214471	1.60515106
chr3	113524332	113524391	60	1	2	0	0.01053319	0	6	5	0.91219088	1.60515106
chr3	113524391	113679817	155427	3	2	0	0.02162467	0	5	3	0.68214471	0.93173516
chr3	113679817	113679876	60	1	3	0	0.05670724	0	5	3	0.45545077	0.93173516
chr3	113679876	113729283	49408	1	3	0	0.05670724	0	5	1	0.45545077	0.30102999
chr3	113729283	113729342	60	1	4	0	0.11390336	0	5	1	0.30102999	0.30102999
chr3	113729342	113897648	168307	6	3	0	0.05670724	0	5	1	0.45545077	0.30102999
chr3	113897648	114005609	107962	2	3	0	0.10122019	0	4	1	0.30102999	0.30102999
chr3	114005609	114005668	60	1	3	0	0.05670724	0	5	1	0.45545077	0.30102999
chr3	114005668	114134010	128343	4	3	0	0.10122019	0	4	1	0.30102999	0.30102999
chr3	114134010	114260384	126375	3	3	0	0.17593012	0	3	1	0.17593012	0.30102999
chr3	114260384	114260443	60	1	3	0	0.10122019	0	4	1	0.30102999	0.30102999
chr3	114260443	114419438	158996	3	3	0	0.17593012	0	3	1	0.17593012	0.30102999
chr3	114419438	114522521	103084	3	4	0	0.18734596	0	4	1	0.18734596	0.30102999
chr3	114522521	114737921	215401	4	3	0	0.10122019	0	4	1	0.30102999	0.30102999
chr3	114737921	114737980	60	1	3	0	0.10122019	0	4	2	0.30102999	0.61140001
chr3	114737980	114801176	63197	1	2	0	0.0429175	0	4	2	0.47744371	0.61140001
chr3	114801176	115046957	245782	4	2	0	0.0429175	0	4	0	0.47744371	0
chr3	115046957	115198347	151391	3	2	0	0.0429175	0	4	1	0.47744371	0.30102999
chr3	115198347	115198406	60	1	2	0	0.0429175	0	4	2	0.47744371	0.61140001
chr3	115198406	115373790	175385	3	2	0	0.0429175	0	4	1	0.47744371	0.30102999
chr3	115373790	115439972	66183	2	2	0	0.0429175	0	4	2	0.47744371	0.61140001
chr3	115439972	115440031	60	1	2	0	0.0429175	0	4	3	0.47744371	0.93173516
chr3	115440031	116085339	645309	12	2	0	0.0429175	0	4	1	0.47744371	0.30102999
chr3	116085339	116233223	147885	3	2	0	0.08289318	0	3	1	0.30102999	0.30102999
chr3	116233223	116460128	226906	3	1	0	0.02438896	0	3	1	0.51676182	0.30102999
chr3	116460128	116587439	127312	1	0	0	0	0	3	1	0.93173516	0.30102999
chr3	116587439	116615441	28003	1	0	0	0	0	3	0	0.93173516	0
chr3	116615441	116615500	60	1	1	0	0.02438896	0	3	0	0.51676182	0
chr3	116615500	116691055	75556	1	0	0	0	0	3	0	0.93173516	0
chr3	116691055	116795512	104458	2	0	0	0	0	3	1	0.93173516	0.30102999
chr3	116795512	116795571	60	1	1	0	0.02438896	0	3	1	0.51676182	0.30102999
chr3	116795571	116898300	102730	1	0	0	0	0	2	1	0.61140001	0.30102999
chr3	116898300	117166718	268419	1	0	0	0	0	1	1	0.30102999	0.30102999
chr3	117516385	117600150	83766	2	0	0	0	0	1	0	0.30102999	0
chr3	117600150	117780353	180204	2	0	0	0	0	1	1	0.30102999	0.30102999
chr3	117780353	117950181	169829	1	0	0	0	0	0	1	0	0.30102999
chr3	117950181	118159343	209163	2	0	0	0	0	0	2	0	0.61140001
chr3	118159343	118159402	60	1	0	0	0	0	2	5	0.61140001	1.60515106
chr3	118159402	118326121	166720	2	0	0	0	0	2	4	0.61140001	1.26272838
chr3	118326121	118408189	82069	2	0	0	0	0	2	5	0.61140001	1.60515106
chr3	118408189	118747220	339032	6	0	0	0	0	2	4	0.61140001	1.26272838
chr3	118747220	118824903	77684	3	0	1	0	0.01091641	3	4	0.93173516	0.76005302
chr3	118824903	118937563	112661	3	0	1	0	0.01091641	2	4	0.61140001	0.76005302
chr3	118937563	119112245	174683	5	0	2	0	0.02162467	3	5	0.93173516	0.68214471
chr3	119112245	119393750	281506	13	0	1	0	0.00478973	3	5	0.93173516	1.02643191
chr3	119393750	119500867	107118	3	0	1	0	0.01091641	3	4	0.93173516	0.76005302
chr3	119500867	119562169	61303	2	0	2	0	0.0429175	3	4	0.93173516	0.47744371
chr3	119562169	119562227	59	1	0	2	0	0.02162467	3	5	0.93173516	0.68214471
chr3	119562227	119720946	158720	3	0	1	0	0.01091641	3	4	0.93173516	0.76005302
chr3	119720946	119778347	57402	1	0	1	0	0.02438896	3	3	0.93173516	0.51676182
chr3	119778347	119812513	34167	2	0	1	0	0.01091641	3	4	0.93173516	0.76005302
chr3	119812513	119842433	29921	1	0	1	0	0.05404976	3	2	0.93173516	0.30102999
chr3	119842433	119903983	61551	2	0	1	0	0.05404976	5	2	1.60515106	0.30102999
chr3	119903983	119904042	60	1	0	1	0	0.05404976	6	2	1.95986592	0.30102999
chr3	119904042	119946568	42527	1	0	1	0	0.1218695	6	1	1.95986592	0.1218695
chr3	119946568	120005319	58752	2	1	1	0.00204627	0.1218695	6	1	1.31360226	0.1218695
chr3	120005319	120005377	59	1	1	1	0.00204627	0.02438896	6	3	1.31360226	0.51676182
chr3	120005377	120081841	76465	1	1	1	0.00204627	0.05404976	6	2	1.31360226	0.30102999
chr3	120081841	120163070	81230	3	1	1	0.00204627	0.1218695	6	1	1.31360226	0.1218695
chr3	120163070	120247727	84658	1	0	1	0	0.1218695	4	1	1.26272838	0.1218695
chr3	120247727	120247786	60	1	0	1	0	0.05404976	4	2	1.26272838	0.30102999
chr3	120247786	120420573	172788	1	0	1	0	0.05404976	3	2	0.93173516	0.30102999
chr3	120420573	120477195	56623	2	1	1	0.02438896	0.05404976	3	2	0.51676182	0.30102999
chr3	120477195	120558027	80833	2	1	1	0.02438896	0.02438896	3	3	0.51676182	0.51676182
chr3	120558027	120663836	105810	3	1	1	0.01091641	0.02438896	4	3	0.76005302	0.51676182
chr3	120663836	120874780	210945	4	1	1	0.01091641	0.05404976	4	2	0.76005302	0.30102999
chr3	120874780	120988822	114043	4	1	1	0.00478973	0.02438896	5	3	1.02643191	0.51676182
chr3	120988822	121150821	162000	3	1	1	0.01091641	0.02438896	4	3	0.76005302	0.51676182
chr3	121150821	121150880	60	1	1	2	0.01091641	0.0429175	4	4	0.76005302	0.47744371
chr3	121150880	121227636	76757	2	1	2	0.01091641	0.08289318	4	3	0.76005302	0.30102999
chr3	121227636	121227695	60	1	1	2	0.00478973	0.08289318	5	3	1.02643191	0.30102999
chr3	121227695	121382148	154454	4	1	1	0.01091641	0.02438896	4	3	0.76005302	0.51676182
chr3	121382148	121466988	84841	2	1	1	0.01091641	0.05404976	4	2	0.76005302	0.30102999
chr3	121466988	121517769	50782	1	1	1	0.01091641	0.1218695	4	1	0.76005302	0.1218695
chr3	121517769	121605050	87282	3	2	1	0.0429175	0.1218695	4	1	0.47744371	0.1218695
chr3	121605050	121641037	35988	2	2	1	0.02162467	0.1218695	5	1	0.68214471	0.1218695
chr3	121641037	121641096	60	1	2	2	0.02162467	0.30102999	5	1	0.68214471	0.05404976
chr3	121641096	121796778	155683	3	2	1	0.02162467	0.1218695	5	1	0.68214471	0.1218695
chr3	121796778	121908309	111532	3	2	1	0.02162467	0.05404976	5	2	0.68214471	0.30102999
chr3	121908309	121908368	60	1	2	1	0.02162467	0.02438896	5	3	0.68214471	0.51676182
chr3	121908368	121954321	45954	2	2	1	0.02162467	0.05404976	5	2	0.68214471	0.30102999
chr3	121954321	122044288	89968	2	2	1	0.0429175	0.05404976	4	2	0.47744371	0.30102999
chr3	122044288	122044347	60	1	2	1	0.0429175	0.02438896	4	3	0.47744371	0.51676182
chr3	122044347	122160985	116639	5	2	1	0.0429175	0.05404976	4	2	0.47744371	0.30102999
chr3	122160985	122288105	127121	3	2	1	0.0429175	0.1218695	4	1	0.47744371	0.1218695
chr3	122288105	122288164	60	1	2	1	0.0429175	0.05404976	4	2	0.47744371	0.30102999
chr3	122288164	122437797	149634	2	2	1	0.0429175	0.1218695	4	1	0.47744371	0.1218695
chr3	122437797	122437856	60	1	2	1	0.02162467	0.1218695	5	1	0.68214471	0.1218695
chr3	122437856	122514570	76715	2	2	1	0.0429175	0.1218695	4	1	0.47744371	0.1218695
chr3	122514570	122514629	60	1	3	1	0.10122019	0.1218695	4	1	0.30102999	0.1218695
chr3	122514629	122578912	64284	2	2	1	0.0429175	0.1218695	4	1	0.47744371	0.1218695
chr3	122578912	123083245	504334	14	2	1	0.0429175	0.05404976	4	2	0.47744371	0.30102999
chr3	123083245	123500428	417184	10	2	0	0.0429175	0	4	2	0.47744371	0.61140001
chr3	123500428	123531370	30943	2	2	1	0.0429175	0.05404976	4	2	0.47744371	0.30102999
chr3	123531370	123531429	60	1	2	1	0.0429175	0.02438896	4	3	0.47744371	0.51676182
chr3	123531429	123632380	100952	2	2	0	0.0429175	0	4	2	0.47744371	0.61140001

chr3	123632380	123632425	46	2	2	0	0.0429175	0	4	3	0.47744371	0.93173516
chr3	123632425	123649920	17496	1	2	0	0.0429175	0	4	2	0.47744371	0.61140001
chr3	123649920	123649979	60	1	3	1	0.10122019	0.02438896	4	3	0.30102999	0.51676182
chr3	123649979	123780392	130414	2	3	1	0.10122019	0.05404976	4	2	0.30102999	0.30102999
chr3	123780392	123902747	122356	2	3	0	0.10122019	0	4	1	0.30102999	0.30102999
chr3	123902747	124462924	560178	13	3	1	0.10122019	0.05404976	4	2	0.30102999	0.30102999
chr3	124462924	124502599	39676	3	3	2	0.10122019	0.1575501	4	2	0.30102999	0.1575501
chr3	124502599	124574302	71704	2	3	2	0.10122019	0.30102999	4	1	0.30102999	0.05404976
chr3	124574302	124646517	72216	2	3	1	0.17593012	0.30102999	3	0	0.17593012	0
chr3	124646517	124719924	73408	2	2	1	0.08289318	0.30102999	3	0	0.30102999	0
chr3	124719924	124759708	39785	2	2	1	0.08289318	0.1218695	3	1	0.30102999	0.1218695
chr3	124759708	124759767	60	1	2	1	0.08289318	0.05404976	3	2	0.30102999	0.30102999
chr3	124759767	124854580	94814	2	2	1	0.08289318	0.30102999	3	0	0.30102999	0
chr3	124854580	124854635	56	1	2	1	0.08289318	0.00478973	3	5	0.30102999	1.02643191
chr3	124854635	124951101	96467	2	2	0	0.08289318	0	3	3	0.30102999	0.93173516
chr3	124951101	124997963	46863	2	2	0	0.08289318	0	3	4	0.30102999	1.26272838
chr3	124997963	124998022	60	1	2	0	0.08289318	0	3	5	0.30102999	1.60515106
chr3	124998022	125193821	195800	4	2	0	0.08289318	0	3	4	0.30102999	1.26272838
chr3	125193821	125208259	14439	2	2	1	0.08289318	0.01091641	3	4	0.30102999	0.76005302
chr3	125208259	125267023	58765	2	2	1	0.08289318	0.02438896	3	3	0.30102999	0.51676182
chr3	125267023	125305163	38141	2	2	1	0.08289318	0.01091641	3	4	0.30102999	0.76005302
chr3	125305163	125395138	89976	1	2	0	0.1575501	0	2	3	0.1575501	0.93173516
chr3	125395138	125395197	60	1	3	0	0.30102999	0	2	3	0.08289318	0.93173516
chr3	125395197	125673197	278001	1	2	0	0.1575501	0	2	3	0.1575501	0.93173516
chr3	125673197	125725325	52129	2	3	1	0.17593012	0.00478973	3	5	0.17593012	1.02643191
chr3	125725325	125803478	78154	2	3	1	0.17593012	0.01091641	3	4	0.17593012	0.76005302
chr3	125803478	125831735	28258	2	3	2	0.17593012	0.02162467	3	5	0.17593012	0.68214471
chr3	125831735	125870295	38561	1	2	2	0.08289318	0.02162467	3	5	0.30102999	0.68214471
chr3	125870295	125870354	60	1	3	2	0.17593012	0.02162467	3	5	0.17593012	0.68214471
chr3	125870354	125928441	58088	2	3	1	0.17593012	0.00478973	3	5	0.17593012	1.02643191
chr3	125928441	126005754	77314	1	3	1	0.30102999	0.00478973	2	5	0.08289318	1.02643191
chr3	126005754	126005813	60	1	4	1	0.47744371	0.00478973	2	5	0.0429175	1.02643191
chr3	126005813	126116239	110427	2	4	1	0.47744371	0.01091641	2	4	0.0429175	0.76005302
chr3	126116239	126221984	105746	3	4	0	0.47744371	0	2	4	0.0429175	1.26272838
chr3	126221984	126235791	13808	2	4	0	0.47744371	0	2	5	0.0429175	1.60515106
chr3	126235791	126390714	154924	4	3	0	0.30102999	0	2	5	0.08289318	1.60515106
chr3	126390714	126501033	110320	2	3	0	0.30102999	0	2	4	0.08289318	1.26272838
chr3	126501033	126550275	49243	2	3	0	0.30102999	0	2	5	0.08289318	1.60515106
chr3	126550275	126550334	60	1	3	0	0.30102999	0	2	6	0.08289318	1.95986592
chr3	126550334	126587595	37262	1	3	0	0.30102999	0	2	5	0.08289318	1.60515106
chr3	126587595	126633495	45901	2	3	0	0.30102999	0	2	6	0.08289318	1.95986592
chr3	126633495	126714543	81049	3	4	0	0.47744371	0	2	6	0.0429175	1.95986592
chr3	126714543	126769875	55333	2	4	0	0.47744371	0	2	5	0.0429175	1.60515106
chr3	126769875	126769929	55	1	4	1	0.47744371	0.00204627	2	6	0.0429175	1.31360226
chr3	126769929	126835066	65138	1	4	1	0.47744371	0.00478973	2	5	0.0429175	1.02643191
chr3	126835066	126896029	60964	2	4	2	0.47744371	0.02162467	2	5	0.0429175	0.68214471
chr3	126896029	126922854	26826	2	4	2	0.47744371	0.01053319	2	6	0.0429175	0.91219088
chr3	126922854	127002510	79657	1	4	2	0.47744371	0.08289318	2	3	0.0429175	0.30102999
chr3	127002510	127033184	30675	2	4	2	0.30102999	0.08289318	3	3	0.10122019	0.30102999
chr3	127033184	127103505	70322	3	4	2	0.30102999	0.02162467	3	5	0.10122019	0.68214471
chr3	127103505	127200388	96884	2	4	2	0.30102999	0.0429175	3	4	0.10122019	0.47744371
chr3	127200388	127243729	43342	1	4	2	0.47744371	0.0429175	2	4	0.0429175	0.47744371
chr3	127243729	127243788	60	1	4	2	0.47744371	0.02162467	2	5	0.0429175	0.68214471
chr3	127243788	127307734	63947	2	4	2	0.47744371	0.0429175	2	4	0.0429175	0.47744371
chr3	127307734	127357611	49878	5	4	2	0.47744371	0.02162467	2	5	0.0429175	0.68214471
chr3	127357611	127385658	28048	2	4	2	0.30102999	0.02162467	3	5	0.10122019	0.68214471
chr3	127385658	127439881	54224	2	4	2	0.47744371	0.02162467	2	5	0.0429175	0.68214471
chr3	127439881	127476170	36290	2	4	2	0.30102999	0.02162467	3	5	0.10122019	0.68214471
chr3	127476170	127541176	65007	3	4	2	0.18734596	0.02162467	4	5	0.18734596	0.68214471
chr3	127541176	127553380	12205	1	3	2	0.10122019	0.02162467	4	5	0.30102999	0.68214471
chr3	127553380	127588039	34660	1	3	2	0.17593012	0.02162467	3	5	0.17593012	0.68214471
chr3	127588039	127588098	60	1	4	2	0.30102999	0.02162467	3	5	0.10122019	0.68214471
chr3	127588098	127647100	59003	1	3	2	0.17593012	0.02162467	3	5	0.17593012	0.68214471
chr3	127647100	127838206	191107	5	3	2	0.10122019	0.02162467	4	5	0.30102999	0.68214471
chr3	127838206	127838262	57	1	4	2	0.11390336	0.02162467	5	5	0.30102999	0.68214471
chr3	127838262	128016653	178392	3	3	2	0.17593012	0.02162467	3	5	0.17593012	0.68214471
chr3	128016653	128199479	182827	7	4	2	0.30102999	0.02162467	3	5	0.10122019	0.68214471
chr3	128199479	128199538	60	1	4	2	0.18734596	0.01053319	4	6	0.18734596	0.91219088
chr3	128199538	128210258	10721	1	4	2	0.30102999	0.01053319	3	6	0.10122019	0.91219088
chr3	128210258	128267008	56751	1	4	2	0.30102999	0.02162467	3	5	0.10122019	0.68214471
chr3	128267008	128338981	71974	3	4	2	0.18734596	0.02162467	4	5	0.18734596	0.68214471
chr3	128338981	128345649	6669	1	4	1	0.18734596	0.00478973	4	5	0.18734596	1.02643191
chr3	128345649	128400925	55277	1	3	1	0.10122019	0.00478973	4	5	0.30102999	1.02643191
chr3	128400925	128400984	60	1	3	2	0.10122019	0.01053319	4	6	0.30102999	0.91219088
chr3	128400984	128467632	66649	1	3	1	0.10122019	0.00478973	4	5	0.30102999	1.02643191
chr3	128467632	128494359	26728	2	4	1	0.18734596	0.00478973	4	5	0.18734596	1.02643191
chr3	128494359	128494418	60	1	4	2	0.18734596	0.02162467	4	5	0.18734596	0.68214471
chr3	128494418	128627419	133002	3	4	1	0.18734596	0.00478973	4	5	0.18734596	1.02643191
chr3	128627419	128686022	58604	1	4	1	0.47744371	0.00478973	2	5	0.0429175	1.02643191
chr3	128686022	128753853	67832	1	3	1	0.30102999	0.00478973	2	5	0.08289318	1.02643191
chr3	128753853	128779848	25996	3	3	1	0.10122019	0.00478973	4	5	0.30102999	1.02643191
chr3	128779848	128926481	146634	4	3	1	0.10122019	0.01091641	4	4	0.30102999	0.76005302
chr3	128926481	128926540	60	1	3	1	0.10122019	0.00478973	4	5	0.30102999	1.02643191
chr3	128926540	129070426	143887	3	3	1	0.17593012	0.00478973	3	5	0.17593012	1.02643191
chr3	129070426	129152947	82522	2	3	1	0.17593012	0.02438896	3	3	0.17593012	0.51676182
chr3	129152947	129504594	351648	10	3	1	0.17593012	0.05404976	3	2	0.17593012	0.30102999
chr3	129504594	129611238	106645	3	4	1	0.30102999	0.05404976	3	2	0.10122019	0.30102999
chr3	129611238	129689840	78603	1	4	1	0.30102999	0.1218695	3	1	0.10122019	0.1218695
chr3	129689840	130003817	313978	6	4	1	0.11390336	0.1218695	5	1	0.30102999	0.1218695
chr3	130003817	130088199	84383	2	4	1	0.11390336	0.05404976	5	2	0.30102999	0.30102999
chr3	130088199	130203237	115039	5	4	1	0.11390336	0.02438896	5	3	0.30102999	0.51676182
chr3	130203237	130329278	126042	2	3	1	0.05670724	0.05404976	5	2	0.45545077	0.30102999
chr3	130329278	130398054	68777	3	4	1	0.11390336	0.05404976	5	2	0.30102999	0.30102999
chr3	130398054	130464051	65998	2	4	1	0.18734596	0.05404976	4	2	0.18734596	0.30102999
chr3	130464051	130464110	60	1	4	1	0.11390336	0.05404976	5	2	0.30102999	0.30102999
chr3	130464110	130515007	50898	1	4	1	0.18734596	0.05404976	4	2	0.18734596	0.30102999
chr3	130515007	130515066	60	1	4	1	0.18734596	0.02438896	4	3	0.18734596	0.51676182
chr3	130515066	130570720	55655	1	3	1	0.10122019	0.02438896	4	3	0.30102999	0.51676182
chr3	130570720	130614137	43418	1	3	1	0.10122019	0.05404976	4	2	0.30102999	0.30102999
chr3	130614137	130670481	56345	2	4	1	0.18734596	0.05404976	4	2	0.18734596	0.30102999
chr3	130670481	130670540	60	1	5	1	0.30102999	0.02438896	4	3	0.11390336	0.51676182
chr3	130670540	130732881	62342	1	3	1	0.10122019	0.02438896	4	3	0.30102999	0.51676182
chr3	130732881	130739696	6816	3	3	3	0.10122019	0.17593012	4	3	0.30102999	0.17593012
chr3	130739696	130739755	60	1	5	3	0.30102999	0.17593012	4	3	0.11390336	0.17593012
chr3	130739755	130829076	89322	2	3	3	0.10122019	0.17593012	4	3	0.30102999	0.17593012
chr3	130829076	130893897	64822	1	3	2	0.10122019	0.08289318	4	3	0.30102999	0.30102999
chr3	130893897	130947406	53510	2	5	2	0.30102999	0.08289318	4	3	0.11390336	0.30102999
chr3	130947406	130992355	44950	2	5	2	0.30102999	0.0429175	4	4	0.11390336	0.47744371
chr3	130992355	131102638	110284	3	5	2	0.30102999	0.08289318	4	3	0.11390336	0.30102999
chr3	131102638	131158201	55564	2	5	2	0.30102999	0.0429175	4	4	0.11390336	0.47744371
chr3	131158201	131307282	149082	3	5	2	0.30102999	0.08289318	4	3	0.11390336	0.30102999
chr3	131307282	131404737	97456	2	4	2	0.18734596	0.08289318	4	3	0.18734596	0.30102999
chr3	131404737	131404796	60	1	4	2	0.11390336	0.08289318	5	3	0.30102999	0.30102999
chr3	131404796	131452933	48138	1	4	2	0.11390336	0.1575501	5	2	0.30102999	0.1575501
chr3	131452933	131452992	60	1	5	2	0.19510895	0.1575501	5	2	0.19510895	0.1575501
chr3	131452992	131488519	35528	1	5	1	0.19510895	0.05404976	5	2	0.19510895	0.30102999
chr3	131488519	131576658	88140	2	4	1	0.11390336	0.05404976	5	2	0.30102999	0.30102999
chr3	131576658	131624243	47586	2	4	2	0.11390336	0.08289318	5	3	0.30102999	0.30102999
chr3	131624243	131624296	54	1	5	2	0.19510895	0.08289318	5	3	0.19510895	0.30102999
chr3	131624296	131692577	68282	1	4	1	0.11390336	0.02438896	5	3	0.30102999	0.51676182
chr3	131692577	131875994	183418	3	3	1	0.10122019	0.30102999	4	0	0.30102999	0
chr3	131875994	131876053	60	1	4	1	0.18734596	0.30102999	4	0	0.18734596	0
chr3	131876053	131946312	70260	1	3	1	0.10122019	0.30102999	4	0	0.30102999	0
chr3	131946312	132036540	90229	2	3	1	0.05670724	0.30102999	5	0	0.45545077	0

chr3	132036540	132087048	50509	2	5	1	0.12309572	0.30102999	6	0	0.30102999	0
chr3	132087048	132278579	191532	5	5	1	0.07511598	0.30102999	7	0	0.43181735	0
chr3	132278579	132319193	40615	2	5	1	0.04407651	0.30102999	8	0	0.58747015	0
chr3	132319193	132319252	60	1	6	1	0.08122616	0.30102999	8	0	0.4250187	0
chr3	132319252	132432055	112804	4	6	1	0.129913	0.30102999	7	0	0.30102999	0
chr3	132432055	132542884	110830	3	6	1	0.08122616	0.30102999	8	0	0.4250187	0
chr3	132542884	132628311	85428	2	6	1	0.02793176	0.30102999	10	0	0.74627054	0
chr3	132628311	132711240	82930	2	7	1	0.03037338	0.30102999	11	0	0.73110763	0
chr3	132711240	132961762	250523	4	7	1	0.05232577	0.30102999	10	0	0.56314362	0
chr3	132961762	133124200	162439	5	7	1	0.03037338	0.30102999	11	0	0.73110763	0
chr3	133124200	133182993	58794	2	6	1	0.02793176	0.30102999	10	0	0.74627054	0
chr3	133182993	133183052	60	1	6	1	0.01518174	0.30102999	11	0	0.9449689	0
chr3	133183052	133305574	122523	2	6	1	0.02793176	0.30102999	10	0	0.74627054	0
chr3	133305574	133306067	494	2	6	1	0.01518174	0.30102999	11	0	0.9449689	0
chr3	133306067	133748256	442190	16	6	1	0.02793176	0.30102999	10	0	0.74627054	0
chr3	133748256	133825556	77301	1	6	1	0.04875589	0.30102999	9	0	0.5732208	0
chr3	133825556	133900325	74770	2	6	1	0.129913	0.30102999	7	0	0.30102999	0
chr3	133900325	133941520	41196	3	6	1	0.08122616	0.30102999	8	0	0.4250187	0
chr3	133941520	134075119	133600	2	6	1	0.129913	0.30102999	7	0	0.30102999	0
chr3	134075119	134120416	45298	2	6	1	0.20064824	0.30102999	6	0	0.20064824	0
chr3	134120416	134120475	60	1	6	1	0.04875589	0.30102999	9	0	0.5732208	0
chr3	134120475	134174021	53547	1	6	1	0.20064824	0.30102999	6	0	0.20064824	0
chr3	134174021	134204702	30682	2	5	1	0.19510895	0.30102999	5	0	0.19510895	0
chr3	134204702	134346680	141979	3	4	1	0.11390336	0.30102999	5	0	0.30102999	0
chr3	134346680	134567381	220702	3	4	0	0.11390336	0	5	0	0.30102999	0
chr3	134567381	134567440	60	1	4	0	0.11390336	0	5	1	0.30102999	0.30102999
chr3	134567440	134636064	68625	1	4	0	0.18734596	0	4	0	0.18734596	0
chr3	134636064	134843569	207506	4	3	0	0.10122019	0	4	0	0.30102999	0
chr3	134843569	135087408	243840	4	3	0	0.03070643	0	6	0	0.63695542	0
chr3	135087408	135470061	382654	2	1	0	0.01091641	0	4	0	0.76005302	0
chr3	135470061	135470120	60	1	2	1	0.0429175	0.1218695	4	1	0.47744371	0.1218695
chr3	135470120	135678138	208019	1	2	0	0.0429175	0	4	1	0.47744371	0.30102999
chr3	135678138	135721189	43052	2	2	0	0.02162467	0	5	1	0.68214471	0.30102999
chr3	135721189	135797226	76038	3	3	1	0.05670724	0.1218695	5	1	0.45545077	0.1218695
chr3	135797226	135797285	60	1	3	1	0.03070643	0.1218695	6	1	0.63695542	0.1218695
chr3	135797285	135904893	107609	2	2	0	0.01053319	0	6	0	0.91219088	0
chr3	135904893	135904952	60	1	2	0	0.00493743	0	7	0	1.16581773	0
chr3	135904952	136240172	335221	7	2	0	0.01053319	0	6	0	0.91219088	0
chr3	136240172	136240231	60	1	2	1	0.01053319	0.30102999	6	0	0.91219088	0
chr3	136240231	136413007	172777	3	2	0	0.01053319	0	6	0	0.91219088	0
chr3	136413007	136451394	38388	2	3	1	0.03070643	0.30102999	6	0	0.63695542	0
chr3	136451394	136713801	262408	5	3	0	0.03070643	0	6	0	0.63695542	0
chr3	136713801	136713860	60	1	3	2	0.03070643	0.30102999	6	1	0.63695542	0.05404976
chr3	136713860	136790987	77128	1	3	1	0.03070643	0.30102999	6	0	0.63695542	0
chr3	136790987	136791046	60	1	3	1	0.01598258	0.30102999	7	0	0.84395715	0
chr3	136791046	137018113	227068	1	3	1	0.03070643	0.30102999	6	0	0.63695542	0
chr3	137018113	137018172	60	1	4	1	0.03812622	0.30102999	7	0	0.60763643	0
chr3	137018172	137208729	190558	1	3	1	0.01598258	0.30102999	7	0	0.84395715	0
chr3	137208729	137208788	60	1	5	1	0.04407651	0.30102999	8	0	0.58747015	0
chr3	137208788	137362614	153827	1	5	1	0.12309572	0.30102999	6	0	0.30102999	0
chr3	137362614	137484278	121665	2	6	1	0.20064824	0.30102999	6	0	0.20064824	0
chr3	137484278	137484323	46	1	7	1	0.30102999	0.30102999	6	0	0.129913	0
chr3	137484323	137567648	83326	1	7	0	0.30102999	0	6	0	0.129913	0
chr3	137567648	137739189	171542	2	6	0	0.20064824	0	6	0	0.20064824	0
chr3	137739189	137811282	72094	3	6	0	0.129913	0	7	0	0.30102999	0
chr3	137811282	137942317	131036	3	5	0	0.12309572	0	6	0	0.30102999	0
chr3	137942317	138027470	85154	3	6	1	0.20064824	0.30102999	6	0	0.20064824	0
chr3	138027470	138043871	16402	2	8	1	0.30102999	0.30102999	7	0	0.13499366	0
chr3	138043871	138116821	72951	2	7	0	0.20469099	0	7	0	0.20469099	0
chr3	138116821	138161861	45041	3	8	1	0.30102999	0.30102999	7	0	0.13499366	0
chr3	138161861	138248190	86330	2	7	1	0.20469099	0.30102999	7	0	0.20469099	0
chr3	138248190	138248249	60	1	8	2	0.20764654	0.61140001	8	0	0.20764654	0
chr3	138248249	138330031	81783	3	8	1	0.20764654	0.30102999	8	0	0.20764654	0
chr3	138330031	138373980	43950	2	7	1	0.13499366	0.30102999	8	0	0.30102999	0
chr3	138373980	138374039	60	1	8	1	0.20764654	0.30102999	8	0	0.20764654	0
chr3	138374039	138598678	224640	3	7	1	0.13499366	0.30102999	8	0	0.30102999	0
chr3	138598678	138598737	60	1	8	2	0.20764654	0.61140001	8	0	0.20764654	0
chr3	138598737	138663176	64440	1	8	2	0.30102999	0.61140001	7	0	0.13499366	0
chr3	138663176	138734331	71156	1	7	2	0.20469099	0.61140001	7	0	0.20469099	0
chr3	138734331	138759414	25084	2	7	2	0.20469099	0.30102999	7	1	0.20469099	0.05404976
chr3	138759414	138824857	65444	1	6	2	0.129913	0.61140001	7	0	0.30102999	0
chr3	138824857	138884907	60051	2	6	2	0.08122616	0.61140001	8	0	0.4250187	0
chr3	138884907	138884966	60	1	7	2	0.13499366	0.61140001	8	0	0.30102999	0
chr3	138884966	138955020	70055	1	6	2	0.129913	0.61140001	7	0	0.30102999	0
chr3	138955020	139026985	71966	2	6	2	0.129913	0.30102999	7	1	0.30102999	0.05404976
chr3	139026985	139075748	48764	1	6	1	0.129913	0.30102999	7	0	0.30102999	0
chr3	139075748	139106930	31183	2	7	1	0.20469099	0.30102999	7	0	0.20469099	0
chr3	139106930	139106989	60	1	7	1	0.20469099	0.1218695	7	1	0.20469099	0.1218695
chr3	139106989	139236275	129287	2	6	1	0.20064824	0.30102999	6	0	0.20064824	0
chr3	139236275	139244030	7756	2	8	1	0.20764654	0.30102999	8	0	0.20764654	0
chr3	139244030	139279676	35647	1	5	1	0.04407651	0.30102999	8	0	0.58747015	0
chr3	139279676	139346029	66354	2	4	1	0.02074938	0.30102999	8	0	0.79906872	0
chr3	139346029	139346088	60	1	5	1	0.04407651	0.30102999	8	0	0.58747015	0
chr3	139346088	139517043	170956	2	4	1	0.02074938	0.30102999	8	0	0.79906872	0
chr3	139517043	139517102	60	1	5	1	0.04407651	0.30102999	8	0	0.58747015	0
chr3	139517102	139652108	135007	1	4	1	0.02074938	0.30102999	8	0	0.79906872	0
chr3	139652108	139652167	60	1	4	1	0.02074938	0.05404976	8	2	0.79906872	0.30102999
chr3	139652167	139688200	36034	1	4	1	0.02074938	0.1218695	8	1	0.79906872	0.1218695
chr3	139688200	139773831	85632	3	5	1	0.04407651	0.1218695	8	1	0.58747015	0.1218695
chr3	139773831	139894784	120954	4	6	1	0.08122616	0.1218695	8	1	0.4250187	0.1218695
chr3	139894784	139950208	55425	2	7	1	0.13499366	0.1218695	8	1	0.30102999	0.1218695
chr3	139950208	140185431	235224	4	6	1	0.08122616	0.30102999	8	0	0.4250187	0
chr3	140185431	140185482	52	1	7	1	0.13499366	0.30102999	8	0	0.30102999	0
chr3	140185482	140331909	146428	3	6	1	0.08122616	0.30102999	8	0	0.4250187	0
chr3	140331909	140402569	70661	2	7	1	0.13499366	0.30102999	8	0	0.30102999	0
chr3	140402569	140406803	4235	2	8	1	0.20764654	0.30102999	8	0	0.20764654	0
chr3	140406803	140636967	230165	2	7	1	0.13499366	0.30102999	8	0	0.30102999	0
chr3	140636967	140637026	60	1	8	1	0.13872638	0.30102999	9	0	0.30102999	0
chr3	140637026	140682697	45672	1	6	1	0.04875589	0.30102999	9	0	0.5732208	0
chr3	140682697	140695270	12574	1	6	1	0.08122616	0.30102999	8	0	0.4250187	0
chr3	140695270	140772392	77123	1	5	1	0.04407651	0.30102999	8	0	0.58747015	0
chr3	140772392	140772451	60	1	5	1	0.02473314	0.30102999	9	0	0.76806864	0
chr3	140772451	140823922	51472	1	5	1	0.04407651	0.30102999	8	0	0.58747015	0
chr3	140823922	140823981	60	1	6	1	0.08122616	0.1218695	8	1	0.4250187	0.1218695
chr3	140823981	140867384	43404	1	5	1	0.04407651	0.1218695	8	1	0.58747015	0.1218695
chr3	140867384	140867443	60	1	5	2	0.02473314	0.30102999	9	1	0.76806864	0.05404976
chr3	140867443	140969043	101601	1	5	1	0.02473314	0.1218695	9	1	0.76806864	0.1218695
chr3	140969043	141073018	103976	3	7	1	0.08584816	0.1218695	9	1	0.42015402	0.1218695
chr3	141073018	141073077	60	1	7	2	0.08584816	0.30102999	9	1	0.42015402	0.05404976
chr3	141073077	141201268	128192	2	6	2	0.04875589	0.30102999	9	1	0.5732208	0.05404976
chr3	141201268	141248662	47395	2	7	2	0.08584816	0.30102999	9	1	0.42015402	0.05404976
chr3	141248662	141289786	41125	1	6	2	0.04875589	0.30102999	9	1	0.5732208	0.05404976
chr3	141289786	141328804	39019	2	6	2	0.02793176	0.30102999	10	1	0.74627054	0.05404976
chr3	141328804	141385743	56940	1	6	2	0.04875589	0.30102999	9	1	0.5732208	0.05404976
chr3	141385743	141459467	73725	2	7	2	0.08584816	0.30102999	9	1	0.42015402	0.05404976
chr3	141459467	141596567	137101	5	7	2	0.05232577	0.30102999	10	1	0.56314362	0.05404976
chr3	141596567	141671185	74619	2	7	2	0.08584816	0.30102999	9	1	0.42015402	0.05404976
chr3	141671185	141841093	169909	6	7	2	0.05232577	0.30102999	10	1	0.56314362	0.05404976
chr3	141841093	141896333	55241	2	6	2	0.02793176	0.30102999	10	1	0.74627054	0.05404976
chr3	141896333	141953305	56973	4	7	2	0.05232577	0.30102999	10	1	0.56314362	0.05404976
chr3	141953305	142278146	324842	8	6	2	0.02793176	0.30102999	10	1	0.74627054	0.05404976
chr3	142278146	142342355	64210	1	6	2	0.02793176	0.61140001	10	0	0.74627054	0
chr3	142342355	142368809	26455	2	7	2	0.05232577	0.30102999	10	1	0.56314362	0.05404976
chr3	142368809	142442647	73839	2	7	2	0.05232577	0.61140001	10	0	0.56314362	0

chr3	142442647	142512674	70028	2	6	2	0.02793176	0.61140001	10	0	0.74627054	0
chr3	142512674	142512733	60	1	6	2	0.02793176	0.30102999	10	1	0.74627054	0.05404976
chr3	142512733	142577172	64440	2	6	2	0.02793176	0.61140001	10	0	0.74627054	0
chr3	142577172	142723924	146753	4	6	2	0.01518174	0.61140001	11	0	0.9449689	0
chr3	142723924	142723983	60	1	8	2	0.05490675	0.61140001	11	0	0.55623409	0
chr3	142723983	142779437	55455	1	7	2	0.03037338	0.61140001	11	0	0.73110763	0
chr3	142779437	142894708	115272	2	6	2	0.01518174	0.61140001	11	0	0.9449689	0
chr3	142894708	142984456	89749	2	5	2	0.00666883	0.61140001	11	0	1.20557689	0
chr3	142984456	143156586	172131	3	4	2	0.02074938	0.61140001	8	0	0.79906872	0
chr3	143156586	143156645	60	1	5	2	0.04407651	0.61140001	8	0	0.58747015	0
chr3	143156645	143212526	55882	1	5	2	0.12309572	0.61140001	6	0	0.30102999	0
chr3	143212526	143271117	58592	1	5	2	0.19510895	0.61140001	5	0	0.19510895	0
chr3	143271117	143271176	60	1	5	2	0.04407651	0.61140001	8	0	0.58747015	0
chr3	143271176	143337628	66453	1	5	1	0.04407651	0.30102999	8	0	0.58747015	0
chr3	143337628	143375800	38173	1	4	1	0.06713722	0.30102999	6	0	0.44141547	0
chr3	143375800	143498841	123042	4	4	1	0.06713722	0.1218695	6	1	0.44141547	0.1218695
chr3	143498841	143722633	223793	5	4	1	0.06713722	0.30102999	6	0	0.44141547	0
chr3	143722633	144065039	342407	3	4	0	0.06713722	0	6	0	0.44141547	0
chr3	144065039	144065098	60	1	4	0	0.03812622	0	7	0	0.60763643	0
chr3	144065098	144163463	98366	2	2	0	0.01053319	0	6	0	0.91219088	0
chr3	144163463	144344443	180981	1	2	0	0.0429175	0	4	0	0.47744371	0
chr3	144344443	145050142	705700	4	2	0	0.08289318	0	3	0	0.30102999	0
chr3	145050142	145050201	60	1	3	1	0.10122019	0.30102999	4	0	0.30102999	0
chr3	145050201	145399406	349206	2	3	0	0.17593012	0	3	0	0.17593012	0
chr3	145399406	145519243	119838	2	4	1	0.06713722	0.30102999	6	0	0.44141547	0
chr3	145519243	145783995	264753	2	3	0	0.03070643	0	6	0	0.63695542	0
chr3	145783995	145784054	60	1	4	1	0.03812622	0.30102999	7	0	0.60763643	0
chr3	145784054	145938580	154527	3	3	1	0.05670724	0.30102999	5	0	0.45545077	0
chr3	145938580	145980330	41751	3	4	1	0.11390336	0.30102999	5	0	0.30102999	0
chr3	145980330	146064060	83731	1	4	1	0.18734596	0.30102999	4	0	0.18734596	0
chr3	146064060	146151230	87171	2	5	1	0.19510895	0.30102999	5	0	0.19510895	0
chr3	146151230	146151289	60	1	6	1	0.129913	0.30102999	7	0	0.30102999	0
chr3	146151289	146213605	62317	1	5	1	0.19510895	0.30102999	5	0	0.19510895	0
chr3	146213605	146246235	32631	1	5	0	0.19510895	0	5	0	0.19510895	0
chr3	146246235	146323213	76979	4	6	0	0.129913	0	7	0	0.30102999	0
chr3	146323213	146619160	295948	2	6	0	0.20064824	0	6	0	0.20064824	0
chr3	146619160	146760112	140953	3	6	0	0.04875589	0	9	0	0.5732208	0
chr3	146760112	146958826	198715	1	6	0	0.129913	0	7	0	0.30102999	0
chr3	146958826	146958885	60	1	6	0	0.129913	0	7	1	0.30102999	0.30102999
chr3	146958885	147041039	82155	1	6	0	0.20064824	0	6	1	0.20064824	0.30102999
chr3	147041039	147130658	89620	3	4	0	0.11390336	0	5	1	0.30102999	0.30102999
chr3	147130658	147130717	60	1	6	0	0.20064824	0	6	1	0.20064824	0.30102999
chr3	147130717	147219530	88814	1	5	0	0.12309572	0	6	1	0.30102999	0.30102999
chr3	147219530	147370868	151339	1	5	0	0.19510895	0	5	1	0.19510895	0.30102999
chr3	147370868	147370927	60	1	5	0	0.12309572	0	6	1	0.30102999	0.30102999
chr3	147370927	147514205	143279	1	5	0	0.19510895	0	5	1	0.19510895	0.30102999
chr3	147514205	147514264	60	1	5	0	0.12309572	0	6	1	0.30102999	0.30102999
chr3	147514264	147625502	111239	1	4	0	0.06713722	0	6	0	0.44141547	0
chr3	147625502	147719481	93980	2	5	0	0.12309572	0	6	1	0.30102999	0.30102999
chr3	147719481	147719540	60	1	6	1	0.129913	0.1218695	7	1	0.30102999	0.1218695
chr3	147719540	147857159	137620	1	6	0	0.129913	0	7	0	0.30102999	0
chr3	147857159	147857218	60	1	8	0	0.13872638	0	9	1	0.30102999	0.30102999
chr3	147857218	147963917	106700	2	7	0	0.08584816	0	9	1	0.42015402	0.30102999
chr3	147963917	147963976	60	1	8	0	0.13872638	0	9	2	0.30102999	0.61140001
chr3	147963976	148118658	154683	1	8	0	0.13872638	0	9	1	0.30102999	0.30102999
chr3	148118658	148118717	60	1	9	0	0.09154957	0	11	1	0.41444892	0.30102999
chr3	148118717	148247003	128287	1	8	0	0.05490675	0	11	1	0.55623409	0.30102999
chr3	148247003	148425748	178746	2	8	0	0.05490675	0	11	0	0.55623409	0
chr3	148425748	148491324	65577	3	9	0	0.09154957	0	11	0	0.41444892	0
chr3	148491324	148577961	86638	4	10	0	0.14303407	0	11	0	0.30102999	0
chr3	148577961	148965690	387730	14	9	0	0.09154957	0	11	0	0.41444892	0
chr3	148965690	149211013	245324	6	10	0	0.14303407	0	11	0	0.30102999	0
chr3	149211013	149307704	96692	4	11	0	0.21200206	0	11	0	0.21200206	0
chr3	149307704	149368351	60648	2	11	1	0.14385241	0.30102999	12	0	0.30102999	0
chr3	149368351	149414576	46226	1	11	0	0.14385241	0	12	0	0.30102999	0
chr3	149414576	149458747	44172	2	12	0	0.21227046	0	12	0	0.21227046	0
chr3	149458747	149458806	60	1	12	0	0.14385241	0	13	0	0.30102999	0
chr3	149458806	149514722	55917	2	12	0	0.21227046	0	12	0	0.21227046	0
chr3	149514722	149589811	75090	1	12	0	0.30102999	0	11	0	0.14385241	0
chr3	149589811	149749169	159359	5	13	0	0.21200206	0	13	0	0.21200206	0
chr3	149749169	149961703	212535	2	13	0	0.41271556	0	11	0	0.09334429	0
chr3	149961703	149961762	60	1	13	1	0.41271556	0.30102999	11	0	0.09334429	0
chr3	149961762	150156002	194241	2	12	1	0.41314172	0.30102999	10	0	0.09290028	0
chr3	150156002	150156059	58	1	12	1	0.21227046	0.1218695	12	1	0.21227046	0.1218695
chr3	150156059	150177694	21636	2	12	1	0.30102999	0.1218695	11	1	0.14385241	0.1218695
chr3	150177694	150293480	115787	4	12	1	0.30102999	0.05404976	11	2	0.14385241	0.30102999
chr3	150293480	150461964	168485	5	12	1	0.41314172	0.05404976	10	2	0.09290028	0.30102999
chr3	150461964	150587537	125574	2	11	1	0.30102999	0.1218695	10	1	0.14303407	0.1218695
chr3	150587537	150622480	34944	2	11	1	0.21200206	0.1218695	11	1	0.21200206	0.1218695
chr3	150622480	150651767	29288	3	12	1	0.30102999	0.1218695	11	1	0.14385241	0.1218695
chr3	150651767	150690789	39023	1	12	1	0.41314172	0.1218695	10	1	0.09290028	0.1218695
chr3	150690789	150769222	78434	1	12	1	0.41314172	0.30102999	10	0	0.09290028	0
chr3	150769222	150983589	214368	8	12	1	0.30102999	0.30102999	11	0	0.14385241	0
chr3	150983589	151156344	172756	10	12	1	0.30102999	0.1218695	11	1	0.14385241	0.1218695
chr3	151156344	151167962	11619	1	11	1	0.21200206	0.1218695	11	1	0.21200206	0.1218695
chr3	151167962	151287291	119330	1	10	0	0.14303407	0	11	1	0.30102999	0.30102999
chr3	151287291	151441231	153941	3	11	0	0.21200206	0	11	1	0.21200206	0.30102999
chr3	151441231	151531699	90469	1	11	0	0.21200206	0	11	0	0.21200206	0
chr3	151531699	151599643	67945	4	12	0	0.30102999	0	11	0	0.14385241	0
chr3	151599643	151970271	370629	2	10	0	0.14303407	0	11	0	0.30102999	0
chr3	151970271	151970330	60	1	11	0	0.21200206	0	11	0	0.21200206	0
chr3	151970330	152016859	46530	1	10	0	0.14303407	0	11	0	0.30102999	0
chr3	152016859	152101637	84779	2	8	0	0.20764654	0	8	0	0.20764654	0
chr3	152101637	152101696	60	1	8	0	0.13872638	0	9	0	0.30102999	0
chr3	152101696	152309548	207853	3	6	0	0.129913	0	7	0	0.30102999	0
chr3	152309548	152424263	114716	2	8	0	0.30102999	0	7	0	0.13499366	0
chr3	152424263	152510621	86359	2	8	0	0.20764654	0	8	0	0.20764654	0
chr3	152510621	152510680	60	1	9	0	0.20975986	0	9	0	0.20975986	0
chr3	152510680	152555518	44839	1	8	0	0.13872638	0	9	0	0.30102999	0
chr3	152555518	152555577	60	1	9	0	0.20975986	0	9	0	0.20975986	0
chr3	152555577	152655882	100306	1	6	0	0.08122616	0	8	0	0.4250187	0
chr3	152655882	152771666	115785	1	5	0	0.04407651	0	8	0	0.58747015	0
chr3	152771666	152881655	109990	2	8	0	0.13872638	0	9	0	0.30102999	0
chr3	152881655	152881714	60	1	11	0	0.30102999	0	10	0	0.14303407	0
chr3	152881714	152940063	58350	1	10	0	0.30102999	0	9	0	0.14135546	0
chr3	152940063	153870694	930632	12	9	0	0.20975986	0	9	0	0.20975986	0
chr3	153870694	154002390	131697	3	8	0	0.13872638	0	9	0	0.30102999	0
chr3	154002390	154268572	266183	6	9	0	0.20975986	0	9	0	0.20975986	0
chr3	154268572	155106491	837920	8	9	0	0.42015402	0	7	0	0.08584816	0
chr3	155106491	155161736	55246	2	10	0	0.4167287	0	8	0	0.08923391	0
chr3	155161736	155241172	79437	2	12	0	0.72109894	0	8	0	0.03209037	0
chr3	155241172	155306696	65525	1	11	0	0.55623409	0	8	0	0.05490675	0
chr3	155306696	155353417	46722	2	11	0	0.41444892	0	9	0	0.09154957	0
chr3	155353417	155406126	52710	2	11	0	0.30102999	0	10	0	0.14303407	0
chr3	155406126	155406185	60	1	12	0	0.30102999	0	11	0	0.14385241	0
chr3	155406185	155539979	133795	3	11	0	0.21200206	0	11	0	0.21200206	0
chr3	155539979	155637577	97599	4	11	0	0.30102999	0	10	0	0.14303407	0
chr3	155637577	155944603	307027	5	11	0	0.41444892	0	9	0	0.09154957	0
chr3	155944603	155976549	31947	2	12	0	0.55190077	0	9	0	0.0565833	0
chr3	155976549	156070811	94263	2	11	0	0.41444892	0	9	0	0.09154957	0
chr3	156070811	156070870	60	1	12	0	0.30102999	0	11	0	0.14385241	0
chr3	156070870	156194659	123790	3	12	0	0.41314172	0	10	0	0.09290028	0
chr3	156194659	156399266	204608	6	12	0	0.30102999	0	11	0	0.14385241	0
chr3	156399266	156500239	100974	2	12	0	0.41314172	0	10	0	0.09290028	0

chr3	156500239	156543647	43409	1	12	0	0.55190077	0	9	0	0.0565833	0
chr3	156543647	156606494	62848	1	11	0	0.41444892	0	9	0	0.09154957	0
chr3	156606494	156712591	106098	3	11	0	0.30102999	0	10	0	0.14303407	0
chr3	156712591	156712650	60	1	12	0	0.30102999	0	11	0	0.14385241	0
chr3	156712650	156869731	157082	3	11	0	0.21200206	0	11	0	0.21200206	0
chr3	156869731	156932441	62711	1	11	0	0.30102999	0	10	0	0.14303407	0
chr3	156932441	157082218	149778	4	12	0	0.41314172	0	10	0	0.09290028	0
chr3	157082218	157311107	228890	5	11	0	0.30102999	0	10	0	0.14303407	0
chr3	157311107	157311166	60	1	12	0	0.41314172	0	10	0	0.09290028	0
chr3	157311166	157649680	338515	3	11	0	0.30102999	0	10	0	0.14303407	0
chr3	157649680	157649739	60	1	11	0	0.21200206	0	11	0	0.21200206	0
chr3	157649739	157716637	66899	1	11	0	0.30102999	0	10	0	0.14303407	0
chr3	157716637	157839904	123268	2	10	0	0.21118145	0	10	0	0.21118145	0
chr3	157839904	158210336	370433	9	10	0	0.14303407	0	11	0	0.30102999	0
chr3	158210336	158272621	62286	1	10	0	0.21118145	0	10	0	0.21118145	0
chr3	158272621	158272680	60	1	10	0	0.14303407	0	11	0	0.30102999	0
chr3	158272680	158414913	142234	7	10	0	0.21118145	0	10	0	0.21118145	0
chr3	158414913	158546836	131924	6	11	0	0.21200206	0	11	0	0.21200206	0
chr3	158546836	158730176	183341	1	11	0	0.30102999	0	10	0	0.14303407	0
chr3	158730176	158786919	56744	2	11	0	0.21200206	0	11	0	0.21200206	0
chr3	158786919	158786978	60	1	12	0	0.14385241	0	13	0	0.30102999	0
chr3	158786978	158955242	168265	4	11	0	0.21200206	0	11	0	0.21200206	0
chr3	158955242	159012665	57424	3	12	0	0.30102999	0	11	0	0.14385241	0
chr3	159012665	159164049	151385	3	11	0	0.21200206	0	11	0	0.21200206	0
chr3	159164049	159198524	34476	2	11	0	0.14385241	0	12	0	0.30102999	0
chr3	159198524	159280043	81520	2	11	0	0.21200206	0	11	0	0.21200206	0
chr3	159280043	159328038	47996	1	11	0	0.30102999	0	10	0	0.14303407	0
chr3	159328038	159477095	149058	4	11	0	0.21200206	0	11	0	0.21200206	0
chr3	159477095	159662649	185555	5	12	0	0.30102999	0	11	0	0.14385241	0
chr3	159662649	159849796	187148	5	12	0	0.21227046	0	12	0	0.21227046	0
chr3	159849796	159984949	135154	2	11	0	0.14385241	0	12	0	0.30102999	0
chr3	159984949	159985008	60	1	11	0	0.09334429	0	13	0	0.41271556	0
chr3	159985008	160085301	100294	2	11	0	0.14385241	0	12	0	0.30102999	0
chr3	160085301	160279160	193860	7	11	0	0.21200206	0	11	0	0.21200206	0
chr3	160279160	160279219	60	1	11	0	0.14385241	0	12	0	0.30102999	0
chr3	160279219	160679651	400433	7	11	0	0.21200206	0	11	0	0.21200206	0
chr3	160679651	160679710	60	1	11	0	0.14385241	0	12	0	0.30102999	0
chr3	160679710	161075157	395448	7	11	0	0.21200206	0	11	0	0.21200206	0
chr3	161075157	161123418	48262	1	11	0	0.55623409	0	8	0	0.05490675	0
chr3	161123418	161216835	93418	2	9	0	0.30102999	0	8	0	0.13872638	0
chr3	161216835	161537949	321115	2	8	0	0.20764654	0	8	0	0.20764654	0
chr3	161537949	161903614	365666	2	7	0	0.13499366	0	8	0	0.30102999	0
chr3	161903614	162262697	359084	2	3	0	0.05670724	0	5	0	0.45545077	0
chr3	162262697	162262756	60	1	3	0	0.03070643	0	6	0	0.63695542	0
chr3	162262756	162725478	462723	4	3	0	0.05670724	0	5	0	0.45545077	0
chr3	162725478	162725537	60	1	5	0	0.12309572	0	6	0	0.30102999	0
chr3	162725537	162928148	202612	2	5	0	0.19510895	0	5	0	0.19510895	0
chr3	162928148	162928207	60	1	5	0	0.12309572	0	6	0	0.30102999	0
chr3	162928207	163133456	205250	2	5	0	0.19510895	0	5	0	0.19510895	0
chr3	163133456	163482098	348643	5	6	0	0.30102999	0	5	0	0.12309572	0
chr3	163482098	163532726	50629	1	5	0	0.19510895	0	5	0	0.19510895	0
chr3	163532726	163643791	111066	1	5	0	0.30102999	0	4	0	0.11390336	0
chr3	163643791	163643850	60	1	6	0	0.44141547	0	4	0	0.06713722	0
chr3	163643850	163768664	124815	2	5	0	0.30102999	0	4	0	0.11390336	0
chr3	163768664	163926377	157714	4	5	0	0.19510895	0	5	0	0.19510895	0
chr3	163926377	163926436	60	1	6	0	0.30102999	0	5	0	0.12309572	0
chr3	163926436	164370152	443717	6	5	0	0.30102999	0	4	0	0.11390336	0
chr3	164370152	164370211	60	1	5	0	0.19510895	0	5	0	0.19510895	0
chr3	164370211	164569733	199523	1	5	0	0.30102999	0	4	0	0.11390336	0
chr3	164569733	164569792	60	1	6	0	0.44141547	0	4	1	0.06713722	0.30102999
chr3	164569792	164710200	140409	1	6	0	0.44141547	0	4	0	0.06713722	0
chr3	164710200	164863594	153395	3	5	0	0.30102999	0	4	0	0.11390336	0
chr3	164863594	164912215	48622	2	6	0	0.44141547	0	4	0	0.06713722	0
chr3	164912215	165013751	101537	2	7	0	0.60763643	0	4	0	0.03812622	0
chr3	165013751	165013810	60	1	7	0	0.60763643	0	4	1	0.03812622	0.30102999
chr3	165013810	165119608	105799	1	5	0	0.45545077	0	3	1	0.05670724	0.30102999
chr3	165119608	165385037	265430	2	5	0	0.45545077	0	3	0	0.05670724	0
chr3	165385037	165655987	270951	4	6	0	0.63695542	0	3	0	0.03070643	0
chr3	165655987	165806091	150105	2	6	0	0.44141547	0	4	0	0.06713722	0
chr3	165806091	165806150	60	1	7	0	0.30102999	0	6	0	0.129913	0
chr3	165806150	165891343	85194	1	5	0	0.30102999	0	4	0	0.11390336	0
chr3	165891343	166121899	230557	2	4	0	0.30102999	0	3	0	0.10122019	0
chr3	166121899	166121958	60	1	9	0	0.42015402	0	7	0	0.08584816	0
chr3	166121958	166269102	147145	1	6	0	0.129913	0	7	0	0.30102999	0
chr3	166269102	166269161	60	1	7	0	0.20469099	0	7	1	0.20469099	0.30102999
chr3	166269161	166471358	202198	1	6	0	0.20064824	0	6	1	0.20064824	0.30102999
chr3	166471358	166527551	56194	2	7	0	0.20469099	0	7	1	0.20469099	0.30102999
chr3	166527551	166659726	132176	1	7	0	0.30102999	0	6	1	0.129913	0.30102999
chr3	166659726	166918267	258542	3	7	0	0.20469099	0	7	1	0.20469099	0.30102999
chr3	166918267	166918326	60	1	7	1	0.20469099	0.1218695	7	1	0.20469099	0.1218695
chr3	166918326	167090692	172367	4	7	0	0.20469099	0	7	1	0.20469099	0.30102999
chr3	167090692	167159761	69070	2	6	0	0.129913	0	7	1	0.30102999	0.30102999
chr3	167159761	167189506	29746	2	6	1	0.129913	0.1218695	7	1	0.30102999	0.1218695
chr3	167189506	167287982	98477	2	6	0	0.129913	0	7	1	0.30102999	0.30102999
chr3	167287982	167371614	83633	3	7	0	0.20469099	0	7	1	0.20469099	0.30102999
chr3	167371614	167540761	169148	7	9	0	0.42015402	0	7	1	0.08584816	0.30102999
chr3	167540761	167776506	235746	4	9	1	0.42015402	0.1218695	7	1	0.08584816	0.1218695
chr3	167776506	167837277	60772	2	9	1	0.30102999	0.1218695	8	1	0.13872638	0.1218695
chr3	167837277	167837336	60	1	11	1	0.41444892	0.1218695	9	1	0.09154957	0.1218695
chr3	167837336	167978166	140831	1	11	0	0.41444892	0	9	1	0.09154957	0.30102999
chr3	167978166	167978225	60	1	12	0	0.30102999	0	11	1	0.14385241	0.30102999
chr3	167978225	168219616	241392	2	12	0	0.41314172	0	10	1	0.09290028	0.30102999
chr3	168219616	168274402	54787	2	12	0	0.41314172	0	10	0	0.09290028	0
chr3	168274402	168347450	73049	2	12	0	0.41314172	0	10	1	0.09290028	0.30102999
chr3	168347450	168429212	81763	1	12	0	0.41314172	0	10	0	0.09290028	0
chr3	168429212	168524592	95381	1	11	0	0.30102999	0	10	0	0.14303407	0
chr3	168524592	168802607	278016	4	11	0	0.21200206	0	11	1	0.21200206	0.30102999
chr3	168802607	168824043	21437	2	12	0	0.30102999	0	11	1	0.14385241	0.30102999
chr3	168824043	169195165	371123	9	11	0	0.21200206	0	11	1	0.21200206	0.30102999
chr3	169195165	169195224	60	1	13	0	0.30102999	0	12	1	0.14385241	0.30102999
chr3	169195224	169272415	77192	2	12	0	0.30102999	0	11	1	0.14385241	0.30102999
chr3	169272415	169272474	60	1	12	0	0.21227046	0	12	1	0.21227046	0.30102999
chr3	169272474	169685517	413044	12	12	0	0.30102999	0	11	1	0.14385241	0.30102999
chr3	169685517	169788584	103068	3	13	0	0.41271556	0	11	1	0.09334429	0.30102999
chr3	169788584	169825757	37174	1	11	0	0.30102999	0	10	1	0.14303407	0.30102999
chr3	169825757	169825816	60	1	11	0	0.30102999	0	10	2	0.14303407	0.61140001
chr3	169825816	169999578	173763	4	11	0	0.30102999	0	10	1	0.14303407	0.30102999
chr3	169999578	170178481	178904	7	12	0	0.41314172	0	10	1	0.09290028	0.30102999
chr3	170178481	170265595	87115	2	11	0	0.30102999	0	10	1	0.14303407	0.30102999
chr3	170265595	170377634	112040	2	10	0	0.21118145	0	10	1	0.21118145	0.30102999
chr3	170377634	170422887	45254	2	11	0	0.30102999	0	10	1	0.14303407	0.30102999
chr3	170422887	170422946	60	1	12	0	0.41314172	0	10	1	0.09290028	0.30102999
chr3	170422946	170546140	123195	2	9	0	0.30102999	0	8	1	0.13872638	0.30102999
chr3	170546140	170621985	75846	4	12	0	0.72109894	0	8	1	0.03209037	0.30102999
chr3	170621985	170681646	59662	1	12	0	0.72109894	0	8	0	0.03209037	0
chr3	170681646	170681705	60	1	12	0	0.55190077	0	9	0	0.0565833	0
chr3	170681705	171125616	443912	9	12	0	0.72109894	0	8	0	0.03209037	0
chr3	171125616	171329410	203795	4	12	0	0.72109894	0	8	1	0.03209037	0.30102999
chr3	171329410	171329469	60	1	12	0	0.30102999	0	11	1	0.14385241	0.30102999
chr3	171329469	171379837	50369	1	12	0	0.41314172	0	10	0	0.09290028	0
chr3	171379837	171516011	136175	4	13	0	0.5498098	0	10	0	0.0574087	0
chr3	171516011	171737139	221129	3	13	0	0.5498098	0	10	1	0.0574087	0.30102999
chr3	171737139	171737198	60	1	13	0	0.41271556	0	11	1	0.09334429	0.30102999
chr3	171737198	171806594	69397	2	13	0	0.5498098	0	10	0	0.0574087	0

chr3	171806594	171806653	60	1	13	0	0.41271556	0	11	0	0.09334429	0
chr3	171806653	171904548	97896	3	13	0	0.5498098	0	10	0	0.0574087	0
chr3	171904548	171904607	60	1	13	0	0.30102999	0	12	0	0.14385241	0
chr3	171904607	172016586	111980	2	13	0	0.41271556	0	11	0	0.09334429	0
chr3	172016586	172058885	42300	1	13	0	0.5498098	0	10	0	0.0574087	0
chr3	172058885	172350612	291728	8	13	0	0.71538971	0	9	0	0.0331093	0
chr3	172350612	172538403	187792	6	13	0	0.41271556	0	11	0	0.09334429	0
chr3	172538403	172560272	21870	1	12	0	0.30102999	0	11	0	0.14385241	0
chr3	172560272	172632382	72111	1	11	0	0.30102999	0	10	0	0.14303407	0
chr3	172632382	172694812	62431	1	11	0	0.41444892	0	9	0	0.09154957	0
chr3	172694812	172735030	40219	2	13	0	0.5498098	0	10	1	0.0574087	0.30102999
chr3	172735030	172735089	60	1	13	0	0.41271556	0	11	1	0.09334429	0.30102999
chr3	172735089	172798434	63346	2	13	0	0.5498098	0	10	0	0.0574087	0
chr3	172798434	172798493	60	1	13	0	0.41271556	0	11	1	0.09334429	0.30102999
chr3	172798493	172835272	36780	1	13	0	0.5498098	0	10	0	0.0574087	0
chr3	172835272	172835331	60	1	14	0	0.5498098	0	11	0	0.0574087	0
chr3	172835331	173076361	241031	2	13	0	0.41271556	0	11	0	0.09334429	0
chr3	173076361	173198136	121776	2	13	0	0.5498098	0	10	0	0.0574087	0
chr3	173198136	173198195	60	1	13	0	0.41271556	0	11	0	0.09334429	0
chr3	173198195	173302774	104580	3	13	0	0.5498098	0	10	0	0.0574087	0
chr3	173302774	173411152	108379	2	12	0	0.41314172	0	10	0	0.09290028	0
chr3	173411152	173458666	47515	2	13	0	0.5498098	0	10	0	0.0574087	0
chr3	173458666	173458725	60	1	13	0	0.41271556	0	11	0	0.09334429	0
chr3	173458725	173539520	80796	2	12	0	0.41314172	0	10	0	0.09290028	0
chr3	173539520	173614890	75371	2	13	0	0.30102999	0	12	0	0.14385241	0
chr3	173614890	173740143	125254	2	13	0	0.41271556	0	11	0	0.09334429	0
chr3	173740143	173830379	90237	2	12	0	0.41314172	0	10	0	0.09290028	0
chr3	173830379	174033302	202924	4	10	0	0.30102999	0	9	0	0.14135546	0
chr3	174033302	174378043	344742	4	9	0	0.20975986	0	9	0	0.20975986	0
chr3	174378043	174713426	335384	7	10	0	0.30102999	0	9	0	0.14135546	0
chr3	174713426	174821844	108419	2	9	0	0.30102999	0	8	0	0.13872638	0
chr3	174821844	174821903	60	1	10	0	0.30102999	0	9	0	0.14135546	0
chr3	174821903	174955077	133175	2	9	0	0.30102999	0	8	0	0.13872638	0
chr3	174955077	175008609	53533	1	8	0	0.30102999	0	7	0	0.13499366	0
chr3	175008609	175060188	51580	1	7	0	0.30102999	0	6	0	0.129913	0
chr3	175060188	175125133	64946	2	7	0	0.20469099	0	7	0	0.20469099	0
chr3	175125133	175268482	143350	3	6	0	0.20064824	0	6	0	0.20064824	0
chr3	175268482	175347833	79352	2	5	0	0.12309572	0	6	0	0.30102999	0
chr3	175347833	175347892	60	1	6	0	0.20064824	0	6	0	0.20064824	0
chr3	175347892	175468381	120490	2	5	0	0.12309572	0	6	0	0.30102999	0
chr3	175468381	175468440	60	1	6	0	0.20064824	0	6	1	0.20064824	0.30102999
chr3	175468440	175568222	99783	1	5	0	0.12309572	0	6	1	0.30102999	0.30102999
chr3	175568222	175568281	60	1	6	0	0.20064824	0	6	1	0.20064824	0.30102999
chr3	175568281	175699047	130767	1	3	0	0.03070643	0	6	1	0.63695542	0.30102999
chr3	175699047	175809788	110742	1	3	0	0.03070643	0	6	0	0.63695542	0
chr3	175809788	176025124	215337	2	5	0	0.07511598	0	7	0	0.43181735	0
chr3	176025124	176157699	132576	2	7	0	0.20469099	0	7	0	0.20469099	0
chr3	176157699	176157758	60	1	8	0	0.30102999	0	7	0	0.13499366	0
chr3	176157758	176272287	114530	1	7	0	0.30102999	0	6	0	0.129913	0
chr3	176272287	176272346	60	1	7	0	0.30102999	0	6	1	0.129913	0.30102999
chr3	176272346	176483587	211242	2	4	0	0.06713722	0	6	0	0.44141547	0
chr3	176483587	176551757	68171	2	5	0	0.12309572	0	6	0	0.30102999	0
chr3	176551757	176845144	293388	6	4	0	0.06713722	0	6	0	0.44141547	0
chr3	176845144	177197497	352354	5	5	0	0.12309572	0	6	0	0.30102999	0
chr3	177197497	177329227	131731	3	7	0	0.30102999	0	6	0	0.129913	0
chr3	177329227	177560267	231041	3	8	0	0.4250187	0	6	0	0.08122616	0
chr3	177560267	177710016	149750	1	7	0	0.30102999	0	6	0	0.129913	0
chr3	177710016	177710075	60	1	8	0	0.4250187	0	6	0	0.08122616	0
chr3	177710075	178017330	307256	2	6	0	0.20064824	0	6	0	0.20064824	0
chr3	178017330	178017389	60	1	9	1	0.42015402	0.30102999	7	0	0.08584816	0
chr3	178017389	178164664	147276	2	8	1	0.30102999	0.30102999	7	0	0.13499366	0
chr3	178164664	178284365	119702	3	9	1	0.42015402	0.30102999	7	0	0.08584816	0
chr3	178284365	178420467	136103	3	8	1	0.30102999	0.30102999	7	0	0.13499366	0
chr3	178420467	178420526	60	1	10	1	0.30102999	0.30102999	9	0	0.14135546	0
chr3	178420526	178471351	50826	1	9	1	0.30102999	0.30102999	8	0	0.13872638	0
chr3	178471351	178471410	60	1	9	1	0.20975986	0.30102999	9	0	0.20975986	0
chr3	178471410	178521877	50468	1	9	1	0.30102999	0.30102999	8	0	0.13872638	0
chr3	178521877	178521936	60	1	11	1	0.30102999	0.30102999	10	0	0.14303407	0
chr3	178521936	178622708	100773	2	11	1	0.41444892	0.30102999	9	0	0.09154957	0
chr3	178622708	178622767	60	1	11	1	0.21200206	0.30102999	11	0	0.21200206	0
chr3	178622767	178742237	119471	1	10	1	0.14303407	0.30102999	11	0	0.30102999	0
chr3	178742237	178742296	60	1	10	1	0.09290028	0.30102999	12	0	0.41314172	0
chr3	178742296	178764048	21753	1	10	1	0.21118145	0.30102999	10	0	0.21118145	0
chr3	178764048	178764107	60	1	11	1	0.30102999	0.30102999	10	0	0.14303407	0
chr3	178764107	178879119	115013	3	10	1	0.21118145	0.30102999	10	0	0.21118145	0
chr3	178879119	178879178	60	1	11	1	0.30102999	0.30102999	10	0	0.14303407	0
chr3	178879178	179095030	215853	7	11	1	0.41444892	0.30102999	9	0	0.09154957	0
chr3	179095030	179196273	101244	3	11	1	0.55623409	0.30102999	8	0	0.05490675	0
chr3	179196273	179277087	80815	1	11	1	0.73110763	0.30102999	7	0	0.03037338	0
chr3	179277087	179306327	29241	3	11	1	0.55623409	0.30102999	8	0	0.05490675	0
chr3	179306327	179328710	22384	1	10	1	0.56314362	0.30102999	7	0	0.05232577	0
chr3	179328710	179389917	61208	2	10	1	0.4167287	0.30102999	8	0	0.08923391	0
chr3	179389917	179448056	58140	3	10	1	0.30102999	0.30102999	9	0	0.14135546	0
chr3	179448056	179483463	35408	1	10	1	0.4167287	0.30102999	8	0	0.08923391	0
chr3	179483463	179553430	69968	3	10	1	0.30102999	0.30102999	9	0	0.14135546	0
chr3	179553430	179593210	39781	1	9	1	0.20975986	0.30102999	9	0	0.20975986	0
chr3	179593210	179666115	72906	3	9	1	0.20975986	0.1218695	9	1	0.20975986	0.1218695
chr3	179666115	179666174	60	1	9	2	0.14135546	0.30102999	10	1	0.30102999	0.05404976
chr3	179666174	179734228	68055	2	8	2	0.08923391	0.30102999	10	1	0.4167287	0.05404976
chr3	179734228	179734287	60	1	9	2	0.14135546	0.30102999	10	1	0.30102999	0.05404976
chr3	179734287	179753977	19691	1	8	2	0.08923391	0.30102999	10	1	0.4167287	0.05404976
chr3	179753977	179859594	105618	1	8	2	0.20764654	0.30102999	8	1	0.20764654	0.05404976
chr3	179859594	179859653	60	1	8	2	0.13872638	0.30102999	9	1	0.30102999	0.05404976
chr3	179859653	179899339	39687	1	8	2	0.20764654	0.30102999	8	1	0.20764654	0.05404976
chr3	179899339	180022216	122878	1	7	1	0.13499366	0.1218695	8	1	0.30102999	0.1218695
chr3	180022216	180261965	239750	2	6	1	0.129913	0.30102999	7	0	0.30102999	0
chr3	180261965	180262024	60	1	6	1	0.129913	0.1218695	7	1	0.30102999	0.1218695
chr3	180262024	180320911	58888	1	5	1	0.07511598	0.1218695	7	1	0.43181735	0.1218695
chr3	180320911	180325725	4815	2	6	1	0.129913	0.1218695	7	1	0.30102999	0.1218695
chr3	180325725	180380061	54337	1	5	1	0.07511598	0.1218695	7	1	0.43181735	0.1218695
chr3	180380061	180421243	41183	1	5	1	0.12309572	0.1218695	6	1	0.30102999	0.1218695
chr3	180421243	180550890	129648	2	4	1	0.06713722	0.1218695	6	1	0.44141547	0.1218695
chr3	180550890	180631203	80314	2	4	1	0.06713722	0.05404976	6	2	0.44141547	0.30102999
chr3	180631203	180651152	19950	2	5	1	0.12309572	0.05404976	6	2	0.30102999	0.30102999
chr3	180651152	180980750	329599	5	4	1	0.06713722	0.05404976	6	2	0.44141547	0.30102999
chr3	180980750	181101964	121215	1	4	1	0.11390336	0.05404976	5	2	0.30102999	0.30102999
chr3	181101964	181292708	190745	1	4	1	0.11390336	0.1218695	5	1	0.30102999	0.1218695
chr3	181292708	181292767	60	1	4	1	0.06713722	0.1218695	6	1	0.44141547	0.1218695
chr3	181292767	181360636	67870	1	4	1	0.11390336	0.1218695	5	1	0.30102999	0.1218695
chr3	181360636	181360695	60	1	5	1	0.19510895	0.1218695	5	1	0.19510895	0.1218695
chr3	181360695	181472338	111644	4	5	0	0.19510895	0	5	0	0.19510895	0
chr3	181472338	181648319	175982	3	5	0	0.12309572	0	6	0	0.30102999	0
chr3	181648319	181648378	60	1	6	0	0.20064824	0	6	0	0.20064824	0
chr3	181648378	181991155	342778	2	5	0	0.12309572	0	6	0	0.30102999	0
chr3	181991155	182245548	254394	3	5	1	0.07511598	0.30102999	7	0	0.43181735	0
chr3	182245548	182245607	60	1	6	2	0.129913	0.30102999	7	1	0.30102999	0.05404976
chr3	182245607	182458878	213272	2	6	1	0.129913	0.30102999	7	0	0.30102999	0
chr3	182458878	182458937	60	1	6	1	0.08122616	0.30102999	8	0	0.4250187	0
chr3	182458937	182607505	148569	2	5	1	0.07511598	0.30102999	7	0	0.43181735	0
chr3	182607505	182790188	182684	5	6	1	0.08122616	0.30102999	8	0	0.4250187	0
chr3	182790188	182858917	68730	3	6	2	0.08122616	0.30102999	8	1	0.4250187	0.05404976
chr3	182858917	182989790	130874	3	6	2	0.08122616	0.1575501	8	2	0.4250187	0.1575501
chr3	182989790	183051329	61540	2	6	2	0.129913	0.1575501	7	2	0.30102999	0.1575501
chr3	183051329	183245731	194403	7	7	2	0.13499366	0.1575501	8	2	0.30102999	0.1575501

chr3	183245731	183660527	414797	14	7	2	0.08584816	0.1575501	9	2	0.42015402	0.1575501
chr3	183660527	183660580	54	1	7	2	0.03037338	0.1575501	11	2	0.73110763	0.1575501
chr3	183660580	183752847	92268	2	7	2	0.05232577	0.1575501	10	2	0.56314362	0.1575501
chr3	183752847	183778261	25415	2	8	2	0.08923391	0.1575501	10	2	0.4167287	0.1575501
chr3	183778261	184036953	258693	15	8	2	0.13872638	0.1575501	9	2	0.30102999	0.1575501
chr3	184036953	184084548	47596	4	7	2	0.08584816	0.1575501	9	2	0.42015402	0.1575501
chr3	184084548	184523379	438832	11	8	2	0.13872638	0.1575501	9	2	0.30102999	0.1575501
chr3	184523379	184523438	60	1	8	2	0.08923391	0.1575501	10	2	0.4167287	0.1575501
chr3	184523438	184548606	25169	1	8	2	0.13872638	0.1575501	9	2	0.30102999	0.1575501
chr3	184548606	184604158	55553	1	7	2	0.08584816	0.1575501	9	2	0.42015402	0.1575501
chr3	184604158	184604217	60	1	7	2	0.05232577	0.1575501	10	2	0.56314362	0.1575501
chr3	184604217	184684444	80228	2	7	2	0.05232577	0.30102999	10	1	0.56314362	0.05404976
chr3	184684444	184684503	60	1	8	2	0.03209037	0.30102999	12	1	0.72109894	0.05404976
chr3	184684503	184724704	40202	1	8	2	0.08923391	0.30102999	10	1	0.4167287	0.05404976
chr3	184724704	184724763	60	1	9	2	0.14135546	0.30102999	10	1	0.30102999	0.05404976
chr3	184724763	184799629	74867	1	9	0	0.14135546	0	10	1	0.30102999	0.30102999
chr3	184799629	184869065	69437	3	9	0	0.0331093	0	13	1	0.71538971	0.30102999
chr3	184869065	184869124	60	1	10	0	0.0574087	0	13	1	0.5498098	0.30102999
chr3	184869124	184906121	36998	1	10	0	0.09290028	0	12	1	0.41314172	0.30102999
chr3	184906121	184935913	29793	2	9	0	0.09154957	0	11	1	0.41444892	0.30102999
chr3	184935913	184935965	53	1	9	1	0.0565833	0.1218695	12	1	0.55190077	0.1218695
chr3	184935965	184966251	30287	1	9	0	0.0565833	0	12	1	0.55190077	0.30102999
chr3	184966251	185036237	69987	1	9	0	0.20975986	0	9	1	0.20975986	0.30102999
chr3	185036237	185036296	60	1	9	0	0.14135546	0	10	1	0.30102999	0.30102999
chr3	185036296	185169017	132722	3	9	0	0.20975986	0	9	1	0.20975986	0.30102999
chr3	185169017	185210992	41976	2	9	1	0.20975986	0.05404976	9	2	0.20975986	0.30102999
chr3	185210992	185252583	41592	1	9	0	0.20975986	0	9	2	0.20975986	0.61140001
chr3	185252583	185307872	55290	1	9	0	0.30102999	0	8	2	0.13872638	0.61140001
chr3	185307872	185307931	60	1	9	1	0.20975986	0.05404976	9	2	0.20975986	0.30102999
chr3	185307931	185348867	40937	1	8	1	0.20764654	0.05404976	8	2	0.20764654	0.30102999
chr3	185348867	185419048	70182	1	8	1	0.30102999	0.05404976	7	2	0.13499366	0.30102999
chr3	185419048	185463211	44164	2	8	2	0.30102999	0.1575501	7	2	0.13499366	0.1575501
chr3	185463211	185463270	60	1	9	2	0.42015402	0.1575501	7	2	0.08584816	0.1575501
chr3	185463270	185539081	75812	2	8	2	0.30102999	0.1575501	7	2	0.13499366	0.1575501
chr3	185539081	185539140	60	1	9	2	0.20975986	0.1575501	9	2	0.20975986	0.1575501
chr3	185539140	185711222	172083	4	9	2	0.42015402	0.1575501	7	2	0.08584816	0.1575501
chr3	185711222	185823405	112184	2	7	2	0.20469099	0.1575501	7	2	0.20469099	0.1575501
chr3	185823405	185823458	54	1	8	2	0.30102999	0.1575501	7	2	0.13499366	0.1575501
chr3	185823458	185969574	146117	3	6	2	0.129913	0.1575501	7	2	0.30102999	0.1575501
chr3	185969574	186235972	266399	4	6	2	0.129913	0.30102999	7	1	0.30102999	0.05404976
chr3	186235972	186236031	60	1	6	2	0.129913	0.1575501	7	2	0.30102999	0.1575501
chr3	186236031	186303354	67324	4	5	2	0.07511598	0.1575501	7	2	0.43181735	0.1575501
chr3	186303354	186303413	60	1	6	2	0.129913	0.1575501	7	2	0.30102999	0.1575501
chr3	186303413	186370669	67257	2	5	2	0.07511598	0.1575501	7	2	0.43181735	0.1575501
chr3	186370669	186394793	24125	2	6	2	0.129913	0.1575501	7	2	0.30102999	0.1575501
chr3	186394793	186470781	75989	2	6	2	0.129913	0.30102999	7	1	0.30102999	0.05404976
chr3	186470781	186518971	48191	5	6	2	0.129913	0.1575501	7	2	0.30102999	0.1575501
chr3	186518971	186760613	241643	5	6	2	0.129913	0.30102999	7	1	0.30102999	0.05404976
chr3	186760613	186794754	34142	1	5	2	0.07511598	0.30102999	7	1	0.43181735	0.05404976
chr3	186794754	186794813	60	1	5	2	0.07511598	0.1575501	7	2	0.43181735	0.1575501
chr3	186794813	186936981	142169	3	5	2	0.07511598	0.30102999	7	1	0.43181735	0.05404976
chr3	186936981	187086869	149889	4	4	2	0.03812622	0.30102999	7	1	0.60763643	0.05404976
chr3	187086869	187454155	367287	8	5	2	0.07511598	0.30102999	7	1	0.43181735	0.05404976
chr3	187454155	187637107	182953	2	4	2	0.03812622	0.61140001	7	0	0.60763643	0
chr3	187637107	187637166	60	1	5	2	0.07511598	0.30102999	7	1	0.43181735	0.05404976
chr3	187637166	187832390	195225	2	5	1	0.07511598	0.30102999	7	0	0.43181735	0
chr3	187832390	187894262	61873	2	6	1	0.129913	0.1218695	7	1	0.30102999	0.1218695
chr3	187894262	187894321	60	1	6	1	0.08122616	0.1218695	8	1	0.4250187	0.1218695
chr3	187894321	187943305	48985	1	5	1	0.04407651	0.1218695	8	1	0.58747015	0.1218695
chr3	187943305	188123870	180566	4	5	1	0.07511598	0.1218695	7	1	0.43181735	0.1218695
chr3	188123870	188123928	59	1	7	1	0.13499366	0.1218695	8	1	0.30102999	0.1218695
chr3	188123928	188288589	164662	3	7	1	0.20469099	0.1218695	7	1	0.20469099	0.1218695
chr3	188288589	188288648	60	1	7	1	0.13499366	0.1218695	8	1	0.30102999	0.1218695
chr3	188288648	188389234	100587	2	6	1	0.129913	0.1218695	7	1	0.30102999	0.1218695
chr3	188389234	188538435	149202	4	7	1	0.20469099	0.1218695	7	1	0.20469099	0.1218695
chr3	188538435	188607606	69172	2	6	1	0.129913	0.1218695	7	1	0.30102999	0.1218695
chr3	188607606	188710871	103266	2	8	1	0.30102999	0.1218695	7	1	0.13499366	0.1218695
chr3	188710871	188822766	111896	2	8	1	0.20764654	0.1218695	8	1	0.20764654	0.1218695
chr3	188822766	188997660	174895	4	8	1	0.13872638	0.1218695	9	1	0.30102999	0.1218695
chr3	188997660	189083620	85961	2	9	1	0.14135546	0.1218695	10	1	0.30102999	0.1218695
chr3	189083620	189083679	60	1	11	1	0.30102999	0.1218695	10	1	0.14303407	0.1218695
chr3	189083679	189319473	235795	2	9	1	0.14135546	0.1218695	10	1	0.30102999	0.1218695
chr3	189319473	189319532	60	1	10	1	0.21118145	0.1218695	10	1	0.21118145	0.1218695
chr3	189319532	189456465	136934	3	9	1	0.14135546	0.1218695	10	1	0.30102999	0.1218695
chr3	189456465	189507656	51192	1	8	1	0.13872638	0.1218695	9	1	0.30102999	0.1218695
chr3	189507656	189688682	181027	5	8	1	0.13872638	0.30102999	9	0	0.30102999	0
chr3	189688682	189947899	259218	6	6	1	0.08122616	0.30102999	8	0	0.4250187	0
chr3	189947899	189947958	60	1	6	1	0.08122616	0.1218695	8	1	0.4250187	0.1218695
chr3	189947958	190030816	82859	1	5	1	0.04407651	0.1218695	8	1	0.58747015	0.1218695
chr3	190030816	190205705	174890	4	6	1	0.08122616	0.1218695	8	1	0.4250187	0.1218695
chr3	190205705	190205764	60	1	7	1	0.08584816	0.1218695	9	1	0.42015402	0.1218695
chr3	190205764	190604567	398804	7	6	1	0.08122616	0.30102999	8	0	0.4250187	0
chr3	190604567	190811038	206472	1	5	1	0.04407651	0.30102999	8	0	0.58747015	0
chr3	190811038	191290431	479394	8	5	1	0.07511598	0.30102999	7	0	0.43181735	0
chr3	191290431	191413759	123329	2	5	1	0.07511598	0.1218695	7	1	0.43181735	0.1218695
chr3	191413759	191413818	60	1	7	1	0.13499366	0.1218695	8	1	0.30102999	0.1218695
chr3	191413818	191742477	328660	2	6	1	0.08122616	0.1218695	8	1	0.4250187	0.1218695
chr3	191742477	192005036	262560	5	7	1	0.13499366	0.1218695	8	1	0.30102999	0.1218695
chr3	192005036	192005095	60	1	10	1	0.4167287	0.1218695	8	1	0.08923391	0.1218695
chr3	192005095	192059737	54643	1	10	1	0.4167287	0.30102999	8	0	0.08923391	0
chr3	192059737	192142365	82629	2	9	1	0.30102999	0.30102999	8	0	0.13872638	0
chr3	192142365	192241574	99210	2	8	1	0.30102999	0.30102999	7	0	0.13499366	0
chr3	192241574	192437045	195472	5	8	1	0.20764654	0.30102999	8	0	0.20764654	0
chr3	192437045	192470785	33741	2	8	1	0.13872638	0.30102999	9	0	0.30102999	0
chr3	192470785	192586367	115583	3	8	1	0.20764654	0.30102999	8	0	0.20764654	0
chr3	192586367	192633769	47403	2	8	1	0.08923391	0.30102999	10	0	0.4167287	0
chr3	192633769	193109853	476085	8	8	1	0.13872638	0.30102999	9	0	0.30102999	0
chr3	193109853	193157462	47610	2	9	1	0.20975986	0.30102999	9	0	0.20975986	0
chr3	193157462	193267743	110282	3	9	1	0.14135546	0.30102999	10	0	0.30102999	0
chr3	193267743	193267802	60	1	9	1	0.09154957	0.1218695	11	1	0.41444892	0.1218695
chr3	193267802	193384963	117162	2	9	1	0.14135546	0.30102999	10	0	0.30102999	0
chr3	193384963	193385022	60	1	9	1	0.14135546	0.1218695	10	1	0.30102999	0.1218695
chr3	193385022	193413976	28955	1	9	1	0.14135546	0.30102999	10	0	0.30102999	0
chr3	193413976	193579266	165291	2	8	1	0.13872638	0.30102999	9	0	0.30102999	0
chr3	193579266	193579325	60	1	9	1	0.14135546	0.30102999	10	0	0.30102999	0
chr3	193579325	193816606	237282	4	9	1	0.20975986	0.30102999	9	0	0.20975986	0
chr3	193816606	194115999	299394	7	9	1	0.20975986	0.1218695	9	1	0.20975986	0.1218695
chr3	194115999	194151878	35880	3	9	1	0.20975986	0.05404976	9	2	0.20975986	0.30102999
chr3	194151878	194195900	44023	1	9	1	0.30102999	0.05404976	8	2	0.13872638	0.30102999
chr3	194195900	194246923	51024	1	9	1	0.30102999	0.1218695	8	1	0.13872638	0.1218695
chr3	194246923	194362861	115939	4	9	1	0.20975986	0.1218695	9	1	0.20975986	0.1218695
chr3	194362861	194520577	157717	2	9	1	0.30102999	0.1218695	8	1	0.13872638	0.1218695
chr3	194520577	194851175	330599	5	9	1	0.30102999	0.05404976	8	2	0.13872638	0.30102999
chr3	194851175	194851234	60	1	10	1	0.4167287	0.05404976	8	2	0.08923391	0.30102999
chr3	194851234	194958399	107166	2	9	1	0.42015402	0.05404976	7	2	0.08584816	0.30102999
chr3	194958399	195000153	41755	3	11	1	0.55623409	0.05404976	8	2	0.05490675	0.30102999
chr3	195000153	195027281	27129	1	10	1	0.56314362	0.05404976	7	2	0.05232577	0.30102999
chr3	195027281	195060930	33650	1	9	1	0.42015402	0.05404976	7	2	0.08584816	0.30102999
chr3	195060930	195101733	40804	2	10	1	0.56314362	0.05404976	7	2	0.05232577	0.30102999
chr3	195101733	195101792	60	1	11	2	0.73110763	0.1575501	7	2	0.03037338	0.1575501
chr3	195101792	195243902	142111	3	9	2	0.5732208	0.1575501	6	2	0.04875589	0.1575501
chr3	195243902	195296510	52609	2	10	2	0.56314362	0.1575501	7	2	0.05232577	0.1575501
chr3	195296510	195425934	129425	2	10	2	0.56314362	0.30102999	7	1	0.05232577	0.05404976
chr3	195425934	195482573	56640	4	10	1	0.56314362	0.1218695	7	1	0.05232577	0.1218695

chr3	195482573	195482632	60	1	10	2	0.56314362	0.30102999	7	1	0.05232577	0.05404976
chr3	195482632	195740357	257726	5	10	1	0.56314362	0.1218695	7	1	0.05232577	0.1218695
chr3	195740357	195789463	49107	3	11	2	0.73110763	0.30102999	7	1	0.03037338	0.05404976
chr3	195789463	195840894	51432	4	11	2	0.73110763	0.1575501	7	2	0.03037338	0.1575501
chr3	195840894	195908864	67971	1	11	1	0.73110763	0.1218695	7	1	0.03037338	0.1218695
chr3	195908864	196120031	211168	8	11	1	0.55623409	0.1218695	8	1	0.05490675	0.1218695
chr3	196120031	196120090	60	1	11	1	0.41444892	0.1218695	9	1	0.09154957	0.1218695
chr3	196120090	196293986	173897	4	11	1	0.55623409	0.1218695	8	1	0.05490675	0.1218695
chr3	196293986	196443776	149791	4	11	1	0.41444892	0.1218695	9	1	0.09154957	0.1218695
chr3	196443776	196468474	24699	1	11	1	0.73110763	0.1218695	7	1	0.03037338	0.1218695
chr3	196468474	196502693	34220	2	12	1	0.92532268	0.1218695	7	1	0.01667721	0.1218695
chr3	196502693	196509171	6479	2	12	1	0.55190077	0.1218695	9	1	0.0565833	0.1218695
chr3	196509171	196599138	89968	2	11	1	0.55623409	0.1218695	8	1	0.05490675	0.1218695
chr3	196599138	196731338	132201	7	11	1	0.73110763	0.1218695	7	1	0.03037338	0.1218695
chr3	196731338	196731395	58	1	11	1	0.55623409	0.05404976	8	2	0.05490675	0.30102999
chr3	196731395	196771500	40106	2	11	1	0.55623409	0.1218695	8	1	0.05490675	0.1218695
chr3	196771500	196771557	58	1	11	1	0.55623409	0.05404976	8	2	0.05490675	0.30102999
chr3	196771557	196960822	189266	4	11	1	0.55623409	0.1218695	8	1	0.05490675	0.1218695
chr3	196960822	196960881	60	1	11	1	0.41444892	0.1218695	9	1	0.09154957	0.1218695
chr3	196960881	197023273	62393	1	11	0	0.55623409	0	8	1	0.05490675	0.30102999
chr3	197023273	197238597	215325	3	10	0	0.56314362	0	7	1	0.05232577	0.30102999
chr3	197238597	197310451	71855	3	10	0	0.56314362	0	7	2	0.05232577	0.61140001
chr3	197310451	197446442	135992	3	9	0	0.42015402	0	7	2	0.08584816	0.61140001
chr3	197446442	197446501	60	1	10	0	0.56314362	0	7	2	0.05232577	0.61140001
chr3	197446501	197478171	31671	1	9	0	0.5732208	0	6	1	0.04875589	0.30102999
chr3	197478171	197516977	38807	1	9	0	0.76806864	0	5	1	0.02473314	0.30102999
chr3	197516977	197517036	60	1	10	0	0.74627054	0	6	2	0.02793176	0.61140001
chr3	197517036	197544104	27069	1	8	0	0.4250187	0	6	2	0.08122616	0.61140001
chr3	197544104	197608333	64230	3	8	0	0.30102999	0	7	2	0.13499366	0.61140001
chr3	197608333	197608392	60	1	9	0	0.42015402	0	7	2	0.08584816	0.61140001
chr3	197608392	197681739	73348	2	8	0	0.30102999	0	7	1	0.13499366	0.30102999
chr3	197681739	197681798	60	1	9	0	0.30102999	0	8	2	0.13872638	0.61140001
chr3	197681798	197766732	84935	3	8	0	0.4250187	0	6	1	0.08122616	0.30102999
chr3	197766732	197766791	60	1	8	0	0.30102999	0	7	1	0.13499366	0.30102999
chr3	197766791	197840339	73549	1	7	0	0.20469099	0	7	1	0.20469099	0.30102999
chr4	71552	71611	60	1	3	1	0.93173516	0.00204627	0	6	0	1.31360226
chr4	71611	221449	149839	2	3	1	0.93173516	0.01091641	0	4	0	0.76005302
chr4	221449	221508	60	1	3	2	0.93173516	0.01053319	0	6	0	0.91219088
chr4	221508	259110	37603	1	3	2	0.93173516	0.02162467	0	5	0	0.68214471
chr4	259110	259169	60	1	3	3	0.93173516	0.01598258	0	7	0	0.84395715
chr4	259169	315565	56397	1	3	2	0.93173516	0.01053319	0	6	0	0.91219088
chr4	315565	365626	50062	4	3	3	0.93173516	0.01598258	0	7	0	0.84395715
chr4	365626	522551	156926	5	3	5	0.93173516	0.02473314	0	9	0	0.76806864
chr4	522551	628500	105950	2	3	4	0.93173516	0.01077081	0	9	0	1.01542894
chr4	628500	755208	126709	4	3	4	0.93173516	0.00530919	0	10	0	1.2568129
chr4	755208	864692	109485	3	3	5	0.93173516	0.00666883	0	11	0	1.20557689
chr4	864692	927780	63089	1	3	5	0.93173516	0.01320236	0	10	0	0.97390707
chr4	927780	927839	60	1	3	7	0.93173516	0.05232577	0	10	0	0.56314362
chr4	927839	1004652	76814	2	3	6	0.93173516	0.02793176	0	10	0	0.74627054
chr4	1004652	1004711	60	1	3	6	0.93173516	0.01518174	0	11	0	0.9449689
chr4	1004711	1146766	142056	2	3	6	0.93173516	0.02793176	0	10	0	0.74627054
chr4	1146766	1319275	172510	5	3	6	0.93173516	0.01518174	0	11	0	0.9449689
chr4	1319275	1364014	44740	1	3	6	0.93173516	0.04875589	0	9	0	0.5732208
chr4	1364014	1403261	39248	1	3	5	0.93173516	0.02473314	0	9	0	0.76806864
chr4	1403261	1563818	160558	2	3	5	0.93173516	0.01320236	0	10	0	0.97390707
chr4	1563818	1600380	36563	2	3	7	0.93173516	0.03037338	0	11	0	0.73110763
chr4	1600380	1600439	60	1	3	7	0.51676182	0.03037338	1	11	0.02438896	0.73110763
chr4	1600439	1678585	78147	1	3	7	0.51676182	0.08584816	1	9	0.02438896	0.42015402
chr4	1678585	1800380	121796	3	3	6	0.93173516	0.08122616	0	8	0	0.4250187
chr4	1800380	1875256	74877	2	3	6	0.51676182	0.04875589	1	9	0.02438896	0.5732208
chr4	1875256	1944311	69056	2	3	6	0.51676182	0.01518174	1	11	0.02438896	0.9449689
chr4	1944311	1944370	60	1	3	6	0.51676182	0.00778066	1	12	0.02438896	1.17038931
chr4	1944370	1980946	36577	1	3	6	0.93173516	0.01518174	0	11	0	0.9449689
chr4	1980946	2021560	40615	3	3	6	0.93173516	0.00778066	0	12	0	1.17038931
chr4	2021560	2068404	46845	1	3	6	0.93173516	0.01518174	0	11	0	0.9449689
chr4	2068404	2110275	41872	1	3	6	0.93173516	0.02793176	0	10	0	0.74627054
chr4	2110275	2158354	48080	2	3	6	0.93173516	0.01518174	0	11	0	0.9449689
chr4	2158354	2233676	75323	2	3	7	0.93173516	0.00859896	0	13	0	1.14735594
chr4	2233676	2598422	364747	7	3	7	0.93173516	0.01667721	0	12	0	0.92532268
chr4	2598422	2808212	209791	4	3	6	0.93173516	0.00778066	0	12	0	1.17038931
chr4	2808212	2867343	59132	2	3	6	0.93173516	0.01518174	0	11	0	0.9449689
chr4	2867343	3174976	307634	8	3	6	0.93173516	0.00778066	0	12	0	1.17038931
chr4	3174976	3175035	60	1	3	6	0.93173516	0.00373523	0	13	0	1.42397267
chr4	3175035	3434016	258982	6	3	6	0.93173516	0.00778066	0	12	0	1.17038931
chr4	3434016	3434075	60	1	3	6	0.93173516	0.00373523	0	13	0	1.42397267
chr4	3434075	3483540	49466	1	3	6	0.93173516	0.00778066	0	12	0	1.17038931
chr4	3483540	3527216	43677	1	2	6	0.61140001	0.00778066	0	12	0	1.17038931
chr4	3527216	3580260	53045	1	2	5	0.61140001	0.00317045	0	12	0	1.46403142
chr4	3580260	3580319	60	1	3	5	0.93173516	0.00141024	0	13	0	1.7506523
chr4	3580319	3636834	56516	1	2	5	0.61140001	0.00317045	0	12	0	1.46403142
chr4	3636834	3776843	140010	3	2	5	0.30102999	0.00317045	1	12	0.05404976	1.46403142
chr4	3776843	3845038	68196	1	2	4	0.30102999	0.00108487	1	12	0.05404976	1.81706455
chr4	3845038	3845097	60	1	2	5	0.30102999	0.00317045	1	12	0.05404976	1.46403142
chr4	3845097	4207758	362662	1	2	5	0.30102999	0.02473314	1	9	0.05404976	0.76806864
chr4	4207758	4207817	60	1	3	5	0.51676182	0.02473314	1	9	0.02438896	0.76806864
chr4	4207817	4249253	41437	1	3	4	0.51676182	0.01077081	1	9	0.02438896	1.01542894
chr4	4249253	4283574	34322	2	3	4	0.51676182	0.00530919	1	10	0.02438896	1.2568129
chr4	4283574	4283633	60	1	3	5	0.51676182	0.01320236	1	10	0.02438896	0.97390707
chr4	4283633	4331339	47707	1	3	4	0.51676182	0.00530919	1	10	0.02438896	1.2568129
chr4	4331339	4370244	38906	1	3	4	0.51676182	0.01077081	1	9	0.02438896	1.01542894
chr4	4370244	4441881	71638	1	2	4	0.61140001	0.01077081	0	9	0	1.01542894
chr4	4441881	4485131	43251	2	2	4	0.61140001	0.00530919	0	10	0	1.2568129
chr4	4485131	4534990	49860	1	2	4	0.61140001	0.01077081	0	9	0	1.01542894
chr4	4534990	4564313	29324	2	2	5	0.61140001	0.02473314	0	9	0	0.76806864
chr4	4564313	4673343	109031	3	3	5	0.51676182	0.00666883	1	11	0.02438896	1.20557689
chr4	4673343	4899535	226193	4	3	4	0.51676182	0.00530919	1	10	0.02438896	1.2568129
chr4	4899535	4979855	80321	2	3	6	0.51676182	0.01518174	1	11	0.02438896	0.9449689
chr4	4979855	5036659	56805	2	3	6	0.51676182	0.00778066	1	12	0.02438896	1.17038931
chr4	5036659	5036718	60	1	3	8	0.51676182	0.03209037	1	12	0.02438896	0.72109894
chr4	5036718	5139896	103179	2	3	6	0.51676182	0.00778066	1	12	0.02438896	1.17038931
chr4	5139896	5139955	60	1	3	7	0.51676182	0.01667721	1	12	0.02438896	0.92532268
chr4	5139955	5211906	71952	1	3	7	0.51676182	0.03037338	1	11	0.02438896	0.73110763
chr4	5211906	5279133	67228	1	3	6	0.51676182	0.01518174	1	11	0.02438896	0.9449689
chr4	5279133	5375619	96487	3	3	6	0.51676182	0.00778066	1	12	0.02438896	1.17038931
chr4	5375619	5449861	74243	2	3	6	0.51676182	0.01518174	1	11	0.02438896	0.9449689
chr4	5449861	5506529	56669	2	3	6	0.51676182	0.00778066	1	12	0.02438896	1.17038931
chr4	5506529	5722587	216059	6	3	6	0.30102999	0.00778066	2	12	0.08289318	1.17038931
chr4	5722587	5747161	24575	2	3	6	0.30102999	0.00166733	2	14	0.08289318	1.70763027
chr4	5747161	5877140	129980	2	3	6	0.30102999	0.00778066	2	12	0.08289318	1.17038931
chr4	5877140	5877199	60	1	3	7	0.30102999	0.00859896	2	13	0.08289318	1.14735594
chr4	5877199	5948790	71592	2	3	7	0.30102999	0.01667721	2	12	0.08289318	0.92532268
chr4	5948790	5948849	60	1	3	8	0.30102999	0.01767679	2	13	0.08289318	0.91308053
chr4	5948849	6016300	67452	1	3	8	0.51676182	0.03209037	1	12	0.02438896	0.72109894
chr4	6016300	6409495	393196	8	3	7	0.51676182	0.01667721	1	12	0.02438896	0.92532268
chr4	6409495	6409554	60	1	3	7	0.30102999	0.01667721	2	12	0.08289318	0.92532268
chr4	6409554	6544577	135024	3	3	6	0.51676182	0.00778066	1	12	0.02438896	1.17038931
chr4	6544577	6544636	60	1	3	6	0.30102999	0.00778066	2	12	0.08289318	1.17038931
chr4	6544636	6698609	153974	3	3	6	0.51676182	0.00778066	1	12	0.02438896	1.17038931
chr4	6698609	6826017	127409	4	3	6	0.30102999	0.00778066	2	12	0.08289318	1.17038931
chr4	6826017	6871516	45500	1	3	6	0.30102999	0.01518174	2	11	0.08289318	0.9449689
chr4	6871516	6924403	52888	1	3	6	0.30102999	0.02793176	2	10	0.08289318	0.74627054
chr4	6924403	6924462	60	1	3	6	0.30102999	0.00778066	2	12	0.08289318	1.17038931
chr4	6924462	7100541	176080	4	3	6	0.30102999	0.01518174	2	11	0.08289318	0.9449689
chr4	7100541	7212835	112295	3	3	6	0.30102999	0.00778066	2	12	0.08289318	1.17038931

chr4	7212835	7212894	60	1	3	6	0.30102999	0.00373523	2	13	0.08289318	1.42397267
chr4	7212894	7633947	421054	9	3	6	0.30102999	0.00778066	2	12	0.08289318	1.17038931
chr4	7633947	7668912	34966	2	3	6	0.30102999	0.00373523	2	13	0.08289318	1.42397267
chr4	7668912	7731835	62924	2	3	6	0.30102999	0.00778066	2	12	0.08289318	1.17038931
chr4	7731835	7760991	29157	2	3	6	0.30102999	0.00373523	2	13	0.08289318	1.42397267
chr4	7760991	7831152	70162	1	3	6	0.30102999	0.00778066	2	12	0.08289318	1.17038931
chr4	7831152	7880957	49806	1	3	6	0.30102999	0.01518174	2	11	0.08289318	0.9449689
chr4	7880957	7938986	58030	2	3	6	0.30102999	0.00373523	2	13	0.08289318	1.42397267
chr4	7938986	8019397	80412	3	3	6	0.17593012	0.00166733	3	14	0.17593012	1.70763027
chr4	8019397	8072127	52731	2	3	6	0.30102999	0.00166733	2	14	0.08289318	1.70763027
chr4	8072127	8113835	41709	2	3	6	0.17593012	0.00166733	3	14	0.17593012	1.70763027
chr4	8113835	8224525	110691	3	3	6	0.30102999	0.00166733	2	14	0.08289318	1.70763027
chr4	8224525	8308781	84257	2	3	6	0.51676182	0.00166733	1	14	0.02438896	1.70763027
chr4	8308781	8368180	59400	2	3	6	0.51676182	6.86E-04	1	15	0.02438896	2.02387299
chr4	8368180	8461355	93176	2	3	6	0.51676182	0.00778066	1	12	0.02438896	1.17038931
chr4	8461355	8575359	114005	3	3	6	0.51676182	0.00373523	1	13	0.02438896	1.42397267
chr4	8575359	8607464	32106	1	3	5	0.93173516	0.00666883	0	11	0	1.20557689
chr4	8607464	8874214	266751	6	3	2	0.93173516	0.00221948	0	8	0	1.44210395
chr4	8874214	8882456	8243	1	2	2	0.61140001	0.00221948	0	8	0	1.44210395
chr4	8882456	8900505	18050	3	2	2	0.61140001	9.54E-04	0	9	0	1.74076927
chr4	8900505	8900564	60	1	3	2	0.93173516	3.90E-04	0	10	0	2.0620585
chr4	8900564	9627944	727381	1	1	2	0.30102999	3.90E-04	0	10	0	2.0620585
chr4	9627944	9766686	138743	2	1	5	0.30102999	0.00141024	0	13	0	1.7506523
chr4	9766686	9829086	62401	2	1	6	0.30102999	0.00373523	0	13	0	1.42397267
chr4	9829086	9865168	36083	1	1	5	0.30102999	0.01320236	0	10	0	0.97390707
chr4	9865168	9896172	31005	1	1	2	0.30102999	0.00221948	0	8	0	1.44210395
chr4	9896172	10076446	180275	5	1	4	0.30102999	0.02074938	0	8	0	0.79906872
chr4	10076446	10252290	175845	2	1	3	0.30102999	0.0079614	0	8	0	1.07548421
chr4	10252290	10417389	165100	1	0	3	0	0.0079614	0	8	0	1.07548421
chr4	10417389	10641640	224252	5	0	7	0	0.01667721	1	12	0.30102999	0.92532268
chr4	10641640	10747885	106246	3	0	8	0	0.03209037	1	12	0.30102999	0.72109894
chr4	10747885	10865325	117441	1	0	8	0	0.05490675	1	11	0.30102999	0.55623409
chr4	10865325	10865384	60	1	0	9	0	0.09154957	1	11	0.30102999	0.41444892
chr4	10865384	11091079	225696	1	0	6	0	0.04875589	1	9	0.30102999	0.5732208
chr4	11091079	11091138	60	1	0	7	0	0.08584816	1	9	0.30102999	0.42015402
chr4	11091138	11280802	189665	1	0	3	0	0.0079614	1	8	0.30102999	1.07548421
chr4	11280802	11400736	119935	3	0	4	0	0.02074938	1	8	0.30102999	0.79906872
chr4	11400736	11401761	1026	2	0	10	0	0.14303407	1	11	0.30102999	0.30102999
chr4	11401761	11431746	29986	1	0	6	0	0.04875589	1	9	0.30102999	0.5732208
chr4	11431746	11612389	180644	1	0	6	0	0.08122616	1	8	0.30102999	0.4250187
chr4	11612389	11612448	60	1	0	6	0	0.04875589	1	9	0.30102999	0.5732208
chr4	11612448	11750089	137642	1	0	3	0	0.01598258	1	7	0.30102999	0.84395715
chr4	11750089	11857246	107158	2	0	4	0	0.02074938	1	8	0.30102999	0.79906872
chr4	11857246	11857305	60	1	0	7	0	0.03037338	1	11	0.30102999	0.73110763
chr4	11857305	11977464	120160	1	0	7	0	0.05232577	1	10	0.30102999	0.56314362
chr4	11977464	12273633	296170	2	0	6	0	0.30102999	1	5	0.30102999	0.12309572
chr4	12273633	12273692	60	1	0	8	0	0.08923391	1	10	0.30102999	0.4167287
chr4	12273692	12382630	108939	1	0	8	0	0.20764654	1	8	0.30102999	0.20764654
chr4	12382630	12382689	60	1	0	8	0	0.13872638	1	9	0.30102999	0.30102999
chr4	12382689	12496097	113409	1	0	8	0	0.20764654	1	8	0.30102999	0.20764654
chr4	12496097	12496156	60	1	0	9	0	0.09154957	1	11	0.30102999	0.41444892
chr4	12496156	12595138	98983	1	0	5	0	0.07511598	1	7	0.30102999	0.43181735
chr4	12595138	12713420	118283	1	0	5	0	0.07511598	0	7	0	0.43181735
chr4	12713420	12802689	89270	2	0	8	0	0.30102999	0	7	0	0.13499366
chr4	12802689	12976287	173599	1	0	5	0	0.07511598	0	7	0	0.43181735
chr4	12976287	12976346	60	1	0	5	0	0.04407651	0	8	0	0.58747015
chr4	12976346	13214984	238639	1	0	5	0	0.07511598	0	7	0	0.43181735
chr4	13214984	13343352	128369	1	0	4	0	0.11390336	0	5	0	0.30102999
chr4	13343352	13369949	26598	2	0	5	0	0.07511598	0	7	0	0.43181735
chr4	13369949	13481105	111157	3	0	4	0	0.06713722	0	6	0	0.44141547
chr4	13481105	13542775	61671	2	0	6	0	0.129913	0	7	0	0.30102999
chr4	13542775	13542834	60	1	0	6	0	0.08122616	0	8	0	0.4250187
chr4	13542834	13625717	82884	2	0	5	0	0.07511598	0	7	0	0.43181735
chr4	13625717	13628601	2885	2	0	5	0	0.02473314	0	9	0	0.76806864
chr4	13628601	13718621	90021	1	0	4	0	0.02074938	0	8	0	0.79906872
chr4	13718621	13820583	101963	2	0	4	0	0.01077081	0	9	0	1.01542894
chr4	13820583	13889044	68462	2	0	5	0	0.00666883	0	11	0	1.20557689
chr4	13889044	13950562	61519	2	0	5	0	0.00141024	0	13	0	1.7506523
chr4	13950562	14062753	112192	1	0	5	0	0.00317045	0	12	0	1.46403142
chr4	14062753	14062812	60	1	0	8	0	0.00909834	0	14	0	1.13428373
chr4	14062812	14358235	295424	3	0	7	0	0.00859896	0	13	0	1.14735594
chr4	14358235	14358294	60	1	0	7	0	0.00413023	0	14	0	1.39918918
chr4	14358294	14454449	96156	1	0	6	0	0.00778066	0	12	0	1.17038931
chr4	14454449	14578096	123648	3	0	6	0	0.00373523	0	13	0	1.42397267
chr4	14578096	14631374	53279	2	0	8	0	0.00909834	0	14	0	1.13428373
chr4	14631374	14631433	60	1	0	10	0	0.03344703	0	14	0	0.71353304
chr4	14631433	14692174	60742	1	0	9	0	0.01817691	0	14	0	0.90719478
chr4	14692174	14692233	60	1	0	10	0	0.01817691	0	15	0	0.90719478
chr4	14692233	14787897	95665	2	0	9	0	0.00926608	0	15	0	1.13004284
chr4	14787897	14820108	32212	1	0	8	0	0.00433294	0	15	0	1.3873209
chr4	14820108	14987869	167762	4	0	8	0	0.00909834	0	14	0	1.13428373
chr4	14987869	15135527	147659	5	0	10	0	0.01817691	0	15	0	0.90719478
chr4	15135527	15302739	167213	1	0	9	0	0.00926608	0	15	0	1.13004284
chr4	15302739	15371494	68756	3	0	9	0	0.00433294	0	16	0	1.3873209
chr4	15371494	15478358	106865	4	0	10	0	0.00909834	0	16	0	1.13428373
chr4	15478358	15685537	207180	5	0	9	0	0.00926608	0	15	0	1.13004284
chr4	15685537	15724416	38880	1	0	9	0	0.01817691	0	14	0	0.90719478
chr4	15724416	15768035	43620	2	0	10	0	0.03344703	0	14	0	0.71353304
chr4	15768035	15768094	60	1	0	10	0	0.01817691	0	15	0	0.90719478
chr4	15768094	15818178	50085	1	0	10	0	0.03344703	0	14	0	0.71353304
chr4	15818178	15855129	36952	2	0	12	0	0.09290028	0	14	0	0.41314172
chr4	15855129	16034980	179852	6	0	10	0	0.03344703	0	14	0	0.71353304
chr4	16034980	16246453	211474	4	0	10	0	0.0574087	0	13	0	0.5498098
chr4	16246453	16246512	60	1	0	11	0	0.0574087	0	14	0	0.5498098
chr4	16246512	16358433	111922	1	0	11	0	0.09334429	0	13	0	0.41271556
chr4	16358433	16482734	124302	1	0	10	0	0.0574087	0	13	0	0.5498098
chr4	16482734	16482793	60	1	0	10	0	0.03344703	0	14	0	0.71353304
chr4	16482793	16554028	71236	1	0	9	0	0.01817691	0	14	0	0.90719478
chr4	16554028	16590372	36345	2	1	9	0.30102999	0.01817691	0	14	0	0.90719478
chr4	16590372	16773872	183501	6	1	9	0.30102999	0.00926608	0	15	0	1.13004284
chr4	16773872	16809426	35555	1	1	9	0.30102999	0.01817691	0	14	0	0.90719478
chr4	16809426	16833244	23819	2	1	9	0.1218695	0.01817691	1	14	0.1218695	0.90719478
chr4	16833244	16899621	66378	2	1	9	0.1218695	0.00926608	1	15	0.1218695	1.13004284
chr4	16899621	16900656	1036	2	1	10	0.1218695	0.01817691	1	15	0.1218695	0.90719478
chr4	16900656	17074820	174165	2	1	9	0.1218695	0.00926608	1	15	0.1218695	1.13004284
chr4	17074820	17074879	60	1	1	9	0.1218695	0.00433294	1	16	0.1218695	1.3873209
chr4	17074879	17193258	118380	1	1	9	0.1218695	0.00926608	1	15	0.1218695	1.13004284
chr4	17193258	17488306	295049	3	0	9	0	0.00926608	1	15	0.30102999	1.13004284
chr4	17488306	17600224	111919	5	0	10	0	0.01817691	1	15	0.30102999	0.90719478
chr4	17600224	17625188	24965	2	1	10	0.1218695	0.01817691	1	15	0.1218695	0.90719478
chr4	17625188	17653684	28497	1	1	9	0.1218695	0.00926608	1	15	0.1218695	1.13004284
chr4	17653684	17689872	36189	1	0	9	0	0.00926608	1	15	0.30102999	1.13004284
chr4	17689872	17745251	55380	1	0	9	0	0.01817691	1	14	0.30102999	0.90719478
chr4	17745251	17745310	60	1	1	9	0.1218695	0.01817691	1	14	0.1218695	0.90719478
chr4	17745310	17845097	99788	4	0	9	0	0.01817691	1	14	0.30102999	0.90719478
chr4	17845097	17883744	38648	2	0	10	0	0.01817691	1	15	0.30102999	0.90719478
chr4	17883744	17963667	79924	2	0	10	0	0.03344703	1	14	0.30102999	0.71353304
chr4	17963667	18015153	51487	1	0	8	0	0.01767679	1	13	0.30102999	0.91308053
chr4	18015153	18015212	60	1	0	8	0	0.00909834	1	14	0.30102999	1.13428373
chr4	18015212	18201194	185983	1	0	7	0	0.03037338	1	11	0.30102999	0.73110763
chr4	18201194	18201253	60	1	0	8	0	0.05490675	1	11	0.30102999	0.55623409
chr4	18201253	18391482	190230	1	0	7	0	0.03037338	1	11	0.30102999	0.73110763
chr4	18391482	18512406	120925	1	0	5	0	0.04407651	1	8	0.30102999	0.58747015
chr4	18512406	18633460	121055	1	0	4	0	0.18734596	1	4	0.30102999	0.18734596

chr4	18633460	18633519	60	1	0	4	0	0.11390336	1	5	0.30102999	0.30102999
chr4	18633519	18839589	206071	1	0	3	0	0.17593012	1	3	0.30102999	0.17593012
chr4	18839589	18839648	60	1	0	3	0	0.05670724	1	5	0.30102999	0.45545077
chr4	18839648	19176896	337249	2	0	0	0	0	0	2	0	0.61140001
chr4	19176896	19370059	193164	2	0	0	0	0	0	1	0	0.30102999
chr4	19370059	19591913	221855	3	0	3	0	0.17593012	0	3	0	0.17593012
chr4	19591913	19694001	102089	2	0	7	0	0.60763643	0	4	0	0.03812622
chr4	19694001	19792069	98069	2	0	9	0	0.76806864	1	5	0.30102999	0.02473314
chr4	19792069	19792128	60	1	0	9	0	0.5732208	1	6	0.30102999	0.04875589
chr4	19792128	19990818	198691	2	0	6	0	0.20064824	1	6	0.30102999	0.20064824
chr4	19990818	20216503	225686	2	0	9	0	0.14135546	2	10	0.61140001	0.30102999
chr4	20216503	20254688	38186	2	0	11	0	0.0574087	2	14	0.61140001	0.5498098
chr4	20254688	20294750	40063	2	0	11	0	0.0331093	2	15	0.61140001	0.71538971
chr4	20294750	20324879	30130	1	0	11	0	0.0574087	2	14	0.61140001	0.5498098
chr4	20324879	20324938	60	1	0	12	0	0.09290028	2	14	0.61140001	0.41314172
chr4	20324938	20394896	69959	2	0	11	0	0.0574087	2	14	0.61140001	0.5498098
chr4	20394896	20394955	60	1	0	12	0	0.01667721	2	17	0.61140001	0.92532268
chr4	20394955	20490678	95724	2	0	11	0	0.01767679	1	16	0.30102999	0.91308053
chr4	20490678	20541187	50510	3	0	11	0	0.00859896	2	17	0.61140001	1.14735594
chr4	20541187	20563947	22761	1	0	11	0	0.00859896	1	17	0.30102999	1.14735594
chr4	20563947	20564006	60	1	0	12	0	0.01667721	1	17	0.30102999	0.92532268
chr4	20564006	20620650	56645	2	0	11	0	0.00859896	1	17	0.30102999	1.14735594
chr4	20620650	20714840	94191	3	0	12	0	0.01667721	1	17	0.30102999	0.92532268
chr4	20714840	20714899	60	1	0	13	0	0.03037338	1	17	0.30102999	0.73110763
chr4	20714899	21403260	688362	19	0	12	0	0.01667721	1	17	0.30102999	0.92532268
chr4	21403260	21482176	78917	3	0	11	0	0.00859896	1	17	0.30102999	1.14735594
chr4	21482176	21602980	120805	3	0	10	0	0.00413023	1	17	0.30102999	1.39918918
chr4	21602980	21868325	265346	4	0	10	0	0.00909834	1	16	0.30102999	1.13428373
chr4	21868325	21868384	60	1	0	12	0	0.01667721	1	17	0.30102999	0.92532268
chr4	21868384	22006216	137833	2	0	10	0	0.00909834	1	16	0.30102999	1.13428373
chr4	22006216	22006275	60	1	0	10	0	0.00413023	1	17	0.30102999	1.39918918
chr4	22006275	22176651	170377	1	0	9	0	0.00926608	1	15	0.30102999	1.13004284
chr4	22176651	22422757	246107	3	0	8	0	0.03209037	1	12	0.30102999	0.72109894
chr4	22422757	22459005	36249	2	0	8	0	0.01767679	1	13	0.30102999	0.91308053
chr4	22459005	22459064	60	1	0	8	0	0.00909834	1	14	0.30102999	1.13428373
chr4	22459064	22493944	34881	1	0	8	0	0.03209037	1	12	0.30102999	0.72109894
chr4	22493944	22526538	32595	1	0	8	0	0.05490675	1	11	0.30102999	0.55623409
chr4	22526538	22716554	190017	2	0	6	0	0.129913	1	7	0.30102999	0.30102999
chr4	22716554	22762063	45510	1	0	5	0	0.07511598	1	7	0.30102999	0.43181735
chr4	22762063	22927820	165758	2	0	3	0	0.01598258	1	7	0.30102999	0.84395715
chr4	22927820	23211015	283196	3	0	6	0	0.04875589	1	9	0.30102999	0.5732208
chr4	23211015	23313776	102762	1	0	4	0	0.01077081	1	9	0.30102999	1.01542894
chr4	23313776	23515063	201288	1	0	1	0	3.39E-04	1	8	0.30102999	1.94674965
chr4	23515063	23703416	188354	2	0	2	0	9.54E-04	1	9	0.30102999	1.74076927
chr4	23703416	23756696	53281	2	0	4	0	0.00530919	1	10	0.30102999	1.2568129
chr4	23756696	23756755	60	1	0	4	0	0.00247414	1	11	0.30102999	1.52371709
chr4	23756755	23806994	50240	2	0	3	0	0.00378107	1	9	0.30102999	1.33111237
chr4	23806994	23807053	60	1	0	4	0	0.00530919	1	10	0.30102999	1.2568129
chr4	23807053	23886460	79408	2	0	1	0	3.39E-04	1	8	0.30102999	1.94674965
chr4	23886460	23886517	58	1	0	4	0	0.00530919	1	10	0.30102999	1.2568129
chr4	23886517	24068622	182106	1	0	1	0	4.80E-05	1	10	0.30102999	2.6588298
chr4	24068622	24284414	215793	2	0	2	0	3.90E-04	1	10	0.30102999	2.0620585
chr4	24284414	24284473	60	1	0	6	0	0.00373523	1	13	0.30102999	1.42397267
chr4	24284473	24521837	237365	2	0	6	0	0.00778066	1	12	0.30102999	1.17038931
chr4	24521837	24548281	26445	2	0	7	0	0.01667721	1	12	0.30102999	0.92532268
chr4	24548281	24700363	152083	3	0	6	0	0.00778066	1	12	0.30102999	1.17038931
chr4	24700363	24798376	98014	2	0	7	0	0.00183109	1	15	0.30102999	1.68336308
chr4	24798376	24854752	56377	2	0	8	0	0.00433294	1	15	0.30102999	1.3873209
chr4	24854752	24854811	60	1	0	9	0	0.00926608	1	15	0.30102999	1.13004284
chr4	24854811	24893167	38357	1	0	8	0	0.00433294	1	15	0.30102999	1.3873209
chr4	24893167	24893226	60	1	0	8	0	0.00188722	1	16	0.30102999	1.67551277
chr4	24893226	25008199	114974	3	0	7	0	7.41E-04	1	16	0.30102999	2.00316618
chr4	25008199	25175412	167214	3	0	7	0	0.00183109	1	15	0.30102999	1.68336308
chr4	25175412	25277869	102458	3	0	8	0	0.00188722	1	16	0.30102999	1.67551277
chr4	25277869	25315638	37770	2	0	9	0	0.00433294	1	16	0.30102999	1.3873209
chr4	25315638	25352246	36609	1	0	9	0	0.00926608	1	15	0.30102999	1.13004284
chr4	25352246	25352305	60	1	0	10	0	0.01817691	1	15	0.30102999	0.90719478
chr4	25352305	25420099	67795	3	0	9	0	0.00926608	1	15	0.30102999	1.13004284
chr4	25420099	25661606	241508	3	0	8	0	0.00433294	1	15	0.30102999	1.3873209
chr4	25661606	25725177	63572	5	0	9	0	0.00926608	1	15	0.30102999	1.13004284
chr4	25725177	25831923	106747	3	0	10	0	0.01817691	1	15	0.30102999	0.90719478
chr4	25831923	25831982	60	1	0	11	0	0.01767679	1	16	0.30102999	0.91308053
chr4	25831982	25903066	71085	2	0	10	0	0.01817691	1	15	0.30102999	0.90719478
chr4	25903066	25941873	38808	2	0	10	0	0.00909834	1	16	0.30102999	1.13428373
chr4	25941873	25941932	60	1	0	11	0	0.01767679	1	16	0.30102999	0.91308053
chr4	25941932	26156709	214778	1	0	9	0	0.00926608	1	15	0.30102999	1.13004284
chr4	26156709	26156768	60	1	0	9	0	0.00433294	1	16	0.30102999	1.3873209
chr4	26156768	26252617	95850	1	0	9	0	0.01817691	1	14	0.30102999	0.90719478
chr4	26252617	26252676	60	1	0	9	0	0.00926608	1	15	0.30102999	1.13004284
chr4	26252676	26344351	91676	1	0	9	0	0.0331093	1	13	0.30102999	0.71538971
chr4	26344351	26376036	31686	2	0	9	0	0.01817691	1	14	0.30102999	0.90719478
chr4	26376036	26376095	60	1	0	9	0	0.00926608	1	15	0.30102999	1.13004284
chr4	26376095	26459600	83506	2	0	8	0	0.01767679	1	13	0.30102999	0.91308053
chr4	26459600	26585136	125537	3	0	8	0	0.00433294	1	15	0.30102999	1.3873209
chr4	26585136	26585195	60	1	0	9	0	0.00433294	1	16	0.30102999	1.3873209
chr4	26585195	26641819	56625	2	0	9	0	0.00926608	1	15	0.30102999	1.13004284
chr4	26641819	26675431	33613	1	0	8	0	0.01767679	1	13	0.30102999	0.91308053
chr4	26675431	26756765	81335	2	0	8	0	0.03209037	1	12	0.30102999	0.72109894
chr4	26756765	26865330	108566	2	0	8	0	0.13872638	1	9	0.30102999	0.30102999
chr4	26865330	26921275	55946	1	0	8	0	0.20764654	1	8	0.30102999	0.20764654
chr4	26921275	26959278	38004	1	0	7	0	0.13499366	1	8	0.30102999	0.30102999
chr4	26959278	26959337	60	1	0	7	0	0.08584816	1	9	0.30102999	0.42015402
chr4	26959337	27025347	66011	1	0	7	0	0.20469099	1	7	0.30102999	0.20469099
chr4	27025347	27269113	243767	4	0	8	0	0.30102999	1	7	0.30102999	0.13499366
chr4	27269113	27574902	305790	2	0	5	0	0.19510895	1	5	0.30102999	0.19510895
chr4	27574902	27574961	60	1	0	7	0	0.20469099	1	7	0.30102999	0.20469099
chr4	27574961	27840623	265663	2	0	4	0	0.30102999	1	3	0.30102999	0.10122019
chr4	27840623	28005975	165353	2	0	4	0	0.11390336	1	5	0.30102999	0.30102999
chr4	28005975	28161143	155169	1	0	4	0	0.18734596	1	4	0.30102999	0.18734596
chr4	28161143	28357602	196460	1	0	3	0	0.17593012	1	3	0.30102999	0.17593012
chr4	28357602	28451250	93649	1	0	3	0	0.30102999	1	2	0.30102999	0.08289318
chr4	28451250	28764611	313362	2	0	3	0	0.30102999	0	2	0	0.08289318
chr4	28764611	28955673	191063	3	0	3	0	0.17593012	1	3	0.30102999	0.17593012
chr4	28955673	28955732	60	1	0	3	0	0.05670724	1	5	0.30102999	0.45545077
chr4	28955732	29277508	321777	3	0	3	0	0.17593012	0	3	0	0.17593012
chr4	29277508	29455453	177946	1	0	0	0	0	0	2	0	0.61140001
chr4	29455453	29455512	60	1	0	0	0	0	0	4	0	1.26272838
chr4	29455512	29894102	438591	3	0	0	0	0	0	3	0	0.93173516
chr4	29894102	29894161	60	1	0	3	0	0.03070643	0	6	0	0.63695542
chr4	29894161	30182135	287975	1	0	3	0	0.10122019	0	4	0	0.30102999
chr4	30182135	30182194	60	1	0	4	0	0.11390336	1	5	0.30102999	0.30102999
chr4	30182194	30597944	415751	2	0	3	0	0.10122019	0	4	0	0.30102999
chr4	30597944	30598003	60	1	0	4	0	0.03812622	1	7	0.30102999	0.60763643
chr4	30598003	30725133	127131	1	0	2	0	0.08289318	1	3	0.30102999	0.30102999
chr4	30725133	31213207	488075	9	0	0	0	0	0	3	0	0.93173516
chr4	31213207	31576042	362836	3	0	0	0	0	1	4	0.30102999	1.26272838
chr4	31576042	31576101	60	1	0	1	0	0.01091641	1	4	0.30102999	0.76005302
chr4	31576101	31768439	192339	1	0	0	0	0	1	4	0.30102999	1.26272838
chr4	31768439	31768498	60	1	0	1	0	0.01091641	1	4	0.30102999	0.76005302
chr4	31768498	32023735	255238	1	0	0	0	0	0	3	0	0.93173516
chr4	33080365	33080424	60	1	0	2	0	0.08289318	1	3	0.30102999	0.30102999
chr4	33080424	33281326	200903	1	0	0	0	0	1	3	0.30102999	0.93173516
chr4	33281326	33446367	165042	2	0	2	0	0.02162467	1	5	0.30102999	0.68214471
chr4	33446367	33617563	171197	2	0	2	0	0.01053319	1	6	0.30102999	0.91219088

chr4	33617563	34033992	416430	3	0	0	0	0	1	2	0.30102999	0.61140001
chr4	34033992	34106870	72879	2	0	0	0	0	1	4	0.30102999	1.26272838
chr4	34106870	34260744	153875	3	0	0	0	0	1	5	0.30102999	1.60515106
chr4	34260744	34833500	572757	3	0	0	0	0	1	2	0.30102999	0.61140001
chr4	34833500	35303722	470223	5	0	0	0	0	1	3	0.30102999	0.93173516
chr4	35303722	35485786	182065	2	0	2	0	0.08289318	1	3	0.30102999	0.30102999
chr4	35485786	35698021	212236	2	0	4	0	0.18734596	1	4	0.30102999	0.18734596
chr4	35698021	35935924	237904	2	0	4	0	0.11390336	1	5	0.30102999	0.30102999
chr4	35935924	35935983	60	1	0	6	0	0.20064824	1	6	0.30102999	0.20064824
chr4	35935983	36093572	157590	3	0	5	0	0.30102999	1	4	0.30102999	0.11390336
chr4	36093572	36093631	60	1	0	6	0	0.30102999	1	5	0.30102999	0.12309572
chr4	36093631	36272268	178638	5	0	6	0	0.63695542	1	3	0.30102999	0.03070643
chr4	36272268	36272327	60	1	0	7	0	0.20469099	1	7	0.30102999	0.20469099
chr4	36272327	36434278	161952	3	0	7	0	0.43181735	1	5	0.30102999	0.07511598
chr4	36434278	36434337	60	1	0	8	0	0.20764654	1	8	0.30102999	0.20764654
chr4	36434337	36593341	159005	2	0	7	0	0.13499366	1	8	0.30102999	0.30102999
chr4	36593341	36833002	239662	3	0	7	0	0.01667721	1	12	0.30102999	0.92532268
chr4	36833002	37114858	281857	1	0	6	0	0.02793176	1	10	0.30102999	0.74627054
chr4	37114858	37236591	121734	1	0	4	0	0.01077081	1	9	0.30102999	1.01542894
chr4	37236591	37300587	63997	2	1	4	0.1218695	0.01077081	1	9	0.1218695	1.01542894
chr4	37300587	37300646	60	1	1	4	0.1218695	0.00530919	1	10	0.1218695	1.2568129
chr4	37300646	37455978	155333	4	1	3	0.1218695	0.00378107	1	9	0.1218695	1.33111237
chr4	37455978	37456037	60	1	1	3	0.1218695	0.00170589	1	10	0.1218695	1.61091002
chr4	37456037	37559630	103594	2	1	3	0.1218695	0.01598258	1	7	0.1218695	0.84395715
chr4	37559630	37590493	30864	2	1	3	0.1218695	0.00170589	1	10	0.1218695	1.61091002
chr4	37590493	37684725	94233	2	0	3	0	0.0079614	1	8	0.30102999	1.07548421
chr4	37684725	37684784	60	1	1	3	0.1218695	0.0079614	1	8	0.1218695	1.07548421
chr4	37684784	37726834	42051	1	1	2	0.1218695	0.00221948	1	8	0.1218695	1.44210395
chr4	37726834	37836222	109389	1	0	2	0	0.00221948	1	8	0.30102999	1.44210395
chr4	37836222	37850275	14054	2	0	3	0	7.28E-04	1	11	0.30102999	1.91540774
chr4	37850275	37911055	60781	1	0	2	0	0.00221948	1	8	0.30102999	1.44210395
chr4	37911055	37953144	42090	2	1	2	0.1218695	0.00221948	1	8	0.1218695	1.44210395
chr4	37953144	37953203	60	1	1	3	0.1218695	0.0079614	1	8	0.1218695	1.07548421
chr4	37953203	38029393	76191	2	0	3	0	0.01598258	1	7	0.30102999	0.84395715
chr4	38029393	38074093	44701	2	2	3	0.30102999	0.00378107	1	9	0.05404976	1.33111237
chr4	38074093	38211375	137283	3	2	3	0.30102999	0.0079614	1	8	0.05404976	1.07548421
chr4	38211375	38211434	60	1	2	3	0.30102999	0.00378107	1	9	0.05404976	1.33111237
chr4	38211434	38290242	78809	1	2	2	0.30102999	9.54E-04	1	9	0.05404976	1.74076927
chr4	38290242	38290301	60	1	2	2	0.30102999	3.90E-04	1	10	0.05404976	2.0620585
chr4	38290301	38452743	162443	1	0	2	0	9.54E-04	1	9	0.30102999	1.74076927
chr4	38452743	38512216	59474	2	1	2	0.1218695	9.54E-04	1	9	0.1218695	1.74076927
chr4	38512216	38626231	114016	2	1	1	0.1218695	1.30E-04	1	9	0.1218695	2.29264207
chr4	38626231	38626290	60	1	1	4	0.1218695	0.01077081	1	9	0.1218695	1.01542894
chr4	38626290	38683980	57691	2	1	1	0.1218695	1.30E-04	1	9	0.1218695	2.29264207
chr4	38683980	38684039	60	1	1	4	0.1218695	0.01077081	1	9	0.1218695	1.01542894
chr4	38684039	38730524	46486	1	1	3	0.1218695	0.00378107	1	9	0.1218695	1.33111237
chr4	38730524	38879793	149270	5	1	2	0.1218695	9.54E-04	1	9	0.1218695	1.74076927
chr4	38879793	38926934	47142	1	1	1	0.1218695	1.30E-04	1	9	0.1218695	2.29264207
chr4	38926934	38968751	41818	2	1	2	0.1218695	9.54E-04	1	9	0.1218695	1.74076927
chr4	38968751	39021117	52367	2	1	4	0.1218695	0.01077081	1	9	0.1218695	1.01542894
chr4	39021117	39021176	60	1	1	5	0.1218695	0.00666883	1	11	0.1218695	1.20557689
chr4	39021176	39090404	69229	1	1	5	0.1218695	0.01320236	1	10	0.1218695	0.97390707
chr4	39090404	39160988	70585	2	1	5	0.1218695	0.02473314	1	9	0.1218695	0.76806864
chr4	39160988	39219586	58599	2	1	5	0.1218695	0.01320236	1	10	0.1218695	0.97390707
chr4	39219586	39276568	56983	3	1	5	0.1218695	0.00666883	1	11	0.1218695	1.20557689
chr4	39276568	39552971	276404	10	1	5	0.1218695	0.00317045	1	12	0.1218695	1.46403142
chr4	39552971	39619343	66373	3	1	5	0.1218695	0.00141024	1	13	0.1218695	1.7506523
chr4	39619343	39698881	79539	2	1	3	0.1218695	2.93E-04	1	12	0.1218695	2.24559516
chr4	39698881	39750115	51235	3	1	5	0.1218695	0.00141024	1	13	0.1218695	1.7506523
chr4	39750115	39781062	30948	1	1	4	0.1218695	4.45E-04	1	13	0.1218695	2.13824703
chr4	39781062	39942183	161122	4	1	4	0.30102999	4.45E-04	0	13	0	2.13824703
chr4	39942183	40027878	85696	3	2	4	0.61140001	4.45E-04	0	13	0	2.13824703
chr4	40027878	40121747	93870	3	2	5	0.61140001	0.00141024	0	13	0	1.7506523
chr4	40121747	40153440	31694	2	2	6	0.61140001	0.00373523	0	13	0	1.42397267
chr4	40153440	40189332	35893	1	2	6	0.61140001	0.01518174	0	11	0	0.9449689
chr4	40189332	40239298	49967	4	2	6	0.61140001	0.00778066	0	12	0	1.17038931
chr4	40239298	40348687	109390	2	2	5	0.61140001	0.00666883	0	11	0	1.20557689
chr4	40348687	40379060	30374	1	2	4	0.61140001	0.00247414	0	11	0	1.52371709
chr4	40379060	40430483	51424	2	2	5	0.61140001	0.00666883	0	11	0	1.20557689
chr4	40430483	40513326	82844	2	2	4	0.61140001	0.00247414	0	11	0	1.52371709
chr4	40513326	40588332	75007	3	2	5	0.61140001	0.00666883	0	11	0	1.20557689
chr4	40588332	40629327	40996	1	2	4	0.61140001	0.00247414	0	11	0	1.52371709
chr4	40629327	40748354	119028	1	1	4	0.30102999	0.00247414	0	11	0	1.52371709
chr4	40748354	40937135	188782	7	1	5	0.30102999	0.00666883	0	11	0	1.20557689
chr4	40937135	41016268	79134	2	1	5	0.30102999	0.01320236	0	10	0	0.97390707
chr4	41016268	41087376	71109	2	1	5	0.30102999	0.02473314	0	9	0	0.76806864
chr4	41087376	41156822	69447	1	1	5	0.30102999	0.04407651	0	8	0	0.58747015
chr4	41156822	41188018	31197	2	1	5	0.30102999	0.02473314	0	9	0	0.76806864
chr4	41188018	41188077	60	1	1	5	0.30102999	0.01320236	0	10	0	0.97390707
chr4	41188077	41229854	41778	1	1	5	0.30102999	0.02473314	0	9	0	0.76806864
chr4	41229854	41299928	70075	3	1	5	0.30102999	0.04407651	0	8	0	0.58747015
chr4	41299928	41368384	68457	1	1	4	0.30102999	0.03812622	0	7	0	0.60763643
chr4	41368384	41444356	75973	1	1	3	0.30102999	0.01598258	0	7	0	0.84395715
chr4	41444356	41531279	86924	3	1	4	0.30102999	0.02074938	0	8	0	0.79906872
chr4	41531279	41574063	42785	2	1	4	0.30102999	0.01077081	0	9	0	1.01542894
chr4	41574063	41618637	44575	1	1	4	0.30102999	0.02074938	0	8	0	0.79906872
chr4	41618637	41652135	33499	1	0	4	0	0.02074938	0	8	0	0.79906872
chr4	41652135	41748674	96540	3	0	4	0	0.01077081	0	9	0	1.01542894
chr4	41748674	41779782	31109	2	1	4	0.30102999	0.01077081	0	9	0	1.01542894
chr4	41779782	41856258	76477	1	1	4	0.30102999	0.03812622	0	7	0	0.60763643
chr4	41856258	41956926	100669	1	0	4	0	0.03812622	0	7	0	0.60763643
chr4	41956926	41995541	38616	2	0	4	0	0.02074938	0	8	0	0.79906872
chr4	41995541	42028806	33266	2	0	4	0	0.01077081	0	9	0	1.01542894
chr4	42028806	42028865	60	1	1	4	0.30102999	0.01077081	0	9	0	1.01542894
chr4	42028865	42068656	39792	1	1	4	0.30102999	0.02074938	0	8	0	0.79906872
chr4	42068656	42175062	106407	3	1	3	0.30102999	0.03070643	0	6	0	0.63695542
chr4	42175062	42352811	177750	1	0	2	0	0.02162467	0	5	0	0.68214471
chr4	42352811	42414756	61946	2	0	2	0	0.02162467	1	5	0.30102999	0.68214471
chr4	42414756	42459482	44727	2	0	2	0	0.01053319	1	6	0.30102999	0.91219088
chr4	42459482	42526844	67363	3	0	3	0	0.0079614	1	8	0.30102999	1.07548421
chr4	42526844	42626588	99745	2	0	3	0	0.03070643	1	6	0.30102999	0.63695542
chr4	42626588	42626647	60	1	0	3	0	0.0079614	1	8	0.30102999	1.07548421
chr4	42626647	42716301	89655	1	0	2	0	0.00493743	1	7	0.30102999	1.16581773
chr4	42716301	42716360	60	1	0	3	0	0.01598258	1	7	0.30102999	0.84395715
chr4	42716360	42898190	181831	1	0	2	0	0.01053319	1	6	0.30102999	0.91219088
chr4	42898190	43048195	150006	3	0	2	0	0.01053319	2	6	0.61140001	0.91219088
chr4	43048195	43048254	60	1	0	3	0	0.01598258	2	7	0.61140001	0.84395715
chr4	43048254	43219963	171710	1	0	2	0	0.00493743	2	7	0.61140001	1.16581773
chr4	43219963	43389038	169076	1	0	2	0	0.00493743	1	7	0.30102999	1.16581773
chr4	43389038	43427202	38165	2	0	4	0	0.03812622	1	7	0.30102999	0.60763643
chr4	43427202	43642857	215656	2	0	3	0	0.01598258	1	7	0.30102999	0.84395715
chr4	43642857	43761244	118388	2	0	4	0	0.03812622	2	7	0.61140001	0.60763643
chr4	43761244	43761303	60	1	1	7	0.05404976	0.13499366	2	8	0.30102999	0.30102999
chr4	43761303	43851921	90619	1	1	6	0.05404976	0.08122616	2	8	0.30102999	0.4250187
chr4	43851921	43851980	60	1	1	6	0.05404976	0.04875589	2	9	0.30102999	0.5732208
chr4	43851980	44089485	237506	2	1	5	0.05404976	0.07511598	2	7	0.30102999	0.43181735
chr4	44089485	44089544	60	1	1	6	0.05404976	0.01518174	2	11	0.30102999	0.9449689
chr4	44089544	44220949	131406	2	1	6	0.05404976	0.04875589	2	9	0.30102999	0.5732208
chr4	44220949	44221008	60	1	1	7	0.05404976	0.05232577	2	10	0.30102999	0.56314362
chr4	44221008	44379893	158886	4	1	6	0.05404976	0.04875589	2	9	0.30102999	0.5732208
chr4	44379893	44379952	60	1	1	7	0.05404976	0.05232577	2	10	0.30102999	0.56314362
chr4	44379952	44419538	39587	1	1	5	0.05404976	0.02473314	2	9	0.30102999	0.76806864
chr4	44419538	44551234	131697	2	1	5	0.05404976	0.12309572	2	6	0.30102999	0.30102999
chr4	44551234	44771097	219864	8	1	5	0.05404976	0.04407651	2	8	0.30102999	0.58747015

chr4	44771097	44771156	60	1	1	5	0.05404976	0.02473314	2	9	0.30102999	0.76806864
chr4	44771156	44850644	79489	1	1	3	0.05404976	0.0079614	2	8	0.30102999	1.07548421
chr4	44850644	45106338	255695	2	1	2	0.05404976	0.00493743	2	7	0.30102999	1.16581773
chr4	45106338	45106397	60	1	1	3	0.05404976	0.0079614	2	8	0.30102999	1.07548421
chr4	45106397	45254680	148284	1	1	3	0.05404976	0.01598258	2	7	0.30102999	0.84395715
chr4	45254680	45376149	121470	2	1	4	0.05404976	0.03812622	2	7	0.30102999	0.60763643
chr4	45376149	45518972	142824	2	1	4	0.05404976	0.06713722	2	6	0.30102999	0.44141547
chr4	45518972	45755051	236080	1	1	2	0.05404976	0.02162467	2	5	0.30102999	0.68214471
chr4	45755051	45847432	92382	1	1	2	0.1218695	0.02162467	1	5	0.1218695	0.68214471
chr4	45847432	45847491	60	1	1	4	0.1218695	0.11390336	1	5	0.1218695	0.30102999
chr4	45847491	46033652	186162	1	0	4	0	0.11390336	1	5	0.30102999	0.30102999
chr4	46033652	46456211	422560	11	0	4	0	0.06713722	1	6	0.30102999	0.44141547
chr4	46456211	46580693	124483	2	0	4	0	0.06713722	2	6	0.61140001	0.44141547
chr4	46580693	46731529	150837	2	0	4	0	0.06713722	3	6	0.93173516	0.44141547
chr4	46731529	46731588	60	1	0	5	0	0.12309572	3	6	0.93173516	0.30102999
chr4	46731588	46877670	146083	3	0	4	0	0.06713722	3	6	0.93173516	0.44141547
chr4	46877670	46911136	33467	2	0	6	0	0.20064824	3	6	0.93173516	0.20064824
chr4	46911136	46966676	55541	1	0	5	0	0.12309572	3	6	0.93173516	0.30102999
chr4	46966676	46994955	28280	3	1	5	0.02438896	0.12309572	3	6	0.51676182	0.30102999
chr4	46994955	47035150	40196	1	1	4	0.02438896	0.11390336	3	5	0.51676182	0.30102999
chr4	47035150	47163305	128156	2	0	4	0	0.11390336	3	5	0.93173516	0.30102999
chr4	47163305	47175094	11790	2	0	5	0	0.19510895	4	5	1.26272838	0.19510895
chr4	52689101	52711931	22831	2	2	1	0.08289318	0.05404976	3	2	0.30102999	0.30102999
chr4	52711931	52711990	60	1	2	1	0.01053319	0.01091641	6	4	0.91219088	0.76005302
chr4	52711990	52743955	31966	1	2	1	0.01053319	0.05404976	6	2	0.91219088	0.30102999
chr4	52743955	52859933	115979	3	2	1	0.00493743	0.05404976	7	2	1.16581773	0.30102999
chr4	52859933	52893117	33185	2	2	1	0.00493743	0.01091641	7	4	1.16581773	0.76005302
chr4	52893117	52944500	51384	2	2	1	0.00493743	0.02438896	7	3	1.16581773	0.51676182
chr4	52944500	53025164	80665	4	2	1	0.00493743	0.01091641	7	4	1.16581773	0.76005302
chr4	53025164	53294292	269129	5	2	2	0.00493743	0.0429175	7	4	1.16581773	0.47744371
chr4	53294292	53294351	60	1	2	2	0.00493743	0.02162467	7	5	1.16581773	0.68214471
chr4	53294351	53416699	122349	2	2	2	0.00493743	0.0429175	7	4	1.16581773	0.47744371
chr4	53416699	53705275	288577	8	2	2	0.00493743	0.02162467	7	5	1.16581773	0.68214471
chr4	53705275	53729457	24183	2	2	2	0.00221948	0.02162467	8	5	1.44210395	0.68214471
chr4	53729457	54060909	331453	11	3	2	0.0079614	0.02162467	8	5	1.07548421	0.68214471
chr4	54060909	54134010	73102	2	2	2	0.00221948	0.02162467	8	5	1.44210395	0.68214471
chr4	54134010	54218619	84610	2	2	2	0.00221948	0.0429175	8	4	1.44210395	0.47744371
chr4	54218619	54373639	155021	5	2	2	0.00221948	0.02162467	8	5	1.44210395	0.68214471
chr4	54373639	54766075	392437	6	2	2	0.00221948	0.0429175	8	4	1.44210395	0.47744371
chr4	54766075	55048646	282572	9	2	2	0.00221948	0.08289318	8	3	1.44210395	0.30102999
chr4	55048646	55048705	60	1	2	2	0.00221948	0.0429175	8	4	1.44210395	0.47744371
chr4	55048705	55179152	130448	4	2	2	0.00221948	0.08289318	8	3	1.44210395	0.30102999
chr4	55179152	55179211	60	1	2	2	0.00221948	0.0429175	8	4	1.44210395	0.47744371
chr4	55179211	55252228	73018	1	2	2	0.00493743	0.0429175	7	4	1.16581773	0.47744371
chr4	55252228	55252287	60	1	2	2	0.00493743	0.02162467	7	5	1.16581773	0.68214471
chr4	55252287	55409467	157181	1	2	2	0.00493743	0.0429175	7	4	1.16581773	0.47744371
chr4	55409467	55532008	122542	1	2	2	0.02162467	0.0429175	5	4	0.68214471	0.47744371
chr4	55532008	55593629	61622	3	2	3	0.02162467	0.10122019	5	4	0.68214471	0.30102999
chr4	55593629	55606492	12864	2	2	3	0.02162467	0.05670724	5	5	0.68214471	0.45545077
chr4	55606492	55759075	152584	1	2	3	0.02162467	0.10122019	5	4	0.68214471	0.30102999
chr4	55759075	55759134	60	1	2	3	0.00493743	0.10122019	7	4	1.16581773	0.30102999
chr4	55759134	55879220	120087	1	2	3	0.01053319	0.10122019	6	4	0.91219088	0.30102999
chr4	55879220	55980398	101179	2	2	3	0.0429175	0.10122019	4	4	0.47744371	0.30102999
chr4	55980398	56016702	36305	2	2	3	0.01053319	0.10122019	6	4	0.91219088	0.30102999
chr4	56016702	56154217	137516	1	1	3	0.00204627	0.10122019	6	4	1.31360226	0.30102999
chr4	56154217	56218849	64633	2	1	3	0.00478973	0.17593012	5	3	1.02643191	0.17593012
chr4	56218849	56265560	46712	1	1	3	0.01091641	0.17593012	4	3	0.76005302	0.17593012
chr4	56265560	56294580	29021	2	1	3	0.00478973	0.10122019	5	4	1.02643191	0.30102999
chr4	56294580	56294639	60	1	1	3	3.39E-04	0.10122019	8	4	1.94674965	0.30102999
chr4	56294639	56369907	75269	2	1	3	8.47E-04	0.10122019	7	4	1.62048027	0.30102999
chr4	56369907	56500620	130714	7	1	4	3.39E-04	0.11390336	8	5	1.94674965	0.30102999
chr4	56500620	56629617	128998	1	1	3	0.00478973	0.05670724	5	5	1.02643191	0.45545077
chr4	56629617	56697858	68242	1	1	3	0.00478973	0.10122019	5	4	1.02643191	0.30102999
chr4	56697858	56885547	187690	6	1	3	0.00204627	0.10122019	6	4	1.31360226	0.30102999
chr4	56885547	56946386	60840	3	1	3	8.47E-04	0.10122019	7	4	1.62048027	0.30102999
chr4	56946386	56946445	60	1	1	3	8.47E-04	0.03070643	7	6	1.62048027	0.63695542
chr4	56946445	56986699	40255	1	1	3	8.47E-04	0.05670724	7	5	1.62048027	0.45545077
chr4	56986699	57082664	95966	2	2	3	0.00221948	0.05670724	8	5	1.44210395	0.45545077
chr4	57082664	57110994	28331	2	2	3	9.54E-04	0.05670724	9	5	1.74076927	0.45545077
chr4	57110994	57194063	83070	2	2	3	9.54E-04	0.10122019	9	4	1.74076927	0.30102999
chr4	57194063	57194122	60	1	3	3	0.00378107	0.10122019	9	4	1.33111237	0.30102999
chr4	57194122	57248082	53961	1	3	3	0.00378107	0.17593012	9	3	1.33111237	0.17593012
chr4	57248082	57280535	32454	1	2	3	9.54E-04	0.17593012	9	3	1.74076927	0.17593012
chr4	57280535	57309910	29376	2	2	3	9.54E-04	0.10122019	9	4	1.74076927	0.30102999
chr4	57309910	57309969	60	1	2	3	9.54E-04	0.05670724	9	5	1.74076927	0.45545077
chr4	57309969	57395445	85477	2	2	3	0.00221948	0.05670724	8	5	1.44210395	0.45545077
chr4	57395445	57395504	60	1	2	3	9.54E-04	0.05670724	9	5	1.74076927	0.45545077
chr4	57395504	57514428	118925	1	2	1	0.00221948	0.01091641	8	4	1.44210395	0.76005302
chr4	57514428	57544216	29789	2	2	1	9.54E-04	0.01091641	9	4	1.74076927	0.76005302
chr4	57544216	57640757	96542	1	2	1	0.00493743	0.01091641	7	4	1.16581773	0.76005302
chr4	57640757	57680620	39864	3	2	3	0.00493743	0.10122019	7	4	1.16581773	0.30102999
chr4	57680620	57829686	149067	4	1	1	0.00204627	0.01091641	6	4	1.31360226	0.76005302
chr4	57829686	57842295	12610	3	1	3	0.00204627	0.10122019	6	4	1.31360226	0.30102999
chr4	57842295	57916058	73764	3	1	2	0.00204627	0.0429175	6	4	1.31360226	0.47744371
chr4	57916058	57958486	42429	2	1	2	0.00204627	0.08289318	6	3	1.31360226	0.30102999
chr4	57958486	57973903	15418	2	1	2	8.47E-04	0.0429175	7	4	1.62048027	0.47744371
chr4	57973903	58183131	209229	2	1	0	8.47E-04	0	7	4	1.62048027	1.26272838
chr4	58183131	58183190	60	1	2	0	0.00493743	0	7	4	1.16581773	1.26272838
chr4	58183190	58329324	146135	1	0	0	0	0	6	4	1.95986592	1.26272838
chr4	58329324	58365361	36038	2	0	0	0	0	6	5	1.95986592	1.60515106
chr4	58365361	58365420	60	1	1	0	0.00204627	0	6	5	1.31360226	1.60515106
chr4	58365420	58469391	103972	1	1	0	0.00478973	0	5	5	1.02643191	1.60515106
chr4	58469391	58604510	135120	1	1	0	0.00478973	0	5	3	1.02643191	0.93173516
chr4	58604510	58850244	245735	1	1	0	0.01091641	0	4	1	0.76005302	0.30102999
chr4	58850244	59026086	175843	1	0	0	0	0	2	1	0.61140001	0.30102999
chr4	59026086	59258450	232365	2	0	0	0	0	2	2	0.61140001	0.61140001
chr4	59258450	59258509	60	1	0	1	0	0.05404976	2	2	0.61140001	0.30102999
chr4	59598916	59598975	60	1	0	2	0	0.08289318	2	3	0.61140001	0.30102999
chr4	59911076	60392154	481079	3	0	1	0	0.1218695	2	1	0.61140001	0.1218695
chr4	60392154	60687007	294854	4	0	2	0	0.30102999	2	1	0.61140001	0.05404976
chr4	60687007	60854315	167309	3	0	2	0	0.30102999	3	1	0.93173516	0.05404976
chr4	60854315	61343360	489046	3	0	1	0	0.1218695	3	1	0.93173516	0.1218695
chr4	61343360	61343419	60	1	0	1	0	0.05404976	3	2	0.93173516	0.30102999
chr4	61343419	61619455	276037	2	0	1	0	0.30102999	3	0	0.93173516	0
chr4	61619455	61619514	60	1	0	2	0	0.30102999	3	1	0.93173516	0.05404976
chr4	61619514	61862486	242973	1	0	2	0	0.61140001	3	0	0.93173516	0
chr4	61862486	62129501	267016	3	0	3	0	0.51676182	3	1	0.93173516	0.02438896
chr4	62129501	62362825	233325	4	0	3	0	0.51676182	4	1	1.26272838	0.02438896
chr4	62362825	62534191	171367	4	1	3	0.01091641	0.51676182	4	1	0.76005302	0.02438896
chr4	62534191	63086274	552084	10	1	3	0.00478973	0.51676182	5	1	1.02643191	0.02438896
chr4	63086274	63393653	307380	2	0	2	0	0.30102999	4	1	1.26272838	0.05404976
chr4	63393653	63393712	60	1	0	4	0	0.47744371	4	2	1.26272838	0.0429175
chr4	63393712	63636530	242819	1	0	4	0	0.76005302	3	1	0.93173516	0.01091641
chr4	63636530	63813565	177036	1	0	0	0	0	3	1	0.93173516	0.30102999
chr4	64367629	64669283	301655	2	0	0	0	0	2	1	0.61140001	0.30102999
chr4	64669283	64788828	119546	2	0	1	0	0.1218695	2	1	0.61140001	0.1218695
chr4	64788828	65876082	1087255	9	0	0	0	0	2	1	0.61140001	0.30102999
chr4	65876082	65991088	115007	2	0	1	0	0.1218695	3	1	0.93173516	0.1218695
chr4	65991088	66119071	127984	1	0	0	0	0	3	1	0.93173516	0.30102999
chr4	66119071	66230823	111753	2	0	0	0	0	2	1	0.61140001	0.30102999
chr4	66230823	66279978	49156	2	0	2	0	0.1575501	2	2	0.61140001	0.1575501
chr4	66279978	66391688	111711	3	0	2	0	0.1575501	3	2	0.93173516	0.1575501
chr4	66391688	66838599	446912	5	0	0	0	0	3	1	0.93173516	0.30102999
chr4	66838599	66966827	128229	2	0	0	0	0	3	2	0.93173516	0.61140001

chr4	66966827	66966886	60	1	0	2	0	0.1575501	3	2	0.93173516	0.1575501
chr4	66966886	67129288	162403	2	0	0	0	0	3	1	0.93173516	0.30102999
chr4	67129288	67189303	60016	3	0	0	0	0	3	2	0.93173516	0.61140001
chr4	67189303	67346550	157248	1	0	0	0	0	2	1	0.61140001	0.30102999
chr4	67346550	67346609	60	1	0	1	0	0.1218695	2	1	0.61140001	0.1218695
chr4	67346609	67743365	396757	2	0	0	0	0	2	0	0.61140001	0
chr4	67743365	67743424	60	1	0	2	0	0.08289318	3	3	0.93173516	0.30102999
chr4	67743424	67958904	215481	1	0	2	0	0.1575501	3	2	0.93173516	0.1575501
chr4	67958904	68193194	234291	1	0	2	0	0.30102999	3	1	0.93173516	0.05404976
chr4	68193194	68193253	60	1	0	2	0	0.1575501	4	2	1.26272838	0.1575501
chr4	68193253	68383183	189931	3	0	2	0	0.1575501	3	2	0.93173516	0.1575501
chr4	68383183	68383242	60	1	0	2	0	0.1575501	4	2	1.26272838	0.1575501
chr4	68383242	68912989	529748	14	0	2	0	0.1575501	3	2	0.93173516	0.1575501
chr4	68912989	68946958	33970	2	0	3	0	0.17593012	3	3	0.93173516	0.17593012
chr4	68946958	69092624	145667	4	0	3	0	0.30102999	3	2	0.93173516	0.08289318
chr4	69092624	69198495	105872	4	0	4	0	0.47744371	3	2	0.93173516	0.0429175
chr4	69198495	69213780	15286	2	0	4	0	0.47744371	4	2	1.26272838	0.0429175
chr4	69213780	69402791	189012	1	0	2	0	0.1575501	3	2	0.93173516	0.1575501
chr4	69402791	69402845	55	1	0	2	0	0.1575501	4	2	1.26272838	0.1575501
chr4	69402845	69718248	315404	1	0	2	0	0.61140001	2	0	0.61140001	0
chr4	69718248	69809232	90985	2	1	3	0.02438896	0.93173516	3	0	0.51676182	0
chr4	69809232	69809291	60	1	1	3	0.00478973	0.93173516	5	0	1.02643191	0
chr4	69809291	69897369	88079	1	1	2	0.00478973	0.61140001	5	0	1.02643191	0
chr4	69897369	69961874	64506	1	1	2	0.01091641	0.61140001	4	0	0.76005302	0
chr4	69961874	70014042	52169	2	1	2	0.01091641	0.30102999	4	1	0.76005302	0.05404976
chr4	70014042	70014101	60	1	1	4	0.01091641	0.76005302	4	1	0.76005302	0.01091641
chr4	70014101	70182118	168018	2	1	2	0.01091641	0.30102999	4	1	0.76005302	0.05404976
chr4	70182118	70182177	60	1	1	3	0.01091641	0.17593012	4	3	0.76005302	0.17593012
chr4	70182177	70330499	148323	1	1	3	0.01091641	0.30102999	4	2	0.76005302	0.08289318
chr4	70330499	70330558	60	1	2	4	0.02162467	0.47744371	5	2	0.68214471	0.0429175
chr4	70330558	70355326	24769	1	2	3	0.02162467	0.30102999	5	2	0.68214471	0.08289318
chr4	70355326	70356682	1357	2	2	3	0.01053319	0.30102999	6	2	0.91219088	0.08289318
chr4	70356682	70405132	48451	1	1	3	0.00204627	0.30102999	6	2	1.31360226	0.08289318
chr4	70405132	70456773	51642	2	1	4	0.00204627	0.30102999	6	3	1.31360226	0.10122019
chr4	70456773	70596245	139473	2	1	4	0.01091641	0.30102999	4	3	0.76005302	0.10122019
chr4	70596245	70667736	71492	2	1	4	8.47E-04	0.30102999	7	3	1.62048027	0.10122019
chr4	70667736	70667795	60	1	1	6	8.47E-04	0.63695542	7	3	1.62048027	0.03070643
chr4	70667795	70797273	129479	2	1	6	0.00204627	0.63695542	6	3	1.31360226	0.03070643
chr4	70797273	71070208	272936	9	1	5	0.00204627	0.45545077	6	3	1.31360226	0.05670724
chr4	71070208	71230260	160053	6	1	6	0.00204627	0.63695542	6	3	1.31360226	0.03070643
chr4	71230260	71251451	21192	1	1	5	0.00204627	0.45545077	6	3	1.31360226	0.05670724
chr4	71251451	71251510	60	1	1	5	0.00204627	0.30102999	6	4	1.31360226	0.11390336
chr4	71251510	71275265	23756	1	1	5	0.00204627	0.45545077	6	3	1.31360226	0.05670724
chr4	71275265	71379309	104045	4	1	6	0.00204627	0.63695542	6	3	1.31360226	0.03070643
chr4	71379309	71379368	60	1	1	6	0.00204627	0.44141547	6	4	1.31360226	0.06713722
chr4	71379368	71472584	93217	2	1	6	0.00204627	0.63695542	6	3	1.31360226	0.03070643
chr4	71472584	71472643	60	1	1	6	0.00204627	0.44141547	6	4	1.31360226	0.06713722
chr4	71472643	71555968	83326	5	1	6	0.00204627	0.63695542	6	3	1.31360226	0.03070643
chr4	71555968	71628262	72295	3	1	6	0.00204627	0.44141547	6	4	1.31360226	0.06713722
chr4	71628262	71701939	73678	2	1	6	0.00204627	0.63695542	6	3	1.31360226	0.03070643
chr4	71701939	71725250	23312	2	1	7	0.00204627	0.84395715	6	3	1.31360226	0.01598258
chr4	71725250	71796651	71402	2	1	8	0.00204627	0.58747015	6	5	1.31360226	0.04407651
chr4	71796651	71824777	28127	1	1	7	0.00204627	0.60763643	6	4	1.31360226	0.03812622
chr4	71824777	71875751	50975	1	1	6	0.00204627	0.44141547	6	4	1.31360226	0.06713722
chr4	71875751	71892429	16679	3	1	6	0.00204627	0.30102999	6	5	1.31360226	0.12309572
chr4	71892429	72037455	145027	2	1	6	0.00204627	0.44141547	6	4	1.31360226	0.06713722
chr4	72037455	72074858	37404	2	1	7	0.00204627	0.43181735	6	5	1.31360226	0.07511598
chr4	72074858	72074917	60	1	1	7	0.00204627	0.30102999	6	6	1.31360226	0.129913
chr4	72074917	72150396	75480	2	1	6	0.00204627	0.63695542	6	3	1.31360226	0.03070643
chr4	72150396	72150455	60	1	1	6	0.00204627	0.44141547	6	4	1.31360226	0.06713722
chr4	72150455	72184996	34542	1	1	6	0.00204627	0.63695542	6	3	1.31360226	0.03070643
chr4	72184996	72263311	78316	2	1	6	0.00478973	0.63695542	5	3	1.02643191	0.03070643
chr4	72263311	72263364	54	1	1	7	0.00478973	0.84395715	5	3	1.02643191	0.01598258
chr4	72263364	72388732	125369	3	1	6	0.00478973	0.63695542	5	3	1.02643191	0.03070643
chr4	72388732	72694632	305901	6	1	6	0.00204627	0.63695542	6	3	1.31360226	0.03070643
chr4	72694632	72852167	157536	1	1	6	0.00478973	0.63695542	5	3	1.02643191	0.03070643
chr4	72852167	73013520	161354	4	1	5	0.00478973	0.45545077	5	3	1.02643191	0.05670724
chr4	73013520	73075562	62043	1	1	5	0.01091641	0.45545077	4	3	0.76005302	0.05670724
chr4	73075562	73227532	151971	3	1	5	0.01091641	0.68214471	4	2	0.76005302	0.02162467
chr4	73227532	73330508	102977	2	1	4	0.02438896	0.47744371	3	2	0.51676182	0.0429175
chr4	73330508	73373561	43054	2	1	4	0.02438896	0.30102999	3	3	0.51676182	0.10122019
chr4	73373561	73495623	122063	2	1	4	0.02438896	0.47744371	3	2	0.51676182	0.0429175
chr4	73495623	73930473	434851	5	1	4	0.02438896	0.30102999	3	3	0.51676182	0.10122019
chr4	73930473	73930532	60	1	2	4	0.08289318	0.30102999	3	3	0.30102999	0.10122019
chr4	73930532	73994060	63529	2	1	4	0.02438896	0.30102999	3	3	0.51676182	0.10122019
chr4	73994060	74027044	32985	2	1	4	0.02438896	0.18734596	3	4	0.51676182	0.18734596
chr4	74027044	74076511	49468	2	1	5	0.02438896	0.30102999	3	4	0.51676182	0.11390336
chr4	74076511	74115854	39344	1	1	5	0.02438896	0.45545077	3	3	0.51676182	0.05670724
chr4	74115854	74279312	163459	3	1	5	0.01091641	0.45545077	4	3	0.76005302	0.05670724
chr4	74279312	74391127	111816	5	1	5	0.00478973	0.45545077	5	3	1.02643191	0.05670724
chr4	74391127	74391186	60	1	1	6	0.00478973	0.63695542	5	3	1.02643191	0.03070643
chr4	74391186	74484902	93717	2	1	6	0.01091641	0.63695542	4	3	0.76005302	0.03070643
chr4	74484902	74703204	218303	4	1	6	0.02438896	0.63695542	3	3	0.51676182	0.03070643
chr4	74703204	74703789	586	2	1	7	0.02438896	0.84395715	3	3	0.51676182	0.01598258
chr4	74703789	74769169	65381	2	1	6	0.02438896	0.63695542	3	3	0.51676182	0.03070643
chr4	74769169	74769228	60	1	2	6	0.0429175	0.63695542	4	3	0.47744371	0.03070643
chr4	74769228	74852953	83726	2	1	6	0.01091641	0.63695542	4	3	0.76005302	0.03070643
chr4	74852953	74862821	9869	1	1	4	0.01091641	0.30102999	4	3	0.76005302	0.10122019
chr4	74862821	74963200	100380	6	2	4	0.0429175	0.30102999	4	3	0.47744371	0.10122019
chr4	74963200	74963259	60	1	2	6	0.0429175	0.63695542	4	3	0.47744371	0.03070643
chr4	74963259	75239841	276583	6	1	6	0.01091641	0.63695542	4	3	0.76005302	0.03070643
chr4	75239841	75246872	7032	2	1	7	0.01091641	0.84395715	4	3	0.76005302	0.01598258
chr4	75246872	75631511	384640	2	1	6	0.01091641	0.63695542	4	3	0.76005302	0.03070643
chr4	75631511	75718360	86850	3	1	6	0.00204627	0.63695542	6	3	1.31360226	0.03070643
chr4	75718360	75718419	60	1	1	7	0.00204627	0.84395715	6	3	1.31360226	0.01598258
chr4	75718419	75971503	253085	5	1	4	0.00204627	0.30102999	6	3	1.31360226	0.10122019
chr4	75971503	76264904	293402	2	1	3	0.00204627	0.17593012	6	3	1.31360226	0.17593012
chr4	76264904	76417312	152409	3	1	4	0.00204627	0.18734596	6	4	1.31360226	0.18734596
chr4	76417312	76453612	36301	3	1	4	8.47E-04	0.18734596	7	4	1.62048027	0.18734596
chr4	76453612	76521417	67806	2	1	3	8.47E-04	0.10122019	7	4	1.62048027	0.30102999
chr4	76521417	76715073	193657	8	2	3	0.00493743	0.10122019	7	4	1.16581773	0.30102999
chr4	76715073	77053787	338715	14	1	3	8.47E-04	0.10122019	7	4	1.62048027	0.30102999
chr4	77053787	77164727	110941	3	1	3	8.47E-04	0.17593012	7	3	1.62048027	0.17593012
chr4	77164727	77506547	341821	11	1	3	8.47E-04	0.10122019	7	4	1.62048027	0.30102999
chr4	77506547	77556611	50065	1	1	3	0.00204627	0.10122019	6	4	1.31360226	0.30102999
chr4	77556611	77639859	83249	2	1	3	0.00478973	0.10122019	5	4	1.02643191	0.30102999
chr4	77639859	77747719	107861	4	1	3	0.00204627	0.05670724	6	5	1.31360226	0.45545077
chr4	77747719	77873619	125901	2	1	3	0.00204627	0.10122019	6	4	1.31360226	0.30102999
chr4	77873619	77902158	28540	2	1	3	0.00204627	0.05670724	6	5	1.31360226	0.45545077
chr4	77902158	77977442	75285	2	1	3	0.00204627	0.10122019	6	4	1.31360226	0.30102999
chr4	77977442	77977501	60	1	1	3	0.00204627	0.05670724	6	5	1.31360226	0.45545077
chr4	77977501	78080175	102675	2	1	3	0.00478973	0.10122019	5	4	1.02643191	0.30102999
chr4	78080175	78085456	5282	3	1	3	0.00204627	0.10122019	6	4	1.31360226	0.30102999
chr4	78085456	78162792	77337	1	1	3	0.00478973	0.10122019	5	4	1.02643191	0.30102999
chr4	78162792	78162851	60	1	1	3	0.00478973	0.05670724	5	5	1.02643191	0.45545077
chr4	78162851	78215223	52373	1	1	3	0.00478973	0.10122019	5	4	1.02643191	0.30102999
chr4	78215223	78215282	60	1	1	3	0.00204627	0.10122019	6	4	1.31360226	0.30102999
chr4	78215282	78272446	57165	1	1	3	0.00478973	0.10122019	5	4	1.02643191	0.30102999
chr4	78272446	78272505	60	1	1	4	0.00478973	0.11390336	5	5	1.02643191	0.30102999
chr4	78272505	78432964	160460	3	1	3	0.00478973	0.05670724	5	5	1.02643191	0.45545077
chr4	78432964	78529027	96064	2	1	3	0.00478973	0.10122019	5	4	1.02643191	0.30102999
chr4	78529027	78601448	72422	2	1	4	0.00478973	0.11390336	5	5	1.02643191	0.30102999
chr4	78601448	78601507	60	1	1	5	0.00478973	0.19510895	5	5	1.02643191	0.19510895
chr4	78601507	78647247	45741	2	1	4	0.00478973	0.11390336	5	5	1.02643191	0.30102999

chr4	78647247	78804617	157371	4	1	5	0.00478973	0.19510895	5	5	1.02643191	0.19510895
chr4	78804617	79076040	271424	7	1	6	0.00204627	0.30102999	6	5	1.31360226	0.12309572
chr4	79076040	79307240	231201	5	1	6	0.00478973	0.44141547	5	4	1.02643191	0.06713722
chr4	79307240	79408088	100849	2	1	6	0.00478973	0.63695542	5	3	1.02643191	0.03070643
chr4	79408088	79408147	60	1	1	6	0.00478973	0.30102999	5	5	1.02643191	0.12309572
chr4	79408147	79701251	293105	6	1	6	0.00478973	0.63695542	5	3	1.02643191	0.03070643
chr4	79701251	79741037	39787	2	1	6	0.00204627	0.63695542	6	3	1.31360226	0.03070643
chr4	79741037	79869478	128442	9	1	6	0.00204627	0.44141547	6	4	1.31360226	0.06713722
chr4	79869478	79972194	102717	2	1	5	0.00204627	0.30102999	6	4	1.31360226	0.11390336
chr4	79972194	80020176	47983	2	1	6	0.00204627	0.44141547	6	4	1.31360226	0.06713722
chr4	80020176	80020235	60	1	1	6	0.00204627	0.30102999	6	5	1.31360226	0.12309572
chr4	80020235	80103546	83312	1	1	6	0.00204627	0.44141547	6	4	1.31360226	0.06713722
chr4	80103546	80103605	60	1	1	6	0.00204627	0.30102999	6	5	1.31360226	0.12309572
chr4	80103605	80188542	84938	1	1	6	0.00478973	0.44141547	5	4	1.02643191	0.06713722
chr4	80188542	80188601	60	1	1	6	0.00478973	0.30102999	5	5	1.02643191	0.12309572
chr4	80188601	80243920	55320	2	1	6	0.01091641	0.30102999	4	5	0.76005302	0.12309572
chr4	80243920	80327877	83958	1	1	5	0.01091641	0.19510895	4	5	0.76005302	0.19510895
chr4	80327877	80394761	66885	2	1	6	0.01091641	0.30102999	4	5	0.76005302	0.12309572
chr4	80394761	80394820	60	1	1	8	0.01091641	0.58747015	4	5	0.76005302	0.04407651
chr4	80394820	80490393	95574	1	1	7	0.01091641	0.43181735	4	5	0.76005302	0.07511598
chr4	80490393	80490452	60	1	1	8	0.01091641	0.58747015	4	5	0.76005302	0.04407651
chr4	80490452	80597312	106861	1	1	7	0.01091641	0.43181735	4	5	0.76005302	0.07511598
chr4	80597312	80597371	60	1	1	8	0.00478973	0.58747015	5	5	1.02643191	0.04407651
chr4	80597371	80707668	110298	1	1	7	0.02438896	0.43181735	3	5	0.51676182	0.07511598
chr4	80707668	80707727	60	1	1	8	0.02438896	0.58747015	3	5	0.51676182	0.04407651
chr4	80707727	80872939	165213	4	1	7	0.02438896	0.43181735	3	5	0.51676182	0.07511598
chr4	80872939	81289268	416330	10	1	8	0.02438896	0.58747015	3	5	0.51676182	0.04407651
chr4	81289268	81408980	119713	2	1	7	0.02438896	0.43181735	3	5	0.51676182	0.07511598
chr4	81408980	81447997	39018	2	1	9	0.02438896	0.76806864	3	5	0.51676182	0.02473314
chr4	81447997	81504230	56234	1	1	8	0.02438896	0.58747015	3	5	0.51676182	0.04407651
chr4	81504230	81702044	197815	6	1	9	0.02438896	0.5732208	3	6	0.51676182	0.04875589
chr4	81702044	81702103	60	1	1	10	0.02438896	0.74627054	3	6	0.51676182	0.02793176
chr4	81702103	81798577	96475	2	1	8	0.02438896	0.4250187	3	6	0.51676182	0.08122616
chr4	81798577	81884651	86075	2	1	6	0.02438896	0.30102999	3	5	0.51676182	0.12309572
chr4	81884651	81898468	13818	2	1	7	0.02438896	0.43181735	3	5	0.51676182	0.07511598
chr4	81898468	81970919	72452	1	1	4	0.02438896	0.11390336	3	5	0.51676182	0.30102999
chr4	81970919	81970978	60	1	1	4	0.02438896	0.06713722	3	6	0.51676182	0.44141547
chr4	81970978	82158097	187120	4	1	4	0.05404976	0.06713722	2	6	0.30102999	0.44141547
chr4	82158097	82251719	93623	1	1	4	0.05404976	0.18734596	2	4	0.30102999	0.18734596
chr4	82251719	82312971	61253	1	1	3	0.05404976	0.17593012	2	3	0.30102999	0.17593012
chr4	82312971	82373903	60933	2	1	3	0.05404976	0.03070643	2	6	0.30102999	0.63695542
chr4	82373903	82378778	4876	2	1	3	0.05404976	0.01598258	2	7	0.30102999	0.84395715
chr4	82378778	82472611	93834	2	1	3	0.05404976	0.03070643	2	6	0.30102999	0.63695542
chr4	82472611	82583953	111343	1	1	3	0.05404976	0.05670724	2	5	0.30102999	0.45545077
chr4	82583953	82584012	60	1	1	3	0.05404976	0.03070643	2	6	0.30102999	0.63695542
chr4	82584012	82777709	193698	2	1	2	0.05404976	0.0429175	2	4	0.30102999	0.47744371
chr4	82777709	82981798	204090	2	1	2	0.05404976	0.02162467	2	5	0.30102999	0.68214471
chr4	82981798	83072285	90488	2	1	6	0.05404976	0.129913	2	7	0.30102999	0.30102999
chr4	83072285	83155020	82736	1	1	5	0.05404976	0.12309572	2	6	0.30102999	0.30102999
chr4	83155020	83155079	60	1	1	5	0.05404976	0.07511598	2	7	0.30102999	0.43181735
chr4	83155079	83381760	226682	3	1	5	0.05404976	0.19510895	2	5	0.30102999	0.19510895
chr4	83381760	83382059	300	2	1	5	0.05404976	0.12309572	2	6	0.30102999	0.30102999
chr4	83382059	83429357	47299	1	1	4	0.05404976	0.18734596	2	4	0.30102999	0.18734596
chr4	83429357	83486706	57350	1	1	4	0.05404976	0.30102999	2	3	0.30102999	0.10122019
chr4	83486706	83522978	36273	1	1	4	0.05404976	0.47744371	2	2	0.30102999	0.0429175
chr4	83522978	83555656	32679	2	1	4	0.02438896	0.30102999	3	3	0.51676182	0.10122019
chr4	83555656	83740092	184437	7	1	5	0.02438896	0.30102999	3	4	0.51676182	0.11390336
chr4	83740092	83785496	45405	1	1	4	0.02438896	0.30102999	3	3	0.51676182	0.10122019
chr4	83785496	83900040	114545	4	1	5	0.02438896	0.30102999	3	4	0.51676182	0.11390336
chr4	83900040	84036024	135985	6	1	5	0.02438896	0.19510895	3	5	0.51676182	0.19510895
chr4	84036024	84315063	279040	4	1	5	0.05404976	0.19510895	2	5	0.30102999	0.19510895
chr4	84315063	84374297	59235	3	1	5	0.02438896	0.19510895	3	5	0.51676182	0.19510895
chr4	84374297	84374356	60	1	1	5	0.02438896	0.12309572	3	6	0.51676182	0.30102999
chr4	84374356	84380357	6002	2	1	5	0.02438896	0.19510895	3	5	0.51676182	0.19510895
chr4	84380357	84380416	60	1	1	5	0.02438896	0.12309572	3	6	0.51676182	0.30102999
chr4	84380416	84400066	19651	2	1	5	0.02438896	0.30102999	3	4	0.51676182	0.11390336
chr4	84400066	84489988	89923	2	1	5	0.02438896	0.45545077	3	3	0.51676182	0.05670724
chr4	84489988	84584470	94483	4	1	5	0.02438896	0.30102999	3	4	0.51676182	0.11390336
chr4	84584470	84766562	182093	1	1	4	0.02438896	0.18734596	3	4	0.51676182	0.18734596
chr4	84766562	84891504	124943	3	1	4	0.01091641	0.18734596	4	4	0.76005302	0.18734596
chr4	84891504	84891563	60	1	1	4	0.01091641	0.11390336	4	5	0.76005302	0.30102999
chr4	84891563	84965324	73762	1	1	4	0.01091641	0.30102999	4	3	0.76005302	0.10122019
chr4	84965324	85109176	143853	2	1	3	0.01091641	0.30102999	4	2	0.76005302	0.08289318
chr4	85109176	85109235	60	1	1	3	0.01091641	0.17593012	4	3	0.76005302	0.17593012
chr4	85109235	85206674	97440	1	1	2	0.01091641	0.1575501	4	2	0.76005302	0.1575501
chr4	85206674	85206733	60	1	1	2	0.01091641	0.08289318	4	3	0.76005302	0.30102999
chr4	85206733	85338191	131459	1	1	2	0.05404976	0.1575501	2	2	0.30102999	0.1575501
chr4	85338191	85417374	79184	1	1	1	0.05404976	0.1218695	2	1	0.30102999	0.1218695
chr4	85417374	85417433	60	1	1	2	0.05404976	0.08289318	2	3	0.30102999	0.30102999
chr4	85417433	85510609	93177	1	1	1	0.1218695	0.02438896	1	3	0.1218695	0.51676182
chr4	85510609	85540594	29986	1	1	1	0.1218695	0.05404976	1	2	0.1218695	0.30102999
chr4	85540594	85590841	50248	3	1	1	0.1218695	0.02438896	1	3	0.1218695	0.51676182
chr4	85590841	85590900	60	1	1	1	0.1218695	0.01091641	1	4	0.1218695	0.76005302
chr4	85590900	85672613	81714	2	1	0	0.1218695	0	1	2	0.1218695	0.61140001
chr4	85672613	85672672	60	1	1	1	0.1218695	0.05404976	1	2	0.1218695	0.30102999
chr4	85672672	85729284	56613	1	1	0	0.1218695	0	1	2	0.1218695	0.61140001
chr4	85729284	85759812	30529	2	1	0	0.1218695	0	1	3	0.1218695	0.93173516
chr4	85759812	85759871	60	1	1	2	0.05404976	0.08289318	2	3	0.30102999	0.30102999
chr4	85759871	85853466	93596	2	1	1	0.1218695	0.02438896	1	3	0.1218695	0.51676182
chr4	85853466	86171521	318056	5	1	1	0.05404976	0.02438896	2	3	0.30102999	0.51676182
chr4	86171521	86261894	90374	2	1	2	0.05404976	0.08289318	2	3	0.30102999	0.30102999
chr4	86261894	86438402	176509	4	1	3	0.05404976	0.17593012	2	3	0.30102999	0.17593012
chr4	86438402	86491895	53494	2	1	4	0.05404976	0.18734596	2	4	0.30102999	0.18734596
chr4	86491895	86753901	262007	6	1	2	0.05404976	0.08289318	2	3	0.30102999	0.30102999
chr4	86753901	86790618	36718	2	1	2	0.05404976	0.0429175	2	4	0.30102999	0.47744371
chr4	86790618	86851864	61247	3	1	3	0.05404976	0.10122019	2	4	0.30102999	0.30102999
chr4	86851864	86937755	85892	3	1	3	0.02438896	0.10122019	3	4	0.51676182	0.30102999
chr4	86937755	87028610	90856	2	1	2	0.02438896	0.08289318	3	3	0.51676182	0.30102999
chr4	87028610	87028669	60	1	1	2	0.01091641	0.0429175	4	4	0.76005302	0.47744371
chr4	87028669	87197339	168671	3	1	2	0.01091641	0.08289318	4	3	0.76005302	0.30102999
chr4	87197339	87197398	60	1	1	2	0.00478973	0.08289318	5	3	1.02643191	0.30102999
chr4	87197398	87277410	80013	1	1	2	0.00478973	0.1575501	5	2	1.02643191	0.1575501
chr4	87277410	87277469	60	1	1	3	0.00478973	0.30102999	5	2	1.02643191	0.08289318
chr4	87277469	87319118	41650	1	1	2	0.00478973	0.1575501	5	2	1.02643191	0.1575501
chr4	87319118	87319177	60	1	1	2	0.00478973	0.08289318	5	3	1.02643191	0.30102999
chr4	87319177	87370663	51487	1	1	2	0.00478973	0.30102999	5	1	1.02643191	0.05404976
chr4	87370663	87514492	143830	3	1	3	0.00478973	0.51676182	5	1	1.02643191	0.02438896
chr4	87514492	87622446	107955	3	1	3	0.00478973	0.30102999	5	2	1.02643191	0.08289318
chr4	87622446	87684240	61795	2	1	4	0.00478973	0.30102999	5	3	1.02643191	0.10122019
chr4	87684240	87769729	85490	2	1	3	0.00478973	0.30102999	5	2	1.02643191	0.08289318
chr4	87769729	87808967	39239	2	1	3	0.00478973	0.51676182	5	1	1.02643191	0.02438896
chr4	87808967	87850983	42017	2	1	3	0.00478973	0.30102999	5	2	1.02643191	0.08289318
chr4	87850983	87851042	60	1	1	3	0.00478973	0.17593012	5	3	1.02643191	0.17593012
chr4	87851042	87920856	69815	1	1	3	0.00478973	0.30102999	5	2	1.02643191	0.08289318
chr4	87920856	88349207	428352	12	1	3	0.01091641	0.51676182	4	1	0.76005302	0.02438896
chr4	88349207	88436596	87390	3	1	3	0.01091641	0.30102999	4	2	0.76005302	0.08289318
chr4	88436596	88488792	52197	2	1	4	0.01091641	0.30102999	4	3	0.76005302	0.10122019
chr4	88488792	88488851	60	1	1	4	0.01091641	0.18734596	4	4	0.76005302	0.18734596
chr4	88488851	88578170	89320	3	1	3	0.01091641	0.17593012	4	3	0.76005302	0.17593012
chr4	88578170	88584658	6489	2	1	3	0.00478973	0.10122019	5	4	1.02643191	0.30102999
chr4	88584658	88723816	139159	2	1	3	0.00478973	0.17593012	5	3	1.02643191	0.17593012
chr4	88723816	88805863	82048	3	1	4	0.00478973	0.18734596	5	4	1.02643191	0.18734596
chr4	88805863	88900063	94201	2	1	3	0.00478973	0.10122019	5	4	1.02643191	0.30102999
chr4	88900063	88900122	60	1	1	4	0.00478973	0.18734596	5	4	1.02643191	0.18734596

chr4	88900122	88949541	49420	2	1	4	0.01091641	0.18734596	4	4	0.76005302	0.18734596
chr4	88949541	89052197	102657	4	1	5	0.00478973	0.19510895	5	5	1.02643191	0.19510895
chr4	89052197	89052251	55	1	1	6	0.00478973	0.30102999	5	5	1.02643191	0.12309572
chr4	89052251	89183609	131359	2	1	5	0.02438896	0.19510895	3	5	0.51676182	0.19510895
chr4	89183609	89183668	60	1	1	6	0.01091641	0.30102999	4	5	0.76005302	0.12309572
chr4	89183668	89198376	14709	2	1	6	0.02438896	0.44141547	3	4	0.51676182	0.06713722
chr4	89198376	89577303	378928	8	1	5	0.02438896	0.30102999	3	4	0.51676182	0.11390336
chr4	89577303	89648163	70861	2	1	5	0.05404976	0.30102999	2	4	0.30102999	0.11390336
chr4	89648163	89648222	60	1	1	6	0.05404976	0.44141547	2	4	0.30102999	0.06713722
chr4	89648222	89791253	143032	3	1	5	0.05404976	0.30102999	2	4	0.30102999	0.11390336
chr4	89791253	89856353	65101	1	1	5	0.05404976	0.45545077	2	3	0.30102999	0.05670724
chr4	89856353	89856412	60	1	1	6	0.05404976	0.63695542	2	3	0.30102999	0.03070643
chr4	89856412	89951120	94709	2	1	5	0.05404976	0.68214471	2	2	0.30102999	0.02162467
chr4	89951120	89951179	60	1	1	5	0.05404976	0.30102999	2	4	0.30102999	0.11390336
chr4	89951179	90462350	511172	9	1	5	0.05404976	0.68214471	2	2	0.30102999	0.02162467
chr4	90462350	90638181	175832	2	1	6	0.05404976	0.63695542	2	3	0.30102999	0.03070643
chr4	90638181	90647688	9508	2	2	8	0.1575501	1.07548421	2	3	0.1575501	0.0079614
chr4	90647688	90684820	37133	1	1	7	0.05404976	1.16581773	2	2	0.30102999	0.00493743
chr4	90684820	90722644	37825	1	1	6	0.05404976	0.91219088	2	2	0.30102999	0.01053319
chr4	90722644	90797095	74452	2	1	5	0.05404976	0.68214471	2	2	0.30102999	0.02162467
chr4	90797095	90833151	36057	2	2	5	0.08289318	0.68214471	3	2	0.30102999	0.02162467
chr4	90833151	90902061	68911	2	1	5	0.02438896	0.68214471	3	2	0.51676182	0.02162467
chr4	90902061	91047347	145287	1	1	5	0.05404976	0.68214471	2	2	0.30102999	0.02162467
chr4	91047347	91147550	100204	2	2	5	0.1575501	0.68214471	2	2	0.1575501	0.02162467
chr4	91147550	91213084	65535	2	1	2	0.05404976	0.1575501	2	2	0.30102999	0.1575501
chr4	91213084	91213143	60	1	1	3	0.05404976	0.30102999	2	2	0.30102999	0.08289318
chr4	91213143	91292877	79735	2	1	2	0.05404976	0.1575501	2	2	0.30102999	0.1575501
chr4	91292877	91292936	60	1	2	4	0.1575501	0.30102999	2	3	0.1575501	0.10122019
chr4	91292936	91399112	106177	3	1	2	0.05404976	0.1575501	2	2	0.30102999	0.1575501
chr4	91399112	91464466	65355	3	1	2	0.02438896	0.1575501	3	2	0.51676182	0.1575501
chr4	91464466	91464525	60	1	1	2	0.01091641	0.1575501	4	2	0.76005302	0.1575501
chr4	91464525	91603233	138709	3	1	2	0.02438896	0.1575501	3	2	0.51676182	0.1575501
chr4	91603233	91603292	60	1	1	3	0.01091641	0.30102999	4	2	0.76005302	0.08289318
chr4	91603292	91651077	47786	1	1	2	0.02438896	0.1575501	3	2	0.51676182	0.1575501
chr4	91651077	91699333	48257	2	1	3	0.02438896	0.30102999	3	2	0.51676182	0.08289318
chr4	91699333	91760887	61555	3	1	2	0.02438896	0.1575501	3	2	0.51676182	0.1575501
chr4	91760887	92037915	277029	4	1	1	0.05404976	0.1218695	2	1	0.30102999	0.1218695
chr4	92037915	92117968	80054	2	1	2	0.05404976	0.30102999	2	1	0.30102999	0.05404976
chr4	92117968	92118027	60	1	1	4	0.05404976	0.47744371	2	2	0.30102999	0.0429175
chr4	92118027	92202997	84971	1	1	3	0.05404976	0.51676182	2	1	0.30102999	0.02438896
chr4	92202997	92331091	128095	3	1	3	0.02438896	0.51676182	3	1	0.51676182	0.02438896
chr4	92331091	92331150	60	1	1	4	0.02438896	0.76005302	3	1	0.51676182	0.01091641
chr4	92331150	92593100	261951	3	1	3	0.02438896	0.51676182	3	1	0.51676182	0.02438896
chr4	92593100	92854003	260904	1	1	2	0.05404976	0.61140001	2	0	0.30102999	0
chr4	92854003	92854062	60	1	1	2	0.05404976	0.30102999	2	1	0.30102999	0.05404976
chr4	92854062	93028413	174352	1	1	2	0.1218695	0.30102999	1	1	0.1218695	0.05404976
chr4	93028413	93090575	62163	2	1	3	0.1218695	0.30102999	1	2	0.1218695	0.08289318
chr4	93090575	93202223	111649	1	1	2	0.1218695	0.1575501	1	2	0.1218695	0.1575501
chr4	93202223	93226245	24023	2	1	3	0.05404976	0.17593012	2	3	0.30102999	0.17593012
chr4	93226245	93306651	80407	2	1	3	0.1218695	0.30102999	1	2	0.1218695	0.08289318
chr4	93306651	93359924	53274	2	1	3	0.05404976	0.17593012	2	3	0.30102999	0.17593012
chr4	93359924	93434401	74478	2	1	4	0.05404976	0.30102999	2	3	0.30102999	0.10122019
chr4	93434401	93554942	120542	2	1	4	0.1218695	0.30102999	1	3	0.1218695	0.10122019
chr4	93554942	93712943	158002	5	1	5	0.05404976	0.30102999	2	4	0.30102999	0.11390336
chr4	93712943	93753109	40167	2	1	6	0.05404976	0.44141547	2	4	0.30102999	0.06713722
chr4	93753109	93834596	81488	1	1	6	0.05404976	0.63695542	2	3	0.30102999	0.03070643
chr4	93834596	93867292	32697	1	1	5	0.05404976	0.45545077	2	3	0.30102999	0.05670724
chr4	93867292	94032035	164744	4	1	5	0.05404976	0.30102999	2	4	0.30102999	0.11390336
chr4	94032035	94139865	107831	2	1	4	0.05404976	0.18734596	2	4	0.30102999	0.18734596
chr4	94139865	94213879	74015	1	1	4	0.05404976	0.30102999	2	3	0.30102999	0.10122019
chr4	94213879	94213938	60	1	1	5	0.02438896	0.45545077	3	3	0.51676182	0.05670724
chr4	94213938	94326388	112451	2	1	4	0.02438896	0.47744371	3	2	0.51676182	0.0429175
chr4	94326388	94326447	60	1	1	5	0.02438896	0.45545077	3	3	0.51676182	0.05670724
chr4	94326447	94362727	36281	1	1	4	0.02438896	0.30102999	3	3	0.51676182	0.10122019
chr4	94362727	94462921	100195	3	1	5	0.02438896	0.45545077	3	3	0.51676182	0.05670724
chr4	94462921	94575566	112646	3	1	3	0.02438896	0.17593012	3	3	0.51676182	0.17593012
chr4	94575566	94575625	60	1	1	5	0.02438896	0.45545077	3	3	0.51676182	0.05670724
chr4	94575625	94693423	117799	2	1	4	0.02438896	0.30102999	3	3	0.51676182	0.10122019
chr4	94693423	94859936	166514	2	1	3	0.02438896	0.17593012	3	3	0.51676182	0.17593012
chr4	94859936	95099249	239314	2	1	3	0.05404976	0.17593012	2	3	0.30102999	0.17593012
chr4	95099249	95147332	48084	2	1	4	0.02438896	0.30102999	3	3	0.51676182	0.10122019
chr4	95147332	95210716	63385	3	1	4	0.02438896	0.11390336	3	5	0.51676182	0.30102999
chr4	95210716	95269953	59238	2	1	4	0.05404976	0.18734596	2	4	0.30102999	0.18734596
chr4	95269953	95406121	136169	2	1	3	0.05404976	0.10122019	2	4	0.30102999	0.30102999
chr4	95406121	95496808	90688	3	1	4	0.05404976	0.18734596	2	4	0.30102999	0.18734596
chr4	95496808	95548979	52172	1	1	3	0.05404976	0.10122019	2	4	0.30102999	0.30102999
chr4	95548979	95585351	36373	1	1	3	0.05404976	0.30102999	2	2	0.30102999	0.08289318
chr4	95585351	95585410	60	1	1	4	0.05404976	0.47744371	2	2	0.30102999	0.0429175
chr4	95585410	95730539	145130	2	1	3	0.05404976	0.30102999	2	2	0.30102999	0.08289318
chr4	95730539	95730598	60	1	1	4	0.05404976	0.47744371	2	2	0.30102999	0.0429175
chr4	95730598	95996087	265490	5	1	2	0.05404976	0.1575501	2	2	0.30102999	0.1575501
chr4	95996087	96075791	79705	3	1	3	0.05404976	0.30102999	2	2	0.30102999	0.08289318
chr4	96075791	96522408	446618	10	1	3	0.05404976	0.17593012	2	3	0.30102999	0.17593012
chr4	96522408	96522467	60	1	1	4	0.05404976	0.18734596	2	4	0.30102999	0.18734596
chr4	96522467	96648516	126050	1	1	3	0.05404976	0.17593012	2	3	0.30102999	0.17593012
chr4	96648516	96761384	112869	1	1	3	0.1218695	0.30102999	1	2	0.1218695	0.08289318
chr4	96761384	96762490	1107	2	2	3	0.30102999	0.30102999	1	2	0.05404976	0.08289318
chr4	96762490	96793717	31228	2	2	3	0.30102999	0.17593012	1	3	0.05404976	0.17593012
chr4	96793717	96895658	101942	1	2	1	0.30102999	0.02438896	1	3	0.05404976	0.51676182
chr4	96895658	96895717	60	1	2	2	0.30102999	0.08289318	1	3	0.05404976	0.30102999
chr4	96895717	97055574	159858	1	2	2	0.61140001	0.08289318	0	3	0	0.30102999
chr4	97055574	97192180	136607	1	1	0	0.30102999	0	0	1	0	0.30102999
chr4	97192180	97444707	252528	3	1	0	0.30102999	0	0	0	0	0
chr4	97444707	97444766	60	1	1	3	0.30102999	0.51676182	0	1	0	0.02438896
chr4	97444766	97547349	102584	1	1	2	0.30102999	0.61140001	0	0	0	0
chr4	97547349	97754958	207610	3	1	2	0.30102999	0.1575501	0	2	0	0.1575501
chr4	97754958	97828558	73601	2	1	3	0.30102999	0.05670724	0	5	0	0.45545077
chr4	97828558	97828617	60	1	1	4	0.30102999	0.06713722	0	6	0	0.44141547
chr4	97828617	97979500	150884	1	1	3	0.30102999	0.10122019	0	4	0	0.30102999
chr4	97979500	98175038	195539	2	1	5	0.1218695	0.30102999	1	4	0.1218695	0.11390336
chr4	98175038	98175097	60	1	1	6	0.1218695	0.20064824	1	6	0.1218695	0.20064824
chr4	98175097	98250538	75442	1	1	5	0.1218695	0.19510895	1	5	0.1218695	0.19510895
chr4	98250538	98390236	139699	2	1	5	0.1218695	0.30102999	1	4	0.1218695	0.11390336
chr4	98390236	98390295	60	1	1	5	0.1218695	0.19510895	1	5	0.1218695	0.19510895
chr4	98390295	98438893	48599	1	1	4	0.1218695	0.18734596	1	4	0.1218695	0.18734596
chr4	98438893	98480065	41173	2	1	5	0.1218695	0.19510895	1	5	0.1218695	0.19510895
chr4	98480065	98537526	57462	2	1	6	0.1218695	0.30102999	1	5	0.1218695	0.12309572
chr4	98537526	98537585	60	1	1	8	0.1218695	0.4250187	1	6	0.1218695	0.08122616
chr4	98537585	98570154	32570	1	1	6	0.1218695	0.30102999	1	5	0.1218695	0.12309572
chr4	98570154	98633824	63671	1	1	5	0.1218695	0.30102999	1	4	0.1218695	0.11390336
chr4	98633824	98678419	44596	2	1	5	0.1218695	0.12309572	1	6	0.1218695	0.30102999
chr4	98678419	98722563	44145	1	1	5	0.1218695	0.19510895	1	5	0.1218695	0.19510895
chr4	98722563	98722622	60	1	1	6	0.1218695	0.20064824	1	6	0.1218695	0.20064824
chr4	98722622	98816366	93745	2	1	5	0.1218695	0.30102999	1	4	0.1218695	0.11390336
chr4	98816366	98816425	60	1	1	5	0.1218695	0.12309572	1	6	0.1218695	0.30102999
chr4	98816425	98865081	48657	1	1	5	0.1218695	0.19510895	1	5	0.1218695	0.19510895
chr4	98865081	99113952	248872	7	1	5	0.1218695	0.12309572	1	6	0.1218695	0.30102999
chr4	99113952	99273606	159655	4	1	5	0.1218695	0.07511598	1	7	0.1218695	0.43181735
chr4	99273606	99527704	254099	7	1	5	0.1218695	0.04407651	1	8	0.1218695	0.58747015
chr4	99527704	99792178	264475	3	1	5	0.1218695	0.12309572	1	6	0.1218695	0.30102999
chr4	99792178	99888335	96158	4	1	5	0.1218695	0.07511598	1	7	0.1218695	0.43181735
chr4	99888335	99929531	41197	2	1	5	0.1218695	0.04407651	1	8	0.1218695	0.58747015
chr4	99929531	99982702	53172	1	1	5	0.1218695	0.07511598	1	7	0.1218695	0.43181735
chr4	99982702	99982761	60	1	1	6	0.1218695	0.129913	1	7	0.1218695	0.30102999

chr4	99982761	100124832	142072	3	1	5	0.1218695	0.07511598	1	7	0.1218695	0.43181735
chr4	100124832	100256424	131593	6	1	6	0.1218695	0.08122616	1	7	0.1218695	0.4250187
chr4	100256424	100333812	77389	3	1	7	0.1218695	0.13499366	1	8	0.1218695	0.30102999
chr4	100333812	100384021	50210	2	1	7	0.1218695	0.20469099	1	7	0.1218695	0.20469099
chr4	100384021	100384080	60	1	1	7	0.1218695	0.13499366	1	8	0.1218695	0.30102999
chr4	100384080	100740926	356847	6	1	7	0.1218695	0.20469099	1	7	0.1218695	0.20469099
chr4	100740926	100786644	45719	3	1	7	0.1218695	0.13499366	1	8	0.1218695	0.30102999
chr4	100786644	100908940	122297	4	1	7	0.1218695	0.20469099	1	7	0.1218695	0.20469099
chr4	100908940	101160815	251876	7	1	8	0.1218695	0.30102999	1	7	0.1218695	0.13499366
chr4	101160815	101234168	73354	1	1	7	0.1218695	0.20469099	1	7	0.1218695	0.20469099
chr4	101234168	101319111	84944	2	1	7	0.1218695	0.30102999	1	6	0.1218695	0.129913
chr4	101319111	101368859	49749	1	1	7	0.1218695	0.43181735	1	5	0.1218695	0.07511598
chr4	101368859	101368918	60	1	2	7	0.30102999	0.43181735	1	5	0.05404976	0.07511598
chr4	101368918	101439015	70098	2	1	6	0.1218695	0.30102999	1	5	0.1218695	0.12309572
chr4	101439015	101710473	271459	3	1	7	0.1218695	0.43181735	1	5	0.1218695	0.07511598
chr4	101710473	101944715	234243	2	1	6	0.1218695	0.30102999	1	5	0.1218695	0.12309572
chr4	101944715	102042948	98234	3	1	7	0.1218695	0.43181735	1	5	0.1218695	0.07511598
chr4	102042948	102076881	33934	2	1	7	0.1218695	0.30102999	1	6	0.1218695	0.129913
chr4	102076881	102148259	71379	2	1	6	0.1218695	0.30102999	1	5	0.1218695	0.12309572
chr4	102148259	102209343	61085	2	1	5	0.1218695	0.30102999	1	4	0.1218695	0.11390336
chr4	102209343	102209402	60	1	1	6	0.1218695	0.30102999	1	5	0.1218695	0.12309572
chr4	102209402	102255190	45789	1	1	5	0.1218695	0.19510895	1	5	0.1218695	0.19510895
chr4	102255190	102277137	21948	2	1	5	0.05404976	0.19510895	2	5	0.30102999	0.19510895
chr4	102277137	102405772	128636	2	1	5	0.1218695	0.30102999	1	4	0.1218695	0.11390336
chr4	102405772	102735054	329283	6	1	5	0.05404976	0.19510895	2	5	0.30102999	0.19510895
chr4	102735054	102735113	60	1	1	6	0.05404976	0.30102999	2	5	0.30102999	0.12309572
chr4	102735113	102751261	16149	1	1	5	0.05404976	0.19510895	2	5	0.30102999	0.19510895
chr4	102751261	102951250	199990	4	1	5	0.1218695	0.19510895	1	5	0.1218695	0.19510895
chr4	102951250	102981260	30011	2	1	5	0.05404976	0.19510895	2	5	0.30102999	0.19510895
chr4	102981260	103025117	43858	1	1	5	0.1218695	0.19510895	1	5	0.1218695	0.19510895
chr4	103025117	103183535	158419	1	1	4	0.1218695	0.11390336	1	5	0.1218695	0.30102999
chr4	103183535	103245089	61555	3	1	5	0.05404976	0.19510895	2	5	0.30102999	0.19510895
chr4	103245089	103362256	117168	1	1	5	0.05404976	0.30102999	2	4	0.30102999	0.11390336
chr4	103362256	103517270	155015	4	1	5	0.05404976	0.19510895	2	5	0.30102999	0.19510895
chr4	103517270	103517329	60	1	1	5	0.05404976	0.12309572	2	6	0.30102999	0.30102999
chr4	103517329	103671156	153828	3	1	4	0.05404976	0.06713722	2	6	0.30102999	0.44141547
chr4	103671156	103739038	67883	1	1	4	0.1218695	0.06713722	1	6	0.1218695	0.44141547
chr4	103739038	103941448	202411	5	1	3	0.1218695	0.03070643	1	6	0.1218695	0.63695542
chr4	103941448	104118000	176553	6	1	2	0.30102999	0.0429175	0	4	0	0.47744371
chr4	104118000	104250788	132789	1	1	2	0.30102999	0.08289318	0	3	0	0.30102999
chr4	104250788	104420567	169780	3	1	2	0.30102999	0.0429175	0	4	0	0.47744371
chr4	104420567	104420626	60	1	1	3	0.30102999	0.05670724	0	5	0	0.45545077
chr4	104420626	104638978	218353	4	1	2	0.30102999	0.08289318	0	3	0	0.30102999
chr4	104638978	104699853	60876	1	1	2	0.30102999	0.1575501	0	2	0	0.1575501
chr4	104699853	104826929	127077	1	1	1	0.30102999	0.1218695	0	1	0	0.1218695
chr4	104826929	104955534	128606	1	0	1	0	0.1218695	0	1	0	0.1218695
chr4	104955534	104955593	60	1	0	2	0	0.30102999	0	1	0	0.05404976
chr4	104955593	105342698	387106	2	0	1	0	0.30102999	0	0	0	0
chr4	105342698	105404237	61540	3	0	2	0	0.0429175	0	4	0	0.47744371
chr4	105404237	105460758	56522	1	0	1	0	0.01091641	0	4	0	0.76005302
chr4	105460758	105460817	60	1	0	1	0	0.01091641	1	4	0.30102999	0.76005302
chr4	105460817	105549175	88359	1	0	1	0	0.02438896	0	3	0	0.51676182
chr4	105549175	105658948	109774	1	0	1	0	0.05404976	0	2	0	0.30102999
chr4	105658948	105807123	148176	2	0	3	0	0.30102999	0	2	0	0.08289318
chr4	105807123	105807182	60	1	0	3	0	0.17593012	1	3	0.30102999	0.17593012
chr4	105807182	105935157	127976	1	0	3	0	0.30102999	1	2	0.30102999	0.08289318
chr4	105935157	105935216	60	1	0	5	0	0.45545077	1	3	0.30102999	0.05670724
chr4	105935216	106081316	146101	1	0	5	0	0.68214471	1	2	0.30102999	0.02162467
chr4	106081316	106111334	30019	2	0	6	0	0.63695542	1	3	0.30102999	0.03070643
chr4	106111334	106196087	84754	3	0	6	0	0.91219088	1	2	0.30102999	0.01053319
chr4	106196087	106196146	60	1	0	6	0	0.44141547	1	4	0.30102999	0.06713722
chr4	106196146	106268111	71966	1	0	6	0	0.63695542	1	3	0.30102999	0.03070643
chr4	106268111	106290817	22707	2	0	7	0	0.84395715	2	3	0.61140001	0.01598258
chr4	106290817	106377862	87046	2	0	6	0	0.63695542	2	3	0.61140001	0.03070643
chr4	106377862	106377921	60	1	0	6	0	0.63695542	3	3	0.93173516	0.03070643
chr4	106377921	106510610	132690	3	0	6	0	0.91219088	2	2	0.61140001	0.01053319
chr4	106510610	106624225	113616	2	0	5	0	0.68214471	2	2	0.61140001	0.02162467
chr4	106624225	106632002	7778	2	0	7	0	1.16581773	3	2	0.93173516	0.00493743
chr4	106632002	106766692	134691	3	0	6	0	0.91219088	2	2	0.61140001	0.01053319
chr4	106766692	106766751	60	1	0	6	0	0.91219088	3	2	0.93173516	0.01053319
chr4	106766751	106861302	94552	2	0	4	0	0.47744371	2	2	0.61140001	0.0429175
chr4	106861302	106861361	60	1	0	6	0	0.91219088	3	2	0.93173516	0.01053319
chr4	106861361	106892598	31238	1	0	5	0	0.68214471	3	2	0.93173516	0.02162467
chr4	106892598	106967123	74526	1	0	5	0	0.68214471	2	2	0.61140001	0.02162467
chr4	106967123	106967182	60	1	0	6	0	0.63695542	2	3	0.61140001	0.03070643
chr4	106967182	107113962	146781	3	0	5	0	0.45545077	2	3	0.61140001	0.05670724
chr4	107113962	107168501	54540	2	0	6	0	0.30102999	2	5	0.61140001	0.12309572
chr4	107168501	107200317	31817	1	0	6	0	0.63695542	2	3	0.61140001	0.03070643
chr4	107200317	107248637	48321	2	0	6	0	0.63695542	3	3	0.93173516	0.03070643
chr4	107248637	107473485	224849	2	0	6	0	0.63695542	1	3	0.30102999	0.03070643
chr4	107473485	107473544	60	1	0	6	0	0.44141547	2	4	0.61140001	0.06713722
chr4	107473544	107583802	110259	1	0	5	0	0.30102999	2	4	0.61140001	0.11390336
chr4	107583802	107743558	159757	1	0	4	0	0.18734596	1	4	0.30102999	0.18734596
chr4	107743558	107845369	101812	1	0	3	0	0.17593012	1	3	0.30102999	0.17593012
chr4	107845369	107880092	34724	2	0	3	0	0.17593012	2	3	0.61140001	0.17593012
chr4	107880092	107880151	60	1	0	5	0	0.30102999	2	4	0.61140001	0.11390336
chr4	107880151	107912408	32258	1	0	3	0	0.17593012	1	3	0.30102999	0.17593012
chr4	107912408	107943133	30726	1	0	3	0	0.30102999	1	2	0.30102999	0.08289318
chr4	107943133	107943192	60	1	0	6	0	0.44141547	2	4	0.61140001	0.06713722
chr4	107943192	107975509	32318	1	0	4	0	0.18734596	1	4	0.30102999	0.18734596
chr4	107975509	108044509	69001	2	0	5	0	0.19510895	1	5	0.30102999	0.19510895
chr4	108044509	108088649	44141	1	0	4	0	0.18734596	1	4	0.30102999	0.18734596
chr4	108088649	108088708	60	1	0	4	0	0.11390336	1	5	0.30102999	0.30102999
chr4	108088708	108349141	260434	2	0	3	0	0.30102999	1	2	0.30102999	0.08289318
chr4	108349141	108349200	60	1	0	4	0	0.30102999	1	3	0.30102999	0.10122019
chr4	108349200	108535203	186004	2	0	2	0	0.08289318	1	3	0.30102999	0.30102999
chr4	108535203	108581102	45900	2	0	4	0	0.30102999	1	3	0.30102999	0.10122019
chr4	108581102	108581161	60	1	0	5	0	0.45545077	1	3	0.30102999	0.05670724
chr4	108581161	108611876	30716	1	0	3	0	0.17593012	1	3	0.30102999	0.17593012
chr4	108611876	108700165	88290	3	0	3	0	0.10122019	1	4	0.30102999	0.30102999
chr4	108700165	108746998	46834	2	0	4	0	0.11390336	1	5	0.30102999	0.30102999
chr4	108746998	108747057	60	1	0	6	0	0.30102999	1	5	0.30102999	0.12309572
chr4	108747057	108817183	70127	2	0	5	0	0.30102999	1	4	0.30102999	0.11390336
chr4	108817183	108917377	100195	3	0	4	0	0.18734596	1	4	0.30102999	0.18734596
chr4	108917377	108917436	60	1	0	4	0	0.11390336	1	5	0.30102999	0.30102999
chr4	108917436	108969448	52013	1	0	4	0	0.18734596	1	4	0.30102999	0.18734596
chr4	108969448	109089484	120037	2	0	3	0	0.10122019	1	4	0.30102999	0.30102999
chr4	109089484	109190196	100713	2	0	3	0	0.05670724	1	5	0.30102999	0.45545077
chr4	109190196	109460889	270694	2	0	4	0	0.11390336	2	5	0.61140001	0.30102999
chr4	109460889	109460948	60	1	0	8	0	0.58747015	2	5	0.61140001	0.04407651
chr4	109460948	109572534	111587	3	0	7	0	0.43181735	2	5	0.61140001	0.07511598
chr4	109572534	109572593	60	1	0	8	0	0.58747015	2	5	0.61140001	0.04407651
chr4	109572593	109588360	15768	1	0	7	0	0.43181735	2	5	0.61140001	0.07511598
chr4	109588360	109735096	146737	3	0	7	0	0.43181735	3	5	0.93173516	0.07511598
chr4	109735096	109770011	34916	1	0	7	0	0.43181735	2	5	0.61140001	0.07511598
chr4	109770011	109895638	125628	3	0	8	0	0.58747015	2	5	0.61140001	0.04407651
chr4	109895638	109895697	60	1	0	8	0	0.58747015	3	5	0.93173516	0.04407651
chr4	109895697	109936041	40345	1	0	8	0	0.79906872	3	4	0.93173516	0.02074938
chr4	109936041	109985205	49165	1	0	6	0	0.44141547	2	4	0.61140001	0.06713722
chr4	109985205	110056590	71386	2	0	5	0	0.30102999	2	4	0.61140001	0.11390336
chr4	110056590	110056649	60	1	0	6	0	0.44141547	3	4	0.93173516	0.06713722
chr4	110056649	110098857	42209	1	0	6	0	0.44141547	2	4	0.61140001	0.06713722
chr4	110098857	110221757	122901	4	0	8	0	0.79906872	3	4	0.93173516	0.02074938
chr4	110221757	110461040	239284	8	0	8	0	0.4250187	3	6	0.93173516	0.08122616

chr4	110461040	110546964	85925	2	0	8	0	0.58747015	3	5	0.93173516	0.04407651
chr4	110546964	110547023	60	1	0	9	0	0.76806864	3	5	0.93173516	0.02473314
chr4	110547023	110581353	34331	1	0	8	0	0.79906872	3	4	0.93173516	0.02074938
chr4	110581353	110581412	60	1	0	8	0	0.58747015	3	5	0.93173516	0.04407651
chr4	110581412	110641930	60519	4	0	8	0	0.79906872	3	4	0.93173516	0.02074938
chr4	110641930	110723152	81223	2	0	7	0	0.60763643	3	4	0.93173516	0.03812622
chr4	110723152	110723211	60	1	0	8	0	0.58747015	3	5	0.93173516	0.04407651
chr4	110723211	110754405	31195	2	0	7	0	0.60763643	3	4	0.93173516	0.03812622
chr4	110754405	110834202	79798	1	0	6	0	0.44141547	3	4	0.93173516	0.06713722
chr4	110834202	110834261	60	1	0	7	0	0.60763643	3	4	0.93173516	0.03812622
chr4	110834261	110970511	136251	4	0	7	0	0.60763643	2	4	0.61140001	0.03812622
chr4	110970511	110980932	10422	2	0	8	0	0.58747015	2	5	0.61140001	0.04407651
chr4	110980932	111084948	104017	3	0	7	0	0.43181735	2	5	0.61140001	0.07511598
chr4	111084948	111085007	60	1	0	7	0	0.30102999	2	6	0.61140001	0.129913
chr4	111085007	111119550	34544	1	0	6	0	0.30102999	2	5	0.61140001	0.12309572
chr4	111119550	111254831	135282	2	0	6	0	0.44141547	2	4	0.61140001	0.06713722
chr4	111254831	111254890	60	1	0	6	0	0.30102999	2	5	0.61140001	0.12309572
chr4	111254890	111390285	135396	1	0	5	0	0.19510895	2	5	0.61140001	0.19510895
chr4	111390285	111390344	60	1	0	5	0	0.12309572	2	6	0.61140001	0.30102999
chr4	111390344	111482623	92280	2	0	5	0	0.45545077	1	3	0.30102999	0.05670724
chr4	111482623	111544856	62234	2	0	5	0	0.30102999	1	4	0.30102999	0.11390336
chr4	111544856	111548981	4126	1	0	3	0	0.10122019	1	4	0.30102999	0.30102999
chr4	111548981	111559992	11012	3	0	3	0	0.10122019	2	4	0.61140001	0.30102999
chr4	111559992	111782109	222118	2	0	3	0	0.10122019	1	4	0.30102999	0.30102999
chr4	111782109	111812232	30124	2	0	5	0	0.19510895	1	5	0.30102999	0.19510895
chr4	111812232	111938423	126192	1	0	3	0	0.10122019	1	4	0.30102999	0.30102999
chr4	111938423	112077828	139406	1	0	3	0	0.10122019	0	4	0	0.30102999
chr4	112077828	112077887	60	1	0	5	0	0.19510895	0	5	0	0.19510895
chr4	112077887	112287659	209773	2	0	5	0	0.30102999	0	4	0	0.11390336
chr4	112287659	112441994	154336	1	0	5	0	0.45545077	0	3	0	0.05670724
chr4	112441994	112442053	60	1	0	5	0	0.45545077	1	3	0.30102999	0.05670724
chr4	112442053	112598562	156510	1	0	4	0	0.30102999	0	3	0	0.10122019
chr4	112598562	112598621	60	1	0	5	0	0.19510895	0	5	0	0.19510895
chr4	112598621	112753564	154944	1	0	5	0	0.30102999	0	4	0	0.11390336
chr4	112753564	112828683	75120	2	0	6	0	0.44141547	0	4	0	0.06713722
chr4	112828683	112828742	60	1	0	6	0	0.30102999	0	5	0	0.12309572
chr4	112828742	112979202	150461	1	0	6	0	0.44141547	0	4	0	0.06713722
chr4	112979202	113069250	90049	2	1	6	0.30102999	0.44141547	0	4	0	0.06713722
chr4	113069250	113069309	60	1	1	6	0.1218695	0.44141547	1	4	0.1218695	0.06713722
chr4	113069309	113098795	29487	1	1	6	0.30102999	0.44141547	0	4	0	0.06713722
chr4	113098795	113098854	60	1	1	6	0.1218695	0.44141547	1	4	0.1218695	0.06713722
chr4	113098854	113200226	101373	2	1	5	0.1218695	0.30102999	1	4	0.1218695	0.11390336
chr4	113200226	113249039	48814	2	1	6	0.05404976	0.44141547	2	4	0.30102999	0.06713722
chr4	113249039	113292159	43121	2	1	6	0.05404976	0.30102999	2	5	0.30102999	0.12309572
chr4	113292159	113375690	83532	2	1	6	0.05404976	0.44141547	2	4	0.30102999	0.06713722
chr4	113375690	113462245	86556	3	1	6	0.02438896	0.30102999	3	5	0.51676182	0.12309572
chr4	113462245	113462304	60	1	1	6	0.01091641	0.30102999	4	5	0.76005302	0.12309572
chr4	113462304	113610439	148136	5	1	6	0.01091641	0.44141547	4	4	0.76005302	0.06713722
chr4	113610439	113838424	227986	4	1	5	0.01091641	0.30102999	4	4	0.76005302	0.11390336
chr4	113838424	113838483	60	1	1	7	0.01091641	0.43181735	4	5	0.76005302	0.07511598
chr4	113838483	113908051	69569	1	1	7	0.01091641	0.60763643	4	4	0.76005302	0.03812622
chr4	113908051	114153329	245279	6	1	5	0.01091641	0.30102999	4	4	0.76005302	0.11390336
chr4	114153329	114153388	60	1	1	6	0.01091641	0.44141547	4	4	0.76005302	0.06713722
chr4	114153388	114228114	74727	2	1	5	0.01091641	0.45545077	4	3	0.76005302	0.05670724
chr4	114228114	114375455	147342	3	1	5	0.02438896	0.68214471	3	2	0.51676182	0.02162467
chr4	114375455	114375514	60	1	1	5	0.02438896	0.45545077	3	3	0.51676182	0.05670724
chr4	114375514	114530322	154809	4	0	5	0	0.45545077	3	3	0.93173516	0.05670724
chr4	114530322	114530381	60	1	0	6	0	0.44141547	3	4	0.93173516	0.06713722
chr4	114530381	114822723	292343	4	0	5	0	0.45545077	3	3	0.93173516	0.05670724
chr4	114822723	114824166	1444	2	0	6	0	0.44141547	3	4	0.93173516	0.06713722
chr4	114824166	114915647	91482	2	0	5	0	0.30102999	3	4	0.93173516	0.11390336
chr4	114915647	115146765	231119	1	0	5	0	0.30102999	2	4	0.61140001	0.11390336
chr4	115146765	115146824	60	1	0	6	0	0.30102999	2	5	0.61140001	0.12309572
chr4	115146824	115313299	166476	1	0	5	0	0.45545077	1	3	0.30102999	0.05670724
chr4	115313299	115531861	218563	2	0	4	0	0.30102999	1	3	0.30102999	0.10122019
chr4	115531861	115565870	34010	2	0	5	0	0.45545077	1	3	0.30102999	0.05670724
chr4	115565870	116194418	628549	10	0	4	0	0.30102999	1	3	0.30102999	0.10122019
chr4	116194418	116194477	60	1	0	4	0	0.11390336	1	5	0.30102999	0.30102999
chr4	116194477	116494580	300104	2	0	3	0	0.10122019	0	4	0	0.30102999
chr4	116494580	116681173	186594	2	0	3	0	0.17593012	0	3	0	0.17593012
chr4	116681173	116681232	60	1	0	3	0	0.10122019	0	4	0	0.30102999
chr4	116681232	116833697	152466	1	0	1	0	0.02438896	0	3	0	0.51676182
chr4	116833697	116913753	80057	1	0	1	0	0.05404976	0	2	0	0.30102999
chr4	116913753	117064946	151194	1	0	1	0	0.1218695	0	1	0	0.1218695
chr4	117064946	117212297	147352	1	0	0	0	0	0	1	0	0.30102999
chr4	117212297	117466385	254089	3	0	0	0	0	0	2	0	0.61140001
chr4	117466385	117676119	209735	3	0	2	0	0.1575501	0	2	0	0.1575501
chr4	117676119	117676178	60	1	0	2	0	0.08289318	0	3	0	0.30102999
chr4	117676178	118005405	329228	2	0	1	0	0.05404976	0	2	0	0.30102999
chr4	118005405	118005464	60	1	0	3	0	0.10122019	0	4	0	0.30102999
chr4	118005464	118097129	91666	1	0	0	0	0	0	4	0	1.26272838
chr4	118097129	118165551	68423	2	0	1	0	0.01091641	0	4	0	0.76005302
chr4	118165551	118165610	60	1	0	2	0	0.02162467	0	5	0	0.68214471
chr4	118165610	118273037	107428	1	0	1	0	0.01091641	0	4	0	0.76005302
chr4	118273037	118362538	89502	2	0	2	0	0.0429175	0	4	0	0.47744371
chr4	118362538	118608253	245716	3	0	2	0	0.08289318	0	3	0	0.30102999
chr4	118608253	118810256	202004	2	1	2	0.1218695	0.0429175	1	4	0.1218695	0.47744371
chr4	118810256	118810315	60	1	1	3	0.1218695	0.10122019	1	4	0.1218695	0.30102999
chr4	118810315	118926950	116636	1	1	2	0.1218695	0.08289318	1	3	0.1218695	0.30102999
chr4	118926950	119026148	99199	4	1	2	0.05404976	0.08289318	2	3	0.30102999	0.30102999
chr4	119026148	119256687	230540	6	1	2	0.05404976	0.0429175	2	4	0.30102999	0.47744371
chr4	119256687	119461777	205091	3	1	3	0.05404976	0.10122019	2	4	0.30102999	0.30102999
chr4	119461777	119606253	144477	3	1	3	0.05404976	0.05670724	2	5	0.30102999	0.45545077
chr4	119606253	119631970	25718	2	1	4	0.05404976	0.11390336	2	5	0.30102999	0.30102999
chr4	119631970	119854428	222459	7	2	4	0.08289318	0.11390336	3	5	0.30102999	0.30102999
chr4	119854428	119949827	95400	2	2	3	0.08289318	0.05670724	3	5	0.30102999	0.45545077
chr4	119949827	120003254	53428	1	2	2	0.08289318	0.02162467	3	5	0.30102999	0.68214471
chr4	120003254	120072168	68915	2	1	2	0.02438896	0.02162467	3	5	0.51676182	0.68214471
chr4	120072168	120136208	64041	1	0	2	0	0.0429175	2	4	0.61140001	0.47744371
chr4	120136208	120177994	41787	2	0	3	0	0.10122019	2	4	0.61140001	0.30102999
chr4	120177994	120240141	62148	1	0	3	0	0.17593012	2	3	0.61140001	0.17593012
chr4	120240141	120241854	1714	2	1	3	0.02438896	0.17593012	3	3	0.51676182	0.17593012
chr4	120241854	120241913	60	1	1	3	0.02438896	0.10122019	3	4	0.51676182	0.30102999
chr4	120241913	120811566	569654	7	0	3	0	0.17593012	2	3	0.61140001	0.17593012
chr4	120811566	120980576	169011	2	0	3	0	0.17593012	1	3	0.30102999	0.17593012
chr4	120980576	121287085	306510	6	0	3	0	0.10122019	1	4	0.30102999	0.30102999
chr4	121287085	121392348	105264	2	0	3	0	0.10122019	2	4	0.61140001	0.30102999
chr4	121392348	121529861	137514	1	0	3	0	0.10122019	1	4	0.30102999	0.30102999
chr4	121529861	121529920	60	1	0	3	0	0.05670724	1	5	0.30102999	0.45545077
chr4	121529920	121675720	145801	2	0	3	0	0.10122019	1	4	0.30102999	0.30102999
chr4	121675720	121742447	66728	2	0	3	0	0.10122019	2	4	0.61140001	0.30102999
chr4	121742447	121770514	28068	2	0	4	0	0.11390336	2	5	0.61140001	0.30102999
chr4	121770514	121770573	60	1	0	5	0	0.19510895	4	5	1.26272838	0.19510895
chr4	121770573	122053528	282956	4	0	4	0	0.11390336	2	5	0.61140001	0.30102999
chr4	122053528	122108355	54828	3	1	4	0.05404976	0.11390336	2	5	0.30102999	0.30102999
chr4	122108355	122374162	265808	7	1	5	0.05404976	0.19510895	2	5	0.30102999	0.19510895
chr4	122374162	122374221	60	1	1	5	0.02438896	0.19510895	3	5	0.51676182	0.19510895
chr4	122374221	122604574	230354	2	1	5	0.05404976	0.19510895	2	5	0.30102999	0.19510895
chr4	122604574	122639200	34627	2	1	5	0.02438896	0.19510895	3	5	0.51676182	0.19510895
chr4	122639200	122680210	41011	2	1	5	0.01091641	0.19510895	4	5	0.76005302	0.19510895
chr4	122680210	122737578	57369	3	1	5	0.00478973	0.19510895	5	5	1.02643191	0.19510895
chr4	122737578	122740610	3033	2	1	5	0.00478973	0.30102999	5	4	1.02643191	0.11390336
chr4	122740610	122754453	13844	3	1	5	0.00478973	0.19510895	5	5	1.02643191	0.19510895
chr4	122754453	122791428	36976	1	0	5	0	0.19510895	5	5	1.60515106	0.19510895

chr4	122791428	122791483	56	1	0	5	0	0.19510895	6	5	1.95986592	0.19510895
chr4	122791483	122821223	29741	1	0	5	0	0.19510895	4	5	1.26272838	0.19510895
chr4	122821223	123068008	246786	5	1	5	0.01091641	0.19510895	4	5	0.76005302	0.19510895
chr4	123068008	123283775	215768	7	1	6	0.01091641	0.30102999	4	5	0.76005302	0.12309572
chr4	123283775	123423512	139738	3	1	6	0.01091641	0.44141547	4	4	0.76005302	0.06713722
chr4	123423512	123423571	60	1	1	6	0.01091641	0.30102999	4	5	0.76005302	0.12309572
chr4	123423571	123534022	110452	1	1	6	0.01091641	0.44141547	4	4	0.76005302	0.06713722
chr4	123534022	123539291	5270	2	1	7	0.01091641	0.60763643	4	4	0.76005302	0.03812622
chr4	123539291	123632308	93018	1	1	6	0.02438896	0.44141547	3	4	0.51676182	0.06713722
chr4	123632308	123664642	32335	2	1	6	0.02438896	0.63695542	3	3	0.51676182	0.03070643
chr4	123664642	123753909	89268	1	1	4	0.02438896	0.30102999	3	3	0.51676182	0.10122019
chr4	123753909	123753968	60	1	2	4	0.0429175	0.30102999	4	3	0.47744371	0.10122019
chr4	123753968	123949660	195693	6	2	4	0.08289318	0.30102999	3	3	0.30102999	0.10122019
chr4	123949660	124093635	143976	3	2	4	0.08289318	0.47744371	3	2	0.30102999	0.0429175
chr4	124093635	124177218	83584	2	2	3	0.08289318	0.30102999	3	2	0.30102999	0.08289318
chr4	124177218	124669993	492776	7	1	3	0.02438896	0.30102999	3	2	0.51676182	0.08289318
chr4	124669993	124737537	67545	2	1	5	0.02438896	0.68214471	3	2	0.51676182	0.02162467
chr4	124737537	124851620	114084	1	0	1	0	0.05404976	3	2	0.93173516	0.30102999
chr4	124851620	124976418	124799	1	0	1	0	0.1218695	2	1	0.61140001	0.1218695
chr4	124976418	125079051	102634	1	0	1	0	0.1218695	1	1	0.30102999	0.1218695
chr4	125079051	125224636	145586	1	0	0	0	0	1	1	0.30102999	0.30102999
chr4	125224636	125224695	60	1	2	0	0.1575501	0	2	1	0.1575501	0.30102999
chr4	125224695	125437394	212700	3	1	0	0.05404976	0	2	1	0.30102999	0.30102999
chr4	125437394	125437453	60	1	2	0	0.1575501	0	2	1	0.1575501	0.30102999
chr4	125437453	125631656	194204	3	0	0	0	0	2	1	0.61140001	0.30102999
chr4	125631656	125631715	60	1	1	0	0.05404976	0	2	2	0.30102999	0.61140001
chr4	125631715	126039775	408061	3	0	0	0	0	2	2	0.61140001	0.61140001
chr4	126039775	126308658	268884	5	0	1	0	0.05404976	2	2	0.61140001	0.30102999
chr4	126308658	126308717	60	1	0	1	0	0.05404976	3	2	0.93173516	0.30102999
chr4	126308717	126582726	274010	4	0	0	0	0	2	2	0.61140001	0.61140001
chr4	126582726	126582785	60	1	0	0	0	0	3	2	0.93173516	0.61140001
chr4	126582785	126615813	33029	1	0	0	0	0	1	2	0.30102999	0.61140001
chr4	126615813	126695127	79315	2	0	1	0	0.05404976	1	2	0.30102999	0.30102999
chr4	126695127	127096729	401603	2	0	1	0	0.05404976	0	2	0	0.30102999
chr4	127096729	127252263	155535	2	0	1	0	0.05404976	1	2	0.30102999	0.30102999
chr4	127252263	127453132	200870	1	0	1	0	0.1218695	0	1	0	0.1218695
chr4	127453132	127722426	269295	3	0	1	0	0.05404976	0	2	0	0.30102999
chr4	127722426	127722485	60	1	0	2	0	0.0429175	0	4	0	0.47744371
chr4	127722485	127837499	115015	1	0	2	0	0.08289318	0	3	0	0.30102999
chr4	127837499	127936558	99060	1	0	2	0	0.1575501	0	2	0	0.1575501
chr4	127936558	127994626	58069	2	0	2	0	0.08289318	1	3	0.30102999	0.30102999
chr4	127994626	128056015	61390	2	0	3	0	0.05670724	1	5	0.30102999	0.45545077
chr4	128056015	128056074	60	1	0	4	0	0.11390336	1	5	0.30102999	0.30102999
chr4	128056074	128249974	193901	1	0	2	0	0.1575501	1	2	0.30102999	0.1575501
chr4	128249974	128406045	156072	2	0	2	0	0.1575501	2	2	0.61140001	0.1575501
chr4	128406045	128406104	60	1	1	3	0.05404976	0.17593012	2	3	0.30102999	0.17593012
chr4	128406104	128559394	153291	2	1	1	0.1218695	0.1218695	1	1	0.1218695	0.1218695
chr4	128559394	128590322	30929	2	1	3	0.1218695	0.17593012	1	3	0.1218695	0.17593012
chr4	128590322	128658605	68284	2	1	1	0.1218695	0.1218695	1	1	0.1218695	0.1218695
chr4	128658605	128710070	51466	3	1	2	0.05404976	0.08289318	2	3	0.30102999	0.30102999
chr4	128710070	128776518	66449	1	1	1	0.05404976	0.02438896	2	3	0.30102999	0.51676182
chr4	128776518	128807193	30676	1	0	1	0	0.02438896	2	3	0.61140001	0.51676182
chr4	128807193	128854159	46967	4	0	2	0	0.02162467	2	5	0.61140001	0.68214471
chr4	128854159	128901636	47478	2	0	3	0	0.05670724	2	5	0.61140001	0.45545077
chr4	128901636	128901695	60	1	0	3	0	0.01598258	2	7	0.61140001	0.84395715
chr4	128901695	128949664	47970	1	0	2	0	0.00493743	2	7	0.61140001	1.16581773
chr4	128949664	128981343	31680	1	0	2	0	0.01053319	2	6	0.61140001	0.91219088
chr4	128981343	129258959	277617	7	0	3	0	0.03070643	2	6	0.61140001	0.63695542
chr4	129258959	129631786	372828	5	0	3	0	0.03070643	3	6	0.93173516	0.63695542
chr4	129631786	129631845	60	1	1	3	0.02438896	0.03070643	3	6	0.51676182	0.63695542
chr4	129631845	129688570	56726	1	1	2	0.02438896	0.01053319	3	6	0.51676182	0.91219088
chr4	129688570	129857933	169364	5	1	2	0.05404976	0.0429175	2	4	0.30102999	0.47744371
chr4	129857933	129920976	63044	2	1	2	0.05404976	0.02162467	2	5	0.30102999	0.68214471
chr4	129920976	129965196	44221	1	1	2	0.05404976	0.0429175	2	4	0.30102999	0.47744371
chr4	129965196	130204489	239294	3	0	2	0	0.08289318	0	3	0	0.30102999
chr4	130204489	130677429	472941	4	0	2	0	0.02162467	0	5	0	0.68214471
chr4	130677429	130746617	69189	2	0	3	0	0.05670724	0	5	0	0.45545077
chr4	130746617	130746676	60	1	0	3	0	0.03070643	0	6	0	0.63695542
chr4	130746676	130791586	44911	1	0	1	0	0.01091641	0	4	0	0.76005302
chr4	130791586	130946954	155369	2	0	1	0	0.02438896	0	3	0	0.51676182
chr4	131400699	131645763	245065	3	0	0	0	0	0	2	0	0.61140001
chr4	131645763	131916402	270640	1	0	0	0	0	0	1	0	0.30102999
chr4	132962005	133803315	841311	6	0	0	0	0	0	1	0	0.30102999
chr4	133803315	133939036	135722	2	0	1	0	0.05404976	0	2	0	0.30102999
chr4	133939036	134207962	268927	3	0	0	0	0	0	1	0	0.30102999
chr4	134207962	134396504	188543	2	0	1	0	0.05404976	0	2	0	0.30102999
chr4	134396504	134635512	239009	2	0	0	0	0	0	2	0	0.61140001
chr4	134635512	134766607	131096	2	0	1	0	0.01091641	0	4	0	0.76005302
chr4	134766607	134889916	123310	1	0	0	0	0	0	4	0	1.26272838
chr4	134889916	134889975	60	1	0	0	0	0	0	5	0	1.60515106
chr4	134889975	135023283	133309	1	0	0	0	0	0	4	0	1.26272838
chr4	135023283	135137668	114386	1	0	0	0	0	0	3	0	0.93173516
chr4	135347238	135347297	60	1	0	0	0	0	0	3	0	0.93173516
chr4	135347297	135992638	645342	4	0	0	0	0	0	2	0	0.61140001
chr4	135992638	136277833	285196	2	0	0	0	0	0	1	0	0.30102999
chr4	136277833	136421727	143895	3	0	0	0	0	3	1	0.93173516	0.30102999
chr4	136421727	136587298	165572	2	0	0	0	0	4	1	1.26272838	0.30102999
chr4	136587298	136912336	325039	3	0	0	0	0	4	2	1.26272838	0.61140001
chr4	136912336	137197947	285612	4	0	1	0	0.05404976	4	2	1.26272838	0.30102999
chr4	137197947	137460908	262962	2	0	1	0	0.05404976	2	2	0.61140001	0.30102999
chr4	137460908	137815553	354646	2	0	1	0	0.05404976	1	2	0.30102999	0.30102999
chr4	137815553	137815612	60	1	0	1	0	0.01091641	5	4	1.60515106	0.76005302
chr4	137815612	138128986	313375	2	0	1	0	0.1218695	2	1	0.61140001	0.1218695
chr4	138128986	138129045	60	1	0	1	0	0.01091641	3	4	0.93173516	0.76005302
chr4	138129045	138324113	195069	1	0	0	0	0	3	1	0.93173516	0.30102999
chr4	138324113	138441850	117738	2	0	0	0	0	3	2	0.93173516	0.61140001
chr4	138441850	138560114	118265	2	1	4	0.01091641	0.30102999	4	3	0.76005302	0.10122019
chr4	138560114	138727743	167630	1	0	3	0	0.17593012	3	3	0.93173516	0.17593012
chr4	138727743	138812274	84532	2	0	4	0	0.30102999	3	3	0.93173516	0.10122019
chr4	138812274	138999382	187109	1	0	1	0	0.30102999	3	0	0.93173516	0
chr4	138999382	139074661	75280	2	1	2	0.01091641	0.61140001	4	0	0.76005302	0
chr4	139074661	139218284	143624	4	1	1	0.02438896	0.30102999	3	0	0.51676182	0
chr4	139218284	139218343	60	1	1	1	0.02438896	0.1218695	3	1	0.51676182	0.1218695
chr4	139218343	139291840	73498	1	0	1	0	0.1218695	3	1	0.93173516	0.1218695
chr4	139291840	139291899	60	1	0	1	0	0.1218695	4	1	1.26272838	0.1218695
chr4	139291899	139431686	139788	1	0	1	0	0.30102999	3	0	0.93173516	0
chr4	139431686	139431745	60	1	1	1	0.02438896	0.30102999	3	0	0.51676182	0
chr4	139431745	139517235	85491	1	0	1	0	0.30102999	3	0	0.93173516	0
chr4	139517235	139648168	130934	2	0	3	0	0.30102999	3	2	0.93173516	0.08289318
chr4	139648168	139648227	60	1	0	3	0	0.17593012	5	3	1.60515106	0.17593012
chr4	139648227	139818470	170244	1	0	3	0	0.51676182	4	1	1.26272838	0.02438896
chr4	139818470	139869927	51458	2	0	3	0	0.30102999	4	2	1.26272838	0.08289318
chr4	139869927	139869986	60	1	0	3	0	0.17593012	4	3	1.26272838	0.17593012
chr4	139869986	139957548	87563	1	0	3	0	0.30102999	4	2	1.26272838	0.08289318
chr4	139957548	139993208	35661	3	0	3	0	0.17593012	5	3	1.60515106	0.17593012
chr4	139993208	140046328	53121	1	0	1	0	0.02438896	5	3	1.60515106	0.51676182
chr4	140046328	140046386	59	1	0	1	0	0.02438896	6	3	1.95986592	0.51676182
chr4	140046386	140096835	50450	1	0	1	0	0.05404976	6	2	1.95986592	0.30102999
chr4	140096835	140096894	60	1	1	1	0.00204627	0.05404976	6	2	1.31360226	0.30102999
chr4	140096894	140125726	28833	1	0	1	0	0.05404976	6	2	1.95986592	0.30102999
chr4	140125726	140192161	66436	1	0	1	0	0.1218695	5	1	1.60515106	0.1218695
chr4	140192161	140192220	60	1	1	1	0.00478973	0.1218695	5	1	1.02643191	0.1218695
chr4	140192220	140211404	19185	1	1	0	0.00478973	0	5	1	1.02643191	0.30102999
chr4	140211404	140281998	70595	2	1	0	0.00204627	0	6	1	1.31360226	0.30102999

chr4	140281998	140427323	145326	3	0	0	0	0	5	1	1.60515106	0.30102999
chr4	140427323	140427382	60	1	0	2	0	0.1575501	5	2	1.60515106	0.1575501
chr4	140427382	140532854	105473	3	0	1	0	0.1218695	5	1	1.60515106	0.1218695
chr4	140532854	140532913	60	1	0	2	0	0.1575501	5	2	1.60515106	0.1575501
chr4	140532913	140625423	92511	2	0	1	0	0.1218695	5	1	1.60515106	0.1218695
chr4	140625423	140639077	13655	1	0	0	0	0	5	1	1.60515106	0.30102999
chr4	140639077	140715344	76268	2	0	1	0	0.1218695	5	1	1.60515106	0.1218695
chr4	140715344	140810126	94783	3	0	1	0	0.1218695	6	1	1.95986592	0.1218695
chr4	140810126	140885078	74953	1	0	1	0	0.1218695	5	1	1.60515106	0.1218695
chr4	140885078	140965269	80192	3	0	3	0	0.51676182	5	1	1.60515106	0.02438896
chr4	140965269	141008421	43153	1	0	2	0	0.30102999	5	1	1.60515106	0.05404976
chr4	141008421	141068980	60560	2	0	2	0	0.30102999	6	1	1.95986592	0.05404976
chr4	141068980	141074868	5889	2	0	3	0	0.51676182	6	1	1.95986592	0.02438896
chr4	141074868	141177144	102277	1	0	2	0	0.30102999	6	1	1.95986592	0.05404976
chr4	141177144	141177203	60	1	0	3	0	0.30102999	6	2	1.95986592	0.08289318
chr4	141177203	141205615	28413	1	0	2	0	0.30102999	6	1	1.95986592	0.05404976
chr4	141205615	141425400	219786	7	0	1	0	0.1218695	6	1	1.95986592	0.1218695
chr4	141425400	141471387	45988	1	0	1	0	0.30102999	6	0	1.95986592	0
chr4	141471387	141481076	9690	2	1	1	0.00204627	0.1218695	6	1	1.31360226	0.1218695
chr4	141481076	141481132	57	1	1	1	0.00204627	0.05404976	6	2	1.31360226	0.30102999
chr4	141481132	141554854	73723	1	0	1	0	0.1218695	6	1	1.95986592	0.1218695
chr4	141554854	141781182	226329	3	0	1	0	0.1218695	5	1	1.60515106	0.1218695
chr4	141781182	141818146	36965	1	0	0	0	0	5	1	1.60515106	0.30102999
chr4	141818146	141873736	55591	1	0	0	0	0	5	0	1.60515106	0
chr4	141873736	142049839	176104	4	0	1	0	0.30102999	5	0	1.60515106	0
chr4	142049839	142127120	77282	2	0	3	0	0.93173516	5	0	1.60515106	0
chr4	142127120	142155315	28196	3	0	4	0	0.47744371	5	2	1.60515106	0.0429175
chr4	142155315	142194513	39199	1	0	4	0	0.76005302	5	1	1.60515106	0.01091641
chr4	142194513	142194572	60	1	0	5	0	1.02643191	5	1	1.60515106	0.00478973
chr4	142194572	142274902	80331	1	0	4	0	0.76005302	5	1	1.60515106	0.01091641
chr4	142274902	142274961	60	1	0	4	0	0.11390336	5	5	1.60515106	0.30102999
chr4	142274961	142602182	327222	3	0	4	0	0.47744371	5	2	1.60515106	0.0429175
chr4	142602182	142602241	60	1	0	4	0	0.11390336	5	5	1.60515106	0.30102999
chr4	142602241	142654058	51818	1	0	4	0	0.30102999	5	3	1.60515106	0.10122019
chr4	142654058	142676976	22919	2	0	5	0	0.45545077	5	3	1.60515106	0.05670724
chr4	142676976	142845871	168896	1	0	4	0	0.47744371	5	2	1.60515106	0.0429175
chr4	142845871	142941573	95703	2	0	4	0	0.30102999	5	3	1.60515106	0.10122019
chr4	142941573	143003352	61780	3	0	5	0	0.45545077	5	3	1.60515106	0.05670724
chr4	143003352	143159084	155733	3	0	4	0	0.47744371	5	2	1.60515106	0.0429175
chr4	143159084	143195842	36759	2	0	6	0	0.91219088	5	2	1.60515106	0.01053319
chr4	143195842	143195901	60	1	0	6	0	0.63695542	5	3	1.60515106	0.03070643
chr4	143195901	143324115	128215	3	0	5	0	0.45545077	5	3	1.60515106	0.05670724
chr4	143324115	143324174	60	1	0	5	0	0.30102999	5	4	1.60515106	0.11390336
chr4	143324174	143435290	111117	2	0	4	0	0.30102999	5	3	1.60515106	0.10122019
chr4	143435290	143479103	43814	2	0	5	0	0.45545077	5	3	1.60515106	0.05670724
chr4	143479103	143479162	60	1	0	6	0	0.30102999	5	5	1.60515106	0.12309572
chr4	143479162	143585146	105985	2	0	6	0	0.44141547	5	4	1.60515106	0.06713722
chr4	143585146	143652628	67483	3	0	6	0	0.30102999	5	5	1.60515106	0.12309572
chr4	143652628	143784539	131912	3	0	6	0	0.63695542	5	3	1.60515106	0.03070643
chr4	143784539	143884358	99820	2	0	6	0	0.20064824	5	6	1.60515106	0.20064824
chr4	143884358	144108953	224596	2	0	4	0	0.30102999	5	3	1.60515106	0.10122019
chr4	144108953	144134794	25842	3	1	6	0.00478973	0.63695542	5	3	1.02643191	0.03070643
chr4	144134794	144212055	77262	1	0	4	0	0.30102999	5	3	1.60515106	0.10122019
chr4	144212055	144212114	60	1	0	4	0	0.30102999	6	3	1.95986592	0.10122019
chr4	144212114	144336861	124748	3	0	4	0	0.30102999	5	3	1.60515106	0.10122019
chr4	144336861	144588607	251747	7	0	4	0	0.47744371	5	2	1.60515106	0.0429175
chr4	144588607	144659362	70756	2	1	4	0.00478973	0.47744371	5	2	1.02643191	0.0429175
chr4	144659362	144744584	85223	2	1	4	0.00478973	0.30102999	5	3	1.02643191	0.10122019
chr4	144744584	144842647	98064	2	1	5	0.00478973	0.12309572	5	6	1.02643191	0.30102999
chr4	144842647	145096624	253978	5	1	4	0.00478973	0.06713722	5	6	1.02643191	0.44141547
chr4	145096624	145096683	60	1	2	4	0.02162467	0.06713722	5	6	0.68214471	0.44141547
chr4	145096683	145188628	91946	1	2	4	0.0429175	0.11390336	4	5	0.47744371	0.30102999
chr4	145188628	145275116	86489	2	2	5	0.0429175	0.19510895	4	5	0.47744371	0.19510895
chr4	145275116	145413845	138730	1	1	5	0.01091641	0.19510895	4	5	0.76005302	0.19510895
chr4	145413845	145581078	167234	3	1	5	0.01091641	0.12309572	4	6	0.76005302	0.30102999
chr4	145581078	145633141	52064	2	1	4	0.01091641	0.11390336	4	5	0.76005302	0.30102999
chr4	145633141	145659615	26475	2	1	5	0.01091641	0.12309572	4	6	0.76005302	0.30102999
chr4	145659615	145923269	263655	3	1	4	0.01091641	0.11390336	4	5	0.76005302	0.30102999
chr4	145923269	145963820	40552	2	1	4	0.01091641	0.06713722	4	6	0.76005302	0.44141547
chr4	145963820	145963879	60	1	1	5	0.01091641	0.12309572	4	6	0.76005302	0.30102999
chr4	145963879	146029280	65402	1	1	4	0.01091641	0.06713722	4	6	0.76005302	0.44141547
chr4	146029280	146091590	62311	3	1	4	0.02438896	0.06713722	3	6	0.51676182	0.44141547
chr4	146091590	146123562	31973	2	1	4	0.01091641	0.06713722	4	6	0.76005302	0.44141547
chr4	146123562	146401995	278434	2	1	4	0.02438896	0.06713722	3	6	0.51676182	0.44141547
chr4	146401995	146402054	60	1	2	4	0.0429175	0.06713722	4	6	0.47744371	0.44141547
chr4	146402054	146435581	33528	1	1	4	0.02438896	0.06713722	3	6	0.51676182	0.44141547
chr4	146435581	146560672	125092	4	1	5	0.02438896	0.07511598	3	7	0.51676182	0.43181735
chr4	146560672	146560731	60	1	2	6	0.0429175	0.08122616	4	8	0.47744371	0.4250187
chr4	146560731	146658315	97585	2	2	4	0.0429175	0.06713722	4	6	0.47744371	0.44141547
chr4	146658315	146744613	86299	2	2	4	0.08289318	0.06713722	3	6	0.30102999	0.44141547
chr4	146744613	146791626	47014	2	2	6	0.0429175	0.129913	4	7	0.47744371	0.30102999
chr4	146791626	146823886	32261	1	2	5	0.0429175	0.12309572	4	6	0.47744371	0.30102999
chr4	146823886	146865223	41338	1	2	5	0.08289318	0.12309572	3	6	0.30102999	0.30102999
chr4	146865223	146931478	66256	2	2	7	0.0429175	0.20469099	4	7	0.47744371	0.20469099
chr4	146931478	146931537	60	1	3	7	0.10122019	0.20469099	4	7	0.30102999	0.20469099
chr4	146931537	147044764	113228	1	2	6	0.08289318	0.30102999	3	5	0.30102999	0.12309572
chr4	147044764	147044823	60	1	2	7	0.08289318	0.43181735	3	5	0.30102999	0.07511598
chr4	147044823	147096418	51596	2	2	5	0.08289318	0.30102999	3	4	0.30102999	0.11390336
chr4	147096418	147096477	60	1	3	7	0.17593012	0.43181735	3	5	0.17593012	0.07511598
chr4	147096477	147215062	118586	3	2	4	0.1575501	0.11390336	2	5	0.1575501	0.30102999
chr4	147215062	147265444	50383	3	3	4	0.30102999	0.11390336	2	5	0.08289318	0.30102999
chr4	147265444	147362825	97382	3	3	7	0.30102999	0.30102999	2	6	0.08289318	0.129913
chr4	147362825	147397047	34223	2	3	8	0.30102999	0.4250187	2	6	0.08289318	0.08122616
chr4	147397047	147435177	38131	1	3	7	0.30102999	0.30102999	2	6	0.08289318	0.129913
chr4	147435177	147435236	60	1	3	7	0.30102999	0.13499366	2	8	0.08289318	0.30102999
chr4	147435236	147499662	64427	1	3	6	0.30102999	0.129913	2	7	0.08289318	0.30102999
chr4	147499662	147562144	62483	1	2	6	0.1575501	0.129913	2	7	0.1575501	0.30102999
chr4	147562144	147563184	1041	2	2	7	0.08289318	0.13499366	3	8	0.30102999	0.30102999
chr4	147563184	147741367	178184	4	2	7	0.1575501	0.13499366	2	8	0.1575501	0.30102999
chr4	147741367	147741426	60	1	2	8	0.1575501	0.20764654	2	8	0.1575501	0.20764654
chr4	147741426	147795919	54494	1	2	7	0.1575501	0.13499366	2	8	0.1575501	0.30102999
chr4	147795919	147795978	60	1	2	7	0.08289318	0.13499366	3	8	0.30102999	0.30102999
chr4	147795978	147863777	67800	2	2	7	0.1575501	0.13499366	2	8	0.1575501	0.30102999
chr4	147863777	147890375	26599	2	2	7	0.08289318	0.13499366	3	8	0.30102999	0.30102999
chr4	147890375	147981332	90958	1	2	6	0.08289318	0.08122616	3	8	0.30102999	0.4250187
chr4	147981332	148238435	257104	1	2	6	0.1575501	0.08122616	2	8	0.1575501	0.4250187
chr4	148238435	148257949	19515	2	2	6	0.1575501	0.04875589	2	9	0.1575501	0.5732208
chr4	148257949	148258008	60	1	2	7	0.1575501	0.08584816	2	9	0.1575501	0.42015402
chr4	148258008	148270365	12358	1	2	6	0.1575501	0.04875589	2	9	0.1575501	0.5732208
chr4	148270365	148406895	136531	2	2	5	0.1575501	0.02473314	2	9	0.1575501	0.76806864
chr4	148406895	148406954	60	1	2	6	0.1575501	0.04875589	2	9	0.1575501	0.5732208
chr4	148406954	148555569	148616	4	2	5	0.1575501	0.04407651	2	8	0.1575501	0.58747015
chr4	148555569	148559297	3729	2	2	6	0.1575501	0.04875589	2	9	0.1575501	0.5732208
chr4	148559297	148601450	42154	1	2	6	0.1575501	0.08122616	2	8	0.1575501	0.4250187
chr4	148601450	148639085	37636	2	2	8	0.1575501	0.20764654	2	8	0.1575501	0.20764654
chr4	148639085	148744063	104979	3	1	7	0.05404976	0.13499366	2	8	0.30102999	0.30102999
chr4	148744063	148772165	28103	2	1	7	0.05404976	0.08584816	2	9	0.30102999	0.42015402
chr4	148772165	148804576	32412	1	1	6	0.05404976	0.08122616	2	8	0.30102999	0.4250187
chr4	148804576	148876480	71905	2	1	6	0.05404976	0.129913	2	7	0.30102999	0.30102999
chr4	148876480	148911418	34939	2	1	6	0.05404976	0.08122616	2	8	0.30102999	0.4250187
chr4	148911418	148944614	33197	3	1	6	0.02438896	0.08122616	3	8	0.51676182	0.4250187
chr4	148944614	149010193	65580	2	1	6	0.02438896	0.129913	3	7	0.51676182	0.30102999
chr4	149010193	149181251	171059	5	1	7	0.02438896	0.20469099	3	7	0.51676182	0.20469099
chr4	149181251	149245228	63978	1	1	7	0.05404976	0.20469099	2	7	0.30102999	0.20469099

chr4	149245228	149245287	60	1	1	7	0.05404976	0.13499366	2	8	0.30102999	0.30102999
chr4	149245287	149284619	39333	1	1	6	0.05404976	0.129913	2	7	0.30102999	0.30102999
chr4	149284619	149467140	182522	2	1	5	0.05404976	0.07511598	2	7	0.30102999	0.43181735
chr4	149467140	149555306	88167	1	1	3	0.05404976	0.03070643	2	6	0.30102999	0.63695542
chr4	149555306	149695604	140299	1	1	3	0.05404976	0.17593012	2	3	0.30102999	0.17593012
chr4	149695604	149695663	60	1	2	4	0.1575501	0.18734596	2	4	0.1575501	0.18734596
chr4	149695663	149892825	197163	1	0	4	0	0.18734596	2	4	0.61140001	0.18734596
chr4	149892825	149892884	60	1	0	6	0	0.04875589	2	9	0.61140001	0.5732208
chr4	149892884	149970772	77889	1	0	6	0	0.129913	2	7	0.61140001	0.30102999
chr4	149970772	149970831	60	1	0	8	0	0.30102999	2	7	0.61140001	0.13499366
chr4	149970831	150233681	262851	2	0	3	0	0.17593012	1	3	0.30102999	0.17593012
chr4	150233681	150233740	60	1	1	4	0.05404976	0.30102999	2	3	0.30102999	0.10122019
chr4	150233740	150325481	91742	1	0	4	0	0.30102999	2	3	0.61140001	0.10122019
chr4	150325481	150325540	60	1	0	6	0	0.129913	2	7	0.61140001	0.30102999
chr4	150325540	150605535	279996	3	0	5	0	0.19510895	2	5	0.61140001	0.19510895
chr4	150605535	150605594	60	1	0	6	0	0.20064824	2	6	0.61140001	0.20064824
chr4	150605594	150690325	84732	1	0	6	0	0.30102999	2	5	0.61140001	0.12309572
chr4	150690325	150741996	51672	1	0	5	0	0.19510895	2	5	0.61140001	0.19510895
chr4	150741996	150742055	60	1	0	6	0	0.30102999	2	5	0.61140001	0.12309572
chr4	150742055	150807105	65051	1	0	5	0	0.19510895	2	5	0.61140001	0.19510895
chr4	150807105	150807164	60	1	1	5	0.05404976	0.19510895	2	5	0.30102999	0.19510895
chr4	150807164	150944669	137506	1	0	4	0	0.11390336	2	5	0.61140001	0.30102999
chr4	150944669	151023663	78995	1	0	3	0	0.05670724	2	5	0.61140001	0.45545077
chr4	151023663	151062397	38735	2	2	4	0.1575501	0.11390336	2	5	0.1575501	0.30102999
chr4	151062397	151138773	76377	2	1	4	0.05404976	0.11390336	2	5	0.30102999	0.30102999
chr4	151138773	151211578	72806	3	2	5	0.1575501	0.12309572	2	6	0.1575501	0.30102999
chr4	151211578	151211637	60	1	2	5	0.1575501	0.07511598	2	7	0.1575501	0.43181735
chr4	151211637	151242375	30739	1	2	5	0.1575501	0.12309572	2	6	0.1575501	0.30102999
chr4	151242375	151312601	70227	1	1	5	0.05404976	0.12309572	2	6	0.30102999	0.30102999
chr4	151312601	151312660	60	1	1	5	0.05404976	0.07511598	2	7	0.30102999	0.43181735
chr4	151312660	151419552	106893	2	1	3	0.05404976	0.01598258	2	7	0.30102999	0.84395715
chr4	151419552	151470134	50583	1	1	2	0.05404976	0.01053319	2	6	0.30102999	0.91219088
chr4	151470134	151503983	33850	3	2	2	0.1575501	0.01053319	2	6	0.1575501	0.91219088
chr4	151503983	151624865	120883	3	1	2	0.05404976	0.01053319	2	6	0.30102999	0.91219088
chr4	151624865	151678774	53910	2	2	2	0.1575501	0.01053319	2	6	0.1575501	0.91219088
chr4	151678774	151678833	60	1	2	3	0.1575501	0.01598258	2	7	0.1575501	0.84395715
chr4	151678833	152092541	413709	9	2	3	0.1575501	0.03070643	2	6	0.1575501	0.63695542
chr4	152092541	152092600	60	1	2	4	0.1575501	0.06713722	2	6	0.1575501	0.44141547
chr4	152092600	152201339	108740	2	2	3	0.1575501	0.03070643	2	6	0.1575501	0.63695542
chr4	152201339	152201398	60	1	2	4	0.1575501	0.06713722	2	6	0.1575501	0.44141547
chr4	152201398	152261714	60317	1	2	4	0.1575501	0.11390336	2	5	0.1575501	0.30102999
chr4	152261714	152409995	148282	2	2	3	0.1575501	0.05670724	2	5	0.1575501	0.45545077
chr4	152409995	152466467	56473	2	2	3	0.1575501	0.01598258	2	7	0.1575501	0.84395715
chr4	152466467	152548183	81717	1	2	3	0.30102999	0.03070643	1	6	0.05404976	0.63695542
chr4	152548183	152592030	43848	1	2	2	0.30102999	0.0429175	1	4	0.05404976	0.47744371
chr4	152592030	152640630	48601	1	2	2	0.61140001	0.0429175	0	4	0	0.47744371
chr4	152640630	152720434	79805	1	1	2	0.30102999	0.0429175	0	4	0	0.47744371
chr4	152720434	152720493	60	1	1	2	0.1218695	0.0429175	1	4	0.1218695	0.47744371
chr4	152720493	152877808	157316	1	1	2	0.1218695	0.08289318	1	3	0.1218695	0.30102999
chr4	152877808	153008656	130849	2	2	3	0.30102999	0.17593012	1	3	0.05404976	0.17593012
chr4	153008656	153179401	170746	1	2	2	0.30102999	0.1575501	1	2	0.05404976	0.1575501
chr4	153179401	153242458	63058	1	1	2	0.1218695	0.1575501	1	2	0.1218695	0.1575501
chr4	153242458	153259046	16589	2	1	3	0.1218695	0.30102999	1	2	0.1218695	0.08289318
chr4	153259046	153317903	58858	1	1	1	0.1218695	0.05404976	1	2	0.1218695	0.30102999
chr4	153317903	153387189	69287	3	2	1	0.30102999	0.05404976	1	2	0.05404976	0.30102999
chr4	153387189	153458987	71799	2	2	4	0.30102999	0.47744371	1	2	0.05404976	0.0429175
chr4	153458987	153459046	60	1	2	4	0.30102999	0.30102999	1	3	0.05404976	0.10122019
chr4	153459046	153575250	116205	1	2	4	0.30102999	0.47744371	1	2	0.05404976	0.0429175
chr4	153575250	153575309	60	1	2	4	0.30102999	0.18734596	1	4	0.05404976	0.18734596
chr4	153575309	153599033	23725	1	2	4	0.30102999	0.30102999	1	3	0.05404976	0.10122019
chr4	153599033	153690877	91845	2	1	4	0.1218695	0.30102999	1	3	0.1218695	0.10122019
chr4	153690877	153985249	294373	6	1	4	0.1218695	0.18734596	1	4	0.1218695	0.18734596
chr4	153985249	154260061	274813	6	1	4	0.1218695	0.30102999	1	3	0.1218695	0.10122019
chr4	154260061	154296780	36720	3	2	4	0.30102999	0.18734596	1	4	0.05404976	0.18734596
chr4	154296780	154368868	72089	2	2	3	0.30102999	0.17593012	1	3	0.05404976	0.17593012
chr4	154368868	154400503	31636	1	1	3	0.1218695	0.17593012	1	3	0.1218695	0.17593012
chr4	154400503	154505770	105268	4	1	4	0.1218695	0.30102999	1	3	0.1218695	0.10122019
chr4	154505770	154505829	60	1	2	4	0.30102999	0.18734596	1	4	0.05404976	0.18734596
chr4	154505829	154621801	115973	3	1	4	0.1218695	0.18734596	1	4	0.1218695	0.18734596
chr4	154621801	154626457	4657	2	2	4	0.30102999	0.18734596	1	4	0.05404976	0.18734596
chr4	154626457	154635451	8995	1	2	3	0.30102999	0.10122019	1	4	0.05404976	0.30102999
chr4	154635451	154664258	28808	1	1	3	0.1218695	0.10122019	1	4	0.1218695	0.30102999
chr4	154664258	154767721	103464	2	1	2	0.1218695	0.0429175	1	4	0.1218695	0.47744371
chr4	154767721	154767780	60	1	1	4	0.1218695	0.18734596	1	4	0.1218695	0.18734596
chr4	154767780	155003587	235808	1	1	3	0.1218695	0.10122019	1	4	0.1218695	0.30102999
chr4	155003587	155157246	153660	2	2	4	0.30102999	0.18734596	1	4	0.05404976	0.18734596
chr4	155157246	155257114	99869	5	2	5	0.30102999	0.30102999	1	4	0.05404976	0.11390336
chr4	155257114	155311705	54592	1	1	4	0.1218695	0.18734596	1	4	0.1218695	0.18734596
chr4	155311705	155382205	70501	2	1	4	0.1218695	0.06713722	1	6	0.1218695	0.44141547
chr4	155382205	155490843	108639	2	1	3	0.1218695	0.03070643	1	6	0.1218695	0.63695542
chr4	155490843	155527838	36996	2	1	1	0.1218695	0.01091641	1	4	0.1218695	0.76005302
chr4	155527838	155527892	55	1	1	3	0.1218695	0.03070643	1	6	0.1218695	0.63695542
chr4	155527892	155662218	134327	2	1	2	0.1218695	0.02162467	1	5	0.1218695	0.68214471
chr4	155662218	155671011	8794	1	1	1	0.1218695	0.01091641	1	4	0.1218695	0.76005302
chr4	155671011	155801974	130964	3	1	1	0.1218695	0.02438896	1	3	0.1218695	0.51676182
chr4	155801974	155904890	102917	2	2	2	0.30102999	0.08289318	1	3	0.05404976	0.30102999
chr4	155904890	155904949	60	1	2	4	0.30102999	0.11390336	1	5	0.05404976	0.30102999
chr4	155904949	156026296	121348	1	2	3	0.30102999	0.10122019	1	4	0.05404976	0.30102999
chr4	156026296	156232623	206328	4	2	3	0.30102999	0.05670724	1	5	0.05404976	0.45545077
chr4	156232623	156294417	61795	3	2	3	0.30102999	0.10122019	1	4	0.05404976	0.30102999
chr4	156294417	156370372	75956	1	2	3	0.61140001	0.10122019	0	4	0	0.30102999
chr4	156370372	156370431	60	1	2	4	0.61140001	0.11390336	0	5	0	0.30102999
chr4	156370431	156523928	153498	2	2	3	0.61140001	0.10122019	0	4	0	0.30102999
chr4	156523928	156523987	60	1	2	3	0.61140001	0.05670724	0	5	0	0.45545077
chr4	156523987	156631641	107655	2	2	3	0.61140001	0.10122019	0	4	0	0.30102999
chr4	156631641	156631700	60	1	2	3	0.61140001	0.05670724	0	5	0	0.45545077
chr4	156631700	156750995	119296	2	2	3	0.61140001	0.10122019	0	4	0	0.30102999
chr4	156750995	156945393	194399	6	2	4	0.61140001	0.11390336	0	5	0	0.30102999
chr4	156945393	156945452	60	1	2	5	0.61140001	0.19510895	0	5	0	0.19510895
chr4	156945452	157152567	207116	1	1	5	0.30102999	0.19510895	0	5	0	0.19510895
chr4	157152567	157152626	60	1	1	6	0.30102999	0.20064824	0	6	0	0.20064824
chr4	157152626	157261335	108710	1	0	6	0	0.20064824	0	6	0	0.20064824
chr4	157261335	157261394	60	1	1	6	0.30102999	0.20064824	0	6	0	0.20064824
chr4	157261394	157343163	81770	1	0	6	0	0.20064824	0	6	0	0.20064824
chr4	157343163	157518678	175516	2	0	6	0	0.129913	0	7	0	0.30102999
chr4	157518678	157518737	60	1	1	6	0.30102999	0.129913	0	7	0	0.30102999
chr4	157518737	157732013	213277	2	0	6	0	0.20064824	0	6	0	0.20064824
chr4	157732013	157840497	108485	3	0	7	0	0.20469099	0	7	0	0.20469099
chr4	157840497	157949206	108710	2	0	6	0	0.20064824	0	6	0	0.20064824
chr4	157949206	158002931	53726	2	0	8	0	0.30102999	0	7	0	0.13499366
chr4	158002931	158103500	100570	2	0	6	0	0.129913	0	7	0	0.30102999
chr4	158103500	158142160	38661	2	0	6	0	0.08122616	0	8	0	0.4250187
chr4	158142160	158142219	60	1	0	7	0	0.13499366	0	8	0	0.30102999
chr4	158142219	158191729	49511	1	0	7	0	0.20469099	0	7	0	0.20469099
chr4	158191729	158270949	79221	3	0	7	0	0.13499366	1	8	0.30102999	0.30102999
chr4	158270949	158271008	60	1	0	8	0	0.13872638	1	9	0.30102999	0.30102999
chr4	158271008	158503743	232736	2	0	7	0	0.08584816	1	9	0.30102999	0.42015402
chr4	158503743	158572472	68730	2	0	9	0	0.14135546	1	10	0.30102999	0.30102999
chr4	158572472	158572531	60	1	0	10	0	0.21118145	1	10	0.30102999	0.21118145
chr4	158572531	158670491	97961	1	0	8	0	0.08923391	1	10	0.30102999	0.4167287
chr4	158670491	158794224	123734	1	0	7	0	0.08584816	1	9	0.30102999	0.42015402
chr4	158794224	158908493	114270	2	0	8	0	0.13872638	1	9	0.30102999	0.30102999
chr4	158908493	158908552	60	1	1	11	0.1218695	0.30102999	1	10	0.1218695	0.14303407
chr4	158908552	158983859	75308	1	1	10	0.1218695	0.21118145	1	10	0.1218695	0.21118145

chr4	158983859	159091434	107576	3	1	12	0.1218695	0.41314172	1	10	0.1218695	0.09290028
chr4	159091434	159132513	41080	1	1	11	0.1218695	0.30102999	1	10	0.1218695	0.14303407
chr4	159132513	159132572	60	1	1	12	0.1218695	0.41314172	1	10	0.1218695	0.09290028
chr4	159132572	159263283	130712	2	1	11	0.1218695	0.30102999	1	10	0.1218695	0.14303407
chr4	159263283	159263342	60	1	1	13	0.1218695	0.5498098	1	10	0.1218695	0.0574087
chr4	159263342	159382823	119482	2	1	12	0.1218695	0.41314172	1	10	0.1218695	0.09290028
chr4	159382823	159502583	119761	2	1	9	0.1218695	0.14135546	1	10	0.1218695	0.30102999
chr4	159502583	159554549	51967	1	0	9	0	0.14135546	1	10	0.30102999	0.30102999
chr4	159554549	159593954	39406	2	0	10	0	0.21118145	1	10	0.30102999	0.21118145
chr4	159593954	159642658	48705	3	0	11	0	0.30102999	1	10	0.30102999	0.14303407
chr4	159642658	159807064	164407	4	0	9	0	0.14135546	1	10	0.30102999	0.30102999
chr4	159807064	159894456	87393	3	0	10	0	0.21118145	1	10	0.30102999	0.21118145
chr4	159894456	159956250	61795	2	0	9	0	0.14135546	1	10	0.30102999	0.30102999
chr4	159956250	159985792	29543	1	0	7	0	0.05232577	1	10	0.30102999	0.56314362
chr4	159985792	160101955	116164	1	0	6	0	0.02793176	0	10	0	0.74627054
chr4	160101955	160102014	60	1	0	7	0	0.05232577	0	10	0	0.56314362
chr4	160102014	160189268	87255	1	0	6	0	0.02793176	0	10	0	0.74627054
chr4	160189268	160355412	166145	5	0	7	0	0.05232577	0	10	0	0.56314362
chr4	160355412	160429414	74003	1	0	7	0	0.08584816	0	9	0	0.42015402
chr4	160429414	160517264	87851	1	0	6	0	0.129913	0	7	0	0.30102999
chr4	160517264	160517323	60	1	0	6	0	0.08122616	0	8	0	0.4250187
chr4	160517323	160689333	172011	1	0	5	0	0.04407651	0	8	0	0.58747015
chr4	160689333	160806765	117433	1	0	5	0	0.07511598	0	7	0	0.43181735
chr4	160806765	160806824	60	1	0	6	0	0.02793176	0	10	0	0.74627054
chr4	160806824	160897361	90538	1	0	4	0	0.00530919	0	10	0	1.2568129
chr4	160897361	161147440	250080	1	0	1	0	8.47E-04	0	7	0	1.62048027
chr4	161147440	161147499	60	1	0	1	0	3.39E-04	0	8	0	1.94674965
chr4	161147499	161477490	329992	2	0	0	0	0	0	2	0	0.61140001
chr4	161477490	161515746	38257	2	0	1	0	8.47E-04	0	7	0	1.62048027
chr4	161515746	161768683	252938	1	0	1	0	0.00204627	0	6	0	1.31360226
chr4	161768683	161768742	60	1	0	3	0	0.00378107	0	9	0	1.33111237
chr4	161768742	161929917	161176	1	0	3	0	0.0079614	0	8	0	1.07548421
chr4	161929917	161929976	60	1	0	4	0	0.01077081	0	9	0	1.01542894
chr4	161929976	162186946	256971	1	0	3	0	0.00378107	0	9	0	1.33111237
chr4	162186946	162307554	120609	2	0	3	0	7.28E-04	0	11	0	1.91540774
chr4	162307554	162371010	63457	1	0	3	0	0.00170589	0	10	0	1.61091002
chr4	162371010	162461146	90137	3	0	4	0	0.00530919	0	10	0	1.2568129
chr4	162461146	162461205	60	1	0	5	0	0.00666883	1	11	0.30102999	1.20557689
chr4	162461205	162888176	426972	9	0	5	0	0.01320236	0	10	0	0.97390707
chr4	162888176	162954756	66581	1	0	5	0	0.02473314	0	9	0	0.76806864
chr4	162954756	163084866	130111	3	0	5	0	0.01320236	0	10	0	0.97390707
chr4	163084866	163084925	60	1	0	7	0	0.00859896	1	13	0.30102999	1.14735594
chr4	163084925	163164521	79597	1	0	5	0	0.00666883	0	11	0	1.20557689
chr4	163164521	163366922	202402	1	0	4	0	0.00530919	0	10	0	1.2568129
chr4	163366922	163435850	68929	2	0	5	0	0.01320236	0	10	0	0.97390707
chr4	163435850	163435909	60	1	0	8	0	0.08923391	0	10	0	0.4167287
chr4	163435909	163669490	233582	1	0	6	0	0.02793176	0	10	0	0.74627054
chr4	163669490	163669549	60	1	0	6	0	0.02793176	1	10	0.30102999	0.74627054
chr4	163669549	164054351	384803	2	0	3	0	0.01598258	0	7	0	0.84395715
chr4	164054351	164245296	190946	4	0	4	0	0.02074938	0	8	0	0.79906872
chr4	164245296	164271409	26114	5	0	5	0	0.01320236	1	10	0.30102999	0.97390707
chr4	164271409	164392415	121007	1	0	4	0	0.00530919	1	10	0.30102999	1.2568129
chr4	164392415	164829766	437352	10	0	3	0	0.00170589	0	10	0	1.61091002
chr4	164829766	164866110	36345	2	0	3	0	7.28E-04	0	11	0	1.91540774
chr4	164866110	164941281	75172	1	0	3	0	0.00170589	0	10	0	1.61091002
chr4	164941281	165032745	91465	2	1	4	0.1218695	0.00530919	1	10	0.1218695	1.2568129
chr4	165032745	165119308	86564	2	1	6	0.1218695	0.02793176	1	10	0.1218695	0.74627054
chr4	165119308	165119367	60	1	1	8	0.1218695	0.03209037	1	12	0.1218695	0.72109894
chr4	165119367	165153490	34124	1	1	8	0.1218695	0.05490675	1	11	0.1218695	0.55623409
chr4	165153490	165229132	75643	1	0	8	0	0.08923391	1	10	0.30102999	0.4167287
chr4	165229132	165229191	60	1	0	9	0	0.14135546	1	10	0.30102999	0.30102999
chr4	165229191	165306519	77329	1	0	9	0	0.14135546	0	10	0	0.30102999
chr4	165306519	165306578	60	1	0	10	0	0.09290028	0	12	0	0.41314172
chr4	165306578	165411087	104510	1	0	9	0	0.0565833	0	12	0	0.55190077
chr4	165411087	165622245	211159	2	0	9	0	0.0565833	1	12	0.30102999	0.55190077
chr4	165622245	165622304	60	1	1	9	0.1218695	0.0565833	1	12	0.1218695	0.55190077
chr4	165622304	165717299	94996	1	0	9	0	0.09154957	1	11	0.30102999	0.41444892
chr4	165717299	165879671	162373	2	0	9	0	0.14135546	1	10	0.30102999	0.30102999
chr4	165879671	166000248	120578	4	1	9	0.1218695	0.09154957	1	11	0.1218695	0.41444892
chr4	166000248	166067425	67178	2	1	9	0.1218695	0.20975986	1	9	0.1218695	0.20975986
chr4	166067425	166090263	22839	2	1	9	0.1218695	0.09154957	1	11	0.1218695	0.41444892
chr4	166090263	166132145	41883	1	1	9	0.1218695	0.14135546	1	10	0.1218695	0.30102999
chr4	166132145	166263354	131210	2	1	6	0.1218695	0.08122616	1	8	0.1218695	0.4250187
chr4	166263354	166310670	47317	2	1	7	0.1218695	0.08584816	1	9	0.1218695	0.42015402
chr4	166310670	166389020	78351	4	1	7	0.1218695	0.05232577	1	10	0.1218695	0.56314362
chr4	166389020	166450866	61847	2	1	7	0.1218695	0.08584816	1	9	0.1218695	0.42015402
chr4	166450866	166583516	132651	1	1	6	0.1218695	0.04875589	1	9	0.1218695	0.5732208
chr4	166583516	166730570	147055	1	0	5	0	0.02473314	1	9	0.30102999	0.76806864
chr4	166730570	166730629	60	1	0	7	0	0.05232577	1	10	0.30102999	0.56314362
chr4	166730629	166795159	64531	1	0	7	0	0.08584816	1	9	0.30102999	0.42015402
chr4	166795159	166795216	58	1	1	8	0.1218695	0.08923391	1	10	0.1218695	0.4167287
chr4	166795216	166848583	53368	1	0	7	0	0.05232577	1	10	0.30102999	0.56314362
chr4	166848583	166848642	60	1	0	7	0	0.05232577	2	10	0.61140001	0.56314362
chr4	166848642	166886588	37947	1	0	6	0	0.02793176	1	10	0.30102999	0.74627054
chr4	166886588	166916289	29702	1	0	6	0	0.04875589	1	9	0.30102999	0.5732208
chr4	166916289	166952081	35793	2	0	7	0	0.08584816	1	9	0.30102999	0.42015402
chr4	166952081	167023386	71306	2	0	6	0	0.08122616	1	8	0.30102999	0.4250187
chr4	167023386	167023445	60	1	1	6	0.1218695	0.04875589	1	9	0.1218695	0.5732208
chr4	167023445	167061918	38474	1	0	6	0	0.04875589	1	9	0.30102999	0.5732208
chr4	167061918	167105152	43235	1	0	5	0	0.04407651	1	8	0.30102999	0.58747015
chr4	167105152	167282122	176971	1	0	4	0	0.02074938	1	8	0.30102999	0.79906872
chr4	167282122	167282181	60	1	1	5	0.1218695	0.04407651	1	8	0.1218695	0.58747015
chr4	167282181	167396654	114474	1	0	4	0	0.03812622	1	7	0.30102999	0.60763643
chr4	167396654	167487741	91088	1	0	2	0	0.01053319	1	6	0.30102999	0.91219088
chr4	167487741	167649124	161384	2	0	3	0	0.03070643	1	6	0.30102999	0.63695542
chr4	167649124	167843635	194512	7	0	5	0	0.07511598	1	7	0.30102999	0.43181735
chr4	167843635	167843694	60	1	0	6	0	0.129913	1	7	0.30102999	0.30102999
chr4	167843694	167921523	77830	2	0	5	0	0.07511598	1	7	0.30102999	0.43181735
chr4	167921523	167921579	57	1	0	6	0	0.129913	1	7	0.30102999	0.30102999
chr4	167921579	168023294	101716	2	0	4	0	0.11390336	1	5	0.30102999	0.30102999
chr4	168023294	168023353	60	1	0	5	0	0.07511598	1	7	0.30102999	0.43181735
chr4	168023353	168108601	85249	2	0	5	0	0.19510895	1	5	0.30102999	0.19510895
chr4	168108601	168139439	30839	2	0	6	0	0.20064824	1	6	0.30102999	0.20064824
chr4	168139439	168139498	60	1	0	6	0	0.129913	1	7	0.30102999	0.30102999
chr4	168139498	168515593	376096	4	0	6	0	0.20064824	1	6	0.30102999	0.20064824
chr4	168515593	168596767	81175	2	1	6	0.1218695	0.129913	1	7	0.1218695	0.30102999
chr4	168596767	168596826	60	1	1	6	0.1218695	0.08122616	1	8	0.1218695	0.4250187
chr4	168596826	168732895	136070	1	1	6	0.1218695	0.20064824	1	6	0.1218695	0.20064824
chr4	168732895	168732954	60	1	1	8	0.1218695	0.4250187	1	6	0.1218695	0.08122616
chr4	168732954	168959029	226076	2	1	7	0.1218695	0.30102999	1	6	0.1218695	0.129913
chr4	168959029	169105794	146766	4	1	9	0.1218695	0.42015402	1	7	0.1218695	0.08584816
chr4	169105794	169137607	31814	2	1	9	0.1218695	0.30102999	1	8	0.1218695	0.13872638
chr4	169137607	169137666	60	1	1	9	0.05404976	0.30102999	2	8	0.30102999	0.13872638
chr4	169137666	169142954	5289	1	1	9	0.05404976	0.42015402	2	7	0.30102999	0.08584816
chr4	169142954	169197208	54255	1	1	9	0.1218695	0.5732208	1	6	0.1218695	0.04875589
chr4	169197208	169387735	190528	5	1	9	0.1218695	0.42015402	1	7	0.1218695	0.08584816
chr4	169387735	169505795	118061	4	1	9	0.05404976	0.42015402	2	7	0.30102999	0.08584816
chr4	169505795	169559180	53386	1	1	9	0.05404976	0.5732208	2	6	0.30102999	0.04875589
chr4	169559180	169621423	62244	3	1	9	0.05404976	0.42015402	2	7	0.30102999	0.08584816
chr4	169621423	169669525	48103	3	1	9	0.05404976	0.30102999	2	8	0.30102999	0.13872638
chr4	169669525	169819593	150069	5	1	9	0.05404976	0.20975986	2	9	0.30102999	0.20975986
chr4	169819593	169819652	60	1	1	10	0.05404976	0.30102999	2	9	0.30102999	0.14135546
chr4	169819652	169910667	91016	2	1	9	0.1218695	0.30102999	1	8	0.1218695	0.13872638
chr4	169910667	169910726	60	1	1	10	0.05404976	0.30102999	2	9	0.30102999	0.14135546

chr4	169910726	169926557	15832	1	1	10	0.1218695	0.30102999	1	9	0.1218695	0.14135546
chr4	169926557	170016091	89535	2	1	10	0.1218695	0.21118145	1	10	0.1218695	0.21118145
chr4	170016091	170016150	60	1	1	11	0.1218695	0.30102999	1	10	0.1218695	0.14303407
chr4	170016150	170109541	93392	2	1	10	0.1218695	0.21118145	1	10	0.1218695	0.21118145
chr4	170109541	170177217	67677	2	0	10	0	0.30102999	1	9	0.30102999	0.14135546
chr4	170177217	170191669	14453	3	1	10	0.1218695	0.21118145	1	10	0.1218695	0.21118145
chr4	170191669	170292905	101237	1	0	10	0	0.21118145	1	10	0.30102999	0.21118145
chr4	170292905	170345752	52848	2	0	11	0	0.30102999	1	10	0.30102999	0.14303407
chr4	170345752	170384111	38360	2	1	11	0.1218695	0.30102999	1	10	0.1218695	0.14303407
chr4	170384111	170458692	74582	2	0	9	0	0.14135546	1	10	0.30102999	0.30102999
chr4	170458692	170458751	60	1	0	10	0	0.21118145	1	10	0.30102999	0.21118145
chr4	170458751	170506572	47822	1	0	9	0	0.14135546	1	10	0.30102999	0.30102999
chr4	170506572	170557213	50642	2	0	11	0	0.30102999	1	10	0.30102999	0.14303407
chr4	170557213	170613242	56030	1	0	10	0	0.30102999	1	9	0.30102999	0.14135546
chr4	170613242	170613301	60	1	1	10	0.1218695	0.30102999	1	9	0.1218695	0.14135546
chr4	170613301	170638268	24968	1	1	9	0.1218695	0.20975986	1	9	0.1218695	0.20975986
chr4	170638268	170676342	38075	1	0	8	0	0.13872638	1	9	0.30102999	0.30102999
chr4	170676342	170717982	41641	1	0	8	0	0.20764654	1	8	0.30102999	0.20764654
chr4	170717982	170822356	104375	1	0	8	0	0.30102999	0	7	0	0.13499366
chr4	170822356	170921438	99083	3	0	8	0	0.13872638	0	9	0	0.30102999
chr4	170921438	170924853	3416	2	1	8	0.1218695	0.13872638	1	9	0.1218695	0.30102999
chr4	170924853	171009584	84732	3	1	6	0.30102999	0.04875589	0	9	0	0.5732208
chr4	171009584	171061324	51741	1	1	5	0.30102999	0.07511598	0	7	0	0.43181735
chr4	171061324	171157969	96646	1	1	3	0.30102999	0.05670724	0	5	0	0.45545077
chr4	171157969	171264950	106982	1	0	3	0	0.05670724	0	5	0	0.45545077
chr4	171264950	171471410	206461	2	0	5	0	0.07511598	0	7	0	0.43181735
chr4	171471410	171516361	44952	2	0	5	0	0.02473314	0	9	0	0.76806864
chr4	171516361	171516420	60	1	0	6	0	0.02793176	0	10	0	0.74627054
chr4	171516420	171587962	71543	1	0	4	0	0.11390336	0	5	0	0.30102999
chr4	171587962	171794591	206630	1	0	3	0	0.10122019	0	4	0	0.30102999
chr4	171794591	171881647	87057	2	0	3	0	0.03070643	0	6	0	0.63695542
chr4	171881647	171881706	60	1	0	6	0	0.20064824	0	6	0	0.20064824
chr4	171881706	172013457	131752	1	0	2	0	0.08289318	0	3	0	0.30102999
chr4	172013457	172152864	139408	2	0	2	0	0.0429175	0	4	0	0.47744371
chr4	172152864	172152923	60	1	0	3	0	0.03070643	1	6	0.30102999	0.63695542
chr4	172152923	172369753	216831	1	0	3	0	0.05670724	1	5	0.30102999	0.45545077
chr4	172369753	172496219	126467	2	0	5	0	0.12309572	1	6	0.30102999	0.30102999
chr4	172496219	172496278	60	1	0	5	0	0.07511598	1	7	0.30102999	0.43181735
chr4	172496278	172602774	106497	1	0	3	0	0.05670724	1	5	0.30102999	0.45545077
chr4	172602774	172741297	138524	2	0	3	0	0.05670724	2	5	0.61140001	0.45545077
chr4	172741297	172930618	189322	4	0	6	0	0.129913	2	7	0.61140001	0.30102999
chr4	172930618	172930677	60	1	0	7	0	0.20469099	2	7	0.61140001	0.20469099
chr4	172930677	172958857	28181	1	0	6	0	0.129913	2	7	0.61140001	0.30102999
chr4	172958857	173123156	164300	4	0	7	0	0.05232577	2	10	0.61140001	0.56314362
chr4	173123156	173191490	68335	3	0	8	0	0.08923391	2	10	0.61140001	0.4167287
chr4	173191490	173191549	60	1	0	9	0	0.09154957	2	11	0.61140001	0.41444892
chr4	173191549	173225040	33492	1	0	9	0	0.09154957	1	11	0.30102999	0.41444892
chr4	173225040	173265171	40132	2	0	10	0	0.14303407	1	11	0.30102999	0.30102999
chr4	173265171	173265230	60	1	0	10	0	0.09290028	1	12	0.30102999	0.41314172
chr4	173265230	173320227	54998	1	0	9	0	0.20975986	1	9	0.30102999	0.20975986
chr4	173320227	173320286	60	1	0	10	0	0.30102999	2	9	0.61140001	0.14135546
chr4	173320286	173389757	69472	2	0	10	0	0.4167287	2	8	0.61140001	0.08923391
chr4	173389757	173389816	60	1	0	10	0	0.30102999	2	9	0.61140001	0.14135546
chr4	173389816	173429556	39741	1	0	10	0	0.4167287	2	8	0.61140001	0.08923391
chr4	173429556	173499640	70085	1	0	9	0	0.30102999	2	8	0.61140001	0.13872638
chr4	173499640	173515167	15528	2	0	10	0	0.30102999	2	9	0.61140001	0.14135546
chr4	173515167	173557224	42058	1	0	9	0	0.30102999	2	8	0.61140001	0.13872638
chr4	173557224	173630484	73261	2	0	9	0	0.20975986	2	9	0.61140001	0.20975986
chr4	173630484	173775754	145271	4	0	10	0	0.21118145	2	10	0.61140001	0.21118145
chr4	173775754	173855435	79682	2	0	9	0	0.14135546	2	10	0.61140001	0.30102999
chr4	173855435	173895390	39956	1	0	9	0	0.20975986	2	9	0.61140001	0.20975986
chr4	173895390	173995028	99639	3	0	9	0	0.14135546	2	10	0.61140001	0.30102999
chr4	173995028	173995087	60	1	0	10	0	0.21118145	2	10	0.61140001	0.21118145
chr4	173995087	174079435	84349	1	0	10	0	0.30102999	2	9	0.61140001	0.14135546
chr4	174079435	174198459	119025	3	0	9	0	0.20975986	2	9	0.61140001	0.20975986
chr4	174198459	174223341	24883	2	0	10	0	0.30102999	2	9	0.61140001	0.14135546
chr4	174223341	174253742	30402	1	0	9	0	0.20975986	2	9	0.61140001	0.20975986
chr4	174253742	174292768	39027	2	0	9	0	0.20975986	3	9	0.93173516	0.20975986
chr4	174292768	174314470	21703	4	0	10	0	0.30102999	3	9	0.93173516	0.14135546
chr4	174314470	174364254	49785	1	0	10	0	0.4167287	3	8	0.93173516	0.08923391
chr4	174364254	174410419	46166	1	0	9	0	0.42015402	2	7	0.61140001	0.08584816
chr4	174410419	174449344	38926	1	0	9	0	0.42015402	1	7	0.30102999	0.08584816
chr4	174449344	174530338	80995	3	0	9	0	0.30102999	1	8	0.30102999	0.13872638
chr4	174530338	174530397	60	1	0	9	0	0.20975986	1	9	0.30102999	0.20975986
chr4	174530397	174596651	66255	1	0	9	0	0.30102999	1	8	0.30102999	0.13872638
chr4	174596651	174596710	60	1	0	9	0	0.30102999	2	8	0.61140001	0.13872638
chr4	174596710	174817531	220822	1	0	8	0	0.20764654	2	8	0.61140001	0.20764654
chr4	174817531	174941335	123805	2	0	8	0	0.13872638	2	9	0.61140001	0.30102999
chr4	174941335	175090510	149176	1	0	8	0	0.4250187	2	6	0.61140001	0.08122616
chr4	175090510	175196826	106317	2	0	7	0	0.43181735	1	5	0.30102999	0.07511598
chr4	175196826	175196885	60	1	0	7	0	0.30102999	1	6	0.30102999	0.129913
chr4	175196885	175224884	28000	1	0	6	0	0.20064824	1	6	0.30102999	0.20064824
chr4	175224884	175239927	15044	2	0	6	0	0.20064824	2	6	0.61140001	0.20064824
chr4	175239927	175260095	20169	1	0	6	0	0.20064824	1	6	0.30102999	0.20064824
chr4	175260095	175260154	60	1	0	6	0	0.20064824	2	6	0.61140001	0.20064824
chr4	175260154	175344518	84365	1	0	5	0	0.12309572	1	6	0.30102999	0.30102999
chr4	175344518	175413193	68676	2	0	5	0	0.07511598	1	7	0.30102999	0.43181735
chr4	175413193	175413252	60	1	0	5	0	0.04407651	1	8	0.30102999	0.58747015
chr4	175413252	175564227	150976	2	0	5	0	0.19510895	1	5	0.30102999	0.19510895
chr4	175564227	175595488	31262	2	0	5	0	0.04407651	1	8	0.30102999	0.58747015
chr4	175595488	175595547	60	1	0	6	0	0.08122616	2	8	0.61140001	0.4250187
chr4	175595547	175710121	114575	2	0	6	0	0.08122616	1	8	0.30102999	0.4250187
chr4	175710121	175852205	142085	3	0	6	0	0.08122616	0	8	0	0.4250187
chr4	175852205	176113231	261027	3	0	9	0	0.30102999	1	8	0.30102999	0.13872638
chr4	176113231	176113290	60	1	0	9	0	0.20975986	2	9	0.61140001	0.20975986
chr4	176113290	176270886	157597	1	0	8	0	0.13872638	1	9	0.30102999	0.30102999
chr4	176270886	176379542	108657	2	1	8	0.1218695	0.13872638	1	9	0.1218695	0.30102999
chr4	176379542	176379601	60	1	1	9	0.1218695	0.20975986	1	9	0.1218695	0.20975986
chr4	176379601	176585881	206281	3	0	9	0	0.20975986	1	9	0.30102999	0.20975986
chr4	176585881	176618459	32579	1	0	8	0	0.13872638	1	9	0.30102999	0.30102999
chr4	176618459	176618518	60	1	0	8	0	0.13872638	2	9	0.61140001	0.30102999
chr4	176618518	176783955	165438	3	0	8	0	0.13872638	1	9	0.30102999	0.30102999
chr4	176783955	177135324	351370	9	0	8	0	0.13872638	0	9	0	0.30102999
chr4	177135324	177236652	101329	5	0	8	0	0.13872638	1	9	0.30102999	0.30102999
chr4	177236652	177331486	94835	2	0	9	0	0.20975986	1	9	0.30102999	0.20975986
chr4	177331486	177498192	166707	2	0	10	0	0.30102999	1	9	0.30102999	0.14135546
chr4	177498192	177498251	60	1	0	11	0	0.41444892	2	9	0.61140001	0.09154957
chr4	177498251	177605065	106815	1	0	10	0	0.30102999	1	9	0.30102999	0.14135546
chr4	177605065	177632728	27664	1	0	10	0	0.4167287	0	8	0	0.08923391
chr4	177632728	177685102	52375	1	0	9	0	0.30102999	0	8	0	0.13872638
chr4	177685102	177738230	53129	2	0	9	0	0.20975986	1	9	0.30102999	0.20975986
chr4	177738230	177738289	60	1	0	10	0	0.30102999	2	9	0.61140001	0.14135546
chr4	177738289	177854172	115884	1	0	9	0	0.30102999	2	8	0.61140001	0.13872638
chr4	177854172	178104943	250772	1	0	8	0	0.30102999	2	7	0.61140001	0.13499366
chr4	178104943	178105002	60	1	0	9	0	0.42015402	2	7	0.61140001	0.08584816
chr4	178105002	178233073	128072	1	0	7	0	0.20469099	2	7	0.61140001	0.20469099
chr4	178233073	178293029	59957	2	0	4	0	0.06713722	2	6	0.61140001	0.44141547
chr4	178293029	178293088	60	1	0	6	0	0.129913	2	7	0.61140001	0.30102999
chr4	178293088	178478423	185336	3	0	4	0	0.06713722	2	6	0.61140001	0.44141547
chr4	178478423	178668115	189693	2	0	3	0	0.05670724	2	5	0.61140001	0.45545077
chr4	178668115	178748736	80622	3	0	3	0	0.03070643	2	6	0.61140001	0.63695542
chr4	178748736	178829254	80519	3	0	4	0	0.06713722	2	6	0.61140001	0.44141547
chr4	178829254	178996524	167271	3	0	3	0	0.10122019	1	4	0.30102999	0.30102999
chr4	178996524	179459635	463112	2	0	3	0	0.10122019	0	4	0	0.30102999

chr4	179459635	179459694	60	1	0	5	0	0.12309572	0	6	0	0.30102999
chr4	179459694	179738779	279086	2	0	2	0	0.1575501	0	2	0	0.1575501
chr4	179738779	179738838	60	1	0	3	0	0.30102999	0	2	0	0.08289318
chr4	180166540	180417434	250895	3	0	1	0	0.1218695	0	1	0	0.1218695
chr4	180417434	180613104	195671	1	0	1	0	0.30102999	0	0	0	0
chr4	180613104	180955202	342099	3	0	3	0	0.10122019	0	4	0	0.30102999
chr4	180955202	181418338	463137	4	0	2	0	0.0429175	0	4	0	0.47744371
chr4	181418338	181418397	60	1	0	2	0	0.02162467	0	5	0	0.68214471
chr4	181418397	181772578	354182	4	0	2	0	0.0429175	0	4	0	0.47744371
chr4	181772578	182302280	529703	5	0	2	0	0.02162467	1	5	0.30102999	0.68214471
chr4	182302280	182453736	151457	2	0	2	0	0.00221948	1	8	0.30102999	1.44210395
chr4	182453736	182453795	60	1	0	3	0	0.0079614	1	8	0.30102999	1.07548421
chr4	182453795	182620988	167194	1	0	3	0	0.03070643	1	6	0.30102999	0.63695542
chr4	182620988	182817097	196110	2	0	4	0	0.06713722	1	6	0.30102999	0.44141547
chr4	182817097	182920816	103720	2	0	5	0	0.04407651	1	8	0.30102999	0.58747015
chr4	182920816	182990240	69425	2	0	6	0	0.08122616	1	8	0.30102999	0.4250187
chr4	182990240	183063613	73374	2	0	6	0	0.04875589	1	9	0.30102999	0.5732208
chr4	183063613	183106730	43118	2	0	6	0	0.08122616	1	8	0.30102999	0.4250187
chr4	183106730	183171959	65230	1	0	6	0	0.129913	1	7	0.30102999	0.30102999
chr4	183171959	183247611	75653	2	0	7	0	0.20469099	1	7	0.30102999	0.20469099
chr4	183247611	183337339	89729	3	0	7	0	0.08584816	1	9	0.30102999	0.42015402
chr4	183337339	183462363	125025	2	0	7	0	0.13499366	1	8	0.30102999	0.30102999
chr4	183462363	183523607	61245	3	0	8	0	0.05490675	1	11	0.30102999	0.55623409
chr4	183523607	183608864	85258	2	0	7	0	0.03037338	1	11	0.30102999	0.73110763
chr4	183608864	183658223	49360	2	0	8	0	0.05490675	1	11	0.30102999	0.55623409
chr4	183658223	183714630	56408	2	0	9	0	0.0565833	1	12	0.30102999	0.55190077
chr4	183714630	183751234	36605	1	0	8	0	0.08923391	1	10	0.30102999	0.4167287
chr4	183751234	183836772	85539	5	0	8	0	0.13872638	1	9	0.30102999	0.30102999
chr4	183836772	183935653	98882	1	0	8	0	0.20764654	1	8	0.30102999	0.20764654
chr4	183935653	184018370	82718	2	0	8	0	0.13872638	1	9	0.30102999	0.30102999
chr4	184018370	184018428	59	1	0	10	0	0.14303407	1	11	0.30102999	0.30102999
chr4	184018428	184087638	69211	1	0	10	0	0.21118145	1	10	0.30102999	0.21118145
chr4	184087638	184087697	60	1	0	10	0	0.14303407	1	11	0.30102999	0.30102999
chr4	184087697	184186817	99121	2	0	8	0	0.20764654	1	8	0.30102999	0.20764654
chr4	184186817	184239016	52200	3	0	8	0	0.03209037	1	12	0.30102999	0.72109894
chr4	184239016	184291513	52498	1	0	8	0	0.08923391	1	10	0.30102999	0.4167287
chr4	184291513	184368385	76873	2	0	9	0	0.14135546	1	10	0.30102999	0.30102999
chr4	184368385	184368444	60	1	0	9	0	0.09154957	1	11	0.30102999	0.41444892
chr4	184368444	184432129	63686	2	0	8	0	0.08923391	1	10	0.30102999	0.4167287
chr4	184432129	184567533	135405	2	0	7	0	0.13499366	1	8	0.30102999	0.30102999
chr4	184567533	184567592	60	1	0	9	0	0.09154957	1	11	0.30102999	0.41444892
chr4	184567592	184606012	38421	2	0	8	0	0.05490675	1	11	0.30102999	0.55623409
chr4	184606012	184618668	12657	1	0	8	0	0.08923391	1	10	0.30102999	0.4167287
chr4	184618668	184628037	9370	2	0	9	0	0.14135546	1	10	0.30102999	0.30102999
chr4	184628037	184717878	89842	1	0	9	0	0.20975986	1	9	0.30102999	0.20975986
chr4	184717878	184717937	60	1	0	9	0	0.14135546	1	10	0.30102999	0.30102999
chr4	184717937	184799413	81477	1	0	9	0	0.20975986	1	9	0.30102999	0.20975986
chr4	184799413	184900899	101487	3	0	9	0	0.14135546	1	10	0.30102999	0.30102999
chr4	184900899	185009999	109101	2	0	8	0	0.08923391	1	10	0.30102999	0.4167287
chr4	185009999	185010058	60	1	0	10	0	0.21118145	1	10	0.30102999	0.21118145
chr4	185010058	185093813	83756	2	0	8	0	0.08923391	1	10	0.30102999	0.4167287
chr4	185093813	185248234	154422	4	0	10	0	0.21118145	1	10	0.30102999	0.21118145
chr4	185248234	185309073	60840	3	0	11	0	0.21200206	1	11	0.30102999	0.21200206
chr4	185309073	185366838	57766	2	0	10	0	0.14303407	1	11	0.30102999	0.30102999
chr4	185366838	185395650	28813	2	0	12	0	0.30102999	1	11	0.30102999	0.14385241
chr4	185395650	185544552	148903	2	0	10	0	0.14303407	1	11	0.30102999	0.30102999
chr4	185544552	185553590	9039	3	0	11	0	0.21200206	1	11	0.30102999	0.21200206
chr4	185553590	185722451	168862	8	0	11	0	0.30102999	1	10	0.30102999	0.14303407
chr4	185722451	185787268	64818	2	0	12	0	0.21227046	1	12	0.30102999	0.21227046
chr4	185787268	185921677	134410	1	0	11	0	0.21200206	1	11	0.30102999	0.21200206
chr4	185921677	185955325	33649	2	0	11	0	0.14385241	1	12	0.30102999	0.30102999
chr4	185955325	186001583	46259	1	0	10	0	0.09290028	1	12	0.30102999	0.41314172
chr4	186001583	186001642	60	1	0	11	0	0.14385241	1	12	0.30102999	0.30102999
chr4	186001642	186103582	101941	3	0	10	0	0.09290028	1	12	0.30102999	0.41314172
chr4	186103582	186132514	28933	2	0	11	0	0.14385241	1	12	0.30102999	0.30102999
chr4	186132514	186366551	234038	11	0	11	0	0.09334429	1	13	0.30102999	0.41271556
chr4	186366551	186435311	68761	2	0	11	0	0.14385241	1	12	0.30102999	0.30102999
chr4	186435311	186435948	638	2	0	12	0	0.0565833	1	15	0.30102999	0.55190077
chr4	186435948	186444614	8667	1	0	12	0	0.09290028	1	14	0.30102999	0.41314172
chr4	186444614	186454600	9987	1	0	11	0	0.0574087	1	14	0.30102999	0.5498098
chr4	186454600	186454659	60	1	0	12	0	0.0565833	1	15	0.30102999	0.55190077
chr4	186454659	186518650	63992	1	0	12	0	0.09290028	1	14	0.30102999	0.41314172
chr4	186518650	186559340	40691	1	0	11	0	0.0574087	1	14	0.30102999	0.5498098
chr4	186559340	186615258	55919	1	0	11	0	0.21200206	1	11	0.30102999	0.21200206
chr4	186615258	186615317	60	1	0	12	0	0.30102999	1	11	0.30102999	0.14385241
chr4	186615317	186663704	48388	1	0	11	0	0.30102999	1	10	0.30102999	0.14303407
chr4	186663704	186696480	32777	2	0	11	0	0.21200206	1	11	0.30102999	0.21200206
chr4	186696480	186817320	120841	2	0	11	0	0.30102999	1	10	0.30102999	0.14303407
chr4	186817320	186817379	60	1	0	12	0	0.41314172	1	10	0.30102999	0.09290028
chr4	186817379	186997805	180427	3	0	11	0	0.41444892	1	9	0.30102999	0.09154957
chr4	186997805	187153401	155597	3	0	10	0	0.30102999	1	9	0.30102999	0.14135546
chr4	187153401	187153460	60	1	0	12	0	0.41314172	1	10	0.30102999	0.09290028
chr4	187153460	187201142	47683	2	0	10	0	0.30102999	1	9	0.30102999	0.14135546
chr4	187201142	187209940	8799	2	0	11	0	0.30102999	1	10	0.30102999	0.14303407
chr4	187209940	187268079	58140	1	0	10	0	0.30102999	1	9	0.30102999	0.14135546
chr4	187268079	187370897	102819	1	0	9	0	0.20975986	1	9	0.30102999	0.20975986
chr4	187370897	187370956	60	1	0	10	0	0.14303407	1	11	0.30102999	0.30102999
chr4	187370956	187499602	128647	2	0	9	0	0.20975986	1	9	0.30102999	0.20975986
chr4	187499602	187542770	43169	1	0	8	0	0.13872638	1	9	0.30102999	0.30102999
chr4	187542770	187613922	71153	2	0	7	0	0.08584816	1	9	0.30102999	0.42015402
chr4	187613922	187613981	60	1	0	9	0	0.14135546	1	10	0.30102999	0.30102999
chr4	187613981	187698927	84947	2	0	7	0	0.08584816	1	9	0.30102999	0.42015402
chr4	187698927	187755458	56532	1	0	7	0	0.13499366	1	8	0.30102999	0.30102999
chr4	187755458	187755517	60	1	0	7	0	0.08584816	1	9	0.30102999	0.42015402
chr4	187755517	187831029	75513	1	0	7	0	0.13499366	1	8	0.30102999	0.30102999
chr4	187831029	187831086	58	1	0	8	0	0.20764654	1	8	0.30102999	0.20764654
chr4	187831086	187916974	85889	1	0	8	0	0.30102999	1	7	0.30102999	0.13499366
chr4	187916974	187979723	62750	2	0	8	0	0.20764654	1	8	0.30102999	0.20764654
chr4	187979723	188039365	59643	2	0	8	0	0.08923391	1	10	0.30102999	0.4167287
chr4	188039365	188191938	152574	3	0	10	0	0.21118145	1	10	0.30102999	0.21118145
chr4	188191938	188238599	46662	1	0	10	0	0.30102999	1	9	0.30102999	0.14135546
chr4	188238599	188285378	46780	1	0	9	0	0.20975986	1	9	0.30102999	0.20975986
chr4	188285378	188405222	119845	1	0	8	0	0.20764654	1	8	0.30102999	0.20764654
chr4	188405222	188533654	128433	2	0	8	0	0.30102999	1	7	0.30102999	0.13499366
chr4	188533654	188533713	60	1	0	8	0	0.20764654	1	8	0.30102999	0.20764654
chr4	188533713	188706640	172928	3	0	8	0	0.30102999	1	7	0.30102999	0.13499366
chr4	188706640	188706699	60	1	0	8	0	0.13872638	1	9	0.30102999	0.30102999
chr4	188706699	188851793	145095	2	0	8	0	0.20764654	1	8	0.30102999	0.20764654
chr4	188851793	189064967	213175	7	0	8	0	0.03209037	1	12	0.30102999	0.72109894
chr4	189064967	189149917	84951	3	0	9	0	0.0331093	1	13	0.30102999	0.71538971
chr4	189149917	189247732	97816	1	0	8	0	0.01767679	1	13	0.30102999	0.91308053
chr4	189247732	189318237	70506	1	0	8	0	0.01767679	0	13	0	0.91308053
chr4	189318237	189374480	56244	2	0	8	0	0.00909834	0	14	0	1.13428373
chr4	189374480	189457938	83459	2	0	8	0	0.00909834	1	14	0.30102999	1.13428373
chr4	189457938	189457997	60	1	0	8	0	0.00188722	1	16	0.30102999	1.67551277
chr4	189457997	189564094	106098	1	0	8	0	0.00433294	0	15	0	1.3873209
chr4	189564094	189564153	60	1	0	10	0	0.01817691	0	15	0	0.90719478
chr4	189564153	189749994	185842	3	0	10	0	0.03344703	0	14	0	0.71353304
chr4	189749994	189844755	94762	1	0	9	0	0.01817691	0	14	0	0.90719478
chr4	189844755	189844814	60	1	0	9	0	0.00926608	0	15	0	1.13004284
chr4	189844814	189914168	69355	1	0	9	0	0.0331093	0	13	0	0.71538971
chr4	189914168	189914227	60	1	0	11	0	0.0574087	0	14	0	0.5498098
chr4	189914227	189968308	54082	1	0	9	0	0.01817691	0	14	0	0.90719478
chr4	189968308	190049274	80967	1	0	7	0	0.00413023	0	14	0	1.39918918

chr4	190049274	190175815	126542	2	0	7	0	0.03037338	0	11	0	0.73110763
chr4	190175815	190350992	175178	2	0	6	0	0.08122616	0	8	0	0.4250187
chr4	190350992	190422533	71542	2	0	7	0	0.05232577	0	10	0	0.56314362
chr4	190422533	190619807	197275	3	0	4	0	0.03812622	0	7	0	0.60763643
chr4	190619807	190650266	30460	2	0	5	0	0.04407651	0	8	0	0.58747015
chr4	190650266	190767055	116790	2	0	2	0	0.01053319	0	6	0	0.91219088
chr4	190767055	190896674	129620	1	0	3	0	0.0079614	0	8	0	1.07548421
chr5	151737	207981	56245	2	2	1	0.1575501	0.1218695	2	1	0.1575501	0.1218695
chr5	207981	364170	156190	4	2	1	0.1575501	0.05404976	2	2	0.1575501	0.30102999
chr5	364170	527552	163383	4	3	1	0.30102999	0.02438896	2	3	0.08289318	0.51676182
chr5	527552	820365	292814	5	3	1	0.17593012	0.02438896	3	3	0.17593012	0.51676182
chr5	820365	871692	51328	2	4	1	0.30102999	0.01091641	3	4	0.10122019	0.76005302
chr5	871692	1021681	149990	2	3	1	0.17593012	0.01091641	3	4	0.17593012	0.76005302
chr5	1021681	1174543	152863	4	4	1	0.30102999	0.01091641	3	4	0.10122019	0.76005302
chr5	1174543	1266671	92129	3	5	1	0.45545077	0.01091641	3	4	0.05670724	0.76005302
chr5	1266671	1340550	73880	1	4	1	0.30102999	0.01091641	3	4	0.10122019	0.76005302
chr5	1340550	1404604	64055	2	5	1	0.45545077	0.01091641	3	4	0.05670724	0.76005302
chr5	1404604	1498180	93577	2	4	1	0.30102999	0.02438896	3	3	0.10122019	0.51676182
chr5	1498180	1673040	174861	4	5	1	0.45545077	0.01091641	3	4	0.05670724	0.76005302
chr5	1673040	1799145	126106	3	5	2	0.45545077	0.0429175	3	4	0.05670724	0.47744371
chr5	1799145	1933186	134042	2	5	2	0.45545077	0.08289318	3	3	0.05670724	0.30102999
chr5	1933186	2059946	126761	3	5	2	0.45545077	0.0429175	3	4	0.05670724	0.47744371
chr5	2059946	2167697	107752	2	5	1	0.45545077	0.01091641	3	4	0.05670724	0.76005302
chr5	2167697	2359386	191690	3	5	1	0.45545077	0.02438896	3	3	0.05670724	0.51676182
chr5	2359386	2359444	59	1	5	1	0.45545077	0.01091641	3	4	0.05670724	0.76005302
chr5	2359444	2700742	341299	4	5	1	0.45545077	0.02438896	3	3	0.05670724	0.51676182
chr5	2700742	3104552	403811	7	6	1	0.63695542	0.02438896	3	3	0.03070643	0.51676182
chr5	3104552	3192024	87473	1	6	1	0.91219088	0.02438896	2	3	0.01053319	0.51676182
chr5	3192024	3286528	94505	1	5	0	0.68214471	0	2	1	0.02162467	0.30102999
chr5	3286528	3404299	117772	2	5	0	0.45545077	0	3	1	0.05670724	0.30102999
chr5	3404299	3404358	60	1	6	0	0.44141547	0	4	1	0.06713722	0.30102999
chr5	3404358	3503104	98747	1	5	0	0.45545077	0	3	1	0.05670724	0.30102999
chr5	3503104	3503163	60	1	5	0	0.45545077	0	3	2	0.05670724	0.61140001
chr5	3503163	3660708	157546	2	5	0	0.45545077	0	3	1	0.05670724	0.30102999
chr5	3660708	3660767	60	1	5	0	0.45545077	0	3	3	0.05670724	0.93173516
chr5	3660767	4065664	404898	6	5	0	0.45545077	0	3	2	0.05670724	0.61140001
chr5	4065664	4349436	283773	4	5	0	0.30102999	0	4	2	0.11390336	0.61140001
chr5	4349436	4382440	33005	2	5	0	0.19510895	0	5	2	0.19510895	0.61140001
chr5	4382440	4641409	258970	3	5	0	0.19510895	0	5	1	0.19510895	0.30102999
chr5	4641409	4641468	60	1	7	0	0.30102999	0	6	1	0.129913	0.30102999
chr5	4641468	4894797	253330	2	6	0	0.44141547	0	4	1	0.06713722	0.30102999
chr5	4894797	4894856	60	1	6	0	0.44141547	0	4	2	0.06713722	0.61140001
chr5	4894856	5123280	228425	3	4	0	0.18734596	0	4	2	0.18734596	0.61140001
chr5	5123280	5290095	166816	4	4	0	0.18734596	0	4	3	0.18734596	0.93173516
chr5	5290095	5290154	60	1	5	0	0.30102999	0	4	3	0.11390336	0.93173516
chr5	5290154	5407990	117837	2	5	0	0.30102999	0	4	2	0.11390336	0.61140001
chr5	5407990	5408049	60	1	5	0	0.30102999	0	4	4	0.11390336	1.26272838
chr5	5408049	5471345	63297	1	5	0	0.30102999	0	4	3	0.11390336	0.93173516
chr5	5471345	5591790	120446	1	5	0	0.30102999	0	4	2	0.11390336	0.61140001
chr5	5591790	5804136	212347	2	5	0	0.19510895	0	5	2	0.19510895	0.61140001
chr5	5804136	5804195	60	1	6	0	0.30102999	0	5	4	0.12309572	1.26272838
chr5	5804195	5956060	151866	1	3	0	0.05670724	0	5	3	0.45545077	0.93173516
chr5	5956060	6112711	156652	1	2	0	0.02162467	0	5	3	0.68214471	0.93173516
chr5	6112711	6112770	60	1	2	0	0.02162467	0	5	4	0.68214471	1.26272838
chr5	6112770	6250083	137314	1	2	0	0.08289318	0	3	3	0.30102999	0.93173516
chr5	6250083	6341184	91102	2	2	0	0.1575501	0	2	3	0.1575501	0.93173516
chr5	6341184	6414428	73245	2	2	0	0.08289318	0	3	3	0.30102999	0.93173516
chr5	6414428	6414487	60	1	2	0	0.08289318	0	3	4	0.30102999	1.26272838
chr5	6414487	6578122	163636	2	2	0	0.30102999	0	1	2	0.05404976	0.61140001
chr5	6578122	6632269	54148	2	2	0	0.08289318	0	3	4	0.30102999	1.26272838
chr5	6632269	6707258	74990	2	2	0	0.08289318	0	3	3	0.30102999	0.93173516
chr5	6707258	6753894	46637	2	3	0	0.17593012	0	3	3	0.17593012	0.93173516
chr5	6753894	6753953	60	1	4	0	0.30102999	0	3	3	0.10122019	0.93173516
chr5	6753953	6910791	156839	2	3	0	0.17593012	0	3	3	0.17593012	0.93173516
chr5	6910791	6910850	60	1	4	0	0.30102999	0	3	3	0.10122019	0.93173516
chr5	6910850	7144564	233715	2	4	0	0.30102999	0	3	2	0.10122019	0.61140001
chr5	7144564	7144623	60	1	5	0	0.45545077	0	3	2	0.05670724	0.61140001
chr5	7144623	7315227	170605	1	4	0	0.30102999	0	3	2	0.10122019	0.61140001
chr5	7315227	7315286	60	1	4	0	0.30102999	0	3	3	0.10122019	0.93173516
chr5	7315286	7383218	67933	1	3	0	0.17593012	0	3	3	0.17593012	0.93173516
chr5	7383218	7570834	187617	5	3	0	0.17593012	0	3	2	0.17593012	0.61140001
chr5	7570834	7639686	68853	1	3	0	0.17593012	0	3	1	0.17593012	0.30102999
chr5	7639686	7749693	110008	3	4	0	0.30102999	0	3	1	0.10122019	0.30102999
chr5	7749693	7827355	77663	2	4	0	0.18734596	0	4	1	0.18734596	0.30102999
chr5	7827355	8288012	460658	5	5	0	0.30102999	0	4	1	0.11390336	0.30102999
chr5	8288012	8288071	60	1	8	0	0.79906872	0	4	1	0.02074938	0.30102999
chr5	8288071	8561544	273474	2	7	0	0.60763643	0	4	1	0.03812622	0.30102999
chr5	8561544	8561603	60	1	8	0	0.58747015	0	5	1	0.04407651	0.30102999
chr5	8561603	8747001	185399	2	7	0	0.43181735	0	5	1	0.07511598	0.30102999
chr5	8747001	8982448	235448	2	7	0	0.30102999	0	6	1	0.129913	0.30102999
chr5	8982448	9181533	199086	5	7	0	0.13499366	0	8	1	0.30102999	0.30102999
chr5	9181533	9323329	141797	3	7	0	0.20469099	0	7	1	0.20469099	0.30102999
chr5	9323329	9323388	60	1	8	0	0.20764654	0	8	1	0.20764654	0.30102999
chr5	9323388	9440670	117283	2	8	0	0.30102999	0	7	1	0.13499366	0.30102999
chr5	9440670	9691889	251220	6	8	0	0.20764654	0	8	1	0.20764654	0.30102999
chr5	9691889	9762627	70739	1	7	0	0.13499366	0	8	1	0.30102999	0.30102999
chr5	9762627	9843524	80898	2	7	0	0.13499366	0	8	2	0.30102999	0.61140001
chr5	9843524	9843583	60	1	8	0	0.20764654	0	8	2	0.20764654	0.61140001
chr5	9843583	10378871	535289	7	7	0	0.13499366	0	8	2	0.30102999	0.61140001
chr5	10378871	10435064	56194	3	7	0	0.08584816	0	9	2	0.42015402	0.61140001
chr5	10435064	10586857	151794	4	7	0	0.13499366	0	8	2	0.30102999	0.61140001
chr5	10586857	10707514	120658	4	7	0	0.08584816	0	9	2	0.42015402	0.61140001
chr5	10707514	10746297	38784	2	6	0	0.04875589	0	9	2	0.5732208	0.61140001
chr5	10746297	10930114	183818	2	6	0	0.20064824	0	6	2	0.20064824	0.61140001
chr5	10930114	11199684	269571	5	6	0	0.129913	0	7	2	0.30102999	0.61140001
chr5	11199684	11255648	55965	1	6	0	0.20064824	0	6	2	0.20064824	0.61140001
chr5	11255648	11255707	60	1	6	0	0.129913	0	7	2	0.30102999	0.61140001
chr5	11255707	11549410	293704	6	6	0	0.20064824	0	6	2	0.20064824	0.61140001
chr5	11549410	11609433	60024	1	6	0	0.20064824	0	6	1	0.20064824	0.30102999
chr5	11609433	11767693	158261	4	4	0	0.06713722	0	6	1	0.44141547	0.30102999
chr5	11767693	11843306	75614	2	5	0	0.12309572	0	6	1	0.30102999	0.30102999
chr5	11843306	11897092	53787	2	6	0	0.20064824	0	6	1	0.20064824	0.30102999
chr5	11897092	11985200	88109	1	5	0	0.12309572	0	6	1	0.30102999	0.30102999
chr5	11985200	12284415	299216	1	4	0	0.06713722	0	6	1	0.44141547	0.30102999
chr5	12284415	12464991	180577	1	3	0	0.05670724	0	5	1	0.45545077	0.30102999
chr5	12464991	12699724	234734	3	1	0	0.02438896	0	3	1	0.51676182	0.30102999
chr5	12699724	13266637	566914	2	0	0	0	0	1	0	0.30102999	0
chr5	13266637	13266696	60	1	1	0	0.05404976	0	2	1	0.30102999	0.30102999
chr5	13266696	13505023	238328	1	0	0	0	0	2	1	0.61140001	0.30102999
chr5	13505023	13690522	185500	2	0	0	0	0	6	1	1.95986592	0.30102999
chr5	13690522	14561601	871080	18	2	0	0.01053319	0	6	2	0.91219088	0.61140001
chr5	14561601	14678686	117086	4	3	0	0.01598258	0	7	2	0.84395715	0.61140001
chr5	14678686	14685383	6698	2	3	0	0.01598258	0	7	3	0.84395715	0.93173516
chr5	14685383	14755975	70593	2	3	0	0.01598258	0	7	1	0.84395715	0.30102999
chr5	14755975	14783968	27994	2	3	0	0.01598258	0	7	3	0.84395715	0.93173516
chr5	14783968	14822095	38128	1	3	0	0.01598258	0	7	2	0.84395715	0.61140001
chr5	14822095	14945851	123757	2	2	0	0.00493743	0	7	2	1.16581773	0.61140001
chr5	14945851	14945910	60	1	4	0	0.02074938	0	8	2	0.79906872	0.61140001
chr5	14945910	15032877	86968	1	4	0	0.03812622	0	7	2	0.60763643	0.61140001
chr5	15032877	15234290	201414	3	5	0	0.07511598	0	7	2	0.43181735	0.61140001
chr5	15234290	15300320	66031	2	6	0	0.129913	0	7	2	0.30102999	0.61140001
chr5	15300320	15509048	208729	1	5	0	0.12309572	0	6	2	0.30102999	0.61140001
chr5	15509048	15509107	60	1	5	0	0.07511598	0	7	2	0.43181735	0.61140001
chr5	15509107	15601832	92726	2	5	0	0.12309572	0	6	2	0.30102999	0.61140001

chr5	15601832	15676173	74342	2	4	0	0.11390336	0	5	2	0.30102999	0.61140001
chr5	15676173	15877944	201772	4	3	0	0.05670724	0	5	2	0.45545077	0.61140001
chr5	15877944	15904484	26541	2	5	0	0.12309572	0	6	2	0.30102999	0.61140001
chr5	15904484	15995450	90967	2	4	0	0.06713722	0	6	2	0.44141547	0.61140001
chr5	15995450	16057648	62199	1	4	0	0.11390336	0	5	1	0.30102999	0.30102999
chr5	16057648	16119901	62254	2	6	0	0.20064824	0	6	1	0.20064824	0.30102999
chr5	16119901	16207386	87486	2	5	0	0.12309572	0	6	1	0.30102999	0.30102999
chr5	16207386	16372182	164797	1	4	0	0.11390336	0	5	1	0.30102999	0.30102999
chr5	16372182	16451599	79418	1	4	0	0.18734596	0	4	1	0.18734596	0.30102999
chr5	16451599	16456553	4955	2	5	0	0.19510895	0	5	1	0.19510895	0.30102999
chr5	16456553	16503362	46810	3	5	0	0.12309572	0	6	1	0.30102999	0.30102999
chr5	16503362	16590184	86823	2	4	0	0.11390336	0	5	1	0.30102999	0.30102999
chr5	16590184	16590243	60	1	5	0	0.19510895	0	5	1	0.19510895	0.30102999
chr5	16590243	16620812	30570	1	4	0	0.11390336	0	5	1	0.30102999	0.30102999
chr5	16620812	16620871	60	1	4	0	0.11390336	0	5	2	0.30102999	0.61140001
chr5	16620871	16660012	39142	1	3	0	0.05670724	0	5	2	0.45545077	0.61140001
chr5	16660012	16779642	119631	2	1	0	0.00478973	0	5	2	1.02643191	0.61140001
chr5	16779642	16855353	75712	3	2	0	0.02162467	0	5	2	0.68214471	0.61140001
chr5	16855353	17014228	158876	2	1	0	0.00478973	0	5	2	1.02643191	0.61140001
chr5	17014228	17014287	60	1	2	0	0.01053319	0	6	2	0.91219088	0.61140001
chr5	17014287	17256984	242698	4	2	0	0.02162467	0	5	2	0.68214471	0.61140001
chr5	17256984	17425663	168680	5	3	0	0.05670724	0	5	2	0.45545077	0.61140001
chr5	17425663	17425722	60	1	3	0	0.01598258	0	7	2	0.84395715	0.61140001
chr5	17425722	17652752	227031	1	1	0	0.01091641	0	4	0	0.76005302	0
chr5	17652752	17811948	159197	3	2	0	0.0429175	0	4	0	0.47744371	0
chr5	17811948	17812007	60	1	2	0	0.02162467	0	5	0	0.68214471	0
chr5	17812007	18098980	286974	2	1	0	0.01091641	0	4	0	0.76005302	0
chr5	18098980	18215617	116638	1	0	0	0	0	4	0	1.26272838	0
chr5	18215617	18215676	60	1	0	0	0	0	4	1	1.26272838	0.30102999
chr5	18215676	18561815	346140	2	0	0	0	0	4	0	1.26272838	0
chr5	18561815	18703074	141260	2	1	0	0.00478973	0	5	1	1.02643191	0.30102999
chr5	18703074	18703133	60	1	4	0	0.02074938	0	8	1	0.79906872	0.30102999
chr5	18703133	18996995	293863	2	1	0	0.00478973	0	5	1	1.02643191	0.30102999
chr5	18996995	18997054	60	1	3	0	0.0079614	0	8	1	1.07548421	0.30102999
chr5	18997054	19204238	207185	1	1	0	0.00478973	0	5	1	1.02643191	0.30102999
chr5	19204238	19327188	122951	2	2	0	0.02162467	0	5	1	0.68214471	0.30102999
chr5	19327188	19327247	60	1	4	0	0.06713722	0	6	1	0.44141547	0.30102999
chr5	19327247	19453333	126087	1	3	0	0.03070643	0	6	1	0.63695542	0.30102999
chr5	19453333	19453392	60	1	4	0	0.06713722	0	6	1	0.44141547	0.30102999
chr5	19453392	19537833	84442	2	3	0	0.03070643	0	6	1	0.63695542	0.30102999
chr5	19537833	19606080	68248	2	2	0	0.02162467	0	5	1	0.68214471	0.30102999
chr5	19606080	19668883	62804	1	2	0	0.0429175	0	4	1	0.47744371	0.30102999
chr5	19668883	19668942	60	1	5	0	0.12309572	0	6	2	0.30102999	0.61140001
chr5	19668942	19793209	124268	2	3	0	0.10122019	0	4	2	0.30102999	0.61140001
chr5	19793209	19886283	93075	3	3	1	0.05670724	0.05404976	5	2	0.45545077	0.30102999
chr5	19886283	20049711	163429	3	3	1	0.05670724	0.1218695	5	1	0.45545077	0.1218695
chr5	20049711	20225601	175891	4	3	1	0.03070643	0.05404976	6	2	0.63695542	0.30102999
chr5	20225601	20474807	249207	3	3	1	0.05670724	0.05404976	5	2	0.45545077	0.30102999
chr5	20474807	20563995	89189	1	3	1	0.10122019	0.05404976	4	2	0.30102999	0.30102999
chr5	20563995	20693629	129635	2	2	1	0.0429175	0.05404976	4	2	0.47744371	0.30102999
chr5	20693629	20792200	98572	1	2	0	0.0429175	0	4	1	0.47744371	0.30102999
chr5	20792200	21001561	209362	3	2	0	0.02162467	0	5	1	0.68214471	0.30102999
chr5	21001561	21001620	60	1	2	1	0.02162467	0.1218695	5	1	0.68214471	0.1218695
chr5	21001620	21173362	171743	1	2	0	0.0429175	0	4	1	0.47744371	0.30102999
chr5	21173362	21173421	60	1	3	0	0.10122019	0	4	1	0.30102999	0.30102999
chr5	21173421	21269457	96037	1	2	0	0.0429175	0	4	1	0.47744371	0.30102999
chr5	21269457	21594606	325150	2	1	0	0.01091641	0	4	1	0.76005302	0.30102999
chr5	21594606	21594665	60	1	2	1	0.02162467	0.1218695	5	1	0.68214471	0.1218695
chr5	21594665	21854398	259734	4	1	0	0.00478973	0	5	1	1.02643191	0.30102999
chr5	21854398	21854457	60	1	3	0	0.05670724	0	5	1	0.45545077	0.30102999
chr5	21854457	22187544	333088	4	1	0	0.00478973	0	5	1	1.02643191	0.30102999
chr5	22187544	22246012	58469	1	1	0	0.00478973	0	5	0	1.02643191	0
chr5	22246012	22306108	60097	2	3	0	0.05670724	0	5	0	0.45545077	0
chr5	22306108	22691751	385644	8	3	0	0.03070643	0	6	0	0.63695542	0
chr5	22691751	22814561	122811	2	2	0	0.02162467	0	5	0	0.68214471	0
chr5	22814561	22814620	60	1	2	1	0.02162467	0.30102999	5	0	0.68214471	0
chr5	22814620	23000373	185754	2	1	1	0.00478973	0.30102999	5	0	1.02643191	0
chr5	23000373	23000432	60	1	2	1	0.02162467	0.30102999	5	0	0.68214471	0
chr5	23000432	23117939	117508	1	1	0	0.00478973	0	5	0	1.02643191	0
chr5	23117939	23283337	165399	3	2	0	0.00493743	0	7	0	1.16581773	0
chr5	23283337	23524228	240892	2	2	0	0.01053319	0	6	0	0.91219088	0
chr5	23524228	23524286	59	1	2	1	0.01053319	0.30102999	6	0	0.91219088	0
chr5	23524286	23607162	82877	1	1	1	0.00204627	0.30102999	6	0	1.31360226	0
chr5	23607162	23821705	214544	1	0	0	0	0	6	0	1.95986592	0
chr5	23821705	23968179	146475	1	0	0	0	0	5	0	1.60515106	0
chr5	23968179	24072689	104511	4	0	1	0	0.1218695	5	1	1.60515106	0.1218695
chr5	24072689	24178293	105605	2	0	1	0	0.1218695	4	1	1.26272838	0.1218695
chr5	24178293	24188509	10217	2	1	1	0.00204627	0.1218695	6	1	1.31360226	0.1218695
chr5	24188509	24644745	456237	6	0	1	0	0.1218695	4	1	1.26272838	0.1218695
chr5	24644745	24666557	21813	2	1	1	0.01091641	0.1218695	4	1	0.76005302	0.1218695
chr5	24666557	24976470	309914	2	0	1	0	0.1218695	4	1	1.26272838	0.1218695
chr5	24976470	25104177	127708	1	0	1	0	0.1218695	3	1	0.93173516	0.1218695
chr5	25104177	25166170	61994	2	0	2	0	0.30102999	3	1	0.93173516	0.05404976
chr5	25166170	25281091	114922	1	0	1	0	0.1218695	2	1	0.61140001	0.1218695
chr5	25281091	25469388	188298	1	0	0	0	0	2	1	0.61140001	0.30102999
chr5	26068066	26287290	219225	2	0	0	0	0	2	1	0.61140001	0.30102999
chr5	26287290	26768618	481329	4	0	1	0	0.05404976	2	2	0.61140001	0.30102999
chr5	26768618	26946671	178054	5	0	1	0	0.05404976	3	2	0.93173516	0.30102999
chr5	26946671	27098938	152268	3	0	0	0	0	3	0	0.93173516	0
chr5	27098938	27350139	251202	3	0	0	0	0	4	0	1.26272838	0
chr5	27350139	27350198	60	1	0	2	0	0.30102999	4	1	1.26272838	0.05404976
chr5	27350198	27831015	480818	2	0	1	0	0.1218695	4	1	1.26272838	0.1218695
chr5	27831015	27979981	148967	1	0	1	0	0.30102999	3	0	0.93173516	0
chr5	27979981	27980040	60	1	0	2	0	0.61140001	3	0	0.93173516	0
chr5	27980040	28393916	413877	2	0	1	0	0.30102999	3	0	0.93173516	0
chr5	28393916	29048764	654849	6	0	2	0	0.61140001	3	0	0.93173516	0
chr5	29048764	29152795	104032	2	0	2	0	0.30102999	3	1	0.93173516	0.05404976
chr5	29152795	29371965	219171	1	0	1	0	0.1218695	2	1	0.61140001	0.1218695
chr5	29371965	29760863	388899	3	1	1	0.05404976	0.05404976	2	2	0.30102999	0.30102999
chr5	29760863	29760922	60	1	1	1	0.02438896	0.05404976	3	2	0.51676182	0.30102999
chr5	29760922	29903010	142089	1	0	1	0	0.05404976	1	2	0.30102999	0.30102999
chr5	30349974	30601125	251152	2	0	1	0	0.30102999	0	0	0	0
chr5	30601125	30875060	273936	2	0	1	0	0.05404976	2	2	0.61140001	0.30102999
chr5	30875060	31055478	180419	2	0	1	0	0.05404976	3	2	0.93173516	0.30102999
chr5	31055478	31162841	107364	2	1	2	0.01091641	0.1575501	4	2	0.76005302	0.1575501
chr5	31162841	31162900	60	1	1	2	0.00478973	0.1575501	5	2	1.02643191	0.1575501
chr5	31162900	31250950	88051	2	1	2	0.01091641	0.1575501	4	2	0.76005302	0.1575501
chr5	31250950	31251006	57	1	1	2	0.00204627	0.1575501	6	2	1.31360226	0.1575501
chr5	31251006	31294123	43118	2	1	1	0.00204627	0.05404976	6	2	1.31360226	0.30102999
chr5	31294123	31451628	157506	5	1	2	0.00204627	0.1575501	6	2	1.31360226	0.1575501
chr5	31451628	31681161	229534	6	2	3	0.01053319	0.30102999	6	2	0.91219088	0.08289318
chr5	31681161	31754704	73544	2	2	3	0.00493743	0.30102999	7	2	1.16581773	0.08289318
chr5	31754704	31810074	55371	2	3	3	0.01598258	0.30102999	7	2	0.84395715	0.08289318
chr5	31810074	31810133	60	1	3	4	0.01598258	0.47744371	7	2	0.84395715	0.0429175
chr5	31810133	32008279	198147	4	3	3	0.01598258	0.30102999	7	2	0.84395715	0.08289318
chr5	32008279	32149723	141445	6	3	4	0.01598258	0.47744371	7	2	0.84395715	0.0429175
chr5	32149723	32242242	92520	3	3	4	0.03070643	0.47744371	6	2	0.63695542	0.0429175
chr5	32242242	32263300	21059	1	3	3	0.03070643	0.30102999	6	2	0.63695542	0.08289318
chr5	32263300	32271018	7719	2	3	3	0.03070643	0.17593012	6	3	0.63695542	0.17593012
chr5	32271018	32280502	9485	2	3	4	0.03070643	0.30102999	6	3	0.63695542	0.10122019
chr5	32280502	32864953	584452	14	3	3	0.03070643	0.30102999	6	2	0.63695542	0.08289318
chr5	32864953	32933499	68547	2	3	3	0.03070643	0.17593012	6	3	0.63695542	0.17593012
chr5	32933499	33120488	186990	2	3	3	0.03070643	0.30102999	6	2	0.63695542	0.08289318
chr5	33120488	33120547	60	1	3	4	0.03070643	0.30102999	6	3	0.63695542	0.10122019

chr5	33120547	33247771	127225	2	3	3	0.03070643	0.17593012	6	3	0.63695542	0.17593012
chr5	33247771	33287601	39831	1	3	2	0.03070643	0.08289318	6	3	0.63695542	0.30102999
chr5	33287601	33402372	114772	1	3	2	0.03070643	0.1575501	6	2	0.63695542	0.1575501
chr5	33402372	33508899	106528	2	3	2	0.03070643	0.30102999	6	1	0.63695542	0.05404976
chr5	33508899	33508958	60	1	3	2	0.03070643	0.1575501	6	2	0.63695542	0.1575501
chr5	33508958	33683120	174163	3	3	2	0.03070643	0.30102999	6	1	0.63695542	0.05404976
chr5	33683120	33683173	54	1	3	2	0.03070643	0.1575501	6	2	0.63695542	0.1575501
chr5	33683173	33719802	36630	1	2	2	0.01053319	0.1575501	6	2	0.91219088	0.1575501
chr5	33719802	33764808	45007	1	2	2	0.01053319	0.30102999	6	1	0.91219088	0.05404976
chr5	33764808	33881366	116559	2	2	1	0.02162467	0.1218695	5	1	0.68214471	0.1218695
chr5	33881366	33947411	66046	2	2	2	0.01053319	0.30102999	6	1	0.91219088	0.05404976
chr5	33947411	34073756	126346	8	2	2	0.01053319	0.1575501	6	2	0.91219088	0.1575501
chr5	34073756	34410322	336567	2	2	2	0.0429175	0.1575501	4	2	0.47744371	0.1575501
chr5	34410322	34559420	149099	2	2	2	0.01053319	0.1575501	6	2	0.91219088	0.1575501
chr5	34559420	34611593	52174	2	2	2	0.00493743	0.1575501	7	2	1.16581773	0.1575501
chr5	34611593	34663720	52128	1	2	2	0.01053319	0.1575501	6	2	0.91219088	0.1575501
chr5	34663720	34663779	60	1	2	3	0.01053319	0.30102999	6	2	0.91219088	0.08289318
chr5	34663779	34685933	22155	1	2	3	0.02162467	0.30102999	5	2	0.68214471	0.08289318
chr5	34685933	34744070	58138	1	2	2	0.02162467	0.1575501	5	2	0.68214471	0.1575501
chr5	34744070	34796020	51951	2	2	2	0.01053319	0.1575501	6	2	0.91219088	0.1575501
chr5	34796020	34847496	51477	3	2	3	0.00493743	0.30102999	7	2	1.16581773	0.08289318
chr5	34847496	34913930	66435	2	2	3	0.01053319	0.30102999	6	2	0.91219088	0.08289318
chr5	34913930	35010242	96313	4	2	3	0.00493743	0.30102999	7	2	1.16581773	0.08289318
chr5	35010242	35049017	38776	2	2	3	0.01053319	0.30102999	6	2	0.91219088	0.08289318
chr5	35049017	35049069	53	1	2	3	0.00493743	0.30102999	7	2	1.16581773	0.08289318
chr5	35049069	35155562	106494	2	2	3	0.01053319	0.30102999	6	2	0.91219088	0.08289318
chr5	35155562	35155621	60	1	2	4	0.01053319	0.47744371	6	2	0.91219088	0.0429175
chr5	35155621	35213780	58160	1	2	4	0.02162467	0.47744371	5	2	0.68214471	0.0429175
chr5	35213780	35430004	216225	2	2	4	0.0429175	0.47744371	4	2	0.47744371	0.0429175
chr5	35430004	35430063	60	1	2	4	0.02162467	0.47744371	5	2	0.68214471	0.0429175
chr5	35430063	36110857	680795	17	2	4	0.0429175	0.47744371	4	2	0.47744371	0.0429175
chr5	36110857	36110916	60	1	2	4	0.02162467	0.47744371	5	2	0.68214471	0.0429175
chr5	36110916	36520866	409951	10	2	4	0.0429175	0.47744371	4	2	0.47744371	0.0429175
chr5	36520866	36520925	60	1	2	4	0.02162467	0.47744371	5	2	0.68214471	0.0429175
chr5	36520925	36655780	134856	3	2	4	0.0429175	0.47744371	4	2	0.47744371	0.0429175
chr5	36655780	36655839	60	1	2	4	0.02162467	0.47744371	5	2	0.68214471	0.0429175
chr5	36655839	36809705	153867	2	2	3	0.0429175	0.30102999	4	2	0.47744371	0.08289318
chr5	36809705	36809764	60	1	2	4	0.02162467	0.30102999	5	3	0.68214471	0.10122019
chr5	36809764	36944458	134695	2	2	4	0.02162467	0.47744371	5	2	0.68214471	0.0429175
chr5	36944458	37016145	71688	2	2	3	0.0429175	0.30102999	4	2	0.47744371	0.08289318
chr5	37016145	37061019	44875	2	2	4	0.0429175	0.47744371	4	2	0.47744371	0.0429175
chr5	37061019	37158302	97284	4	2	3	0.0429175	0.30102999	4	2	0.47744371	0.08289318
chr5	37158302	37239267	80966	2	2	3	0.02162467	0.30102999	5	2	0.68214471	0.08289318
chr5	37239267	37292092	52826	1	2	3	0.02162467	0.51676182	5	1	0.68214471	0.02438896
chr5	37292092	37292146	55	1	2	3	0.01053319	0.51676182	6	1	0.91219088	0.02438896
chr5	37292146	37437095	144950	3	2	3	0.02162467	0.51676182	5	1	0.68214471	0.02438896
chr5	37437095	37437154	60	1	2	3	0.02162467	0.30102999	5	2	0.68214471	0.08289318
chr5	37437154	37516603	79450	2	2	3	0.02162467	0.51676182	5	1	0.68214471	0.02438896
chr5	37516603	37516662	60	1	2	3	0.02162467	0.30102999	5	2	0.68214471	0.08289318
chr5	37516662	37605145	88484	2	2	3	0.08289318	0.30102999	3	2	0.30102999	0.08289318
chr5	37605145	37605199	55	1	2	3	0.02162467	0.30102999	5	2	0.68214471	0.08289318
chr5	37605199	37648574	43376	1	2	3	0.08289318	0.30102999	3	2	0.30102999	0.08289318
chr5	37648574	37858734	210161	5	2	3	0.1575501	0.30102999	2	2	0.1575501	0.08289318
chr5	37858734	38031882	173149	1	2	3	0.30102999	0.30102999	1	2	0.05404976	0.08289318
chr5	38031882	38367928	336047	4	2	3	0.30102999	0.51676182	1	1	0.05404976	0.02438896
chr5	38367928	38464482	96555	4	2	3	0.30102999	0.30102999	1	2	0.05404976	0.08289318
chr5	38464482	38506683	42202	1	2	3	0.30102999	0.51676182	1	1	0.05404976	0.02438896
chr5	38506683	38506742	60	1	2	5	0.1575501	1.02643191	2	1	0.1575501	0.00478973
chr5	38506742	38530648	23907	1	2	4	0.1575501	0.76005302	2	1	0.1575501	0.01091641
chr5	38530648	38530707	60	1	2	4	0.08289318	0.76005302	3	1	0.30102999	0.01091641
chr5	38530707	38594140	63434	2	1	4	0.02438896	0.76005302	3	1	0.51676182	0.01091641
chr5	38594140	38812557	218418	2	1	3	0.05404976	0.51676182	2	1	0.30102999	0.02438896
chr5	38812557	39153581	341025	8	1	2	0.05404976	0.30102999	2	1	0.30102999	0.05404976
chr5	39153581	39228187	74607	2	1	2	0.05404976	0.61140001	2	0	0.30102999	0
chr5	39228187	39383383	155197	4	1	2	0.05404976	0.30102999	2	1	0.30102999	0.05404976
chr5	39383383	39480926	97544	1	1	2	0.05404976	0.61140001	2	0	0.30102999	0
chr5	39480926	39739415	258490	1	1	1	0.05404976	0.30102999	2	0	0.30102999	0
chr5	39739415	39962973	223559	2	1	1	0.05404976	0.1218695	2	1	0.30102999	0.1218695
chr5	39962973	39963032	60	1	2	1	0.1575501	0.1218695	2	1	0.1575501	0.1218695
chr5	39963032	40227465	264434	1	2	0	0.1575501	0	2	1	0.1575501	0.30102999
chr5	40227465	40308038	80574	2	2	0	0.08289318	0	3	1	0.30102999	0.30102999
chr5	40308038	40481922	173885	2	2	0	0.0429175	0	4	1	0.47744371	0.30102999
chr5	40481922	40632544	150623	2	3	1	0.10122019	0.1218695	4	1	0.30102999	0.1218695
chr5	40632544	40746633	114090	3	3	1	0.10122019	0.05404976	4	2	0.30102999	0.30102999
chr5	40746633	40833824	87192	4	3	1	0.05670724	0.05404976	5	2	0.45545077	0.30102999
chr5	40833824	40928690	94867	3	3	1	0.10122019	0.05404976	4	2	0.30102999	0.30102999
chr5	40928690	40969921	41232	2	3	2	0.10122019	0.1575501	4	2	0.30102999	0.1575501
chr5	40969921	41135465	165545	5	3	3	0.10122019	0.30102999	4	2	0.30102999	0.08289318
chr5	41135465	41213572	78108	4	3	4	0.10122019	0.30102999	4	3	0.30102999	0.10122019
chr5	41213572	41458084	244513	5	3	2	0.10122019	0.08289318	4	3	0.30102999	0.30102999
chr5	41458084	41585063	126980	4	3	3	0.05670724	0.17593012	5	3	0.45545077	0.17593012
chr5	41585063	41730846	145784	4	3	3	0.05670724	0.30102999	5	2	0.45545077	0.08289318
chr5	41730846	41842822	111977	2	3	2	0.05670724	0.1575501	5	2	0.45545077	0.1575501
chr5	41842822	41989920	147099	5	3	3	0.05670724	0.30102999	5	2	0.45545077	0.08289318
chr5	41989920	42088106	98187	1	3	2	0.10122019	0.1575501	4	2	0.30102999	0.1575501
chr5	42088106	42278512	190407	1	2	2	0.0429175	0.30102999	4	1	0.47744371	0.05404976
chr5	42278512	42429800	151289	1	2	2	0.0429175	0.61140001	4	0	0.47744371	0
chr5	42429800	42583800	154001	4	3	2	0.10122019	0.61140001	4	0	0.30102999	0
chr5	42583800	42629208	45409	2	3	3	0.05670724	0.93173516	5	0	0.45545077	0
chr5	42629208	42720939	91732	2	3	2	0.05670724	0.61140001	5	0	0.45545077	0
chr5	42720939	42720998	60	1	3	3	0.05670724	0.93173516	5	0	0.45545077	0
chr5	42720998	42746480	25483	1	2	3	0.0429175	0.93173516	4	0	0.47744371	0
chr5	42746480	42804840	58361	1	2	2	0.0429175	0.61140001	4	0	0.47744371	0
chr5	42804840	42975303	170464	4	2	2	0.02162467	0.61140001	5	0	0.68214471	0
chr5	42975303	42975362	60	1	3	3	0.03070643	0.93173516	6	0	0.63695542	0
chr5	42975362	43245321	269960	6	2	3	0.01053319	0.93173516	6	0	0.91219088	0
chr5	43245321	43532256	286936	9	2	3	0.0429175	0.93173516	4	0	0.47744371	0
chr5	43532256	43780363	248108	6	2	3	0.08289318	0.93173516	3	0	0.30102999	0
chr5	43780363	44087549	307187	4	2	4	0.08289318	1.26272838	3	0	0.30102999	0
chr5	44087549	44268534	180986	1	2	2	0.08289318	0.61140001	3	0	0.30102999	0
chr5	44268534	44268593	60	1	2	2	0.0429175	0.61140001	4	0	0.47744371	0
chr5	44268593	44310596	42004	1	2	2	0.08289318	0.61140001	3	0	0.30102999	0
chr5	44310596	44310655	60	1	3	2	0.17593012	0.61140001	3	0	0.17593012	0
chr5	44310655	44360929	50275	1	2	2	0.1575501	0.61140001	2	0	0.1575501	0
chr5	44360929	44383854	22926	1	1	2	0.05404976	0.61140001	2	0	0.30102999	0
chr5	44383854	44541440	157587	2	1	3	0.05404976	0.51676182	2	1	0.30102999	0.02438896
chr5	44541440	44716199	174760	1	1	1	0.1218695	0.1218695	1	1	0.1218695	0.1218695
chr5	44716199	44873491	157293	4	2	1	0.30102999	0.1218695	1	1	0.05404976	0.1218695
chr5	49690172	49881538	191367	3	1	1	0.30102999	0.1218695	0	1	0	0.1218695
chr5	49881538	50123913	242376	7	1	1	0.30102999	0.00478973	0	5	0	1.02643191
chr5	50123913	50531399	407487	3	0	1	0	0.00478973	0	5	0	1.02643191
chr5	50531399	50690001	158603	6	1	2	0.30102999	0.01053319	0	6	0	0.91219088
chr5	50690001	50840788	150788	1	0	2	0	0.02162467	0	5	0	0.68214471
chr5	50840788	50989500	148713	2	0	3	0	0.05670724	0	5	0	0.45545077
chr5	50989500	51426826	437327	2	0	2	0	0.0429175	0	4	0	0.47744371
chr5	51426826	51426885	60	1	0	3	0	0.05670724	1	5	0.30102999	0.45545077
chr5	51426885	51714304	287420	1	0	3	0	0.10122019	1	4	0.30102999	0.30102999
chr5	51714304	51986573	272270	2	0	5	0	0.30102999	1	4	0.30102999	0.11390336
chr5	51986573	52061544	74972	2	0	8	0	0.4250187	1	6	0.30102999	0.08122616
chr5	52061544	52338001	276458	8	0	9	0	0.5732208	1	6	0.30102999	0.04875589
chr5	52338001	52363039	25039	2	0	10	0	0.74627054	2	6	0.61140001	0.02793176
chr5	52363039	52468974	105936	3	0	9	0	0.5732208	2	6	0.61140001	0.04875589
chr5	52468974	52571472	102499	2	0	10	0	0.74627054	2	6	0.61140001	0.02793176
chr5	52571472	52699922	128451	2	0	10	0	0.74627054	3	6	0.93173516	0.02793176

chr5	52699922	52699981	60	1	0	12	0	1.17038931	3	6	0.93173516	0.00778066
chr5	52699981	52855758	155778	2	0	10	0	0.74627054	2	6	0.61140001	0.02793176
chr5	52855758	52855817	60	1	0	10	0	0.56314362	2	7	0.61140001	0.05232577
chr5	52855817	52899327	43511	1	0	10	0	0.56314362	1	7	0.30102999	0.05232577
chr5	52899327	52967429	68103	4	0	11	0	0.55623409	1	8	0.30102999	0.05490675
chr5	52967429	53092696	125268	1	0	10	0	0.56314362	1	7	0.30102999	0.05232577
chr5	53092696	53180594	87899	2	0	10	0	0.56314362	2	7	0.61140001	0.05232577
chr5	53180594	53180653	60	1	0	12	0	0.72109894	2	8	0.61140001	0.03209037
chr5	53180653	53322946	142294	4	0	11	0	0.55623409	1	8	0.30102999	0.05490675
chr5	53322946	53470931	147986	4	0	11	0	0.55623409	2	8	0.61140001	0.05490675
chr5	53470931	53470990	60	1	0	13	0	0.91308053	2	8	0.61140001	0.01767679
chr5	53470990	53692422	221433	3	0	11	0	0.55623409	2	8	0.61140001	0.05490675
chr5	53692422	53752188	59767	3	0	12	0	0.72109894	2	8	0.61140001	0.03209037
chr5	53752188	53834035	81848	1	0	11	0	0.55623409	2	8	0.61140001	0.05490675
chr5	53834035	53884916	50882	1	0	10	0	0.4167287	2	8	0.61140001	0.08923391
chr5	53884916	53884975	60	1	0	10	0	0.30102999	2	9	0.61140001	0.14135546
chr5	53884975	54274998	390024	3	0	10	0	0.30102999	1	9	0.30102999	0.14135546
chr5	54274998	54327250	52253	4	0	11	0	0.41444892	1	9	0.30102999	0.09154957
chr5	54327250	54405935	78686	2	0	10	0	0.30102999	1	9	0.30102999	0.14135546
chr5	54405935	54405993	59	1	0	11	0	0.41444892	1	9	0.30102999	0.09154957
chr5	54405993	54422851	16859	2	0	9	0	0.20975986	1	9	0.30102999	0.20975986
chr5	54422851	54453341	30491	2	0	9	0	0.30102999	1	8	0.30102999	0.13872638
chr5	54453341	54528474	75134	4	0	9	0	0.20975986	1	9	0.30102999	0.20975986
chr5	54528474	54600713	72240	4	0	10	0	0.30102999	1	9	0.30102999	0.14135546
chr5	54600713	54662700	61988	2	0	9	0	0.20975986	1	9	0.30102999	0.20975986
chr5	54662700	54763860	101161	3	0	8	0	0.13872638	1	9	0.30102999	0.30102999
chr5	54763860	54813475	49616	3	0	10	0	0.30102999	1	9	0.30102999	0.14135546
chr5	54813475	54867957	54483	1	0	9	0	0.20975986	1	9	0.30102999	0.20975986
chr5	54867957	54868016	60	1	0	10	0	0.30102999	1	9	0.30102999	0.14135546
chr5	54868016	54922296	54281	1	0	9	0	0.20975986	1	9	0.30102999	0.20975986
chr5	54922296	54922355	60	1	0	10	0	0.21118145	1	10	0.30102999	0.21118145
chr5	54922355	54965591	43237	2	0	10	0	0.30102999	1	9	0.30102999	0.14135546
chr5	54965591	55005121	39531	2	0	10	0	0.21118145	1	10	0.30102999	0.21118145
chr5	55005121	55034974	29854	1	0	8	0	0.08923391	1	10	0.30102999	0.4167287
chr5	55034974	55072833	37860	1	0	7	0	0.05232577	1	10	0.30102999	0.56314362
chr5	55072833	55072892	60	1	0	8	0	0.08923391	1	10	0.30102999	0.4167287
chr5	55072892	55150650	77759	1	0	7	0	0.08584816	1	9	0.30102999	0.42015402
chr5	55150650	55155516	4867	2	0	8	0	0.13872638	1	9	0.30102999	0.30102999
chr5	55155516	55178855	23340	1	0	7	0	0.08584816	1	9	0.30102999	0.42015402
chr5	55178855	55178914	60	1	0	8	0	0.13872638	1	9	0.30102999	0.30102999
chr5	55178914	55212864	33951	1	0	7	0	0.08584816	1	9	0.30102999	0.42015402
chr5	55212864	55259296	46433	3	0	8	0	0.08923391	1	10	0.30102999	0.4167287
chr5	55259296	55300294	40999	1	0	7	0	0.08584816	1	9	0.30102999	0.42015402
chr5	55300294	55338255	37962	2	0	8	0	0.13872638	1	9	0.30102999	0.30102999
chr5	55338255	55403185	64931	2	0	8	0	0.13872638	0	9	0	0.30102999
chr5	55403185	55407375	4191	1	0	8	0	0.20764654	0	8	0	0.20764654
chr5	55407375	55463797	56423	1	0	5	0	0.07511598	0	7	0	0.43181735
chr5	55463797	55506966	43170	1	0	3	0	0.01598258	0	7	0	0.84395715
chr5	55506966	55548789	41824	2	0	4	0	0.03812622	0	7	0	0.60763643
chr5	55548789	55548848	60	1	0	7	0	0.20469099	0	7	0	0.20469099
chr5	55548848	55613293	64446	1	0	5	0	0.07511598	0	7	0	0.43181735
chr5	55613293	55613352	60	1	0	6	0	0.129913	0	7	0	0.30102999
chr5	55613352	55822480	209129	2	0	4	0	0.03812622	0	7	0	0.60763643
chr5	55822480	55822539	60	1	0	5	0	0.04407651	0	8	0	0.58747015
chr5	55822539	55897011	74473	1	0	4	0	0.02074938	0	8	0	0.79906872
chr5	55897011	56049292	152282	1	0	3	0	0.0079614	0	8	0	1.07548421
chr5	56049292	56107595	58304	2	0	4	0	0.02074938	0	8	0	0.79906872
chr5	56107595	56142367	34773	2	0	7	0	0.13499366	0	8	0	0.30102999
chr5	56142367	56173104	30738	1	0	5	0	0.04407651	0	8	0	0.58747015
chr5	56173104	56229094	55991	2	0	3	0	0.0079614	0	8	0	1.07548421
chr5	56229094	56346762	117669	2	0	5	0	0.04407651	0	8	0	0.58747015
chr5	56346762	56346821	60	1	0	9	0	0.30102999	0	8	0	0.13872638
chr5	56346821	56538065	191245	4	0	8	0	0.30102999	0	7	0	0.13499366
chr5	56538065	56560366	22302	1	0	8	0	0.4250187	0	6	0	0.08122616
chr5	56560366	56560425	60	1	0	8	0	0.30102999	0	7	0	0.13499366
chr5	56560425	56819300	258876	2	0	6	0	0.30102999	0	5	0	0.12309572
chr5	56819300	56819359	60	1	0	8	0	0.4250187	0	6	0	0.08122616
chr5	56819359	56921560	102202	1	0	6	0	0.20064824	0	6	0	0.20064824
chr5	56921560	56921619	60	1	0	6	0	0.129913	0	7	0	0.30102999
chr5	56921619	57072959	151341	1	0	6	0	0.20064824	0	6	0	0.20064824
chr5	57072959	57073018	60	1	0	9	0	0.42015402	0	7	0	0.08584816
chr5	57073018	57367145	294128	2	0	6	0	0.20064824	0	6	0	0.20064824
chr5	57367145	57367204	60	1	0	8	0	0.20764654	0	8	0	0.20764654
chr5	57367204	57439562	72359	1	0	8	0	0.30102999	0	7	0	0.13499366
chr5	57439562	57439621	60	1	0	9	0	0.30102999	0	8	0	0.13872638
chr5	57439621	57550782	111162	1	0	8	0	0.30102999	0	7	0	0.13499366
chr5	57550782	57706809	156028	2	0	8	0	0.20764654	0	8	0	0.20764654
chr5	57706809	57753040	46232	2	0	8	0	0.08923391	0	10	0	0.4167287
chr5	57753040	57864290	111251	2	0	8	0	0.13872638	0	9	0	0.30102999
chr5	57864290	57941456	77167	1	0	7	0	0.08584816	0	9	0	0.42015402
chr5	57941456	57975607	34152	2	0	8	0	0.08923391	0	10	0	0.4167287
chr5	57975607	58019626	44020	2	0	9	0	0.09154957	0	11	0	0.41444892
chr5	58019626	58019685	60	1	0	9	0	0.0565833	0	12	0	0.55190077
chr5	58019685	58159718	140034	2	0	8	0	0.05490675	0	11	0	0.55623409
chr5	58159718	58159777	60	1	0	9	0	0.09154957	0	11	0	0.41444892
chr5	58159777	58267314	107538	1	0	9	0	0.14135546	0	10	0	0.30102999
chr5	58267314	58295609	28296	2	0	9	0	0.09154957	0	11	0	0.41444892
chr5	58295609	58438276	142668	4	0	9	0	0.0565833	0	12	0	0.55190077
chr5	58438276	58480998	42723	1	0	9	0	0.09154957	0	11	0	0.41444892
chr5	58480998	58512914	31917	2	0	10	0	0.14303407	0	11	0	0.30102999
chr5	58512914	58885517	372604	8	0	10	0	0.09290028	0	12	0	0.41314172
chr5	58885517	58962510	76994	1	0	9	0	0.0565833	0	12	0	0.55190077
chr5	58962510	58962569	60	1	0	10	0	0.09290028	0	12	0	0.41314172
chr5	58962569	58982671	20103	1	0	10	0	0.14303407	0	11	0	0.30102999
chr5	58982671	59040702	58032	2	0	9	0	0.09154957	0	11	0	0.41444892
chr5	59040702	59188595	147894	3	0	11	0	0.21200206	0	11	0	0.21200206
chr5	59188595	59251109	62515	4	0	11	0	0.14385241	0	12	0	0.30102999
chr5	59251109	59251168	60	1	0	12	0	0.21227046	0	12	0	0.21227046
chr5	59251168	59354424	103257	2	0	10	0	0.09290028	0	12	0	0.41314172
chr5	59354424	59481493	127070	1	0	10	0	0.14303407	0	11	0	0.30102999
chr5	59481493	59559993	78501	1	0	9	0	0.09154957	0	11	0	0.41444892
chr5	59559993	59560052	60	1	0	10	0	0.14303407	0	11	0	0.30102999
chr5	59560052	59836718	276667	4	0	9	0	0.09154957	0	11	0	0.41444892
chr5	59836718	59965756	129039	4	0	9	0	0.0565833	0	12	0	0.55190077
chr5	59965756	59965815	60	1	0	10	0	0.09290028	0	12	0	0.41314172
chr5	59965815	59996606	30792	1	0	9	0	0.09154957	0	11	0	0.41444892
chr5	59996606	60080096	83491	1	0	9	0	0.14135546	0	10	0	0.30102999
chr5	60080096	60080155	60	1	0	10	0	0.14303407	0	11	0	0.30102999
chr5	60080155	60169681	89527	2	0	8	0	0.08923391	0	10	0	0.4167287
chr5	60169681	60187882	18202	2	0	10	0	0.09290028	0	12	0	0.41314172
chr5	60187882	60241304	53423	2	0	8	0	0.08923391	0	10	0	0.4167287
chr5	60241304	60371469	130166	2	0	8	0	0.13872638	0	9	0	0.30102999
chr5	60371469	60506762	135294	4	0	8	0	0.08923391	0	10	0	0.4167287
chr5	60506762	60619698	112937	1	0	7	0	0.13499366	0	8	0	0.30102999
chr5	60619698	60705946	86249	1	0	6	0	0.08122616	0	8	0	0.4250187
chr5	60705946	60806393	100448	2	0	8	0	0.13872638	0	9	0	0.30102999
chr5	60806393	60806452	60	1	0	11	0	0.30102999	0	10	0	0.14303407
chr5	60806452	60839232	32781	1	0	11	0	0.41444892	0	9	0	0.09154957
chr5	60839232	61000963	161732	2	0	10	0	0.30102999	0	9	0	0.14135546
chr5	61000963	61113050	112088	2	0	12	0	0.55190077	0	9	0	0.0565833
chr5	61113050	61305129	192080	1	0	10	0	0.30102999	0	9	0	0.14135546
chr5	61305129	61484564	179436	1	0	9	0	0.20975986	0	9	0	0.20975986
chr5	61484564	61484623	60	1	0	10	0	0.30102999	0	9	0	0.14135546
chr5	61484623	61709724	225102	5	0	7	0	0.30102999	0	6	0	0.129913

chr5	61709724	61807108	97385	3	0	9	0	0.30102999	0	8	0	0.13872638
chr5	61807108	61876775	69668	2	0	8	0	0.20764654	0	8	0	0.20764654
chr5	61876775	61985998	109224	2	0	4	0	0.03812622	0	7	0	0.60763643
chr5	61985998	61986057	60	1	0	5	0	0.07511598	0	7	0	0.43181735
chr5	61986057	62167606	181550	1	0	5	0	0.12309572	0	6	0	0.30102999
chr5	62167606	62167665	60	1	0	5	0	0.07511598	0	7	0	0.43181735
chr5	62167665	62567143	399479	3	0	3	0	0.01598258	0	7	0	0.84395715
chr5	62567143	62763067	195925	2	0	7	0	0.08584816	0	9	0	0.42015402
chr5	62763067	62839696	76630	2	0	8	0	0.13872638	0	9	0	0.30102999
chr5	62839696	62839755	60	1	0	9	0	0.20975986	0	9	0	0.20975986
chr5	62839755	62904604	64850	1	0	8	0	0.20764654	0	8	0	0.20764654
chr5	62904604	63146963	242360	1	0	8	0	0.30102999	0	7	0	0.13499366
chr5	63146963	63147022	60	1	0	9	0	0.14135546	0	10	0	0.30102999
chr5	63147022	63256289	109268	1	0	9	0	0.20975986	0	9	0	0.20975986
chr5	63256289	63462871	206583	4	0	9	0	0.14135546	0	10	0	0.30102999
chr5	63462871	63564788	101918	4	0	10	0	0.09290028	0	12	0	0.41314172
chr5	63564788	63607543	42756	2	0	10	0	0.0574087	0	13	0	0.5498098
chr5	63607543	63607602	60	1	0	10	0	0.03344703	0	14	0	0.71353304
chr5	63607602	63795860	188259	4	0	10	0	0.0574087	0	13	0	0.5498098
chr5	63795860	63908578	112719	2	0	10	0	0.09290028	0	12	0	0.41314172
chr5	63908578	63908637	60	1	0	12	0	0.21227046	0	12	0	0.21227046
chr5	63908637	63976761	68125	1	0	11	0	0.14385241	0	12	0	0.30102999
chr5	63976761	64124088	147328	4	0	13	0	0.21200206	0	13	0	0.21200206
chr5	64124088	64181337	57250	2	0	14	0	0.30102999	0	13	0	0.14303407
chr5	64181337	64267604	86268	3	0	15	0	0.41444892	0	13	0	0.09154957
chr5	64267604	64267652	49	1	0	15	0	0.30102999	0	14	0	0.14135546
chr5	64267652	64402468	134817	2	0	14	0	0.30102999	0	13	0	0.14303407
chr5	64402468	64445253	42786	3	0	16	0	0.55623409	0	13	0	0.05490675
chr5	64445253	64595917	150665	2	0	14	0	0.30102999	0	13	0	0.14303407
chr5	64595917	64595974	58	1	0	16	0	0.55623409	0	13	0	0.05490675
chr5	64595974	64629923	33950	1	0	14	0	0.30102999	0	13	0	0.14303407
chr5	64629923	64883293	253371	6	0	13	0	0.30102999	0	12	0	0.14385241
chr5	64883293	64883352	60	1	0	14	0	0.30102999	0	13	0	0.14303407
chr5	64883352	64958702	75351	2	0	13	0	0.41271556	0	11	0	0.09334429
chr5	64958702	64970883	12182	3	0	14	0	0.41314172	0	12	0	0.09290028
chr5	64970883	65000171	29289	1	0	13	0	0.30102999	0	12	0	0.14385241
chr5	65000171	65271952	271782	7	0	12	0	0.21227046	0	12	0	0.21227046
chr5	65271952	65368029	96078	4	0	12	0	0.09290028	0	14	0	0.41314172
chr5	65368029	65368086	58	1	0	12	0	0.0565833	0	15	0	0.55190077
chr5	65368086	65453958	85873	2	0	12	0	0.14385241	0	13	0	0.30102999
chr5	65453958	65635983	182026	6	0	12	0	0.09290028	0	14	0	0.41314172
chr5	65635983	65756386	120404	2	0	12	0	0.14385241	0	13	0	0.30102999
chr5	65756386	65954504	198119	4	0	11	0	0.09334429	0	13	0	0.41271556
chr5	65954504	66091472	136969	2	0	9	0	0.0565833	0	12	0	0.55190077
chr5	66091472	66223106	131635	3	0	9	0	0.0331093	0	13	0	0.71538971
chr5	66223106	66287220	64115	2	0	9	0	0.09154957	0	11	0	0.41444892
chr5	66287220	66287279	60	1	0	9	0	0.0565833	0	12	0	0.55190077
chr5	66287279	66342718	55440	1	0	8	0	0.03209037	0	12	0	0.72109894
chr5	66342718	66392796	50079	2	0	9	0	0.0565833	0	12	0	0.55190077
chr5	66392796	66449372	56577	2	0	9	0	0.0331093	0	13	0	0.71538971
chr5	66449372	66449431	60	1	0	11	0	0.09334429	0	13	0	0.41271556
chr5	66449431	66492404	42974	1	0	9	0	0.0331093	0	13	0	0.71538971
chr5	66492404	66579723	87320	1	0	8	0	0.01767679	0	13	0	0.91308053
chr5	66579723	66646622	66900	2	0	9	0	0.0331093	0	13	0	0.71538971
chr5	66646622	66773116	126495	1	0	7	0	0.00859896	0	13	0	1.14735594
chr5	66773116	67015853	242738	2	0	5	0	0.00141024	0	13	0	1.7506523
chr5	67015853	67015912	60	1	0	6	0	0.00166733	0	14	0	1.70763027
chr5	67015912	67174998	159087	1	0	3	0	2.93E-04	0	12	0	2.24559516
chr5	67174998	67175057	60	1	0	4	0	0.00108487	0	12	0	1.81706455
chr5	67175057	67320572	145516	1	0	2	0	9.54E-04	0	9	0	1.74076927
chr5	67320572	67444100	123529	1	0	2	0	0.00221948	0	8	0	1.44210395
chr5	67444100	67508101	64002	1	0	2	0	0.00493743	0	7	0	1.16581773
chr5	67508101	67641500	133400	5	0	2	0	0.00221948	0	8	0	1.44210395
chr5	67641500	67641559	60	1	0	2	0	3.90E-04	0	10	0	2.0620585
chr5	67641559	67914183	272625	1	0	2	0	0.00221948	0	8	0	1.44210395
chr5	67914183	68167910	253728	3	0	2	0	9.54E-04	0	9	0	1.74076927
chr5	68167910	68425519	257610	4	0	2	0	0.00221948	0	8	0	1.44210395
chr5	68425519	68520433	94915	5	0	2	0	0.00493743	0	7	0	1.16581773
chr5	68520433	68791331	270899	9	0	1	0	0.02438896	0	3	0	0.51676182
chr5	68791331	70748823	1957493	1	0	0	0	0	0	2	0	0.61140001
chr5	70748823	70895484	146662	5	0	5	0	0.07511598	0	7	0	0.43181735
chr5	70895484	71298356	402873	7	0	8	0	0.13872638	0	9	0	0.30102999
chr5	71298356	71436565	138210	3	0	9	0	0.0565833	0	12	0	0.55190077
chr5	71436565	71460780	24216	2	0	13	0	0.21200206	0	13	0	0.21200206
chr5	71460780	71502802	42023	1	0	12	0	0.14385241	0	13	0	0.30102999
chr5	71502802	71515732	12931	2	0	12	0	0.09290028	0	14	0	0.41314172
chr5	71515732	71699601	183870	5	0	10	0	0.03344703	0	14	0	0.71353304
chr5	71699601	71886668	187068	4	0	12	0	0.09290028	0	14	0	0.41314172
chr5	71886668	71886727	60	1	0	13	0	0.05490675	0	16	0	0.55623409
chr5	71886727	72115386	228660	2	0	13	0	0.14303407	0	14	0	0.30102999
chr5	72115386	72115445	60	1	0	15	0	0.13872638	0	16	0	0.30102999
chr5	72115445	72161456	46012	1	0	14	0	0.14135546	0	15	0	0.30102999
chr5	72161456	72204364	42909	1	0	13	0	0.09154957	0	15	0	0.41444892
chr5	72204364	72204423	60	1	0	13	0	0.05490675	0	16	0	0.55623409
chr5	72204423	72243184	38762	1	0	12	0	0.03209037	0	16	0	0.72109894
chr5	72243184	72288525	45342	1	0	12	0	0.09290028	0	14	0	0.41314172
chr5	72288525	72288584	60	1	0	13	0	0.14303407	0	14	0	0.30102999
chr5	72288584	72337791	49208	1	0	12	0	0.14385241	0	13	0	0.30102999
chr5	72337791	72417501	79711	3	0	12	0	0.09290028	0	14	0	0.41314172
chr5	72417501	72427454	9954	3	0	12	0	0.0565833	0	15	0	0.55190077
chr5	72427454	72470617	43164	2	0	11	0	0.0331093	0	15	0	0.71538971
chr5	72470617	72470676	60	1	0	12	0	0.0565833	0	15	0	0.55190077
chr5	72470676	72527647	56972	1	0	11	0	0.0331093	0	15	0	0.71538971
chr5	72527647	72527706	60	1	0	12	0	0.0565833	0	15	0	0.55190077
chr5	72527706	72660687	132982	1	0	11	0	0.09334429	0	13	0	0.41271556
chr5	72660687	72742476	81790	2	0	10	0	0.0574087	0	13	0	0.5498098
chr5	72742476	72987520	245045	7	0	8	0	0.01767679	0	13	0	0.91308053
chr5	72987520	72987579	60	1	0	10	0	0.0574087	0	13	0	0.5498098
chr5	72987579	73044400	56822	1	0	8	0	0.01767679	0	13	0	0.91308053
chr5	73044400	73084686	40287	1	0	8	0	0.03209037	0	12	0	0.72109894
chr5	73084686	73158692	74007	2	0	10	0	0.09290028	0	12	0	0.41314172
chr5	73158692	73158751	60	1	0	10	0	0.0574087	0	13	0	0.5498098
chr5	73158751	73201951	43201	1	0	10	0	0.09290028	0	12	0	0.41314172
chr5	73201951	73237181	35231	2	0	11	0	0.14385241	0	12	0	0.30102999
chr5	73237181	73285938	48758	1	0	11	0	0.21200206	0	11	0	0.21200206
chr5	73285938	73470360	184423	1	0	9	0	0.14135546	0	10	0	0.30102999
chr5	73470360	73600171	129812	2	0	9	0	0.09154957	0	11	0	0.41444892
chr5	73600171	73708380	108210	2	0	11	0	0.14385241	0	12	0	0.30102999
chr5	73708380	73708439	60	1	0	13	0	0.14303407	0	14	0	0.30102999
chr5	73708439	73794436	85998	1	0	12	0	0.21227046	0	12	0	0.21227046
chr5	73794436	73924114	129679	1	0	11	0	0.14385241	0	12	0	0.30102999
chr5	73924114	73985153	61040	2	0	12	0	0.21227046	0	12	0	0.21227046
chr5	73985153	73985211	59	1	0	14	0	0.30102999	0	13	0	0.14303407
chr5	73985211	74054704	69494	3	0	13	0	0.21200206	0	13	0	0.21200206
chr5	74054704	74119791	65088	4	0	13	0	0.14303407	0	14	0	0.30102999
chr5	74119791	74119850	60	1	0	14	0	0.14135546	0	15	0	0.30102999
chr5	74119850	74173584	53735	1	0	14	0	0.21118145	0	14	0	0.21118145
chr5	74173584	74241701	68118	1	0	13	0	0.21200206	0	13	0	0.21200206
chr5	74241701	74241760	60	1	0	14	0	0.30102999	0	13	0	0.14303407
chr5	74241760	74407784	166025	3	0	12	0	0.21227046	0	12	0	0.21227046
chr5	74407784	74407843	60	1	0	14	0	0.41314172	0	12	0	0.09290028
chr5	74407843	74484578	76736	1	0	13	0	0.30102999	0	12	0	0.14385241
chr5	74484578	74510646	26069	1	0	12	0	0.21227046	0	12	0	0.21227046
chr5	74510646	74510705	60	1	0	13	0	0.30102999	0	12	0	0.14385241

chr5	74510705	74632161	121457	1	0	12	0	0.21227046	0	12	0	0.21227046
chr5	74632161	74706843	74683	5	0	12	0	0.14385241	0	13	0	0.30102999
chr5	74706843	74706902	60	1	0	14	0	0.14135546	0	15	0	0.30102999
chr5	74706902	74772552	65651	1	0	13	0	0.14303407	0	14	0	0.30102999
chr5	74772552	74772611	60	1	0	13	0	0.09154957	0	15	0	0.41444892
chr5	74772611	74822073	49463	1	0	12	0	0.0565833	0	15	0	0.55190077
chr5	74822073	74973724	151652	5	0	13	0	0.09154957	0	15	0	0.41444892
chr5	74973724	74973782	59	1	0	15	0	0.13872638	0	16	0	0.30102999
chr5	74973782	75003621	29840	2	0	14	0	0.08923391	0	16	0	0.4167287
chr5	75003621	75003680	60	1	0	15	0	0.13872638	0	16	0	0.30102999
chr5	75003680	75033997	30318	1	0	14	0	0.14135546	0	15	0	0.30102999
chr5	75033997	75141221	107225	2	0	15	0	0.20975986	0	15	0	0.20975986
chr5	75141221	75141280	60	1	0	15	0	0.13872638	0	16	0	0.30102999
chr5	75141280	75271248	129969	1	0	14	0	0.08923391	0	16	0	0.4167287
chr5	75271248	75389702	118455	1	0	14	0	0.14135546	0	15	0	0.30102999
chr5	75389702	75389761	60	1	0	15	0	0.13872638	0	16	0	0.30102999
chr5	75389761	75494672	104912	2	0	14	0	0.14135546	0	15	0	0.30102999
chr5	75494672	75626399	131728	2	0	12	0	0.09290028	0	14	0	0.41314172
chr5	75626399	75626458	60	1	0	14	0	0.21118145	0	14	0	0.21118145
chr5	75626458	75720002	93545	2	0	10	0	0.0574087	0	13	0	0.5498098
chr5	75720002	75757455	37454	2	0	11	0	0.0574087	0	14	0	0.5498098
chr5	75757455	75788687	31233	2	0	13	0	0.09154957	0	15	0	0.41444892
chr5	75788687	75788746	60	1	0	14	0	0.14135546	0	15	0	0.30102999
chr5	75788746	75852895	64150	1	0	14	0	0.21118145	0	14	0	0.21118145
chr5	75852895	75911769	58875	1	0	12	0	0.09290028	0	14	0	0.41314172
chr5	75911769	75946977	35209	4	0	12	0	0.0565833	0	15	0	0.55190077
chr5	75946977	76003175	56199	1	0	10	0	0.01817691	0	15	0	0.90719478
chr5	76003175	76169880	166706	5	0	10	0	0.0574087	0	13	0	0.5498098
chr5	76169880	76329022	159143	6	0	10	0	0.03344703	0	14	0	0.71353304
chr5	76329022	76504233	175212	4	0	10	0	0.01817691	0	15	0	0.90719478
chr5	76504233	76525095	20863	2	0	13	0	0.09154957	0	15	0	0.41444892
chr5	76525095	76525154	60	1	0	14	0	0.14135546	0	15	0	0.30102999
chr5	76525154	76605107	79954	2	0	13	0	0.09154957	0	15	0	0.41444892
chr5	76605107	76651897	46791	2	0	14	0	0.08923391	0	16	0	0.4167287
chr5	76651897	76651956	60	1	0	15	0	0.13872638	0	16	0	0.30102999
chr5	76651956	76706606	54651	2	0	14	0	0.08923391	0	16	0	0.4167287
chr5	76706606	76748249	41644	2	0	13	0	0.05490675	0	16	0	0.55623409
chr5	76748249	76784414	36166	2	0	13	0	0.14303407	0	14	0	0.30102999
chr5	76784414	77021060	236647	4	0	13	0	0.21200206	0	13	0	0.21200206
chr5	77021060	77072493	51434	2	0	13	0	0.14303407	0	14	0	0.30102999
chr5	77072493	77072542	50	1	0	14	0	0.21118145	0	14	0	0.21118145
chr5	77072542	77523319	450778	8	0	14	0	0.30102999	0	13	0	0.14303407
chr5	77523319	77635231	111913	3	0	15	0	0.41444892	0	13	0	0.09154957
chr5	77635231	77658239	23009	2	0	15	0	0.30102999	0	14	0	0.14135546
chr5	77658239	77684699	26461	2	0	16	0	0.20764654	0	16	0	0.20764654
chr5	77684699	77715374	30676	1	0	15	0	0.20975986	0	15	0	0.20975986
chr5	77715374	77755098	39725	1	0	14	0	0.21118145	0	14	0	0.21118145
chr5	77755098	77786678	31581	1	0	13	0	0.21200206	0	13	0	0.21200206
chr5	77786678	77786737	60	1	0	13	0	0.14303407	0	14	0	0.30102999
chr5	77786737	77833478	46742	2	0	13	0	0.30102999	0	12	0	0.14385241
chr5	77833478	77833537	60	1	0	13	0	0.14303407	0	14	0	0.30102999
chr5	77833537	77900667	67131	2	0	12	0	0.21227046	0	12	0	0.21227046
chr5	77900667	77900726	60	1	0	12	0	0.09290028	0	14	0	0.41314172
chr5	77900726	77977430	76705	1	0	12	0	0.21227046	0	12	0	0.21227046
chr5	77977430	78075795	98366	1	0	11	0	0.30102999	0	10	0	0.14303407
chr5	78075795	78111599	35805	2	0	11	0	0.21200206	0	11	0	0.21200206
chr5	78111599	78199103	87505	3	0	12	0	0.09290028	0	14	0	0.41314172
chr5	78199103	78234294	35192	2	0	14	0	0.21118145	0	14	0	0.21118145
chr5	78234294	78280105	45812	1	0	13	0	0.21200206	0	13	0	0.21200206
chr5	78280105	78317194	37090	1	0	13	0	0.41271556	0	11	0	0.09334429
chr5	78317194	78368121	50928	1	0	13	0	0.5498098	0	10	0	0.0574087
chr5	78368121	78411563	43443	3	0	14	0	0.5498098	0	11	0	0.0574087
chr5	78411563	78411617	55	1	0	14	0	0.30102999	0	13	0	0.14303407
chr5	78411617	78559013	147397	3	0	11	0	0.09334429	0	13	0	0.41271556
chr5	78559013	78617472	58460	2	0	10	0	0.0574087	0	13	0	0.5498098
chr5	78617472	78617531	60	1	0	12	0	0.0565833	0	15	0	0.55190077
chr5	78617531	78671747	54217	1	0	11	0	0.09334429	0	13	0	0.41271556
chr5	78671747	78671806	60	1	0	11	0	0.0574087	0	14	0	0.5498098
chr5	78671806	78693347	21542	1	0	9	0	0.01817691	0	14	0	0.90719478
chr5	78693347	78789518	96172	4	0	10	0	0.03344703	0	14	0	0.71353304
chr5	78789518	78831700	42183	2	0	14	0	0.08923391	0	16	0	0.4167287
chr5	78831700	78973533	141834	4	0	11	0	0.0574087	0	14	0	0.5498098
chr5	78973533	78973592	60	1	0	14	0	0.14135546	0	15	0	0.30102999
chr5	78973592	79034055	60464	1	0	12	0	0.09290028	0	14	0	0.41314172
chr5	79034055	79034114	60	1	0	14	0	0.21118145	0	14	0	0.21118145
chr5	79034114	79084822	50709	2	0	12	0	0.09290028	0	14	0	0.41314172
chr5	79084822	79084879	58	1	0	13	0	0.14303407	0	14	0	0.30102999
chr5	79084879	79254138	169260	2	0	11	0	0.09334429	0	13	0	0.41271556
chr5	79254138	79336096	81959	2	0	10	0	0.14303407	0	11	0	0.30102999
chr5	79336096	79411777	75682	2	0	8	0	0.05490675	0	11	0	0.55623409
chr5	79411777	79507683	95907	3	0	9	0	0.09154957	0	11	0	0.41444892
chr5	79507683	79539194	31512	2	0	9	0	0.0565833	0	12	0	0.55190077
chr5	79539194	79539253	60	1	0	11	0	0.09334429	0	13	0	0.41271556
chr5	79539253	79578155	38903	1	0	10	0	0.0574087	0	13	0	0.5498098
chr5	79578155	79616014	37860	2	0	10	0	0.03344703	0	14	0	0.71353304
chr5	79616014	79678634	62621	1	0	10	0	0.0574087	0	13	0	0.5498098
chr5	79678634	79736096	57463	1	0	9	0	0.0331093	0	13	0	0.71538971
chr5	79736096	79775071	38976	3	0	9	0	0.01817691	0	14	0	0.90719478
chr5	79775071	79844193	69123	3	0	10	0	0.01817691	0	15	0	0.90719478
chr5	79844193	79844252	60	1	0	11	0	0.0331093	0	15	0	0.71538971
chr5	79844252	79933552	89301	1	0	10	0	0.01817691	0	15	0	0.90719478
chr5	79933552	79951089	17538	2	0	11	0	0.0331093	0	15	0	0.71538971
chr5	79951089	80021305	70217	2	0	13	0	0.05490675	0	16	0	0.55623409
chr5	80021305	80021364	60	1	0	14	0	0.08923391	0	16	0	0.4167287
chr5	80021364	80057447	36084	1	0	12	0	0.03209037	0	16	0	0.72109894
chr5	80057447	80169014	111568	2	0	11	0	0.0331093	0	15	0	0.71538971
chr5	80169014	80254203	85190	1	0	10	0	0.01817691	0	15	0	0.90719478
chr5	80254203	80366805	112603	2	0	8	0	0.00433294	0	15	0	1.3873209
chr5	80366805	80366864	60	1	0	10	0	0.01817691	0	15	0	0.90719478
chr5	80366864	80469317	102454	2	0	9	0	0.00926608	0	15	0	1.13004284
chr5	80469317	80469376	60	1	0	10	0	0.01817691	0	15	0	0.90719478
chr5	80469376	80608340	138965	4	0	10	0	0.03344703	0	14	0	0.71353304
chr5	80608340	80868550	260211	6	0	9	0	0.01817691	0	14	0	0.90719478
chr5	80868550	80899450	30901	2	0	10	0	0.03344703	0	14	0	0.71353304
chr5	80899450	80993322	93873	2	0	8	0	0.01767679	0	13	0	0.91308053
chr5	80993322	81027222	33901	2	0	9	0	0.01817691	0	14	0	0.90719478
chr5	81027222	81054919	27698	3	0	10	0	0.03344703	0	14	0	0.71353304
chr5	81054919	81165326	110408	1	0	7	0	0.00859896	0	13	0	1.14735594
chr5	81165326	81264022	98697	1	0	6	0	0.00373523	0	13	0	1.42397267
chr5	81264022	81318891	54870	3	0	6	0	0.00166733	0	14	0	1.70763027
chr5	81318891	81354382	35492	2	0	9	0	0.01817691	0	14	0	0.90719478
chr5	81354382	81413270	58889	1	0	8	0	0.00909834	0	14	0	1.13428373
chr5	81413270	81460235	46966	2	0	8	0	0.00433294	0	15	0	1.3873209
chr5	81460235	81460289	55	1	0	9	0	0.00926608	0	15	0	1.13004284
chr5	81460289	81520566	60278	1	0	8	0	0.00433294	0	15	0	1.3873209
chr5	81520566	81520625	60	1	0	10	0	0.01817691	0	15	0	0.90719478
chr5	81520625	81548472	27848	1	0	10	0	0.03344703	0	14	0	0.71353304
chr5	81548472	81573682	25211	1	0	10	0	0.0574087	0	13	0	0.5498098
chr5	81573682	81573741	60	1	0	11	0	0.0574087	0	14	0	0.5498098
chr5	81573741	81647728	73988	2	0	10	0	0.0574087	0	13	0	0.5498098
chr5	81647728	81714588	66861	1	0	9	0	0.0331093	0	13	0	0.71538971
chr5	81714588	81838069	123482	1	0	9	0	0.0565833	0	12	0	0.55190077
chr5	81838069	81838128	60	1	0	10	0	0.09290028	0	12	0	0.41314172
chr5	81838128	81997374	159247	1	0	9	0	0.0565833	0	12	0	0.55190077

chr5	81997374	82088238	90865	2	0	10	0	0.09290028	0	12	0	0.41314172
chr5	82088238	82088297	60	1	0	13	0	0.09154957	0	15	0	0.41444892
chr5	82088297	82317796	229500	1	0	13	0	0.14303407	0	14	0	0.30102999
chr5	82317796	82317855	60	1	0	14	0	0.21118145	0	14	0	0.21118145
chr5	82317855	82360571	42717	1	0	14	0	0.30102999	0	13	0	0.14303407
chr5	82360571	82367584	7014	2	0	14	0	0.21118145	0	14	0	0.21118145
chr5	82367584	82472836	105253	3	0	14	0	0.14135546	0	15	0	0.30102999
chr5	82472836	82506382	33547	2	0	15	0	0.20975986	0	15	0	0.20975986
chr5	82506382	82506441	60	1	0	17	0	0.42015402	0	15	0	0.08584816
chr5	82506441	82600225	93785	3	0	17	0	0.56314362	0	14	0	0.05232577
chr5	82600225	82648926	48702	1	0	15	0	0.30102999	0	14	0	0.14135546
chr5	82648926	82648985	60	1	0	16	0	0.4167287	0	14	0	0.08923391
chr5	82648985	82744321	95337	1	0	16	0	0.55623409	0	13	0	0.05490675
chr5	82744321	82779349	35029	1	0	15	0	0.41444892	0	13	0	0.09154957
chr5	82779349	82835213	55865	3	0	15	0	0.30102999	0	14	0	0.14135546
chr5	82835213	82995331	160119	5	0	15	0	0.41444892	0	13	0	0.09154957
chr5	82995331	83244757	249427	4	0	14	0	0.30102999	0	13	0	0.14303407
chr5	83244757	83356252	111496	2	0	13	0	0.21200206	0	13	0	0.21200206
chr5	83356252	83356309	58	1	0	15	0	0.30102999	0	14	0	0.14135546
chr5	83356309	83481832	125524	3	0	12	0	0.14385241	0	13	0	0.30102999
chr5	83481832	83481891	60	1	0	13	0	0.21200206	0	13	0	0.21200206
chr5	83481891	83561183	79293	1	0	12	0	0.14385241	0	13	0	0.30102999
chr5	83561183	83638398	77216	2	0	8	0	0.05490675	0	11	0	0.55623409
chr5	83638398	83638457	60	1	0	13	0	0.21200206	0	13	0	0.21200206
chr5	83638457	83769645	131189	2	0	8	0	0.03209037	0	12	0	0.72109894
chr5	83769645	83924954	155310	1	0	7	0	0.03037338	0	11	0	0.73110763
chr5	83924954	83925013	60	1	0	10	0	0.14303407	0	11	0	0.30102999
chr5	83925013	84194538	269526	1	0	6	0	0.20064824	0	6	0	0.20064824
chr5	84194538	84299608	105071	2	0	7	0	0.30102999	0	6	0	0.129913
chr5	84299608	84349709	50102	2	0	8	0	0.4250187	0	6	0	0.08122616
chr5	84819841	84984575	164735	3	0	3	0	0.51676182	0	1	0	0.02438896
chr5	84984575	84984634	60	1	0	11	0	0.41444892	0	9	0	0.09154957
chr5	84984634	85199271	214638	1	0	10	0	0.30102999	0	9	0	0.14135546
chr5	85199271	85199330	60	1	0	12	0	0.55190077	0	9	0	0.0565833
chr5	85199330	85381873	182544	1	0	11	0	0.41444892	0	9	0	0.09154957
chr5	85381873	85381932	60	1	0	11	0	0.30102999	0	10	0	0.14303407
chr5	85381932	85624656	242725	2	0	10	0	0.30102999	0	9	0	0.14135546
chr5	85624656	85712443	87788	2	0	11	0	0.30102999	0	10	0	0.14303407
chr5	85712443	85712502	60	1	0	14	0	0.21118145	0	14	0	0.21118145
chr5	85712502	85954487	241986	4	0	13	0	0.21200206	0	13	0	0.21200206
chr5	85954487	86254118	299632	2	0	13	0	0.30102999	0	12	0	0.14385241
chr5	86254118	86377115	122998	2	0	14	0	0.30102999	0	13	0	0.14303407
chr5	86377115	86487774	110660	1	0	13	0	0.21200206	0	13	0	0.21200206
chr5	86487774	86519499	31726	1	0	10	0	0.09290028	0	12	0	0.41314172
chr5	86519499	86523177	3679	2	0	11	0	0.14385241	0	12	0	0.30102999
chr5	86523177	86682676	159500	4	0	9	0	0.0565833	0	12	0	0.55190077
chr5	86682676	86718879	36204	4	0	8	0	0.05490675	0	11	0	0.55623409
chr5	86718879	86904001	185123	3	0	9	0	0.09154957	0	11	0	0.41444892
chr5	86904001	86904060	60	1	0	9	0	0.0565833	0	12	0	0.55190077
chr5	86904060	87050542	146483	1	0	7	0	0.03037338	0	11	0	0.73110763
chr5	87050542	87249953	199412	2	0	7	0	0.01667721	0	12	0	0.92532268
chr5	87249953	87250012	60	1	0	12	0	0.09290028	0	14	0	0.41314172
chr5	87250012	87615824	365813	4	0	10	0	0.0574087	0	13	0	0.5498098
chr5	87615824	87686865	71042	1	0	9	0	0.0331093	0	13	0	0.71538971
chr5	87686865	87686924	60	1	0	10	0	0.0574087	0	13	0	0.5498098
chr5	87686924	87734527	47604	1	0	5	0	0.00666883	0	11	0	1.20557689
chr5	87734527	87928008	193482	1	0	4	0	0.00247414	0	11	0	1.52371709
chr5	87928008	88016273	88266	4	0	4	0	0.00108487	0	12	0	1.81706455
chr5	88016273	88078676	62404	1	0	4	0	0.00247414	0	11	0	1.52371709
chr5	88078676	88232646	153971	5	0	5	0	0.00666883	0	11	0	1.20557689
chr5	88232646	88348206	115561	1	0	5	0	0.04407651	0	8	0	0.58747015
chr5	88348206	88450293	102088	1	0	4	0	0.02074938	0	8	0	0.79906872
chr5	88450293	88593277	142985	2	0	4	0	0.01077081	0	9	0	1.01542894
chr5	88593277	88593336	60	1	0	9	0	0.14135546	0	10	0	0.30102999
chr5	88593336	88860218	266883	2	0	3	0	0.00378107	0	9	0	1.33111237
chr5	88860218	88860277	60	1	0	8	0	0.08923391	0	10	0	0.4167287
chr5	88860277	89028093	167817	1	0	8	0	0.30102999	0	7	0	0.13499366
chr5	89028093	89253207	225115	1	0	6	0	0.129913	0	7	0	0.30102999
chr5	89253207	89253266	60	1	0	7	0	0.13499366	0	8	0	0.30102999
chr5	89253266	89500084	246819	2	0	6	0	0.129913	0	7	0	0.30102999
chr5	89500084	89689882	189799	1	0	5	0	0.12309572	0	6	0	0.30102999
chr5	89689882	89734050	44169	3	0	5	0	0.07511598	0	7	0	0.43181735
chr5	89734050	89734109	60	1	0	7	0	0.13499366	0	8	0	0.30102999
chr5	89734109	90077119	343011	10	0	7	0	0.20469099	0	7	0	0.20469099
chr5	90077119	90281252	204134	5	0	5	0	0.07511598	0	7	0	0.43181735
chr5	90281252	90321595	40344	2	0	7	0	0.13499366	0	8	0	0.30102999
chr5	90321595	90460776	139182	4	0	7	0	0.20469099	0	7	0	0.20469099
chr5	90460776	90677117	216342	3	0	4	0	0.18734596	0	4	0	0.18734596
chr5	90677117	90752413	75297	2	0	3	0	0.30102999	0	2	0	0.08289318
chr5	90752413	90904045	151633	1	0	2	0	0.1575501	0	2	0	0.1575501
chr5	90904045	90904104	60	1	0	4	0	0.18734596	0	4	0	0.18734596
chr5	90904104	91086399	182296	1	0	0	0	0	0	3	0	0.93173516
chr5	91086399	91086458	60	1	0	0	0	0	0	4	0	1.26272838
chr5	91086458	91327086	240629	1	0	0	0	0	0	2	0	0.61140001
chr5	91327086	91327145	60	1	0	1	0	0.05404976	0	2	0	0.30102999
chr5	91327145	91542432	215288	2	0	0	0	0	0	1	0	0.30102999
chr5	91542432	91542491	60	1	0	0	0	0	0	2	0	0.61140001
chr5	91542491	91705071	162581	1	0	0	0	0	0	1	0	0.30102999
chr5	91705071	91705130	60	1	0	1	0	0.05404976	0	2	0	0.30102999
chr5	91705130	91981946	276817	3	0	1	0	0.30102999	0	0	0	0
chr5	91981946	91982005	60	1	1	6	0.30102999	1.31360226	0	1	0	0.00204627
chr5	91982005	92105563	123559	1	0	6	0	1.31360226	0	1	0	0.00204627
chr5	92105563	92235441	129879	2	0	7	0	1.16581773	0	2	0	0.00493743
chr5	92235441	92316026	80586	2	0	8	0	1.07548421	0	3	0	0.0079614
chr5	92316026	92316085	60	1	0	8	0	0.20764654	0	8	0	0.20764654
chr5	92316085	92528810	212726	1	0	7	0	0.20469099	0	7	0	0.20469099
chr5	92528810	92661284	132475	2	0	8	0	0.30102999	0	7	0	0.13499366
chr5	92661284	92661343	60	1	0	13	0	0.5498098	0	10	0	0.0574087
chr5	92661343	92740716	79374	1	0	12	0	0.55190077	0	9	0	0.0565833
chr5	92740716	92740775	60	1	0	13	0	0.5498098	0	10	0	0.0574087
chr5	92740775	92806839	66065	1	0	13	0	0.71538971	0	9	0	0.0331093
chr5	92806839	92856328	49490	2	0	14	0	0.90719478	0	9	0	0.01817691
chr5	92856328	92922775	66448	1	0	13	0	0.71538971	0	9	0	0.0331093
chr5	92922775	92925352	2578	2	0	14	0	0.71353304	0	10	0	0.03344703
chr5	92925352	93016278	90927	2	0	14	0	0.90719478	0	9	0	0.01817691
chr5	93016278	93016337	60	1	0	15	0	1.13004284	0	9	0	0.00926608
chr5	93016337	93076070	59734	1	0	14	0	0.90719478	0	9	0	0.01817691
chr5	93076070	93076129	60	1	0	14	0	0.5498098	0	11	0	0.0574087
chr5	93076129	93198058	121930	2	0	14	0	0.90719478	0	9	0	0.01817691
chr5	93198058	93233473	35416	1	0	13	0	0.71538971	0	9	0	0.0331093
chr5	93233473	93294580	61108	3	0	13	0	0.91308053	0	8	0	0.01767679
chr5	93294580	93323162	28583	2	0	14	0	0.41314172	0	12	0	0.09290028
chr5	93323162	93424409	101248	2	0	12	0	0.72109894	0	8	0	0.03209037
chr5	93424409	93424468	60	1	0	14	0	0.30102999	0	13	0	0.14303407
chr5	93424468	93582960	158493	3	0	14	0	0.41314172	0	12	0	0.09290028
chr5	93582960	93789795	206836	6	0	15	0	0.41444892	0	13	0	0.09154957
chr5	93789795	93859253	69459	1	0	14	0	0.41314172	0	12	0	0.09290028
chr5	93859253	93953135	93883	3	0	14	0	0.5498098	0	11	0	0.0574087
chr5	93953135	94014622	61488	1	0	13	0	0.41271556	0	11	0	0.09334429
chr5	94014622	94043256	28635	2	0	13	0	0.30102999	0	12	0	0.14385241
chr5	94043256	94114792	71537	1	0	13	0	0.41271556	0	11	0	0.09334429
chr5	94114792	94146497	31706	2	0	14	0	0.5498098	0	11	0	0.0574087
chr5	94146497	94208884	62388	1	0	12	0	0.30102999	0	11	0	0.14385241
chr5	94208884	94288903	80020	2	0	11	0	0.21200206	0	11	0	0.21200206

chr5	94288903	94288962	60	1	0	14	0	0.5498098	0	11	0	0.0574087
chr5	94288962	94316727	27766	1	0	13	0	0.41271556	0	11	0	0.09334429
chr5	94316727	94373819	57093	1	0	12	0	0.30102999	0	11	0	0.14385241
chr5	94373819	94478206	104388	2	0	10	0	0.21118145	0	10	0	0.21118145
chr5	94478206	94528574	50369	2	0	11	0	0.21200206	0	11	0	0.21200206
chr5	94528574	94528633	60	1	0	11	0	0.14385241	0	12	0	0.30102999
chr5	94528633	94683232	154600	3	0	10	0	0.09290028	0	12	0	0.41314172
chr5	94683232	94683291	60	1	0	11	0	0.14385241	0	12	0	0.30102999
chr5	94683291	94800050	116760	2	0	9	0	0.09154957	0	11	0	0.41444892
chr5	94800050	94856474	56425	2	0	9	0	0.0331093	0	13	0	0.71538971
chr5	94856474	94927175	70702	2	0	9	0	0.0565833	0	12	0	0.55190077
chr5	94927175	95072617	145443	3	0	8	0	0.08923391	0	10	0	0.4167287
chr5	95072617	95152468	79852	3	0	9	0	0.14135546	0	10	0	0.30102999
chr5	95152468	95188548	36081	2	0	9	0	0.09154957	0	11	0	0.41444892
chr5	95188548	95188603	56	1	0	11	0	0.14385241	0	12	0	0.30102999
chr5	95188603	95275711	87109	2	0	11	0	0.21200206	0	11	0	0.21200206
chr5	95275711	95275770	60	1	0	11	0	0.14385241	0	12	0	0.30102999
chr5	95275770	95353621	77852	2	0	10	0	0.21118145	0	10	0	0.21118145
chr5	95353621	95353680	60	1	0	11	0	0.14385241	0	12	0	0.30102999
chr5	95353680	95469240	115561	3	0	10	0	0.14303407	0	11	0	0.30102999
chr5	95469240	95580933	111694	1	0	10	0	0.21118145	0	10	0	0.21118145
chr5	95580933	95655412	74480	2	0	12	0	0.21227046	0	12	0	0.21227046
chr5	95655412	95821236	165825	2	0	9	0	0.20975986	0	9	0	0.20975986
chr5	95821236	96031510	210275	2	0	8	0	0.13872638	0	9	0	0.30102999
chr5	96031510	96110300	78791	3	0	9	0	0.14135546	0	10	0	0.30102999
chr5	96110300	96181765	71466	2	0	7	0	0.13499366	0	8	0	0.30102999
chr5	96181765	96212200	30436	1	0	7	0	0.30102999	0	6	0	0.129913
chr5	96212200	96212257	58	1	0	8	0	0.4250187	0	6	0	0.08122616
chr5	96212257	96273324	61068	3	0	7	0	0.43181735	0	5	0	0.07511598
chr5	96273324	96339119	65796	1	0	7	0	0.60763643	0	4	0	0.03812622
chr5	96339119	96339178	60	1	0	8	0	0.79906872	0	4	0	0.02074938
chr5	96339178	96427846	88669	2	0	7	0	1.16581773	0	2	0	0.00493743
chr5	96427846	96577967	150122	5	0	5	0	0.68214471	0	2	0	0.02162467
chr5	96577967	96578026	60	1	0	7	0	1.16581773	0	2	0	0.00493743
chr5	96578026	96762669	184644	2	0	6	0	0.91219088	0	2	0	0.01053319
chr5	96762669	96762728	60	1	0	7	0	1.16581773	0	2	0	0.00493743
chr5	96762728	96899081	136354	1	0	6	0	0.91219088	0	2	0	0.01053319
chr5	96899081	96899140	60	1	0	7	0	1.16581773	0	2	0	0.00493743
chr5	96899140	97051910	152771	1	0	6	0	0.91219088	0	2	0	0.01053319
chr5	97051910	97051969	60	1	0	7	0	1.16581773	0	2	0	0.00493743
chr5	97051969	97310976	259008	2	0	6	0	0.91219088	0	2	0	0.01053319
chr5	97310976	97311035	60	1	0	9	0	1.33111237	0	3	0	0.00378107
chr5	97311035	97555591	244557	1	0	9	0	1.74076927	0	2	0	9.54E-04
chr5	97555591	97663374	107784	2	0	9	0	0.5732208	0	6	0	0.04875589
chr5	97663374	97663433	60	1	0	14	0	1.70763027	0	6	0	0.00166733
chr5	97663433	97705034	41602	1	0	9	0	1.01542894	0	4	0	0.01077081
chr5	97705034	97790274	85241	1	0	8	0	0.79906872	0	4	0	0.02074938
chr5	97790274	97968817	178544	2	0	9	0	1.01542894	0	4	0	0.01077081
chr5	97968817	97968876	60	1	0	10	0	1.2568129	0	4	0	0.00530919
chr5	97968876	98191915	223040	3	0	9	0	1.01542894	0	4	0	0.01077081
chr5	98191915	98217825	25911	2	0	12	0	1.17038931	0	6	0	0.00778066
chr5	98217825	98390918	173094	2	0	12	0	1.81706455	0	4	0	0.00108487
chr5	98390918	98390977	60	1	0	12	0	0.92532268	0	7	0	0.01667721
chr5	98390977	98584007	193031	1	0	11	0	0.9449689	0	6	0	0.01518174
chr5	98584007	98584066	60	1	0	13	0	1.42397267	0	6	0	0.00373523
chr5	98584066	98775350	191285	1	0	13	0	1.7506523	0	5	0	0.00141024
chr5	98775350	98775409	60	1	0	13	0	0.71538971	0	9	0	0.0331093
chr5	98775409	99011063	235655	1	0	13	0	0.91308053	0	8	0	0.01767679
chr5	99011063	99011122	60	1	0	15	0	0.41444892	0	13	0	0.09154957
chr5	99011122	99272936	261815	1	0	15	0	0.55190077	0	12	0	0.0565833
chr5	99272936	99458486	185551	2	0	14	0	0.5498098	0	11	0	0.0574087
chr5	99458486	99458545	60	1	0	16	0	0.55623409	0	13	0	0.05490675
chr5	99458545	99784028	325484	3	0	15	0	0.55190077	0	12	0	0.0565833
chr5	99784028	99874854	90827	1	0	10	0	0.56314362	0	7	0	0.05232577
chr5	99874854	100001989	127136	2	0	9	0	0.42015402	0	7	0	0.08584816
chr5	100001989	100002048	60	1	0	15	0	0.90719478	0	10	0	0.01817691
chr5	100002048	100160174	158127	3	0	9	0	0.5732208	0	6	0	0.04875589
chr5	100160174	100302977	142804	5	0	15	0	0.90719478	0	10	0	0.01817691
chr5	100302977	100303036	60	1	0	16	0	0.91308053	0	11	0	0.01767679
chr5	100303036	100508209	205174	3	0	8	0	0.20764654	0	8	0	0.20764654
chr5	100508209	100508268	60	1	0	8	0	0.13872638	0	9	0	0.30102999
chr5	100508268	100650791	142524	1	0	7	0	0.08584816	0	9	0	0.42015402
chr5	100650791	100814258	163468	2	0	8	0	0.05490675	0	11	0	0.55623409
chr5	100814258	100908297	94040	2	0	11	0	0.14385241	0	12	0	0.30102999
chr5	100908297	100996616	88320	1	0	9	0	0.0565833	0	12	0	0.55190077
chr5	100996616	101148949	152334	1	0	6	0	0.08122616	0	8	0	0.4250187
chr5	101148949	101572563	423615	3	0	6	0	0.44141547	0	4	0	0.06713722
chr5	101572563	101583133	10571	2	0	7	0	0.30102999	0	6	0	0.129913
chr5	101583133	101627144	44012	1	0	7	0	0.43181735	0	5	0	0.07511598
chr5	101627144	101729935	102792	3	0	9	0	0.30102999	0	8	0	0.13872638
chr5	101729935	101832497	102563	3	0	9	0	0.42015402	0	7	0	0.08584816
chr5	101832497	101834242	1746	2	0	12	0	0.41314172	0	10	0	0.09290028
chr5	101834242	102183237	348996	3	0	12	0	0.55190077	0	9	0	0.0565833
chr5	102183237	102526581	343345	9	0	11	0	0.55623409	0	8	0	0.05490675
chr5	102526581	102594041	67461	1	0	11	0	0.73110763	0	7	0	0.03037338
chr5	102594041	102612576	18536	1	0	11	0	0.9449689	0	6	0	0.01518174
chr5	102612576	102732128	119553	1	0	9	0	1.01542894	0	4	0	0.01077081
chr5	102732128	102732187	60	1	0	9	0	0.76806864	0	5	0	0.02473314
chr5	102732187	103185751	453565	4	0	7	0	0.60763643	0	4	0	0.03812622
chr5	103185751	103185810	60	1	0	11	0	0.73110763	0	7	0	0.03037338
chr5	103185810	103409776	223967	1	0	10	0	0.74627054	0	6	0	0.02793176
chr5	103409776	103409835	60	1	0	11	0	0.9449689	0	6	0	0.01518174
chr5	103409835	103566362	156528	1	0	10	0	1.2568129	0	4	0	0.00530919
chr5	103566362	103566421	60	1	0	11	0	1.52371709	0	4	0	0.00247414
chr5	103566421	103618818	52398	1	0	10	0	2.0620585	0	2	0	3.90E-04
chr5	103618818	103770041	151224	1	0	9	0	1.74076927	0	2	0	9.54E-04
chr5	103770041	103770100	60	1	0	9	0	1.01542894	0	4	0	0.01077081
chr5	103770100	103924672	154573	1	0	8	0	1.44210395	0	2	0	0.00221948
chr5	103924672	103924731	60	1	0	8	0	1.07548421	0	3	0	0.0079614
chr5	103924731	104118070	193340	2	0	2	0	0.61140001	0	0	0	0
chr5	104118070	104118129	60	1	0	7	0	0.60763643	0	4	0	0.03812622
chr5	104118129	104246304	128176	1	0	4	0	0.18734596	0	4	0	0.18734596
chr5	104246304	104246363	60	1	0	5	0	0.30102999	0	4	0	0.11390336
chr5	104246363	104412706	166344	2	0	3	0	0.30102999	0	2	0	0.08289318
chr5	104412706	104644094	231389	3	0	3	0	0.17593012	0	3	0	0.17593012
chr5	104644094	104823902	179809	1	0	2	0	0.1575501	0	2	0	0.1575501
chr5	104823902	104823961	60	1	0	2	0	0.0429175	0	4	0	0.47744371
chr5	105623245	105796347	173103	2	0	1	0	0.05404976	0	2	0	0.30102999
chr5	105796347	105796406	60	1	0	2	0	0.01053319	0	6	0	0.91219088
chr5	105796406	106034651	238246	2	0	1	0	0.00204627	0	6	0	1.31360226
chr5	106034651	106034710	60	1	0	4	0	0.03812622	0	7	0	0.60763643
chr5	106034710	106188878	154169	1	0	3	0	0.03070643	0	6	0	0.63695542
chr5	106188878	106296486	107609	2	0	4	0	0.06713722	0	6	0	0.44141547
chr5	106296486	106296545	60	1	0	5	0	0.12309572	0	6	0	0.30102999
chr5	106296545	106677056	380512	2	0	4	0	0.06713722	0	6	0	0.44141547
chr5	106677056	106860246	183191	5	0	6	0	0.20064824	0	6	0	0.20064824
chr5	106860246	106911886	51641	2	0	7	0	0.20469099	0	7	0	0.20469099
chr5	106911886	107083896	172011	3	0	5	0	0.07511598	0	7	0	0.43181735
chr5	107083896	107083955	60	1	0	6	0	0.129913	0	7	0	0.30102999
chr5	107083955	107250021	166067	2	0	5	0	0.19510895	0	5	0	0.19510895
chr5	107250021	107331137	81117	1	0	5	0	0.30102999	0	4	0	0.11390336
chr5	107331137	107331196	60	1	0	7	0	0.20469099	0	7	0	0.20469099
chr5	107331196	107396366	65171	1	0	6	0	0.129913	0	7	0	0.30102999
chr5	107396366	107461547	65182	1	0	5	0	0.07511598	0	7	0	0.43181735

chr5	107461547	107461606	60	1	0	6	0	0.08122616	0	8	0	0.4250187
chr5	107461606	107644164	182559	3	0	6	0	0.129913	0	7	0	0.30102999
chr5	107644164	107644223	60	1	0	7	0	0.13499366	0	8	0	0.30102999
chr5	107644223	107787100	142878	2	0	7	0	0.20469099	0	7	0	0.20469099
chr5	107787100	107787159	60	1	0	8	0	0.30102999	0	7	0	0.13499366
chr5	107787159	107966441	179283	1	0	6	0	0.129913	0	7	0	0.30102999
chr5	107966441	107966500	60	1	0	7	0	0.13499366	0	8	0	0.30102999
chr5	107966500	108083526	117027	1	0	7	0	0.20469099	0	7	0	0.20469099
chr5	108083526	108083585	60	1	1	8	0.30102999	0.30102999	0	7	0	0.13499366
chr5	108083585	108135211	51627	1	1	8	0.30102999	0.58747015	0	5	0	0.04407651
chr5	108135211	108175612	40402	1	1	7	0.30102999	0.43181735	0	5	0	0.07511598
chr5	108175612	108175671	60	1	1	8	0.30102999	0.20764654	0	8	0	0.20764654
chr5	108175671	108281818	106148	2	1	8	0.30102999	0.4250187	0	6	0	0.08122616
chr5	108281818	108317680	35863	2	1	10	0.30102999	0.56314362	0	7	0	0.05232577
chr5	108317680	108363286	45607	1	1	8	0.30102999	0.4250187	0	6	0	0.08122616
chr5	108363286	108396682	33397	1	1	8	0.30102999	0.58747015	0	5	0	0.04407651
chr5	108396682	108493634	96953	3	1	8	0.30102999	0.4250187	0	6	0	0.08122616
chr5	108493634	108493693	60	1	1	10	0.30102999	0.56314362	0	7	0	0.05232577
chr5	108493693	108523238	29546	1	0	10	0	0.56314362	0	7	0	0.05232577
chr5	108523238	108653767	130530	2	0	9	0	0.5732208	0	6	0	0.04875589
chr5	108653767	108653826	60	1	0	9	0	0.42015402	0	7	0	0.08584816
chr5	108653826	108730382	76557	2	0	9	0	0.5732208	0	6	0	0.04875589
chr5	108730382	108905068	174687	2	0	6	0	0.20064824	0	6	0	0.20064824
chr5	108905068	108978641	73574	2	1	6	0.30102999	0.129913	0	7	0	0.30102999
chr5	108978641	109072537	93897	2	1	7	0.30102999	0.20469099	0	7	0	0.20469099
chr5	109072537	109153155	80619	3	1	7	0.30102999	0.13499366	0	8	0	0.30102999
chr5	109153155	109181554	28400	1	1	6	0.30102999	0.08122616	0	8	0	0.4250187
chr5	109181554	109181613	60	1	1	7	0.30102999	0.13499366	0	8	0	0.30102999
chr5	109181613	109272288	90676	1	1	7	0.30102999	0.20469099	0	7	0	0.20469099
chr5	109272288	109516665	244378	1	0	6	0	0.129913	0	7	0	0.30102999
chr5	109516665	109516724	60	1	0	7	0	0.05232577	0	10	0	0.56314362
chr5	109516724	109692030	175307	1	0	7	0	0.08584816	0	9	0	0.42015402
chr5	109692030	109813485	121456	3	1	7	0.30102999	0.03037338	0	11	0	0.73110763
chr5	109813485	109851548	38064	1	1	6	0.30102999	0.02793176	0	10	0	0.74627054
chr5	109851548	109863434	11887	2	2	6	0.61140001	0.02793176	0	10	0	0.74627054
chr5	109863434	109925123	61690	1	1	6	0.30102999	0.02793176	0	10	0	0.74627054
chr5	109925123	110023143	98021	2	0	6	0	0.02793176	0	10	0	0.74627054
chr5	110023143	110077753	54611	2	1	6	0.30102999	0.02793176	0	10	0	0.74627054
chr5	110077753	110077812	60	1	1	6	0.30102999	0.01518174	0	11	0	0.9449689
chr5	110077812	110153967	76156	2	1	6	0.30102999	0.02793176	0	10	0	0.74627054
chr5	110153967	110525673	371707	6	1	6	0.30102999	0.01518174	0	11	0	0.9449689
chr5	110525673	110570434	44762	1	0	6	0	0.01518174	0	11	0	0.9449689
chr5	110570434	110644342	73909	3	0	7	0	0.01667721	0	12	0	0.92532268
chr5	110644342	110644401	60	1	1	8	0.30102999	0.03209037	0	12	0	0.72109894
chr5	110644401	110782376	137976	2	0	8	0	0.03209037	0	12	0	0.72109894
chr5	110782376	111008513	226138	5	0	11	0	0.09334429	0	13	0	0.41271556
chr5	111008513	111008572	60	1	0	12	0	0.14385241	0	13	0	0.30102999
chr5	111008572	111084651	76080	1	0	11	0	0.09334429	0	13	0	0.41271556
chr5	111084651	111084710	60	1	0	12	0	0.09290028	0	14	0	0.41314172
chr5	111084710	111194538	109829	1	0	11	0	0.0574087	0	14	0	0.5498098
chr5	111194538	111528822	334285	6	0	10	0	0.03344703	0	14	0	0.71353304
chr5	111528822	111528881	60	1	0	11	0	0.0574087	0	14	0	0.5498098
chr5	111528881	111549545	20665	1	0	10	0	0.03344703	0	14	0	0.71353304
chr5	111549545	111599291	49747	1	0	9	0	0.01817691	0	14	0	0.90719478
chr5	111599291	111599350	60	1	0	10	0	0.03344703	0	14	0	0.71353304
chr5	111599350	111678466	79117	2	0	10	0	0.0574087	0	13	0	0.5498098
chr5	111678466	111756788	78323	4	0	10	0	0.01817691	0	15	0	0.90719478
chr5	111756788	112026277	269490	2	0	8	0	0.00909834	0	14	0	1.13428373
chr5	112026277	112065690	39414	2	0	9	0	0.00926608	0	15	0	1.13004284
chr5	112065690	112159202	93513	5	0	8	0	0.00433294	0	15	0	1.3873209
chr5	112159202	112361367	202166	7	1	8	0.30102999	0.00433294	0	15	0	1.3873209
chr5	112361367	112487104	125738	3	0	8	0	0.00433294	0	15	0	1.3873209
chr5	112487104	112535189	48086	1	0	6	0	0.00166733	0	14	0	1.70763027
chr5	112535189	112630129	94941	2	0	6	0	0.00778066	0	12	0	1.17038931
chr5	112630129	112630176	48	1	0	6	0	0.00166733	0	14	0	1.70763027
chr5	112630176	112689256	59081	1	0	6	0	0.00778066	0	12	0	1.17038931
chr5	112689256	112768686	79431	1	0	6	0	0.01518174	0	11	0	0.9449689
chr5	112768686	112828983	60298	2	1	6	0.30102999	0.01518174	0	11	0	0.9449689
chr5	112828983	112883976	54994	2	1	7	0.30102999	0.00859896	0	13	0	1.14735594
chr5	112883976	112884035	60	1	1	8	0.30102999	0.01767679	0	13	0	0.91308053
chr5	112884035	112929668	45634	1	1	6	0.30102999	0.00373523	0	13	0	1.42397267
chr5	112929668	112975695	46028	2	1	7	0.30102999	0.00859896	0	13	0	1.14735594
chr5	112975695	113144517	168823	1	1	5	0.30102999	0.01320236	0	10	0	0.97390707
chr5	113144517	113353747	209231	1	1	5	0.30102999	0.07511598	0	7	0	0.43181735
chr5	113353747	113560958	207212	2	1	6	0.30102999	0.129913	0	7	0	0.30102999
chr5	113560958	113561017	60	1	1	8	0.30102999	0.05490675	0	11	0	0.55623409
chr5	113561017	113602530	41514	1	1	7	0.30102999	0.03037338	0	11	0	0.73110763
chr5	113602530	113602589	60	1	1	8	0.30102999	0.01767679	0	13	0	0.91308053
chr5	113602589	113700181	97593	1	1	8	0.30102999	0.03209037	0	12	0	0.72109894
chr5	113700181	113719832	19652	2	1	9	0.30102999	0.0565833	0	12	0	0.55190077
chr5	113719832	113798839	79008	2	1	7	0.30102999	0.08584816	0	9	0	0.42015402
chr5	113798839	113798898	60	1	1	8	0.30102999	0.08923391	0	10	0	0.4167287
chr5	113798898	113831998	33101	1	0	8	0	0.08923391	0	10	0	0.4167287
chr5	113831998	113832057	60	1	0	8	0	0.05490675	0	11	0	0.55623409
chr5	113832057	113872800	40744	1	0	7	0	0.05232577	0	10	0	0.56314362
chr5	113872800	113872859	60	1	0	7	0	0.03037338	0	11	0	0.73110763
chr5	113872859	113996482	123624	1	0	7	0	0.05232577	0	10	0	0.56314362
chr5	113996482	113996541	60	1	0	14	0	0.30102999	0	13	0	0.14303407
chr5	113996541	114065392	68852	1	0	13	0	0.30102999	0	12	0	0.14385241
chr5	114065392	114158839	93448	1	0	13	0	0.41271556	0	11	0	0.09334429
chr5	114158839	114158898	60	1	0	13	0	0.30102999	0	12	0	0.14385241
chr5	114158898	114392528	233631	1	0	11	0	0.30102999	0	10	0	0.14303407
chr5	114392528	114462055	69528	2	1	11	0.30102999	0.21200206	0	11	0	0.21200206
chr5	114462055	114518075	56021	3	1	14	0.30102999	0.5498098	0	11	0	0.0574087
chr5	114518075	114518134	60	1	1	14	0.30102999	0.30102999	0	13	0	0.14303407
chr5	114518134	114557744	39611	1	0	14	0	0.30102999	0	13	0	0.14303407
chr5	114557744	114615445	57702	1	0	14	0	0.41314172	0	12	0	0.09290028
chr5	114615445	114739019	123575	1	0	13	0	0.30102999	0	12	0	0.14385241
chr5	114739019	114926109	187091	5	0	13	0	0.21200206	0	13	0	0.21200206
chr5	114926109	114956855	30747	4	0	14	0	0.30102999	0	13	0	0.14303407
chr5	114956855	115284593	327739	6	0	13	0	0.21200206	0	13	0	0.21200206
chr5	115284593	115454851	170259	4	0	11	0	0.14385241	0	12	0	0.30102999
chr5	115454851	115508329	53479	1	0	9	0	0.0565833	0	12	0	0.55190077
chr5	115508329	115551734	43406	2	0	10	0	0.09290028	0	12	0	0.41314172
chr5	115551734	115551793	60	1	0	11	0	0.14385241	0	12	0	0.30102999
chr5	115551793	115578519	26727	1	0	10	0	0.14303407	0	11	0	0.30102999
chr5	115578519	115628066	49548	1	0	9	0	0.14135546	0	10	0	0.30102999
chr5	115628066	115781606	153541	4	0	9	0	0.09154957	0	11	0	0.41444892
chr5	115781606	115852365	70760	3	0	11	0	0.21200206	0	11	0	0.21200206
chr5	115852365	115852424	60	1	0	12	0	0.30102999	0	11	0	0.14385241
chr5	115852424	115878132	25709	1	0	11	0	0.30102999	0	10	0	0.14303407
chr5	115878132	115910167	32036	1	0	11	0	0.41444892	0	9	0	0.09154957
chr5	115910167	115910223	57	1	0	11	0	0.30102999	0	10	0	0.14303407
chr5	115910223	116150712	240490	2	0	10	0	0.4167287	0	8	0	0.08923391
chr5	116150712	116150771	60	1	0	10	0	0.30102999	0	9	0	0.14135546
chr5	116150771	116605943	455173	2	0	10	0	0.56314362	0	7	0	0.05232577
chr5	116605943	116606002	60	1	0	11	0	0.41444892	0	9	0	0.09154957
chr5	116606002	116893086	287085	2	0	9	0	1.33111237	0	3	0	0.00378107
chr5	116893086	116893145	60	1	0	9	0	1.01542894	0	4	0	0.01077081
chr5	116893145	117116161	223017	2	0	4	0	0.18734596	0	4	0	0.18734596
chr5	117116161	117116220	60	1	0	5	0	0.30102999	0	4	0	0.11390336
chr5	117116220	117337960	221741	1	0	4	0	0.18734596	0	4	0	0.18734596
chr5	117337960	117338019	60	1	0	7	0	0.20469099	0	7	0	0.20469099
chr5	117338019	117455204	117186	1	0	6	0	0.129913	0	7	0	0.30102999

chr5	117455204	117657010	201807	1	0	5	0	0.07511598	0	7	0	0.43181735
chr5	117657010	117657069	60	1	0	6	0	0.00166733	0	14	0	1.70763027
chr5	117657069	117715795	58727	1	0	6	0	0.00373523	0	13	0	1.42397267
chr5	117715795	117715854	60	1	0	8	0	0.01767679	0	13	0	0.91308053
chr5	117715854	117869418	153565	2	0	8	0	0.03209037	0	12	0	0.72109894
chr5	117869418	118088313	218896	2	0	8	0	0.01767679	0	13	0	0.91308053
chr5	118088313	118280766	192454	4	0	7	0	0.00859896	0	13	0	1.14735594
chr5	118280766	118299388	18623	2	0	8	0	0.01767679	0	13	0	0.91308053
chr5	118299388	118356972	57585	2	0	8	0	0.03209037	0	12	0	0.72109894
chr5	118356972	118414744	57773	2	0	8	0	0.00909834	0	14	0	1.13428373
chr5	118414744	118507580	92837	2	0	7	0	0.00859896	0	13	0	1.14735594
chr5	118507580	118651618	144039	5	0	8	0	0.00909834	0	14	0	1.13428373
chr5	118651618	118753545	101928	2	0	7	0	0.00859896	0	13	0	1.14735594
chr5	118753545	118812999	59455	2	0	8	0	0.01767679	0	13	0	0.91308053
chr5	118812999	118837814	24816	1	0	7	0	0.00859896	0	13	0	1.14735594
chr5	118837814	118913520	75707	1	0	6	0	0.00373523	0	13	0	1.42397267
chr5	118913520	118968466	54947	2	0	8	0	0.01767679	0	13	0	0.91308053
chr5	118968466	119121544	153079	1	0	7	0	0.00859896	0	13	0	1.14735594
chr5	119121544	119230040	108497	1	0	7	0	0.01667721	0	12	0	0.92532268
chr5	119230040	119354507	124468	1	0	5	0	0.00666883	0	11	0	1.20557689
chr5	119354507	119472895	118389	1	0	3	0	0.00378107	0	9	0	1.33111237
chr5	119472895	119472954	60	1	0	4	0	0.01077081	0	9	0	1.01542894
chr5	119472954	119595933	122980	1	0	2	0	0.01053319	0	6	0	0.91219088
chr5	119595933	119696984	101052	1	0	2	0	0.02162467	0	5	0	0.68214471
chr5	119696984	119776567	79584	2	0	2	0	0.01053319	0	6	0	0.91219088
chr5	119776567	119776626	60	1	0	3	0	0.01598258	0	7	0	0.84395715
chr5	119776626	119816036	39411	1	0	2	0	0.0429175	0	4	0	0.47744371
chr5	119816036	119954182	138147	3	0	1	0	0.02438896	0	3	0	0.51676182
chr5	119954182	120022491	68310	1	0	1	0	0.05404976	0	2	0	0.30102999
chr5	120022491	120085675	63185	2	0	1	0	0.02438896	0	3	0	0.51676182
chr5	120085675	120367158	281484	2	0	0	0	0	0	1	0	0.30102999
chr5	120367158	120449277	82120	2	0	1	0	0.05404976	0	2	0	0.30102999
chr5	120449277	120449336	60	1	0	3	0	0.03070643	0	6	0	0.63695542
chr5	120449336	120848731	399396	2	0	1	0	0.05404976	0	2	0	0.30102999
chr5	120848731	120848790	60	1	0	4	0	0.03812622	0	7	0	0.60763643
chr5	120848790	120991141	142352	1	0	3	0	0.03070643	0	6	0	0.63695542
chr5	120991141	120991200	60	1	0	3	0	0.01598258	0	7	0	0.84395715
chr5	120991200	121188438	197239	2	0	2	0	0.02162467	0	5	0	0.68214471
chr5	121188438	121249950	61513	2	0	3	0	0.01598258	0	7	0	0.84395715
chr5	121249950	121250009	60	1	0	4	0	0.02074938	0	8	0	0.79906872
chr5	121250009	121599315	349307	6	0	4	0	0.03812622	0	7	0	0.60763643
chr5	121599315	121599374	60	1	0	9	0	0.20975986	0	9	0	0.20975986
chr5	121599374	121659945	60572	1	0	7	0	0.08584816	0	9	0	0.42015402
chr5	121659945	121729626	69682	2	0	7	0	0.20469099	0	7	0	0.20469099
chr5	121729626	121975965	246340	5	0	9	0	0.20975986	0	9	0	0.20975986
chr5	121975965	122127751	151787	3	0	9	0	0.09154957	0	11	0	0.41444892
chr5	122127751	122165645	37895	2	0	9	0	0.0565833	0	12	0	0.55190077
chr5	122165645	122281809	116165	3	0	9	0	0.09154957	0	11	0	0.41444892
chr5	122281809	122361508	79700	1	0	9	0	0.14135546	0	10	0	0.30102999
chr5	122361508	122628967	267460	5	0	8	0	0.20764654	0	8	0	0.20764654
chr5	122628967	122629026	60	1	0	9	0	0.30102999	0	8	0	0.13872638
chr5	122629026	122694152	65127	1	0	8	0	0.30102999	0	7	0	0.13499366
chr5	122694152	122748132	53981	2	0	8	0	0.4250187	0	6	0	0.08122616
chr5	122748132	122748189	58	1	0	8	0	0.20764654	0	8	0	0.20764654
chr5	122748189	122911510	163322	4	0	8	0	0.4250187	0	6	0	0.08122616
chr5	122911510	123109346	197837	2	0	7	0	0.43181735	0	5	0	0.07511598
chr5	123109346	123290915	181570	2	0	7	0	0.13499366	0	8	0	0.30102999
chr5	123290915	123290974	60	1	0	7	0	0.08584816	0	9	0	0.42015402
chr5	123290974	123457514	166541	1	0	5	0	0.07511598	0	7	0	0.43181735
chr5	123457514	123522792	65279	1	0	4	0	0.06713722	0	6	0	0.44141547
chr5	123522792	123614800	92009	1	0	3	0	0.03070643	0	6	0	0.63695542
chr5	123614800	123783181	168382	1	0	3	0	0.10122019	0	4	0	0.30102999
chr5	123783181	123968674	185494	2	0	3	0	0.17593012	0	3	0	0.17593012
chr5	123968674	123968733	60	1	0	4	0	0.30102999	0	3	0	0.10122019
chr5	123968733	124043544	74812	2	0	3	0	0.51676182	0	1	0	0.02438896
chr5	124043544	124043603	60	1	0	4	0	0.30102999	0	3	0	0.10122019
chr5	124043603	124159005	115403	2	0	3	0	0.51676182	0	1	0	0.02438896
chr5	124159005	124363282	204278	3	0	4	0	0.18734596	0	4	0	0.18734596
chr5	124363282	124363341	60	1	0	4	0	0.11390336	0	5	0	0.30102999
chr5	124363341	124530336	166996	1	0	4	0	0.30102999	0	3	0	0.10122019
chr5	124530336	124846125	315790	2	0	4	0	0.47744371	0	2	0	0.0429175
chr5	124846125	124846184	60	1	0	7	0	0.60763643	0	4	0	0.03812622
chr5	124846184	125007374	161191	1	0	6	0	0.44141547	0	4	0	0.06713722
chr5	125007374	125007433	60	1	0	6	0	0.30102999	0	5	0	0.12309572
chr5	125007433	125141620	134188	1	0	6	0	0.63695542	0	3	0	0.03070643
chr5	125141620	125297047	155428	1	0	5	0	0.45545077	0	3	0	0.05670724
chr5	125297047	125475206	178160	2	0	5	0	0.07511598	0	7	0	0.43181735
chr5	125475206	125475265	60	1	0	8	0	0.20764654	0	8	0	0.20764654
chr5	125475265	125774098	298834	2	0	8	0	0.30102999	0	7	0	0.13499366
chr5	125774098	125794639	20542	2	0	9	0	0.20975986	0	9	0	0.20975986
chr5	125794639	125829518	34880	2	0	10	0	0.30102999	0	9	0	0.14135546
chr5	125829518	125882578	53061	2	0	9	0	0.30102999	0	8	0	0.13872638
chr5	125882578	125882637	60	1	0	10	0	0.30102999	0	9	0	0.14135546
chr5	125882637	125993510	110874	4	0	9	0	0.20975986	0	9	0	0.20975986
chr5	125993510	126161864	168355	4	0	9	0	0.30102999	0	8	0	0.13872638
chr5	126161864	126397126	235263	5	0	8	0	0.30102999	0	7	0	0.13499366
chr5	126397126	126567625	170500	3	0	8	0	0.20764654	0	8	0	0.20764654
chr5	126567625	126599054	31430	2	0	9	0	0.20975986	0	9	0	0.20975986
chr5	126599054	126599113	60	1	0	9	0	0.14135546	0	10	0	0.30102999
chr5	126599113	126694535	95423	2	0	9	0	0.20975986	0	9	0	0.20975986
chr5	126694535	126694594	60	1	0	9	0	0.14135546	0	10	0	0.30102999
chr5	126694594	126793496	98903	3	0	9	0	0.20975986	0	9	0	0.20975986
chr5	126793496	126865996	72501	1	0	8	0	0.30102999	0	7	0	0.13499366
chr5	126865996	126866055	60	1	0	8	0	0.20764654	0	8	0	0.20764654
chr5	126866055	126934857	68803	3	0	8	0	0.30102999	0	7	0	0.13499366
chr5	126934857	126934916	60	1	0	8	0	0.20764654	0	8	0	0.20764654
chr5	126934916	127046164	111249	1	0	7	0	0.30102999	0	6	0	0.129913
chr5	127046164	127046223	60	1	0	7	0	0.20469099	0	7	0	0.20469099
chr5	127046223	127270100	223878	3	0	6	0	0.129913	0	7	0	0.30102999
chr5	127270100	127270159	60	1	0	7	0	0.20469099	0	7	0	0.20469099
chr5	127270159	127375077	104919	1	0	6	0	0.20064824	0	6	0	0.20064824
chr5	127375077	127714554	339478	8	0	6	0	0.129913	0	7	0	0.30102999
chr5	127714554	127767803	53250	1	0	6	0	0.30102999	0	5	0	0.12309572
chr5	127767803	127799372	31570	1	0	6	0	0.44141547	0	4	0	0.06713722
chr5	127799372	127822101	22730	2	0	6	0	0.20064824	0	6	0	0.20064824
chr5	127822101	127850808	28708	1	0	5	0	0.12309572	0	6	0	0.30102999
chr5	127850808	127873620	22813	1	0	5	0	0.30102999	0	4	0	0.11390336
chr5	127873620	127873679	60	1	0	5	0	0.12309572	0	6	0	0.30102999
chr5	127873679	127922519	48841	1	0	4	0	0.06713722	0	6	0	0.44141547
chr5	127922519	127922578	60	1	0	5	0	0.12309572	0	6	0	0.30102999
chr5	127922578	127976136	53559	1	0	5	0	0.19510895	0	5	0	0.19510895
chr5	127976136	128062650	86515	1	0	5	0	0.30102999	0	4	0	0.11390336
chr5	128062650	128062709	60	1	0	7	0	0.30102999	0	6	0	0.129913
chr5	128062709	128238488	175780	1	0	7	0	0.43181735	0	5	0	0.07511598
chr5	128238488	128316143	77656	2	0	7	0	0.30102999	0	6	0	0.129913
chr5	128316143	128440739	124597	4	0	7	0	0.13499366	0	8	0	0.30102999
chr5	128440739	128445513	4775	2	0	7	0	0.08584816	0	9	0	0.42015402
chr5	128445513	128606033	160521	1	0	7	0	0.20469099	0	7	0	0.20469099
chr5	128606033	128770640	164608	1	0	6	0	0.30102999	0	5	0	0.12309572
chr5	128770640	128770699	60	1	0	6	0	0.20064824	0	6	0	0.20064824
chr5	128770699	128831078	60380	1	0	6	0	0.30102999	0	5	0	0.12309572
chr5	128831078	128831137	60	1	0	6	0	0.20064824	0	6	0	0.20064824
chr5	128831137	128932258	101122	2	0	6	0	0.30102999	0	5	0	0.12309572
chr5	128932258	128990085	57828	2	0	6	0	0.20064824	0	6	0	0.20064824

chr5	128990085	129025955	35871	1	0	5	0	0.12309572	0	6	0	0.30102999
chr5	129025955	129157545	131591	3	0	5	0	0.19510895	0	5	0	0.19510895
chr5	129157545	129309601	152057	2	0	5	0	0.30102999	0	4	0	0.11390336
chr5	129309601	129309660	60	1	0	5	0	0.12309572	0	6	0	0.30102999
chr5	129309660	129439531	129872	2	0	5	0	0.30102999	0	4	0	0.11390336
chr5	129439531	129521150	81620	2	0	4	0	0.30102999	0	3	0	0.10122019
chr5	129521150	129521209	60	1	0	4	0	0.11390336	0	5	0	0.30102999
chr5	129521209	129687092	165884	1	0	3	0	0.10122019	0	4	0	0.30102999
chr5	129687092	129687151	60	1	0	3	0	0.05670724	0	5	0	0.45545077
chr5	129687151	129747565	60415	1	0	3	0	0.10122019	0	4	0	0.30102999
chr5	129747565	129747624	60	1	0	3	0	0.05670724	0	5	0	0.45545077
chr5	129747624	129876046	128423	1	0	3	0	0.17593012	0	3	0	0.17593012
chr5	129876046	130006441	130396	2	0	3	0	0.10122019	0	4	0	0.30102999
chr5	130006441	130166702	160262	2	0	3	0	0.0079614	0	8	0	1.07548421
chr5	130166702	130278278	111577	2	0	8	0	0.08923391	0	10	0	0.4167287
chr5	130278278	130278337	60	1	0	10	0	0.14303407	0	11	0	0.30102999
chr5	130278337	130515797	237461	2	0	8	0	0.05490675	0	11	0	0.55623409
chr5	130515797	130515856	60	1	0	8	0	0.01767679	0	13	0	0.91308053
chr5	130515856	130535411	19556	1	0	8	0	0.03209037	0	12	0	0.72109894
chr5	130535411	130652568	117158	2	0	8	0	0.05490675	0	11	0	0.55623409
chr5	130652568	130698970	46403	2	0	8	0	0.03209037	0	12	0	0.72109894
chr5	130698970	130699029	60	1	0	8	0	0.01767679	0	13	0	0.91308053
chr5	130699029	130831319	132291	4	0	8	0	0.03209037	0	12	0	0.72109894
chr5	130831319	130928339	97021	3	0	8	0	0.01767679	0	13	0	0.91308053
chr5	130928339	130977930	49592	2	0	8	0	0.03209037	0	12	0	0.72109894
chr5	130977930	131044312	66383	3	0	8	0	0.01767679	0	13	0	0.91308053
chr5	131044312	131190312	146001	5	0	9	0	0.0331093	0	13	0	0.71538971
chr5	131190312	131190371	60	1	0	10	0	0.0574087	0	13	0	0.5498098
chr5	131190371	131251648	61278	1	0	9	0	0.0331093	0	13	0	0.71538971
chr5	131251648	131251707	60	1	0	12	0	0.14385241	0	13	0	0.30102999
chr5	131251707	131302175	50469	1	0	11	0	0.09334429	0	13	0	0.41271556
chr5	131302175	131484039	181865	7	0	11	0	0.0574087	0	14	0	0.5498098
chr5	131484039	131484098	60	1	0	12	0	0.09290028	0	14	0	0.41314172
chr5	131484098	131528360	44263	1	0	11	0	0.0574087	0	14	0	0.5498098
chr5	131528360	131528419	60	1	0	12	0	0.09290028	0	14	0	0.41314172
chr5	131528419	131724586	196168	4	0	11	0	0.0574087	0	14	0	0.5498098
chr5	131724586	131778715	54130	2	0	12	0	0.0565833	0	15	0	0.55190077
chr5	131778715	131953901	175187	5	0	13	0	0.09154957	0	15	0	0.41444892
chr5	131953901	132009648	55748	4	0	11	0	0.0331093	0	15	0	0.71538971
chr5	132009648	132093891	84244	2	0	10	0	0.03344703	0	14	0	0.71353304
chr5	132093891	132197917	104027	4	0	9	0	0.01817691	0	14	0	0.90719478
chr5	132197917	132270440	72524	8	0	10	0	0.03344703	0	14	0	0.71353304
chr5	132270440	132270499	60	1	0	10	0	0.01817691	0	15	0	0.90719478
chr5	132270499	132400648	130150	4	0	10	0	0.03344703	0	14	0	0.71353304
chr5	132400648	132543216	142569	3	0	11	0	0.0574087	0	14	0	0.5498098
chr5	132543216	132659634	116419	3	0	12	0	0.0565833	0	15	0	0.55190077
chr5	132659634	132745729	86096	2	0	12	0	0.09290028	0	14	0	0.41314172
chr5	132745729	132745788	60	1	0	12	0	0.01667721	0	17	0	0.92532268
chr5	132745788	132820420	74633	2	0	12	0	0.03209037	0	16	0	0.72109894
chr5	132820420	132943728	123309	2	0	11	0	0.0331093	0	15	0	0.71538971
chr5	132943728	132998301	54574	2	0	11	0	0.01767679	0	16	0	0.91308053
chr5	132998301	132998360	60	1	0	12	0	0.03209037	0	16	0	0.72109894
chr5	132998360	133096935	98576	1	0	11	0	0.0574087	0	14	0	0.5498098
chr5	133096935	133096994	60	1	0	11	0	0.0331093	0	15	0	0.71538971
chr5	133096994	133171114	74121	1	0	11	0	0.0574087	0	14	0	0.5498098
chr5	133171114	133171173	60	1	0	11	0	0.0331093	0	15	0	0.71538971
chr5	133171173	133295735	124563	1	0	8	0	0.01767679	0	13	0	0.91308053
chr5	133295735	133325740	30006	1	0	7	0	0.00859896	0	13	0	1.14735594
chr5	133325740	133325799	60	1	0	9	0	0.00926608	0	15	0	1.13004284
chr5	133325799	133338007	12209	1	0	8	0	0.00909834	0	14	0	1.13428373
chr5	133338007	133395340	57334	2	0	9	0	0.01817691	0	14	0	0.90719478
chr5	133395340	133395399	60	1	0	9	0	0.00926608	0	15	0	1.13004284
chr5	133395399	133428495	33097	1	0	8	0	0.00433294	0	15	0	1.3873209
chr5	133428495	133481929	53435	4	0	8	0	0.00188722	0	16	0	1.67551277
chr5	133481929	133560263	78335	3	0	6	0	2.57E-04	0	16	0	2.37598969
chr5	133560263	133657551	97289	3	0	7	0	7.41E-04	0	16	0	2.00316618
chr5	133657551	133695676	38126	1	0	6	0	6.86E-04	0	15	0	2.02387299
chr5	133695676	133716502	20827	2	0	6	0	0.00373523	0	13	0	1.42397267
chr5	133716502	133738926	22425	3	0	6	0	6.86E-04	0	15	0	2.02387299
chr5	133738926	133746561	7636	2	0	5	0	5.83E-04	0	14	0	2.06733314
chr5	133746561	133845877	99317	1	0	5	0	0.00141024	0	13	0	1.7506523
chr5	133845877	133845936	60	1	0	7	0	7.41E-04	0	16	0	2.00316618
chr5	133845936	133909957	64022	2	0	6	0	0.00166733	0	14	0	1.70763027
chr5	133909957	133937242	27286	2	0	6	0	6.86E-04	0	15	0	2.02387299
chr5	133937242	134036265	99024	2	0	6	0	0.00166733	0	14	0	1.70763027
chr5	134036265	134087365	51101	3	0	6	0	6.86E-04	0	15	0	2.02387299
chr5	134087365	134164933	77569	3	0	5	0	2.22E-04	0	15	0	2.41659593
chr5	134164933	134236385	71453	3	0	5	0	5.83E-04	0	14	0	2.06733314
chr5	134236385	134257631	21247	2	0	5	0	7.71E-05	0	16	0	2.80176103
chr5	134257631	134296200	38570	1	0	5	0	2.22E-04	0	15	0	2.41659593
chr5	134296200	134305174	8975	2	0	6	0	2.57E-04	0	16	0	2.37598969
chr5	134305174	134442297	137124	2	0	5	0	5.83E-04	0	14	0	2.06733314
chr5	134442297	134442356	60	1	0	6	0	8.69E-05	0	17	0	2.76830915
chr5	134442356	134495421	53066	1	0	6	0	2.57E-04	0	16	0	2.37598969
chr5	134495421	134604936	109516	1	0	6	0	0.00166733	0	14	0	1.70763027
chr5	134604936	134670438	65503	2	0	7	0	0.00413023	0	14	0	1.39918918
chr5	134670438	134670497	60	1	0	8	0	0.00909834	0	14	0	1.13428373
chr5	134670497	134714599	44103	1	0	7	0	0.00413023	0	14	0	1.39918918
chr5	134714599	134837808	123210	3	0	4	0	1.70E-04	0	14	0	2.48919369
chr5	134837808	134837867	60	1	0	5	0	5.83E-04	0	14	0	2.06733314
chr5	134837867	134870691	32825	1	0	4	0	1.70E-04	0	14	0	2.48919369
chr5	134870691	135172706	302016	3	0	4	0	4.45E-04	0	13	0	2.13824703
chr5	135172706	135272480	99775	5	0	6	0	0.00166733	0	14	0	1.70763027
chr5	135272480	135288803	16324	2	0	9	0	0.01817691	0	14	0	0.90719478
chr5	135288803	135416143	127341	4	0	6	0	0.00373523	0	13	0	1.42397267
chr5	135416143	135508234	92092	4	0	7	0	0.00413023	0	14	0	1.39918918
chr5	135508234	135583508	75275	5	0	8	0	0.00909834	0	14	0	1.13428373
chr5	135583508	135633485	49978	3	0	9	0	0.00926608	0	15	0	1.13004284
chr5	135633485	135673896	40412	2	0	10	0	0.01817691	0	15	0	0.90719478
chr5	135673896	135734222	60327	1	0	9	0	0.00926608	0	15	0	1.13004284
chr5	135734222	135995553	261332	2	0	8	0	0.00433294	0	15	0	1.3873209
chr5	135995553	136141997	146445	2	0	9	0	0.00926608	0	15	0	1.13004284
chr5	136141997	136142056	60	1	0	10	0	0.01817691	0	15	0	0.90719478
chr5	136142056	136352234	210179	3	0	9	0	0.00926608	0	15	0	1.13004284
chr5	136352234	136453101	100868	3	0	11	0	0.0331093	0	15	0	0.71538971
chr5	136453101	136505662	52562	1	0	10	0	0.01817691	0	15	0	0.90719478
chr5	136505662	136602752	97091	3	0	9	0	0.00926608	0	15	0	1.13004284
chr5	136602752	136780316	177565	3	0	9	0	0.01817691	0	14	0	0.90719478
chr5	136780316	136827007	46692	2	0	10	0	0.03344703	0	14	0	0.71353304
chr5	136827007	137240552	413546	13	0	11	0	0.0574087	0	14	0	0.5498098
chr5	137240552	137273608	33057	2	0	11	0	0.09334429	0	13	0	0.41271556
chr5	137273608	137295350	21743	3	0	11	0	0.0574087	0	14	0	0.5498098
chr5	137295350	137356797	61448	2	0	9	0	0.01817691	0	14	0	0.90719478
chr5	137356797	137469718	112922	4	0	11	0	0.0574087	0	14	0	0.5498098
chr5	137469718	137492617	22900	2	0	11	0	0.0331093	0	15	0	0.71538971
chr5	137492617	137519410	26794	1	0	10	0	0.03344703	0	14	0	0.71353304
chr5	137519410	137574933	55524	2	0	9	0	0.0331093	0	13	0	0.71538971
chr5	137574933	137626434	51502	3	0	9	0	0.01817691	0	14	0	0.90719478
chr5	137626434	137708108	81675	3	0	9	0	0.0331093	0	13	0	0.71538971
chr5	137708108	137759797	51690	2	0	10	0	0.0574087	0	13	0	0.5498098
chr5	137759797	137851716	91920	4	0	10	0	0.01817691	0	15	0	0.90719478
chr5	137851716	137906720	55005	3	0	11	0	0.0331093	0	15	0	0.71538971
chr5	137906720	138052592	145873	2	0	10	0	0.03344703	0	14	0	0.71353304
chr5	138052592	138052651	60	1	0	10	0	0.01817691	0	15	0	0.90719478

chr5	138052651	138119087	66437	1	0	9	0	0.00926608	0	15	0	1.13004284
chr5	138119087	138268912	149826	3	0	9	0	0.0331093	0	13	0	0.71538971
chr5	138268912	138308907	39996	1	0	8	0	0.03209037	0	12	0	0.72109894
chr5	138308907	138308966	60	1	1	9	0.30102999	0.0331093	0	13	0	0.71538971
chr5	138308966	138411206	102241	2	1	8	0.30102999	0.05490675	0	11	0	0.55623409
chr5	138411206	138439097	27892	1	1	7	0.30102999	0.05232577	0	10	0	0.56314362
chr5	138439097	138464022	24926	2	1	7	0.30102999	0.03037338	0	11	0	0.73110763
chr5	138464022	138576104	112083	2	0	6	0	0.02793176	0	10	0	0.74627054
chr5	138576104	138576163	60	1	1	8	0.30102999	0.08923391	0	10	0	0.4167287
chr5	138576163	138631938	55776	1	0	8	0	0.08923391	0	10	0	0.4167287
chr5	138631938	138631997	60	1	0	9	0	0.0331093	0	13	0	0.71538971
chr5	138631997	138753046	121050	7	0	8	0	0.01767679	0	13	0	0.91308053
chr5	138753046	138927861	174816	2	0	7	0	0.03037338	0	11	0	0.73110763
chr5	138927861	139037798	109938	3	0	6	0	0.01518174	0	11	0	0.9449689
chr5	139037798	139037857	60	1	0	8	0	0.00909834	0	14	0	1.13428373
chr5	139037857	139137264	99408	2	0	6	0	0.00166733	0	14	0	1.70763027
chr5	139137264	139223498	86235	3	0	6	0	6.86E-04	0	15	0	2.02387299
chr5	139223498	139299777	76280	3	0	7	0	0.00183109	0	15	0	1.68336308
chr5	139299777	139555702	255926	6	0	9	0	0.00926608	0	15	0	1.13004284
chr5	139555702	139623279	67578	2	0	8	0	0.00433294	0	15	0	1.3873209
chr5	139623279	139623337	59	1	0	10	0	0.01817691	0	15	0	0.90719478
chr5	139623337	139648521	25185	1	0	9	0	0.00926608	0	15	0	1.13004284
chr5	139648521	139713291	64771	1	0	8	0	0.00433294	0	15	0	1.3873209
chr5	139713291	139940834	227544	7	0	9	0	0.00926608	0	15	0	1.13004284
chr5	139940834	140024110	83277	4	0	10	0	0.01817691	0	15	0	0.90719478
chr5	140024110	140026698	2589	2	0	10	0	0.00909834	0	16	0	1.13428373
chr5	140026698	140348951	322254	12	0	10	0	0.01817691	0	15	0	0.90719478
chr5	140348951	140603311	254361	17	0	10	0	0.00909834	0	16	0	1.13428373
chr5	140603311	140626204	22894	1	0	10	0	0.01817691	0	15	0	0.90719478
chr5	140626204	140682380	56177	2	0	10	0	0.03344703	0	14	0	0.71353304
chr5	140682380	140698320	15941	2	0	10	0	0.00909834	0	16	0	1.13428373
chr5	140698320	140699278	959	3	0	10	0	0.00413023	0	17	0	1.39918918
chr5	140699278	140905904	206627	5	0	10	0	0.00909834	0	16	0	1.13428373
chr5	140905904	140966681	60778	2	0	10	0	0.01817691	0	15	0	0.90719478
chr5	140966681	141112393	145713	9	0	10	0	0.00909834	0	16	0	1.13428373
chr5	141112393	141329234	216842	4	0	10	0	0.01817691	0	15	0	0.90719478
chr5	141329234	141391993	62760	3	0	9	0	0.00926608	0	15	0	1.13004284
chr5	141391993	141392037	45	1	0	10	0	0.01817691	0	15	0	0.90719478
chr5	141392037	141505819	113783	1	0	9	0	0.00926608	0	15	0	1.13004284
chr5	141505819	141533709	27891	2	0	8	0	0.00433294	0	15	0	1.3873209
chr5	141533709	141617510	83802	1	0	7	0	0.00413023	0	14	0	1.39918918
chr5	141617510	141708006	90497	4	0	8	0	0.00909834	0	14	0	1.13428373
chr5	141708006	141781422	73417	1	0	8	0	0.01767679	0	13	0	0.91308053
chr5	141781422	141877113	95692	1	0	5	0	0.00317045	0	12	0	1.46403142
chr5	141877113	142006212	129100	2	0	5	0	0.01320236	0	10	0	0.97390707
chr5	142006212	142301852	295641	6	0	5	0	0.07511598	0	7	0	0.43181735
chr5	142301852	142301911	60	1	0	5	0	0.04407651	0	8	0	0.58747015
chr5	142301911	142329740	27830	1	0	4	0	0.02074938	0	8	0	0.79906872
chr5	142329740	142500738	170999	3	0	4	0	0.03812622	0	7	0	0.60763643
chr5	142500738	142607457	106720	2	0	4	0	0.06713722	0	6	0	0.44141547
chr5	142607457	142688518	81062	2	0	5	0	0.04407651	0	8	0	0.58747015
chr5	142688518	142859458	170941	4	0	4	0	0.02074938	0	8	0	0.79906872
chr5	142859458	142990952	131495	2	0	5	0	0.04407651	0	8	0	0.58747015
chr5	142990952	142991011	60	1	0	6	0	0.08122616	0	8	0	0.4250187
chr5	142991011	143289610	298600	3	0	4	0	0.06713722	0	6	0	0.44141547
chr5	143289610	143485669	196060	2	0	4	0	0.03812622	0	7	0	0.60763643
chr5	143485669	143620852	135184	7	0	7	0	0.05232577	0	10	0	0.56314362
chr5	143620852	143733617	112766	2	0	7	0	0.08584816	0	9	0	0.42015402
chr5	143733617	143882271	148655	4	0	7	0	0.05232577	0	10	0	0.56314362
chr5	143882271	144027588	145318	2	0	9	0	0.14135546	0	10	0	0.30102999
chr5	144027588	144141705	114118	2	0	10	0	0.21118145	0	10	0	0.21118145
chr5	144141705	144298775	157071	1	0	9	0	0.14135546	0	10	0	0.30102999
chr5	144298775	144298834	60	1	0	9	0	0.09154957	0	11	0	0.41444892
chr5	144298834	144528930	230097	1	0	4	0	0.00530919	0	10	0	1.2568129
chr5	144528930	144618270	89341	1	0	4	0	0.06713722	0	6	0	0.44141547
chr5	144618270	144777001	158732	2	0	4	0	0.03812622	0	7	0	0.60763643
chr5	144777001	144777060	60	1	0	6	0	0.129913	0	7	0	0.30102999
chr5	144777060	145067292	290233	2	0	4	0	0.03812622	0	7	0	0.60763643
chr5	145067292	145250672	183381	5	0	5	0	0.07511598	0	7	0	0.43181735
chr5	145250672	145250731	60	1	0	5	0	0.04407651	0	8	0	0.58747015
chr5	145250731	145317535	66805	1	0	5	0	0.07511598	0	7	0	0.43181735
chr5	145317535	145405642	88108	2	0	4	0	0.03812622	0	7	0	0.60763643
chr5	145405642	145492927	87286	3	0	4	0	0.02074938	0	8	0	0.79906872
chr5	145492927	145758233	265307	7	0	5	0	0.04407651	0	8	0	0.58747015
chr5	145758233	145758292	60	1	1	5	0.30102999	0.04407651	0	8	0	0.58747015
chr5	145758292	145830751	72460	1	0	5	0	0.04407651	0	8	0	0.58747015
chr5	145830751	145883015	52265	3	0	6	0	0.04875589	0	9	0	0.5732208
chr5	145883015	145904094	21080	3	0	8	0	0.01767679	0	13	0	0.91308053
chr5	145904094	145969193	65100	1	0	7	0	0.01667721	0	12	0	0.92532268
chr5	145969193	146015634	46442	1	0	7	0	0.03037338	0	11	0	0.73110763
chr5	146015634	146015693	60	1	0	7	0	0.01667721	0	12	0	0.92532268
chr5	146015693	146111506	95814	2	0	6	0	0.00778066	0	12	0	1.17038931
chr5	146111506	146164041	52536	2	0	7	0	0.00859896	0	13	0	1.14735594
chr5	146164041	146295856	131816	3	0	8	0	0.01767679	0	13	0	0.91308053
chr5	146295856	146328248	32393	1	0	7	0	0.00859896	0	13	0	1.14735594
chr5	146328248	146379171	50924	1	0	6	0	0.00373523	0	13	0	1.42397267
chr5	146379171	146416339	37169	2	0	7	0	0.00859896	0	13	0	1.14735594
chr5	146416339	146602755	186417	4	0	8	0	0.01767679	0	13	0	0.91308053
chr5	146602755	146602814	60	1	0	9	0	0.0331093	0	13	0	0.71538971
chr5	146602814	146641446	38633	1	0	8	0	0.01767679	0	13	0	0.91308053
chr5	146641446	146753168	111723	2	0	7	0	0.03037338	0	11	0	0.73110763
chr5	146753168	146753227	60	1	0	8	0	0.03209037	0	12	0	0.72109894
chr5	146753227	146823310	70084	2	0	8	0	0.05490675	0	11	0	0.55623409
chr5	146823310	146823369	60	1	0	8	0	0.03209037	0	12	0	0.72109894
chr5	146823369	146866462	43094	1	0	8	0	0.05490675	0	11	0	0.55623409
chr5	146866462	147092055	225594	4	0	8	0	0.08923391	0	10	0	0.4167287
chr5	147092055	147137334	45280	2	0	9	0	0.0331093	0	13	0	0.71538971
chr5	147137334	147395908	258575	6	0	10	0	0.0574087	0	13	0	0.5498098
chr5	147395908	147765543	369636	8	0	9	0	0.0331093	0	13	0	0.71538971
chr5	147765543	147902806	137264	4	0	9	0	0.01817691	0	14	0	0.90719478
chr5	147902806	147928184	25379	1	0	9	0	0.0331093	0	13	0	0.71538971
chr5	147928184	147975724	47541	1	0	8	0	0.03209037	0	12	0	0.72109894
chr5	147975724	148016535	40812	2	0	8	0	0.01767679	0	13	0	0.91308053
chr5	148016535	148038066	21532	2	0	9	0	0.0331093	0	13	0	0.71538971
chr5	148038066	148102669	64604	1	0	8	0	0.01767679	0	13	0	0.91308053
chr5	148102669	148102728	60	1	0	9	0	0.01817691	0	14	0	0.90719478
chr5	148102728	148207292	104565	2	0	8	0	0.01767679	0	13	0	0.91308053
chr5	148207292	148207351	60	1	0	8	0	0.00909834	0	14	0	1.13428373
chr5	148207351	148208032	682	1	0	7	0	0.00413023	0	14	0	1.39918918
chr5	148208032	148252038	44007	1	0	7	0	0.00859896	0	13	0	1.14735594
chr5	148252038	148252097	60	1	0	8	0	0.01767679	0	13	0	0.91308053
chr5	148252097	148304476	52380	1	0	7	0	0.03037338	0	11	0	0.73110763
chr5	148304476	148420123	115648	2	0	6	0	0.04875589	0	9	0	0.5732208
chr5	148420123	148420182	60	1	0	6	0	0.02793176	0	10	0	0.74627054
chr5	148420182	148488978	68797	2	0	6	0	0.04875589	0	9	0	0.5732208
chr5	148488978	148519668	30691	2	0	7	0	0.03037338	0	11	0	0.73110763
chr5	148519668	148639951	120284	5	0	7	0	0.00859896	0	13	0	1.14735594
chr5	148639951	148640010	60	1	1	8	0.30102999	0.01767679	0	13	0	0.91308053
chr5	148640010	148676818	36809	1	1	7	0.30102999	0.00859896	0	13	0	1.14735594
chr5	148676818	148783744	106927	5	1	6	0.30102999	0.00778066	0	12	0	1.17038931
chr5	148783744	148783803	60	1	1	7	0.30102999	0.00859896	0	13	0	1.14735594
chr5	148783803	148985478	201676	7	0	7	0	0.00859896	0	13	0	1.14735594
chr5	148985478	148985537	60	1	0	8	0	0.01767679	0	13	0	0.91308053
chr5	148985537	149091881	106345	2	0	8	0	0.03209037	0	12	0	0.72109894

chr5	149091881	149091940	60	1	0	8	0	0.01767679	0	13	0	0.91308053
chr5	149091940	149190659	98720	3	0	8	0	0.03209037	0	12	0	0.72109894
chr5	149190659	149190718	60	1	0	8	0	0.01767679	0	13	0	0.91308053
chr5	149190718	149225661	34944	1	0	8	0	0.03209037	0	12	0	0.72109894
chr5	149225661	149238841	13181	2	0	8	0	0.05490675	0	11	0	0.55623409
chr5	149238841	149310691	71851	3	0	8	0	0.03209037	0	12	0	0.72109894
chr5	149310691	149372929	62239	3	0	8	0	0.00909834	0	14	0	1.13428373
chr5	149372929	149456918	83990	2	0	8	0	0.01767679	0	13	0	0.91308053
chr5	149456918	149488198	31281	2	0	8	0	0.00909834	0	14	0	1.13428373
chr5	149488198	149488255	58	1	0	8	0	0.00433294	0	15	0	1.3873209
chr5	149488255	149534421	46167	3	0	8	0	0.00909834	0	14	0	1.13428373
chr5	149534421	149571293	36873	3	0	8	0	0.00433294	0	15	0	1.3873209
chr5	149571293	149601496	30204	2	0	9	0	0.00433294	0	16	0	1.3873209
chr5	149601496	149645528	44033	2	0	9	0	0.00926608	0	15	0	1.13004284
chr5	149645528	149645587	60	1	0	10	0	0.00909834	0	16	0	1.13428373
chr5	149645587	149724544	78958	1	0	10	0	0.01817691	0	15	0	0.90719478
chr5	149724544	149758765	34222	1	0	10	0	0.03344703	0	14	0	0.71353304
chr5	149758765	149779027	20263	3	0	10	0	0.01817691	0	15	0	0.90719478
chr5	149779027	149899981	120955	3	0	10	0	0.0574087	0	13	0	0.5498098
chr5	149899981	149921879	21899	3	0	11	0	0.0574087	0	14	0	0.5498098
chr5	149921879	149933476	11598	1	0	9	0	0.0331093	0	13	0	0.71538971
chr5	149933476	150040974	107499	4	0	9	0	0.01817691	0	14	0	0.90719478
chr5	150040974	150050296	9323	1	0	9	0	0.0565833	0	12	0	0.55190077
chr5	150050296	150070524	20229	2	0	10	0	0.09290028	0	12	0	0.41314172
chr5	150070524	150138814	68291	4	0	10	0	0.0574087	0	13	0	0.5498098
chr5	150138814	150176098	37285	2	0	10	0	0.01817691	0	15	0	0.90719478
chr5	150176098	150280038	103941	2	0	10	0	0.0574087	0	13	0	0.5498098
chr5	150280038	150283559	3522	2	0	11	0	0.0331093	0	15	0	0.71538971
chr5	150283559	150403830	120272	2	0	10	0	0.01817691	0	15	0	0.90719478
chr5	150403830	150459548	55719	3	0	11	0	0.01767679	0	16	0	0.91308053
chr5	150459548	150515017	55470	1	0	11	0	0.0331093	0	15	0	0.71538971
chr5	150515017	150534761	19745	2	0	12	0	0.0565833	0	15	0	0.55190077
chr5	150534761	150604966	70206	4	0	12	0	0.03209037	0	16	0	0.72109894
chr5	150604966	150680422	75457	2	0	12	0	0.0565833	0	15	0	0.55190077
chr5	150680422	150738907	58486	3	0	12	0	0.01667721	0	17	0	0.92532268
chr5	150738907	150861311	122405	2	0	12	0	0.0565833	0	15	0	0.55190077
chr5	150861311	150863648	2338	2	0	12	0	0.03209037	0	16	0	0.72109894
chr5	150863648	150925466	61819	1	0	11	0	0.0331093	0	15	0	0.71538971
chr5	150925466	151007188	81723	2	0	11	0	0.0574087	0	14	0	0.5498098
chr5	151007188	151048914	41727	2	0	11	0	0.0331093	0	15	0	0.71538971
chr5	151048914	151126010	77097	4	0	12	0	0.0565833	0	15	0	0.55190077
chr5	151126010	151154377	28368	1	0	12	0	0.09290028	0	14	0	0.41314172
chr5	151154377	151154436	60	1	0	12	0	0.0565833	0	15	0	0.55190077
chr5	151154436	151179692	25257	1	0	12	0	0.09290028	0	14	0	0.41314172
chr5	151179692	151277117	97426	4	0	12	0	0.0565833	0	15	0	0.55190077
chr5	151277117	151304167	27051	1	0	11	0	0.0574087	0	14	0	0.5498098
chr5	151304167	151328344	24178	2	0	11	0	0.0331093	0	15	0	0.71538971
chr5	151328344	151328403	60	1	0	12	0	0.0565833	0	15	0	0.55190077
chr5	151328403	151451354	122952	1	0	8	0	0.01767679	0	13	0	0.91308053
chr5	151451354	151530718	79365	1	0	8	0	0.08923391	0	10	0	0.4167287
chr5	151530718	151771598	240881	2	0	8	0	0.13872638	0	9	0	0.30102999
chr5	151771598	152026494	254897	2	0	6	0	0.04875589	0	9	0	0.5732208
chr5	152026494	152140747	114254	2	0	8	0	0.08923391	0	10	0	0.4167287
chr5	152140747	152140806	60	1	0	8	0	0.05490675	0	11	0	0.55623409
chr5	152140806	152335779	194974	2	0	6	0	0.08122616	0	8	0	0.4250187
chr5	152335779	152335838	60	1	0	7	0	0.08584816	0	9	0	0.42015402
chr5	152335838	152448325	112488	1	0	5	0	0.04407651	0	8	0	0.58747015
chr5	152448325	152639569	191245	1	0	4	0	0.02074938	0	8	0	0.79906872
chr5	152639569	152639628	60	1	0	5	0	0.01320236	0	10	0	0.97390707
chr5	152639628	152873470	233843	2	0	5	0	0.02473314	0	9	0	0.76806864
chr5	152873470	152951731	78262	2	0	6	0	0.00778066	1	12	0.30102999	1.17038931
chr5	152951731	153134583	182853	3	0	6	0	0.00778066	0	12	0	1.17038931
chr5	153134583	153361800	227218	3	0	6	0	0.01518174	0	11	0	0.9449689
chr5	153361800	153630341	268542	6	0	6	0	0.00778066	0	12	0	1.17038931
chr5	153630341	153735212	104872	2	0	5	0	0.00317045	0	12	0	1.46403142
chr5	153735212	153856715	121504	6	0	6	0	0.00778066	0	12	0	1.17038931
chr5	153856715	154051814	195100	2	0	6	0	0.01518174	0	11	0	0.9449689
chr5	154051814	154130623	78810	2	0	6	0	0.129913	0	7	0	0.30102999
chr5	154130623	154130682	60	1	0	6	0	0.04875589	0	9	0	0.5732208
chr5	154130682	154251303	120622	4	0	3	0	0.01598258	0	7	0	0.84395715
chr5	154251303	154287403	36101	3	0	4	0	0.03812622	0	7	0	0.60763643
chr5	154287403	154364775	77373	3	0	2	0	0.00493743	0	7	0	1.16581773
chr5	154364775	154433622	68848	2	0	3	0	0.01598258	0	7	0	0.84395715
chr5	154433622	154821114	387493	2	0	1	0	0.00204627	0	6	0	1.31360226
chr5	154821114	154821173	60	1	0	3	0	0.01598258	1	7	0.30102999	0.84395715
chr5	154821173	154886233	65061	1	0	2	0	0.02162467	1	5	0.30102999	0.68214471
chr5	154886233	155217429	331197	2	0	2	0	0.0429175	1	4	0.30102999	0.47744371
chr5	155217429	155217488	60	1	0	2	0	0.01053319	1	6	0.30102999	0.91219088
chr5	155217488	155312750	95263	1	0	2	0	0.02162467	1	5	0.30102999	0.68214471
chr5	155312750	155312809	60	1	0	2	0	0.01053319	1	6	0.30102999	0.91219088
chr5	155312809	155471543	158735	2	0	1	0	0.00478973	1	5	0.30102999	1.02643191
chr5	155471543	155702760	231218	3	0	1	0	0.01091641	1	4	0.30102999	0.76005302
chr5	155702760	155771638	68879	2	0	1	0	0.00478973	1	5	0.30102999	1.02643191
chr5	155771638	155771694	57	1	0	2	0	0.02162467	1	5	0.30102999	0.68214471
chr5	155771694	155874457	102764	2	0	2	0	0.0429175	1	4	0.30102999	0.47744371
chr5	155874457	155874516	60	1	0	3	0	0.00378107	1	9	0.30102999	1.33111237
chr5	155874516	156016255	141740	2	0	3	0	0.0079614	1	8	0.30102999	1.07548421
chr5	156016255	156016313	59	1	0	4	0	0.02074938	1	8	0.30102999	0.79906872
chr5	156016313	156126541	110229	2	0	4	0	0.03812622	1	7	0.30102999	0.60763643
chr5	156126541	156187836	61296	2	0	4	0	0.02074938	1	8	0.30102999	0.79906872
chr5	156187836	156696416	508581	13	0	5	0	0.04407651	1	8	0.30102999	0.58747015
chr5	156696416	156822261	125846	4	0	5	0	0.07511598	1	7	0.30102999	0.43181735
chr5	156822261	156904492	82232	3	0	5	0	0.04407651	1	8	0.30102999	0.58747015
chr5	156904492	157105233	200742	5	0	5	0	0.12309572	1	6	0.30102999	0.30102999
chr5	157105233	157105292	60	1	0	5	0	0.07511598	1	7	0.30102999	0.43181735
chr5	157105292	157216337	111046	5	0	5	0	0.12309572	1	6	0.30102999	0.30102999
chr5	157216337	157368536	152200	5	0	5	0	0.07511598	1	7	0.30102999	0.43181735
chr5	157368536	157455904	87369	2	0	5	0	0.04407651	1	8	0.30102999	0.58747015
chr5	157455904	157455963	60	1	1	5	0.1218695	0.04407651	1	8	0.1218695	0.58747015
chr5	157455963	157648230	192268	1	1	5	0.30102999	0.04407651	0	8	0	0.58747015
chr5	157648230	157776310	128081	2	1	6	0.30102999	0.04875589	0	9	0	0.5732208
chr5	157776310	158158808	382499	4	1	5	0.30102999	0.02473314	0	9	0	0.76806864
chr5	158158808	158223377	64570	1	1	5	0.30102999	0.04407651	0	8	0	0.58747015
chr5	158223377	158223430	54	1	1	6	0.30102999	0.08122616	0	8	0	0.4250187
chr5	158223430	158297333	73904	1	0	4	0	0.02074938	0	8	0	0.79906872
chr5	158297333	158333349	36017	2	0	5	0	0.02473314	0	9	0	0.76806864
chr5	158333349	158333408	60	1	1	5	0.30102999	0.01320236	0	10	0	0.97390707
chr5	158333408	158368362	34955	1	0	5	0	0.01320236	0	10	0	0.97390707
chr5	158368362	158401240	32879	2	0	6	0	0.02793176	0	10	0	0.74627054
chr5	158401240	158445611	44372	2	1	6	0.30102999	0.02793176	0	10	0	0.74627054
chr5	158445611	158659917	214307	5	0	5	0	0.01320236	0	10	0	0.97390707
chr5	158659917	158829101	169185	5	1	5	0.30102999	0.01320236	0	10	0	0.97390707
chr5	158829101	158965994	136894	2	1	5	0.30102999	0.00666883	0	11	0	1.20557689
chr5	158965994	159214687	248694	1	1	4	0.30102999	0.00530919	0	10	0	1.2568129
chr5	159214687	159388467	173781	4	1	5	0.30102999	0.01320236	0	10	0	0.97390707
chr5	159388467	159658065	269599	6	0	4	0	0.01077081	0	9	0	1.01542894
chr5	159658065	159734450	76386	2	0	3	0	0.0079614	0	8	0	1.07548421
chr5	159734450	159992942	258493	11	0	4	0	0.01077081	0	9	0	1.01542894
chr5	159992942	159993001	60	1	0	5	0	0.02473314	0	9	0	0.76806864
chr5	159993001	160148716	155716	4	0	4	0	0.02074938	0	8	0	0.79906872
chr5	160148716	160185425	36710	2	0	5	0	0.02473314	0	9	0	0.76806864
chr5	160185425	160185484	60	1	0	6	0	0.00778066	0	12	0	1.17038931
chr5	160185484	160278769	93286	2	0	5	0	0.02473314	0	9	0	0.76806864
chr5	160278769	160278828	60	1	0	6	0	0.00778066	0	12	0	1.17038931

chr5	160278828	160461919	183092	1	0	5	0	0.00317045	0	12	0	1.46403142
chr5	160461919	160622747	160829	1	0	5	0	0.01320236	0	10	0	0.97390707
chr5	160622747	160720878	98132	2	0	6	0	0.02793176	0	10	0	0.74627054
chr5	160720878	160765376	44499	2	0	7	0	0.05232577	0	10	0	0.56314362
chr5	160765376	160827300	61925	1	0	5	0	0.02473314	0	9	0	0.76806864
chr5	160827300	160914332	87033	2	0	5	0	0.04407651	0	8	0	0.58747015
chr5	160914332	161028885	114554	2	0	5	0	0.07511598	0	7	0	0.43181735
chr5	161028885	161116093	87209	2	0	5	0	0.04407651	0	8	0	0.58747015
chr5	161116093	161166374	50282	3	0	5	0	0.02473314	0	9	0	0.76806864
chr5	161166374	161166433	60	1	0	6	0	0.04875589	0	9	0	0.5732208
chr5	161166433	161658087	491655	8	0	5	0	0.04407651	0	8	0	0.58747015
chr5	161658087	161658146	60	1	0	6	0	0.08122616	0	8	0	0.4250187
chr5	161658146	161880865	222720	1	0	5	0	0.12309572	0	6	0	0.30102999
chr5	161880865	161929256	48392	2	0	5	0	0.12309572	1	6	0.30102999	0.30102999
chr5	161929256	162031937	102682	2	0	8	0	0.20764654	1	8	0.30102999	0.20764654
chr5	162031937	162223207	191271	1	0	7	0	0.13499366	1	8	0.30102999	0.30102999
chr5	162223207	162223266	60	1	0	8	0	0.20764654	1	8	0.30102999	0.20764654
chr5	162223266	162373422	150157	1	0	4	0	0.06713722	1	6	0.30102999	0.44141547
chr5	162373422	162688104	314683	2	0	3	0	0.05670724	1	5	0.30102999	0.45545077
chr5	162688104	162688163	60	1	0	4	0	0.11390336	1	5	0.30102999	0.30102999
chr5	162688163	162885689	197527	7	0	3	0	0.05670724	1	5	0.30102999	0.45545077
chr5	162885689	163163839	278151	7	0	3	0	0.03070643	1	6	0.30102999	0.63695542
chr5	163163839	163813766	649928	6	0	5	0	0.12309572	1	6	0.30102999	0.30102999
chr5	163813766	163910547	96782	1	0	2	0	0.0429175	1	4	0.30102999	0.47744371
chr5	163910547	164166144	255598	3	0	0	0	0	1	4	0.30102999	1.26272838
chr5	164166144	164166203	60	1	0	1	0	0.01091641	1	4	0.30102999	0.76005302
chr5	164166203	164828290	662088	5	0	1	0	0.01091641	0	4	0	0.76005302
chr5	164828290	164828349	60	1	0	2	0	0.0429175	0	4	0	0.47744371
chr5	164828349	164973086	144738	1	0	2	0	0.08289318	0	3	0	0.30102999
chr5	164973086	165212295	239210	2	0	0	0	0	0	1	0	0.30102999
chr5	165212295	165402101	189807	2	0	0	0	0	0	2	0	0.61140001
chr5	165768313	166125609	357297	3	0	1	0	0.05404976	0	2	0	0.30102999
chr5	166125609	166385157	259549	2	0	5	0	0.45545077	0	3	0	0.05670724
chr5	166385157	166719422	334266	3	0	5	0	0.45545077	1	3	0.30102999	0.05670724
chr5	166719422	166798936	79515	2	0	5	0	0.30102999	1	4	0.30102999	0.11390336
chr5	166798936	166798995	60	1	0	6	0	0.30102999	1	5	0.30102999	0.12309572
chr5	166798995	166844073	45079	1	0	6	0	0.44141547	1	4	0.30102999	0.06713722
chr5	166844073	166891389	47317	2	0	6	0	0.30102999	1	5	0.30102999	0.12309572
chr5	166891389	166951793	60405	1	0	6	0	0.44141547	1	4	0.30102999	0.06713722
chr5	166951793	167021249	69457	2	0	6	0	0.30102999	1	5	0.30102999	0.12309572
chr5	167021249	167150012	128764	3	0	6	0	0.20064824	1	6	0.30102999	0.20064824
chr5	167150012	167267486	117475	3	0	6	0	0.129913	1	7	0.30102999	0.30102999
chr5	167267486	167361258	93773	2	0	6	0	0.20064824	1	6	0.30102999	0.20064824
chr5	167361258	167565005	203748	5	0	6	0	0.129913	1	7	0.30102999	0.30102999
chr5	167565005	167666325	101321	3	0	7	0	0.20469099	1	7	0.30102999	0.20469099
chr5	167666325	167706507	40183	2	0	8	0	0.20764654	2	8	0.61140001	0.20764654
chr5	167706507	167982982	276476	7	0	8	0	0.30102999	2	7	0.61140001	0.13499366
chr5	167982982	168011390	28409	1	0	8	0	0.30102999	1	7	0.30102999	0.13499366
chr5	168011390	168165013	153624	2	0	7	0	0.20469099	1	7	0.30102999	0.20469099
chr5	168165013	168317542	152530	5	0	8	0	0.30102999	1	7	0.30102999	0.13499366
chr5	168317542	168490985	173444	4	0	7	0	0.20469099	1	7	0.30102999	0.20469099
chr5	168490985	168491044	60	1	0	8	0	0.30102999	1	7	0.30102999	0.13499366
chr5	168491044	168554764	63721	1	0	7	0	0.20469099	1	7	0.30102999	0.20469099
chr5	168554764	168603478	48715	2	0	8	0	0.30102999	1	7	0.30102999	0.13499366
chr5	168603478	168603537	60	1	0	8	0	0.20764654	1	8	0.30102999	0.20764654
chr5	168603537	168693007	89471	3	0	6	0	0.129913	1	7	0.30102999	0.30102999
chr5	168693007	168778608	85602	3	0	7	0	0.13499366	1	8	0.30102999	0.30102999
chr5	168778608	168778667	60	1	0	8	0	0.20764654	1	8	0.30102999	0.20764654
chr5	168778667	168983162	204496	1	0	7	0	0.30102999	1	6	0.30102999	0.129913
chr5	168983162	168983221	60	1	0	7	0	0.30102999	2	6	0.61140001	0.129913
chr5	168983221	169117384	134164	3	0	7	0	0.43181735	2	5	0.61140001	0.07511598
chr5	169117384	169145700	28317	2	0	7	0	0.20469099	2	7	0.61140001	0.20469099
chr5	169145700	169191047	45348	1	0	7	0	0.43181735	2	5	0.61140001	0.07511598
chr5	169191047	169267734	76688	2	0	7	0	0.43181735	1	5	0.30102999	0.07511598
chr5	169267734	169267784	51	1	0	7	0	0.30102999	1	6	0.30102999	0.129913
chr5	169267784	169479290	211507	4	0	7	0	0.43181735	1	5	0.30102999	0.07511598
chr5	169479290	169536555	57266	5	0	7	0	0.43181735	2	5	0.61140001	0.07511598
chr5	169536555	169629965	93411	2	0	6	0	0.44141547	1	4	0.30102999	0.06713722
chr5	169629965	169630023	59	1	0	7	0	0.30102999	1	6	0.30102999	0.129913
chr5	169630023	169675170	45148	1	0	6	0	0.20064824	1	6	0.30102999	0.20064824
chr5	169675170	169708041	32872	2	0	6	0	0.44141547	1	4	0.30102999	0.06713722
chr5	169708041	169708085	45	1	0	6	0	0.30102999	2	5	0.61140001	0.12309572
chr5	169708085	169763048	54964	1	0	6	0	0.44141547	1	4	0.30102999	0.06713722
chr5	169763048	169849631	86584	4	0	6	0	0.30102999	1	5	0.30102999	0.12309572
chr5	169849631	170109334	259704	6	0	8	0	0.58747015	2	5	0.61140001	0.04407651
chr5	170109334	170214178	104845	2	0	7	0	0.43181735	1	5	0.30102999	0.07511598
chr5	170214178	170214237	60	1	0	7	0	0.43181735	2	5	0.61140001	0.07511598
chr5	170214237	170262676	48440	2	0	6	0	0.30102999	1	5	0.30102999	0.12309572
chr5	170262676	170319564	56889	2	0	7	0	0.20469099	2	7	0.61140001	0.20469099
chr5	170319564	170319623	60	1	0	7	0	0.20469099	3	7	0.93173516	0.20469099
chr5	170319623	170470509	150887	3	0	6	0	0.20064824	3	6	0.93173516	0.20064824
chr5	170470509	170496921	26413	1	0	6	0	0.44141547	3	4	0.93173516	0.06713722
chr5	170496921	170570384	73464	1	0	5	0	0.30102999	2	4	0.61140001	0.11390336
chr5	170570384	170570443	60	1	0	5	0	0.12309572	2	6	0.61140001	0.30102999
chr5	170570443	170726981	156539	3	0	4	0	0.18734596	2	4	0.61140001	0.18734596
chr5	170726981	170737914	10934	2	0	6	0	0.30102999	2	5	0.61140001	0.12309572
chr5	170737914	170820185	82272	1	0	6	0	0.44141547	2	4	0.61140001	0.06713722
chr5	170820185	170834717	14533	4	0	7	0	0.60763643	2	4	0.61140001	0.03812622
chr5	170834717	170881134	46418	2	0	6	0	0.44141547	2	4	0.61140001	0.06713722
chr5	170881134	170881185	52	1	0	7	0	0.60763643	2	4	0.61140001	0.03812622
chr5	170881185	170963376	82192	1	0	7	0	0.84395715	2	3	0.61140001	0.01598258
chr5	170963376	170963435	60	1	0	7	0	0.84395715	3	3	0.93173516	0.01598258
chr5	170963435	171149889	186455	1	0	5	0	0.45545077	2	3	0.61140001	0.05670724
chr5	171149889	171239417	89529	1	0	5	0	0.45545077	1	3	0.30102999	0.05670724
chr5	171239417	171343555	104139	2	0	5	0	0.45545077	0	3	0	0.05670724
chr5	171343555	171384561	41007	3	0	7	0	0.60763643	0	4	0	0.03812622
chr5	171384561	171469909	85349	2	0	6	0	0.63695542	0	3	0	0.03070643
chr5	171469909	171469968	60	1	0	6	0	0.44141547	0	4	0	0.06713722
chr5	171469968	171503864	33897	1	0	5	0	0.30102999	0	4	0	0.11390336
chr5	171503864	171503923	60	1	0	6	0	0.44141547	1	4	0.30102999	0.06713722
chr5	171503923	171561208	57286	1	0	6	0	0.63695542	1	3	0.30102999	0.03070643
chr5	171561208	171561267	60	1	0	7	0	0.84395715	1	3	0.30102999	0.01598258
chr5	171561267	171612630	51364	1	0	6	0	0.63695542	1	3	0.30102999	0.03070643
chr5	171612630	171671174	58545	2	0	6	0	0.91219088	0	2	0	0.01053319
chr5	171671174	171761860	90687	3	0	6	0	0.63695542	1	3	0.30102999	0.03070643
chr5	171761860	171810211	48352	3	0	6	0	0.63695542	2	3	0.61140001	0.03070643
chr5	171810211	171962738	152528	2	0	6	0	0.91219088	1	2	0.30102999	0.01053319
chr5	171962738	172075680	112943	1	0	5	0	0.68214471	1	2	0.30102999	0.02162467
chr5	172075680	172281000	205321	5	0	6	0	0.91219088	1	2	0.30102999	0.01053319
chr5	172281000	172333670	52671	2	0	4	0	0.47744371	1	2	0.30102999	0.0429175
chr5	172333670	172379518	45849	4	0	5	0	0.68214471	1	2	0.30102999	0.02162467
chr5	172379518	172412610	33093	4	0	7	0	1.16581773	1	2	0.30102999	0.00493743
chr5	172412610	172443883	31274	1	0	6	0	0.91219088	1	2	0.30102999	0.01053319
chr5	172443883	172518724	74842	2	0	5	0	0.68214471	1	2	0.30102999	0.02162467
chr5	172518724	172693535	174812	6	0	7	0	1.16581773	1	2	0.30102999	0.00493743
chr5	172693535	172752386	58852	3	0	6	0	0.91219088	0	2	0	0.01053319
chr5	172752386	172779361	26976	1	0	5	0	0.68214471	0	2	0	0.02162467
chr5	172779361	173266253	486893	6	0	4	0	0.47744371	0	2	0	0.0429175
chr5	173266253	173266312	60	1	0	4	0	0.47744371	1	2	0.30102999	0.0429175
chr5	173266312	173320205	53894	2	0	4	0	0.47744371	0	2	0	0.0429175
chr5	173320205	173387338	67134	2	0	4	0	1.26272838	0	0	0	0
chr5	173387338	173387397	60	1	0	4	0	0.47744371	0	2	0	0.0429175
chr5	173387397	173453764	66368	1	0	4	0	0.76005302	0	1	0	0.01091641
chr5	173453764	173515045	61282	2	0	3	0	0.51676182	0	1	0	0.02438896

chr5	173515045	173515104	60	1	0	5	0	0.68214471	0	2	0	0.02162467
chr5	173515104	173534799	19696	1	0	3	0	0.51676182	0	1	0	0.02438896
chr5	173534799	173656118	121320	1	0	2	0	0.30102999	0	1	0	0.05404976
chr5	173656118	173656177	60	1	0	3	0	0.51676182	0	1	0	0.02438896
chr5	173656177	173912734	256558	2	0	2	0	0.30102999	0	1	0	0.05404976
chr5	173912734	173912793	60	1	0	4	0	0.30102999	0	3	0	0.10122019
chr5	173912793	174034345	121553	1	0	3	0	0.17593012	0	3	0	0.17593012
chr5	174034345	174034404	60	1	0	3	0	0.05670724	0	5	0	0.45545077
chr5	174034404	174153419	119016	1	0	3	0	0.30102999	0	2	0	0.08289318
chr5	174153419	174154900	1482	2	0	3	0	0.30102999	1	2	0.30102999	0.08289318
chr5	174154900	174226486	71587	1	0	2	0	0.1575501	1	2	0.30102999	0.1575501
chr5	174226486	174375130	148645	2	0	2	0	0.02162467	1	5	0.30102999	0.68214471
chr5	174375130	174407546	32417	2	1	2	0.1218695	0.00493743	1	7	0.1218695	1.16581773
chr5	174407546	174481609	74064	1	1	2	0.1218695	0.01053319	1	6	0.1218695	0.91219088
chr5	174481609	174481668	60	1	1	3	0.1218695	0.03070643	1	6	0.1218695	0.63695542
chr5	174481668	174849572	367905	2	1	2	0.30102999	0.08289318	0	3	0	0.30102999
chr5	174849572	174868099	18528	2	1	3	0.30102999	0.01598258	0	7	0	0.84395715
chr5	174868099	174954512	86414	3	1	3	0.30102999	0.05670724	0	5	0	0.45545077
chr5	174954512	174954571	60	1	1	3	0.30102999	0.03070643	0	6	0	0.63695542
chr5	174954571	175027689	73119	2	1	3	0.30102999	0.05670724	0	5	0	0.45545077
chr5	175027689	175109981	82293	2	1	4	0.30102999	0.03812622	0	7	0	0.60763643
chr5	175109981	175110040	60	1	1	5	0.30102999	0.07511598	0	7	0	0.43181735
chr5	175110040	175225949	115910	2	1	4	0.30102999	0.03812622	0	7	0	0.60763643
chr5	175225949	175226008	60	1	1	5	0.30102999	0.07511598	0	7	0	0.43181735
chr5	175226008	175273482	47475	2	1	4	0.30102999	0.03812622	0	7	0	0.60763643
chr5	175273482	175273541	60	1	1	5	0.30102999	0.07511598	0	7	0	0.43181735
chr5	175273541	175437847	164307	2	1	4	0.30102999	0.03812622	0	7	0	0.60763643
chr5	175437847	175437906	60	1	2	5	0.30102999	0.07511598	1	7	0.05404976	0.43181735
chr5	175437906	175576602	138697	1	1	5	0.1218695	0.07511598	1	7	0.1218695	0.43181735
chr5	175576602	175576661	60	1	1	6	0.1218695	0.08122616	1	8	0.1218695	0.4250187
chr5	175576661	175719197	142537	1	1	6	0.1218695	0.129913	1	7	0.1218695	0.30102999
chr5	175719197	175719256	60	1	1	7	0.05404976	0.20469099	2	7	0.30102999	0.20469099
chr5	175719256	175775326	56071	1	1	6	0.05404976	0.129913	2	7	0.30102999	0.30102999
chr5	175775326	175779383	4058	2	3	6	0.30102999	0.129913	2	7	0.08289318	0.30102999
chr5	175779383	175793937	14555	3	3	7	0.30102999	0.20469099	2	7	0.08289318	0.20469099
chr5	175793937	175816210	22274	1	2	7	0.1575501	0.20469099	2	7	0.1575501	0.20469099
chr5	175816210	175890496	74287	3	1	7	0.05404976	0.20469099	2	7	0.30102999	0.20469099
chr5	175890496	175890555	60	1	3	7	0.30102999	0.20469099	2	7	0.08289318	0.20469099
chr5	175890555	175936428	45874	1	3	6	0.30102999	0.129913	2	7	0.08289318	0.30102999
chr5	175936428	175936487	60	1	5	6	0.45545077	0.04875589	3	9	0.05670724	0.5732208
chr5	175936487	175974728	38242	2	5	5	0.45545077	0.02473314	3	9	0.05670724	0.76806864
chr5	175974728	176013026	38299	2	5	5	0.45545077	0.01320236	3	10	0.05670724	0.97390707
chr5	176013026	176033642	20617	2	5	5	0.45545077	0.02473314	3	9	0.05670724	0.76806864
chr5	176033642	176033692	51	1	5	6	0.45545077	0.02793176	3	10	0.05670724	0.74627054
chr5	176033692	176056299	22608	2	4	6	0.30102999	0.02793176	3	10	0.10122019	0.74627054
chr5	176056299	176127129	70831	1	4	6	0.30102999	0.04875589	3	9	0.10122019	0.5732208
chr5	176127129	176178201	51073	2	4	7	0.30102999	0.08584816	3	9	0.10122019	0.42015402
chr5	176178201	176267486	89286	1	3	5	0.30102999	0.12309572	2	6	0.08289318	0.30102999
chr5	176267486	176309698	42213	1	3	5	0.30102999	0.19510895	2	5	0.08289318	0.19510895
chr5	176309698	176309749	52	1	4	5	0.47744371	0.19510895	2	5	0.0429175	0.19510895
chr5	176309749	176311879	2131	1	3	5	0.30102999	0.19510895	2	5	0.08289318	0.19510895
chr5	176311879	176409575	97697	4	3	5	0.30102999	0.12309572	2	6	0.08289318	0.30102999
chr5	176409575	176409634	60	1	4	5	0.47744371	0.12309572	2	6	0.0429175	0.30102999
chr5	176409634	176433008	23375	1	3	5	0.30102999	0.12309572	2	6	0.08289318	0.30102999
chr5	176433008	176488608	55601	2	3	5	0.30102999	0.07511598	2	7	0.08289318	0.43181735
chr5	176488608	176516236	27629	3	5	5	0.68214471	0.04407651	2	8	0.02162467	0.58747015
chr5	176516236	176563025	46790	1	4	5	0.47744371	0.04407651	2	8	0.0429175	0.58747015
chr5	176563025	176637907	74883	2	4	5	0.47744371	0.07511598	2	7	0.0429175	0.43181735
chr5	176637907	176637966	60	1	4	6	0.47744371	0.04875589	2	9	0.0429175	0.5732208
chr5	176637966	176662911	24946	1	3	6	0.30102999	0.04875589	2	9	0.08289318	0.5732208
chr5	176662911	176729171	66261	1	3	6	0.30102999	0.08122616	2	8	0.08289318	0.4250187
chr5	176729171	176776603	47433	3	4	6	0.30102999	0.08122616	3	8	0.10122019	0.4250187
chr5	176776603	176776662	60	1	4	6	0.30102999	0.04875589	3	9	0.10122019	0.5732208
chr5	176776662	176816297	39636	2	3	6	0.17593012	0.04875589	3	9	0.17593012	0.5732208
chr5	176816297	176866053	49757	2	3	6	0.17593012	0.08122616	3	8	0.17593012	0.4250187
chr5	176866053	176883275	17223	2	3	6	0.17593012	0.04875589	3	9	0.17593012	0.5732208
chr5	176883275	176904798	21524	2	3	5	0.30102999	0.02473314	2	9	0.08289318	0.76806864
chr5	176904798	177013902	109105	2	3	5	0.30102999	0.07511598	2	7	0.08289318	0.43181735
chr5	177013902	177013961	60	1	3	5	0.30102999	0.04407651	2	8	0.08289318	0.58747015
chr5	177013961	177107471	93511	2	3	5	0.30102999	0.07511598	2	7	0.08289318	0.43181735
chr5	177107471	177297107	189637	2	3	5	0.30102999	0.12309572	2	6	0.08289318	0.30102999
chr5	177297107	177421886	124780	2	2	5	0.1575501	0.12309572	2	6	0.1575501	0.30102999
chr5	177421886	177422760	875	2	3	5	0.17593012	0.12309572	3	6	0.17593012	0.30102999
chr5	177422760	177537467	114708	1	2	5	0.1575501	0.12309572	2	6	0.1575501	0.30102999
chr5	177537467	177569245	31779	2	2	5	0.1575501	0.07511598	2	7	0.1575501	0.43181735
chr5	177569245	177756155	186911	8	2	6	0.1575501	0.129913	2	7	0.1575501	0.30102999
chr5	177756155	177832393	76239	2	2	6	0.1575501	0.08122616	2	8	0.1575501	0.4250187
chr5	177832393	177832452	60	1	3	6	0.30102999	0.08122616	2	8	0.08289318	0.4250187
chr5	177832452	177870827	38376	1	2	6	0.1575501	0.129913	2	7	0.1575501	0.30102999
chr5	177870827	177870886	60	1	2	7	0.1575501	0.20469099	2	7	0.1575501	0.20469099
chr5	177870886	177913026	42141	1	2	7	0.30102999	0.30102999	1	6	0.05404976	0.129913
chr5	177913026	177956938	43913	1	2	6	0.30102999	0.30102999	1	5	0.05404976	0.12309572
chr5	177956938	178038828	81891	1	2	6	0.30102999	0.44141547	1	4	0.05404976	0.06713722
chr5	178038828	178078984	40157	3	2	6	0.30102999	0.30102999	1	5	0.05404976	0.12309572
chr5	178078984	178079043	60	1	2	6	0.30102999	0.20064824	1	6	0.05404976	0.20064824
chr5	178079043	178177337	98295	2	2	6	0.30102999	0.30102999	1	5	0.05404976	0.12309572
chr5	178177337	178231925	54589	2	2	6	0.1575501	0.20064824	2	6	0.1575501	0.20064824
chr5	178231925	178231984	60	1	2	6	0.1575501	0.04875589	2	9	0.1575501	0.5732208
chr5	178231984	178290142	58159	1	2	6	0.30102999	0.04875589	1	9	0.05404976	0.5732208
chr5	178290142	178290201	60	1	2	7	0.30102999	0.03037338	1	11	0.05404976	0.73110763
chr5	178290201	178358506	68306	1	2	6	0.30102999	0.01518174	1	11	0.05404976	0.9449689
chr5	178358506	178392976	34471	1	2	6	0.30102999	0.04875589	1	9	0.05404976	0.5732208
chr5	178392976	178415365	22390	2	2	7	0.30102999	0.08584816	1	9	0.05404976	0.42015402
chr5	178415365	178415423	59	1	2	7	0.30102999	0.05232577	1	10	0.05404976	0.56314362
chr5	178415423	178457067	41645	1	2	6	0.30102999	0.02793176	1	10	0.05404976	0.74627054
chr5	178457067	178507278	50212	3	2	6	0.1575501	0.02793176	2	10	0.1575501	0.74627054
chr5	178507278	178507337	60	1	2	7	0.1575501	0.03037338	2	11	0.1575501	0.73110763
chr5	178507337	178545281	37945	1	2	7	0.1575501	0.08584816	2	9	0.1575501	0.42015402
chr5	178545281	178597059	51779	1	2	6	0.1575501	0.04875589	2	9	0.1575501	0.5732208
chr5	178597059	178597118	60	1	2	6	0.1575501	0.02793176	2	10	0.1575501	0.74627054
chr5	178597118	178669297	72180	2	2	6	0.1575501	0.08122616	2	8	0.1575501	0.4250187
chr5	178669297	178727850	58554	2	2	7	0.1575501	0.13499366	2	8	0.1575501	0.30102999
chr5	178727850	178830351	102502	2	2	6	0.1575501	0.129913	2	7	0.1575501	0.30102999
chr5	178830351	178830410	60	1	3	6	0.30102999	0.04875589	2	9	0.08289318	0.5732208
chr5	178830410	178917535	87126	1	2	6	0.1575501	0.04875589	2	9	0.1575501	0.5732208
chr5	178917535	178917594	60	1	2	7	0.1575501	0.05232577	2	10	0.1575501	0.56314362
chr5	178917594	179048214	130621	2	2	5	0.1575501	0.02473314	2	9	0.1575501	0.76806864
chr5	179048214	179123736	75523	2	2	5	0.1575501	0.01320236	2	10	0.1575501	0.97390707
chr5	179123736	179165418	41683	2	2	5	0.30102999	0.01320236	1	10	0.05404976	0.97390707
chr5	179165418	179222229	56812	3	2	6	0.1575501	0.02793176	2	10	0.1575501	0.74627054
chr5	179222229	179323908	101680	8	2	7	0.1575501	0.03037338	2	11	0.1575501	0.73110763
chr5	179323908	179382640	58733	1	2	6	0.1575501	0.02793176	2	10	0.1575501	0.74627054
chr5	179382640	179422255	39616	1	2	5	0.1575501	0.01320236	2	10	0.1575501	0.97390707
chr5	179422255	179446567	24313	2	2	5	0.1575501	0.00666883	2	11	0.1575501	1.20557689
chr5	179446567	179446626	60	1	2	6	0.1575501	0.01518174	2	11	0.1575501	0.9449689
chr5	179446626	179467521	20896	1	2	5	0.1575501	0.00666883	2	11	0.1575501	1.20557689
chr5	179467521	179491865	24345	1	2	5	0.1575501	0.01320236	2	10	0.1575501	0.97390707
chr5	179491865	179541490	49626	2	2	5	0.1575501	0.02473314	2	9	0.1575501	0.76806864
chr5	179541490	179541544	55	1	2	5	0.1575501	0.01320236	2	10	0.1575501	0.97390707
chr5	179541544	179621281	79738	2	2	5	0.1575501	0.02473314	2	9	0.1575501	0.76806864
chr5	179621281	179621340	60	1	2	5	0.1575501	0.01320236	2	10	0.1575501	0.97390707
chr5	179621340	179698774	77435	2	2	5	0.1575501	0.04407651	2	8	0.1575501	0.58747015
chr5	179698774	179807137	108364	4	2	5	0.1575501	0.02473314	2	9	0.1575501	0.76806864
chr5	179807137	179878423	71287	1	2	5	0.1575501	0.04407651	2	8	0.1575501	0.58747015

chr5	179878423	179922418	43996	2	2	5	0.1575501	0.02473314	2	9	0.1575501	0.76806864
chr5	179922418	180031741	109324	4	2	6	0.1575501	0.02793176	2	10	0.1575501	0.74627054
chr5	180031741	180040671	8931	1	2	6	0.1575501	0.04875589	2	9	0.1575501	0.5732208
chr5	180040671	180075444	34774	1	2	5	0.1575501	0.04407651	2	8	0.1575501	0.58747015
chr5	180075444	180152467	77024	2	2	5	0.1575501	0.02473314	2	9	0.1575501	0.76806864
chr5	180152467	180235736	83270	3	2	5	0.1575501	0.04407651	2	8	0.1575501	0.58747015
chr5	180235736	180481959	246224	2	2	4	0.30102999	0.06713722	1	6	0.05404976	0.44141547
chr5	180481959	180482018	60	1	2	5	0.30102999	0.12309572	1	6	0.05404976	0.30102999
chr5	180482018	180487584	5567	2	2	5	0.30102999	0.19510895	1	5	0.05404976	0.19510895
chr5	180487584	180487643	60	1	2	5	0.30102999	0.12309572	1	6	0.05404976	0.30102999
chr5	180487643	180552404	64762	1	1	4	0.1218695	0.06713722	1	6	0.1218695	0.44141547
chr5	180552404	180626328	73925	2	1	4	0.1218695	0.11390336	1	5	0.1218695	0.30102999
chr5	180626328	180652442	26115	2	1	4	0.1218695	0.06713722	1	6	0.1218695	0.44141547
chr5	180652442	180684501	32060	1	0	4	0	0.06713722	1	6	0.30102999	0.44141547
chr6	170426	255350	84925	2	2	0	0.08289318	0	3	1	0.30102999	0.30102999
chr6	255350	588213	332864	7	3	0	0.10122019	0	4	2	0.30102999	0.61140001
chr6	588213	732288	144076	3	2	0	0.08289318	0	3	2	0.30102999	0.61140001
chr6	732288	980386	248099	5	3	0	0.10122019	0	4	2	0.30102999	0.61140001
chr6	980386	1057094	76709	2	3	0	0.05670724	0	5	2	0.45545077	0.61140001
chr6	1057094	1057153	60	1	4	0	0.11390336	0	5	2	0.30102999	0.61140001
chr6	1057153	1106749	49597	1	3	0	0.05670724	0	5	2	0.45545077	0.61140001
chr6	1106749	1369777	263029	4	3	0	0.03070643	0	6	2	0.63695542	0.61140001
chr6	1369777	1470685	100909	2	4	0	0.02074938	0	8	2	0.79906872	0.61140001
chr6	1470685	1691836	221152	3	4	0	0.03812622	0	7	2	0.60763643	0.61140001
chr6	1691836	1911795	219960	4	3	0	0.01598258	0	7	2	0.84395715	0.61140001
chr6	1911795	1911854	60	1	3	1	0.01598258	0.05404976	7	2	0.84395715	0.30102999
chr6	1911854	1960845	48992	1	3	0	0.01598258	0	7	2	0.84395715	0.61140001
chr6	1960845	1960904	60	1	4	0	0.03812622	0	7	2	0.60763643	0.61140001
chr6	1960904	2155812	194909	4	4	0	0.06713722	0	6	2	0.44141547	0.61140001
chr6	2155812	2232459	76648	1	3	0	0.03070643	0	6	2	0.63695542	0.61140001
chr6	2232459	2308517	76059	2	3	1	0.03070643	0.05404976	6	2	0.63695542	0.30102999
chr6	2308517	2380300	71784	1	2	1	0.01053319	0.05404976	6	2	0.91219088	0.30102999
chr6	2380300	2435118	54819	2	3	1	0.03070643	0.05404976	6	2	0.63695542	0.30102999
chr6	2435118	2516240	81123	1	3	1	0.05670724	0.05404976	5	2	0.45545077	0.30102999
chr6	2516240	2634208	117969	3	4	1	0.11390336	0.05404976	5	2	0.30102999	0.30102999
chr6	2634208	2893856	259649	6	4	1	0.06713722	0.05404976	6	2	0.44141547	0.30102999
chr6	2893856	2977261	83406	1	4	1	0.06713722	0.1218695	6	1	0.44141547	0.1218695
chr6	2977261	2977320	60	1	5	1	0.12309572	0.1218695	6	1	0.30102999	0.1218695
chr6	2977320	3153933	176614	3	5	0	0.19510895	0	5	1	0.19510895	0.30102999
chr6	3153933	3153992	60	1	5	0	0.07511598	0	7	1	0.43181735	0.30102999
chr6	3153992	3224544	70553	2	5	0	0.19510895	0	5	1	0.19510895	0.30102999
chr6	3224544	3224603	60	1	5	1	0.12309572	0.1218695	6	1	0.30102999	0.1218695
chr6	3224603	3290680	66078	1	5	0	0.19510895	0	5	1	0.19510895	0.30102999
chr6	3290680	3369564	78885	1	5	0	0.30102999	0	4	1	0.11390336	0.30102999
chr6	3369564	3437192	67629	2	5	0	0.19510895	0	5	1	0.19510895	0.30102999
chr6	3437192	3527033	89842	2	5	0	0.12309572	0	6	1	0.30102999	0.30102999
chr6	3527033	3527092	60	1	5	0	0.07511598	0	7	1	0.43181735	0.30102999
chr6	3527092	3794072	266981	3	4	0	0.03812622	0	7	1	0.60763643	0.30102999
chr6	3794072	3849877	55806	2	4	0	0.02074938	0	8	1	0.79906872	0.30102999
chr6	3849877	3898778	48902	1	3	0	0.0079614	0	8	1	1.07548421	0.30102999
chr6	3898778	3939209	40432	1	3	0	0.01598258	0	7	1	0.84395715	0.30102999
chr6	3939209	4145434	206226	4	4	0	0.03812622	0	7	1	0.60763643	0.30102999
chr6	4145434	4208273	62840	2	5	0	0.04407651	0	8	1	0.58747015	0.30102999
chr6	4208273	4436059	227787	3	5	0	0.07511598	0	7	1	0.43181735	0.30102999
chr6	4436059	4436118	60	1	6	0	0.08122616	0	8	1	0.4250187	0.30102999
chr6	4436118	4489797	53680	1	5	0	0.04407651	0	8	1	0.58747015	0.30102999
chr6	4489797	4674674	184878	4	5	0	0.02473314	0	9	1	0.76806864	0.30102999
chr6	4674674	4755760	81087	2	6	0	0.04875589	0	9	1	0.5732208	0.30102999
chr6	4755760	4791460	35701	2	7	0	0.08584816	0	9	1	0.42015402	0.30102999
chr6	4791460	4996186	204727	3	7	0	0.20469099	0	7	1	0.20469099	0.30102999
chr6	4996186	5114122	117937	2	5	0	0.07511598	0	7	1	0.43181735	0.30102999
chr6	5114122	5114181	60	1	5	0	0.04407651	0	8	1	0.58747015	0.30102999
chr6	5114181	5244107	129927	3	4	0	0.02074938	0	8	1	0.79906872	0.30102999
chr6	5244107	5354461	110355	3	6	0	0.08122616	0	8	1	0.4250187	0.30102999
chr6	5354461	5431389	76929	1	5	0	0.04407651	0	8	1	0.58747015	0.30102999
chr6	5431389	5541120	109732	3	6	0	0.08122616	0	8	1	0.4250187	0.30102999
chr6	5541120	5541179	60	1	7	0	0.13499366	0	8	1	0.30102999	0.30102999
chr6	5541179	5658806	117628	2	7	0	0.20469099	0	7	1	0.20469099	0.30102999
chr6	5658806	5793644	134839	3	7	0	0.30102999	0	6	1	0.129913	0.30102999
chr6	5793644	6062974	269331	4	8	0	0.4250187	0	6	1	0.08122616	0.30102999
chr6	6062974	6063033	60	1	10	0	0.74627054	0	6	1	0.02793176	0.30102999
chr6	6063033	6167357	104325	1	9	0	0.5732208	0	6	1	0.04875589	0.30102999
chr6	6167357	6204433	37077	2	9	0	0.30102999	0	8	1	0.13872638	0.30102999
chr6	6204433	6321984	117552	2	8	0	0.30102999	0	7	1	0.13499366	0.30102999
chr6	6321984	6361756	39773	2	8	0	0.20764654	0	8	1	0.20764654	0.30102999
chr6	6361756	6464734	102979	2	9	0	0.30102999	0	8	1	0.13872638	0.30102999
chr6	6464734	6849238	384505	6	8	0	0.30102999	0	7	1	0.13499366	0.30102999
chr6	6849238	6849297	60	1	8	0	0.20764654	0	8	1	0.20764654	0.30102999
chr6	6849297	6935627	86331	1	8	0	0.30102999	0	7	1	0.13499366	0.30102999
chr6	6935627	7108164	172538	1	7	0	0.30102999	0	6	1	0.129913	0.30102999
chr6	7108164	7210686	102523	3	7	1	0.30102999	0.1218695	6	1	0.129913	0.1218695
chr6	7210686	7621929	411244	6	7	0	0.30102999	0	6	1	0.129913	0.30102999
chr6	7621929	7621988	60	1	7	0	0.20469099	0	7	1	0.20469099	0.30102999
chr6	7621988	7725598	103611	1	7	0	0.20469099	0	7	0	0.20469099	0
chr6	7725598	7797658	72061	1	6	0	0.20064824	0	6	0	0.20064824	0
chr6	7797658	7849301	51644	2	7	0	0.20469099	0	7	0	0.20469099	0
chr6	7849301	7957626	108326	2	6	0	0.30102999	0	5	0	0.12309572	0
chr6	7957626	8101948	144323	4	6	0	0.20064824	0	6	0	0.20064824	0
chr6	8101948	8195167	93220	1	5	0	0.30102999	0	4	0	0.11390336	0
chr6	8195167	8195226	60	1	5	0	0.30102999	0	4	1	0.11390336	0.30102999
chr6	8195226	8434586	239361	2	5	0	0.45545077	0	3	1	0.05670724	0.30102999
chr6	8434586	8548707	114122	1	4	0	0.30102999	0	3	1	0.10122019	0.30102999
chr6	8548707	8548766	60	1	4	1	0.30102999	0.05404976	3	2	0.10122019	0.30102999
chr6	8548766	8749552	200787	2	3	1	0.30102999	0.1218695	2	1	0.08289318	0.1218695
chr6	8749552	8866195	116644	1	3	1	0.30102999	0.30102999	2	0	0.08289318	0
chr6	8866195	9472850	606656	1	3	0	0.30102999	0	2	0	0.08289318	0
chr6	9472850	9472909	60	1	4	1	0.18734596	0.1218695	4	1	0.18734596	0.1218695
chr6	9472909	9621501	148593	1	4	1	0.30102999	0.1218695	3	1	0.10122019	0.1218695
chr6	9621501	9621560	60	1	4	2	0.18734596	0.30102999	4	1	0.18734596	0.05404976
chr6	9621560	9734281	112722	1	4	2	0.18734596	0.61140001	4	0	0.18734596	0
chr6	9734281	9734340	60	1	5	2	0.30102999	0.61140001	4	0	0.11390336	0
chr6	9734340	9826171	91832	2	4	2	0.18734596	0.61140001	4	0	0.18734596	0
chr6	9826171	9826230	60	1	6	2	0.44141547	0.30102999	4	1	0.06713722	0.05404976
chr6	9826230	9866302	40073	1	6	2	0.44141547	0.61140001	4	0	0.06713722	0
chr6	9866302	9866361	60	1	6	2	0.30102999	0.61140001	5	0	0.12309572	0
chr6	9866361	9900601	34241	1	6	2	0.44141547	0.61140001	4	0	0.06713722	0
chr6	9900601	9977741	77141	2	6	1	0.44141547	0.30102999	4	0	0.06713722	0
chr6	9977741	9997843	20103	2	6	2	0.20064824	0.30102999	6	1	0.20064824	0.05404976
chr6	9997843	10044791	46949	1	6	2	0.44141547	0.30102999	4	1	0.06713722	0.05404976
chr6	10044791	10211683	166893	4	6	2	0.44141547	0.1575501	4	2	0.06713722	0.1575501
chr6	10211683	10400666	188984	3	6	2	0.129913	0.1575501	7	2	0.30102999	0.1575501
chr6	10400666	10586358	185693	7	6	2	0.08122616	0.1575501	8	2	0.4250187	0.1575501
chr6	10586358	10633187	46830	2	6	2	0.02793176	0.1575501	10	2	0.74627054	0.1575501
chr6	10633187	10633246	60	1	6	2	0.01518174	0.1575501	11	2	0.9449689	0.1575501
chr6	10633246	10707685	74440	2	6	2	0.02793176	0.1575501	10	2	0.74627054	0.1575501
chr6	10707685	10731042	23358	1	6	2	0.04875589	0.1575501	9	2	0.5732208	0.1575501
chr6	10731042	10731096	55	1	6	2	0.02793176	0.1575501	10	2	0.74627054	0.1575501
chr6	10731096	10803947	72852	2	6	2	0.04875589	0.1575501	9	2	0.5732208	0.1575501
chr6	10803947	10804006	60	1	6	2	0.02793176	0.1575501	10	2	0.74627054	0.1575501
chr6	10804006	10961538	157533	4	6	2	0.04875589	0.1575501	9	2	0.5732208	0.1575501
chr6	10961538	10961597	60	1	6	2	0.02793176	0.1575501	10	2	0.74627054	0.1575501
chr6	10961597	11002644	41048	1	6	2	0.02793176	0.30102999	10	1	0.74627054	0.05404976
chr6	11002644	11083745	81102	2	6	2	0.08122616	0.30102999	8	1	0.4250187	0.05404976
chr6	11083745	11083804	60	1	6	2	0.02793176	0.30102999	10	1	0.74627054	0.05404976

chr6	11083804	11210276	126473	2	5	2	0.07511598	0.30102999	7	1	0.43181735	0.05404976
chr6	11210276	11210335	60	1	6	2	0.04875589	0.1575501	9	2	0.5732208	0.1575501
chr6	11210335	11244604	34270	1	5	2	0.07511598	0.30102999	7	1	0.43181735	0.05404976
chr6	11244604	11327614	83011	1	5	1	0.07511598	0.1218695	7	1	0.43181735	0.1218695
chr6	11327614	11327673	60	1	6	1	0.04875589	0.1218695	9	1	0.5732208	0.1218695
chr6	11327673	11891911	564239	10	5	1	0.07511598	0.1218695	7	1	0.43181735	0.1218695
chr6	11891911	12164735	272825	4	5	1	0.19510895	0.30102999	5	0	0.19510895	0
chr6	12164735	12296224	131490	5	5	1	0.12309572	0.30102999	6	0	0.30102999	0
chr6	12296224	12405000	108777	2	5	1	0.19510895	0.30102999	5	0	0.19510895	0
chr6	12405000	12405059	60	1	5	1	0.07511598	0.30102999	7	0	0.43181735	0
chr6	12405059	12581384	176326	2	5	1	0.12309572	0.30102999	6	0	0.30102999	0
chr6	12581384	12581443	60	1	5	1	0.12309572	0.1218695	6	1	0.30102999	0.1218695
chr6	12581443	12719161	137719	1	5	1	0.19510895	0.1218695	5	1	0.19510895	0.1218695
chr6	12719161	12775509	56349	2	5	1	0.12309572	0.1218695	6	1	0.30102999	0.1218695
chr6	12775509	12974593	199085	3	5	1	0.19510895	0.1218695	5	1	0.19510895	0.1218695
chr6	12974593	13030769	56177	2	6	1	0.20064824	0.1218695	6	1	0.20064824	0.1218695
chr6	13030769	13030828	60	1	6	1	0.129913	0.1218695	7	1	0.30102999	0.1218695
chr6	13030828	13137655	106828	2	6	1	0.20064824	0.1218695	6	1	0.20064824	0.1218695
chr6	13137655	13248873	111219	2	5	1	0.12309572	0.30102999	6	0	0.30102999	0
chr6	13248873	13268031	19159	2	6	1	0.129913	0.30102999	7	0	0.30102999	0
chr6	13268031	13311751	43721	1	5	1	0.30102999	0.30102999	4	0	0.11390336	0
chr6	13311751	13324065	12315	2	5	2	0.30102999	0.61140001	4	0	0.11390336	0
chr6	13324065	13469664	145600	3	5	1	0.30102999	0.30102999	4	0	0.11390336	0
chr6	13469664	13529695	60032	2	5	2	0.30102999	0.61140001	4	0	0.11390336	0
chr6	13529695	13806728	277034	8	5	1	0.30102999	0.30102999	4	0	0.11390336	0
chr6	13806728	13806787	60	1	5	2	0.30102999	0.61140001	4	0	0.11390336	0
chr6	13806787	13891671	84885	1	5	1	0.30102999	0.30102999	4	0	0.11390336	0
chr6	13891671	13891730	60	1	5	1	0.19510895	0.30102999	5	0	0.19510895	0
chr6	13891730	13978235	86506	2	5	1	0.30102999	0.30102999	4	0	0.11390336	0
chr6	13978235	14044891	66657	2	5	1	0.19510895	0.30102999	5	0	0.19510895	0
chr6	14044891	14122940	78050	1	5	1	0.30102999	0.30102999	4	0	0.11390336	0
chr6	14122940	14122999	60	1	5	1	0.07511598	0.30102999	7	0	0.43181735	0
chr6	14122999	14183664	60666	1	5	0	0.12309572	0	6	0	0.30102999	0
chr6	14183664	14311206	127543	1	5	0	0.19510895	0	5	0	0.19510895	0
chr6	14311206	14417234	106029	1	4	0	0.11390336	0	5	0	0.30102999	0
chr6	14417234	14545576	128343	1	4	0	0.18734596	0	4	0	0.18734596	0
chr6	14545576	14545635	60	1	4	0	0.11390336	0	5	0	0.30102999	0
chr6	14545635	14647002	101368	1	4	0	0.18734596	0	4	0	0.18734596	0
chr6	14647002	14710122	63121	2	5	0	0.30102999	0	4	0	0.11390336	0
chr6	14710122	14885265	175144	1	4	0	0.18734596	0	4	0	0.18734596	0
chr6	14885265	15081806	196542	2	3	0	0.10122019	0	4	0	0.30102999	0
chr6	15081806	15253135	171330	2	3	0	0.17593012	0	3	0	0.17593012	0
chr6	15253135	15312132	58998	2	4	0	0.30102999	0	3	0	0.10122019	0
chr6	15312132	15374392	62261	2	6	0	0.63695542	0	3	0	0.03070643	0
chr6	15374392	15374446	55	1	6	0	0.30102999	0	5	0	0.12309572	0
chr6	15374446	15580399	205954	3	5	0	0.19510895	0	5	0	0.19510895	0
chr6	15580399	15615549	35151	2	6	1	0.30102999	0.30102999	5	0	0.12309572	0
chr6	15615549	15794379	178831	2	5	1	0.30102999	0.30102999	4	0	0.11390336	0
chr6	15794379	15915522	121144	2	6	1	0.44141547	0.30102999	4	0	0.06713722	0
chr6	15915522	15915581	60	1	6	1	0.30102999	0.30102999	5	0	0.12309572	0
chr6	15915581	16063023	147443	1	6	1	0.44141547	0.30102999	4	0	0.06713722	0
chr6	16063023	16249176	186154	7	5	1	0.30102999	0.30102999	4	0	0.11390336	0
chr6	16249176	16249235	60	1	6	1	0.44141547	0.30102999	4	0	0.06713722	0
chr6	16249235	16295732	46498	2	5	1	0.30102999	0.30102999	4	0	0.11390336	0
chr6	16295732	16318467	22736	2	6	1	0.44141547	0.30102999	4	0	0.06713722	0
chr6	16318467	16441442	122976	3	7	1	0.60763643	0.30102999	4	0	0.03812622	0
chr6	16441442	16441501	60	1	7	1	0.43181735	0.30102999	5	0	0.07511598	0
chr6	16441501	16566479	124979	3	7	1	0.60763643	0.30102999	4	0	0.03812622	0
chr6	16566479	16566538	60	1	7	1	0.43181735	0.30102999	5	0	0.07511598	0
chr6	16566538	16623615	57078	1	6	1	0.30102999	0.30102999	5	0	0.12309572	0
chr6	16623615	16658098	34484	2	6	1	0.20064824	0.30102999	6	0	0.20064824	0
chr6	16658098	16846787	188690	4	7	1	0.30102999	0.30102999	6	0	0.129913	0
chr6	16846787	17013088	166302	2	7	1	0.20469099	0.30102999	7	0	0.20469099	0
chr6	17013088	17013147	60	1	7	1	0.08584816	0.30102999	9	0	0.42015402	0
chr6	17013147	17227851	214705	2	7	1	0.13499366	0.30102999	8	0	0.30102999	0
chr6	17227851	17284900	57050	1	7	0	0.13499366	0	8	0	0.30102999	0
chr6	17284900	17507441	222542	7	7	0	0.08584816	0	9	0	0.42015402	0
chr6	17507441	17609453	102013	2	7	0	0.13499366	0	8	0	0.30102999	0
chr6	17609453	17637670	28218	2	7	1	0.13499366	0.30102999	8	0	0.30102999	0
chr6	17637670	17972394	334725	9	7	0	0.13499366	0	8	0	0.30102999	0
chr6	17972394	18093033	120640	2	7	0	0.20469099	0	7	0	0.20469099	0
chr6	18093033	18160005	66973	3	7	0	0.13499366	0	8	0	0.30102999	0
chr6	18160005	18160064	60	1	7	1	0.13499366	0.30102999	8	0	0.30102999	0
chr6	18160064	18389392	229329	5	7	0	0.13499366	0	8	0	0.30102999	0
chr6	18389392	18596430	207039	4	6	0	0.129913	0	7	0	0.30102999	0
chr6	18596430	18648575	52146	2	6	0	0.08122616	0	8	0	0.4250187	0
chr6	18648575	19190219	541645	4	6	0	0.129913	0	7	0	0.30102999	0
chr6	19190219	19355845	165627	1	5	0	0.12309572	0	6	0	0.30102999	0
chr6	19355845	19501625	145781	2	5	0	0.07511598	0	7	0	0.43181735	0
chr6	19501625	19595112	93488	1	5	0	0.12309572	0	6	0	0.30102999	0
chr6	19595112	19840220	245109	4	5	0	0.07511598	0	7	0	0.43181735	0
chr6	19840220	19936116	95897	3	5	0	0.04407651	0	8	0	0.58747015	0
chr6	19936116	20031401	95286	2	5	0	0.02473314	0	9	0	0.76806864	0
chr6	20031401	20126873	95473	3	6	0	0.04875589	0	9	0	0.5732208	0
chr6	20126873	20152890	26018	2	6	1	0.04875589	0.30102999	9	0	0.5732208	0
chr6	20152890	20314434	161545	3	6	1	0.08122616	0.30102999	8	0	0.4250187	0
chr6	20314434	20649563	335130	6	5	1	0.04407651	0.30102999	8	0	0.58747015	0
chr6	20649563	20801967	152405	3	5	0	0.04407651	0	8	0	0.58747015	0
chr6	20801967	20802026	60	1	5	1	0.04407651	0.30102999	8	0	0.58747015	0
chr6	20802026	20903400	101375	2	5	0	0.07511598	0	7	0	0.43181735	0
chr6	20903400	20951453	48054	1	5	0	0.12309572	0	6	0	0.30102999	0
chr6	20951453	21043768	92316	3	5	0	0.07511598	0	7	0	0.43181735	0
chr6	21043768	21082139	38372	2	5	0	0.04407651	0	8	0	0.58747015	0
chr6	21082139	21082836	698	2	5	0	0.02473314	0	9	0	0.76806864	0
chr6	21082836	21124309	41474	1	5	0	0.04407651	0	8	0	0.58747015	0
chr6	21124309	21198286	73978	2	5	0	0.12309572	0	6	0	0.30102999	0
chr6	21198286	21198345	60	1	5	0	0.07511598	0	7	0	0.43181735	0
chr6	21198345	21390294	191950	2	5	0	0.12309572	0	6	0	0.30102999	0
chr6	21390294	21533907	143614	2	5	0	0.07511598	0	7	0	0.43181735	0
chr6	21533907	22002973	469067	8	5	0	0.12309572	0	6	0	0.30102999	0
chr6	22002973	22054876	51904	1	5	0	0.30102999	0	4	0	0.11390336	0
chr6	22054876	22139102	84227	1	4	0	0.18734596	0	4	0	0.18734596	0
chr6	22139102	22571462	432361	5	3	0	0.17593012	0	3	0	0.17593012	0
chr6	22571462	22571899	438	2	4	0	0.18734596	0	4	0	0.18734596	0
chr6	22571899	22731995	160097	2	3	0	0.10122019	0	4	0	0.30102999	0
chr6	22731995	22873626	141632	2	4	0	0.18734596	0	4	0	0.18734596	0
chr6	22873626	22942655	69030	1	3	0	0.10122019	0	4	0	0.30102999	0
chr6	22942655	23087351	144697	1	3	0	0.17593012	0	3	0	0.17593012	0
chr6	23087351	23087410	60	1	3	0	0.10122019	0	4	0	0.30102999	0
chr6	23087410	23436971	349562	3	3	0	0.17593012	0	3	0	0.17593012	0
chr6	23436971	23953528	516558	6	5	1	0.30102999	0.30102999	4	0	0.11390336	0
chr6	23953528	24128396	174869	2	4	1	0.18734596	0.30102999	4	0	0.18734596	0
chr6	24128396	25043507	915112	29	5	1	0.30102999	0.30102999	4	0	0.11390336	0
chr6	25043507	25194059	150553	4	6	1	0.44141547	0.30102999	4	0	0.06713722	0
chr6	25194059	25251093	57035	2	6	1	0.30102999	0.30102999	5	0	0.12309572	0
chr6	25251093	25320250	69158	2	6	1	0.129913	0.30102999	7	0	0.30102999	0
chr6	25320250	25320309	60	1	7	1	0.08584816	0.30102999	9	0	0.42015402	0
chr6	25320309	25368796	48488	1	6	1	0.04875589	0.30102999	9	0	0.5732208	0
chr6	25368796	25416765	47970	2	6	1	0.02793176	0.30102999	10	0	0.74627054	0
chr6	25416765	25416824	60	1	7	1	0.05232577	0.30102999	10	0	0.56314362	0
chr6	25416824	25441075	24252	1	7	1	0.08584816	0.30102999	9	0	0.42015402	0
chr6	25441075	25493003	51929	1	6	1	0.04875589	0.30102999	9	0	0.5732208	0
chr6	25493003	25530829	37827	2	6	1	0.02793176	0.30102999	10	0	0.74627054	0
chr6	25530829	25594700	63872	2	6	1	0.08122616	0.30102999	8	0	0.4250187	0

chr6	25594700	25594757	58	1	6	1	0.02793176	0.30102999	10	0	0.74627054	0
chr6	25594757	25726301	131545	4	6	1	0.08122616	0.30102999	8	0	0.4250187	0
chr6	25726301	25773847	47547	3	6	1	0.04875589	0.30102999	9	0	0.5732208	0
chr6	25773847	25826797	52951	2	5	1	0.02473314	0.30102999	9	0	0.76806864	0
chr6	25826797	25826856	60	1	6	1	0.04875589	0.30102999	9	0	0.5732208	0
chr6	25826856	26033385	206530	6	5	1	0.02473314	0.30102999	9	0	0.76806864	0
chr6	26033385	26104480	71096	2	5	1	0.04407651	0.30102999	8	0	0.58747015	0
chr6	26104480	26107921	3442	2	6	1	0.04875589	0.30102999	9	0	0.5732208	0
chr6	26107921	26124355	16435	2	6	1	0.02793176	0.30102999	10	0	0.74627054	0
chr6	26124355	26125025	671	3	7	1	0.03037338	0.30102999	11	0	0.73110763	0
chr6	26125025	26285413	160389	16	7	1	0.05232577	0.30102999	10	0	0.56314362	0
chr6	26285413	26390395	104983	2	6	1	0.02793176	0.30102999	10	0	0.74627054	0
chr6	26390395	26428978	38584	4	7	1	0.05232577	0.30102999	10	0	0.56314362	0
chr6	26428978	26503057	74080	4	6	1	0.02793176	0.30102999	10	0	0.74627054	0
chr6	26503057	26544373	41317	2	7	1	0.05232577	0.30102999	10	0	0.56314362	0
chr6	26544373	26598356	53984	1	6	1	0.04875589	0.30102999	9	0	0.5732208	0
chr6	26598356	26642819	44464	1	6	1	0.129913	0.30102999	7	0	0.30102999	0
chr6	26642819	26689276	46458	1	5	1	0.07511598	0.30102999	7	0	0.43181735	0
chr6	26689276	26999929	310654	2	5	1	0.04407651	0.30102999	8	0	0.58747015	0
chr6	26999929	26999988	60	1	7	2	0.05232577	0.61140001	10	0	0.56314362	0
chr6	26999988	27092982	92995	2	7	2	0.08584816	0.61140001	9	0	0.42015402	0
chr6	27092982	27115339	22358	4	6	2	0.04875589	0.61140001	9	0	0.5732208	0
chr6	27115339	27155070	39732	1	6	2	0.08122616	0.61140001	8	0	0.4250187	0
chr6	27155070	27218227	63158	1	5	1	0.07511598	0.30102999	7	0	0.43181735	0
chr6	27218227	27472009	253783	5	5	1	0.30102999	0.30102999	4	0	0.11390336	0
chr6	27472009	27551763	79755	3	5	1	0.19510895	0.30102999	5	0	0.19510895	0
chr6	27551763	28110946	559184	28	6	1	0.20064824	0.30102999	6	0	0.20064824	0
chr6	28110946	28145142	34197	1	5	1	0.12309572	0.30102999	6	0	0.30102999	0
chr6	28145142	28471522	326381	9	4	1	0.06713722	0.30102999	6	0	0.44141547	0
chr6	28471522	28471581	60	1	6	1	0.20064824	0.30102999	6	0	0.20064824	0
chr6	28471581	28501276	29696	1	5	1	0.12309572	0.30102999	6	0	0.30102999	0
chr6	28501276	28537621	36346	1	4	1	0.06713722	0.30102999	6	0	0.44141547	0
chr6	28537621	28648254	110634	3	5	1	0.12309572	0.30102999	6	0	0.30102999	0
chr6	28648254	28682928	34675	2	5	1	0.07511598	0.30102999	7	0	0.43181735	0
chr6	28682928	28746429	63502	1	5	1	0.12309572	0.30102999	6	0	0.30102999	0
chr6	28746429	28802854	56426	1	5	1	0.19510895	0.30102999	5	0	0.19510895	0
chr6	28802854	28802913	60	1	5	1	0.12309572	0.30102999	6	0	0.30102999	0
chr6	28802913	28949288	146376	3	5	1	0.19510895	0.30102999	5	0	0.19510895	0
chr6	28949288	28949347	60	1	5	1	0.12309572	0.30102999	6	0	0.30102999	0
chr6	28949347	29045777	96431	2	5	1	0.19510895	0.30102999	5	0	0.19510895	0
chr6	29045777	29045836	60	1	5	1	0.07511598	0.30102999	7	0	0.43181735	0
chr6	29045836	29140581	94746	2	4	1	0.11390336	0.30102999	5	0	0.30102999	0
chr6	29140581	29140640	60	1	4	1	0.02074938	0.30102999	8	0	0.79906872	0
chr6	29140640	29184205	43566	1	4	1	0.03812622	0.30102999	7	0	0.60763643	0
chr6	29184205	29184264	60	1	5	1	0.04407651	0.30102999	8	0	0.58747015	0
chr6	29184264	29259856	75593	1	4	1	0.02074938	0.30102999	8	0	0.79906872	0
chr6	29259856	29259915	60	1	4	1	0.01077081	0.30102999	9	0	1.01542894	0
chr6	29259915	29426204	166290	5	4	1	0.06713722	0.30102999	6	0	0.44141547	0
chr6	29426204	29527431	101228	4	4	1	0.03812622	0.30102999	7	0	0.60763643	0
chr6	29527431	29553660	26230	3	5	1	0.07511598	0.30102999	7	0	0.43181735	0
chr6	29553660	29638898	85239	2	5	1	0.12309572	0.30102999	6	0	0.30102999	0
chr6	29638898	29638957	60	1	5	1	0.07511598	0.30102999	7	0	0.43181735	0
chr6	29638957	29694190	55234	1	4	1	0.03812622	0.30102999	7	0	0.60763643	0
chr6	29694190	29719673	25484	2	4	1	0.02074938	0.30102999	8	0	0.79906872	0
chr6	29719673	29945375	225703	5	5	1	0.04407651	0.30102999	8	0	0.58747015	0
chr6	29945375	30308038	362664	16	5	1	0.02473314	0.30102999	9	0	0.76806864	0
chr6	30308038	30314498	6461	2	5	1	0.01320236	0.30102999	10	0	0.97390707	0
chr6	30314498	30406771	92274	2	5	1	0.02473314	0.30102999	9	0	0.76806864	0
chr6	30406771	30529936	123166	5	5	1	0.04407651	0.30102999	8	0	0.58747015	0
chr6	30529936	30554673	24738	2	5	1	0.02473314	0.30102999	9	0	0.76806864	0
chr6	30554673	30554732	60	1	6	1	0.04875589	0.30102999	9	0	0.5732208	0
chr6	30554732	30593903	39172	2	5	1	0.02473314	0.30102999	9	0	0.76806864	0
chr6	30593903	30595286	1384	2	6	1	0.04875589	0.30102999	9	0	0.5732208	0
chr6	30595286	30629636	34351	1	5	1	0.02473314	0.30102999	9	0	0.76806864	0
chr6	30629636	30629695	60	1	5	1	0.00666883	0.30102999	11	0	1.20557689	0
chr6	30629695	30657076	27382	2	5	1	0.02473314	0.30102999	9	0	0.76806864	0
chr6	30657076	30688370	31295	3	6	1	0.02793176	0.30102999	10	0	0.74627054	0
chr6	30688370	30688425	56	1	9	1	0.14135546	0.30102999	10	0	0.30102999	0
chr6	30688425	30698013	9589	1	7	1	0.05232577	0.30102999	10	0	0.56314362	0
chr6	30698013	30825584	127572	3	7	1	0.05232577	0.1218695	10	1	0.56314362	0.1218695
chr6	30825584	30860350	34767	3	8	1	0.08923391	0.1218695	10	1	0.4167287	0.1218695
chr6	30860350	30921958	61609	7	8	1	0.05490675	0.1218695	11	1	0.55623409	0.1218695
chr6	30921958	31078993	157036	2	8	1	0.08923391	0.1218695	10	1	0.4167287	0.1218695
chr6	31078993	31106231	27239	3	9	2	0.09154957	0.30102999	11	1	0.41444892	0.05404976
chr6	31106231	31120603	14373	3	10	2	0.14303407	0.30102999	11	1	0.30102999	0.05404976
chr6	31120603	31277858	157256	6	9	2	0.09154957	0.30102999	11	1	0.41444892	0.05404976
chr6	31277858	31348684	70827	1	8	2	0.05490675	0.30102999	11	1	0.55623409	0.05404976
chr6	31348684	31513065	164382	5	9	2	0.09154957	0.30102999	11	1	0.41444892	0.05404976
chr6	31513065	31525215	12151	1	9	2	0.14135546	0.30102999	10	1	0.30102999	0.05404976
chr6	31525215	31525274	60	1	10	2	0.21118145	0.30102999	10	1	0.21118145	0.05404976
chr6	31525274	31541471	16198	1	9	2	0.14135546	0.30102999	10	1	0.30102999	0.05404976
chr6	31541471	31548413	6943	5	10	2	0.14303407	0.30102999	11	1	0.30102999	0.05404976
chr6	31548413	31583427	35015	1	10	2	0.21118145	0.30102999	10	1	0.21118145	0.05404976
chr6	31583427	31626810	43384	4	11	2	0.30102999	0.30102999	10	1	0.14303407	0.05404976
chr6	31626810	31659133	32324	7	11	1	0.30102999	0.1218695	10	1	0.14303407	0.1218695
chr6	31659133	31659192	60	1	11	2	0.30102999	0.30102999	10	1	0.14303407	0.05404976
chr6	31659192	31688818	29627	5	10	2	0.21118145	0.61140001	10	0	0.21118145	0
chr6	31688818	31695676	6859	2	11	2	0.30102999	0.61140001	10	0	0.14303407	0
chr6	31695676	31720807	25132	2	11	1	0.30102999	0.30102999	10	0	0.14303407	0
chr6	31720807	31797657	76851	9	12	1	0.30102999	0.30102999	11	0	0.14385241	0
chr6	31797657	31804641	6985	3	12	1	0.30102999	0.1218695	11	1	0.14385241	0.1218695
chr6	31804641	31857843	53203	5	12	1	0.30102999	0.30102999	11	0	0.14385241	0
chr6	31857843	31902174	44332	1	12	1	0.41314172	0.30102999	10	0	0.09290028	0
chr6	31902174	31905114	2941	2	12	1	0.30102999	0.30102999	11	0	0.14385241	0
chr6	31905114	31922818	17705	2	12	1	0.41314172	0.30102999	10	0	0.09290028	0
chr6	31922818	31939009	16192	4	12	1	0.30102999	0.30102999	11	0	0.14385241	0
chr6	31939009	31939821	813	1	11	1	0.30102999	0.30102999	10	0	0.14303407	0
chr6	31939821	32035188	95368	1	10	1	0.21118145	0.30102999	10	0	0.21118145	0
chr6	32035188	32091117	55930	2	10	1	0.30102999	0.30102999	9	0	0.14135546	0
chr6	32091117	32097503	6387	2	11	1	0.21200206	0.30102999	11	0	0.21200206	0
chr6	32097503	32117630	20128	1	11	1	0.30102999	0.30102999	10	0	0.14303407	0
chr6	32117630	32128956	11327	1	11	1	0.41444892	0.30102999	9	0	0.09154957	0
chr6	32128956	32149461	20506	3	10	1	0.30102999	0.30102999	9	0	0.14135546	0
chr6	32149461	32154733	5273	1	9	1	0.20975986	0.30102999	9	0	0.20975986	0
chr6	32154733	32160025	5293	2	10	1	0.30102999	0.30102999	9	0	0.14135546	0
chr6	32160025	32189171	29147	2	11	1	0.21200206	0.30102999	11	0	0.21200206	0
chr6	32189171	32245268	56098	2	11	1	0.14385241	0.30102999	12	0	0.30102999	0
chr6	32245268	32261671	16404	1	11	1	0.21200206	0.30102999	11	0	0.21200206	0
chr6	32261671	32430137	168467	4	12	1	0.30102999	0.30102999	11	0	0.14385241	0
chr6	32430137	32485232	55096	2	13	1	0.41271556	0.30102999	11	0	0.09334429	0
chr6	32485232	32523396	38165	1	12	1	0.30102999	0.30102999	11	0	0.14385241	0
chr6	32523396	32604038	80643	2	11	1	0.21200206	0.30102999	11	0	0.21200206	0
chr6	32604038	32658104	54067	1	11	1	0.41444892	0.30102999	9	0	0.09154957	0
chr6	32658104	32731014	72911	2	7	1	0.20469099	0.30102999	7	0	0.20469099	0
chr6	32731014	32731073	60	1	7	1	0.08584816	0.30102999	9	0	0.42015402	0
chr6	32731073	32781919	50847	1	7	1	0.13499366	0.30102999	8	0	0.30102999	0
chr6	32781919	32908075	126157	9	6	1	0.129913	0.30102999	7	0	0.30102999	0
chr6	32908075	32920040	11966	1	5	1	0.07511598	0.30102999	7	0	0.43181735	0
chr6	32920040	32939206	19167	2	5	1	0.04407651	0.30102999	8	0	0.58747015	0
chr6	32939206	33052205	113000	5	5	1	0.12309572	0.30102999	6	0	0.30102999	0
chr6	33052205	33162841	110637	5	5	1	0.19510895	0.30102999	5	0	0.19510895	0
chr6	33162841	33201680	38840	4	5	1	0.12309572	0.30102999	6	0	0.30102999	0
chr6	33201680	33201739	60	1	5	1	0.07511598	0.30102999	7	0	0.43181735	0
chr6	33201739	33377086	175348	13	5	1	0.12309572	0.30102999	6	0	0.30102999	0

chr6	33377086	33389795	12710	4	5	1	0.07511598	0.30102999	7	0	0.43181735	0
chr6	33389795	33496935	107141	2	5	1	0.12309572	0.30102999	6	0	0.30102999	0
chr6	33496935	33557887	60953	4	5	1	0.07511598	0.30102999	7	0	0.43181735	0
chr6	33557887	33588535	30649	1	5	1	0.12309572	0.30102999	6	0	0.30102999	0
chr6	33588535	33588591	57	1	5	1	0.07511598	0.30102999	7	0	0.43181735	0
chr6	33588591	33677306	88716	3	5	1	0.12309572	0.30102999	6	0	0.30102999	0
chr6	33677306	33693416	16111	2	6	1	0.20064824	0.30102999	6	0	0.20064824	0
chr6	33693416	33748896	55481	1	5	1	0.12309572	0.30102999	6	0	0.30102999	0
chr6	33748896	33770834	21939	2	5	1	0.07511598	0.30102999	7	0	0.43181735	0
chr6	33770834	33963393	192560	2	5	1	0.12309572	0.30102999	6	0	0.30102999	0
chr6	33963393	33977355	13963	2	5	1	0.07511598	0.30102999	7	0	0.43181735	0
chr6	33977355	34034924	57570	2	6	1	0.129913	0.30102999	7	0	0.30102999	0
chr6	34034924	34071292	36369	2	7	1	0.20469099	0.30102999	7	0	0.20469099	0
chr6	34071292	34071351	60	1	8	1	0.30102999	0.30102999	7	0	0.13499366	0
chr6	34071351	34086729	15379	1	6	1	0.129913	0.30102999	7	0	0.30102999	0
chr6	34086729	34163154	76426	1	5	1	0.07511598	0.30102999	7	0	0.43181735	0
chr6	34163154	34163213	60	1	5	1	0.04407651	0.30102999	8	0	0.58747015	0
chr6	34163213	34209939	46727	1	5	1	0.07511598	0.30102999	7	0	0.43181735	0
chr6	34209939	34263395	53457	1	5	1	0.12309572	0.30102999	6	0	0.30102999	0
chr6	34263395	34332310	68916	3	6	1	0.20064824	0.30102999	6	0	0.20064824	0
chr6	34332310	34471102	138793	4	7	1	0.30102999	0.30102999	6	0	0.129913	0
chr6	34471102	34520505	49404	2	7	1	0.30102999	0.1218695	6	1	0.129913	0.1218695
chr6	34520505	34520561	57	1	7	1	0.20469099	0.1218695	7	1	0.20469099	0.1218695
chr6	34520561	34555153	34593	1	7	1	0.30102999	0.1218695	6	1	0.129913	0.1218695
chr6	34555153	34578199	23047	1	7	0	0.30102999	0	6	1	0.129913	0.30102999
chr6	34578199	34578258	60	1	7	0	0.20469099	0	7	1	0.20469099	0.30102999
chr6	34578258	34614753	36496	1	7	0	0.20469099	0	7	0	0.20469099	0
chr6	34614753	34649402	34650	1	7	0	0.30102999	0	6	0	0.129913	0
chr6	34649402	34700092	50691	2	7	1	0.30102999	0.30102999	6	0	0.129913	0
chr6	34700092	34734425	34334	2	7	1	0.20469099	0.30102999	7	0	0.20469099	0
chr6	34734425	34824605	90181	2	7	1	0.30102999	0.30102999	6	0	0.129913	0
chr6	34824605	34850792	26188	1	7	0	0.30102999	0	6	0	0.129913	0
chr6	34850792	34965512	114721	4	7	0	0.20469099	0	7	0	0.20469099	0
chr6	34965512	35031548	66037	2	7	1	0.20469099	0.30102999	7	0	0.20469099	0
chr6	35031548	35219348	187801	8	7	0	0.20469099	0	7	0	0.20469099	0
chr6	35219348	35250592	31245	3	7	0	0.13499366	0	8	0	0.30102999	0
chr6	35250592	35426963	176372	6	7	0	0.20469099	0	7	0	0.20469099	0
chr6	35426963	35489509	62547	8	7	0	0.13499366	0	8	0	0.30102999	0
chr6	35489509	35790836	301328	8	7	1	0.13499366	0.30102999	8	0	0.30102999	0
chr6	35790836	35840824	49989	1	6	1	0.08122616	0.30102999	8	0	0.4250187	0
chr6	35840824	35840883	60	1	6	2	0.08122616	0.61140001	8	0	0.4250187	0
chr6	35840883	35869457	28575	1	6	1	0.08122616	0.30102999	8	0	0.4250187	0
chr6	35869457	35869516	60	1	7	1	0.13499366	0.30102999	8	0	0.30102999	0
chr6	35869516	35927384	57869	1	7	1	0.20469099	0.30102999	7	0	0.20469099	0
chr6	35927384	35997490	70107	2	6	1	0.129913	0.30102999	7	0	0.30102999	0
chr6	35997490	36040640	43151	3	7	1	0.13499366	0.30102999	8	0	0.30102999	0
chr6	36040640	36070415	29776	2	7	2	0.13499366	0.61140001	8	0	0.30102999	0
chr6	36070415	36103006	32592	1	6	2	0.08122616	0.61140001	8	0	0.4250187	0
chr6	36103006	36187934	84929	2	6	1	0.129913	0.30102999	7	0	0.30102999	0
chr6	36187934	36358592	170659	5	6	2	0.129913	0.61140001	7	0	0.30102999	0
chr6	36358592	36410520	51929	2	6	2	0.08122616	0.61140001	8	0	0.4250187	0
chr6	36410520	36437855	27336	2	7	2	0.08584816	0.61140001	9	0	0.42015402	0
chr6	36437855	36513924	76070	2	6	1	0.04875589	0.30102999	9	0	0.5732208	0
chr6	36513924	36778123	264200	11	6	1	0.129913	0.30102999	7	0	0.30102999	0
chr6	36778123	37029712	251590	11	6	1	0.08122616	0.30102999	8	0	0.4250187	0
chr6	37029712	37029771	60	1	6	1	0.04875589	0.30102999	9	0	0.5732208	0
chr6	37029771	37179962	150192	5	6	1	0.08122616	0.30102999	8	0	0.4250187	0
chr6	37179962	37349084	169123	5	6	1	0.04875589	0.30102999	9	0	0.5732208	0
chr6	37349084	37403430	54347	3	6	1	0.02793176	0.30102999	10	0	0.74627054	0
chr6	37403430	37509377	105948	3	6	1	0.129913	0.30102999	7	0	0.30102999	0
chr6	37509377	37509436	60	1	6	1	0.04875589	0.30102999	9	0	0.5732208	0
chr6	37509436	37605888	96453	1	6	1	0.129913	0.30102999	7	0	0.30102999	0
chr6	37605888	37785349	179462	2	6	1	0.20064824	0.30102999	6	0	0.20064824	0
chr6	37785349	37849793	64445	3	6	1	0.08122616	0.30102999	8	0	0.4250187	0
chr6	37849793	37849852	60	1	6	1	0.04875589	0.30102999	9	0	0.5732208	0
chr6	37849852	37897789	47938	1	6	1	0.08122616	0.30102999	8	0	0.4250187	0
chr6	37897789	37932443	34655	1	6	1	0.129913	0.30102999	7	0	0.30102999	0
chr6	37932443	37979660	47218	1	5	1	0.12309572	0.30102999	6	0	0.30102999	0
chr6	37979660	38068871	89212	2	4	1	0.06713722	0.30102999	6	0	0.44141547	0
chr6	38068871	38142161	73291	2	5	1	0.12309572	0.30102999	6	0	0.30102999	0
chr6	38142161	38180792	38632	2	5	1	0.07511598	0.30102999	7	0	0.43181735	0
chr6	38180792	38180851	60	1	6	1	0.129913	0.30102999	7	0	0.30102999	0
chr6	38180851	38252496	71646	2	6	1	0.20064824	0.30102999	6	0	0.20064824	0
chr6	38252496	38252555	60	1	6	1	0.129913	0.30102999	7	0	0.30102999	0
chr6	38252555	38418836	166282	3	3	1	0.05670724	0.30102999	5	0	0.45545077	0
chr6	38418836	38483074	64239	3	4	1	0.11390336	0.30102999	5	0	0.30102999	0
chr6	38483074	38545447	62374	2	5	1	0.19510895	0.30102999	5	0	0.19510895	0
chr6	38545447	38593281	47835	1	4	1	0.11390336	0.30102999	5	0	0.30102999	0
chr6	38593281	38754721	161441	4	4	1	0.06713722	0.30102999	6	0	0.44141547	0
chr6	38754721	38885196	130476	3	4	1	0.11390336	0.30102999	5	0	0.30102999	0
chr6	38885196	39003892	118697	3	3	1	0.05670724	0.30102999	5	0	0.45545077	0
chr6	39003892	39003951	60	1	3	1	0.03070643	0.30102999	6	0	0.63695542	0
chr6	39003951	39507808	503858	13	3	1	0.05670724	0.30102999	5	0	0.45545077	0
chr6	39507808	39581432	73625	1	3	1	0.10122019	0.30102999	4	0	0.30102999	0
chr6	39581432	39581491	60	1	3	1	0.10122019	0.1218695	4	1	0.30102999	0.1218695
chr6	39581491	39654656	73166	1	3	1	0.10122019	0.30102999	4	0	0.30102999	0
chr6	39654656	39654715	60	1	4	1	0.06713722	0.30102999	6	0	0.44141547	0
chr6	39654715	39734163	79449	1	4	1	0.11390336	0.30102999	5	0	0.30102999	0
chr6	39734163	39734222	60	1	5	1	0.19510895	0.30102999	5	0	0.19510895	0
chr6	39734222	39828689	94468	3	4	0	0.11390336	0	5	0	0.30102999	0
chr6	39828689	39862003	33315	2	5	1	0.19510895	0.30102999	5	0	0.19510895	0
chr6	39862003	39937893	75891	3	5	0	0.19510895	0	5	0	0.19510895	0
chr6	39937893	39937952	60	1	5	1	0.19510895	0.30102999	5	0	0.19510895	0
chr6	39937952	40001906	63955	1	4	0	0.11390336	0	5	0	0.30102999	0
chr6	40001906	40254912	253007	2	3	0	0.05670724	0	5	0	0.45545077	0
chr6	40254912	40321633	66722	2	3	1	0.05670724	0.30102999	5	0	0.45545077	0
chr6	40321633	40569149	247517	5	4	1	0.11390336	0.30102999	5	0	0.30102999	0
chr6	40569149	40841720	272572	2	3	1	0.05670724	0.30102999	5	0	0.45545077	0
chr6	40841720	40999441	157722	3	4	1	0.11390336	0.30102999	5	0	0.30102999	0
chr6	40999441	41367026	367586	14	4	1	0.11390336	0.1218695	5	1	0.30102999	0.1218695
chr6	41367026	41745384	378359	8	4	1	0.18734596	0.1218695	4	1	0.18734596	0.1218695
chr6	41745384	41757310	11927	3	4	1	0.11390336	0.05404976	5	2	0.30102999	0.30102999
chr6	41757310	41900722	143413	5	4	1	0.11390336	0.1218695	5	1	0.30102999	0.1218695
chr6	41900722	41975433	74712	6	4	1	0.11390336	0.05404976	5	2	0.30102999	0.30102999
chr6	41975433	42141736	166304	5	4	1	0.11390336	0.1218695	5	1	0.30102999	0.1218695
chr6	42141736	42196370	54635	2	4	0	0.11390336	0	5	1	0.30102999	0.30102999
chr6	42196370	42196429	60	1	4	2	0.06713722	0.30102999	6	1	0.44141547	0.05404976
chr6	42196429	42286013	89585	2	4	1	0.06713722	0.1218695	6	1	0.44141547	0.1218695
chr6	42286013	42362358	76346	2	4	1	0.11390336	0.1218695	5	1	0.30102999	0.1218695
chr6	42362358	42362417	60	1	4	2	0.11390336	0.30102999	5	1	0.30102999	0.05404976
chr6	42362417	42457654	95238	2	4	1	0.11390336	0.1218695	5	1	0.30102999	0.1218695
chr6	42457654	42713196	255543	7	4	1	0.06713722	0.1218695	6	1	0.44141547	0.1218695
chr6	42713196	42980710	267515	13	4	1	0.11390336	0.1218695	5	1	0.30102999	0.1218695
chr6	42980710	42993872	13163	3	5	1	0.19510895	0.1218695	5	1	0.19510895	0.1218695
chr6	42993872	43026343	32472	3	4	1	0.11390336	0.1218695	5	1	0.30102999	0.1218695
chr6	43026343	43031965	5623	2	5	1	0.12309572	0.1218695	6	1	0.30102999	0.1218695
chr6	43031965	43235217	203253	5	4	1	0.11390336	0.1218695	5	1	0.30102999	0.1218695
chr6	43235217	43235276	60	1	4	1	0.06713722	0.1218695	6	1	0.44141547	0.1218695
chr6	43235276	43399596	164321	3	4	1	0.11390336	0.1218695	5	1	0.30102999	0.1218695
chr6	43399596	43419498	19903	3	4	1	0.03812622	0.1218695	7	1	0.60763643	0.1218695
chr6	43419498	43473935	54438	3	6	1	0.129913	0.1218695	7	1	0.30102999	0.1218695
chr6	43473935	43558779	84845	7	6	1	0.08122616	0.1218695	8	1	0.4250187	0.1218695
chr6	43558779	43608442	49664	3	5	1	0.07511598	0.1218695	7	1	0.43181735	0.1218695
chr6	43608442	43635525	27084	3	6	1	0.129913	0.1218695	7	1	0.30102999	0.1218695

chr6	43635525	43644080	8556	2	6	1	0.08122616	0.1218695	8	1	0.4250187	0.1218695
chr6	43644080	43738066	93987	2	5	1	0.07511598	0.1218695	7	1	0.43181735	0.1218695
chr6	43738066	43798208	60143	4	6	1	0.129913	0.1218695	7	1	0.30102999	0.1218695
chr6	43798208	43969565	171358	1	6	0	0.129913	0	7	1	0.30102999	0.30102999
chr6	43969565	44002922	33358	4	7	0	0.13499366	0	8	1	0.30102999	0.30102999
chr6	44002922	44083725	80804	1	6	0	0.08122616	0	8	1	0.4250187	0.30102999
chr6	44083725	44146874	63150	1	5	0	0.04407651	0	8	1	0.58747015	0.30102999
chr6	44146874	44146933	60	1	5	0	0.02473314	0	9	1	0.76806864	0.30102999
chr6	44146933	44249729	102797	5	5	0	0.04407651	0	8	1	0.58747015	0.30102999
chr6	44249729	44358073	108345	5	5	0	0.02473314	0	9	1	0.76806864	0.30102999
chr6	44358073	44415140	57068	5	5	0	0.01320236	0	10	1	0.97390707	0.30102999
chr6	44415140	44596012	180873	1	5	0	0.04407651	0	8	1	0.58747015	0.30102999
chr6	44596012	44796618	200607	2	6	0	0.08122616	0	8	1	0.4250187	0.30102999
chr6	44796618	44902611	105994	4	7	0	0.08584816	0	9	1	0.42015402	0.30102999
chr6	44902611	44902670	60	1	7	0	0.05232577	0	10	1	0.56314362	0.30102999
chr6	44902670	44971472	68803	1	6	0	0.08122616	0	8	1	0.4250187	0.30102999
chr6	44971472	45020461	48990	1	6	0	0.129913	0	7	1	0.30102999	0.30102999
chr6	45020461	45073711	53251	1	5	0	0.07511598	0	7	1	0.43181735	0.30102999
chr6	45073711	45128523	54813	2	6	0	0.129913	0	7	1	0.30102999	0.30102999
chr6	45128523	45179039	50517	3	8	0	0.13872638	0	9	1	0.30102999	0.30102999
chr6	45179039	45341709	162671	3	7	0	0.13499366	0	8	1	0.30102999	0.30102999
chr6	45341709	45910266	568558	7	6	0	0.20064824	0	6	1	0.20064824	0.30102999
chr6	45910266	45980219	69954	2	5	0	0.12309572	0	6	1	0.30102999	0.30102999
chr6	45980219	45980278	60	1	6	0	0.20064824	0	6	1	0.20064824	0.30102999
chr6	45980278	46054890	74613	2	5	0	0.12309572	0	6	1	0.30102999	0.30102999
chr6	46054890	46112152	57263	2	5	0	0.12309572	0	6	0	0.30102999	0
chr6	46112152	47004310	892159	24	4	0	0.06713722	0	6	0	0.44141547	0
chr6	47004310	47170572	166263	2	5	0	0.12309572	0	6	0	0.30102999	0
chr6	47170572	47501506	330935	7	5	0	0.07511598	0	7	0	0.43181735	0
chr6	47501506	47592779	91274	2	4	0	0.11390336	0	5	0	0.30102999	0
chr6	47592779	47592838	60	1	4	0	0.06713722	0	6	0	0.44141547	0
chr6	47592838	47665567	72730	2	4	0	0.30102999	0	3	0	0.10122019	0
chr6	47665567	47876997	211431	4	3	0	0.17593012	0	3	0	0.17593012	0
chr6	47876997	47936534	59538	1	3	0	0.30102999	0	2	0	0.08289318	0
chr6	47936534	48168800	232267	3	2	0	0.1575501	0	2	0	0.1575501	0
chr6	48168800	48168859	60	1	3	0	0.30102999	0	2	0	0.08289318	0
chr6	48168859	48217975	49117	1	1	0	0.1218695	0	1	0	0.1218695	0
chr6	48217975	48381374	163400	1	0	0	0	0	1	0	0.30102999	0
chr6	48381374	48540177	158804	2	2	0	0.30102999	0	1	0	0.05404976	0
chr6	48540177	48540236	60	1	2	0	0.08289318	0	3	0	0.30102999	0
chr6	48540236	48682849	142614	1	1	0	0.02438896	0	3	0	0.51676182	0
chr6	48682849	48853381	170533	2	2	0	0.08289318	0	3	0	0.30102999	0
chr6	48853381	49520226	666846	8	1	0	0.02438896	0	3	0	0.51676182	0
chr6	49520226	49524620	4395	2	2	0	0.08289318	0	3	0	0.30102999	0
chr6	49524620	49705011	180392	5	1	0	0.02438896	0	3	0	0.51676182	0
chr6	49705011	49754264	49254	2	1	0	0.01091641	0	4	0	0.76005302	0
chr6	49754264	49754310	47	1	2	0	0.0429175	0	4	0	0.47744371	0
chr6	49754310	50153612	399303	6	1	0	0.01091641	0	4	0	0.76005302	0
chr6	50153612	50519405	365794	3	3	0	0.03070643	0	6	0	0.63695542	0
chr6	50519405	50519464	60	1	3	0	0.01598258	0	7	0	0.84395715	0
chr6	50519464	50677418	157955	1	3	0	0.03070643	0	6	0	0.63695542	0
chr6	50677418	50787061	109644	3	4	0	0.06713722	0	6	0	0.44141547	0
chr6	50787061	50855651	68591	2	6	0	0.20064824	0	6	0	0.20064824	0
chr6	50855651	50938895	83245	1	5	0	0.12309572	0	6	0	0.30102999	0
chr6	50938895	50938954	60	1	5	0	0.07511598	0	7	0	0.43181735	0
chr6	50938954	51188587	249634	2	3	0	0.03070643	0	6	0	0.63695542	0
chr6	51188587	51285782	97196	3	4	0	0.03812622	0	7	0	0.60763643	0
chr6	51285782	51356400	70619	1	4	0	0.06713722	0	6	0	0.44141547	0
chr6	51356400	51565572	209173	5	4	0	0.03812622	0	7	0	0.60763643	0
chr6	51565572	51565631	60	1	4	0	0.03812622	0	7	1	0.60763643	0.30102999
chr6	51565631	51676030	110400	2	4	0	0.03812622	0	7	0	0.60763643	0
chr6	51676030	51732872	56843	2	4	0	0.02074938	0	8	0	0.79906872	0
chr6	51732872	51732931	60	1	4	0	0.02074938	0	8	1	0.79906872	0.30102999
chr6	51732931	51781690	48760	1	4	0	0.02074938	0	8	0	0.79906872	0
chr6	51781690	51781749	60	1	4	0	0.01077081	0	9	0	1.01542894	0
chr6	51781749	51824641	42893	1	4	0	0.03812622	0	7	0	0.60763643	0
chr6	51824641	51856209	31569	2	4	0	0.02074938	0	8	0	0.79906872	0
chr6	51856209	51918238	62030	2	4	0	0.01077081	0	9	0	1.01542894	0
chr6	51918238	51918297	60	1	5	0	0.02473314	0	9	0	0.76806864	0
chr6	51918297	52018676	100380	3	4	0	0.02074938	0	8	0	0.79906872	0
chr6	52018676	52018735	60	1	5	0	0.02473314	0	9	0	0.76806864	0
chr6	52018735	52054898	36164	2	5	0	0.07511598	0	7	0	0.43181735	0
chr6	52054898	52103749	48852	2	5	0	0.04407651	0	8	0	0.58747015	0
chr6	52103749	52174776	71028	3	5	0	0.04407651	0	8	1	0.58747015	0.30102999
chr6	52174776	52174835	60	1	5	0	0.02473314	0	9	1	0.76806864	0.30102999
chr6	52174835	52248137	73303	1	4	0	0.03812622	0	7	1	0.60763643	0.30102999
chr6	52248137	52318918	70782	1	3	0	0.01598258	0	7	1	0.84395715	0.30102999
chr6	52318918	52436596	117679	5	4	0	0.01077081	0	9	1	1.01542894	0.30102999
chr6	52436596	52536762	100167	1	4	0	0.02074938	0	8	1	0.79906872	0.30102999
chr6	52536762	52577491	40730	2	5	0	0.04407651	0	8	1	0.58747015	0.30102999
chr6	52577491	52605937	28447	2	6	0	0.08122616	0	8	1	0.4250187	0.30102999
chr6	52605937	52883117	277181	7	5	0	0.04407651	0	8	1	0.58747015	0.30102999
chr6	52883117	52883176	60	1	5	0	0.02473314	0	9	1	0.76806864	0.30102999
chr6	52883176	53043731	160556	4	3	0	0.00378107	0	9	1	1.33111237	0.30102999
chr6	53043731	53043790	60	1	5	0	0.02473314	0	9	1	0.76806864	0.30102999
chr6	53043790	53133054	89265	2	3	0	0.00378107	0	9	1	1.33111237	0.30102999
chr6	53133054	53166598	33545	2	4	0	0.01077081	0	9	1	1.01542894	0.30102999
chr6	53166598	53207323	40726	1	4	0	0.02074938	0	8	1	0.79906872	0.30102999
chr6	53207323	53277044	69722	1	3	0	0.01598258	0	7	1	0.84395715	0.30102999
chr6	53277044	53363094	86051	3	4	0	0.02074938	0	8	1	0.79906872	0.30102999
chr6	53363094	53394023	30930	1	3	0	0.01598258	0	7	1	0.84395715	0.30102999
chr6	53394023	53555361	161339	2	2	0	0.00493743	0	7	1	1.16581773	0.30102999
chr6	53555361	53662780	107420	2	3	0	0.01598258	0	7	1	0.84395715	0.30102999
chr6	53662780	53687073	24294	1	3	0	0.03070643	0	6	1	0.63695542	0.30102999
chr6	53687073	53687132	60	1	4	0	0.03812622	0	7	1	0.60763643	0.30102999
chr6	53687132	53713420	26289	1	2	0	0.01053319	0	6	1	0.91219088	0.30102999
chr6	53713420	53713479	60	1	2	0	0.00493743	0	7	1	1.16581773	0.30102999
chr6	53713479	53767421	53943	1	2	0	0.01053319	0	6	1	0.91219088	0.30102999
chr6	53767421	53767480	60	1	2	0	0.00493743	0	7	1	1.16581773	0.30102999
chr6	53767480	53853420	85941	1	1	0	8.47E-04	0	7	1	1.62048027	0.30102999
chr6	53853420	53853479	60	1	2	0	0.00493743	0	7	1	1.16581773	0.30102999
chr6	53853479	53947911	94433	2	2	0	0.01053319	0	6	1	0.91219088	0.30102999
chr6	53947911	53989414	41504	2	3	0	0.01598258	0	7	1	0.84395715	0.30102999
chr6	53989414	54054700	65287	1	2	0	0.00493743	0	7	1	1.16581773	0.30102999
chr6	54054700	54131101	76402	1	2	0	0.01053319	0	6	1	0.91219088	0.30102999
chr6	54131101	54191632	60532	1	2	0	0.02162467	0	5	0	0.68214471	0
chr6	54191632	54191685	54	1	2	0	9.54E-04	0	9	0	1.74076927	0
chr6	54191685	54254838	63154	1	2	0	0.00221948	0	8	0	1.44210395	0
chr6	54254838	54380391	125554	1	2	0	0.00493743	0	7	0	1.16581773	0
chr6	54380391	54380450	60	1	2	0	0.00221948	0	8	0	1.44210395	0
chr6	54380450	54628342	247893	2	1	0	3.39E-04	0	8	0	1.94674965	0
chr6	54628342	54739321	110980	3	3	0	0.0079614	0	8	0	1.07548421	0
chr6	54739321	54739380	60	1	4	0	0.01077081	0	9	0	1.01542894	0
chr6	54739380	54813743	74364	1	4	0	0.02074938	0	8	0	0.79906872	0
chr6	54813743	54918661	104919	1	2	0	0.01053319	0	6	0	0.91219088	0
chr6	54918661	55044728	126068	2	2	1	0.01053319	0.30102999	6	0	0.91219088	0
chr6	55044728	55044787	60	1	3	1	0.0079614	0.30102999	8	0	1.07548421	0
chr6	55044787	55147137	102351	2	3	1	0.01598258	0.30102999	7	0	0.84395715	0
chr6	55147137	55211976	64840	2	3	1	0.0079614	0.30102999	8	0	1.07548421	0
chr6	55211976	55212035	60	1	5	1	0.04407651	0.30102999	8	0	0.58747015	0
chr6	55212035	55329426	117392	2	3	0	0.0079614	0	8	0	1.07548421	0
chr6	55329426	55458153	128728	3	5	0	0.02473314	0	9	0	0.76806864	0
chr6	55458153	55458212	60	1	5	0	0.01320236	0	10	0	0.97390707	0
chr6	55458212	55625277	167066	2	5	0	0.02473314	0	9	0	0.76806864	0

chr6	55625277	55659244	33968	2	5	1	0.01320236	0.30102999	10	0	0.97390707	0
chr6	55659244	55711436	52193	2	5	1	0.02473314	0.30102999	9	0	0.76806864	0
chr6	55711436	55864341	152906	2	4	0	0.02074938	0	8	0	0.79906872	0
chr6	55864341	55864400	60	1	5	0	0.01320236	0	10	0	0.97390707	0
chr6	55864400	55955844	91445	2	5	0	0.07511598	0	7	0	0.43181735	0
chr6	55955844	55955903	60	1	5	0	0.02473314	0	9	0	0.76806864	0
chr6	55955903	55990444	34542	1	5	0	0.04407651	0	8	0	0.58747015	0
chr6	55990444	56091285	100842	2	4	0	0.02074938	0	8	0	0.79906872	0
chr6	56091285	56091344	60	1	6	0	0.01518174	0	11	1	0.9449689	0.30102999
chr6	56091344	56212219	120876	2	3	0	0.00170589	0	10	1	1.61091002	0.30102999
chr6	56212219	56252017	39799	2	4	0	0.00530919	0	10	1	1.2568129	0.30102999
chr6	56252017	56323554	71538	2	5	1	0.01320236	0.1218695	10	1	0.97390707	0.1218695
chr6	56323554	56323613	60	1	5	1	0.00666883	0.1218695	11	1	1.20557689	0.1218695
chr6	56323613	56366197	42585	1	3	1	0.00170589	0.1218695	10	1	1.61091002	0.1218695
chr6	56366197	56433379	67183	1	2	1	3.90E-04	0.1218695	10	1	2.0620585	0.1218695
chr6	56433379	56508516	75138	2	1	1	4.80E-05	0.1218695	10	1	2.6588298	0.1218695
chr6	56508516	56616119	107604	2	1	1	8.47E-04	0.1218695	7	1	1.62048027	0.1218695
chr6	56616119	56682232	66114	1	1	0	0.00204627	0	6	1	1.31360226	0.30102999
chr6	56682232	57075262	393031	10	1	0	0.00478973	0	5	1	1.02643191	0.30102999
chr6	57075262	57149417	74156	1	0	0	0	0	5	1	1.60515106	0.30102999
chr6	57149417	57246989	97573	3	0	0	0	0	4	1	1.26272838	0.30102999
chr6	57246989	57297527	50539	1	0	0	0	0	4	0	1.26272838	0
chr6	57297527	57329882	32356	2	2	0	0.0429175	0	4	0	0.47744371	0
chr6	57329882	57329941	60	1	3	0	0.10122019	0	4	0	0.30102999	0
chr6	57329941	57393140	63200	1	1	0	0.02438896	0	3	0	0.51676182	0
chr6	57393140	57686216	293077	5	1	0	0.02438896	0	3	1	0.51676182	0.30102999
chr6	57686216	58014532	328317	1	1	0	0.1218695	0	1	0	0.1218695	0
chr6	61982931	62367058	384128	2	0	1	0	0.30102999	4	0	1.26272838	0
chr6	62367058	62367117	60	1	0	1	0	0.30102999	5	0	1.60515106	0
chr6	62367117	62757818	390702	8	0	1	0	0.30102999	4	0	1.26272838	0
chr6	62757818	62887221	129404	4	0	1	0	0.1218695	5	1	1.60515106	0.1218695
chr6	62887221	62944608	57388	1	0	1	0	0.1218695	4	1	1.26272838	0.1218695
chr6	62944608	63197995	253388	3	0	1	0	0.1218695	3	1	0.93173516	0.1218695
chr6	63197995	63608317	410323	3	0	1	0	0.30102999	3	0	0.93173516	0
chr6	63608317	63608376	60	1	1	1	0.01091641	0.30102999	4	0	0.76005302	0
chr6	63608376	63928733	320358	2	0	1	0	0.30102999	4	0	1.26272838	0
chr6	63928733	63928792	60	1	1	1	0.01091641	0.30102999	4	0	0.76005302	0
chr6	63928792	64016348	87557	2	0	0	0	0	3	0	0.93173516	0
chr6	64016348	64147240	130893	3	1	0	0.01091641	0	4	0	0.76005302	0
chr6	64147240	64212199	64960	2	1	0	0.02438896	0	3	0	0.51676182	0
chr6	64212199	64292547	80349	2	1	0	0.01091641	0	4	0	0.76005302	0
chr6	64292547	64505835	213289	5	1	0	0.02438896	0	3	0	0.51676182	0
chr6	64505835	64619267	113433	3	1	0	0.01091641	0	4	0	0.76005302	0
chr6	64619267	65087308	468042	3	1	0	0.00478973	0	5	0	1.02643191	0
chr6	65087308	65270447	183140	1	0	0	0	0	4	0	1.26272838	0
chr6	65270447	65321229	50783	2	1	0	0.01091641	0	4	0	0.76005302	0
chr6	65321229	65321288	60	1	2	0	0.0429175	0	4	1	0.47744371	0.30102999
chr6	65321288	65495896	174609	3	2	0	0.0429175	0	4	0	0.47744371	0
chr6	65495896	65662415	166520	2	1	0	0.01091641	0	4	0	0.76005302	0
chr6	65662415	65794022	131608	3	2	0	0.0429175	0	4	0	0.47744371	0
chr6	65794022	65972753	178732	2	3	0	0.05670724	0	5	0	0.45545077	0
chr6	65972753	66008616	35864	2	3	0	0.03070643	0	6	1	0.63695542	0.30102999
chr6	66008616	66064180	55565	1	3	0	0.05670724	0	5	1	0.45545077	0.30102999
chr6	66064180	66064239	60	1	3	0	0.03070643	0	6	1	0.63695542	0.30102999
chr6	66064239	66205374	141136	3	3	0	0.05670724	0	5	1	0.45545077	0.30102999
chr6	66205374	66205433	60	1	3	0	0.03070643	0	6	1	0.63695542	0.30102999
chr6	66205433	66280326	74894	1	3	0	0.10122019	0	4	0	0.30102999	0
chr6	66280326	66316494	36169	2	3	0	0.05670724	0	5	0	0.45545077	0
chr6	66316494	66407551	91058	2	3	0	0.10122019	0	4	0	0.30102999	0
chr6	66407551	66407610	60	1	3	0	0.05670724	0	5	0	0.45545077	0
chr6	66407610	66497520	89911	1	2	0	0.0429175	0	4	0	0.47744371	0
chr6	66497520	66536131	38612	2	2	0	0.02162467	0	5	0	0.68214471	0
chr6	66536131	66596815	60685	1	2	0	0.0429175	0	4	0	0.47744371	0
chr6	66596815	67164064	567250	5	1	0	0.01091641	0	4	0	0.76005302	0
chr6	67164064	67396853	232790	2	1	0	0.02438896	0	3	0	0.51676182	0
chr6	67396853	67576468	179616	1	1	0	0.05404976	0	2	0	0.30102999	0
chr6	67576468	68686165	1109698	8	0	0	0	0	1	0	0.30102999	0
chr6	68686165	69053845	367681	3	0	1	0	0.30102999	2	0	0.61140001	0
chr6	69053845	69053904	60	1	0	2	0	0.61140001	2	0	0.61140001	0
chr6	69053904	69346523	292620	2	0	1	0	0.30102999	2	0	0.61140001	0
chr6	69346523	69347332	810	2	0	1	0	0.1218695	2	1	0.61140001	0.1218695
chr6	69347332	69484862	137531	2	0	0	0	0	2	0	0.61140001	0
chr6	69484862	69484921	60	1	0	1	0	0.1218695	2	1	0.61140001	0.1218695
chr6	69484921	69713520	228600	4	0	0	0	0	2	1	0.61140001	0.30102999
chr6	69713520	69772817	59298	2	1	0	0.05404976	0	2	1	0.30102999	0.30102999
chr6	69772817	69772876	60	1	1	0	0.01091641	0	4	1	0.76005302	0.30102999
chr6	69772876	69811169	38294	1	1	0	0.02438896	0	3	1	0.51676182	0.30102999
chr6	69811169	69844050	32882	1	1	0	0.02438896	0	3	0	0.51676182	0
chr6	69844050	69880154	36105	2	1	0	0.01091641	0	4	0	0.76005302	0
chr6	69880154	70071174	191021	4	1	0	0.00478973	0	5	1	1.02643191	0.30102999
chr6	70071174	70386079	314906	4	2	0	0.02162467	0	5	1	0.68214471	0.30102999
chr6	70386079	70386138	60	1	2	0	0.01053319	0	6	1	0.91219088	0.30102999
chr6	70386138	70566080	179943	4	1	0	0.00204627	0	6	1	1.31360226	0.30102999
chr6	70566080	70566139	60	1	2	0	0.01053319	0	6	1	0.91219088	0.30102999
chr6	70566139	71071757	505619	12	1	0	0.00204627	0	6	1	1.31360226	0.30102999
chr6	71071757	71137999	66243	2	1	0	8.47E-04	0	7	1	1.62048027	0.30102999
chr6	71137999	71298394	160396	5	2	0	0.00493743	0	7	1	1.16581773	0.30102999
chr6	71298394	71464771	166378	4	2	0	0.00221948	0	8	1	1.44210395	0.30102999
chr6	71464771	71567306	102536	3	2	0	0.00493743	0	7	1	1.16581773	0.30102999
chr6	71567306	71569123	1818	2	2	0	0.00221948	0	8	1	1.44210395	0.30102999
chr6	71569123	71633035	63913	2	2	0	0.00493743	0	7	1	1.16581773	0.30102999
chr6	71633035	71651793	18759	2	2	0	0.00221948	0	8	1	1.44210395	0.30102999
chr6	71651793	71816253	164461	2	1	0	3.39E-04	0	8	1	1.94674965	0.30102999
chr6	71816253	71816312	60	1	1	0	1.30E-04	0	9	2	2.29264207	0.61140001
chr6	71816312	71879888	63577	1	1	0	0.00204627	0	6	2	1.31360226	0.61140001
chr6	71879888	72003258	123371	1	1	0	0.00204627	0	6	1	1.31360226	0.30102999
chr6	72003258	72003317	60	1	1	0	3.39E-04	0	8	1	1.94674965	0.30102999
chr6	72003317	72043240	39924	3	1	0	0.00204627	0	6	1	1.31360226	0.30102999
chr6	72043240	72098992	55753	3	1	0	3.39E-04	0	8	1	1.94674965	0.30102999
chr6	72098992	72125934	26943	1	1	0	8.47E-04	0	7	1	1.62048027	0.30102999
chr6	72125934	72252519	126586	4	2	0	0.00493743	0	7	1	1.16581773	0.30102999
chr6	72252519	72385947	133429	2	1	0	8.47E-04	0	7	1	1.62048027	0.30102999
chr6	72385947	72597333	211387	3	2	0	0.00493743	0	7	1	1.16581773	0.30102999
chr6	72597333	72670292	72960	2	3	0	0.00378107	0	9	1	1.33111237	0.30102999
chr6	72670292	72802144	131853	4	3	0	0.00378107	0	9	2	1.33111237	0.61140001
chr6	72802144	72845116	42973	1	2	0	9.54E-04	0	9	2	1.74076927	0.61140001
chr6	72845116	72970802	125687	2	2	0	0.00221948	0	8	2	1.44210395	0.61140001
chr6	72970802	72970861	60	1	2	0	9.54E-04	0	9	2	1.74076927	0.61140001
chr6	72970861	73017052	46192	1	2	0	0.01053319	0	6	2	0.91219088	0.61140001
chr6	73017052	73110574	93523	2	1	0	0.00204627	0	6	2	1.31360226	0.61140001
chr6	73110574	73110633	60	1	2	0	0.01053319	0	6	2	0.91219088	0.61140001
chr6	73110633	73120895	10263	1	1	0	0.00478973	0	5	2	1.02643191	0.61140001
chr6	73120895	73228354	107460	1	1	0	0.01091641	0	4	2	0.76005302	0.61140001
chr6	73228354	73228413	60	1	1	0	0.00478973	0	5	2	1.02643191	0.61140001
chr6	73228413	73520690	292278	5	1	0	0.01091641	0	4	2	0.76005302	0.61140001
chr6	73520690	73565943	45254	2	1	0	0.00204627	0	6	2	1.31360226	0.61140001
chr6	73565943	73601499	35557	1	1	0	0.00204627	0	6	1	1.31360226	0.30102999
chr6	73601499	73783522	182024	4	1	0	8.47E-04	0	7	1	1.62048027	0.30102999
chr6	73783522	73972542	189021	4	1	0	0.00204627	0	6	1	1.31360226	0.30102999
chr6	73972542	73972596	55	1	2	0	0.01053319	0	6	1	0.91219088	0.30102999
chr6	73972596	74059598	87003	2	2	0	0.02162467	0	5	1	0.68214471	0.30102999
chr6	74059598	74201991	142394	5	1	0	0.01091641	0	4	1	0.76005302	0.30102999
chr6	74201991	74202047	57	1	2	0	0.01053319	0	6	1	0.91219088	0.30102999
chr6	74202047	74407262	205216	4	1	0	0.00478973	0	5	1	1.02643191	0.30102999

chr6	74407262	74407321	60	1	2	0	0.00493743	0	7	1	1.16581773	0.30102999
chr6	74407321	74468644	61324	2	1	0	0.00478973	0	5	1	1.02643191	0.30102999
chr6	74468644	74528260	59617	3	2	0	0.02162467	0	5	1	0.68214471	0.30102999
chr6	74528260	74890711	362452	5	1	0	0.00478973	0	5	1	1.02643191	0.30102999
chr6	74890711	74973157	82447	1	1	0	0.01091641	0	4	1	0.76005302	0.30102999
chr6	74973157	74973216	60	1	1	0	0.00478973	0	5	1	1.02643191	0.30102999
chr6	74973216	75138045	164830	2	1	0	0.01091641	0	4	0	0.76005302	0
chr6	75138045	75138104	60	1	1	0	0.00478973	0	5	0	1.02643191	0
chr6	75138104	75283958	145855	2	1	0	0.01091641	0	4	0	0.76005302	0
chr6	75283958	75350910	66953	2	1	0	0.00204627	0	6	0	1.31360226	0
chr6	75350910	75560010	209101	3	2	0	0.01053319	0	6	1	0.91219088	0.30102999
chr6	75560010	75705310	145301	1	1	0	0.00478973	0	5	1	1.02643191	0.30102999
chr6	75705310	75705369	60	1	1	0	0.00478973	0	5	2	1.02643191	0.61140001
chr6	75705369	75795148	89780	1	1	0	0.01091641	0	4	2	0.76005302	0.61140001
chr6	75795148	75893111	97964	4	1	0	0.00478973	0	5	2	1.02643191	0.61140001
chr6	75893111	75963130	70020	4	1	1	0.00478973	0.05404976	5	2	1.02643191	0.30102999
chr6	75963130	76072444	109315	3	0	0	0	0	4	1	1.26272838	0.30102999
chr6	76072444	76072679	236	2	1	0	0.00478973	0	5	2	1.02643191	0.61140001
chr6	76072679	76165034	92356	2	0	0	0	0	4	1	1.26272838	0.30102999
chr6	76165034	76551082	386049	11	0	0	0	0	4	2	1.26272838	0.61140001
chr6	76551082	76744026	192945	5	0	0	0	0	4	1	1.26272838	0.30102999
chr6	76744026	76786151	42126	2	0	0	0	0	5	1	1.60515106	0.30102999
chr6	76786151	76869202	83052	1	0	0	0	0	4	1	1.26272838	0.30102999
chr6	76869202	77173382	304181	2	0	0	0	0	3	1	0.93173516	0.30102999
chr6	77173382	77173441	60	1	0	0	0	0	5	2	1.60515106	0.61140001
chr6	77173441	77369645	196205	2	0	0	0	0	4	0	1.26272838	0
chr6	77369645	77575676	206032	2	0	0	0	0	1	0	0.30102999	0
chr6	77575676	77575735	60	1	0	0	0	0	2	1	0.61140001	0.30102999
chr6	77575735	78643149	1067415	11	0	0	0	0	1	0	0.30102999	0
chr6	78643149	78825593	182445	3	0	0	0	0	1	2	0.30102999	0.61140001
chr6	78825593	78825652	60	1	0	1	0	0.05404976	1	2	0.30102999	0.30102999
chr6	78825652	78926616	100965	1	0	0	0	0	1	2	0.30102999	0.61140001
chr6	78926616	79141799	215184	1	0	0	0	0	1	1	0.30102999	0.30102999
chr6	79141799	79326430	184632	2	1	0	0.02438896	0	3	1	0.51676182	0.30102999
chr6	79326430	79571628	245199	2	1	0	0.01091641	0	4	2	0.76005302	0.61140001
chr6	79571628	79735637	164010	5	1	0	0.00478973	0	5	2	1.02643191	0.61140001
chr6	79735637	79910951	175315	3	2	0	0.01053319	0	6	2	0.91219088	0.61140001
chr6	79910951	79911010	60	1	3	0	0.03070643	0	6	2	0.63695542	0.61140001
chr6	79911010	79981068	70059	2	2	0	0.01053319	0	6	2	0.91219088	0.61140001
chr6	79981068	80113727	132660	2	1	0	0.00204627	0	6	1	1.31360226	0.30102999
chr6	80113727	80635984	522258	11	3	0	0.03070643	0	6	2	0.63695542	0.61140001
chr6	80635984	80739840	103857	2	1	0	0.00204627	0	6	1	1.31360226	0.30102999
chr6	80739840	80752027	12188	2	2	0	0.01053319	0	6	2	0.91219088	0.61140001
chr6	80752027	80837316	85290	2	1	0	0.00204627	0	6	2	1.31360226	0.61140001
chr6	80837316	80989674	152359	2	0	0	0	0	4	1	1.26272838	0.30102999
chr6	80989674	81053470	63797	2	0	0	0	0	4	2	1.26272838	0.61140001
chr6	81053470	81091901	38432	2	0	0	0	0	5	2	1.60515106	0.61140001
chr6	81091901	81496783	404883	4	0	0	0	0	4	1	1.26272838	0.30102999
chr6	81496783	81765437	268655	1	0	0	0	0	3	1	0.93173516	0.30102999
chr6	81765437	82004749	239313	1	0	0	0	0	2	1	0.61140001	0.30102999
chr6	82363693	82577829	214137	4	0	0	0	0	1	0	0.30102999	0
chr6	82742906	82840207	97302	2	0	0	0	0	2	0	0.61140001	0
chr6	82840207	82880600	40394	2	0	0	0	0	3	1	0.93173516	0.30102999
chr6	82880600	82901165	20566	2	1	0	0.01091641	0	4	1	0.76005302	0.30102999
chr6	82901165	82901224	60	1	1	1	0.01091641	0.1218695	4	1	0.76005302	0.1218695
chr6	82901224	82943930	42707	2	0	1	0	0.1218695	4	1	1.26272838	0.1218695
chr6	82943930	83575511	631582	6	1	1	0.00478973	0.05404976	5	2	1.02643191	0.30102999
chr6	83575511	83609207	33697	2	1	1	0.00204627	0.02438896	6	3	1.31360226	0.51676182
chr6	83609207	83708198	98992	3	1	1	0.00204627	0.01091641	6	4	1.31360226	0.76005302
chr6	83708198	83838681	130484	3	1	1	0.01091641	0.02438896	4	3	0.76005302	0.51676182
chr6	83838681	83878133	39453	2	1	1	0.00478973	0.01091641	5	4	1.02643191	0.76005302
chr6	83878133	83898531	20399	3	1	2	0.00478973	0.0429175	5	4	1.02643191	0.47744371
chr6	83898531	83963461	64931	3	1	1	0.00478973	0.01091641	5	4	1.02643191	0.76005302
chr6	83963461	84108173	144713	3	1	1	0.01091641	0.01091641	4	4	0.76005302	0.76005302
chr6	84108173	84884683	776511	17	1	2	0.00478973	0.0429175	5	4	1.02643191	0.47744371
chr6	84884683	84910643	25961	1	1	1	0.01091641	0.01091641	4	4	0.76005302	0.76005302
chr6	84910643	84980651	70009	1	0	1	0	0.01091641	3	4	0.93173516	0.76005302
chr6	84980651	84980710	60	1	0	2	0	0.0429175	3	4	0.93173516	0.47744371
chr6	84980710	85220808	240099	2	0	1	0	0.01091641	3	4	0.93173516	0.76005302
chr6	85220808	85385036	164229	2	1	2	0.01091641	0.0429175	4	4	0.76005302	0.47744371
chr6	85385036	85385095	60	1	1	2	0.00478973	0.0429175	5	4	1.02643191	0.47744371
chr6	85385095	85451167	66073	1	1	2	0.00478973	0.08289318	5	3	1.02643191	0.30102999
chr6	85451167	85566641	115475	1	1	0	0.00478973	0	5	3	1.02643191	0.93173516
chr6	85566641	85566700	60	1	1	0	0.00478973	0	5	4	1.02643191	1.26272838
chr6	85566700	85683552	116853	1	1	0	0.00478973	0	5	3	1.02643191	0.93173516
chr6	85683552	85867422	183871	1	0	0	0	0	5	3	1.60515106	0.93173516
chr6	85867422	85934568	67147	2	1	0	0.00478973	0	5	4	1.02643191	1.26272838
chr6	85934568	86080434	145867	2	0	0	0	0	5	3	1.60515106	0.93173516
chr6	86080434	86080493	60	1	1	0	0.00478973	0	5	4	1.02643191	1.26272838
chr6	86080493	86430805	350313	9	0	0	0	0	3	3	0.93173516	0.93173516
chr6	86430805	86655009	224205	3	0	0	0	0	2	3	0.61140001	0.93173516
chr6	86655009	86754494	99486	2	0	0	0	0	2	4	0.61140001	1.26272838
chr6	86754494	86884296	129803	2	1	0	0.05404976	0	2	4	0.30102999	1.26272838
chr6	86884296	87041125	156830	3	1	0	0.01091641	0	4	4	0.76005302	1.26272838
chr6	87041125	87041184	60	1	1	1	0.01091641	0.01091641	4	4	0.76005302	0.76005302
chr6	87041184	87178844	137661	1	1	0	0.01091641	0	4	3	0.76005302	0.93173516
chr6	87178844	87463929	285086	5	1	0	0.00204627	0	6	3	1.31360226	0.93173516
chr6	87463929	87534787	70859	2	1	0	0.00204627	0	6	4	1.31360226	1.26272838
chr6	87534787	87649685	114899	2	1	0	0.00478973	0	5	4	1.02643191	1.26272838
chr6	87649685	87649744	60	1	1	0	0.00204627	0	6	4	1.31360226	1.26272838
chr6	87649744	87862808	213065	7	1	0	0.00478973	0	5	4	1.02643191	1.26272838
chr6	87862808	87973967	111160	3	1	2	0.00478973	0.0429175	5	4	1.02643191	0.47744371
chr6	87973967	88032817	58851	2	1	2	0.01091641	0.0429175	4	4	0.76005302	0.47744371
chr6	88032817	88255387	222571	9	1	2	0.01091641	0.08289318	4	3	0.76005302	0.30102999
chr6	88255387	88293352	37966	2	1	2	0.02438896	0.08289318	3	3	0.51676182	0.30102999
chr6	88293352	88417066	123715	6	1	2	0.01091641	0.08289318	4	3	0.76005302	0.30102999
chr6	88417066	88569431	152366	2	1	1	0.01091641	0.02438896	4	3	0.76005302	0.51676182
chr6	88569431	88624018	54588	1	1	1	0.02438896	0.02438896	3	3	0.51676182	0.51676182
chr6	88624018	88768460	144443	1	1	1	0.02438896	0.05404976	3	2	0.51676182	0.30102999
chr6	88768460	88862223	93764	5	2	1	0.02162467	0.05404976	5	2	0.68214471	0.30102999
chr6	88862223	88977956	115734	1	2	0	0.0429175	0	4	2	0.47744371	0.61140001
chr6	88977956	89033980	56025	1	2	0	0.08289318	0	3	2	0.30102999	0.61140001
chr6	89033980	89227714	193735	2	2	0	0.0429175	0	4	2	0.47744371	0.61140001
chr6	89227714	89339942	112229	2	2	1	0.02162467	0.02438896	5	3	0.68214471	0.51676182
chr6	89339942	89388107	48166	1	2	1	0.02162467	0.05404976	5	2	0.68214471	0.30102999
chr6	89388107	89781268	393162	8	1	1	0.01091641	0.05404976	4	2	0.76005302	0.30102999
chr6	89781268	89826057	44790	2	1	2	0.00478973	0.1575501	5	2	1.02643191	0.1575501
chr6	89826057	90241133	415077	12	1	1	0.00478973	0.05404976	5	2	1.02643191	0.30102999
chr6	90241133	90453427	212295	5	1	2	0.00478973	0.1575501	5	2	1.02643191	0.1575501
chr6	90453427	90707105	253679	7	1	1	0.00478973	0.05404976	5	2	1.02643191	0.30102999
chr6	90707105	90865892	158788	3	1	0	0.00478973	0	5	2	1.02643191	0.61140001
chr6	90865892	90900628	34737	2	1	1	0.00478973	0.05404976	5	2	1.02643191	0.30102999
chr6	90900628	90900687	60	1	1	2	0.00478973	0.1575501	5	2	1.02643191	0.1575501
chr6	90900687	91004146	103460	2	1	1	0.00478973	0.05404976	5	2	1.02643191	0.30102999
chr6	91004146	91281399	277254	5	1	1	0.01091641	0.1218695	4	1	0.76005302	0.1218695
chr6	91281399	91281458	60	1	1	1	0.01091641	0.05404976	4	2	0.76005302	0.30102999
chr6	91281458	91379512	98055	1	1	1	0.01091641	0.1218695	4	1	0.76005302	0.1218695
chr6	91379512	91598727	219216	1	0	0	0	0	3	1	0.93173516	0.30102999
chr6	91598727	91833379	234653	1	0	0	0	0	2	0	0.61140001	0
chr6	91833379	92045753	212375	1	0	0	0	0	1	0	0.30102999	0
chr6	92203313	92203372	60	1	0	0	0	0	1	0	0.30102999	0
chr6	92573175	93007777	434603	4	0	0	0	0	1	0	0.30102999	0
chr6	93007777	93007836	60	1	1	0	0.05404976	0	2	1	0.30102999	0.30102999
chr6	93007836	93176783	168948	1	0	0	0	0	2	1	0.61140001	0.30102999

chr6	93176783	93342107	165325	2	0	0	0	0	4	1	1.26272838	0.30102999
chr6	93342107	93502656	160550	1	0	0	0	0	3	1	0.93173516	0.30102999
chr6	93502656	93553893	51238	2	0	0	0	0	4	1	1.26272838	0.30102999
chr6	93553893	93609026	55134	1	0	0	0	0	3	1	0.93173516	0.30102999
chr6	93609026	93720661	111636	2	0	1	0	0.1218695	3	1	0.93173516	0.1218695
chr6	93720661	94091543	370883	6	0	0	0	0	2	1	0.61140001	0.30102999
chr6	94091543	94181249	89707	4	0	0	0	0	3	1	0.93173516	0.30102999
chr6	94181249	94292552	111304	2	0	1	0	0.1218695	4	1	1.26272838	0.1218695
chr6	94292552	94487830	195279	1	0	1	0	0.1218695	3	1	0.93173516	0.1218695
chr6	94487830	94586500	98671	2	0	2	0	0.30102999	3	1	0.93173516	0.05404976
chr6	94586500	94586559	60	1	0	2	0	0.1575501	3	2	0.93173516	0.1575501
chr6	94586559	94783161	196603	1	0	1	0	0.1218695	2	1	0.61140001	0.1218695
chr6	94783161	95078973	295813	2	0	0	0	0	1	1	0.30102999	0.30102999
chr6	95078973	95079032	60	1	0	2	0	0.30102999	2	1	0.61140001	0.05404976
chr6	95079032	95148784	69753	1	0	0	0	0	2	1	0.61140001	0.30102999
chr6	95417726	95584631	166906	2	0	0	0	0	2	0	0.61140001	0
chr6	95584631	95584690	60	1	0	0	0	0	4	1	1.26272838	0.30102999
chr6	95584690	96037310	452621	2	0	0	0	0	3	0	0.93173516	0
chr6	96037310	96400185	362876	5	1	0	0.01091641	0	4	1	0.76005302	0.30102999
chr6	96400185	96400244	60	1	1	1	0.00478973	0.05404976	5	2	1.02643191	0.30102999
chr6	96400244	96533522	133279	4	1	1	0.01091641	0.1218695	4	1	0.76005302	0.1218695
chr6	96533522	96569190	35669	2	1	2	0.01091641	0.1575501	4	2	0.76005302	0.1575501
chr6	96569190	96842882	273693	3	1	1	0.01091641	0.05404976	4	2	0.76005302	0.30102999
chr6	96842882	96976463	133582	2	2	2	0.00493743	0.1575501	7	2	1.16581773	0.1575501
chr6	96976463	96976522	60	1	2	2	0.00493743	0.08289318	7	3	1.16581773	0.30102999
chr6	96976522	96984286	7765	1	2	2	0.01053319	0.08289318	6	3	0.91219088	0.30102999
chr6	96984286	97010479	26194	1	2	1	0.01053319	0.1218695	6	1	0.91219088	0.1218695
chr6	97010479	97010537	59	1	2	1	0.00493743	0.1218695	7	1	1.16581773	0.1218695
chr6	97010537	97163116	152580	3	2	0	0.00493743	0	7	1	1.16581773	0.30102999
chr6	97163116	97446683	283568	7	3	0	0.01598258	0	7	1	0.84395715	0.30102999
chr6	97446683	97489438	42756	1	2	0	0.01053319	0	6	1	0.91219088	0.30102999
chr6	97489438	98035068	545631	13	2	0	0.01053319	0	6	2	0.91219088	0.61140001
chr6	98035068	98035127	60	1	2	0	0.01053319	0	6	3	0.91219088	0.93173516
chr6	98035127	98124957	89831	1	1	0	0.00478973	0	5	1	1.02643191	0.30102999
chr6	98124957	98219291	94335	1	1	0	0.01091641	0	4	1	0.76005302	0.30102999
chr6	98219291	98219350	60	1	1	0	0.00478973	0	5	2	1.02643191	0.61140001
chr6	98219350	98319087	99738	1	1	0	0.00478973	0	5	1	1.02643191	0.30102999
chr6	98319087	98319146	60	1	1	0	0.00204627	0	6	3	1.31360226	0.93173516
chr6	98319146	98463256	144111	1	1	0	0.05404976	0	2	2	0.30102999	0.61140001
chr6	98463256	98726579	263324	2	1	0	0.1218695	0	1	0	0.1218695	0
chr6	98726579	99082388	355810	3	1	0	0.1218695	0	1	1	0.1218695	0.30102999
chr6	99082388	99082447	60	1	2	0	0.02162467	0	5	3	0.68214471	0.93173516
chr6	99082447	99284234	201788	3	1	0	0.02438896	0	3	3	0.51676182	0.93173516
chr6	99284234	99285919	1686	2	1	0	0.01091641	0	4	3	0.76005302	0.93173516
chr6	99285919	99394111	108193	2	1	0	0.05404976	0	2	3	0.30102999	0.93173516
chr6	99394111	99394170	60	1	1	1	0.01091641	0.01091641	4	4	0.76005302	0.76005302
chr6	99394170	99461950	67781	1	1	1	0.02438896	0.02438896	3	3	0.51676182	0.51676182
chr6	99461950	99609664	147715	1	1	1	0.05404976	0.02438896	2	3	0.30102999	0.51676182
chr6	99609664	99609723	60	1	1	2	0.02438896	0.0429175	3	4	0.51676182	0.47744371
chr6	99609723	99728805	119083	1	1	1	0.02438896	0.02438896	3	3	0.51676182	0.51676182
chr6	99728805	99817320	88516	3	1	1	0.01091641	0.02438896	4	3	0.76005302	0.51676182
chr6	99817320	99817473	154	2	1	2	0.01091641	0.08289318	4	3	0.76005302	0.30102999
chr6	99817473	99872538	55066	2	1	1	0.01091641	0.02438896	4	3	0.76005302	0.51676182
chr6	99872538	99891473	18936	2	1	2	0.01091641	0.08289318	4	3	0.76005302	0.30102999
chr6	99891473	99961506	70034	2	1	1	0.02438896	0.02438896	3	3	0.51676182	0.51676182
chr6	99961506	100260928	299423	7	1	2	0.00478973	0.0429175	5	4	1.02643191	0.47744371
chr6	100260928	100415543	154616	3	2	2	0.02162467	0.0429175	5	4	0.68214471	0.47744371
chr6	100415543	100415602	60	1	2	2	0.01053319	0.0429175	6	4	0.91219088	0.47744371
chr6	100415602	100510551	94950	2	2	2	0.02162467	0.0429175	5	4	0.68214471	0.47744371
chr6	100510551	100510610	60	1	2	2	0.01053319	0.0429175	6	4	0.91219088	0.47744371
chr6	100510610	100642782	132173	2	2	2	0.02162467	0.0429175	5	4	0.68214471	0.47744371
chr6	100642782	100794653	151872	2	2	2	0.01053319	0.0429175	6	4	0.91219088	0.47744371
chr6	100794653	100794712	60	1	2	3	0.01053319	0.10122019	6	4	0.91219088	0.30102999
chr6	100794712	100957238	162527	4	1	2	0.00204627	0.0429175	6	4	1.31360226	0.47744371
chr6	100957238	101099518	142281	3	1	2	0.00478973	0.08289318	5	3	1.02643191	0.30102999
chr6	101099518	101151700	52183	1	1	2	0.00478973	0.1575501	5	2	1.02643191	0.1575501
chr6	101151700	101151759	60	1	1	2	0.00204627	0.1575501	6	2	1.31360226	0.1575501
chr6	101151759	101200326	48568	1	1	0	0.00204627	0	6	2	1.31360226	0.61140001
chr6	101200326	101254389	54064	2	2	0	0.01053319	0	6	2	0.91219088	0.61140001
chr6	101254389	101318404	64016	3	2	0	0.0429175	0	4	2	0.47744371	0.61140001
chr6	101318404	101401778	83375	1	2	0	0.0429175	0	4	1	0.47744371	0.30102999
chr6	101401778	101401837	60	1	2	2	0.02162467	0.30102999	5	1	0.68214471	0.05404976
chr6	101401837	101568080	166244	1	2	1	0.0429175	0.30102999	4	0	0.47744371	0
chr6	101568080	101568139	60	1	3	1	0.10122019	0.30102999	4	0	0.30102999	0
chr6	101568139	101737120	168982	1	2	1	0.0429175	0.30102999	4	0	0.47744371	0
chr6	101737120	101944677	207558	2	1	1	0.01091641	0.30102999	4	0	0.76005302	0
chr6	101944677	101944736	60	1	1	2	0.01091641	0.61140001	4	0	0.76005302	0
chr6	101944736	102192907	248172	5	1	1	0.01091641	0.30102999	4	0	0.76005302	0
chr6	102192907	102444345	251439	4	1	0	0.01091641	0	4	0	0.76005302	0
chr6	102444345	102513743	69399	3	2	0	0.0429175	0	4	0	0.47744371	0
chr6	102513743	102772530	258788	2	1	0	0.01091641	0	4	0	0.76005302	0
chr6	102772530	102931873	159344	2	1	1	0.01091641	0.30102999	4	0	0.76005302	0
chr6	102931873	103179934	248062	1	0	1	0	0.30102999	4	0	1.26272838	0
chr6	103179934	103405100	225167	2	0	1	0	0.30102999	2	0	0.61140001	0
chr6	104137729	104454132	316404	3	0	0	0	0	3	0	0.93173516	0
chr6	104454132	104564985	110854	2	0	0	0	0	4	0	1.26272838	0
chr6	104564985	104815168	250184	3	0	1	0	0.30102999	4	0	1.26272838	0
chr6	104815168	104978927	163760	2	1	1	0.01091641	0.30102999	4	0	0.76005302	0
chr6	104978927	105133746	154820	2	2	1	0.0429175	0.30102999	4	0	0.47744371	0
chr6	105133746	105133805	60	1	2	1	0.02162467	0.1218695	5	1	0.68214471	0.1218695
chr6	105133805	105306830	173026	5	2	0	0.02162467	0	5	0	0.68214471	0
chr6	105306830	105548869	242040	6	2	0	0.02162467	0	5	1	0.68214471	0.30102999
chr6	105548869	106127139	578271	9	2	0	0.01053319	0	6	1	0.91219088	0.30102999
chr6	106127139	106127198	60	1	2	0	0.00493743	0	7	1	1.16581773	0.30102999
chr6	106127198	106257017	129820	1	2	0	0.01053319	0	6	1	0.91219088	0.30102999
chr6	106257017	106383483	126467	1	2	0	0.0429175	0	4	1	0.47744371	0.30102999
chr6	106383483	106543543	160061	2	3	0	0.05670724	0	5	1	0.45545077	0.30102999
chr6	106543543	106557540	13998	2	4	0	0.11390336	0	5	1	0.30102999	0.30102999
chr6	106557540	106660565	103026	2	3	0	0.10122019	0	4	1	0.30102999	0.30102999
chr6	106660565	106756225	95661	3	3	0	0.05670724	0	5	1	0.45545077	0.30102999
chr6	106756225	106885657	129433	3	3	0	0.01598258	0	7	1	0.84395715	0.30102999
chr6	106885657	107021243	135587	4	3	1	0.01598258	0.1218695	7	1	0.84395715	0.1218695
chr6	107021243	107186150	164908	3	3	1	0.03070643	0.1218695	6	1	0.63695542	0.1218695
chr6	107186150	107186209	60	1	4	1	0.06713722	0.1218695	6	1	0.44141547	0.1218695
chr6	107186209	107289757	103549	3	3	1	0.03070643	0.1218695	6	1	0.63695542	0.1218695
chr6	107289757	107397093	107337	2	3	1	0.05670724	0.1218695	5	1	0.45545077	0.1218695
chr6	107397093	107555834	158742	3	3	1	0.03070643	0.1218695	6	1	0.63695542	0.1218695
chr6	107555834	107555893	60	1	3	2	0.03070643	0.30102999	6	1	0.63695542	0.05404976
chr6	107555893	107595372	39480	1	3	2	0.05670724	0.30102999	5	1	0.45545077	0.05404976
chr6	107595372	107681884	86513	2	3	1	0.05670724	0.1218695	5	1	0.45545077	0.1218695
chr6	107681884	107681943	60	1	3	2	0.03070643	0.30102999	6	1	0.63695542	0.05404976
chr6	107681943	107814126	132184	3	3	2	0.05670724	0.30102999	5	1	0.45545077	0.05404976
chr6	107814126	107865086	50961	1	3	1	0.05670724	0.1218695	5	1	0.45545077	0.1218695
chr6	107865086	107906482	41397	2	3	1	0.05670724	0.05404976	5	2	0.45545077	0.30102999
chr6	107906482	108040267	133786	3	3	2	0.05670724	0.1575501	5	2	0.45545077	0.1575501
chr6	108040267	108040324	58	1	4	3	0.11390336	0.30102999	5	2	0.30102999	0.08289318
chr6	108040324	108109054	68731	2	3	3	0.05670724	0.30102999	5	2	0.45545077	0.08289318
chr6	108109054	108134831	25778	1	3	2	0.05670724	0.1575501	5	2	0.45545077	0.1575501
chr6	108134831	108227875	93045	2	3	2	0.10122019	0.1575501	4	2	0.30102999	0.1575501
chr6	108227875	108342425	114551	3	3	2	0.05670724	0.1575501	5	2	0.45545077	0.1575501
chr6	108342425	108342484	60	1	3	2	0.03070643	0.1575501	6	2	0.63695542	0.1575501
chr6	108342484	108385404	42921	1	3	2	0.05670724	0.1575501	5	2	0.45545077	0.1575501
chr6	108385404	108395268	9865	2	3	2	0.05670724	0.08289318	5	3	0.45545077	0.30102999
chr6	108395268	108488469	93202	1	3	2	0.10122019	0.08289318	4	3	0.30102999	0.30102999

chr6	108488469	108509490	21022	2	3	2	0.05670724	0.08289318	5	3	0.45545077	0.30102999
chr6	108509490	108645210	135721	4	3	2	0.10122019	0.08289318	4	3	0.30102999	0.30102999
chr6	108645210	108729564	84355	2	3	2	0.17593012	0.08289318	3	3	0.17593012	0.30102999
chr6	108729564	108729623	60	1	3	2	0.10122019	0.08289318	4	3	0.30102999	0.30102999
chr6	108729623	108768497	38875	1	2	2	0.0429175	0.08289318	4	3	0.47744371	0.30102999
chr6	108768497	109066059	297563	7	2	2	0.08289318	0.1575501	3	2	0.30102999	0.1575501
chr6	109066059	109066118	60	1	2	3	0.08289318	0.30102999	3	2	0.30102999	0.08289318
chr6	109066118	109121485	55368	1	2	2	0.08289318	0.1575501	3	2	0.30102999	0.1575501
chr6	109121485	109121544	60	1	2	2	0.0429175	0.1575501	4	2	0.47744371	0.1575501
chr6	109121544	109225480	103937	3	2	2	0.08289318	0.1575501	3	2	0.30102999	0.1575501
chr6	109225480	109249282	23803	2	2	2	0.0429175	0.1575501	4	2	0.47744371	0.1575501
chr6	109249282	109249341	60	1	3	2	0.10122019	0.1575501	4	2	0.30102999	0.1575501
chr6	109249341	109334883	85543	2	2	2	0.08289318	0.1575501	3	2	0.30102999	0.1575501
chr6	109334883	109450490	115608	4	3	2	0.17593012	0.1575501	3	2	0.17593012	0.1575501
chr6	109450490	109450549	60	1	3	2	0.10122019	0.1575501	4	2	0.30102999	0.1575501
chr6	109450549	109634985	184437	3	2	2	0.08289318	0.1575501	3	2	0.30102999	0.1575501
chr6	109634985	109692872	57888	2	2	3	0.08289318	0.30102999	3	2	0.30102999	0.08289318
chr6	109692872	109721292	28421	2	2	3	0.0429175	0.30102999	4	2	0.47744371	0.08289318
chr6	109721292	109801874	80583	2	2	2	0.0429175	0.1575501	4	2	0.47744371	0.1575501
chr6	109801874	109820288	18415	2	2	3	0.0429175	0.30102999	4	2	0.47744371	0.08289318
chr6	109820288	110064342	244055	5	2	2	0.0429175	0.1575501	4	2	0.47744371	0.1575501
chr6	110064342	110123960	59619	1	2	2	0.08289318	0.1575501	3	2	0.30102999	0.1575501
chr6	110123960	110124019	60	1	2	3	0.08289318	0.30102999	3	2	0.30102999	0.08289318
chr6	110124019	110243528	119510	2	2	2	0.08289318	0.1575501	3	2	0.30102999	0.1575501
chr6	110243528	110301858	58331	2	2	1	0.08289318	0.05404976	3	2	0.30102999	0.30102999
chr6	110301858	110434581	132724	2	2	1	0.08289318	0.1218695	3	1	0.30102999	0.1218695
chr6	110434581	110481954	47374	2	2	1	0.0429175	0.1218695	4	1	0.47744371	0.1218695
chr6	110481954	110575427	93474	2	2	1	0.08289318	0.1218695	3	1	0.30102999	0.1218695
chr6	110575427	110607187	31761	2	2	1	0.0429175	0.1218695	4	1	0.47744371	0.1218695
chr6	110607187	110720327	113141	3	2	1	0.0429175	0.05404976	4	2	0.47744371	0.30102999
chr6	110720327	110727203	6877	2	2	2	0.0429175	0.1575501	4	2	0.47744371	0.1575501
chr6	110727203	110869110	141908	3	2	1	0.0429175	0.05404976	4	2	0.47744371	0.30102999
chr6	110869110	110869169	60	1	2	2	0.0429175	0.1575501	4	2	0.47744371	0.1575501
chr6	110869169	110932222	63054	2	2	1	0.0429175	0.05404976	4	2	0.47744371	0.30102999
chr6	110932222	110985114	52893	2	2	1	0.08289318	0.05404976	3	2	0.30102999	0.30102999
chr6	110985114	111212812	227699	7	2	1	0.0429175	0.05404976	4	2	0.47744371	0.30102999
chr6	111212812	111286981	74170	3	2	2	0.0429175	0.1575501	4	2	0.47744371	0.1575501
chr6	111286981	111321617	34637	1	2	1	0.0429175	0.05404976	4	2	0.47744371	0.30102999
chr6	111321617	111371082	49466	2	2	1	0.02162467	0.05404976	5	2	0.68214471	0.30102999
chr6	111371082	111426241	55160	1	2	1	0.0429175	0.05404976	4	2	0.47744371	0.30102999
chr6	111426241	111768179	341939	14	2	2	0.0429175	0.1575501	4	2	0.47744371	0.1575501
chr6	111768179	111936808	168630	4	2	2	0.02162467	0.1575501	5	2	0.68214471	0.1575501
chr6	111936808	112167791	230984	5	2	2	0.0429175	0.1575501	4	2	0.47744371	0.1575501
chr6	112167791	112167850	60	1	2	2	0.02162467	0.1575501	5	2	0.68214471	0.1575501
chr6	112167850	112486396	318547	8	2	2	0.0429175	0.1575501	4	2	0.47744371	0.1575501
chr6	112486396	112574984	88589	2	2	2	0.08289318	0.1575501	3	2	0.30102999	0.1575501
chr6	112574984	112606743	31760	2	2	2	0.0429175	0.1575501	4	2	0.47744371	0.1575501
chr6	112606743	112763483	156741	2	2	2	0.08289318	0.1575501	3	2	0.30102999	0.1575501
chr6	112763483	112763542	60	1	3	2	0.17593012	0.1575501	3	2	0.17593012	0.1575501
chr6	112763542	113044405	280864	2	2	2	0.1575501	0.1575501	2	2	0.1575501	0.1575501
chr6	113044405	113141536	97132	2	2	2	0.08289318	0.1575501	3	2	0.30102999	0.1575501
chr6	113141536	113141595	60	1	2	3	0.08289318	0.30102999	3	2	0.30102999	0.08289318
chr6	113141595	113301556	159962	1	2	3	0.1575501	0.30102999	2	2	0.1575501	0.08289318
chr6	113301556	113381983	80428	1	2	2	0.1575501	0.1575501	2	2	0.1575501	0.1575501
chr6	113381983	113579004	197022	2	2	1	0.1575501	0.1218695	2	1	0.1575501	0.1218695
chr6	113579004	113579063	60	1	2	1	0.0429175	0.1218695	4	1	0.47744371	0.1218695
chr6	113579063	113904236	325174	2	2	1	0.1575501	0.1218695	2	1	0.1575501	0.1218695
chr6	113904236	114179637	275402	4	2	0	0.1575501	0	2	1	0.1575501	0.30102999
chr6	114179637	114376864	197228	7	2	1	0.1575501	0.1218695	2	1	0.1575501	0.1218695
chr6	114376864	114940863	564000	7	2	0	0.1575501	0	2	1	0.1575501	0.30102999
chr6	114940863	115190171	249309	3	2	1	0.1575501	0.1218695	2	1	0.1575501	0.1218695
chr6	115190171	115701467	511297	2	1	0	0.05404976	0	2	0	0.30102999	0
chr6	115701467	115701526	60	1	3	1	0.30102999	0.30102999	2	0	0.08289318	0
chr6	115701526	115985253	283728	2	2	1	0.1575501	0.30102999	2	0	0.1575501	0
chr6	115985253	115985312	60	1	2	2	0.1575501	0.30102999	2	1	0.1575501	0.05404976
chr6	115985312	116125497	140186	1	1	2	0.05404976	0.30102999	2	1	0.30102999	0.05404976
chr6	116125497	116262708	137212	2	1	2	0.02438896	0.1575501	3	2	0.51676182	0.1575501
chr6	116262708	116289909	27202	2	2	2	0.0429175	0.1575501	4	2	0.47744371	0.1575501
chr6	116289909	116480091	190183	4	2	2	0.1575501	0.1575501	2	2	0.1575501	0.1575501
chr6	116480091	116698123	218033	7	3	2	0.30102999	0.1575501	2	2	0.08289318	0.1575501
chr6	116698123	116804635	106513	3	3	2	0.30102999	0.08289318	2	3	0.08289318	0.30102999
chr6	116804635	116804694	60	1	3	2	0.10122019	0.08289318	4	3	0.30102999	0.30102999
chr6	116804694	117454715	650022	12	3	2	0.17593012	0.08289318	3	3	0.17593012	0.30102999
chr6	117454715	117629934	175220	3	2	2	0.08289318	0.08289318	3	3	0.30102999	0.30102999
chr6	117629934	117681198	51265	3	3	2	0.17593012	0.08289318	3	3	0.17593012	0.30102999
chr6	117681198	117824987	143790	3	2	2	0.08289318	0.08289318	3	3	0.30102999	0.30102999
chr6	117824987	117846542	21556	1	2	2	0.1575501	0.08289318	2	3	0.1575501	0.30102999
chr6	117846542	117901307	54766	4	3	2	0.30102999	0.08289318	2	3	0.08289318	0.30102999
chr6	117901307	117955439	54133	1	3	1	0.30102999	0.02438896	2	3	0.08289318	0.51676182
chr6	117955439	118202946	247508	3	2	1	0.1575501	0.05404976	2	2	0.1575501	0.30102999
chr6	118202946	118203005	60	1	2	1	0.08289318	0.05404976	3	2	0.30102999	0.30102999
chr6	118203005	118270842	67838	1	2	1	0.1575501	0.05404976	2	2	0.1575501	0.30102999
chr6	118270842	118337197	66356	2	2	1	0.1575501	0.02438896	2	3	0.1575501	0.51676182
chr6	118337197	118417529	80333	2	2	1	0.08289318	0.02438896	3	3	0.30102999	0.51676182
chr6	118417529	118507150	89622	3	3	1	0.17593012	0.02438896	3	3	0.17593012	0.51676182
chr6	118507150	118638707	131558	2	3	1	0.30102999	0.02438896	2	3	0.08289318	0.51676182
chr6	118638707	118830249	191543	4	3	1	0.10122019	0.02438896	4	3	0.30102999	0.51676182
chr6	118830249	118830308	60	1	3	1	0.05670724	0.02438896	5	3	0.45545077	0.51676182
chr6	118830308	118931017	100710	3	3	1	0.10122019	0.02438896	4	3	0.30102999	0.51676182
chr6	118931017	119373008	441992	10	3	1	0.10122019	0.05404976	4	2	0.30102999	0.30102999
chr6	119373008	119641834	268827	10	3	1	0.05670724	0.05404976	5	2	0.45545077	0.30102999
chr6	119641834	119664466	22633	2	3	1	0.05670724	0.02438896	5	3	0.45545077	0.51676182
chr6	119664466	119834413	169948	2	3	1	0.10122019	0.05404976	4	2	0.30102999	0.30102999
chr6	119834413	119834472	60	1	3	1	0.05670724	0.02438896	5	3	0.45545077	0.51676182
chr6	119834472	120127891	293420	2	3	1	0.17593012	0.02438896	3	3	0.17593012	0.51676182
chr6	120127891	120320336	192446	3	3	1	0.10122019	0.02438896	4	3	0.30102999	0.51676182
chr6	120320336	120670412	350077	3	1	1	0.01091641	0.1218695	4	1	0.76005302	0.1218695
chr6	120670412	120670471	60	1	1	1	0.01091641	0.05404976	4	2	0.76005302	0.30102999
chr6	120670471	120810231	139761	1	1	0	0.02438896	0	3	2	0.51676182	0.61140001
chr6	120810231	121056833	246603	3	1	0	0.02438896	0	3	1	0.51676182	0.30102999
chr6	121056833	121056892	60	1	1	0	0.00478973	0	5	1	1.02643191	0.30102999
chr6	121056892	121410561	353670	2	1	0	0.01091641	0	4	1	0.76005302	0.30102999
chr6	121410561	121494947	84387	3	1	0	0.00478973	0	5	1	1.02643191	0.30102999
chr6	121494947	121604976	110030	2	1	0	0.01091641	0	4	1	0.76005302	0.30102999
chr6	121604976	121629158	24183	2	2	0	0.02162467	0	5	1	0.68214471	0.30102999
chr6	121629158	121767601	138444	4	1	0	0.00478973	0	5	1	1.02643191	0.30102999
chr6	121767601	121825781	58181	1	1	0	0.01091641	0	4	1	0.76005302	0.30102999
chr6	121825781	121947706	121926	1	1	0	0.02438896	0	3	1	0.51676182	0.30102999
chr6	121947706	121998186	50481	2	2	0	0.0429175	0	4	1	0.47744371	0.30102999
chr6	121998186	122068477	70292	2	2	1	0.0429175	0.1218695	4	1	0.47744371	0.1218695
chr6	122068477	122539011	470535	4	2	0	0.0429175	0	4	1	0.47744371	0.30102999
chr6	122539011	122539070	60	1	2	0	0.02162467	0	5	1	0.68214471	0.30102999
chr6	122539070	122792021	252952	3	2	0	0.0429175	0	4	1	0.47744371	0.30102999
chr6	122792021	122996250	204230	5	2	1	0.0429175	0.1218695	4	1	0.47744371	0.1218695
chr6	122996250	123046273	50024	2	3	1	0.10122019	0.1218695	4	1	0.30102999	0.1218695
chr6	123046273	123046332	60	1	3	1	0.05670724	0.1218695	5	1	0.45545077	0.1218695
chr6	123046332	123380660	334329	7	3	1	0.10122019	0.1218695	4	1	0.30102999	0.1218695
chr6	123380660	123380719	60	1	3	1	0.10122019	0.05404976	4	2	0.30102999	0.30102999
chr6	123380719	123539625	158907	1	2	1	0.0429175	0.05404976	4	2	0.47744371	0.30102999
chr6	123539625	123838001	298377	7	2	1	0.0429175	0.02438896	4	3	0.47744371	0.51676182
chr6	123838001	123888421	50421	1	2	1	0.08289318	0.02438896	3	3	0.30102999	0.51676182
chr6	123888421	123888480	60	1	2	2	0.08289318	0.08289318	3	3	0.30102999	0.30102999
chr6	123888480	123957926	69447	1	2	1	0.08289318	0.02438896	3	3	0.30102999	0.51676182
chr6	123957926	123957984	59	1	2	1	0.0429175	0.02438896	4	3	0.47744371	0.51676182

chr6	123957984	124125473	167490	2	2	0	0.0429175	0	4	3	0.47744371	0.93173516
chr6	124125473	124125532	60	1	2	1	0.0429175	0.02438896	4	3	0.47744371	0.51676182
chr6	124125532	124166602	41071	1	1	0	0.01091641	0	4	3	0.76005302	0.93173516
chr6	124166602	124351985	185384	4	1	1	0.01091641	0.02438896	4	3	0.76005302	0.51676182
chr6	124351985	124526914	174930	5	1	2	0.01091641	0.08289318	4	3	0.76005302	0.30102999
chr6	124526914	124653671	126758	3	2	2	0.02162467	0.08289318	5	3	0.68214471	0.30102999
chr6	124653671	124717712	64042	2	1	2	0.00478973	0.08289318	5	3	1.02643191	0.30102999
chr6	124717712	124883439	165728	3	0	2	0	0.08289318	4	3	1.26272838	0.30102999
chr6	124883439	125083105	199667	6	0	2	0	0.08289318	5	3	1.60515106	0.30102999
chr6	125083105	125304535	221431	3	0	1	0	0.02438896	4	3	1.26272838	0.51676182
chr6	125304535	126298928	994394	23	0	1	0	0.02438896	5	3	1.60515106	0.51676182
chr6	126298928	126298987	60	1	0	1	0	0.02438896	6	3	1.95986592	0.51676182
chr6	126298987	126391126	92140	2	0	0	0	0	6	3	1.95986592	0.93173516
chr6	126391126	126707405	316280	3	0	0	0	0	5	3	1.60515106	0.93173516
chr6	126707405	126749767	42363	2	0	0	0	0	6	3	1.95986592	0.93173516
chr6	126749767	126801112	51346	2	1	0	0.00204627	0	6	3	1.31360226	0.93173516
chr6	126801112	126974958	173847	3	1	0	0.00478973	0	5	3	1.02643191	0.93173516
chr6	126974958	127130705	155748	3	1	1	0.00204627	0.02438896	6	3	1.31360226	0.51676182
chr6	127130705	127518222	387518	8	2	1	0.01053319	0.02438896	6	3	0.91219088	0.51676182
chr6	127518222	127595065	76844	3	2	1	0.00493743	0.02438896	7	3	1.16581773	0.51676182
chr6	127595065	127663255	68191	4	3	1	0.01598258	0.02438896	7	3	0.84395715	0.51676182
chr6	127663255	128036580	373326	7	2	1	0.00493743	0.02438896	7	3	1.16581773	0.51676182
chr6	128036580	128263597	227018	6	3	1	0.01598258	0.02438896	7	3	0.84395715	0.51676182
chr6	128263597	128263656	60	1	3	1	0.0079614	0.02438896	8	3	1.07548421	0.51676182
chr6	128263656	128345254	81599	2	3	1	0.01598258	0.02438896	7	3	0.84395715	0.51676182
chr6	128345254	128450892	105639	3	3	1	0.0079614	0.02438896	8	3	1.07548421	0.51676182
chr6	128450892	128505706	54815	1	1	1	8.47E-04	0.02438896	7	3	1.62048027	0.51676182
chr6	128505706	128683120	177415	4	1	1	0.00204627	0.02438896	6	3	1.31360226	0.51676182
chr6	128683120	128774748	91629	2	1	1	0.00478973	0.02438896	5	3	1.02643191	0.51676182
chr6	128774748	128899143	124396	3	2	1	0.00493743	0.02438896	7	3	1.16581773	0.51676182
chr6	128899143	128965749	66607	2	3	1	0.01598258	0.02438896	7	3	0.84395715	0.51676182
chr6	128965749	129052149	86401	1	2	1	0.00493743	0.02438896	7	3	1.16581773	0.51676182
chr6	129052149	129331414	279266	5	2	2	0.00221948	0.08289318	8	3	1.44210395	0.30102999
chr6	129331414	129474072	142659	4	3	2	0.00378107	0.08289318	9	3	1.33111237	0.30102999
chr6	129474072	129573328	99257	2	3	2	0.0079614	0.08289318	8	3	1.07548421	0.30102999
chr6	129573328	129646590	73263	1	3	2	0.01598258	0.08289318	7	3	0.84395715	0.30102999
chr6	129646590	129706728	60139	1	3	1	0.01598258	0.02438896	7	3	0.84395715	0.51676182
chr6	129706728	129771998	65271	2	3	1	0.00170589	0.02438896	10	3	1.61091002	0.51676182
chr6	129771998	129823921	51924	1	3	1	0.00170589	0.05404976	10	2	1.61091002	0.30102999
chr6	129823921	129899080	75160	2	3	1	0.00378107	0.05404976	9	2	1.33111237	0.30102999
chr6	129899080	130031098	132019	3	3	1	0.0079614	0.05404976	8	2	1.07548421	0.30102999
chr6	130031098	130031325	228	2	3	1	0.0079614	0.02438896	8	3	1.07548421	0.51676182
chr6	130031325	130133930	102606	2	3	1	0.01598258	0.02438896	7	3	0.84395715	0.51676182
chr6	130133930	130133989	60	1	3	1	0.0079614	0.02438896	8	3	1.07548421	0.51676182
chr6	130133989	130250885	116897	2	3	1	0.01598258	0.05404976	7	2	0.84395715	0.30102999
chr6	130250885	130250944	60	1	3	2	0.0079614	0.08289318	8	3	1.07548421	0.30102999
chr6	130250944	130432126	181183	2	3	2	0.01598258	0.08289318	7	3	0.84395715	0.30102999
chr6	130432126	130505715	73590	2	3	1	0.03070643	0.05404976	6	2	0.63695542	0.30102999
chr6	130505715	130505772	58	1	3	2	0.03070643	0.08289318	6	3	0.63695542	0.30102999
chr6	130505772	130609194	103423	3	3	1	0.03070643	0.02438896	6	3	0.63695542	0.51676182
chr6	130609194	130658059	48866	3	3	1	0.01598258	0.02438896	7	3	0.84395715	0.51676182
chr6	130658059	130658118	60	1	3	1	0.0079614	0.01091641	8	4	1.07548421	0.76005302
chr6	130658118	130687834	29717	1	3	1	0.01598258	0.01091641	7	4	0.84395715	0.76005302
chr6	130687834	130706288	18455	1	3	1	0.01598258	0.05404976	7	2	0.84395715	0.30102999
chr6	130706288	130763034	56747	3	3	1	0.0079614	0.05404976	8	2	1.07548421	0.30102999
chr6	130763034	130916564	153531	2	3	1	0.01598258	0.05404976	7	2	0.84395715	0.30102999
chr6	130916564	130916623	60	1	3	2	0.01598258	0.1575501	7	2	0.84395715	0.1575501
chr6	130916623	131046165	129543	1	2	1	0.00493743	0.05404976	7	2	1.16581773	0.30102999
chr6	131046165	131156645	110481	2	2	1	9.54E-04	0.02438896	9	3	1.74076927	0.51676182
chr6	131156645	131160901	4257	2	3	2	0.00378107	0.08289318	9	3	1.33111237	0.30102999
chr6	131160901	131247722	86822	3	2	2	9.54E-04	0.08289318	9	3	1.74076927	0.30102999
chr6	131247722	131247781	60	1	3	2	0.00378107	0.08289318	9	3	1.33111237	0.30102999
chr6	131247781	131525694	277914	5	2	2	9.54E-04	0.08289318	9	3	1.74076927	0.30102999
chr6	131525694	131525753	60	1	3	2	0.00378107	0.08289318	9	3	1.33111237	0.30102999
chr6	131525753	131604489	78737	2	2	2	0.00221948	0.08289318	8	3	1.44210395	0.30102999
chr6	131604489	131785777	181289	2	2	2	0.02162467	0.08289318	5	3	0.68214471	0.30102999
chr6	131785777	131785836	60	1	2	2	0.01053319	0.08289318	6	3	0.91219088	0.30102999
chr6	131785836	131895827	109992	1	2	2	0.02162467	0.08289318	5	3	0.68214471	0.30102999
chr6	131895827	131940995	45169	4	2	2	0.02162467	0.0429175	5	4	0.68214471	0.47744371
chr6	131940995	131995331	54337	3	2	2	0.00493743	0.0429175	7	4	1.16581773	0.47744371
chr6	131995331	132270859	275529	9	2	2	0.01053319	0.0429175	6	4	0.91219088	0.47744371
chr6	132270859	132388801	117943	1	2	2	0.01053319	0.08289318	6	3	0.91219088	0.30102999
chr6	132388801	132388860	60	1	2	2	0.00493743	0.08289318	7	3	1.16581773	0.30102999
chr6	132388860	132470533	81674	1	2	2	0.01053319	0.08289318	6	3	0.91219088	0.30102999
chr6	132470533	132633795	163263	2	2	2	0.01053319	0.1575501	6	2	0.91219088	0.1575501
chr6	132633795	132637184	3390	2	3	2	0.03070643	0.08289318	6	3	0.63695542	0.30102999
chr6	132637184	132714172	76989	1	3	2	0.03070643	0.1575501	6	2	0.63695542	0.1575501
chr6	132714172	132814183	100012	3	3	2	0.03070643	0.08289318	6	3	0.63695542	0.30102999
chr6	132814183	132939023	124841	6	3	2	0.03070643	0.0429175	6	4	0.63695542	0.47744371
chr6	132939023	133013571	74549	2	3	2	0.03070643	0.1575501	6	2	0.63695542	0.1575501
chr6	133013571	133081900	68330	1	2	2	0.01053319	0.1575501	6	2	0.91219088	0.1575501
chr6	133081900	133108227	26328	2	2	2	0.01053319	0.08289318	6	3	0.91219088	0.30102999
chr6	133108227	133138620	30394	1	2	2	0.02162467	0.08289318	5	3	0.68214471	0.30102999
chr6	133138620	133179434	40815	1	2	2	0.02162467	0.1575501	5	2	0.68214471	0.1575501
chr6	133179434	133301132	121699	2	2	2	0.01053319	0.1575501	6	2	0.91219088	0.1575501
chr6	133301132	133301191	60	1	2	2	0.01053319	0.08289318	6	3	0.91219088	0.30102999
chr6	133301191	133769222	468032	6	2	2	0.01053319	0.1575501	6	2	0.91219088	0.1575501
chr6	133769222	133769280	59	1	2	2	0.01053319	0.08289318	6	3	0.91219088	0.30102999
chr6	133769280	133850590	81311	1	2	2	0.01053319	0.1575501	6	2	0.91219088	0.1575501
chr6	133850590	133850649	60	1	3	2	0.03070643	0.1575501	6	2	0.63695542	0.1575501
chr6	133850649	133885517	34869	1	2	2	0.01053319	0.1575501	6	2	0.91219088	0.1575501
chr6	133885517	134062091	176575	3	2	2	0.01053319	0.08289318	6	3	0.91219088	0.30102999
chr6	134062091	134142379	80289	1	2	1	0.01053319	0.05404976	6	2	0.91219088	0.30102999
chr6	134142379	134213163	70785	2	3	1	0.03070643	0.05404976	6	2	0.63695542	0.30102999
chr6	134213163	134284021	70859	3	3	1	0.03070643	0.02438896	6	3	0.63695542	0.51676182
chr6	134284021	134310589	26569	3	3	2	0.03070643	0.08289318	6	3	0.63695542	0.30102999
chr6	134310589	134364589	54001	2	3	1	0.03070643	0.02438896	6	3	0.63695542	0.51676182
chr6	134364589	134388004	23416	2	3	2	0.03070643	0.08289318	6	3	0.63695542	0.30102999
chr6	134388004	134600903	212900	4	3	1	0.03070643	0.02438896	6	3	0.63695542	0.51676182
chr6	134600903	134600962	60	1	3	2	0.03070643	0.0429175	6	4	0.63695542	0.47744371
chr6	134600962	135185886	584925	8	3	2	0.03070643	0.08289318	6	3	0.63695542	0.30102999
chr6	135185886	135185945	60	1	3	3	0.03070643	0.17593012	6	3	0.63695542	0.17593012
chr6	135185945	135303692	117748	4	3	2	0.03070643	0.08289318	6	3	0.63695542	0.30102999
chr6	135303692	135303751	60	1	3	2	0.01598258	0.08289318	7	3	0.84395715	0.30102999
chr6	135303751	135353872	50122	2	2	2	0.0429175	0.08289318	4	3	0.47744371	0.30102999
chr6	135353872	135429926	76055	3	2	2	0.02162467	0.08289318	5	3	0.68214471	0.30102999
chr6	135429926	135429985	60	1	2	3	0.02162467	0.17593012	5	3	0.68214471	0.17593012
chr6	135429985	135565749	135765	3	2	2	0.0429175	0.08289318	4	3	0.47744371	0.30102999
chr6	135565749	135565808	60	1	2	2	0.02162467	0.08289318	5	3	0.68214471	0.30102999
chr6	135565808	135618240	52433	2	2	2	0.0429175	0.08289318	4	3	0.47744371	0.30102999
chr6	135618240	135679346	61107	3	2	2	0.02162467	0.08289318	5	3	0.68214471	0.30102999
chr6	135679346	135787352	108007	2	2	2	0.0429175	0.08289318	4	3	0.47744371	0.30102999
chr6	135787352	135843857	56506	3	2	2	0.02162467	0.08289318	5	3	0.68214471	0.30102999
chr6	135843857	135945295	101439	2	2	2	0.01053319	0.08289318	6	3	0.91219088	0.30102999
chr6	135945295	135996327	51033	2	2	2	0.00493743	0.08289318	7	3	1.16581773	0.30102999
chr6	135996327	136142531	146205	1	2	2	0.01053319	0.08289318	6	3	0.91219088	0.30102999
chr6	136142531	136268689	126159	4	2	2	0.00493743	0.08289318	7	3	1.16581773	0.30102999
chr6	136268689	136317924	49236	1	2	2	0.01053319	0.08289318	6	3	0.91219088	0.30102999
chr6	136317924	136317983	60	1	2	2	0.00493743	0.08289318	7	3	1.16581773	0.30102999
chr6	136317983	136372355	54373	1	2	2	0.02162467	0.08289318	5	3	0.68214471	0.30102999
chr6	136372355	136513883	141529	4	2	3	0.02162467	0.17593012	5	3	0.68214471	0.17593012
chr6	136513883	136537669	23787	2	2	3	0.00493743	0.17593012	7	3	1.16581773	0.17593012
chr6	136537669	136582393	44725	2	2	3	0.01053319	0.17593012	6	3	0.91219088	0.17593012
chr6	136582393	136704809	122417	2	2	3	0.0429175	0.17593012	4	3	0.47744371	0.17593012

chr6	136704809	136704864	56	1	2	3	0.02162467	0.17593012	5	3	0.68214471	0.17593012
chr6	136704864	137019615	314752	8	2	3	0.0429175	0.17593012	4	3	0.47744371	0.17593012
chr6	137019615	137070086	50472	2	2	3	0.02162467	0.17593012	5	3	0.68214471	0.17593012
chr6	137070086	137147457	77372	2	2	3	0.02162467	0.30102999	5	2	0.68214471	0.08289318
chr6	137147457	137147511	55	1	2	3	0.01053319	0.30102999	6	2	0.91219088	0.08289318
chr6	137147511	137175714	28204	1	2	3	0.02162467	0.30102999	5	2	0.68214471	0.08289318
chr6	137175714	137219255	43542	1	2	3	0.0429175	0.30102999	4	2	0.47744371	0.08289318
chr6	137219255	137219314	60	1	2	3	0.0429175	0.17593012	4	3	0.47744371	0.17593012
chr6	137219314	137322108	102795	2	2	3	0.0429175	0.30102999	4	2	0.47744371	0.08289318
chr6	137322108	137469001	146894	3	2	3	0.0429175	0.17593012	4	3	0.47744371	0.17593012
chr6	137469001	137620298	151298	4	2	2	0.0429175	0.08289318	4	3	0.47744371	0.30102999
chr6	137620298	137620357	60	1	2	2	0.02162467	0.08289318	5	3	0.68214471	0.30102999
chr6	137620357	137813478	193122	2	2	2	0.0429175	0.08289318	4	3	0.47744371	0.30102999
chr6	137813478	138095257	281780	3	2	2	0.0429175	0.1575501	4	2	0.47744371	0.1575501
chr6	138095257	138195072	99816	1	1	2	0.01091641	0.1575501	4	2	0.76005302	0.1575501
chr6	138195072	138203754	8683	2	1	2	0.00478973	0.1575501	5	2	1.02643191	0.1575501
chr6	138203754	138295199	91446	1	1	1	0.01091641	0.05404976	4	2	0.76005302	0.30102999
chr6	138295199	138419878	124680	3	1	2	0.01091641	0.1575501	4	2	0.76005302	0.1575501
chr6	138419878	138434120	14243	2	1	2	0.00478973	0.1575501	5	2	1.02643191	0.1575501
chr6	138434120	138502451	68332	1	1	2	0.01091641	0.1575501	4	2	0.76005302	0.1575501
chr6	138502451	138537500	35050	2	1	3	0.01091641	0.30102999	4	2	0.76005302	0.08289318
chr6	138537500	138816531	279032	6	1	2	0.01091641	0.1575501	4	2	0.76005302	0.1575501
chr6	138816531	139066203	249673	5	1	2	0.00478973	0.1575501	5	2	1.02643191	0.1575501
chr6	139066203	139066262	60	1	1	3	0.00478973	0.30102999	5	2	1.02643191	0.08289318
chr6	139066262	139121816	55555	2	1	3	0.01091641	0.30102999	4	2	0.76005302	0.08289318
chr6	139121816	139183708	61893	1	1	3	0.02438896	0.30102999	3	2	0.51676182	0.08289318
chr6	139183708	139183767	60	1	1	4	0.02438896	0.47744371	3	2	0.51676182	0.0429175
chr6	139183767	139262380	78614	2	1	3	0.05404976	0.30102999	2	2	0.30102999	0.08289318
chr6	139262380	139285267	22888	2	1	3	0.02438896	0.30102999	3	2	0.51676182	0.08289318
chr6	139285267	139437634	152368	3	1	3	0.05404976	0.30102999	2	2	0.30102999	0.08289318
chr6	139437634	139693619	255986	7	1	4	0.05404976	0.47744371	2	2	0.30102999	0.0429175
chr6	139693619	139800754	107136	1	1	4	0.05404976	0.76005302	2	1	0.30102999	0.01091641
chr6	139800754	140335592	534839	5	0	4	0	0.76005302	2	1	0.61140001	0.01091641
chr6	140335592	140335651	60	1	1	4	0.05404976	0.76005302	2	1	0.30102999	0.01091641
chr6	140335651	140669555	333905	2	0	0	0	0	2	0	0.61140001	0
chr6	140669555	141166056	496502	5	0	0	0	0	3	0	0.93173516	0
chr6	141166056	141528317	362262	3	0	0	0	0	2	0	0.61140001	0
chr6	141528317	141757845	229529	2	0	1	0	0.05404976	3	2	0.93173516	0.30102999
chr6	141757845	141894754	136910	2	0	2	0	0.08289318	3	3	0.93173516	0.30102999
chr6	141894754	141970442	75689	2	0	2	0	0.08289318	4	3	1.26272838	0.30102999
chr6	141970442	142143478	173037	3	1	2	0.01091641	0.08289318	4	3	0.76005302	0.30102999
chr6	142143478	142143537	60	1	2	2	0.0429175	0.08289318	4	3	0.47744371	0.30102999
chr6	142143537	142200482	56946	1	1	2	0.01091641	0.08289318	4	3	0.76005302	0.30102999
chr6	142200482	142302646	102165	1	1	1	0.01091641	0.02438896	4	3	0.76005302	0.51676182
chr6	142302646	142401965	99320	1	0	1	0	0.02438896	4	3	1.26272838	0.51676182
chr6	142401965	142490715	88751	4	0	2	0	0.08289318	4	3	1.26272838	0.30102999
chr6	142490715	142804538	313824	8	1	2	0.01091641	0.08289318	4	3	0.76005302	0.30102999
chr6	142804538	142880252	75715	2	1	2	0.00478973	0.08289318	5	3	1.02643191	0.30102999
chr6	142880252	143658780	778529	14	1	2	0.00204627	0.08289318	6	3	1.31360226	0.30102999
chr6	143658780	143695792	37013	1	1	2	0.00204627	0.1575501	6	2	1.31360226	0.1575501
chr6	143695792	143921803	226012	5	1	2	0.00478973	0.1575501	5	2	1.02643191	0.1575501
chr6	143921803	143921862	60	1	2	2	0.02162467	0.1575501	5	2	0.68214471	0.1575501
chr6	143921862	144145419	223558	5	1	2	0.00478973	0.1575501	5	2	1.02643191	0.1575501
chr6	144145419	144183388	37970	2	2	2	0.02162467	0.1575501	5	2	0.68214471	0.1575501
chr6	144183388	144223245	39858	1	2	2	0.0429175	0.1575501	4	2	0.47744371	0.1575501
chr6	144223245	144315564	92320	3	2	2	0.02162467	0.08289318	5	3	0.68214471	0.30102999
chr6	144315564	144328897	13334	2	2	2	0.01053319	0.08289318	6	3	0.91219088	0.30102999
chr6	144328897	144499914	171018	5	1	2	0.01091641	0.08289318	4	3	0.76005302	0.30102999
chr6	144499914	144499973	60	1	1	3	0.01091641	0.17593012	4	3	0.76005302	0.17593012
chr6	144499973	144692464	192492	3	1	2	0.01091641	0.08289318	4	3	0.76005302	0.30102999
chr6	144692464	144692523	60	1	2	3	0.0429175	0.17593012	4	3	0.47744371	0.17593012
chr6	144692523	144945977	253455	5	0	3	0	0.17593012	4	3	1.26272838	0.17593012
chr6	144945977	144946036	60	1	0	4	0	0.30102999	4	3	1.26272838	0.10122019
chr6	144946036	145057662	111627	2	0	3	0	0.17593012	4	3	1.26272838	0.17593012
chr6	145057662	145057721	60	1	1	4	0.01091641	0.30102999	4	3	0.76005302	0.10122019
chr6	145057721	145105155	47435	1	1	3	0.01091641	0.17593012	4	3	0.76005302	0.17593012
chr6	145105155	145283553	178399	2	1	2	0.01091641	0.08289318	4	3	0.76005302	0.30102999
chr6	145283553	145283612	60	1	2	2	0.0429175	0.08289318	4	3	0.47744371	0.30102999
chr6	145283612	145473927	190316	1	2	1	0.0429175	0.05404976	4	2	0.47744371	0.30102999
chr6	145473927	145664481	190555	1	2	0	0.1575501	0	2	2	0.1575501	0.61140001
chr6	145664481	145734378	69898	1	1	0	0.05404976	0	2	2	0.30102999	0.61140001
chr6	145734378	145734437	60	1	1	2	0.02438896	0.0429175	3	4	0.51676182	0.47744371
chr6	145734437	145982152	247716	3	1	2	0.05404976	0.08289318	2	3	0.30102999	0.30102999
chr6	145982152	146042379	60228	1	0	2	0	0.1575501	2	2	0.61140001	0.1575501
chr6	146042379	146055872	13494	2	0	3	0	0.17593012	2	3	0.61140001	0.17593012
chr6	146055872	146132701	76830	1	0	2	0	0.1575501	2	2	0.61140001	0.1575501
chr6	146132701	146132760	60	1	1	2	0.05404976	0.1575501	2	2	0.30102999	0.1575501
chr6	146132760	146179896	47137	1	1	1	0.05404976	0.05404976	2	2	0.30102999	0.30102999
chr6	146179896	146231330	51435	3	1	2	0.05404976	0.1575501	2	2	0.30102999	0.1575501
chr6	146231330	146313209	81880	3	0	2	0	0.1575501	2	2	0.61140001	0.1575501
chr6	146313209	146351002	37794	2	0	3	0	0.30102999	2	2	0.61140001	0.08289318
chr6	146351002	146434860	83859	3	0	3	0	0.17593012	3	3	0.93173516	0.17593012
chr6	146434860	146494044	59185	1	0	3	0	0.17593012	2	3	0.61140001	0.17593012
chr6	146494044	146589430	95387	3	1	4	0.05404976	0.30102999	2	3	0.30102999	0.10122019
chr6	146589430	146673396	83967	3	2	4	0.1575501	0.30102999	2	3	0.1575501	0.10122019
chr6	146673396	146703664	30269	2	2	5	0.1575501	0.45545077	2	3	0.1575501	0.05670724
chr6	146703664	146758280	54617	1	2	4	0.1575501	0.30102999	2	3	0.1575501	0.10122019
chr6	146758280	146875909	117630	1	1	4	0.05404976	0.30102999	2	3	0.30102999	0.10122019
chr6	146875909	146934166	58258	2	1	5	0.05404976	0.45545077	2	3	0.30102999	0.05670724
chr6	146934166	146988824	54659	1	1	3	0.05404976	0.17593012	2	3	0.30102999	0.17593012
chr6	146988824	146988883	60	1	2	3	0.1575501	0.17593012	2	3	0.1575501	0.17593012
chr6	146988883	147045031	56149	1	1	3	0.05404976	0.17593012	2	3	0.30102999	0.17593012
chr6	147045031	147045090	60	1	1	3	0.02438896	0.17593012	3	3	0.51676182	0.17593012
chr6	147045090	147085277	40188	1	1	2	0.02438896	0.08289318	3	3	0.51676182	0.30102999
chr6	147085277	147163745	78469	2	1	2	0.02438896	0.0429175	3	4	0.51676182	0.47744371
chr6	147163745	147293829	130085	2	0	2	0	0.0429175	1	4	0.30102999	0.47744371
chr6	147293829	147588250	294422	4	0	1	0	0.02438896	1	3	0.30102999	0.51676182
chr6	147588250	147588309	60	1	0	3	0	0.10122019	1	4	0.30102999	0.30102999
chr6	147588309	147660528	72220	3	0	1	0	0.02438896	1	3	0.30102999	0.51676182
chr6	147660528	147825506	164979	2	0	1	0	0.05404976	1	2	0.30102999	0.30102999
chr6	147825506	147868215	42710	2	0	2	0	0.08289318	2	3	0.61140001	0.30102999
chr6	147868215	147868274	60	1	1	2	0.05404976	0.08289318	2	3	0.30102999	0.30102999
chr6	147868274	148056029	187756	2	0	2	0	0.30102999	2	1	0.61140001	0.05404976
chr6	148056029	148056088	60	1	1	2	0.05404976	0.1575501	2	2	0.30102999	0.1575501
chr6	148056088	148220934	164847	1	0	2	0	0.1575501	2	2	0.61140001	0.1575501
chr6	148220934	148456242	235309	1	0	2	0	0.30102999	2	1	0.61140001	0.05404976
chr6	148456242	148589994	133753	2	1	2	0.05404976	0.30102999	2	1	0.30102999	0.05404976
chr6	148589994	148701499	111506	3	2	2	0.1575501	0.30102999	2	1	0.1575501	0.05404976
chr6	148701499	148701558	60	1	2	2	0.08289318	0.30102999	3	1	0.30102999	0.05404976
chr6	148701558	148937387	235830	7	1	2	0.05404976	0.30102999	2	1	0.30102999	0.05404976
chr6	148937387	148937446	60	1	2	2	0.08289318	0.30102999	3	1	0.30102999	0.05404976
chr6	148937446	149172520	235075	4	1	2	0.02438896	0.30102999	3	1	0.51676182	0.05404976
chr6	149172520	149236628	64109	2	1	2	0.00478973	0.30102999	5	1	1.02643191	0.05404976
chr6	149236628	149262591	25964	2	2	2	0.02162467	0.30102999	5	1	0.68214471	0.05404976
chr6	149262591	149397602	135012	2	2	2	0.0429175	0.30102999	4	1	0.47744371	0.05404976
chr6	149397602	149397661	60	1	2	2	0.02162467	0.1575501	5	2	0.68214471	0.1575501
chr6	149397661	149564095	166435	2	2	1	0.0429175	0.05404976	4	2	0.47744371	0.30102999
chr6	149564095	149564154	60	1	2	2	0.0429175	0.1575501	4	2	0.47744371	0.1575501
chr6	149564154	149690745	126592	2	2	2	0.08289318	0.1575501	3	2	0.30102999	0.1575501
chr6	149690745	149718897	28153	3	2	2	0.08289318	0.08289318	3	3	0.30102999	0.30102999
chr6	149718897	149777551	58655	1	2	2	0.08289318	0.1575501	3	2	0.30102999	0.1575501
chr6	149777551	149855785	78235	1	2	1	0.08289318	0.05404976	3	2	0.30102999	0.30102999
chr6	149855785	149855844	60	1	2	1	0.0429175	0.05404976	4	2	0.47744371	0.30102999
chr6	149855844	149946190	90347	2	1	1	0.01091641	0.05404976	4	2	0.76005302	0.30102999

chr6	149946190	150132442	186253	6	1	1	0.01091641	0.02438896	4	3	0.76005302	0.51676182
chr6	150132442	150209800	77359	3	1	1	0.01091641	0.05404976	4	2	0.76005302	0.30102999
chr6	150209800	150353992	144193	3	1	1	0.02438896	0.05404976	3	2	0.51676182	0.30102999
chr6	150353992	150476449	122458	4	1	1	0.01091641	0.05404976	4	2	0.76005302	0.30102999
chr6	150476449	150642806	166358	4	1	1	0.01091641	0.1218695	4	1	0.76005302	0.1218695
chr6	150642806	150642865	60	1	1	1	0.00478973	0.1218695	5	1	1.02643191	0.1218695
chr6	150642865	150702522	59658	1	1	1	0.01091641	0.1218695	4	1	0.76005302	0.1218695
chr6	150702522	150981103	278582	5	2	1	0.0429175	0.1218695	4	1	0.47744371	0.1218695
chr6	150981103	151116095	134993	2	1	1	0.01091641	0.1218695	4	1	0.76005302	0.1218695
chr6	151116095	151630579	514485	11	1	1	0.01091641	0.05404976	4	2	0.76005302	0.30102999
chr6	151630579	151734887	104309	3	0	1	0	0.05404976	4	2	1.26272838	0.30102999
chr6	151734887	151734946	60	1	1	1	0.01091641	0.05404976	4	2	0.76005302	0.30102999
chr6	151734946	151907174	172229	4	0	1	0	0.05404976	4	2	1.26272838	0.30102999
chr6	151907174	151907232	59	1	1	1	0.01091641	0.05404976	4	2	0.76005302	0.30102999
chr6	151907232	152159730	252499	5	0	1	0	0.05404976	4	2	1.26272838	0.30102999
chr6	152159730	152298586	138857	3	0	1	0	0.05404976	3	2	0.93173516	0.30102999
chr6	152298586	152325493	26908	2	0	1	0	0.02438896	3	3	0.93173516	0.51676182
chr6	152325493	152367078	41586	2	0	1	0	0.01091641	5	4	1.60515106	0.76005302
chr6	152367078	152456275	89198	3	0	1	0	0.00478973	5	5	1.60515106	1.02643191
chr6	152456275	152456334	60	1	0	2	0	0.02162467	5	5	1.60515106	0.68214471
chr6	152456334	152590823	134490	2	0	2	0	0.02162467	3	5	0.93173516	0.68214471
chr6	152590823	152826726	235904	5	0	2	0	0.02162467	2	5	0.61140001	0.68214471
chr6	152826726	153298731	472006	9	0	2	0	0.02162467	1	5	0.30102999	0.68214471
chr6	153298731	153311349	12619	3	1	3	0.1218695	0.05670724	1	5	0.1218695	0.45545077
chr6	153311349	153417094	105746	5	0	3	0	0.05670724	1	5	0.30102999	0.45545077
chr6	153417094	153543129	126036	3	0	3	0	0.03070643	1	6	0.30102999	0.63695542
chr6	153543129	153543188	60	1	0	4	0	0.06713722	1	6	0.30102999	0.44141547
chr6	153543188	153650250	107063	1	0	2	0	0.01053319	1	6	0.30102999	0.91219088
chr6	153650250	153650309	60	1	0	3	0	0.03070643	1	6	0.30102999	0.63695542
chr6	153650309	153938070	287762	2	0	2	0	0.01053319	1	6	0.30102999	0.91219088
chr6	153938070	153981410	43341	2	0	4	0	0.06713722	1	6	0.30102999	0.44141547
chr6	153981410	154122864	141455	1	0	2	0	0.01053319	1	6	0.30102999	0.91219088
chr6	154122864	154295758	172895	3	0	2	0	0.01053319	2	6	0.61140001	0.91219088
chr6	154295758	154412610	116853	2	0	1	0	0.01091641	1	4	0.30102999	0.76005302
chr6	154412610	154414433	1824	2	0	2	0	0.0429175	1	4	0.30102999	0.47744371
chr6	154414433	154491524	77092	2	0	1	0	0.01091641	1	4	0.30102999	0.76005302
chr6	154491524	154535443	43920	2	1	2	0.1218695	0.0429175	1	4	0.1218695	0.47744371
chr6	154535443	154567925	32483	1	0	1	0	0.01091641	1	4	0.30102999	0.76005302
chr6	154567925	154705203	137279	3	0	1	0	0.00478973	2	5	0.61140001	1.02643191
chr6	154705203	154751749	46547	3	0	2	0	0.00493743	2	7	0.61140001	1.16581773
chr6	154751749	154827208	75460	2	0	1	0	8.47E-04	2	7	0.61140001	1.62048027
chr6	154827208	155059401	232194	3	0	1	0	0.00204627	2	6	0.61140001	1.31360226
chr6	155059401	155126517	67117	3	0	1	0	8.47E-04	2	7	0.61140001	1.62048027
chr6	155126517	155392484	265968	4	0	1	0	8.47E-04	3	7	0.93173516	1.62048027
chr6	155392484	155392543	60	1	0	2	0	0.00493743	3	7	0.93173516	1.16581773
chr6	155392543	155470535	77993	2	0	1	0	0.00478973	3	5	0.93173516	1.02643191
chr6	155470535	155565936	95402	2	0	1	0	0.01091641	3	4	0.93173516	0.76005302
chr6	155565936	156423608	857673	11	0	1	0	0.01091641	2	4	0.61140001	0.76005302
chr6	156423608	156423667	60	1	0	2	0	0.02162467	2	5	0.61140001	0.68214471
chr6	156423667	156585724	162058	1	0	2	0	0.0429175	2	4	0.61140001	0.47744371
chr6	156585724	156699568	113845	1	0	1	0	0.05404976	2	2	0.61140001	0.30102999
chr6	156699568	156895627	196060	2	0	1	0	0.1218695	2	1	0.61140001	0.1218695
chr6	156895627	156895686	60	1	0	1	0	0.05404976	2	2	0.61140001	0.30102999
chr6	156895686	157120030	224345	2	0	1	0	0.1218695	1	1	0.30102999	0.1218695
chr6	157120030	157120089	60	1	0	1	0	0.05404976	2	2	0.61140001	0.30102999
chr6	157120089	157336025	215937	4	0	1	0	0.05404976	1	2	0.30102999	0.30102999
chr6	157336025	157386210	50186	2	0	1	0	0.02438896	1	3	0.30102999	0.51676182
chr6	157386210	157386269	60	1	0	1	0	0.01091641	1	4	0.30102999	0.76005302
chr6	157386269	157553034	166766	2	0	1	0	0.02438896	1	3	0.30102999	0.51676182
chr6	157553034	158144137	591104	7	0	1	0	0.01091641	1	4	0.30102999	0.76005302
chr6	158144137	158211502	67366	2	1	1	0.1218695	0.01091641	1	4	0.1218695	0.76005302
chr6	158211502	158211561	60	1	1	1	0.1218695	0.00478973	1	5	0.1218695	1.02643191
chr6	158211561	158296076	84516	1	0	1	0	0.01091641	1	4	0.30102999	0.76005302
chr6	158296076	158406859	110784	4	0	2	0	0.0429175	1	4	0.30102999	0.47744371
chr6	158406859	158441992	35134	2	1	2	0.1218695	0.02162467	1	5	0.1218695	0.68214471
chr6	158441992	158680478	238487	7	0	2	0	0.02162467	1	5	0.30102999	0.68214471
chr6	158680478	158734787	54310	2	0	3	0	0.05670724	1	5	0.30102999	0.45545077
chr6	158734787	159210371	475585	12	0	3	0	0.01598258	1	7	0.30102999	0.84395715
chr6	159210371	159266498	56128	3	0	4	0	0.03812622	1	7	0.30102999	0.60763643
chr6	159266498	159354145	87648	3	0	4	0	0.02074938	2	8	0.61140001	0.79906872
chr6	159354145	159398227	44083	2	0	4	0	0.01077081	2	9	0.61140001	1.01542894
chr6	159398227	159398527	301	2	0	5	0	0.02473314	2	9	0.61140001	0.76806864
chr6	159398527	159456484	57958	1	0	5	0	0.04407651	2	8	0.61140001	0.58747015
chr6	159456484	159615687	159204	2	0	5	0	0.07511598	2	7	0.61140001	0.43181735
chr6	159615687	159615746	60	1	0	5	0	0.04407651	2	8	0.61140001	0.58747015
chr6	159615746	159671491	55746	2	0	5	0	0.07511598	2	7	0.61140001	0.43181735
chr6	159671491	159671548	58	1	0	5	0	0.04407651	2	8	0.61140001	0.58747015
chr6	159671548	159820916	149369	1	0	5	0	0.07511598	2	7	0.61140001	0.43181735
chr6	159820916	159820975	60	1	0	5	0	0.04407651	2	8	0.61140001	0.58747015
chr6	159820975	159921850	100876	1	0	5	0	0.07511598	2	7	0.61140001	0.43181735
chr6	159921850	159921909	60	1	0	5	0	0.04407651	3	8	0.93173516	0.58747015
chr6	159921909	160021134	99226	1	0	4	0	0.03812622	2	7	0.61140001	0.60763643
chr6	160021134	160147521	126388	4	0	4	0	0.02074938	2	8	0.61140001	0.79906872
chr6	160147521	160329038	181518	11	1	4	0.05404976	0.02074938	2	8	0.30102999	0.79906872
chr6	160329038	160485814	156777	3	0	4	0	0.02074938	2	8	0.61140001	0.79906872
chr6	160485814	160707401	221588	7	0	5	0	0.04407651	2	8	0.61140001	0.58747015
chr6	160707401	161007816	300416	9	0	5	0	0.02473314	2	9	0.61140001	0.76806864
chr6	161007816	161159595	151780	3	0	5	0	0.04407651	2	8	0.61140001	0.58747015
chr6	161159595	161413772	254178	4	0	5	0	0.07511598	2	7	0.61140001	0.43181735
chr6	161413772	161470451	56680	3	0	5	0	0.04407651	2	8	0.61140001	0.58747015
chr6	161470451	161552266	81816	2	0	5	0	0.07511598	2	7	0.61140001	0.43181735
chr6	161552266	161687022	134757	4	0	5	0	0.04407651	3	8	0.93173516	0.58747015
chr6	161687022	161769668	82647	2	0	5	0	0.07511598	2	7	0.61140001	0.43181735
chr6	161769668	161769727	60	1	0	5	0	0.02473314	3	9	0.93173516	0.76806864
chr6	161769727	161812380	42654	1	0	5	0	0.02473314	2	9	0.61140001	0.76806864
chr6	161812380	161951125	138746	2	0	5	0	0.04407651	1	8	0.30102999	0.58747015
chr6	161951125	162009551	58427	1	0	4	0	0.02074938	1	8	0.30102999	0.79906872
chr6	162009551	162009610	60	1	0	4	0	0.02074938	2	8	0.61140001	0.79906872
chr6	162009610	162349416	339807	7	0	4	0	0.03812622	1	7	0.30102999	0.60763643
chr6	162349416	162394395	44980	2	0	4	0	0.03812622	2	7	0.61140001	0.60763643
chr6	162394395	162465962	71568	2	0	4	0	0.02074938	2	8	0.61140001	0.79906872
chr6	162465962	162466021	60	1	0	4	0	0.02074938	3	8	0.93173516	0.79906872
chr6	162466021	162541411	75391	1	0	4	0	0.03812622	2	7	0.61140001	0.60763643
chr6	162541411	162609575	68165	1	0	4	0	0.03812622	1	7	0.30102999	0.60763643
chr6	162609575	162609634	60	1	0	4	0	0.03812622	2	7	0.61140001	0.60763643
chr6	162609634	162683727	74094	1	0	4	0	0.03812622	1	7	0.30102999	0.60763643
chr6	162683727	162683777	51	1	0	4	0	0.02074938	1	8	0.30102999	0.79906872
chr6	162683777	162748288	64512	1	0	4	0	0.03812622	1	7	0.30102999	0.60763643
chr6	162748288	162748347	60	1	0	4	0	0.03812622	2	7	0.61140001	0.60763643
chr6	162748347	162976542	228196	3	0	4	0	0.03812622	1	7	0.30102999	0.60763643
chr6	162976542	162976601	60	1	0	4	0	0.03812622	2	7	0.61140001	0.60763643
chr6	162976601	163050344	73744	1	0	4	0	0.03812622	1	7	0.30102999	0.60763643
chr6	163050344	163310837	260494	6	0	4	0	0.02074938	1	8	0.30102999	0.79906872
chr6	163310837	163483254	172418	3	0	4	0	0.03812622	1	7	0.30102999	0.60763643
chr6	163483254	163731800	248547	4	0	4	0	0.06713722	1	6	0.30102999	0.44141547
chr6	163731800	164159702	427903	8	0	3	0	0.03070643	1	6	0.30102999	0.63695542
chr6	164159702	164325361	165660	1	0	3	0	0.05670724	1	5	0.30102999	0.45545077
chr6	164325361	164954424	629064	4	0	2	0	0.02162467	1	5	0.30102999	0.68214471
chr6	164954424	165203779	249356	2	0	3	0	0.03070643	1	6	0.30102999	0.63695542
chr6	165203779	165443883	240105	2	0	4	0	0.06713722	1	6	0.30102999	0.44141547
chr6	165443883	165667663	223781	2	0	4	0	0.18734596	1	4	0.30102999	0.18734596
chr6	165667663	165745016	77354	3	0	5	0	0.12309572	1	6	0.30102999	0.30102999
chr6	165745016	165779301	34286	1	0	4	0	0.06713722	1	6	0.30102999	0.44141547
chr6	165779301	165886755	107455	2	0	3	0	0.05670724	1	5	0.30102999	0.45545077

chr6	165886755	165988219	101465	4	0	3	0	0.03070643	1	6	0.30102999	0.63695542
chr6	165988219	165988278	60	1	0	4	0	0.02074938	2	8	0.61140001	0.79906872
chr6	165988278	166071046	82769	2	0	4	0	0.03812622	2	7	0.61140001	0.60763643
chr6	166071046	166203130	132085	3	0	5	0	0.04407651	2	8	0.61140001	0.58747015
chr6	166203130	166393240	190111	2	0	5	0	0.07511598	2	7	0.61140001	0.43181735
chr6	166393240	166393299	60	1	0	5	0	0.04407651	2	8	0.61140001	0.58747015
chr6	166393299	166571083	177785	3	0	5	0	0.07511598	2	7	0.61140001	0.43181735
chr6	166571083	166659302	88220	4	0	5	0	0.04407651	2	8	0.61140001	0.58747015
chr6	166659302	166754981	95680	3	0	5	0	0.02473314	2	9	0.61140001	0.76806864
chr6	166754981	166782268	27288	2	0	5	0	0.01320236	2	10	0.61140001	0.97390707
chr6	166782268	166964963	182696	5	0	5	0	0.07511598	2	7	0.61140001	0.43181735
chr6	166964963	166965020	58	1	0	5	0	0.04407651	2	8	0.61140001	0.58747015
chr6	166965020	166998263	33244	1	0	5	0	0.07511598	2	7	0.61140001	0.43181735
chr6	166998263	167052569	54307	2	0	5	0	0.04407651	2	8	0.61140001	0.58747015
chr6	167052569	167194713	142145	3	0	4	0	0.03812622	2	7	0.61140001	0.60763643
chr6	167194713	167424098	229386	6	0	4	0	0.06713722	2	6	0.61140001	0.44141547
chr6	167424098	167453607	29510	3	0	4	0	0.03812622	2	7	0.61140001	0.60763643
chr6	167453607	167543365	89759	2	0	4	0	0.06713722	2	6	0.61140001	0.44141547
chr6	167543365	167770869	227505	7	0	4	0	0.03812622	2	7	0.61140001	0.60763643
chr6	167770869	167770928	60	1	0	5	0	0.07511598	2	7	0.61140001	0.43181735
chr6	167770928	167881373	110446	1	0	4	0	0.06713722	2	6	0.61140001	0.44141547
chr6	167881373	168303033	421661	1	0	3	0	0.05670724	1	5	0.30102999	0.45545077
chr6	168303033	168312764	9732	3	0	4	0	0.06713722	2	6	0.61140001	0.44141547
chr6	168312764	168776873	464110	9	0	4	0	0.06713722	1	6	0.30102999	0.44141547
chr6	168776873	168907643	130771	3	0	4	0	0.03812622	1	7	0.30102999	0.60763643
chr6	168907643	168954929	47287	2	0	5	0	0.07511598	1	7	0.30102999	0.43181735
chr6	168954929	169011336	56408	2	0	7	0	0.20469099	1	7	0.30102999	0.20469099
chr6	169011336	169011395	60	1	0	7	0	0.20469099	2	7	0.61140001	0.20469099
chr6	169011395	169200278	188884	3	0	6	0	0.129913	2	7	0.61140001	0.30102999
chr6	169200278	169261781	61504	2	0	8	0	0.30102999	2	7	0.61140001	0.13499366
chr6	169261781	169506562	244782	3	0	7	0	0.20469099	2	7	0.61140001	0.20469099
chr6	169506562	169591031	84470	1	0	6	0	0.20064824	2	6	0.61140001	0.20064824
chr6	169591031	169653664	62634	1	0	6	0	0.20064824	1	6	0.30102999	0.20064824
chr6	169653664	169655578	1915	2	0	6	0	0.129913	1	7	0.30102999	0.30102999
chr6	169655578	169786414	130837	2	0	6	0	0.20064824	1	6	0.30102999	0.20064824
chr6	169786414	169936233	149820	4	0	6	0	0.129913	1	7	0.30102999	0.30102999
chr6	169936233	170088905	152673	4	0	6	0	0.129913	2	7	0.61140001	0.30102999
chr6	170088905	170088964	60	1	1	6	0.02438896	0.129913	3	7	0.51676182	0.30102999
chr6	170088964	170201866	112903	4	1	5	0.05404976	0.12309572	2	6	0.30102999	0.30102999
chr6	170201866	170228733	26868	2	1	5	0.05404976	0.07511598	2	7	0.30102999	0.43181735
chr6	170228733	170460340	231608	2	0	5	0	0.19510895	1	5	0.30102999	0.19510895
chr6	170460340	170460399	60	1	0	6	0	0.129913	2	7	0.61140001	0.30102999
chr6	170460399	170726796	266398	5	0	5	0	0.19510895	2	5	0.61140001	0.19510895
chr6	170726796	170890108	163313	4	0	4	0	0.11390336	2	5	0.61140001	0.30102999
chr7	92532	159259	66728	3	2	0	0.30102999	0	1	1	0.05404976	0.30102999
chr7	159259	195085	35827	2	3	0	0.51676182	0	1	2	0.02438896	0.61140001
chr7	195085	227824	32740	1	2	0	0.30102999	0	1	1	0.05404976	0.30102999
chr7	227824	558409	330586	1	1	0	0.1218695	0	1	1	0.1218695	0.30102999
chr7	558409	764921	206513	3	4	0	0.76005302	0	1	2	0.01091641	0.61140001
chr7	764921	764980	60	1	4	1	0.47744371	0.05404976	2	2	0.0429175	0.30102999
chr7	764980	926169	161190	4	4	1	0.76005302	0.05404976	1	2	0.01091641	0.30102999
chr7	926169	995544	69376	2	4	2	0.47744371	0.1575501	2	2	0.0429175	0.1575501
chr7	995544	995602	59	1	5	2	0.30102999	0.08289318	4	3	0.11390336	0.30102999
chr7	995602	1070935	75334	3	5	2	0.30102999	0.1575501	4	2	0.11390336	0.1575501
chr7	1070935	1087076	16142	1	5	2	0.45545077	0.1575501	3	2	0.05670724	0.1575501
chr7	1087076	1127485	40410	1	4	2	0.76005302	0.1575501	1	2	0.01091641	0.1575501
chr7	1127485	1197306	69822	2	4	2	0.76005302	0.08289318	1	3	0.01091641	0.30102999
chr7	1197306	1197365	60	1	4	2	0.47744371	0.08289318	2	3	0.0429175	0.30102999
chr7	1197365	1245696	48332	1	4	2	0.76005302	0.08289318	1	3	0.01091641	0.30102999
chr7	1245696	1245755	60	1	4	2	0.76005302	0.0429175	1	4	0.01091641	0.47744371
chr7	1245755	1292812	47058	1	4	2	0.76005302	0.08289318	1	3	0.01091641	0.30102999
chr7	1292812	1292871	60	1	4	3	0.76005302	0.17593012	1	3	0.01091641	0.17593012
chr7	1292871	1468185	175315	2	4	3	0.76005302	0.30102999	1	2	0.01091641	0.08289318
chr7	1468185	1541154	72970	2	4	3	0.76005302	0.17593012	1	3	0.01091641	0.17593012
chr7	1541154	1541213	60	1	5	3	0.68214471	0.17593012	2	3	0.02162467	0.17593012
chr7	1541213	1613355	72143	2	5	2	0.68214471	0.08289318	2	3	0.02162467	0.30102999
chr7	1613355	1694406	81052	2	5	2	0.68214471	0.1575501	2	2	0.02162467	0.1575501
chr7	1694406	1694465	60	1	6	2	0.91219088	0.1575501	2	2	0.01053319	0.1575501
chr7	1694465	1737760	43296	1	5	2	1.02643191	0.1575501	1	2	0.00478973	0.1575501
chr7	1737760	1737819	60	1	5	3	1.02643191	0.30102999	1	2	0.00478973	0.08289318
chr7	1737819	1779563	41745	1	5	3	1.02643191	0.51676182	1	1	0.00478973	0.02438896
chr7	1779563	1828396	48834	2	5	3	0.68214471	0.51676182	2	1	0.02162467	0.02438896
chr7	1828396	1828455	60	1	5	3	0.68214471	0.30102999	2	2	0.02162467	0.08289318
chr7	1828455	1886724	58270	1	5	2	0.68214471	0.1575501	2	2	0.02162467	0.1575501
chr7	1886724	1886783	60	1	5	2	0.30102999	0.08289318	4	3	0.11390336	0.30102999
chr7	1886783	1958292	71510	1	5	2	0.45545077	0.08289318	3	3	0.05670724	0.30102999
chr7	1958292	2033390	75099	2	5	2	0.45545077	0.30102999	3	1	0.05670724	0.05404976
chr7	2033390	2112167	78778	2	5	2	0.68214471	0.30102999	2	1	0.02162467	0.05404976
chr7	2112167	2112226	60	1	5	3	0.68214471	0.51676182	2	1	0.02162467	0.02438896
chr7	2112226	2149076	36851	1	4	3	0.47744371	0.51676182	2	1	0.0429175	0.02438896
chr7	2149076	2149134	59	1	5	3	0.68214471	0.30102999	2	2	0.02162467	0.08289318
chr7	2149134	2570278	421145	9	4	1	0.47744371	0.1218695	2	1	0.0429175	0.1218695
chr7	2570278	2664449	94172	2	3	1	0.51676182	0.1218695	1	1	0.02438896	0.1218695
chr7	2664449	2913754	249306	5	3	0	0.51676182	0	1	1	0.02438896	0.30102999
chr7	2913754	2960602	46849	2	3	1	0.51676182	0.1218695	1	1	0.02438896	0.1218695
chr7	2960602	3305603	345002	6	3	0	0.51676182	0	1	1	0.02438896	0.30102999
chr7	3305603	3347071	41469	2	3	1	0.30102999	0.1218695	2	1	0.08289318	0.1218695
chr7	3347071	3772851	425781	8	3	0	0.30102999	0	2	1	0.08289318	0.30102999
chr7	3772851	3772910	60	1	3	1	0.30102999	0.1218695	2	1	0.08289318	0.1218695
chr7	3772910	3859349	86440	2	3	1	0.51676182	0.1218695	1	1	0.02438896	0.1218695
chr7	3859349	3956823	97475	3	3	2	0.51676182	0.30102999	1	1	0.02438896	0.05404976
chr7	3956823	3956882	60	1	3	2	0.30102999	0.30102999	2	1	0.08289318	0.05404976
chr7	3956882	4017824	60943	1	3	2	0.51676182	0.30102999	1	1	0.02438896	0.05404976
chr7	4017824	4017883	60	1	4	2	0.76005302	0.30102999	1	1	0.01091641	0.05404976
chr7	4017883	4063875	45993	1	3	2	0.51676182	0.30102999	1	1	0.02438896	0.05404976
chr7	4063875	4063934	60	1	3	2	0.17593012	0.30102999	3	1	0.17593012	0.05404976
chr7	4063934	4115793	51860	1	3	2	0.30102999	0.30102999	2	1	0.08289318	0.05404976
chr7	4115793	4289827	174035	5	4	2	0.47744371	0.30102999	2	1	0.0429175	0.05404976
chr7	4289827	4289886	60	1	4	3	0.30102999	0.30102999	3	2	0.10122019	0.08289318
chr7	4289886	4374077	84192	1	4	3	0.47744371	0.30102999	2	2	0.0429175	0.08289318
chr7	4374077	4468533	94457	1	4	3	0.76005302	0.30102999	1	2	0.01091641	0.08289318
chr7	4468533	4468592	60	1	4	3	0.47744371	0.30102999	2	2	0.0429175	0.08289318
chr7	4468592	4548182	79591	1	4	2	0.47744371	0.1575501	2	2	0.0429175	0.1575501
chr7	4548182	4617323	69142	2	6	2	0.91219088	0.08289318	2	3	0.01053319	0.30102999
chr7	4617323	4701866	84544	1	6	2	0.91219088	0.1575501	2	2	0.01053319	0.1575501
chr7	4701866	4701925	60	1	6	3	0.91219088	0.30102999	2	2	0.01053319	0.08289318
chr7	4701925	4783775	81851	1	6	3	1.31360226	0.30102999	1	2	0.00204627	0.08289318
chr7	4783775	4783834	60	1	6	3	0.91219088	0.30102999	2	2	0.01053319	0.08289318
chr7	4783834	4899774	115941	2	6	2	0.91219088	0.1575501	2	2	0.01053319	0.1575501
chr7	4899774	5040666	140893	4	6	3	0.91219088	0.30102999	2	2	0.01053319	0.08289318
chr7	5040666	5105390	64725	1	6	2	0.91219088	0.1575501	2	2	0.01053319	0.1575501
chr7	5105390	5151503	46114	1	5	2	0.68214471	0.1575501	2	2	0.02162467	0.1575501
chr7	5151503	5390677	239175	4	5	2	1.02643191	0.1575501	1	2	0.00478973	0.1575501
chr7	5390677	5492967	102291	3	5	3	1.02643191	0.17593012	1	3	0.00478973	0.17593012
chr7	5492967	5520557	27591	2	6	3	1.31360226	0.17593012	1	3	0.00204627	0.17593012
chr7	5520557	5520616	60	1	6	4	0.91219088	0.30102999	2	3	0.01053319	0.10122019
chr7	5520616	5561223	40608	2	6	3	0.91219088	0.17593012	2	3	0.01053319	0.17593012
chr7	5561223	5617875	56653	1	6	2	0.91219088	0.08289318	2	3	0.01053319	0.30102999
chr7	5617875	5864851	246977	5	5	2	0.68214471	0.08289318	2	3	0.02162467	0.30102999
chr7	5864851	6036490	171640	2	5	2	0.68214471	0.1575501	2	2	0.02162467	0.1575501
chr7	6036490	6202675	166186	3	5	1	0.68214471	0.05404976	2	2	0.02162467	0.30102999
chr7	6202675	6202734	60	1	5	1	0.68214471	0.02438896	2	3	0.02162467	0.51676182
chr7	6202734	6383450	180717	3	4	1	0.47744371	0.02438896	2	3	0.0429175	0.51676182
chr7	6383450	6383509	60	1	5	1	0.68214471	0.02438896	2	3	0.02162467	0.51676182

chr7	6383509	6536408	152900	3	3	0	0.30102999	0	2	1	0.08289318	0.30102999
chr7	6536408	6659432	123025	4	3	1	0.30102999	0.1218695	2	1	0.08289318	0.1218695
chr7	6659432	6777262	117831	2	3	0	0.30102999	0	2	1	0.08289318	0.30102999
chr7	6777262	6870884	93623	2	3	0	0.17593012	0	3	1	0.17593012	0.30102999
chr7	6870884	7137521	266638	3	3	0	0.10122019	0	4	1	0.30102999	0.30102999
chr7	7137521	7268479	130959	3	3	0	0.05670724	0	5	2	0.45545077	0.61140001
chr7	7268479	7335136	66658	1	3	0	0.10122019	0	4	2	0.30102999	0.61140001
chr7	7335136	7462326	127191	2	3	0	0.10122019	0	4	1	0.30102999	0.30102999
chr7	7462326	7462385	60	1	3	0	0.05670724	0	5	1	0.45545077	0.30102999
chr7	7462385	7554879	92495	2	3	0	0.10122019	0	4	1	0.30102999	0.30102999
chr7	7554879	7554938	60	1	3	0	0.05670724	0	5	2	0.45545077	0.61140001
chr7	7554938	7690102	135165	3	3	0	0.17593012	0	3	2	0.17593012	0.61140001
chr7	7690102	7770105	80004	2	3	0	0.10122019	0	4	2	0.30102999	0.61140001
chr7	7770105	7770164	60	1	3	1	0.05670724	0.02438896	5	3	0.45545077	0.51676182
chr7	7770164	8024246	254083	5	3	0	0.05670724	0	5	2	0.45545077	0.61140001
chr7	8024246	8024305	60	1	3	1	0.03070643	0.02438896	6	3	0.63695542	0.51676182
chr7	8024305	8122630	98326	2	3	0	0.05670724	0	5	2	0.45545077	0.61140001
chr7	8122630	8383976	261347	6	3	0	0.05670724	0	5	1	0.45545077	0.30102999
chr7	8383976	8384035	60	1	3	0	0.03070643	0	6	2	0.63695542	0.61140001
chr7	8384035	8519960	135926	2	3	0	0.05670724	0	5	2	0.45545077	0.61140001
chr7	8519960	8520019	60	1	3	0	0.03070643	0	6	2	0.63695542	0.61140001
chr7	8520019	8602125	82107	1	3	0	0.10122019	0	4	1	0.30102999	0.30102999
chr7	8602125	8717542	115418	2	3	0	0.17593012	0	3	1	0.17593012	0.30102999
chr7	8717542	8872243	154702	2	2	0	0.08289318	0	3	1	0.30102999	0.30102999
chr7	8872243	8872302	60	1	2	0	0.08289318	0	3	2	0.30102999	0.61140001
chr7	8872302	9197157	324856	3	2	0	0.08289318	0	3	1	0.30102999	0.30102999
chr7	9197157	9271055	73899	2	2	0	0.0429175	0	4	1	0.47744371	0.30102999
chr7	9271055	9271114	60	1	2	0	0.02162467	0	5	1	0.68214471	0.30102999
chr7	9271114	9737453	466340	3	2	0	0.08289318	0	3	1	0.30102999	0.30102999
chr7	9737453	10146085	408633	3	1	0	0.02438896	0	3	1	0.51676182	0.30102999
chr7	10146085	10146144	60	1	2	0	0.0429175	0	4	2	0.47744371	0.61140001
chr7	10146144	10246323	100180	1	1	0	0.02438896	0	3	2	0.51676182	0.61140001
chr7	10246323	10511035	264713	3	1	2	0.02438896	0.1575501	3	2	0.51676182	0.1575501
chr7	10511035	10609866	98832	3	2	2	0.08289318	0.1575501	3	2	0.30102999	0.1575501
chr7	10609866	10676324	66459	2	3	2	0.17593012	0.1575501	3	2	0.17593012	0.1575501
chr7	10676324	10747918	71595	2	3	2	0.10122019	0.1575501	4	2	0.30102999	0.1575501
chr7	10747918	10795079	47162	1	3	1	0.10122019	0.05404976	4	2	0.30102999	0.30102999
chr7	10795079	10958707	163629	2	3	1	0.05670724	0.05404976	5	2	0.45545077	0.30102999
chr7	10958707	11173509	214803	6	3	1	0.03070643	0.05404976	6	2	0.63695542	0.30102999
chr7	11173509	11173568	60	1	3	1	0.03070643	0.02438896	6	3	0.63695542	0.51676182
chr7	11173568	11302301	128734	2	3	1	0.05670724	0.02438896	5	3	0.45545077	0.51676182
chr7	11302301	11445872	143572	4	3	2	0.05670724	0.08289318	5	3	0.45545077	0.30102999
chr7	11445872	11628329	182458	3	3	1	0.05670724	0.02438896	5	3	0.45545077	0.51676182
chr7	11628329	11754376	126048	2	2	1	0.02162467	0.02438896	5	3	0.68214471	0.51676182
chr7	11754376	11843297	88922	3	2	1	0.01053319	0.02438896	6	3	0.91219088	0.51676182
chr7	11843297	11974866	131570	2	2	1	0.01053319	0.05404976	6	2	0.91219088	0.30102999
chr7	11974866	11974925	60	1	2	1	0.01053319	0.02438896	6	3	0.91219088	0.51676182
chr7	11974925	12193099	218175	2	2	1	0.02162467	0.02438896	5	3	0.68214471	0.51676182
chr7	12193099	12258124	65026	3	2	1	0.00493743	0.02438896	7	3	1.16581773	0.51676182
chr7	12258124	12305539	47416	1	2	1	0.02162467	0.02438896	5	3	0.68214471	0.51676182
chr7	12305539	12351994	46456	1	2	1	0.0429175	0.02438896	4	3	0.47744371	0.51676182
chr7	12351994	12352046	53	1	3	1	0.05670724	0.02438896	5	3	0.45545077	0.51676182
chr7	12352046	12409890	57845	1	2	1	0.02162467	0.02438896	5	3	0.68214471	0.51676182
chr7	12409890	12409949	60	1	2	1	0.01053319	0.02438896	6	3	0.91219088	0.51676182
chr7	12409949	12534238	124290	3	2	1	0.02162467	0.02438896	5	3	0.68214471	0.51676182
chr7	12534238	12594626	60389	2	2	1	0.01053319	0.02438896	6	3	0.91219088	0.51676182
chr7	12594626	12728910	134285	3	2	0	0.02162467	0	5	3	0.68214471	0.93173516
chr7	12728910	12920612	191703	2	2	0	0.0429175	0	4	3	0.47744371	0.93173516
chr7	12920612	13218621	298010	3	2	0	0.08289318	0	3	3	0.30102999	0.93173516
chr7	13218621	13218680	60	1	2	1	0.08289318	0.02438896	3	3	0.30102999	0.51676182
chr7	13218680	13327050	108371	1	2	0	0.08289318	0	3	2	0.30102999	0.61140001
chr7	13327050	13327109	60	1	2	0	0.0429175	0	4	2	0.47744371	0.61140001
chr7	13327109	13642792	315684	2	2	0	0.0429175	0	4	1	0.47744371	0.30102999
chr7	13642792	13642851	60	1	2	0	0.0429175	0	4	2	0.47744371	0.61140001
chr7	13642851	13926260	283410	2	2	0	0.0429175	0	4	1	0.47744371	0.30102999
chr7	13926260	13946246	19987	2	3	1	0.10122019	0.02438896	4	3	0.30102999	0.51676182
chr7	13946246	13978897	32652	1	3	1	0.10122019	0.05404976	4	2	0.30102999	0.30102999
chr7	13978897	14097341	118445	2	3	1	0.17593012	0.05404976	3	2	0.17593012	0.30102999
chr7	14097341	14188314	90974	2	3	2	0.17593012	0.1575501	3	2	0.17593012	0.1575501
chr7	14188314	14216326	28013	2	3	2	0.10122019	0.1575501	4	2	0.30102999	0.1575501
chr7	14216326	14326541	110216	3	3	2	0.17593012	0.1575501	3	2	0.17593012	0.1575501
chr7	14326541	14378115	51575	1	2	2	0.08289318	0.1575501	3	2	0.30102999	0.1575501
chr7	14378115	14443860	65746	2	2	3	0.08289318	0.30102999	3	2	0.30102999	0.08289318
chr7	14443860	14491342	47483	2	3	3	0.17593012	0.30102999	3	2	0.17593012	0.08289318
chr7	14491342	14545745	54404	1	3	2	0.17593012	0.1575501	3	2	0.17593012	0.1575501
chr7	14545745	14545804	60	1	3	2	0.10122019	0.1575501	4	2	0.30102999	0.1575501
chr7	14545804	14581695	35892	1	3	2	0.17593012	0.1575501	3	2	0.17593012	0.1575501
chr7	14581695	14661078	79384	3	3	3	0.17593012	0.30102999	3	2	0.17593012	0.08289318
chr7	14661078	14760901	99824	3	3	3	0.17593012	0.17593012	3	3	0.17593012	0.17593012
chr7	14760901	14802209	41309	2	3	3	0.05670724	0.17593012	5	3	0.45545077	0.17593012
chr7	14802209	14849628	47420	2	3	4	0.05670724	0.30102999	5	3	0.45545077	0.10122019
chr7	14849628	14951256	101629	2	2	4	0.0429175	0.30102999	4	3	0.47744371	0.10122019
chr7	14951256	15016581	65326	2	3	4	0.05670724	0.30102999	5	3	0.45545077	0.10122019
chr7	15016581	15016640	60	1	3	5	0.05670724	0.45545077	5	3	0.45545077	0.05670724
chr7	15016640	15079109	62470	1	2	5	0.0429175	0.45545077	4	3	0.47744371	0.05670724
chr7	15079109	15173336	94228	2	2	4	0.0429175	0.30102999	4	3	0.47744371	0.10122019
chr7	15173336	15173395	60	1	3	5	0.10122019	0.45545077	4	3	0.30102999	0.05670724
chr7	15173395	15307077	133683	3	2	4	0.0429175	0.30102999	4	3	0.47744371	0.10122019
chr7	15307077	15431079	124003	4	2	5	0.0429175	0.45545077	4	3	0.47744371	0.05670724
chr7	15431079	15870023	438945	7	2	4	0.0429175	0.30102999	4	3	0.47744371	0.10122019
chr7	15870023	15870082	60	1	4	4	0.11390336	0.30102999	5	3	0.30102999	0.10122019
chr7	15870082	15926980	56899	1	2	3	0.0429175	0.17593012	4	3	0.47744371	0.17593012
chr7	15926980	15994292	67313	2	3	3	0.10122019	0.17593012	4	3	0.30102999	0.17593012
chr7	15994292	16111821	117530	1	2	3	0.0429175	0.17593012	4	3	0.47744371	0.17593012
chr7	16111821	16161200	49380	2	2	3	0.02162467	0.17593012	5	3	0.68214471	0.17593012
chr7	16161200	16255671	94472	2	2	1	0.02162467	0.02438896	5	3	0.68214471	0.51676182
chr7	16255671	16381765	126095	2	2	1	0.0429175	0.02438896	4	3	0.47744371	0.51676182
chr7	16381765	16381824	60	1	3	2	0.05670724	0.08289318	5	3	0.45545077	0.30102999
chr7	16381824	16441305	59482	1	3	2	0.10122019	0.08289318	4	3	0.30102999	0.30102999
chr7	16441305	16441364	60	1	3	3	0.10122019	0.17593012	4	3	0.30102999	0.17593012
chr7	16441364	16535901	94538	2	3	3	0.17593012	0.17593012	3	3	0.17593012	0.17593012
chr7	16535901	16639522	103622	3	2	2	0.1575501	0.08289318	2	3	0.1575501	0.30102999
chr7	16639522	16639576	55	1	3	2	0.30102999	0.08289318	2	3	0.08289318	0.30102999
chr7	16639576	16722390	82815	2	2	1	0.1575501	0.02438896	2	3	0.1575501	0.51676182
chr7	16722390	16823903	101514	4	3	1	0.17593012	0.02438896	3	3	0.17593012	0.51676182
chr7	16823903	17074337	250435	6	2	1	0.1575501	0.02438896	2	3	0.1575501	0.51676182
chr7	17074337	17074396	60	1	2	2	0.1575501	0.08289318	2	3	0.1575501	0.30102999
chr7	17074396	17222770	148375	1	2	1	0.1575501	0.02438896	2	3	0.1575501	0.51676182
chr7	17222770	17492810	270041	5	2	2	0.1575501	0.08289318	2	3	0.1575501	0.30102999
chr7	17492810	17748786	255977	1	2	2	0.30102999	0.08289318	1	3	0.05404976	0.30102999
chr7	17748786	17861254	112469	2	1	0	0.1218695	0	1	1	0.1218695	0.30102999
chr7	17861254	17920474	59221	2	1	0	0.1218695	0	1	2	0.1218695	0.61140001
chr7	17920474	17920533	60	1	2	2	0.30102999	0.1575501	1	2	0.05404976	0.1575501
chr7	17920533	18067565	147033	2	2	1	0.30102999	0.1218695	1	1	0.05404976	0.1218695
chr7	18067565	18141854	74290	2	3	2	0.51676182	0.08289318	1	3	0.02438896	0.30102999
chr7	18141854	18270106	128253	2	3	2	0.51676182	0.1575501	1	2	0.02438896	0.1575501
chr7	18270106	18270165	60	1	3	3	0.51676182	0.17593012	1	3	0.02438896	0.17593012
chr7	18270165	18360977	90813	1	3	3	0.51676182	0.30102999	1	2	0.02438896	0.08289318
chr7	18360977	18637858	276882	4	2	3	0.30102999	0.51676182	1	1	0.05404976	0.02438896
chr7	18637858	18788707	150850	4	4	3	0.76005302	0.51676182	1	1	0.01091641	0.02438896
chr7	18788707	18788761	55	1	4	3	0.76005302	0.30102999	1	2	0.01091641	0.08289318
chr7	18788761	18868958	80198	1	3	3	0.51676182	0.30102999	1	2	0.02438896	0.08289318
chr7	18868958	18875267	6310	2	3	4	0.51676182	0.47744371	1	2	0.02438896	0.0429175
chr7	18875267	18925823	50557	1	3	3	0.51676182	0.30102999	1	2	0.02438896	0.08289318

chr7	18925823	18963231	37409	2	4	3	0.76005302	0.30102999	1	2	0.01091641	0.08289318
chr7	18963231	18991887	28657	1	3	3	0.51676182	0.30102999	1	2	0.02438896	0.08289318
chr7	18991887	19039777	47891	3	3	4	0.51676182	0.47744371	1	2	0.02438896	0.0429175
chr7	19039777	19095444	55668	1	3	4	0.51676182	0.76005302	1	1	0.02438896	0.01091641
chr7	19095444	19095503	60	1	3	4	0.30102999	0.76005302	2	1	0.08289318	0.01091641
chr7	19095503	19185418	89916	3	3	4	0.51676182	0.76005302	1	1	0.02438896	0.01091641
chr7	19185418	19335351	149934	1	3	3	0.51676182	0.51676182	1	1	0.02438896	0.02438896
chr7	19335351	19335410	60	1	3	3	0.30102999	0.51676182	2	1	0.08289318	0.02438896
chr7	19335410	19506913	171504	1	3	2	0.30102999	0.30102999	2	1	0.08289318	0.05404976
chr7	19506913	19616004	109092	2	3	2	0.17593012	0.30102999	3	1	0.17593012	0.05404976
chr7	19616004	19761586	145583	2	3	3	0.17593012	0.30102999	3	2	0.17593012	0.08289318
chr7	19761586	19791510	29925	3	3	3	0.17593012	0.17593012	3	3	0.17593012	0.17593012
chr7	19791510	19845493	53984	1	3	3	0.17593012	0.30102999	3	2	0.17593012	0.08289318
chr7	19845493	19983239	137747	1	2	3	0.1575501	0.51676182	2	1	0.1575501	0.02438896
chr7	19983239	20180778	197540	2	2	2	0.1575501	0.30102999	2	1	0.1575501	0.05404976
chr7	20180778	20308338	127561	3	2	1	0.30102999	0.1218695	1	1	0.05404976	0.1218695
chr7	20308338	20308397	60	1	2	2	0.1575501	0.30102999	2	1	0.1575501	0.05404976
chr7	20308397	20513161	204765	5	2	1	0.30102999	0.1218695	1	1	0.05404976	0.1218695
chr7	20513161	20513220	60	1	2	2	0.30102999	0.30102999	1	1	0.05404976	0.05404976
chr7	20513220	20601079	87860	1	2	1	0.30102999	0.1218695	1	1	0.05404976	0.1218695
chr7	20601079	20647463	46385	2	2	1	0.1575501	0.1218695	2	1	0.1575501	0.1218695
chr7	20647463	20689690	42228	2	3	2	0.17593012	0.1575501	3	2	0.17593012	0.1575501
chr7	20689690	20890868	201179	5	3	2	0.30102999	0.1575501	2	2	0.08289318	0.1575501
chr7	20890868	20890927	60	1	3	2	0.30102999	0.08289318	2	3	0.08289318	0.30102999
chr7	20890927	21266273	375347	3	3	1	0.30102999	0.05404976	2	2	0.08289318	0.30102999
chr7	21266273	21457292	191020	3	3	2	0.30102999	0.08289318	2	3	0.08289318	0.30102999
chr7	21457292	21505732	48441	2	3	3	0.30102999	0.17593012	2	3	0.08289318	0.17593012
chr7	21505732	21603894	98163	4	3	2	0.30102999	0.1575501	2	2	0.08289318	0.1575501
chr7	21603894	21603953	60	1	3	2	0.17593012	0.1575501	3	2	0.17593012	0.1575501
chr7	21603953	21644667	40715	1	3	2	0.30102999	0.1575501	2	2	0.08289318	0.1575501
chr7	21644667	21729443	84777	2	3	1	0.30102999	0.05404976	2	2	0.08289318	0.30102999
chr7	21729443	21729502	60	1	3	1	0.17593012	0.05404976	3	2	0.17593012	0.30102999
chr7	21729502	21784060	54559	1	3	1	0.30102999	0.05404976	2	2	0.08289318	0.30102999
chr7	21784060	21784119	60	1	4	1	0.47744371	0.05404976	2	2	0.0429175	0.30102999
chr7	21784119	21857833	73715	2	3	1	0.30102999	0.05404976	2	2	0.08289318	0.30102999
chr7	21857833	21857892	60	1	4	1	0.47744371	0.05404976	2	2	0.0429175	0.30102999
chr7	21857892	21940938	83047	2	4	0	0.47744371	0	2	2	0.0429175	0.61140001
chr7	21940938	21984723	43786	3	4	0	0.11390336	0	5	2	0.30102999	0.61140001
chr7	21984723	22293469	308747	5	4	0	0.30102999	0	3	2	0.10122019	0.61140001
chr7	22293469	22328002	34534	2	4	1	0.30102999	0.05404976	3	2	0.10122019	0.30102999
chr7	22328002	22328061	60	1	4	2	0.18734596	0.1575501	4	2	0.18734596	0.1575501
chr7	22328061	22393332	65272	2	4	2	0.30102999	0.1575501	3	2	0.10122019	0.1575501
chr7	22393332	22393391	60	1	4	3	0.18734596	0.30102999	4	2	0.18734596	0.08289318
chr7	22393391	22497220	103830	2	4	2	0.30102999	0.1575501	3	2	0.10122019	0.1575501
chr7	22497220	22527063	29844	2	4	4	0.30102999	0.47744371	3	2	0.10122019	0.0429175
chr7	22527063	22574223	47161	1	4	3	0.30102999	0.30102999	3	2	0.10122019	0.08289318
chr7	22574223	22609575	35353	1	4	2	0.30102999	0.1575501	3	2	0.10122019	0.1575501
chr7	22609575	22609634	60	1	4	4	0.30102999	0.47744371	3	2	0.10122019	0.0429175
chr7	22609634	22719884	110251	2	4	1	0.30102999	0.05404976	3	2	0.10122019	0.30102999
chr7	22719884	22855197	135314	4	4	2	0.30102999	0.1575501	3	2	0.10122019	0.1575501
chr7	22855197	22859924	4728	2	4	2	0.18734596	0.1575501	4	2	0.18734596	0.1575501
chr7	22859924	22935369	75446	2	4	2	0.30102999	0.1575501	3	2	0.10122019	0.1575501
chr7	22935369	23004041	68673	2	4	2	0.30102999	0.08289318	3	3	0.10122019	0.30102999
chr7	23004041	23004100	60	1	4	2	0.18734596	0.08289318	4	3	0.18734596	0.30102999
chr7	23004100	23205582	201483	4	4	2	0.18734596	0.1575501	4	2	0.18734596	0.1575501
chr7	23205582	23270882	65301	3	4	1	0.18734596	0.05404976	4	2	0.18734596	0.30102999
chr7	23270882	23340533	69652	4	4	1	0.30102999	0.05404976	3	2	0.10122019	0.30102999
chr7	23340533	23417837	77305	3	4	1	0.18734596	0.05404976	4	2	0.18734596	0.30102999
chr7	23417837	23417896	60	1	4	2	0.18734596	0.1575501	4	2	0.18734596	0.1575501
chr7	23417896	23493995	76100	2	3	2	0.17593012	0.1575501	3	2	0.17593012	0.1575501
chr7	23493995	23494054	60	1	4	2	0.18734596	0.1575501	4	2	0.18734596	0.1575501
chr7	23494054	23564766	70713	2	3	2	0.17593012	0.1575501	3	2	0.17593012	0.1575501
chr7	23564766	23981753	416988	10	3	3	0.17593012	0.17593012	3	3	0.17593012	0.17593012
chr7	23981753	24038809	57057	1	3	3	0.17593012	0.30102999	3	2	0.17593012	0.08289318
chr7	24038809	24126329	87521	1	3	2	0.17593012	0.1575501	3	2	0.17593012	0.1575501
chr7	24126329	24215940	89612	2	3	2	0.17593012	0.08289318	3	3	0.17593012	0.30102999
chr7	24215940	24288957	73018	1	3	2	0.30102999	0.08289318	2	3	0.08289318	0.30102999
chr7	24288957	24327835	38879	3	3	4	0.30102999	0.18734596	2	4	0.08289318	0.18734596
chr7	24327835	24469114	141280	1	3	3	0.30102999	0.10122019	2	4	0.08289318	0.30102999
chr7	24469114	24703338	234225	4	3	1	0.30102999	0.01091641	2	4	0.08289318	0.76005302
chr7	24703338	24846488	143151	3	3	0	0.30102999	0	2	2	0.08289318	0.61140001
chr7	24846488	25025817	179330	6	3	0	0.17593012	0	3	2	0.17593012	0.61140001
chr7	25025817	25158549	132733	1	3	0	0.30102999	0	2	2	0.08289318	0.61140001
chr7	25158549	25219261	60713	3	3	0	0.30102999	0	2	3	0.08289318	0.93173516
chr7	25219261	25673259	453999	5	3	1	0.30102999	0.02438896	2	3	0.08289318	0.51676182
chr7	25673259	25673318	60	1	4	1	0.47744371	0.02438896	2	3	0.0429175	0.51676182
chr7	25673318	25743493	70176	1	3	1	0.30102999	0.02438896	2	3	0.08289318	0.51676182
chr7	25743493	25827607	84115	1	2	1	0.1575501	0.02438896	2	3	0.1575501	0.51676182
chr7	25827607	25827666	60	1	2	1	0.1575501	0.01091641	2	4	0.1575501	0.76005302
chr7	25827666	25979147	151482	1	2	1	0.30102999	0.02438896	1	3	0.05404976	0.51676182
chr7	25979147	25979206	60	1	2	1	0.0429175	0.02438896	4	3	0.47744371	0.51676182
chr7	25979206	26207388	228183	3	2	1	0.1575501	0.02438896	2	3	0.1575501	0.51676182
chr7	26207388	26207447	60	1	4	1	0.18734596	0.02438896	4	3	0.18734596	0.51676182
chr7	26207447	26223793	16347	1	3	1	0.10122019	0.02438896	4	3	0.30102999	0.51676182
chr7	26223793	26237479	13687	2	3	1	0.17593012	0.02438896	3	3	0.17593012	0.51676182
chr7	26237479	26333612	96134	3	3	1	0.10122019	0.02438896	4	3	0.30102999	0.51676182
chr7	26333612	26404648	71037	1	3	1	0.17593012	0.02438896	3	3	0.17593012	0.51676182
chr7	26404648	26497881	93234	3	4	1	0.30102999	0.02438896	3	3	0.10122019	0.51676182
chr7	26497881	26574723	76843	2	4	1	0.18734596	0.01091641	4	4	0.18734596	0.76005302
chr7	26574723	26708215	133493	4	4	2	0.18734596	0.0429175	4	4	0.18734596	0.47744371
chr7	26708215	26765127	56913	2	4	2	0.18734596	0.02162467	4	5	0.18734596	0.68214471
chr7	26765127	26853441	88315	2	4	1	0.18734596	0.00478973	4	5	0.18734596	1.02643191
chr7	26853441	26916260	62820	1	4	1	0.30102999	0.00478973	3	5	0.10122019	1.02643191
chr7	26916260	26916319	60	1	4	2	0.30102999	0.02162467	3	5	0.10122019	0.68214471
chr7	26916319	27062058	145740	3	4	1	0.30102999	0.00478973	3	5	0.10122019	1.02643191
chr7	27062058	27236652	174595	16	4	2	0.30102999	0.02162467	3	5	0.10122019	0.68214471
chr7	27236652	27347452	110801	3	4	1	0.30102999	0.00478973	3	5	0.10122019	1.02643191
chr7	27347452	27347511	60	1	5	1	0.45545077	0.00478973	3	5	0.05670724	1.02643191
chr7	27347511	27434792	87282	1	4	1	0.30102999	0.00478973	3	5	0.10122019	1.02643191
chr7	27434792	27594647	159856	4	4	1	0.30102999	0.00204627	3	6	0.10122019	1.31360226
chr7	27594647	27869489	274843	7	4	1	0.30102999	0.00478973	3	5	0.10122019	1.02643191
chr7	27869489	27943174	73686	2	4	2	0.30102999	0.02162467	3	5	0.10122019	0.68214471
chr7	27943174	27988510	45337	2	5	2	0.45545077	0.02162467	3	5	0.05670724	0.68214471
chr7	27988510	28031516	43007	1	5	1	0.45545077	0.00478973	3	5	0.05670724	1.02643191
chr7	28031516	28031575	60	1	5	1	0.45545077	0.00204627	3	6	0.05670724	1.31360226
chr7	28031575	28103668	72094	1	5	1	0.45545077	0.00478973	3	5	0.05670724	1.02643191
chr7	28103668	28153691	50024	1	3	1	0.17593012	0.01091641	3	4	0.17593012	0.76005302
chr7	28153691	28153750	60	1	4	2	0.18734596	0.0429175	4	4	0.18734596	0.47744371
chr7	28153750	28238832	85083	2	3	2	0.10122019	0.0429175	4	4	0.30102999	0.47744371
chr7	28238832	28304410	65579	2	4	2	0.18734596	0.0429175	4	4	0.18734596	0.47744371
chr7	28304410	28304469	60	1	4	3	0.18734596	0.10122019	4	4	0.18734596	0.30102999
chr7	28304469	28387089	82621	2	4	2	0.18734596	0.0429175	4	4	0.18734596	0.47744371
chr7	28387089	28452432	65344	3	4	4	0.18734596	0.18734596	4	4	0.18734596	0.18734596
chr7	28452432	28566079	113648	3	5	4	0.30102999	0.18734596	4	4	0.11390336	0.18734596
chr7	28566079	28644935	78857	2	5	3	0.30102999	0.10122019	4	4	0.11390336	0.30102999
chr7	28644935	28692978	48044	2	5	4	0.30102999	0.18734596	4	4	0.11390336	0.18734596
chr7	28692978	28829293	136316	2	5	3	0.30102999	0.10122019	4	4	0.11390336	0.30102999
chr7	28829293	28829352	60	1	5	4	0.30102999	0.18734596	4	4	0.11390336	0.18734596
chr7	28829352	28992956	163605	2	5	1	0.30102999	0.01091641	4	4	0.11390336	0.76005302
chr7	28992956	29070295	77340	3	5	4	0.30102999	0.18734596	4	4	0.11390336	0.18734596
chr7	29070295	29135056	64762	2	5	3	0.45545077	0.10122019	3	4	0.05670724	0.30102999
chr7	29135056	29135115	60	1	5	4	0.45545077	0.18734596	3	4	0.05670724	0.18734596
chr7	29135115	29231988	96874	3	5	3	0.45545077	0.10122019	3	4	0.05670724	0.30102999
chr7	29231988	29394282	162295	5	5	4	0.45545077	0.18734596	3	4	0.05670724	0.18734596

chr7	29394282	29519922	125641	3	4	4	0.30102999	0.18734596	3	4	0.10122019	0.18734596
chr7	29519922	29552759	32838	2	4	4	0.18734596	0.18734596	4	4	0.18734596	0.18734596
chr7	29552759	29800160	247402	6	3	4	0.10122019	0.18734596	4	4	0.30102999	0.18734596
chr7	29800160	29800219	60	1	4	4	0.11390336	0.18734596	5	4	0.30102999	0.18734596
chr7	29800219	29959824	159606	3	3	4	0.10122019	0.18734596	4	4	0.30102999	0.18734596
chr7	29959824	29959878	55	1	5	4	0.19510895	0.18734596	5	4	0.19510895	0.18734596
chr7	29959878	30013689	53812	1	3	4	0.05670724	0.18734596	5	4	0.45545077	0.18734596
chr7	30013689	30013748	60	1	3	4	0.03070643	0.18734596	6	4	0.63695542	0.18734596
chr7	30013748	30085170	71423	2	3	3	0.03070643	0.10122019	6	4	0.63695542	0.30102999
chr7	30085170	30101770	16601	2	3	4	0.03070643	0.18734596	6	4	0.63695542	0.18734596
chr7	30101770	30186521	84752	2	3	3	0.10122019	0.10122019	4	4	0.30102999	0.30102999
chr7	30186521	30201914	15394	3	3	6	0.10122019	0.30102999	4	5	0.30102999	0.12309572
chr7	30201914	30263143	61230	1	3	6	0.30102999	0.30102999	2	5	0.08289318	0.12309572
chr7	30263143	30341627	78485	2	3	3	0.30102999	0.10122019	2	4	0.08289318	0.30102999
chr7	30341627	30341686	60	1	3	4	0.17593012	0.18734596	3	4	0.17593012	0.18734596
chr7	30341686	30410392	68707	2	3	3	0.30102999	0.10122019	2	4	0.08289318	0.30102999
chr7	30410392	30410451	60	1	4	3	0.47744371	0.10122019	2	4	0.0429175	0.30102999
chr7	30410451	30497755	87305	2	3	3	0.30102999	0.10122019	2	4	0.08289318	0.30102999
chr7	30497755	30497814	60	1	3	4	0.30102999	0.11390336	2	5	0.08289318	0.30102999
chr7	30497814	30590273	92460	2	3	3	0.30102999	0.17593012	2	3	0.08289318	0.17593012
chr7	30590273	30649261	58989	1	3	2	0.30102999	0.08289318	2	3	0.08289318	0.30102999
chr7	30649261	30649320	60	1	3	3	0.30102999	0.05670724	2	5	0.08289318	0.45545077
chr7	30649320	30739193	89874	2	3	2	0.30102999	0.02162467	2	5	0.08289318	0.68214471
chr7	30739193	30795638	56446	2	3	3	0.30102999	0.05670724	2	5	0.08289318	0.45545077
chr7	30795638	31019110	223473	6	3	4	0.30102999	0.11390336	2	5	0.08289318	0.30102999
chr7	31019110	31019168	59	1	3	4	0.30102999	0.06713722	2	6	0.08289318	0.44141547
chr7	31019168	31102323	83156	1	3	3	0.30102999	0.03070643	2	6	0.08289318	0.63695542
chr7	31102323	31242136	139814	4	3	3	0.30102999	0.05670724	2	5	0.08289318	0.45545077
chr7	31242136	31379833	137698	2	3	3	0.30102999	0.03070643	2	6	0.08289318	0.63695542
chr7	31379833	31379892	60	1	3	3	0.17593012	0.01598258	3	7	0.17593012	0.84395715
chr7	31379892	31469156	89265	1	3	3	0.30102999	0.01598258	2	7	0.08289318	0.84395715
chr7	31469156	31558457	89302	2	3	4	0.30102999	0.02074938	2	8	0.08289318	0.79906872
chr7	31558457	31558516	60	1	3	4	0.17593012	0.02074938	3	8	0.17593012	0.79906872
chr7	31558516	31623936	65421	2	2	4	0.1575501	0.02074938	2	8	0.1575501	0.79906872
chr7	31623936	31623995	60	1	4	4	0.30102999	0.02074938	3	8	0.10122019	0.79906872
chr7	31623995	31731788	107794	3	3	4	0.17593012	0.06713722	3	6	0.17593012	0.44141547
chr7	31731788	32010412	278625	6	3	3	0.17593012	0.05670724	3	5	0.17593012	0.45545077
chr7	32010412	32010471	60	1	4	3	0.30102999	0.05670724	3	5	0.10122019	0.45545077
chr7	32010471	32044755	34285	1	3	2	0.17593012	0.02162467	3	5	0.17593012	0.68214471
chr7	32044755	32113218	68464	1	3	2	0.17593012	0.0429175	3	4	0.17593012	0.47744371
chr7	32113218	32371664	258447	7	3	3	0.17593012	0.10122019	3	4	0.17593012	0.30102999
chr7	32371664	32527322	155659	2	3	2	0.17593012	0.08289318	3	3	0.17593012	0.30102999
chr7	32527322	32527381	60	1	3	3	0.17593012	0.10122019	3	4	0.17593012	0.30102999
chr7	32527381	32598515	71135	1	3	2	0.17593012	0.08289318	3	3	0.17593012	0.30102999
chr7	32598515	32598574	60	1	3	2	0.10122019	0.08289318	4	3	0.30102999	0.30102999
chr7	32598574	32651634	53061	1	2	2	0.0429175	0.08289318	4	3	0.47744371	0.30102999
chr7	32651634	32651693	60	1	2	2	0.02162467	0.0429175	5	4	0.68214471	0.47744371
chr7	32651693	32700095	48403	1	2	2	0.02162467	0.08289318	5	3	0.68214471	0.30102999
chr7	32700095	32766307	66213	1	2	1	0.02162467	0.02438896	5	3	0.68214471	0.51676182
chr7	32766307	32837184	70878	4	3	1	0.05670724	0.02438896	5	3	0.45545077	0.51676182
chr7	32837184	32919238	82055	2	2	1	0.02162467	0.02438896	5	3	0.68214471	0.51676182
chr7	32919238	32945415	26178	1	2	0	0.02162467	0	5	3	0.68214471	0.93173516
chr7	32945415	32997734	52320	1	2	0	0.0429175	0	4	3	0.47744371	0.93173516
chr7	32997734	33074691	76958	1	1	0	0.02438896	0	3	3	0.51676182	0.93173516
chr7	33074691	33098391	23701	2	1	1	0.01091641	0.02438896	4	3	0.76005302	0.51676182
chr7	33098391	33422141	323751	7	1	1	0.02438896	0.02438896	3	3	0.51676182	0.51676182
chr7	33422141	33527096	104956	3	1	1	0.02438896	0.00478973	3	5	0.51676182	1.02643191
chr7	33527096	33707412	180317	4	1	0	0.02438896	0	3	5	0.51676182	1.60515106
chr7	33707412	33766232	58821	2	1	3	0.02438896	0.05670724	3	5	0.51676182	0.45545077
chr7	33766232	34058830	292599	6	1	2	0.02438896	0.02162467	3	5	0.51676182	0.68214471
chr7	34058830	34101615	42786	2	1	3	0.02438896	0.05670724	3	5	0.51676182	0.45545077
chr7	34101615	34194602	92988	3	1	4	0.01091641	0.11390336	4	5	0.76005302	0.30102999
chr7	34194602	34296783	102182	1	1	4	0.02438896	0.11390336	3	5	0.51676182	0.30102999
chr7	34296783	34296842	60	1	1	5	0.02438896	0.19510895	3	5	0.51676182	0.19510895
chr7	34296842	34460336	163495	3	1	3	0.02438896	0.05670724	3	5	0.51676182	0.45545077
chr7	34460336	34640648	180313	6	1	3	0.02438896	0.03070643	3	6	0.51676182	0.63695542
chr7	34640648	34795835	155188	3	1	2	0.02438896	0.01053319	3	6	0.51676182	0.91219088
chr7	34795835	34795894	60	1	1	4	0.01091641	0.06713722	4	6	0.76005302	0.44141547
chr7	34795894	34937566	141673	3	1	4	0.02438896	0.06713722	3	6	0.51676182	0.44141547
chr7	34937566	34993875	56310	3	1	4	0.02438896	0.03812622	3	7	0.51676182	0.60763643
chr7	34993875	35034997	41123	1	1	3	0.05404976	0.01598258	2	7	0.30102999	0.84395715
chr7	35034997	35077993	42997	1	1	2	0.05404976	0.01053319	2	6	0.30102999	0.91219088
chr7	35077993	35078052	60	1	1	2	0.01091641	0.01053319	4	6	0.76005302	0.91219088
chr7	35078052	35288201	210150	1	1	1	0.02438896	0.00204627	3	6	0.51676182	1.31360226
chr7	35288201	35290583	2383	2	1	1	0.01091641	0.00204627	4	6	0.76005302	1.31360226
chr7	35290583	35367246	76664	1	1	1	0.02438896	0.00204627	3	6	0.51676182	1.31360226
chr7	35367246	35367305	60	1	1	1	0.01091641	0.00204627	4	6	0.76005302	1.31360226
chr7	35367305	35524603	157299	1	1	1	0.02438896	0.00478973	3	5	0.51676182	1.02643191
chr7	35524603	35524662	60	1	1	1	0.00478973	0.00478973	5	5	1.02643191	1.02643191
chr7	35524662	35677930	153269	1	1	1	0.01091641	0.00478973	4	5	0.76005302	1.02643191
chr7	35677930	35791206	113277	3	1	3	0.01091641	0.03070643	4	6	0.76005302	0.63695542
chr7	35791206	35849355	58150	2	1	4	0.00478973	0.06713722	5	6	1.02643191	0.44141547
chr7	35849355	35920325	70971	1	0	4	0	0.11390336	5	5	1.60515106	0.30102999
chr7	35920325	36142309	221985	3	0	4	0	0.11390336	4	5	1.26272838	0.30102999
chr7	36142309	36209446	67138	2	0	4	0	0.11390336	5	5	1.60515106	0.30102999
chr7	36209446	36209505	60	1	0	4	0	0.06713722	5	6	1.60515106	0.44141547
chr7	36209505	36301255	91751	2	0	4	0	0.11390336	4	5	1.26272838	0.30102999
chr7	36301255	36447347	146093	4	1	4	0.00478973	0.06713722	5	6	1.02643191	0.44141547
chr7	36447347	36547449	100103	4	2	4	0.02162467	0.06713722	5	6	0.68214471	0.44141547
chr7	36547449	36713598	166150	5	2	4	0.01053319	0.06713722	6	6	0.91219088	0.44141547
chr7	36713598	36893957	180360	3	2	4	0.02162467	0.11390336	5	5	0.68214471	0.30102999
chr7	36893957	36958891	64935	4	2	5	0.02162467	0.19510895	5	5	0.68214471	0.19510895
chr7	36958891	36979511	20621	1	2	4	0.02162467	0.11390336	5	5	0.68214471	0.30102999
chr7	36979511	36979570	60	1	2	4	0.01053319	0.11390336	6	5	0.91219088	0.30102999
chr7	36979570	37240973	261404	6	2	4	0.02162467	0.11390336	5	5	0.68214471	0.30102999
chr7	37240973	37343082	102110	3	2	4	0.0429175	0.11390336	4	5	0.47744371	0.30102999
chr7	37343082	37635127	292046	5	2	4	0.0429175	0.06713722	4	6	0.47744371	0.44141547
chr7	37635127	37780600	145474	4	2	4	0.02162467	0.06713722	5	6	0.68214471	0.44141547
chr7	37780600	38423746	643147	15	2	4	0.0429175	0.06713722	4	6	0.47744371	0.44141547
chr7	38423746	38574510	150765	4	2	5	0.0429175	0.07511598	4	7	0.47744371	0.43181735
chr7	38574510	38574568	59	1	4	5	0.18734596	0.07511598	4	7	0.18734596	0.43181735
chr7	38574568	38698304	123737	3	3	5	0.10122019	0.07511598	4	7	0.30102999	0.43181735
chr7	38698304	38826767	128464	2	2	5	0.0429175	0.07511598	4	7	0.47744371	0.43181735
chr7	38826767	38826826	60	1	4	5	0.18734596	0.07511598	4	7	0.18734596	0.43181735
chr7	38826826	38860853	34028	1	3	5	0.10122019	0.07511598	4	7	0.30102999	0.43181735
chr7	38860853	38937687	76835	2	3	4	0.10122019	0.03812622	4	7	0.30102999	0.60763643
chr7	38937687	38937745	59	1	4	4	0.18734596	0.03812622	4	7	0.18734596	0.60763643
chr7	38937745	39010591	72847	1	3	4	0.10122019	0.06713722	4	6	0.30102999	0.44141547
chr7	39010591	39010650	60	1	3	5	0.10122019	0.12309572	4	6	0.30102999	0.30102999
chr7	39010650	39103000	92351	2	2	5	0.0429175	0.12309572	4	6	0.47744371	0.30102999
chr7	39103000	39221557	118558	3	3	5	0.10122019	0.12309572	4	6	0.30102999	0.30102999
chr7	39221557	39247234	25678	2	4	5	0.18734596	0.12309572	4	6	0.18734596	0.30102999
chr7	39247234	39379995	132762	3	4	4	0.30102999	0.06713722	3	6	0.10122019	0.44141547
chr7	39379995	39431147	51153	1	4	4	0.30102999	0.11390336	3	5	0.10122019	0.30102999
chr7	39431147	39504317	73171	3	4	4	0.18734596	0.11390336	4	5	0.18734596	0.30102999
chr7	39504317	39695542	191226	5	3	4	0.10122019	0.11390336	4	5	0.30102999	0.30102999
chr7	39695542	39847384	151843	2	3	3	0.10122019	0.05670724	4	5	0.30102999	0.45545077
chr7	39847384	39847443	60	1	3	4	0.10122019	0.11390336	4	5	0.30102999	0.30102999
chr7	39847443	39996107	148665	3	3	3	0.10122019	0.05670724	4	5	0.30102999	0.45545077
chr7	39996107	40068236	72130	2	3	2	0.10122019	0.08289318	4	3	0.30102999	0.30102999
chr7	40068236	40132520	64285	3	2	2	0.0429175	0.08289318	4	3	0.47744371	0.30102999
chr7	40132520	40304148	171629	4	2	1	0.0429175	0.02438896	4	3	0.47744371	0.51676182
chr7	40304148	40304207	60	1	2	3	0.01053319	0.17593012	6	3	0.91219088	0.17593012
chr7	40304207	40379983	75777	2	2	0	0.0429175	0	4	3	0.47744371	0.93173516

chr7	40379983	40680672	300690	8	2	0	0.02162467	0	5	3	0.68214471	0.93173516
chr7	40680672	40789006	108335	2	2	0	0.02162467	0	5	2	0.68214471	0.61140001
chr7	40789006	40817945	28940	2	2	0	0.01053319	0	6	3	0.91219088	0.93173516
chr7	40817945	40917509	99565	3	2	0	0.02162467	0	5	3	0.68214471	0.93173516
chr7	40917509	40917568	60	1	2	0	0.01053319	0	6	3	0.91219088	0.93173516
chr7	40917568	41084640	167073	1	2	0	0.02162467	0	5	3	0.68214471	0.93173516
chr7	41084640	41280084	195445	1	1	0	0.05404976	0	2	3	0.30102999	0.93173516
chr7	41280084	41280143	60	1	1	2	0.05404976	0.08289318	2	3	0.30102999	0.30102999
chr7	41280143	41546705	266563	1	1	2	0.1218695	0.08289318	1	3	0.1218695	0.30102999
chr7	41546705	41750898	204194	3	0	2	0	0.08289318	1	3	0.30102999	0.30102999
chr7	41750898	41915483	164586	1	0	1	0	0.02438896	1	3	0.30102999	0.51676182
chr7	41915483	42003745	88263	1	0	0	0	0	1	3	0.30102999	0.93173516
chr7	42003745	42079615	75871	2	0	1	0	0.02438896	1	3	0.30102999	0.51676182
chr7	42079615	42079670	56	1	1	3	0.05404976	0.05670724	2	5	0.30102999	0.45545077
chr7	42079670	42116391	36722	1	0	1	0	0.01091641	2	4	0.61140001	0.76005302
chr7	42116391	42188032	71642	1	0	1	0	0.02438896	2	3	0.61140001	0.51676182
chr7	42188032	42262821	74790	2	1	3	0.05404976	0.10122019	2	4	0.30102999	0.30102999
chr7	42262821	42441843	179023	2	0	1	0	0.01091641	2	4	0.61140001	0.76005302
chr7	42441843	42664387	222545	2	1	5	0.05404976	0.19510895	2	5	0.30102999	0.19510895
chr7	42664387	42664446	60	1	2	5	0.1575501	0.19510895	2	5	0.1575501	0.19510895
chr7	42664446	42807367	142922	1	2	4	0.1575501	0.11390336	2	5	0.1575501	0.30102999
chr7	42807367	42944511	137145	2	2	4	0.1575501	0.06713722	2	6	0.1575501	0.44141547
chr7	42944511	42949108	4598	2	2	5	0.1575501	0.07511598	2	7	0.1575501	0.43181735
chr7	42949108	42974342	25235	3	2	6	0.1575501	0.129913	2	7	0.1575501	0.30102999
chr7	42974342	43070435	96094	1	2	4	0.1575501	0.03812622	2	7	0.1575501	0.60763643
chr7	43070435	43139655	69221	1	2	3	0.1575501	0.01598258	2	7	0.1575501	0.84395715
chr7	43139655	43139714	60	1	2	3	0.08289318	0.01598258	3	7	0.30102999	0.84395715
chr7	43139714	43160630	20917	1	2	2	0.08289318	0.00493743	3	7	0.30102999	1.16581773
chr7	43160630	43267465	106836	2	2	2	0.08289318	0.01053319	3	6	0.30102999	0.91219088
chr7	43267465	43399629	132165	4	3	2	0.17593012	0.01053319	3	6	0.17593012	0.91219088
chr7	43399629	43399688	60	1	3	3	0.17593012	0.01598258	3	7	0.17593012	0.84395715
chr7	43399688	43483743	84056	2	3	2	0.17593012	0.00493743	3	7	0.17593012	1.16581773
chr7	43483743	43613764	130022	3	2	2	0.08289318	0.00493743	3	7	0.30102999	1.16581773
chr7	43613764	43613823	60	1	3	2	0.10122019	0.00493743	4	7	0.30102999	1.16581773
chr7	43613823	43631148	17326	1	3	2	0.10122019	0.01053319	4	6	0.30102999	0.91219088
chr7	43631148	43631207	60	1	3	3	0.05670724	0.03070643	5	6	0.45545077	0.63695542
chr7	43631207	43721565	90359	3	3	2	0.05670724	0.01053319	5	6	0.45545077	0.91219088
chr7	43721565	43844311	122747	3	3	1	0.05670724	0.00204627	5	6	0.45545077	1.31360226
chr7	43844311	43844370	60	1	3	1	0.03070643	0.00204627	6	6	0.63695542	1.31360226
chr7	43844370	43906135	61766	1	3	1	0.10122019	0.00204627	4	6	0.30102999	1.31360226
chr7	43906135	43945453	39319	4	3	3	0.10122019	0.03070643	4	6	0.30102999	0.63695542
chr7	43945453	43945512	60	1	3	3	0.03070643	0.03070643	6	6	0.63695542	0.63695542
chr7	43945512	44085092	139581	2	3	2	0.05670724	0.01053319	5	6	0.45545077	0.91219088
chr7	44085092	44103691	18600	2	3	2	0.03070643	0.01053319	6	6	0.63695542	0.91219088
chr7	44103691	44123435	19745	4	3	4	0.03070643	0.06713722	6	6	0.63695542	0.44141547
chr7	44123435	44146194	22760	1	3	3	0.03070643	0.03070643	6	6	0.63695542	0.63695542
chr7	44146194	44158547	12354	1	3	2	0.03070643	0.01053319	6	6	0.63695542	0.91219088
chr7	44158547	44179545	20999	4	3	1	0.03070643	0.00204627	6	6	0.63695542	1.31360226
chr7	44179545	44179591	47	1	3	3	0.03070643	0.03070643	6	6	0.63695542	0.63695542
chr7	44179591	44220292	40702	1	3	1	0.03070643	0.00204627	6	6	0.63695542	1.31360226
chr7	44220292	44243081	22790	2	3	1	0.03070643	8.47E-04	6	7	0.63695542	1.62048027
chr7	44243081	44243140	60	1	3	3	0.03070643	0.01598258	6	7	0.63695542	0.84395715
chr7	44243140	44359362	116223	3	3	1	0.03070643	8.47E-04	6	7	0.63695542	1.62048027
chr7	44359362	44423686	64325	2	3	1	0.05670724	8.47E-04	5	7	0.45545077	1.62048027
chr7	44423686	44501992	78307	2	3	1	0.05670724	0.00204627	5	6	0.45545077	1.31360226
chr7	44501992	44530729	28738	2	3	1	0.03070643	0.00204627	6	6	0.63695542	1.31360226
chr7	44530729	44572817	42089	1	3	1	0.05670724	0.00204627	5	6	0.45545077	1.31360226
chr7	44572817	44618716	45900	3	3	2	0.05670724	0.01053319	5	6	0.45545077	0.91219088
chr7	44618716	44646934	28219	4	3	2	0.03070643	0.01053319	6	6	0.63695542	0.91219088
chr7	44646934	44748537	101604	3	3	1	0.03070643	0.00204627	6	6	0.63695542	1.31360226
chr7	44748537	44787686	39150	2	3	1	0.03070643	8.47E-04	6	7	0.63695542	1.62048027
chr7	44787686	44837459	49774	1	3	1	0.05670724	8.47E-04	5	7	0.45545077	1.62048027
chr7	44837459	44886899	49441	2	3	1	0.10122019	8.47E-04	4	7	0.30102999	1.62048027
chr7	44886899	44924081	37183	1	3	1	0.10122019	0.00204627	4	6	0.30102999	1.31360226
chr7	44924081	44999529	75449	2	3	1	0.05670724	0.00204627	5	6	0.45545077	1.31360226
chr7	44999529	45103560	104032	3	3	2	0.05670724	0.01053319	5	6	0.45545077	0.91219088
chr7	45103560	45145929	42370	1	2	2	0.02162467	0.01053319	5	6	0.68214471	0.91219088
chr7	45145929	45179106	33178	1	2	2	0.0429175	0.01053319	4	6	0.47744371	0.91219088
chr7	45179106	45179165	60	1	2	3	0.0429175	0.03070643	4	6	0.47744371	0.63695542
chr7	45179165	45223788	44624	1	2	3	0.08289318	0.03070643	3	6	0.30102999	0.63695542
chr7	45223788	45285096	61309	2	2	3	0.08289318	0.01598258	3	7	0.30102999	0.84395715
chr7	45285096	45482804	197709	1	2	2	0.08289318	0.02162467	3	5	0.30102999	0.68214471
chr7	45482804	45618484	135681	2	2	2	0.0429175	0.02162467	4	5	0.47744371	0.68214471
chr7	45618484	45618543	60	1	2	3	0.0429175	0.05670724	4	5	0.47744371	0.45545077
chr7	45618543	45680560	62018	1	0	2	0	0.02162467	1	5	0.30102999	0.68214471
chr7	45680560	45724596	44037	2	0	2	0	0.01053319	1	6	0.30102999	0.91219088
chr7	45724596	45789334	64739	4	0	4	0	0.06713722	1	6	0.30102999	0.44141547
chr7	45789334	45929080	139747	1	0	3	0	0.03070643	1	6	0.30102999	0.63695542
chr7	45929080	45953318	24239	2	0	4	0	0.06713722	1	6	0.30102999	0.44141547
chr7	45953318	46028055	74738	1	0	3	0	0.05670724	1	5	0.30102999	0.45545077
chr7	46028055	46256488	228434	1	0	0	0	0	0	4	0	1.26272838
chr7	46256488	46392037	135550	1	0	0	0	0	0	3	0	0.93173516
chr7	46392037	46392096	60	1	0	1	0	0.01091641	2	4	0.61140001	0.76005302
chr7	46392096	46557430	165335	1	0	0	0	0	2	3	0.61140001	0.93173516
chr7	46557430	46700759	143330	1	0	0	0	0	2	1	0.61140001	0.30102999
chr7	46700759	46700818	60	1	0	0	0	0	2	2	0.61140001	0.61140001
chr7	46700818	46889750	188933	3	0	0	0	0	2	1	0.61140001	0.30102999
chr7	46889750	46889809	60	1	0	1	0	0.05404976	3	2	0.93173516	0.30102999
chr7	46889809	47034481	144673	1	0	1	0	0.1218695	2	1	0.61140001	0.1218695
chr7	47034481	47126917	92437	1	0	1	0	0.1218695	1	1	0.30102999	0.1218695
chr7	47126917	47276534	149618	2	0	2	0	0.1575501	1	2	0.30102999	0.1575501
chr7	47276534	47455744	179211	4	1	2	0.1218695	0.08289318	1	3	0.1218695	0.30102999
chr7	47455744	47455803	60	1	1	3	0.05404976	0.17593012	2	3	0.30102999	0.17593012
chr7	47455803	47533605	77803	1	1	2	0.05404976	0.08289318	2	3	0.30102999	0.30102999
chr7	47533605	47678537	144933	4	2	2	0.08289318	0.08289318	3	3	0.30102999	0.30102999
chr7	47678537	48378052	699516	17	2	2	0.0429175	0.08289318	4	3	0.47744371	0.30102999
chr7	48378052	49001278	623227	9	2	2	0.08289318	0.08289318	3	3	0.30102999	0.30102999
chr7	49001278	49130189	128912	2	2	2	0.0429175	0.08289318	4	3	0.47744371	0.30102999
chr7	49130189	49312227	182039	1	1	2	0.01091641	0.1575501	4	2	0.76005302	0.1575501
chr7	49312227	49371988	59762	1	1	1	0.01091641	0.05404976	4	2	0.76005302	0.30102999
chr7	49371988	49372045	58	1	2	1	0.02162467	0.05404976	5	2	0.68214471	0.30102999
chr7	49372045	49494872	122828	1	2	1	0.08289318	0.05404976	3	2	0.30102999	0.30102999
chr7	49494872	49620805	125934	2	2	2	0.08289318	0.1575501	3	2	0.30102999	0.1575501
chr7	49620805	49762649	141845	2	2	2	0.08289318	0.08289318	3	3	0.30102999	0.30102999
chr7	49762649	49762708	60	1	2	2	0.08289318	0.0429175	3	4	0.30102999	0.47744371
chr7	49762708	49906586	143879	3	2	2	0.1575501	0.0429175	2	4	0.1575501	0.47744371
chr7	49906586	50023023	116438	4	2	2	0.08289318	0.02162467	3	5	0.30102999	0.68214471
chr7	50023023	50070783	47761	2	2	2	0.08289318	0.01053319	3	6	0.30102999	0.91219088
chr7	50070783	50148050	77268	2	1	2	0.05404976	0.01053319	2	6	0.30102999	0.91219088
chr7	50148050	50238182	90133	3	1	2	0.05404976	0.00493743	2	7	0.30102999	1.16581773
chr7	50238182	50238241	60	1	2	2	0.08289318	0.00493743	3	7	0.30102999	1.16581773
chr7	50238241	50353062	114822	1	1	2	0.02438896	0.00493743	3	7	0.51676182	1.16581773
chr7	50353062	50594898	241837	7	1	3	0.02438896	0.01598258	3	7	0.51676182	0.84395715
chr7	50594898	50719810	124913	3	1	3	0.02438896	0.03070643	3	6	0.51676182	0.63695542
chr7	50719810	50750203	30394	2	1	3	0.02438896	0.01598258	3	7	0.51676182	0.84395715
chr7	50750203	50815106	64904	1	1	3	0.05404976	0.01598258	2	7	0.30102999	0.84395715
chr7	50815106	50892603	77498	2	1	3	0.1218695	0.01598258	1	7	0.1218695	0.84395715
chr7	50892603	50892662	60	1	1	3	0.05404976	0.01598258	2	7	0.30102999	0.84395715
chr7	50892662	51376150	483489	9	1	3	0.1218695	0.01598258	1	7	0.1218695	0.84395715
chr7	51376150	51376208	59	1	1	3	0.05404976	0.01598258	2	7	0.30102999	0.84395715
chr7	51376208	51520786	144579	1	0	3	0	0.03070643	1	6	0.30102999	0.63695542
chr7	51520786	51805320	284535	2	0	2	0	0.01053319	1	6	0.30102999	0.91219088
chr7	51805320	52068063	262744	2	0	2	0	0.02162467	0	5	0	0.68214471
chr7	52068063	52068122	60	1	0	2	0	0.01053319	0	6	0	0.91219088

chr7	52068122	52182150	114029	1	0	2	0	0.02162467	0	5	0	0.68214471
chr7	52182150	52182209	60	1	1	2	0.30102999	0.02162467	0	5	0	0.68214471
chr7	52182209	52317316	135108	1	0	1	0	0.01091641	0	4	0	0.76005302
chr7	52317316	52793551	476236	3	0	1	0	0.05404976	0	2	0	0.30102999
chr7	52793551	52957325	163775	2	0	2	0	0.0429175	0	4	0	0.47744371
chr7	52957325	53180432	223108	3	0	2	0	0.0429175	1	4	0.30102999	0.47744371
chr7	53180432	53605427	424996	3	0	1	0	0.05404976	1	2	0.30102999	0.30102999
chr7	53605427	53605486	60	1	0	1	0	0.02438896	1	3	0.30102999	0.51676182
chr7	53605486	53724423	118938	1	0	0	0	0	1	3	0.30102999	0.93173516
chr7	53724423	53724482	60	1	0	0	0	0	2	4	0.61140001	1.26272838
chr7	53724482	53825056	100575	2	0	0	0	0	2	3	0.61140001	0.93173516
chr7	53825056	54412818	587763	6	1	1	0.05404976	0.02438896	2	3	0.30102999	0.51676182
chr7	54412818	54412877	60	1	2	1	0.1575501	0.02438896	2	3	0.1575501	0.51676182
chr7	54412877	54604609	191733	2	1	1	0.05404976	0.02438896	2	3	0.30102999	0.51676182
chr7	54604609	54823177	218569	6	1	1	0.05404976	0.01091641	2	4	0.30102999	0.76005302
chr7	54823177	54924763	101587	2	2	1	0.1575501	0.01091641	2	4	0.1575501	0.76005302
chr7	54924763	54971953	47191	2	2	1	0.0429175	0.01091641	4	4	0.47744371	0.76005302
chr7	54971953	55147087	175135	2	2	1	0.08289318	0.01091641	3	4	0.30102999	0.76005302
chr7	55147087	55446925	299839	6	2	1	0.0429175	0.01091641	4	4	0.47744371	0.76005302
chr7	55446925	55569321	122397	3	2	1	0.08289318	0.01091641	3	4	0.30102999	0.76005302
chr7	55569321	55569380	60	1	2	1	0.0429175	0.01091641	4	4	0.47744371	0.76005302
chr7	55569380	55638029	68650	2	2	1	0.08289318	0.01091641	3	4	0.30102999	0.76005302
chr7	55638029	55638088	60	1	2	1	0.0429175	0.01091641	4	4	0.47744371	0.76005302
chr7	55638088	56082888	444801	10	2	1	0.08289318	0.02438896	3	3	0.30102999	0.51676182
chr7	56082888	56147677	64790	2	1	1	0.02438896	0.02438896	3	3	0.51676182	0.51676182
chr7	56147677	56169989	22313	3	1	2	0.02438896	0.08289318	3	3	0.51676182	0.30102999
chr7	56169989	56267361	97373	1	1	0	0.02438896	0	3	3	0.51676182	0.93173516
chr7	56267361	56566840	299480	1	1	0	0.05404976	0	2	3	0.30102999	0.93173516
chr7	56566840	56566899	60	1	1	1	0.05404976	0.02438896	2	3	0.30102999	0.51676182
chr7	56566899	56786860	219962	2	1	1	0.05404976	0.05404976	2	2	0.30102999	0.30102999
chr7	56786860	56996079	209220	1	0	0	0	0	0	1	0	0.30102999
chr7	62833583	63108972	275390	2	0	0	0	0	1	0	0.30102999	0
chr7	63108972	63374309	265338	2	3	0	0.17593012	0	3	1	0.17593012	0.30102999
chr7	63374309	63374368	60	1	4	0	0.18734596	0	4	1	0.18734596	0.30102999
chr7	63374368	63494989	120622	2	3	0	0.10122019	0	4	1	0.30102999	0.30102999
chr7	63494989	63919428	424440	7	2	0	0.08289318	0	3	1	0.30102999	0.30102999
chr7	63919428	63919487	60	1	4	0	0.18734596	0	4	2	0.18734596	0.61140001
chr7	63919487	64117428	197942	5	2	0	0.0429175	0	4	2	0.47744371	0.61140001
chr7	64117428	64170916	53489	3	4	0	0.18734596	0	4	2	0.18734596	0.61140001
chr7	64170916	64290160	119245	2	4	0	0.18734596	0	4	1	0.18734596	0.30102999
chr7	64290160	64362759	72600	3	5	0	0.19510895	0	5	2	0.19510895	0.61140001
chr7	64362759	64399980	37222	1	5	0	0.30102999	0	4	2	0.11390336	0.61140001
chr7	64399980	64435453	35474	1	4	0	0.18734596	0	4	2	0.18734596	0.61140001
chr7	64435453	64691936	256484	2	3	0	0.10122019	0	4	2	0.30102999	0.61140001
chr7	64691936	64691995	60	1	4	0	0.18734596	0	4	2	0.18734596	0.61140001
chr7	64691995	64754859	62865	1	2	0	0.08289318	0	3	2	0.30102999	0.61140001
chr7	64754859	64786270	31412	1	2	0	0.08289318	0	3	1	0.30102999	0.30102999
chr7	64786270	64866014	79745	3	3	0	0.17593012	0	3	1	0.17593012	0.30102999
chr7	64866014	64927758	61745	3	4	0	0.11390336	0	5	2	0.30102999	0.61140001
chr7	64927758	65070919	143162	1	3	0	0.10122019	0	4	2	0.30102999	0.61140001
chr7	65070919	65217105	146187	1	2	0	0.0429175	0	4	1	0.47744371	0.30102999
chr7	65217105	65217164	60	1	3	1	0.10122019	0.1218695	4	1	0.30102999	0.1218695
chr7	65217164	65269867	52704	1	3	0	0.10122019	0	4	1	0.30102999	0.30102999
chr7	65269867	65426422	156556	2	1	0	0.01091641	0	4	1	0.76005302	0.30102999
chr7	65426422	65426481	60	1	3	0	0.10122019	0	4	1	0.30102999	0.30102999
chr7	65426481	65580686	154206	2	3	0	0.17593012	0	3	1	0.17593012	0.30102999
chr7	65580686	65618072	37387	2	3	0	0.17593012	0	3	2	0.17593012	0.61140001
chr7	65618072	65618131	60	1	3	1	0.17593012	0.05404976	3	2	0.17593012	0.30102999
chr7	65618131	65726503	108373	2	3	1	0.17593012	0.1218695	3	1	0.17593012	0.1218695
chr7	65726503	65726562	60	1	4	1	0.30102999	0.05404976	3	2	0.10122019	0.30102999
chr7	65726562	65782693	56132	1	2	1	0.08289318	0.05404976	3	2	0.30102999	0.30102999
chr7	65782693	65886456	103764	4	2	0	0.08289318	0	3	2	0.30102999	0.61140001
chr7	65886456	65972673	86218	2	2	1	0.08289318	0.05404976	3	2	0.30102999	0.30102999
chr7	65972673	66246289	273617	8	3	1	0.17593012	0.05404976	3	2	0.17593012	0.30102999
chr7	66246289	66246348	60	1	3	1	0.17593012	0.02438896	3	3	0.17593012	0.51676182
chr7	66246348	66389043	142696	3	2	1	0.1575501	0.05404976	2	2	0.1575501	0.30102999
chr7	66389043	66418437	29395	2	3	1	0.17593012	0.05404976	3	2	0.17593012	0.30102999
chr7	66418437	66540178	121742	3	3	1	0.30102999	0.05404976	2	2	0.08289318	0.30102999
chr7	66540178	66730616	190439	4	2	1	0.1575501	0.05404976	2	2	0.1575501	0.30102999
chr7	66730616	66824455	93840	2	4	1	0.47744371	0.05404976	2	2	0.0429175	0.30102999
chr7	66824455	66937687	113233	2	1	1	0.05404976	0.05404976	2	2	0.30102999	0.30102999
chr7	66937687	66937746	60	1	1	1	0.05404976	0.02438896	2	3	0.30102999	0.51676182
chr7	66937746	67230326	292581	1	1	1	0.05404976	0.05404976	2	2	0.30102999	0.30102999
chr7	67230326	67230385	60	1	2	1	0.1575501	0.05404976	2	2	0.1575501	0.30102999
chr7	67230385	67429596	199212	1	2	1	0.30102999	0.05404976	1	2	0.05404976	0.30102999
chr7	67429596	67616356	186761	2	1	1	0.1218695	0.30102999	1	0	0.1218695	0
chr7	67616356	68065098	448743	2	0	1	0	0.30102999	0	0	0	0
chr7	68065098	68065157	60	1	0	1	0	0.30102999	1	0	0.30102999	0
chr7	68194327	68363806	169480	2	0	0	0	0	1	0	0.30102999	0
chr7	68363806	68564123	200318	2	1	0	0.05404976	0	2	1	0.30102999	0.30102999
chr7	68564123	68694925	130803	2	1	0	0.01091641	0	4	1	0.76005302	0.30102999
chr7	68694925	68953512	258588	3	1	0	0.02438896	0	3	1	0.51676182	0.30102999
chr7	68953512	68953571	60	1	2	0	0.08289318	0	3	1	0.30102999	0.30102999
chr7	68953571	69130237	176667	3	1	0	0.02438896	0	3	1	0.51676182	0.30102999
chr7	69130237	69130296	60	1	1	0	0.01091641	0	4	1	0.76005302	0.30102999
chr7	69130296	69225022	94727	2	1	0	0.02438896	0	3	1	0.51676182	0.30102999
chr7	69225022	69330796	105775	3	2	0	0.08289318	0	3	1	0.30102999	0.30102999
chr7	69330796	69492624	161829	3	1	0	0.02438896	0	3	1	0.51676182	0.30102999
chr7	69492624	69563017	70394	2	2	0	0.02162467	0	5	1	0.68214471	0.30102999
chr7	69563017	69709373	146357	2	2	0	0.0429175	0	4	1	0.47744371	0.30102999
chr7	69709373	69709432	60	1	3	0	0.10122019	0	4	1	0.30102999	0.30102999
chr7	69709432	69822421	112990	2	2	0	0.0429175	0	4	1	0.47744371	0.30102999
chr7	69822421	69869807	47387	2	3	0	0.10122019	0	4	1	0.30102999	0.30102999
chr7	69869807	69869866	60	1	3	0	0.05670724	0	5	1	0.45545077	0.30102999
chr7	69869866	70037607	167742	4	3	0	0.30102999	0	2	1	0.08289318	0.30102999
chr7	70037607	70037666	60	1	3	0	0.10122019	0	4	1	0.30102999	0.30102999
chr7	70037666	70070355	32690	1	3	0	0.30102999	0	2	1	0.08289318	0.30102999
chr7	70070355	70163569	93215	2	1	0	0.05404976	0	2	1	0.30102999	0.30102999
chr7	70163569	70220687	57119	2	2	0	0.08289318	0	3	1	0.30102999	0.30102999
chr7	70220687	70356011	135325	2	2	0	0.0429175	0	4	1	0.47744371	0.30102999
chr7	70356011	70356070	60	1	3	0	0.05670724	0	5	1	0.45545077	0.30102999
chr7	70356070	70594168	238099	2	2	0	0.0429175	0	4	1	0.47744371	0.30102999
chr7	70594168	70624379	30212	2	2	0	0.02162467	0	5	1	0.68214471	0.30102999
chr7	70624379	70624438	60	1	4	0	0.11390336	0	5	1	0.30102999	0.30102999
chr7	70624438	70690330	65893	1	3	0	0.05670724	0	5	1	0.45545077	0.30102999
chr7	70690330	70690389	60	1	3	0	0.05670724	0	5	2	0.45545077	0.61140001
chr7	70690389	70800643	110255	2	3	0	0.10122019	0	4	1	0.30102999	0.30102999
chr7	70800643	70825260	24618	2	3	0	0.05670724	0	5	1	0.45545077	0.30102999
chr7	70825260	70899755	74496	2	4	0	0.06713722	0	6	1	0.44141547	0.30102999
chr7	70899755	70981781	82027	2	4	0	0.18734596	0	4	1	0.18734596	0.30102999
chr7	70981781	71036391	54611	2	5	1	0.19510895	0.1218695	5	1	0.19510895	0.1218695
chr7	71036391	71095535	59145	1	1	1	0.01091641	0.1218695	4	1	0.76005302	0.1218695
chr7	71095535	71095594	60	1	1	1	0.01091641	0.02438896	4	3	0.76005302	0.51676182
chr7	71095594	71178081	82488	2	1	1	0.01091641	0.05404976	4	2	0.76005302	0.30102999
chr7	71178081	71345199	167119	5	1	1	0.01091641	0.02438896	4	3	0.76005302	0.51676182
chr7	71345199	72159859	814661	13	1	1	0.02438896	0.02438896	3	3	0.51676182	0.51676182
chr7	72159859	72286151	126293	4	2	1	0.0429175	0.00204627	4	6	0.47744371	1.31360226
chr7	72286151	72365957	79807	2	2	1	0.02162467	0.00204627	5	6	0.68214471	1.31360226
chr7	72365957	72726578	360622	1	2	1	0.02162467	0.01091641	5	4	0.68214471	0.76005302
chr7	72726578	72745047	18470	2	5	1	0.19510895	0.01091641	5	4	0.19510895	0.76005302
chr7	72745047	72772522	27476	2	5	1	0.19510895	0.00478973	5	5	0.19510895	1.02643191
chr7	72772522	73096428	323907	9	7	1	0.43181735	0.00478973	5	5	0.07511598	1.02643191
chr7	73096428	73107089	10662	3	8	1	0.4250187	0.00478973	6	5	0.08122616	1.02643191
chr7	73107089	73150418	43330	3	8	1	0.58747015	0.00478973	5	5	0.04407651	1.02643191

chr7	73150418	73152527	2110	2	8	1	0.4250187	0.00478973	6	5	0.08122616	1.02643191
chr7	73152527	73183363	30837	1	8	1	0.4250187	0.01091641	6	4	0.08122616	0.76005302
chr7	73183363	73246898	63536	1	7	1	0.30102999	0.01091641	6	4	0.129913	0.76005302
chr7	73246898	73313449	66552	4	7	1	0.30102999	0.00478973	6	5	0.129913	1.02643191
chr7	73313449	73313508	60	1	8	1	0.4250187	0.00478973	6	5	0.08122616	1.02643191
chr7	73313508	73445798	132291	1	7	1	0.30102999	0.00478973	6	5	0.129913	1.02643191
chr7	73445798	73525221	79424	2	7	1	0.30102999	0.01091641	6	4	0.129913	0.76005302
chr7	73525221	73700208	174988	8	8	1	0.4250187	0.00478973	6	5	0.08122616	1.02643191
chr7	73700208	73773985	73778	2	8	1	0.4250187	0.01091641	6	4	0.08122616	0.76005302
chr7	73773985	73901713	127729	4	8	1	0.30102999	0.01091641	7	4	0.13499366	0.76005302
chr7	73901713	73944662	42950	2	10	1	0.4167287	0.01091641	8	4	0.08923391	0.76005302
chr7	73944662	74024374	79713	3	11	1	0.55623409	0.01091641	8	4	0.05490675	0.76005302
chr7	74024374	74090455	66082	1	8	1	0.30102999	0.01091641	7	4	0.13499366	0.76005302
chr7	74090455	74119570	29116	1	7	1	0.20469099	0.02438896	7	3	0.20469099	0.51676182
chr7	74119570	74339044	219475	2	6	1	0.20064824	0.05404976	6	2	0.20064824	0.30102999
chr7	74339044	74481540	142497	1	5	1	0.12309572	0.05404976	6	2	0.30102999	0.30102999
chr7	74481540	74485767	4228	1	5	1	0.19510895	0.05404976	5	2	0.19510895	0.30102999
chr7	74485767	74485826	60	1	6	1	0.30102999	0.05404976	5	2	0.12309572	0.30102999
chr7	74485826	75062013	576188	1	4	1	0.11390336	0.05404976	5	2	0.30102999	0.30102999
chr7	75062013	75160961	98949	2	4	1	0.03812622	0.05404976	7	2	0.60763643	0.30102999
chr7	75160961	75197522	36562	2	6	1	0.129913	0.05404976	7	2	0.30102999	0.30102999
chr7	75197522	75278702	81181	2	4	1	0.06713722	0.05404976	6	2	0.44141547	0.30102999
chr7	75278702	75278761	60	1	5	1	0.12309572	0.05404976	6	2	0.30102999	0.30102999
chr7	75278761	75546088	267328	6	4	1	0.06713722	0.05404976	6	2	0.44141547	0.30102999
chr7	75546088	75729577	183490	5	4	1	0.06713722	0.1218695	6	1	0.44141547	0.1218695
chr7	75729577	75853698	124122	2	3	1	0.03070643	0.1218695	6	1	0.63695542	0.1218695
chr7	75853698	76139282	285585	11	3	1	0.03070643	0.05404976	6	2	0.63695542	0.30102999
chr7	76139282	76144740	5459	2	4	1	0.03812622	0.05404976	7	2	0.60763643	0.30102999
chr7	76144740	76200157	55418	1	4	1	0.03812622	0.1218695	7	1	0.60763643	0.1218695
chr7	76200157	76230206	30050	2	4	1	0.03812622	0.05404976	7	2	0.60763643	0.30102999
chr7	76230206	76476855	246650	1	4	1	0.11390336	0.1218695	5	1	0.30102999	0.1218695
chr7	76476855	76583962	107108	1	4	0	0.11390336	0	5	1	0.30102999	0.30102999
chr7	76583962	76724633	140672	1	3	0	0.17593012	0	3	1	0.17593012	0.30102999
chr7	76724633	76724692	60	1	3	0	0.10122019	0	4	1	0.30102999	0.30102999
chr7	76724692	76882615	157924	3	3	0	0.17593012	0	3	1	0.17593012	0.30102999
chr7	76882615	76941286	58672	1	2	0	0.08289318	0	3	1	0.30102999	0.30102999
chr7	76941286	76980543	39258	2	2	0	0.08289318	0	3	2	0.30102999	0.61140001
chr7	76980543	77954661	974119	20	2	0	0.1575501	0	2	2	0.1575501	0.61140001
chr7	77954661	78015051	60391	2	2	0	0.08289318	0	3	2	0.30102999	0.61140001
chr7	78015051	78095241	80191	2	2	0	0.1575501	0	2	2	0.1575501	0.61140001
chr7	78095241	78256506	161266	4	2	0	0.08289318	0	3	2	0.30102999	0.61140001
chr7	78256506	78364961	108456	3	2	0	0.08289318	0	3	1	0.30102999	0.30102999
chr7	78364961	78554817	189857	4	2	0	0.1575501	0	2	1	0.1575501	0.30102999
chr7	78554817	78711132	156316	2	1	0	0.05404976	0	2	0	0.30102999	0
chr7	78711132	79241694	530563	11	1	0	0.05404976	0	2	1	0.30102999	0.30102999
chr7	79241694	79554278	312585	4	1	0	0.02438896	0	3	1	0.51676182	0.30102999
chr7	79554278	79790594	236317	3	2	0	0.08289318	0	3	1	0.30102999	0.30102999
chr7	79790594	79790653	60	1	3	0	0.10122019	0	4	1	0.30102999	0.30102999
chr7	79790653	79903642	112990	3	2	0	0.08289318	0	3	1	0.30102999	0.30102999
chr7	79903642	79903701	60	1	2	0	0.02162467	0	5	1	0.68214471	0.30102999
chr7	79903701	80371841	468141	9	2	0	0.08289318	0	3	1	0.30102999	0.30102999
chr7	80371841	80430058	58218	2	2	0	0.02162467	0	5	1	0.68214471	0.30102999
chr7	80430058	80546138	116081	4	3	0	0.05670724	0	5	1	0.45545077	0.30102999
chr7	80546138	80730949	184812	2	2	0	0.02162467	0	5	1	0.68214471	0.30102999
chr7	80730949	80731008	60	1	3	1	0.05670724	0.1218695	5	1	0.45545077	0.1218695
chr7	80731008	80878634	147627	1	3	0	0.05670724	0	5	1	0.45545077	0.30102999
chr7	80878634	80878693	60	1	3	1	0.05670724	0.1218695	5	1	0.45545077	0.1218695
chr7	80878693	80998965	120273	2	2	0	0.02162467	0	5	1	0.68214471	0.30102999
chr7	80998965	81208524	209560	2	3	2	0.05670724	0.30102999	5	1	0.45545077	0.05404976
chr7	81208524	81208583	60	1	3	3	0.05670724	0.51676182	5	1	0.45545077	0.02438896
chr7	81208583	81329811	121229	2	2	1	0.0429175	0.1218695	4	1	0.47744371	0.1218695
chr7	81329811	81388038	58228	7	2	3	0.0429175	0.51676182	4	1	0.47744371	0.02438896
chr7	81388038	81639246	251209	4	2	2	0.08289318	0.30102999	3	1	0.30102999	0.05404976
chr7	81639246	81679933	40688	2	3	2	0.10122019	0.30102999	4	1	0.30102999	0.05404976
chr7	81679933	81850748	170816	4	3	2	0.17593012	0.30102999	3	1	0.17593012	0.05404976
chr7	81850748	81964573	113826	3	3	1	0.17593012	0.1218695	3	1	0.17593012	0.1218695
chr7	81964573	82027636	63064	2	3	0	0.17593012	0	3	1	0.17593012	0.30102999
chr7	82027636	82159009	131374	3	3	1	0.17593012	0.05404976	3	2	0.17593012	0.30102999
chr7	82159009	82159068	60	1	3	1	0.10122019	0.05404976	4	2	0.30102999	0.30102999
chr7	82159068	82231161	72094	1	3	0	0.10122019	0	4	2	0.30102999	0.61140001
chr7	82231161	82231220	60	1	4	0	0.18734596	0	4	2	0.18734596	0.61140001
chr7	82231220	82356348	125129	1	3	0	0.10122019	0	4	2	0.30102999	0.61140001
chr7	82356348	82460632	104285	2	2	0	0.08289318	0	3	2	0.30102999	0.61140001
chr7	82460632	82460691	60	1	3	0	0.10122019	0	4	2	0.30102999	0.61140001
chr7	82460691	82682438	221748	4	2	0	0.08289318	0	3	2	0.30102999	0.61140001
chr7	82682438	82736112	53675	2	2	0	0.0429175	0	4	2	0.47744371	0.61140001
chr7	82736112	82779289	43178	1	2	0	0.08289318	0	3	2	0.30102999	0.61140001
chr7	82779289	82870498	91210	1	2	0	0.1575501	0	2	2	0.1575501	0.61140001
chr7	82870498	82995159	124662	2	4	0	0.30102999	0	3	2	0.10122019	0.61140001
chr7	82995159	83037696	42538	1	3	0	0.17593012	0	3	2	0.17593012	0.61140001
chr7	83037696	83123092	85397	2	2	0	0.08289318	0	3	2	0.30102999	0.61140001
chr7	83123092	83123151	60	1	4	0	0.30102999	0	3	2	0.10122019	0.61140001
chr7	83123151	83229423	106273	2	3	0	0.17593012	0	3	2	0.17593012	0.61140001
chr7	83229423	83229482	60	1	4	0	0.30102999	0	3	2	0.10122019	0.61140001
chr7	83229482	83329365	99884	2	3	0	0.17593012	0	3	2	0.17593012	0.61140001
chr7	83329365	83636840	307476	4	4	0	0.30102999	0	3	2	0.10122019	0.61140001
chr7	83636840	83739821	102982	2	3	0	0.17593012	0	3	2	0.17593012	0.61140001
chr7	83739821	83791547	51727	1	3	0	0.30102999	0	2	2	0.08289318	0.61140001
chr7	83791547	83973024	181478	3	3	0	0.17593012	0	3	2	0.17593012	0.61140001
chr7	83973024	84509221	536198	5	1	0	0.1218695	0	1	2	0.1218695	0.61140001
chr7	84509221	84763387	254167	5	0	0	0	0	1	1	0.30102999	0.30102999
chr7	84763387	84763446	60	1	0	1	0	0.1218695	1	1	0.30102999	0.1218695
chr7	84763446	85258816	495371	3	0	0	0	0	1	1	0.30102999	0.30102999
chr7	85258816	85434850	176035	2	0	1	0	0.1218695	1	1	0.30102999	0.1218695
chr7	85434850	85654444	219595	1	0	0	0	0	1	1	0.30102999	0.30102999
chr7	85654444	85901986	247543	2	1	0	0.1218695	0	1	1	0.1218695	0.30102999
chr7	85901986	86052401	150416	2	1	0	0.02438896	0	3	1	0.51676182	0.30102999
chr7	86052401	86145909	93509	2	2	0	0.08289318	0	3	1	0.30102999	0.30102999
chr7	86145909	86259620	113712	1	2	0	0.08289318	0	3	0	0.30102999	0
chr7	86259620	86259679	60	1	2	0	0.0429175	0	4	0	0.47744371	0
chr7	86259679	86288504	28826	1	2	0	0.08289318	0	3	0	0.30102999	0
chr7	86288504	86394499	105996	3	2	0	0.08289318	0	3	1	0.30102999	0.30102999
chr7	86394499	86394558	60	1	2	0	0.0429175	0	4	1	0.47744371	0.30102999
chr7	86394558	86493791	99234	2	2	0	0.08289318	0	3	1	0.30102999	0.30102999
chr7	86493791	86541509	47719	2	2	0	0.0429175	0	4	1	0.47744371	0.30102999
chr7	86541509	87280113	738605	19	2	0	0.02162467	0	5	1	0.68214471	0.30102999
chr7	87280113	87538522	258410	8	2	0	0.01053319	0	6	1	0.91219088	0.30102999
chr7	87538522	87607681	69160	2	2	0	0.02162467	0	5	1	0.68214471	0.30102999
chr7	87607681	87643160	35480	2	2	0	0.01053319	0	6	1	0.91219088	0.30102999
chr7	87643160	87760675	117516	2	2	0	0.01053319	0	6	0	0.91219088	0
chr7	87760675	87760734	60	1	3	0	0.03070643	0	6	1	0.63695542	0.30102999
chr7	87760734	87844670	83937	2	2	0	0.01053319	0	6	0	0.91219088	0
chr7	87844670	87908580	63911	3	2	0	0.00493743	0	7	0	1.16581773	0
chr7	87908580	88026410	117831	1	2	0	0.01053319	0	6	0	0.91219088	0
chr7	88026410	88118032	91623	1	2	0	0.02162467	0	5	0	0.68214471	0
chr7	88118032	88118091	60	1	2	0	0.01053319	0	6	1	0.91219088	0.30102999
chr7	88118091	88244937	126847	1	2	0	0.02162467	0	5	1	0.68214471	0.30102999
chr7	88244937	88376367	131431	1	2	0	0.0429175	0	4	0	0.47744371	0
chr7	88376367	88424763	48397	1	2	0	0.08289318	0	3	0	0.30102999	0
chr7	88424763	88424822	60	1	2	0	0.0429175	0	4	0	0.47744371	0
chr7	88424822	88642670	217849	4	2	0	0.1575501	0	2	0	0.1575501	0
chr7	88642670	88897540	254871	6	2	0	0.1575501	0	2	1	0.1575501	0.30102999
chr7	88897540	89038216	140677	3	2	0	0.08289318	0	3	1	0.30102999	0.30102999
chr7	89038216	89038275	60	1	2	0	0.0429175	0	4	1	0.47744371	0.30102999

chr7	89038275	89322628	284354	2	2	0	0.08289318	0	3	1	0.30102999	0.30102999
chr7	89322628	89445171	122544	2	2	0	0.0429175	0	4	1	0.47744371	0.30102999
chr7	89445171	89445230	60	1	2	0	0.02162467	0	5	1	0.68214471	0.30102999
chr7	89445230	89627519	182290	1	2	0	0.0429175	0	4	1	0.47744371	0.30102999
chr7	89627519	89917070	289552	8	2	0	0.08289318	0	3	1	0.30102999	0.30102999
chr7	89917070	89982216	65147	2	2	0	0.08289318	0	3	0	0.30102999	0
chr7	89982216	90043788	61573	4	2	0	0.08289318	0	3	1	0.30102999	0.30102999
chr7	90043788	90112283	68496	2	2	0	0.02162467	0	5	1	0.68214471	0.30102999
chr7	90112283	90227502	115220	2	2	0	0.0429175	0	4	1	0.47744371	0.30102999
chr7	90227502	90227561	60	1	2	0	0.02162467	0	5	1	0.68214471	0.30102999
chr7	90227561	90366644	139084	3	2	0	0.0429175	0	4	1	0.47744371	0.30102999
chr7	90366644	90747356	380713	9	2	0	0.02162467	0	5	1	0.68214471	0.30102999
chr7	90747356	90792406	45051	2	3	0	0.05670724	0	5	1	0.45545077	0.30102999
chr7	90792406	90897006	104601	2	3	0	0.05670724	0	5	0	0.45545077	0
chr7	90897006	90968721	71716	2	3	0	0.01598258	0	7	1	0.84395715	0.30102999
chr7	90968721	90988735	20015	1	3	0	0.03070643	0	6	1	0.63695542	0.30102999
chr7	90988735	91145751	157017	1	3	0	0.10122019	0	4	1	0.30102999	0.30102999
chr7	91145751	91251883	106133	2	3	0	0.05670724	0	5	1	0.45545077	0.30102999
chr7	91251883	91251942	60	1	3	0	0.01598258	0	7	1	0.84395715	0.30102999
chr7	91251942	91404315	152374	1	3	0	0.01598258	0	7	0	0.84395715	0
chr7	91404315	91457963	53649	1	2	0	0.00493743	0	7	0	1.16581773	0
chr7	91457963	91571480	113518	3	2	0	0.00493743	0	7	1	1.16581773	0.30102999
chr7	91571480	91571539	60	1	3	0	0.01598258	0	7	1	0.84395715	0.30102999
chr7	91571539	91762089	190551	4	3	0	0.03070643	0	6	1	0.63695542	0.30102999
chr7	91762089	91829045	66957	2	3	0	0.05670724	0	5	1	0.45545077	0.30102999
chr7	91829045	91873423	44379	2	3	0	0.10122019	0	4	1	0.30102999	0.30102999
chr7	91873423	91914301	40879	2	3	0	0.05670724	0	5	1	0.45545077	0.30102999
chr7	91914301	92247424	333124	10	3	0	0.03070643	0	6	1	0.63695542	0.30102999
chr7	92247424	92279132	31709	1	3	0	0.03070643	0	6	0	0.63695542	0
chr7	92279132	92312387	33256	2	3	0	0.01598258	0	7	0	0.84395715	0
chr7	92312387	92441015	128629	4	3	0	0.01598258	0	7	1	0.84395715	0.30102999
chr7	92441015	92762042	321028	4	3	0	0.03070643	0	6	1	0.63695542	0.30102999
chr7	92762042	92793467	31426	2	4	0	0.03812622	0	7	1	0.60763643	0.30102999
chr7	92793467	92869219	75753	2	3	0	0.01598258	0	7	1	0.84395715	0.30102999
chr7	92869219	92940523	71305	3	3	0	0.03070643	0	6	1	0.63695542	0.30102999
chr7	92940523	93014702	74180	1	3	0	0.05670724	0	5	1	0.45545077	0.30102999
chr7	93014702	93113485	98784	3	4	0	0.11390336	0	5	1	0.30102999	0.30102999
chr7	93113485	93128868	15384	2	4	0	0.03812622	0	7	1	0.60763643	0.30102999
chr7	93128868	93170460	41593	1	4	0	0.11390336	0	5	1	0.30102999	0.30102999
chr7	93170460	93199449	28990	1	4	0	0.18734596	0	4	1	0.18734596	0.30102999
chr7	93199449	93233925	34477	2	4	1	0.18734596	0.1218695	4	1	0.18734596	0.1218695
chr7	93233925	93233984	60	1	4	1	0.03812622	0.1218695	7	1	0.60763643	0.1218695
chr7	93233984	93389236	155253	2	4	1	0.30102999	0.1218695	3	1	0.10122019	0.1218695
chr7	93389236	93389295	60	1	4	1	0.11390336	0.1218695	5	1	0.30102999	0.1218695
chr7	93389295	93472023	82729	1	4	0	0.18734596	0	4	1	0.18734596	0.30102999
chr7	93472023	93536746	64724	4	4	0	0.30102999	0	3	1	0.10122019	0.30102999
chr7	93536746	93633040	96295	6	4	0	0.18734596	0	4	1	0.18734596	0.30102999
chr7	93633040	93633099	60	1	4	1	0.18734596	0.1218695	4	1	0.18734596	0.1218695
chr7	93633099	93797640	164542	1	4	0	0.18734596	0	4	1	0.18734596	0.30102999
chr7	93797640	93971384	173745	3	5	0	0.07511598	0	7	1	0.43181735	0.30102999
chr7	93971384	94047784	76401	2	4	0	0.06713722	0	6	1	0.44141547	0.30102999
chr7	94047784	94082821	35038	3	5	0	0.04407651	0	8	1	0.58747015	0.30102999
chr7	94082821	94180435	97615	2	4	0	0.03812622	0	7	1	0.60763643	0.30102999
chr7	94180435	94229044	48610	3	4	0	0.02074938	0	8	1	0.79906872	0.30102999
chr7	94229044	94376650	147607	4	4	0	0.03812622	0	7	1	0.60763643	0.30102999
chr7	94376650	94399796	23147	1	4	0	0.06713722	0	6	1	0.44141547	0.30102999
chr7	94399796	94399855	60	1	4	0	0.03812622	0	7	1	0.60763643	0.30102999
chr7	94399855	94533676	133822	2	4	0	0.03812622	0	7	0	0.60763643	0
chr7	94533676	94813618	279943	7	4	0	0.03812622	0	7	1	0.60763643	0.30102999
chr7	94813618	94882150	68533	2	4	0	0.03812622	0	7	0	0.60763643	0
chr7	94882150	94989596	107447	3	4	0	0.03812622	0	7	1	0.60763643	0.30102999
chr7	94989596	95034510	44915	3	5	0	0.07511598	0	7	1	0.43181735	0.30102999
chr7	95034510	95063886	29377	1	5	0	0.12309572	0	6	1	0.30102999	0.30102999
chr7	95063886	95115411	51526	1	5	0	0.12309572	0	6	0	0.30102999	0
chr7	95115411	95167037	51627	2	5	0	0.07511598	0	7	0	0.43181735	0
chr7	95167037	95168537	1501	2	5	0	0.07511598	0	7	1	0.43181735	0.30102999
chr7	95168537	95388063	219527	4	4	0	0.11390336	0	5	1	0.30102999	0.30102999
chr7	95388063	95495600	107538	3	5	0	0.19510895	0	5	1	0.19510895	0.30102999
chr7	95495600	95495659	60	1	5	0	0.12309572	0	6	1	0.30102999	0.30102999
chr7	95495659	95579648	83990	2	5	0	0.19510895	0	5	1	0.19510895	0.30102999
chr7	95579648	95614200	34553	1	3	0	0.05670724	0	5	1	0.45545077	0.30102999
chr7	95614200	95659720	45521	1	3	0	0.05670724	0	5	0	0.45545077	0
chr7	95659720	95659779	60	1	4	0	0.06713722	0	6	0	0.44141547	0
chr7	95659779	95723689	63911	1	3	0	0.03070643	0	6	0	0.63695542	0
chr7	95723689	95749059	25371	2	3	0	0.03070643	0	6	1	0.63695542	0.30102999
chr7	95749059	95814120	65062	1	3	0	0.05670724	0	5	1	0.45545077	0.30102999
chr7	95814120	95863771	49652	4	4	0	0.11390336	0	5	1	0.30102999	0.30102999
chr7	95863771	95971363	107593	2	4	0	0.18734596	0	4	1	0.18734596	0.30102999
chr7	95971363	95971422	60	1	5	0	0.30102999	0	4	1	0.11390336	0.30102999
chr7	95971422	96055262	83841	1	4	0	0.18734596	0	4	1	0.18734596	0.30102999
chr7	96055262	96276166	220905	3	3	0	0.10122019	0	4	1	0.30102999	0.30102999
chr7	96276166	96536170	260005	7	4	0	0.18734596	0	4	1	0.18734596	0.30102999
chr7	96536170	96710984	174815	5	4	0	0.11390336	0	5	1	0.30102999	0.30102999
chr7	96710984	96786684	75701	2	4	0	0.18734596	0	4	1	0.18734596	0.30102999
chr7	96786684	96969273	182590	2	3	0	0.17593012	0	3	0	0.17593012	0
chr7	96969273	97038238	68966	2	3	0	0.10122019	0	4	0	0.30102999	0
chr7	97038238	97242449	204212	2	3	0	0.17593012	0	3	0	0.17593012	0
chr7	97242449	97242508	60	1	3	0	0.17593012	0	3	1	0.17593012	0.30102999
chr7	97242508	97428148	185641	3	3	0	0.17593012	0	3	0	0.17593012	0
chr7	97428148	97483869	55722	1	2	0	0.08289318	0	3	0	0.30102999	0
chr7	97483869	97488141	4273	3	2	0	0.08289318	0	3	1	0.30102999	0.30102999
chr7	97488141	97597642	109502	4	3	0	0.17593012	0	3	1	0.17593012	0.30102999
chr7	97597642	97597701	60	1	3	0	0.17593012	0	3	2	0.17593012	0.61140001
chr7	97597701	97652881	55181	1	2	0	0.08289318	0	3	1	0.30102999	0.30102999
chr7	97652881	97749450	96570	2	2	0	0.0429175	0	4	1	0.47744371	0.30102999
chr7	97749450	97749509	60	1	4	0	0.18734596	0	4	1	0.18734596	0.30102999
chr7	97749509	97852707	103199	2	3	0	0.10122019	0	4	1	0.30102999	0.30102999
chr7	97852707	97982004	129298	6	4	0	0.18734596	0	4	1	0.18734596	0.30102999
chr7	97982004	97982063	60	1	4	0	0.11390336	0	5	1	0.30102999	0.30102999
chr7	97982063	98019013	36951	1	4	0	0.18734596	0	4	1	0.18734596	0.30102999
chr7	98019013	98082325	63313	2	4	0	0.18734596	0	4	2	0.18734596	0.61140001
chr7	98082325	98082384	60	1	4	0	0.18734596	0	4	3	0.18734596	0.93173516
chr7	98082384	98194831	112448	1	4	0	0.18734596	0	4	2	0.18734596	0.61140001
chr7	98194831	98194890	60	1	4	0	0.11390336	0	5	2	0.30102999	0.61140001
chr7	98194890	98367904	173015	4	4	0	0.18734596	0	4	1	0.18734596	0.30102999
chr7	98367904	98448384	80481	2	4	0	0.11390336	0	5	1	0.30102999	0.30102999
chr7	98448384	98521861	73478	2	3	0	0.10122019	0	4	1	0.30102999	0.30102999
chr7	98521861	98521920	60	1	5	1	0.19510895	0.1218695	5	1	0.19510895	0.1218695
chr7	98521920	98573355	51436	1	4	1	0.18734596	0.1218695	4	1	0.18734596	0.1218695
chr7	98573355	98647166	73812	2	3	0	0.17593012	0	3	1	0.17593012	0.30102999
chr7	98647166	98687423	40258	2	4	0	0.30102999	0	3	1	0.10122019	0.30102999
chr7	98687423	98708711	21289	2	4	1	0.30102999	0.1218695	3	1	0.10122019	0.1218695
chr7	98708711	98784437	75727	3	5	1	0.19510895	0.05404976	5	2	0.19510895	0.30102999
chr7	98784437	98814636	30200	2	5	2	0.12309572	0.1575501	6	2	0.30102999	0.1575501
chr7	98814636	98898001	83366	3	6	2	0.20064824	0.1575501	6	2	0.20064824	0.1575501
chr7	98898001	98976760	78760	2	6	2	0.20064824	0.61140001	6	0	0.20064824	0
chr7	98976760	98976819	60	1	7	2	0.30102999	0.1575501	6	2	0.129913	0.1575501
chr7	98976819	99017203	40385	3	6	1	0.20064824	0.05404976	6	2	0.20064824	0.30102999
chr7	99017203	99222926	205724	10	7	1	0.30102999	0.05404976	6	2	0.129913	0.30102999
chr7	99222926	99271187	48262	1	7	1	0.30102999	0.1218695	6	1	0.129913	0.1218695
chr7	99271187	99307257	36071	2	8	1	0.4250187	0.1218695	6	1	0.08122616	0.1218695
chr7	99307257	99307316	60	1	8	1	0.20764654	0.05404976	8	2	0.20764654	0.30102999
chr7	99307316	99500904	193589	4	8	1	0.30102999	0.05404976	7	2	0.13499366	0.30102999
chr7	99500904	99633336	132433	5	9	1	0.42015402	0.02438896	7	3	0.08584816	0.51676182

chr7	99633336	99633395	60	1	9	1	0.30102999	0.02438896	8	3	0.13872638	0.51676182
chr7	99633395	99656999	23605	1	9	1	0.42015402	0.02438896	7	3	0.08584816	0.51676182
chr7	99656999	99671423	14425	2	8	1	0.30102999	0.02438896	7	3	0.13499366	0.51676182
chr7	99671423	99688525	17103	3	9	1	0.42015402	0.02438896	7	3	0.08584816	0.51676182
chr7	99688525	99718475	29951	8	9	1	0.30102999	0.02438896	8	3	0.13872638	0.51676182
chr7	99718475	99767299	48825	5	9	2	0.30102999	0.08289318	8	3	0.13872638	0.30102999
chr7	99767299	99945627	178329	4	9	1	0.42015402	0.02438896	7	3	0.08584816	0.51676182
chr7	99945627	99974771	29145	2	9	2	0.30102999	0.0429175	8	4	0.13872638	0.47744371
chr7	99974771	100067865	93095	4	9	1	0.30102999	0.02438896	8	3	0.13872638	0.51676182
chr7	100067865	100083726	15862	3	10	2	0.4167287	0.0429175	8	4	0.08923391	0.47744371
chr7	100083726	100198681	114956	2	10	1	0.4167287	0.02438896	8	3	0.08923391	0.51676182
chr7	100198681	100279058	80378	6	11	1	0.55623409	0.02438896	8	3	0.05490675	0.51676182
chr7	100279058	100320692	41635	3	12	1	0.72109894	0.01091641	8	4	0.03209037	0.76005302
chr7	100320692	100421854	101163	3	11	1	0.55623409	0.01091641	8	4	0.05490675	0.76005302
chr7	100421854	100649012	227159	6	12	1	0.55190077	0.01091641	9	4	0.0565833	0.76005302
chr7	100649012	100701931	52920	2	13	1	0.5498098	0.01091641	10	4	0.0574087	0.76005302
chr7	100701931	100733164	31234	2	14	1	0.71353304	0.01091641	10	4	0.03344703	0.76005302
chr7	100733164	100733213	50	1	16	1	0.91308053	0.01091641	11	4	0.01767679	0.76005302
chr7	100733213	100814592	81380	8	15	1	0.90719478	0.01091641	10	4	0.01817691	0.76005302
chr7	100814592	100886535	71944	7	16	1	1.13428373	0.01091641	10	4	0.00909834	0.76005302
chr7	100886535	100967178	80644	2	15	1	0.90719478	0.01091641	10	4	0.01817691	0.76005302
chr7	100967178	100998791	31614	2	15	1	0.90719478	0.02438896	10	3	0.01817691	0.51676182
chr7	100998791	101049499	50709	1	14	1	0.71353304	0.02438896	10	3	0.03344703	0.51676182
chr7	101049499	101092135	42637	1	13	1	0.5498098	0.02438896	10	3	0.0574087	0.51676182
chr7	101092135	101149639	57505	2	14	1	0.71353304	0.02438896	10	3	0.03344703	0.51676182
chr7	101149639	101149698	60	1	16	1	1.13428373	0.02438896	10	3	0.00909834	0.51676182
chr7	101149698	101269072	119375	4	15	1	0.90719478	0.02438896	10	3	0.01817691	0.51676182
chr7	101269072	101409332	140261	3	14	1	0.90719478	0.02438896	9	3	0.01817691	0.51676182
chr7	101409332	101463620	54289	1	13	1	0.71538971	0.02438896	9	3	0.0331093	0.51676182
chr7	101463620	101463679	60	1	15	1	0.90719478	0.02438896	10	3	0.01817691	0.51676182
chr7	101463679	101572570	108892	2	13	1	0.71538971	0.05404976	9	2	0.0331093	0.30102999
chr7	101572570	101658905	86336	3	13	1	0.71538971	0.02438896	9	3	0.0331093	0.51676182
chr7	101658905	101790733	131829	3	11	1	0.41444892	0.02438896	9	3	0.09154957	0.51676182
chr7	101790733	101790792	60	1	12	1	0.55190077	0.02438896	9	3	0.0565833	0.51676182
chr7	101790792	101836705	45914	1	9	1	0.30102999	0.05404976	8	2	0.13872638	0.30102999
chr7	101836705	101892906	56202	1	8	0	0.20764654	0	8	0	0.20764654	0
chr7	101892906	101931864	38959	2	9	0	0.30102999	0	8	0	0.13872638	0
chr7	101931864	101972013	40150	1	7	0	0.13499366	0	8	0	0.30102999	0
chr7	101972013	102053011	80999	3	7	0	0.20469099	0	7	0	0.20469099	0
chr7	102053011	102053070	60	1	8	0	0.20764654	0	8	0	0.20764654	0
chr7	102053070	102108989	55920	3	8	0	0.30102999	0	7	0	0.13499366	0
chr7	102108989	102114142	5154	2	7	0	0.20469099	0	7	0	0.20469099	0
chr7	102114142	102353540	239399	1	6	0	0.129913	0	7	0	0.30102999	0
chr7	102353540	102448618	95079	3	7	0	0.20469099	0	7	0	0.20469099	0
chr7	102448618	102491806	43189	2	7	0	0.13499366	0	8	0	0.30102999	0
chr7	102491806	102555946	64141	2	6	0	0.08122616	0	8	0	0.4250187	0
chr7	102555946	102939691	383746	6	6	0	0.129913	0	7	0	0.30102999	0
chr7	102939691	102988391	48701	3	6	0	0.129913	0	7	1	0.30102999	0.30102999
chr7	102988391	102988450	60	1	7	0	0.20469099	0	7	1	0.20469099	0.30102999
chr7	102988450	103032213	43764	1	6	0	0.129913	0	7	1	0.30102999	0.30102999
chr7	103032213	103071413	39201	2	7	0	0.20469099	0	7	1	0.20469099	0.30102999
chr7	103071413	103194117	122705	2	5	0	0.07511598	0	7	1	0.43181735	0.30102999
chr7	103194117	103248033	53917	2	5	0	0.04407651	0	8	1	0.58747015	0.30102999
chr7	103248033	103248092	60	1	7	0	0.05232577	0	10	1	0.56314362	0.30102999
chr7	103248092	103294597	46506	1	6	0	0.129913	0	7	1	0.30102999	0.30102999
chr7	103294597	103389855	95259	2	6	0	0.129913	0	7	0	0.30102999	0
chr7	103389855	103389914	60	1	7	0	0.08584816	0	9	1	0.42015402	0.30102999
chr7	103389914	103433940	44027	1	6	0	0.08122616	0	8	1	0.4250187	0.30102999
chr7	103433940	103479678	45739	1	6	0	0.129913	0	7	1	0.30102999	0.30102999
chr7	103479678	103479737	60	1	7	0	0.20469099	0	7	1	0.20469099	0.30102999
chr7	103479737	103664675	184939	5	6	0	0.129913	0	7	1	0.30102999	0.30102999
chr7	103664675	103664734	60	1	7	0	0.20469099	0	7	1	0.20469099	0.30102999
chr7	103664734	103767027	102294	2	6	0	0.129913	0	7	1	0.30102999	0.30102999
chr7	103767027	103767086	60	1	7	0	0.20469099	0	7	1	0.20469099	0.30102999
chr7	103767086	103835708	68623	2	6	0	0.129913	0	7	1	0.30102999	0.30102999
chr7	103835708	103947715	112008	2	6	0	0.129913	0	7	0	0.30102999	0
chr7	103947715	103997140	49426	2	7	0	0.20469099	0	7	1	0.20469099	0.30102999
chr7	103997140	103997199	60	1	9	0	0.42015402	0	7	1	0.08584816	0.30102999
chr7	103997199	104051443	54245	1	9	0	0.42015402	0	7	0	0.08584816	0
chr7	104051443	104100018	48576	2	9	0	0.20975986	0	9	0	0.20975986	0
chr7	104100018	104130970	30953	1	9	0	0.30102999	0	8	0	0.13872638	0
chr7	104130970	104179715	48746	1	7	0	0.20469099	0	7	0	0.20469099	0
chr7	104179715	104241701	61987	2	7	0	0.13499366	0	8	0	0.30102999	0
chr7	104241701	104284479	42779	2	7	0	0.05232577	0	10	0	0.56314362	0
chr7	104284479	104380817	96339	2	7	0	0.08584816	0	9	0	0.42015402	0
chr7	104380817	104477768	96952	2	6	0	0.08122616	0	8	0	0.4250187	0
chr7	104477768	104477827	60	1	7	0	0.08584816	0	9	0	0.42015402	0
chr7	104477827	104558966	81140	2	6	0	0.08122616	0	8	0	0.4250187	0
chr7	104558966	104657247	98282	3	6	0	0.129913	0	7	0	0.30102999	0
chr7	104657247	104730300	73054	2	6	0	0.08122616	0	8	0	0.4250187	0
chr7	104730300	104765232	34933	2	6	0	0.08122616	0	8	1	0.4250187	0.30102999
chr7	104765232	104861250	96019	2	6	0	0.08122616	0	8	0	0.4250187	0
chr7	104861250	105044594	183345	5	7	0	0.13499366	0	8	0	0.30102999	0
chr7	105044594	105044653	60	1	8	0	0.20764654	0	8	0	0.20764654	0
chr7	105044653	105177121	132469	3	7	0	0.13499366	0	8	0	0.30102999	0
chr7	105177121	105303894	126774	4	6	0	0.08122616	0	8	0	0.4250187	0
chr7	105303894	105465969	162076	3	5	0	0.07511598	0	7	0	0.43181735	0
chr7	105465969	105466028	60	1	5	0	0.04407651	0	8	0	0.58747015	0
chr7	105466028	105549271	83244	3	5	0	0.07511598	0	7	0	0.43181735	0
chr7	105549271	105549330	60	1	5	0	0.04407651	0	8	1	0.58747015	0.30102999
chr7	105549330	105642012	92683	2	5	0	0.12309572	0	6	0	0.30102999	0
chr7	105642012	105673543	31532	2	5	0	0.07511598	0	7	1	0.43181735	0.30102999
chr7	105673543	105747316	73774	1	5	0	0.07511598	0	7	0	0.43181735	0
chr7	105747316	105747375	60	1	5	0	0.02473314	0	9	0	0.76806864	0
chr7	105747375	105796199	48825	1	5	0	0.04407651	0	8	0	0.58747015	0
chr7	105796199	105829983	33785	2	5	0	0.04407651	0	8	1	0.58747015	0.30102999
chr7	105829983	105904293	74311	1	5	0	0.04407651	0	8	0	0.58747015	0
chr7	105904293	106014461	110169	2	5	0	0.19510895	0	5	0	0.19510895	0
chr7	106014461	106088963	74503	3	5	0	0.04407651	0	8	0	0.58747015	0
chr7	106088963	106223291	134329	3	5	0	0.07511598	0	7	0	0.43181735	0
chr7	106223291	106299963	76673	2	5	0	0.12309572	0	6	0	0.30102999	0
chr7	106299963	106513296	213334	6	5	0	0.07511598	0	7	0	0.43181735	0
chr7	106513296	106572973	59678	4	5	0	0.04407651	0	8	0	0.58747015	0
chr7	106572973	106573032	60	1	5	0	0.02473314	0	9	0	0.76806864	0
chr7	106573032	106762468	189437	4	5	0	0.04407651	0	8	0	0.58747015	0
chr7	106762468	106974294	211827	5	4	0	0.02074938	0	8	0	0.79906872	0
chr7	106974294	107002808	28515	2	4	0	0.01077081	0	9	0	1.01542894	0
chr7	107002808	107065863	63056	1	4	0	0.02074938	0	8	0	0.79906872	0
chr7	107065863	107065922	60	1	5	0	0.04407651	0	8	0	0.58747015	0
chr7	107065922	107188581	122660	3	4	0	0.03812622	0	7	0	0.60763643	0
chr7	107188581	107312634	124054	5	5	0	0.04407651	0	8	0	0.58747015	0
chr7	107312634	107350605	37972	2	5	0	0.02473314	0	9	0	0.76806864	0
chr7	107350605	107493398	142794	4	5	0	0.04407651	0	8	0	0.58747015	0
chr7	107493398	107493457	60	1	5	0	0.02473314	0	9	0	0.76806864	0
chr7	107493457	107556010	62554	2	5	0	0.04407651	0	8	0	0.58747015	0
chr7	107556010	107678029	122020	5	5	0	0.02473314	0	9	0	0.76806864	0
chr7	107678029	107678088	60	1	5	1	0.02473314	0.30102999	9	0	0.76806864	0
chr7	107678088	107757792	79705	1	5	0	0.04407651	0	8	0	0.58747015	0
chr7	107757792	107838455	80664	2	5	0	0.12309572	0	6	0	0.30102999	0
chr7	107838455	107838514	60	1	5	0	0.02473314	0	9	0	0.76806864	0
chr7	107838514	107973591	135078	3	5	0	0.07511598	0	7	0	0.43181735	0
chr7	107973591	108040063	66473	2	5	0	0.04407651	0	8	0	0.58747015	0
chr7	108040063	108113195	73133	2	4	0	0.02074938	0	8	0	0.79906872	0
chr7	108113195	108321116	207922	5	4	0	0.11390336	0	5	0	0.30102999	0

chr7	108321116	108440818	119703	2	4	0	0.18734596	0	4	0	0.18734596	0
chr7	108440818	108526931	86114	3	4	0	0.11390336	0	5	0	0.30102999	0
chr7	108526931	108596565	69635	2	5	0	0.19510895	0	5	0	0.19510895	0
chr7	108596565	108672760	76196	1	4	0	0.11390336	0	5	0	0.30102999	0
chr7	108672760	108717625	44866	1	3	0	0.05670724	0	5	0	0.45545077	0
chr7	108717625	108876280	158656	3	3	0	0.10122019	0	4	0	0.30102999	0
chr7	108876280	108876339	60	1	3	0	0.05670724	0	5	0	0.45545077	0
chr7	108876339	108931936	55598	1	3	0	0.10122019	0	4	0	0.30102999	0
chr7	108931936	109105351	173416	3	3	0	0.17593012	0	3	0	0.17593012	0
chr7	109105351	109105410	60	1	3	0	0.05670724	0	5	0	0.45545077	0
chr7	109105410	109173035	67626	2	3	0	0.10122019	0	4	0	0.30102999	0
chr7	109173035	109211744	38710	1	2	0	0.0429175	0	4	0	0.47744371	0
chr7	109211744	109319033	107290	2	1	0	0.01091641	0	4	0	0.76005302	0
chr7	109319033	109319092	60	1	2	0	0.0429175	0	4	0	0.47744371	0
chr7	109319092	109483610	164519	3	1	0	0.01091641	0	4	0	0.76005302	0
chr7	109483610	109612590	128981	4	3	0	0.10122019	0	4	0	0.30102999	0
chr7	109612590	109689563	76974	3	4	0	0.18734596	0	4	0	0.18734596	0
chr7	109689563	109850636	161074	3	3	0	0.10122019	0	4	0	0.30102999	0
chr7	109850636	109850695	60	1	4	0	0.18734596	0	4	0	0.18734596	0
chr7	109850695	109889645	38951	1	3	0	0.10122019	0	4	0	0.30102999	0
chr7	109889645	110187166	297522	6	3	0	0.17593012	0	3	0	0.17593012	0
chr7	110187166	110276505	89340	3	3	0	0.10122019	0	4	0	0.30102999	0
chr7	110276505	110602839	326335	8	5	0	0.30102999	0	4	0	0.11390336	0
chr7	110602839	110602898	60	1	5	0	0.19510895	0	5	0	0.19510895	0
chr7	110602898	110674867	71970	1	5	0	0.30102999	0	4	0	0.11390336	0
chr7	110674867	110731344	56478	2	5	0	0.45545077	0	3	0	0.05670724	0
chr7	110731344	110731403	60	1	5	0	0.30102999	0	4	0	0.11390336	0
chr7	110731403	110767384	35982	1	5	0	0.45545077	0	3	0	0.05670724	0
chr7	110767384	110767443	60	1	5	0	0.30102999	0	4	0	0.11390336	0
chr7	110767443	110839892	72450	2	5	0	0.45545077	0	3	0	0.05670724	0
chr7	110839892	110904950	65059	1	5	0	0.68214471	0	2	0	0.02162467	0
chr7	110904950	111105151	200202	4	5	0	0.45545077	0	3	0	0.05670724	0
chr7	111105151	111130599	25449	2	6	0	0.63695542	0	3	0	0.03070643	0
chr7	111130599	111149166	18568	2	6	0	0.44141547	0	4	0	0.06713722	0
chr7	111149166	111202026	52861	1	5	0	0.45545077	0	3	0	0.05670724	0
chr7	111202026	111274604	72579	2	4	0	0.30102999	0	3	0	0.10122019	0
chr7	111274604	111304031	29428	2	4	0	0.11390336	0	5	0	0.30102999	0
chr7	111304031	111366863	62833	1	4	0	0.18734596	0	4	0	0.18734596	0
chr7	111366863	111474710	107848	2	3	0	0.10122019	0	4	0	0.30102999	0
chr7	111474710	111503892	29183	2	5	0	0.19510895	0	5	0	0.19510895	0
chr7	111503892	111580210	76319	1	3	0	0.05670724	0	5	0	0.45545077	0
chr7	111580210	111613396	33187	1	3	0	0.10122019	0	4	0	0.30102999	0
chr7	111613396	111647224	33829	2	4	0	0.18734596	0	4	0	0.18734596	0
chr7	111647224	111647283	60	1	4	0	0.06713722	0	6	0	0.44141547	0
chr7	111647283	111741420	94138	2	4	0	0.18734596	0	4	0	0.18734596	0
chr7	111741420	111741479	60	1	5	0	0.19510895	0	5	0	0.19510895	0
chr7	111741479	111840999	99521	2	4	0	0.18734596	0	4	0	0.18734596	0
chr7	111840999	112095843	254845	8	4	0	0.06713722	0	6	0	0.44141547	0
chr7	112095843	112095902	60	1	5	0	0.12309572	0	6	0	0.30102999	0
chr7	112095902	112124983	29082	1	3	0	0.05670724	0	5	0	0.45545077	0
chr7	112124983	112211255	86273	2	2	0	0.0429175	0	4	0	0.47744371	0
chr7	112211255	112320660	109406	2	2	0	0.08289318	0	3	0	0.30102999	0
chr7	112320660	112378119	57460	2	2	0	0.0429175	0	4	0	0.47744371	0
chr7	112378119	112409979	31861	2	2	0	0.02162467	0	5	0	0.68214471	0
chr7	112409979	112459365	49387	2	2	0	0.01053319	0	6	0	0.91219088	0
chr7	112459365	112494208	34844	1	2	0	0.0429175	0	4	0	0.47744371	0
chr7	112494208	112494267	60	1	3	0	0.10122019	0	4	0	0.30102999	0
chr7	112494267	112683880	189614	4	2	0	0.1575501	0	2	0	0.1575501	0
chr7	112683880	112979837	295958	7	2	0	0.08289318	0	3	0	0.30102999	0
chr7	112979837	113282818	302982	7	3	0	0.17593012	0	3	0	0.17593012	0
chr7	113282818	113338567	55750	1	2	0	0.08289318	0	3	0	0.30102999	0
chr7	113338567	113391485	52919	2	3	0	0.17593012	0	3	0	0.17593012	0
chr7	113391485	113429675	38191	2	4	0	0.30102999	0	3	0	0.10122019	0
chr7	113429675	113558493	128819	6	4	0	0.18734596	0	4	0	0.18734596	0
chr7	113558493	113712256	153764	3	3	0	0.10122019	0	4	0	0.30102999	0
chr7	113712256	113885474	173219	5	3	0	0.05670724	0	5	0	0.45545077	0
chr7	113885474	113885533	60	1	4	0	0.11390336	0	5	0	0.30102999	0
chr7	113885533	114385008	499476	12	4	0	0.18734596	0	4	0	0.18734596	0
chr7	114385008	114474569	89562	4	5	0	0.30102999	0	4	0	0.11390336	0
chr7	114474569	114577454	102886	2	4	0	0.18734596	0	4	0	0.18734596	0
chr7	114577454	114899975	322522	8	5	0	0.19510895	0	5	0	0.19510895	0
chr7	114899975	114900034	60	1	5	0	0.12309572	0	6	0	0.30102999	0
chr7	114900034	115038104	138071	2	4	0	0.06713722	0	6	0	0.44141547	0
chr7	115038104	115099128	61025	2	5	0	0.12309572	0	6	0	0.30102999	0
chr7	115099128	115147607	48480	1	4	0	0.06713722	0	6	0	0.44141547	0
chr7	115147607	115243213	95607	3	4	0	0.03812622	0	7	0	0.60763643	0
chr7	115243213	115319336	76124	2	4	0	0.06713722	0	6	0	0.44141547	0
chr7	115319336	115479345	160010	4	5	0	0.07511598	0	7	0	0.43181735	0
chr7	115479345	115581915	102571	2	4	0	0.03812622	0	7	0	0.60763643	0
chr7	115581915	115670743	88829	3	4	0	0.02074938	0	8	0	0.79906872	0
chr7	115670743	115775118	104376	2	4	0	0.03812622	0	7	0	0.60763643	0
chr7	115775118	115898680	123563	3	4	0	0.02074938	0	8	0	0.79906872	0
chr7	115898680	116060004	161325	3	4	0	0.03812622	0	7	0	0.60763643	0
chr7	116060004	116625128	565125	20	4	0	0.02074938	0	8	0	0.79906872	0
chr7	116625128	116692089	66962	2	4	0	0.06713722	0	6	0	0.44141547	0
chr7	116692089	116692148	60	1	5	0	0.07511598	0	7	0	0.43181735	0
chr7	116692148	116870046	177899	5	4	0	0.03812622	0	7	0	0.60763643	0
chr7	116870046	117114170	244125	8	4	0	0.06713722	0	6	0	0.44141547	0
chr7	117114170	117581083	466914	11	3	0	0.03070643	0	6	0	0.63695542	0
chr7	117581083	117581142	60	1	4	0	0.03812622	0	7	0	0.60763643	0
chr7	117581142	117701929	120788	3	3	0	0.01598258	0	7	0	0.84395715	0
chr7	117701929	117701988	60	1	4	0	0.03812622	0	7	0	0.60763643	0
chr7	117701988	117825700	123713	2	4	0	0.06713722	0	6	0	0.44141547	0
chr7	117825700	117916326	90627	4	4	0	0.03812622	0	7	0	0.60763643	0
chr7	117916326	118349222	432897	9	4	0	0.06713722	0	6	0	0.44141547	0
chr7	118349222	118467051	117830	3	4	0	0.18734596	0	4	0	0.18734596	0
chr7	118467051	118538355	71305	2	4	0	0.11390336	0	5	0	0.30102999	0
chr7	118538355	118640924	102570	3	4	0	0.18734596	0	4	0	0.18734596	0
chr7	118640924	118773037	132114	2	3	0	0.10122019	0	4	0	0.30102999	0
chr7	118773037	118773096	60	1	3	0	0.05670724	0	5	0	0.45545077	0
chr7	118773096	118866671	93576	2	3	0	0.10122019	0	4	0	0.30102999	0
chr7	118866671	118866730	60	1	4	0	0.11390336	0	5	0	0.30102999	0
chr7	118866730	119058769	192040	4	4	0	0.18734596	0	4	0	0.18734596	0
chr7	119058769	119120843	62075	2	4	0	0.11390336	0	5	0	0.30102999	0
chr7	119120843	119223662	102820	2	3	0	0.05670724	0	5	0	0.45545077	0
chr7	119223662	119223721	60	1	5	0	0.19510895	0	5	0	0.19510895	0
chr7	119223721	119408972	185252	4	4	0	0.11390336	0	5	0	0.30102999	0
chr7	119408972	119542788	133817	2	3	0	0.05670724	0	5	0	0.45545077	0
chr7	119542788	119600024	57237	2	4	0	0.11390336	0	5	0	0.30102999	0
chr7	119600024	119661718	61695	1	4	0	0.18734596	0	4	0	0.18734596	0
chr7	119661718	119836734	175017	4	3	0	0.10122019	0	4	0	0.30102999	0
chr7	119836734	119836793	60	1	4	0	0.18734596	0	4	0	0.18734596	0
chr7	119836793	119988606	151814	3	3	0	0.10122019	0	4	0	0.30102999	0
chr7	119988606	120025982	37377	1	2	0	0.0429175	0	4	0	0.47744371	0
chr7	120025982	120026041	60	1	3	0	0.10122019	0	4	0	0.30102999	0
chr7	120026041	120144894	118854	2	2	0	0.0429175	0	4	0	0.47744371	0
chr7	120144894	120144953	60	1	3	0	0.05670724	0	5	0	0.45545077	0
chr7	120144953	120211721	66769	2	2	0	0.0429175	0	4	0	0.47744371	0
chr7	120211721	120300317	88597	3	2	0	0.02162467	0	5	0	0.68214471	0
chr7	120300317	120387875	87559	2	2	0	0.0429175	0	4	0	0.47744371	0
chr7	120387875	120508015	120141	4	2	1	0.02162467	0.30102999	5	0	0.68214471	0
chr7	120508015	120539024	31010	1	2	0	0.02162467	0	5	0	0.68214471	0
chr7	120539024	120539083	60	1	5	0	0.19510895	0	5	0	0.19510895	0
chr7	120539083	120581132	42050	1	4	0	0.11390336	0	5	0	0.30102999	0

chr7	120581132	120673969	92838	3	3	0	0.05670724	0	5	0	0.45545077	0
chr7	120673969	120674028	60	1	4	0	0.11390336	0	5	0	0.30102999	0
chr7	120674028	120780926	106899	3	3	0	0.05670724	0	5	0	0.45545077	0
chr7	120780926	120901907	120982	3	2	0	0.02162467	0	5	0	0.68214471	0
chr7	120901907	120901966	60	1	3	0	0.05670724	0	5	0	0.45545077	0
chr7	120901966	120998057	96092	2	2	0	0.02162467	0	5	0	0.68214471	0
chr7	120998057	120998116	60	1	2	0	0.01053319	0	6	0	0.91219088	0
chr7	120998116	121101368	103253	3	2	0	0.02162467	0	5	0	0.68214471	0
chr7	121101368	121347421	246054	6	2	0	0.01053319	0	6	0	0.91219088	0
chr7	121347421	121381748	34328	2	4	0	0.03812622	0	7	0	0.60763643	0
chr7	121381748	121512486	130739	3	3	0	0.03070643	0	6	0	0.63695542	0
chr7	121512486	121652294	139809	5	3	0	0.01598258	0	7	0	0.84395715	0
chr7	121652294	121701346	49053	2	5	0	0.07511598	0	7	0	0.43181735	0
chr7	121701346	121758401	57056	2	6	0	0.08122616	0	8	0	0.4250187	0
chr7	121758401	121809720	51320	2	6	0	0.129913	0	7	0	0.30102999	0
chr7	121809720	121959920	150201	3	5	0	0.07511598	0	7	0	0.43181735	0
chr7	121959920	121959979	60	1	6	0	0.129913	0	7	0	0.30102999	0
chr7	121959979	122091427	131449	3	5	0	0.07511598	0	7	0	0.43181735	0
chr7	122091427	122091486	60	1	7	0	0.20469099	0	7	0	0.20469099	0
chr7	122091486	122161350	69865	1	6	0	0.129913	0	7	0	0.30102999	0
chr7	122161350	122242786	81437	2	6	0	0.20064824	0	6	0	0.20064824	0
chr7	122242786	122291630	48845	2	7	0	0.30102999	0	6	0	0.129913	0
chr7	122291630	122412493	120864	5	6	0	0.20064824	0	6	0	0.20064824	0
chr7	122412493	122453614	41122	2	6	0	0.129913	0	7	0	0.30102999	0
chr7	122453614	122787185	333572	9	5	0	0.12309572	0	6	0	0.30102999	0
chr7	122787185	122879594	92410	3	6	0	0.20064824	0	6	0	0.20064824	0
chr7	122879594	122986200	106607	3	7	0	0.30102999	0	6	0	0.129913	0
chr7	122986200	122986259	60	1	8	0	0.30102999	0	7	0	0.13499366	0
chr7	122986259	123047169	60911	1	7	0	0.30102999	0	6	0	0.129913	0
chr7	123047169	123047228	60	1	7	0	0.20469099	0	7	0	0.20469099	0
chr7	123047228	123092563	45336	1	5	0	0.12309572	0	6	0	0.30102999	0
chr7	123092563	123142667	50105	2	5	0	0.07511598	0	7	0	0.43181735	0
chr7	123142667	123142726	60	1	7	0	0.08584816	0	9	0	0.42015402	0
chr7	123142726	123235242	92517	2	6	0	0.20064824	0	6	0	0.20064824	0
chr7	123235242	123266642	31401	2	6	0	0.129913	0	7	0	0.30102999	0
chr7	123266642	123266701	60	1	6	0	0.08122616	0	8	0	0.4250187	0
chr7	123266701	123384451	117751	2	6	0	0.20064824	0	6	0	0.20064824	0
chr7	123384451	123488183	103733	3	6	0	0.129913	0	7	0	0.30102999	0
chr7	123488183	123550345	62163	2	6	0	0.20064824	0	6	0	0.20064824	0
chr7	123550345	123627320	76976	2	5	0	0.12309572	0	6	0	0.30102999	0
chr7	123627320	123627379	60	1	6	0	0.20064824	0	6	0	0.20064824	0
chr7	123627379	123710958	83580	2	4	0	0.18734596	0	4	0	0.18734596	0
chr7	123710958	123711017	60	1	5	0	0.12309572	0	6	0	0.30102999	0
chr7	123711017	123840714	129698	3	4	0	0.18734596	0	4	0	0.18734596	0
chr7	123840714	123840773	60	1	4	0	0.11390336	0	5	0	0.30102999	0
chr7	123840773	123911816	71044	1	3	0	0.05670724	0	5	0	0.45545077	0
chr7	123911816	123952516	40701	2	4	0	0.11390336	0	5	0	0.30102999	0
chr7	123952516	124136443	183928	4	2	0	0.02162467	0	5	0	0.68214471	0
chr7	124136443	124136502	60	1	3	0	0.05670724	0	5	0	0.45545077	0
chr7	124136502	124568925	432424	11	3	0	0.10122019	0	4	0	0.30102999	0
chr7	124568925	124607233	38309	1	3	0	0.17593012	0	3	0	0.17593012	0
chr7	124607233	124607292	60	1	4	0	0.30102999	0	3	0	0.10122019	0
chr7	124607292	124671238	63947	1	2	0	0.08289318	0	3	0	0.30102999	0
chr7	124671238	124695381	24144	2	2	0	0.0429175	0	4	0	0.47744371	0
chr7	124695381	124968066	272686	5	2	0	0.08289318	0	3	0	0.30102999	0
chr7	124968066	124968125	60	1	2	0	0.0429175	0	4	0	0.47744371	0
chr7	124968125	125533178	565054	11	2	0	0.08289318	0	3	0	0.30102999	0
chr7	125533178	125533237	60	1	3	0	0.17593012	0	3	0	0.17593012	0
chr7	125533237	125610054	76818	2	2	0	0.08289318	0	3	0	0.30102999	0
chr7	125610054	126025603	415550	9	3	0	0.17593012	0	3	0	0.17593012	0
chr7	126025603	126199642	174040	5	5	0	0.45545077	0	3	0	0.05670724	0
chr7	126199642	126363805	164164	3	4	0	0.30102999	0	3	0	0.10122019	0
chr7	126363805	126711483	347679	10	5	0	0.45545077	0	3	0	0.05670724	0
chr7	126711483	126832119	120637	2	5	0	0.68214471	0	2	0	0.02162467	0
chr7	126832119	126882726	50608	2	5	0	0.45545077	0	3	0	0.05670724	0
chr7	126882726	126921477	38752	2	5	0	0.45545077	0	3	1	0.05670724	0.30102999
chr7	126921477	126961130	39654	1	4	0	0.30102999	0	3	1	0.10122019	0.30102999
chr7	126961130	127023981	62852	3	4	0	0.30102999	0	3	2	0.10122019	0.61140001
chr7	127023981	127103527	79547	3	4	0	0.18734596	0	4	2	0.18734596	0.61140001
chr7	127103527	127167239	63713	1	4	0	0.30102999	0	3	2	0.10122019	0.61140001
chr7	127167239	127254746	87508	4	4	0	0.47744371	0	2	2	0.0429175	0.61140001
chr7	127254746	127287308	32563	2	5	0	0.45545077	0	3	2	0.05670724	0.61140001
chr7	127287308	127341251	53944	1	4	0	0.30102999	0	3	2	0.10122019	0.61140001
chr7	127341251	127447514	106264	2	4	0	0.47744371	0	2	2	0.0429175	0.61140001
chr7	127447514	127506726	59213	2	4	0	0.30102999	0	3	2	0.10122019	0.61140001
chr7	127506726	127569334	62609	2	5	0	0.45545077	0	3	2	0.05670724	0.61140001
chr7	127569334	127569388	55	1	6	0	0.44141547	0	4	2	0.06713722	0.61140001
chr7	127569388	127816572	247185	6	4	0	0.18734596	0	4	2	0.18734596	0.61140001
chr7	127816572	127892164	75593	3	4	0	0.11390336	0	5	2	0.30102999	0.61140001
chr7	127892164	128002386	110223	4	4	0	0.06713722	0	6	2	0.44141547	0.61140001
chr7	128002386	128002445	60	1	5	0	0.12309572	0	6	2	0.30102999	0.61140001
chr7	128002445	128187067	184623	4	5	0	0.19510895	0	5	2	0.19510895	0.61140001
chr7	128187067	128187126	60	1	6	0	0.20064824	0	6	2	0.20064824	0.61140001
chr7	128187126	128319733	132608	1	5	0	0.19510895	0	5	2	0.19510895	0.61140001
chr7	128319733	128403716	83984	4	6	0	0.30102999	0	5	2	0.12309572	0.61140001
chr7	128403716	128412897	9182	2	7	0	0.43181735	0	5	2	0.07511598	0.61140001
chr7	128412897	128499016	86120	3	5	0	0.19510895	0	5	2	0.19510895	0.61140001
chr7	128499016	128504637	5622	2	7	1	0.43181735	0.05404976	5	2	0.07511598	0.30102999
chr7	128504637	128640881	136245	3	6	1	0.30102999	0.05404976	5	2	0.12309572	0.30102999
chr7	128640881	128680847	39967	2	6	1	0.20064824	0.05404976	6	2	0.20064824	0.30102999
chr7	128680847	128680906	60	1	6	1	0.129913	0.05404976	7	2	0.30102999	0.30102999
chr7	128680906	128711683	30778	1	6	1	0.20064824	0.05404976	6	2	0.20064824	0.30102999
chr7	128711683	128803026	91344	2	4	1	0.06713722	0.05404976	6	2	0.44141547	0.30102999
chr7	128803026	128803085	60	1	6	1	0.129913	0.05404976	7	2	0.30102999	0.30102999
chr7	128803085	128837765	34681	1	4	1	0.03812622	0.05404976	7	2	0.60763643	0.30102999
chr7	128837765	128890693	52929	2	4	1	0.06713722	0.05404976	6	2	0.44141547	0.30102999
chr7	128890693	128936237	45545	1	4	1	0.06713722	0.1218695	6	1	0.44141547	0.1218695
chr7	128936237	128996486	60250	1	4	1	0.06713722	0.30102999	6	0	0.44141547	0
chr7	128996486	129045658	49173	2	6	1	0.20064824	0.30102999	6	0	0.20064824	0
chr7	129045658	129106898	61241	2	7	1	0.30102999	0.30102999	6	0	0.129913	0
chr7	129106898	129119872	12975	2	7	1	0.13499366	0.30102999	8	0	0.30102999	0
chr7	129119872	129154874	35003	1	7	0	0.13499366	0	8	0	0.30102999	0
chr7	129154874	129210637	55764	2	8	0	0.20764654	0	8	0	0.20764654	0
chr7	129210637	129285324	74688	3	10	0	0.4167287	0	8	0	0.08923391	0
chr7	129285324	129350287	64964	2	9	0	0.30102999	0	8	0	0.13872638	0
chr7	129350287	129395053	44767	1	8	0	0.30102999	0	7	0	0.13499366	0
chr7	129395053	129395112	60	1	8	0	0.20764654	0	8	0	0.20764654	0
chr7	129395112	129463006	67895	2	8	0	0.30102999	0	7	0	0.13499366	0
chr7	129463006	129519395	56390	1	8	0	0.4250187	0	6	0	0.08122616	0
chr7	129519395	129519454	60	1	8	0	0.4250187	0	6	1	0.08122616	0.30102999
chr7	129519454	129616566	97113	2	8	0	0.4250187	0	6	0	0.08122616	0
chr7	129616566	129658149	41584	2	8	0	0.30102999	0	7	0	0.13499366	0
chr7	129658149	129658203	55	1	9	0	0.42015402	0	7	0	0.08584816	0
chr7	129658203	129825166	166964	4	7	0	0.20469099	0	7	0	0.20469099	0
chr7	129825166	129847959	22794	2	6	0	0.129913	0	7	0	0.30102999	0
chr7	129847959	129929601	81643	2	5	0	0.07511598	0	7	0	0.43181735	0
chr7	129929601	129929660	60	1	7	0	0.20469099	0	7	0	0.20469099	0
chr7	129929660	129984923	55264	2	5	0	0.07511598	0	7	0	0.43181735	0
chr7	129984923	130039897	54975	3	7	0	0.13499366	0	8	0	0.30102999	0
chr7	130039897	130078604	38708	2	6	0	0.129913	0	7	0	0.30102999	0
chr7	130078604	130130099	51496	1	5	0	0.07511598	0	7	0	0.43181735	0
chr7	130130099	130148949	18851	2	6	0	0.129913	0	7	0	0.30102999	0
chr7	130148949	130307998	159050	1	5	0	0.07511598	0	7	0	0.43181735	0
chr7	130307998	130308057	60	1	6	0	0.08122616	0	8	0	0.4250187	0

chr7	130308057	130347791	39735	1	6	0	0.129913	0	7	0	0.30102999	0
chr7	130347791	130354220	6430	2	6	0	0.08122616	0	8	0	0.4250187	0
chr7	130354220	130417851	63632	3	6	0	0.04875589	0	9	0	0.5732208	0
chr7	130417851	130458190	40340	2	6	0	0.01518174	0	11	0	0.9449689	0
chr7	130458190	130458249	60	1	7	0	0.03037338	0	11	0	0.73110763	0
chr7	130458249	130537984	79736	2	6	0	0.04875589	0	9	0	0.5732208	0
chr7	130537984	130538043	60	1	7	0	0.03037338	0	11	0	0.73110763	0
chr7	130538043	130572174	34132	1	7	0	0.08584816	0	9	0	0.42015402	0
chr7	130572174	130593514	21341	1	6	0	0.04875589	0	9	0	0.5732208	0
chr7	130593514	130712214	118701	3	6	0	0.08122616	0	8	0	0.4250187	0
chr7	130712214	130849462	137249	5	6	0	0.04875589	0	9	0	0.5732208	0
chr7	130849462	131023381	173920	5	7	0	0.08584816	0	9	0	0.42015402	0
chr7	131023381	131084069	60689	1	7	0	0.13499366	0	8	0	0.30102999	0
chr7	131084069	131120938	36870	2	7	0	0.08584816	0	9	0	0.42015402	0
chr7	131120938	131120997	60	1	7	0	0.05232577	0	10	0	0.56314362	0
chr7	131120997	131237966	116970	2	6	0	0.08122616	0	8	0	0.4250187	0
chr7	131237966	131370007	132042	5	7	0	0.05232577	0	10	0	0.56314362	0
chr7	131370007	131403218	33212	2	7	0	0.03037338	0	11	0	0.73110763	0
chr7	131403218	131480152	76935	2	7	0	0.05232577	0	10	0	0.56314362	0
chr7	131480152	131480211	60	1	8	0	0.05490675	0	11	0	0.55623409	0
chr7	131480211	131577036	96826	2	7	0	0.03037338	0	11	0	0.73110763	0
chr7	131577036	131707973	130938	3	7	0	0.05232577	0	10	0	0.56314362	0
chr7	131707973	131827920	119948	3	7	0	0.13499366	0	8	0	0.30102999	0
chr7	131827920	131827979	60	1	7	0	0.05232577	0	10	0	0.56314362	0
chr7	131827979	131876810	48832	1	7	0	0.08584816	0	9	0	0.42015402	0
chr7	131876810	131948767	71958	1	5	0	0.04407651	0	8	0	0.58747015	0
chr7	131948767	131948826	60	1	6	0	0.08122616	0	8	1	0.4250187	0.30102999
chr7	131948826	131984127	35302	1	5	0	0.04407651	0	8	1	0.58747015	0.30102999
chr7	131984127	131984186	60	1	5	0	0.02473314	0	9	1	0.76806864	0.30102999
chr7	131984186	132020408	36223	1	5	0	0.04407651	0	8	1	0.58747015	0.30102999
chr7	132020408	132020467	60	1	5	0	0.02473314	0	9	1	0.76806864	0.30102999
chr7	132020467	132059549	39083	1	5	0	0.04407651	0	8	1	0.58747015	0.30102999
chr7	132059549	132059608	60	1	6	0	0.04875589	0	9	2	0.5732208	0.61140001
chr7	132059608	132169510	109903	2	5	0	0.07511598	0	7	2	0.43181735	0.61140001
chr7	132169510	132169569	60	1	6	0	0.129913	0	7	3	0.30102999	0.93173516
chr7	132169569	132234470	64902	1	5	0	0.07511598	0	7	3	0.43181735	0.93173516
chr7	132234470	132234529	60	1	5	0	0.04407651	0	8	3	0.58747015	0.93173516
chr7	132234529	132281375	46847	1	4	0	0.02074938	0	8	2	0.79906872	0.61140001
chr7	132281375	132281434	60	1	5	0	0.04407651	0	8	2	0.58747015	0.61140001
chr7	132281434	132334303	52870	1	5	0	0.07511598	0	7	1	0.43181735	0.30102999
chr7	132334303	132346241	11939	2	5	0	0.04407651	0	8	1	0.58747015	0.30102999
chr7	132346241	132480379	134139	2	4	0	0.06713722	0	6	1	0.44141547	0.30102999
chr7	132480379	132600441	120063	3	5	0	0.12309572	0	6	1	0.30102999	0.30102999
chr7	132600441	132662185	61745	1	4	0	0.06713722	0	6	1	0.44141547	0.30102999
chr7	132662185	133314916	652732	14	4	0	0.03812622	0	7	1	0.60763643	0.30102999
chr7	133314916	133394713	79798	1	4	0	0.03812622	0	7	0	0.60763643	0
chr7	133394713	133394772	60	1	4	0	0.02074938	0	8	0	0.79906872	0
chr7	133394772	133602454	207683	5	4	0	0.03812622	0	7	0	0.60763643	0
chr7	133602454	133788973	186520	5	4	0	0.02074938	0	8	0	0.79906872	0
chr7	133788973	133933772	144800	3	4	0	0.06713722	0	6	0	0.44141547	0
chr7	133933772	134063115	129344	6	4	0	0.03812622	0	7	0	0.60763643	0
chr7	134063115	134104629	41515	2	4	1	0.03812622	0.30102999	7	0	0.60763643	0
chr7	134104629	134202238	97610	3	4	1	0.02074938	0.30102999	8	0	0.79906872	0
chr7	134202238	134332164	129927	3	5	1	0.02473314	0.30102999	9	0	0.76806864	0
chr7	134332164	134564635	232472	7	6	1	0.04875589	0.30102999	9	0	0.5732208	0
chr7	134564635	134702458	137824	3	6	1	0.08122616	0.30102999	8	0	0.4250187	0
chr7	134702458	134784510	82053	1	5	1	0.04407651	0.30102999	8	0	0.58747015	0
chr7	134784510	134853829	69320	3	4	1	0.02074938	0.30102999	8	0	0.79906872	0
chr7	134853829	134923456	69628	3	4	1	0.03812622	0.30102999	7	0	0.60763643	0
chr7	134923456	134968430	44975	2	5	1	0.07511598	0.30102999	7	0	0.43181735	0
chr7	134968430	134968489	60	1	6	1	0.04875589	0.30102999	9	0	0.5732208	0
chr7	134968489	134998314	29826	1	5	1	0.02473314	0.30102999	9	0	0.76806864	0
chr7	134998314	135048841	50528	1	5	1	0.04407651	0.30102999	8	0	0.58747015	0
chr7	135048841	135069843	21003	1	3	1	0.01598258	0.30102999	7	0	0.84395715	0
chr7	135069843	135099056	29214	5	3	1	0.0079614	0.30102999	8	0	1.07548421	0
chr7	135099056	135099115	60	1	4	1	0.02074938	0.30102999	8	0	0.79906872	0
chr7	135099115	135165201	66087	1	4	1	0.03812622	0.30102999	7	0	0.60763643	0
chr7	135165201	135165260	60	1	4	1	0.02074938	0.30102999	8	0	0.79906872	0
chr7	135165260	135308257	142998	4	4	1	0.03812622	0.30102999	7	0	0.60763643	0
chr7	135308257	135491891	183635	5	4	1	0.06713722	0.30102999	6	0	0.44141547	0
chr7	135491891	135491950	60	1	4	1	0.03812622	0.30102999	7	0	0.60763643	0
chr7	135491950	135739797	247848	8	4	1	0.06713722	0.30102999	6	0	0.44141547	0
chr7	135739797	135739856	60	1	4	1	0.03812622	0.30102999	7	0	0.60763643	0
chr7	135739856	135865434	125579	2	4	1	0.06713722	0.30102999	6	0	0.44141547	0
chr7	135865434	135865493	60	1	4	1	0.03812622	0.30102999	7	0	0.60763643	0
chr7	135865493	135923764	58272	1	4	1	0.06713722	0.30102999	6	0	0.44141547	0
chr7	135923764	136063988	140225	3	4	1	0.11390336	0.30102999	5	0	0.30102999	0
chr7	136063988	136092407	28420	1	3	1	0.05670724	0.30102999	5	0	0.45545077	0
chr7	136092407	136540747	448341	10	3	1	0.10122019	0.30102999	4	0	0.30102999	0
chr7	136540747	136540806	60	1	3	1	0.05670724	0.30102999	5	0	0.45545077	0
chr7	136540806	136699615	158810	4	2	1	0.02162467	0.30102999	5	0	0.68214471	0
chr7	136699615	136699674	60	1	3	1	0.05670724	0.30102999	5	0	0.45545077	0
chr7	136699674	136805859	106186	2	3	1	0.10122019	0.30102999	4	0	0.30102999	0
chr7	136805859	137302808	496950	11	3	1	0.05670724	0.30102999	5	0	0.45545077	0
chr7	137302808	137436005	133198	4	4	1	0.11390336	0.30102999	5	0	0.30102999	0
chr7	137436005	137544187	108183	2	3	1	0.05670724	0.30102999	5	0	0.45545077	0
chr7	137544187	137597659	53473	2	4	1	0.06713722	0.30102999	6	0	0.44141547	0
chr7	137597659	137597707	49	1	5	1	0.12309572	0.30102999	6	0	0.30102999	0
chr7	137597707	137645389	47683	1	4	1	0.11390336	0.30102999	5	0	0.30102999	0
chr7	137645389	137735357	89969	3	5	1	0.19510895	0.30102999	5	0	0.19510895	0
chr7	137735357	137878359	143003	4	5	1	0.12309572	0.30102999	6	0	0.30102999	0
chr7	137878359	137914662	36304	2	5	1	0.07511598	0.30102999	7	0	0.43181735	0
chr7	137914662	138080879	166218	4	5	2	0.07511598	0.61140001	7	0	0.43181735	0
chr7	138080879	138119953	39075	2	7	2	0.20469099	0.61140001	7	0	0.20469099	0
chr7	138119953	138290465	170513	7	7	2	0.13499366	0.61140001	8	0	0.30102999	0
chr7	138290465	138320582	30118	1	6	2	0.08122616	0.61140001	8	0	0.4250187	0
chr7	138320582	138453585	133004	2	6	2	0.129913	0.61140001	7	0	0.30102999	0
chr7	138453585	138471032	17448	2	6	2	0.04875589	0.61140001	9	0	0.5732208	0
chr7	138471032	138522005	50974	1	6	2	0.08122616	0.61140001	8	0	0.4250187	0
chr7	138522005	138588196	66192	1	5	2	0.07511598	0.61140001	7	0	0.43181735	0
chr7	138588196	138588255	60	1	5	2	0.04407651	0.61140001	8	0	0.58747015	0
chr7	138588255	138637247	48993	1	5	2	0.19510895	0.61140001	5	0	0.19510895	0
chr7	138637247	138637306	60	1	6	2	0.30102999	0.61140001	5	0	0.12309572	0
chr7	138637306	138677003	39698	1	5	2	0.19510895	0.61140001	5	0	0.19510895	0
chr7	138677003	138810389	133387	6	6	2	0.20064824	0.61140001	6	0	0.20064824	0
chr7	138810389	138810448	60	1	6	2	0.129913	0.61140001	7	0	0.30102999	0
chr7	138810448	138853229	42782	2	6	2	0.20064824	0.61140001	6	0	0.20064824	0
chr7	138853229	138992700	139472	4	5	2	0.12309572	0.61140001	6	0	0.30102999	0
chr7	138992700	138992759	60	1	5	2	0.07511598	0.61140001	7	0	0.43181735	0
chr7	138992759	139026872	34114	1	5	2	0.12309572	0.61140001	6	0	0.30102999	0
chr7	139026872	139195913	169042	5	5	2	0.19510895	0.61140001	5	0	0.19510895	0
chr7	139195913	139195972	60	1	5	3	0.19510895	0.93173516	5	0	0.19510895	0
chr7	139195972	139281037	85066	2	4	3	0.11390336	0.93173516	5	0	0.30102999	0
chr7	139281037	139433177	152141	4	5	3	0.19510895	0.93173516	5	0	0.19510895	0
chr7	139433177	139491890	58714	2	5	3	0.12309572	0.93173516	6	0	0.30102999	0
chr7	139491890	139614665	122776	3	5	3	0.19510895	0.93173516	5	0	0.19510895	0
chr7	139614665	139614724	60	1	5	3	0.12309572	0.93173516	6	0	0.30102999	0
chr7	139614724	139657476	42753	2	4	2	0.06713722	0.61140001	6	0	0.44141547	0
chr7	139657476	139657535	60	1	5	2	0.12309572	0.61140001	6	0	0.30102999	0
chr7	139657535	139724030	66496	1	5	2	0.19510895	0.61140001	5	0	0.19510895	0
chr7	139724030	139724089	60	1	6	2	0.30102999	0.61140001	5	0	0.12309572	0
chr7	139724089	139816977	92889	2	5	2	0.19510895	0.61140001	5	0	0.19510895	0
chr7	139816977	139966793	149817	3	5	2	0.30102999	0.61140001	4	0	0.11390336	0
chr7	139966793	139966852	60	1	5	2	0.19510895	0.61140001	5	0	0.19510895	0

chr7	139966852	140043326	76475	2	4	2	0.11390336	0.61140001	5	0	0.30102999	0
chr7	140043326	140043385	60	1	4	2	0.06713722	0.61140001	6	0	0.44141547	0
chr7	140043385	140234201	190817	5	4	2	0.11390336	0.61140001	5	0	0.30102999	0
chr7	140234201	140274928	40728	2	4	2	0.06713722	0.61140001	6	0	0.44141547	0
chr7	140274928	140306327	31400	1	3	2	0.03070643	0.61140001	6	0	0.63695542	0
chr7	140306327	140375777	69451	2	3	2	0.05670724	0.61140001	5	0	0.45545077	0
chr7	140375777	140453998	78222	4	3	2	0.03070643	0.30102999	6	1	0.63695542	0.05404976
chr7	140453998	140454057	60	1	3	2	0.01598258	0.30102999	7	1	0.84395715	0.05404976
chr7	140454057	140534481	80425	2	3	2	0.03070643	0.30102999	6	1	0.63695542	0.05404976
chr7	140534481	140620376	85896	3	4	2	0.06713722	0.1575501	6	2	0.44141547	0.1575501
chr7	140620376	140671659	51284	1	4	2	0.06713722	0.30102999	6	1	0.44141547	0.05404976
chr7	140671659	140740863	69205	2	4	1	0.06713722	0.1218695	6	1	0.44141547	0.1218695
chr7	140740863	140803904	63042	2	4	1	0.06713722	0.05404976	6	2	0.44141547	0.30102999
chr7	140803904	140869417	65514	1	4	1	0.06713722	0.1218695	6	1	0.44141547	0.1218695
chr7	140869417	140869476	60	1	5	1	0.12309572	0.1218695	6	1	0.30102999	0.1218695
chr7	140869476	140951427	81952	2	4	1	0.06713722	0.1218695	6	1	0.44141547	0.1218695
chr7	140951427	140951486	60	1	5	1	0.12309572	0.1218695	6	1	0.30102999	0.1218695
chr7	140951486	141405033	453548	10	4	1	0.06713722	0.1218695	6	1	0.44141547	0.1218695
chr7	141405033	141405092	60	1	5	1	0.12309572	0.1218695	6	1	0.30102999	0.1218695
chr7	141405092	141438657	33566	2	4	1	0.06713722	0.1218695	6	1	0.44141547	0.1218695
chr7	141438657	141464180	25524	3	5	1	0.12309572	0.1218695	6	1	0.30102999	0.1218695
chr7	141464180	141597935	133756	4	5	1	0.07511598	0.1218695	7	1	0.43181735	0.1218695
chr7	141597935	141730228	132294	4	5	1	0.07511598	0.05404976	7	2	0.43181735	0.30102999
chr7	141730228	141805616	75389	2	4	1	0.03812622	0.05404976	7	2	0.60763643	0.30102999
chr7	141805616	142041570	235955	6	5	1	0.07511598	0.05404976	7	2	0.43181735	0.30102999
chr7	142041570	142429393	387824	4	3	1	0.01598258	0.05404976	7	2	0.84395715	0.30102999
chr7	142429393	142487095	57703	2	3	1	0.03070643	0.05404976	6	2	0.63695542	0.30102999
chr7	142487095	142487154	60	1	3	2	0.03070643	0.1575501	6	2	0.63695542	0.1575501
chr7	142487154	142561592	74439	2	3	1	0.03070643	0.05404976	6	2	0.63695542	0.30102999
chr7	142561592	142580484	18893	2	3	2	0.03070643	0.1575501	6	2	0.63695542	0.1575501
chr7	142580484	142637276	56793	3	3	1	0.03070643	0.05404976	6	2	0.63695542	0.30102999
chr7	142637276	142763460	126185	4	3	2	0.01598258	0.1575501	7	2	0.84395715	0.1575501
chr7	142763460	142798038	34579	2	3	2	0.0079614	0.1575501	8	2	1.07548421	0.1575501
chr7	142798038	143175538	377501	19	3	2	0.01598258	0.1575501	7	2	0.84395715	0.1575501
chr7	143175538	143425418	249881	2	3	2	0.0079614	0.1575501	8	2	1.07548421	0.1575501
chr7	143425418	143426039	622	3	4	2	0.02074938	0.1575501	8	2	0.79906872	0.1575501
chr7	143426039	143626560	200522	2	4	2	0.03812622	0.1575501	7	2	0.60763643	0.1575501
chr7	143626560	143853032	226473	7	4	2	0.02074938	0.1575501	8	2	0.79906872	0.1575501
chr7	143853032	143952435	99404	1	4	2	0.03812622	0.1575501	7	2	0.60763643	0.1575501
chr7	143952435	144075261	122827	1	4	1	0.03812622	0.05404976	7	2	0.60763643	0.30102999
chr7	144075261	144134169	58909	2	4	1	0.02074938	0.05404976	8	2	0.79906872	0.30102999
chr7	144134169	144204173	70005	2	3	1	0.03070643	0.05404976	6	2	0.63695542	0.30102999
chr7	144204173	144245804	41632	1	2	1	0.01053319	0.05404976	6	2	0.91219088	0.30102999
chr7	144245804	144245863	60	1	2	3	0.01053319	0.30102999	6	2	0.91219088	0.08289318
chr7	144245863	144288503	42641	1	2	2	0.01053319	0.1575501	6	2	0.91219088	0.1575501
chr7	144288503	144340908	52406	2	3	2	0.03070643	0.1575501	6	2	0.63695542	0.1575501
chr7	144340908	144379972	39065	1	2	2	0.01053319	0.1575501	6	2	0.91219088	0.1575501
chr7	144379972	144419845	39874	2	2	3	0.01053319	0.30102999	6	2	0.91219088	0.08289318
chr7	144419845	144462961	43117	1	2	2	0.01053319	0.1575501	6	2	0.91219088	0.1575501
chr7	144462961	144520633	57673	2	2	2	0.00221948	0.1575501	8	2	1.44210395	0.1575501
chr7	144520633	144866746	346114	7	2	3	0.00221948	0.30102999	8	2	1.44210395	0.08289318
chr7	144866746	144947347	80602	2	1	3	3.39E-04	0.30102999	8	2	1.94674965	0.08289318
chr7	144947347	144947406	60	1	2	3	0.00221948	0.17593012	8	3	1.44210395	0.17593012
chr7	144947406	145026181	78776	1	1	3	3.39E-04	0.17593012	8	3	1.94674965	0.17593012
chr7	145026181	145026240	60	1	2	3	0.00221948	0.17593012	8	3	1.44210395	0.17593012
chr7	145026240	145064958	38719	1	2	3	0.00221948	0.30102999	8	2	1.44210395	0.08289318
chr7	145064958	145133696	68739	1	2	2	0.00221948	0.1575501	8	2	1.44210395	0.1575501
chr7	145133696	145178038	44343	1	1	2	3.39E-04	0.1575501	8	2	1.94674965	0.1575501
chr7	145178038	145208793	30756	2	1	3	3.39E-04	0.30102999	8	2	1.94674965	0.08289318
chr7	145208793	145349255	140463	3	1	3	8.47E-04	0.30102999	7	2	1.62048027	0.08289318
chr7	145349255	145397096	47842	2	1	3	8.47E-04	0.17593012	7	3	1.62048027	0.17593012
chr7	145397096	145525078	127983	3	1	3	0.00204627	0.17593012	6	3	1.31360226	0.17593012
chr7	145525078	145556874	31797	2	1	3	8.47E-04	0.17593012	7	3	1.62048027	0.17593012
chr7	145556874	145650396	93523	2	1	3	0.00204627	0.17593012	6	3	1.31360226	0.17593012
chr7	145650396	145650455	60	1	2	3	0.01053319	0.17593012	6	3	0.91219088	0.17593012
chr7	145650455	145726212	75758	2	2	3	0.02162467	0.17593012	5	3	0.68214471	0.17593012
chr7	145726212	145781576	55365	1	1	3	0.00478973	0.17593012	5	3	1.02643191	0.17593012
chr7	145781576	145824684	43109	2	1	3	0.00204627	0.17593012	6	3	1.31360226	0.17593012
chr7	145824684	145824743	60	1	1	4	0.00204627	0.30102999	6	3	1.31360226	0.10122019
chr7	145824743	145912108	87366	2	1	3	0.00204627	0.17593012	6	3	1.31360226	0.17593012
chr7	145912108	145971019	58912	2	1	3	8.47E-04	0.17593012	7	3	1.62048027	0.17593012
chr7	145971019	146017032	46014	2	1	3	3.39E-04	0.17593012	8	3	1.94674965	0.17593012
chr7	146017032	146017091	60	1	2	3	0.00221948	0.17593012	8	3	1.44210395	0.17593012
chr7	146017091	146236230	219140	4	1	3	3.39E-04	0.17593012	8	3	1.94674965	0.17593012
chr7	146236230	146333829	97600	3	2	3	0.00221948	0.17593012	8	3	1.44210395	0.17593012
chr7	146333829	146367408	33580	1	2	3	0.00493743	0.17593012	7	3	1.16581773	0.17593012
chr7	146367408	146451347	83940	2	2	3	0.01053319	0.17593012	6	3	0.91219088	0.17593012
chr7	146451347	146498949	47603	2	2	3	0.00493743	0.17593012	7	3	1.16581773	0.17593012
chr7	146498949	146587249	88301	2	2	3	0.01053319	0.17593012	6	3	0.91219088	0.17593012
chr7	146587249	146587308	60	1	2	3	0.00493743	0.17593012	7	3	1.16581773	0.17593012
chr7	146587308	146730472	143165	2	2	3	0.01053319	0.17593012	6	3	0.91219088	0.17593012
chr7	146730472	146763404	32933	2	2	3	0.00493743	0.17593012	7	3	1.16581773	0.17593012
chr7	146763404	146805297	41894	1	2	3	0.01053319	0.17593012	6	3	0.91219088	0.17593012
chr7	146805297	146981028	175732	4	1	3	0.00204627	0.17593012	6	3	1.31360226	0.17593012
chr7	146981028	147147025	165998	5	2	3	0.01053319	0.17593012	6	3	0.91219088	0.17593012
chr7	147147025	147292374	145350	3	2	3	0.02162467	0.17593012	5	3	0.68214471	0.17593012
chr7	147292374	147335706	43333	2	2	3	0.01053319	0.17593012	6	3	0.91219088	0.17593012
chr7	147335706	147366936	31231	2	2	3	0.00493743	0.17593012	7	3	1.16581773	0.17593012
chr7	147366936	147366995	60	1	2	3	0.00221948	0.17593012	8	3	1.44210395	0.17593012
chr7	147366995	147417054	50060	1	2	3	0.00493743	0.17593012	7	3	1.16581773	0.17593012
chr7	147417054	147449218	32165	1	2	3	0.01053319	0.17593012	6	3	0.91219088	0.17593012
chr7	147449218	147477663	28446	2	3	3	0.03070643	0.17593012	6	3	0.63695542	0.17593012
chr7	147477663	147521169	43507	2	3	3	0.01598258	0.17593012	7	3	0.84395715	0.17593012
chr7	147521169	147740163	218995	4	3	3	0.03070643	0.17593012	6	3	0.63695542	0.17593012
chr7	147740163	147740222	60	1	3	3	0.01598258	0.17593012	7	3	0.84395715	0.17593012
chr7	147740222	147847752	107531	2	3	2	0.01598258	0.08289318	7	3	0.84395715	0.30102999
chr7	147847752	147949695	101944	3	3	2	0.0079614	0.08289318	8	3	1.07548421	0.30102999
chr7	147949695	147949754	60	1	3	3	0.0079614	0.17593012	8	3	1.07548421	0.17593012
chr7	147949754	148023519	73766	1	3	3	0.01598258	0.17593012	7	3	0.84395715	0.17593012
chr7	148023519	148255537	232019	5	3	3	0.01598258	0.30102999	7	2	0.84395715	0.08289318
chr7	148255537	148293029	37493	2	3	3	0.0079614	0.30102999	8	2	1.07548421	0.08289318
chr7	148293029	148370007	76979	3	3	3	0.0079614	0.17593012	8	3	1.07548421	0.17593012
chr7	148370007	148427042	57036	1	3	3	0.0079614	0.30102999	8	2	1.07548421	0.08289318
chr7	148427042	148630241	203200	6	3	3	0.0079614	0.17593012	8	3	1.07548421	0.17593012
chr7	148630241	148630300	60	1	3	3	0.00378107	0.17593012	9	3	1.33111237	0.17593012
chr7	148630300	148700922	70623	1	3	3	0.0079614	0.17593012	8	3	1.07548421	0.17593012
chr7	148700922	148774870	73949	4	3	3	0.0079614	0.10122019	8	4	1.07548421	0.30102999
chr7	148774870	148779627	4758	2	3	3	0.00378107	0.10122019	9	4	1.33111237	0.30102999
chr7	148779627	149006885	227259	7	3	3	0.0079614	0.10122019	8	4	1.07548421	0.30102999
chr7	149006885	149062776	55892	1	3	3	0.01598258	0.10122019	7	4	0.84395715	0.30102999
chr7	149062776	149128443	65668	1	3	2	0.01598258	0.0429175	7	4	0.84395715	0.47744371
chr7	149128443	149128502	60	1	3	2	0.0079614	0.0429175	8	4	1.07548421	0.47744371
chr7	149128502	149248703	120202	4	3	2	0.01598258	0.0429175	7	4	0.84395715	0.47744371
chr7	149248703	149280479	31777	2	3	3	0.01598258	0.10122019	7	4	0.84395715	0.30102999
chr7	149280479	149330227	49749	1	3	3	0.03070643	0.10122019	6	4	0.63695542	0.30102999
chr7	149330227	149415472	85246	2	3	3	0.03070643	0.17593012	6	3	0.63695542	0.17593012
chr7	149415472	149668072	252601	7	3	3	0.03070643	0.10122019	6	4	0.63695542	0.30102999
chr7	149668072	149810321	142250	2	3	2	0.03070643	0.0429175	6	4	0.63695542	0.47744371
chr7	149810321	150499948	689628	21	3	3	0.03070643	0.10122019	6	4	0.63695542	0.30102999
chr7	150499948	150588506	88559	3	3	2	0.03070643	0.0429175	6	4	0.63695542	0.47744371
chr7	150588506	150588565	60	1	3	3	0.03070643	0.10122019	6	4	0.63695542	0.30102999
chr7	150588565	150655713	67149	2	3	2	0.03070643	0.0429175	6	4	0.63695542	0.47744371
chr7	150655713	150659831	4119	2	3	3	0.03070643	0.10122019	6	4	0.63695542	0.30102999
chr7	150659831	150754875	95045	5	3	2	0.03070643	0.0429175	6	4	0.63695542	0.47744371

chr7	150754875	150882916	128042	6	3	3	0.03070643	0.10122019	6	4	0.63695542	0.30102999
chr7	150882916	151042488	159573	4	3	2	0.05670724	0.0429175	5	4	0.45545077	0.47744371
chr7	151042488	151065059	22572	2	3	3	0.05670724	0.10122019	5	4	0.45545077	0.30102999
chr7	151065059	151385392	320334	6	3	3	0.05670724	0.17593012	5	3	0.45545077	0.17593012
chr7	151385392	151438034	52643	2	4	3	0.06713722	0.17593012	6	3	0.44141547	0.17593012
chr7	151438034	151573677	135644	4	3	3	0.03070643	0.17593012	6	3	0.63695542	0.17593012
chr7	151573677	151602419	28743	2	3	3	0.01598258	0.17593012	7	3	0.84395715	0.17593012
chr7	151602419	151602478	60	1	3	3	0.0079614	0.17593012	8	3	1.07548421	0.17593012
chr7	151602478	151711773	109296	4	3	3	0.01598258	0.17593012	7	3	0.84395715	0.17593012
chr7	151711773	151748853	37081	2	3	3	0.0079614	0.17593012	8	3	1.07548421	0.17593012
chr7	151748853	151748912	60	1	3	4	0.0079614	0.30102999	8	3	1.07548421	0.10122019
chr7	151748912	151776649	27738	1	3	4	0.03070643	0.30102999	6	3	0.63695542	0.10122019
chr7	151776649	151917504	140856	3	3	3	0.05670724	0.17593012	5	3	0.45545077	0.17593012
chr7	151917504	152008358	90855	2	3	2	0.05670724	0.08289318	5	3	0.45545077	0.30102999
chr7	152008358	152008417	60	1	3	3	0.03070643	0.17593012	6	3	0.63695542	0.17593012
chr7	152008417	152032715	24299	1	3	3	0.05670724	0.17593012	5	3	0.45545077	0.17593012
chr7	152032715	152351866	319152	4	2	3	0.02162467	0.17593012	5	3	0.68214471	0.17593012
chr7	152351866	152418291	66426	2	2	2	0.02162467	0.08289318	5	3	0.68214471	0.30102999
chr7	152418291	152516257	97967	3	2	2	0.0429175	0.08289318	4	3	0.47744371	0.30102999
chr7	152516257	152516316	60	1	2	2	0.02162467	0.08289318	5	3	0.68214471	0.30102999
chr7	152516316	152552113	35798	1	2	2	0.0429175	0.08289318	4	3	0.47744371	0.30102999
chr7	152552113	152552172	60	1	2	3	0.0429175	0.17593012	4	3	0.47744371	0.17593012
chr7	152552172	152765094	212923	2	2	2	0.0429175	0.08289318	4	3	0.47744371	0.30102999
chr7	152765094	152765153	60	1	2	3	0.0429175	0.17593012	4	3	0.47744371	0.17593012
chr7	152765153	152848370	83218	1	2	2	0.0429175	0.08289318	4	3	0.47744371	0.30102999
chr7	152848370	153021983	173614	1	1	2	0.01091641	0.08289318	4	3	0.76005302	0.30102999
chr7	153021983	153157388	135406	2	1	3	0.01091641	0.17593012	4	3	0.76005302	0.17593012
chr7	153157388	153157447	60	1	2	3	0.0429175	0.17593012	4	3	0.47744371	0.17593012
chr7	153157447	153569109	411663	2	1	3	0.01091641	0.17593012	4	3	0.76005302	0.17593012
chr7	153569109	153617970	48862	2	2	3	0.0429175	0.10122019	4	4	0.47744371	0.30102999
chr7	153617970	153618029	60	1	3	3	0.10122019	0.10122019	4	4	0.30102999	0.30102999
chr7	153618029	153761547	143519	4	2	3	0.0429175	0.10122019	4	4	0.47744371	0.30102999
chr7	153761547	153761606	60	1	3	3	0.10122019	0.10122019	4	4	0.30102999	0.30102999
chr7	153761606	153891336	129731	2	2	3	0.0429175	0.10122019	4	4	0.47744371	0.30102999
chr7	153891336	154263945	372610	10	3	3	0.10122019	0.10122019	4	4	0.30102999	0.30102999
chr7	154263945	154946297	682353	18	3	3	0.05670724	0.10122019	5	4	0.45545077	0.30102999
chr7	154946297	155008840	62544	1	3	3	0.10122019	0.10122019	4	4	0.30102999	0.30102999
chr7	155008840	155255740	246901	5	3	2	0.10122019	0.0429175	4	4	0.30102999	0.47744371
chr7	155255740	155257298	1559	3	3	3	0.10122019	0.10122019	4	4	0.30102999	0.30102999
chr7	155257298	155330437	73140	2	3	2	0.10122019	0.0429175	4	4	0.30102999	0.47744371
chr7	155330437	155330496	60	1	3	3	0.10122019	0.10122019	4	4	0.30102999	0.30102999
chr7	155330496	155707594	377099	8	3	2	0.10122019	0.0429175	4	4	0.30102999	0.47744371
chr7	155707594	155707653	60	1	3	2	0.05670724	0.0429175	5	4	0.45545077	0.47744371
chr7	155707653	155911833	204181	3	3	2	0.10122019	0.0429175	4	4	0.30102999	0.47744371
chr7	155911833	156052598	140766	4	3	2	0.03070643	0.0429175	6	4	0.63695542	0.47744371
chr7	156052598	156235636	183039	2	3	2	0.10122019	0.08289318	4	3	0.30102999	0.30102999
chr7	156235636	156407328	171693	2	2	2	0.0429175	0.08289318	4	3	0.47744371	0.30102999
chr7	156407328	156431093	23766	2	2	3	0.02162467	0.17593012	5	3	0.68214471	0.17593012
chr7	156431093	156475623	44531	4	2	3	0.02162467	0.10122019	5	4	0.68214471	0.30102999
chr7	156475623	156589139	113517	2	2	3	0.0429175	0.10122019	4	4	0.47744371	0.30102999
chr7	156589139	156589198	60	1	2	3	0.02162467	0.10122019	5	4	0.68214471	0.30102999
chr7	156589198	156684752	95555	2	2	3	0.0429175	0.10122019	4	4	0.47744371	0.30102999
chr7	156684752	156922311	237560	6	2	3	0.02162467	0.10122019	5	4	0.68214471	0.30102999
chr7	156922311	156969269	46959	2	3	3	0.05670724	0.10122019	5	4	0.45545077	0.30102999
chr7	156969269	157009703	40435	2	3	3	0.03070643	0.10122019	6	4	0.63695542	0.30102999
chr7	157009703	157133297	123595	3	3	3	0.05670724	0.10122019	5	4	0.45545077	0.30102999
chr7	157133297	157133356	60	1	5	3	0.12309572	0.10122019	6	4	0.30102999	0.30102999
chr7	157133356	157258597	125242	3	5	3	0.19510895	0.10122019	5	4	0.19510895	0.30102999
chr7	157258597	157319939	61343	1	5	2	0.19510895	0.0429175	5	4	0.19510895	0.47744371
chr7	157319939	157345353	25415	3	5	2	0.12309572	0.0429175	6	4	0.30102999	0.47744371
chr7	157345353	157422753	77401	4	5	3	0.12309572	0.10122019	6	4	0.30102999	0.30102999
chr7	157422753	157422812	60	1	5	3	0.12309572	0.05670724	6	5	0.30102999	0.45545077
chr7	157422812	157463498	40687	1	5	3	0.19510895	0.05670724	5	5	0.19510895	0.45545077
chr7	157463498	157598312	134815	4	5	3	0.19510895	0.03070643	5	6	0.19510895	0.63695542
chr7	157598312	157643650	45339	1	4	3	0.11390336	0.03070643	5	6	0.30102999	0.63695542
chr7	157643650	157875046	231397	5	2	3	0.02162467	0.03070643	5	6	0.68214471	0.63695542
chr7	157875046	157875105	60	1	4	3	0.11390336	0.03070643	5	6	0.30102999	0.63695542
chr7	157875105	158020297	145193	3	3	3	0.05670724	0.03070643	5	6	0.45545077	0.63695542
chr7	158020297	158110290	89994	3	3	4	0.05670724	0.06713722	5	6	0.45545077	0.44141547
chr7	158110290	158201462	91173	2	2	4	0.02162467	0.06713722	5	6	0.68214471	0.44141547
chr7	158201462	158269643	68182	2	2	4	0.02162467	0.03812622	5	7	0.68214471	0.60763643
chr7	158269643	158365342	95700	4	2	4	0.02162467	0.02074938	5	8	0.68214471	0.79906872
chr7	158365342	158365401	60	1	3	4	0.05670724	0.02074938	5	8	0.45545077	0.79906872
chr7	158365401	158599209	233809	4	3	3	0.05670724	0.03070643	5	6	0.45545077	0.63695542
chr7	158599209	158695253	96045	2	2	2	0.02162467	0.01053319	5	6	0.68214471	0.91219088
chr7	158695253	158738830	43578	1	2	2	0.0429175	0.02162467	4	5	0.47744371	0.68214471
chr7	158738830	158738889	60	1	3	2	0.05670724	0.02162467	5	5	0.45545077	0.68214471
chr7	158738889	158827238	88350	2	2	1	0.08289318	0.00478973	3	5	0.30102999	1.02643191
chr7	158827238	158861586	34349	2	2	1	0.0429175	0.00478973	4	5	0.47744371	1.02643191
chr7	158861586	158909738	48153	1	2	1	0.08289318	0.00478973	3	5	0.30102999	1.02643191
chr7	158909738	159088636	178899	1	2	1	0.1575501	0.05404976	2	2	0.1575501	0.30102999
chr8	221611	379418	157808	3	0	2	0	0.0429175	0	4	0	0.47744371
chr8	379418	442326	62909	2	0	3	0	0.0079614	0	8	0	1.07548421
chr8	442326	627655	185330	2	0	3	0	0.01598258	0	7	0	0.84395715
chr8	627655	714325	86671	2	0	6	0	0.129913	0	7	0	0.30102999
chr8	714325	714384	60	1	0	8	0	0.13872638	0	9	0	0.30102999
chr8	714384	770001	55618	1	0	8	0	0.20764654	0	8	0	0.20764654
chr8	770001	770060	60	1	1	9	0.30102999	0.20975986	0	9	0	0.20975986
chr8	770060	843383	73324	1	1	9	0.30102999	0.30102999	0	8	0	0.13872638
chr8	843383	977218	133836	2	1	9	0.30102999	0.20975986	0	9	0	0.20975986
chr8	977218	1603483	626266	11	1	9	0.30102999	0.09154957	0	11	0	0.41444892
chr8	1603483	1941854	338372	9	1	9	0.30102999	0.14135546	0	10	0	0.30102999
chr8	1941854	2088726	146873	4	1	9	0.30102999	0.09154957	0	11	0	0.41444892
chr8	2088726	2088785	60	1	1	11	0.30102999	0.21200206	0	11	0	0.21200206
chr8	2088785	2206158	117374	2	0	11	0	0.21200206	0	11	0	0.21200206
chr8	2206158	2206217	60	1	0	11	0	0.14385241	0	12	0	0.30102999
chr8	2206217	2308985	102769	1	0	11	0	0.21200206	0	11	0	0.21200206
chr8	2308985	2433783	124799	1	0	10	0	0.21118145	0	10	0	0.21118145
chr8	2433783	2559331	125549	2	0	9	0	0.14135546	0	10	0	0.30102999
chr8	2559331	2625411	66081	1	0	9	0	0.20975986	0	9	0	0.20975986
chr8	2625411	2625470	60	1	0	9	0	0.14135546	0	10	0	0.30102999
chr8	2625470	2794429	168960	2	0	9	0	0.20975986	0	9	0	0.20975986
chr8	2794429	2832422	37994	2	0	9	0	0.14135546	0	10	0	0.30102999
chr8	2832422	2832481	60	1	0	10	0	0.14303407	0	11	0	0.30102999
chr8	2832481	2966378	133898	2	0	10	0	0.30102999	0	9	0	0.14135546
chr8	2966378	2966437	60	1	0	10	0	0.14303407	0	11	0	0.30102999
chr8	2966437	3006264	39828	1	0	10	0	0.30102999	0	9	0	0.14135546
chr8	3006264	3122008	115745	2	0	9	0	0.20975986	0	9	0	0.20975986
chr8	3122008	3186077	64070	2	0	10	0	0.21118145	0	10	0	0.21118145
chr8	3186077	3276220	90144	2	0	8	0	0.20764654	0	8	0	0.20764654
chr8	3276220	3430348	154129	4	0	8	0	0.08923391	0	10	0	0.4167287
chr8	3430348	3513372	83025	2	0	8	0	0.13872638	0	9	0	0.30102999
chr8	3513372	3513431	60	1	0	9	0	0.14135546	0	10	0	0.30102999
chr8	3513431	3583303	69873	1	0	8	0	0.08923391	0	10	0	0.4167287
chr8	3583303	3583362	60	1	0	9	0	0.09154957	0	11	0	0.41444892
chr8	3583362	3636535	53174	1	0	8	0	0.05490675	0	11	0	0.55623409
chr8	3636535	3710810	74276	2	0	10	0	0.14303407	0	11	0	0.30102999
chr8	3710810	3754418	43609	2	0	11	0	0.21200206	0	11	0	0.21200206
chr8	3754418	3754477	60	1	0	11	0	0.14385241	0	12	0	0.30102999
chr8	3754477	3809137	54661	1	0	10	0	0.14303407	0	11	0	0.30102999
chr8	3809137	3908980	99844	2	0	9	0	0.20975986	0	9	0	0.20975986
chr8	3908980	3958726	49747	2	0	10	0	0.21118145	0	10	0	0.21118145
chr8	3958726	4173694	214969	4	0	9	0	0.20975986	0	9	0	0.20975986
chr8	4173694	4301326	127633	2	0	7	0	0.08584816	0	9	0	0.42015402

chr8	4301326	4301385	60	1	0	9	0	0.20975986	0	9	0	0.20975986
chr8	4301385	4660008	358624	7	0	8	0	0.13872638	0	9	0	0.30102999
chr8	4660008	4660067	60	1	0	8	0	0.08923391	0	10	0	0.4167287
chr8	4660067	4725168	65102	1	0	8	0	0.13872638	0	9	0	0.30102999
chr8	4725168	5232698	507531	7	0	6	0	0.04875589	0	9	0	0.5732208
chr8	5232698	5454489	221792	1	0	4	0	0.01077081	0	9	0	1.01542894
chr8	5454489	5721369	266881	2	0	3	0	0.01598258	0	7	0	0.84395715
chr8	5721369	5721428	60	1	1	4	0.30102999	0.03812622	0	7	0	0.60763643
chr8	5721428	5829649	108222	1	0	2	0	0.00493743	0	7	0	1.16581773
chr8	5829649	5921954	92306	2	0	3	0	0.01598258	0	7	0	0.84395715
chr8	5921954	5922013	60	1	0	4	0	0.03812622	0	7	0	0.60763643
chr8	5922013	6261022	339010	3	0	2	0	0.00493743	0	7	0	1.16581773
chr8	6261022	6550066	289045	8	0	4	0	0.01077081	0	9	0	1.01542894
chr8	6550066	6746478	196413	4	0	2	0	0.01053319	0	6	0	0.91219088
chr8	6746478	6914076	167599	3	0	2	0	0.02162467	0	5	0	0.68214471
chr8	8130630	8130689	60	1	0	2	0	0.08289318	1	3	0.30102999	0.30102999
chr8	8130689	8353662	222974	2	0	2	0	0.08289318	0	3	0	0.30102999
chr8	8353662	8353721	60	1	0	4	0	0.11390336	0	5	0	0.30102999
chr8	8353721	8557157	203437	2	0	3	0	0.10122019	0	4	0	0.30102999
chr8	8557157	8621658	64502	2	1	3	0.1218695	0.05670724	1	5	0.1218695	0.45545077
chr8	8621658	8684645	62988	2	1	4	0.1218695	0.01077081	1	9	0.1218695	1.01542894
chr8	8684645	8857854	173210	2	1	4	0.30102999	0.01077081	0	9	0	1.01542894
chr8	8857854	8891761	33908	2	2	5	0.61140001	0.02473314	0	9	0	0.76806864
chr8	8891761	9097498	205738	4	1	4	0.30102999	0.01077081	0	9	0	1.01542894
chr8	9097498	9261291	163794	3	1	4	0.30102999	0.00530919	0	10	0	1.2568129
chr8	9261291	9313799	52509	2	2	5	0.61140001	0.01320236	0	10	0	0.97390707
chr8	9313799	9372240	58442	1	1	5	0.30102999	0.01320236	0	10	0	0.97390707
chr8	9372240	9423529	51290	1	1	4	0.30102999	0.00530919	0	10	0	1.2568129
chr8	9423529	9423588	60	1	2	4	0.30102999	0.00530919	1	10	0.05404976	1.2568129
chr8	9423588	9556782	133195	2	2	4	0.61140001	0.00530919	0	10	0	1.2568129
chr8	9556782	9556841	60	1	2	6	0.61140001	0.02793176	0	10	0	0.74627054
chr8	9556841	9615475	58635	2	2	4	0.61140001	0.00530919	0	10	0	1.2568129
chr8	9615475	9638929	23455	2	2	6	0.61140001	0.02793176	0	10	0	0.74627054
chr8	9638929	9638988	60	1	2	6	0.30102999	0.02793176	1	10	0.05404976	0.74627054
chr8	9638988	9687615	48628	1	2	4	0.30102999	0.01077081	1	9	0.05404976	1.01542894
chr8	9687615	9730056	42442	2	2	5	0.30102999	0.02473314	1	9	0.05404976	0.76806864
chr8	9730056	9759684	29629	2	2	7	0.30102999	0.05232577	1	10	0.05404976	0.56314362
chr8	9759684	9819189	59506	2	2	7	0.61140001	0.08584816	0	9	0	0.42015402
chr8	9819189	9819248	60	1	2	7	0.61140001	0.03037338	0	11	0	0.73110763
chr8	9819248	9918050	98803	1	2	7	0.61140001	0.08584816	0	9	0	0.42015402
chr8	9918050	9992928	74879	1	2	6	0.61140001	0.04875589	0	9	0	0.5732208
chr8	9992928	10029423	36496	2	2	6	0.61140001	0.02793176	0	10	0	0.74627054
chr8	10029423	10065376	35954	2	2	8	0.61140001	0.08923391	0	10	0	0.4167287
chr8	10065376	10190619	125244	2	2	8	0.61140001	0.13872638	0	9	0	0.30102999
chr8	10190619	10358352	167734	4	2	8	0.61140001	0.08923391	0	10	0	0.4167287
chr8	10358352	11012211	653860	20	2	8	0.61140001	0.05490675	0	11	0	0.55623409
chr8	11012211	11374000	361790	14	2	7	0.61140001	0.03037338	0	11	0	0.73110763
chr8	11374000	11516619	142620	3	2	7	0.61140001	0.05232577	0	10	0	0.56314362
chr8	11516619	11567555	50937	2	2	7	0.61140001	0.03037338	0	11	0	0.73110763
chr8	11567555	11732948	165394	7	1	7	0.30102999	0.03037338	0	11	0	0.73110763
chr8	11732948	11841842	108895	2	1	6	0.30102999	0.02793176	0	10	0	0.74627054
chr8	11841842	11841901	60	1	1	7	0.30102999	0.03037338	0	11	0	0.73110763
chr8	11841901	12586472	744572	2	0	5	0	0.07511598	0	7	0	0.43181735
chr8	12586472	12824735	238264	3	0	5	0	0.19510895	0	5	0	0.19510895
chr8	12824735	12824794	60	1	0	5	0	0.12309572	0	6	0	0.30102999
chr8	12824794	12942604	117811	3	0	5	0	0.19510895	0	5	0	0.19510895
chr8	12942604	13083029	140426	3	0	5	0	0.30102999	0	4	0	0.11390336
chr8	13083029	13083088	60	1	0	6	0	0.30102999	0	5	0	0.12309572
chr8	13083088	13131825	48738	1	0	5	0	0.30102999	0	4	0	0.11390336
chr8	13131825	13162770	30946	2	0	6	0	0.30102999	0	5	0	0.12309572
chr8	13162770	13251151	88382	3	0	6	0	0.129913	0	7	0	0.30102999
chr8	13251151	13357172	106022	3	0	8	0	0.13872638	0	9	0	0.30102999
chr8	13357172	13357231	60	1	0	9	0	0.14135546	0	10	0	0.30102999
chr8	13357231	13558023	200793	3	0	9	0	0.20975986	0	9	0	0.20975986
chr8	13558023	13558082	60	1	0	9	0	0.20975986	1	9	0.30102999	0.20975986
chr8	13558082	13828325	270244	1	0	9	0	0.20975986	0	9	0	0.20975986
chr8	13828325	13947536	119212	2	0	10	0	0.14303407	0	11	0	0.30102999
chr8	13947536	13978884	31349	2	0	12	0	0.14385241	0	13	0	0.30102999
chr8	13978884	14062679	83796	2	0	11	0	0.09334429	0	13	0	0.41271556
chr8	14062679	14226231	163553	4	0	11	0	0.14385241	0	12	0	0.30102999
chr8	14226231	14308550	82320	2	0	10	0	0.09290028	0	12	0	0.41314172
chr8	14308550	14834145	525596	14	0	11	0	0.14385241	0	12	0	0.30102999
chr8	14834145	14921546	87402	2	0	11	0	0.21200206	0	11	0	0.21200206
chr8	14921546	15000092	78547	2	0	11	0	0.14385241	0	12	0	0.30102999
chr8	15000092	15093359	93268	4	0	11	0	0.09334429	0	13	0	0.41271556
chr8	15093359	15149147	55789	2	0	12	0	0.14385241	0	13	0	0.30102999
chr8	15149147	15480646	331500	3	0	9	0	0.0565833	0	12	0	0.55190077
chr8	15480646	15480705	60	1	0	10	0	0.09290028	0	12	0	0.41314172
chr8	15480705	15581080	100376	2	0	9	0	0.0565833	0	12	0	0.55190077
chr8	15581080	15610795	29716	2	0	9	0	0.0331093	0	13	0	0.71538971
chr8	15610795	15672380	61586	1	0	8	0	0.03209037	0	12	0	0.72109894
chr8	15672380	15966905	294526	2	0	8	0	0.20764654	0	8	0	0.20764654
chr8	15966905	16051358	84454	3	0	8	0	0.08923391	0	10	0	0.4167287
chr8	16051358	16051417	60	1	0	8	0	0.05490675	0	11	0	0.55623409
chr8	16051417	16100711	49295	1	0	8	0	0.08923391	0	10	0	0.4167287
chr8	16100711	16100770	60	1	0	11	0	0.21200206	0	11	0	0.21200206
chr8	16100770	16240968	140199	1	0	11	0	0.30102999	0	10	0	0.14303407
chr8	16240968	16241027	60	1	0	11	0	0.21200206	0	11	0	0.21200206
chr8	16241027	16416649	175623	1	0	9	0	0.20975986	0	9	0	0.20975986
chr8	16416649	16416708	60	1	0	9	0	0.09154957	0	11	0	0.41444892
chr8	16416708	16558744	142037	1	0	8	0	0.08923391	0	10	0	0.4167287
chr8	16558744	16706628	147885	2	0	8	0	0.05490675	0	11	0	0.55623409
chr8	16706628	16706687	60	1	0	9	0	0.0565833	0	12	0	0.55190077
chr8	16706687	16850815	144129	1	0	9	0	0.09154957	0	11	0	0.41444892
chr8	16850815	16935276	84462	2	0	8	0	0.05490675	0	11	0	0.55623409
chr8	16935276	16963022	27747	2	0	9	0	0.09154957	0	11	0	0.41444892
chr8	16963022	17183450	220429	6	0	8	0	0.05490675	0	11	0	0.55623409
chr8	17183450	17183509	60	1	0	9	0	0.09154957	0	11	0	0.41444892
chr8	17183509	17401992	218484	2	0	8	0	0.05490675	0	11	0	0.55623409
chr8	17401992	17629688	227697	9	0	9	0	0.09154957	0	11	0	0.41444892
chr8	17629688	17665600	35913	2	0	10	0	0.14303407	0	11	0	0.30102999
chr8	17665600	17721945	56346	1	0	9	0	0.09154957	0	11	0	0.41444892
chr8	17721945	17801244	79300	4	0	9	0	0.09154957	1	11	0.30102999	0.41444892
chr8	17801244	17919917	118674	4	0	10	0	0.14303407	1	11	0.30102999	0.30102999
chr8	17919917	18079493	159577	2	0	9	0	0.14135546	1	10	0.30102999	0.30102999
chr8	18079493	18079552	60	1	0	9	0	0.09154957	1	11	0.30102999	0.41444892
chr8	18079552	18331170	251619	3	0	8	0	0.05490675	1	11	0.30102999	0.55623409
chr8	18331170	18385511	54342	2	0	9	0	0.09154957	1	11	0.30102999	0.41444892
chr8	18385511	18417614	32104	1	0	9	0	0.14135546	1	10	0.30102999	0.30102999
chr8	18417614	18559625	142012	2	0	8	0	0.13872638	1	9	0.30102999	0.30102999
chr8	18559625	18559684	60	1	0	10	0	0.21118145	1	10	0.30102999	0.21118145
chr8	18559684	18604444	44761	1	0	9	0	0.14135546	1	10	0.30102999	0.30102999
chr8	18604444	18666293	61850	1	0	9	0	0.20975986	1	9	0.30102999	0.20975986
chr8	18666293	18719365	53073	1	0	8	0	0.13872638	1	9	0.30102999	0.30102999
chr8	18719365	18845845	126481	2	0	8	0	0.13872638	0	9	0	0.30102999
chr8	18845845	18880540	34696	2	0	9	0	0.20975986	0	9	0	0.20975986
chr8	18880540	19040050	159511	3	0	9	0	0.14135546	0	10	0	0.30102999
chr8	19040050	19412081	372032	9	0	9	0	0.09154957	0	11	0	0.41444892
chr8	19412081	19456271	44191	1	0	8	0	0.05490675	0	11	0	0.55623409
chr8	19456271	19456722	452	2	0	8	0	0.03209037	0	12	0	0.72109894
chr8	19456722	19591008	134287	4	0	9	0	0.0565833	0	12	0	0.55190077
chr8	19591008	19810898	219891	3	0	9	0	0.14135546	0	10	0	0.30102999
chr8	19810898	19810957	60	1	0	9	0	0.09154957	0	11	0	0.41444892
chr8	19810957	19873112	62156	1	0	8	0	0.08923391	0	10	0	0.4167287

chr8	19873112	20056388	183277	4	0	8	0	0.13872638	0	9	0	0.30102999
chr8	20056388	20165020	108633	4	0	9	0	0.20975986	0	9	0	0.20975986
chr8	20165020	20468341	303322	3	0	8	0	0.13872638	0	9	0	0.30102999
chr8	20468341	20690353	222013	1	0	8	0	0.20764654	0	8	0	0.20764654
chr8	20690353	20690412	60	1	0	8	0	0.13872638	0	9	0	0.30102999
chr8	20690412	20908353	217942	2	0	7	0	0.13499366	0	8	0	0.30102999
chr8	20908353	21132854	224502	2	0	8	0	0.08923391	0	10	0	0.4167287
chr8	21132854	21132913	60	1	0	9	0	0.09154957	0	11	0	0.41444892
chr8	21132913	21242166	109254	1	0	7	0	0.05232577	0	10	0	0.56314362
chr8	21242166	21493893	251728	2	0	6	0	0.02793176	0	10	0	0.74627054
chr8	21493893	21577614	83722	2	0	7	0	0.00859896	0	13	0	1.14735594
chr8	21577614	21820202	242589	6	0	8	0	0.00909834	0	14	0	1.13428373
chr8	21820202	21883863	63662	3	0	8	0	0.01767679	0	13	0	0.91308053
chr8	21883863	21883922	60	1	0	8	0	0.00909834	0	14	0	1.13428373
chr8	21883922	21901426	17505	1	0	8	0	0.01767679	0	13	0	0.91308053
chr8	21901426	21901478	53	1	0	9	0	0.01817691	0	14	0	0.90719478
chr8	21901478	21964615	63138	5	0	8	0	0.03209037	0	12	0	0.72109894
chr8	21964615	21964660	46	1	0	9	0	0.01817691	0	14	0	0.90719478
chr8	21964660	21995524	30865	2	0	9	0	0.0331093	0	13	0	0.71538971
chr8	21995524	22021055	25532	4	0	9	0	0.01817691	0	14	0	0.90719478
chr8	22021055	22087590	66536	2	0	9	0	0.0331093	0	13	0	0.71538971
chr8	22087590	22546205	458616	17	0	9	0	0.01817691	0	14	0	0.90719478
chr8	22546205	22607913	61709	2	0	9	0	0.0331093	0	13	0	0.71538971
chr8	22607913	22878680	270768	7	0	9	0	0.01817691	0	14	0	0.90719478
chr8	22878680	22878739	60	1	0	10	0	0.03344703	0	14	0	0.71353304
chr8	22878739	22959441	80703	3	0	9	0	0.01817691	0	14	0	0.90719478
chr8	22959441	22995148	35708	2	0	8	0	0.00909834	0	14	0	1.13428373
chr8	22995148	22995207	60	1	0	9	0	0.01817691	0	14	0	0.90719478
chr8	22995207	23081265	86059	3	0	7	0	0.00413023	0	14	0	1.39918918
chr8	23081265	23148333	67069	2	0	7	0	0.00859896	0	13	0	1.14735594
chr8	23148333	23289322	140990	4	0	7	0	0.00183109	0	15	0	1.68336308
chr8	23289322	23357497	68176	3	0	8	0	0.00433294	0	15	0	1.3873209
chr8	23357497	23427849	70353	3	0	9	0	0.00926608	0	15	0	1.13004284
chr8	23427849	23466216	38368	1	0	8	0	0.00433294	0	15	0	1.3873209
chr8	23466216	23539339	73124	1	0	6	0	0.00778066	0	12	0	1.17038931
chr8	23539339	23614962	75624	2	0	7	0	0.01667721	0	12	0	0.92532268
chr8	23614962	23643346	28385	2	0	8	0	0.00909834	0	14	0	1.13428373
chr8	23643346	23827988	184643	3	0	7	0	0.00859896	0	13	0	1.14735594
chr8	23827988	23828047	60	1	0	8	0	0.00909834	0	14	0	1.13428373
chr8	23828047	23947786	119740	1	0	8	0	0.05490675	0	11	0	0.55623409
chr8	23947786	24036657	88872	2	0	9	0	0.09154957	0	11	0	0.41444892
chr8	24036657	24036716	60	1	0	10	0	0.0574087	0	13	0	0.5498098
chr8	24036716	24168873	132158	2	0	8	0	0.05490675	0	11	0	0.55623409
chr8	24168873	24168932	60	1	0	9	0	0.09154957	0	11	0	0.41444892
chr8	24168932	24263232	94301	2	0	8	0	0.05490675	0	11	0	0.55623409
chr8	24263232	24304724	41493	2	0	9	0	0.09154957	0	11	0	0.41444892
chr8	24304724	24351287	46564	2	0	9	0	0.01817691	0	14	0	0.90719478
chr8	24351287	24351346	60	1	0	10	0	0.03344703	0	14	0	0.71353304
chr8	24351346	24384376	33031	1	0	10	0	0.0574087	0	13	0	0.5498098
chr8	24384376	24493086	108711	1	0	9	0	0.0565833	0	12	0	0.55190077
chr8	24493086	24610445	117360	2	0	9	0	0.0331093	0	13	0	0.71538971
chr8	24610445	24773595	163151	1	0	9	0	0.20975986	0	9	0	0.20975986
chr8	24773595	24811819	38225	4	0	9	0	0.14135546	0	10	0	0.30102999
chr8	24811819	24918107	106289	1	0	9	0	0.20975986	0	9	0	0.20975986
chr8	24918107	25190093	271987	5	0	9	0	0.0565833	0	12	0	0.55190077
chr8	25190093	25190152	60	1	0	10	0	0.09290028	0	12	0	0.41314172
chr8	25190152	25323072	132921	4	0	10	0	0.14303407	0	11	0	0.30102999
chr8	25323072	25323131	60	1	0	10	0	0.09290028	0	12	0	0.41314172
chr8	25323131	25364736	41606	2	0	9	0	0.0565833	0	12	0	0.55190077
chr8	25364736	25399721	34986	1	0	9	0	0.09154957	0	11	0	0.41444892
chr8	25399721	25482789	83069	2	0	9	0	0.14135546	0	10	0	0.30102999
chr8	25482789	25605849	123061	2	0	9	0	0.20975986	0	9	0	0.20975986
chr8	25605849	25745312	139464	4	0	10	0	0.09290028	0	12	0	0.41314172
chr8	25745312	25889490	144179	2	0	9	0	0.14135546	0	10	0	0.30102999
chr8	25889490	25889549	60	1	0	9	0	0.09154957	0	11	0	0.41444892
chr8	25889549	25995736	106188	2	0	9	0	0.14135546	0	10	0	0.30102999
chr8	25995736	26102739	107004	2	0	9	0	0.20975986	0	9	0	0.20975986
chr8	26102739	26102798	60	1	0	10	0	0.14303407	0	11	0	0.30102999
chr8	26102798	26162268	59471	1	0	9	0	0.09154957	0	11	0	0.41444892
chr8	26162268	26196470	34203	1	0	9	0	0.14135546	0	10	0	0.30102999
chr8	26196470	26196529	60	1	0	11	0	0.30102999	0	10	0	0.14303407
chr8	26196529	26265643	69115	2	0	10	0	0.21118145	0	10	0	0.21118145
chr8	26265643	26584168	318526	5	0	9	0	0.20975986	0	9	0	0.20975986
chr8	26584168	26584227	60	1	0	10	0	0.30102999	0	9	0	0.14135546
chr8	26584227	26655855	71629	2	0	8	0	0.13872638	0	9	0	0.30102999
chr8	26655855	26707917	52063	1	0	8	0	0.20764654	0	8	0	0.20764654
chr8	26707917	26707976	60	1	0	9	0	0.30102999	0	8	0	0.13872638
chr8	26707976	26876978	169003	2	0	9	0	0.42015402	0	7	0	0.08584816
chr8	26876978	26978519	101542	3	0	9	0	0.20975986	0	9	0	0.20975986
chr8	26978519	27040563	62045	1	0	9	0	0.30102999	0	8	0	0.13872638
chr8	27040563	27098086	57524	2	0	10	0	0.4167287	0	8	0	0.08923391
chr8	27098086	27143694	45609	1	0	9	0	0.42015402	0	7	0	0.08584816
chr8	27143694	27162783	19090	1	0	7	0	0.20469099	0	7	0	0.20469099
chr8	27162783	27227862	65080	3	0	7	0	0.13499366	0	8	0	0.30102999
chr8	27227862	27319856	91995	4	0	10	0	0.30102999	0	9	0	0.14135546
chr8	27319856	27402336	82481	2	0	9	0	0.20975986	0	9	0	0.20975986
chr8	27402336	27402395	60	1	0	10	0	0.30102999	0	9	0	0.14135546
chr8	27402395	27499756	97362	4	0	9	0	0.20975986	0	9	0	0.20975986
chr8	27499756	27530515	30760	1	0	8	0	0.13872638	0	9	0	0.30102999
chr8	27530515	27579235	48721	1	0	8	0	0.20764654	0	8	0	0.20764654
chr8	27579235	27614313	35079	2	0	8	0	0.20764654	1	8	0.30102999	0.20764654
chr8	27614313	27667902	53590	1	0	8	0	0.20764654	0	8	0	0.20764654
chr8	27667902	27667961	60	1	0	8	0	0.13872638	0	9	0	0.30102999
chr8	27667961	27711797	43837	2	0	8	0	0.20764654	0	8	0	0.20764654
chr8	27711797	27711856	60	1	0	10	0	0.4167287	0	8	0	0.08923391
chr8	27711856	27846302	134447	3	0	9	0	0.30102999	0	8	0	0.13872638
chr8	27846302	27886331	40030	2	0	10	0	0.4167287	0	8	0	0.08923391
chr8	27886331	27886390	60	1	0	10	0	0.30102999	0	9	0	0.14135546
chr8	27886390	28047665	161276	4	0	8	0	0.13872638	0	9	0	0.30102999
chr8	28047665	28240972	193308	7	0	8	0	0.20764654	0	8	0	0.20764654
chr8	28240972	28241031	60	1	0	9	0	0.30102999	0	8	0	0.13872638
chr8	28241031	28340940	99910	2	0	7	0	0.13499366	0	8	0	0.30102999
chr8	28340940	28421560	80621	3	0	8	0	0.20764654	0	8	0	0.20764654
chr8	28421560	28421619	60	1	0	9	0	0.30102999	0	8	0	0.13872638
chr8	28421619	28529407	107789	1	0	5	0	0.12309572	0	6	0	0.30102999
chr8	28529407	28611216	81810	2	0	4	0	0.06713722	0	6	0	0.44141547
chr8	28611216	28645371	34156	2	0	5	0	0.07511598	0	7	0	0.43181735
chr8	28645371	28645430	60	1	0	7	0	0.13499366	0	8	0	0.30102999
chr8	28645430	28671090	25661	1	0	6	0	0.08122616	0	8	0	0.4250187
chr8	28671090	28752645	81556	2	0	6	0	0.20064824	0	6	0	0.20064824
chr8	28752645	28752704	60	1	0	6	0	0.129913	0	7	0	0.30102999
chr8	28752704	28848846	96143	2	0	5	0	0.12309572	0	6	0	0.30102999
chr8	28848846	28906717	57872	1	0	4	0	0.06713722	0	6	0	0.44141547
chr8	28906717	28948689	41973	2	0	4	0	0.06713722	1	6	0.30102999	0.44141547
chr8	28948689	28948748	60	1	0	6	0	0.20064824	1	6	0.30102999	0.20064824
chr8	28948748	28981639	32892	1	0	5	0	0.12309572	1	6	0.30102999	0.30102999
chr8	28981639	29197146	215508	4	0	5	0	0.19510895	0	5	0	0.19510895
chr8	29197146	29243085	45940	1	0	4	0	0.18734596	0	4	0	0.18734596
chr8	29243085	29493828	250744	2	0	4	0	0.18734596	1	4	0.30102999	0.18734596
chr8	29493828	29596171	102344	2	0	6	0	0.30102999	1	5	0.30102999	0.12309572
chr8	29596171	29743350	147180	2	0	5	0	0.19510895	1	5	0.30102999	0.19510895
chr8	29743350	29743409	60	1	0	6	0	0.30102999	1	5	0.30102999	0.12309572
chr8	29743409	29923461	180053	2	0	5	0	0.19510895	1	5	0.30102999	0.19510895
chr8	29923461	30021700	98240	4	0	5	0	0.19510895	2	5	0.61140001	0.19510895

chr8	30021700	30066728	45029	1	0	5	0	0.19510895	1	5	0.30102999	0.19510895
chr8	30066728	30243957	177230	3	0	6	0	0.30102999	1	5	0.30102999	0.12309572
chr8	30243957	30469997	226041	8	0	8	0	0.58747015	1	5	0.30102999	0.04407651
chr8	30469997	30511086	41090	1	0	7	0	0.43181735	1	5	0.30102999	0.07511598
chr8	30511086	30567333	56248	3	0	8	0	0.58747015	1	5	0.30102999	0.04407651
chr8	30567333	30567389	57	1	0	8	0	0.4250187	1	6	0.30102999	0.08122616
chr8	30567389	30666890	99502	2	0	8	0	0.58747015	1	5	0.30102999	0.04407651
chr8	30666890	30853483	186594	4	0	8	0	0.30102999	1	7	0.30102999	0.13499366
chr8	30853483	30853542	60	1	0	9	0	0.42015402	1	7	0.30102999	0.08584816
chr8	30853542	30921916	68375	3	0	8	0	0.30102999	0	7	0	0.13499366
chr8	30921916	30921975	60	1	0	9	0	0.30102999	0	8	0	0.13872638
chr8	30921975	30973984	52010	1	0	9	0	0.42015402	0	7	0	0.08584816
chr8	30973984	31031059	57076	3	0	10	0	0.56314362	0	7	0	0.05232577
chr8	31031059	31158406	127348	2	0	12	0	0.72109894	1	8	0.30102999	0.03209037
chr8	31158406	31396934	238529	2	0	12	0	0.55190077	1	9	0.30102999	0.0565833
chr8	31396934	31396993	60	1	0	12	0	0.41314172	1	10	0.30102999	0.09290028
chr8	31396993	31497313	100321	2	0	11	0	0.30102999	1	10	0.30102999	0.14303407
chr8	31497313	31650428	153116	3	0	10	0	0.21118145	1	10	0.30102999	0.21118145
chr8	31650428	31787247	136820	4	0	11	0	0.30102999	1	10	0.30102999	0.14303407
chr8	31787247	31874423	87177	2	0	10	0	0.21118145	1	10	0.30102999	0.21118145
chr8	31874423	31874482	60	1	0	11	0	0.30102999	1	10	0.30102999	0.14303407
chr8	31874482	31920148	45667	1	0	10	0	0.21118145	1	10	0.30102999	0.21118145
chr8	31920148	31973643	53496	2	0	11	0	0.30102999	1	10	0.30102999	0.14303407
chr8	31973643	32005107	31465	1	0	10	0	0.30102999	1	9	0.30102999	0.14135546
chr8	32005107	32067028	61922	1	0	9	0	0.20975986	1	9	0.30102999	0.20975986
chr8	32067028	32067087	60	1	1	9	0.1218695	0.14135546	1	10	0.1218695	0.30102999
chr8	32067087	32132178	65092	1	1	9	0.1218695	0.20975986	1	9	0.1218695	0.20975986
chr8	32132178	32132237	60	1	1	10	0.1218695	0.30102999	1	9	0.1218695	0.14135546
chr8	32132237	32282892	150656	3	1	9	0.1218695	0.20975986	1	9	0.1218695	0.20975986
chr8	32282892	32282951	60	1	1	11	0.1218695	0.41444892	1	9	0.1218695	0.09154957
chr8	32282951	32380233	97283	2	1	10	0.1218695	0.30102999	1	9	0.1218695	0.14135546
chr8	32380233	32380292	60	1	1	10	0.1218695	0.21118145	1	10	0.1218695	0.21118145
chr8	32380292	32502397	122106	2	1	9	0.1218695	0.20975986	1	9	0.1218695	0.20975986
chr8	32502397	32543147	40751	2	1	9	0.1218695	0.14135546	1	10	0.1218695	0.30102999
chr8	32543147	32586023	42877	1	1	7	0.1218695	0.08584816	1	9	0.1218695	0.42015402
chr8	32586023	32666749	80727	1	1	5	0.1218695	0.02473314	1	9	0.1218695	0.76806864
chr8	32666749	32817154	150406	2	1	3	0.1218695	0.0079614	1	8	0.1218695	1.07548421
chr8	32817154	32859696	42543	2	1	3	0.05404976	0.0079614	2	8	0.30102999	1.07548421
chr8	32859696	32859755	60	1	1	3	0.05404976	0.00170589	2	10	0.30102999	1.61091002
chr8	32859755	32923289	63535	1	1	3	0.05404976	0.00378107	2	9	0.30102999	1.33111237
chr8	32923289	33119221	195933	1	1	3	0.1218695	0.01598258	1	7	0.1218695	0.84395715
chr8	33119221	33254022	134802	3	1	2	0.1218695	0.01053319	1	6	0.1218695	0.91219088
chr8	33254022	34639082	1385061	16	1	1	0.1218695	0.00478973	1	5	0.1218695	1.02643191
chr8	34639082	34714943	75862	2	1	5	0.1218695	0.07511598	1	7	0.1218695	0.43181735
chr8	34714943	34715002	60	1	1	6	0.1218695	0.129913	1	7	0.1218695	0.30102999
chr8	34715002	35167151	452150	5	1	5	0.1218695	0.12309572	1	6	0.1218695	0.30102999
chr8	35167151	35167210	60	1	1	7	0.1218695	0.13499366	1	8	0.1218695	0.30102999
chr8	35167210	35268882	101673	2	1	5	0.30102999	0.12309572	0	6	0	0.30102999
chr8	35268882	35410653	141772	4	1	7	0.1218695	0.13499366	1	8	0.1218695	0.30102999
chr8	35410653	35620127	209475	6	1	7	0.1218695	0.08584816	1	9	0.1218695	0.42015402
chr8	35620127	35698220	78094	1	1	7	0.1218695	0.13499366	1	8	0.1218695	0.30102999
chr8	35698220	35907864	209645	1	1	6	0.1218695	0.08122616	1	8	0.1218695	0.4250187
chr8	35907864	36014509	106646	2	2	9	0.30102999	0.20975986	1	9	0.05404976	0.20975986
chr8	36014509	36149008	134500	1	2	7	0.61140001	0.20469099	0	7	0	0.20469099
chr8	36149008	36361881	212874	2	2	5	0.61140001	0.07511598	0	7	0	0.43181735
chr8	36361881	36361940	60	1	2	7	0.61140001	0.08584816	0	9	0	0.42015402
chr8	36361940	36622844	260905	2	1	4	0.30102999	0.06713722	0	6	0	0.44141547
chr8	36622844	36622903	60	1	1	6	0.30102999	0.08122616	0	8	0	0.4250187
chr8	36622903	36703080	80178	2	1	3	0.30102999	0.05670724	0	5	0	0.45545077
chr8	36703080	36746755	43676	2	2	3	0.61140001	0.05670724	0	5	0	0.45545077
chr8	36746755	36746814	60	1	2	4	0.30102999	0.03812622	1	7	0.05404976	0.60763643
chr8	36746814	36854135	107322	2	2	3	0.30102999	0.03070643	1	6	0.05404976	0.63695542
chr8	36854135	36854194	60	1	3	4	0.30102999	0.06713722	2	6	0.08289318	0.44141547
chr8	36854194	37032208	178015	1	3	4	0.30102999	0.30102999	2	3	0.08289318	0.10122019
chr8	37032208	37228320	196113	2	4	4	0.47744371	0.30102999	2	3	0.0429175	0.10122019
chr8	37228320	37419308	190989	2	4	4	0.18734596	0.30102999	4	3	0.18734596	0.10122019
chr8	37419308	37538561	119254	2	5	4	0.30102999	0.30102999	4	3	0.11390336	0.10122019
chr8	37538561	37596744	58184	2	5	5	0.30102999	0.45545077	4	3	0.11390336	0.05670724
chr8	37596744	37627128	30385	2	5	5	0.30102999	0.30102999	4	4	0.11390336	0.11390336
chr8	37627128	37662934	35807	2	5	5	0.19510895	0.30102999	5	4	0.19510895	0.11390336
chr8	37662934	37727921	64988	2	5	3	0.19510895	0.10122019	5	4	0.19510895	0.30102999
chr8	37727921	37792736	64816	2	5	3	0.12309572	0.10122019	6	4	0.30102999	0.30102999
chr8	37792736	37827202	34467	2	5	2	0.12309572	0.0429175	6	4	0.30102999	0.47744371
chr8	37827202	37903880	76679	1	5	2	0.19510895	0.0429175	5	4	0.19510895	0.47744371
chr8	37903880	38061694	157815	7	5	4	0.12309572	0.18734596	6	4	0.30102999	0.18734596
chr8	38061694	38061753	60	1	5	4	0.07511598	0.18734596	7	4	0.43181735	0.18734596
chr8	38061753	38105379	43627	1	5	3	0.07511598	0.10122019	7	4	0.43181735	0.30102999
chr8	38105379	38133227	27849	5	6	3	0.129913	0.10122019	7	4	0.30102999	0.30102999
chr8	38133227	38157045	23819	1	5	3	0.07511598	0.10122019	7	4	0.43181735	0.30102999
chr8	38157045	38226918	69874	2	5	3	0.12309572	0.10122019	6	4	0.30102999	0.30102999
chr8	38226918	38298466	71549	3	5	3	0.19510895	0.10122019	5	4	0.19510895	0.30102999
chr8	38298466	38366279	67814	5	5	3	0.07511598	0.10122019	7	4	0.43181735	0.30102999
chr8	38366279	38430838	64560	2	5	3	0.07511598	0.05670724	7	5	0.43181735	0.45545077
chr8	38430838	38665576	234739	6	6	3	0.08122616	0.05670724	8	5	0.4250187	0.45545077
chr8	38665576	38764939	99364	3	6	4	0.08122616	0.11390336	8	5	0.4250187	0.30102999
chr8	38764939	38840455	75517	1	5	3	0.07511598	0.05670724	7	5	0.43181735	0.45545077
chr8	38840455	38899473	59019	7	5	4	0.07511598	0.11390336	7	5	0.43181735	0.30102999
chr8	38899473	38899532	60	1	5	4	0.04407651	0.11390336	8	5	0.58747015	0.30102999
chr8	38899532	39008931	109400	2	5	4	0.07511598	0.11390336	7	5	0.43181735	0.30102999
chr8	39008931	39008990	60	1	5	4	0.04407651	0.11390336	8	5	0.58747015	0.30102999
chr8	39008990	39068643	59654	1	5	4	0.07511598	0.11390336	7	5	0.43181735	0.30102999
chr8	39068643	39103677	35035	2	5	5	0.07511598	0.19510895	7	5	0.43181735	0.19510895
chr8	39103677	39309648	205972	5	5	5	0.12309572	0.19510895	6	5	0.30102999	0.19510895
chr8	39309648	39441924	132277	3	4	5	0.06713722	0.19510895	6	5	0.44141547	0.19510895
chr8	39441924	39494815	52892	2	4	4	0.06713722	0.11390336	6	5	0.44141547	0.30102999
chr8	39494815	39550120	55306	3	5	4	0.12309572	0.11390336	6	5	0.30102999	0.30102999
chr8	39550120	39550179	60	1	5	5	0.12309572	0.19510895	6	5	0.30102999	0.19510895
chr8	39550179	39581315	31137	1	5	4	0.12309572	0.11390336	6	5	0.30102999	0.30102999
chr8	39581315	39678598	97284	2	4	3	0.06713722	0.05670724	6	5	0.44141547	0.45545077
chr8	39678598	39678657	60	1	4	6	0.06713722	0.30102999	6	5	0.44141547	0.12309572
chr8	39678657	39718590	39934	1	4	5	0.06713722	0.19510895	6	5	0.44141547	0.19510895
chr8	39718590	39831828	113239	3	4	5	0.11390336	0.19510895	5	5	0.30102999	0.19510895
chr8	39831828	39899128	67301	2	3	4	0.17593012	0.18734596	3	4	0.17593012	0.18734596
chr8	39899128	39899187	60	1	3	4	0.17593012	0.11390336	3	5	0.17593012	0.30102999
chr8	39899187	40012313	113127	1	3	3	0.17593012	0.05670724	3	5	0.17593012	0.45545077
chr8	40012313	40012372	60	1	4	3	0.30102999	0.05670724	3	5	0.10122019	0.45545077
chr8	40012372	40089227	76856	1	4	3	0.30102999	0.10122019	3	4	0.10122019	0.30102999
chr8	40089227	40199169	109943	1	3	3	0.17593012	0.17593012	3	3	0.17593012	0.17593012
chr8	40199169	40384710	185542	1	3	2	0.17593012	0.1575501	3	2	0.17593012	0.1575501
chr8	40384710	40384769	60	1	3	3	0.10122019	0.17593012	4	3	0.30102999	0.17593012
chr8	40384769	40423721	38953	1	3	3	0.17593012	0.17593012	3	3	0.17593012	0.17593012
chr8	40423721	40456589	32869	2	3	4	0.17593012	0.30102999	3	3	0.17593012	0.10122019
chr8	40456589	40540260	83672	2	3	3	0.17593012	0.17593012	3	3	0.17593012	0.17593012
chr8	40540260	40580158	39899	2	3	3	0.17593012	0.05670724	3	5	0.17593012	0.45545077
chr8	40580158	40625263	45106	1	3	3	0.17593012	0.10122019	3	4	0.17593012	0.30102999
chr8	40625263	40760338	135076	2	3	3	0.17593012	0.17593012	3	3	0.17593012	0.17593012
chr8	40760338	40760397	60	1	3	3	0.10122019	0.17593012	4	3	0.30102999	0.17593012
chr8	40760397	40905973	145577	1	3	3	0.17593012	0.17593012	3	3	0.17593012	0.17593012
chr8	40905973	40906032	60	1	3	3	0.17593012	0.10122019	3	4	0.17593012	0.30102999
chr8	40906032	41113322	207291	1	3	3	0.17593012	0.30102999	3	2	0.17593012	0.08289318
chr8	41113322	41228429	115108	4	3	3	0.10122019	0.30102999	4	2	0.30102999	0.08289318
chr8	41228429	41345468	117040	3	3	3	0.05670724	0.30102999	5	2	0.45545077	0.08289318
chr8	41345468	41387646	42179	2	4	3	0.11390336	0.30102999	5	2	0.30102999	0.08289318
chr8	41387646	41387705	60	1	5	3	0.19510895	0.30102999	5	2	0.19510895	0.08289318

chr8	41387705	41559087	171383	7	4	3	0.11390336	0.30102999	5	2	0.30102999	0.08289318
chr8	41559087	41559138	52	1	5	3	0.19510895	0.30102999	5	2	0.19510895	0.08289318
chr8	41559138	41653582	94445	2	4	3	0.11390336	0.30102999	5	2	0.30102999	0.08289318
chr8	41653582	41653641	60	1	4	3	0.11390336	0.17593012	5	3	0.30102999	0.17593012
chr8	41653641	41690380	36740	1	4	3	0.11390336	0.30102999	5	2	0.30102999	0.08289318
chr8	41690380	41690439	60	1	4	3	0.06713722	0.30102999	6	2	0.44141547	0.08289318
chr8	41690439	41735639	45201	1	4	3	0.06713722	0.51676182	6	1	0.44141547	0.02438896
chr8	41735639	41735698	60	1	5	3	0.12309572	0.51676182	6	1	0.30102999	0.02438896
chr8	41735698	41792326	56629	1	4	3	0.06713722	0.51676182	6	1	0.44141547	0.02438896
chr8	41792326	41850544	58219	3	4	3	0.06713722	0.30102999	6	2	0.44141547	0.08289318
chr8	41850544	41850603	60	1	4	3	0.06713722	0.17593012	6	3	0.44141547	0.17593012
chr8	41850603	42056223	205621	5	4	3	0.06713722	0.30102999	6	2	0.44141547	0.08289318
chr8	42056223	42056282	60	1	5	3	0.12309572	0.30102999	6	2	0.30102999	0.08289318
chr8	47681335	47681394	60	1	4	0	0.11390336	0	5	0	0.30102999	0
chr8	47681394	47868796	187403	1	3	0	0.05670724	0	5	0	0.45545077	0
chr8	47868796	47948697	79902	2	3	1	0.03070643	0.30102999	6	0	0.63695542	0
chr8	47948697	47948756	60	1	5	1	0.12309572	0.30102999	6	0	0.30102999	0
chr8	47948756	48274815	326060	6	3	1	0.03070643	0.30102999	6	0	0.63695542	0
chr8	48274815	48487662	212848	5	4	1	0.06713722	0.30102999	6	0	0.44141547	0
chr8	48487662	48547114	59453	2	4	1	0.03812622	0.30102999	7	0	0.60763643	0
chr8	48547114	48962388	415275	14	4	1	0.01077081	0.30102999	9	0	1.01542894	0
chr8	48962388	49091300	128913	2	4	1	0.00247414	0.30102999	11	0	1.52371709	0
chr8	49091300	49091359	60	1	5	1	0.00317045	0.30102999	12	0	1.46403142	0
chr8	49091359	49330207	238849	2	4	1	0.00530919	0.30102999	10	0	1.2568129	0
chr8	49330207	49534174	203968	2	3	1	0.01598258	0.30102999	7	0	0.84395715	0
chr8	49534174	49623026	88853	1	3	1	0.03070643	0.30102999	6	0	0.63695542	0
chr8	49623026	49646409	23384	3	3	1	0.03070643	0.1218695	6	1	0.63695542	0.1218695
chr8	49646409	49830999	184591	1	3	1	0.10122019	0.1218695	4	1	0.30102999	0.1218695
chr8	49830999	49990465	159467	2	3	1	0.17593012	0.30102999	3	0	0.17593012	0
chr8	49990465	50253137	262673	2	2	1	0.08289318	0.30102999	3	0	0.30102999	0
chr8	50253137	50447771	194635	2	3	1	0.17593012	0.30102999	3	0	0.17593012	0
chr8	50447771	50968855	521085	8	3	1	0.17593012	0.1218695	3	1	0.17593012	0.1218695
chr8	50968855	51078336	109482	2	3	0	0.17593012	0	3	0	0.17593012	0
chr8	51078336	51245330	166995	4	3	1	0.17593012	0.1218695	3	1	0.17593012	0.1218695
chr8	51245330	51362233	116904	2	3	1	0.17593012	0.30102999	3	0	0.17593012	0
chr8	51362233	51395140	32908	2	3	1	0.17593012	0.1218695	3	1	0.17593012	0.1218695
chr8	51395140	51543169	148030	4	3	1	0.17593012	0.30102999	3	0	0.17593012	0
chr8	51543169	51640761	97593	3	3	1	0.10122019	0.30102999	4	0	0.30102999	0
chr8	51640761	52570948	930188	12	3	1	0.05670724	0.30102999	5	0	0.45545077	0
chr8	52570948	52672673	101726	3	3	1	0.03070643	0.30102999	6	0	0.63695542	0
chr8	52672673	52672732	60	1	4	1	0.03812622	0.30102999	7	0	0.60763643	0
chr8	52672732	52714573	41842	1	4	1	0.06713722	0.30102999	6	0	0.44141547	0
chr8	52714573	53024779	310207	7	5	1	0.12309572	0.30102999	6	0	0.30102999	0
chr8	53024779	53133343	108565	2	4	1	0.06713722	0.30102999	6	0	0.44141547	0
chr8	53133343	53273925	140583	5	5	1	0.12309572	0.30102999	6	0	0.30102999	0
chr8	53273925	53318890	44966	2	6	1	0.20064824	0.30102999	6	0	0.20064824	0
chr8	53318890	53363992	45103	1	5	1	0.12309572	0.30102999	6	0	0.30102999	0
chr8	53363992	53364051	60	1	5	1	0.07511598	0.30102999	7	0	0.43181735	0
chr8	53364051	53467396	103346	1	3	1	0.01598258	0.30102999	7	0	0.84395715	0
chr8	53467396	53607735	140340	4	3	1	0.03070643	0.30102999	6	0	0.63695542	0
chr8	53607735	53694659	86925	2	3	1	0.05670724	0.30102999	5	0	0.45545077	0
chr8	53694659	53694718	60	1	3	1	0.01598258	0.30102999	7	0	0.84395715	0
chr8	53694718	54068813	374096	2	3	0	0.05670724	0	5	0	0.45545077	0
chr8	54068813	54151519	82707	3	3	0	0.10122019	0	4	0	0.30102999	0
chr8	54151519	54237949	86431	1	2	0	0.0429175	0	4	0	0.47744371	0
chr8	54237949	54238008	60	1	2	0	0.01053319	0	6	0	0.91219088	0
chr8	54238008	54426040	188033	1	2	0	0.0429175	0	4	0	0.47744371	0
chr8	54426040	54628150	202111	3	3	0	0.10122019	0	4	0	0.30102999	0
chr8	54628150	55273058	644909	11	3	1	0.10122019	0.30102999	4	0	0.30102999	0
chr8	55273058	55372900	99843	4	3	1	0.05670724	0.30102999	5	0	0.45545077	0
chr8	55372900	55445546	72647	1	3	1	0.10122019	0.30102999	4	0	0.30102999	0
chr8	55445546	55538020	92475	3	3	1	0.05670724	0.30102999	5	0	0.45545077	0
chr8	55538020	55538079	60	1	3	1	0.03070643	0.30102999	6	0	0.63695542	0
chr8	55538079	55613593	75515	1	3	1	0.05670724	0.30102999	5	0	0.45545077	0
chr8	55613593	55613652	60	1	3	1	0.03070643	0.30102999	6	0	0.63695542	0
chr8	55613652	55994867	381216	3	3	1	0.10122019	0.30102999	4	0	0.30102999	0
chr8	55994867	56081496	86630	3	3	1	0.05670724	0.30102999	5	0	0.45545077	0
chr8	56081496	56147083	65588	2	4	1	0.11390336	0.30102999	5	0	0.30102999	0
chr8	56147083	56192971	45889	2	4	1	0.02074938	0.30102999	8	0	0.79906872	0
chr8	56192971	56383853	190883	4	4	1	0.03812622	0.30102999	7	0	0.60763643	0
chr8	56383853	56383912	60	1	4	1	0.02074938	0.30102999	8	0	0.79906872	0
chr8	56383912	56525071	141160	2	4	1	0.03812622	0.30102999	7	0	0.60763643	0
chr8	56525071	56525130	60	1	4	1	0.02074938	0.30102999	8	0	0.79906872	0
chr8	56525130	56607820	82691	1	4	1	0.03812622	0.30102999	7	0	0.60763643	0
chr8	56607820	56652014	44195	1	4	1	0.06713722	0.30102999	6	0	0.44141547	0
chr8	56652014	56776724	124711	5	4	1	0.02074938	0.30102999	8	0	0.79906872	0
chr8	56776724	56886235	109512	2	4	1	0.03812622	0.30102999	7	0	0.60763643	0
chr8	56886235	56922544	36310	1	4	1	0.11390336	0.30102999	5	0	0.30102999	0
chr8	56922544	56986184	63641	2	4	1	0.06713722	0.30102999	6	0	0.44141547	0
chr8	56986184	57025855	39672	1	4	0	0.11390336	0	5	0	0.30102999	0
chr8	57025855	57075931	50077	2	4	0	0.06713722	0	6	0	0.44141547	0
chr8	57075931	57229343	153413	5	4	0	0.03812622	0	7	0	0.60763643	0
chr8	57229343	57229402	60	1	4	0	0.02074938	0	8	0	0.79906872	0
chr8	57229402	57294135	64734	1	4	0	0.03812622	0	7	0	0.60763643	0
chr8	57294135	57414265	120131	3	4	0	0.06713722	0	6	0	0.44141547	0
chr8	57414265	57481595	67331	2	4	1	0.06713722	0.30102999	6	0	0.44141547	0
chr8	57481595	57903463	421869	5	4	0	0.06713722	0	6	0	0.44141547	0
chr8	57903463	57903522	60	1	5	1	0.12309572	0.30102999	6	0	0.30102999	0
chr8	57903522	58006167	102646	1	4	1	0.06713722	0.30102999	6	0	0.44141547	0
chr8	58006167	58006226	60	1	4	1	0.06713722	0.1218695	6	1	0.44141547	0.1218695
chr8	58006226	58049907	43682	1	4	0	0.06713722	0	6	1	0.44141547	0.30102999
chr8	58049907	58195802	145896	2	4	0	0.06713722	0	6	0	0.44141547	0
chr8	58195802	58244889	49088	3	4	1	0.06713722	0.30102999	6	0	0.44141547	0
chr8	58244889	58432828	187940	1	4	1	0.11390336	0.30102999	5	0	0.30102999	0
chr8	58432828	58832685	399858	3	4	1	0.18734596	0.30102999	4	0	0.18734596	0
chr8	58832685	58995874	163190	4	4	1	0.11390336	0.30102999	5	0	0.30102999	0
chr8	58995874	58995933	60	1	5	1	0.07511598	0.30102999	7	0	0.43181735	0
chr8	58995933	59043056	47124	2	4	1	0.06713722	0.30102999	6	0	0.44141547	0
chr8	59043056	59043115	60	1	5	1	0.12309572	0.30102999	6	0	0.30102999	0
chr8	59043115	59356817	313703	5	4	1	0.06713722	0.30102999	6	0	0.44141547	0
chr8	59356817	59718149	361333	7	4	1	0.11390336	0.30102999	5	0	0.30102999	0
chr8	59718149	59768370	50222	3	4	1	0.06713722	0.30102999	6	0	0.44141547	0
chr8	59768370	59851839	83470	2	4	1	0.11390336	0.30102999	5	0	0.30102999	0
chr8	59851839	59969281	117443	4	4	1	0.06713722	0.30102999	6	0	0.44141547	0
chr8	59969281	60017957	48677	1	4	1	0.11390336	0.30102999	5	0	0.30102999	0
chr8	60017957	60018016	60	1	5	1	0.19510895	0.30102999	5	0	0.19510895	0
chr8	60018016	60209502	191487	2	4	1	0.18734596	0.30102999	4	0	0.18734596	0
chr8	60209502	60209561	60	1	5	1	0.19510895	0.30102999	5	0	0.19510895	0
chr8	60209561	60306035	96475	1	4	1	0.11390336	0.30102999	5	0	0.30102999	0
chr8	60306035	60306094	60	1	5	1	0.19510895	0.30102999	5	0	0.19510895	0
chr8	60306094	60500981	194888	2	4	1	0.18734596	0.30102999	4	0	0.18734596	0
chr8	60500981	60637560	136580	2	5	1	0.19510895	0.30102999	5	0	0.19510895	0
chr8	60637560	60637619	60	1	6	1	0.30102999	0.1218695	5	1	0.12309572	0.1218695
chr8	60637619	60779958	142340	1	5	1	0.30102999	0.1218695	4	1	0.11390336	0.1218695
chr8	60779958	60953463	173506	1	4	1	0.18734596	0.1218695	4	1	0.18734596	0.1218695
chr8	60953463	60953522	60	1	4	2	0.18734596	0.30102999	4	1	0.18734596	0.05404976
chr8	60953522	61139456	185935	2	4	0	0.18734596	0	4	0	0.18734596	0
chr8	61139456	61178243	38788	3	4	0	0.11390336	0	5	0	0.30102999	0
chr8	61178243	61421463	243221	2	4	0	0.18734596	0	4	0	0.18734596	0
chr8	61421463	61421522	60	1	4	1	0.11390336	0.30102999	5	0	0.30102999	0
chr8	61421522	61605492	183971	4	4	1	0.30102999	0.30102999	3	0	0.10122019	0
chr8	61605492	61605551	60	1	4	1	0.18734596	0.30102999	4	0	0.18734596	0
chr8	61605551	61915189	309639	5	3	1	0.10122019	0.30102999	4	0	0.30102999	0
chr8	61915189	61915248	60	1	4	1	0.18734596	0.30102999	4	0	0.18734596	0

chr8	61915248	62121726	206479	2	3	1	0.10122019	0.30102999	4	0	0.30102999	0
chr8	62121726	62121785	60	1	4	1	0.18734596	0.30102999	4	0	0.18734596	0
chr8	62121785	62256117	134333	2	3	0	0.10122019	0	4	0	0.30102999	0
chr8	62256117	62370995	114879	4	4	1	0.18734596	0.30102999	4	0	0.18734596	0
chr8	62370995	62460604	89610	3	5	1	0.19510895	0.30102999	5	0	0.19510895	0
chr8	62460604	62460663	60	1	5	1	0.12309572	0.30102999	6	0	0.30102999	0
chr8	62460663	62505567	44905	1	5	0	0.12309572	0	6	0	0.30102999	0
chr8	62505567	62537287	31721	1	5	0	0.19510895	0	5	0	0.19510895	0
chr8	62537287	62580779	43493	2	5	1	0.19510895	0.30102999	5	0	0.19510895	0
chr8	62580779	62580832	54	1	5	1	0.12309572	0.30102999	6	0	0.30102999	0
chr8	62580832	62621940	41109	1	4	1	0.06713722	0.30102999	6	0	0.44141547	0
chr8	62621940	63072717	450778	3	4	1	0.30102999	0.30102999	3	0	0.10122019	0
chr8	63072717	63199019	126303	2	7	1	0.60763643	0.30102999	4	0	0.03812622	0
chr8	63199019	63199078	60	1	7	2	0.30102999	0.61140001	6	0	0.129913	0
chr8	63199078	63283467	84390	2	6	2	0.44141547	0.61140001	4	0	0.06713722	0
chr8	63283467	63338725	55259	2	7	2	0.43181735	0.61140001	5	0	0.07511598	0
chr8	63338725	63386986	48262	2	7	2	0.30102999	0.61140001	6	0	0.129913	0
chr8	63386986	63387045	60	1	7	2	0.20469099	0.61140001	7	0	0.20469099	0
chr8	63387045	63427135	40091	1	7	1	0.30102999	0.30102999	6	0	0.129913	0
chr8	63427135	63462169	35035	1	6	1	0.20064824	0.30102999	6	0	0.20064824	0
chr8	63462169	63462228	60	1	6	1	0.129913	0.30102999	7	0	0.30102999	0
chr8	63462228	63517033	54806	1	5	1	0.12309572	0.30102999	6	0	0.30102999	0
chr8	63517033	63569712	52680	2	5	2	0.12309572	0.61140001	6	0	0.30102999	0
chr8	63569712	63569771	60	1	5	2	0.12309572	0.30102999	6	1	0.30102999	0.05404976
chr8	63569771	63779676	209906	4	5	2	0.30102999	0.30102999	4	1	0.11390336	0.05404976
chr8	63779676	63779735	60	1	5	2	0.12309572	0.30102999	6	1	0.30102999	0.05404976
chr8	63779735	63903369	123635	4	5	2	0.30102999	0.30102999	4	1	0.11390336	0.05404976
chr8	63903369	64443494	540126	11	5	2	0.45545077	0.30102999	3	1	0.05670724	0.05404976
chr8	64443494	64443553	60	1	5	2	0.19510895	0.30102999	5	1	0.19510895	0.05404976
chr8	64443553	64507981	64429	1	5	2	0.30102999	0.30102999	4	1	0.11390336	0.05404976
chr8	64507981	64709711	201731	2	5	2	0.45545077	0.30102999	3	1	0.05670724	0.05404976
chr8	64709711	64709770	60	1	5	2	0.07511598	0.30102999	7	1	0.43181735	0.05404976
chr8	64709770	64921244	211475	1	5	2	0.30102999	0.30102999	4	1	0.11390336	0.05404976
chr8	64921244	65113979	192736	1	5	2	0.45545077	0.30102999	3	1	0.05670724	0.05404976
chr8	65113979	65114038	60	1	5	3	0.45545077	0.51676182	3	1	0.05670724	0.02438896
chr8	65114038	65275659	161622	2	4	2	0.47744371	0.30102999	2	1	0.0429175	0.05404976
chr8	65275659	65275718	60	1	5	3	0.45545077	0.51676182	3	1	0.05670724	0.02438896
chr8	65275718	65302085	26368	1	5	2	0.68214471	0.30102999	2	1	0.02162467	0.05404976
chr8	65302085	65302144	60	1	5	2	0.45545077	0.30102999	3	1	0.05670724	0.05404976
chr8	65302144	65427599	125456	1	5	1	0.45545077	0.1218695	3	1	0.05670724	0.1218695
chr8	65427599	65495515	67917	2	5	1	0.30102999	0.1218695	4	1	0.11390336	0.1218695
chr8	65495515	65555119	59605	2	4	1	0.30102999	0.1218695	3	1	0.10122019	0.1218695
chr8	65555119	65657261	102143	3	5	1	0.30102999	0.1218695	4	1	0.11390336	0.1218695
chr8	65657261	66231528	574268	5	5	1	0.19510895	0.1218695	5	1	0.19510895	0.1218695
chr8	66231528	66231587	60	1	5	1	0.07511598	0.1218695	7	1	0.43181735	0.1218695
chr8	66231587	66409867	178281	2	5	1	0.12309572	0.1218695	6	1	0.30102999	0.1218695
chr8	66409867	66515100	105234	3	5	1	0.04407651	0.1218695	8	1	0.58747015	0.1218695
chr8	66515100	66545979	30880	4	5	1	0.02473314	0.1218695	9	1	0.76806864	0.1218695
chr8	66545979	66582143	36165	1	5	1	0.04407651	0.1218695	8	1	0.58747015	0.1218695
chr8	66582143	66622671	40529	1	5	1	0.07511598	0.1218695	7	1	0.43181735	0.1218695
chr8	66622671	66647080	24410	3	5	1	0.04407651	0.1218695	8	1	0.58747015	0.1218695
chr8	66647080	66647139	60	1	5	1	0.02473314	0.1218695	9	1	0.76806864	0.1218695
chr8	66647139	66913877	266739	3	5	1	0.04407651	0.1218695	8	1	0.58747015	0.1218695
chr8	66913877	67012006	98130	3	5	1	0.01320236	0.1218695	10	1	0.97390707	0.1218695
chr8	67012006	67128015	116010	6	6	1	0.02793176	0.1218695	10	1	0.74627054	0.1218695
chr8	67128015	67128074	60	1	7	1	0.01667721	0.1218695	12	1	0.92532268	0.1218695
chr8	67128074	67296789	168716	1	6	1	0.00778066	0.1218695	12	1	1.17038931	0.1218695
chr8	67296789	67492176	195388	7	6	1	0.00373523	0.1218695	13	1	1.42397267	0.1218695
chr8	67492176	67492235	60	1	8	1	0.01767679	0.1218695	13	1	0.91308053	0.1218695
chr8	67492235	67543387	51153	2	7	1	0.01667721	0.1218695	12	1	0.92532268	0.1218695
chr8	67543387	67644481	101095	4	5	1	0.00666883	0.1218695	11	1	1.20557689	0.1218695
chr8	67644481	67644540	60	1	7	1	0.03037338	0.1218695	11	1	0.73110763	0.1218695
chr8	67644540	67684227	39688	1	6	1	0.01518174	0.1218695	11	1	0.9449689	0.1218695
chr8	67684227	67971446	287220	6	5	1	0.00666883	0.1218695	11	1	1.20557689	0.1218695
chr8	67971446	68074066	102621	4	7	2	0.03037338	0.30102999	11	1	0.73110763	0.05404976
chr8	68074066	68111201	37136	1	6	1	0.01518174	0.1218695	11	1	0.9449689	0.1218695
chr8	68111201	68299291	188091	4	5	1	0.00666883	0.1218695	11	1	1.20557689	0.1218695
chr8	68299291	68334426	35136	2	7	2	0.03037338	0.30102999	11	1	0.73110763	0.05404976
chr8	68334426	68454209	119784	2	6	2	0.01518174	0.30102999	11	1	0.9449689	0.05404976
chr8	68454209	68536490	82282	3	7	2	0.03037338	0.30102999	11	1	0.73110763	0.05404976
chr8	68536490	68595543	59054	1	7	1	0.05232577	0.1218695	10	1	0.56314362	0.1218695
chr8	68595543	68675428	79886	3	9	1	0.14135546	0.1218695	10	1	0.30102999	0.1218695
chr8	68675428	68675487	60	1	10	1	0.21118145	0.1218695	10	1	0.21118145	0.1218695
chr8	68675487	68900021	224535	2	8	1	0.13872638	0.1218695	9	1	0.30102999	0.1218695
chr8	68900021	68965391	65371	3	9	1	0.09154957	0.1218695	11	1	0.41444892	0.1218695
chr8	68965391	69050748	85358	3	7	1	0.08584816	0.1218695	9	1	0.42015402	0.1218695
chr8	69050748	69143736	92989	2	7	1	0.13499366	0.1218695	8	1	0.30102999	0.1218695
chr8	69143736	69392095	248360	6	7	1	0.08584816	0.1218695	9	1	0.42015402	0.1218695
chr8	69392095	69510342	118248	2	5	1	0.02473314	0.1218695	9	1	0.76806864	0.1218695
chr8	69510342	69552714	42373	2	6	1	0.04875589	0.1218695	9	1	0.5732208	0.1218695
chr8	69552714	69552773	60	1	8	1	0.08923391	0.1218695	10	1	0.4167287	0.1218695
chr8	69552773	69610616	57844	1	7	1	0.08584816	0.1218695	9	1	0.42015402	0.1218695
chr8	69610616	69680919	70304	2	5	1	0.02473314	0.1218695	9	1	0.76806864	0.1218695
chr8	69680919	69730931	50013	2	6	1	0.04875589	0.1218695	9	1	0.5732208	0.1218695
chr8	69730931	69836784	105854	1	5	1	0.02473314	0.1218695	9	1	0.76806864	0.1218695
chr8	69836784	69867283	30500	2	5	1	0.01320236	0.1218695	10	1	0.97390707	0.1218695
chr8	69867283	70029644	162362	2	5	1	0.04407651	0.1218695	8	1	0.58747015	0.1218695
chr8	70029644	70106192	76549	1	5	1	0.12309572	0.30102999	6	0	0.30102999	0
chr8	70106192	70279598	173407	1	5	0	0.19510895	0	5	0	0.19510895	0
chr8	70279598	70279656	59	1	5	0	0.12309572	0	6	0	0.30102999	0
chr8	70279656	70441544	161889	2	5	0	0.19510895	0	5	0	0.19510895	0
chr8	70441544	70498689	57146	2	5	0	0.12309572	0	6	0	0.30102999	0
chr8	70498689	70498748	60	1	5	0	0.12309572	0	6	1	0.30102999	0.30102999
chr8	70498748	70594464	95717	2	5	0	0.30102999	0	4	1	0.11390336	0.30102999
chr8	70594464	70594523	60	1	5	1	0.19510895	0.1218695	5	1	0.19510895	0.1218695
chr8	70594523	70973880	379358	6	5	0	0.30102999	0	4	1	0.11390336	0.30102999
chr8	70973880	71024709	50830	2	5	0	0.19510895	0	5	1	0.19510895	0.30102999
chr8	71024709	71070488	45780	1	4	0	0.18734596	0	4	1	0.18734596	0.30102999
chr8	71070488	71168325	97838	3	4	1	0.18734596	0.1218695	4	1	0.18734596	0.1218695
chr8	71168325	71208972	40648	1	4	0	0.18734596	0	4	1	0.18734596	0.30102999
chr8	71208972	71580594	371623	9	5	0	0.12309572	0	6	1	0.30102999	0.30102999
chr8	71580594	71777173	196580	3	5	0	0.30102999	0	4	1	0.11390336	0.30102999
chr8	71777173	71973754	196582	1	5	0	0.30102999	0	4	0	0.11390336	0
chr8	71973754	72110296	136543	2	6	0	0.20064824	0	6	0	0.20064824	0
chr8	72110296	72110355	60	1	6	0	0.08122616	0	8	0	0.4250187	0
chr8	72110355	72268801	158447	3	6	0	0.20064824	0	6	0	0.20064824	0
chr8	72268801	72340784	71984	2	6	0	0.08122616	0	8	0	0.4250187	0
chr8	72340784	72468057	127274	3	7	0	0.08584816	0	9	0	0.42015402	0
chr8	72468057	72694454	226398	1	7	0	0.20469099	0	7	0	0.20469099	0
chr8	72694454	72754973	60520	2	7	0	0.13499366	0	8	0	0.30102999	0
chr8	72754973	72755471	499	2	8	0	0.08923391	0	10	0	0.4167287	0
chr8	72755471	72823573	68103	1	7	0	0.08584816	0	9	0	0.42015402	0
chr8	72823573	72870665	47093	1	7	0	0.20469099	0	7	0	0.20469099	0
chr8	72870665	72958765	88101	1	7	0	0.30102999	0	6	0	0.129913	0
chr8	72958765	73019805	61041	2	7	0	0.13499366	0	8	0	0.30102999	0
chr8	73019805	73019864	60	1	7	0	0.08584816	0	9	1	0.42015402	0.30102999
chr8	73019864	73198685	178822	1	6	0	0.04875589	0	9	1	0.5732208	0.30102999
chr8	73198685	73423429	224745	3	6	0	0.08122616	0	8	0	0.4250187	0
chr8	73423429	73480220	56792	1	5	0	0.04407651	0	8	0	0.58747015	0
chr8	73480220	73596927	116708	3	5	0	0.02473314	0	9	1	0.76806864	0.30102999
chr8	73596927	73596986	60	1	6	0	0.04875589	0	9	1	0.5732208	0.30102999
chr8	73596986	73726809	129824	3	6	0	0.04875589	0	9	0	0.5732208	0
chr8	73726809	73762081	35273	1	5	0	0.02473314	0	9	0	0.76806864	0
chr8	73762081	73850463	88383	3	5	0	0.01320236	0	10	1	0.97390707	0.30102999

chr8	73850463	73925241	74779	1	5	0	0.01320236	0	10	0	0.97390707	0
chr8	73925241	73982182	56942	3	7	0	0.05232577	0	10	0	0.56314362	0
chr8	73982182	74203225	221044	6	7	0	0.05232577	0	10	1	0.56314362	0.30102999
chr8	74203225	74203282	58	1	7	0	0.01667721	0	12	1	0.92532268	0.30102999
chr8	74203282	74214226	10945	1	6	0	0.01518174	0	11	1	0.9449689	0.30102999
chr8	74214226	74226543	12318	2	7	0	0.03037338	0	11	1	0.73110763	0.30102999
chr8	74226543	74325115	98573	1	6	0	0.01518174	0	11	1	0.9449689	0.30102999
chr8	74325115	74565684	240570	6	6	0	0.00778066	0	12	1	1.17038931	0.30102999
chr8	74565684	74603322	37639	2	6	0	0.00373523	0	13	1	1.42397267	0.30102999
chr8	74603322	74654966	51645	1	6	0	0.00778066	0	12	1	1.17038931	0.30102999
chr8	74654966	74731758	76793	2	6	0	0.01518174	0	11	0	0.9449689	0
chr8	74731758	74731817	60	1	6	0	0.00778066	0	12	0	1.17038931	0
chr8	74731817	74789791	57975	1	6	0	0.02793176	0	10	0	0.74627054	0
chr8	74789791	74860448	70658	2	6	0	0.01518174	0	11	0	0.9449689	0
chr8	74860448	74893709	33262	3	6	0	0.00778066	0	12	0	1.17038931	0
chr8	74893709	75147258	253550	4	6	0	0.01518174	0	11	0	0.9449689	0
chr8	75147258	75210473	63216	4	6	0	0.00778066	0	12	0	1.17038931	0
chr8	75210473	75397018	186546	3	6	0	0.08122616	0	8	0	0.4250187	0
chr8	75397018	75586021	189004	2	6	0	0.04875589	0	9	0	0.5732208	0
chr8	75586021	75586080	60	1	6	0	0.02793176	0	10	0	0.74627054	0
chr8	75586080	75878199	292120	4	6	0	0.08122616	0	8	0	0.4250187	0
chr8	75878199	75878258	60	1	6	0	0.04875589	0	9	0	0.5732208	0
chr8	75878258	75989752	111495	2	6	0	0.08122616	0	8	0	0.4250187	0
chr8	75989752	75989811	60	1	6	0	0.04875589	0	9	0	0.5732208	0
chr8	75989811	76069530	79720	1	6	0	0.08122616	0	8	0	0.4250187	0
chr8	76069530	76149281	79752	1	6	0	0.129913	0	7	0	0.30102999	0
chr8	76149281	76149340	60	1	6	0	0.08122616	0	8	0	0.4250187	0
chr8	76149340	76564258	414919	7	6	0	0.129913	0	7	0	0.30102999	0
chr8	76564258	76564317	60	1	7	0	0.20469099	0	7	0	0.20469099	0
chr8	76564317	76687035	122719	1	7	0	0.30102999	0	6	0	0.129913	0
chr8	76687035	76794376	107342	2	7	0	0.20469099	0	7	1	0.20469099	0.30102999
chr8	76794376	76941461	147086	2	8	0	0.30102999	0	7	1	0.13499366	0.30102999
chr8	76941461	76941520	60	1	8	0	0.20764654	0	8	1	0.20764654	0.30102999
chr8	76941520	77119820	178301	2	8	0	0.30102999	0	7	1	0.13499366	0.30102999
chr8	77119820	77119879	60	1	8	0	0.13872638	0	9	1	0.30102999	0.30102999
chr8	77119879	77376307	256429	2	8	0	0.30102999	0	7	1	0.13499366	0.30102999
chr8	77376307	77376366	60	1	8	0	0.13872638	0	9	1	0.30102999	0.30102999
chr8	77376366	77648477	272112	4	8	0	0.30102999	0	7	1	0.13499366	0.30102999
chr8	77648477	78075125	426649	7	8	0	0.4250187	0	6	1	0.08122616	0.30102999
chr8	78075125	78357218	282094	1	6	0	0.20064824	0	6	1	0.20064824	0.30102999
chr8	78357218	78372122	14905	2	7	0	0.13499366	0	8	1	0.30102999	0.30102999
chr8	78372122	78639744	267623	1	6	0	0.20064824	0	6	0	0.20064824	0
chr8	78639744	78961227	321484	1	5	0	0.19510895	0	5	0	0.19510895	0
chr8	78961227	78961286	60	1	5	0	0.12309572	0	6	0	0.30102999	0
chr8	78961286	79434843	473558	4	5	0	0.19510895	0	5	0	0.19510895	0
chr8	79434843	79514785	79943	2	5	0	0.30102999	0	4	0	0.11390336	0
chr8	79514785	79645911	131127	2	4	0	0.18734596	0	4	0	0.18734596	0
chr8	79645911	79763966	118056	3	4	0	0.03812622	0	7	0	0.60763643	0
chr8	79763966	79967045	203080	2	7	0	0.08584816	0	9	0	0.42015402	0
chr8	79967045	80082254	115210	2	8	0	0.08923391	0	10	0	0.4167287	0
chr8	80082254	80440116	357863	3	8	0	0.05490675	0	11	0	0.55623409	0
chr8	80440116	80559960	119845	2	9	0	0.0565833	0	12	0	0.55190077	0
chr8	80559960	80560019	60	1	10	0	0.0574087	0	13	0	0.5498098	0
chr8	80560019	80892766	332748	5	10	0	0.14303407	0	11	0	0.30102999	0
chr8	80892766	80892825	60	1	10	0	0.0574087	0	13	0	0.5498098	0
chr8	80892825	80948496	55672	1	9	0	0.0331093	0	13	0	0.71538971	0
chr8	80948496	80992615	44120	1	9	0	0.09154957	0	11	0	0.41444892	0
chr8	80992615	81043036	50422	3	10	0	0.0574087	0	13	0	0.5498098	0
chr8	81043036	81070358	27323	2	10	1	0.03344703	0.30102999	14	0	0.71353304	0
chr8	81070358	81198379	128022	2	10	1	0.09290028	0.30102999	12	0	0.41314172	0
chr8	81198379	81198438	60	1	10	1	0.0574087	0.30102999	13	0	0.5498098	0
chr8	81198438	81422563	224126	2	10	1	0.09290028	0.30102999	12	0	0.41314172	0
chr8	81422563	81422622	60	1	10	1	0.03344703	0.1218695	14	1	0.71353304	0.1218695
chr8	81422622	81432974	10353	2	10	1	0.0574087	0.1218695	13	1	0.5498098	0.1218695
chr8	81432974	81669008	236035	4	10	0	0.0574087	0	13	1	0.5498098	0.30102999
chr8	81669008	81720620	51613	1	10	0	0.09290028	0	12	1	0.41314172	0.30102999
chr8	81720620	81855024	134405	3	10	1	0.09290028	0.1218695	12	1	0.41314172	0.1218695
chr8	81855024	81888045	33022	1	10	1	0.14303407	0.1218695	11	1	0.30102999	0.1218695
chr8	81888045	81925288	37244	1	10	0	0.14303407	0	11	1	0.30102999	0.30102999
chr8	81925288	82019092	93805	4	10	0	0.09290028	0	12	1	0.41314172	0.30102999
chr8	82019092	82019151	60	1	10	1	0.09290028	0.1218695	12	1	0.41314172	0.1218695
chr8	82019151	82193925	174775	2	9	0	0.0565833	0	12	1	0.55190077	0.30102999
chr8	82193925	82197238	3314	2	10	1	0.09290028	0.1218695	12	1	0.41314172	0.1218695
chr8	82197238	82392629	195392	3	9	1	0.0565833	0.1218695	12	1	0.55190077	0.1218695
chr8	82392629	82427223	34595	1	9	0	0.0565833	0	12	1	0.55190077	0.30102999
chr8	82427223	82570356	143134	2	9	0	0.09154957	0	11	1	0.41444892	0.30102999
chr8	82570356	82646435	76080	3	9	0	0.0565833	0	12	1	0.55190077	0.30102999
chr8	82646435	82752514	106080	3	9	0	0.09154957	0	11	1	0.41444892	0.30102999
chr8	82752514	82799697	47184	1	9	0	0.14135546	0	10	1	0.30102999	0.30102999
chr8	82799697	83157905	358209	2	7	0	0.20469099	0	7	1	0.20469099	0.30102999
chr8	83157905	83157964	60	1	9	0	0.30102999	0	8	1	0.13872638	0.30102999
chr8	83157964	83365015	207052	2	6	0	0.129913	0	7	1	0.30102999	0.30102999
chr8	83365015	83591368	226354	1	4	0	0.11390336	0	5	0	0.30102999	0
chr8	83591368	83756025	164658	1	4	0	0.18734596	0	4	0	0.18734596	0
chr8	83756025	83756084	60	1	4	0	0.11390336	0	5	0	0.30102999	0
chr8	83756084	84280819	524736	3	4	0	0.18734596	0	4	0	0.18734596	0
chr8	84280819	84772109	491291	4	5	0	0.19510895	0	5	1	0.19510895	0.30102999
chr8	84772109	85022127	250019	3	7	0	0.13499366	0	8	1	0.30102999	0.30102999
chr8	85022127	85197315	175189	4	8	0	0.13872638	0	9	1	0.30102999	0.30102999
chr8	85197315	85229365	32051	2	8	0	0.05490675	0	11	1	0.55623409	0.30102999
chr8	85229365	85409009	179645	3	8	0	0.13872638	0	9	1	0.30102999	0.30102999
chr8	85409009	85521751	112743	3	9	0	0.20975986	0	9	1	0.20975986	0.30102999
chr8	85521751	85521810	60	1	9	1	0.14135546	0.1218695	10	1	0.30102999	0.1218695
chr8	85521810	85750950	229141	4	9	0	0.20975986	0	9	1	0.20975986	0.30102999
chr8	85750950	85833804	82855	2	9	0	0.30102999	0	8	1	0.13872638	0.30102999
chr8	85833804	85888656	54853	1	7	0	0.13499366	0	8	1	0.30102999	0.30102999
chr8	85888656	86134206	245551	7	7	0	0.08584816	0	9	1	0.42015402	0.30102999
chr8	86134206	86168328	34123	2	7	1	0.03037338	0.1218695	11	1	0.73110763	0.1218695
chr8	86168328	86393161	224834	7	7	0	0.03037338	0	11	1	0.73110763	0.30102999
chr8	86393161	86467090	73930	1	7	0	0.05232577	0	10	1	0.56314362	0.30102999
chr8	86467090	86467149	60	1	7	1	0.05232577	0.1218695	10	1	0.56314362	0.1218695
chr8	86467149	86842195	375047	1	7	1	0.08584816	0.1218695	9	1	0.42015402	0.1218695
chr8	86842195	87067106	224912	3	8	1	0.13872638	0.1218695	9	1	0.30102999	0.1218695
chr8	87067106	87067165	60	1	8	1	0.08923391	0.1218695	10	1	0.4167287	0.1218695
chr8	87067165	87101942	34778	2	8	0	0.08923391	0	10	1	0.4167287	0.30102999
chr8	87101942	87102001	60	1	8	1	0.08923391	0.1218695	10	1	0.4167287	0.1218695
chr8	87102001	87410774	308774	5	7	1	0.05232577	0.1218695	10	1	0.56314362	0.1218695
chr8	87410774	87449811	39038	2	8	1	0.08923391	0.1218695	10	1	0.4167287	0.1218695
chr8	87449811	87530387	80577	2	8	0	0.20764654	0	8	1	0.20764654	0.30102999
chr8	87530387	87844380	313994	8	8	1	0.08923391	0.1218695	10	1	0.4167287	0.1218695
chr8	87844380	88076388	232009	5	9	1	0.14135546	0.1218695	10	1	0.30102999	0.1218695
chr8	88076388	88076447	60	1	9	1	0.09154957	0.1218695	11	1	0.41444892	0.1218695
chr8	88076447	88124813	48367	1	9	0	0.09154957	0	11	1	0.41444892	0.30102999
chr8	88124813	88204890	80078	2	9	0	0.0565833	0	12	1	0.55190077	0.30102999
chr8	88204890	88233814	28925	2	9	1	0.0565833	0.1218695	12	1	0.55190077	0.1218695
chr8	88233814	88493859	260046	5	9	0	0.09154957	0	11	1	0.41444892	0.30102999
chr8	88493859	88493918	60	1	9	0	0.0565833	0	12	1	0.55190077	0.30102999
chr8	88493918	88884933	391016	4	9	0	0.09154957	0	11	1	0.41444892	0.30102999
chr8	88884933	89180109	295177	5	8	0	0.08923391	0	10	1	0.4167287	0.30102999
chr8	89180109	89288872	108764	2	8	0	0.30102999	0	7	0	0.13499366	0
chr8	89288872	89288931	60	1	8	0	0.08923391	0	10	0	0.4167287	0
chr8	89288931	89339365	50435	1	8	0	0.13872638	0	9	0	0.30102999	0
chr8	89339365	89408099	68735	1	8	0	0.20764654	0	8	0	0.20764654	0
chr8	89408099	89408158	60	1	8	0	0.13872638	0	9	0	0.30102999	0

chr8	89408158	89832250	424093	4	8	0	0.20764654	0	8	0	0.20764654	0
chr8	89832250	89959514	127265	2	8	0	0.05490675	0	11	0	0.55623409	0
chr8	89959514	89959573	60	1	8	0	0.03209037	0	12	0	0.72109894	0
chr8	89959573	90271287	311715	1	8	0	0.13872638	0	9	0	0.30102999	0
chr8	90271287	90356558	85272	2	9	0	0.20975986	0	9	0	0.20975986	0
chr8	90356558	90539895	183338	2	9	0	0.30102999	0	8	0	0.13872638	0
chr8	90539895	90539954	60	1	9	0	0.20975986	0	9	0	0.20975986	0
chr8	90539954	90734760	194807	2	9	0	0.30102999	0	8	0	0.13872638	0
chr8	90734760	90802628	67869	5	9	0	0.20975986	0	9	0	0.20975986	0
chr8	90802628	90921818	119191	1	9	0	0.30102999	0	8	0	0.13872638	0
chr8	90921818	91049041	127224	6	9	0	0.20975986	0	9	0	0.20975986	0
chr8	91049041	91125928	76888	4	9	0	0.09154957	0	11	0	0.41444892	0
chr8	91125928	91125987	60	1	9	0	0.0565833	0	12	0	0.55190077	0
chr8	91125987	91325764	199778	1	9	0	0.09154957	0	11	0	0.41444892	0
chr8	91325764	91325823	60	1	9	0	0.0331093	0	13	0	0.71538971	0
chr8	91325823	91611770	285948	3	9	0	0.09154957	0	11	0	0.41444892	0
chr8	91611770	91667198	55429	2	9	0	0.0565833	0	12	0	0.55190077	0
chr8	91667198	91712507	45310	2	10	0	0.09290028	0	12	0	0.41314172	0
chr8	91712507	91775040	62534	1	10	0	0.14303407	0	11	0	0.30102999	0
chr8	91775040	91893357	118318	3	9	0	0.09154957	0	11	0	0.41444892	0
chr8	91893357	91996633	103277	2	9	0	0.30102999	0	8	0	0.13872638	0
chr8	91996633	91996692	60	1	9	0	0.14135546	0	10	0	0.30102999	0
chr8	91996692	92017958	21267	1	8	0	0.13872638	0	9	0	0.30102999	0
chr8	92017958	92261799	243842	7	8	0	0.20764654	0	8	0	0.20764654	0
chr8	92261799	92527471	265673	6	9	0	0.30102999	0	8	0	0.13872638	0
chr8	92527471	92527530	60	1	9	0	0.20975986	0	9	0	0.20975986	0
chr8	92527530	92650333	122804	2	8	0	0.20764654	0	8	0	0.20764654	0
chr8	92650333	92650392	60	1	9	0	0.20975986	0	9	0	0.20975986	0
chr8	92650392	92918168	267777	2	8	0	0.30102999	0	7	0	0.13499366	0
chr8	92918168	92918227	60	1	10	0	0.4167287	0	8	0	0.08923391	0
chr8	92918227	92971254	53028	1	9	0	0.30102999	0	8	0	0.13872638	0
chr8	92971254	92999161	27908	2	9	0	0.14135546	0	10	0	0.30102999	0
chr8	92999161	93029826	30666	2	10	0	0.21118145	0	10	0	0.21118145	0
chr8	93029826	93061154	31329	1	10	0	0.30102999	0	9	0	0.14135546	0
chr8	93061154	93088389	27236	1	10	0	0.4167287	0	8	0	0.08923391	0
chr8	93088389	93194969	106581	2	8	0	0.30102999	0	7	0	0.13499366	0
chr8	93194969	93335136	140168	2	8	0	0.20764654	0	8	0	0.20764654	0
chr8	93335136	93335195	60	1	8	0	0.13872638	0	9	0	0.30102999	0
chr8	93335195	93469030	133836	1	8	0	0.20764654	0	8	0	0.20764654	0
chr8	93469030	93662591	193562	1	7	0	0.13499366	0	8	0	0.30102999	0
chr8	93662591	93767760	105170	1	7	0	0.20469099	0	7	0	0.20469099	0
chr8	93767760	93824090	56331	2	8	0	0.30102999	0	7	0	0.13499366	0
chr8	93824090	93824149	60	1	8	0	0.05490675	0	11	0	0.55623409	0
chr8	93824149	93945663	121515	2	8	0	0.20764654	0	8	0	0.20764654	0
chr8	93945663	94081963	136301	3	8	0	0.08923391	0	10	0	0.4167287	0
chr8	94081963	94320302	238340	3	7	0	0.08584816	0	9	0	0.42015402	0
chr8	94320302	94320361	60	1	7	0	0.01667721	0	12	0	0.92532268	0
chr8	94320361	94430294	109934	1	7	0	0.08584816	0	9	0	0.42015402	0
chr8	94430294	94465088	34795	2	7	0	0.05232577	0	10	0	0.56314362	0
chr8	94465088	94465147	60	1	8	0	0.03209037	0	12	0	0.72109894	0
chr8	94465147	94550070	84924	1	8	0	0.05490675	0	11	0	0.55623409	0
chr8	94550070	94622311	72242	2	8	0	0.03209037	0	12	0	0.72109894	0
chr8	94622311	94737899	115589	3	8	0	0.00433294	0	15	0	1.3873209	0
chr8	94737899	94784854	46956	4	9	0	0.00433294	0	16	0	1.3873209	0
chr8	94784854	94992353	207500	3	8	0	0.00433294	0	15	0	1.3873209	0
chr8	94992353	95106370	114018	2	10	0	0.00909834	0	16	0	1.13428373	0
chr8	95106370	95220701	114332	4	10	0	0.00413023	0	17	0	1.39918918	0
chr8	95220701	95582623	361923	10	11	0	0.00859896	0	17	0	1.14735594	0
chr8	95582623	95718151	135529	2	11	0	0.01767679	0	16	0	0.91308053	0
chr8	95718151	95718209	59	1	11	0	0.00859896	0	17	0	1.14735594	0
chr8	95718209	95787879	69671	2	10	0	0.00909834	0	16	0	1.13428373	0
chr8	95787879	95906483	118605	6	10	0	0.00413023	0	17	0	1.39918918	0
chr8	95906483	95944304	37822	1	10	0	0.00909834	0	16	0	1.13428373	0
chr8	95944304	96039968	95665	2	10	0	0.01817691	0	15	0	0.90719478	0
chr8	96039968	96070073	30106	2	10	0	0.00909834	0	16	0	1.13428373	0
chr8	96070073	96257651	187579	3	10	0	0.01817691	0	15	0	0.90719478	0
chr8	96257651	96257710	60	1	11	0	0.0331093	0	15	0	0.71538971	0
chr8	96257710	96269237	11528	2	11	0	0.0574087	0	14	0	0.5498098	0
chr8	96269237	96432872	163636	3	11	0	0.0331093	0	15	0	0.71538971	0
chr8	96432872	96432931	60	1	11	0	0.01767679	0	16	0	0.91308053	0
chr8	96432931	96530476	97546	1	11	0	0.0331093	0	15	0	0.71538971	0
chr8	96530476	96764742	234267	2	11	0	0.0574087	0	14	0	0.5498098	0
chr8	96764742	96846254	81513	2	11	0	0.0331093	0	15	0	0.71538971	0
chr8	96846254	96846313	60	1	13	0	0.05490675	0	16	0	0.55623409	0
chr8	96846313	96936902	90590	1	12	0	0.14385241	0	13	0	0.30102999	0
chr8	96936902	97014872	77971	1	12	0	0.30102999	0	11	0	0.14385241	0
chr8	97014872	97014931	60	1	12	0	0.21227046	0	12	0	0.21227046	0
chr8	97014931	97180081	165151	2	12	0	0.30102999	0	11	0	0.14385241	0
chr8	97180081	97599692	419612	12	12	0	0.09290028	0	14	0	0.41314172	0
chr8	97599692	97670469	70778	3	13	0	0.14303407	0	14	0	0.30102999	0
chr8	97670469	97744108	73640	3	13	0	0.09154957	0	15	0	0.41444892	0
chr8	97744108	97839787	95680	3	13	0	0.05490675	0	16	0	0.55623409	0
chr8	97839787	97894579	54793	2	13	0	0.03037338	0	17	0	0.73110763	0
chr8	97894579	97894638	60	1	14	0	0.05232577	0	17	0	0.56314362	0
chr8	97894638	98078238	183601	4	13	0	0.03037338	0	17	0	0.73110763	0
chr8	98078238	98156892	78655	3	14	0	0.05232577	0	17	0	0.56314362	0
chr8	98156892	98237667	80776	1	13	0	0.03037338	0	17	0	0.73110763	0
chr8	98237667	98237726	60	1	13	1	0.03037338	0.30102999	17	0	0.73110763	0
chr8	98237726	98285813	48088	1	13	0	0.03037338	0	17	0	0.73110763	0
chr8	98285813	98324149	38337	4	14	0	0.05232577	0	17	0	0.56314362	0
chr8	98324149	98324208	60	1	15	0	0.08584816	0	17	0	0.42015402	0
chr8	98324208	98432309	108102	2	14	0	0.05232577	0	17	0	0.56314362	0
chr8	98432309	98560897	128589	1	14	0	0.08923391	0	16	0	0.4167287	0
chr8	98560897	98560956	60	1	15	0	0.13872638	0	16	0	0.30102999	0
chr8	98560956	98673425	112470	2	14	0	0.08923391	0	16	0	0.4167287	0
chr8	98673425	98906274	232850	7	13	0	0.05490675	0	16	0	0.55623409	0
chr8	98906274	99005555	99282	3	13	0	0.03037338	0	17	0	0.73110763	0
chr8	99005555	99266324	260770	9	15	1	0.08584816	0.30102999	17	0	0.42015402	0
chr8	99266324	99291095	24772	1	14	1	0.21118145	0.30102999	14	0	0.21118145	0
chr8	99291095	99417167	126073	3	14	1	0.14135546	0.30102999	15	0	0.30102999	0
chr8	99417167	99417226	60	1	15	1	0.08584816	0.30102999	17	0	0.42015402	0
chr8	99417226	99527300	110075	2	15	0	0.13872638	0	16	0	0.30102999	0
chr8	99527300	99735118	207819	6	15	1	0.08584816	0.30102999	17	0	0.42015402	0
chr8	99735118	99873949	138832	3	14	1	0.05232577	0.30102999	17	0	0.56314362	0
chr8	99873949	99956048	82100	2	15	1	0.08584816	0.30102999	17	0	0.42015402	0
chr8	99956048	99995666	39619	5	16	1	0.08122616	0.30102999	18	0	0.4250187	0
chr8	99995666	100129249	133584	2	15	1	0.04875589	0.30102999	18	0	0.5732208	0
chr8	100129249	100129308	60	1	16	1	0.08122616	0.30102999	18	0	0.4250187	0
chr8	100129308	100205095	75788	2	15	1	0.04875589	0.30102999	18	0	0.5732208	0
chr8	100205095	100290947	85853	3	16	1	0.08122616	0.30102999	18	0	0.4250187	0
chr8	100290947	100344417	53471	1	14	1	0.02793176	0.30102999	18	0	0.74627054	0
chr8	100344417	100396443	52027	1	14	1	0.05232577	0.30102999	17	0	0.56314362	0
chr8	100396443	100396502	60	1	16	1	0.08122616	0.30102999	18	0	0.4250187	0
chr8	100396502	100463947	67446	1	14	1	0.05232577	0.30102999	17	0	0.56314362	0
chr8	100463947	100493833	29887	2	14	1	0.02793176	0.30102999	18	0	0.74627054	0
chr8	100493833	100576059	82227	4	16	1	0.04407651	0.30102999	19	0	0.58747015	0
chr8	100576059	100994343	418285	10	15	0	0.04875589	0	18	0	0.5732208	0
chr8	100994343	101164772	170430	5	14	0	0.02793176	0	18	0	0.74627054	0
chr8	101164772	101251572	86801	2	14	0	0.05232577	0	17	0	0.56314362	0
chr8	101251572	101545472	293901	4	15	0	0.04875589	0	18	0	0.5732208	0
chr8	101545472	101630576	85105	2	15	0	0.08584816	0	17	0	0.42015402	0
chr8	101630576	101675051	44476	2	15	0	0.04875589	0	18	0	0.5732208	0
chr8	101675051	101731057	56007	4	16	0	0.08122616	0	18	0	0.4250187	0
chr8	101731057	101864506	133450	1	15	0	0.04875589	0	18	0	0.5732208	0

chr8	101864506	101947980	83475	2	14	0	0.02793176	0	18	0	0.74627054	0
chr8	101947980	101953221	5242	2	15	0	0.04875589	0	18	0	0.5732208	0
chr8	101953221	102067097	113877	1	15	0	0.13872638	0	16	0	0.30102999	0
chr8	102067097	102137815	70719	2	16	0	0.13499366	0	17	0	0.30102999	0
chr8	102137815	102273350	135536	6	17	0	0.129913	0	18	0	0.30102999	0
chr8	102273350	102273409	60	1	17	0	0.07511598	0	19	0	0.43181735	0
chr8	102273409	102381990	108582	1	16	0	0.04407651	0	19	0	0.58747015	0
chr8	102381990	102422643	40654	1	16	0	0.08122616	0	18	0	0.4250187	0
chr8	102422643	102611301	188659	4	16	0	0.13499366	0	17	0	0.30102999	0
chr8	102611301	102698924	87624	4	16	0	0.08122616	0	18	0	0.4250187	0
chr8	102698924	102698975	52	1	16	0	0.0079614	0	21	0	1.07548421	0
chr8	102698975	102731660	32686	1	16	0	0.02074938	0	20	0	0.79906872	0
chr8	102731660	102755472	23813	2	17	0	0.03812622	0	20	0	0.60763643	0
chr8	102755472	102803078	47607	1	17	0	0.07511598	0	19	0	0.43181735	0
chr8	102803078	102803133	56	1	17	0	0.03812622	0	20	0	0.60763643	0
chr8	102803133	102868211	65079	1	16	0	0.02074938	0	20	0	0.79906872	0
chr8	102868211	102968972	100762	2	16	0	0.04407651	0	19	0	0.58747015	0
chr8	102968972	103113904	144933	5	17	0	0.03812622	0	20	0	0.60763643	0
chr8	103113904	103205695	91792	2	17	0	0.01598258	0	21	0	0.84395715	0
chr8	103205695	103247710	42016	1	17	0	0.03812622	0	20	0	0.60763643	0
chr8	103247710	103389163	141454	3	16	0	0.02074938	0	20	0	0.79906872	0
chr8	103389163	103515061	125899	4	17	0	0.03812622	0	20	0	0.60763643	0
chr8	103515061	103589803	74743	3	16	0	0.02074938	0	20	0	0.79906872	0
chr8	103589803	103589862	60	1	17	0	0.03812622	0	20	0	0.60763643	0
chr8	103589862	103664630	74769	2	16	0	0.02074938	0	20	0	0.79906872	0
chr8	103664630	103769626	104997	1	16	0	0.08122616	0	18	0	0.4250187	0
chr8	103769626	103769685	60	1	17	0	0.129913	0	18	0	0.30102999	0
chr8	103769685	103841492	71808	1	16	0	0.13499366	0	17	0	0.30102999	0
chr8	103841492	103841551	60	1	16	0	0.08122616	0	18	0	0.4250187	0
chr8	103841551	103954240	112690	3	15	0	0.04875589	0	18	0	0.5732208	0
chr8	103954240	103954299	60	1	15	0	0.02473314	0	19	0	0.76806864	0
chr8	103954299	104102022	147724	3	15	0	0.08584816	0	17	0	0.42015402	0
chr8	104102022	104102081	60	1	15	0	0.04875589	0	18	0	0.5732208	0
chr8	104102081	104197966	95886	2	15	0	0.08584816	0	17	0	0.42015402	0
chr8	104197966	104198025	60	1	15	0	0.04875589	0	18	0	0.5732208	0
chr8	104198025	104499594	301570	11	15	0	0.08584816	0	17	0	0.42015402	0
chr8	104499594	104853259	353666	9	15	0	0.04875589	0	18	0	0.5732208	0
chr8	104853259	104853318	60	1	15	0	0.02473314	0	19	0	0.76806864	0
chr8	104853318	105087630	234313	7	14	0	0.05232577	0	17	0	0.56314362	0
chr8	105087630	105225087	137458	2	14	0	0.08923391	0	16	0	0.4167287	0
chr8	105225087	105261842	36756	1	14	0	0.21118145	0	14	0	0.21118145	0
chr8	105261842	105358106	96265	2	12	0	0.09290028	0	14	0	0.41314172	0
chr8	105358106	105394000	35895	1	10	0	0.0574087	0	13	0	0.5498098	0
chr8	105394000	105436584	42585	1	10	0	0.09290028	0	12	0	0.41314172	0
chr8	105436584	105476947	40364	2	12	0	0.09290028	0	14	0	0.41314172	0
chr8	105476947	105477006	60	1	12	0	0.0565833	0	15	0	0.55190077	0
chr8	105477006	105509388	32383	1	12	0	0.09290028	0	14	0	0.41314172	0
chr8	105509388	105544140	34753	2	12	0	0.03209037	0	16	0	0.72109894	0
chr8	105544140	105564361	20222	2	12	0	0.00778066	0	18	0	1.17038931	0
chr8	105564361	105610940	46580	2	12	0	0.01667721	0	17	0	0.92532268	0
chr8	105610940	105788086	177147	1	12	0	0.0565833	0	15	0	0.55190077	0
chr8	105788086	105914640	126555	1	11	0	0.0331093	0	15	0	0.71538971	0
chr8	105914640	105914699	60	1	11	0	0.01767679	0	16	0	0.91308053	0
chr8	105914699	106240525	325827	2	11	0	0.0331093	0	15	0	0.71538971	0
chr8	106240525	106240584	60	1	11	0	0.01767679	0	16	0	0.91308053	0
chr8	106240584	106319209	78626	1	10	0	0.00909834	0	16	0	1.13428373	0
chr8	106319209	106400112	80904	1	10	0	0.01817691	0	15	0	0.90719478	0
chr8	106400112	106461292	61181	3	11	0	0.0331093	0	15	0	0.71538971	0
chr8	106461292	106503389	42098	2	11	0	0.01767679	0	16	0	0.91308053	0
chr8	106503389	106503448	60	1	12	0	0.03209037	0	16	0	0.72109894	0
chr8	106503448	106639222	135775	2	11	0	0.0574087	0	14	0	0.5498098	0
chr8	106639222	106705607	66386	2	12	0	0.0565833	0	15	0	0.55190077	0
chr8	106705607	106746062	40456	2	14	0	0.14135546	0	15	0	0.30102999	0
chr8	106746062	107025536	279475	4	14	0	0.21118145	0	14	0	0.21118145	0
chr8	107025536	107104146	78611	2	15	0	0.30102999	0	14	0	0.14135546	0
chr8	107104146	107243362	139217	1	13	0	0.14303407	0	14	0	0.30102999	0
chr8	107243362	107307812	64451	2	13	0	0.09154957	0	15	0	0.41444892	0
chr8	107307812	107441071	133260	4	15	0	0.13872638	0	16	0	0.30102999	0
chr8	107441071	107472460	31390	2	15	0	0.08584816	0	17	0	0.42015402	0
chr8	107472460	107561136	88677	2	14	0	0.08923391	0	16	0	0.4167287	0
chr8	107561136	107561195	60	1	15	0	0.08584816	0	17	0	0.42015402	0
chr8	107561195	107669943	108749	2	14	0	0.08923391	0	16	0	0.4167287	0
chr8	107669943	107733014	63072	3	14	0	0.05232577	0	17	0	0.56314362	0
chr8	107733014	107764000	30987	2	14	0	0.21118145	0	14	0	0.21118145	0
chr8	107764000	107782117	18118	2	15	0	0.13872638	0	16	0	0.30102999	0
chr8	107782117	107852627	70511	1	14	0	0.14135546	0	15	0	0.30102999	0
chr8	107852627	108145484	292858	2	14	0	0.21118145	0	14	0	0.21118145	0
chr8	108145484	108262935	117452	3	15	0	0.20975986	0	15	0	0.20975986	0
chr8	108262935	108348430	85496	3	15	0	0.08584816	0	17	0	0.42015402	0
chr8	108348430	108413208	64779	1	15	0	0.13872638	0	16	0	0.30102999	0
chr8	108413208	108448796	35589	2	15	0	0.08584816	0	17	0	0.42015402	0
chr8	108448796	108512442	63647	1	14	0	0.21118145	0	14	0	0.21118145	0
chr8	108512442	108633452	121011	1	13	0	0.30102999	0	12	0	0.14385241	0
chr8	108633452	108762923	129472	1	12	0	0.30102999	0	11	0	0.14385241	0
chr8	108762923	108917256	154334	1	11	0	0.21200206	0	11	0	0.21200206	0
chr8	108917256	108942691	25436	2	12	0	0.21227046	0	12	0	0.21227046	0
chr8	108942691	109009030	66340	1	10	0	0.09290028	0	12	0	0.41314172	0
chr8	109009030	109058489	49460	1	10	0	0.14303407	0	11	0	0.30102999	0
chr8	109058489	109248528	190040	5	11	0	0.21200206	0	11	0	0.21200206	0
chr8	109248528	109498859	250332	5	11	0	0.30102999	0	10	0	0.14303407	0
chr8	109498859	109567858	69000	1	10	0	0.21118145	0	10	0	0.21118145	0
chr8	109567858	109619675	51818	2	10	0	0.14303407	0	11	0	0.30102999	0
chr8	109619675	109677828	58154	2	11	0	0.14385241	0	12	0	0.30102999	0
chr8	109677828	109677887	60	1	12	0	0.21227046	0	12	0	0.21227046	0
chr8	109677887	109774130	96244	2	12	0	0.30102999	0	11	0	0.14385241	0
chr8	109774130	109862169	88040	2	11	0	0.30102999	0	10	0	0.14303407	0
chr8	109862169	110051157	188989	1	10	0	0.4167287	0	8	0	0.08923391	0
chr8	110051157	110453542	402386	10	9	0	0.30102999	0	8	0	0.13872638	0
chr8	110453542	110527427	73886	2	9	0	0.42015402	0	7	0	0.08584816	0
chr8	110527427	110573111	45685	2	9	0	0.30102999	0	8	0	0.13872638	0
chr8	110573111	110922797	349687	5	9	0	0.42015402	0	7	0	0.08584816	0
chr8	110922797	110995144	72348	4	9	0	0.20975986	0	9	0	0.20975986	0
chr8	110995144	111297761	302618	2	9	0	0.42015402	0	7	0	0.08584816	0
chr8	111297761	111297820	60	1	9	0	0.30102999	0	8	0	0.13872638	0
chr8	111297820	111519183	221364	1	8	0	0.30102999	0	7	0	0.13499366	0
chr8	111519183	111661171	141989	1	5	0	0.19510895	0	5	0	0.19510895	0
chr8	111661171	111951271	290101	2	3	0	0.10122019	0	4	0	0.30102999	0
chr8	111951271	112359493	408223	4	2	0	0.08289318	0	3	0	0.30102999	0
chr8	112359493	112451182	91690	2	3	0	0.05670724	0	5	0	0.45545077	0
chr8	112451182	112451241	60	1	8	0	0.30102999	0	7	0	0.13499366	0
chr8	112451241	112932561	481321	3	7	0	0.20469099	0	7	0	0.20469099	0
chr8	112932561	113163217	230657	2	10	0	0.4167287	0	8	0	0.08923391	0
chr8	113163217	113163276	60	1	12	0	0.30102999	0	11	0	0.14385241	0
chr8	113163276	113236651	73376	1	12	0	0.41314172	0	10	0	0.09290028	0
chr8	113236651	113352093	115443	3	12	0	0.21227046	0	12	0	0.21227046	0
chr8	113352093	113393695	41603	2	13	0	0.14303407	0	14	0	0.30102999	0
chr8	113393695	113443874	50180	2	13	0	0.14303407	0	14	1	0.30102999	0.30102999
chr8	113443874	113589924	146051	3	13	0	0.21200206	0	13	1	0.21200206	0.30102999
chr8	113589924	113702121	112198	2	13	0	0.30102999	0	12	0	0.14385241	0
chr8	113702121	113732737	30617	2	13	0	0.09154957	0	15	1	0.41444892	0.30102999
chr8	113732737	113878724	145988	3	13	0	0.21200206	0	13	0	0.21200206	0
chr8	113878724	113903030	24307	2	13	0	0.09154957	0	15	1	0.41444892	0.30102999
chr8	113903030	114006907	103878	2	13	0	0.21200206	0	13	0	0.21200206	0
chr8	114006907	114173007	166101	4	13	0	0.21200206	0	13	1	0.21200206	0.30102999

chr8	114173007	114317923	144917	4	13	0	0.14303407	0	14	1	0.30102999	0.30102999
chr8	114317923	114380869	62947	2	13	0	0.09154957	0	15	1	0.41444892	0.30102999
chr8	114380869	114450308	69440	1	13	0	0.09154957	0	15	0	0.41444892	0
chr8	114450308	114633352	183045	1	12	0	0.0565833	0	15	0	0.55190077	0
chr8	114633352	114863900	230549	1	12	0	0.14385241	0	13	0	0.30102999	0
chr8	114863900	114962376	98477	1	11	0	0.09334429	0	13	0	0.41271556	0
chr8	114962376	115124554	162179	1	11	0	0.14385241	0	12	0	0.30102999	0
chr8	115124554	115430637	306084	2	8	0	0.13872638	0	9	0	0.30102999	0
chr8	115430637	115430696	60	1	8	0	0.08923391	0	10	0	0.4167287	0
chr8	115430696	115656140	225445	1	8	0	0.20764654	0	8	0	0.20764654	0
chr8	115656140	115803228	147089	2	10	0	0.0574087	0	13	0	0.5498098	0
chr8	115803228	115803287	60	1	11	0	0.01767679	0	16	0	0.91308053	0
chr8	115803287	116002879	199593	2	10	0	0.0574087	0	13	0	0.5498098	0
chr8	116002879	116002938	60	1	11	0	0.00859896	0	17	0	1.14735594	0
chr8	116002938	116617156	614219	7	10	0	0.03344703	0	14	0	0.71353304	0
chr8	116617156	116676778	59623	1	10	0	0.0574087	0	13	0	0.5498098	0
chr8	116676778	116948828	272051	2	10	0	0.14303407	0	11	0	0.30102999	0
chr8	116948828	116948887	60	1	12	0	0.21227046	0	12	0	0.21227046	0
chr8	116948887	117197436	248550	4	12	0	0.30102999	0	11	0	0.14385241	0
chr8	117197436	117197495	60	1	13	0	0.21200206	0	13	0	0.21200206	0
chr8	117197495	117656808	459314	2	13	0	0.30102999	0	12	0	0.14385241	0
chr8	117656808	117699497	42690	2	13	0	0.21200206	0	13	0	0.21200206	0
chr8	117699497	117884939	185443	4	13	0	0.30102999	0	12	0	0.14385241	0
chr8	117884939	118391347	506409	9	14	0	0.30102999	0	13	0	0.14303407	0
chr8	118391347	118806849	415503	5	14	0	0.21118145	0	14	0	0.21118145	0
chr8	118806849	119418329	611481	12	14	0	0.14135546	0	15	0	0.30102999	0
chr8	119418329	119682877	264549	6	15	0	0.20975986	0	15	0	0.20975986	0
chr8	119682877	119886789	203913	2	15	0	0.13872638	0	16	0	0.30102999	0
chr8	119886789	119957106	70318	3	15	0	0.08584816	0	17	0	0.42015402	0
chr8	119957106	120079510	122405	2	15	0	0.13872638	0	16	0	0.30102999	0
chr8	120079510	120079562	53	1	15	0	0.08584816	0	17	0	0.42015402	0
chr8	120079562	120429746	350185	6	15	0	0.13872638	0	16	0	0.30102999	0
chr8	120429746	120580415	150670	4	16	0	0.20764654	0	16	0	0.20764654	0
chr8	120580415	120602816	22402	2	17	0	0.20469099	0	17	0	0.20469099	0
chr8	120602816	120651018	48203	2	17	0	0.42015402	0	15	0	0.08584816	0
chr8	120651018	121444240	793223	22	17	0	0.129913	0	18	0	0.30102999	0
chr8	121444240	121747270	303031	12	17	0	0.07511598	0	19	0	0.43181735	0
chr8	121747270	122142883	395614	4	17	0	0.129913	0	18	0	0.30102999	0
chr8	122142883	122329549	186667	1	16	0	0.20764654	0	16	0	0.20764654	0
chr8	122329549	122494596	165048	1	15	0	0.30102999	0	14	0	0.14135546	0
chr8	122494596	122637617	143022	1	12	0	0.41314172	0	10	0	0.09290028	0
chr8	122637617	122637676	60	1	12	0	0.30102999	0	11	0	0.14385241	0
chr8	122637676	122653162	15487	2	12	0	0.41314172	0	10	0	0.09290028	0
chr8	122653162	122653221	60	1	12	0	0.30102999	0	11	0	0.14385241	0
chr8	122653221	122863357	210137	1	12	0	0.92532268	0	7	0	0.01667721	0
chr8	122863357	122863416	60	1	12	0	0.72109894	0	8	0	0.03209037	0
chr8	122863416	123165226	301811	3	8	0	0.79906872	0	4	0	0.02074938	0
chr8	123165226	123165285	60	1	11	0	0.73110763	0	7	0	0.03037338	0
chr8	123165285	123431150	265866	1	11	0	0.9449689	0	6	0	0.01518174	0
chr8	123431150	123538325	107176	2	13	0	0.30102999	0	12	0	0.14385241	0
chr8	123538325	123538384	60	1	14	0	0.41314172	0	12	0	0.09290028	0
chr8	123538384	123743989	205606	2	12	0	0.30102999	0	11	0	0.14385241	0
chr8	123743989	123812877	68889	2	13	0	0.21200206	0	13	0	0.21200206	0
chr8	123812877	123852933	40057	2	14	0	0.21118145	0	14	0	0.21118145	0
chr8	123852933	124042821	189889	4	14	0	0.30102999	0	13	0	0.14303407	0
chr8	124042821	124115445	72625	3	14	0	0.05232577	0	17	0	0.56314362	0
chr8	124115445	124403872	288428	13	15	0	0.08584816	0	17	0	0.42015402	0
chr8	124403872	124453740	49869	2	15	0	0.02473314	0	19	0	0.76806864	0
chr8	124453740	124453799	60	1	16	0	0.02074938	0	20	0	0.79906872	0
chr8	124453799	124551697	97899	2	16	0	0.04407651	0	19	0	0.58747015	0
chr8	124551697	124706038	154342	4	17	0	0.03812622	0	20	0	0.60763643	0
chr8	124706038	124746836	40799	1	17	0	0.07511598	0	19	0	0.43181735	0
chr8	124746836	124922842	176007	4	16	0	0.04407651	0	19	0	0.58747015	0
chr8	124922842	125025913	103072	3	17	0	0.07511598	0	19	0	0.43181735	0
chr8	125025913	125058412	32500	3	17	0	0.03812622	0	20	0	0.60763643	0
chr8	125058412	125137486	79075	2	16	0	0.04407651	0	19	0	0.58747015	0
chr8	125137486	125137545	60	1	17	0	0.07511598	0	19	0	0.43181735	0
chr8	125137545	125908133	770589	18	16	0	0.04407651	0	19	0	0.58747015	0
chr8	125908133	126011610	103478	4	17	0	0.07511598	0	19	0	0.43181735	0
chr8	126011610	126061312	49703	2	17	0	0.129913	0	18	0	0.30102999	0
chr8	126061312	126102049	40738	2	17	0	0.07511598	0	19	0	0.43181735	0
chr8	126102049	126131821	29773	1	16	0	0.04407651	0	19	0	0.58747015	0
chr8	126131821	126163413	31593	1	15	0	0.02473314	0	19	0	0.76806864	0
chr8	126163413	126449694	286282	7	16	0	0.04407651	0	19	0	0.58747015	0
chr8	126449694	126800956	351263	3	15	0	0.02473314	0	19	0	0.76806864	0
chr8	126800956	127095547	294592	5	16	0	0.04407651	0	19	0	0.58747015	0
chr8	127095547	127146431	50885	1	16	0	0.08122616	0	18	0	0.4250187	0
chr8	127146431	127247560	101130	1	15	0	0.04875589	0	18	0	0.5732208	0
chr8	127247560	127389078	141519	1	15	0	0.08584816	0	17	0	0.42015402	0
chr8	127389078	127770332	381255	6	15	0	0.71538971	0	11	0	0.0331093	0
chr8	127770332	127770391	60	1	16	0	0.20764654	0	16	0	0.20764654	0
chr8	127770391	128052290	281900	2	16	0	0.30102999	0	15	0	0.13872638	0
chr8	128052290	128052349	60	1	17	0	0.20469099	0	17	0	0.20469099	0
chr8	128052349	128174165	121817	1	17	0	0.73110763	0	13	0	0.03037338	0
chr8	128174165	128232861	58697	1	15	0	0.41444892	0	13	0	0.09154957	0
chr8	128232861	128401403	168543	3	15	0	0.30102999	0	14	0	0.14135546	0
chr8	128401403	128479520	78118	2	17	0	0.56314362	0	14	0	0.05232577	0
chr8	128479520	128707459	227940	1	15	0	0.30102999	0	14	0	0.14135546	0
chr8	128707459	128828456	120998	6	15	0	0.20975986	0	15	0	0.20975986	0
chr8	128828456	128886097	57642	2	15	0	0.13872638	0	16	0	0.30102999	0
chr8	128886097	128931603	45507	1	15	0	0.30102999	0	14	0	0.14135546	0
chr8	128931603	129046130	114528	5	15	0	0.20975986	0	15	0	0.20975986	0
chr8	129046130	129147723	101594	3	14	0	0.30102999	0	13	0	0.14303407	0
chr8	129147723	129161861	14139	2	15	0	0.20975986	0	15	0	0.20975986	0
chr8	129161861	129235191	73331	2	14	0	0.30102999	0	13	0	0.14303407	0
chr8	129235191	129381645	146455	1	14	0	0.41314172	0	12	0	0.09290028	0
chr8	129381645	129381704	60	1	15	0	0.41444892	0	13	0	0.09154957	0
chr8	129381704	129505388	123685	1	14	0	0.30102999	0	13	0	0.14303407	0
chr8	129505388	129505447	60	1	14	0	0.08923391	0	16	0	0.4167287	0
chr8	129505447	129641936	136490	1	14	0	0.14135546	0	15	0	0.30102999	0
chr8	129641936	129903075	261140	3	15	0	0.13872638	0	16	0	0.30102999	0
chr8	129903075	129903134	60	1	15	0	0.08584816	0	17	0	0.42015402	0
chr8	129903134	130089962	186829	1	14	0	0.21118145	0	14	0	0.21118145	0
chr8	130089962	130250255	160294	1	12	0	0.09290028	0	14	0	0.41314172	0
chr8	130250255	130505862	255608	7	13	0	0.09154957	0	15	0	0.41444892	0
chr8	130505862	130606639	100778	3	14	0	0.14135546	0	15	0	0.30102999	0
chr8	130606639	130764114	157476	3	14	0	0.21118145	0	14	0	0.21118145	0
chr8	130764114	130788501	24388	2	15	0	0.20975986	0	15	0	0.20975986	0
chr8	130788501	130857383	68883	1	15	0	0.41444892	0	13	0	0.09154957	0
chr8	130857383	130887756	30374	2	16	0	0.55623409	0	13	0	0.05490675	0
chr8	130887756	130887815	60	1	16	0	0.20764654	0	16	0	0.20764654	0
chr8	130887815	130978746	90932	3	16	0	0.4167287	0	14	0	0.08923391	0
chr8	130978746	131042840	64095	3	16	0	0.30102999	0	15	0	0.13872638	0
chr8	131042840	131380111	337272	8	15	0	0.20975986	0	15	0	0.20975986	0
chr8	131380111	131465845	85735	1	15	0	0.30102999	0	14	0	0.14135546	0
chr8	131465845	131651428	185584	1	15	0	0.41444892	0	13	0	0.09154957	0
chr8	131651428	131651487	60	1	15	0	0.30102999	0	14	0	0.14135546	0
chr8	131651487	131755007	103521	1	15	0	0.41444892	0	13	0	0.09154957	0
chr8	131755007	131880114	125108	3	13	0	0.21200206	0	13	0	0.21200206	0
chr8	131880114	131955707	75594	2	14	0	0.21118145	0	14	0	0.21118145	0
chr8	131955707	132046763	91057	2	13	0	0.14303407	0	14	0	0.30102999	0
chr8	132046763	132089671	42909	1	13	0	0.21200206	0	13	0	0.21200206	0
chr8	132089671	132201216	111546	2	14	0	0.30102999	0	13	0	0.14303407	0
chr8	132201216	132201275	60	1	15	0	0.30102999	0	14	0	0.14135546	0

chr8	132201275	132435042	233768	1	13	0	0.14303407	0	14	0	0.30102999	0
chr8	132435042	132495569	60528	1	13	0	0.21200206	0	13	0	0.21200206	0
chr8	132495569	132667661	172093	1	13	0	0.30102999	0	12	0	0.14385241	0
chr8	132667661	132849059	181399	2	14	0	0.21118145	0	14	0	0.21118145	0
chr8	132849059	132849118	60	1	14	0	0.08923391	0	16	0	0.4167287	0
chr8	132849118	132942574	93457	1	14	0	0.30102999	0	13	0	0.14303407	0
chr8	132942574	133150205	207632	5	14	0	0.41314172	0	12	0	0.09290028	0
chr8	133150205	133318584	168380	5	14	0	0.21118145	0	14	0	0.21118145	0
chr8	133318584	133396634	78051	3	14	0	0.02793176	0	18	0	0.74627054	0
chr8	133396634	133584392	187759	3	14	0	0.05232577	0	17	0	0.56314362	0
chr8	133584392	134031884	447493	12	14	0	0.02793176	0	18	0	0.74627054	0
chr8	134031884	134147170	115287	6	15	0	0.04875589	0	18	0	0.5732208	0
chr8	134147170	134233364	86195	2	15	0	0.08584816	0	17	0	0.42015402	0
chr8	134233364	134307889	74526	6	14	0	0.08923391	0	16	0	0.4167287	0
chr8	134307889	134585098	277210	5	14	0	0.14135546	0	15	0	0.30102999	0
chr8	134585098	134649598	64501	2	14	0	0.08923391	0	16	0	0.4167287	0
chr8	134649598	134649657	60	1	15	0	0.08584816	0	17	0	0.42015402	0
chr8	134649657	134746618	96962	1	15	0	0.13872638	0	16	0	0.30102999	0
chr8	134746618	135055947	309330	3	15	0	0.08584816	0	17	0	0.42015402	0
chr8	135055947	135056006	60	1	16	0	0.13499366	0	17	0	0.30102999	0
chr8	135056006	135253032	197027	1	14	0	0.08923391	0	16	0	0.4167287	0
chr8	135253032	135416835	163804	1	11	0	0.0574087	0	14	0	0.5498098	0
chr8	135416835	135498647	81813	1	11	0	0.09334429	0	13	0	0.41271556	0
chr8	135498647	135558228	59582	2	11	0	0.0574087	0	14	0	0.5498098	0
chr8	135558228	135558287	60	1	12	0	0.0565833	0	15	0	0.55190077	0
chr8	135558287	135592578	34292	1	10	0	0.03344703	0	14	0	0.71353304	0
chr8	135592578	135696501	103924	2	10	0	0.0574087	0	13	0	0.5498098	0
chr8	135696501	135768464	71964	2	11	0	0.0331093	0	15	0	0.71538971	0
chr8	135768464	135803456	34993	2	11	0	0.01767679	0	16	0	0.91308053	0
chr8	135803456	135898618	95163	3	11	0	0.09334429	0	13	0	0.41271556	0
chr8	135898618	135898677	60	1	11	0	0.0331093	0	15	0	0.71538971	0
chr8	135898677	136533616	634940	6	10	0	0.09290028	0	12	0	0.41314172	0
chr8	136533616	136554927	21312	1	10	0	0.21118145	0	10	0	0.21118145	0
chr8	136554927	136809209	254283	4	10	0	0.30102999	0	9	0	0.14135546	0
chr8	136809209	136958296	149088	1	9	0	0.30102999	0	8	0	0.13872638	0
chr8	136958296	137325845	367550	3	9	0	0.5732208	0	6	0	0.04875589	0
chr8	137325845	137325904	60	1	9	0	0.42015402	0	7	0	0.08584816	0
chr8	137325904	137552141	226238	1	7	0	0.60763643	0	4	0	0.03812622	0
chr8	137552141	137633745	81605	2	8	0	0.79906872	0	4	0	0.02074938	0
chr8	137633745	137882966	249222	2	1	0	0.01091641	0	4	0	0.76005302	0
chr8	137882966	138006813	123848	1	1	0	0.02438896	0	3	0	0.51676182	0
chr8	138006813	138129879	123067	2	2	0	0.08289318	0	3	0	0.30102999	0
chr8	138129879	138129938	60	1	8	0	0.30102999	0	7	0	0.13499366	0
chr8	138129938	138391645	261708	2	8	0	0.4250187	0	6	0	0.08122616	0
chr8	138391645	138617053	225409	2	9	0	0.30102999	0	8	0	0.13872638	0
chr8	138617053	138796141	179089	2	9	0	0.20975986	0	9	0	0.20975986	0
chr8	138796141	138936007	139867	2	9	0	0.14135546	0	10	0	0.30102999	0
chr8	138936007	139030616	94610	2	10	0	0.14303407	0	11	0	0.30102999	0
chr8	139030616	139155151	124536	3	11	0	0.21200206	0	11	0	0.21200206	0
chr8	139155151	139229070	73920	3	12	0	0.30102999	0	11	0	0.14385241	0
chr8	139229070	139338537	109468	2	11	0	0.30102999	0	10	0	0.14303407	0
chr8	139338537	139380072	41536	2	12	0	0.30102999	0	11	0	0.14385241	0
chr8	139380072	139413945	33874	1	11	0	0.21200206	0	11	0	0.21200206	0
chr8	139413945	139465677	51733	2	11	0	0.14385241	0	12	0	0.30102999	0
chr8	139465677	139904014	438338	10	12	0	0.14385241	0	13	0	0.30102999	0
chr8	139904014	139904073	60	1	13	0	0.21200206	0	13	0	0.21200206	0
chr8	139904073	140297689	393617	3	12	0	0.14385241	0	13	0	0.30102999	0
chr8	140297689	140297748	60	1	13	0	0.21200206	0	13	1	0.21200206	0.30102999
chr8	140297748	140427685	129938	1	12	0	0.21227046	0	12	1	0.21227046	0.30102999
chr8	140427685	140682898	255214	3	12	0	0.14385241	0	13	1	0.30102999	0.30102999
chr8	140682898	140945526	262629	7	13	0	0.21200206	0	13	1	0.21200206	0.30102999
chr8	140945526	140945585	60	1	14	0	0.21118145	0	14	1	0.21118145	0.30102999
chr8	140945585	141174389	228805	5	14	0	0.30102999	0	13	1	0.14303407	0.30102999
chr8	141174389	141216384	41996	2	14	0	0.21118145	0	14	1	0.21118145	0.30102999
chr8	141216384	141216443	60	1	16	0	0.4167287	0	14	1	0.08923391	0.30102999
chr8	141216443	141285919	69477	1	16	0	0.55623409	0	13	1	0.05490675	0.30102999
chr8	141285919	141301047	15129	1	15	0	0.41444892	0	13	1	0.09154957	0.30102999
chr8	141301047	141301106	60	1	15	0	0.30102999	0	14	1	0.14135546	0.30102999
chr8	141301106	141364555	63450	1	15	0	0.41444892	0	13	1	0.09154957	0.30102999
chr8	141364555	141415716	51162	2	16	0	0.4167287	0	14	1	0.08923391	0.30102999
chr8	141415716	141447774	32059	1	15	0	0.41444892	0	13	1	0.09154957	0.30102999
chr8	141447774	141447833	60	1	15	0	0.30102999	0	14	1	0.14135546	0.30102999
chr8	141447833	141709192	261360	6	15	0	0.41444892	0	13	1	0.09154957	0.30102999
chr8	141709192	141709251	60	1	15	0	0.30102999	0	14	1	0.14135546	0.30102999
chr8	141709251	141813629	104379	4	15	0	0.41444892	0	13	1	0.09154957	0.30102999
chr8	141813629	141900696	87068	2	14	0	0.30102999	0	13	1	0.14303407	0.30102999
chr8	141900696	141900755	60	1	14	0	0.21118145	0	14	1	0.21118145	0.30102999
chr8	141900755	142080027	179273	4	14	0	0.30102999	0	13	1	0.14303407	0.30102999
chr8	142080027	142270535	190509	7	15	0	0.30102999	0	14	1	0.14135546	0.30102999
chr8	142270535	142308354	37820	1	14	0	0.21118145	0	14	1	0.21118145	0.30102999
chr8	142308354	142390136	81783	1	13	0	0.14303407	0	14	1	0.30102999	0.30102999
chr8	142390136	142390195	60	1	14	0	0.21118145	0	14	1	0.21118145	0.30102999
chr8	142390195	142501148	110954	2	13	0	0.21200206	0	13	1	0.21200206	0.30102999
chr8	142501148	142501207	60	1	14	0	0.30102999	0	13	1	0.14303407	0.30102999
chr8	142501207	142624972	123766	2	13	0	0.21200206	0	13	1	0.21200206	0.30102999
chr8	142624972	142680770	55799	2	14	0	0.30102999	0	13	1	0.14303407	0.30102999
chr8	142680770	142680829	60	1	15	0	0.41444892	0	13	1	0.09154957	0.30102999
chr8	142680829	142757798	76970	1	13	0	0.21200206	0	13	1	0.21200206	0.30102999
chr8	142757798	142840194	82397	1	12	0	0.14385241	0	13	1	0.30102999	0.30102999
chr8	142840194	142909763	69570	3	12	0	0.14385241	0	13	2	0.30102999	0.61140001
chr8	142909763	143053394	143632	3	12	0	0.21227046	0	12	2	0.21227046	0.61140001
chr8	143053394	143118366	64973	1	12	0	0.30102999	0	11	2	0.14385241	0.61140001
chr8	143118366	143282786	164421	2	12	0	0.72109894	0	8	2	0.03209037	0.61140001
chr8	143282786	143282845	60	1	12	0	0.41314172	0	10	2	0.09290028	0.61140001
chr8	143282845	143371909	89065	2	12	0	0.72109894	0	8	2	0.03209037	0.61140001
chr8	143371909	143371968	60	1	12	0	0.55190077	0	9	2	0.0565833	0.61140001
chr8	143371968	143416464	44497	1	12	0	0.72109894	0	8	2	0.03209037	0.61140001
chr8	143416464	143448617	32154	1	11	0	0.55623409	0	8	2	0.05490675	0.61140001
chr8	143448617	143568457	119841	2	11	0	0.41444892	0	9	2	0.09154957	0.61140001
chr8	143568457	143577971	9515	2	11	0	0.30102999	0	10	3	0.14303407	0.93173516
chr8	143577971	143692418	114448	2	11	0	0.41444892	0	9	3	0.09154957	0.93173516
chr8	143692418	143742406	49989	2	12	0	0.41314172	0	10	3	0.09290028	0.93173516
chr8	143742406	143744955	2550	2	12	0	0.21227046	0	12	3	0.21227046	0.93173516
chr8	143744955	143783029	38075	2	12	0	0.41314172	0	10	2	0.09290028	0.61140001
chr8	143783029	143917351	134323	7	12	0	0.14385241	0	13	2	0.30102999	0.61140001
chr8	143917351	144172190	254840	6	12	0	0.09290028	0	14	2	0.41314172	0.61140001
chr8	144172190	144331033	158844	4	15	1	0.30102999	0.05404976	14	2	0.14135546	0.30102999
chr8	144331033	144331088	56	1	15	1	0.20975986	0.05404976	15	2	0.20975986	0.30102999
chr8	144331088	144375125	44038	2	15	1	0.30102999	0.05404976	14	2	0.14135546	0.30102999
chr8	144375125	144466341	91217	3	15	1	0.20975986	0.05404976	15	2	0.20975986	0.30102999
chr8	144466341	144466391	51	1	15	1	0.13872638	0.05404976	16	2	0.30102999	0.30102999
chr8	144466391	144567236	100846	3	15	1	0.20975986	0.05404976	15	2	0.20975986	0.30102999
chr8	144567236	144567295	60	1	15	1	0.13872638	0.05404976	16	2	0.30102999	0.30102999
chr8	144567295	144682427	115133	4	15	1	0.20975986	0.05404976	15	2	0.20975986	0.30102999
chr8	144682427	144682486	60	1	15	1	0.13872638	0.05404976	16	2	0.30102999	0.30102999
chr8	144682486	144732073	49588	3	15	1	0.20975986	0.05404976	15	2	0.20975986	0.30102999
chr8	144732073	144799904	67832	2	15	1	0.30102999	0.05404976	14	2	0.14135546	0.30102999
chr8	144799904	144799957	54	1	16	1	0.4167287	0.05404976	14	2	0.08923391	0.30102999
chr8	144799957	144881451	81495	2	15	1	0.30102999	0.05404976	14	2	0.14135546	0.30102999
chr8	144881451	144990160	108710	6	17	1	0.42015402	0.05404976	15	2	0.08584816	0.30102999
chr8	144990160	145138849	148690	6	18	1	0.5732208	0.05404976	15	2	0.04875589	0.30102999
chr8	145138849	145225218	86370	7	20	1	1.01542894	0.05404976	15	2	0.01077081	0.30102999
chr8	145225218	145324529	99312	1	18	1	0.5732208	0.05404976	15	2	0.04875589	0.30102999
chr8	145324529	145493555	169027	1	18	1	0.74627054	0.05404976	14	2	0.02793176	0.30102999

chr8	145493555	145722243	228689	13	20	1	1.01542894	0.05404976	15	2	0.01077081	0.30102999
chr8	145722243	145777917	55675	3	19	1	1.20557689	0.05404976	13	2	0.00666883	0.30102999
chr8	145777917	145811171	33255	1	18	1	0.9449689	0.05404976	13	2	0.01518174	0.30102999
chr8	145811171	145811230	60	1	18	1	0.74627054	0.05404976	14	2	0.02793176	0.30102999
chr8	145811230	145921823	110594	2	18	1	1.17038931	0.05404976	12	2	0.00778066	0.30102999
chr8	145921823	145921882	60	1	19	1	1.46403142	0.05404976	12	2	0.00317045	0.30102999
chr8	145921882	146005188	83307	2	17	1	0.92532268	0.05404976	12	2	0.01667721	0.30102999
chr8	146005188	146015737	10550	2	18	1	1.17038931	0.05404976	12	2	0.00778066	0.30102999
chr8	146015737	146121090	105354	5	17	1	0.92532268	0.05404976	12	2	0.01667721	0.30102999
chr8	146121090	146157954	36865	1	15	1	0.71538971	0.05404976	11	2	0.0331093	0.30102999
chr8	146157954	146174033	16080	1	13	1	0.41271556	0.05404976	11	2	0.09334429	0.30102999
chr8	146174033	146280020	105988	1	12	1	0.30102999	0.05404976	11	2	0.14385241	0.30102999
chr9	271257	329684	58428	2	2	0	0.30102999	0	1	2	0.05404976	0.61140001
chr9	329684	329743	60	1	3	0	0.10122019	0	4	2	0.30102999	0.61140001
chr9	329743	534531	204789	4	3	0	0.17593012	0	3	2	0.17593012	0.61140001
chr9	534531	866584	332054	7	3	0	0.17593012	0	3	4	0.17593012	1.26272838
chr9	866584	910389	43806	1	3	0	0.17593012	0	3	3	0.17593012	0.93173516
chr9	910389	973803	63415	2	3	1	0.17593012	0.02438896	3	3	0.17593012	0.51676182
chr9	973803	973862	60	1	3	1	0.17593012	0.01091641	3	4	0.17593012	0.76005302
chr9	973862	1079463	105602	2	3	1	0.17593012	0.05404976	3	2	0.17593012	0.30102999
chr9	1079463	1079522	60	1	3	1	0.17593012	0.00478973	3	5	0.17593012	1.02643191
chr9	1079522	1178939	99418	1	3	1	0.17593012	0.01091641	3	4	0.17593012	0.76005302
chr9	1178939	1288054	109116	1	3	0	0.17593012	0	3	4	0.17593012	1.26272838
chr9	1288054	1288113	60	1	4	0	0.30102999	0	3	5	0.10122019	1.60515106
chr9	1288113	1411750	123638	2	4	0	0.30102999	0	3	4	0.10122019	1.26272838
chr9	1411750	1511200	99451	2	4	0	0.18734596	0	4	4	0.18734596	1.26272838
chr9	1511200	1511259	60	1	4	1	0.18734596	0.01091641	4	4	0.18734596	0.76005302
chr9	1511259	1579408	68150	1	4	1	0.30102999	0.01091641	3	4	0.10122019	0.76005302
chr9	1579408	1579467	60	1	4	1	0.30102999	0.00478973	3	5	0.10122019	1.02643191
chr9	1579467	1665294	85828	1	4	0	0.30102999	0	3	4	0.10122019	1.26272838
chr9	1665294	1803603	138310	2	3	0	0.30102999	0	2	4	0.08289318	1.26272838
chr9	1803603	1869792	66190	2	3	1	0.30102999	0.00478973	2	5	0.08289318	1.02643191
chr9	1869792	1869851	60	1	3	1	0.30102999	0.00204627	2	6	0.08289318	1.31360226
chr9	1869851	1950345	80495	1	3	1	0.30102999	0.00478973	2	5	0.08289318	1.02643191
chr9	1950345	2141771	191427	3	3	1	0.30102999	0.01091641	2	4	0.08289318	0.76005302
chr9	2141771	2267812	126042	3	3	1	0.30102999	0.00478973	2	5	0.08289318	1.02643191
chr9	2267812	2267871	60	1	3	1	0.17593012	0.00204627	3	6	0.17593012	1.31360226
chr9	2267871	2385395	117525	1	3	1	0.30102999	0.00204627	2	6	0.08289318	1.31360226
chr9	2385395	2385454	60	1	3	1	0.30102999	8.47E-04	2	7	0.08289318	1.62048027
chr9	2385454	2494816	109363	1	3	1	0.30102999	0.00204627	2	6	0.08289318	1.31360226
chr9	2494816	2494875	60	1	4	1	0.47744371	0.00204627	2	6	0.0429175	1.31360226
chr9	2494875	2737667	242793	4	3	1	0.30102999	0.00204627	2	6	0.08289318	1.31360226
chr9	2737667	2805577	67911	1	3	1	0.30102999	0.00478973	2	5	0.08289318	1.02643191
chr9	2805577	2876716	71140	2	3	2	0.30102999	0.02162467	2	5	0.08289318	0.68214471
chr9	2876716	3168699	291984	5	3	0	0.30102999	0	2	5	0.08289318	1.60515106
chr9	3168699	3323081	154383	4	3	1	0.30102999	0.00204627	2	6	0.08289318	1.31360226
chr9	3323081	3519377	196297	4	3	1	0.30102999	0.00478973	2	5	0.08289318	1.02643191
chr9	3519377	3754972	235596	4	3	2	0.30102999	0.02162467	2	5	0.08289318	0.68214471
chr9	3754972	3755031	60	1	3	2	0.17593012	0.02162467	3	5	0.17593012	0.68214471
chr9	3755031	3896743	141713	2	3	1	0.17593012	0.01091641	3	4	0.17593012	0.76005302
chr9	3896743	3896802	60	1	4	1	0.30102999	0.00478973	3	5	0.10122019	1.02643191
chr9	3896802	3972272	75471	2	3	1	0.17593012	0.02438896	3	3	0.17593012	0.51676182
chr9	3972272	3972331	60	1	4	1	0.30102999	0.02438896	3	3	0.10122019	0.51676182
chr9	3972331	4051530	79200	1	3	1	0.17593012	0.05404976	3	2	0.17593012	0.30102999
chr9	4051530	4116616	65087	1	3	1	0.17593012	0.1218695	3	1	0.17593012	0.1218695
chr9	4116616	4156589	39974	2	3	1	0.10122019	0.02438896	4	3	0.30102999	0.51676182
chr9	4156589	4156648	60	1	3	1	0.10122019	0.01091641	4	4	0.30102999	0.76005302
chr9	4156648	4210276	53629	1	3	1	0.17593012	0.01091641	3	4	0.17593012	0.76005302
chr9	4210276	4210335	60	1	3	2	0.10122019	0.0429175	4	4	0.30102999	0.47744371
chr9	4210335	4277620	67286	1	3	1	0.10122019	0.02438896	4	3	0.30102999	0.51676182
chr9	4277620	4357696	80077	1	3	1	0.17593012	0.05404976	3	2	0.17593012	0.30102999
chr9	4357696	4428574	70879	1	3	0	0.17593012	0	3	2	0.17593012	0.61140001
chr9	4428574	4516059	87486	3	4	0	0.18734596	0	4	2	0.18734596	0.61140001
chr9	4516059	4970093	454035	9	4	0	0.18734596	0	4	1	0.18734596	0.30102999
chr9	4970093	5132181	162089	5	4	1	0.18734596	0.02438896	4	3	0.18734596	0.51676182
chr9	5132181	5291649	159469	2	4	1	0.18734596	0.05404976	4	2	0.18734596	0.30102999
chr9	5291649	5291708	60	1	4	2	0.18734596	0.08289318	4	3	0.18734596	0.30102999
chr9	5291708	5420471	128764	2	4	2	0.30102999	0.08289318	3	3	0.10122019	0.30102999
chr9	5420471	5485609	65139	1	4	2	0.47744371	0.08289318	2	3	0.0429175	0.30102999
chr9	5485609	5558534	72926	2	4	2	0.30102999	0.0429175	3	4	0.10122019	0.47744371
chr9	5558534	5558593	60	1	4	2	0.18734596	0.0429175	4	4	0.18734596	0.47744371
chr9	5558593	5615590	56998	1	4	2	0.30102999	0.0429175	3	4	0.10122019	0.47744371
chr9	5615590	5775569	159980	2	4	1	0.30102999	0.01091641	3	4	0.10122019	0.76005302
chr9	5775569	5827172	51604	2	5	2	0.45545077	0.0429175	3	4	0.05670724	0.47744371
chr9	5827172	5947662	120491	2	4	2	0.30102999	0.0429175	3	4	0.10122019	0.47744371
chr9	5947662	5947721	60	1	5	2	0.45545077	0.0429175	3	4	0.05670724	0.47744371
chr9	5947721	6198539	250819	3	4	2	0.47744371	0.0429175	2	4	0.0429175	0.47744371
chr9	6198539	6198598	60	1	5	2	0.68214471	0.0429175	2	4	0.02162467	0.47744371
chr9	6198598	6330667	132070	3	4	2	0.47744371	0.0429175	2	4	0.0429175	0.47744371
chr9	6330667	6330726	60	1	4	2	0.47744371	0.02162467	2	5	0.0429175	0.68214471
chr9	6330726	6715000	384275	7	3	2	0.30102999	0.0429175	2	4	0.08289318	0.47744371
chr9	6715000	6763489	48490	1	3	2	0.30102999	0.08289318	2	3	0.08289318	0.30102999
chr9	6763489	6813496	50008	1	3	2	0.30102999	0.1575501	2	2	0.08289318	0.1575501
chr9	6813496	6884557	71062	2	4	2	0.47744371	0.08289318	2	3	0.0429175	0.30102999
chr9	6884557	7047206	162650	3	3	2	0.30102999	0.08289318	2	3	0.08289318	0.30102999
chr9	7047206	7202935	155730	3	3	2	0.30102999	0.1575501	2	2	0.08289318	0.1575501
chr9	7202935	7341462	138528	1	3	1	0.30102999	0.05404976	2	2	0.08289318	0.30102999
chr9	7341462	7493625	152164	2	4	1	0.47744371	0.05404976	2	2	0.0429175	0.30102999
chr9	7493625	7493684	60	1	4	1	0.30102999	0.05404976	3	2	0.10122019	0.30102999
chr9	7493684	7608025	114342	1	3	1	0.30102999	0.05404976	2	2	0.08289318	0.30102999
chr9	7608025	7608084	60	1	3	2	0.30102999	0.1575501	2	2	0.08289318	0.1575501
chr9	7608084	7935806	327723	3	3	2	0.51676182	0.1575501	1	2	0.02438896	0.1575501
chr9	7935806	8217768	281963	2	3	2	0.30102999	0.1575501	2	2	0.08289318	0.1575501
chr9	8217768	8364878	147111	3	4	2	0.47744371	0.08289318	2	3	0.0429175	0.30102999
chr9	8364878	8364937	60	1	4	2	0.47744371	0.0429175	2	4	0.0429175	0.47744371
chr9	8364937	8467234	102298	2	4	2	0.76005302	0.0429175	1	4	0.01091641	0.47744371
chr9	8467234	8528790	61557	2	4	2	0.47744371	0.02162467	2	5	0.0429175	0.68214471
chr9	8528790	8528849	60	1	4	2	0.47744371	0.00493743	2	7	0.0429175	1.16581773
chr9	8528849	9363262	834414	16	4	2	0.76005302	0.00493743	1	7	0.01091641	1.16581773
chr9	9363262	9363321	60	1	4	2	0.47744371	0.00493743	2	7	0.0429175	1.16581773
chr9	9363321	9400328	37008	1	4	2	0.47744371	0.01053319	2	6	0.0429175	0.91219088
chr9	9400328	9433647	33320	1	4	2	0.76005302	0.01053319	1	6	0.01091641	0.91219088
chr9	9433647	9514302	80656	1	4	2	0.76005302	0.02162467	1	5	0.01091641	0.68214471
chr9	9514302	9819064	304763	7	4	2	0.30102999	0.02162467	3	5	0.10122019	0.68214471
chr9	9819064	9872132	53069	1	4	1	0.30102999	0.00478973	3	5	0.10122019	1.02643191
chr9	9872132	10060015	187884	4	4	0	0.30102999	0	3	5	0.10122019	1.60515106
chr9	10060015	10060074	60	1	4	1	0.30102999	0.00478973	3	5	0.10122019	1.02643191
chr9	10060074	10212135	152062	3	3	0	0.17593012	0	3	5	0.17593012	1.60515106
chr9	10212135	10286986	74852	1	3	0	0.17593012	0	3	4	0.17593012	1.26272838
chr9	10286986	10372530	85545	2	2	0	0.08289318	0	3	4	0.30102999	1.26272838
chr9	10372530	10372589	60	1	2	1	0.08289318	0.01091641	3	4	0.30102999	0.76005302
chr9	10372589	10554031	181443	3	1	0	0.02438896	0	3	3	0.51676182	0.93173516
chr9	10554031	10581502	27472	2	1	0	0.02438896	0	3	4	0.51676182	1.26272838
chr9	10581502	10686721	105220	1	1	0	0.02438896	0	3	3	0.51676182	0.93173516
chr9	10686721	10786075	99355	1	1	0	0.05404976	0	2	3	0.30102999	0.93173516
chr9	10786075	10882873	96799	2	1	1	0.05404976	0.02438896	2	3	0.30102999	0.51676182
chr9	10882873	10882932	60	1	1	1	0.05404976	0.01091641	2	4	0.30102999	0.76005302
chr9	10882932	11037444	154513	1	1	1	0.1218695	0.02438896	1	3	0.1218695	0.51676182
chr9	11037444	11219608	182165	1	1	1	0.1218695	0.05404976	1	2	0.1218695	0.30102999
chr9	11219608	11457340	237733	1	1	1	0.30102999	0.05404976	0	2	0	0.30102999
chr9	11457340	11573590	116251	2	1	1	0.30102999	0.02438896	0	3	0	0.51676182
chr9	11573590	11760141	186552	2	1	1	0.30102999	0.1218695	0	1	0	0.1218695
chr9	11760141	11760200	60	1	1	1	0.1218695	0.05404976	1	2	0.1218695	0.30102999
chr9	11760200	11818291	58092	1	1	1	0.30102999	0.05404976	0	2	0	0.30102999

chr9	11818291	11818350	60	1	1	1	0.1218695	0.02438896	1	3	0.1218695	0.51676182
chr9	11818350	12196201	377852	3	1	1	0.30102999	0.02438896	0	3	0	0.51676182
chr9	12196201	12275118	78918	1	1	1	0.30102999	0.05404976	0	2	0	0.30102999
chr9	12275118	12275177	60	1	2	2	0.30102999	0.08289318	1	3	0.05404976	0.30102999
chr9	12275177	12405964	130788	1	2	1	0.30102999	0.02438896	1	3	0.05404976	0.51676182
chr9	12405964	12406020	57	1	2	1	0.1575501	0.01091641	2	4	0.1575501	0.76005302
chr9	12406020	12698598	292579	4	2	1	0.30102999	0.02438896	1	3	0.05404976	0.51676182
chr9	12698598	13003652	305055	4	2	1	0.61140001	0.05404976	0	2	0	0.30102999
chr9	13003652	13003711	60	1	2	1	0.61140001	0.02438896	0	3	0	0.51676182
chr9	13003711	13106806	103096	1	2	1	0.61140001	0.05404976	0	2	0	0.30102999
chr9	13106806	13136195	29390	3	2	1	0.30102999	0.02438896	1	3	0.05404976	0.51676182
chr9	13136195	13183475	47281	1	2	1	0.30102999	0.05404976	1	2	0.05404976	0.30102999
chr9	13183475	13217213	33739	2	2	1	0.30102999	0.02438896	1	3	0.05404976	0.51676182
chr9	13217213	13275993	58781	3	2	1	0.30102999	0.01091641	1	4	0.05404976	0.76005302
chr9	13275993	13374245	98253	2	2	2	0.30102999	0.0429175	1	4	0.05404976	0.47744371
chr9	13374245	13595855	221611	3	2	2	0.1575501	0.0429175	2	4	0.1575501	0.47744371
chr9	13595855	13595914	60	1	3	2	0.30102999	0.0429175	2	4	0.08289318	0.47744371
chr9	13595914	13752900	156987	1	3	2	0.30102999	0.08289318	2	3	0.08289318	0.30102999
chr9	13752900	13752959	60	1	3	2	0.30102999	0.0429175	2	4	0.08289318	0.47744371
chr9	13752959	13877280	124322	1	3	2	0.30102999	0.1575501	2	2	0.08289318	0.1575501
chr9	13877280	13877339	60	1	3	2	0.17593012	0.08289318	3	3	0.17593012	0.30102999
chr9	13877339	13959104	81766	1	3	1	0.17593012	0.02438896	3	3	0.17593012	0.51676182
chr9	13959104	13959163	60	1	4	1	0.18734596	0.00478973	4	5	0.18734596	1.02643191
chr9	13959163	14087982	128820	2	4	1	0.18734596	0.02438896	4	3	0.18734596	0.51676182
chr9	14087982	14088041	60	1	6	1	0.20064824	0.01091641	6	4	0.20064824	0.76005302
chr9	14088041	14179792	91752	2	4	1	0.11390336	0.02438896	5	3	0.30102999	0.51676182
chr9	14179792	14210863	31072	1	4	1	0.18734596	0.02438896	4	3	0.18734596	0.51676182
chr9	14210863	14270425	59563	1	3	1	0.17593012	0.02438896	3	3	0.17593012	0.51676182
chr9	14270425	14360221	89797	3	4	1	0.30102999	0.01091641	3	4	0.10122019	0.76005302
chr9	14360221	14397186	36966	1	3	1	0.17593012	0.01091641	3	4	0.17593012	0.76005302
chr9	14397186	14506695	109510	2	3	2	0.17593012	0.0429175	3	4	0.17593012	0.47744371
chr9	14506695	14542140	35446	2	4	4	0.30102999	0.18734596	3	4	0.10122019	0.18734596
chr9	14542140	14610351	68212	1	3	3	0.17593012	0.10122019	3	4	0.17593012	0.30102999
chr9	14610351	14639912	29562	2	3	3	0.17593012	0.05670724	3	5	0.17593012	0.45545077
chr9	14639912	14680180	40269	2	3	4	0.10122019	0.06713722	4	6	0.30102999	0.44141547
chr9	14680180	14720392	40213	1	3	4	0.17593012	0.06713722	3	6	0.17593012	0.44141547
chr9	14720392	14887042	166651	4	3	3	0.17593012	0.03070643	3	6	0.17593012	0.63695542
chr9	14887042	14887101	60	1	3	3	0.10122019	0.03070643	4	6	0.30102999	0.63695542
chr9	14887101	14956477	69377	1	3	2	0.10122019	0.01053319	4	6	0.30102999	0.91219088
chr9	14956477	15040963	84487	1	3	1	0.17593012	0.00204627	3	6	0.17593012	1.31360226
chr9	15040963	15104458	63496	2	3	1	0.10122019	0.00204627	4	6	0.30102999	1.31360226
chr9	15104458	15185751	81294	1	3	1	0.30102999	0.00478973	2	5	0.08289318	1.02643191
chr9	15185751	15267912	82162	2	3	1	0.30102999	0.02438896	2	3	0.08289318	0.51676182
chr9	15267912	15267965	54	1	3	1	0.30102999	0.00478973	2	5	0.08289318	1.02643191
chr9	15267965	15305901	37937	1	3	1	0.51676182	0.01091641	1	4	0.02438896	0.76005302
chr9	15305901	15378135	72235	1	3	1	0.51676182	0.02438896	1	3	0.02438896	0.51676182
chr9	15378135	15378194	60	1	3	2	0.51676182	0.02162467	1	5	0.02438896	0.68214471
chr9	15378194	15433811	55618	1	3	2	0.51676182	0.0429175	1	4	0.02438896	0.47744371
chr9	15433811	15433870	60	1	3	2	0.51676182	0.02162467	1	5	0.02438896	0.68214471
chr9	15433870	15482839	48970	2	3	2	0.51676182	0.0429175	1	4	0.02438896	0.47744371
chr9	15482839	15482898	60	1	3	2	0.30102999	0.02162467	2	5	0.08289318	0.68214471
chr9	15482898	15549731	66834	1	3	2	0.51676182	0.02162467	1	5	0.02438896	0.68214471
chr9	15549731	15624173	74443	3	3	2	0.30102999	0.02162467	2	5	0.08289318	0.68214471
chr9	15624173	16124090	499918	9	3	2	0.51676182	0.02162467	1	5	0.02438896	0.68214471
chr9	16124090	16307883	183794	3	3	1	0.93173516	0.02438896	0	3	0	0.51676182
chr9	16307883	16418754	110872	2	3	1	0.51676182	0.02438896	1	3	0.02438896	0.51676182
chr9	16418754	16434825	16072	2	3	1	0.30102999	0.02438896	2	3	0.08289318	0.51676182
chr9	16434825	16434884	60	1	3	1	0.30102999	0.01091641	2	4	0.08289318	0.76005302
chr9	16434884	16526550	91667	2	3	1	0.30102999	0.02438896	2	3	0.08289318	0.51676182
chr9	16526550	16527656	1107	2	3	1	0.30102999	0.01091641	2	4	0.08289318	0.76005302
chr9	16527656	16593358	65703	1	3	1	0.51676182	0.02438896	1	3	0.02438896	0.51676182
chr9	16593358	16705259	111902	4	4	1	0.76005302	0.02438896	1	3	0.01091641	0.51676182
chr9	16705259	17020094	314836	4	2	1	0.30102999	0.05404976	1	2	0.05404976	0.30102999
chr9	17020094	17076538	56445	2	2	1	0.1575501	0.05404976	2	2	0.1575501	0.30102999
chr9	17076538	17195765	119228	2	2	1	0.30102999	0.05404976	1	2	0.05404976	0.30102999
chr9	17195765	17195824	60	1	2	1	0.30102999	0.02438896	1	3	0.05404976	0.51676182
chr9	17195824	17298850	103027	2	2	1	0.61140001	0.05404976	0	2	0	0.30102999
chr9	17298850	17342315	43466	2	2	1	0.61140001	0.02438896	0	3	0	0.51676182
chr9	17342315	17386482	44168	2	2	1	0.61140001	0.01091641	0	4	0	0.76005302
chr9	17386482	17466848	80367	1	1	1	0.30102999	0.01091641	0	4	0	0.76005302
chr9	17466848	17502885	36038	2	1	2	0.30102999	0.0429175	0	4	0	0.47744371
chr9	17502885	17502944	60	1	2	2	0.61140001	0.0429175	0	4	0	0.47744371
chr9	17502944	17578717	75774	1	2	2	0.61140001	0.08289318	0	3	0	0.30102999
chr9	17578717	17630872	52156	1	1	2	0.30102999	0.08289318	0	3	0	0.30102999
chr9	17630872	17675345	44474	2	1	2	0.30102999	0.0429175	0	4	0	0.47744371
chr9	17675345	17709124	33780	2	2	2	0.61140001	0.0429175	0	4	0	0.47744371
chr9	17709124	17796728	87605	2	2	2	0.61140001	0.08289318	0	3	0	0.30102999
chr9	17796728	17851059	54332	1	2	1	0.61140001	0.02438896	0	3	0	0.51676182
chr9	17851059	17985541	134483	2	2	1	0.30102999	0.02438896	1	3	0.05404976	0.51676182
chr9	17985541	17985600	60	1	2	1	0.08289318	0.02438896	3	3	0.30102999	0.51676182
chr9	17985600	18186747	201148	1	2	1	0.1575501	0.02438896	2	3	0.1575501	0.51676182
chr9	18186747	18186806	60	1	2	1	0.08289318	0.01091641	3	4	0.30102999	0.76005302
chr9	18186806	18288868	102063	1	2	1	0.1575501	0.01091641	2	4	0.1575501	0.76005302
chr9	18288868	18398255	109388	1	2	1	0.30102999	0.02438896	1	3	0.05404976	0.51676182
chr9	18398255	18474451	76197	2	2	1	0.30102999	0.01091641	1	4	0.05404976	0.76005302
chr9	18474451	18533248	58798	2	2	1	0.30102999	0.00478973	1	5	0.05404976	1.02643191
chr9	18533248	18574273	41026	2	2	1	0.0429175	0.00478973	4	5	0.47744371	1.02643191
chr9	18574273	18639234	64962	1	2	1	0.08289318	0.00478973	3	5	0.30102999	1.02643191
chr9	18639234	18681089	41856	2	2	1	0.0429175	0.00478973	4	5	0.47744371	1.02643191
chr9	18681089	18743296	62208	1	2	1	0.1575501	0.00478973	2	5	0.1575501	1.02643191
chr9	18743296	18892980	149685	3	1	1	0.05404976	0.00478973	2	5	0.30102999	1.02643191
chr9	18892980	18950918	57939	1	1	1	0.1218695	0.00478973	1	5	0.1218695	1.02643191
chr9	18950918	19022646	71729	2	1	1	0.1218695	0.01091641	1	4	0.1218695	0.76005302
chr9	19022646	19213822	191177	7	1	1	0.1218695	0.00478973	1	5	0.1218695	1.02643191
chr9	19213822	19213881	60	1	1	1	0.05404976	0.00478973	2	5	0.30102999	1.02643191
chr9	19213881	19296099	82219	1	1	1	0.1218695	0.00478973	1	5	0.1218695	1.02643191
chr9	19296099	19296158	60	1	1	1	0.05404976	0.00478973	2	5	0.30102999	1.02643191
chr9	19296158	19357623	61466	2	1	1	0.1218695	0.00478973	1	5	0.1218695	1.02643191
chr9	19357623	19441347	83725	3	1	1	0.02438896	0.00478973	3	5	0.51676182	1.02643191
chr9	19441347	19511524	70178	3	1	1	0.01091641	0.00478973	4	5	0.76005302	1.02643191
chr9	19511524	19511583	60	1	1	1	0.00204627	0.00478973	6	5	1.31360226	1.02643191
chr9	19511583	19597233	85651	2	1	1	0.00204627	0.01091641	6	4	1.31360226	0.76005302
chr9	19597233	19597292	60	1	1	2	0.00204627	0.02162467	6	5	1.31360226	0.68214471
chr9	19597292	19692238	94947	2	1	1	0.00204627	0.00478973	6	5	1.31360226	1.02643191
chr9	19692238	19785974	93737	2	1	1	0.01091641	0.00478973	4	5	0.76005302	1.02643191
chr9	19785974	19786033	60	1	1	2	0.00478973	0.01053319	5	6	1.02643191	0.91219088
chr9	19786033	19814697	28665	1	1	2	0.02438896	0.02162467	3	5	0.51676182	0.68214471
chr9	19814697	19918661	103965	1	1	2	0.05404976	0.02162467	2	5	0.30102999	0.68214471
chr9	19918661	19993826	75166	2	1	4	0.02438896	0.06713722	3	6	0.51676182	0.44141547
chr9	19993826	20121574	127749	1	1	4	0.05404976	0.06713722	2	6	0.30102999	0.44141547
chr9	20121574	20218996	97423	2	1	4	0.02438896	0.06713722	3	6	0.51676182	0.44141547
chr9	20218996	20385331	166336	3	1	3	0.02438896	0.03070643	3	6	0.51676182	0.63695542
chr9	20385331	20422504	37174	2	1	4	0.01091641	0.06713722	4	6	0.76005302	0.44141547
chr9	20422504	20422563	60	1	1	4	0.00478973	0.06713722	5	6	1.02643191	0.44141547
chr9	20422563	20456703	34141	1	1	4	0.01091641	0.06713722	4	6	0.76005302	0.44141547
chr9	20456703	20509690	52988	2	1	4	0.00478973	0.06713722	5	6	1.02643191	0.44141547
chr9	20509690	20520469	10780	2	1	4	0.00204627	0.06713722	6	6	1.31360226	0.44141547
chr9	20520469	20550077	29609	2	1	5	0.00204627	0.12309572	6	6	1.31360226	0.30102999
chr9	20550077	20874659	324583	9	1	5	8.47E-04	0.12309572	7	6	1.62048027	0.30102999
chr9	20874659	20909825	35167	2	1	6	8.47E-04	0.20064824	7	6	1.62048027	0.20064824
chr9	20909825	21140864	231040	6	1	5	0.00204627	0.12309572	6	6	1.31360226	0.30102999
chr9	21140864	21141927	1064	2	1	6	0.00204627	0.20064824	6	6	1.31360226	0.20064824
chr9	21141927	21347678	205752	6	1	5	0.00204627	0.12309572	6	6	1.31360226	0.30102999
chr9	21347678	21583983	236306	11	1	6	0.00204627	0.20064824	6	6	1.31360226	0.20064824

chr9	21583983	21678040	94058	2	1	6	0.00204627	0.129913	6	7	1.31360226	0.30102999
chr9	21678040	21678099	60	1	1	6	0.00204627	0.08122616	6	8	1.31360226	0.4250187
chr9	21678099	21743410	65312	1	0	6	0	0.08122616	6	8	1.95986592	0.4250187
chr9	21743410	21743469	60	1	0	7	0	0.13499366	6	8	1.95986592	0.30102999
chr9	21743469	21805329	61861	1	0	6	0	0.08122616	6	8	1.95986592	0.4250187
chr9	21805329	21905380	100052	3	0	6	0	0.129913	6	7	1.95986592	0.30102999
chr9	21905380	21905439	60	1	0	6	0	0.08122616	6	8	1.95986592	0.4250187
chr9	21905439	22009029	103591	8	0	5	0	0.07511598	6	7	1.95986592	0.43181735
chr9	22009029	22241139	232111	2	0	4	0	0.03812622	6	7	1.95986592	0.60763643
chr9	22241139	22326224	85086	1	0	4	0	0.03812622	4	7	1.26272838	0.60763643
chr9	22326224	22416608	90385	1	0	3	0	0.05670724	2	5	0.61140001	0.45545077
chr9	22416608	22519885	103278	1	0	1	0	0.00478973	2	5	0.61140001	1.02643191
chr9	22519885	22519944	60	1	0	1	0	8.47E-04	3	7	0.93173516	1.62048027
chr9	22519944	22648510	128567	1	0	1	0	0.00478973	2	5	0.61140001	1.02643191
chr9	22648510	22700103	51594	1	0	1	0	0.01091641	2	4	0.61140001	0.76005302
chr9	22700103	22700162	60	1	0	2	0	0.01053319	2	6	0.61140001	0.91219088
chr9	22700162	22765222	65061	1	0	1	0	0.00204627	2	6	0.61140001	1.31360226
chr9	22765222	22899584	134363	2	0	2	0	0.01053319	2	6	0.61140001	0.91219088
chr9	22899584	23002377	102794	2	0	4	0	0.06713722	2	6	0.61140001	0.44141547
chr9	23002377	23598116	595740	4	0	3	0	0.05670724	1	5	0.30102999	0.45545077
chr9	23598116	23598175	60	1	0	4	0	0.11390336	1	5	0.30102999	0.30102999
chr9	23598175	23690332	92158	2	0	2	0	0.0429175	1	4	0.30102999	0.47744371
chr9	23690332	23789157	98826	3	0	1	0	0.01091641	1	4	0.30102999	0.76005302
chr9	23789157	23933357	144201	4	0	1	0	0.00478973	1	5	0.30102999	1.02643191
chr9	23933357	24146307	212951	2	0	1	0	0.01091641	1	4	0.30102999	0.76005302
chr9	24146307	24146366	60	1	0	2	0	0.0429175	1	4	0.30102999	0.47744371
chr9	24146366	24694811	548446	3	0	1	0	0.05404976	1	2	0.30102999	0.30102999
chr9	24694811	24837887	143077	1	0	1	0	0.1218695	1	1	0.30102999	0.1218695
chr9	24837887	24942407	104521	2	0	1	0	0.30102999	1	0	0.30102999	0
chr9	24942407	25205505	263099	2	0	0	0	0	1	0	0.30102999	0
chr9	25205505	25738780	533276	4	0	1	0	0.1218695	1	1	0.30102999	0.1218695
chr9	25738780	25738839	60	1	0	2	0	0.30102999	1	1	0.30102999	0.05404976
chr9	25738839	26023775	284937	2	0	0	0	0	1	1	0.30102999	0.30102999
chr9	26023775	26096341	72567	2	0	0	0	0	2	5	0.61140001	1.60515106
chr9	26096341	26321885	225545	3	0	1	0	0.00478973	2	5	0.61140001	1.02643191
chr9	26321885	26321944	60	1	0	3	0	0.05670724	2	5	0.61140001	0.45545077
chr9	26321944	26464989	143046	1	0	2	0	0.02162467	2	5	0.61140001	0.68214471
chr9	26464989	26999600	534612	9	1	4	0.05404976	0.06713722	2	6	0.30102999	0.44141547
chr9	26999600	27029070	29471	2	1	4	0.05404976	0.03812622	2	7	0.30102999	0.60763643
chr9	27029070	27230105	201036	6	1	5	0.05404976	0.04407651	2	8	0.30102999	0.58747015
chr9	27230105	27263898	33794	2	1	6	0.05404976	0.08122616	2	8	0.30102999	0.4250187
chr9	27263898	27374468	110571	2	1	5	0.05404976	0.04407651	2	8	0.30102999	0.58747015
chr9	27374468	27415871	41404	2	1	7	0.05404976	0.13499366	2	8	0.30102999	0.30102999
chr9	27415871	27455716	39846	1	1	6	0.05404976	0.08122616	2	8	0.30102999	0.4250187
chr9	27455716	27487943	32228	2	1	6	0.02438896	0.08122616	3	8	0.51676182	0.4250187
chr9	27487943	27524323	36381	1	1	5	0.02438896	0.04407651	3	8	0.51676182	0.58747015
chr9	27524323	27556704	32382	2	1	6	0.02438896	0.08122616	3	8	0.51676182	0.4250187
chr9	27556704	27626876	70173	2	1	6	0.02438896	0.129913	3	7	0.51676182	0.30102999
chr9	27626876	27626935	60	1	1	7	0.02438896	0.13499366	3	8	0.51676182	0.30102999
chr9	27626935	28014370	387436	4	1	6	0.02438896	0.129913	3	7	0.51676182	0.30102999
chr9	28014370	28063806	49437	1	1	6	0.02438896	0.20064824	3	6	0.51676182	0.20064824
chr9	28063806	28139825	76020	1	1	5	0.02438896	0.12309572	3	6	0.51676182	0.30102999
chr9	28139825	28213365	73541	2	1	6	0.02438896	0.129913	3	7	0.51676182	0.30102999
chr9	28213365	28335486	122122	3	1	7	0.02438896	0.20469099	3	7	0.51676182	0.20469099
chr9	28335486	28428131	92646	2	1	7	0.02438896	0.43181735	3	5	0.51676182	0.07511598
chr9	28428131	28462162	34032	2	1	7	0.02438896	0.30102999	3	6	0.51676182	0.129913
chr9	28462162	28519628	57467	1	1	5	0.02438896	0.19510895	3	5	0.51676182	0.19510895
chr9	28519628	28606661	87034	2	1	5	0.02438896	0.30102999	3	4	0.51676182	0.11390336
chr9	28606661	28606720	60	1	1	6	0.02438896	0.44141547	3	4	0.51676182	0.06713722
chr9	28606720	28887418	280699	6	1	4	0.02438896	0.30102999	3	3	0.51676182	0.10122019
chr9	28887418	29022489	135072	2	1	3	0.02438896	0.17593012	3	3	0.51676182	0.17593012
chr9	29022489	29022548	60	1	1	5	0.02438896	0.45545077	3	3	0.51676182	0.05670724
chr9	29022548	29137041	114494	1	0	2	0	0.08289318	3	3	0.93173516	0.30102999
chr9	29137041	29255382	118342	2	0	2	0	0.08289318	2	3	0.61140001	0.30102999
chr9	29255382	29457747	202366	1	0	2	0	0.08289318	1	3	0.30102999	0.30102999
chr9	29457747	29601835	144089	2	0	4	0	0.30102999	1	3	0.30102999	0.10122019
chr9	29601835	29601894	60	1	0	6	0	0.63695542	1	3	0.30102999	0.03070643
chr9	29601894	29753333	151440	1	0	3	0	0.17593012	1	3	0.30102999	0.17593012
chr9	29753333	29753392	60	1	0	3	0	0.10122019	1	4	0.30102999	0.30102999
chr9	29753392	30144402	391011	2	0	2	0	0.08289318	0	3	0	0.30102999
chr9	30144402	30144461	60	1	0	2	0	0.0429175	0	4	0	0.47744371
chr9	30144461	30247394	102934	1	0	2	0	0.08289318	0	3	0	0.30102999
chr9	30247394	30424069	176676	2	0	2	0	0.08289318	1	3	0.30102999	0.30102999
chr9	30424069	30424128	60	1	0	3	0	0.10122019	1	4	0.30102999	0.30102999
chr9	30424128	30660176	236049	3	0	3	0	0.17593012	1	3	0.30102999	0.17593012
chr9	30660176	30660235	60	1	0	4	0	0.30102999	1	3	0.30102999	0.10122019
chr9	30660235	30810232	149998	2	0	3	0	0.30102999	1	2	0.30102999	0.08289318
chr9	30810232	30810291	60	1	0	3	0	0.30102999	2	2	0.61140001	0.08289318
chr9	30810291	31144867	334577	3	0	2	0	0.1575501	2	2	0.61140001	0.1575501
chr9	31144867	31367313	222447	4	1	2	0.05404976	0.1575501	2	2	0.30102999	0.1575501
chr9	31367313	31510599	143287	3	1	2	0.05404976	0.08289318	2	3	0.30102999	0.30102999
chr9	31510599	31642000	131402	1	1	2	0.05404976	0.1575501	2	2	0.30102999	0.1575501
chr9	31642000	31723924	81925	2	1	2	0.01091641	0.1575501	4	2	0.76005302	0.1575501
chr9	31723924	31723983	60	1	1	3	0.01091641	0.30102999	4	2	0.76005302	0.08289318
chr9	31723983	31891415	167433	1	1	2	0.01091641	0.1575501	4	2	0.76005302	0.1575501
chr9	31891415	31891474	60	1	1	2	0.01091641	0.08289318	4	3	0.76005302	0.30102999
chr9	31891474	32030469	138996	1	1	2	0.02438896	0.08289318	3	3	0.51676182	0.30102999
chr9	32030469	32030528	60	1	1	2	0.02438896	0.0429175	3	4	0.51676182	0.47744371
chr9	32030528	32205770	175243	1	1	2	0.02438896	0.08289318	3	3	0.51676182	0.30102999
chr9	32205770	32293241	87472	2	1	2	0.02438896	0.0429175	3	4	0.51676182	0.47744371
chr9	32293241	32293300	60	1	1	2	0.01091641	0.02162467	4	5	0.76005302	0.68214471
chr9	32293300	32342016	48717	1	1	2	0.02438896	0.02162467	3	5	0.51676182	0.68214471
chr9	32342016	32384264	42249	1	1	2	0.02438896	0.0429175	3	4	0.51676182	0.47744371
chr9	32384264	32456057	71794	4	2	2	0.08289318	0.0429175	3	4	0.30102999	0.47744371
chr9	32456057	32486915	30859	2	2	3	0.0429175	0.05670724	4	5	0.47744371	0.45545077
chr9	32486915	32558887	71973	4	2	3	0.08289318	0.10122019	3	4	0.30102999	0.30102999
chr9	32558887	32608748	49862	2	2	3	0.0429175	0.10122019	4	4	0.47744371	0.30102999
chr9	32608748	32721784	113037	2	2	2	0.08289318	0.0429175	3	4	0.30102999	0.47744371
chr9	32721784	32759755	37972	2	2	3	0.0429175	0.10122019	4	4	0.47744371	0.30102999
chr9	32759755	32844623	84869	2	2	2	0.0429175	0.08289318	4	3	0.47744371	0.30102999
chr9	32844623	32844682	60	1	2	2	0.0429175	0.01053319	4	6	0.47744371	0.91219088
chr9	32844682	32982795	138114	2	2	2	0.08289318	0.02162467	3	5	0.30102999	0.68214471
chr9	32982795	33035827	53033	3	2	3	0.08289318	0.03070643	3	6	0.30102999	0.63695542
chr9	33035827	33165475	129649	6	2	4	0.08289318	0.06713722	3	6	0.30102999	0.44141547
chr9	33165475	33248412	82938	1	2	3	0.08289318	0.03070643	3	6	0.30102999	0.63695542
chr9	33248412	33347012	98601	4	3	3	0.10122019	0.03070643	4	6	0.30102999	0.63695542
chr9	33347012	33441267	94256	3	3	3	0.05670724	0.03070643	5	6	0.45545077	0.63695542
chr9	33441267	33473272	32006	2	3	4	0.05670724	0.03812622	5	7	0.45545077	0.60763643
chr9	33473272	33557425	84154	1	2	4	0.0429175	0.03812622	4	7	0.47744371	0.60763643
chr9	33557425	33664927	107503	1	1	4	0.02438896	0.03812622	3	7	0.51676182	0.60763643
chr9	33664927	33777785	112859	3	2	5	0.08289318	0.07511598	3	7	0.30102999	0.43181735
chr9	33777785	33777844	60	1	2	5	0.0429175	0.07511598	4	7	0.47744371	0.43181735
chr9	33777844	33862241	84398	2	1	5	0.01091641	0.07511598	4	7	0.76005302	0.43181735
chr9	33862241	33862300	60	1	2	5	0.0429175	0.07511598	4	7	0.47744371	0.43181735
chr9	33862300	33941822	79523	2	2	5	0.08289318	0.07511598	3	7	0.30102999	0.43181735
chr9	33941822	33941881	60	1	2	5	0.0429175	0.07511598	4	7	0.47744371	0.43181735
chr9	33941881	33971753	29873	1	1	5	0.02438896	0.07511598	3	7	0.51676182	0.43181735
chr9	33971753	34017112	45360	1	1	4	0.02438896	0.03812622	3	7	0.51676182	0.60763643
chr9	34017112	34085974	68863	2	1	4	0.00478973	0.03812622	5	7	1.02643191	0.60763643
chr9	34085974	34086033	60	1	2	4	0.02162467	0.03812622	5	7	0.68214471	0.60763643
chr9	34086033	34115673	29641	1	1	4	0.00478973	0.03812622	5	7	1.02643191	0.60763643
chr9	34115673	34115732	60	1	2	4	0.01053319	0.02074938	6	8	0.91219088	0.79906872
chr9	34115732	34188026	72295	2	2	4	0.01053319	0.03812622	6	7	0.91219088	0.60763643
chr9	34188026	34257917	69892	1	2	4	0.02162467	0.03812622	5	7	0.68214471	0.60763643

chr9	34257917	34257975	59	1	2	5	0.02162467	0.07511598	5	7	0.68214471	0.43181735
chr9	34257975	34310936	52962	1	2	4	0.02162467	0.03812622	5	7	0.68214471	0.60763643
chr9	34310936	34336250	25315	2	2	4	0.01053319	0.03812622	6	7	0.91219088	0.60763643
chr9	34336250	34381835	45586	2	2	5	0.01053319	0.04407651	6	8	0.91219088	0.58747015
chr9	34381835	34418362	36528	1	2	5	0.02162467	0.04407651	5	8	0.68214471	0.58747015
chr9	34418362	34472098	53737	1	2	3	0.02162467	0.0079614	5	8	0.68214471	1.07548421
chr9	34472098	34520945	48848	1	2	3	0.02162467	0.01598258	5	7	0.68214471	0.84395715
chr9	34520945	34588605	67661	3	2	3	0.01053319	0.01598258	6	7	0.91219088	0.84395715
chr9	34588605	34588663	59	1	2	4	0.01053319	0.03812622	6	7	0.91219088	0.60763643
chr9	34588663	34618506	29844	3	1	3	0.00204627	0.01598258	6	7	1.31360226	0.84395715
chr9	34618506	34690988	72483	8	1	4	0.00204627	0.03812622	6	7	1.31360226	0.60763643
chr9	34690988	34745832	54845	2	2	4	0.01053319	0.03812622	6	7	0.91219088	0.60763643
chr9	34745832	34866614	120783	1	2	3	0.01053319	0.01598258	6	7	0.91219088	0.84395715
chr9	34866614	34996134	129521	4	2	3	0.01053319	0.0079614	6	8	0.91219088	1.07548421
chr9	34996134	35090704	94571	5	2	3	0.01053319	0.01598258	6	7	0.91219088	0.84395715
chr9	35090704	35196492	105789	6	2	3	0.00493743	0.0079614	7	8	1.16581773	1.07548421
chr9	35196492	35277729	81238	3	2	4	0.00493743	0.02074938	7	8	1.16581773	0.79906872
chr9	35277729	35404384	126656	3	2	4	0.00493743	0.03812622	7	7	1.16581773	0.60763643
chr9	35404384	35404443	60	1	2	4	0.00493743	0.02074938	7	8	1.16581773	0.79906872
chr9	35404443	35664900	260458	7	2	4	0.00493743	0.03812622	7	7	1.16581773	0.60763643
chr9	35664900	35708126	43227	5	2	4	0.00493743	0.06713722	7	6	1.16581773	0.44141547
chr9	35708126	36100542	392417	16	2	4	0.00493743	0.03812622	7	7	1.16581773	0.60763643
chr9	36100542	36171237	70696	2	1	4	0.00204627	0.03812622	6	7	1.31360226	0.60763643
chr9	36171237	36344858	173622	5	2	4	0.01053319	0.03812622	6	7	0.91219088	0.60763643
chr9	36344858	36589598	244741	4	2	4	0.02162467	0.03812622	5	7	0.68214471	0.60763643
chr9	36589598	36657289	67692	3	2	4	0.01053319	0.03812622	6	7	0.91219088	0.60763643
chr9	36657289	36669396	12108	1	2	4	0.02162467	0.03812622	5	7	0.68214471	0.60763643
chr9	36669396	36749860	80465	1	1	4	0.00478973	0.06713722	5	6	1.02643191	0.44141547
chr9	36749860	36846481	96622	1	1	2	0.00478973	0.0429175	5	4	1.02643191	0.47744371
chr9	36846481	36893592	47112	2	1	3	0.00204627	0.05670724	6	5	1.31360226	0.45545077
chr9	36893592	36940263	46672	2	1	5	0.00204627	0.19510895	6	5	1.31360226	0.19510895
chr9	36940263	36992764	52502	2	1	6	0.00204627	0.30102999	6	5	1.31360226	0.12309572
chr9	36992764	37087995	95232	2	1	4	0.00204627	0.18734596	6	4	1.31360226	0.18734596
chr9	37087995	37088054	60	1	1	5	0.00204627	0.30102999	6	4	1.31360226	0.11390336
chr9	37088054	37156220	68167	2	1	4	0.00204627	0.18734596	6	4	1.31360226	0.18734596
chr9	37156220	37186847	30628	1	1	4	0.00478973	0.18734596	5	4	1.02643191	0.18734596
chr9	37186847	37288449	101603	2	1	4	0.00478973	0.30102999	5	3	1.02643191	0.10122019
chr9	37288449	37327772	39324	1	1	4	0.00478973	0.47744371	5	2	1.02643191	0.0429175
chr9	37327772	37327830	59	1	1	4	0.00478973	0.30102999	5	3	1.02643191	0.10122019
chr9	37327830	37460967	133138	3	1	2	0.01091641	0.1575501	4	2	0.76005302	0.1575501
chr9	37460967	37501694	40728	3	1	2	0.00478973	0.1575501	5	2	1.02643191	0.1575501
chr9	37501694	37501753	60	1	1	2	0.00204627	0.08289318	6	3	1.31360226	0.30102999
chr9	37501753	37596391	94639	2	1	1	0.00478973	0.1218695	5	1	1.02643191	0.1218695
chr9	37596391	37660586	64196	1	1	1	0.01091641	0.1218695	4	1	0.76005302	0.1218695
chr9	37660586	37711328	50743	1	0	1	0	0.1218695	4	1	1.26272838	0.1218695
chr9	37711328	37760851	49524	2	0	2	0	0.08289318	4	3	1.26272838	0.30102999
chr9	37760851	37767193	6343	2	0	2	0	0.08289318	5	3	1.60515106	0.30102999
chr9	37767193	37857269	90077	3	0	1	0	0.05404976	4	2	1.26272838	0.30102999
chr9	37857269	37857328	60	1	1	1	0.00478973	0.05404976	5	2	1.02643191	0.30102999
chr9	37857328	37960064	102737	3	1	1	0.01091641	0.05404976	4	2	0.76005302	0.30102999
chr9	37960064	38143707	183644	3	1	1	0.01091641	0.1218695	4	1	0.76005302	0.1218695
chr9	38143707	38205685	61979	1	1	1	0.02438896	0.1218695	3	1	0.51676182	0.1218695
chr9	38205685	38663271	457587	8	0	1	0	0.1218695	3	1	0.93173516	0.1218695
chr9	38663271	38768291	105021	1	0	1	0	0.30102999	3	0	0.93173516	0
chr9	38768291	39156954	388664	2	0	0	0	0	1	0	0.30102999	0
chr9	71035346	71095721	60376	2	1	0	0.05404976	0	2	2	0.30102999	0.61140001
chr9	71095721	71217555	121835	4	1	0	0.05404976	0	2	3	0.30102999	0.93173516
chr9	71217555	71491639	274085	6	1	0	0.05404976	0	2	4	0.30102999	1.26272838
chr9	71491639	71491698	60	1	2	0	0.1575501	0	2	4	0.1575501	1.26272838
chr9	71491698	71649155	157458	4	1	0	0.05404976	0	2	4	0.30102999	1.26272838
chr9	71649155	71917699	268545	6	3	0	0.30102999	0	2	4	0.08289318	1.26272838
chr9	71917699	71977019	59321	2	3	1	0.30102999	0.01091641	2	4	0.08289318	0.76005302
chr9	71977019	71994158	17140	2	3	1	0.17593012	0.01091641	3	4	0.17593012	0.76005302
chr9	71994158	72083648	89491	3	2	1	0.08289318	0.01091641	3	4	0.30102999	0.76005302
chr9	72083648	72083707	60	1	3	1	0.17593012	0.01091641	3	4	0.17593012	0.76005302
chr9	72083707	72137166	53460	1	2	1	0.08289318	0.01091641	3	4	0.30102999	0.76005302
chr9	72137166	72222462	85297	2	2	1	0.1575501	0.01091641	2	4	0.1575501	0.76005302
chr9	72222462	72272234	49773	1	1	1	0.05404976	0.01091641	2	4	0.30102999	0.76005302
chr9	72272234	72272293	60	1	2	1	0.1575501	0.01091641	2	4	0.1575501	0.76005302
chr9	72272293	72441389	169097	3	1	1	0.05404976	0.01091641	2	4	0.30102999	0.76005302
chr9	72441389	72464871	23483	2	2	1	0.1575501	0.01091641	2	4	0.1575501	0.76005302
chr9	72464871	72464930	60	1	4	1	0.47744371	0.01091641	2	4	0.0429175	0.76005302
chr9	72464930	72702982	238053	3	3	1	0.30102999	0.01091641	2	4	0.08289318	0.76005302
chr9	72702982	72703041	60	1	4	1	0.30102999	0.01091641	3	4	0.10122019	0.76005302
chr9	72703041	72758637	55597	2	4	1	0.47744371	0.01091641	2	4	0.0429175	0.76005302
chr9	72758637	72937200	178564	3	3	1	0.30102999	0.01091641	2	4	0.08289318	0.76005302
chr9	72937200	72968479	31280	2	3	1	0.17593012	0.01091641	3	4	0.17593012	0.76005302
chr9	72968479	73309372	340894	7	2	1	0.08289318	0.01091641	3	4	0.30102999	0.76005302
chr9	73309372	73462272	152901	4	1	1	0.02438896	0.01091641	3	4	0.51676182	0.76005302
chr9	73462272	73462331	60	1	1	1	0.02438896	0.00478973	3	5	0.51676182	1.02643191
chr9	73462331	73588788	126458	4	1	1	0.02438896	0.01091641	3	4	0.51676182	0.76005302
chr9	73588788	73688965	100178	3	1	1	0.02438896	0.00478973	3	5	0.51676182	1.02643191
chr9	73688965	73736251	47287	2	2	1	0.08289318	0.00478973	3	5	0.30102999	1.02643191
chr9	73736251	73861618	125368	2	1	1	0.02438896	0.01091641	3	4	0.51676182	0.76005302
chr9	73861618	74298697	437080	9	1	1	0.02438896	0.00478973	3	5	0.51676182	1.02643191
chr9	74298697	74340500	41804	1	1	1	0.02438896	0.01091641	3	4	0.51676182	0.76005302
chr9	74340500	74360324	19825	2	2	1	0.08289318	0.01091641	3	4	0.30102999	0.76005302
chr9	74360324	74360381	58	1	2	1	0.08289318	0.00478973	3	5	0.30102999	1.02643191
chr9	74360381	74481204	120824	2	1	1	0.02438896	0.00478973	3	5	0.51676182	1.02643191
chr9	74481204	74587593	106390	4	1	1	0.01091641	0.00478973	4	5	0.76005302	1.02643191
chr9	74587593	74666008	78416	2	1	1	0.00478973	0.00478973	5	5	1.02643191	1.02643191
chr9	74666008	74666067	60	1	2	1	0.02162467	0.00478973	5	5	0.68214471	1.02643191
chr9	74666067	74810467	144401	3	2	1	0.0429175	0.00478973	4	5	0.47744371	1.02643191
chr9	74810467	74866180	55714	1	2	1	0.08289318	0.00478973	3	5	0.30102999	1.02643191
chr9	74866180	74902358	36179	2	2	1	0.0429175	0.00478973	4	5	0.47744371	1.02643191
chr9	74902358	74902417	60	1	2	1	0.02162467	0.00478973	5	5	0.68214471	1.02643191
chr9	74902417	74976850	74434	3	2	1	0.0429175	0.00478973	4	5	0.47744371	1.02643191
chr9	74976850	75081164	104315	2	2	1	0.0429175	0.01091641	4	4	0.47744371	0.76005302
chr9	75081164	75162520	81357	2	2	1	0.0429175	0.02438896	4	3	0.47744371	0.51676182
chr9	75162520	75162579	60	1	2	2	0.0429175	0.02162467	4	5	0.47744371	0.68214471
chr9	75162579	75242873	80295	2	2	1	0.0429175	0.02438896	4	3	0.47744371	0.51676182
chr9	75242873	75276569	33697	1	2	1	0.08289318	0.02438896	3	3	0.30102999	0.51676182
chr9	75276569	75315456	38888	2	2	2	0.08289318	0.08289318	3	3	0.30102999	0.30102999
chr9	75315456	75315515	60	1	2	2	0.0429175	0.08289318	4	3	0.47744371	0.30102999
chr9	75315515	75354943	39429	1	2	0	0.0429175	0	4	3	0.47744371	0.93173516
chr9	75354943	75355001	59	1	3	0	0.10122019	0	4	3	0.30102999	0.93173516
chr9	75355001	75451157	96157	2	3	0	0.17593012	0	3	3	0.17593012	0.93173516
chr9	75451157	75451216	60	1	3	1	0.10122019	0.02438896	4	3	0.30102999	0.51676182
chr9	75451216	75487096	35881	1	3	0	0.10122019	0	4	3	0.30102999	0.93173516
chr9	75487096	75555121	68026	2	1	0	0.01091641	0	4	3	0.76005302	0.93173516
chr9	75555121	75555180	60	1	2	1	0.0429175	0.02438896	4	3	0.47744371	0.51676182
chr9	75555180	75759304	204125	4	1	0	0.02438896	0	3	3	0.51676182	0.93173516
chr9	75759304	75759363	60	1	1	1	0.02438896	0.01091641	3	4	0.51676182	0.76005302
chr9	75759363	75893526	134164	2	1	0	0.02438896	0	3	3	0.51676182	0.93173516
chr9	75893526	75893585	60	1	2	1	0.08289318	0.01091641	3	4	0.30102999	0.76005302
chr9	75893585	76321602	428018	4	1	0	0.1218695	0	1	3	0.1218695	0.93173516
chr9	76321602	76413786	92185	2	1	0	0.05404976	0	2	3	0.30102999	0.93173516
chr9	76413786	76508702	94917	2	2	0	0.08289318	0	3	3	0.30102999	0.93173516
chr9	76508702	76508761	60	1	2	1	0.08289318	0.02438896	3	3	0.30102999	0.51676182
chr9	76508761	76998451	489691	3	2	0	0.1575501	0	2	3	0.1575501	0.93173516
chr9	76998451	76998510	60	1	2	0	0.1575501	0	2	4	0.1575501	1.26272838
chr9	76998510	77089238	90729	1	2	0	0.1575501	0	2	3	0.1575501	0.93173516
chr9	77089238	77211373	122136	4	2	0	0.08289318	0	3	3	0.30102999	0.93173516
chr9	77211373	77563714	352342	8	2	0	0.1575501	0	2	3	0.1575501	0.93173516

chr9	77563714	78004387	440674	10	2	0	0.08289318	0	3	3	0.30102999	0.93173516
chr9	78004387	78086127	81741	2	3	0	0.17593012	0	3	3	0.17593012	0.93173516
chr9	78086127	78086186	60	1	3	0	0.10122019	0	4	3	0.30102999	0.93173516
chr9	78086186	78307262	221077	1	3	0	0.17593012	0	3	3	0.17593012	0.93173516
chr9	78307262	78547409	240148	2	2	0	0.08289318	0	3	3	0.30102999	0.93173516
chr9	78547409	78702938	155530	3	2	0	0.1575501	0	2	3	0.1575501	0.93173516
chr9	78702938	79528912	825975	18	2	0	0.1575501	0	2	2	0.1575501	0.61140001
chr9	79528912	79583399	54488	2	3	0	0.30102999	0	2	2	0.08289318	0.61140001
chr9	79583399	80356196	772798	16	2	0	0.1575501	0	2	2	0.1575501	0.61140001
chr9	80356196	80804457	448262	7	2	0	0.1575501	0	2	1	0.1575501	0.30102999
chr9	80804457	80915162	110706	3	2	0	0.1575501	0	2	3	0.1575501	0.93173516
chr9	80915162	81186684	271523	3	1	0	0.05404976	0	2	2	0.30102999	0.61140001
chr9	81186684	81400580	213897	2	1	0	0.1218695	0	1	2	0.1218695	0.61140001
chr9	81400580	81488146	87567	2	2	0	0.1575501	0	2	2	0.1575501	0.61140001
chr9	81488146	81488205	60	1	2	0	0.1575501	0	2	3	0.1575501	0.93173516
chr9	81488205	81596633	108429	1	2	0	0.1575501	0	2	2	0.1575501	0.61140001
chr9	81596633	81978151	381519	3	2	0	0.30102999	0	1	2	0.05404976	0.61140001
chr9	81978151	82269003	290853	5	2	1	0.30102999	0.02438896	1	3	0.05404976	0.51676182
chr9	82269003	82355013	86011	2	1	1	0.1218695	0.02438896	1	3	0.1218695	0.51676182
chr9	82355013	82355072	60	1	2	1	0.1575501	0.02438896	2	3	0.1575501	0.51676182
chr9	82355072	82449959	94888	1	1	0	0.05404976	0	2	2	0.30102999	0.61140001
chr9	82449959	83002927	552969	7	1	0	0.05404976	0	2	3	0.30102999	0.93173516
chr9	83002927	83002986	60	1	1	1	0.05404976	0.01091641	2	4	0.30102999	0.76005302
chr9	83002986	83175374	172389	2	1	0	0.05404976	0	2	2	0.30102999	0.61140001
chr9	83175374	83691625	516252	5	1	0	0.05404976	0	2	1	0.30102999	0.30102999
chr9	83691625	83959649	268025	2	1	0	0.05404976	0	2	3	0.30102999	0.93173516
chr9	83959649	84115644	155996	2	1	0	0.05404976	0	2	1	0.30102999	0.30102999
chr9	84115644	84198666	83023	2	2	0	0.1575501	0	2	2	0.1575501	0.61140001
chr9	84198666	84782517	583852	12	2	0	0.1575501	0	2	3	0.1575501	0.93173516
chr9	84782517	84782576	60	1	2	1	0.1575501	0.02438896	2	3	0.1575501	0.51676182
chr9	84782576	85032783	250208	2	2	0	0.1575501	0	2	2	0.1575501	0.61140001
chr9	85032783	85070123	37341	2	2	1	0.1575501	0.01091641	2	4	0.1575501	0.76005302
chr9	85070123	85070182	60	1	2	2	0.1575501	0.0429175	2	4	0.1575501	0.47744371
chr9	85070182	85409863	339682	4	2	1	0.1575501	0.01091641	2	4	0.1575501	0.76005302
chr9	85409863	85409922	60	1	2	2	0.1575501	0.0429175	2	4	0.1575501	0.47744371
chr9	85409922	85605326	195405	2	2	1	0.1575501	0.01091641	2	4	0.1575501	0.76005302
chr9	85605326	85640798	35473	2	2	2	0.1575501	0.0429175	2	4	0.1575501	0.47744371
chr9	85640798	85753828	113031	2	2	2	0.1575501	0.08289318	2	3	0.1575501	0.30102999
chr9	85753828	85857357	103530	2	2	2	0.1575501	0.0429175	2	4	0.1575501	0.47744371
chr9	85857357	86188974	331618	7	2	1	0.1575501	0.01091641	2	4	0.1575501	0.76005302
chr9	86188974	86248532	59559	1	2	0	0.1575501	0	2	4	0.1575501	1.26272838
chr9	86248532	86356860	108329	3	2	0	0.1575501	0	2	3	0.1575501	0.93173516
chr9	86356860	86496264	139405	4	2	0	0.1575501	0	2	4	0.1575501	1.26272838
chr9	86496264	86496323	60	1	2	2	0.1575501	0.0429175	2	4	0.1575501	0.47744371
chr9	86496323	86570445	74123	2	2	0	0.1575501	0	2	4	0.1575501	1.26272838
chr9	86570445	86592592	22148	3	2	2	0.1575501	0.0429175	2	4	0.1575501	0.47744371
chr9	86592592	87039456	446865	7	2	1	0.1575501	0.01091641	2	4	0.1575501	0.76005302
chr9	87039456	87430380	390925	6	2	2	0.1575501	0.0429175	2	4	0.1575501	0.47744371
chr9	87430380	87476029	45650	1	2	1	0.1575501	0.01091641	2	4	0.1575501	0.76005302
chr9	87476029	87595872	119844	2	2	1	0.1575501	0.02438896	2	3	0.1575501	0.51676182
chr9	87595872	87654624	58753	2	2	0	0.1575501	0	2	2	0.1575501	0.61140001
chr9	87654624	87926084	271461	3	2	1	0.1575501	0.02438896	2	3	0.1575501	0.51676182
chr9	87926084	87926143	60	1	2	1	0.1575501	0.01091641	2	4	0.1575501	0.76005302
chr9	87926143	88142954	216812	2	2	1	0.1575501	0.02438896	2	3	0.1575501	0.51676182
chr9	88142954	88167757	24804	2	2	1	0.1575501	0.01091641	2	4	0.1575501	0.76005302
chr9	88167757	88202561	34805	1	2	1	0.1575501	0.02438896	2	3	0.1575501	0.51676182
chr9	88202561	88202620	60	1	2	2	0.1575501	0.08289318	2	3	0.1575501	0.30102999
chr9	88202620	88298964	96345	3	2	2	0.1575501	0.1575501	2	2	0.1575501	0.1575501
chr9	88298964	88345162	46199	2	2	2	0.1575501	0.08289318	2	3	0.1575501	0.30102999
chr9	88345162	88713319	368158	8	2	1	0.1575501	0.05404976	2	2	0.1575501	0.30102999
chr9	88713319	89077436	364118	9	2	1	0.08289318	0.05404976	3	2	0.30102999	0.30102999
chr9	89077436	89346554	269119	2	2	0	0.08289318	0	3	2	0.30102999	0.61140001
chr9	89346554	89589995	243442	3	2	0	0.1575501	0	2	1	0.1575501	0.30102999
chr9	89589995	89590054	60	1	3	1	0.17593012	0.05404976	3	2	0.17593012	0.30102999
chr9	89590054	89655874	65821	1	3	1	0.30102999	0.05404976	2	2	0.08289318	0.30102999
chr9	89655874	89805789	149916	4	3	2	0.30102999	0.1575501	2	2	0.08289318	0.1575501
chr9	89805789	89947529	141741	1	3	2	0.30102999	0.30102999	2	1	0.08289318	0.05404976
chr9	89947529	90033495	85967	1	3	1	0.30102999	0.1218695	2	1	0.08289318	0.1218695
chr9	90033495	90100688	67194	2	3	1	0.30102999	0.05404976	2	2	0.08289318	0.30102999
chr9	90100688	90132550	31863	2	3	2	0.30102999	0.1575501	2	2	0.08289318	0.1575501
chr9	90132550	90216070	83521	2	3	1	0.30102999	0.05404976	2	2	0.08289318	0.30102999
chr9	90216070	90342208	126139	4	3	3	0.30102999	0.30102999	2	2	0.08289318	0.08289318
chr9	90342208	90503818	161611	4	3	3	0.30102999	0.17593012	2	3	0.08289318	0.17593012
chr9	90503818	90619639	115822	2	3	2	0.30102999	0.08289318	2	3	0.08289318	0.30102999
chr9	90619639	90721528	101890	2	2	2	0.1575501	0.1575501	2	2	0.1575501	0.1575501
chr9	90721528	90803353	81826	2	2	2	0.1575501	0.08289318	2	3	0.1575501	0.30102999
chr9	90803353	91007979	204627	4	2	2	0.1575501	0.0429175	2	4	0.1575501	0.47744371
chr9	91007979	91093348	85370	3	2	2	0.1575501	0.02162467	2	5	0.1575501	0.68214471
chr9	91093348	91093407	60	1	2	3	0.1575501	0.05670724	2	5	0.1575501	0.45545077
chr9	91093407	91190032	96626	3	1	3	0.05404976	0.05670724	2	5	0.30102999	0.45545077
chr9	91190032	91680525	490494	8	1	2	0.05404976	0.02162467	2	5	0.30102999	0.68214471
chr9	91680525	91770153	89629	2	1	1	0.05404976	0.02438896	2	3	0.30102999	0.51676182
chr9	91770153	91770201	49	1	1	2	0.05404976	0.0429175	2	4	0.30102999	0.47744371
chr9	91770201	92014232	244032	8	1	1	0.05404976	0.02438896	2	3	0.30102999	0.51676182
chr9	92014232	92221377	207146	3	1	1	0.05404976	0.05404976	2	2	0.30102999	0.30102999
chr9	92221377	92297837	76461	3	1	2	0.05404976	0.08289318	2	3	0.30102999	0.30102999
chr9	92297837	92484319	186483	1	1	2	0.1218695	0.08289318	1	3	0.1218695	0.30102999
chr9	92484319	92484378	60	1	1	2	0.1218695	0.02162467	1	5	0.1218695	0.68214471
chr9	92484378	92751038	266661	1	1	1	0.1218695	0.02438896	1	3	0.1218695	0.51676182
chr9	92751038	92751097	60	1	1	1	0.05404976	0.02438896	2	3	0.30102999	0.51676182
chr9	92751097	92875550	124454	1	1	0	0.05404976	0	2	3	0.30102999	0.93173516
chr9	92875550	92965804	90255	1	1	0	0.05404976	0	2	1	0.30102999	0.30102999
chr9	92965804	93372163	406360	4	1	0	0.05404976	0	2	0	0.30102999	0
chr9	93372163	93480848	108686	3	1	1	0.05404976	0.30102999	2	0	0.30102999	0
chr9	93480848	93480907	60	1	1	1	0.05404976	0.05404976	2	2	0.30102999	0.30102999
chr9	93480907	93564261	83355	1	1	0	0.1218695	0	1	2	0.1218695	0.61140001
chr9	93564261	93626871	62611	2	1	0	0.1218695	0	1	3	0.1218695	0.93173516
chr9	93626871	93979576	352706	5	1	1	0.05404976	0.02438896	2	3	0.30102999	0.51676182
chr9	93979576	94087595	108020	3	1	2	0.05404976	0.08289318	2	3	0.30102999	0.30102999
chr9	94087595	94087654	60	1	1	2	0.05404976	0.0429175	2	4	0.30102999	0.47744371
chr9	94087654	94176162	88509	2	1	2	0.05404976	0.08289318	2	3	0.30102999	0.30102999
chr9	94176162	94358815	182654	2	1	1	0.05404976	0.02438896	2	3	0.30102999	0.51676182
chr9	94358815	94358874	60	1	1	1	0.05404976	0.01091641	2	4	0.30102999	0.76005302
chr9	94358874	94439471	80598	2	1	1	0.05404976	0.02438896	2	3	0.30102999	0.51676182
chr9	94439471	94485287	45817	2	1	1	0.05404976	0.01091641	2	4	0.30102999	0.76005302
chr9	94485287	94649669	164383	3	1	1	0.05404976	0.02438896	2	3	0.30102999	0.51676182
chr9	94649669	94649728	60	1	1	2	0.05404976	0.08289318	2	3	0.30102999	0.30102999
chr9	94649728	94702713	52986	1	1	2	0.1218695	0.08289318	1	3	0.1218695	0.30102999
chr9	94702713	94768966	66254	2	1	2	0.1218695	0.0429175	1	4	0.1218695	0.47744371
chr9	94768966	94769025	60	1	1	2	0.1218695	0.01053319	1	6	0.1218695	0.91219088
chr9	94769025	94841748	72724	3	1	2	0.1218695	0.02162467	1	5	0.1218695	0.68214471
chr9	94841748	94874944	33197	2	1	2	0.05404976	0.01053319	2	6	0.30102999	0.91219088
chr9	94874944	94946306	71363	2	1	1	0.05404976	0.00204627	2	6	0.30102999	1.31360226
chr9	94946306	95009801	63496	2	1	1	0.05404976	0.00478973	2	5	0.30102999	1.02643191
chr9	95009801	95055693	45893	2	1	1	0.05404976	0.00204627	2	6	0.30102999	1.31360226
chr9	95055693	95183326	127634	6	1	2	0.05404976	0.01053319	2	6	0.30102999	0.91219088
chr9	95183326	95228774	45449	1	1	1	0.05404976	0.00204627	2	6	0.30102999	1.31360226
chr9	95228774	95780412	551639	14	1	1	0.1218695	0.00204627	1	6	0.1218695	1.31360226
chr9	95780412	95780465	54	1	1	1	0.1218695	8.47E-04	1	7	0.1218695	1.62048027
chr9	95780465	95936288	155824	4	1	1	0.1218695	0.00204627	1	6	0.1218695	1.31360226
chr9	95936288	96000537	64250	2	1	2	0.1218695	0.01053319	1	6	0.1218695	0.91219088
chr9	96000537	96000591	55	1	1	4	0.1218695	0.06713722	1	6	0.1218695	0.44141547
chr9	96000591	96031663	31073	1	1	3	0.1218695	0.03070643	1	6	0.1218695	0.63695542
chr9	96031663	96080473	48811	1	1	2	0.1218695	0.01053319	1	6	0.1218695	0.91219088

chr9	96080473	96080532	60	1	1	3	0.1218695	0.03070643	1	6	0.1218695	0.63695542
chr9	96080532	96191008	110477	3	1	2	0.1218695	0.01053319	1	6	0.1218695	0.91219088
chr9	96191008	96285544	94537	4	1	3	0.1218695	0.01598258	1	7	0.1218695	0.84395715
chr9	96285544	96285603	60	1	1	3	0.1218695	0.0079614	1	8	0.1218695	1.07548421
chr9	96285603	96447813	162211	3	1	3	0.1218695	0.01598258	1	7	0.1218695	0.84395715
chr9	96447813	96681493	233681	2	1	2	0.1218695	0.00493743	1	7	0.1218695	1.16581773
chr9	96681493	96794163	112671	2	1	1	0.1218695	0.00204627	1	6	0.1218695	1.31360226
chr9	96794163	96827115	32953	2	1	2	0.1218695	0.00493743	1	7	0.1218695	1.16581773
chr9	96827115	96888888	61774	1	1	2	0.1218695	0.01053319	1	6	0.1218695	0.91219088
chr9	96888888	96932958	44071	2	1	2	0.1218695	0.00493743	1	7	0.1218695	1.16581773
chr9	96932958	96933017	60	1	1	3	0.1218695	0.01598258	1	7	0.1218695	0.84395715
chr9	96933017	97023815	90799	2	1	3	0.30102999	0.01598258	0	7	0	0.84395715
chr9	97023815	97055632	31818	2	1	2	0.30102999	0.00493743	0	7	0	1.16581773
chr9	97055632	97223165	167534	2	1	2	0.30102999	0.01053319	0	6	0	0.91219088
chr9	97223165	97320282	97118	3	1	3	0.30102999	0.03070643	0	6	0	0.63695542
chr9	97320282	97375641	55360	2	1	2	0.30102999	0.02162467	0	5	0	0.68214471
chr9	97375641	97380088	4448	2	1	2	0.30102999	0.01053319	0	6	0	0.91219088
chr9	97380088	97387158	7071	1	1	1	0.30102999	0.00478973	0	5	0	1.02643191
chr9	97387158	97522014	134857	2	1	0	0.30102999	0	0	5	0	1.60515106
chr9	97522014	97522073	60	1	2	1	0.61140001	0.00478973	0	5	0	1.02643191
chr9	97522073	97587748	65676	2	2	0	0.61140001	0	0	5	0	1.60515106
chr9	97587748	97587807	60	1	2	0	0.30102999	0	1	5	0.05404976	1.60515106
chr9	97587807	97827936	240130	5	2	0	0.61140001	0	0	5	0	1.60515106
chr9	97827936	97843085	15150	2	2	1	0.61140001	0.00478973	0	5	0	1.02643191
chr9	97843085	98078300	235216	5	2	1	0.61140001	0.02438896	0	3	0	0.51676182
chr9	98078300	98078359	60	1	2	1	0.61140001	0.01091641	0	4	0	0.76005302
chr9	98078359	98200891	122533	2	2	0	0.61140001	0	0	4	0	1.26272838
chr9	98200891	98200950	60	1	2	0	0.61140001	0	0	5	0	1.60515106
chr9	98200950	98233617	32668	1	1	0	0.30102999	0	0	5	0	1.60515106
chr9	98233617	98373804	140188	3	1	0	0.30102999	0	0	6	0	1.95986592
chr9	98373804	98513438	139635	2	0	0	0	0	0	6	0	1.95986592
chr9	98513438	99160554	647117	11	0	0	0	0	0	5	0	1.60515106
chr9	99160554	99178917	18364	2	0	0	0	0	0	4	0	1.26272838
chr9	99178917	99178976	60	1	0	0	0	0	0	5	0	1.60515106
chr9	99178976	99223270	44295	1	0	0	0	0	0	4	0	1.26272838
chr9	99223270	99343936	120667	3	0	1	0	0.01091641	0	4	0	0.76005302
chr9	99343936	99343995	60	1	0	1	0	0.00478973	0	5	0	1.02643191
chr9	99343995	99373477	29483	1	0	0	0	0	0	5	0	1.60515106
chr9	99373477	99443439	69963	1	0	0	0	0	0	4	0	1.26272838
chr9	99443439	99479475	36037	2	0	1	0	0.01091641	0	4	0	0.76005302
chr9	99479475	99524233	44759	2	0	1	0	0.00478973	0	5	0	1.02643191
chr9	99524233	99583447	59215	2	1	1	0.30102999	0.00478973	0	5	0	1.02643191
chr9	99583447	99583506	60	1	1	3	0.30102999	0.05670724	0	5	0	0.45545077
chr9	99583506	99640257	56752	1	0	3	0	0.05670724	0	5	0	0.45545077
chr9	99640257	99723609	83353	1	0	2	0	0.02162467	0	5	0	0.68214471
chr9	99723609	99954624	231016	6	1	2	0.30102999	0.01053319	0	6	0	0.91219088
chr9	99954624	100147447	192824	5	1	3	0.30102999	0.03070643	0	6	0	0.63695542
chr9	100147447	100222949	75503	3	1	4	0.30102999	0.06713722	0	6	0	0.44141547
chr9	100222949	100340699	117751	2	1	3	0.30102999	0.03070643	0	6	0	0.63695542
chr9	100340699	100340758	60	1	1	4	0.30102999	0.06713722	0	6	0	0.44141547
chr9	100340758	100368484	27727	2	1	3	0.30102999	0.03070643	0	6	0	0.63695542
chr9	100368484	100368543	60	1	1	4	0.30102999	0.06713722	0	6	0	0.44141547
chr9	100368543	100506926	138384	4	0	3	0	0.03070643	0	6	0	0.63695542
chr9	100506926	100618226	111301	3	0	4	0	0.06713722	0	6	0	0.44141547
chr9	100618226	100702830	84605	2	0	2	0	0.02162467	0	5	0	0.68214471
chr9	100702830	100702889	60	1	0	4	0	0.06713722	0	6	0	0.44141547
chr9	100702889	100880177	177289	5	0	3	0	0.03070643	0	6	0	0.63695542
chr9	100880177	100880236	60	1	0	5	0	0.12309572	0	6	0	0.30102999
chr9	100880236	100922724	42489	2	0	3	0	0.03070643	0	6	0	0.63695542
chr9	100922724	100966794	44071	2	0	4	0	0.06713722	0	6	0	0.44141547
chr9	100966794	100966853	60	1	1	4	0.30102999	0.06713722	0	6	0	0.44141547
chr9	100966853	101052229	85377	2	0	4	0	0.06713722	0	6	0	0.44141547
chr9	101052229	101103733	51505	1	0	3	0	0.03070643	0	6	0	0.63695542
chr9	101103733	101366913	263181	6	1	3	0.30102999	0.03070643	0	6	0	0.63695542
chr9	101366913	101366972	60	1	1	4	0.30102999	0.06713722	0	6	0	0.44141547
chr9	101366972	101513362	146391	2	0	4	0	0.06713722	0	6	0	0.44141547
chr9	101513362	101547430	34069	1	0	3	0	0.03070643	0	6	0	0.63695542
chr9	101547430	101577905	30476	2	1	3	0.30102999	0.03070643	0	6	0	0.63695542
chr9	101577905	101612063	34159	1	0	3	0	0.03070643	0	6	0	0.63695542
chr9	101612063	101833091	221029	6	0	4	0	0.06713722	0	6	0	0.44141547
chr9	101833091	101891260	58170	2	0	4	0	0.11390336	0	5	0	0.30102999
chr9	101891260	101891315	56	1	0	4	0	0.06713722	0	6	0	0.44141547
chr9	101891315	101940944	49630	2	0	4	0	0.11390336	0	5	0	0.30102999
chr9	101940944	101982997	42054	2	0	5	0	0.19510895	0	5	0	0.19510895
chr9	101982997	101988398	5402	2	1	5	0.30102999	0.19510895	0	5	0	0.19510895
chr9	101988398	101989757	1360	1	1	4	0.30102999	0.11390336	0	5	0	0.30102999
chr9	101989757	101989816	60	1	1	4	0.30102999	0.06713722	0	6	0	0.44141547
chr9	101989816	102042279	52464	1	0	4	0	0.06713722	0	6	0	0.44141547
chr9	102042279	102112257	69979	2	0	5	0	0.12309572	0	6	0	0.30102999
chr9	102112257	102227981	115725	1	0	5	0	0.19510895	0	5	0	0.19510895
chr9	102227981	102472075	244095	2	0	5	0	0.30102999	0	4	0	0.11390336
chr9	102472075	102589027	116953	2	0	3	0	0.17593012	0	3	0	0.17593012
chr9	102589027	102596175	7149	2	0	4	0	0.11390336	0	5	0	0.30102999
chr9	102596175	102627513	31339	2	0	4	0	0.18734596	0	4	0	0.18734596
chr9	102627513	102669932	42420	2	0	4	0	0.11390336	0	5	0	0.30102999
chr9	102669932	102867687	197756	4	0	3	0	0.10122019	0	4	0	0.30102999
chr9	102867687	102867746	60	1	0	4	0	0.18734596	0	4	0	0.18734596
chr9	102867746	102947123	79378	2	0	3	0	0.17593012	0	3	0	0.17593012
chr9	102947123	103027144	80022	3	0	3	0	0.10122019	0	4	0	0.30102999
chr9	103027144	103027203	60	1	0	3	0	0.05670724	0	5	0	0.45545077
chr9	103027203	103070825	43623	1	0	3	0	0.10122019	0	4	0	0.30102999
chr9	103070825	103135416	64592	2	0	3	0	0.05670724	0	5	0	0.45545077
chr9	103135416	103231512	96097	4	0	4	0	0.11390336	0	5	0	0.30102999
chr9	103231512	103607071	375560	6	0	5	0	0.19510895	0	5	0	0.19510895
chr9	103607071	103728118	121048	1	0	3	0	0.05670724	0	5	0	0.45545077
chr9	103728118	103827939	99822	2	0	2	0	0.02162467	0	5	0	0.68214471
chr9	103827939	103995306	167368	4	0	3	0	0.05670724	0	5	0	0.45545077
chr9	103995306	104032248	36943	2	0	4	0	0.11390336	0	5	0	0.30102999
chr9	104032248	104131381	99134	2	0	2	0	0.02162467	0	5	0	0.68214471
chr9	104131381	104198104	66724	5	0	4	0	0.11390336	0	5	0	0.30102999
chr9	104198104	104245853	47750	1	0	3	0	0.10122019	0	4	0	0.30102999
chr9	104245853	104307086	61234	1	0	1	0	0.01091641	0	4	0	0.76005302
chr9	104307086	104307137	52	1	1	1	0.30102999	0.01091641	0	4	0	0.76005302
chr9	104307137	104356270	49134	1	0	1	0	0.01091641	0	4	0	0.76005302
chr9	104356270	104449425	93156	3	0	2	0	0.0429175	0	4	0	0.47744371
chr9	104449425	104531971	82547	3	1	3	0.30102999	0.10122019	0	4	0	0.30102999
chr9	104531971	104602509	70539	2	1	3	0.30102999	0.05670724	0	5	0	0.45545077
chr9	104602509	104755162	152654	1	0	3	0	0.10122019	0	4	0	0.30102999
chr9	104755162	104991055	235894	3	0	4	0	0.11390336	0	5	0	0.30102999
chr9	104991055	105106710	115656	1	0	2	0	0.08289318	0	3	0	0.30102999
chr9	105106710	105190105	83396	1	0	1	0	0.02438896	0	3	0	0.51676182
chr9	105190105	105190164	60	1	0	1	0	0.01091641	0	4	0	0.76005302
chr9	105190164	105350080	159917	2	0	0	0	0	0	2	0	0.61140001
chr9	105350080	105350139	60	1	0	0	0	0	0	3	0	0.93173516
chr9	105350139	105502955	152817	2	0	0	0	0	0	2	0	0.61140001
chr9	105502955	105774723	271769	3	0	0	0	0	0	3	0	0.93173516
chr9	105774723	105811446	36724	2	0	1	0	0.02438896	0	3	0	0.51676182
chr9	105811446	106007887	196442	2	0	0	0	0	0	3	0	0.93173516
chr9	106007887	106433699	425813	3	0	0	0	0	0	2	0	0.61140001
chr9	106433699	106621864	188166	2	0	3	0	0.10122019	0	4	0	0.30102999
chr9	106621864	106700012	78149	2	0	3	0	0.03070643	0	6	0	0.63695542
chr9	106700012	106700071	60	1	0	5	0	0.12309572	0	6	0	0.30102999
chr9	106700071	106901490	201420	2	0	3	0	0.03070643	0	6	0	0.63695542
chr9	106901490	107026058	124569	1	0	2	0	0.02162467	0	5	0	0.68214471

chr9	107026058	107062792	36735	2	0	4	0	0.11390336	1	5	0.30102999	0.30102999
chr9	107062792	107156480	93689	1	0	4	0	0.18734596	1	4	0.30102999	0.18734596
chr9	107156480	107293452	136973	2	0	4	0	0.18734596	0	4	0	0.18734596
chr9	107293452	107293511	60	1	0	5	0	0.12309572	1	6	0.30102999	0.30102999
chr9	107293511	107386168	92658	2	0	3	0	0.10122019	1	4	0.30102999	0.30102999
chr9	107386168	107420201	34034	2	0	4	0	0.11390336	1	5	0.30102999	0.30102999
chr9	107420201	107460164	39964	1	0	3	0	0.10122019	1	4	0.30102999	0.30102999
chr9	107460164	107510848	50685	1	0	3	0	0.17593012	1	3	0.30102999	0.17593012
chr9	107510848	107648702	137855	6	0	5	0	0.30102999	1	4	0.30102999	0.11390336
chr9	107648702	107810022	161321	3	0	6	0	0.30102999	1	5	0.30102999	0.12309572
chr9	107810022	108021895	211874	3	0	4	0	0.30102999	0	3	0	0.10122019
chr9	108021895	108021954	60	1	0	5	0	0.45545077	0	3	0	0.05670724
chr9	108021954	108118605	96652	2	0	3	0	0.17593012	0	3	0	0.17593012
chr9	108118605	108197017	78413	2	0	5	0	0.45545077	0	3	0	0.05670724
chr9	108197017	108197076	60	1	0	5	0	0.30102999	0	4	0	0.11390336
chr9	108197076	108276411	79336	1	0	4	0	0.30102999	0	3	0	0.10122019
chr9	108276411	108382256	105846	2	0	3	0	0.17593012	0	3	0	0.17593012
chr9	108382256	108424804	42549	2	0	3	0	0.10122019	0	4	0	0.30102999
chr9	108424804	108487745	62942	1	0	2	0	0.0429175	0	4	0	0.47744371
chr9	108487745	108535449	47705	1	0	0	0	0	0	4	0	1.26272838
chr9	108535449	108865371	329923	2	0	0	0	0	0	3	0	0.93173516
chr9	108865371	108986615	121245	2	0	2	0	0.08289318	0	3	0	0.30102999
chr9	108986615	109043854	57240	2	0	4	0	0.30102999	0	3	0	0.10122019
chr9	109043854	109163320	119467	2	0	1	0	0.02438896	0	3	0	0.51676182
chr9	109163320	109163379	60	1	0	2	0	0.08289318	0	3	0	0.30102999
chr9	109163379	109621337	457959	5	0	1	0	0.05404976	0	2	0	0.30102999
chr9	109621337	109653113	31777	2	0	2	0	0.1575501	0	2	0	0.1575501
chr9	109653113	109737164	84052	2	0	1	0	0.05404976	0	2	0	0.30102999
chr9	109737164	110046402	309239	5	0	0	0	0	0	2	0	0.61140001
chr9	110046402	110093089	46688	2	0	1	0	0.02438896	0	3	0	0.51676182
chr9	110093089	110273555	180467	3	0	1	0	0.05404976	0	2	0	0.30102999
chr9	110273555	110317852	44298	2	0	1	0	0.02438896	0	3	0	0.51676182
chr9	110317852	110672051	354200	4	0	1	0	0.05404976	0	2	0	0.30102999
chr9	110672051	110861863	189813	3	0	1	0	0.02438896	0	3	0	0.51676182
chr9	110861863	111047632	185770	2	0	2	0	0.02162467	0	5	0	0.68214471
chr9	111047632	111047691	60	1	0	2	0	0.01053319	0	6	0	0.91219088
chr9	111047691	111341780	294090	1	0	1	0	0.00204627	0	6	0	1.31360226
chr9	111341780	111518840	177061	2	0	2	0	0.01053319	0	6	0	0.91219088
chr9	111518840	111518899	60	1	1	3	0.30102999	0.03070643	0	6	0	0.63695542
chr9	111518899	111672482	153584	4	0	3	0	0.03070643	0	6	0	0.63695542
chr9	111672482	111754969	82488	5	1	3	0.30102999	0.03070643	0	6	0	0.63695542
chr9	111754969	111868855	113887	5	1	2	0.30102999	0.01053319	0	6	0	0.91219088
chr9	111868855	111868914	60	1	1	3	0.30102999	0.03070643	0	6	0	0.63695542
chr9	111868914	111970294	101381	3	1	2	0.30102999	0.01053319	0	6	0	0.91219088
chr9	111970294	111970351	58	1	2	3	0.61140001	0.03070643	0	6	0	0.63695542
chr9	111970351	112258440	288090	6	2	2	0.61140001	0.01053319	0	6	0	0.91219088
chr9	112258440	112482995	224556	5	2	2	0.30102999	0.01053319	1	6	0.05404976	0.91219088
chr9	112482995	112483054	60	1	2	3	0.30102999	0.03070643	1	6	0.05404976	0.63695542
chr9	112483054	112633360	150307	3	2	2	0.30102999	0.01053319	1	6	0.05404976	0.91219088
chr9	112633360	112772526	139167	3	1	2	0.1218695	0.01053319	1	6	0.1218695	0.91219088
chr9	112772526	112820649	48124	1	1	2	0.1218695	0.02162467	1	5	0.1218695	0.68214471
chr9	112820649	112981516	160868	4	1	1	0.1218695	0.00478973	1	5	0.1218695	1.02643191
chr9	112981516	113285506	303991	6	1	1	0.1218695	0.01091641	1	4	0.1218695	0.76005302
chr9	113285506	113285565	60	1	1	2	0.1218695	0.0429175	1	4	0.1218695	0.47744371
chr9	113285565	113327458	41894	1	1	2	0.30102999	0.0429175	0	4	0	0.47744371
chr9	113327458	113366472	39015	2	1	2	0.30102999	0.02162467	0	5	0	0.68214471
chr9	113366472	113431266	64795	1	1	1	0.30102999	0.00478973	0	5	0	1.02643191
chr9	113431266	113562937	131672	3	0	1	0	0.00478973	0	5	0	1.02643191
chr9	113562937	113562988	52	1	0	2	0	0.02162467	0	5	0	0.68214471
chr9	113562988	113677240	114253	2	0	1	0	0.00478973	0	5	0	1.02643191
chr9	113677240	113784710	107471	3	0	2	0	0.02162467	0	5	0	0.68214471
chr9	113784710	113784769	60	1	0	2	0	0.01053319	0	6	0	0.91219088
chr9	113784769	113860744	75976	2	0	2	0	0.08289318	0	3	0	0.30102999
chr9	113860744	113919284	58541	1	0	1	0	0.05404976	0	2	0	0.30102999
chr9	113919284	113948285	29002	2	0	1	0	0.02438896	0	3	0	0.51676182
chr9	113948285	114061747	113463	1	0	1	0	0.05404976	0	2	0	0.30102999
chr9	114061747	114147303	85557	2	0	1	0	0.02438896	0	3	0	0.51676182
chr9	114147303	114147362	60	1	0	1	0	0.01091641	0	4	0	0.76005302
chr9	114147362	114198483	51122	1	0	1	0	0.02438896	0	3	0	0.51676182
chr9	114198483	114325417	126935	5	0	1	0	0.01091641	0	4	0	0.76005302
chr9	114325417	114423267	97851	3	0	1	0	0.05404976	0	2	0	0.30102999
chr9	114423267	114423325	59	1	1	1	0.30102999	0.02438896	0	3	0	0.51676182
chr9	114423325	114472435	49111	1	1	1	0.30102999	0.1218695	0	1	0	0.1218695
chr9	114472435	114538198	65764	2	1	0	0.30102999	0	0	1	0	0.30102999
chr9	114538198	114661532	123335	3	1	1	0.30102999	0.1218695	0	1	0	0.1218695
chr9	114661532	114661591	60	1	1	1	0.30102999	0.02438896	0	3	0	0.51676182
chr9	114661591	114762420	100830	2	0	0	0	0	0	3	0	0.93173516
chr9	114762420	114805946	43527	2	1	0	0.30102999	0	0	3	0	0.93173516
chr9	114805946	115095046	289101	7	1	1	0.30102999	0.02438896	0	3	0	0.51676182
chr9	115095046	115167877	72832	3	1	1	0.30102999	0.01091641	0	4	0	0.76005302
chr9	115167877	115167936	60	1	1	1	0.30102999	0.00478973	0	5	0	1.02643191
chr9	115167936	115252623	84688	2	1	1	0.30102999	0.01091641	0	4	0	0.76005302
chr9	115252623	115449078	196456	4	0	1	0	0.01091641	0	4	0	0.76005302
chr9	115449078	115552278	103201	4	0	1	0	0.00478973	0	5	0	1.02643191
chr9	115552278	115747015	194738	5	0	1	0	0.02438896	0	3	0	0.51676182
chr9	115747015	115808690	61676	2	0	1	0	0.01091641	0	4	0	0.76005302
chr9	115808690	115925701	117012	2	0	1	0	0.00478973	0	5	0	1.02643191
chr9	115925701	115925760	60	1	0	1	0	0.00204627	0	6	0	1.31360226
chr9	115925760	116035393	109634	2	0	1	0	0.00478973	0	5	0	1.02643191
chr9	116035393	116041198	5806	2	0	1	0	0.00204627	0	6	0	1.31360226
chr9	116041198	116113079	71882	6	0	3	0	0.03070643	0	6	0	0.63695542
chr9	116113079	116641235	528157	14	0	3	0	0.01598258	0	7	0	0.84395715
chr9	116641235	116708691	67457	1	0	3	0	0.03070643	0	6	0	0.63695542
chr9	116708691	116756926	48236	2	1	3	0.30102999	0.03070643	0	6	0	0.63695542
chr9	116756926	116756983	58	1	1	3	0.30102999	0.01598258	0	7	0	0.84395715
chr9	116756983	116792658	35676	1	1	3	0.30102999	0.03070643	0	6	0	0.63695542
chr9	116792658	116859141	66484	2	0	3	0	0.03070643	0	6	0	0.63695542
chr9	116859141	116859193	53	1	0	3	0	0.01598258	0	7	0	0.84395715
chr9	116859193	116953141	93949	2	0	3	0	0.03070643	0	6	0	0.63695542
chr9	116953141	116980592	27452	2	1	3	0.30102999	0.03070643	0	6	0	0.63695542
chr9	116980592	117054834	74243	2	0	3	0	0.05670724	0	5	0	0.45545077
chr9	117054834	117054887	54	1	0	3	0	0.03070643	0	6	0	0.63695542
chr9	117054887	117082833	27947	1	0	2	0	0.01053319	0	6	0	0.91219088
chr9	117082833	117093071	10239	1	0	2	0	0.02162467	0	5	0	0.68214471
chr9	117093071	117127735	34665	1	0	2	0	0.0429175	0	4	0	0.47744371
chr9	117127735	117127794	60	1	1	2	0.30102999	0.0429175	0	4	0	0.47744371
chr9	117127794	117136983	9190	1	0	2	0	0.0429175	0	4	0	0.47744371
chr9	117136983	117170419	33437	1	0	2	0	0.08289318	0	3	0	0.30102999
chr9	117170419	117207191	36773	2	0	2	0	0.0429175	0	4	0	0.47744371
chr9	117207191	117311675	104485	3	0	2	0	0.0429175	1	4	0.30102999	0.47744371
chr9	117311675	117585649	273975	7	0	2	0	0.08289318	1	3	0.30102999	0.30102999
chr9	117585649	117621345	35697	2	0	2	0	0.0429175	1	4	0.30102999	0.47744371
chr9	117621345	117782964	161620	3	0	2	0	0.0429175	0	4	0	0.47744371
chr9	117782964	117783023	60	1	0	3	0	0.10122019	0	4	0	0.30102999
chr9	117783023	117853370	70348	2	0	2	0	0.08289318	0	3	0	0.30102999
chr9	117853370	117879236	25867	2	0	2	0	0.0429175	0	4	0	0.47744371
chr9	117879236	117932660	53425	2	0	3	0	0.10122019	0	4	0	0.30102999
chr9	117932660	117980055	47396	2	0	3	0	0.05670724	0	5	0	0.45545077
chr9	117980055	118054094	74040	2	0	2	0	0.02162467	0	5	0	0.68214471
chr9	118054094	118128604	74511	2	0	2	0	0.08289318	0	3	0	0.30102999
chr9	118128604	118164654	36051	2	0	2	0	0.08289318	1	3	0.30102999	0.30102999
chr9	118164654	118164713	60	1	0	3	0	0.10122019	1	4	0.30102999	0.30102999
chr9	118164713	118206312	41600	1	0	3	0	0.17593012	1	3	0.30102999	0.17593012
chr9	118206312	118206371	60	1	0	4	0	0.30102999	1	3	0.30102999	0.10122019

chr9	118206371	118529695	323325	3	0	3	0	0.51676182	1	1	0.30102999	0.02438896
chr9	118529695	118529754	60	1	0	4	0	0.18734596	1	4	0.30102999	0.18734596
chr9	118529754	118650708	120955	1	0	4	0	0.30102999	0	3	0	0.10122019
chr9	118650708	118677120	26413	3	0	5	0	0.45545077	0	3	0	0.05670724
chr9	118677120	118839359	162240	1	0	4	0	0.30102999	0	3	0	0.10122019
chr9	118839359	118839418	60	1	0	4	0	0.18734596	1	4	0.30102999	0.18734596
chr9	118839418	119059513	220096	3	0	4	0	0.30102999	0	3	0	0.10122019
chr9	119059513	119127934	68422	3	0	5	0	0.45545077	0	3	0	0.05670724
chr9	119127934	119187479	59546	5	0	5	0	0.19510895	0	5	0	0.19510895
chr9	119187479	119380611	193133	4	0	5	0	0.12309572	0	6	0	0.30102999
chr9	119380611	119419472	38862	2	0	5	0	0.07511598	0	7	0	0.43181735
chr9	119419472	119457998	38527	4	0	5	0	0.04407651	0	8	0	0.58747015
chr9	119457998	119576527	118530	2	0	5	0	0.07511598	0	7	0	0.43181735
chr9	119576527	120086013	509487	10	0	5	0	0.04407651	0	8	0	0.58747015
chr9	120086013	120120407	34395	1	0	4	0	0.02074938	0	8	0	0.79906872
chr9	120120407	120296568	176162	2	0	3	0	0.0079614	0	8	0	1.07548421
chr9	120296568	120435101	138534	1	0	2	0	0.00493743	0	7	0	1.16581773
chr9	120435101	120708959	273859	4	0	2	0	0.01053319	0	6	0	0.91219088
chr9	120708959	120709018	60	1	0	4	0	0.06713722	0	6	0	0.44141547
chr9	120709018	121139703	430686	3	0	3	0	0.03070643	0	6	0	0.63695542
chr9	121139703	121432168	292466	2	0	2	0	0.01053319	0	6	0	0.91219088
chr9	121432168	121432227	60	1	0	3	0	0.01598258	0	7	0	0.84395715
chr9	121432227	121540083	107857	1	0	2	0	0.01053319	0	6	0	0.91219088
chr9	121540083	121672929	132847	2	0	2	0	0.00493743	0	7	0	1.16581773
chr9	121672929	121929222	256294	2	0	1	0	0.00204627	0	6	0	1.31360226
chr9	121929222	122004122	74901	1	0	1	0	0.01091641	0	4	0	0.76005302
chr9	122004122	122115885	111764	4	0	2	0	0.0429175	0	4	0	0.47744371
chr9	122115885	122159398	43514	2	0	2	0	0.02162467	0	5	0	0.68214471
chr9	122159398	122159457	60	1	0	2	0	0.01053319	0	6	0	0.91219088
chr9	122159457	122361030	201574	2	0	2	0	0.0429175	0	4	0	0.47744371
chr9	122361030	122547094	186065	2	0	2	0	0.00493743	0	7	0	1.16581773
chr9	122547094	122547153	60	1	0	3	0	0.01598258	0	7	0	0.84395715
chr9	122547153	122762080	214928	2	0	2	0	0.00493743	0	7	0	1.16581773
chr9	122762080	122762139	60	1	0	2	0	0.00221948	0	8	0	1.44210395
chr9	122762139	122905740	143602	1	0	2	0	0.00493743	0	7	0	1.16581773
chr9	122905740	123010959	105220	2	0	2	0	0.01053319	0	6	0	0.91219088
chr9	123010959	123088173	77215	2	0	2	0	0.00493743	0	7	0	1.16581773
chr9	123088173	123088232	60	1	1	2	0.30102999	0.00493743	0	7	0	1.16581773
chr9	123088232	123182061	93830	3	0	2	0	0.01053319	0	6	0	0.91219088
chr9	123182061	123182120	60	1	1	2	0.30102999	0.01053319	0	6	0	0.91219088
chr9	123182120	123341444	159325	3	0	2	0	0.01053319	0	6	0	0.91219088
chr9	123341444	123341503	60	1	0	2	0	0.00493743	0	7	0	1.16581773
chr9	123341503	123414289	72787	2	0	2	0	0.02162467	0	5	0	0.68214471
chr9	123414289	123414348	60	1	1	2	0.30102999	0.02162467	0	5	0	0.68214471
chr9	123414348	123546488	132141	3	0	2	0	0.02162467	0	5	0	0.68214471
chr9	123546488	123550447	3960	2	0	2	0	0.01053319	0	6	0	0.91219088
chr9	123550447	123636598	86152	3	0	2	0	0.00493743	0	7	0	1.16581773
chr9	123636598	123860664	224067	8	0	2	0	0.00493743	1	7	0.30102999	1.16581773
chr9	123860664	124001726	141063	5	0	2	0	0.00221948	1	8	0.30102999	1.44210395
chr9	124001726	124291196	289471	9	0	2	0	0.00221948	2	8	0.61140001	1.44210395
chr9	124291196	124423026	131831	2	0	2	0	0.00493743	2	7	0.61140001	1.16581773
chr9	124423026	124423085	60	1	0	2	0	0.00221948	2	8	0.61140001	1.44210395
chr9	124423085	124584365	161281	4	0	2	0	0.00493743	2	7	0.61140001	1.16581773
chr9	124584365	124628147	43783	1	0	2	0	0.00493743	1	7	0.30102999	1.16581773
chr9	124628147	124628206	60	1	0	2	0	0.00221948	1	8	0.30102999	1.44210395
chr9	124628206	124664562	36357	1	0	2	0	0.00493743	1	7	0.30102999	1.16581773
chr9	124664562	124918446	253885	7	0	2	0	0.00493743	2	7	0.61140001	1.16581773
chr9	124918446	124918505	60	1	0	3	0	0.0079614	2	8	0.61140001	1.07548421
chr9	124918505	124977052	58548	2	0	2	0	0.00493743	2	7	0.61140001	1.16581773
chr9	124977052	125042127	65076	3	0	3	0	0.01598258	2	7	0.61140001	0.84395715
chr9	125042127	125151302	109176	3	0	3	0	0.01598258	1	7	0.30102999	0.84395715
chr9	125151302	125228887	77586	3	0	3	0	0.0079614	1	8	0.30102999	1.07548421
chr9	125228887	125330788	101902	2	0	3	0	0.01598258	1	7	0.30102999	0.84395715
chr9	125330788	125376609	45822	2	0	3	0	0.0079614	1	8	0.30102999	1.07548421
chr9	125376609	125520006	143398	2	0	3	0	0.03070643	1	6	0.30102999	0.63695542
chr9	125520006	125782637	262632	10	0	4	0	0.06713722	1	6	0.30102999	0.44141547
chr9	125782637	125815519	32883	2	0	4	0	0.11390336	1	5	0.30102999	0.30102999
chr9	125815519	126027907	212389	9	0	4	0	0.06713722	1	6	0.30102999	0.44141547
chr9	126027907	126202182	174276	4	0	3	0	0.03070643	1	6	0.30102999	0.63695542
chr9	126202182	126270264	68083	2	0	3	0	0.03070643	0	6	0	0.63695542
chr9	126270264	126270323	60	1	0	3	0	0.03070643	1	6	0.30102999	0.63695542
chr9	126270323	126340823	70501	1	0	3	0	0.03070643	0	6	0	0.63695542
chr9	126340823	126370587	29765	2	0	4	0	0.06713722	0	6	0	0.44141547
chr9	126370587	126480075	109489	2	0	3	0	0.03070643	0	6	0	0.63695542
chr9	126480075	126480134	60	1	0	4	0	0.03812622	0	7	0	0.60763643
chr9	126480134	126598579	118446	3	0	3	0	0.01598258	0	7	0	0.84395715
chr9	126598579	126723048	124470	2	0	2	0	0.01053319	0	6	0	0.91219088
chr9	126723048	126780380	57333	2	1	2	0.30102999	0.01053319	0	6	0	0.91219088
chr9	126780380	126795325	14946	3	1	2	0.30102999	0.00493743	0	7	0	1.16581773
chr9	126795325	127019350	224026	2	1	2	0.30102999	0.02162467	0	5	0	0.68214471
chr9	127019350	127023652	4303	2	1	3	0.30102999	0.03070643	0	6	0	0.63695542
chr9	127023652	127130392	106741	2	1	2	0.30102999	0.01053319	0	6	0	0.91219088
chr9	127130392	127130451	60	1	1	3	0.30102999	0.03070643	0	6	0	0.63695542
chr9	127130451	127254078	123628	3	1	3	0.30102999	0.05670724	0	5	0	0.45545077
chr9	127254078	127254125	48	1	1	3	0.30102999	0.01598258	0	7	0	0.84395715
chr9	127254125	127319305	65181	3	1	3	0.30102999	0.03070643	0	6	0	0.63695542
chr9	127319305	127707232	387928	13	1	3	0.30102999	0.01598258	0	7	0	0.84395715
chr9	127707232	127749171	41940	1	1	3	0.30102999	0.05670724	0	5	0	0.45545077
chr9	127749171	127818144	68974	2	1	3	0.30102999	0.10122019	0	4	0	0.30102999
chr9	127818144	127867709	49566	2	1	3	0.30102999	0.05670724	0	5	0	0.45545077
chr9	127867709	128464047	596339	16	1	3	0.30102999	0.10122019	0	4	0	0.30102999
chr9	128464047	128464106	60	1	1	3	0.30102999	0.05670724	0	5	0	0.45545077
chr9	128464106	128557214	93109	3	1	3	0.30102999	0.30102999	0	2	0	0.08289318
chr9	128557214	128557273	60	1	1	3	0.30102999	0.17593012	0	3	0	0.17593012
chr9	128557273	128640000	82728	2	1	3	0.30102999	0.30102999	0	2	0	0.08289318
chr9	128640000	128652844	12845	2	1	3	0.30102999	0.10122019	0	4	0	0.30102999
chr9	128652844	128689438	36595	2	1	3	0.30102999	0.30102999	0	2	0	0.08289318
chr9	128689438	128689497	60	1	1	3	0.30102999	0.10122019	0	4	0	0.30102999
chr9	128689497	128711704	22208	1	1	3	0.30102999	0.17593012	0	3	0	0.17593012
chr9	128711704	128870221	158518	1	0	2	0	0.08289318	0	3	0	0.30102999
chr9	128870221	129103689	233469	3	0	2	0	0.0429175	0	4	0	0.47744371
chr9	129103689	129199679	95991	3	0	3	0	0.10122019	0	4	0	0.30102999
chr9	129199679	129356475	156797	3	0	2	0	0.08289318	0	3	0	0.30102999
chr9	129356475	129356534	60	1	0	3	0	0.10122019	0	4	0	0.30102999
chr9	129356534	129451759	95226	3	0	3	0	0.17593012	0	3	0	0.17593012
chr9	129451759	129509718	57960	2	0	3	0	0.10122019	0	4	0	0.30102999
chr9	129509718	129571350	61633	2	0	4	0	0.18734596	0	4	0	0.18734596
chr9	129571350	129597516	26167	2	0	4	0	0.03812622	0	7	0	0.60763643
chr9	129597516	129597575	60	1	0	4	0	0.02074938	0	8	0	0.79906872
chr9	129597575	129688382	90808	2	0	4	0	0.03812622	0	7	0	0.60763643
chr9	129688382	129736810	48429	2	0	4	0	0.02074938	0	8	0	0.79906872
chr9	129736810	129849593	112784	4	0	4	0	0.01077081	0	9	0	1.01542894
chr9	129849593	129849682	90	2	0	4	0	0.00530919	0	10	0	1.2568129
chr9	129849682	129928144	78463	2	0	4	0	0.01077081	0	9	0	1.01542894
chr9	129928144	130013475	85332	3	0	4	0	0.00530919	0	10	0	1.2568129
chr9	130013475	130041612	28138	2	0	4	0	0.00247414	0	11	0	1.52371709
chr9	130041612	130120104	78493	2	0	4	0	0.00530919	0	10	0	1.2568129
chr9	130120104	130120163	60	1	0	5	0	0.01320236	0	10	0	0.97390707
chr9	130120163	130169441	49279	1	0	4	0	0.00530919	0	10	0	1.2568129
chr9	130169441	130197466	28026	2	0	4	0	0.00247414	0	11	0	1.52371709
chr9	130197466	130413926	216461	9	0	5	0	0.00666883	0	11	0	1.20557689
chr9	130413926	130491547	77622	2	0	5	0	0.01320236	0	10	0	0.97390707
chr9	130491547	130533312	41766	4	0	5	0	0.00666883	0	11	0	1.20557689
chr9	130533312	130533364	53	1	0	5	0	0.00317045	0	12	0	1.46403142

chr9	130533364	130596070	62707	3	0	5	0	0.00666883	0	11	0	1.20557689
chr9	130596070	130699312	103243	7	0	5	0	0.00317045	1	12	0.30102999	1.46403142
chr9	130699312	130735438	36127	3	0	6	0	0.00778066	1	12	0.30102999	1.17038931
chr9	130735438	130825542	90105	1	0	6	0	0.02793176	1	10	0.30102999	0.74627054
chr9	130825542	130886800	61259	2	1	6	0.1218695	0.02793176	1	10	0.1218695	0.74627054
chr9	130886800	130923464	36665	2	1	7	0.1218695	0.03037338	1	11	0.1218695	0.73110763
chr9	130923464	130985100	61637	2	2	7	0.30102999	0.03037338	1	11	0.05404976	0.73110763
chr9	130985100	131090997	105898	5	1	6	0.1218695	0.01518174	1	11	0.1218695	0.9449689
chr9	131090997	131271757	180761	10	2	7	0.30102999	0.03037338	1	11	0.05404976	0.73110763
chr9	131271757	131306786	35030	1	2	6	0.30102999	0.01518174	1	11	0.05404976	0.9449689
chr9	131306786	131395721	88936	3	2	5	0.30102999	0.00666883	1	11	0.05404976	1.20557689
chr9	131395721	131416330	20610	2	2	6	0.30102999	0.01518174	1	11	0.05404976	0.9449689
chr9	131416330	131485931	69602	5	2	7	0.30102999	0.03037338	1	11	0.05404976	0.73110763
chr9	131485931	131503843	17913	1	2	6	0.30102999	0.01518174	1	11	0.05404976	0.9449689
chr9	131503843	131525429	21587	2	2	6	0.1575501	0.01518174	2	11	0.1575501	0.9449689
chr9	131525429	131551269	25841	1	2	6	0.1575501	0.02793176	2	10	0.1575501	0.74627054
chr9	131551269	131633235	81967	5	2	6	0.30102999	0.02793176	1	10	0.05404976	0.74627054
chr9	131633235	131689374	56140	2	2	6	0.30102999	0.01518174	1	11	0.05404976	0.9449689
chr9	131689374	131689418	45	1	2	7	0.30102999	0.03037338	1	11	0.05404976	0.73110763
chr9	131689418	131708122	18705	2	1	7	0.1218695	0.03037338	1	11	0.1218695	0.73110763
chr9	131708122	131708179	58	1	2	7	0.30102999	0.03037338	1	11	0.05404976	0.73110763
chr9	131708179	131824464	116286	4	1	6	0.1218695	0.02793176	1	10	0.1218695	0.74627054
chr9	131824464	131824523	60	1	2	6	0.30102999	0.01518174	1	11	0.05404976	0.9449689
chr9	131824523	131852122	27600	3	2	6	0.30102999	0.02793176	1	10	0.05404976	0.74627054
chr9	131852122	131867543	15422	1	1	6	0.1218695	0.02793176	1	10	0.1218695	0.74627054
chr9	131867543	131899913	32371	2	1	6	0.1218695	0.01518174	1	11	0.1218695	0.9449689
chr9	131899913	131899966	54	1	2	6	0.30102999	0.01518174	1	11	0.05404976	0.9449689
chr9	131899966	131955293	55328	1	2	6	0.30102999	0.02793176	1	10	0.05404976	0.74627054
chr9	131955293	131987266	31974	1	2	5	0.30102999	0.01320236	1	10	0.05404976	0.97390707
chr9	131987266	131987325	60	1	2	6	0.30102999	0.02793176	1	10	0.05404976	0.74627054
chr9	131987325	132043687	56363	1	1	6	0.1218695	0.04875589	1	9	0.1218695	0.5732208
chr9	132043687	132091097	47411	1	1	5	0.1218695	0.02473314	1	9	0.1218695	0.76806864
chr9	132091097	132091156	60	1	1	5	0.1218695	0.01320236	1	10	0.1218695	0.97390707
chr9	132091156	132315993	224838	4	1	4	0.1218695	0.00530919	1	10	0.1218695	1.2568129
chr9	132315993	132389432	73440	2	1	5	0.1218695	0.01320236	1	10	0.1218695	0.97390707
chr9	132389432	132395475	6044	1	1	5	0.30102999	0.01320236	0	10	0	0.97390707
chr9	132395475	132396763	1289	2	1	6	0.30102999	0.02793176	0	10	0	0.74627054
chr9	132396763	132399197	2435	2	1	6	0.1218695	0.02793176	1	10	0.1218695	0.74627054
chr9	132399197	132461752	62556	2	1	5	0.30102999	0.01320236	0	10	0	0.97390707
chr9	132461752	132484969	23218	2	1	6	0.30102999	0.02793176	0	10	0	0.74627054
chr9	132484969	132573552	88584	2	1	5	0.30102999	0.01320236	0	10	0	0.97390707
chr9	132573552	132584673	11122	1	1	4	0.30102999	0.01077081	0	9	0	1.01542894
chr9	132584673	132612490	27818	3	1	4	0.30102999	0.00530919	0	10	0	1.2568129
chr9	132612490	132690322	77833	2	1	4	0.30102999	0.00247414	0	11	0	1.52371709
chr9	132690322	132690381	60	1	1	5	0.30102999	0.00666883	0	11	0	1.20557689
chr9	132690381	132755571	65191	1	1	4	0.30102999	0.00247414	0	11	0	1.52371709
chr9	132755571	132755630	60	1	1	4	0.1218695	0.00247414	1	11	0.1218695	1.52371709
chr9	132755630	132803682	48053	1	1	4	0.1218695	0.00530919	1	10	0.1218695	1.2568129
chr9	132803682	132803741	60	1	1	5	0.1218695	0.00666883	1	11	0.1218695	1.20557689
chr9	132803741	132863494	59754	2	1	4	0.30102999	0.00247414	0	11	0	1.52371709
chr9	132863494	132902903	39410	1	1	4	0.30102999	0.00530919	0	10	0	1.2568129
chr9	132902903	132995601	92699	2	1	4	0.30102999	0.01077081	0	9	0	1.01542894
chr9	132995601	132995660	60	1	1	4	0.1218695	0.00530919	1	10	0.1218695	1.2568129
chr9	132995660	133044416	48757	1	1	3	0.30102999	0.00170589	0	10	0	1.61091002
chr9	133044416	133297198	252783	1	0	3	0	0.00170589	0	10	0	1.61091002
chr9	133297198	133297257	60	1	0	7	0	0.05232577	0	10	0	0.56314362
chr9	133297257	133376145	78889	2	0	5	0	0.04407651	0	8	0	0.58747015
chr9	133376145	133376204	60	1	0	7	0	0.08584816	0	9	0	0.42015402
chr9	133376204	133481689	105486	2	0	5	0	0.04407651	0	8	0	0.58747015
chr9	133481689	133481748	60	1	0	5	0	0.04407651	1	8	0.30102999	0.58747015
chr9	133481748	133548267	66520	1	0	4	0	0.03812622	0	7	0	0.60763643
chr9	133548267	133590274	42008	4	0	5	0	0.07511598	0	7	0	0.43181735
chr9	133590274	133590333	60	1	0	5	0	0.07511598	1	7	0.30102999	0.43181735
chr9	133590333	133768611	178279	4	0	5	0	0.07511598	0	7	0	0.43181735
chr9	133768611	133774868	6258	2	0	6	0	0.08122616	0	8	0	0.4250187
chr9	133774868	133931680	156813	4	0	6	0	0.129913	0	7	0	0.30102999
chr9	133931680	134053698	122019	4	0	4	0	0.03812622	0	7	0	0.60763643
chr9	134053698	134104967	51270	3	0	6	0	0.129913	0	7	0	0.30102999
chr9	134104967	134184586	79620	5	0	6	0	0.08122616	0	8	0	0.4250187
chr9	134184586	134305643	121058	3	0	6	0	0.04875589	0	9	0	0.5732208
chr9	134305643	134501267	195625	6	0	5	0	0.02473314	0	9	0	0.76806864
chr9	134501267	134585960	84694	3	0	6	0	0.04875589	0	9	0	0.5732208
chr9	134585960	134612145	26186	1	0	5	0	0.02473314	0	9	0	0.76806864
chr9	134612145	134663883	51739	2	0	5	0	0.01320236	0	10	0	0.97390707
chr9	134663883	134785337	121455	2	0	4	0	0.01077081	0	9	0	1.01542894
chr9	134785337	134881700	96364	3	0	6	0	0.02793176	0	10	0	0.74627054
chr9	134881700	135007324	125625	4	0	6	0	0.00778066	0	12	0	1.17038931
chr9	135007324	135053804	46481	3	0	7	0	0.00859896	0	13	0	1.14735594
chr9	135053804	135087840	34037	2	0	7	0	0.00413023	0	14	0	1.39918918
chr9	135087840	135161840	74001	3	0	6	0	0.00373523	0	13	0	1.42397267
chr9	135161840	135211814	49975	2	0	6	0	0.01518174	0	11	0	0.9449689
chr9	135211814	135261940	50127	2	0	6	0	0.00778066	0	12	0	1.17038931
chr9	135261940	135261999	60	1	0	6	0	0.00373523	0	13	0	1.42397267
chr9	135261999	135380272	118274	3	0	6	0	0.00778066	0	12	0	1.17038931
chr9	135380272	135380331	60	1	0	6	0	0.00166733	0	14	0	1.70763027
chr9	135380331	135461246	80916	2	0	5	0	0.00317045	0	12	0	1.46403142
chr9	135461246	135549768	88523	3	0	5	0	0.00141024	0	13	0	1.7506523
chr9	135549768	135600047	50280	2	0	7	0	0.00859896	0	13	0	1.14735594
chr9	135600047	135600104	58	1	0	8	0	0.01767679	0	13	0	0.91308053
chr9	135600104	135763086	162983	5	0	8	0	0.03209037	0	12	0	0.72109894
chr9	135763086	135787853	24768	3	0	8	0	0.01767679	0	13	0	0.91308053
chr9	135787853	135813928	26076	1	0	8	0	0.03209037	0	12	0	0.72109894
chr9	135813928	136036942	223015	8	0	7	0	0.01667721	0	12	0	0.92532268
chr9	136036942	136137479	100538	2	0	6	0	0.00778066	0	12	0	1.17038931
chr9	136137479	136137536	58	1	0	7	0	0.01667721	0	12	0	0.92532268
chr9	136137536	136354510	216975	5	0	6	0	0.00778066	0	12	0	1.17038931
chr9	136354510	136407014	52505	2	0	7	0	0.01667721	0	12	0	0.92532268
chr9	136407014	136407071	58	1	1	7	0.30102999	0.01667721	0	12	0	0.92532268
chr9	136407071	136538934	131864	2	1	5	0.30102999	0.00666883	0	11	0	1.20557689
chr9	136538934	136586798	47865	1	1	5	0.30102999	0.01320236	0	10	0	0.97390707
chr9	136586798	136586857	60	1	1	5	0.30102999	0.00317045	0	12	0	1.46403142
chr9	136586857	136605402	18546	1	1	5	0.30102999	0.01320236	0	10	0	0.97390707
chr9	136605402	136605456	55	1	1	5	0.30102999	0.00666883	0	11	0	1.20557689
chr9	136605456	136656889	51434	1	1	5	0.30102999	0.01320236	0	10	0	0.97390707
chr9	136656889	136755057	98169	3	1	6	0.30102999	0.02793176	0	10	0	0.74627054
chr9	136755057	136828326	73270	2	1	6	0.30102999	0.04875589	0	9	0	0.5732208
chr9	136828326	136861477	33152	2	1	6	0.30102999	0.00778066	0	12	0	1.17038931
chr9	136861477	136912981	51505	1	0	6	0	0.00778066	0	12	0	1.17038931
chr9	136912981	136974334	61354	1	0	6	0	0.08122616	0	8	0	0.4250187
chr9	136974334	137192259	217926	1	0	5	0	0.04407651	0	8	0	0.58747015
chr9	137192259	137226998	34740	1	0	4	0	0.02074938	0	8	0	0.79906872
chr9	137226998	137227053	56	1	0	5	0	0.04407651	0	8	0	0.58747015
chr9	137227053	137291508	64456	1	0	4	0	0.03812622	0	7	0	0.60763643
chr9	137291508	137297534	6027	2	0	6	0	0.01518174	0	11	0	0.9449689
chr9	137297534	137384121	86588	3	0	6	0	0.02793176	0	10	0	0.74627054
chr9	137384121	137668297	284177	7	0	6	0	0.01518174	0	11	0	0.9449689
chr9	137668297	137887660	219364	6	0	6	0	0.02793176	0	10	0	0.74627054
chr9	137887660	137989962	102303	2	0	6	0	0.01518174	0	11	0	0.9449689
chr9	137989962	138205167	215206	3	0	6	0	0.02793176	0	10	0	0.74627054
chr9	138205167	138205226	60	1	0	6	0	0.00778066	0	12	0	1.17038931
chr9	138205226	138392136	186911	5	0	6	0	0.02793176	0	10	0	0.74627054
chr9	138392136	138417341	25206	3	0	6	0	0.01518174	0	11	0	0.9449689
chr9	138417341	138454735	37395	3	0	6	0	0.00778066	0	12	0	1.17038931
chr9	138454735	138517845	63111	1	0	6	0	0.01518174	0	11	0	0.9449689

chr9	138517845	138569148	51304	1	0	6	0	0.02793176	0	10	0	0.74627054
chr9	138569148	138691533	122386	4	0	7	0	0.05232577	0	10	0	0.56314362
chr9	138691533	138758222	66690	2	0	7	0	0.03037338	0	11	0	0.73110763
chr9	138758222	138758281	60	1	0	7	0	0.01667721	0	12	0	0.92532268
chr9	138758281	138824836	66556	1	0	6	0	0.04875589	0	9	0	0.5732208
chr9	138824836	138880824	55989	3	1	7	0.30102999	0.08584816	0	9	0	0.42015402
chr9	138880824	139088882	208059	5	1	7	0.1218695	0.08584816	1	9	0.1218695	0.42015402
chr9	139088882	139088941	60	1	1	7	0.1218695	0.05232577	1	10	0.1218695	0.56314362
chr9	139088941	139253928	164988	7	1	7	0.1218695	0.08584816	1	9	0.1218695	0.42015402
chr9	139253928	139299672	45745	3	2	7	0.30102999	0.08584816	1	9	0.05404976	0.42015402
chr9	139299672	139299731	60	1	2	7	0.30102999	0.05232577	1	10	0.05404976	0.56314362
chr9	139299731	139362970	63240	3	2	7	0.30102999	0.08584816	1	9	0.05404976	0.42015402
chr9	139362970	139363029	60	1	3	7	0.51676182	0.05232577	1	10	0.02438896	0.56314362
chr9	139363029	139378050	15022	1	1	7	0.1218695	0.08584816	1	9	0.1218695	0.42015402
chr9	139378050	139378107	58	1	2	7	0.30102999	0.08584816	1	9	0.05404976	0.42015402
chr9	139378107	139432550	54444	2	1	6	0.1218695	0.04875589	1	9	0.1218695	0.5732208
chr9	139432550	139432609	60	1	3	6	0.30102999	0.04875589	2	9	0.08289318	0.5732208
chr9	139432609	139503031	70423	1	2	6	0.1575501	0.04875589	2	9	0.1575501	0.5732208
chr9	139503031	139574425	71395	5	3	6	0.30102999	0.04875589	2	9	0.08289318	0.5732208
chr9	139574425	139607528	33104	2	3	7	0.30102999	0.08584816	2	9	0.08289318	0.42015402
chr9	139607528	139687162	79635	3	2	6	0.1575501	0.04875589	2	9	0.1575501	0.5732208
chr9	139687162	139687722	561	2	3	7	0.30102999	0.08584816	2	9	0.08289318	0.42015402
chr9	139687722	139722914	35193	2	2	6	0.1575501	0.04875589	2	9	0.1575501	0.5732208
chr9	139722914	139982580	259667	8	3	7	0.30102999	0.08584816	2	9	0.08289318	0.42015402
chr9	139982580	140087146	104567	5	4	7	0.47744371	0.05232577	2	10	0.0429175	0.56314362
chr9	140087146	140110329	23184	2	4	7	0.47744371	0.08584816	2	9	0.0429175	0.42015402
chr9	140110329	140110374	46	1	4	7	0.30102999	0.08584816	3	9	0.10122019	0.42015402
chr9	140110374	140164421	54048	1	4	6	0.30102999	0.08122616	3	8	0.10122019	0.4250187
chr9	140164421	140219048	54628	3	4	6	0.30102999	0.04875589	3	9	0.10122019	0.5732208
chr9	140219048	140219107	60	1	4	7	0.30102999	0.05232577	3	10	0.10122019	0.56314362
chr9	140219107	140287676	68570	1	4	6	0.30102999	0.04875589	3	9	0.10122019	0.5732208
chr9	140287676	140378700	91025	2	4	5	0.30102999	0.02473314	3	9	0.10122019	0.76806864
chr9	140378700	140378759	60	1	4	6	0.30102999	0.02793176	3	10	0.10122019	0.74627054
chr9	140378759	140482538	103780	3	4	4	0.30102999	0.02074938	3	8	0.10122019	0.79906872
chr9	140482538	140560792	78255	3	2	4	0.1575501	0.02074938	2	8	0.1575501	0.79906872
chr9	140560792	140560851	60	1	2	4	0.08289318	0.01077081	3	9	0.30102999	1.01542894
chr9	140560851	140590918	30068	1	2	3	0.08289318	0.0079614	3	8	0.30102999	1.07548421
chr9	140590918	140590977	60	1	3	3	0.17593012	0.00378107	3	9	0.17593012	1.33111237
chr9	140590977	140657526	66550	1	1	3	0.30102999	0.0079614	0	8	0	1.07548421
chr9	140657526	140707451	49926	1	1	3	0.30102999	0.03070643	0	6	0	0.63695542
chr9	140707451	140707504	54	1	2	3	0.61140001	0.03070643	0	6	0	0.63695542
chr9	140707504	140785691	78188	2	1	3	0.30102999	0.03070643	0	6	0	0.63695542
chr9	140785691	140878861	93171	2	1	2	0.30102999	0.02162467	0	5	0	0.68214471
chr9	140878861	141008915	130055	1	0	1	0	0.00478973	0	5	0	1.02643191
chr10	148206	148265	60	1	9	0	0.30102999	0	8	2	0.13872638	0.61140001
chr10	148265	248464	100200	2	7	0	0.13499366	0	8	2	0.30102999	0.61140001
chr10	248464	248523	60	1	8	0	0.20764654	0	8	2	0.20764654	0.61140001
chr10	248523	314639	66117	1	7	0	0.13499366	0	8	2	0.30102999	0.61140001
chr10	314639	314698	60	1	9	0	0.30102999	0	8	2	0.13872638	0.61140001
chr10	314698	412075	97378	2	7	0	0.13499366	0	8	2	0.30102999	0.61140001
chr10	412075	463007	50933	2	11	0	0.55623409	0	8	2	0.05490675	0.61140001
chr10	463007	556875	93869	3	12	0	0.55190077	0	9	2	0.0565833	0.61140001
chr10	556875	556934	60	1	12	0	0.41314172	0	10	2	0.09290028	0.61140001
chr10	556934	728425	171492	3	11	0	0.30102999	0	10	2	0.14303407	0.61140001
chr10	728425	873893	145469	4	13	0	0.5498098	0	10	2	0.0574087	0.61140001
chr10	873893	972595	98703	2	12	0	0.41314172	0	10	2	0.09290028	0.61140001
chr10	972595	1460405	487811	15	13	0	0.5498098	0	10	2	0.0574087	0.61140001
chr10	1460405	1793817	333413	6	11	0	0.30102999	0	10	2	0.14303407	0.61140001
chr10	1793817	2036190	242374	3	9	0	0.20975986	0	9	2	0.20975986	0.61140001
chr10	2036190	2096163	59974	1	7	0	0.13499366	0	8	1	0.30102999	0.30102999
chr10	2096163	2245932	149770	2	6	0	0.129913	0	7	1	0.30102999	0.30102999
chr10	2245932	2318372	72441	2	8	0	0.20764654	0	8	2	0.20764654	0.61140001
chr10	2318372	2318431	60	1	10	0	0.4167287	0	8	2	0.08923391	0.61140001
chr10	2318431	2417549	99119	1	10	0	0.4167287	0	8	1	0.08923391	0.30102999
chr10	2417549	2417608	60	1	11	0	0.41444892	0	9	1	0.09154957	0.30102999
chr10	2417608	2692948	275341	3	6	0	0.08122616	0	8	0	0.4250187	0
chr10	2692948	2693007	60	1	8	0	0.20764654	0	8	0	0.20764654	0
chr10	2693007	2921442	228436	3	6	0	0.08122616	0	8	0	0.4250187	0
chr10	2921442	3000869	79428	2	8	0	0.08923391	0	10	1	0.4167287	0.30102999
chr10	3000869	3124061	123193	3	8	0	0.05490675	0	11	1	0.55623409	0.30102999
chr10	3124061	3124120	60	1	9	0	0.09154957	0	11	1	0.41444892	0.30102999
chr10	3124120	3177933	53814	1	8	0	0.05490675	0	11	1	0.55623409	0.30102999
chr10	3177933	3178802	870	2	10	0	0.14303407	0	11	1	0.30102999	0.30102999
chr10	3178802	3233421	54620	6	11	0	0.21200206	0	11	1	0.21200206	0.30102999
chr10	3233421	3335606	102186	1	10	0	0.14303407	0	11	0	0.30102999	0
chr10	3335606	3335665	60	1	10	0	0.09290028	0	12	0	0.41314172	0
chr10	3335665	3469802	134138	2	8	0	0.03209037	0	12	0	0.72109894	0
chr10	3469802	3691660	221859	4	11	0	0.14385241	0	12	0	0.30102999	0
chr10	3691660	3735020	43361	2	13	0	0.30102999	0	12	0	0.14385241	0
chr10	3735020	3735079	60	1	14	0	0.41314172	0	12	0	0.09290028	0
chr10	3735079	3821288	86210	1	11	0	0.14385241	0	12	0	0.30102999	0
chr10	3821288	3823295	2008	3	11	0	0.14385241	0	12	1	0.30102999	0.30102999
chr10	3823295	3935490	112196	2	11	0	0.14385241	0	12	0	0.30102999	0
chr10	3935490	3935549	60	1	14	0	0.41314172	0	12	1	0.09290028	0.30102999
chr10	3935549	4049156	113608	1	12	0	0.21227046	0	12	1	0.21227046	0.30102999
chr10	4049156	4049215	60	1	12	0	0.14385241	0	13	1	0.30102999	0.30102999
chr10	4049215	4093817	44603	1	12	0	0.21227046	0	12	0	0.21227046	0
chr10	4093817	4133446	39630	2	14	0	0.41314172	0	12	0	0.09290028	0
chr10	4133446	4133505	60	1	14	0	0.30102999	0	13	0	0.14303407	0
chr10	4133505	4318888	185384	2	13	0	0.21200206	0	13	0	0.21200206	0
chr10	4318888	4318947	60	1	13	0	0.14303407	0	14	1	0.30102999	0.30102999
chr10	4318947	4396320	77374	1	13	0	0.21200206	0	13	0	0.21200206	0
chr10	4396320	4459570	63251	1	12	0	0.14385241	0	13	0	0.30102999	0
chr10	4459570	4584377	124808	3	12	0	0.14385241	0	13	1	0.30102999	0.30102999
chr10	4584377	4667016	82640	2	14	0	0.21118145	0	14	1	0.21118145	0.30102999
chr10	4667016	4823143	156128	2	12	0	0.09290028	0	14	1	0.41314172	0.30102999
chr10	4823143	4823202	60	1	15	0	0.30102999	0	14	2	0.14135546	0.61140001
chr10	4823202	4890258	67057	2	13	0	0.14303407	0	14	2	0.30102999	0.61140001
chr10	4890258	5042551	152294	3	13	0	0.21200206	0	13	2	0.21200206	0.61140001
chr10	5042551	5042610	60	1	15	0	0.20975986	0	15	2	0.20975986	0.61140001
chr10	5042610	5204511	161902	4	14	0	0.21118145	0	14	2	0.21118145	0.61140001
chr10	5204511	5302335	97825	3	14	0	0.30102999	0	13	2	0.14303407	0.61140001
chr10	5302335	5302394	60	1	15	0	0.30102999	0	14	2	0.14135546	0.61140001
chr10	5302394	5462228	159835	4	13	0	0.14303407	0	14	2	0.30102999	0.61140001
chr10	5462228	5488808	26581	2	14	0	0.21118145	0	14	2	0.21118145	0.61140001
chr10	5488808	5488867	60	1	15	0	0.30102999	0	14	2	0.14135546	0.61140001
chr10	5488867	5540688	51822	1	13	0	0.14303407	0	14	2	0.30102999	0.61140001
chr10	5540688	5568343	27656	1	13	0	0.21200206	0	13	2	0.21200206	0.61140001
chr10	5568343	5640964	72622	1	12	0	0.14385241	0	13	2	0.30102999	0.61140001
chr10	5640964	5641023	60	1	14	0	0.21118145	0	14	2	0.21118145	0.61140001
chr10	5641023	5772977	131955	6	13	0	0.14303407	0	14	2	0.30102999	0.61140001
chr10	5772977	5800865	27889	1	13	0	0.14303407	0	14	1	0.30102999	0.30102999
chr10	5800865	5800924	60	1	14	0	0.21118145	0	14	1	0.21118145	0.30102999
chr10	5800924	5838784	37861	1	13	0	0.14303407	0	14	1	0.30102999	0.30102999
chr10	5838784	5915788	77005	2	13	0	0.14303407	0	14	2	0.30102999	0.61140001
chr10	5915788	5955708	39921	1	12	0	0.09290028	0	14	2	0.41314172	0.61140001
chr10	5955708	6018355	62648	2	12	0	0.0565833	0	15	2	0.55190077	0.61140001
chr10	6018355	6018411	57	1	13	0	0.09154957	0	15	2	0.41444892	0.61140001
chr10	6018411	6135036	116626	3	13	0	0.14303407	0	14	2	0.30102999	0.61140001
chr10	6135036	6135095	60	1	13	0	0.09154957	0	15	2	0.41444892	0.61140001
chr10	6135095	6247262	112168	2	13	0	0.14303407	0	14	2	0.30102999	0.61140001
chr10	6247262	6370563	123302	5	12	0	0.09290028	0	14	2	0.41314172	0.61140001
chr10	6370563	6449053	78491	2	13	0	0.14303407	0	14	2	0.30102999	0.61140001
chr10	6449053	6449112	60	1	14	0	0.21118145	0	14	2	0.21118145	0.61140001

chr10	6449112	6600019	150908	4	13	0	0.14303407	0	14	2	0.30102999	0.61140001
chr10	6600019	6633590	33572	1	13	0	0.21200206	0	13	2	0.21200206	0.61140001
chr10	6633590	7002284	368695	6	14	0	0.30102999	0	13	2	0.14303407	0.61140001
chr10	7002284	7002343	60	1	15	0	0.41444892	0	13	2	0.09154957	0.61140001
chr10	7002343	7069636	67294	1	14	0	0.30102999	0	13	2	0.14303407	0.61140001
chr10	7069636	7131652	62017	1	13	0	0.21200206	0	13	2	0.21200206	0.61140001
chr10	7131652	7131711	60	1	14	0	0.30102999	0	13	2	0.14303407	0.61140001
chr10	7131711	7250691	118981	2	12	0	0.14385241	0	13	2	0.30102999	0.61140001
chr10	7250691	7250750	60	1	14	0	0.30102999	0	13	2	0.14303407	0.61140001
chr10	7250750	7351799	101050	2	12	0	0.14385241	0	13	2	0.30102999	0.61140001
chr10	7351799	7386119	34321	2	13	0	0.21200206	0	13	2	0.21200206	0.61140001
chr10	7386119	7416722	30604	2	14	0	0.30102999	0	13	2	0.14303407	0.61140001
chr10	7416722	7470791	54070	1	13	0	0.21200206	0	13	2	0.21200206	0.61140001
chr10	7470791	7565181	94391	1	12	0	0.14385241	0	13	2	0.30102999	0.61140001
chr10	7565181	7565240	60	1	13	0	0.21200206	0	13	2	0.21200206	0.61140001
chr10	7565240	7627978	62739	5	11	0	0.09334429	0	13	2	0.41271556	0.61140001
chr10	7627978	7722648	94671	2	11	0	0.14385241	0	12	2	0.30102999	0.61140001
chr10	7722648	7772056	49409	2	12	0	0.14385241	0	13	2	0.30102999	0.61140001
chr10	7772056	7814191	42136	4	10	0	0.0574087	0	13	2	0.5498098	0.61140001
chr10	7814191	7891298	77108	2	10	0	0.0574087	0	13	1	0.5498098	0.30102999
chr10	7891298	7934635	43338	2	10	0	0.0574087	0	13	2	0.5498098	0.61140001
chr10	7934635	8116684	182050	7	11	0	0.09334429	0	13	2	0.41271556	0.61140001
chr10	8116684	8215858	99175	1	9	0	0.0331093	0	13	1	0.71538971	0.30102999
chr10	8215858	8588248	372391	2	7	0	0.05232577	0	10	1	0.56314362	0.30102999
chr10	8588248	8588307	60	1	7	0	0.03037338	0	11	1	0.73110763	0.30102999
chr10	8588307	8683328	95022	1	6	0	0.02793176	0	10	1	0.74627054	0.30102999
chr10	8683328	8774576	91249	1	5	0	0.02473314	0	9	1	0.76806864	0.30102999
chr10	8774576	8994869	220294	1	5	0	0.07511598	0	7	1	0.43181735	0.30102999
chr10	8994869	9190795	195927	1	2	0	0.0429175	0	4	0	0.47744371	0
chr10	9190795	9352537	161743	2	3	0	0.10122019	0	4	0	0.30102999	0
chr10	9352537	9513006	160470	1	2	0	0.0429175	0	4	0	0.47744371	0
chr10	9513006	9645787	132782	1	1	0	0.01091641	0	4	0	0.76005302	0
chr10	9846848	9846907	60	1	3	0	0.05670724	0	5	0	0.45545077	0
chr10	9846907	10047084	200178	1	3	0	0.10122019	0	4	0	0.30102999	0
chr10	10047084	10221742	174659	1	2	0	0.0429175	0	4	0	0.47744371	0
chr10	10221742	10221801	60	1	5	0	0.04407651	0	8	0	0.58747015	0
chr10	10221801	10358905	137105	1	5	0	0.07511598	0	7	0	0.43181735	0
chr10	10358905	10358964	60	1	6	0	0.08122616	0	8	0	0.4250187	0
chr10	10358964	10797860	438897	2	5	0	0.04407651	0	8	0	0.58747015	0
chr10	10797860	10844699	46840	2	6	0	0.02793176	0	10	0	0.74627054	0
chr10	10844699	11091335	246637	2	6	0	0.04875589	0	9	0	0.5732208	0
chr10	11091335	11371351	280017	9	6	0	0.02793176	0	10	0	0.74627054	0
chr10	11371351	11576958	205608	5	6	0	0.01518174	0	11	0	0.9449689	0
chr10	11576958	11611794	34837	2	9	0	0.0565833	0	12	0	0.55190077	0
chr10	11611794	11722207	110414	2	8	0	0.03209037	0	12	0	0.72109894	0
chr10	11722207	11722266	60	1	9	0	0.0565833	0	12	0	0.55190077	0
chr10	11722266	11794942	72677	3	9	0	0.09154957	0	11	0	0.41444892	0
chr10	11794942	11895834	100893	2	8	0	0.05490675	0	11	0	0.55623409	0
chr10	11895834	12114557	218724	6	9	0	0.09154957	0	11	0	0.41444892	0
chr10	12114557	12114616	60	1	11	0	0.21200206	0	11	0	0.21200206	0
chr10	12114616	12228207	113592	5	10	0	0.14303407	0	11	0	0.30102999	0
chr10	12228207	12280441	52235	3	13	0	0.41271556	0	11	0	0.09334429	0
chr10	12280441	12280500	60	1	14	0	0.5498098	0	11	0	0.0574087	0
chr10	12280500	12446689	166190	3	12	0	0.30102999	0	11	0	0.14385241	0
chr10	12446689	12512466	65778	1	11	0	0.21200206	0	11	0	0.21200206	0
chr10	12512466	12539603	27138	1	10	0	0.14303407	0	11	0	0.30102999	0
chr10	12539603	12613843	74241	2	10	0	0.14303407	0	11	1	0.30102999	0.30102999
chr10	12613843	12706318	92476	3	11	0	0.21200206	0	11	1	0.21200206	0.30102999
chr10	12706318	12766606	60289	1	10	0	0.14303407	0	11	1	0.30102999	0.30102999
chr10	12766606	12857914	91309	2	10	0	0.21118145	0	10	0	0.21118145	0
chr10	12857914	13001474	143561	4	10	0	0.14303407	0	11	0	0.30102999	0
chr10	13001474	13160865	159392	4	15	0	0.55190077	0	12	0	0.0565833	0
chr10	13160865	13160924	60	1	15	0	0.41444892	0	13	0	0.09154957	0
chr10	13160924	13251149	90226	3	14	0	0.41314172	0	12	0	0.09290028	0
chr10	13251149	13251208	60	1	14	0	0.21118145	0	14	0	0.21118145	0
chr10	13251208	13271658	20451	1	14	0	0.30102999	0	13	0	0.14303407	0
chr10	13271658	13336604	64947	1	14	0	0.41314172	0	12	0	0.09290028	0
chr10	13336604	13380327	43724	1	13	0	0.30102999	0	12	0	0.14385241	0
chr10	13380327	13483445	103119	1	13	0	0.41271556	0	11	0	0.09334429	0
chr10	13483445	13642272	158828	4	12	0	0.30102999	0	11	0	0.14385241	0
chr10	13642272	13773041	130770	4	13	0	0.41271556	0	11	0	0.09334429	0
chr10	13773041	13880015	106975	3	13	0	0.30102999	0	12	0	0.14385241	0
chr10	13880015	13904118	24104	2	13	0	0.21200206	0	13	0	0.21200206	0
chr10	13904118	13904177	60	1	13	0	0.09154957	0	15	0	0.41444892	0
chr10	13904177	14050271	146095	3	13	0	0.14303407	0	14	0	0.30102999	0
chr10	14050271	14050330	60	1	14	0	0.21118145	0	14	0	0.21118145	0
chr10	14050330	14108879	58550	1	14	0	0.41314172	0	12	0	0.09290028	0
chr10	14108879	14158802	49924	2	14	0	0.30102999	0	13	0	0.14303407	0
chr10	14158802	14268036	109235	2	13	0	0.30102999	0	12	0	0.14385241	0
chr10	14268036	14329017	60982	2	14	0	0.30102999	0	13	0	0.14303407	0
chr10	14329017	14372661	43645	1	14	0	0.41314172	0	12	0	0.09290028	0
chr10	14372661	14496155	123495	3	13	0	0.30102999	0	12	0	0.14385241	0
chr10	14496155	14496214	60	1	13	0	0.21200206	0	13	0	0.21200206	0
chr10	14496214	14635361	139148	2	13	0	0.41271556	0	11	0	0.09334429	0
chr10	14635361	14724086	88726	2	13	0	0.5498098	0	10	0	0.0574087	0
chr10	14724086	14778772	54687	1	12	0	0.41314172	0	10	0	0.09290028	0
chr10	14778772	14869993	91222	3	12	0	0.30102999	0	11	0	0.14385241	0
chr10	14869993	15009438	139446	4	11	0	0.30102999	0	10	0	0.14303407	0
chr10	15009438	15106486	97049	3	11	0	0.30102999	0	10	1	0.14303407	0.30102999
chr10	15106486	15150846	44361	4	10	0	0.21118145	0	10	1	0.21118145	0.30102999
chr10	15150846	15415335	264490	7	9	0	0.14135546	0	10	1	0.30102999	0.30102999
chr10	15415335	15504365	89031	1	7	0	0.05232577	0	10	1	0.56314362	0.30102999
chr10	15504365	15728129	223765	6	8	0	0.08923391	0	10	1	0.4167287	0.30102999
chr10	15728129	15824162	96034	2	6	0	0.02793176	0	10	1	0.74627054	0.30102999
chr10	15824162	15975054	150893	3	8	0	0.08923391	0	10	1	0.4167287	0.30102999
chr10	15975054	15975113	60	1	9	0	0.14135546	0	10	1	0.30102999	0.30102999
chr10	15975113	16042161	67049	1	9	0	0.14135546	0	10	0	0.30102999	0
chr10	16042161	16151160	109000	1	7	0	0.05232577	0	10	0	0.56314362	0
chr10	16151160	16300979	149820	1	6	0	0.02793176	0	10	0	0.74627054	0
chr10	16300979	16301038	60	1	8	0	0.03209037	0	12	1	0.72109894	0.30102999
chr10	16301038	16446128	145091	1	6	0	0.00778066	0	12	1	1.17038931	0.30102999
chr10	16446128	16503642	57515	2	7	0	0.00859896	0	13	1	1.14735594	0.30102999
chr10	16503642	16503701	60	1	9	0	0.0331093	0	13	1	0.71538971	0.30102999
chr10	16503701	16553412	49712	2	7	0	0.00859896	0	13	0	1.14735594	0
chr10	16553412	16553471	60	1	7	0	0.00859896	0	13	1	1.14735594	0.30102999
chr10	16553471	16736998	183528	3	7	0	0.00859896	0	13	0	1.14735594	0
chr10	16736998	16786323	49326	2	8	0	0.01767679	0	13	0	0.91308053	0
chr10	16786323	16786382	60	1	9	0	0.0331093	0	13	0	0.71538971	0
chr10	16786382	16911673	125292	3	8	0	0.01767679	0	13	0	0.91308053	0
chr10	16911673	16944583	32911	2	9	0	0.0331093	0	13	0	0.71538971	0
chr10	16944583	16992117	47535	1	8	0	0.01767679	0	13	0	0.91308053	0
chr10	16992117	17051087	58971	1	7	0	0.00859896	0	13	0	1.14735594	0
chr10	17051087	17539377	488291	14	8	0	0.01767679	0	13	0	0.91308053	0
chr10	17539377	17539436	60	1	9	0	0.01817691	0	14	0	0.90719478	0
chr10	17539436	17622519	83084	1	8	0	0.01767679	0	13	0	0.91308053	0
chr10	17622519	17622578	60	1	9	0	0.01817691	0	14	0	0.90719478	0
chr10	17622578	17688018	65441	2	7	0	0.00413023	0	14	0	1.39918918	0
chr10	17688018	17726891	38874	1	6	0	0.00166733	0	14	0	1.70763027	0
chr10	17726891	17757105	30215	2	6	0	6.86E-04	0	15	0	2.02387299	0
chr10	17757105	17758440	1336	2	7	0	0.00183109	0	15	0	1.68336308	0
chr10	17758440	18393455	635016	4	4	0	0.00247414	0	11	0	1.52371709	0
chr10	18393455	18439840	46386	1	3	0	0.00170589	0	10	0	1.61091002	0
chr10	18439840	18439899	60	1	3	0	7.28E-04	0	11	0	1.91540774	0
chr10	18439899	18515359	75461	1	3	0	0.00378107	0	9	0	1.33111237	0

chr10	18515359	18549858	34500	2	3	0	0.00170589	0	10	0	1.61091002	0
chr10	18549858	18618376	68519	3	3	0	2.93E-04	0	12	0	2.24559516	0
chr10	18618376	18652192	33817	1	3	0	7.28E-04	0	11	0	1.91540774	0
chr10	18652192	18699314	47123	2	3	0	0.00170589	0	10	0	1.61091002	0
chr10	18699314	18699373	60	1	3	0	2.93E-04	0	12	0	2.24559516	0
chr10	18699373	18949530	250158	5	3	0	0.00378107	0	9	0	1.33111237	0
chr10	18949530	19010007	60478	3	3	0	0.00170589	0	10	0	1.61091002	0
chr10	19010007	19252784	242778	2	3	0	0.0079614	0	8	0	1.07548421	0
chr10	19252784	19346555	93772	2	3	0	0.00378107	0	9	0	1.33111237	0
chr10	19346555	19536529	189975	3	3	0	7.28E-04	0	11	0	1.91540774	0
chr10	19536529	19650514	113986	1	3	0	0.00170589	0	10	0	1.61091002	0
chr10	19650514	19650573	60	1	3	0	7.28E-04	0	11	0	1.91540774	0
chr10	19650573	19906612	256040	4	3	0	0.00378107	0	9	0	1.33111237	0
chr10	19906612	20105838	199227	3	3	0	0.0079614	0	8	0	1.07548421	0
chr10	20105838	20311068	205231	5	4	0	0.01077081	0	9	0	1.01542894	0
chr10	20311068	20357071	46004	2	4	0	0.00108487	0	12	0	1.81706455	0
chr10	20357071	20357130	60	1	6	0	0.00778066	0	12	0	1.17038931	0
chr10	20357130	20506343	149214	3	4	0	0.00530919	0	10	0	1.2568129	0
chr10	20506343	20506402	60	1	6	0	0.01518174	0	11	0	0.9449689	0
chr10	20506402	20589017	82616	1	6	0	0.02793176	0	10	0	0.74627054	0
chr10	20589017	20589076	60	1	6	0	0.01518174	0	11	0	0.9449689	0
chr10	20589076	20695175	106100	1	5	0	0.00666883	0	11	0	1.20557689	0
chr10	20695175	20825532	130358	2	6	0	0.01518174	0	11	0	0.9449689	0
chr10	20825532	20825591	60	1	7	0	0.00859896	0	13	0	1.14735594	0
chr10	20825591	21070477	244887	2	6	0	0.00778066	0	12	0	1.17038931	0
chr10	21070477	21102908	32432	2	7	0	0.01667721	0	12	0	0.92532268	0
chr10	21102908	21445496	342589	7	6	0	0.01518174	0	11	0	0.9449689	0
chr10	21445496	21551827	106332	3	6	0	0.00778066	0	12	0	1.17038931	0
chr10	21551827	21757087	205261	2	5	0	0.00317045	0	12	0	1.46403142	0
chr10	21757087	21883121	126035	5	7	0	0.00859896	0	13	0	1.14735594	0
chr10	21883121	22002747	119627	2	7	0	0.01667721	0	12	0	0.92532268	0
chr10	22002747	22065396	62650	2	7	0	0.03037338	0	11	0	0.73110763	0
chr10	22065396	22146175	80780	2	7	0	0.01667721	0	12	0	0.92532268	0
chr10	22146175	22146234	60	1	7	0	0.00859896	0	13	0	1.14735594	0
chr10	22146234	22217411	71178	1	7	0	0.01667721	0	12	0	0.92532268	0
chr10	22217411	22217470	60	1	7	0	0.00859896	0	13	0	1.14735594	0
chr10	22217470	22262394	44925	1	6	0	0.01518174	0	11	0	0.9449689	0
chr10	22262394	22394622	132229	3	7	0	0.01667721	0	12	0	0.92532268	0
chr10	22394622	22532652	138031	3	9	0	0.0331093	0	13	0	0.71538971	0
chr10	22532652	22606298	73647	2	9	0	0.01817691	0	14	0	0.90719478	0
chr10	22606298	22639692	33395	3	9	0	0.0331093	0	13	0	0.71538971	0
chr10	22639692	22825792	186101	4	9	0	0.01817691	0	14	0	0.90719478	0
chr10	22825792	22844450	18659	2	9	0	0.00926608	0	15	0	1.13004284	0
chr10	22844450	22950715	106266	2	9	0	0.0331093	0	13	0	0.71538971	0
chr10	22950715	22991713	40999	2	9	0	0.01817691	0	14	0	0.90719478	0
chr10	22991713	23041802	50090	1	9	0	0.0565833	0	12	0	0.55190077	0
chr10	23041802	23041861	60	1	10	0	0.09290028	0	12	0	0.41314172	0
chr10	23041861	23089529	47669	1	10	0	0.14303407	0	11	0	0.30102999	0
chr10	23089529	23089588	60	1	10	0	0.0574087	0	13	0	0.5498098	0
chr10	23089588	23190112	100525	1	10	0	0.09290028	0	12	0	0.41314172	0
chr10	23190112	23220899	30788	2	10	0	0.03344703	0	14	0	0.71353304	0
chr10	23220899	23257288	36390	2	10	0	0.01817691	0	15	0	0.90719478	0
chr10	23257288	23377491	120204	3	9	0	0.0331093	0	13	0	0.71538971	0
chr10	23377491	23377550	60	1	10	0	0.0574087	0	13	0	0.5498098	0
chr10	23377550	23409844	32295	1	9	0	0.0331093	0	13	0	0.71538971	0
chr10	23409844	23447940	38097	2	9	0	0.00433294	0	16	0	1.3873209	0
chr10	23447940	23553937	105998	3	7	0	0.00413023	0	14	0	1.39918918	0
chr10	23553937	23609705	55769	2	9	0	0.00926608	0	15	0	1.13004284	0
chr10	23609705	23617487	7783	2	9	0	0.00433294	0	16	0	1.3873209	0
chr10	23617487	23678708	61222	2	10	0	0.00909834	0	16	0	1.13428373	0
chr10	23678708	23678767	60	1	10	0	0.00413023	0	17	0	1.39918918	0
chr10	23678767	23725958	47192	1	9	0	0.00433294	0	16	0	1.3873209	0
chr10	23725958	23899594	173637	1	7	0	0.00183109	0	15	0	1.68336308	0
chr10	23899594	24005940	106347	2	7	0	2.70E-04	0	17	0	2.36290411	0
chr10	24005940	24060390	54451	3	7	0	8.69E-05	0	18	1	2.76830915	0.30102999
chr10	24060390	24177159	116770	2	7	0	2.70E-04	0	17	1	2.36290411	0.30102999
chr10	24177159	24283796	106638	3	8	0	2.57E-04	0	18	1	2.37598969	0.30102999
chr10	24283796	24400329	116534	3	8	0	7.41E-04	0	17	0	2.00316618	0
chr10	24400329	24526574	126246	3	8	0	0.00188722	0	16	0	1.67551277	0
chr10	24526574	24562280	35707	2	7	0	0.00183109	0	15	0	1.68336308	0
chr10	24562280	24604763	42484	3	7	0	7.41E-04	0	16	0	2.00316618	0
chr10	24604763	24716756	111994	2	6	0	6.86E-04	0	15	0	2.02387299	0
chr10	24716756	24716815	60	1	7	0	7.41E-04	0	16	0	2.00316618	0
chr10	24716815	25089237	372423	7	6	0	2.57E-04	0	16	0	2.37598969	0
chr10	25089237	25181647	92411	3	6	0	6.86E-04	0	15	0	2.02387299	0
chr10	25181647	25279466	97820	2	6	0	0.00166733	0	14	0	1.70763027	0
chr10	25279466	25482892	203427	5	6	0	2.57E-04	0	16	0	2.37598969	0
chr10	25482892	25534772	51881	1	6	0	6.86E-04	0	15	0	2.02387299	0
chr10	25534772	25578708	43937	1	6	0	0.00166733	0	14	0	1.70763027	0
chr10	25578708	25697468	118761	2	6	0	0.00373523	0	13	0	1.42397267	0
chr10	25697468	25735095	37628	1	6	0	0.01518174	0	11	0	0.9449689	0
chr10	25735095	25773461	38367	1	5	0	0.01320236	0	10	0	0.97390707	0
chr10	25773461	26106103	332643	5	5	0	0.02473314	0	9	0	0.76806864	0
chr10	26106103	26459434	353332	9	6	0	0.04875589	0	9	0	0.5732208	0
chr10	26459434	26611986	152553	3	5	0	0.02473314	0	9	0	0.76806864	0
chr10	26611986	26727223	115238	2	6	0	0.04875589	0	9	0	0.5732208	0
chr10	26727223	26727268	46	1	6	0	0.02793176	0	10	0	0.74627054	0
chr10	26727268	26822412	95145	2	5	0	0.01320236	0	10	0	0.97390707	0
chr10	26822412	26822471	60	1	6	0	0.02793176	0	10	0	0.74627054	0
chr10	26822471	26858131	35661	2	5	0	0.01320236	0	10	0	0.97390707	0
chr10	26858131	26991816	133686	1	5	0	0.02473314	0	9	0	0.76806864	0
chr10	26991816	27091081	99266	3	6	0	0.02793176	0	10	0	0.74627054	0
chr10	27091081	27147044	55964	1	5	0	0.01320236	0	10	0	0.97390707	0
chr10	27147044	27147103	60	1	5	0	0.00317045	0	12	0	1.46403142	0
chr10	27147103	27266067	118965	1	5	0	0.02473314	0	9	0	0.76806864	0
chr10	27266067	27294439	28373	1	5	0	0.01320236	0	10	0	0.97390707	0
chr10	27294439	27313441	19003	2	5	0	0.00666883	0	11	0	1.20557689	0
chr10	27313441	27313500	60	1	6	0	0.01518174	0	11	0	0.9449689	0
chr10	27313500	27415826	102327	3	5	0	0.01320236	0	10	0	0.97390707	0
chr10	27415826	27444025	28200	2	5	0	0.00666883	0	11	0	1.20557689	0
chr10	27444025	27444075	51	1	6	0	0.01518174	0	11	0	0.9449689	0
chr10	27444075	27461727	17653	1	5	0	0.00666883	0	11	0	1.20557689	0
chr10	27461727	27520665	58939	3	5	0	0.00317045	0	12	0	1.46403142	0
chr10	27520665	27529036	8372	2	6	0	0.00778066	0	12	0	1.17038931	0
chr10	27529036	27578447	49412	1	6	0	0.01518174	0	11	0	0.9449689	0
chr10	27578447	27578506	60	1	7	0	0.03037338	0	11	0	0.73110763	0
chr10	27578506	27634143	55638	1	7	0	0.05232577	0	10	0	0.56314362	0
chr10	27634143	27875075	240933	4	5	0	0.01320236	0	10	0	0.97390707	0
chr10	27875075	27985285	110211	3	5	0	0.02473314	0	9	0	0.76806864	0
chr10	27985285	28036032	50748	2	6	0	0.00373523	0	13	0	1.42397267	0
chr10	28036032	28101342	65311	1	6	0	0.01518174	0	11	0	0.9449689	0
chr10	28101342	28151475	50134	1	6	0	0.02793176	0	10	0	0.74627054	0
chr10	28151475	28196671	45197	1	6	0	0.04875589	0	9	0	0.5732208	0
chr10	28196671	28347467	150797	5	6	0	0.02793176	0	10	0	0.74627054	0
chr10	28347467	28489598	142132	3	6	0	0.04875589	0	9	0	0.5732208	0
chr10	28489598	28489657	60	1	6	0	0.02793176	0	10	0	0.74627054	0
chr10	28489657	28649980	160324	5	6	0	0.04875589	0	9	0	0.5732208	0
chr10	28649980	28824570	174591	3	5	0	0.02473314	0	9	0	0.76806864	0
chr10	28824570	28911499	86930	3	7	0	0.08584816	0	9	0	0.42015402	0
chr10	28911499	28911558	60	1	8	0	0.13872638	0	9	0	0.30102999	0
chr10	28911558	29017336	105779	2	7	0	0.08584816	0	9	0	0.42015402	0
chr10	29017336	29017395	60	1	8	0	0.08923391	0	10	0	0.4167287	0
chr10	29017395	29149171	131777	2	7	0	0.08584816	0	9	0	0.42015402	0
chr10	29149171	29149230	60	1	8	0	0.05490675	0	11	0	0.55623409	0

chr10	29149230	29309540	160311	3	8	0	0.13872638	0	9	0	0.30102999	0	
chr10	29309540	29309599	60	1	9	0	0.14135546	0	10	0	0.30102999	0	
chr10	29309599	29494622	185024	1	8	0	0.08923391	0	10	0	0.4167287	0	
chr10	29494622	29582455	87834	2	8	0	0.05490675	0	11	0	0.55623409	0	
chr10	29582455	29664214	81760	2	9	0	0.09154957	0	11	0	0.41444892	0	
chr10	29664214	29764262	100049	2	9	0	0.0331093	0	13	0	0.71538971	0	
chr10	29764262	29923752	159491	6	9	0	0.01817691	0	14	0	0.90719478	0	
chr10	29923752	30017357	93606	2	9	0	0.0565833	0	12	0	0.55190077	0	
chr10	30017357	30135457	118101	2	8	0	0.08923391	0	10	0	0.4167287	0	
chr10	30135457	30190964	55508	2	9	1	0.0565833	0.30102999	12	0	0.55190077	0	
chr10	30190964	30275294	84331	1	9	1	0.14135546	0.30102999	10	0	0.30102999	0	
chr10	30275294	30406831	131538	3	7	1	0.05232577	0.30102999	10	0	0.56314362	0	
chr10	30406831	30537801	130971	1	6	1	0.02793176	0.30102999	10	0	0.74627054	0	
chr10	30537801	30537860	60	1	6	1	0.02793176	0.1218695	10	1	0.74627054	0.1218695	
chr10	30537860	30661355	123496	3	5	0	0.01320236	0	10	0	0.97390707	0	
chr10	30661355	30661414	60	1	5	0	0.01320236	0	10	1	0.97390707	0.30102999	
chr10	30661414	30728137	66724	1	5	0	0.01320236	0	10	0	0.97390707	0	
chr10	30728137	30750028	21892	4	6	0	0.02793176	0	10	0	0.74627054	0	
chr10	30750028	30750087	60	1	7	0	0.03037338	0	11	0	0.73110763	0	
chr10	30750087	30822470	72384	1	6	0	0.02793176	0	10	0	0.74627054	0	
chr10	30822470	30961827	139358	2	4	0	0.02074938	0	8	0	0.79906872	0	
chr10	30961827	31156591	194765	2	3	0	0.01598258	0	7	0	0.84395715	0	
chr10	31156591	31156650	60	1	4	0	0.03812622	0	7	0	0.60763643	0	
chr10	31156650	31291873	135224	3	3	0	0.01598258	0	7	0	0.84395715	0	
chr10	31291873	31291932	60	1	4	0	0.03812622	0	7	0	0.60763643	0	
chr10	31291932	31387211	95280	1	3	0	0.01598258	0	7	0	0.84395715	0	
chr10	31387211	31507862	120652	2	4	0	0.02074938	0	8	0	0.79906872	0	
chr10	31507862	31551563	43702	2	5	0	0.04407651	0	8	0	0.58747015	0	
chr10	31551563	31637549	85987	2	4	0	0.02074938	0	8	0	0.79906872	0	
chr10	31637549	31637608	60	1	5	0	0.04407651	0	8	0	0.58747015	0	
chr10	31637608	31715824	78217	2	5	0	0.07511598	0	7	0	0.43181735	0	
chr10	31715824	32012867	297044	5	5	0	0.12309572	0	6	0	0.30102999	0	
chr10	32012867	32166608	153742	4	3	0	0.03070643	0	6	0	0.63695542	0	
chr10	32166608	32197507	30900	2	4	0	0.03812622	0	7	0	0.60763643	0	
chr10	32197507	32300151	102645	2	3	0	0.01598258	0	7	0	0.84395715	0	
chr10	32300151	32300210	60	1	4	0	0.02074938	0	8	0	0.79906872	0	
chr10	32300210	32505928	205719	5	3	0	0.0079614	0	8	0	1.07548421	0	
chr10	32505928	32505987	60	1	5	0	0.04407651	0	8	0	0.58747015	0	
chr10	32505987	32627962	121976	2	4	0	0.03812622	0	7	0	0.60763643	0	
chr10	32627962	32628021	60	1	4	0	0.02074938	0	8	0	0.79906872	0	
chr10	32628021	32779348	151328	3	4	0	0.03812622	0	7	0	0.60763643	0	
chr10	32779348	32952767	173420	4	5	0	0.07511598	0	7	0	0.43181735	0	
chr10	32952767	32978070	25304	2	5	0	0.02473314	0	9	0	0.76806864	0	
chr10	32978070	33093075	115006	2	5	0	0.04407651	0	8	0	0.58747015	0	
chr10	33093075	33093134	60	1	5	0	0.02473314	0	9	0	0.76806864	0	
chr10	33093134	33165392	72259	3	5	0	0.04407651	0	8	0	0.58747015	0	
chr10	33165392	33224403	59012	1	4	0	0.03812622	0	7	0	0.60763643	0	
chr10	33224403	33244547	20145	3	4	0	0.02074938	0	8	0	0.79906872	0	
chr10	33244547	33491224	246678	3	3	0	0.03070643	0	6	0	0.63695542	0	
chr10	33491224	33491282	59	1	4	0	0.03812622	0	7	0	0.60763643	0	
chr10	33491282	33550158	58877	1	3	0	0.01598258	0	7	0	0.84395715	0	
chr10	33550158	33582780	32623	1	3	0	0.03070643	0	6	0	0.63695542	0	
chr10	33582780	33582839	60	1	4	0	0.06713722	0	6	0	0.44141547	0	
chr10	33582839	33606406	23568	1	3	0	0.03070643	0	6	0	0.63695542	0	
chr10	33606406	33778244	171839	3	3	0	0.01598258	0	7	0	0.84395715	0	
chr10	33778244	33778303	60	1	3	0	0.0079614	0	8	0	1.07548421	0	
chr10	33778303	33938406	160104	1	3	0	0.01598258	0	7	0	0.84395715	0	
chr10	33938406	34057481	119076	2	4	0	0.03812622	0	7	0	0.60763643	0	
chr10	34057481	34232509	175029	2	5	0	0.07511598	0	7	0	0.43181735	0	
chr10	34232509	34232568	60	1	6	0	0.00778066	0	12	0	1.17038931	0	
chr10	34232568	34484667	252100	3	5	0	0.01320236	0	10	0	0.97390707	0	
chr10	34484667	34484725	59	1	6	0	0.02793176	0	10	0	0.74627054	0	
chr10	34484725	34537239	52515	1	5	0	0.01320236	0	10	0	0.97390707	0	
chr10	34537239	34537298	60	1	5	0	0.00666883	0	11	0	1.20557689	0	
chr10	34537298	34626197	88900	2	5	0	0.01320236	0	10	0	0.97390707	0	
chr10	34626197	34715611	89415	3	5	0	0.00666883	0	11	0	1.20557689	0	
chr10	34715611	34715670	60	1	5	0	0.00317045	0	12	0	1.46403142	0	
chr10	34715670	34885774	170105	5	5	0	0.01320236	0	10	0	0.97390707	0	
chr10	34885774	34985340	99567	2	5	0	0.04407651	0	8	0	0.58747015	0	
chr10	34985340	35391918	406579	10	6	0	0.04875589	0	9	1	0.5732208	0.30102999	
chr10	35391918	35530457	138540	3	4	0	0.01077081	0	9	1	1.01542894	0.30102999	
chr10	35530457	35605447	74991	1	4	0	0.03812622	0	7	1	0.60763643	0.30102999	
chr10	35605447	35652737	47291	2	5	0	0.07511598	0	7	1	0.43181735	0.30102999	
chr10	35652737	35760318	107582	2	4	0	0.03812622	0	7	1	0.60763643	0.30102999	
chr10	35760318	35790460	30143	2	5	0	0.04407651	0	8	1	0.58747015	0.30102999	
chr10	35790460	35823303	32844	1	4	0	0.02074938	0	8	1	0.79906872	0.30102999	
chr10	35823303	35823354	52	1	5	0	0.02473314	0	9	1	0.76806864	0.30102999	
chr10	35823354	35927794	104441	3	3	0	0.03070643	0	6	1	0.63695542	0.30102999	
chr10	35927794	36238765	310972	3	1	0	0.00478973	0	5	1	1.02643191	0.30102999	
chr10	36238765	36238824	60	1	1	0	0.00204627	0	6	1	1.31360226	0.30102999	
chr10	36238824	36418814	179991	1	1	0	0.00478973	0	5	1	1.02643191	0.30102999	
chr10	36418814	36912799	493986	4	1	0	0.02438896	0	3	0	0.51676182	0	
chr10	42889244	42969765	80522	2	1	0	0.02438896	0	3	1	0.51676182	0.30102999	
chr10	42969765	42998583	28819	3	1	0	0.02438896	0	3	2	0.51676182	0.61140001	
chr10	42998583	43238249	239667	5	1	0	0.01091641	0	4	2	0.76005302	0.61140001	
chr10	43238249	43818029	579781	13	2	0	0.0429175	0	4	2	0.47744371	0.61140001	
chr10	43818029	43818088	60	1	2	0	0.02162467	0	5	2	0.68214471	0.61140001	
chr10	43818088	43868297	50210	1	2	0	0.0429175	0	4	2	0.47744371	0.61140001	
chr10	43868297	43868356	60	1	2	0	0.02162467	0	5	2	0.68214471	0.61140001	
chr10	43868356	43949913	81558	2	2	0	0.0429175	0	4	1	0.47744371	0.30102999	
chr10	43949913	43949972	60	1	2	0	0.0429175	0	4	2	0.47744371	0.61140001	
chr10	43949972	44017493	67522	2	1	0	0.02438896	0	3	2	0.51676182	0.61140001	
chr10	44017493	44104662	87170	3	2	0	0.08289318	0	3	2	0.30102999	0.61140001	
chr10	44104662	44139435	34774	2	2	0	0.0429175	0	4	2	0.47744371	0.61140001	
chr10	44139435	44353706	214272	4	2	0	0.08289318	0	3	2	0.30102999	0.61140001	
chr10	44353706	44353765	60	1	2	0	0.0429175	0	4	3	0.47744371	0.93173516	
chr10	44353765	44484281	130517	2	2	0	0.0429175	0	4	2	0.47744371	0.61140001	
chr10	44484281	44706857	222577	2	2	0	0.0429175	0	4	3	0.47744371	0.93173516	
chr10	44706857	44706916	60	1	2	0	0.02162467	0	5	3	0.68214471	0.93173516	
chr10	44706916	44867043	160128	2	1	0	0.00478973	0	5	2	1.02643191	0.61140001	
chr10	44867043	44877427	10385	2	1	0	0.02438896	0	3	2	0.51676182	0.61140001	
chr10	44877427	44879020	1594	2	1	0	0.00478973	0	5	2	1.02643191	0.61140001	
chr10	44879020	44962222	83203	1	1	0	0.01091641	0	4	2	0.76005302	0.61140001	
chr10	44962222	45104413	142192	2	1	0	0.01091641	0	4	3	0.76005302	0.93173516	
chr10	45104413	45104472	60	1	1	0	0.01091641	0	4	4	0.76005302	1.26272838	
chr10	45104472	45349813	245342	2	1	0	0.05404976	0	2	3	0.30102999	0.93173516	
chr10	45349813	45679808	329996	9	0	0	0	0	2	3	0.61140001	0.93173516	
chr10	45679808	45872062	192255	4	0	0	0	0	2	2	0.61140001	0.61140001	
chr10	45872062	45981326	109265	3	0	0	0	0	1	2	0.30102999	0.61140001	
chr10	45981326	46158097	176772	5	1	0	0.1218695	0	1	2	0.1218695	0.61140001	
chr10	46158097	46158215	119	2	1	0	0.1218695	0	1	3	0.1218695	0.93173516	
chr10	47604818	47655146	50329	2	0	0	0	0	1	3	0.30102999	0.93173516	
chr10	47655146	48387727	732582	6	0	0	0	0	1	2	0.30102999	0.61140001	
chr10	48387727	48533609	145883	4	0	0	0	0	1	1	0.30102999	0.30102999	
chr10	48533609	49390457	856849	2	0	0	0	0	1	3	0.30102999	0.93173516	
chr10	49390457	49390516	60	1	1	0	0.1218695	0	1	6	0.1218695	1.95986592	
chr10	49390516	49514027	123512	2	0	0	0	0	1	4	0.30102999	1.26272838	
chr10	49514027	49741476	227450	8	1	0	0.1218695	0	1	4	0.1218695	1.26272838	
chr10	49741476	49781623	40148	2	1	1	0.1218695	0.01091641	1	4	0.1218695	0.76005302	
chr10	49781623	49900501	118879	3	1	1	0.1218695	0.00478973	1	5	0.1218695	1.02643191	
chr10	49900501	49944155	43655	2	2	1	0.30102999	0.00204627	1	6	0.05404976	1.31360226	
chr10	49944155	49944212	58	1	2	1	0.30102999	8.47E-04	1	7	0.05404976	1.62048027	
chr10	49944212	50057312	113101	2	2	1	0.30102999	0.00204627	1	6	0.05404976	1.31360226	
chr10	50057312	50358762	301451	7	3	1	0.17593012	0.00204627	3	6	0.17593012	1.31360226	

chr10	50358762	50580284	221523	3	2	1	0.30102999	0.00204627	1	6	0.05404976	1.31360226
chr10	50580284	50580343	60	1	2	1	0.30102999	8.47E-04	1	7	0.05404976	1.62048027
chr10	50580343	50668502	88160	1	1	1	0.1218695	0.00478973	1	5	0.1218695	1.02643191
chr10	50668502	50765972	97471	3	1	1	0.1218695	0.02438896	1	3	0.1218695	0.51676182
chr10	50765972	50766031	60	1	1	1	0.1218695	0.01091641	1	4	0.1218695	0.76005302
chr10	50766031	50849538	83508	1	1	0	0.1218695	0	1	4	0.1218695	1.26272838
chr10	50849538	50916848	67311	3	1	1	0.1218695	0.01091641	1	4	0.1218695	0.76005302
chr10	50916848	50916907	60	1	1	2	0.1218695	0.0429175	1	4	0.1218695	0.47744371
chr10	50916907	50968591	51685	2	1	2	0.1218695	0.08289318	1	3	0.1218695	0.30102999
chr10	50968591	50968645	55	1	1	2	0.1218695	0.0429175	1	4	0.1218695	0.47744371
chr10	50968645	51026371	57727	1	1	0	0.1218695	0	1	4	0.1218695	1.26272838
chr10	51026371	51549555	523185	2	1	0	0.1218695	0	1	3	0.1218695	0.93173516
chr10	51549555	51664079	114525	6	1	1	0.1218695	0.02438896	1	3	0.1218695	0.51676182
chr10	51664079	51804961	140883	1	1	0	0.1218695	0	1	3	0.1218695	0.93173516
chr10	51804961	51805020	60	1	2	0	0.1575501	0	2	4	0.1575501	1.26272838
chr10	51805020	52006170	201151	1	1	0	0.05404976	0	2	4	0.30102999	1.26272838
chr10	52006170	52036781	30612	2	1	0	0.02438896	0	3	4	0.51676182	1.26272838
chr10	52036781	52036840	60	1	1	1	0.01091641	0.01091641	4	4	0.76005302	0.76005302
chr10	52036840	52066864	30025	1	1	1	0.05404976	0.01091641	2	4	0.30102999	0.76005302
chr10	52066864	52108756	41893	1	1	0	0.05404976	0	2	3	0.30102999	0.93173516
chr10	52108756	52206675	97920	3	2	0	0.1575501	0	2	3	0.1575501	0.93173516
chr10	52206675	52279593	72919	2	2	0	0.08289318	0	3	4	0.30102999	1.26272838
chr10	52279593	52566413	286821	6	2	0	0.0429175	0	4	4	0.47744371	1.26272838
chr10	52566413	52777344	210932	4	2	0	0.08289318	0	3	4	0.30102999	1.26272838
chr10	52777344	52857542	80199	3	2	1	0.08289318	0.00478973	3	5	0.30102999	1.02643191
chr10	52857542	53032642	175101	4	2	0	0.08289318	0	3	5	0.30102999	1.60515106
chr10	53032642	53102430	69789	3	2	1	0.08289318	0.00478973	3	5	0.30102999	1.02643191
chr10	53102430	53227459	125030	2	2	0	0.08289318	0	3	5	0.30102999	1.60515106
chr10	53227459	53269263	41805	2	3	0	0.17593012	0	3	5	0.17593012	1.60515106
chr10	53269263	53361898	92636	2	3	0	0.17593012	0	3	4	0.17593012	1.26272838
chr10	53361898	53361957	60	1	3	0	0.17593012	0	3	5	0.17593012	1.60515106
chr10	53361957	53746679	384723	9	3	0	0.17593012	0	3	4	0.17593012	1.26272838
chr10	53746679	53807219	60541	1	3	0	0.17593012	0	3	3	0.17593012	0.93173516
chr10	53807219	53982674	175456	4	3	0	0.17593012	0	3	4	0.17593012	1.26272838
chr10	53982674	54213409	230736	5	3	0	0.17593012	0	3	3	0.17593012	0.93173516
chr10	54213409	54584632	371224	3	3	0	0.30102999	0	2	3	0.08289318	0.93173516
chr10	54584632	54676207	91576	1	3	0	0.51676182	0	1	1	0.02438896	0.30102999
chr10	54676207	54810783	134577	1	2	0	0.30102999	0	1	1	0.05404976	0.30102999
chr10	54810783	55186702	375920	3	2	0	0.30102999	0	1	3	0.05404976	0.93173516
chr10	55186702	55236904	50203	2	2	0	0.30102999	0	1	4	0.05404976	1.26272838
chr10	55236904	55236963	60	1	2	0	0.30102999	0	1	5	0.05404976	1.60515106
chr10	55236963	55355871	118909	1	2	0	0.30102999	0	1	4	0.05404976	1.26272838
chr10	55355871	55480375	124505	2	3	0	0.51676182	0	1	4	0.02438896	1.26272838
chr10	55480375	55516193	35819	1	3	0	0.51676182	0	1	2	0.02438896	0.61140001
chr10	55516193	55516252	60	1	4	0	0.76005302	0	1	2	0.01091641	0.61140001
chr10	55516252	55754373	238122	5	3	0	0.51676182	0	1	2	0.02438896	0.61140001
chr10	55754373	55803078	48706	1	3	0	0.51676182	0	1	1	0.02438896	0.30102999
chr10	55803078	55803137	60	1	3	0	0.51676182	0	1	2	0.02438896	0.61140001
chr10	55803137	55925472	122336	2	3	0	0.51676182	0	1	1	0.02438896	0.30102999
chr10	55925472	55985113	59642	2	3	0	0.51676182	0	1	2	0.02438896	0.61140001
chr10	55985113	56014996	29884	2	3	0	0.51676182	0	1	5	0.02438896	1.60515106
chr10	56014996	56131740	116745	3	3	0	0.51676182	0	1	3	0.02438896	0.93173516
chr10	56131740	56319908	188169	3	3	0	0.51676182	0	1	2	0.02438896	0.61140001
chr10	56319908	56369329	49422	3	3	0	0.30102999	0	2	5	0.08289318	1.60515106
chr10	56369329	56394602	25274	2	3	0	0.30102999	0	2	6	0.08289318	1.95986592
chr10	56394602	56486116	91515	2	3	0	0.30102999	0	2	5	0.08289318	1.60515106
chr10	56486116	56564594	78479	2	4	0	0.47744371	0	2	6	0.0429175	1.95986592
chr10	56564594	56676461	111868	1	3	0	0.30102999	0	2	4	0.08289318	1.26272838
chr10	56676461	56877260	200800	2	2	0	0.1575501	0	2	4	0.1575501	1.26272838
chr10	56877260	56877319	60	1	2	1	0.1575501	0.01091641	2	4	0.1575501	0.76005302
chr10	56877319	57038923	161605	2	2	0	0.1575501	0	2	1	0.1575501	0.30102999
chr10	57038923	57038982	60	1	2	0	0.1575501	0	2	2	0.1575501	0.61140001
chr10	57038982	57091487	52506	1	2	0	0.1575501	0	2	1	0.1575501	0.30102999
chr10	57091487	57303429	211943	3	1	0	0.05404976	0	2	1	0.30102999	0.30102999
chr10	57303429	57470290	166862	4	1	0	0.05404976	0	2	3	0.30102999	0.93173516
chr10	57470290	57723213	252924	1	1	0	0.1218695	0	1	3	0.1218695	0.93173516
chr10	57723213	57723272	60	1	1	0	0.1218695	0	1	4	0.1218695	1.26272838
chr10	57723272	57831487	108216	1	0	0	0	0	1	3	0.30102999	0.93173516
chr10	57831487	58121294	289808	2	0	0	0	0	1	2	0.30102999	0.61140001
chr10	58121294	58121349	56	1	0	0	0	0	1	3	0.30102999	0.93173516
chr10	58121349	58421494	300146	2	0	0	0	0	1	2	0.30102999	0.61140001
chr10	58421494	58686145	264652	1	0	0	0	0	1	1	0.30102999	0.30102999
chr10	58686145	59028244	342100	2	0	0	0	0	0	1	0	0.30102999
chr10	59028244	59209294	181051	2	0	0	0	0	1	1	0.30102999	0.30102999
chr10	59209294	59452586	243293	3	0	0	0	0	1	3	0.30102999	0.93173516
chr10	59452586	59582257	129672	2	0	0	0	0	1	5	0.30102999	1.60515106
chr10	59582257	59960546	378290	5	1	0	0.1218695	0	1	5	0.1218695	1.60515106
chr10	59960546	59974608	14063	1	1	0	0.1218695	0	1	4	0.1218695	1.26272838
chr10	59974608	60033417	58810	1	1	0	0.1218695	0	1	3	0.1218695	0.93173516
chr10	60033417	60123346	89930	3	2	0	0.30102999	0	1	4	0.05404976	1.26272838
chr10	60123346	60196887	73542	4	2	0	0.30102999	0	1	5	0.05404976	1.60515106
chr10	60196887	60417076	220190	4	2	0	0.30102999	0	1	4	0.05404976	1.26272838
chr10	60417076	60417135	60	1	2	0	0.30102999	0	1	5	0.05404976	1.60515106
chr10	60417135	60516571	99437	2	2	0	0.30102999	0	1	4	0.05404976	1.26272838
chr10	60516571	60516630	60	1	2	0	0.30102999	0	1	5	0.05404976	1.60515106
chr10	60516630	60574007	57378	2	2	0	0.30102999	0	1	4	0.05404976	1.26272838
chr10	60574007	60885982	311976	3	2	0	0.30102999	0	1	3	0.05404976	0.93173516
chr10	60885982	61006839	120858	5	3	0	0.30102999	0	2	3	0.08289318	0.93173516
chr10	61006839	61008352	1514	2	3	0	0.10122019	0	4	3	0.30102999	0.93173516
chr10	61008352	61062589	54238	1	3	0	0.30102999	0	2	3	0.08289318	0.93173516
chr10	61062589	61122141	59553	3	3	0	0.30102999	0	2	4	0.08289318	1.26272838
chr10	61122141	61122196	56	1	3	1	0.30102999	0.00204627	2	6	0.08289318	1.31360226
chr10	61122196	61188677	66482	1	3	0	0.30102999	0	2	6	0.08289318	1.95986592
chr10	61188677	61361946	173270	2	4	0	0.18734596	0	4	6	0.18734596	1.95986592
chr10	61361946	61410711	48766	2	4	1	0.18734596	8.47E-04	4	7	0.18734596	1.62048027
chr10	61410711	61551500	140790	3	4	0	0.18734596	0	4	7	0.18734596	2.3278427
chr10	61551500	61551559	60	1	4	1	0.18734596	8.47E-04	4	7	0.18734596	1.62048027
chr10	61551559	61714342	162784	4	4	0	0.18734596	0	4	7	0.18734596	2.3278427
chr10	61714342	61831928	117587	2	4	0	0.18734596	0	4	6	0.18734596	1.95986592
chr10	61831928	61831987	60	1	5	0	0.30102999	0	4	6	0.11390336	1.95986592
chr10	61831987	62205877	373891	8	4	0	0.18734596	0	4	6	0.18734596	1.95986592
chr10	62205877	62435823	229947	4	5	0	0.30102999	0	4	6	0.11390336	1.95986592
chr10	62435823	62539893	104071	2	4	0	0.18734596	0	4	6	0.18734596	1.95986592
chr10	62539893	62548013	8121	3	5	0	0.30102999	0	4	6	0.11390336	1.95986592
chr10	62548013	62918280	370268	6	4	0	0.18734596	0	4	6	0.18734596	1.95986592
chr10	62918280	64269235	1350956	23	3	0	0.10122019	0	4	5	0.30102999	1.60515106
chr10	64269235	64269294	60	1	3	1	0.10122019	0.00478973	4	5	0.30102999	1.02643191
chr10	64269294	64323297	54004	1	3	0	0.10122019	0	4	5	0.30102999	1.60515106
chr10	64323297	64481443	158147	4	3	0	0.17593012	0	3	5	0.17593012	1.60515106
chr10	64481443	64892035	410593	6	2	0	0.08289318	0	3	5	0.30102999	1.60515106
chr10	64892035	64967583	75549	5	2	0	0.08289318	0	3	6	0.30102999	1.95986592
chr10	64967583	64979679	12097	2	2	1	0.0429175	0.00204627	4	6	0.47744371	1.31360226
chr10	64979679	65024484	44806	1	2	1	0.08289318	0.00204627	3	6	0.30102999	1.31360226
chr10	65024484	65226304	201821	5	2	1	0.1575501	0.00204627	2	6	0.1575501	1.31360226
chr10	65226304	65347281	120978	3	2	1	0.30102999	0.00204627	1	6	0.05404976	1.31360226
chr10	65347281	65402430	55150	1	2	1	0.30102999	0.00478973	1	5	0.05404976	1.02643191
chr10	65402430	65528431	126002	1	1	1	0.1218695	0.00478973	1	5	0.1218695	1.02643191
chr10	65528431	65528490	60	1	1	1	0.1218695	0.00204627	1	6	0.1218695	1.31360226
chr10	65528490	65784589	256100	3	1	0	0.1218695	0	1	5	0.1218695	1.60515106
chr10	65784589	65871872	87284	2	1	1	0.1218695	0.00204627	1	6	0.1218695	1.31360226
chr10	65871872	65938133	66262	1	1	0	0.1218695	0	1	4	0.1218695	1.26272838
chr10	65938133	66265376	327244	2	0	0	0	0	1	2	0.30102999	0.61140001
chr10	66265376	66456772	191397	2	0	0	0	0	1	1	0.30102999	0.30102999
chr10	66456772	66985030	528259	5	0	0	0	0	1	3	0.30102999	0.93173516

chr10	66985030	66985089	60	1	0	0	0	0	1	4	0.30102999	1.26272838
chr10	66985089	67399303	414215	2	0	0	0	0	1	3	0.30102999	0.93173516
chr10	67399303	67454101	54799	2	1	0	0.1218695	0	1	3	0.1218695	0.93173516
chr10	67454101	67637905	183805	2	1	1	0.1218695	0.02438896	1	3	0.1218695	0.51676182
chr10	67637905	67637964	60	1	1	1	0.1218695	0.01091641	1	4	0.1218695	0.76005302
chr10	67637964	67758613	120650	2	1	0	0.1218695	0	1	3	0.1218695	0.93173516
chr10	67758613	67865256	106644	3	1	0	0.1218695	0	1	4	0.1218695	1.26272838
chr10	67865256	67907699	42444	2	1	0	0.05404976	0	2	4	0.30102999	1.26272838
chr10	67907699	68040325	132627	4	1	1	0.05404976	0.01091641	2	4	0.30102999	0.76005302
chr10	68040325	68040384	60	1	1	1	0.05404976	0.00204627	2	6	0.30102999	1.31360226
chr10	68040384	68383364	342981	8	1	1	0.05404976	0.00478973	2	5	0.30102999	1.02643191
chr10	68383364	68443835	60472	2	1	1	0.05404976	0.00204627	2	6	0.30102999	1.31360226
chr10	68443835	68599432	155598	4	1	1	0.02438896	0.00204627	3	6	0.51676182	1.31360226
chr10	68599432	68686933	87502	3	1	1	0.02438896	8.47E-04	3	7	0.51676182	1.62048027
chr10	68686933	68687691	759	2	1	1	0.01091641	8.47E-04	4	7	0.76005302	1.62048027
chr10	68687691	68779472	91782	2	1	1	0.01091641	0.00204627	4	6	0.76005302	1.31360226
chr10	68779472	68779531	60	1	1	1	0.01091641	3.39E-04	4	8	0.76005302	1.94674965
chr10	68779531	68843366	63836	1	1	1	0.01091641	8.47E-04	4	7	0.76005302	1.62048027
chr10	68843366	69064338	220973	6	1	1	0.00478973	8.47E-04	5	7	1.02643191	1.62048027
chr10	69064338	69281681	217344	4	1	1	0.00478973	0.00204627	5	6	1.02643191	1.31360226
chr10	69281681	69281740	60	1	1	2	0.00478973	0.00493743	5	7	1.02643191	1.16581773
chr10	69281740	69408480	126741	2	1	2	0.00478973	0.01053319	5	6	1.02643191	0.91219088
chr10	69408480	69408539	60	1	1	2	0.00478973	0.00493743	5	7	1.02643191	1.16581773
chr10	69408539	69647176	238638	5	1	2	0.00478973	0.01053319	5	6	1.02643191	0.91219088
chr10	69647176	69748511	101336	5	2	2	0.02162467	0.01053319	5	6	0.68214471	0.91219088
chr10	69748511	69832863	84353	2	2	2	0.02162467	0.0429175	5	4	0.68214471	0.47744371
chr10	69832863	69881541	48679	3	2	2	0.02162467	0.02162467	5	5	0.68214471	0.68214471
chr10	69881541	69921538	39998	1	2	2	0.0429175	0.02162467	4	5	0.47744371	0.68214471
chr10	69921538	69991540	70003	1	2	2	0.0429175	0.0429175	4	4	0.47744371	0.47744371
chr10	69991540	69991596	57	1	2	2	0.02162467	0.0429175	5	4	0.68214471	0.47744371
chr10	69991596	70094900	103305	2	2	2	0.0429175	0.0429175	4	4	0.47744371	0.47744371
chr10	70094900	70100962	6063	2	3	2	0.10122019	0.0429175	4	4	0.30102999	0.47744371
chr10	70100962	70229949	128988	3	2	2	0.0429175	0.0429175	4	4	0.47744371	0.47744371
chr10	70229949	70230008	60	1	3	2	0.03070643	0.0429175	6	4	0.63695542	0.47744371
chr10	70230008	70286588	56581	2	2	2	0.02162467	0.0429175	5	4	0.68214471	0.47744371
chr10	70286588	70322043	35456	2	3	3	0.05670724	0.10122019	5	4	0.45545077	0.30102999
chr10	70322043	70362973	40931	1	3	3	0.10122019	0.10122019	4	4	0.30102999	0.30102999
chr10	70362973	70453499	90527	2	3	2	0.10122019	0.0429175	4	4	0.30102999	0.47744371
chr10	70453499	70522116	68618	3	3	3	0.10122019	0.10122019	4	4	0.30102999	0.30102999
chr10	70522116	70522175	60	1	3	3	0.05670724	0.10122019	5	4	0.45545077	0.30102999
chr10	70522175	70554458	32284	1	3	2	0.10122019	0.08289318	4	3	0.30102999	0.30102999
chr10	70554458	70618242	63785	1	2	2	0.0429175	0.08289318	4	3	0.47744371	0.30102999
chr10	70618242	70764899	146658	9	2	2	0.0429175	0.0429175	4	4	0.47744371	0.47744371
chr10	70764899	70848046	83148	2	2	2	0.0429175	0.08289318	4	3	0.47744371	0.30102999
chr10	70848046	70900599	52554	3	2	2	0.02162467	0.0429175	5	4	0.68214471	0.47744371
chr10	70900599	70900658	60	1	2	2	0.01053319	0.0429175	6	4	0.91219088	0.47744371
chr10	70900658	71218296	317639	7	2	2	0.08289318	0.0429175	3	4	0.30102999	0.47744371
chr10	71218296	71251810	33515	1	2	2	0.08289318	0.08289318	3	3	0.30102999	0.30102999
chr10	71251810	71251869	60	1	2	2	0.0429175	0.08289318	4	3	0.47744371	0.30102999
chr10	71251869	71333003	81135	1	2	2	0.0429175	0.1575501	4	2	0.47744371	0.1575501
chr10	71333003	71516435	183433	2	2	2	0.08289318	0.1575501	3	2	0.30102999	0.1575501
chr10	71516435	71563087	46653	2	2	2	0.0429175	0.08289318	4	3	0.47744371	0.30102999
chr10	71563087	71588406	25320	2	3	2	0.10122019	0.0429175	4	4	0.30102999	0.47744371
chr10	71588406	71647182	58777	1	2	2	0.0429175	0.0429175	4	4	0.47744371	0.47744371
chr10	71647182	71846010	198829	6	3	2	0.10122019	0.0429175	4	4	0.30102999	0.47744371
chr10	71846010	71898932	52923	2	3	2	0.17593012	0.0429175	3	4	0.17593012	0.47744371
chr10	71898932	71898991	60	1	3	2	0.10122019	0.0429175	4	4	0.30102999	0.47744371
chr10	71898991	71983416	84426	3	3	2	0.10122019	0.08289318	4	3	0.30102999	0.30102999
chr10	71983416	72072838	89423	2	3	2	0.10122019	0.1575501	4	2	0.30102999	0.1575501
chr10	72072838	72072897	60	1	3	2	0.05670724	0.08289318	5	3	0.45545077	0.30102999
chr10	72072897	72138939	66043	1	3	2	0.05670724	0.1575501	5	2	0.45545077	0.1575501
chr10	72138939	72169741	30803	1	2	2	0.08289318	0.1575501	3	2	0.30102999	0.1575501
chr10	72169741	72198662	28922	2	2	2	0.08289318	0.08289318	3	3	0.30102999	0.30102999
chr10	72198662	72282361	83700	2	2	2	0.08289318	0.1575501	3	2	0.30102999	0.1575501
chr10	72282361	72327855	45495	2	3	2	0.10122019	0.1575501	4	2	0.30102999	0.1575501
chr10	72327855	72327914	60	1	4	2	0.11390336	0.1575501	5	2	0.30102999	0.1575501
chr10	72327914	72359372	31459	1	3	2	0.10122019	0.1575501	4	2	0.30102999	0.1575501
chr10	72359372	72359916	545	2	3	2	0.10122019	0.08289318	4	3	0.30102999	0.30102999
chr10	72359916	72604311	244396	4	3	2	0.10122019	0.1575501	4	2	0.30102999	0.1575501
chr10	72604311	72646994	42684	4	3	2	0.17593012	0.1575501	3	2	0.17593012	0.1575501
chr10	72646994	72681585	34592	1	3	2	0.30102999	0.1575501	2	2	0.08289318	0.1575501
chr10	72681585	72681644	60	1	3	2	0.17593012	0.1575501	3	2	0.17593012	0.1575501
chr10	72681644	72929328	247685	1	2	1	0.08289318	0.05404976	3	2	0.30102999	0.30102999
chr10	72929328	72929387	60	1	2	1	0.02162467	0.02438896	5	3	0.68214471	0.51676182
chr10	72929387	73028865	99479	1	2	1	0.0429175	0.02438896	4	3	0.47744371	0.51676182
chr10	73028865	73120782	91918	3	3	2	0.10122019	0.08289318	4	3	0.30102999	0.30102999
chr10	73120782	73122419	1638	2	3	2	0.10122019	0.0429175	4	4	0.30102999	0.47744371
chr10	73122419	73148577	26159	1	2	2	0.0429175	0.0429175	4	4	0.47744371	0.47744371
chr10	73148577	73148636	60	1	2	2	0.0429175	0.02162467	4	5	0.47744371	0.68214471
chr10	73148636	73247632	98997	2	2	2	0.08289318	0.0429175	3	4	0.30102999	0.47744371
chr10	73247632	73315953	68322	2	2	2	0.08289318	0.02162467	3	5	0.30102999	0.68214471
chr10	73315953	73406500	90548	2	2	2	0.1575501	0.08289318	2	3	0.1575501	0.30102999
chr10	73406500	73449787	43288	2	2	2	0.08289318	0.08289318	3	3	0.30102999	0.30102999
chr10	73449787	73449846	60	1	2	2	0.0429175	0.0429175	4	4	0.47744371	0.47744371
chr10	73449846	73514717	64872	1	2	2	0.0429175	0.08289318	4	3	0.47744371	0.30102999
chr10	73514717	73514776	60	1	2	2	0.0429175	0.0429175	4	4	0.47744371	0.47744371
chr10	73514776	73607502	92727	2	2	2	0.0429175	0.1575501	4	2	0.47744371	0.1575501
chr10	73607502	73773238	165737	3	2	2	0.08289318	0.1575501	3	2	0.30102999	0.1575501
chr10	73773238	73773297	60	1	2	3	0.08289318	0.30102999	3	2	0.30102999	0.08289318
chr10	73773297	73887835	114539	5	2	2	0.08289318	0.1575501	3	2	0.30102999	0.1575501
chr10	73887835	73975496	87662	3	2	3	0.08289318	0.17593012	3	3	0.30102999	0.17593012
chr10	73975496	74139274	163779	6	2	4	0.08289318	0.11390336	3	5	0.30102999	0.30102999
chr10	74139274	74250019	110746	2	2	3	0.08289318	0.05670724	3	5	0.30102999	0.45545077
chr10	74250019	74542787	292769	9	2	4	0.08289318	0.11390336	3	5	0.30102999	0.30102999
chr10	74542787	74542846	60	1	2	5	0.08289318	0.19510895	3	5	0.30102999	0.19510895
chr10	74542846	74620556	77711	2	2	4	0.08289318	0.11390336	3	5	0.30102999	0.30102999
chr10	74620556	74765001	144446	5	2	5	0.08289318	0.19510895	3	5	0.30102999	0.19510895
chr10	74765001	74804840	39840	2	2	5	0.0429175	0.19510895	4	5	0.47744371	0.19510895
chr10	74804840	74874146	69307	2	2	5	0.02162467	0.19510895	5	5	0.68214471	0.19510895
chr10	74874146	74971246	97101	4	2	5	0.01053319	0.19510895	6	5	0.91219088	0.19510895
chr10	74971246	74971305	60	1	2	5	0.00493743	0.19510895	7	5	1.16581773	0.19510895
chr10	74971305	75051097	79793	5	2	4	0.01053319	0.11390336	6	5	0.91219088	0.30102999
chr10	75051097	75104466	53370	1	2	4	0.02162467	0.11390336	5	5	0.68214471	0.30102999
chr10	75104466	75139618	35153	1	2	4	0.02162467	0.18734596	5	4	0.68214471	0.18734596
chr10	75139618	75166073	26456	2	2	4	0.00493743	0.18734596	7	4	1.16581773	0.18734596
chr10	75166073	75503890	337818	11	2	3	0.00493743	0.10122019	7	4	1.16581773	0.30102999
chr10	75503890	75503949	60	1	2	3	0.00493743	0.03070643	7	6	1.16581773	0.63695542
chr10	75503949	75533929	29981	3	2	3	0.00493743	0.05670724	7	5	1.16581773	0.45545077
chr10	75533929	75674642	140714	5	2	2	0.01053319	0.02162467	6	5	0.91219088	0.68214471
chr10	75674642	75802822	128181	3	2	2	0.01053319	0.01053319	6	6	0.91219088	0.91219088
chr10	75802822	75960610	157789	4	2	3	0.01053319	0.03070643	6	6	0.91219088	0.63695542
chr10	75960610	76074439	113830	2	2	3	0.02162467	0.10122019	5	4	0.68214471	0.30102999
chr10	76074439	76158270	83832	3	2	3	0.01053319	0.05670724	6	5	0.91219088	0.45545077
chr10	76158270	76193162	34893	2	2	4	0.01053319	0.06713722	6	6	0.91219088	0.44141547
chr10	76193162	76227760	34599	1	2	4	0.01053319	0.11390336	6	5	0.91219088	0.30102999
chr10	76227760	76285147	57388	1	2	3	0.01053319	0.05670724	6	5	0.91219088	0.45545077
chr10	76285147	76337573	52427	1	2	3	0.02162467	0.05670724	5	5	0.68214471	0.45545077
chr10	76337573	76337632	60	1	2	4	0.02162467	0.06713722	5	6	0.68214471	0.44141547
chr10	76337632	76468932	131301	2	2	3	0.02162467	0.10122019	5	4	0.68214471	0.30102999
chr10	76468932	76468991	60	1	2	4	0.02162467	0.06713722	5	6	0.68214471	0.44141547
chr10	76468991	76524989	55999	1	2	4	0.02162467	0.11390336	5	5	0.68214471	0.30102999
chr10	76524989	76602646	77658	1	2	3	0.02162467	0.10122019	5	4	0.68214471	0.30102999
chr10	76602646	76694484	91839	2	2	3	0.02162467	0.30102999	5	2	0.68214471	0.08289318
chr10	76694484	76788268	93785	3	2	3	0.02162467	0.17593012	5	3	0.68214471	0.17593012

chr10	76788268	76864463	76196	3	2	2	0.02162467	0.1575501	5	2	0.68214471	0.1575501
chr10	76864463	76864515	53	1	2	3	0.02162467	0.30102999	5	2	0.68214471	0.08289318
chr10	76864515	76936246	71732	2	2	3	0.0429175	0.30102999	4	2	0.47744371	0.08289318
chr10	76936246	76977436	41191	2	2	3	0.08289318	0.30102999	3	2	0.30102999	0.08289318
chr10	76977436	77272227	294792	7	2	3	0.0429175	0.30102999	4	2	0.47744371	0.08289318
chr10	77272227	77272286	60	1	2	3	0.0429175	0.17593012	4	3	0.47744371	0.17593012
chr10	77272286	77483984	211699	5	2	3	0.0429175	0.30102999	4	2	0.47744371	0.08289318
chr10	77483984	77484043	60	1	2	4	0.0429175	0.47744371	4	2	0.47744371	0.0429175
chr10	77484043	77889960	405918	8	2	3	0.0429175	0.30102999	4	2	0.47744371	0.08289318
chr10	77889960	77890019	60	1	3	3	0.10122019	0.30102999	4	2	0.30102999	0.08289318
chr10	77890019	78015106	125088	2	3	3	0.10122019	0.51676182	4	1	0.30102999	0.02438896
chr10	78015106	78146147	131042	3	3	3	0.05670724	0.51676182	5	1	0.45545077	0.02438896
chr10	78146147	78224874	78728	2	3	2	0.05670724	0.30102999	5	1	0.45545077	0.05404976
chr10	78224874	78292060	67187	2	3	3	0.05670724	0.51676182	5	1	0.45545077	0.02438896
chr10	78292060	78517951	225892	4	3	3	0.05670724	0.17593012	5	3	0.45545077	0.17593012
chr10	78517951	78518010	60	1	3	4	0.05670724	0.30102999	5	3	0.45545077	0.10122019
chr10	78518010	78644080	126071	2	2	3	0.02162467	0.17593012	5	3	0.68214471	0.17593012
chr10	78644080	78644139	60	1	3	4	0.03070643	0.30102999	6	3	0.63695542	0.10122019
chr10	78644139	78687209	43071	1	2	4	0.01053319	0.30102999	6	3	0.91219088	0.10122019
chr10	78687209	78839253	152045	3	2	3	0.01053319	0.17593012	6	3	0.91219088	0.17593012
chr10	78839253	78839312	60	1	2	4	0.01053319	0.30102999	6	3	0.91219088	0.10122019
chr10	78839312	79047382	208071	5	2	3	0.01053319	0.17593012	6	3	0.91219088	0.17593012
chr10	79047382	79117234	69853	1	2	3	0.02162467	0.17593012	5	3	0.68214471	0.17593012
chr10	79117234	79158563	41330	2	2	4	0.02162467	0.30102999	5	3	0.68214471	0.10122019
chr10	79158563	79212583	54021	2	2	4	0.01053319	0.30102999	6	3	0.91219088	0.10122019
chr10	79212583	79384197	171615	4	2	3	0.02162467	0.17593012	5	3	0.68214471	0.17593012
chr10	79384197	79625278	241082	6	2	3	0.02162467	0.10122019	5	4	0.68214471	0.30102999
chr10	79625278	79661733	36456	1	2	2	0.02162467	0.0429175	5	4	0.68214471	0.47744371
chr10	79661733	79764590	102858	2	2	1	0.02162467	0.01091641	5	4	0.68214471	0.76005302
chr10	79764590	79799851	35262	1	2	1	0.02162467	0.02438896	5	3	0.68214471	0.51676182
chr10	79799851	79799910	60	1	2	2	0.02162467	0.08289318	5	3	0.68214471	0.30102999
chr10	79799910	79891132	91223	1	2	1	0.0429175	0.02438896	4	3	0.47744371	0.51676182
chr10	79891132	80048946	157815	1	1	1	0.01091641	0.02438896	4	3	0.76005302	0.51676182
chr10	80048946	80049005	60	1	1	3	0.00478973	0.05670724	5	5	1.02643191	0.45545077
chr10	80049005	80103108	54104	1	1	1	0.00478973	0.02438896	5	3	1.02643191	0.51676182
chr10	80103108	80171813	68706	2	2	1	0.02162467	0.02438896	5	3	0.68214471	0.51676182
chr10	80171813	80171872	60	1	2	2	0.02162467	0.02162467	5	5	0.68214471	0.68214471
chr10	80171872	80270710	98839	1	1	2	0.00478973	0.0429175	5	4	1.02643191	0.47744371
chr10	80270710	80381611	110902	2	2	2	0.02162467	0.0429175	5	4	0.68214471	0.47744371
chr10	80381611	80597690	216080	2	2	0	0.02162467	0	5	3	0.68214471	0.93173516
chr10	80597690	80597749	60	1	2	1	0.02162467	0.01091641	5	4	0.68214471	0.76005302
chr10	80597749	80779547	181799	2	1	0	0.01091641	0	4	3	0.76005302	0.93173516
chr10	80779547	80779606	60	1	1	1	0.01091641	0.00478973	4	5	0.76005302	1.02643191
chr10	80779606	80846709	67104	1	1	0	0.01091641	0	4	4	0.76005302	1.26272838
chr10	80846709	81051168	204460	4	1	0	0.01091641	0	4	3	0.76005302	0.93173516
chr10	81051168	81154741	103574	4	2	1	0.02162467	0.02438896	5	3	0.68214471	0.51676182
chr10	81154741	81154800	60	1	2	1	0.02162467	0.01091641	5	4	0.68214471	0.76005302
chr10	81154800	81697501	542702	2	2	1	0.02162467	0.1218695	5	1	0.68214471	0.1218695
chr10	81697501	81702930	5430	2	3	1	0.03070643	0.1218695	6	1	0.63695542	0.1218695
chr10	81702930	81873133	170204	5	3	1	0.03070643	0.05404976	6	2	0.63695542	0.30102999
chr10	81873133	81958740	85608	3	3	1	0.01598258	0.05404976	7	2	0.84395715	0.30102999
chr10	81958740	82109919	151180	2	3	1	0.10122019	0.1218695	4	1	0.30102999	0.1218695
chr10	82109919	82109978	60	1	3	1	0.05670724	0.1218695	5	1	0.45545077	0.1218695
chr10	82109978	82718756	608779	11	2	1	0.0429175	0.1218695	4	1	0.47744371	0.1218695
chr10	82718756	82860481	141726	2	2	1	0.02162467	0.1218695	5	1	0.68214471	0.1218695
chr10	82860481	82860540	60	1	2	2	0.02162467	0.30102999	5	1	0.68214471	0.05404976
chr10	82860540	82947948	87409	1	2	2	0.0429175	0.30102999	4	1	0.47744371	0.05404976
chr10	82947948	83278097	330150	2	2	1	0.0429175	0.1218695	4	1	0.47744371	0.1218695
chr10	83278097	83485997	207901	1	2	1	0.08289318	0.1218695	3	1	0.30102999	0.1218695
chr10	83485997	83662567	176571	2	2	1	0.08289318	0.05404976	3	2	0.30102999	0.30102999
chr10	83662567	83662626	60	1	2	2	0.08289318	0.08289318	3	3	0.30102999	0.30102999
chr10	83662626	83719119	56494	1	2	1	0.08289318	0.02438896	3	3	0.30102999	0.51676182
chr10	83719119	83799128	80010	3	2	1	0.08289318	0.01091641	3	4	0.30102999	0.76005302
chr10	83799128	84098620	299493	5	2	1	0.1575501	0.01091641	2	4	0.1575501	0.76005302
chr10	84098620	84098679	60	1	2	2	0.02162467	0.0429175	5	4	0.68214471	0.47744371
chr10	84098679	84179145	80467	2	2	1	0.0429175	0.01091641	4	4	0.47744371	0.76005302
chr10	84179145	84232776	53632	1	2	1	0.0429175	0.02438896	4	3	0.47744371	0.51676182
chr10	84232776	84232835	60	1	2	1	0.02162467	0.02438896	5	3	0.68214471	0.51676182
chr10	84232835	84561595	328761	5	2	1	0.0429175	0.02438896	4	3	0.47744371	0.51676182
chr10	84561595	84696018	134424	3	2	1	0.0429175	0.01091641	4	4	0.47744371	0.76005302
chr10	84696018	84783427	87410	1	2	1	0.08289318	0.02438896	3	3	0.30102999	0.51676182
chr10	84783427	84835236	51810	1	1	1	0.1218695	0.02438896	1	3	0.1218695	0.51676182
chr10	84835236	84973951	138716	1	1	1	0.1218695	0.05404976	1	2	0.1218695	0.30102999
chr10	84973951	84974010	60	1	1	1	0.05404976	0.01091641	2	4	0.30102999	0.76005302
chr10	84974010	85091232	117223	1	1	1	0.1218695	0.01091641	1	4	0.1218695	0.76005302
chr10	85091232	85091291	60	1	1	2	0.02438896	0.02162467	3	5	0.51676182	0.68214471
chr10	85091291	85283970	192680	2	1	2	0.30102999	0.0429175	0	4	0	0.47744371
chr10	85283970	85284029	60	1	2	2	0.08289318	0.02162467	3	5	0.30102999	0.68214471
chr10	85284029	85484389	200361	1	1	1	0.05404976	0.02438896	2	3	0.30102999	0.51676182
chr10	85484389	85531316	46928	2	1	1	0.05404976	0.01091641	2	4	0.30102999	0.76005302
chr10	85531316	85806428	275113	2	1	1	0.05404976	0.02438896	2	3	0.30102999	0.51676182
chr10	85806428	85806487	60	1	1	1	0.02438896	0.01091641	3	4	0.51676182	0.76005302
chr10	85806487	86134794	328308	10	1	1	0.1218695	0.01091641	1	4	0.1218695	0.76005302
chr10	86134794	86276904	142111	4	1	1	0.30102999	0.01091641	0	4	0	0.76005302
chr10	86276904	86276963	60	1	1	1	0.1218695	0.01091641	1	4	0.1218695	0.76005302
chr10	86276963	86461676	184714	2	1	1	0.30102999	0.01091641	0	4	0	0.76005302
chr10	86461676	86710999	249324	2	1	1	0.30102999	0.00478973	0	5	0	1.02643191
chr10	86710999	86863692	152694	2	1	2	0.1218695	0.01053319	1	6	0.1218695	0.91219088
chr10	86863692	86863751	60	1	1	3	0.05404976	0.01598258	2	7	0.30102999	0.84395715
chr10	86863751	87012456	148706	1	1	3	0.1218695	0.03070643	1	6	0.1218695	0.63695542
chr10	87012456	87012515	60	1	1	3	0.1218695	0.01598258	1	7	0.1218695	0.84395715
chr10	87012515	87192947	180433	1	1	3	0.1218695	0.05670724	1	5	0.1218695	0.45545077
chr10	87192947	87193006	60	1	2	3	0.1575501	0.03070643	2	6	0.1575501	0.63695542
chr10	87193006	87269033	76028	1	2	3	0.1575501	0.05670724	2	5	0.1575501	0.45545077
chr10	87269033	87341577	72545	1	2	2	0.1575501	0.02162467	2	5	0.1575501	0.68214471
chr10	87341577	87376808	35232	2	2	2	0.1575501	0.01053319	2	6	0.1575501	0.91219088
chr10	87376808	87450570	73763	1	2	2	0.30102999	0.02162467	1	5	0.05404976	0.68214471
chr10	87450570	87491139	40570	2	2	2	0.30102999	0.01053319	1	6	0.05404976	0.91219088
chr10	87491139	87537400	46262	2	2	2	0.30102999	0.00221948	1	8	0.05404976	1.44210395
chr10	87537400	87537459	60	1	2	3	0.30102999	0.0079614	1	8	0.05404976	1.07548421
chr10	87537459	87615258	77800	2	2	3	0.30102999	0.01598258	1	7	0.05404976	0.84395715
chr10	87615258	87697774	82517	3	2	3	0.30102999	0.0079614	1	8	0.05404976	1.07548421
chr10	87697774	88426177	728404	16	2	2	0.30102999	0.00221948	1	8	0.05404976	1.44210395
chr10	88426177	88461966	35790	2	3	2	0.51676182	0.00221948	1	8	0.02438896	1.44210395
chr10	88461966	88588266	126301	2	3	2	0.51676182	0.00493743	1	7	0.02438896	1.16581773
chr10	88588266	88628677	40412	2	3	3	0.51676182	0.01598258	1	7	0.02438896	0.84395715
chr10	88628677	88628736	60	1	3	3	0.51676182	0.0079614	1	8	0.02438896	1.07548421
chr10	88628736	88816618	187883	4	3	3	0.93173516	0.0079614	0	8	0	1.07548421
chr10	88816618	88816677	60	1	3	3	0.51676182	0.0079614	1	8	0.02438896	1.07548421
chr10	88816677	89263612	446936	3	3	2	0.51676182	0.00221948	1	8	0.02438896	1.44210395
chr10	89263612	89312414	48803	3	3	4	0.30102999	0.02074938	2	8	0.08289318	0.79906872
chr10	89312414	89422185	109772	1	3	3	0.30102999	0.0079614	2	8	0.08289318	1.07548421
chr10	89422185	89474512	52328	3	3	4	0.30102999	0.02074938	2	8	0.08289318	0.79906872
chr10	89474512	89521012	46501	3	3	6	0.30102999	0.08122616	2	8	0.08289318	0.4250187
chr10	89521012	89550212	29201	2	3	7	0.30102999	0.13499366	2	8	0.08289318	0.30102999
chr10	89550212	89604710	54499	1	3	5	0.30102999	0.04407651	2	8	0.08289318	0.58747015
chr10	89604710	89698253	93544	3	3	6	0.30102999	0.08122616	2	8	0.08289318	0.4250187
chr10	89698253	89851332	153080	4	3	7	0.30102999	0.13499366	2	8	0.08289318	0.30102999
chr10	89851332	90044771	193440	3	3	7	0.51676182	0.13499366	1	8	0.02438896	0.30102999
chr10	90044771	90488964	444194	11	3	7	0.17593012	0.13499366	3	8	0.17593012	0.30102999
chr10	90488964	90560938	71975	2	3	6	0.30102999	0.08122616	2	8	0.08289318	0.4250187
chr10	90560938	90604663	43726	2	3	6	0.17593012	0.08122616	3	8	0.17593012	0.4250187
chr10	90604663	90668512	63850	2	3	5	0.30102999	0.07511598	2	7	0.08289318	0.43181735
chr10	90668512	90708111	39600	3	3	5	0.30102999	0.04407651	2	8	0.08289318	0.58747015

chr10	90708111	90775321	67211	3	3	4	0.30102999	0.02074938	2	8	0.08289318	0.79906872
chr10	90775321	90965786	190466	2	3	4	0.51676182	0.02074938	1	8	0.02438896	0.79906872
chr10	90965786	91003286	37501	2	3	5	0.51676182	0.04407651	1	8	0.02438896	0.58747015
chr10	91003286	91483662	480377	14	3	4	0.51676182	0.02074938	1	8	0.02438896	0.79906872
chr10	91483662	91534283	50622	2	3	4	0.93173516	0.02074938	0	8	0	0.79906872
chr10	91534283	91730726	196444	2	3	4	0.93173516	0.11390336	0	5	0	0.30102999
chr10	91730726	92095432	364707	3	3	3	0.93173516	0.05670724	0	5	0	0.45545077
chr10	92095432	92095491	60	1	3	4	0.93173516	0.06713722	0	6	0	0.44141547
chr10	92095491	92191579	96089	1	3	3	0.93173516	0.03070643	0	6	0	0.63695542
chr10	92191579	92191638	60	1	3	3	0.93173516	0.01598258	0	7	0	0.84395715
chr10	92191638	92264113	72476	1	3	2	0.93173516	0.01053319	0	6	0	0.91219088
chr10	92264113	92363552	99440	1	3	2	0.93173516	0.02162467	0	5	0	0.68214471
chr10	92363552	92363611	60	1	3	3	0.93173516	0.05670724	0	5	0	0.45545077
chr10	92363611	92480156	116546	1	2	3	0.61140001	0.05670724	0	5	0	0.45545077
chr10	92480156	92532862	52707	2	2	3	0.61140001	0.03070643	0	6	0	0.63695542
chr10	92532862	92532921	60	1	2	4	0.61140001	0.06713722	0	6	0	0.44141547
chr10	92532921	92594832	61912	1	2	3	0.61140001	0.05670724	0	5	0	0.45545077
chr10	92594832	92655612	60781	2	2	3	0.61140001	0.03070643	0	6	0	0.63695542
chr10	92655612	92680452	24841	2	2	5	0.61140001	0.12309572	0	6	0	0.30102999
chr10	92680452	92823852	143401	3	2	5	0.61140001	0.07511598	0	7	0	0.43181735
chr10	92823852	92823911	60	1	3	5	0.93173516	0.07511598	0	7	0	0.43181735
chr10	92823911	92950376	126466	2	2	5	0.61140001	0.07511598	0	7	0	0.43181735
chr10	92950376	93008446	58071	4	2	5	0.61140001	0.04407651	0	8	0	0.58747015
chr10	93008446	93041536	33091	2	3	6	0.51676182	0.08122616	1	8	0.02438896	0.4250187
chr10	93041536	93126781	85246	1	2	6	0.61140001	0.08122616	0	8	0	0.4250187
chr10	93126781	93126840	60	1	2	6	0.61140001	0.02793176	0	10	0	0.74627054
chr10	93126840	93204666	77827	1	1	6	0.30102999	0.02793176	0	10	0	0.74627054
chr10	93204666	93272363	67698	2	1	6	0.30102999	0.08122616	0	8	0	0.4250187
chr10	93272363	93306009	33647	2	2	6	0.61140001	0.08122616	0	8	0	0.4250187
chr10	93306009	93306068	60	1	3	6	0.93173516	0.04875589	0	9	0	0.5732208
chr10	93306068	93390021	83954	1	3	6	0.93173516	0.08122616	0	8	0	0.4250187
chr10	93390021	93517827	127807	4	3	6	0.51676182	0.08122616	1	8	0.02438896	0.4250187
chr10	93517827	93590753	72927	1	3	6	0.93173516	0.129913	0	7	0	0.30102999
chr10	93590753	93667685	76933	2	3	5	0.93173516	0.07511598	0	7	0	0.43181735
chr10	93667685	93667744	60	1	3	5	0.93173516	0.04407651	0	8	0	0.58747015
chr10	93667744	93748941	81198	2	3	5	0.93173516	0.12309572	0	6	0	0.30102999
chr10	93748941	93749000	60	1	3	5	0.93173516	0.07511598	0	7	0	0.43181735
chr10	93749000	93789925	40926	1	3	5	0.93173516	0.12309572	0	6	0	0.30102999
chr10	93789925	93854943	65019	1	2	5	0.61140001	0.12309572	0	6	0	0.30102999
chr10	93854943	93904872	49930	2	2	5	0.1575501	0.04407651	2	8	0.1575501	0.58747015
chr10	93904872	93952270	47399	1	2	4	0.1575501	0.06713722	2	6	0.1575501	0.44141547
chr10	93952270	94002962	50693	1	2	4	0.61140001	0.11390336	0	5	0	0.30102999
chr10	94002962	94056824	53863	2	2	4	0.61140001	0.06713722	0	6	0	0.44141547
chr10	94056824	94056883	60	1	2	4	0.61140001	0.02074938	0	8	0	0.79906872
chr10	94056883	94138456	81574	2	1	4	0.30102999	0.02074938	0	8	0	0.79906872
chr10	94138456	94214092	75637	1	1	4	0.30102999	0.03812622	0	7	0	0.60763643
chr10	94214092	94269846	55755	2	2	4	0.30102999	0.03812622	1	7	0.05404976	0.60763643
chr10	94269846	94348354	78509	2	2	4	0.30102999	0.06713722	1	6	0.05404976	0.44141547
chr10	94348354	94393412	45059	1	0	4	0	0.06713722	0	6	0	0.44141547
chr10	94393412	94610553	217142	4	0	4	0	0.01077081	0	9	0	1.01542894
chr10	94610553	94669263	58711	2	0	5	0	0.02473314	0	9	0	0.76806864
chr10	94669263	94669322	60	1	1	5	0.30102999	0.01320236	0	10	0	0.97390707
chr10	94669322	94759139	89818	2	0	5	0	0.01320236	0	10	0	0.97390707
chr10	94759139	95126196	367058	11	1	5	0.30102999	0.01320236	0	10	0	0.97390707
chr10	95126196	95126255	60	1	1	5	0.1218695	0.01320236	1	10	0.1218695	0.97390707
chr10	95126255	95185893	59639	2	1	5	0.1218695	0.04407651	1	8	0.1218695	0.58747015
chr10	95185893	95216629	30737	2	1	5	0.1218695	0.02473314	1	9	0.1218695	0.76806864
chr10	95216629	95287995	71367	2	1	5	0.1218695	0.01320236	1	10	0.1218695	0.97390707
chr10	95287995	95335973	47979	2	2	5	0.30102999	0.01320236	1	10	0.05404976	0.97390707
chr10	95335973	95380478	44506	2	1	5	0.30102999	0.01320236	0	10	0	0.97390707
chr10	95380478	95380537	60	1	1	5	0.1218695	0.01320236	1	10	0.1218695	0.97390707
chr10	95380537	95490522	109986	3	1	5	0.30102999	0.01320236	0	10	0	0.97390707
chr10	95490522	95490581	60	1	1	6	0.30102999	0.02793176	0	10	0	0.74627054
chr10	95490581	95518049	27469	1	1	4	0.30102999	0.00530919	0	10	0	1.2568129
chr10	95518049	95557430	39382	2	1	4	0.1218695	0.00530919	1	10	0.1218695	1.2568129
chr10	95557430	95557489	60	1	1	5	0.1218695	0.01320236	1	10	0.1218695	0.97390707
chr10	95557489	95667849	110361	3	1	4	0.30102999	0.00530919	0	10	0	1.2568129
chr10	95667849	95790945	123097	3	1	4	0.30102999	0.01077081	0	9	0	1.01542894
chr10	95790945	95955990	165046	3	1	4	0.30102999	0.02074938	0	8	0	0.79906872
chr10	95955990	95995733	39744	1	1	4	0.30102999	0.03812622	0	7	0	0.60763643
chr10	95995733	96044614	48882	1	1	4	0.30102999	0.06713722	0	6	0	0.44141547
chr10	96044614	96161373	116760	3	1	4	0.30102999	0.03812622	0	7	0	0.60763643
chr10	96161373	96407740	246368	6	1	4	0.30102999	0.06713722	0	6	0	0.44141547
chr10	96407740	96540436	132697	4	1	4	0.1218695	0.06713722	1	6	0.1218695	0.44141547
chr10	96540436	96540495	60	1	1	5	0.1218695	0.07511598	1	7	0.1218695	0.43181735
chr10	96540495	96612764	72270	2	1	5	0.1218695	0.12309572	1	6	0.1218695	0.30102999
chr10	96612764	96750804	138041	2	1	4	0.1218695	0.06713722	1	6	0.1218695	0.44141547
chr10	96750804	96750863	60	1	1	4	0.1218695	0.03812622	1	7	0.1218695	0.60763643
chr10	96750863	96828377	77515	1	1	4	0.1218695	0.06713722	1	6	0.1218695	0.44141547
chr10	96828377	96872364	43988	3	1	4	0.1218695	0.03812622	1	7	0.1218695	0.60763643
chr10	96872364	97021441	149078	4	1	4	0.1218695	0.02074938	1	8	0.1218695	0.79906872
chr10	97021441	97250768	229328	5	1	4	0.1218695	0.03812622	1	7	0.1218695	0.60763643
chr10	97250768	97315278	64511	3	1	4	0.1218695	0.02074938	1	8	0.1218695	0.79906872
chr10	97315278	97413126	97849	2	1	4	0.30102999	0.02074938	0	8	0	0.79906872
chr10	97413126	97452727	39602	3	1	4	0.1218695	0.02074938	1	8	0.1218695	0.79906872
chr10	97452727	97515782	63056	2	1	4	0.30102999	0.02074938	0	8	0	0.79906872
chr10	97515782	97515834	53	1	1	4	0.30102999	0.01077081	0	9	0	1.01542894
chr10	97515834	97583033	67200	2	1	4	0.30102999	0.02074938	0	8	0	0.79906872
chr10	97583033	97640044	57012	1	1	4	0.30102999	0.03812622	0	7	0	0.60763643
chr10	97640044	97870342	230299	7	1	4	0.1218695	0.02074938	1	8	0.1218695	0.79906872
chr10	97870342	97870401	60	1	1	4	0.1218695	0.01077081	1	9	0.1218695	1.01542894
chr10	97870401	97951720	81320	2	1	4	0.30102999	0.01077081	0	9	0	1.01542894
chr10	97951720	97951779	60	1	1	4	0.1218695	0.01077081	1	9	0.1218695	1.01542894
chr10	97951779	98108056	156278	3	1	4	0.30102999	0.01077081	0	9	0	1.01542894
chr10	98108056	98122164	14109	2	1	5	0.30102999	0.01320236	0	10	0	0.97390707
chr10	98122164	98165030	42867	1	1	5	0.30102999	0.02473314	0	9	0	0.76806864
chr10	98165030	98228549	63520	2	1	6	0.30102999	0.02793176	0	10	0	0.74627054
chr10	98228549	98303837	75289	3	1	6	0.30102999	0.00778066	0	12	0	1.17038931
chr10	98303837	98392798	88962	4	1	7	0.30102999	0.01667721	0	12	0	0.92532268
chr10	98392798	98463178	70381	1	1	6	0.30102999	0.01518174	0	11	0	0.9449689
chr10	98463178	98500712	37535	2	1	7	0.30102999	0.01667721	0	12	0	0.92532268
chr10	98500712	98644785	144074	3	1	5	0.30102999	0.00317045	0	12	0	1.46403142
chr10	98644785	98717404	72620	2	1	5	0.30102999	0.00141024	0	13	0	1.7506523
chr10	98717404	98754514	37111	2	1	6	0.30102999	0.00373523	0	13	0	1.42397267
chr10	98754514	98806816	52303	2	1	6	0.30102999	0.00166733	0	14	0	1.70763027
chr10	98806816	98819448	12633	1	1	6	0.30102999	0.00373523	0	13	0	1.42397267
chr10	98819448	98883136	63689	1	1	5	0.30102999	0.00317045	0	12	0	1.46403142
chr10	98883136	98995077	111942	3	1	5	0.30102999	0.01320236	0	10	0	0.97390707
chr10	98995077	99025824	30748	2	1	6	0.30102999	0.00166733	0	14	0	1.70763027
chr10	99025824	99093245	67422	2	1	4	0.30102999	4.45E-04	0	13	0	2.13824703
chr10	99093245	99150534	57290	1	1	4	0.30102999	0.00108487	0	12	0	1.81706455
chr10	99150534	99150591	58	1	1	5	0.30102999	0.00317045	0	12	0	1.46403142
chr10	99150591	99190618	40028	1	1	5	0.30102999	0.00666883	0	11	0	1.20557689
chr10	99190618	99243856	53239	5	1	5	0.30102999	0.00141024	0	13	0	1.7506523
chr10	99243856	99257808	13953	2	1	6	0.30102999	0.00373523	0	13	0	1.42397267
chr10	99257808	99350514	92707	5	1	7	0.30102999	0.00413023	0	14	0	1.39918918
chr10	99350514	99442232	91719	4	1	6	0.30102999	0.00166733	0	14	0	1.70763027
chr10	99442232	99475560	33329	2	1	7	0.30102999	0.00413023	0	14	0	1.39918918
chr10	99475560	99526638	51079	5	1	7	0.30102999	0.00183109	0	15	0	1.68336308
chr10	99526638	99668746	142109	3	1	6	0.30102999	6.86E-04	0	15	0	2.02387299
chr10	99668746	99733762	65017	3	1	7	0.30102999	0.00183109	0	15	0	1.68336308
chr10	99733762	99836194	102433	3	1	8	0.30102999	0.00433294	0	15	0	1.3873209
chr10	99836194	99914897	78704	1	1	8	0.30102999	0.00909834	0	14	0	1.13428373
chr10	99914897	99990664	75768	2	1	7	0.30102999	0.01667721	0	12	0	0.92532268

chr10	99990664	100074141	83478	2	1	7	0.30102999	0.00859896	0	13	0	1.14735594
chr10	100074141	100074200	60	1	1	7	0.30102999	0.00413023	0	14	0	1.39918918
chr10	100074200	100164382	90183	2	1	6	0.30102999	0.00373523	0	13	0	1.42397267
chr10	100164382	100470191	305810	9	1	4	0.30102999	0.00247414	0	11	0	1.52371709
chr10	100470191	100536851	66661	2	1	4	0.30102999	0.00530919	0	10	0	1.2568129
chr10	100536851	100667784	130934	4	1	4	0.30102999	0.00247414	0	11	0	1.52371709
chr10	100667784	100960668	292885	6	1	4	0.30102999	0.00530919	0	10	0	1.2568129
chr10	100960668	100960727	60	1	1	4	0.30102999	0.00247414	0	11	0	1.52371709
chr10	100960727	101128142	167416	4	1	4	0.30102999	0.00530919	0	10	0	1.2568129
chr10	101128142	101170066	41925	2	1	4	0.30102999	0.00247414	0	11	0	1.52371709
chr10	101170066	101193346	23281	1	1	4	0.30102999	0.00530919	0	10	0	1.2568129
chr10	101193346	101294355	101010	1	0	4	0	0.00530919	0	10	0	1.2568129
chr10	101294355	101294414	60	1	0	6	0	0.02793176	0	10	0	0.74627054
chr10	101294414	101514267	219854	7	0	5	0	0.01320236	0	10	0	0.97390707
chr10	101514267	101542596	28330	2	0	6	0	0.02793176	0	10	0	0.74627054
chr10	101542596	101542655	60	1	0	6	0	0.01518174	0	11	0	0.9449689
chr10	101542655	101704515	161861	4	0	4	0	0.00530919	0	10	0	1.2568129
chr10	101704515	101704574	60	1	1	4	0.30102999	0.00530919	0	10	0	1.2568129
chr10	101704574	101802101	97528	2	1	3	0.30102999	0.00170589	0	10	0	1.61091002
chr10	101802101	101992529	190429	10	1	4	0.30102999	0.00530919	0	10	0	1.2568129
chr10	101992529	102052328	59800	3	0	3	0	0.00378107	0	9	0	1.33111237
chr10	102052328	102080193	27866	2	0	4	0	0.00530919	0	10	0	1.2568129
chr10	102080193	102113993	33801	1	0	4	0	0.01077081	0	9	0	1.01542894
chr10	102113993	102190585	76593	1	0	3	0	0.00378107	0	9	0	1.33111237
chr10	102190585	102309789	119205	6	0	2	0	9.54E-04	0	9	0	1.74076927
chr10	102309789	102428425	118637	1	0	2	0	0.00221948	0	8	0	1.44210395
chr10	102428425	102516459	88035	2	0	4	0	0.02074938	0	8	0	0.79906872
chr10	102516459	102516518	60	1	0	4	0	0.01077081	0	9	0	1.01542894
chr10	102516518	102676543	160026	3	0	3	0	0.00378107	0	9	0	1.33111237
chr10	102676543	102676602	60	1	0	4	0	0.00247414	0	11	0	1.52371709
chr10	102676602	102705186	28585	1	0	3	0	0.00170589	0	10	0	1.61091002
chr10	102705186	102705245	60	1	1	3	0.30102999	0.00170589	0	10	0	1.61091002
chr10	102705245	102777838	72594	5	0	3	0	0.00170589	0	10	0	1.61091002
chr10	102777838	102787703	9866	2	0	3	0	7.28E-04	0	11	0	1.91540774
chr10	102787703	102897441	109739	3	0	3	0	0.00170589	0	10	0	1.61091002
chr10	102897441	102897500	60	1	1	4	0.30102999	0.00247414	0	11	0	1.52371709
chr10	102897500	102987480	89981	1	1	4	0.30102999	0.01077081	0	9	0	1.01542894
chr10	102987480	103049803	62324	1	1	4	0.30102999	0.02074938	0	8	0	0.79906872
chr10	103049803	103049862	60	1	1	5	0.30102999	0.01320236	0	10	0	0.97390707
chr10	103049862	103098408	48547	1	1	4	0.30102999	0.00530919	0	10	0	1.2568129
chr10	103098408	103176029	77622	3	1	4	0.30102999	0.00247414	0	11	0	1.52371709
chr10	103176029	103176088	60	1	1	5	0.30102999	0.00666883	0	11	0	1.20557689
chr10	103176088	103208863	32776	1	1	4	0.30102999	0.00247414	0	11	0	1.52371709
chr10	103208863	103275686	66824	2	1	4	0.30102999	0.00530919	0	10	0	1.2568129
chr10	103275686	103366970	91285	5	1	4	0.30102999	0.01077081	0	9	0	1.01542894
chr10	103366970	103419623	52654	2	1	3	0.30102999	0.00378107	0	9	0	1.33111237
chr10	103419623	103449273	29651	2	1	4	0.30102999	0.01077081	0	9	0	1.01542894
chr10	103449273	103534634	85362	2	1	5	0.30102999	0.01320236	0	10	0	0.97390707
chr10	103534634	103602586	67953	6	1	5	0.1218695	0.00666883	1	11	0.1218695	1.20557689
chr10	103602586	103693010	90425	3	1	6	0.1218695	0.01518174	1	11	0.1218695	0.9449689
chr10	103693010	103693069	60	1	1	6	0.05404976	0.01518174	2	11	0.30102999	0.9449689
chr10	103693069	103750391	57323	2	1	6	0.1218695	0.01518174	1	11	0.1218695	0.9449689
chr10	103750391	103774446	24056	1	1	6	0.30102999	0.01518174	0	11	0	0.9449689
chr10	103774446	103872818	98373	2	1	6	0.30102999	0.04875589	0	9	0	0.5732208
chr10	103872818	103988947	116130	6	1	6	0.30102999	0.02793176	0	10	0	0.74627054
chr10	103988947	104066826	77880	3	1	5	0.30102999	0.01320236	0	10	0	0.97390707
chr10	104066826	104066885	60	1	1	6	0.30102999	0.02793176	0	10	0	0.74627054
chr10	104066885	104130174	63290	2	1	5	0.30102999	0.01320236	0	10	0	0.97390707
chr10	104130174	104130226	53	1	1	6	0.1218695	0.02793176	1	10	0.1218695	0.74627054
chr10	104130226	104177975	47750	2	1	6	0.30102999	0.02793176	0	10	0	0.74627054
chr10	104177975	104178034	60	1	1	6	0.30102999	0.01518174	0	11	0	0.9449689
chr10	104178034	104222014	43981	2	1	5	0.30102999	0.00666883	0	11	0	1.20557689
chr10	104222014	104222073	60	1	1	7	0.30102999	0.03037338	0	11	0	0.73110763
chr10	104222073	104412294	190222	7	1	6	0.30102999	0.01518174	0	11	0	0.9449689
chr10	104412294	104445690	33397	1	1	6	0.30102999	0.02793176	0	10	0	0.74627054
chr10	104445690	104477485	31796	2	1	6	0.1218695	0.02793176	1	10	0.1218695	0.74627054
chr10	104477485	104556998	79514	2	1	6	0.1218695	0.04875589	1	9	0.1218695	0.5732208
chr10	104556998	104557057	60	1	2	6	0.30102999	0.04875589	1	9	0.05404976	0.5732208
chr10	104557057	104575440	18384	1	2	5	0.30102999	0.02473314	1	9	0.05404976	0.76806864
chr10	104575440	104590252	14813	1	2	5	0.61140001	0.02473314	0	9	0	0.76806864
chr10	104590252	104899137	308886	11	2	5	0.30102999	0.02473314	1	9	0.05404976	0.76806864
chr10	104899137	104899196	60	1	2	5	0.1575501	0.02473314	2	9	0.1575501	0.76806864
chr10	104899196	104989009	89814	2	2	4	0.1575501	0.01077081	2	9	0.1575501	1.01542894
chr10	104989009	105166144	177136	6	2	4	0.30102999	0.01077081	1	9	0.05404976	1.01542894
chr10	105166144	105206854	40711	3	2	4	0.1575501	0.01077081	2	9	0.1575501	1.01542894
chr10	105206854	105350220	143367	5	2	4	0.30102999	0.01077081	1	9	0.05404976	1.01542894
chr10	105350220	105374321	24102	2	2	4	0.30102999	0.00530919	1	10	0.05404976	1.2568129
chr10	105374321	105431236	56916	1	2	4	0.61140001	0.00530919	0	10	0	1.2568129
chr10	105431236	105431294	59	1	2	4	0.61140001	0.00247414	0	11	0	1.52371709
chr10	105431294	105727489	296196	7	2	4	0.61140001	0.00530919	0	10	0	1.2568129
chr10	105727489	105727541	53	1	2	5	0.61140001	0.01320236	0	10	0	0.97390707
chr10	105727541	105823642	96102	4	2	4	0.61140001	0.00530919	0	10	0	1.2568129
chr10	105823642	105823701	60	1	2	5	0.61140001	0.00666883	0	11	0	1.20557689
chr10	105823701	105852252	28552	1	2	4	0.61140001	0.00530919	0	10	0	1.2568129
chr10	105852252	105952489	100238	4	2	3	0.61140001	0.00170589	0	10	0	1.61091002
chr10	105952489	105952548	60	1	2	4	0.61140001	0.00530919	0	10	0	1.2568129
chr10	105952548	106021532	68985	2	2	3	0.61140001	0.00170589	0	10	0	1.61091002
chr10	106021532	106021591	60	1	2	4	0.61140001	0.00530919	0	10	0	1.2568129
chr10	106021591	106153921	132331	6	2	3	0.61140001	0.00378107	0	9	0	1.33111237
chr10	106153921	106411883	257963	4	2	3	0.30102999	0.00378107	1	9	0.05404976	1.33111237
chr10	106411883	106411942	60	1	2	4	0.30102999	0.01077081	1	9	0.05404976	1.01542894
chr10	106411942	106437463	25522	1	2	4	0.61140001	0.01077081	0	9	0	1.01542894
chr10	106437463	106611508	174046	4	2	4	0.61140001	0.00530919	0	10	0	1.2568129
chr10	106611508	106643119	31612	1	1	4	0.30102999	0.02074938	0	8	0	0.79906872
chr10	106643119	106675624	32506	1	1	3	0.30102999	0.0079614	0	8	0	1.07548421
chr10	106675624	106753963	78340	2	1	3	0.30102999	0.00170589	0	10	0	1.61091002
chr10	106753963	106817638	63676	1	1	3	0.30102999	0.00378107	0	9	0	1.33111237
chr10	106817638	106861049	43412	1	1	3	0.30102999	0.0079614	0	8	0	1.07548421
chr10	106861049	106972403	111355	2	1	3	0.30102999	0.01598258	0	7	0	0.84395715
chr10	106972403	106972462	60	1	1	4	0.30102999	0.03812622	0	7	0	0.60763643
chr10	106972462	107049651	77190	2	1	3	0.30102999	0.01598258	0	7	0	0.84395715
chr10	107049651	107401835	352185	2	0	1	0	0.00478973	0	5	0	1.02643191
chr10	107401835	107522856	121022	2	1	1	0.30102999	0.00478973	0	5	0	1.02643191
chr10	107522856	107522915	60	1	1	1	0.30102999	0.00204627	0	6	0	1.31360226
chr10	107522915	107669748	146834	1	0	1	0	0.01091641	0	4	0	0.76005302
chr10	107669748	107669807	60	1	0	2	0	0.0429175	0	4	0	0.47744371
chr10	107669807	107816122	146316	1	0	1	0	0.01091641	0	4	0	0.76005302
chr10	107816122	107971155	155034	2	1	1	0.30102999	0.01091641	0	4	0	0.76005302
chr10	107971155	107971214	60	1	2	2	0.61140001	0.02162467	0	5	0	0.68214471
chr10	107971214	108054054	82841	1	1	2	0.30102999	0.02162467	0	5	0	0.68214471
chr10	108054054	108299619	245566	2	1	2	0.30102999	0.01053319	0	6	0	0.91219088
chr10	108299619	108339032	39414	3	1	4	0.30102999	0.03812622	0	7	0	0.60763643
chr10	108339032	108512225	173194	3	0	2	0	0.01053319	0	6	0	0.91219088
chr10	108512225	108512284	60	1	0	4	0	0.02074938	0	8	0	0.79906872
chr10	108512284	108572598	60315	2	0	3	0	0.03070643	0	6	0	0.63695542
chr10	108572598	108641558	68961	2	0	4	0	0.01077081	0	9	0	1.01542894
chr10	108641558	108732272	90715	2	0	4	0	0.02074938	0	8	0	0.79906872
chr10	108732272	108849689	117418	2	0	4	0	0.03812622	0	7	0	0.60763643
chr10	108849689	108950917	101229	2	0	2	0	0.00493743	0	7	0	1.16581773
chr10	108950917	109080795	129879	1	0	1	0	0.00204627	0	6	0	1.31360226
chr10	109080795	109188508	107714	2	0	2	0	0.01053319	0	6	0	0.91219088
chr10	109188508	109279942	91435	2	1	4	0.30102999	0.06713722	0	6	0	0.44141547
chr10	109279942	109280001	60	1	1	5	0.30102999	0.07511598	0	7	0	0.43181735
chr10	109280001	109454771	174771	1	0	1	0	0.00204627	0	6	0	1.31360226

chr10	109454771	109552932	98162	1	0	1	0	0.00478973	0	5	0	1.02643191
chr10	109552932	109552991	60	1	1	1	0.30102999	0.00478973	0	5	0	1.02643191
chr10	109552991	109670302	117312	1	0	1	0	0.01091641	0	4	0	0.76005302
chr10	109670302	109670361	60	1	0	1	0	0.00204627	0	6	0	1.31360226
chr10	109670361	109862404	192044	2	0	1	0	0.01091641	0	4	0	0.76005302
chr10	109862404	110057187	194784	2	0	0	0	0	0	1	0	0.30102999
chr10	110057187	110057246	60	1	0	1	0	0.01091641	0	4	0	0.76005302
chr10	110057246	110242525	185280	2	0	0	0	0	0	1	0	0.30102999
chr10	110242525	110425573	183049	2	0	0	0	0	0	2	0	0.61140001
chr10	110425573	110425632	60	1	1	2	0.30102999	0.0429175	0	4	0	0.47744371
chr10	110425632	110611992	186361	2	1	0	0.30102999	0	0	2	0	0.61140001
chr10	110611992	110729064	117073	1	0	0	0	0	0	2	0	0.61140001
chr10	110729064	110966288	237225	2	0	1	0	0.01091641	0	4	0	0.76005302
chr10	110966288	111037990	71703	2	1	1	0.30102999	0.00478973	0	5	0	1.02643191
chr10	111037990	111038049	60	1	1	3	0.30102999	0.01598258	0	7	0	0.84395715
chr10	111038049	111232394	194346	1	1	2	0.30102999	0.02162467	0	5	0	0.68214471
chr10	111232394	111304044	71651	1	0	2	0	0.02162467	0	5	0	0.68214471
chr10	111304044	111544670	240627	3	0	2	0	0.00493743	1	7	0.30102999	1.16581773
chr10	111544670	111544729	60	1	0	4	0	0.00530919	1	10	0.30102999	1.2568129
chr10	111544729	111628432	83704	1	0	3	0	0.00170589	1	10	0.30102999	1.61091002
chr10	111628432	111639962	11531	1	0	3	0	0.0079614	1	8	0.30102999	1.07548421
chr10	111639962	111640021	60	1	1	3	0.1218695	0.0079614	1	8	0.1218695	1.07548421
chr10	111640021	111731310	91290	3	0	2	0	0.00221948	1	8	0.30102999	1.44210395
chr10	111731310	111731369	60	1	2	2	0.30102999	9.54E-04	1	9	0.05404976	1.74076927
chr10	111731369	111815687	84319	2	2	2	0.30102999	0.00221948	1	8	0.05404976	1.44210395
chr10	111815687	111815746	60	1	2	3	0.30102999	0.0079614	1	8	0.05404976	1.07548421
chr10	111815746	111872613	56868	2	2	2	0.30102999	0.00221948	1	8	0.05404976	1.44210395
chr10	111872613	111973270	100658	1	1	2	0.1218695	0.00221948	1	8	0.1218695	1.44210395
chr10	111973270	112012899	39630	2	1	3	0.1218695	0.00170589	1	10	0.1218695	1.61091002
chr10	112012899	112342409	329511	7	1	3	0.1218695	0.00378107	1	9	0.1218695	1.33111237
chr10	112342409	112405234	62826	1	1	3	0.1218695	0.0079614	1	8	0.1218695	1.07548421
chr10	112405234	112438134	32901	2	2	3	0.30102999	0.0079614	1	8	0.05404976	1.07548421
chr10	112438134	112537387	99254	3	2	3	0.30102999	0.00378107	1	9	0.05404976	1.33111237
chr10	112537387	112635757	98371	2	1	3	0.1218695	0.0079614	1	8	0.1218695	1.07548421
chr10	112635757	112635816	60	1	2	3	0.30102999	0.0079614	1	8	0.05404976	1.07548421
chr10	112635816	112685048	49233	3	1	2	0.1218695	0.00221948	1	8	0.1218695	1.44210395
chr10	112685048	112724426	39379	1	0	2	0	0.00493743	1	7	0.30102999	1.16581773
chr10	112724426	112724485	60	1	0	2	0	0.00221948	1	8	0.30102999	1.44210395
chr10	112724485	112753257	28773	1	0	2	0	0.00493743	1	7	0.30102999	1.16581773
chr10	112753257	112753316	60	1	1	3	0.1218695	0.01598258	1	7	0.1218695	0.84395715
chr10	112753316	112839536	86221	2	0	2	0	0.00493743	1	7	0.30102999	1.16581773
chr10	112839536	112839586	51	1	1	2	0.1218695	0.00221948	1	8	0.1218695	1.44210395
chr10	112839586	112915164	75579	1	1	2	0.1218695	0.01053319	1	6	0.1218695	0.91219088
chr10	112915164	113072857	157694	1	1	1	0.1218695	0.00478973	1	5	0.1218695	1.02643191
chr10	113072857	113072916	60	1	1	1	0.1218695	8.47E-04	1	7	0.1218695	1.62048027
chr10	113072916	113217415	144500	1	1	1	0.1218695	0.01091641	1	4	0.1218695	0.76005302
chr10	113217415	113377242	159828	1	1	1	0.1218695	0.05404976	1	2	0.1218695	0.30102999
chr10	113377242	113377301	60	1	2	1	0.30102999	0.05404976	1	2	0.05404976	0.30102999
chr10	113377301	113702235	324935	1	1	1	0.30102999	0.05404976	0	2	0	0.30102999
chr10	113702235	113702294	60	1	1	2	0.30102999	0.02162467	0	5	0	0.68214471
chr10	113702294	113872224	169931	1	1	1	0.30102999	0.02438896	0	3	0	0.51676182
chr10	113872224	113911315	39092	2	1	1	0.30102999	0.00478973	0	5	0	1.02643191
chr10	113911315	113931990	20676	2	1	1	0.30102999	0.00204627	0	6	0	1.31360226
chr10	113931990	114005776	73787	2	1	2	0.30102999	0.01053319	0	6	0	0.91219088
chr10	114005776	114118051	112276	2	1	1	0.30102999	0.00204627	0	6	0	1.31360226
chr10	114118051	114190333	72283	4	1	2	0.30102999	0.00493743	0	7	0	1.16581773
chr10	114190333	114278859	88527	2	1	1	0.30102999	8.47E-04	0	7	0	1.62048027
chr10	114278859	114339686	60828	1	1	1	0.30102999	0.00204627	0	6	0	1.31360226
chr10	114339686	114428032	88347	3	3	2	0.51676182	0.01053319	1	6	0.02438896	0.91219088
chr10	114428032	114497495	69464	1	2	2	0.30102999	0.01053319	1	6	0.05404976	0.91219088
chr10	114497495	114534908	37414	2	2	3	0.30102999	0.03070643	1	6	0.05404976	0.63695542
chr10	114534908	114534967	60	1	3	3	0.51676182	0.03070643	1	6	0.02438896	0.63695542
chr10	114534967	114595633	60667	1	3	2	0.51676182	0.01053319	1	6	0.02438896	0.91219088
chr10	114595633	114595692	60	1	3	2	0.51676182	0.00493743	1	7	0.02438896	1.16581773
chr10	114595692	114676227	80536	1	3	2	0.51676182	0.01053319	1	6	0.02438896	0.91219088
chr10	114676227	114676286	60	1	3	3	0.51676182	0.03070643	1	6	0.02438896	0.63695542
chr10	114676286	114831514	155229	3	3	2	0.51676182	0.02162467	1	5	0.02438896	0.68214471
chr10	114831514	115204957	373444	5	3	3	0.51676182	0.03070643	1	6	0.02438896	0.63695542
chr10	115204957	115205016	60	1	3	4	0.51676182	0.06713722	1	6	0.02438896	0.44141547
chr10	115205016	115349003	143988	3	3	4	0.51676182	0.11390336	1	5	0.02438896	0.30102999
chr10	115349003	115454781	105779	4	3	4	0.51676182	0.06713722	1	6	0.02438896	0.44141547
chr10	115454781	115542162	87382	4	3	3	0.51676182	0.05670724	1	5	0.02438896	0.45545077
chr10	115542162	115640259	98098	2	3	3	0.51676182	0.10122019	1	4	0.02438896	0.30102999
chr10	115640259	115676805	36547	2	3	3	0.51676182	0.05670724	1	5	0.02438896	0.45545077
chr10	115676805	115687170	10366	2	3	4	0.51676182	0.06713722	1	6	0.02438896	0.44141547
chr10	115687170	115736962	49793	1	3	4	0.51676182	0.11390336	1	5	0.02438896	0.30102999
chr10	115736962	115827937	90976	1	3	4	0.51676182	0.30102999	1	3	0.02438896	0.10122019
chr10	115827937	115827996	60	1	3	4	0.51676182	0.18734596	1	4	0.02438896	0.18734596
chr10	115827996	115921404	93409	2	3	3	0.51676182	0.10122019	1	4	0.02438896	0.30102999
chr10	115921404	116025608	104205	4	3	4	0.51676182	0.11390336	1	5	0.02438896	0.30102999
chr10	116025608	116056380	30773	1	3	4	0.51676182	0.18734596	1	4	0.02438896	0.18734596
chr10	116056380	116092980	36601	2	3	4	0.51676182	0.11390336	1	5	0.02438896	0.30102999
chr10	116092980	116152540	59561	3	3	4	0.51676182	0.06713722	1	6	0.02438896	0.44141547
chr10	116152540	116293190	140651	3	2	4	0.30102999	0.11390336	1	5	0.05404976	0.30102999
chr10	116293190	116293249	60	1	2	4	0.30102999	0.06713722	1	6	0.05404976	0.44141547
chr10	116293249	116405945	112697	2	2	4	0.30102999	0.18734596	1	4	0.05404976	0.18734596
chr10	116405945	116406004	60	1	2	4	0.30102999	0.06713722	1	6	0.05404976	0.44141547
chr10	116406004	116477971	71968	1	2	3	0.30102999	0.10122019	1	4	0.05404976	0.30102999
chr10	116477971	116478030	60	1	3	3	0.51676182	0.10122019	1	4	0.02438896	0.30102999
chr10	116478030	116705895	227866	5	2	3	0.30102999	0.10122019	1	4	0.05404976	0.30102999
chr10	116705895	116786582	80688	2	2	3	0.30102999	0.05670724	1	5	0.05404976	0.45545077
chr10	116786582	116786641	60	1	2	4	0.30102999	0.06713722	1	6	0.05404976	0.44141547
chr10	116786641	116931514	144874	2	2	3	0.30102999	0.10122019	1	4	0.05404976	0.30102999
chr10	116931514	116931573	60	1	2	4	0.30102999	0.18734596	1	4	0.05404976	0.18734596
chr10	116931573	117029558	97986	2	2	3	0.30102999	0.10122019	1	4	0.05404976	0.30102999
chr10	117029558	117029617	60	1	2	4	0.30102999	0.18734596	1	4	0.05404976	0.18734596
chr10	117029617	117096311	66695	2	2	3	0.30102999	0.10122019	1	4	0.05404976	0.30102999
chr10	117096311	117163312	67002	1	2	3	0.30102999	0.17593012	1	3	0.05404976	0.17593012
chr10	117163312	117163371	60	1	2	3	0.30102999	0.10122019	1	4	0.05404976	0.30102999
chr10	117163371	117228984	65614	1	1	3	0.1218695	0.10122019	1	4	0.1218695	0.30102999
chr10	117228984	117229043	60	1	1	3	0.1218695	0.05670724	1	5	0.1218695	0.45545077
chr10	117229043	117274633	45591	1	1	3	0.1218695	0.10122019	1	4	0.1218695	0.30102999
chr10	117274633	117547727	273095	4	1	3	0.30102999	0.10122019	0	4	0	0.30102999
chr10	117547727	117547786	60	1	1	3	0.30102999	0.05670724	0	5	0	0.45545077
chr10	117547786	117665191	117406	2	1	3	0.30102999	0.10122019	0	4	0	0.30102999
chr10	117665191	117723984	58794	2	1	3	0.30102999	0.05670724	0	5	0	0.45545077
chr10	117723984	117853220	129237	2	1	3	0.30102999	0.10122019	0	4	0	0.30102999
chr10	117853220	117896853	43634	2	1	3	0.30102999	0.05670724	0	5	0	0.45545077
chr10	117896853	118091833	194981	4	1	2	0.30102999	0.02162467	0	5	0	0.68214471
chr10	118091833	118181536	89704	3	1	2	0.30102999	0.01053319	0	6	0	0.91219088
chr10	118181536	118222153	40618	2	1	2	0.30102999	0.00221948	0	8	0	1.44210395
chr10	118222153	118305585	83433	1	1	2	0.30102999	0.00493743	0	7	0	1.16581773
chr10	118305585	118401044	95460	5	1	2	0.30102999	0.01053319	0	6	0	0.91219088
chr10	118401044	118425239	24196	1	1	2	0.30102999	0.02162467	0	5	0	0.68214471
chr10	118425239	118472553	47315	2	1	2	0.30102999	0.01053319	0	6	0	0.91219088
chr10	118472553	118536148	63596	2	1	2	0.30102999	0.00493743	0	7	0	1.16581773
chr10	118536148	118738762	202615	4	0	2	0	0.01053319	0	6	0	0.91219088
chr10	118738762	118820046	81285	3	0	2	0	0.00493743	0	7	0	1.16581773
chr10	118820046	118882962	62917	2	1	2	0.30102999	0.00493743	0	7	0	1.16581773
chr10	118882962	118969727	86766	3	1	2	0.30102999	0.00221948	0	8	0	1.44210395
chr10	118969727	119012994	43268	1	1	2	0.30102999	0.01053319	0	6	0	0.91219088
chr10	119012994	119042919	29926	1	0	2	0	0.01053319	0	6	0	0.91219088
chr10	119042919	119078365	35447	2	0	2	0	0.00493743	0	7	0	1.16581773
chr10	119078365	119100470	22106	2	1	2	0.30102999	0.00493743	0	7	0	1.16581773

chr10	119100470	119122473	22004	2	1	2	0.30102999	0.00221948	0	8	0	1.44210395
chr10	119122473	119168883	46411	1	1	2	0.30102999	0.00493743	0	7	0	1.16581773
chr10	119168883	119168942	60	1	1	2	0.30102999	0.00221948	0	8	0	1.44210395
chr10	119168942	119327170	158229	4	1	1	0.30102999	0.00204627	0	6	0	1.31360226
chr10	119327170	119434802	107633	1	1	1	0.30102999	0.00478973	0	5	0	1.02643191
chr10	119434802	119434861	60	1	1	2	0.30102999	0.02162467	0	5	0	0.68214471
chr10	119434861	119561238	126378	1	1	1	0.30102999	0.01091641	0	4	0	0.76005302
chr10	119561238	119685626	124389	1	1	1	0.30102999	0.02438896	0	3	0	0.51676182
chr10	119685626	119780990	95365	2	2	1	0.61140001	0.01091641	0	4	0	0.76005302
chr10	119780990	119805443	24454	2	2	2	0.61140001	0.02162467	0	5	0	0.68214471
chr10	119805443	119813612	8170	1	1	2	0.30102999	0.02162467	0	5	0	0.68214471
chr10	119813612	119925455	111844	3	1	1	0.30102999	0.00478973	0	5	0	1.02643191
chr10	119925455	120054686	129232	3	1	2	0.30102999	0.02162467	0	5	0	0.68214471
chr10	120054686	120306894	252209	4	3	2	0.93173516	0.02162467	0	5	0	0.68214471
chr10	120306894	120508795	201902	6	3	3	0.93173516	0.05670724	0	5	0	0.45545077
chr10	120508795	120562393	53599	1	3	2	0.93173516	0.02162467	0	5	0	0.68214471
chr10	120562393	120678170	115778	1	1	2	0.30102999	0.02162467	0	5	0	0.68214471
chr10	120678170	120728496	50327	2	1	4	0.30102999	0.06713722	0	6	0	0.44141547
chr10	120728496	120807022	78527	2	1	4	0.30102999	0.02074938	0	8	0	0.79906872
chr10	120807022	120807081	60	1	2	4	0.61140001	0.02074938	0	8	0	0.79906872
chr10	120807081	120850434	43354	2	1	4	0.30102999	0.02074938	0	8	0	0.79906872
chr10	120850434	120970915	120482	2	1	3	0.30102999	0.0079614	0	8	0	1.07548421
chr10	120970915	120970974	60	1	1	4	0.30102999	0.02074938	0	8	0	0.79906872
chr10	120970974	121290609	319636	7	1	4	0.30102999	0.03812622	0	7	0	0.60763643
chr10	121290609	121430894	140286	5	1	4	0.30102999	0.02074938	0	8	0	0.79906872
chr10	121430894	121450103	19210	2	1	5	0.30102999	0.04407651	0	8	0	0.58747015
chr10	121450103	121458431	8329	2	1	5	0.30102999	0.02473314	0	9	0	0.76806864
chr10	121458431	121551651	93221	2	1	4	0.30102999	0.01077081	0	9	0	1.01542894
chr10	121551651	121607181	55531	3	1	3	0.30102999	0.0079614	0	8	0	1.07548421
chr10	121607181	121630730	23550	2	1	4	0.30102999	0.01077081	0	9	0	1.01542894
chr10	121630730	121692456	61727	2	1	4	0.30102999	0.02074938	0	8	0	0.79906872
chr10	121692456	121720948	28493	3	1	5	0.30102999	0.04407651	0	8	0	0.58747015
chr10	121720948	121775059	54112	1	1	4	0.30102999	0.02074938	0	8	0	0.79906872
chr10	121775059	121862696	87638	1	1	3	0.30102999	0.01598258	0	7	0	0.84395715
chr10	121862696	122239298	376603	3	1	3	0.30102999	0.03070643	0	6	0	0.63695542
chr10	122239298	122328262	88965	2	1	3	0.30102999	0.05670724	0	5	0	0.45545077
chr10	122328262	122369351	41090	2	1	3	0.30102999	0.03070643	0	6	0	0.63695542
chr10	122369351	122543185	173835	2	1	2	0.30102999	0.01053319	0	6	0	0.91219088
chr10	122543185	122603290	60106	2	1	2	0.30102999	0.00493743	0	7	0	1.16581773
chr10	122603290	122668785	65496	3	1	2	0.30102999	0.00221948	0	8	0	1.44210395
chr10	122668785	122761687	92903	1	1	2	0.30102999	0.01053319	0	6	0	0.91219088
chr10	122761687	122817760	56074	1	1	2	0.30102999	0.0429175	0	4	0	0.47744371
chr10	122817760	122828243	10484	2	1	2	0.30102999	0.02162467	0	5	0	0.68214471
chr10	122828243	122934825	106583	2	1	2	0.30102999	0.0429175	0	4	0	0.47744371
chr10	122934825	122934884	60	1	1	2	0.30102999	0.02162467	0	5	0	0.68214471
chr10	122934884	123025474	90591	1	1	1	0.30102999	0.00478973	0	5	0	1.02643191
chr10	123025474	123116447	90974	2	1	1	0.30102999	0.00204627	0	6	0	1.31360226
chr10	123116447	123208806	92360	2	1	3	0.1218695	0.03070643	1	6	0.1218695	0.63695542
chr10	123208806	123208865	60	1	2	3	0.1575501	0.0079614	2	8	0.1575501	1.07548421
chr10	123208865	123274734	65870	2	2	3	0.1575501	0.03070643	2	6	0.1575501	0.63695542
chr10	123274734	123353262	78529	4	2	3	0.1575501	0.01598258	2	7	0.1575501	0.84395715
chr10	123353262	123468418	115157	1	1	3	0.1218695	0.01598258	1	7	0.1218695	0.84395715
chr10	123468418	123600639	132222	4	1	3	0.1218695	0.0079614	1	8	0.1218695	1.07548421
chr10	123600639	123733571	132933	5	1	3	0.1218695	0.01598258	1	7	0.1218695	0.84395715
chr10	123733571	123792851	59281	1	1	3	0.1218695	0.03070643	1	6	0.1218695	0.63695542
chr10	123792851	123792910	60	1	2	3	0.30102999	0.0079614	1	8	0.05404976	1.07548421
chr10	123792910	123853343	60434	1	1	3	0.1218695	0.0079614	1	8	0.1218695	1.07548421
chr10	123853343	123887322	33980	2	1	3	0.1218695	0.00378107	1	9	0.1218695	1.33111237
chr10	123887322	124083158	195837	6	2	3	0.30102999	0.00378107	1	9	0.05404976	1.33111237
chr10	124083158	124162637	79480	2	2	3	0.30102999	0.0079614	1	8	0.05404976	1.07548421
chr10	124162637	124189949	27313	4	2	3	0.30102999	0.00378107	1	9	0.05404976	1.33111237
chr10	124189949	124500982	311034	5	2	3	0.30102999	0.0079614	1	8	0.05404976	1.07548421
chr10	124500982	124597326	96345	3	2	3	0.30102999	0.00378107	1	9	0.05404976	1.33111237
chr10	124597326	124622346	25021	2	2	3	0.30102999	0.0079614	1	8	0.05404976	1.07548421
chr10	124622346	124701182	78837	2	2	3	0.30102999	0.01598258	1	7	0.05404976	0.84395715
chr10	124701182	124701241	60	1	2	4	0.30102999	0.01077081	1	9	0.05404976	1.01542894
chr10	124701241	124917445	216205	5	2	4	0.30102999	0.03812622	1	7	0.05404976	0.60763643
chr10	124917445	124917504	60	1	2	4	0.30102999	0.01077081	1	9	0.05404976	1.01542894
chr10	124917504	125032104	114601	3	2	4	0.30102999	0.02074938	1	8	0.05404976	0.79906872
chr10	125032104	125121097	88994	2	2	4	0.30102999	0.00530919	1	10	0.05404976	1.2568129
chr10	125121097	125191734	70638	1	1	4	0.1218695	0.02074938	1	8	0.1218695	0.79906872
chr10	125191734	125249962	58229	1	1	4	0.1218695	0.03812622	1	7	0.1218695	0.60763643
chr10	125249962	125250021	60	1	2	4	0.30102999	0.03812622	1	7	0.05404976	0.60763643
chr10	125250021	125429413	179393	1	2	3	0.30102999	0.01598258	1	7	0.05404976	0.84395715
chr10	125429413	125436776	7364	1	2	2	0.30102999	0.00493743	1	7	0.05404976	1.16581773
chr10	125436776	125508873	72098	3	2	3	0.30102999	0.01598258	1	7	0.05404976	0.84395715
chr10	125508873	125573294	64422	2	3	3	0.51676182	0.0079614	1	8	0.02438896	1.07548421
chr10	125573294	125573353	60	1	3	3	0.51676182	0.00378107	1	9	0.02438896	1.33111237
chr10	125573353	125726005	152653	3	3	3	0.51676182	0.01598258	1	7	0.02438896	0.84395715
chr10	125726005	125834018	108014	2	3	2	0.51676182	0.00493743	1	7	0.02438896	1.16581773
chr10	125834018	126061025	227008	3	3	2	0.51676182	0.01053319	1	6	0.02438896	0.91219088
chr10	126061025	126105695	44671	3	3	2	0.51676182	0.00221948	1	8	0.02438896	1.44210395
chr10	126105695	126173860	68166	1	3	2	0.51676182	0.01053319	1	6	0.02438896	0.91219088
chr10	126173860	126434032	260173	6	2	2	0.30102999	0.01053319	1	6	0.05404976	0.91219088
chr10	126434032	126491087	57056	2	3	2	0.51676182	0.01053319	1	6	0.02438896	0.91219088
chr10	126491087	126517320	26234	1	2	2	0.30102999	0.01053319	1	6	0.05404976	0.91219088
chr10	126517320	126581894	64575	2	2	3	0.30102999	0.03070643	1	6	0.05404976	0.63695542
chr10	126581894	126581953	60	1	3	3	0.51676182	0.0079614	1	8	0.02438896	1.07548421
chr10	126581953	126631873	49921	1	2	3	0.30102999	0.0079614	1	8	0.05404976	1.07548421
chr10	126631873	126716425	84553	2	2	3	0.30102999	0.01598258	1	7	0.05404976	0.84395715
chr10	126716425	126809975	93551	2	1	3	0.1218695	0.01598258	1	7	0.1218695	0.84395715
chr10	126809975	126890486	80512	3	2	3	0.1575501	0.01598258	2	7	0.1575501	0.84395715
chr10	126890486	127018302	127817	1	2	3	0.1575501	0.03070643	2	6	0.1575501	0.63695542
chr10	127018302	127141474	123173	2	3	3	0.30102999	0.03070643	2	6	0.08289318	0.63695542
chr10	127141474	127141533	60	1	3	3	0.30102999	0.01598258	2	7	0.08289318	0.84395715
chr10	127141533	127458960	317428	7	2	3	0.1575501	0.01598258	2	7	0.1575501	0.84395715
chr10	127458960	127490711	31752	1	2	3	0.1575501	0.03070643	2	6	0.1575501	0.63695542
chr10	127490711	127496004	5294	1	2	2	0.1575501	0.01053319	2	6	0.1575501	0.91219088
chr10	127496004	127516186	20183	2	2	2	0.1575501	0.00493743	2	7	0.1575501	1.16581773
chr10	127516186	127622872	106687	5	2	2	0.1575501	0.01053319	2	6	0.1575501	0.91219088
chr10	127622872	127644842	21971	1	2	2	0.1575501	0.02162467	2	5	0.1575501	0.68214471
chr10	127644842	127668866	24025	2	2	2	0.1575501	0.01053319	2	6	0.1575501	0.91219088
chr10	127668866	127705569	36704	2	2	2	0.1575501	0.00493743	2	7	0.1575501	1.16581773
chr10	127705569	127856772	151204	3	2	1	0.1575501	0.00478973	2	5	0.1575501	1.02643191
chr10	127856772	128023515	166744	4	2	1	0.1575501	0.00204627	2	6	0.1575501	1.31360226
chr10	128023515	128068360	44846	2	2	2	0.1575501	0.01053319	2	6	0.1575501	0.91219088
chr10	128068360	128068419	60	1	2	2	0.1575501	0.00221948	2	8	0.1575501	1.44210395
chr10	128068419	128161518	93100	2	2	2	0.1575501	0.00493743	2	7	0.1575501	1.16581773
chr10	128161518	128330086	168569	4	2	2	0.1575501	0.00221948	2	8	0.1575501	1.44210395
chr10	128330086	128330145	60	1	2	2	0.1575501	9.54E-04	2	9	0.1575501	1.74076927
chr10	128330145	128526964	196820	3	2	2	0.1575501	0.00221948	2	8	0.1575501	1.44210395
chr10	128526964	128602886	75923	1	2	1	0.1575501	8.47E-04	2	7	0.1575501	1.62048027
chr10	128602886	128771757	168872	1	2	1	0.30102999	8.47E-04	1	7	0.05404976	1.62048027
chr10	128771757	128771816	60	1	2	3	0.30102999	0.00170589	1	10	0.05404976	1.61091002
chr10	128771816	128841378	69563	1	2	3	0.61140001	0.00378107	0	9	0	1.33111237
chr10	128841378	128916634	75257	3	2	3	0.61140001	0.00170589	0	10	0	1.61091002
chr10	128916634	128980591	63958	2	2	4	0.61140001	0.00247414	0	11	0	1.52371709
chr10	128980591	129056189	75599	1	2	3	0.61140001	7.28E-04	0	11	0	1.91540774
chr10	129056189	129100978	44790	2	2	3	0.61140001	2.93E-04	0	12	0	2.24559516
chr10	129100978	129137324	36347	1	2	2	0.61140001	1.52E-04	0	11	0	2.40668355
chr10	129137324	129298806	161483	3	2	2	0.61140001	3.90E-04	0	10	0	2.0620585
chr10	129298806	129539465	240660	2	2	2	0.61140001	9.54E-04	0	9	0	1.74076927
chr10	129539465	129741403	201939	5	2	4	0.61140001	0.00247414	0	11	0	1.52371709
chr10	129741403	129874917	133515	4	2	4	0.61140001	0.00108487	0	12	0	1.81706455

chr10	129874917	129874976	60	1	2	4	0.61140001	5.97E-05	0	15	0	2.87247834
chr10	129874976	129908735	33760	1	1	4	0.30102999	0.00247414	0	11	0	1.52371709
chr10	129908735	129994593	85859	1	1	4	0.30102999	0.00530919	0	10	0	1.2568129
chr10	129994593	130031754	37162	2	1	4	0.30102999	0.00247414	0	11	0	1.52371709
chr10	130031754	130187866	156113	1	0	3	0	7.28E-04	0	11	0	1.91540774
chr10	130187866	130187925	60	1	0	5	0	0.00317045	0	12	0	1.46403142
chr10	130187925	130315999	128075	1	0	4	0	0.00108487	0	12	0	1.81706455
chr10	130315999	130316058	60	1	0	4	0	4.45E-04	0	13	0	2.13824703
chr10	130316058	130513537	197480	1	0	4	0	0.00108487	0	12	0	1.81706455
chr10	130513537	130650432	136896	3	0	4	0	4.45E-04	0	13	0	2.13824703
chr10	130650432	130723155	72724	2	1	4	0.30102999	1.70E-04	0	14	0	2.48919369
chr10	130723155	130723214	60	1	1	5	0.30102999	5.83E-04	0	14	0	2.06733314
chr10	130723214	130811742	88529	1	1	4	0.30102999	1.70E-04	0	14	0	2.48919369
chr10	130811742	130862975	51234	2	1	5	0.30102999	5.83E-04	0	14	0	2.06733314
chr10	130862975	130977858	114884	1	1	4	0.30102999	0.00108487	0	12	0	1.81706455
chr10	130977858	130977917	60	1	1	5	0.1218695	0.00141024	1	13	0.1218695	1.7506523
chr10	130977917	131122317	144401	2	1	3	0.1218695	0.00378107	1	9	0.1218695	1.33111237
chr10	131122317	131122376	60	1	2	4	0.30102999	0.01077081	1	9	0.05404976	1.01542894
chr10	131122376	131197648	75273	1	1	4	0.1218695	0.01077081	1	9	0.1218695	1.01542894
chr10	131197648	131197707	60	1	1	4	0.1218695	0.00247414	1	11	0.1218695	1.52371709
chr10	131197707	131335418	137712	3	1	4	0.1218695	0.00530919	1	10	0.1218695	1.2568129
chr10	131335418	131335477	60	1	1	5	0.1218695	0.00666883	1	11	0.1218695	1.20557689
chr10	131335477	131434194	98718	3	1	3	0.1218695	0.00170589	1	10	0.1218695	1.61091002
chr10	131434194	131561314	127121	3	1	4	0.1218695	0.00108487	1	12	0.1218695	1.81706455
chr10	131561314	131643607	82294	3	2	5	0.30102999	0.00141024	1	13	0.05404976	1.7506523
chr10	131643607	131868882	225276	4	2	4	0.30102999	4.45E-04	1	13	0.05404976	2.13824703
chr10	131868882	131938654	69773	3	2	6	0.30102999	0.00373523	1	13	0.05404976	1.42397267
chr10	131938654	132019546	80893	2	2	5	0.30102999	0.00141024	1	13	0.05404976	1.7506523
chr10	132019546	132082758	63213	2	2	6	0.30102999	0.00373523	1	13	0.05404976	1.42397267
chr10	132082758	132082817	60	1	2	6	0.30102999	0.00166733	1	14	0.05404976	1.70763027
chr10	132082817	132190695	107879	2	2	4	0.61140001	1.70E-04	0	14	0	2.48919369
chr10	132190695	132263824	73130	2	2	5	0.61140001	5.83E-04	0	14	0	2.06733314
chr10	132263824	132365460	101637	1	2	5	0.61140001	0.00666883	0	11	0	1.20557689
chr10	132365460	132418039	52580	1	2	5	0.61140001	0.02473314	0	9	0	0.76806864
chr10	132418039	132468363	50325	2	2	5	0.61140001	0.01320236	0	10	0	0.97390707
chr10	132468363	132580655	112293	2	2	5	0.61140001	0.00666883	0	11	0	1.20557689
chr10	132580655	132680277	99623	1	2	4	0.61140001	0.01077081	0	9	0	1.01542894
chr10	132680277	132789730	109454	2	2	4	0.61140001	0.02074938	0	8	0	0.79906872
chr10	132789730	132789789	60	1	2	4	0.61140001	0.01077081	0	9	0	1.01542894
chr10	132789789	132853145	63357	1	2	4	0.61140001	0.03812622	0	7	0	0.60763643
chr10	132853145	132853204	60	1	2	4	0.61140001	0.02074938	0	8	0	0.79906872
chr10	132853204	132942572	89369	2	2	4	0.61140001	0.03812622	0	7	0	0.60763643
chr10	132942572	132994372	51801	1	1	4	0.30102999	0.03812622	0	7	0	0.60763643
chr10	132994372	133047449	53078	2	1	5	0.30102999	0.01320236	0	10	0	0.97390707
chr10	133047449	133047508	60	1	1	6	0.30102999	0.00778066	0	12	0	1.17038931
chr10	133047508	133155037	107530	2	1	6	0.30102999	0.01518174	0	11	0	0.9449689
chr10	133155037	133245771	90735	2	2	6	0.61140001	6.86E-04	0	15	0	2.02387299
chr10	133245771	133442209	196439	2	2	5	0.61140001	5.83E-04	0	14	0	2.06733314
chr10	133442209	133536778	94570	2	2	6	0.61140001	0.00166733	0	14	0	1.70763027
chr10	133536778	133536837	60	1	2	6	0.61140001	6.86E-04	0	15	0	2.02387299
chr10	133536837	133626790	89954	1	2	5	0.61140001	0.00141024	0	13	0	1.7506523
chr10	133626790	133626849	60	1	2	5	0.30102999	0.00141024	1	13	0.05404976	1.7506523
chr10	133626849	133749950	123102	1	2	5	0.61140001	0.00141024	0	13	0	1.7506523
chr10	133749950	133831041	81092	4	2	5	0.61140001	2.22E-04	0	15	0	2.41659593
chr10	133831041	133914951	83911	1	1	5	0.30102999	5.83E-04	0	14	0	2.06733314
chr10	133914951	133915010	60	1	1	5	0.30102999	2.22E-04	0	15	0	2.41659593
chr10	133915010	133945590	30581	1	1	5	0.30102999	5.83E-04	0	14	0	2.06733314
chr10	133945590	133945649	60	1	1	6	0.30102999	0.00166733	0	14	0	1.70763027
chr10	133945649	133996915	51267	1	1	5	0.30102999	0.00141024	0	13	0	1.7506523
chr10	133996915	134009001	12087	2	2	5	0.61140001	0.00141024	0	13	0	1.7506523
chr10	134009001	134079982	70982	1	1	5	0.30102999	0.00141024	0	13	0	1.7506523
chr10	134079982	134118807	38826	2	1	5	0.30102999	5.83E-04	0	14	0	2.06733314
chr10	134118807	134136680	17874	2	1	6	0.1218695	0.00166733	1	14	0.1218695	1.70763027
chr10	134136680	134177439	40760	1	1	6	0.1218695	0.00373523	1	13	0.1218695	1.42397267
chr10	134177439	134258887	81449	2	1	6	0.1218695	0.00166733	1	14	0.1218695	1.70763027
chr10	134258887	134339232	80346	2	1	6	0.1218695	6.86E-04	1	15	0.1218695	2.02387299
chr10	134339232	134372824	33593	2	1	7	0.1218695	0.00183109	1	15	0.1218695	1.68336308
chr10	134372824	134505182	132359	4	1	8	0.1218695	0.00188722	1	16	0.1218695	1.67551277
chr10	134505182	134704469	199288	5	1	7	0.1218695	7.41E-04	1	16	0.1218695	2.00316618
chr10	134704469	134753939	49471	1	1	7	0.1218695	0.00183109	1	15	0.1218695	1.68336308
chr10	134753939	134832661	78723	1	1	6	0.1218695	0.00166733	1	14	0.1218695	1.70763027
chr10	134832661	134924296	91636	3	1	6	0.1218695	6.86E-04	1	15	0.1218695	2.02387299
chr10	134924296	134996216	71921	1	1	4	0.1218695	0.00247414	1	11	0.1218695	1.52371709
chr10	134996216	135125545	129330	7	1	4	0.1218695	0.00530919	1	10	0.1218695	1.2568129
chr10	135125545	135194119	68575	3	1	3	0.1218695	0.00170589	1	10	0.1218695	1.61091002
chr10	135194119	135234843	40725	4	1	3	0.1218695	7.28E-04	1	11	0.1218695	1.91540774
chr10	135234843	135352372	117530	2	1	2	0.1218695	9.54E-04	1	9	0.1218695	1.74076927
chr10	135352372	135372492	20121	3	1	2	0.1218695	3.90E-04	1	10	0.1218695	2.0620585
chr10	135372492	135404523	32032	1	1	2	0.30102999	0.00221948	0	8	0	1.44210395
chr11	218365	284838	66474	2	1	1	0.1218695	0.01091641	1	4	0.1218695	0.76005302
chr11	284838	353347	68510	3	1	1	0.1218695	8.47E-04	1	7	0.1218695	1.62048027
chr11	353347	403846	50500	2	1	2	0.05404976	9.54E-04	2	9	0.30102999	1.74076927
chr11	403846	446813	42968	1	1	1	0.05404976	3.39E-04	2	8	0.30102999	1.94674965
chr11	446813	508019	61207	1	1	1	0.1218695	3.39E-04	1	8	0.1218695	1.94674965
chr11	508019	561995	53977	2	1	1	0.1218695	1.30E-04	1	9	0.1218695	2.29264207
chr11	561995	562040	46	1	1	2	0.1218695	9.54E-04	1	9	0.1218695	1.74076927
chr11	562040	594984	32945	1	1	1	0.1218695	1.30E-04	1	9	0.1218695	2.29264207
chr11	594984	770222	175239	4	1	1	0.05404976	1.30E-04	2	9	0.30102999	2.29264207
chr11	770222	770281	60	1	2	3	0.1575501	0.00378107	2	9	0.1575501	1.33111237
chr11	770281	872664	102384	2	1	3	0.05404976	0.00378107	2	9	0.30102999	1.33111237
chr11	872664	984637	111974	3	2	3	0.1575501	0.00378107	2	9	0.1575501	1.33111237
chr11	984637	984692	56	1	3	3	0.30102999	0.00378107	2	9	0.08289318	1.33111237
chr11	984692	1048786	64095	1	2	3	0.1575501	0.00378107	2	9	0.1575501	1.33111237
chr11	1048786	1076089	27304	2	2	4	0.1575501	0.01077081	2	9	0.1575501	1.01542894
chr11	1076089	1126866	50778	2	2	3	0.1575501	0.00378107	2	9	0.1575501	1.33111237
chr11	1126866	1222348	95483	1	2	2	0.1575501	9.54E-04	2	9	0.1575501	1.74076927
chr11	1222348	1262638	40291	2	2	2	0.1575501	1.52E-04	2	11	0.1575501	2.40668355
chr11	1262638	1262683	46	1	5	2	0.68214471	1.52E-04	2	11	0.02162467	2.40668355
chr11	1262683	1304929	42247	1	5	2	0.68214471	3.90E-04	2	10	0.02162467	2.0620585
chr11	1304929	1440798	135870	3	4	2	0.47744371	3.90E-04	2	10	0.0429175	2.0620585
chr11	1440798	1440857	60	1	4	3	0.47744371	0.00170589	2	10	0.0429175	1.61091002
chr11	1440857	1481727	40871	1	4	2	0.47744371	3.90E-04	2	10	0.0429175	2.0620585
chr11	1481727	1583707	101981	4	5	2	0.68214471	3.90E-04	2	10	0.02162467	2.0620585
chr11	1583707	1588271	4565	2	6	2	0.91219088	3.90E-04	2	10	0.01053319	2.0620585
chr11	1588271	1634221	45951	1	6	2	1.31360226	3.90E-04	1	10	0.00204627	2.0620585
chr11	1634221	1704417	70197	1	5	2	1.02643191	3.90E-04	1	10	0.00478973	2.0620585
chr11	1704417	1751372	46956	2	5	2	1.02643191	1.52E-04	1	11	0.00478973	2.40668355
chr11	1751372	1792331	40960	1	4	2	0.76005302	1.52E-04	1	11	0.01091641	2.40668355
chr11	1792331	1913233	120903	2	2	2	0.30102999	3.90E-04	1	10	0.05404976	2.0620585
chr11	1913233	1969895	56663	3	3	3	0.51676182	7.28E-04	1	11	0.02438896	1.91540774
chr11	1969895	1969949	55	1	3	3	0.30102999	7.28E-04	2	11	0.08289318	1.91540774
chr11	1969949	2014937	44989	1	2	3	0.1575501	7.28E-04	2	11	0.1575501	1.91540774
chr11	2014937	2045687	30751	1	2	3	0.30102999	7.28E-04	1	11	0.05404976	1.91540774
chr11	2045687	2086532	40846	1	1	3	0.1218695	7.28E-04	1	11	0.1218695	1.91540774
chr11	2086532	2140781	54250	2	2	3	0.30102999	7.28E-04	1	11	0.05404976	1.91540774
chr11	2140781	2140840	60	1	2	4	0.1575501	0.00247414	2	11	0.1575501	1.52371709
chr11	2140840	2153898	13059	1	2	4	0.30102999	0.00247414	1	11	0.05404976	1.52371709
chr11	2153898	2192153	38256	5	2	5	0.30102999	0.00666883	1	11	0.05404976	1.20557689
chr11	2192153	2222733	30581	1	1	5	0.1218695	0.00666883	1	11	0.1218695	1.20557689
chr11	2222733	2290020	67288	2	1	5	0.05404976	0.00666883	2	11	0.30102999	1.20557689
chr11	2290020	2290074	55	1	1	5	0.05404976	0.00317045	2	12	0.30102999	1.46403142
chr11	2290074	2326387	36314	1	1	4	0.05404976	0.00108487	2	12	0.30102999	1.81706455
chr11	2326387	2397123	70737	1	1	4	0.05404976	0.00247414	2	11	0.30102999	1.52371709
chr11	2397123	2397182	60	1	2	4	0.1575501	0.00247414	2	11	0.1575501	1.52371709

chr11	2397182	2425071	27890	1	1	4	0.1218695	0.00530919	1	10	0.1218695	1.2568129
chr11	2425071	2475746	50676	3	1	5	0.1218695	0.01320236	1	10	0.1218695	0.97390707
chr11	2475746	2475805	60	1	1	5	0.05404976	0.00141024	2	13	0.30102999	1.7506523
chr11	2475805	2542755	66951	1	1	5	0.05404976	0.00317045	2	12	0.30102999	1.46403142
chr11	2542755	2618221	75467	1	1	5	0.05404976	0.02473314	2	9	0.30102999	0.76806864
chr11	2618221	2674253	56033	1	1	5	0.1218695	0.02473314	1	9	0.1218695	0.76806864
chr11	2674253	2718566	44314	2	1	5	0.1218695	0.01320236	1	10	0.1218695	0.97390707
chr11	2718566	2718625	60	1	1	5	0.1218695	0.00317045	1	12	0.1218695	1.46403142
chr11	2718625	2861725	143101	2	1	4	0.1218695	0.00108487	1	12	0.1218695	1.81706455
chr11	2861725	2861784	60	1	1	4	0.05404976	4.45E-04	2	13	0.30102999	2.13824703
chr11	2861784	2904974	43191	1	1	4	0.05404976	0.00108487	2	12	0.30102999	1.81706455
chr11	2904974	2916747	11774	2	1	4	0.05404976	4.45E-04	2	13	0.30102999	2.13824703
chr11	2916747	2949663	32917	3	1	5	0.05404976	0.00141024	2	13	0.30102999	1.7506523
chr11	2949663	2985309	35647	2	1	5	0.05404976	5.83E-04	2	14	0.30102999	2.06733314
chr11	2985309	3039842	54534	2	1	5	0.1218695	5.83E-04	1	14	0.1218695	2.06733314
chr11	3039842	3039901	60	1	1	6	0.1218695	0.00166733	1	14	0.1218695	1.70763027
chr11	3039901	3073629	33729	1	1	6	0.1218695	0.00373523	1	13	0.1218695	1.42397267
chr11	3073629	3073688	60	1	1	6	0.05404976	0.00373523	2	13	0.30102999	1.42397267
chr11	3073688	3153394	79707	2	1	5	0.1218695	0.00141024	1	13	0.1218695	1.7506523
chr11	3153394	3153453	60	1	1	6	0.1218695	0.00373523	1	13	0.1218695	1.42397267
chr11	3153453	3202593	49141	1	1	5	0.1218695	0.00141024	1	13	0.1218695	1.7506523
chr11	3202593	3243558	40966	2	1	5	0.05404976	0.00141024	2	13	0.30102999	1.7506523
chr11	3243558	3381940	138383	2	1	6	0.05404976	0.00373523	2	13	0.30102999	1.42397267
chr11	3381940	3381999	60	1	1	7	0.05404976	0.00859896	2	13	0.30102999	1.14735594
chr11	3381999	3669161	287163	3	1	6	0.1218695	0.00373523	1	13	0.1218695	1.42397267
chr11	3669161	3692385	23225	1	1	6	0.30102999	0.00373523	0	13	0	1.42397267
chr11	3692385	3733447	41063	3	1	7	0.1218695	0.00859896	1	13	0.1218695	1.14735594
chr11	3733447	3793127	59681	3	1	7	0.05404976	0.00413023	2	14	0.30102999	1.39918918
chr11	3793127	3793185	59	1	1	8	0.05404976	0.00909834	2	14	0.30102999	1.13428373
chr11	3793185	3852615	59431	2	1	8	0.1218695	0.01767679	1	13	0.1218695	0.91308053
chr11	3852615	3886853	34239	2	1	7	0.1218695	0.00859896	1	13	0.1218695	1.14735594
chr11	3886853	3987618	100766	2	1	5	0.30102999	0.00141024	0	13	0	1.7506523
chr11	3987618	3987677	60	1	1	6	0.1218695	0.00373523	1	13	0.1218695	1.42397267
chr11	3987677	4061418	73742	1	1	5	0.30102999	0.00141024	0	13	0	1.7506523
chr11	4061418	4127246	65829	1	1	5	0.30102999	0.00317045	0	12	0	1.46403142
chr11	4127246	4175364	48119	2	1	6	0.30102999	0.00778066	0	12	0	1.17038931
chr11	4175364	4175423	60	1	1	7	0.30102999	0.01667721	0	12	0	0.92532268
chr11	4175423	4272812	97390	1	1	7	0.30102999	0.03037338	0	11	0	0.73110763
chr11	4272812	4406219	133408	2	1	7	0.30102999	0.01667721	0	12	0	0.92532268
chr11	4406219	4594629	188411	5	1	7	0.30102999	0.00413023	0	14	0	1.39918918
chr11	4594629	4596397	1769	2	1	7	0.30102999	0.00183109	0	15	0	1.68336308
chr11	4596397	5010854	414458	8	1	7	0.30102999	0.00413023	0	14	0	1.39918918
chr11	5010854	5081198	70345	3	1	7	0.30102999	0.00183109	0	15	0	1.68336308
chr11	5081198	5173297	92100	2	1	7	0.30102999	0.00413023	0	14	0	1.39918918
chr11	5173297	5392466	219170	9	1	7	0.30102999	0.00183109	0	15	0	1.68336308
chr11	5392466	5614439	221974	5	1	6	0.30102999	0.00166733	0	14	0	1.70763027
chr11	5614439	5655948	41510	4	1	7	0.30102999	0.00413023	0	14	0	1.39918918
chr11	5655948	5727098	71151	4	1	7	0.1218695	0.00413023	1	14	0.1218695	1.39918918
chr11	5727098	5773545	46448	2	1	7	0.1218695	0.00183109	1	15	0.1218695	1.68336308
chr11	5773545	5805665	32121	2	1	7	0.1218695	7.41E-04	1	16	0.1218695	2.00316618
chr11	5805665	6102019	296355	5	1	7	0.1218695	0.00183109	1	15	0.1218695	1.68336308
chr11	6102019	6102078	60	1	1	8	0.05404976	0.00433294	2	15	0.30102999	1.3873209
chr11	6102078	6232603	130526	5	1	7	0.05404976	0.00183109	2	15	0.30102999	1.68336308
chr11	6232603	6254886	22284	1	1	7	0.1218695	0.00183109	1	15	0.1218695	1.68336308
chr11	6254886	6282812	27927	1	1	7	0.1218695	0.00413023	1	14	0.1218695	1.39918918
chr11	6282812	6282871	60	1	1	7	0.05404976	0.00413023	2	14	0.30102999	1.39918918
chr11	6282871	6340301	57431	2	1	7	0.05404976	0.03037338	2	11	0.30102999	0.73110763
chr11	6340301	6457436	117136	5	1	7	0.05404976	0.01667721	2	12	0.30102999	0.92532268
chr11	6457436	6503772	46337	5	1	8	0.05404976	0.03209037	2	12	0.30102999	0.72109894
chr11	6503772	6592660	88889	2	1	7	0.05404976	0.01667721	2	12	0.30102999	0.92532268
chr11	6592660	6621239	28580	2	1	6	0.05404976	0.00778066	2	12	0.30102999	1.17038931
chr11	6621239	6639238	18000	4	1	6	0.05404976	0.01518174	2	11	0.30102999	0.9449689
chr11	6639238	6639297	60	1	1	6	0.05404976	0.00778066	2	12	0.30102999	1.17038931
chr11	6639297	6675974	36678	2	1	5	0.05404976	0.00317045	2	12	0.30102999	1.46403142
chr11	6675974	6676031	58	1	1	7	0.05404976	0.01667721	2	12	0.30102999	0.92532268
chr11	6676031	6703078	27048	1	1	6	0.05404976	0.00778066	2	12	0.30102999	1.17038931
chr11	6703078	6816185	113108	2	1	5	0.05404976	0.00317045	2	12	0.30102999	1.46403142
chr11	6816185	6920018	103834	3	1	3	0.05404976	2.93E-04	2	12	0.30102999	2.24559516
chr11	6920018	6920077	60	1	1	4	0.05404976	0.00108487	2	12	0.30102999	1.81706455
chr11	6920077	7040544	120468	3	1	3	0.05404976	7.28E-04	2	11	0.30102999	1.91540774
chr11	7040544	7040603	60	1	1	4	0.05404976	0.00108487	2	12	0.30102999	1.81706455
chr11	7040603	7091552	50950	2	1	3	0.05404976	7.28E-04	2	11	0.30102999	1.91540774
chr11	7091552	7160345	68794	3	1	3	0.05404976	2.93E-04	2	12	0.30102999	2.24559516
chr11	7160345	7278825	118481	2	1	3	0.05404976	7.28E-04	2	11	0.30102999	1.91540774
chr11	7278825	7327235	48411	2	1	3	0.05404976	2.93E-04	2	12	0.30102999	2.24559516
chr11	7327235	7353055	25821	1	1	3	0.05404976	7.28E-04	2	11	0.30102999	1.91540774
chr11	7353055	7378670	25616	2	1	4	0.05404976	0.00247414	2	11	0.30102999	1.52371709
chr11	7378670	7447256	68587	2	1	4	0.05404976	0.00108487	2	12	0.30102999	1.81706455
chr11	7447256	7465744	18489	2	1	5	0.05404976	0.00317045	2	12	0.30102999	1.46403142
chr11	7465744	7575495	109752	2	1	5	0.05404976	0.00666883	2	11	0.30102999	1.20557689
chr11	7575495	7575553	59	1	1	5	0.05404976	0.00317045	2	12	0.30102999	1.46403142
chr11	7575553	7616545	40993	1	1	5	0.05404976	0.00666883	2	11	0.30102999	1.20557689
chr11	7616545	7634854	18310	1	1	5	0.05404976	0.01320236	2	10	0.30102999	0.97390707
chr11	7634854	7660068	25215	1	1	5	0.05404976	0.02473314	2	9	0.30102999	0.76806864
chr11	7660068	7712477	52410	2	1	4	0.05404976	0.01077081	2	9	0.30102999	1.01542894
chr11	7712477	7931094	218618	7	1	5	0.05404976	0.02473314	2	9	0.30102999	0.76806864
chr11	7931094	7981483	50390	1	1	5	0.05404976	0.04407651	2	8	0.30102999	0.58747015
chr11	7981483	7981540	58	1	1	5	0.05404976	0.02473314	2	9	0.30102999	0.76806864
chr11	7981540	8016220	34681	1	1	4	0.05404976	0.02074938	2	8	0.30102999	0.79906872
chr11	8016220	8075884	59665	1	1	3	0.05404976	0.0079614	2	8	0.30102999	1.07548421
chr11	8075884	8161528	85645	4	1	3	0.05404976	0.00378107	2	9	0.30102999	1.33111237
chr11	8161528	8257457	95930	3	1	3	0.05404976	0.00170589	2	10	0.30102999	1.61091002
chr11	8257457	8271479	14023	2	1	3	0.05404976	7.28E-04	2	11	0.30102999	1.91540774
chr11	8271479	8338096	66618	1	1	3	0.05404976	0.00170589	2	10	0.30102999	1.61091002
chr11	8338096	8497255	159160	4	1	2	0.05404976	9.54E-04	2	9	0.30102999	1.74076927
chr11	8497255	8655502	158248	5	1	1	0.05404976	1.30E-04	2	9	0.30102999	2.29264207
chr11	8655502	9152612	497111	19	1	2	0.05404976	9.54E-04	2	9	0.30102999	1.74076927
chr11	9152612	9177252	24641	2	1	2	0.05404976	3.90E-04	2	10	0.30102999	2.0620585
chr11	9177252	9200052	22801	1	1	2	0.05404976	9.54E-04	2	9	0.30102999	1.74076927
chr11	9200052	9200111	60	1	1	3	0.05404976	0.00378107	2	9	0.30102999	1.33111237
chr11	9200111	9263840	63730	2	1	3	0.05404976	0.0079614	2	8	0.30102999	1.07548421
chr11	9263840	9407239	143400	3	1	3	0.05404976	0.00170589	2	10	0.30102999	1.61091002
chr11	9407239	9407298	60	1	2	3	0.1575501	0.00170589	2	10	0.1575501	1.61091002
chr11	9407298	9509835	102538	2	2	3	0.1575501	0.0079614	2	8	0.1575501	1.07548421
chr11	9509835	9549484	39650	2	2	3	0.1575501	7.28E-04	2	11	0.1575501	1.91540774
chr11	9549484	9610970	61487	3	2	3	0.1575501	0.00170589	2	10	0.1575501	1.61091002
chr11	9610970	9686182	75213	2	2	3	0.1575501	0.0079614	2	8	0.1575501	1.07548421
chr11	9686182	9686227	46	1	2	3	0.1575501	0.00378107	2	9	0.1575501	1.33111237
chr11	9686227	9762304	76078	2	2	2	0.1575501	0.00221948	2	8	0.1575501	1.44210395
chr11	9762304	9773372	11069	2	2	2	0.1575501	9.54E-04	2	9	0.1575501	1.74076927
chr11	9773372	9860452	87081	3	2	3	0.1575501	0.00378107	2	9	0.1575501	1.33111237
chr11	9860452	9975394	114943	4	2	4	0.1575501	0.01077081	2	9	0.1575501	1.01542894
chr11	9975394	9975453	60	1	2	4	0.1575501	0.00530919	2	10	0.1575501	1.2568129
chr11	9975453	10231123	255671	6	2	3	0.1575501	0.00170589	2	10	0.1575501	1.61091002
chr11	10231123	10301946	70824	1	2	2	0.1575501	0.00221948	2	8	0.1575501	1.44210395
chr11	10301946	10466758	164813	3	2	2	0.1575501	0.00493743	2	7	0.1575501	1.16581773
chr11	10466758	10508882	42125	3	2	2	0.1575501	3.90E-04	2	10	0.1575501	2.0620585
chr11	10508882	10584088	75207	2	2	2	0.1575501	9.54E-04	2	9	0.1575501	1.74076927
chr11	10584088	10647993	63906	2	2	1	0.1575501	8.47E-04	2	7	0.1575501	1.62048027
chr11	10647993	10749516	101524	4	2	1	0.1575501	3.39E-04	2	8	0.1575501	1.94674965
chr11	10749516	10785341	35826	2	2	1	0.1575501	1.30E-04	2	9	0.1575501	2.29264207
chr11	10785341	10785400	60	1	2	2	0.1575501	9.54E-04	2	9	0.1575501	1.74076927
chr11	10785400	11006203	220804	6	2	2	0.30102999	0.00221948	1	8	0.05404976	1.44210395
chr11	11006203	11094778	88576	2	2	3	0.30102999	0.00170589	1	10	0.05404976	1.61091002

chr11	11094778	11094837	60	1	2	3	0.1575501	0.00170589	2	10	0.1575501	1.61091002
chr11	11094837	11310311	215475	2	2	2	0.30102999	9.54E-04	1	9	0.05404976	1.74076927
chr11	11310311	11368811	58501	2	2	2	0.30102999	3.90E-04	1	10	0.05404976	2.0620585
chr11	11368811	11395953	27143	2	2	4	0.30102999	0.00530919	1	10	0.05404976	1.2568129
chr11	11395953	11521697	125745	3	2	5	0.30102999	0.01320236	1	10	0.05404976	0.97390707
chr11	11521697	11614411	92715	2	2	4	0.30102999	0.01077081	1	9	0.05404976	1.01542894
chr11	11614411	11906064	291654	3	2	3	0.30102999	0.00378107	1	9	0.05404976	1.33111237
chr11	11906064	11924291	18228	2	2	4	0.30102999	0.01077081	1	9	0.05404976	1.01542894
chr11	11924291	11978280	53990	4	2	4	0.30102999	0.00530919	1	10	0.05404976	1.2568129
chr11	11978280	11984479	6200	1	2	4	0.30102999	0.02074938	1	8	0.05404976	0.79906872
chr11	11984479	11985627	1149	2	2	5	0.30102999	0.04407651	1	8	0.05404976	0.58747015
chr11	11985627	12051354	65728	2	2	4	0.30102999	0.02074938	1	8	0.05404976	0.79906872
chr11	12051354	12051413	60	1	2	5	0.30102999	0.04407651	1	8	0.05404976	0.58747015
chr11	12051413	12143390	91978	1	2	4	0.30102999	0.02074938	1	8	0.05404976	0.79906872
chr11	12143390	12229467	86078	5	2	4	0.30102999	0.01077081	1	9	0.05404976	1.01542894
chr11	12229467	12316138	86672	5	2	5	0.30102999	0.02473314	1	9	0.05404976	0.76806864
chr11	12316138	12363908	47771	3	2	7	0.30102999	0.08584816	1	9	0.05404976	0.42015402
chr11	12363908	12452212	88305	4	2	7	0.30102999	0.05232577	1	10	0.05404976	0.56314362
chr11	12452212	12551725	99514	2	2	6	0.30102999	0.02793176	1	10	0.05404976	0.74627054
chr11	12551725	12800688	248964	4	2	7	0.30102999	0.05232577	1	10	0.05404976	0.56314362
chr11	12800688	12848899	48212	2	2	7	0.1575501	0.05232577	2	10	0.1575501	0.56314362
chr11	12848899	12896091	47193	1	2	7	0.1575501	0.08584816	2	9	0.1575501	0.42015402
chr11	12896091	12923577	27487	1	2	6	0.30102999	0.04875589	1	9	0.05404976	0.5732208
chr11	12923577	12983369	59793	1	2	5	0.30102999	0.02473314	1	9	0.05404976	0.76806864
chr11	12983369	12983428	60	1	2	5	0.30102999	0.01320236	1	10	0.05404976	0.97390707
chr11	12983428	13226395	242968	2	2	5	0.30102999	0.04407651	1	8	0.05404976	0.58747015
chr11	13226395	13226454	60	1	2	5	0.30102999	0.02473314	1	9	0.05404976	0.76806864
chr11	13226454	13331564	105111	3	2	3	0.61140001	0.00378107	0	9	0	1.33111237
chr11	13331564	13351481	19918	1	2	3	0.61140001	0.03070643	0	6	0	0.63695542
chr11	13351481	13371506	20026	2	2	3	0.30102999	0.03070643	1	6	0.05404976	0.63695542
chr11	13371506	13412886	41381	2	2	3	0.1575501	0.03070643	2	6	0.1575501	0.63695542
chr11	13412886	13412945	60	1	2	3	0.1575501	0.01598258	2	7	0.1575501	0.84395715
chr11	13412945	13484163	71219	2	2	3	0.1575501	0.03070643	2	6	0.1575501	0.63695542
chr11	13484163	13514371	30209	2	2	3	0.1575501	0.01598258	2	7	0.1575501	0.84395715
chr11	13514371	13514430	60	1	2	4	0.1575501	0.02074938	2	8	0.1575501	0.79906872
chr11	13514430	13666963	152534	2	2	3	0.1575501	0.0079614	2	8	0.1575501	1.07548421
chr11	13666963	13705717	38755	3	2	4	0.1575501	0.02074938	2	8	0.1575501	0.79906872
chr11	13705717	13952761	247045	4	2	3	0.1575501	0.0079614	2	8	0.1575501	1.07548421
chr11	13952761	13991832	39072	1	2	3	0.30102999	0.0079614	1	8	0.05404976	1.07548421
chr11	13991832	14034589	42758	2	2	3	0.30102999	0.00378107	1	9	0.05404976	1.33111237
chr11	14034589	14034648	60	1	2	3	0.30102999	0.00170589	1	10	0.05404976	1.61091002
chr11	14034648	14157083	122436	2	2	3	0.30102999	0.0079614	1	8	0.05404976	1.07548421
chr11	14157083	14224682	67600	2	2	3	0.30102999	0.00378107	1	9	0.05404976	1.33111237
chr11	14224682	14224741	60	1	2	3	0.30102999	0.00170589	1	10	0.05404976	1.61091002
chr11	14224741	14362224	137484	4	2	3	0.30102999	0.00378107	1	9	0.05404976	1.33111237
chr11	14362224	14362283	60	1	2	3	0.1575501	0.00378107	2	9	0.1575501	1.33111237
chr11	14362283	14379102	16820	1	2	3	0.1575501	0.0079614	2	8	0.1575501	1.07548421
chr11	14379102	14512134	133033	2	2	3	0.30102999	0.0079614	1	8	0.05404976	1.07548421
chr11	14512134	14512193	60	1	2	3	0.30102999	0.00378107	1	9	0.05404976	1.33111237
chr11	14512193	14570871	58679	2	2	3	0.61140001	0.0079614	0	8	0	1.07548421
chr11	14570871	14659210	88340	4	2	3	0.61140001	0.00378107	0	9	0	1.33111237
chr11	14659210	14709082	49873	1	2	3	0.61140001	0.01598258	0	7	0	0.84395715
chr11	14709082	14768905	59824	2	2	3	0.30102999	0.01598258	1	7	0.05404976	0.84395715
chr11	14768905	14853301	84397	3	2	3	0.30102999	0.00378107	1	9	0.05404976	1.33111237
chr11	14853301	14988973	135673	3	2	3	0.61140001	0.0079614	0	8	0	1.07548421
chr11	14988973	14991115	2143	2	2	4	0.61140001	0.02074938	0	8	0	0.79906872
chr11	14991115	15028697	37583	2	2	2	0.61140001	0.00221948	0	8	0	1.44210395
chr11	15028697	15100083	71387	2	2	3	0.61140001	0.0079614	0	8	0	1.07548421
chr11	15100083	15213597	113515	3	2	4	0.30102999	0.02074938	1	8	0.05404976	0.79906872
chr11	15213597	15261726	48130	1	2	3	0.30102999	0.0079614	1	8	0.05404976	1.07548421
chr11	15261726	15261785	60	1	2	3	0.1575501	0.0079614	2	8	0.1575501	1.07548421
chr11	15261785	15406551	144767	1	2	2	0.30102999	0.00221948	1	8	0.05404976	1.44210395
chr11	15406551	15406610	60	1	2	3	0.30102999	0.0079614	1	8	0.05404976	1.07548421
chr11	15406610	15667251	260642	1	2	1	0.30102999	3.39E-04	1	8	0.05404976	1.94674965
chr11	15667251	15781823	114573	1	2	1	0.30102999	8.47E-04	1	7	0.05404976	1.62048027
chr11	15781823	15781882	60	1	2	2	0.30102999	0.00493743	1	7	0.05404976	1.16581773
chr11	15781882	15995789	213908	1	2	0	0.30102999	0	1	4	0.05404976	1.26272838
chr11	15995789	16027746	31958	2	2	0	0.1575501	0	2	4	0.1575501	1.26272838
chr11	16027746	16027805	60	1	2	1	0.1575501	0.00204627	2	6	0.1575501	1.31360226
chr11	16027805	16122782	94978	2	2	0	0.1575501	0	2	5	0.1575501	1.60515106
chr11	16122782	16122841	60	1	2	1	0.1575501	0.00478973	2	5	0.1575501	1.02643191
chr11	16122841	16302150	179310	4	2	0	0.1575501	0	2	4	0.1575501	1.26272838
chr11	16302150	16502568	200419	5	3	0	0.30102999	0	2	4	0.08289318	1.26272838
chr11	16502568	16593589	91022	2	3	1	0.30102999	0.01091641	2	4	0.08289318	0.76005302
chr11	16593589	16593648	60	1	3	1	0.30102999	0.00204627	2	6	0.08289318	1.31360226
chr11	16593648	16922315	328668	6	3	1	0.30102999	0.00478973	2	5	0.08289318	1.02643191
chr11	16922315	16922374	60	1	3	2	0.30102999	0.02162467	2	5	0.08289318	0.68214471
chr11	16922374	17001335	78962	2	3	1	0.30102999	0.00478973	2	5	0.08289318	1.02643191
chr11	17001335	17124338	123004	4	3	2	0.30102999	0.02162467	2	5	0.08289318	0.68214471
chr11	17124338	17144330	19993	2	3	2	0.17593012	0.02162467	3	5	0.17593012	0.68214471
chr11	17144330	17190929	46600	1	3	2	0.30102999	0.02162467	2	5	0.08289318	0.68214471
chr11	17190929	17223356	32428	2	3	2	0.30102999	0.01053319	2	6	0.08289318	0.91219088
chr11	17223356	17223415	60	1	3	2	0.17593012	0.01053319	3	6	0.17593012	0.91219088
chr11	17223415	17270775	47361	1	3	2	0.17593012	0.02162467	3	5	0.17593012	0.68214471
chr11	17270775	17270834	60	1	3	2	0.17593012	0.01053319	3	6	0.17593012	0.91219088
chr11	17270834	17306149	35316	1	3	2	0.30102999	0.01053319	2	6	0.08289318	0.91219088
chr11	17306149	17352436	46288	3	3	2	0.30102999	0.00493743	2	7	0.08289318	1.16581773
chr11	17352436	17352494	59	1	3	2	0.17593012	0.00493743	3	7	0.17593012	1.16581773
chr11	17352494	17414466	61973	2	3	2	0.30102999	0.00493743	2	7	0.08289318	1.16581773
chr11	17414466	17495767	81302	2	3	2	0.30102999	0.01053319	2	6	0.08289318	0.91219088
chr11	17495767	17495826	60	1	4	2	0.47744371	0.00221948	2	8	0.0429175	1.44210395
chr11	17495826	17515475	19650	1	4	2	0.47744371	0.00493743	2	7	0.0429175	1.16581773
chr11	17515475	17623300	107826	5	4	2	0.30102999	0.00221948	3	8	0.10122019	1.44210395
chr11	17623300	17794177	170878	5	4	2	0.30102999	9.54E-04	3	9	0.10122019	1.74076927
chr11	17794177	17853233	59057	1	4	2	0.30102999	0.00221948	3	8	0.10122019	1.44210395
chr11	17853233	17853292	60	1	4	2	0.18734596	0.00221948	4	8	0.18734596	1.44210395
chr11	17853292	17899801	46510	1	3	2	0.10122019	0.00221948	4	8	0.30102999	1.44210395
chr11	17899801	17981145	81345	2	3	2	0.17593012	0.00221948	3	8	0.17593012	1.44210395
chr11	17981145	18073860	92716	4	3	2	0.17593012	9.54E-04	3	9	0.17593012	1.74076927
chr11	18073860	18169103	95244	2	3	2	0.17593012	0.00493743	3	7	0.17593012	1.16581773
chr11	18169103	18258237	89135	3	3	4	0.17593012	0.01077081	3	9	0.17593012	1.01542894
chr11	18258237	18266880	8644	2	3	4	0.17593012	0.00530919	3	10	0.17593012	1.2568129
chr11	18266880	18309469	42590	2	3	3	0.17593012	0.00378107	3	9	0.17593012	1.33111237
chr11	18309469	18327845	18377	1	3	3	0.51676182	0.00378107	1	9	0.02438896	1.33111237
chr11	18327845	18380207	52363	3	3	4	0.51676182	0.01077081	1	9	0.02438896	1.01542894
chr11	18380207	18428636	48430	1	3	4	0.93173516	0.01077081	0	9	0	1.01542894
chr11	18428636	18428695	60	1	3	4	0.51676182	0.01077081	1	9	0.02438896	1.01542894
chr11	18428695	18503356	74662	3	3	4	0.93173516	0.01077081	0	9	0	1.01542894
chr11	18503356	18536283	32928	3	3	4	0.51676182	0.01077081	1	9	0.02438896	1.01542894
chr11	18536283	18726087	189805	8	3	3	0.51676182	0.00378107	1	9	0.02438896	1.33111237
chr11	18726087	18726133	47	1	3	3	0.51676182	0.0079614	1	8	0.02438896	1.07548421
chr11	18726133	18788855	62723	1	3	2	0.51676182	0.00221948	1	8	0.02438896	1.44210395
chr11	18788855	18886425	97571	2	3	2	0.51676182	9.54E-04	1	9	0.02438896	1.74076927
chr11	18886425	18886484	60	1	3	2	0.17593012	9.54E-04	3	9	0.17593012	1.74076927
chr11	18886484	18952024	65541	1	3	2	0.30102999	0.00221948	2	8	0.08289318	1.44210395
chr11	18952024	19076609	124586	1	3	2	0.30102999	0.00493743	2	7	0.08289318	1.16581773
chr11	19076609	19076668	60	1	3	2	0.17593012	0.00493743	3	7	0.17593012	1.16581773
chr11	19076668	19119951	43284	1	3	2	0.30102999	0.00493743	2	7	0.08289318	1.16581773
chr11	19119951	19168758	48808	2	3	2	0.30102999	0.01053319	2	6	0.08289318	0.91219088
chr11	19168758	19251054	82297	3	3	2	0.17593012	0.00221948	3	8	0.17593012	1.44210395
chr11	19251054	19318040	66987	1	3	2	0.17593012	0.00493743	3	7	0.17593012	1.16581773
chr11	19318040	19376615	58576	1	3	2	0.30102999	0.01053319	2	6	0.08289318	0.91219088
chr11	19376615	19430585	53971	2	3	2	0.30102999	0.00493743	2	7	0.08289318	1.16581773
chr11	19430585	19430644	60	1	3	2	0.30102999	0.00221948	2	8	0.08289318	1.44210395

chr11	19430644	19458752	28109	1	3	1	0.30102999	8.47E-04	2	7	0.08289318	1.62048027
chr11	19458752	19458811	60	1	3	1	0.17593012	8.47E-04	3	7	0.17593012	1.62048027
chr11	19458811	19514209	55399	1	3	1	0.30102999	8.47E-04	2	7	0.08289318	1.62048027
chr11	19514209	19591508	77300	2	3	1	0.30102999	3.39E-04	2	8	0.08289318	1.94674965
chr11	19591508	19664553	73046	1	3	1	0.30102999	8.47E-04	2	7	0.08289318	1.62048027
chr11	19664553	19739690	75138	1	2	1	0.30102999	8.47E-04	1	7	0.05404976	1.62048027
chr11	19739690	19739749	60	1	2	2	0.30102999	0.00221948	1	8	0.05404976	1.44210395
chr11	19739749	19793641	53893	1	2	1	0.30102999	3.39E-04	1	8	0.05404976	1.94674965
chr11	19793641	19825909	32269	1	2	1	0.30102999	8.47E-04	1	7	0.05404976	1.62048027
chr11	19825909	19825968	60	1	3	2	0.30102999	0.00493743	2	7	0.08289318	1.16581773
chr11	19825968	19883334	57367	1	3	2	0.51676182	0.00493743	1	7	0.02438896	1.16581773
chr11	19883334	19905869	22536	2	3	2	0.51676182	0.00221948	1	8	0.02438896	1.44210395
chr11	19905869	19965281	59413	2	3	2	0.51676182	9.54E-04	1	9	0.02438896	1.74076927
chr11	19965281	19965340	60	1	3	2	0.30102999	9.54E-04	2	9	0.08289318	1.74076927
chr11	19965340	20083899	118560	2	3	2	0.51676182	9.54E-04	1	9	0.02438896	1.74076927
chr11	20083899	20141561	57663	1	3	2	0.51676182	0.00221948	1	8	0.02438896	1.44210395
chr11	20141561	20218948	77388	2	4	2	0.76005302	0.00221948	1	8	0.01091641	1.44210395
chr11	20218948	20219007	60	1	4	2	0.47744371	9.54E-04	2	9	0.0429175	1.74076927
chr11	20219007	20439739	220733	4	3	2	0.30102999	9.54E-04	2	9	0.08289318	1.74076927
chr11	20439739	20483972	44234	2	3	2	0.17593012	9.54E-04	3	9	0.17593012	1.74076927
chr11	20483972	20620997	137026	2	3	2	0.17593012	0.00221948	3	8	0.17593012	1.44210395
chr11	20620997	20621056	60	1	3	2	0.10122019	0.00221948	4	8	0.30102999	1.44210395
chr11	20621056	20668383	47328	1	3	2	0.17593012	0.00221948	3	8	0.17593012	1.44210395
chr11	20668383	20804865	136483	4	3	2	0.17593012	9.54E-04	3	9	0.17593012	1.74076927
chr11	20804865	21020109	215245	4	3	2	0.30102999	9.54E-04	2	9	0.08289318	1.74076927
chr11	21020109	21099960	79852	2	3	2	0.51676182	9.54E-04	1	9	0.02438896	1.74076927
chr11	21099960	21210098	110139	2	3	2	0.51676182	0.00221948	1	8	0.02438896	1.44210395
chr11	21210098	21272754	62657	2	3	2	0.51676182	0.00493743	1	7	0.02438896	1.16581773
chr11	21272754	21366340	93587	3	3	2	0.51676182	0.00221948	1	8	0.02438896	1.44210395
chr11	21366340	21366399	60	1	3	2	0.51676182	9.54E-04	1	9	0.02438896	1.74076927
chr11	21366399	21464425	98027	2	3	2	0.51676182	0.00221948	1	8	0.02438896	1.44210395
chr11	21464425	21503026	38602	1	2	2	0.30102999	0.01053319	1	6	0.05404976	0.91219088
chr11	21503026	21503085	60	1	2	2	0.1575501	0.01053319	2	6	0.1575501	0.91219088
chr11	21503085	21549682	46598	1	2	2	0.1575501	0.02162467	2	5	0.1575501	0.68214471
chr11	21549682	21596843	47162	2	2	2	0.1575501	0.01053319	2	6	0.1575501	0.91219088
chr11	21596843	21859560	262718	2	2	2	0.30102999	0.01053319	1	6	0.05404976	0.91219088
chr11	21859560	21859619	60	1	2	2	0.08289318	0.00493743	3	7	0.30102999	1.16581773
chr11	21859619	22137207	277589	1	2	2	0.1575501	0.00493743	2	7	0.1575501	1.16581773
chr11	22137207	22301894	164688	2	2	1	0.30102999	0.00478973	1	5	0.05404976	1.02643191
chr11	22301894	22399498	97605	3	2	1	0.30102999	0.01091641	1	4	0.05404976	0.76005302
chr11	22399498	22476574	77077	1	1	1	0.1218695	0.02438896	1	3	0.1218695	0.51676182
chr11	22476574	22644572	167999	1	1	1	0.1218695	0.05404976	1	2	0.1218695	0.30102999
chr11	22644572	22833595	189024	8	1	1	0.1218695	0.02438896	1	3	0.1218695	0.51676182
chr11	22833595	23160039	326445	3	1	1	0.1218695	0.01091641	1	4	0.1218695	0.76005302
chr11	23160039	23445008	284970	1	1	1	0.1218695	0.02438896	1	3	0.1218695	0.51676182
chr11	23445008	23445067	60	1	1	1	0.1218695	0.01091641	1	4	0.1218695	0.76005302
chr11	23445067	23710376	265310	1	1	1	0.1218695	0.02438896	1	3	0.1218695	0.51676182
chr11	23710376	23710435	60	1	2	1	0.30102999	0.02438896	1	3	0.05404976	0.51676182
chr11	23710435	24063998	353564	3	1	1	0.1218695	0.02438896	1	3	0.1218695	0.51676182
chr11	24063998	24280229	216232	2	1	1	0.1218695	0.01091641	1	4	0.1218695	0.76005302
chr11	24280229	24280288	60	1	2	1	0.30102999	0.01091641	1	4	0.05404976	0.76005302
chr11	24280288	24476279	195992	2	2	1	0.30102999	0.02438896	1	3	0.05404976	0.51676182
chr11	24476279	24522092	45814	2	2	1	0.1575501	0.01091641	2	4	0.1575501	0.76005302
chr11	24522092	24671685	149594	3	2	0	0.1575501	0	2	4	0.1575501	1.26272838
chr11	24671685	24847837	176153	4	2	0	0.1575501	0	2	3	0.1575501	0.93173516
chr11	24847837	25069132	221296	5	2	0	0.30102999	0	1	3	0.05404976	0.93173516
chr11	25069132	25069191	60	1	2	0	0.30102999	0	1	4	0.05404976	1.26272838
chr11	25069191	25129984	60794	1	2	0	0.30102999	0	1	3	0.05404976	0.93173516
chr11	25129984	25380076	250093	1	1	0	0.1218695	0	1	3	0.1218695	0.93173516
chr11	25380076	25552296	172221	1	1	0	0.1218695	0	1	2	0.1218695	0.61140001
chr11	25552296	25677598	125303	1	1	0	0.1218695	0	1	1	0.1218695	0.30102999
chr11	25677598	25887995	210398	2	1	1	0.1218695	0.1218695	1	1	0.1218695	0.1218695
chr11	25887995	25888054	60	1	1	2	0.1218695	0.0429175	1	4	0.1218695	0.47744371
chr11	25888054	26118285	230232	1	1	1	0.1218695	0.02438896	1	3	0.1218695	0.51676182
chr11	26118285	26118344	60	1	1	2	0.1218695	0.08289318	1	3	0.1218695	0.30102999
chr11	26118344	26288841	170498	1	1	0	0.1218695	0	1	3	0.1218695	0.93173516
chr11	26288841	26390509	101669	3	1	0	0.05404976	0	2	3	0.30102999	0.93173516
chr11	26390509	26484665	94157	3	2	4	0.1575501	0.18734596	2	4	0.1575501	0.18734596
chr11	26484665	26556063	71399	1	2	3	0.1575501	0.17593012	2	3	0.1575501	0.17593012
chr11	26556063	26583144	27082	2	2	3	0.1575501	0.10122019	2	4	0.1575501	0.30102999
chr11	26583144	26621222	38079	1	2	2	0.1575501	0.0429175	2	4	0.1575501	0.47744371
chr11	26621222	26720962	99741	2	2	2	0.1575501	0.08289318	2	3	0.1575501	0.30102999
chr11	26720962	26834415	113454	4	2	2	0.1575501	0.0429175	2	4	0.1575501	0.47744371
chr11	26834415	27077147	242733	2	2	1	0.1575501	0.01091641	2	4	0.1575501	0.76005302
chr11	27077147	27077206	60	1	3	3	0.30102999	0.10122019	2	4	0.08289318	0.30102999
chr11	27077206	27129357	52152	1	3	2	0.30102999	0.0429175	2	4	0.08289318	0.47744371
chr11	27129357	27149226	19870	2	4	2	0.18734596	0.0429175	4	4	0.18734596	0.47744371
chr11	27149226	27526576	377351	12	4	2	0.30102999	0.0429175	3	4	0.10122019	0.47744371
chr11	27526576	28104454	577879	11	4	1	0.30102999	0.01091641	3	4	0.10122019	0.76005302
chr11	28104454	28131157	26704	1	3	1	0.17593012	0.02438896	3	3	0.17593012	0.51676182
chr11	28131157	28154111	22955	1	3	1	0.17593012	0.05404976	3	2	0.17593012	0.30102999
chr11	28154111	28154170	60	1	4	1	0.30102999	0.05404976	3	2	0.10122019	0.30102999
chr11	28154170	28197596	43427	1	3	1	0.30102999	0.05404976	2	2	0.08289318	0.30102999
chr11	28197596	28197655	60	1	3	1	0.30102999	0.02438896	2	3	0.08289318	0.51676182
chr11	28197655	28254297	56643	1	3	1	0.30102999	0.05404976	2	2	0.08289318	0.30102999
chr11	28254297	28291889	37593	2	4	1	0.47744371	0.05404976	2	2	0.0429175	0.30102999
chr11	28291889	28349712	57824	3	4	1	0.47744371	0.02438896	2	3	0.0429175	0.51676182
chr11	28349712	28464405	114694	1	3	1	0.51676182	0.02438896	1	3	0.02438896	0.51676182
chr11	28464405	28728052	263648	1	3	1	0.51676182	0.1218695	1	1	0.02438896	0.1218695
chr11	28728052	28728111	60	1	4	1	0.76005302	0.05404976	1	2	0.01091641	0.30102999
chr11	28728111	29062145	334035	2	4	1	0.76005302	0.1218695	1	1	0.01091641	0.1218695
chr11	29062145	29062204	60	1	4	1	0.76005302	0.05404976	1	2	0.01091641	0.30102999
chr11	29062204	29195161	132958	1	2	1	0.30102999	0.05404976	1	2	0.05404976	0.30102999
chr11	29195161	29265863	70703	2	2	1	0.30102999	0.02438896	1	3	0.05404976	0.51676182
chr11	29265863	29404733	138871	2	3	1	0.51676182	0.02438896	1	3	0.02438896	0.51676182
chr11	29404733	29404792	60	1	4	1	0.47744371	0.01091641	2	4	0.0429175	0.76005302
chr11	29404792	29628642	223851	2	3	1	0.30102999	0.02438896	2	3	0.08289318	0.51676182
chr11	29628642	29704738	76097	2	3	1	0.30102999	0.01091641	2	4	0.08289318	0.76005302
chr11	29704738	29772632	67895	1	3	1	0.51676182	0.02438896	1	3	0.02438896	0.51676182
chr11	29772632	29979781	207150	1	3	1	0.51676182	0.05404976	1	2	0.02438896	0.30102999
chr11	29979781	29979840	60	1	3	1	0.30102999	0.05404976	2	2	0.08289318	0.30102999
chr11	29979840	30146135	166296	3	3	1	0.51676182	0.05404976	1	2	0.02438896	0.30102999
chr11	30146135	30253446	107312	2	3	1	0.30102999	0.01091641	2	4	0.08289318	0.76005302
chr11	30253446	30253498	53	1	3	1	0.17593012	0.01091641	3	4	0.17593012	0.76005302
chr11	30253498	30379373	125876	2	2	1	0.1575501	0.02438896	2	3	0.1575501	0.51676182
chr11	30379373	30379432	60	1	3	1	0.10122019	0.01091641	4	4	0.30102999	0.76005302
chr11	30379432	30517013	137582	3	3	1	0.10122019	0.02438896	4	3	0.30102999	0.51676182
chr11	30517013	30601996	84984	3	3	1	0.10122019	0.01091641	4	4	0.30102999	0.76005302
chr11	30601996	30602048	53	1	3	1	0.05670724	0.00478973	5	5	0.45545077	1.02643191
chr11	30602048	30741612	139565	1	3	1	0.05670724	0.01091641	5	4	0.45545077	0.76005302
chr11	30741612	30896464	154853	2	3	1	0.03070643	0.01091641	6	4	0.63695542	0.76005302
chr11	30896464	31052904	156441	5	3	1	0.03070643	0.00478973	6	5	0.63695542	1.02643191
chr11	31052904	31172410	119507	2	2	1	0.01053319	0.00478973	6	5	0.91219088	1.02643191
chr11	31172410	31284538	112129	4	2	1	0.01053319	0.00204627	6	6	0.91219088	1.31360226
chr11	31284538	31391205	106668	3	2	1	0.01053319	0.01091641	6	4	0.91219088	0.76005302
chr11	31391205	31468297	77093	3	2	1	0.01053319	0.00478973	6	5	0.91219088	1.02643191
chr11	31468297	31521664	53368	2	2	1	0.01053319	0.02438896	6	3	0.91219088	0.51676182
chr11	31521664	31561279	39616	2	2	1	0.01053319	0.01091641	6	4	0.91219088	0.76005302
chr11	31561279	31625389	64111	2	2	1	0.01053319	0.02438896	6	3	0.91219088	0.51676182
chr11	31625389	31668393	43005	2	2	1	0.01053319	0.01091641	6	4	0.91219088	0.76005302
chr11	31668393	31822366	153974	4	2	1	0.01053319	0.02438896	6	3	0.91219088	0.51676182
chr11	31822366	31857796	35431	3	2	1	0.01053319	0.01091641	6	4	0.91219088	0.76005302
chr11	31857796	31870663	12868	1	2	1	0.01053319	0.02438896	6	3	0.91219088	0.51676182
chr11	31870663	31926025	55363	2	2	1	0.01053319	0.01091641	6	4	0.91219088	0.76005302

chr11	31926025	32182723	256699	3	2	1	0.01053319	0.00478973	6	5	0.91219088	1.02643191
chr11	32182723	32182782	60	1	4	1	0.06713722	0.00478973	6	5	0.44141547	1.02643191
chr11	32182782	32439154	256373	3	3	1	0.03070643	0.00478973	6	5	0.63695542	1.02643191
chr11	32439154	32562898	123745	2	3	1	0.03070643	0.01091641	6	4	0.63695542	0.76005302
chr11	32562898	33331503	768606	25	3	1	0.03070643	0.00478973	6	5	0.63695542	1.02643191
chr11	33331503	33488703	157201	4	3	2	0.03070643	0.02162467	6	5	0.63695542	0.68214471
chr11	33488703	33730524	241822	6	3	2	0.01598258	0.02162467	7	5	0.84395715	0.68214471
chr11	33730524	33880305	149782	2	3	2	0.03070643	0.02162467	6	5	0.63695542	0.68214471
chr11	33880305	33952093	71789	5	3	2	0.01598258	0.02162467	7	5	0.84395715	0.68214471
chr11	33952093	33952152	60	1	3	2	0.0079614	0.02162467	8	5	1.07548421	0.68214471
chr11	33952152	34072303	120152	1	3	2	0.03070643	0.02162467	6	5	0.63695542	0.68214471
chr11	34072303	34098149	25847	2	4	2	0.06713722	0.02162467	6	5	0.44141547	0.68214471
chr11	34098149	34292369	194221	4	3	1	0.03070643	0.00478973	6	5	0.63695542	1.02643191
chr11	34292369	34292428	60	1	4	1	0.03812622	0.00478973	7	5	0.60763643	1.02643191
chr11	34292428	34350648	58221	2	4	1	0.06713722	0.00478973	6	5	0.44141547	1.02643191
chr11	34350648	34386314	35667	2	4	1	0.03812622	0.00478973	7	5	0.60763643	1.02643191
chr11	34386314	34530961	144648	5	4	1	0.06713722	0.00478973	6	5	0.44141547	1.02643191
chr11	34530961	34651737	120777	1	3	1	0.03070643	0.00478973	6	5	0.63695542	1.02643191
chr11	34651737	34764178	112442	4	4	1	0.06713722	0.00478973	6	5	0.44141547	1.02643191
chr11	34764178	34764237	60	1	5	1	0.12309572	0.00478973	6	5	0.30102999	1.02643191
chr11	34764237	34903851	139615	1	5	1	0.12309572	0.01091641	6	4	0.30102999	0.76005302
chr11	34903851	34933576	29726	2	5	1	0.07511598	0.01091641	7	4	0.43181735	0.76005302
chr11	34933576	34951163	17588	1	4	1	0.03812622	0.01091641	7	4	0.60763643	0.76005302
chr11	34951163	34988333	37171	1	4	0	0.03812622	0	7	4	0.60763643	1.26272838
chr11	34988333	35016838	28506	3	5	0	0.07511598	0	7	4	0.43181735	1.26272838
chr11	35016838	35060987	44150	2	5	0	0.04407651	0	8	4	0.58747015	1.26272838
chr11	35060987	35198117	137131	2	3	0	0.0079614	0	8	3	1.07548421	0.93173516
chr11	35198117	35276999	78883	6	3	0	0.0079614	0	8	4	1.07548421	1.26272838
chr11	35276999	35380848	103850	2	3	0	0.0079614	0	8	3	1.07548421	0.93173516
chr11	35380848	35453639	72792	3	3	0	0.01598258	0	7	3	0.84395715	0.93173516
chr11	35453639	35474376	20738	2	3	0	0.0079614	0	8	4	1.07548421	1.26272838
chr11	35474376	35492366	17991	1	3	0	0.0079614	0	8	3	1.07548421	0.93173516
chr11	35492366	35535716	43351	1	3	0	0.01598258	0	7	3	0.84395715	0.93173516
chr11	35535716	35685126	149411	3	3	0	0.01598258	0	7	4	0.84395715	1.26272838
chr11	35685126	35960609	275484	8	4	0	0.03812622	0	7	4	0.60763643	1.26272838
chr11	35960609	36008617	48009	1	4	0	0.03812622	0	7	3	0.60763643	0.93173516
chr11	36008617	36008676	60	1	4	1	0.03812622	0.02438896	7	3	0.60763643	0.51676182
chr11	36008676	36084793	76118	1	4	0	0.03812622	0	7	3	0.60763643	0.93173516
chr11	36084793	36084852	60	1	4	0	0.03812622	0	7	4	0.60763643	1.26272838
chr11	36084852	36200233	115382	2	4	0	0.06713722	0	6	3	0.44141547	0.93173516
chr11	36200233	36200292	60	1	5	0	0.12309572	0	6	3	0.30102999	0.93173516
chr11	36200292	36309570	109279	2	5	0	0.19510895	0	5	3	0.19510895	0.93173516
chr11	36309570	36420877	111308	3	4	0	0.11390336	0	5	3	0.30102999	0.93173516
chr11	36420877	36445235	24359	1	3	0	0.05670724	0	5	3	0.45545077	0.93173516
chr11	36445235	36472190	26956	1	3	0	0.05670724	0	5	1	0.45545077	0.30102999
chr11	36472190	36537037	64848	2	2	0	0.02162467	0	5	1	0.68214471	0.30102999
chr11	36537037	36614666	77630	4	3	0	0.05670724	0	5	2	0.45545077	0.61140001
chr11	36614666	36680598	65933	2	3	0	0.05670724	0	5	1	0.45545077	0.30102999
chr11	36680598	36801306	120709	1	1	0	0.00478973	0	5	1	1.02643191	0.30102999
chr11	36801306	36973982	172677	1	1	0	0.02438896	0	3	1	0.51676182	0.30102999
chr11	36973982	37126229	152248	1	1	0	0.05404976	0	2	1	0.30102999	0.30102999
chr11	37126229	37305602	179374	2	1	0	0.05404976	0	2	2	0.30102999	0.61140001
chr11	37305602	37305661	60	1	2	0	0.08289318	0	3	2	0.30102999	0.61140001
chr11	37305661	37442379	136719	1	1	0	0.02438896	0	3	2	0.51676182	0.61140001
chr11	37442379	37442438	60	1	1	0	0.01091641	0	4	2	0.76005302	0.61140001
chr11	38868079	38868138	60	1	0	0	0	0	0	2	0	0.61140001
chr11	39233962	39528105	294144	3	0	0	0	0	0	2	0	0.61140001
chr11	39528105	39979653	451549	4	1	0	0.1218695	0	1	2	0.1218695	0.61140001
chr11	39979653	39979712	60	1	2	0	0.1575501	0	2	3	0.1575501	0.93173516
chr11	39979712	40149246	169535	1	2	0	0.1575501	0	2	2	0.1575501	0.61140001
chr11	40149246	40339615	190370	4	2	1	0.1575501	0.05404976	2	2	0.1575501	0.30102999
chr11	40339615	40428726	89112	2	2	1	0.1575501	0.02438896	2	3	0.1575501	0.51676182
chr11	40428726	40694847	266122	4	2	2	0.1575501	0.08289318	2	3	0.1575501	0.30102999
chr11	40694847	40859806	164960	2	2	1	0.1575501	0.02438896	2	3	0.1575501	0.51676182
chr11	40859806	41094392	234587	6	2	2	0.1575501	0.08289318	2	3	0.1575501	0.30102999
chr11	41094392	41385521	291130	5	2	1	0.1575501	0.02438896	2	3	0.1575501	0.51676182
chr11	41385521	41944359	558839	4	1	1	0.05404976	0.02438896	2	3	0.30102999	0.51676182
chr11	41944359	41944418	60	1	1	1	0.05404976	0.01091641	2	4	0.30102999	0.76005302
chr11	41944418	42226159	281742	2	0	0	0	0	1	0	0.30102999	0
chr11	42226159	42226218	60	1	1	0	0.1218695	0	1	1	0.1218695	0.30102999
chr11	42226218	42648087	421870	2	1	0	0.1218695	0	1	0	0.1218695	0
chr11	42648087	42648146	60	1	2	1	0.30102999	0.02438896	1	3	0.05404976	0.51676182
chr11	42648146	42916891	268746	1	2	1	0.30102999	0.1218695	1	1	0.05404976	0.1218695
chr11	42916891	42916950	60	1	2	1	0.30102999	0.05404976	1	2	0.05404976	0.30102999
chr11	42916950	43115284	198335	1	2	1	0.30102999	0.1218695	1	1	0.05404976	0.1218695
chr11	43115284	43275890	160607	2	2	1	0.1575501	0.05404976	2	2	0.1575501	0.30102999
chr11	43275890	43428874	152985	3	2	1	0.08289318	0.05404976	3	2	0.30102999	0.30102999
chr11	43428874	43448401	19528	2	2	1	0.0429175	0.05404976	4	2	0.47744371	0.30102999
chr11	43448401	43619551	171151	6	2	1	0.08289318	0.05404976	3	2	0.30102999	0.30102999
chr11	43619551	43904698	285148	8	2	1	0.0429175	0.05404976	4	2	0.47744371	0.30102999
chr11	43904698	44088886	184189	5	4	1	0.18734596	0.05404976	4	2	0.18734596	0.30102999
chr11	44088886	44282436	193551	7	4	1	0.18734596	0.02438896	4	3	0.18734596	0.51676182
chr11	44282436	44305648	23213	2	4	1	0.11390336	0.02438896	5	3	0.30102999	0.51676182
chr11	44305648	44305707	60	1	5	1	0.19510895	0.02438896	5	3	0.19510895	0.51676182
chr11	44305707	44452984	147278	2	4	1	0.11390336	0.02438896	5	3	0.30102999	0.51676182
chr11	44452984	44596757	143774	2	5	1	0.19510895	0.02438896	5	3	0.19510895	0.51676182
chr11	44596757	44606275	9519	2	5	1	0.12309572	0.02438896	6	3	0.30102999	0.51676182
chr11	44606275	44770056	163782	3	4	1	0.11390336	0.02438896	5	3	0.30102999	0.51676182
chr11	44770056	44903476	133421	2	4	1	0.18734596	0.02438896	4	3	0.18734596	0.51676182
chr11	44903476	45079695	176220	5	3	1	0.10122019	0.02438896	4	3	0.30102999	0.51676182
chr11	45079695	45172978	93284	3	3	1	0.10122019	0.01091641	4	4	0.30102999	0.76005302
chr11	45172978	45224904	51927	2	3	1	0.10122019	0.02438896	4	3	0.30102999	0.51676182
chr11	45224904	45261909	37006	1	3	1	0.17593012	0.02438896	3	3	0.17593012	0.51676182
chr11	45261909	45373328	111420	4	3	1	0.17593012	0.00478973	3	5	0.17593012	1.02643191
chr11	45373328	45373387	60	1	3	2	0.10122019	0.02162467	4	5	0.30102999	0.68214471
chr11	45373387	45427024	53638	1	3	1	0.10122019	0.00478973	4	5	0.30102999	1.02643191
chr11	45427024	45553869	126846	1	2	1	0.0429175	0.01091641	4	4	0.47744371	0.76005302
chr11	45553869	45670806	116938	2	2	2	0.0429175	0.0429175	4	4	0.47744371	0.47744371
chr11	45670806	45670865	60	1	2	2	0.0429175	0.02162467	4	5	0.47744371	0.68214471
chr11	45670865	45898269	227405	4	1	2	0.01091641	0.02162467	4	5	0.76005302	0.68214471
chr11	45898269	45910165	11897	3	1	3	0.01091641	0.05670724	4	5	0.76005302	0.45545077
chr11	45910165	45954150	43986	2	1	3	0.02438896	0.05670724	3	5	0.51676182	0.45545077
chr11	45954150	46027200	73051	2	0	3	0	0.05670724	3	5	0.93173516	0.45545077
chr11	46027200	46027259	60	1	1	3	0.02438896	0.05670724	3	5	0.51676182	0.45545077
chr11	46027259	46238236	210978	4	0	3	0	0.05670724	3	5	0.93173516	0.45545077
chr11	46238236	46371029	132794	3	0	3	0	0.05670724	4	5	1.26272838	0.45545077
chr11	46371029	46371088	60	1	1	3	0.01091641	0.05670724	4	5	0.76005302	0.45545077
chr11	46371088	46465013	93926	3	0	3	0	0.05670724	4	5	1.26272838	0.45545077
chr11	46465013	46465068	56	1	1	3	0.01091641	0.05670724	4	5	0.76005302	0.45545077
chr11	46465068	46534329	69262	1	0	3	0	0.05670724	4	5	1.26272838	0.45545077
chr11	46534329	46599681	65353	3	0	3	0	0.03070643	4	6	1.26272838	0.63695542
chr11	46599681	46599740	60	1	0	3	0	0.03070643	5	6	1.60515106	0.63695542
chr11	46599740	46671881	72142	3	0	3	0	0.03070643	4	6	1.26272838	0.63695542
chr11	46671881	46749922	78042	2	0	3	0	0.03070643	3	6	0.93173516	0.63695542
chr11	46749922	46761033	11112	2	0	3	0	0.03070643	4	6	1.26272838	0.63695542
chr11	46761033	46862005	100973	4	0	3	0	0.03070643	3	6	0.93173516	0.63695542
chr11	46862005	46958120	96116	4	0	3	0	0.03070643	5	6	1.60515106	0.63695542
chr11	46958120	47111104	152985	4	0	3	0	0.03070643	4	6	1.26272838	0.63695542
chr11	47111104	47111163	60	1	0	3	0	0.03070643	5	6	1.60515106	0.63695542
chr11	47111163	47246191	135029	4	0	3	0	0.03070643	4	6	1.26272838	0.63695542
chr11	47246191	47295456	49266	7	0	4	0	0.06713722	4	6	1.26272838	0.44141547
chr11	47295456	47295510	55	1	0	4	0	0.06713722	5	6	1.60515106	0.44141547
chr11	47295510	47707513	412004	20	0	4	0	0.06713722	4	6	1.26272838	0.44141547
chr11	47707513	47841088	133576	4	0	4	0	0.06713722	2	6	0.61140001	0.44141547

chr11	47841088	48032749	191662	3	0	3	0	0.03070643	2	6	0.61140001	0.63695542
chr11	48032749	48342701	309953	7	0	2	0	0.01053319	2	6	0.61140001	0.91219088
chr11	48342701	48466903	124203	2	0	1	0	0.00478973	2	5	0.61140001	1.02643191
chr11	48466903	48588884	121982	1	0	1	0	0.02438896	2	3	0.61140001	0.51676182
chr11	48588884	48588943	60	1	0	1	0	0.01091641	2	4	0.61140001	0.76005302
chr11	48588943	49532643	943701	6	0	0	0	0	0	1	0	0.30102999
chr11	55375127	55375186	60	1	0	0	0	0	1	0	0.30102999	0
chr11	55481624	55481683	60	1	0	0	0	0	2	1	0.61140001	0.30102999
chr11	55481683	55622470	140788	2	0	0	0	0	2	0	0.61140001	0
chr11	55622470	55622529	60	1	0	1	0	0.30102999	2	0	0.61140001	0
chr11	55622529	55663362	40834	1	0	0	0	0	2	0	0.61140001	0
chr11	55663362	55753062	89701	3	0	0	0	0	2	1	0.61140001	0.30102999
chr11	55753062	55816312	63251	2	0	0	0	0	2	2	0.61140001	0.61140001
chr11	55816312	55980347	164036	3	0	0	0	0	2	1	0.61140001	0.30102999
chr11	55980347	56052089	71743	2	0	0	0	0	3	1	0.93173516	0.30102999
chr11	56052089	56124601	72513	2	0	0	0	0	3	2	0.93173516	0.61140001
chr11	56124601	56188940	64340	1	0	0	0	0	2	2	0.61140001	0.61140001
chr11	56188940	56252050	63111	2	0	0	0	0	2	3	0.61140001	0.93173516
chr11	56252050	56313831	61782	1	0	0	0	0	2	2	0.61140001	0.61140001
chr11	56313831	56342108	28278	2	0	0	0	0	2	3	0.61140001	0.93173516
chr11	56342108	56342167	60	1	0	0	0	0	3	3	0.93173516	0.93173516
chr11	56342167	56394889	52723	1	0	0	0	0	2	2	0.61140001	0.61140001
chr11	56394889	56461267	66379	2	0	0	0	0	3	2	0.93173516	0.61140001
chr11	56461267	56610344	149078	3	0	0	0	0	3	3	0.93173516	0.93173516
chr11	56610344	56610403	60	1	0	1	0	0.02438896	6	3	1.95986592	0.51676182
chr11	56610403	56649983	39581	1	0	1	0	0.02438896	3	3	0.93173516	0.51676182
chr11	56649983	56692526	42544	1	0	0	0	0	3	3	0.93173516	0.93173516
chr11	56692526	56746498	53973	2	0	0	0	0	6	3	1.95986592	0.93173516
chr11	56746498	56746557	60	1	0	1	0	0.02438896	6	3	1.95986592	0.51676182
chr11	56746557	56872403	125847	1	0	0	0	0	4	3	1.26272838	0.93173516
chr11	56872403	56975589	103187	2	0	0	0	0	4	5	1.26272838	1.60515106
chr11	56975589	57002518	26930	2	0	2	0	0.02162467	4	5	1.26272838	0.68214471
chr11	57002518	57002574	57	1	0	2	0	0.02162467	5	5	1.60515106	0.68214471
chr11	57002574	57093520	90947	2	0	1	0	0.00478973	4	5	1.26272838	1.02643191
chr11	57093520	57144214	50695	2	0	0	0	0	3	5	0.93173516	1.60515106
chr11	57144214	57175444	31231	5	0	1	0	0.00478973	5	5	1.60515106	1.02643191
chr11	57175444	57238918	63475	2	0	0	0	0	3	5	0.93173516	1.60515106
chr11	57238918	57238965	48	1	0	0	0	0	4	5	1.26272838	1.60515106
chr11	57238965	57327481	88517	3	0	0	0	0	3	5	0.93173516	1.60515106
chr11	57327481	57327540	60	1	0	2	0	0.02162467	3	5	0.93173516	0.68214471
chr11	57327540	57468357	140818	5	0	1	0	0.00478973	3	5	0.93173516	1.02643191
chr11	57468357	57529533	61177	5	0	2	0	0.02162467	3	5	0.93173516	0.68214471
chr11	57529533	57574289	44757	2	0	0	0	0	3	5	0.93173516	1.60515106
chr11	57574289	57574348	60	1	0	2	0	0.02162467	4	5	1.26272838	0.68214471
chr11	57574348	57668293	93946	1	0	0	0	0	4	5	1.26272838	1.60515106
chr11	57668293	57788307	120015	1	0	0	0	0	4	4	1.26272838	1.26272838
chr11	57788307	57944568	156262	4	0	0	0	0	5	4	1.60515106	1.26272838
chr11	57944568	57944627	60	1	0	0	0	0	7	4	2.3278427	1.26272838
chr11	57944627	57984274	39648	1	0	0	0	0	7	3	2.3278427	0.93173516
chr11	57984274	58035563	51290	1	0	0	0	0	6	3	1.95986592	0.93173516
chr11	58035563	58130496	94934	1	0	0	0	0	5	3	1.60515106	0.93173516
chr11	58130496	58201966	71471	1	0	0	0	0	5	2	1.60515106	0.61140001
chr11	58201966	58202025	60	1	1	0	0.00204627	0	6	2	1.31360226	0.61140001
chr11	58202025	58312165	110141	2	1	0	0.01091641	0	4	2	0.76005302	0.61140001
chr11	58312165	58362869	50705	3	1	0	0.00478973	0	5	2	1.02643191	0.61140001
chr11	58362869	58392828	29960	2	1	0	0.00204627	0	6	2	1.31360226	0.61140001
chr11	58392828	58480268	87441	1	0	0	0	0	4	2	1.26272838	0.61140001
chr11	58480268	58891154	410887	8	0	0	0	0	3	2	0.93173516	0.61140001
chr11	58891154	58967316	76163	4	0	0	0	0	4	2	1.26272838	0.61140001
chr11	58967316	59163305	195990	3	0	0	0	0	3	2	0.93173516	0.61140001
chr11	59163305	59295250	131946	3	0	0	0	0	3	3	0.93173516	0.93173516
chr11	59295250	59295309	60	1	0	1	0	0.02438896	3	3	0.93173516	0.51676182
chr11	59295309	59343075	47767	1	0	1	0	0.02438896	2	3	0.61140001	0.51676182
chr11	59343075	59369293	26219	3	0	2	0	0.0429175	2	4	0.61140001	0.47744371
chr11	59369293	59496171	126879	4	0	1	0	0.01091641	2	4	0.61140001	0.76005302
chr11	59496171	59496230	60	1	1	1	0.05404976	0.01091641	2	4	0.30102999	0.76005302
chr11	59496230	59541532	45303	1	0	1	0	0.01091641	2	4	0.61140001	0.76005302
chr11	59541532	59569319	27788	1	0	1	0	0.01091641	1	4	0.30102999	0.76005302
chr11	59569319	59569378	60	1	0	2	0	0.0429175	1	4	0.30102999	0.47744371
chr11	59569378	59611911	42534	2	0	1	0	0.01091641	1	4	0.30102999	0.76005302
chr11	59611911	59626700	14790	2	0	2	0	0.0429175	1	4	0.30102999	0.47744371
chr11	59626700	59679690	52991	1	0	2	0	0.08289318	1	3	0.30102999	0.30102999
chr11	59679690	59739027	59338	1	0	2	0	0.08289318	0	3	0	0.30102999
chr11	59739027	59913096	174070	5	0	2	0	0.0429175	0	4	0	0.47744371
chr11	59913096	60043784	130689	4	1	2	0.1218695	0.0429175	1	4	0.1218695	0.47744371
chr11	60043784	60108734	64951	1	1	2	0.1218695	0.08289318	1	3	0.1218695	0.30102999
chr11	60108734	60355993	247260	8	0	2	0	0.08289318	1	3	0.30102999	0.30102999
chr11	60355993	60356052	60	1	0	2	0	0.0429175	1	4	0.30102999	0.47744371
chr11	60356052	60416190	60139	2	0	2	0	0.08289318	1	3	0.30102999	0.30102999
chr11	60416190	60454540	38351	2	0	2	0	0.0429175	2	4	0.61140001	0.47744371
chr11	60454540	60614447	159908	5	1	2	0.05404976	0.0429175	2	4	0.30102999	0.47744371
chr11	60614447	60942446	328000	14	1	2	0.02438896	0.0429175	3	4	0.51676182	0.47744371
chr11	60942446	60942505	60	1	2	2	0.08289318	0.0429175	3	4	0.30102999	0.47744371
chr11	60942505	61049008	106504	2	1	2	0.02438896	0.0429175	3	4	0.51676182	0.47744371
chr11	61049008	61090525	41518	3	2	2	0.08289318	0.0429175	3	4	0.30102999	0.47744371
chr11	61090525	61113696	23172	5	3	2	0.17593012	0.0429175	3	4	0.17593012	0.47744371
chr11	61113696	61124898	11203	1	2	2	0.08289318	0.0429175	3	4	0.30102999	0.47744371
chr11	61124898	61136645	11748	3	3	3	0.17593012	0.10122019	3	4	0.17593012	0.30102999
chr11	61136645	61161394	24750	1	3	2	0.17593012	0.0429175	3	4	0.17593012	0.47744371
chr11	61161394	61161448	55	1	4	2	0.30102999	0.0429175	3	4	0.10122019	0.47744371
chr11	61161448	61170164	8717	1	4	2	0.30102999	0.08289318	3	3	0.10122019	0.30102999
chr11	61170164	61170400	237	2	4	3	0.18734596	0.17593012	4	3	0.18734596	0.17593012
chr11	61170400	61296134	125735	4	4	2	0.18734596	0.1575501	4	2	0.18734596	0.1575501
chr11	61296134	61296189	56	1	5	2	0.30102999	0.1575501	4	2	0.11390336	0.1575501
chr11	61296189	61328097	31909	1	5	2	0.45545077	0.1575501	3	2	0.05670724	0.1575501
chr11	61328097	61349437	21341	1	4	2	0.30102999	0.1575501	3	2	0.10122019	0.1575501
chr11	61349437	61410075	60639	1	3	2	0.17593012	0.1575501	3	2	0.17593012	0.1575501
chr11	61410075	61452950	42876	2	4	2	0.11390336	0.1575501	5	2	0.30102999	0.1575501
chr11	61452950	61453009	60	1	5	2	0.19510895	0.08289318	5	3	0.19510895	0.30102999
chr11	61453009	61493778	40770	1	3	2	0.05670724	0.08289318	5	3	0.45545077	0.30102999
chr11	61493778	61556671	62894	3	3	2	0.05670724	0.0429175	5	4	0.45545077	0.47744371
chr11	61556671	61561874	5204	2	3	2	0.03070643	0.0429175	6	4	0.63695542	0.47744371
chr11	61561874	61609876	48003	4	4	2	0.06713722	0.0429175	6	4	0.44141547	0.47744371
chr11	61609876	61647974	38099	2	4	2	0.06713722	0.08289318	6	3	0.44141547	0.30102999
chr11	61647974	61656728	8755	2	4	2	0.06713722	0.0429175	6	4	0.44141547	0.47744371
chr11	61656728	61686788	30061	1	3	2	0.03070643	0.0429175	6	4	0.63695542	0.47744371
chr11	61686788	61686840	53	1	4	2	0.06713722	0.0429175	6	4	0.44141547	0.47744371
chr11	61686840	61840997	154158	5	3	2	0.05670724	0.08289318	5	3	0.45545077	0.30102999
chr11	61840997	61841056	60	1	4	2	0.06713722	0.0429175	6	4	0.44141547	0.47744371
chr11	61841056	61911830	70775	1	3	2	0.05670724	0.0429175	5	4	0.45545077	0.47744371
chr11	61911830	61958418	46589	3	4	2	0.11390336	0.0429175	5	4	0.30102999	0.47744371
chr11	61958418	62010050	51633	3	4	2	0.06713722	0.0429175	6	4	0.44141547	0.47744371
chr11	62010050	62038095	28046	1	3	2	0.03070643	0.0429175	6	4	0.63695542	0.47744371
chr11	62038095	62083808	45714	1	2	2	0.01053319	0.0429175	6	4	0.91219088	0.47744371
chr11	62083808	62083867	60	1	3	2	0.03070643	0.0429175	6	4	0.63695542	0.47744371
chr11	62083867	62107443	23577	1	2	1	0.02162467	0.02438896	5	3	0.68214471	0.51676182
chr11	62107443	62124627	17185	1	1	1	0.01091641	0.02438896	4	3	0.76005302	0.51676182
chr11	62124627	62190466	65840	2	0	1	0	0.02438896	4	3	1.26272838	0.51676182
chr11	62190466	62230707	40242	2	1	1	0.00478973	0.02438896	5	3	1.02643191	0.51676182
chr11	62230707	62297505	66799	2	1	1	0.01091641	0.02438896	4	3	0.76005302	0.51676182
chr11	62297505	62299947	2443	2	1	1	0.00478973	0.02438896	5	3	1.02643191	0.51676182
chr11	62299947	62336124	36178	1	1	1	0.01091641	0.02438896	4	3	0.76005302	0.51676182
chr11	62336124	62371439	35316	3	0	1	0	0.02438896	4	3	1.26272838	0.51676182
chr11	62371439	62407089	35651	5	1	1	0.01091641	0.02438896	4	3	0.76005302	0.51676182

chr11	62407089	62407148	60	1	1	1	0.00478973	0.02438896	5	3	1.02643191	0.51676182
chr11	62407148	62475483	68336	4	1	1	0.00478973	0.05404976	5	2	1.02643191	0.30102999
chr11	62475483	62475540	58	1	2	1	0.02162467	0.05404976	5	2	0.68214471	0.30102999
chr11	62475540	62495815	20276	1	0	1	0	0.05404976	5	2	1.60515106	0.30102999
chr11	62495815	62502913	7099	2	0	1	0	0.02438896	5	3	1.60515106	0.51676182
chr11	62502913	62534121	31209	2	0	1	0	0.05404976	5	2	1.60515106	0.30102999
chr11	62534121	62571812	37692	4	0	1	0	0.02438896	5	3	1.60515106	0.51676182
chr11	62571812	62571871	60	1	1	1	0.00478973	0.02438896	5	3	1.02643191	0.51676182
chr11	62571871	62647750	75880	4	0	1	0	0.02438896	5	3	1.60515106	0.51676182
chr11	62647750	62686758	39009	2	0	1	0	0.02438896	4	3	1.26272838	0.51676182
chr11	62686758	62747892	61135	1	0	1	0	0.02438896	2	3	0.61140001	0.51676182
chr11	62747892	62889579	141688	3	0	1	0	0.02438896	3	3	0.93173516	0.51676182
chr11	62889579	62952099	62521	2	0	1	0	0.02438896	4	3	1.26272838	0.51676182
chr11	62952099	62952158	60	1	1	1	0.01091641	0.02438896	4	3	0.76005302	0.51676182
chr11	62952158	63069662	117505	2	1	1	0.02438896	0.02438896	3	3	0.51676182	0.51676182
chr11	63069662	63069721	60	1	1	2	0.02438896	0.0429175	3	4	0.51676182	0.47744371
chr11	63069721	63137588	67868	1	1	2	0.05404976	0.0429175	2	4	0.30102999	0.47744371
chr11	63137588	63143087	5500	2	1	3	0.05404976	0.10122019	2	4	0.30102999	0.30102999
chr11	63143087	63173946	30860	1	1	2	0.05404976	0.0429175	2	4	0.30102999	0.47744371
chr11	63173946	63230830	56885	3	1	2	0.02438896	0.0429175	3	4	0.51676182	0.47744371
chr11	63230830	63230889	60	1	1	3	0.02438896	0.10122019	3	4	0.51676182	0.30102999
chr11	63230889	63278493	47605	1	1	3	0.05404976	0.10122019	2	4	0.30102999	0.30102999
chr11	63278493	63342310	63818	3	1	1	0.05404976	0.01091641	2	4	0.30102999	0.76005302
chr11	63342310	63342369	60	1	1	1	0.02438896	0.01091641	3	4	0.51676182	0.76005302
chr11	63342369	63367171	24803	1	1	1	0.05404976	0.01091641	2	4	0.30102999	0.76005302
chr11	63367171	63426700	59530	3	1	1	0.02438896	0.01091641	3	4	0.51676182	0.76005302
chr11	63426700	63472345	45646	1	1	1	0.05404976	0.02438896	2	3	0.30102999	0.51676182
chr11	63472345	63593273	120929	4	1	1	0.05404976	0.01091641	2	4	0.30102999	0.76005302
chr11	63593273	63593332	60	1	1	3	0.02438896	0.05670724	3	5	0.51676182	0.45545077
chr11	63593332	63666123	72792	5	1	3	0.05404976	0.05670724	2	5	0.30102999	0.45545077
chr11	63666123	63756165	90043	3	1	3	0.1218695	0.10122019	1	4	0.1218695	0.30102999
chr11	63756165	63882963	126799	4	1	3	0.1218695	0.05670724	1	5	0.1218695	0.45545077
chr11	63882963	64358665	475703	25	1	4	0.1218695	0.11390336	1	5	0.1218695	0.30102999
chr11	64358665	64358724	60	1	2	4	0.1575501	0.11390336	2	5	0.1575501	0.30102999
chr11	64358724	64570971	212248	12	1	4	0.05404976	0.11390336	2	5	0.30102999	0.30102999
chr11	64570971	64578407	7437	3	2	4	0.1575501	0.11390336	2	5	0.1575501	0.30102999
chr11	64578407	64672741	94335	2	2	3	0.1575501	0.05670724	2	5	0.1575501	0.45545077
chr11	64672741	64755476	82736	5	3	3	0.30102999	0.05670724	2	5	0.08289318	0.45545077
chr11	64755476	64785672	30197	2	2	3	0.1575501	0.05670724	2	5	0.1575501	0.45545077
chr11	64785672	64848925	63254	6	2	3	0.1575501	0.03070643	2	6	0.1575501	0.63695542
chr11	64848925	64868919	19995	4	3	3	0.30102999	0.03070643	2	6	0.08289318	0.63695542
chr11	64868919	64968857	99939	5	2	3	0.1575501	0.03070643	2	6	0.1575501	0.63695542
chr11	64968857	64968916	60	1	3	3	0.17593012	0.03070643	3	6	0.17593012	0.63695542
chr11	64968916	65057311	88396	2	2	3	0.1575501	0.03070643	2	6	0.1575501	0.63695542
chr11	65057311	65065107	7797	2	3	3	0.30102999	0.03070643	2	6	0.08289318	0.63695542
chr11	65065107	65109190	44084	2	2	3	0.30102999	0.03070643	1	6	0.05404976	0.63695542
chr11	65109190	65109249	60	1	3	3	0.30102999	0.03070643	2	6	0.08289318	0.63695542
chr11	65109249	65142778	33530	2	3	3	0.30102999	0.05670724	2	5	0.08289318	0.45545077
chr11	65142778	65149392	6615	3	3	3	0.30102999	0.01598258	2	7	0.08289318	0.84395715
chr11	65149392	65303260	153869	5	2	3	0.1575501	0.03070643	2	6	0.1575501	0.63695542
chr11	65303260	65318425	15166	2	3	3	0.17593012	0.03070643	3	6	0.17593012	0.63695542
chr11	65318425	65338387	19963	1	3	3	0.30102999	0.03070643	2	6	0.08289318	0.63695542
chr11	65338387	65340664	2278	2	3	3	0.30102999	0.01598258	2	7	0.08289318	0.84395715
chr11	65340664	65361644	20981	1	3	3	0.30102999	0.03070643	2	6	0.08289318	0.63695542
chr11	65361644	65376658	15015	2	3	3	0.10122019	0.03070643	4	6	0.30102999	0.63695542
chr11	65376658	65531771	155114	7	3	3	0.17593012	0.05670724	3	5	0.17593012	0.45545077
chr11	65531771	65531830	60	1	4	3	0.30102999	0.05670724	3	5	0.10122019	0.45545077
chr11	65531830	65544038	12209	1	3	3	0.17593012	0.05670724	3	5	0.17593012	0.45545077
chr11	65544038	65544097	60	1	3	3	0.10122019	0.05670724	4	5	0.30102999	0.45545077
chr11	65544097	65562392	18296	2	3	3	0.17593012	0.05670724	3	5	0.17593012	0.45545077
chr11	65562392	65632191	69800	2	2	3	0.08289318	0.05670724	3	5	0.30102999	0.45545077
chr11	65632191	65649068	16878	3	2	4	0.08289318	0.06713722	3	6	0.30102999	0.44141547
chr11	65649068	65661361	12294	4	2	4	0.0429175	0.06713722	4	6	0.47744371	0.44141547
chr11	65661361	65688272	26912	2	2	4	0.1575501	0.06713722	2	6	0.1575501	0.44141547
chr11	65688272	65724878	36607	1	2	4	0.1575501	0.11390336	2	5	0.1575501	0.30102999
chr11	65724878	65881513	156636	11	4	4	0.18734596	0.11390336	4	5	0.18734596	0.30102999
chr11	65881513	65983969	102457	2	4	4	0.30102999	0.11390336	3	5	0.10122019	0.30102999
chr11	65983969	65984026	58	1	4	4	0.18734596	0.11390336	4	5	0.18734596	0.30102999
chr11	65984026	66046441	62416	3	4	4	0.30102999	0.11390336	3	5	0.10122019	0.30102999
chr11	66046441	66052099	5659	2	4	4	0.18734596	0.11390336	4	5	0.18734596	0.30102999
chr11	66052099	66055962	3864	1	4	4	0.18734596	0.18734596	4	4	0.18734596	0.18734596
chr11	66055962	66063266	7305	1	4	4	0.30102999	0.18734596	3	4	0.10122019	0.18734596
chr11	66063266	66101855	38590	2	4	4	0.30102999	0.11390336	3	5	0.10122019	0.30102999
chr11	66101855	66134825	32971	5	4	4	0.18734596	0.11390336	4	5	0.18734596	0.30102999
chr11	66134825	66157106	22282	1	4	4	0.30102999	0.11390336	3	5	0.10122019	0.30102999
chr11	66157106	66157165	60	1	4	4	0.30102999	0.03812622	3	7	0.10122019	0.60763643
chr11	66157165	66204328	47164	2	3	4	0.17593012	0.03812622	3	7	0.17593012	0.60763643
chr11	66204328	66204387	60	1	4	4	0.30102999	0.03812622	3	7	0.10122019	0.60763643
chr11	66204387	66276296	71910	4	3	4	0.30102999	0.03812622	2	7	0.08289318	0.60763643
chr11	66276296	66276355	60	1	5	4	0.68214471	0.03812622	2	7	0.02162467	0.60763643
chr11	66276355	66316040	39686	2	4	4	0.47744371	0.06713722	2	6	0.0429175	0.44141547
chr11	66316040	66330922	14883	2	5	4	0.68214471	0.06713722	2	6	0.02162467	0.44141547
chr11	66330922	66331133	212	2	5	4	0.68214471	0.02074938	2	8	0.02162467	0.79906872
chr11	66331133	66386395	55263	5	4	4	0.47744371	0.06713722	2	6	0.0429175	0.44141547
chr11	66386395	66397095	10701	2	4	4	0.30102999	0.06713722	3	6	0.10122019	0.44141547
chr11	66397095	66433028	35934	2	4	4	0.47744371	0.06713722	2	6	0.0429175	0.44141547
chr11	66433028	66479188	46161	3	4	4	0.30102999	0.06713722	3	6	0.10122019	0.44141547
chr11	66479188	66613959	134772	4	3	4	0.17593012	0.11390336	3	5	0.17593012	0.30102999
chr11	66613959	66627969	14011	2	4	4	0.30102999	0.11390336	3	5	0.10122019	0.30102999
chr11	66627969	66675429	47461	2	3	4	0.17593012	0.11390336	3	5	0.17593012	0.30102999
chr11	66675429	66888750	213322	6	2	4	0.08289318	0.11390336	3	5	0.30102999	0.30102999
chr11	66888750	67008506	119757	4	2	4	0.0429175	0.11390336	4	5	0.47744371	0.30102999
chr11	67008506	67023899	15394	1	2	4	0.0429175	0.18734596	4	4	0.47744371	0.18734596
chr11	67023899	67042233	18335	1	1	4	0.01091641	0.18734596	4	4	0.76005302	0.18734596
chr11	67042233	67073321	31089	1	1	3	0.01091641	0.10122019	4	4	0.76005302	0.30102999
chr11	67073321	67073380	60	1	2	3	0.0429175	0.10122019	4	4	0.47744371	0.30102999
chr11	67073380	67118996	45617	1	1	3	0.02438896	0.10122019	3	4	0.51676182	0.30102999
chr11	67118996	67132168	13173	2	1	4	0.02438896	0.18734596	3	4	0.51676182	0.18734596
chr11	67132168	67136492	4325	1	1	3	0.02438896	0.10122019	3	4	0.51676182	0.30102999
chr11	67136492	67136551	60	1	1	3	0.01091641	0.10122019	4	4	0.76005302	0.30102999
chr11	67136551	67164667	28117	1	1	3	0.02438896	0.10122019	3	4	0.51676182	0.30102999
chr11	67164667	67203932	39266	2	2	4	0.08289318	0.18734596	3	4	0.30102999	0.18734596
chr11	67203932	67267075	63144	5	2	3	0.08289318	0.10122019	3	4	0.30102999	0.30102999
chr11	67267075	67288861	21787	3	2	3	0.0429175	0.10122019	4	4	0.47744371	0.30102999
chr11	67288861	67288918	58	1	2	4	0.0429175	0.18734596	4	4	0.47744371	0.18734596
chr11	67288918	67325627	36710	1	2	4	0.08289318	0.18734596	3	4	0.30102999	0.18734596
chr11	67325627	67353766	28140	2	2	3	0.08289318	0.10122019	3	4	0.30102999	0.30102999
chr11	67353766	67374037	20272	1	2	2	0.08289318	0.0429175	3	4	0.30102999	0.47744371
chr11	67374037	67375079	1043	2	2	3	0.08289318	0.10122019	3	4	0.30102999	0.30102999
chr11	67375079	67411852	36774	2	2	2	0.08289318	0.0429175	3	4	0.30102999	0.47744371
chr11	67411852	67438358	26507	2	2	2	0.0429175	0.0429175	4	4	0.47744371	0.47744371
chr11	67438358	67768754	330397	1	1	2	0.01091641	0.08289318	4	3	0.76005302	0.30102999
chr11	67768754	67768813	60	1	2	2	0.02162467	0.08289318	5	3	0.68214471	0.30102999
chr11	67768813	67781582	12770	1	1	2	0.00478973	0.08289318	5	3	1.02643191	0.30102999
chr11	67781582	67820414	38833	4	1	2	0.00478973	0.0429175	5	4	1.02643191	0.47744371
chr11	67820414	67888074	67661	2	1	2	0.01091641	0.0429175	4	4	0.76005302	0.47744371
chr11	67888074	67933221	45148	1	1	2	0.01091641	0.08289318	4	3	0.76005302	0.30102999
chr11	67933221	68034067	100847	4	2	2	0.02162467	0.08289318	5	3	0.68214471	0.30102999
chr11	68034067	68115829	81763	2	2	2	0.0429175	0.08289318	4	3	0.47744371	0.30102999
chr11	68115829	68184592	68764	1	2	2	0.08289318	0.08289318	3	3	0.30102999	0.30102999
chr11	68184592	68216761	32170	1	2	2	0.1575501	0.08289318	2	3	0.1575501	0.30102999
chr11	68216761	68229868	13108	1	2	2	0.1575501	0.1575501	2	2	0.1575501	0.1575501
chr11	68229868	68286974	57107	1	2	1	0.1575501	0.05404976	2	2	0.1575501	0.30102999
chr11	68286974	68287033	60	1	2	1	0.1575501	0.02438896	2	3	0.1575501	0.51676182

chr11	68287033	68305307	18275	1	2	1	0.1575501	0.05404976	2	2	0.1575501	0.30102999
chr11	68305307	68336631	31325	1	1	1	0.05404976	0.05404976	2	2	0.30102999	0.30102999
chr11	68336631	68336690	60	1	1	1	0.05404976	0.02438896	2	3	0.30102999	0.51676182
chr11	68336690	68381504	44815	1	1	1	0.05404976	0.1218695	2	1	0.30102999	0.1218695
chr11	68381504	68454441	72938	2	1	1	0.05404976	0.05404976	2	2	0.30102999	0.30102999
chr11	68454441	68475784	21344	3	1	1	0.02438896	0.05404976	3	2	0.51676182	0.30102999
chr11	68475784	68549308	73525	6	1	1	0.02438896	0.02438896	3	3	0.51676182	0.51676182
chr11	68549308	68566737	17430	2	2	1	0.0429175	0.01091641	4	4	0.47744371	0.76005302
chr11	68566737	68601605	34869	1	1	1	0.02438896	0.01091641	3	4	0.51676182	0.76005302
chr11	68601605	68649827	48223	1	1	1	0.02438896	0.02438896	3	3	0.51676182	0.51676182
chr11	68649827	68682282	32456	2	2	1	0.0429175	0.02438896	4	3	0.47744371	0.51676182
chr11	68682282	68771883	89602	2	2	1	0.08289318	0.05404976	3	2	0.30102999	0.30102999
chr11	68771883	68771942	60	1	2	1	0.0429175	0.01091641	4	4	0.47744371	0.76005302
chr11	68771942	68818714	46773	1	2	1	0.0429175	0.02438896	4	3	0.47744371	0.51676182
chr11	68818714	68818773	60	1	2	1	0.02162467	0.02438896	5	3	0.68214471	0.51676182
chr11	68818773	68897871	79099	2	1	1	0.01091641	0.02438896	4	3	0.76005302	0.51676182
chr11	68897871	68928165	30295	2	1	1	0.00478973	0.02438896	5	3	1.02643191	0.51676182
chr11	68928165	69048284	120120	1	1	1	0.01091641	0.02438896	4	3	0.76005302	0.51676182
chr11	69048284	69234367	186084	4	1	1	0.00478973	0.02438896	5	3	1.02643191	0.51676182
chr11	69234367	69587837	353471	6	1	1	0.00478973	0.01091641	5	4	1.02643191	0.76005302
chr11	69587837	69587896	60	1	2	1	0.02162467	0.01091641	5	4	0.68214471	0.76005302
chr11	69587896	69630454	42559	3	1	1	0.00478973	0.01091641	5	4	1.02643191	0.76005302
chr11	69630454	69891573	261120	1	1	1	0.01091641	0.01091641	4	4	0.76005302	0.76005302
chr11	69891573	69891632	60	1	2	1	0.0429175	0.01091641	4	4	0.47744371	0.76005302
chr11	69891632	70129305	237674	9	2	1	0.08289318	0.01091641	3	4	0.30102999	0.76005302
chr11	70129305	70181821	52517	2	2	1	0.1575501	0.01091641	2	4	0.1575501	0.76005302
chr11	70181821	70335116	153296	3	2	0	0.1575501	0	2	3	0.1575501	0.93173516
chr11	70335116	70367700	32585	2	2	1	0.0429175	0.02438896	4	3	0.47744371	0.51676182
chr11	70367700	70647318	279619	5	2	0	0.30102999	0	1	3	0.05404976	0.93173516
chr11	70647318	70647377	60	1	2	0	0.30102999	0	1	4	0.05404976	1.26272838
chr11	70647377	70709208	61832	1	2	0	0.61140001	0	0	4	0	1.26272838
chr11	70709208	70778118	68911	3	2	1	0.61140001	0.01091641	0	4	0	0.76005302
chr11	70778118	70853074	74957	2	2	1	0.61140001	0.00478973	0	5	0	1.02643191
chr11	70853074	71090652	237579	3	2	1	0.61140001	0.00204627	0	6	0	1.31360226
chr11	71090652	71155661	65010	2	2	1	0.1575501	0.00204627	2	6	0.1575501	1.31360226
chr11	71155661	71260287	104627	3	2	1	0.1575501	8.47E-04	2	7	0.1575501	1.62048027
chr11	71260287	71286776	26490	1	2	1	0.1575501	0.00204627	2	6	0.1575501	1.31360226
chr11	71286776	71286835	60	1	5	1	0.68214471	0.00204627	2	6	0.02162467	1.31360226
chr11	71286835	71627541	340707	1	3	1	0.30102999	0.00204627	2	6	0.08289318	1.31360226
chr11	71627541	71648032	20492	2	3	2	0.30102999	0.00221948	2	8	0.08289318	1.44210395
chr11	71648032	71690233	42202	2	3	2	0.51676182	0.00221948	1	8	0.02438896	1.44210395
chr11	71690233	71690292	60	1	3	2	0.30102999	0.00221948	2	8	0.08289318	1.44210395
chr11	71690292	71790023	99732	6	2	2	0.30102999	0.00221948	1	8	0.05404976	1.44210395
chr11	71790023	71849521	59499	6	2	2	0.1575501	0.00221948	2	8	0.1575501	1.44210395
chr11	71849521	72013162	163642	6	2	2	0.30102999	0.00221948	1	8	0.05404976	1.44210395
chr11	72013162	72013220	59	1	3	2	0.51676182	0.00221948	1	8	0.02438896	1.44210395
chr11	72013220	72123775	110556	3	2	2	0.30102999	0.00221948	1	8	0.05404976	1.44210395
chr11	72123775	72287622	163848	4	3	2	0.51676182	0.00221948	1	8	0.02438896	1.44210395
chr11	72287622	72370666	83045	5	3	2	0.30102999	0.00221948	2	8	0.08289318	1.44210395
chr11	72370666	72444713	74048	2	3	2	0.30102999	0.00493743	2	7	0.08289318	1.16581773
chr11	72444713	72664318	219606	7	3	2	0.30102999	0.00221948	2	8	0.08289318	1.44210395
chr11	72664318	72664377	60	1	4	2	0.47744371	0.00221948	2	8	0.0429175	1.44210395
chr11	72664377	72754376	90000	3	3	2	0.30102999	0.00221948	2	8	0.08289318	1.44210395
chr11	72754376	72754435	60	1	3	2	0.10122019	0.00221948	4	8	0.30102999	1.44210395
chr11	72754435	72794794	40360	1	3	2	0.17593012	0.00221948	3	8	0.17593012	1.44210395
chr11	72794794	72851213	56420	1	3	2	0.17593012	0.01053319	3	6	0.17593012	0.91219088
chr11	72851213	72939798	88586	2	4	2	0.30102999	0.01053319	3	6	0.10122019	0.91219088
chr11	72939798	72939853	56	1	4	2	0.18734596	0.01053319	4	6	0.18734596	0.91219088
chr11	72939853	72973271	33419	1	3	2	0.10122019	0.02162467	4	5	0.30102999	0.68214471
chr11	72973271	73023132	49862	3	3	2	0.17593012	0.02162467	3	5	0.17593012	0.68214471
chr11	73023132	73094436	71305	3	3	2	0.10122019	0.02162467	4	5	0.30102999	0.68214471
chr11	73094436	73094490	55	1	3	2	0.05670724	0.02162467	5	5	0.45545077	0.68214471
chr11	73094490	73358393	263904	6	3	2	0.10122019	0.0429175	4	4	0.30102999	0.47744371
chr11	73358393	73419514	61122	3	4	2	0.11390336	0.0429175	5	4	0.30102999	0.47744371
chr11	73419514	73471014	51501	2	5	2	0.19510895	0.0429175	5	4	0.19510895	0.47744371
chr11	73471014	73471067	54	1	5	2	0.12309572	0.0429175	6	4	0.30102999	0.47744371
chr11	73471067	73546540	75474	2	5	2	0.19510895	0.0429175	5	4	0.19510895	0.47744371
chr11	73546540	73638725	92186	3	5	2	0.19510895	0.08289318	5	3	0.19510895	0.30102999
chr11	73638725	73691098	52374	1	5	2	0.19510895	0.1575501	5	2	0.19510895	0.1575501
chr11	73691098	73691152	55	1	6	2	0.30102999	0.1575501	5	2	0.12309572	0.1575501
chr11	73691152	73714700	23549	1	6	1	0.30102999	0.05404976	5	2	0.12309572	0.30102999
chr11	73714700	73749791	35092	2	6	1	0.20064824	0.05404976	6	2	0.20064824	0.30102999
chr11	73749791	73749850	60	1	6	1	0.129913	0.02438896	7	3	0.30102999	0.51676182
chr11	73749850	74062476	312627	11	6	1	0.20064824	0.02438896	6	3	0.20064824	0.51676182
chr11	74062476	74166248	103773	3	5	1	0.12309572	0.02438896	6	3	0.30102999	0.51676182
chr11	74166248	74465067	298820	7	5	1	0.07511598	0.02438896	7	3	0.43181735	0.51676182
chr11	74465067	74571559	106493	2	4	1	0.03812622	0.02438896	7	3	0.60763643	0.51676182
chr11	74571559	74571618	60	1	5	1	0.07511598	0.01091641	7	4	0.43181735	0.76005302
chr11	74571618	74670859	99242	2	4	1	0.18734596	0.01091641	4	4	0.18734596	0.76005302
chr11	74670859	74670918	60	1	4	1	0.03812622	0.01091641	7	4	0.60763643	0.76005302
chr11	74670918	74718704	47787	2	4	1	0.18734596	0.01091641	4	4	0.18734596	0.76005302
chr11	74718704	74718763	60	1	4	1	0.06713722	0.01091641	6	4	0.44141547	0.76005302
chr11	74718763	74805354	86592	1	4	1	0.18734596	0.01091641	4	4	0.18734596	0.76005302
chr11	74805354	74909934	104581	4	4	1	0.11390336	0.01091641	5	4	0.30102999	0.76005302
chr11	74909934	74909993	60	1	5	1	0.19510895	0.01091641	5	4	0.19510895	0.76005302
chr11	74909993	75022580	112588	2	3	1	0.05670724	0.01091641	5	4	0.45545077	0.76005302
chr11	75022580	75022639	60	1	5	1	0.19510895	0.01091641	5	4	0.19510895	0.76005302
chr11	75022639	75049688	27050	1	4	1	0.30102999	0.01091641	3	4	0.10122019	0.76005302
chr11	75049688	75081755	32068	1	3	1	0.17593012	0.01091641	3	4	0.17593012	0.76005302
chr11	75081755	75081814	60	1	3	1	0.17593012	0.00204627	3	6	0.17593012	1.31360226
chr11	75081814	75115956	34143	2	3	1	0.17593012	0.00478973	3	5	0.17593012	1.02643191
chr11	75115956	75155183	39228	4	3	1	0.17593012	0.00204627	3	6	0.17593012	1.31360226
chr11	75155183	75173877	18695	1	2	1	0.08289318	0.00204627	3	6	0.30102999	1.31360226
chr11	75173877	75200685	26809	1	2	1	0.08289318	0.00478973	3	5	0.30102999	1.02643191
chr11	75200685	75219298	18614	1	2	1	0.08289318	0.01091641	3	4	0.30102999	0.76005302
chr11	75219298	75281204	61907	2	3	1	0.10122019	0.00478973	4	5	0.30102999	1.02643191
chr11	75281204	75347017	65814	2	4	1	0.18734596	0.00478973	4	5	0.18734596	1.02643191
chr11	75347017	75457332	110316	3	2	1	0.08289318	0.00478973	3	5	0.30102999	1.02643191
chr11	75457332	75512495	55164	3	4	1	0.30102999	0.00478973	3	5	0.10122019	1.02643191
chr11	75512495	75599908	87414	3	4	1	0.18734596	0.00478973	4	5	0.18734596	1.02643191
chr11	75599908	75652857	52950	2	4	1	0.11390336	0.00478973	5	5	0.30102999	1.02643191
chr11	75652857	75727980	75124	1	3	1	0.10122019	0.00478973	4	5	0.30102999	1.02643191
chr11	75727980	75728039	60	1	4	1	0.11390336	0.00478973	5	5	0.30102999	1.02643191
chr11	75728039	75776836	48798	1	4	1	0.18734596	0.01091641	4	4	0.18734596	0.76005302
chr11	75776836	75852635	75800	1	3	1	0.10122019	0.01091641	4	4	0.30102999	0.76005302
chr11	75852635	75852694	60	1	5	1	0.19510895	0.00478973	5	5	0.19510895	1.02643191
chr11	75852694	75899465	46772	1	4	1	0.11390336	0.00478973	5	5	0.30102999	1.02643191
chr11	75899465	75909729	10265	2	4	2	0.06713722	0.02162467	6	5	0.44141547	0.68214471
chr11	75909729	75922308	12580	2	3	2	0.03070643	0.02162467	6	5	0.63695542	0.68214471
chr11	75922308	76072271	149964	1	3	1	0.05670724	0.01091641	5	4	0.45545077	0.76005302
chr11	76072271	76091112	18842	2	5	1	0.19510895	0.00478973	5	5	0.19510895	1.02643191
chr11	76091112	76091165	54	1	5	1	0.19510895	8.47E-04	5	7	0.19510895	1.62048027
chr11	76091165	76128750	37586	1	5	1	0.19510895	0.00204627	5	6	0.19510895	1.31360226
chr11	76128750	76128809	60	1	7	1	0.43181735	0.00204627	5	6	0.07511598	1.31360226
chr11	76128809	76158015	29207	1	5	1	0.19510895	0.01091641	5	4	0.19510895	0.76005302
chr11	76158015	76175100	17086	2	6	1	0.30102999	0.01091641	5	4	0.12309572	0.76005302
chr11	76175100	76207345	32246	1	6	1	0.44141547	0.01091641	4	4	0.06713722	0.76005302
chr11	76207345	76239435	32091	2	6	1	0.44141547	0.00478973	4	5	0.06713722	1.02643191
chr11	76239435	76239494	60	1	6	1	0.20064824	0.00478973	6	5	0.20064824	1.02643191
chr11	76239494	76376842	137349	2	6	1	0.20064824	0.01091641	6	4	0.20064824	0.76005302
chr11	76376842	76508974	132133	2	4	1	0.11390336	0.01091641	5	4	0.30102999	0.76005302
chr11	76508974	76509032	59	1	5	1	0.19510895	0.00204627	5	6	0.19510895	1.31360226
chr11	76509032	76599142	90111	1	4	1	0.11390336	0.00204627	5	6	0.30102999	1.31360226
chr11	76599142	76637590	38449	2	5	1	0.12309572	8.47E-04	6	7	0.30102999	1.62048027

chr11	76637590	76637648	59	1	7	1	0.30102999	8.47E-04	6	7	0.129913	1.62048027
chr11	76637648	76696684	59037	2	4	1	0.06713722	0.00204627	6	6	0.44141547	1.31360226
chr11	76696684	76696743	60	1	6	1	0.20064824	0.00204627	6	6	0.20064824	1.31360226
chr11	76696743	76752340	55598	1	5	1	0.12309572	0.00478973	6	5	0.30102999	1.02643191
chr11	76752340	76752384	45	1	5	1	0.12309572	0.00204627	6	6	0.30102999	1.31360226
chr11	76752384	76777036	24653	1	5	1	0.19510895	0.00204627	5	6	0.19510895	1.31360226
chr11	76777036	76777089	54	1	5	1	0.12309572	8.47E-04	6	7	0.30102999	1.62048027
chr11	76777089	76875656	98568	4	5	1	0.12309572	0.00478973	6	5	0.30102999	1.02643191
chr11	76875656	76875715	60	1	6	1	0.20064824	0.00204627	6	6	0.20064824	1.31360226
chr11	76875715	76894638	18924	1	5	1	0.12309572	0.00204627	6	6	0.30102999	1.31360226
chr11	76894638	76926116	31479	1	5	1	0.12309572	0.02438896	6	3	0.30102999	0.51676182
chr11	76926116	76956469	30354	2	5	1	0.12309572	0.01091641	6	4	0.30102999	0.76005302
chr11	76956469	76956528	60	1	5	1	0.12309572	0.00478973	6	5	0.30102999	1.02643191
chr11	76956528	77035710	79183	3	5	1	0.12309572	0.01091641	6	4	0.30102999	0.76005302
chr11	77035710	77066757	31048	2	5	1	0.12309572	0.00478973	6	5	0.30102999	1.02643191
chr11	77066757	77103361	36605	1	5	1	0.12309572	0.01091641	6	4	0.30102999	0.76005302
chr11	77103361	77103420	60	1	6	1	0.129913	0.01091641	7	4	0.30102999	0.76005302
chr11	77103420	77130017	26598	1	5	1	0.12309572	0.01091641	6	4	0.30102999	0.76005302
chr11	77130017	77130076	60	1	6	1	0.20064824	0.00478973	6	5	0.20064824	1.02643191
chr11	77130076	77160642	30567	1	6	1	0.20064824	0.02438896	6	3	0.20064824	0.51676182
chr11	77160642	77160701	60	1	7	1	0.30102999	0.02438896	6	3	0.129913	0.51676182
chr11	77160701	77243008	82308	1	6	1	0.20064824	0.02438896	6	3	0.20064824	0.51676182
chr11	77243008	77303540	60533	2	6	1	0.20064824	0.01091641	6	4	0.20064824	0.76005302
chr11	77303540	77346779	43240	2	6	1	0.20064824	0.00478973	6	5	0.20064824	1.02643191
chr11	77346779	77458140	111362	3	6	1	0.20064824	0.01091641	6	4	0.20064824	0.76005302
chr11	77458140	77590215	132076	4	5	1	0.19510895	0.02438896	5	3	0.19510895	0.51676182
chr11	77590215	77590274	60	1	6	1	0.20064824	0.01091641	6	4	0.20064824	0.76005302
chr11	77590274	77672088	81815	2	5	1	0.19510895	0.01091641	5	4	0.19510895	0.76005302
chr11	77672088	77775320	103233	4	6	1	0.30102999	0.00478973	5	5	0.12309572	1.02643191
chr11	77775320	77888679	113360	5	6	1	0.30102999	0.00204627	5	6	0.12309572	1.31360226
chr11	77888679	77888738	60	1	6	1	0.20064824	0.00204627	6	6	0.20064824	1.31360226
chr11	77888738	77926688	37951	1	5	1	0.19510895	0.00478973	5	5	0.19510895	1.02643191
chr11	77926688	77970987	44300	1	4	1	0.11390336	0.00478973	5	5	0.30102999	1.02643191
chr11	77970987	78024310	53324	2	4	1	0.11390336	0.00204627	5	6	0.30102999	1.31360226
chr11	78024310	78093754	69445	3	4	1	0.11390336	8.47E-04	5	7	0.30102999	1.62048027
chr11	78093754	78204103	110350	2	4	1	0.11390336	0.00204627	5	6	0.30102999	1.31360226
chr11	78204103	78204162	60	1	4	1	0.06713722	8.47E-04	6	7	0.44141547	1.62048027
chr11	78204162	78364494	160333	3	4	1	0.18734596	8.47E-04	4	7	0.18734596	1.62048027
chr11	78364494	78364553	60	1	4	2	0.18734596	0.00493743	4	7	0.18734596	1.16581773
chr11	78364553	78399909	35357	1	4	2	0.18734596	0.01053319	4	6	0.18734596	0.91219088
chr11	78399909	78460316	60408	1	4	1	0.18734596	0.00478973	4	5	0.18734596	1.02643191
chr11	78460316	78497793	37478	2	4	1	0.18734596	0.00204627	4	6	0.18734596	1.31360226
chr11	78497793	78625598	127806	2	4	1	0.18734596	0.01091641	4	4	0.18734596	0.76005302
chr11	78625598	78625657	60	1	4	1	0.18734596	0.00478973	4	5	0.18734596	1.02643191
chr11	78625657	78748710	123054	2	4	1	0.47744371	0.01091641	2	4	0.0429175	0.76005302
chr11	78748710	78748769	60	1	4	1	0.18734596	0.01091641	4	4	0.18734596	0.76005302
chr11	78748769	78778362	29594	1	3	1	0.10122019	0.01091641	4	4	0.30102999	0.76005302
chr11	78778362	78898511	120150	2	3	1	0.17593012	0.01091641	3	4	0.17593012	0.76005302
chr11	78898511	78898570	60	1	3	1	0.10122019	0.01091641	4	4	0.30102999	0.76005302
chr11	78898570	78993275	94706	2	3	1	0.17593012	0.05404976	3	2	0.17593012	0.30102999
chr11	78993275	78993334	60	1	3	1	0.17593012	0.01091641	3	4	0.17593012	0.76005302
chr11	78993334	79038442	45109	1	3	1	0.30102999	0.01091641	2	4	0.08289318	0.76005302
chr11	79038442	79038501	60	1	3	1	0.30102999	0.00478973	2	5	0.08289318	1.02643191
chr11	79038501	79218731	180231	3	3	0	0.30102999	0	2	2	0.08289318	0.61140001
chr11	79218731	79218790	60	1	3	0	0.30102999	0	2	3	0.08289318	0.93173516
chr11	79218790	79511172	292383	2	3	0	0.30102999	0	2	2	0.08289318	0.61140001
chr11	79511172	79511231	60	1	3	0	0.30102999	0	2	3	0.08289318	0.93173516
chr11	79511231	79715447	204217	1	2	0	0.1575501	0	2	3	0.1575501	0.93173516
chr11	79715447	79715506	60	1	2	0	0.1575501	0	2	4	0.1575501	1.26272838
chr11	79715506	80112382	396877	2	1	0	0.1218695	0	1	0	0.1218695	0
chr11	80112382	80248939	136558	1	1	0	0.30102999	0	0	0	0	0
chr11	80248939	80369045	120107	2	1	0	0.30102999	0	0	1	0	0.30102999
chr11	80369045	81113118	744074	4	0	0	0	0	0	1	0	0.30102999
chr11	81113118	81467310	354193	4	1	0	0.30102999	0	0	4	0	1.26272838
chr11	81467310	81467369	60	1	1	0	0.30102999	0	0	5	0	1.60515106
chr11	81467369	81582093	114725	1	1	0	0.30102999	0	0	4	0	1.26272838
chr11	81582093	81582152	60	1	1	0	0.05404976	0	2	5	0.30102999	1.60515106
chr11	81582152	81669620	87469	1	1	0	0.05404976	0	2	4	0.30102999	1.26272838
chr11	81669620	81771852	102233	2	2	0	0.1575501	0	2	4	0.1575501	1.26272838
chr11	81771852	81771911	60	1	3	0	0.30102999	0	2	4	0.08289318	1.26272838
chr11	81771911	81961087	189177	2	3	0	0.51676182	0	1	4	0.02438896	1.26272838
chr11	81961087	82031482	70396	2	3	0	0.05670724	0	5	4	0.45545077	1.26272838
chr11	82031482	82031541	60	1	3	1	0.05670724	0.00204627	5	6	0.45545077	1.31360226
chr11	82031541	82441687	410147	3	3	1	0.10122019	0.00204627	4	6	0.30102999	1.31360226
chr11	82441687	82443427	1741	2	4	2	0.18734596	0.01053319	4	6	0.18734596	0.91219088
chr11	82443427	82610479	167053	4	3	2	0.10122019	0.01053319	4	6	0.30102999	0.91219088
chr11	82610479	82902701	292223	9	4	2	0.18734596	0.01053319	4	6	0.18734596	0.91219088
chr11	82902701	82952017	49317	2	4	3	0.11390336	0.03070643	5	6	0.30102999	0.63695542
chr11	82952017	82995386	43370	2	4	2	0.11390336	0.01053319	5	6	0.30102999	0.91219088
chr11	82995386	83083730	88345	2	3	2	0.05670724	0.01053319	5	6	0.45545077	0.91219088
chr11	83083730	83083789	60	1	4	2	0.11390336	0.01053319	5	6	0.30102999	0.91219088
chr11	83083789	83193897	110109	4	3	2	0.05670724	0.01053319	5	6	0.45545077	0.91219088
chr11	83193897	83269449	75553	1	3	1	0.05670724	0.00204627	5	6	0.45545077	1.31360226
chr11	83269449	83537688	268240	7	3	2	0.05670724	0.01053319	5	6	0.45545077	0.91219088
chr11	83537688	83537747	60	1	3	2	0.03070643	0.01053319	6	6	0.63695542	0.91219088
chr11	83537747	83695388	157642	3	3	1	0.03070643	0.00204627	6	6	0.63695542	1.31360226
chr11	83695388	83724570	29183	1	3	1	0.05670724	0.00204627	5	6	0.45545077	1.31360226
chr11	83724570	83724629	60	1	3	2	0.05670724	0.01053319	5	6	0.45545077	0.91219088
chr11	83724629	83952707	228079	4	3	1	0.05670724	0.00204627	5	6	0.45545077	1.31360226
chr11	83952707	84003279	50573	2	3	1	0.05670724	0.00478973	5	5	0.45545077	1.02643191
chr11	84003279	84003338	60	1	5	1	0.19510895	0.00204627	5	6	0.19510895	1.31360226
chr11	84003338	84157984	154647	3	2	1	0.02162467	0.00204627	5	6	0.68214471	1.31360226
chr11	84157984	84214703	56720	2	3	1	0.05670724	0.00204627	5	6	0.45545077	1.31360226
chr11	84214703	84291818	77116	4	4	1	0.11390336	0.00204627	5	6	0.30102999	1.31360226
chr11	84291818	84419443	127626	2	3	1	0.05670724	0.00204627	5	6	0.45545077	1.31360226
chr11	84419443	84419502	60	1	4	1	0.11390336	0.00204627	5	6	0.30102999	1.31360226
chr11	84419502	84539606	120105	2	3	1	0.05670724	0.00204627	5	6	0.45545077	1.31360226
chr11	84539606	84539665	60	1	3	1	0.03070643	0.00204627	6	6	0.63695542	1.31360226
chr11	84539665	84634373	94709	2	3	1	0.05670724	0.00204627	5	6	0.45545077	1.31360226
chr11	84634373	84679010	44638	2	3	1	0.03070643	0.00204627	6	6	0.63695542	1.31360226
chr11	84679010	84784173	105164	2	3	1	0.03070643	0.00478973	6	5	0.63695542	1.02643191
chr11	84784173	84784232	60	1	3	1	0.03070643	0.00204627	6	6	0.63695542	1.31360226
chr11	84784232	84923591	139360	2	3	1	0.03070643	0.00478973	6	5	0.63695542	1.02643191
chr11	84923591	84961220	37630	2	3	1	0.01598258	0.00478973	7	5	0.84395715	1.02643191
chr11	84961220	85242437	281218	6	3	1	0.03070643	0.00478973	6	5	0.63695542	1.02643191
chr11	85242437	85242496	60	1	4	1	0.06713722	0.00478973	6	5	0.44141547	1.02643191
chr11	85242496	85305392	62897	1	3	1	0.03070643	0.00478973	6	5	0.63695542	1.02643191
chr11	85305392	85374716	69325	5	3	2	0.03070643	0.02162467	6	5	0.63695542	0.68214471
chr11	85374716	85406154	31439	3	4	2	0.06713722	0.02162467	6	5	0.44141547	0.68214471
chr11	85406154	85425649	19496	1	3	1	0.03070643	0.00478973	6	5	0.63695542	1.02643191
chr11	85425649	85519860	94212	4	3	1	0.03070643	0.00204627	6	6	0.63695542	1.31360226
chr11	85519860	85519919	60	1	4	2	0.06713722	0.01053319	6	6	0.44141547	0.91219088
chr11	85519919	85566636	46718	1	4	1	0.06713722	0.00204627	6	6	0.44141547	1.31360226
chr11	85566636	85584294	17659	1	4	1	0.06713722	0.00478973	6	5	0.44141547	1.02643191
chr11	85584294	85853795	269502	9	4	1	0.06713722	0.01091641	6	4	0.44141547	0.76005302
chr11	85853795	85961116	107322	1	4	1	0.06713722	0.02438896	6	3	0.44141547	0.51676182
chr11	85961116	85988023	26908	1	3	1	0.03070643	0.02438896	6	3	0.63695542	0.51676182
chr11	85988023	85989122	1100	2	3	1	0.03070643	0.01091641	6	4	0.63695542	0.76005302
chr11	85989122	86049739	60618	2	3	1	0.03070643	0.02438896	6	3	0.63695542	0.51676182
chr11	86049739	86049798	60	1	3	1	0.01598258	0.01091641	7	4	0.84395715	0.76005302
chr11	86049798	86111794	61997	1	3	1	0.03070643	0.01091641	6	4	0.63695542	0.76005302
chr11	86111794	86111843	50	1	3	2	0.03070643	0.0429175	6	4	0.63695542	0.47744371
chr11	86111843	86219862	108020	3	3	1	0.03070643	0.02438896	6	3	0.63695542	0.51676182
chr11	86219862	86219921	60	1	3	2	0.03070643	0.08289318	6	3	0.63695542	0.30102999

chr11	86219921	86317846	97926	2	3	1	0.03070643	0.02438896	6	3	0.63695542	0.51676182
chr11	86317846	86374738	56893	3	3	1	0.01598258	0.02438896	7	3	0.84395715	0.51676182
chr11	86374738	86512129	137392	2	3	1	0.05670724	0.02438896	5	3	0.45545077	0.51676182
chr11	86512129	86586936	74808	1	3	1	0.05670724	0.05404976	5	2	0.45545077	0.30102999
chr11	86586936	86665016	78081	3	3	0	0.05670724	0	5	2	0.45545077	0.61140001
chr11	86665016	86978491	313476	6	2	0	0.0429175	0	4	2	0.47744371	0.61140001
chr11	86978491	87144475	165985	2	2	0	0.08289318	0	3	1	0.30102999	0.30102999
chr11	87144475	87144534	60	1	2	0	0.0429175	0	4	2	0.47744371	0.61140001
chr11	87144534	87324690	180157	1	2	0	0.0429175	0	4	1	0.47744371	0.30102999
chr11	87324690	87450865	126176	2	2	0	0.02162467	0	5	1	0.68214471	0.30102999
chr11	87450865	87547184	96320	2	2	0	0.01053319	0	6	2	0.91219088	0.61140001
chr11	87547184	87785796	238613	1	2	0	0.08289318	0	3	2	0.30102999	0.61140001
chr11	87785796	87846529	60734	2	2	0	0.08289318	0	3	3	0.30102999	0.93173516
chr11	87846529	87850595	4067	2	2	1	0.0429175	0.02438896	4	3	0.47744371	0.51676182
chr11	87850595	87898185	47591	1	2	1	0.08289318	0.02438896	3	3	0.30102999	0.51676182
chr11	87898185	88027109	128925	1	2	0	0.08289318	0	3	3	0.30102999	0.93173516
chr11	88027109	88240997	213889	4	3	0	0.17593012	0	3	3	0.17593012	0.93173516
chr11	88240997	88330392	89396	3	3	0	0.10122019	0	4	4	0.30102999	1.26272838
chr11	88330392	88330451	60	1	3	0	0.10122019	0	4	5	0.30102999	1.60515106
chr11	88330451	88360654	30204	1	3	0	0.10122019	0	4	4	0.30102999	1.26272838
chr11	88360654	88690661	330008	6	3	0	0.10122019	0	4	3	0.30102999	0.93173516
chr11	88690661	88758610	67950	2	3	0	0.10122019	0	4	4	0.30102999	1.26272838
chr11	88758610	88903042	144433	2	3	0	0.17593012	0	3	3	0.17593012	0.93173516
chr11	88903042	88961025	57984	2	4	0	0.18734596	0	4	3	0.18734596	0.93173516
chr11	88961025	89014982	53958	2	4	0	0.18734596	0	4	5	0.18734596	1.60515106
chr11	89014982	89177368	162387	4	4	1	0.18734596	0.00478973	4	5	0.18734596	1.02643191
chr11	89177368	89223489	46122	2	4	1	0.18734596	0.01091641	4	4	0.18734596	0.76005302
chr11	89223489	89223548	60	1	4	1	0.18734596	0.00478973	4	5	0.18734596	1.02643191
chr11	89223548	89346831	123284	1	4	0	0.30102999	0	3	5	0.10122019	1.60515106
chr11	89346831	89404085	57255	1	4	0	0.47744371	0	2	5	0.0429175	1.60515106
chr11	89404085	89466583	62499	1	4	0	0.47744371	0	2	4	0.0429175	1.26272838
chr11	89466583	89655866	189284	1	3	0	0.30102999	0	2	4	0.08289318	1.26272838
chr11	89655866	89655925	60	1	3	1	0.17593012	0.01091641	3	4	0.17593012	0.76005302
chr11	89655925	89885908	229984	2	3	0	0.17593012	0	3	4	0.17593012	1.26272838
chr11	89885908	89954996	69089	3	2	0	0.1575501	0	2	3	0.1575501	0.93173516
chr11	89954996	89956504	1509	2	3	0	0.17593012	0	3	4	0.17593012	1.26272838
chr11	89956504	90090388	133885	2	2	0	0.1575501	0	2	3	0.1575501	0.93173516
chr11	90090388	90090447	60	1	2	1	0.1575501	0.01091641	2	4	0.1575501	0.76005302
chr11	90090447	90569659	479213	3	2	0	0.1575501	0	2	3	0.1575501	0.93173516
chr11	90569659	90629499	59841	3	2	1	0.1575501	0.02438896	2	3	0.1575501	0.51676182
chr11	90629499	90851187	221689	1	1	1	0.05404976	0.02438896	2	3	0.30102999	0.51676182
chr11	90851187	90941251	90065	1	1	0	0.05404976	0	2	2	0.30102999	0.61140001
chr11	90941251	90941310	60	1	3	0	0.30102999	0	2	2	0.08289318	0.61140001
chr11	90941310	91344721	403412	2	0	0	0	0	0	1	0	0.30102999
chr11	91344721	91565235	220515	2	1	0	0.1218695	0	1	1	0.1218695	0.30102999
chr11	91565235	91657662	92428	2	2	1	0.30102999	0.05404976	1	2	0.05404976	0.30102999
chr11	91657662	91657721	60	1	3	1	0.51676182	0.05404976	1	2	0.02438896	0.30102999
chr11	91657721	91833562	175842	1	3	1	0.93173516	0.05404976	0	2	0	0.30102999
chr11	91833562	91833621	60	1	3	1	0.93173516	0.02438896	0	3	0	0.51676182
chr11	91833621	92016730	183110	1	2	1	0.61140001	0.02438896	0	3	0	0.51676182
chr11	92016730	92125358	108629	2	2	1	0.61140001	0.00478973	0	5	0	1.02643191
chr11	92125358	92189620	64263	2	2	1	0.08289318	8.47E-04	3	7	0.30102999	1.62048027
chr11	92189620	92293847	104228	2	2	1	0.1575501	8.47E-04	2	7	0.1575501	1.62048027
chr11	92293847	92343682	49836	2	2	1	0.08289318	8.47E-04	3	7	0.30102999	1.62048027
chr11	92343682	92532149	188468	4	2	1	0.1575501	8.47E-04	2	7	0.1575501	1.62048027
chr11	92532149	92624590	92442	3	3	1	0.17593012	8.47E-04	3	7	0.17593012	1.62048027
chr11	92624590	92783972	159383	2	2	1	0.08289318	8.47E-04	3	7	0.30102999	1.62048027
chr11	92783972	92784031	60	1	3	1	0.17593012	8.47E-04	3	7	0.17593012	1.62048027
chr11	92784031	92924867	140837	3	3	1	0.17593012	0.00204627	3	6	0.17593012	1.31360226
chr11	92924867	93065388	140522	3	3	1	0.17593012	8.47E-04	3	7	0.17593012	1.62048027
chr11	93065388	93097455	32068	2	3	1	0.10122019	8.47E-04	4	7	0.30102999	1.62048027
chr11	93097455	93170758	73304	3	2	1	0.08289318	8.47E-04	3	7	0.30102999	1.62048027
chr11	93170758	93268533	97776	3	3	1	0.17593012	8.47E-04	3	7	0.17593012	1.62048027
chr11	93268533	93295475	26943	2	4	1	0.11390336	8.47E-04	5	7	0.30102999	1.62048027
chr11	93295475	93521439	225965	6	4	1	0.11390336	0.00204627	5	6	0.30102999	1.31360226
chr11	93521439	93540074	18636	2	5	1	0.19510895	0.00204627	5	6	0.19510895	1.31360226
chr11	93540074	93914103	374030	8	5	1	0.12309572	0.00204627	6	6	0.30102999	1.31360226
chr11	93914103	94057525	143423	2	5	1	0.19510895	0.00204627	5	6	0.19510895	1.31360226
chr11	94057525	94111573	54049	2	5	1	0.12309572	0.00204627	6	6	0.30102999	1.31360226
chr11	94111573	94212820	101248	4	4	1	0.11390336	0.00204627	5	6	0.30102999	1.31360226
chr11	94212820	94230159	17340	2	5	1	0.12309572	0.00204627	6	6	0.30102999	1.31360226
chr11	94230159	94278998	48840	2	5	1	0.19510895	0.00204627	5	6	0.19510895	1.31360226
chr11	94278998	94301888	22891	2	5	1	0.12309572	0.00204627	6	6	0.30102999	1.31360226
chr11	94301888	94354534	52647	3	5	2	0.12309572	0.01053319	6	6	0.30102999	0.91219088
chr11	94354534	94532550	178017	5	5	1	0.12309572	0.00204627	6	6	0.30102999	1.31360226
chr11	94532550	94532602	53	1	5	2	0.12309572	0.01053319	6	6	0.30102999	0.91219088
chr11	94532602	94563187	30586	1	4	2	0.11390336	0.01053319	5	6	0.30102999	0.91219088
chr11	94563187	94604015	40829	2	4	3	0.11390336	0.03070643	5	6	0.30102999	0.63695542
chr11	94604015	94604074	60	1	4	3	0.06713722	0.03070643	6	6	0.44141547	0.63695542
chr11	94604074	94705730	101657	2	4	2	0.06713722	0.01053319	6	6	0.44141547	0.91219088
chr11	94705730	94801415	95686	3	4	3	0.06713722	0.03070643	6	6	0.44141547	0.63695542
chr11	94801415	94801474	60	1	5	3	0.12309572	0.01598258	6	7	0.30102999	0.84395715
chr11	94801474	94838253	36780	1	5	2	0.12309572	0.00493743	6	7	0.30102999	1.16581773
chr11	94838253	94926706	88454	2	5	1	0.12309572	8.47E-04	6	7	0.30102999	1.62048027
chr11	94926706	95086530	159825	3	5	1	0.12309572	0.00204627	6	6	0.30102999	1.31360226
chr11	95086530	95502376	415847	5	5	2	0.12309572	0.01053319	6	6	0.30102999	0.91219088
chr11	95502376	95555141	52766	2	5	2	0.12309572	0.02162467	6	5	0.30102999	0.68214471
chr11	95555141	95591810	36670	4	5	3	0.12309572	0.05670724	6	5	0.30102999	0.45545077
chr11	95591810	95692390	100581	3	5	2	0.12309572	0.02162467	6	5	0.30102999	0.68214471
chr11	95692390	95711854	19465	2	5	3	0.12309572	0.05670724	6	5	0.30102999	0.45545077
chr11	95711854	95826861	115008	3	5	2	0.12309572	0.02162467	6	5	0.30102999	0.68214471
chr11	95826861	95874545	47685	1	5	2	0.19510895	0.02162467	5	5	0.19510895	0.68214471
chr11	95874545	95874604	60	1	5	3	0.19510895	0.05670724	5	5	0.19510895	0.45545077
chr11	95874604	96243399	368796	9	5	2	0.19510895	0.02162467	5	5	0.19510895	0.68214471
chr11	96243399	96451886	208488	1	4	2	0.11390336	0.02162467	5	5	0.30102999	0.68214471
chr11	96451886	96451945	60	1	4	3	0.11390336	0.05670724	5	5	0.30102999	0.45545077
chr11	96451945	96553328	101384	1	2	2	0.0429175	0.0429175	4	4	0.47744371	0.47744371
chr11	96553328	96689881	136554	1	1	1	0.30102999	0.05404976	0	2	0	0.30102999
chr11	96689881	96847330	157450	1	1	0	0.30102999	0	0	2	0	0.61140001
chr11	96847330	97111804	264475	1	0	0	0	0	0	2	0	0.61140001
chr11	97111804	97529762	417959	2	0	0	0	0	0	1	0	0.30102999
chr11	97854495	97854554	60	1	0	0	0	0	0	1	0	0.30102999
chr11	98487343	98701155	213813	2	1	0	0.30102999	0	0	4	0	1.26272838
chr11	98701155	98701214	60	1	1	3	0.1218695	0.10122019	1	4	0.1218695	0.30102999
chr11	98701214	98774078	72865	1	1	3	0.30102999	0.10122019	0	4	0	0.30102999
chr11	98774078	98921763	147686	3	1	3	0.1218695	0.10122019	1	4	0.1218695	0.30102999
chr11	98921763	98986795	65033	2	1	3	0.02438896	0.05670724	3	5	0.51676182	0.45545077
chr11	98986795	99048665	61871	1	1	3	0.05404976	0.05670724	2	5	0.30102999	0.45545077
chr11	99048665	99097282	48618	2	1	3	0.02438896	0.05670724	3	5	0.51676182	0.45545077
chr11	99097282	99097341	60	1	2	3	0.08289318	0.05670724	3	5	0.30102999	0.45545077
chr11	99097341	99154240	56900	1	1	3	0.02438896	0.05670724	3	5	0.51676182	0.45545077
chr11	99154240	99154299	60	1	1	3	0.01091641	0.05670724	4	5	0.76005302	0.45545077
chr11	99154299	99293131	138833	3	1	3	0.02438896	0.05670724	3	5	0.51676182	0.45545077
chr11	99293131	99360114	66984	1	1	3	0.02438896	0.10122019	3	4	0.51676182	0.30102999
chr11	99360114	99396183	36070	2	2	3	0.0429175	0.10122019	4	4	0.47744371	0.30102999
chr11	99396183	99615737	219555	4	2	3	0.0429175	0.05670724	4	5	0.47744371	0.45545077
chr11	99615737	100000125	384389	7	2	3	0.0429175	0.10122019	4	4	0.47744371	0.30102999
chr11	100000125	100068799	68675	2	2	3	0.0429175	0.05670724	4	5	0.47744371	0.45545077
chr11	100068799	100068858	60	1	2	3	0.0429175	0.01598258	4	7	0.47744371	0.84395715
chr11	100068858	100103835	34978	1	2	3	0.0429175	0.03070643	4	6	0.47744371	0.63695542
chr11	100103835	100211306	107472	2	1	3	0.01091641	0.03070643	4	6	0.76005302	0.63695542
chr11	100211306	100305324	94019	1	0	3	0	0.03070643	4	6	1.26272838	0.63695542
chr11	100305324	100665956	360633	3	0	3	0	0.03070643	3	6	0.93173516	0.63695542
chr11	100665956	100666015	60	1	0	3	0	0.0079614	3	8	0.93173516	1.07548421

chr11	100666015	100713156	47142	1	0	3	0	0.01598258	2	7	0.61140001	0.84395715
chr11	100713156	100786016	72861	1	0	3	0	0.03070643	2	6	0.61140001	0.63695542
chr11	100786016	100786075	60	1	0	4	0	0.03812622	2	7	0.61140001	0.60763643
chr11	100786075	100845021	58947	1	0	3	0	0.01598258	2	7	0.61140001	0.84395715
chr11	100845021	100845080	60	1	1	4	0.05404976	0.02074938	2	8	0.30102999	0.79906872
chr11	100845080	100909288	64209	2	1	3	0.05404976	0.0079614	2	8	0.30102999	1.07548421
chr11	100909288	100917864	8577	1	1	3	0.05404976	0.03070643	2	6	0.30102999	0.63695542
chr11	100917864	100949346	31483	1	1	3	0.05404976	0.05670724	2	5	0.30102999	0.45545077
chr11	100949346	100949405	60	1	1	3	0.02438896	0.05670724	3	5	0.51676182	0.45545077
chr11	100949405	101000675	51271	1	1	2	0.02438896	0.02162467	3	5	0.51676182	0.68214471
chr11	101000675	101024341	23667	2	1	3	0.02438896	0.03070643	3	6	0.51676182	0.63695542
chr11	101024341	101127880	103540	1	1	2	0.02438896	0.01053319	3	6	0.51676182	0.91219088
chr11	101127880	101127939	60	1	2	4	0.08289318	0.03812622	3	7	0.30102999	0.60763643
chr11	101127939	101323715	195777	1	2	3	0.08289318	0.01598258	3	7	0.30102999	0.84395715
chr11	101323715	101374792	51078	3	3	5	0.10122019	0.07511598	4	7	0.30102999	0.43181735
chr11	101374792	101563332	188541	2	2	5	0.08289318	0.12309572	3	6	0.30102999	0.30102999
chr11	101563332	101659057	95726	2	2	5	0.0429175	0.12309572	4	6	0.47744371	0.30102999
chr11	101659057	101659116	60	1	3	8	0.05670724	0.30102999	5	7	0.45545077	0.13499366
chr11	101659116	101761480	102365	1	3	5	0.05670724	0.12309572	5	6	0.45545077	0.30102999
chr11	101761480	101765744	4265	2	4	6	0.06713722	0.20064824	6	6	0.44141547	0.20064824
chr11	101765744	101786833	21090	2	4	6	0.06713722	0.30102999	6	5	0.44141547	0.12309572
chr11	101786833	101857794	70962	2	4	7	0.06713722	0.20469099	6	7	0.44141547	0.20469099
chr11	101857794	101987284	129491	3	4	5	0.06713722	0.07511598	6	7	0.44141547	0.43181735
chr11	101987284	102080256	92973	2	3	4	0.03070643	0.06713722	6	6	0.63695542	0.44141547
chr11	102080256	102125285	45030	3	3	5	0.03070643	0.12309572	6	6	0.63695542	0.30102999
chr11	102125285	102125344	60	1	3	7	0.03070643	0.30102999	6	6	0.63695542	0.129913
chr11	102125344	102268518	143175	8	3	6	0.03070643	0.20064824	6	6	0.63695542	0.20064824
chr11	102268518	102297802	29285	1	3	6	0.03070643	0.30102999	6	5	0.63695542	0.12309572
chr11	102297802	102319527	21726	2	3	7	0.03070643	0.43181735	6	5	0.63695542	0.07511598
chr11	102319527	102369773	50247	2	3	7	0.03070643	0.30102999	6	6	0.63695542	0.129913
chr11	102369773	102369832	60	1	3	8	0.03070643	0.4250187	6	6	0.63695542	0.08122616
chr11	102369832	102391461	21630	2	2	8	0.01053319	0.58747015	6	5	0.91219088	0.04407651
chr11	102391461	102395665	4205	2	3	8	0.03070643	0.4250187	6	6	0.63695542	0.08122616
chr11	102395665	102466901	71237	2	3	7	0.03070643	0.30102999	6	6	0.63695542	0.129913
chr11	102466901	102494630	27730	1	3	5	0.03070643	0.12309572	6	6	0.63695542	0.30102999
chr11	102494630	102562671	68042	2	3	6	0.03070643	0.20064824	6	6	0.63695542	0.20064824
chr11	102562671	102645479	82809	2	3	6	0.03070643	0.30102999	6	5	0.63695542	0.12309572
chr11	102645479	102651241	5763	3	3	6	0.03070643	0.20064824	6	6	0.63695542	0.20064824
chr11	102651241	102755174	103934	7	3	5	0.03070643	0.12309572	6	6	0.63695542	0.30102999
chr11	102755174	102815341	60168	2	3	4	0.03070643	0.06713722	6	6	0.63695542	0.44141547
chr11	102815341	103106444	291104	6	3	4	0.03070643	0.11390336	6	5	0.63695542	0.30102999
chr11	103106444	103124089	17646	2	3	5	0.03070643	0.19510895	6	5	0.63695542	0.19510895
chr11	103124089	103191875	67787	2	3	4	0.03070643	0.11390336	6	5	0.63695542	0.30102999
chr11	103191875	103306780	114906	3	2	4	0.01053319	0.11390336	6	5	0.91219088	0.30102999
chr11	103306780	103339357	32578	1	2	4	0.01053319	0.18734596	6	4	0.91219088	0.18734596
chr11	103339357	103620440	281084	2	2	2	0.01053319	0.08289318	6	3	0.91219088	0.30102999
chr11	103620440	103829655	209216	2	1	0	0.01091641	0	4	2	0.76005302	0.61140001
chr11	103829655	103914833	85179	3	1	0	0.00478973	0	5	3	1.02643191	0.93173516
chr11	103914833	103955263	40431	1	1	0	0.01091641	0	4	3	0.76005302	0.93173516
chr11	103955263	104050764	95502	3	1	2	0.01091641	0.0429175	4	4	0.76005302	0.47744371
chr11	104050764	104050823	60	1	1	2	0.00478973	0.02162467	5	5	1.02643191	0.68214471
chr11	104050823	104135377	84555	1	1	2	0.01091641	0.0429175	4	4	0.76005302	0.47744371
chr11	104135377	104437152	301776	2	1	2	0.02438896	0.0429175	3	4	0.51676182	0.47744371
chr11	104437152	104437211	60	1	2	3	0.08289318	0.10122019	3	4	0.30102999	0.30102999
chr11	104437211	104757909	320699	2	1	3	0.02438896	0.10122019	3	4	0.51676182	0.30102999
chr11	104757909	104820093	62185	4	2	4	0.08289318	0.11390336	3	5	0.30102999	0.30102999
chr11	104820093	104869033	48941	1	2	3	0.08289318	0.10122019	3	4	0.30102999	0.30102999
chr11	104869033	104962273	93241	4	2	3	0.0429175	0.10122019	4	4	0.47744371	0.30102999
chr11	104962273	105068926	106654	4	2	4	0.0429175	0.11390336	4	5	0.47744371	0.30102999
chr11	105068926	105068985	60	1	2	5	0.0429175	0.19510895	4	5	0.47744371	0.19510895
chr11	105068985	105138232	69248	1	2	4	0.0429175	0.18734596	4	4	0.47744371	0.18734596
chr11	105138232	105252612	114381	3	2	4	0.0429175	0.11390336	4	5	0.47744371	0.30102999
chr11	105252612	105252671	60	1	2	5	0.0429175	0.19510895	4	5	0.47744371	0.19510895
chr11	105252671	105510858	258188	3	2	4	0.0429175	0.11390336	4	5	0.47744371	0.30102999
chr11	105510858	105510917	60	1	2	5	0.0429175	0.19510895	4	5	0.47744371	0.19510895
chr11	105510917	105561988	51072	1	2	5	0.08289318	0.19510895	3	5	0.30102999	0.19510895
chr11	105561988	105623705	61718	2	2	6	0.08289318	0.30102999	3	5	0.30102999	0.12309572
chr11	105623705	105623763	59	1	3	6	0.05670724	0.30102999	5	5	0.45545077	0.12309572
chr11	105623763	105730621	106859	2	3	5	0.10122019	0.19510895	4	5	0.30102999	0.19510895
chr11	105730621	105730680	60	1	3	5	0.03070643	0.19510895	6	5	0.63695542	0.19510895
chr11	105730680	105850492	119813	2	3	5	0.10122019	0.19510895	4	5	0.30102999	0.19510895
chr11	105850492	105922488	71997	1	3	5	0.51676182	0.19510895	1	5	0.02438896	0.19510895
chr11	105922488	105947397	24910	2	3	5	0.51676182	0.30102999	1	4	0.02438896	0.11390336
chr11	105947397	105961442	14046	2	3	5	0.51676182	0.19510895	1	5	0.02438896	0.19510895
chr11	105961442	105961501	60	1	3	5	0.30102999	0.19510895	2	5	0.08289318	0.19510895
chr11	105961501	106031011	69511	1	2	3	0.30102999	0.05670724	1	5	0.05404976	0.45545077
chr11	106031011	106267255	236245	1	2	2	0.30102999	0.1575501	1	2	0.05404976	0.1575501
chr11	106267255	106267314	60	1	2	2	0.1575501	0.1575501	2	2	0.1575501	0.1575501
chr11	106267314	106493972	226659	1	2	2	0.1575501	0.30102999	2	1	0.1575501	0.05404976
chr11	106493972	106557833	63862	2	2	2	0.1575501	0.08289318	2	3	0.1575501	0.30102999
chr11	106557833	106558195	363	2	2	2	0.08289318	0.0429175	3	4	0.30102999	0.47744371
chr11	106558195	106617145	58951	1	2	2	0.1575501	0.1575501	2	2	0.1575501	0.1575501
chr11	106617145	106681146	64002	2	2	3	0.1575501	0.30102999	2	2	0.1575501	0.08289318
chr11	106681146	106681205	60	1	2	4	0.1575501	0.30102999	2	3	0.1575501	0.10122019
chr11	106681205	106737199	55995	1	2	3	0.1575501	0.30102999	2	2	0.1575501	0.08289318
chr11	106737199	106810465	73267	1	2	2	0.1575501	0.1575501	2	2	0.1575501	0.1575501
chr11	106810465	106810524	60	1	2	2	0.08289318	0.1575501	3	2	0.30102999	0.1575501
chr11	106810524	106874508	63985	1	2	2	0.1575501	0.1575501	2	2	0.1575501	0.1575501
chr11	106874508	106959465	84958	1	2	1	0.1575501	0.05404976	2	2	0.1575501	0.30102999
chr11	106959465	107134849	175385	2	2	1	0.08289318	0.02438896	3	3	0.30102999	0.51676182
chr11	107134849	107224406	89558	2	2	2	0.08289318	0.08289318	3	3	0.30102999	0.30102999
chr11	107224406	107260943	36538	2	2	2	0.08289318	0.0429175	3	4	0.30102999	0.47744371
chr11	107260943	107409517	148575	3	2	2	0.08289318	0.08289318	3	3	0.30102999	0.30102999
chr11	107409517	107463643	54127	3	2	3	0.08289318	0.10122019	3	4	0.30102999	0.30102999
chr11	107463643	107463702	60	1	2	4	0.02162467	0.18734596	5	4	0.68214471	0.18734596
chr11	107463702	107580017	116316	4	2	3	0.0429175	0.17593012	4	3	0.47744371	0.17593012
chr11	107580017	107661943	81927	3	2	3	0.02162467	0.17593012	5	3	0.68214471	0.17593012
chr11	107661943	107662002	60	1	2	4	0.02162467	0.30102999	5	3	0.68214471	0.10122019
chr11	107662002	107686511	24510	1	2	3	0.02162467	0.17593012	5	3	0.68214471	0.17593012
chr11	107686511	107729810	43300	4	2	3	0.02162467	0.10122019	5	4	0.68214471	0.30102999
chr11	107729810	107777703	47894	2	3	4	0.05670724	0.18734596	5	4	0.45545077	0.18734596
chr11	107777703	107833574	55872	1	3	2	0.05670724	0.0429175	5	4	0.45545077	0.47744371
chr11	107833574	107959353	125780	3	3	1	0.05670724	0.02438896	5	3	0.45545077	0.51676182
chr11	107959353	108178662	219310	7	3	0	0.05670724	0	5	3	0.45545077	0.93173516
chr11	108178662	108199793	21132	1	2	0	0.02162467	0	5	3	0.68214471	0.93173516
chr11	108199793	108199852	60	1	2	0	0.02162467	0	5	4	0.68214471	1.26272838
chr11	108199852	108592668	392817	12	2	0	0.02162467	0	5	3	0.68214471	0.93173516
chr11	108592668	108673795	81128	2	2	0	0.02162467	0	5	2	0.68214471	0.61140001
chr11	108673795	108728694	54900	1	2	0	0.0429175	0	4	2	0.47744371	0.61140001
chr11	108728694	108844948	116255	4	2	0	0.0429175	0	4	3	0.47744371	0.93173516
chr11	108844948	108845007	60	1	2	0	0.02162467	0	5	3	0.68214471	0.93173516
chr11	108845007	109021349	176343	2	2	0	0.0429175	0	4	3	0.47744371	0.93173516
chr11	109021349	109021408	60	1	2	0	0.02162467	0	5	3	0.68214471	0.93173516
chr11	109021408	109181138	159731	1	2	0	0.02162467	0	5	1	0.68214471	0.30102999
chr11	109181138	109270529	89392	1	2	0	0.0429175	0	4	1	0.47744371	0.30102999
chr11	109270529	109363882	93354	1	2	0	0.08289318	0	3	0	0.30102999	0
chr11	109363882	109529329	165448	2	2	0	0.30102999	0	1	0	0.05404976	0
chr11	109529329	109529388	60	1	2	0	0.30102999	0	1	1	0.05404976	0.30102999
chr11	109529388	109702636	173249	1	2	0	0.30102999	0	1	0	0.05404976	0
chr11	109702636	109830066	127431	2	2	0	0.1575501	0	2	0	0.1575501	0
chr11	109830066	109928090	98025	2	2	1	0.1575501	0.30102999	2	0	0.1575501	0
chr11	109928090	110003380	75291	2	2	1	0.1575501	0.1218695	2	1	0.1575501	0.1218695
chr11	110003380	110159601	156222	4	2	1	0.1575501	0.30102999	2	0	0.1575501	0
chr11	110159601	110404638	245038	5	2	1	0.1575501	0.1218695	2	1	0.1575501	0.1218695

chr11	110404638	110404697	60	1	2	1	0.08289318	0.05404976	3	2	0.30102999	0.30102999
chr11	110404697	110508203	103507	3	2	1	0.08289318	0.1218695	3	1	0.30102999	0.1218695
chr11	110508203	110564363	56161	1	2	0	0.08289318	0	3	1	0.30102999	0.30102999
chr11	110564363	110772180	207818	3	2	0	0.0429175	0	4	2	0.47744371	0.61140001
chr11	110772180	110846327	74148	2	2	1	0.01053319	0.05404976	6	2	0.91219088	0.30102999
chr11	110846327	110967252	120926	2	2	2	0.01053319	0.1575501	6	2	0.91219088	0.1575501
chr11	110967252	110967311	60	1	2	2	0.00221948	0.08289318	8	3	1.44210395	0.30102999
chr11	110967311	111137190	169880	1	2	1	0.00221948	0.05404976	8	2	1.44210395	0.30102999
chr11	111137190	111171889	34700	2	2	1	9.54E-04	0.05404976	9	2	1.74076927	0.30102999
chr11	111171889	111249253	77365	4	2	2	9.54E-04	0.08289318	9	3	1.74076927	0.30102999
chr11	111249253	111386795	137543	3	2	2	9.54E-04	0.1575501	9	2	1.74076927	0.1575501
chr11	111386795	111465924	79130	2	2	2	0.00221948	0.1575501	8	2	1.44210395	0.1575501
chr11	111465924	111465983	60	1	2	2	9.54E-04	0.1575501	9	2	1.74076927	0.1575501
chr11	111465983	111612172	146190	3	2	1	9.54E-04	0.05404976	9	2	1.74076927	0.30102999
chr11	111612172	111756483	144312	7	2	2	9.54E-04	0.1575501	9	2	1.74076927	0.1575501
chr11	111756483	111846294	89812	4	2	2	0.00221948	0.1575501	8	2	1.44210395	0.1575501
chr11	111846294	111846353	60	1	2	2	9.54E-04	0.08289318	9	3	1.74076927	0.30102999
chr11	111846353	111941226	94874	4	2	2	0.00221948	0.08289318	8	3	1.44210395	0.30102999
chr11	111941226	112085865	144640	10	2	2	0.00493743	0.1575501	7	2	1.16581773	0.1575501
chr11	112085865	112099380	13516	2	2	2	0.00493743	0.08289318	7	3	1.16581773	0.30102999
chr11	112099380	112168030	68651	2	2	2	0.00493743	0.1575501	7	2	1.16581773	0.1575501
chr11	112168030	112375293	207264	4	2	2	0.00493743	0.08289318	7	3	1.16581773	0.30102999
chr11	112375293	112440131	64839	1	2	2	0.00493743	0.1575501	7	2	1.16581773	0.1575501
chr11	112440131	112499131	59001	1	2	1	0.00493743	0.05404976	7	2	1.16581773	0.30102999
chr11	112499131	112563267	64137	2	2	1	0.00493743	0.02438896	7	3	1.16581773	0.51676182
chr11	112563267	112563326	60	1	2	2	0.00493743	0.08289318	7	3	1.16581773	0.30102999
chr11	112563326	112879364	316039	3	2	2	0.02162467	0.1575501	5	2	0.68214471	0.1575501
chr11	112879364	112879423	60	1	2	3	0.02162467	0.30102999	5	2	0.68214471	0.08289318
chr11	112879423	112942002	62580	1	1	2	0.00478973	0.1575501	5	2	1.02643191	0.1575501
chr11	112942002	113000227	58226	1	1	1	0.00478973	0.1218695	5	1	1.02643191	0.1218695
chr11	113000227	113111526	111300	2	1	1	0.02438896	0.1218695	3	1	0.51676182	0.1218695
chr11	113111526	113111579	54	1	1	3	0.02438896	0.51676182	3	1	0.51676182	0.02438896
chr11	113111579	113186964	75386	4	1	3	0.05404976	0.51676182	2	1	0.30102999	0.02438896
chr11	113186964	113236999	50036	3	1	3	0.05404976	0.30102999	2	2	0.30102999	0.08289318
chr11	113236999	113293362	56364	3	1	4	0.02438896	0.47744371	3	2	0.51676182	0.0429175
chr11	113293362	113475281	181920	3	1	3	0.02438896	0.30102999	3	2	0.51676182	0.08289318
chr11	113475281	113475340	60	1	1	4	0.02438896	0.18734596	3	4	0.51676182	0.18734596
chr11	113475340	113523276	47937	1	1	4	0.02438896	0.30102999	3	3	0.51676182	0.10122019
chr11	113523276	113566108	42833	1	1	3	0.02438896	0.17593012	3	3	0.51676182	0.17593012
chr11	113566108	113612431	46324	2	1	3	0.02438896	0.10122019	3	4	0.51676182	0.30102999
chr11	113612431	113639596	27166	2	1	4	0.02438896	0.18734596	3	4	0.51676182	0.18734596
chr11	113639596	113856922	217327	8	1	3	0.02438896	0.17593012	3	3	0.51676182	0.17593012
chr11	113856922	113955796	98875	2	1	3	0.05404976	0.17593012	2	3	0.30102999	0.17593012
chr11	113955796	113976501	20706	2	1	3	0.02438896	0.10122019	3	4	0.51676182	0.30102999
chr11	113976501	114030160	53660	2	1	3	0.05404976	0.17593012	2	3	0.30102999	0.17593012
chr11	114030160	114130573	100414	3	1	2	0.05404976	0.08289318	2	3	0.30102999	0.30102999
chr11	114130573	114167383	36811	1	1	1	0.05404976	0.02438896	2	3	0.30102999	0.51676182
chr11	114167383	114167442	60	1	1	2	0.05404976	0.08289318	2	3	0.30102999	0.30102999
chr11	114167442	114269885	102444	1	1	2	0.05404976	0.1575501	2	2	0.30102999	0.1575501
chr11	114269885	114316688	46804	2	1	2	0.02438896	0.1575501	3	2	0.51676182	0.1575501
chr11	114316688	114316747	60	1	1	4	0.02438896	0.47744371	3	2	0.51676182	0.0429175
chr11	114316747	114398562	81816	1	1	2	0.02438896	0.1575501	3	2	0.51676182	0.1575501
chr11	114398562	114465471	66910	3	2	2	0.08289318	0.08289318	3	3	0.30102999	0.30102999
chr11	114465471	114556533	91063	1	1	1	0.02438896	0.02438896	3	3	0.51676182	0.51676182
chr11	114556533	114653661	97129	2	1	1	0.02438896	0.05404976	3	2	0.51676182	0.30102999
chr11	114653661	114845534	191874	1	0	0	0	0	3	1	0.93173516	0.30102999
chr11	114845534	115046874	201341	1	0	0	0	0	3	0	0.93173516	0
chr11	115046874	115046933	60	1	1	0	0.01091641	0	4	0	0.76005302	0
chr11	115046933	115109269	62337	3	1	0	0.02438896	0	3	0	0.51676182	0
chr11	115109269	115141188	31920	2	2	0	0.0429175	0	4	0	0.47744371	0
chr11	115141188	115186132	44945	2	2	0	0.02162467	0	5	0	0.68214471	0
chr11	115186132	115287204	101073	2	2	0	0.0429175	0	4	0	0.47744371	0
chr11	115287204	115334147	46944	1	2	0	0.08289318	0	3	0	0.30102999	0
chr11	115334147	115394072	59926	3	2	0	0.0429175	0	4	0	0.47744371	0
chr11	115394072	115518388	124317	1	1	0	0.02438896	0	3	0	0.51676182	0
chr11	115518388	115661137	142750	2	1	0	0.02438896	0	3	1	0.51676182	0.30102999
chr11	115661137	115915689	254553	2	1	1	0.02438896	0.1218695	3	1	0.51676182	0.1218695
chr11	115915689	116053868	138180	2	1	1	0.01091641	0.1218695	4	1	0.76005302	0.1218695
chr11	116053868	116053927	60	1	1	3	0.00478973	0.51676182	5	1	1.02643191	0.02438896
chr11	116053927	116326867	272941	2	1	1	0.00478973	0.1218695	5	1	1.02643191	0.1218695
chr11	116326867	116326926	60	1	2	1	0.02162467	0.1218695	5	1	0.68214471	0.1218695
chr11	116326926	116462497	135572	1	2	1	0.0429175	0.30102999	4	0	0.47744371	0
chr11	116462497	116623317	160821	2	2	3	0.0429175	0.30102999	4	2	0.47744371	0.08289318
chr11	116623317	116655121	31805	3	3	3	0.05670724	0.17593012	5	3	0.45545077	0.17593012
chr11	116655121	116660613	5493	2	3	3	0.05670724	0.03070643	5	6	0.45545077	0.63695542
chr11	116660613	116728594	67982	2	3	3	0.05670724	0.05670724	5	5	0.45545077	0.45545077
chr11	116728594	116746672	18079	1	3	3	0.05670724	0.10122019	5	4	0.45545077	0.30102999
chr11	116746672	116796887	50216	2	3	3	0.05670724	0.05670724	5	5	0.45545077	0.45545077
chr11	116796887	116824812	27926	2	3	3	0.05670724	0.03070643	5	6	0.45545077	0.63695542
chr11	116824812	116884978	60167	1	3	3	0.05670724	0.05670724	5	5	0.45545077	0.45545077
chr11	116884978	116934241	49264	1	3	3	0.05670724	0.10122019	5	4	0.45545077	0.30102999
chr11	116934241	116973424	39184	1	3	3	0.05670724	0.17593012	5	3	0.45545077	0.17593012
chr11	116973424	116973483	60	1	3	4	0.05670724	0.11390336	5	5	0.45545077	0.30102999
chr11	116973483	117015663	42181	1	3	3	0.05670724	0.05670724	5	5	0.45545077	0.45545077
chr11	117015663	117051076	35414	3	3	3	0.05670724	0.01598258	5	7	0.45545077	0.84395715
chr11	117051076	117067450	16375	1	3	3	0.05670724	0.03070643	5	6	0.45545077	0.63695542
chr11	117067450	117067496	47	1	3	4	0.05670724	0.06713722	5	6	0.45545077	0.44141547
chr11	117067496	117101118	33623	3	3	3	0.05670724	0.03070643	5	6	0.45545077	0.63695542
chr11	117101118	117101170	53	1	3	4	0.05670724	0.03812622	5	7	0.45545077	0.60763643
chr11	117101170	117182655	81486	2	3	3	0.05670724	0.03070643	5	6	0.45545077	0.63695542
chr11	117182655	117209339	26685	3	4	3	0.11390336	0.03070643	5	6	0.30102999	0.63695542
chr11	117209339	117233213	23875	2	4	3	0.06713722	0.03070643	6	6	0.44141547	0.63695542
chr11	117233213	117233262	50	1	4	4	0.06713722	0.03812622	6	7	0.44141547	0.60763643
chr11	117233262	117298552	65291	3	4	4	0.06713722	0.06713722	6	6	0.44141547	0.44141547
chr11	117298552	117343785	45234	1	4	4	0.06713722	0.11390336	6	5	0.44141547	0.30102999
chr11	117343785	117588742	244958	7	4	5	0.06713722	0.19510895	6	5	0.44141547	0.19510895
chr11	117588742	117642607	53866	2	4	5	0.03812622	0.19510895	7	5	0.60763643	0.19510895
chr11	117642607	117642666	60	1	4	5	0.02074938	0.19510895	8	5	0.79906872	0.19510895
chr11	117642666	117729021	86356	3	4	5	0.02074938	0.45545077	8	3	0.79906872	0.05670724
chr11	117729021	117788167	59147	1	4	4	0.02074938	0.30102999	8	3	0.79906872	0.10122019
chr11	117788167	117869275	81109	1	4	3	0.03812622	0.17593012	7	3	0.60763643	0.17593012
chr11	117869275	117869334	60	1	4	4	0.03812622	0.30102999	7	3	0.60763643	0.10122019
chr11	117869334	117914757	45424	1	4	3	0.03812622	0.17593012	7	3	0.60763643	0.17593012
chr11	117914757	117938790	24034	2	4	3	0.03812622	0.10122019	7	4	0.60763643	0.30102999
chr11	117938790	117938849	60	1	5	4	0.07511598	0.11390336	7	5	0.43181735	0.30102999
chr11	117938849	117951830	12982	1	4	4	0.03812622	0.11390336	7	5	0.60763643	0.30102999
chr11	117951830	117981575	29746	2	4	4	0.02074938	0.11390336	8	5	0.79906872	0.30102999
chr11	117981575	118005329	23755	2	4	5	0.02074938	0.19510895	8	5	0.79906872	0.19510895
chr11	118005329	118005388	60	1	5	6	0.04407651	0.30102999	8	5	0.58747015	0.12309572
chr11	118005388	118040771	35384	1	5	5	0.04407651	0.19510895	8	5	0.58747015	0.19510895
chr11	118040771	118089334	48564	2	5	5	0.07511598	0.19510895	7	5	0.43181735	0.19510895
chr11	118089334	118098007	8674	2	5	6	0.02473314	0.20064824	9	6	0.76806864	0.20064824
chr11	118098007	118107924	9918	1	5	6	0.04407651	0.20064824	8	6	0.58747015	0.20064824
chr11	118107924	118128037	20114	1	5	5	0.04407651	0.19510895	8	5	0.58747015	0.19510895
chr11	118128037	118180765	52729	1	5	5	0.07511598	0.19510895	7	5	0.43181735	0.19510895
chr11	118180765	118180824	60	1	5	6	0.07511598	0.20064824	7	6	0.43181735	0.20064824
chr11	118180824	118218146	37323	3	5	6	0.07511598	0.30102999	7	5	0.43181735	0.12309572
chr11	118218146	118230491	12346	2	5	7	0.07511598	0.30102999	7	6	0.43181735	0.129913
chr11	118230491	118250287	19797	1	5	7	0.07511598	0.43181735	7	5	0.43181735	0.07511598
chr11	118250287	118250340	54	1	5	7	0.04407651	0.43181735	8	5	0.58747015	0.07511598
chr11	118250340	118277831	27492	1	5	7	0.04407651	0.60763643	8	4	0.58747015	0.03812622
chr11	118277831	118304830	27000	2	5	7	0.04407651	0.43181735	8	5	0.58747015	0.07511598
chr11	118304830	118339458	34629	3	5	7	0.04407651	0.30102999	8	6	0.58747015	0.129913
chr11	118339458	118448990	109533	8	5	7	0.04407651	0.20469099	8	7	0.58747015	0.20469099

chr11	118448990	118517876	68887	3	5	7	0.04407651	0.30102999	8	6	0.58747015	0.129913
chr11	118517876	118606840	88965	2	4	7	0.02074938	0.30102999	8	6	0.79906872	0.129913
chr11	118606840	118660474	53635	2	4	7	0.01077081	0.30102999	9	6	1.01542894	0.129913
chr11	118660474	118689267	28794	2	5	7	0.02473314	0.30102999	9	6	0.76806864	0.129913
chr11	118689267	118828826	139560	3	5	6	0.02473314	0.20064824	9	6	0.76806864	0.20064824
chr11	118828826	118894302	65477	2	4	6	0.01077081	0.20064824	9	6	1.01542894	0.20064824
chr11	118894302	118898846	4545	2	4	6	0.01077081	0.30102999	9	5	1.01542894	0.12309572
chr11	118898846	118969374	70529	7	4	6	0.01077081	0.20064824	9	6	1.01542894	0.20064824
chr11	118969374	118998204	28831	3	4	5	0.01077081	0.12309572	9	6	1.01542894	0.30102999
chr11	118998204	119180317	182114	10	4	6	0.01077081	0.20064824	9	6	1.01542894	0.20064824
chr11	119180317	119217236	36920	4	3	6	0.00378107	0.20064824	9	6	1.33111237	0.20064824
chr11	119217236	119242814	25579	1	3	6	0.0079614	0.30102999	8	5	1.07548421	0.12309572
chr11	119242814	119288885	46072	2	4	6	0.02074938	0.30102999	8	5	0.79906872	0.12309572
chr11	119288885	119293281	4397	2	4	6	0.01077081	0.20064824	9	6	1.01542894	0.20064824
chr11	119293281	119332166	38886	1	4	5	0.01077081	0.19510895	9	5	1.01542894	0.19510895
chr11	119332166	119332225	60	1	5	5	0.02473314	0.12309572	9	6	0.76806864	0.30102999
chr11	119332225	119446154	113930	1	4	2	0.03812622	0.01053319	7	6	0.60763643	0.91219088
chr11	119446154	119539406	93253	1	3	2	0.01598258	0.01053319	7	6	0.84395715	0.91219088
chr11	119539406	119539465	60	1	3	4	0.01598258	0.03812622	7	7	0.84395715	0.60763643
chr11	119539465	119568149	28685	1	2	4	0.00493743	0.03812622	7	7	1.16581773	0.60763643
chr11	119568149	119596739	28591	1	1	4	8.47E-04	0.03812622	7	7	1.62048027	0.60763643
chr11	119596739	119821294	224556	3	1	4	8.47E-04	0.02074938	7	8	1.62048027	0.79906872
chr11	119821294	119821353	60	1	2	4	0.00493743	0.02074938	7	8	1.16581773	0.79906872
chr11	119821353	119997842	176490	3	2	4	0.00493743	0.03812622	7	7	1.16581773	0.60763643
chr11	119997842	120004507	6666	1	2	4	0.02162467	0.06713722	5	6	0.68214471	0.44141547
chr11	120004507	120100568	96062	2	0	4	0	0.18734596	4	4	1.26272838	0.18734596
chr11	120100568	120139957	39390	3	1	4	0.00478973	0.18734596	5	4	1.02643191	0.18734596
chr11	120139957	120161828	21872	1	1	4	0.01091641	0.30102999	4	3	0.76005302	0.10122019
chr11	120161828	120198698	36871	1	0	4	0	0.30102999	4	3	1.26272838	0.10122019
chr11	120198698	120215064	16367	2	0	4	0	0.18734596	4	4	1.26272838	0.18734596
chr11	120215064	120215123	60	1	1	4	0.01091641	0.18734596	4	4	0.76005302	0.18734596
chr11	120215123	120276871	61749	1	0	4	0	0.18734596	4	4	1.26272838	0.18734596
chr11	120276871	120328862	51992	1	0	4	0	0.30102999	4	3	1.26272838	0.10122019
chr11	120328862	120377974	49113	2	0	4	0	0.47744371	4	2	1.26272838	0.0429175
chr11	120377974	120482877	104904	3	0	4	0	0.18734596	4	4	1.26272838	0.18734596
chr11	120482877	120580844	97968	2	0	4	0	0.30102999	4	3	1.26272838	0.10122019
chr11	120580844	120898032	317189	7	0	4	0	0.18734596	4	4	1.26272838	0.18734596
chr11	120898032	120970864	72833	2	0	4	0	0.30102999	4	3	1.26272838	0.10122019
chr11	120970864	120970923	60	1	1	4	0.00478973	0.18734596	5	4	1.02643191	0.18734596
chr11	120970923	121059837	88915	2	1	4	0.01091641	0.30102999	4	3	0.76005302	0.10122019
chr11	121059837	121059896	60	1	1	4	0.00478973	0.30102999	5	3	1.02643191	0.10122019
chr11	121059896	121164451	104556	1	0	3	0	0.30102999	5	2	1.60515106	0.08289318
chr11	121164451	121172877	8427	2	0	4	0	0.47744371	5	2	1.60515106	0.0429175
chr11	121172877	121172936	60	1	0	4	0	0.30102999	5	3	1.60515106	0.10122019
chr11	121172936	121201620	28685	2	0	3	0	0.17593012	5	3	1.60515106	0.17593012
chr11	121201620	121308861	107242	2	0	4	0	0.30102999	5	3	1.60515106	0.10122019
chr11	121308861	121360841	51981	1	0	3	0	0.17593012	5	3	1.60515106	0.17593012
chr11	121360841	121420666	59826	2	0	3	0	0.17593012	4	3	1.26272838	0.17593012
chr11	121420666	121445069	24404	2	0	4	0	0.30102999	4	3	1.26272838	0.10122019
chr11	121445069	121578616	133548	2	0	3	0	0.17593012	3	3	0.93173516	0.17593012
chr11	121578616	121578675	60	1	1	3	0.02438896	0.17593012	3	3	0.51676182	0.17593012
chr11	121578675	121829034	250360	1	0	3	0	0.17593012	3	3	0.93173516	0.17593012
chr11	121829034	121925788	96755	2	1	3	0.01091641	0.17593012	4	3	0.76005302	0.17593012
chr11	121925788	121925847	60	1	1	3	0.01091641	0.10122019	4	4	0.76005302	0.30102999
chr11	121925847	121997214	71368	2	1	3	0.02438896	0.17593012	3	3	0.51676182	0.17593012
chr11	121997214	122053210	55997	3	1	5	0.02438896	0.45545077	3	3	0.51676182	0.05670724
chr11	122053210	122293668	240459	2	1	3	0.02438896	0.30102999	3	2	0.51676182	0.08289318
chr11	122293668	122293727	60	1	1	4	0.01091641	0.47744371	4	2	0.76005302	0.0429175
chr11	122293727	122467330	173604	1	1	3	0.05404976	0.30102999	2	2	0.30102999	0.08289318
chr11	122467330	122467389	60	1	1	3	0.02438896	0.30102999	3	2	0.51676182	0.08289318
chr11	122467389	122621163	153775	3	0	2	0	0.30102999	3	1	0.93173516	0.05404976
chr11	122621163	122621222	60	1	1	4	0.02438896	0.76005302	3	1	0.51676182	0.01091641
chr11	122621222	122680463	59242	1	1	2	0.02438896	0.30102999	3	1	0.51676182	0.05404976
chr11	122680463	122726414	45952	1	1	1	0.02438896	0.1218695	3	1	0.51676182	0.1218695
chr11	122726414	122726473	60	1	1	1	0.01091641	0.05404976	4	2	0.76005302	0.30102999
chr11	122726473	122756645	30173	1	1	1	0.02438896	0.1218695	3	1	0.51676182	0.1218695
chr11	122756645	122862489	105845	5	1	3	0.02438896	0.51676182	3	1	0.51676182	0.02438896
chr11	122862489	122862548	60	1	1	4	0.02438896	0.76005302	3	1	0.51676182	0.01091641
chr11	122862548	122968498	105951	3	1	3	0.02438896	0.51676182	3	1	0.51676182	0.02438896
chr11	122968498	122995448	26951	2	1	3	0.02438896	0.30102999	3	2	0.51676182	0.08289318
chr11	122995448	123045174	49727	2	1	4	0.02438896	0.30102999	3	3	0.51676182	0.10122019
chr11	123045174	123045233	60	1	1	5	0.02438896	0.45545077	3	3	0.51676182	0.05670724
chr11	123045233	123111261	66029	2	0	4	0	0.47744371	3	2	0.93173516	0.0429175
chr11	123111261	123111320	60	1	1	5	0.02438896	0.45545077	3	3	0.51676182	0.05670724
chr11	123111320	123252491	141172	1	0	5	0	0.45545077	3	3	0.93173516	0.05670724
chr11	123252491	123322959	70469	2	0	5	0	0.19510895	3	5	0.93173516	0.19510895
chr11	123322959	123414357	91399	2	0	5	0	0.19510895	4	5	1.26272838	0.19510895
chr11	123414357	123443097	28741	2	0	5	0	0.12309572	4	6	1.26272838	0.30102999
chr11	123443097	123513991	70895	2	0	5	0	0.19510895	4	5	1.26272838	0.19510895
chr11	123513991	123514050	60	1	1	5	0.01091641	0.19510895	4	5	0.76005302	0.19510895
chr11	123514050	123596435	82386	2	0	5	0	0.19510895	4	5	1.26272838	0.19510895
chr11	123596435	123624201	27767	1	0	5	0	0.19510895	3	5	0.93173516	0.19510895
chr11	123624201	123755593	131393	3	1	5	0.01091641	0.19510895	4	5	0.76005302	0.19510895
chr11	123755593	123755652	60	1	1	6	0.01091641	0.20064824	4	6	0.76005302	0.20064824
chr11	123755652	123789540	33889	1	0	5	0	0.12309572	3	6	0.93173516	0.30102999
chr11	123789540	123789599	60	1	0	5	0	0.07511598	3	7	0.93173516	0.43181735
chr11	123789599	123860285	70687	1	0	5	0	0.12309572	3	6	0.93173516	0.30102999
chr11	123860285	123860344	60	1	1	5	0.01091641	0.12309572	4	6	0.76005302	0.30102999
chr11	123860344	123978575	118232	2	1	4	0.01091641	0.18734596	4	4	0.76005302	0.18734596
chr11	123978575	124013207	34633	3	1	5	0.00478973	0.12309572	5	6	1.02643191	0.30102999
chr11	124013207	124017576	4370	2	1	5	0.00478973	0.07511598	5	7	1.02643191	0.43181735
chr11	124017576	124136185	118610	2	1	4	0.01091641	0.03812622	4	7	0.76005302	0.60763643
chr11	124136185	124176486	40302	1	1	4	0.01091641	0.06713722	4	6	0.76005302	0.44141547
chr11	124176486	124342304	165819	4	1	3	0.01091641	0.03070643	4	6	0.76005302	0.63695542
chr11	124342304	124412036	69733	2	1	3	0.00478973	0.03070643	5	6	1.02643191	0.63695542
chr11	124412036	124530606	118571	4	1	3	0.00478973	0.05670724	5	5	1.02643191	0.45545077
chr11	124530606	124614728	84123	4	1	3	0.00204627	0.03070643	6	6	1.31360226	0.63695542
chr11	124614728	124616502	1775	2	3	3	0.03070643	0.03070643	6	6	0.63695542	0.63695542
chr11	124616502	124706171	89670	5	1	3	0.00204627	0.03070643	6	6	1.31360226	0.63695542
chr11	124706171	124754005	47835	2	1	4	0.00204627	0.06713722	6	6	1.31360226	0.44141547
chr11	124754005	124790755	36751	2	1	3	0.00204627	0.03070643	6	6	1.31360226	0.63695542
chr11	124790755	124797970	7216	2	3	3	0.03070643	0.03070643	6	6	0.63695542	0.63695542
chr11	124797970	124856796	58827	3	3	4	0.03070643	0.06713722	6	6	0.63695542	0.44141547
chr11	124856796	124937670	80875	3	3	3	0.03070643	0.03070643	6	6	0.63695542	0.63695542
chr11	124937670	124937729	60	1	3	4	0.03070643	0.06713722	6	6	0.63695542	0.44141547
chr11	124937729	124956131	18403	2	3	4	0.03070643	0.11390336	6	5	0.63695542	0.30102999
chr11	124956131	124956187	57	1	3	4	0.03070643	0.06713722	6	6	0.63695542	0.44141547
chr11	124956187	125002089	45903	2	3	3	0.03070643	0.05670724	6	5	0.63695542	0.45545077
chr11	125002089	125002148	60	1	3	3	0.03070643	0.03070643	6	6	0.63695542	0.63695542
chr11	125002148	125153581	151434	2	3	3	0.03070643	0.05670724	6	5	0.63695542	0.45545077
chr11	125153581	125176448	22868	2	3	5	0.03070643	0.19510895	6	5	0.63695542	0.19510895
chr11	125176448	125232643	56196	1	3	5	0.03070643	0.30102999	6	4	0.63695542	0.11390336
chr11	125232643	125310410	77768	1	3	4	0.03070643	0.18734596	6	4	0.63695542	0.18734596
chr11	125310410	125490966	180557	5	3	4	0.03070643	0.11390336	6	5	0.63695542	0.30102999
chr11	125490966	125503031	12066	1	3	4	0.05670724	0.11390336	5	5	0.45545077	0.30102999
chr11	125503031	125503090	60	1	3	5	0.05670724	0.12309572	5	6	0.45545077	0.30102999
chr11	125503090	125514435	11346	1	3	4	0.05670724	0.06713722	5	6	0.45545077	0.44141547
chr11	125514435	125522361	7927	2	3	4	0.03070643	0.06713722	6	6	0.63695542	0.44141547
chr11	125522361	125546371	24011	1	3	4	0.05670724	0.11390336	5	5	0.45545077	0.30102999
chr11	125546371	125616613	70243	1	3	3	0.05670724	0.05670724	5	5	0.45545077	0.45545077
chr11	125616613	125699001	82389	1	2	2	0.02162467	0.02162467	5	5	0.68214471	0.68214471
chr11	125699001	125765326	66326	2	2	2	0.01053319	0.02162467	6	5	0.91219088	0.68214471
chr11	125765326	125771834	6509	2	3	3	0.03070643	0.05670724	6	5	0.63695542	0.45545077
chr11	125771834	125774792	2959	2	3	4	0.03070643	0.11390336	6	5	0.63695542	0.30102999

chr11	125774792	125792773	17982	1	1	3	0.00204627	0.05670724	6	5	1.31360226	0.45545077
chr11	125792773	125833462	40690	1	1	3	0.00204627	0.10122019	6	4	1.31360226	0.30102999
chr11	125833462	125864146	30685	2	1	5	0.00204627	0.30102999	6	4	1.31360226	0.11390336
chr11	125864146	125878336	14191	1	1	5	0.00478973	0.30102999	5	4	1.02643191	0.11390336
chr11	125878336	126148026	269691	7	1	6	0.00478973	0.30102999	5	5	1.02643191	0.12309572
chr11	126148026	126156495	8470	1	1	6	0.00478973	0.44141547	5	4	1.02643191	0.06713722
chr11	126156495	126219397	62903	1	1	4	0.01091641	0.18734596	4	4	0.76005302	0.18734596
chr11	126219397	126257061	37665	2	1	4	0.01091641	0.11390336	4	5	0.76005302	0.30102999
chr11	126257061	126299163	42103	3	1	4	0.00478973	0.11390336	5	5	1.02643191	0.30102999
chr11	126299163	126299222	60	1	1	6	0.00204627	0.30102999	6	5	1.31360226	0.12309572
chr11	126299222	126433764	134543	2	1	4	0.00478973	0.11390336	5	5	1.02643191	0.30102999
chr11	126433764	126503765	70002	1	1	4	0.01091641	0.11390336	4	5	0.76005302	0.30102999
chr11	126503765	126503824	60	1	1	5	0.01091641	0.19510895	4	5	0.76005302	0.19510895
chr11	126503824	126622218	118395	3	0	4	0	0.11390336	4	5	1.26272838	0.30102999
chr11	126622218	126654804	32587	2	1	4	0.01091641	0.11390336	4	5	0.76005302	0.30102999
chr11	126654804	126767927	113124	2	1	3	0.01091641	0.10122019	4	4	0.76005302	0.30102999
chr11	126767927	126863276	95350	2	0	3	0	0.17593012	4	3	1.26272838	0.17593012
chr11	126863276	126863335	60	1	0	4	0	0.18734596	4	4	1.26272838	0.18734596
chr11	126863335	126875921	12587	2	0	4	0	0.30102999	4	3	1.26272838	0.10122019
chr11	126875921	127054775	178855	1	0	4	0	0.30102999	3	3	0.93173516	0.10122019
chr11	127054775	127054834	60	1	0	4	0	0.18734596	3	4	0.93173516	0.18734596
chr11	127054834	127154844	100011	1	0	4	0	0.76005302	3	1	0.93173516	0.01091641
chr11	127154844	127255598	100755	1	0	3	0	0.93173516	3	0	0.93173516	0
chr11	127255598	127255657	60	1	0	3	0	0.51676182	3	1	0.93173516	0.02438896
chr11	127255657	127546842	291186	2	0	3	0	0.93173516	2	0	0.61140001	0
chr11	127546842	127546901	60	1	1	5	0.05404976	0.45545077	2	3	0.30102999	0.05670724
chr11	127546901	127771597	224697	1	1	5	0.05404976	1.02643191	2	1	0.30102999	0.00478973
chr11	127771597	127771656	60	1	1	5	0.05404976	0.45545077	2	3	0.30102999	0.05670724
chr11	127771656	127889198	117543	1	0	4	0	0.47744371	2	2	0.61140001	0.0429175
chr11	127889198	127889257	60	1	1	5	0.02438896	0.45545077	3	3	0.51676182	0.05670724
chr11	127889257	128076421	187165	1	1	4	0.02438896	0.47744371	3	2	0.51676182	0.0429175
chr11	128076421	128076480	60	1	2	4	0.0429175	0.30102999	4	3	0.47744371	0.10122019
chr11	128076480	128170880	94401	1	2	4	0.0429175	0.47744371	4	2	0.47744371	0.0429175
chr11	128170880	128170939	60	1	2	5	0.02162467	0.68214471	5	2	0.68214471	0.02162467
chr11	128170939	128331373	160435	1	1	4	0.01091641	0.76005302	4	1	0.76005302	0.01091641
chr11	128331373	128373445	42073	5	1	5	0.01091641	1.02643191	4	1	0.76005302	0.00478973
chr11	128373445	128447967	74523	3	2	5	0.0429175	1.02643191	4	1	0.47744371	0.00478973
chr11	128447967	128578771	130805	1	1	5	0.01091641	1.02643191	4	1	0.76005302	0.00478973
chr11	128578771	128708441	129671	8	1	6	0.01091641	1.31360226	4	1	0.76005302	0.00204627
chr11	128708441	128772225	63785	2	1	4	0.01091641	0.76005302	4	1	0.76005302	0.01091641
chr11	128772225	128772274	50	1	1	5	0.01091641	0.68214471	4	2	0.76005302	0.02162467
chr11	128772274	128894057	121784	5	1	4	0.01091641	0.47744371	4	2	0.76005302	0.0429175
chr11	128894057	128993322	99266	4	1	4	0.01091641	0.30102999	4	3	0.76005302	0.10122019
chr11	128993322	129034189	40868	2	1	5	0.00478973	0.30102999	5	4	1.02643191	0.11390336
chr11	129034189	129061060	26872	2	2	6	0.01053319	0.44141547	6	4	0.91219088	0.06713722
chr11	129061060	129090507	29448	1	2	5	0.01053319	0.30102999	6	4	0.91219088	0.11390336
chr11	129090507	129161900	71394	1	2	5	0.01053319	0.45545077	6	3	0.91219088	0.05670724
chr11	129161900	129255977	94078	1	1	5	0.00478973	0.45545077	5	3	1.02643191	0.05670724
chr11	129255977	129302317	46341	3	1	5	0.00478973	0.30102999	5	4	1.02643191	0.11390336
chr11	129302317	129321760	19444	2	1	7	0.00478973	0.43181735	5	5	1.02643191	0.07511598
chr11	129321760	129361018	39259	1	1	6	0.00478973	0.44141547	5	4	1.02643191	0.06713722
chr11	129361018	129570431	209414	1	1	5	0.00478973	0.45545077	5	3	1.02643191	0.05670724
chr11	129570431	129570490	60	1	1	5	0.00204627	0.30102999	6	4	1.31360226	0.11390336
chr11	129570490	129722493	152004	1	1	5	0.00204627	1.02643191	6	1	1.31360226	0.00478973
chr11	129722493	129722546	54	1	2	5	0.01053319	1.02643191	6	1	0.91219088	0.00478973
chr11	129722546	129764846	42301	3	1	5	0.00204627	1.02643191	6	1	1.31360226	0.00478973
chr11	129764846	129764904	59	1	2	5	0.01053319	0.68214471	6	2	0.91219088	0.02162467
chr11	129764904	129899266	134363	4	2	5	0.01053319	1.02643191	6	1	0.91219088	0.00478973
chr11	129899266	129966779	67514	1	2	5	0.02162467	1.02643191	5	1	0.68214471	0.00478973
chr11	129966779	129992355	25577	2	2	5	0.02162467	0.68214471	5	2	0.68214471	0.02162467
chr11	129992355	130014663	22309	1	2	4	0.02162467	0.47744371	5	2	0.68214471	0.0429175
chr11	130014663	130014722	60	1	2	4	0.02162467	0.30102999	5	3	0.68214471	0.10122019
chr11	130014722	130178562	163841	5	2	4	0.0429175	0.30102999	4	3	0.47744371	0.10122019
chr11	130178562	130285086	106525	3	2	5	0.0429175	0.45545077	4	3	0.47744371	0.05670724
chr11	130285086	130289134	4049	2	2	5	0.0429175	0.30102999	4	4	0.47744371	0.11390336
chr11	130289134	130379600	90467	2	2	4	0.0429175	0.18734596	4	4	0.47744371	0.18734596
chr11	130379600	130413592	33993	2	2	6	0.0429175	0.30102999	4	5	0.47744371	0.12309572
chr11	130413592	130547126	133535	2	2	6	0.0429175	0.44141547	4	4	0.47744371	0.06713722
chr11	130547126	130547185	60	1	2	7	0.0429175	0.43181735	4	5	0.47744371	0.07511598
chr11	130547185	130584263	37079	1	1	6	0.01091641	0.44141547	4	4	0.76005302	0.06713722
chr11	130584263	130635725	51463	1	1	5	0.01091641	0.30102999	4	4	0.76005302	0.11390336
chr11	130635725	130635784	60	1	2	6	0.0429175	0.30102999	4	5	0.47744371	0.12309572
chr11	130635784	130703906	68123	1	1	6	0.01091641	0.30102999	4	5	0.76005302	0.12309572
chr11	130703906	130719758	15853	2	1	7	0.01091641	0.30102999	4	6	0.76005302	0.129913
chr11	130719758	130784198	64441	3	1	7	0.01091641	0.43181735	4	5	0.76005302	0.07511598
chr11	130784198	131013203	229006	3	1	6	0.01091641	0.63695542	4	3	0.76005302	0.03070643
chr11	131013203	131110755	97553	2	1	7	0.01091641	0.84395715	4	3	0.76005302	0.01598258
chr11	131110755	131110814	60	1	1	8	0.01091641	0.79906872	4	4	0.76005302	0.02074938
chr11	131110814	131267471	156658	2	0	7	0	0.84395715	4	3	1.26272838	0.01598258
chr11	131267471	131267530	60	1	0	8	0	0.79906872	4	4	1.26272838	0.02074938
chr11	131267530	131390419	122890	2	0	8	0	1.07548421	4	3	1.26272838	0.0079614
chr11	131390419	131605551	215133	5	0	8	0	0.79906872	4	4	1.26272838	0.02074938
chr11	131605551	131702440	96890	2	0	8	0	0.79906872	3	4	0.93173516	0.02074938
chr11	131702440	131780086	77647	1	0	7	0	0.60763643	3	4	0.93173516	0.03812622
chr11	131780086	132137301	357216	8	1	7	0.02438896	0.60763643	3	4	0.51676182	0.03812622
chr11	132137301	132225369	88069	3	1	6	0.02438896	0.44141547	3	4	0.51676182	0.06713722
chr11	132225369	132225428	60	1	1	6	0.02438896	0.30102999	3	5	0.51676182	0.12309572
chr11	132225428	132333033	107606	2	1	6	0.02438896	0.44141547	3	4	0.51676182	0.06713722
chr11	132333033	132333092	60	1	1	8	0.02438896	0.58747015	3	5	0.51676182	0.04407651
chr11	132333092	132399092	66001	1	1	7	0.02438896	0.43181735	3	5	0.51676182	0.07511598
chr11	132399092	132515235	116144	2	1	7	0.05404976	0.43181735	2	5	0.30102999	0.07511598
chr11	132515235	132641420	126186	3	1	7	0.05404976	0.30102999	2	6	0.30102999	0.129913
chr11	132641420	132817221	175802	3	1	7	0.05404976	0.43181735	2	5	0.30102999	0.07511598
chr11	132817221	132868438	51218	1	1	7	0.05404976	0.60763643	2	4	0.30102999	0.03812622
chr11	132868438	132868497	60	1	1	8	0.05404976	0.79906872	2	4	0.30102999	0.02074938
chr11	132868497	132956834	88338	2	1	7	0.05404976	0.60763643	2	4	0.30102999	0.03812622
chr11	132956834	133009773	52940	2	1	8	0.05404976	0.79906872	2	4	0.30102999	0.02074938
chr11	133009773	133146423	136651	2	1	7	0.05404976	0.60763643	2	4	0.30102999	0.03812622
chr11	133146423	133146481	59	1	1	8	0.05404976	0.4250187	2	6	0.30102999	0.08122616
chr11	133146481	133173311	26831	1	0	8	0	0.58747015	2	5	0.61140001	0.04407651
chr11	133173311	133268419	95109	2	0	7	0	0.43181735	2	5	0.61140001	0.07511598
chr11	133268419	133268478	60	1	0	7	0	0.30102999	2	6	0.61140001	0.129913
chr11	133268478	133420927	152450	3	0	5	0	0.30102999	2	4	0.61140001	0.11390336
chr11	133420927	133531350	110424	1	0	4	0	0.30102999	2	3	0.61140001	0.10122019
chr11	133531350	133600741	69392	1	0	3	0	0.17593012	2	3	0.61140001	0.17593012
chr11	133600741	133700219	99479	2	0	4	0	0.18734596	2	4	0.61140001	0.18734596
chr11	133700219	133715345	15127	2	1	5	0.05404976	0.30102999	2	4	0.30102999	0.11390336
chr11	133715345	133767633	52289	1	0	5	0	0.30102999	2	4	0.61140001	0.11390336
chr11	133767633	133767692	60	1	0	5	0	0.19510895	2	5	0.61140001	0.19510895
chr11	133767692	133842894	75203	1	0	4	0	0.11390336	2	5	0.61140001	0.30102999
chr11	133842894	133842953	60	1	1	4	0.05404976	0.11390336	2	5	0.30102999	0.30102999
chr11	133842953	133926977	84025	1	1	3	0.05404976	0.17593012	2	3	0.30102999	0.17593012
chr11	133926977	133990006	63030	4	1	3	0.05404976	0.10122019	2	4	0.30102999	0.30102999
chr11	133990006	134045024	55019	1	1	2	0.05404976	0.0429175	2	4	0.30102999	0.47744371
chr11	134045024	134045083	60	1	1	2	0.05404976	0.02162467	2	5	0.30102999	0.68214471
chr11	134045083	134381691	336609	11	1	2	0.05404976	0.08289318	2	3	0.30102999	0.30102999
chr11	134381691	134746628	364938	5	1	2	0.05404976	0.1575501	2	2	0.30102999	0.1575501
chr11	134746628	134868407	121780	1	1	1	0.05404976	0.05404976	2	2	0.30102999	0.30102999
chr12	230421	289707	59287	2	5	0	0.07511598	0	7	1	0.43181735	0.30102999
chr12	289707	511504	221798	4	4	0	0.06713722	0	6	1	0.44141547	0.30102999
chr12	511504	608362	96859	3	5	0	0.04407651	0	8	1	0.58747015	0.30102999
chr12	608362	702446	94085	3	7	1	0.13499366	0.1218695	8	1	0.30102999	0.1218695
chr12	702446	702505	60	1	8	1	0.20764654	0.1218695	8	1	0.20764654	0.1218695
chr12	702505	786623	84119	2	7	1	0.13499366	0.1218695	8	1	0.30102999	0.1218695

chr12	786623	835365	48743	1	6	1	0.08122616	0.1218695	8	1	0.4250187	0.1218695
chr12	835365	835424	60	1	7	2	0.13499366	0.30102999	8	1	0.30102999	0.05404976
chr12	835424	921383	85960	2	6	2	0.08122616	0.30102999	8	1	0.4250187	0.05404976
chr12	921383	921442	60	1	9	2	0.20975986	0.30102999	9	1	0.20975986	0.05404976
chr12	921442	1064055	142614	3	9	2	0.30102999	0.30102999	8	1	0.13872638	0.05404976
chr12	1064055	1219472	155418	3	8	2	0.20764654	0.30102999	8	1	0.20764654	0.05404976
chr12	1219472	1258131	38660	2	9	2	0.30102999	0.30102999	8	1	0.13872638	0.05404976
chr12	1258131	1313319	55189	1	9	2	0.42015402	0.30102999	7	1	0.08584816	0.05404976
chr12	1313319	1367681	54363	1	8	2	0.30102999	0.30102999	7	1	0.13499366	0.05404976
chr12	1367681	1367740	60	1	8	2	0.20764654	0.30102999	8	1	0.20764654	0.05404976
chr12	1367740	1399200	31461	1	8	2	0.30102999	0.30102999	7	1	0.13499366	0.05404976
chr12	1399200	1476151	76952	1	7	2	0.20469099	0.30102999	7	1	0.20469099	0.05404976
chr12	1476151	1476210	60	1	9	2	0.30102999	0.30102999	8	1	0.13872638	0.05404976
chr12	1476210	1518073	41864	1	7	2	0.20469099	0.30102999	7	1	0.20469099	0.05404976
chr12	1518073	1518132	60	1	8	2	0.30102999	0.30102999	7	1	0.13499366	0.05404976
chr12	1518132	1644476	126345	3	7	2	0.20469099	0.30102999	7	1	0.20469099	0.05404976
chr12	1644476	1696902	52427	2	7	2	0.20469099	0.1575501	7	2	0.20469099	0.1575501
chr12	1696902	1749581	52680	3	8	2	0.30102999	0.1575501	7	2	0.13499366	0.1575501
chr12	1749581	1863686	114106	5	8	2	0.20764654	0.1575501	8	2	0.20764654	0.1575501
chr12	1863686	1904537	40852	2	7	2	0.13499366	0.1575501	8	2	0.30102999	0.1575501
chr12	1904537	1904585	49	1	8	2	0.20764654	0.1575501	8	2	0.20764654	0.1575501
chr12	1904585	1968642	64058	2	7	2	0.13499366	0.1575501	8	2	0.30102999	0.1575501
chr12	1968642	1995419	26778	2	8	2	0.20764654	0.1575501	8	2	0.20764654	0.1575501
chr12	1995419	2086180	90762	2	7	2	0.13499366	0.1575501	8	2	0.30102999	0.1575501
chr12	2086180	2112401	26222	2	7	3	0.13499366	0.30102999	8	2	0.30102999	0.08289318
chr12	2112401	2230845	118445	2	7	2	0.20469099	0.1575501	7	2	0.20469099	0.1575501
chr12	2230845	2345759	114915	3	7	3	0.20469099	0.30102999	7	2	0.20469099	0.08289318
chr12	2345759	2345818	60	1	7	3	0.13499366	0.30102999	8	2	0.30102999	0.08289318
chr12	2345818	2438871	93054	2	7	3	0.20469099	0.30102999	7	2	0.20469099	0.08289318
chr12	2438871	2623147	184277	4	7	3	0.13499366	0.30102999	8	2	0.30102999	0.08289318
chr12	2623147	2623206	60	1	7	3	0.13499366	0.17593012	8	3	0.30102999	0.17593012
chr12	2623206	3178479	555274	18	7	3	0.20469099	0.17593012	7	3	0.20469099	0.17593012
chr12	3178479	3394129	215651	5	7	3	0.30102999	0.17593012	6	3	0.129913	0.17593012
chr12	3394129	3731272	337144	7	5	3	0.12309572	0.17593012	6	3	0.30102999	0.17593012
chr12	3731272	3850575	119304	2	5	2	0.12309572	0.1575501	6	2	0.30102999	0.1575501
chr12	3850575	3907929	57355	2	5	3	0.12309572	0.30102999	6	2	0.30102999	0.08289318
chr12	3907929	4543321	635393	12	4	3	0.06713722	0.30102999	6	2	0.44141547	0.08289318
chr12	4543321	4595093	51773	2	4	4	0.06713722	0.47744371	6	2	0.44141547	0.0429175
chr12	4595093	4790138	195046	6	4	3	0.06713722	0.30102999	6	2	0.44141547	0.08289318
chr12	4790138	5155203	365066	8	4	2	0.06713722	0.1575501	6	2	0.44141547	0.1575501
chr12	5155203	5221304	66102	3	4	3	0.06713722	0.30102999	6	2	0.44141547	0.08289318
chr12	5221304	5347885	126582	2	5	3	0.12309572	0.30102999	6	2	0.30102999	0.08289318
chr12	5347885	5447696	99812	2	5	3	0.12309572	0.17593012	6	3	0.30102999	0.17593012
chr12	5447696	5447755	60	1	6	4	0.20064824	0.18734596	6	4	0.20064824	0.18734596
chr12	5447755	5513678	65924	1	6	4	0.20064824	0.30102999	6	3	0.20064824	0.10122019
chr12	5513678	5513737	60	1	8	4	0.4250187	0.30102999	6	3	0.08122616	0.10122019
chr12	5513737	5545532	31796	1	8	3	0.4250187	0.17593012	6	3	0.08122616	0.17593012
chr12	5545532	5603350	57819	1	6	3	0.20064824	0.17593012	6	3	0.20064824	0.17593012
chr12	5603350	5672258	68909	2	7	4	0.30102999	0.18734596	6	4	0.129913	0.18734596
chr12	5672258	5672314	57	1	8	4	0.4250187	0.18734596	6	4	0.08122616	0.18734596
chr12	5672314	5738225	65912	1	8	3	0.4250187	0.10122019	6	4	0.08122616	0.30102999
chr12	5738225	5738284	60	1	9	3	0.5732208	0.10122019	6	4	0.04875589	0.30102999
chr12	5738284	5769389	31106	1	9	3	0.5732208	0.17593012	6	3	0.04875589	0.17593012
chr12	5769389	5816806	47418	2	10	3	0.74627054	0.17593012	6	3	0.02793176	0.17593012
chr12	5816806	5816865	60	1	10	4	0.74627054	0.18734596	6	4	0.02793176	0.18734596
chr12	5816865	5916479	99615	2	10	4	0.74627054	0.30102999	6	3	0.02793176	0.10122019
chr12	5916479	5916532	54	1	10	4	0.74627054	0.18734596	6	4	0.02793176	0.18734596
chr12	5916532	5979800	63269	1	9	4	0.5732208	0.18734596	6	4	0.04875589	0.18734596
chr12	5979800	5979859	60	1	9	4	0.5732208	0.11390336	6	5	0.04875589	0.30102999
chr12	5979859	6055308	75450	1	9	3	0.5732208	0.05670724	6	5	0.04875589	0.45545077
chr12	6055308	6147946	92639	3	10	3	0.74627054	0.05670724	6	5	0.02793176	0.45545077
chr12	6147946	6326781	178836	5	9	3	0.5732208	0.10122019	6	4	0.04875589	0.30102999
chr12	6326781	6422116	95336	3	10	3	0.74627054	0.10122019	6	4	0.02793176	0.30102999
chr12	6422116	6496043	73928	4	10	3	0.74627054	0.05670724	6	5	0.02793176	0.45545077
chr12	6496043	6525634	29592	2	11	3	0.9449689	0.05670724	6	5	0.01518174	0.45545077
chr12	6525634	6671514	145881	8	10	3	0.74627054	0.10122019	6	4	0.02793176	0.30102999
chr12	6671514	6732736	61223	3	9	3	0.5732208	0.10122019	6	4	0.04875589	0.30102999
chr12	6732736	6732795	60	1	10	3	0.74627054	0.10122019	6	4	0.02793176	0.30102999
chr12	6732795	6796169	63375	4	9	3	0.5732208	0.10122019	6	4	0.04875589	0.30102999
chr12	6796169	6857172	61004	2	9	2	0.5732208	0.0429175	6	4	0.04875589	0.47744371
chr12	6857172	6857231	60	1	9	3	0.42015402	0.10122019	7	4	0.08584816	0.30102999
chr12	6857231	6929708	72478	7	9	3	0.5732208	0.10122019	6	4	0.04875589	0.30102999
chr12	6929708	7114197	184490	22	9	4	0.42015402	0.18734596	7	4	0.08584816	0.18734596
chr12	7114197	7187618	73422	3	9	4	0.42015402	0.11390336	7	5	0.08584816	0.30102999
chr12	7187618	7278289	90672	4	9	4	0.42015402	0.30102999	7	3	0.08584816	0.10122019
chr12	7278289	7301110	22822	1	8	4	0.30102999	0.30102999	7	3	0.13499366	0.10122019
chr12	7301110	7347854	46745	2	8	4	0.30102999	0.18734596	7	4	0.13499366	0.18734596
chr12	7347854	7495496	147643	2	8	4	0.30102999	0.30102999	7	3	0.13499366	0.10122019
chr12	7495496	7495555	60	1	8	4	0.30102999	0.18734596	7	4	0.13499366	0.18734596
chr12	7495555	7550952	55398	2	8	2	0.30102999	0.08289318	7	3	0.13499366	0.30102999
chr12	7550952	7551009	58	1	8	4	0.30102999	0.18734596	7	4	0.13499366	0.18734596
chr12	7551009	7586015	35007	1	8	2	0.30102999	0.08289318	7	3	0.13499366	0.30102999
chr12	7586015	7653675	67661	2	8	1	0.30102999	0.02438896	7	3	0.13499366	0.51676182
chr12	7653675	7712397	58723	2	8	2	0.30102999	0.08289318	7	3	0.13499366	0.30102999
chr12	7712397	7805008	92612	1	7	2	0.20469099	0.08289318	7	3	0.20469099	0.30102999
chr12	7805008	7817151	12144	2	7	2	0.13499366	0.08289318	8	3	0.30102999	0.30102999
chr12	7817151	7817210	60	1	7	3	0.13499366	0.17593012	8	3	0.30102999	0.17593012
chr12	7817210	7886957	69748	4	7	2	0.13499366	0.08289318	8	3	0.30102999	0.30102999
chr12	7886957	7900049	13093	2	7	3	0.13499366	0.10122019	8	4	0.30102999	0.30102999
chr12	7900049	7945970	45922	1	6	3	0.08122616	0.17593012	8	3	0.4250187	0.17593012
chr12	7945970	7985489	39520	1	6	1	0.08122616	0.02438896	8	3	0.4250187	0.51676182
chr12	7985489	8003473	17985	2	6	1	0.08122616	0.01091641	8	4	0.4250187	0.76005302
chr12	8003473	8053401	49929	1	6	1	0.08122616	0.02438896	8	3	0.4250187	0.51676182
chr12	8053401	8078370	24970	2	6	2	0.08122616	0.08289318	8	3	0.4250187	0.30102999
chr12	8078370	8145133	66764	2	4	2	0.02074938	0.08289318	8	3	0.79906872	0.30102999
chr12	8145133	8145192	60	1	5	2	0.04407651	0.08289318	8	3	0.58747015	0.30102999
chr12	8145192	8238013	92822	3	4	1	0.02074938	0.02438896	8	3	0.79906872	0.51676182
chr12	8238013	8238072	60	1	5	2	0.04407651	0.0429175	8	4	0.58747015	0.47744371
chr12	8238072	8281969	43898	1	4	0	0.02074938	0	8	3	0.79906872	0.93173516
chr12	8281969	8608171	326203	1	4	0	0.02074938	0	8	2	0.79906872	0.61140001
chr12	8608171	8608230	60	1	5	0	0.04407651	0	8	2	0.58747015	0.61140001
chr12	8608230	8667855	59626	1	4	0	0.02074938	0	8	2	0.79906872	0.61140001
chr12	8667855	8759458	91604	4	5	0	0.04407651	0	8	2	0.58747015	0.61140001
chr12	8759458	8798736	39279	2	5	0	0.02473314	0	9	2	0.76806864	0.61140001
chr12	8798736	8985450	186715	4	4	0	0.01077081	0	9	2	1.01542894	0.61140001
chr12	8985450	9020831	35382	2	4	0	0.01077081	0	9	3	1.01542894	0.93173516
chr12	9020831	9095929	75099	3	5	0	0.02473314	0	9	3	0.76806864	0.93173516
chr12	9095929	9095988	60	1	6	0	0.04875589	0	9	3	0.5732208	0.93173516
chr12	9095988	9140586	44599	1	5	0	0.02473314	0	9	2	0.76806864	0.61140001
chr12	9140586	9162281	21696	1	4	0	0.01077081	0	9	2	1.01542894	0.61140001
chr12	9162281	9241766	79486	3	4	0	0.01077081	0	9	3	1.01542894	0.93173516
chr12	9241766	9241825	60	1	4	1	0.01077081	0.02438896	9	3	1.01542894	0.51676182
chr12	9241825	9354933	113109	3	4	0	0.01077081	0	9	3	1.01542894	0.93173516
chr12	9354933	9354990	58	1	5	0	0.01320236	0	10	3	0.97390707	0.93173516
chr12	9354990	9424062	69073	3	5	0	0.02473314	0	9	3	0.76806864	0.93173516
chr12	9424062	9424121	60	1	5	1	0.01320236	0.02438896	10	3	0.97390707	0.51676182
chr12	9424121	9497822	73702	1	5	1	0.02473314	0.02438896	9	3	0.76806864	0.51676182
chr12	9497822	9637323	139502	1	5	1	0.02473314	0.05404976	9	2	0.76806864	0.30102999
chr12	9637323	9713366	76044	2	5	1	0.00666883	0.05404976	11	2	1.20557689	0.30102999
chr12	9713366	9747853	34488	2	5	1	0.00666883	0.02438896	11	3	1.20557689	0.51676182
chr12	9747853	9806341	58489	3	5	2	0.00666883	0.08289318	11	3	1.20557689	0.30102999
chr12	9806341	9881523	75183	3	5	0	0.00666883	0	11	3	1.20557689	0.93173516
chr12	9881523	9881582	60	1	5	0	0.00317045	0	12	3	1.46403142	0.93173516
chr12	9881582	9913160	31579	1	5	0	0.00317045	0	12	2	1.46403142	0.61140001

chr12	9913160	9984895	71736	1	5	0	0.00666883	0	11	2	1.20557689	0.61140001
chr12	9984895	10039266	54372	3	5	3	0.00666883	0.17593012	11	4	1.20557689	0.17593012
chr12	10039266	10039325	60	1	5	5	0.00666883	0.30102999	11	4	1.20557689	0.11390336
chr12	10039325	10101899	62575	1	5	4	0.00666883	0.18734596	11	4	1.20557689	0.18734596
chr12	10101899	10137505	35607	3	5	6	0.00666883	0.44141547	11	4	1.20557689	0.06713722
chr12	10137505	10151724	14220	1	4	6	0.00247414	0.44141547	11	4	1.52371709	0.06713722
chr12	10151724	10162062	10339	2	4	7	0.00247414	0.60763643	11	4	1.52371709	0.03812622
chr12	10162062	10164583	2522	1	4	5	0.00247414	0.30102999	11	4	1.52371709	0.11390336
chr12	10164583	10242688	78106	1	4	5	0.00247414	0.45545077	11	3	1.52371709	0.05670724
chr12	10242688	10467296	224609	7	4	6	0.00247414	0.63695542	11	3	1.52371709	0.03070643
chr12	10467296	10467355	60	1	4	6	0.00247414	0.44141547	11	4	1.52371709	0.06713722
chr12	10467355	10511649	44295	1	4	6	0.00247414	0.63695542	11	3	1.52371709	0.03070643
chr12	10511649	10562264	50616	1	4	6	0.00247414	0.91219088	11	2	1.52371709	0.01053319
chr12	10562264	10602776	40513	4	4	6	0.00247414	0.63695542	11	3	1.52371709	0.03070643
chr12	10602776	10662648	59873	2	4	6	0.00247414	0.91219088	11	2	1.52371709	0.01053319
chr12	10662648	10789795	127148	4	4	6	0.00247414	0.44141547	11	4	1.52371709	0.06713722
chr12	10789795	10826880	37086	1	4	5	0.00247414	0.30102999	11	4	1.52371709	0.11390336
chr12	10826880	10872197	45318	2	4	4	0.00247414	0.18734596	11	4	1.52371709	0.18734596
chr12	10872197	10954311	82115	1	4	4	0.00247414	0.30102999	11	3	1.52371709	0.10122019
chr12	10954311	10958642	4332	1	3	4	7.28E-04	0.30102999	11	3	1.91540774	0.10122019
chr12	10958642	10958701	60	1	3	4	7.28E-04	0.18734596	11	4	1.91540774	0.18734596
chr12	10958701	10958960	260	1	3	4	0.00170589	0.18734596	10	4	1.61091002	0.18734596
chr12	10958960	10961831	2872	2	4	4	0.00530919	0.18734596	10	4	1.2568129	0.18734596
chr12	10961831	10978139	16309	2	4	4	0.00247414	0.18734596	11	4	1.52371709	0.18734596
chr12	10978139	11061373	83235	2	4	3	0.00247414	0.17593012	11	3	1.52371709	0.17593012
chr12	11061373	11091279	29907	3	4	3	0.00247414	0.10122019	11	4	1.52371709	0.30102999
chr12	11091279	11091338	60	1	4	5	0.00247414	0.30102999	11	4	1.52371709	0.11390336
chr12	11091338	11149738	58401	2	4	3	0.00247414	0.10122019	11	4	1.52371709	0.30102999
chr12	11149738	11212877	63140	2	4	2	0.00247414	0.08289318	11	3	1.52371709	0.30102999
chr12	11212877	11331053	118177	2	4	1	0.00247414	0.02438896	11	3	1.52371709	0.51676182
chr12	11331053	11331112	60	1	4	2	0.00247414	0.08289318	11	3	1.52371709	0.30102999
chr12	11331112	11421447	90336	1	4	2	0.00530919	0.08289318	10	3	1.2568129	0.30102999
chr12	11421447	11496728	75282	2	4	3	0.00530919	0.10122019	10	4	1.2568129	0.30102999
chr12	11496728	11496787	60	1	4	4	0.00247414	0.18734596	11	4	1.52371709	0.18734596
chr12	11496787	11539679	42893	1	4	4	0.00530919	0.18734596	10	4	1.2568129	0.18734596
chr12	11539679	11787045	247367	3	4	2	0.00530919	0.08289318	10	3	1.2568129	0.30102999
chr12	11787045	11787104	60	1	4	2	0.00530919	0.0429175	10	4	1.2568129	0.47744371
chr12	11787104	11861203	74100	1	4	2	0.00530919	0.08289318	10	3	1.2568129	0.30102999
chr12	11861203	11896528	35326	2	4	3	0.00530919	0.17593012	10	3	1.2568129	0.17593012
chr12	11896528	11896587	60	1	5	4	0.01320236	0.30102999	10	3	0.97390707	0.10122019
chr12	11896587	11956206	59620	1	4	4	0.00530919	0.30102999	10	3	1.2568129	0.10122019
chr12	11956206	12006403	50198	2	4	4	0.00530919	0.11390336	10	5	1.2568129	0.30102999
chr12	12006403	12074651	68249	2	3	3	0.00170589	0.10122019	10	4	1.61091002	0.30102999
chr12	12074651	12094530	19880	1	3	3	0.00170589	0.17593012	10	3	1.61091002	0.17593012
chr12	12094530	12172284	77755	1	3	3	0.00378107	0.17593012	9	3	1.33111237	0.17593012
chr12	12172284	12172343	60	1	3	3	0.00378107	0.10122019	9	4	1.33111237	0.30102999
chr12	12172343	12356229	183887	5	3	3	0.00378107	0.17593012	9	3	1.33111237	0.17593012
chr12	12356229	12397221	40993	2	3	3	0.00378107	0.10122019	9	4	1.33111237	0.30102999
chr12	12397221	12397280	60	1	3	3	0.00170589	0.10122019	10	4	1.61091002	0.30102999
chr12	12397280	12436291	39012	1	3	3	0.00170589	0.17593012	10	3	1.61091002	0.17593012
chr12	12436291	12549605	113315	5	3	3	0.00170589	0.10122019	10	4	1.61091002	0.30102999
chr12	12549605	12653165	103561	4	3	3	0.00170589	0.05670724	10	5	1.61091002	0.45545077
chr12	12653165	12918924	265760	9	3	4	0.00170589	0.11390336	10	5	1.61091002	0.30102999
chr12	12918924	12982882	63959	4	3	5	0.00170589	0.19510895	10	5	1.61091002	0.19510895
chr12	12982882	13013713	30832	1	3	4	0.00170589	0.11390336	10	5	1.61091002	0.30102999
chr12	13013713	13013772	60	1	3	4	0.00170589	0.06713722	10	6	1.61091002	0.44141547
chr12	13013772	13066504	52733	1	3	4	0.00378107	0.06713722	9	6	1.33111237	0.44141547
chr12	13066504	13066563	60	1	3	5	0.00378107	0.12309572	9	6	1.33111237	0.30102999
chr12	13066563	13240987	174425	4	3	5	0.0079614	0.12309572	8	6	1.07548421	0.30102999
chr12	13240987	13418897	177911	4	3	5	0.01598258	0.12309572	7	6	0.84395715	0.30102999
chr12	13418897	13525405	106509	1	3	4	0.01598258	0.18734596	7	4	0.84395715	0.18734596
chr12	13525405	13529105	3701	2	3	4	0.0079614	0.18734596	8	4	1.07548421	0.18734596
chr12	13529105	13656811	127707	2	2	4	0.00221948	0.18734596	8	4	1.44210395	0.18734596
chr12	13656811	13656870	60	1	2	5	0.00221948	0.30102999	8	4	1.44210395	0.11390336
chr12	13656870	13715102	58233	1	1	5	3.39E-04	0.30102999	8	4	1.94674965	0.11390336
chr12	13715102	13828818	113717	4	1	5	1.30E-04	0.30102999	9	4	2.29264207	0.11390336
chr12	13828818	13906859	78042	1	1	5	8.47E-04	0.30102999	7	4	1.62048027	0.11390336
chr12	13906859	13975539	68681	1	1	4	8.47E-04	0.18734596	7	4	1.62048027	0.18734596
chr12	13975539	13975598	60	1	1	4	3.39E-04	0.06713722	8	6	1.94674965	0.44141547
chr12	13975598	14030273	54676	1	1	4	3.39E-04	0.11390336	8	5	1.94674965	0.30102999
chr12	14030273	14132896	102624	3	1	4	1.30E-04	0.11390336	9	5	2.29264207	0.30102999
chr12	14132896	14572563	439668	5	1	4	8.47E-04	0.11390336	7	5	1.62048027	0.30102999
chr12	14572563	15066956	494394	23	1	5	8.47E-04	0.19510895	7	5	1.62048027	0.19510895
chr12	15066956	15114530	47575	4	1	5	3.39E-04	0.19510895	8	5	1.94674965	0.19510895
chr12	15114530	15129429	14900	2	1	5	1.30E-04	0.12309572	9	6	2.29264207	0.30102999
chr12	15129429	15150448	21020	1	1	5	1.30E-04	0.19510895	9	5	2.29264207	0.19510895
chr12	15150448	15278488	128041	2	1	5	1.30E-04	0.30102999	9	4	2.29264207	0.11390336
chr12	15278488	15470171	191684	4	1	4	1.30E-04	0.18734596	9	4	2.29264207	0.18734596
chr12	15470171	15489311	19141	2	1	5	1.30E-04	0.30102999	9	4	2.29264207	0.11390336
chr12	15489311	15550885	61575	1	1	5	3.39E-04	0.30102999	8	4	1.94674965	0.11390336
chr12	15550885	15550944	60	1	1	5	3.39E-04	0.12309572	8	6	1.94674965	0.30102999
chr12	15550944	15598419	47476	1	1	4	3.39E-04	0.06713722	8	6	1.94674965	0.44141547
chr12	15598419	15704539	106121	2	1	4	3.39E-04	0.18734596	8	4	1.94674965	0.18734596
chr12	15704539	15749687	45149	2	1	4	1.30E-04	0.18734596	9	4	2.29264207	0.18734596
chr12	15749687	15749746	60	1	1	4	1.30E-04	0.11390336	9	5	2.29264207	0.30102999
chr12	15749746	15774015	24270	1	1	4	3.39E-04	0.11390336	8	5	1.94674965	0.30102999
chr12	15774015	15834131	60117	1	1	4	3.39E-04	0.18734596	8	4	1.94674965	0.18734596
chr12	15834131	15908518	74388	3	1	4	3.39E-04	0.11390336	8	5	1.94674965	0.30102999
chr12	15908518	15908577	60	1	1	4	3.39E-04	0.06713722	8	6	1.94674965	0.44141547
chr12	15908577	15940945	32369	1	1	4	8.47E-04	0.30102999	7	3	1.62048027	0.10122019
chr12	15940945	15941004	60	1	1	5	8.47E-04	0.45545077	7	3	1.62048027	0.05670724
chr12	15941004	16037239	96236	1	1	3	8.47E-04	0.30102999	7	2	1.62048027	0.08289318
chr12	16037239	16068366	31128	1	1	3	8.47E-04	0.51676182	7	1	1.62048027	0.02438896
chr12	16068366	16111171	42806	1	1	3	0.00204627	0.51676182	6	1	1.31360226	0.02438896
chr12	16111171	16375710	264540	6	1	3	0.00204627	0.30102999	6	2	1.31360226	0.08289318
chr12	16375710	16506363	130654	3	1	3	0.00204627	0.17593012	6	3	1.31360226	0.17593012
chr12	16506363	16703395	197033	5	1	3	8.47E-04	0.17593012	7	3	1.62048027	0.17593012
chr12	16703395	16732343	28949	1	1	0	0.00478973	0	5	1	1.02643191	0.30102999
chr12	16732343	16760848	28506	1	1	0	0.00478973	0	5	0	1.02643191	0
chr12	16760848	16760907	60	1	1	1	0.00478973	0.05404976	5	2	1.02643191	0.30102999
chr12	16760907	16785646	24740	1	1	1	0.00478973	0.1218695	5	1	1.02643191	0.1218695
chr12	16785646	16785705	60	1	1	1	0.00204627	0.05404976	6	2	1.31360226	0.30102999
chr12	16785705	17251595	465891	3	1	0	0.01091641	0	4	0	0.76005302	0
chr12	17251595	17251654	60	1	1	0	0.00478973	0	5	1	1.02643191	0.30102999
chr12	17251654	17495103	243450	1	1	0	0.00478973	0	5	0	1.02643191	0
chr12	17495103	17495162	60	1	2	1	0.00493743	0.30102999	7	0	1.16581773	0
chr12	17495162	17678813	183652	1	2	1	0.02162467	0.30102999	5	0	0.68214471	0
chr12	17678813	17678872	60	1	2	1	0.02162467	0.05404976	5	2	0.68214471	0.30102999
chr12	17678872	17864394	185523	1	2	0	0.02162467	0	5	2	0.68214471	0.61140001
chr12	17864394	18037107	172714	2	2	0	0.01053319	0	6	2	0.91219088	0.61140001
chr12	18037107	18037166	60	1	2	1	0.00493743	0.02438896	7	3	1.16581773	0.51676182
chr12	18037166	18240372	203207	2	2	0	0.00493743	0	7	3	1.16581773	0.93173516
chr12	18240372	18241916	1545	2	2	1	0.00493743	0.02438896	7	3	1.16581773	0.51676182
chr12	18241916	18443901	201986	2	1	0	8.47E-04	0	7	2	1.62048027	0.61140001
chr12	18443901	18443960	60	1	2	1	0.00221948	0.02438896	8	3	1.44210395	0.51676182
chr12	18443960	18536231	92272	2	1	0	8.47E-04	0	7	3	1.62048027	0.93173516
chr12	18536231	18610625	74395	1	1	0	8.47E-04	0	7	2	1.62048027	0.61140001
chr12	18610625	18644479	33855	2	2	1	0.00493743	0.05404976	7	2	1.16581773	0.30102999
chr12	18644479	18892035	247557	6	2	0	0.00493743	0	7	2	1.16581773	0.61140001
chr12	18892035	18892094	60	1	2	0	0.00493743	0	7	3	1.16581773	0.93173516
chr12	18892094	19042039	149946	1	2	0	0.00493743	0	7	2	1.16581773	0.61140001
chr12	19042039	19346251	304213	5	2	1	0.00493743	0.05404976	7	2	1.16581773	0.30102999
chr12	19346251	19381278	35028	2	3	1	0.01598258	0.05404976	7	2	0.84395715	0.30102999
chr12	19381278	19459675	78398	2	3	1	0.03070643	0.05404976	6	2	0.63695542	0.30102999

chr12	19459675	19459728	54	1	3	2	0.03070643	0.1575501	6	2	0.63695542	0.1575501
chr12	19459728	19525493	65766	1	2	1	0.01053319	0.1218695	6	1	0.91219088	0.1218695
chr12	19525493	19602761	77269	2	1	1	0.00478973	0.1218695	5	1	1.02643191	0.1218695
chr12	19602761	19602820	60	1	2	2	0.01053319	0.1575501	6	2	0.91219088	0.1575501
chr12	19602820	19615573	12754	1	1	1	0.00478973	0.1218695	5	1	1.02643191	0.1218695
chr12	19615573	19744674	129102	2	0	1	0	0.1218695	5	1	1.60515106	0.1218695
chr12	19744674	19875175	130502	2	0	2	0	0.1575501	5	2	1.60515106	0.1575501
chr12	19875175	19875234	60	1	0	3	0	0.30102999	5	2	1.60515106	0.08289318
chr12	19875234	20038565	163332	1	0	2	0	0.30102999	5	1	1.60515106	0.05404976
chr12	20038565	20038624	60	1	1	2	0.00478973	0.1575501	5	2	1.02643191	0.1575501
chr12	20038624	20210481	171858	2	1	2	0.00478973	0.30102999	5	1	1.02643191	0.05404976
chr12	20210481	20210540	60	1	1	3	0.00478973	0.51676182	5	1	1.02643191	0.02438896
chr12	20210540	20410956	200417	1	1	2	0.00478973	0.30102999	5	1	1.02643191	0.05404976
chr12	20410956	20547035	136080	2	2	2	0.02162467	0.30102999	5	1	0.68214471	0.05404976
chr12	20547035	20547094	60	1	2	3	0.02162467	0.51676182	5	1	0.68214471	0.02438896
chr12	20547094	20643583	96490	2	1	3	0.00478973	0.51676182	5	1	1.02643191	0.02438896
chr12	20643583	20874888	231306	5	1	3	0.00478973	0.30102999	5	2	1.02643191	0.08289318
chr12	20874888	20951594	76707	3	1	4	0.00478973	0.47744371	5	2	1.02643191	0.0429175
chr12	20951594	20968685	17092	2	1	4	0.00204627	0.47744371	6	2	1.31360226	0.0429175
chr12	20968685	21069145	100461	2	1	4	0.00204627	0.76005302	6	1	1.31360226	0.01091641
chr12	21069145	21261409	192265	4	1	4	0.00204627	0.47744371	6	2	1.31360226	0.0429175
chr12	21261409	21326181	64773	3	1	6	8.47E-04	0.91219088	7	2	1.62048027	0.01053319
chr12	21326181	21427381	101201	2	1	5	0.00204627	0.68214471	6	2	1.31360226	0.02162467
chr12	21427381	21445371	17991	2	2	5	0.00493743	0.45545077	7	3	1.16581773	0.05670724
chr12	21445371	21531577	86207	4	2	5	3.90E-04	0.45545077	10	3	2.0620585	0.05670724
chr12	21531577	21532429	853	2	2	8	3.90E-04	1.07548421	10	3	2.0620585	0.0079614
chr12	21532429	21636284	103856	2	2	5	3.90E-04	0.45545077	10	3	2.0620585	0.05670724
chr12	21636284	21661429	25146	2	2	7	3.90E-04	0.84395715	10	3	2.0620585	0.01598258
chr12	21661429	21689158	27730	3	3	7	0.00170589	0.84395715	10	3	1.61091002	0.01598258
chr12	21689158	21741592	52435	3	2	6	3.90E-04	0.63695542	10	3	2.0620585	0.03070643
chr12	21741592	21787942	46351	1	2	5	3.90E-04	0.45545077	10	3	2.0620585	0.05670724
chr12	21787942	21852627	64686	1	2	4	3.90E-04	0.30102999	10	3	2.0620585	0.10122019
chr12	21852627	21852686	60	1	3	5	0.00170589	0.45545077	10	3	1.61091002	0.05670724
chr12	21852686	22017368	164683	4	2	5	3.90E-04	0.45545077	10	3	2.0620585	0.05670724
chr12	22017368	22017427	60	1	3	5	7.28E-04	0.45545077	11	3	1.91540774	0.05670724
chr12	22017427	22069981	52555	1	3	5	0.00170589	0.45545077	10	3	1.61091002	0.05670724
chr12	22069981	22089612	19632	2	3	6	0.00170589	0.63695542	10	3	1.61091002	0.03070643
chr12	22089612	22204517	114906	1	3	5	0.00170589	0.45545077	10	3	1.61091002	0.05670724
chr12	22204517	22239923	35407	3	3	5	7.28E-04	0.45545077	11	3	1.91540774	0.05670724
chr12	22239923	22401995	162073	2	3	4	7.28E-04	0.30102999	11	3	1.91540774	0.10122019
chr12	22401995	22440065	38071	2	3	5	7.28E-04	0.45545077	11	3	1.91540774	0.05670724
chr12	22440065	22440123	59	1	3	6	7.28E-04	0.63695542	11	3	1.91540774	0.03070643
chr12	22440123	22478115	37993	1	3	6	7.28E-04	0.91219088	11	2	1.91540774	0.01053319
chr12	22478115	22506561	28447	1	2	6	1.52E-04	0.91219088	11	2	2.40668355	0.01053319
chr12	22506561	22602778	96218	1	2	5	1.52E-04	0.68214471	11	2	2.40668355	0.02162467
chr12	22602778	22627734	24957	2	2	5	1.52E-04	0.45545077	11	3	2.40668355	0.05670724
chr12	22627734	22674213	46480	2	2	4	1.52E-04	0.47744371	11	2	2.40668355	0.0429175
chr12	22674213	22694990	20778	2	2	4	1.52E-04	0.30102999	11	3	2.40668355	0.10122019
chr12	22694990	22695049	60	1	3	4	7.28E-04	0.30102999	11	3	1.91540774	0.10122019
chr12	22695049	22784758	89710	2	2	3	3.90E-04	0.17593012	10	3	2.0620585	0.17593012
chr12	22784758	22838216	53459	3	2	2	9.54E-04	0.08289318	9	3	1.74076927	0.30102999
chr12	22838216	22892201	53986	2	2	3	3.90E-04	0.17593012	10	3	2.0620585	0.17593012
chr12	22892201	23011040	118840	1	2	2	9.54E-04	0.08289318	9	3	1.74076927	0.30102999
chr12	23011040	23155279	144240	1	1	1	1.30E-04	0.02438896	9	3	2.29264207	0.51676182
chr12	23155279	23265912	110634	1	1	1	1.30E-04	0.05404976	9	2	2.29264207	0.30102999
chr12	23265912	23386854	120943	1	1	1	1.30E-04	0.1218695	9	1	2.29264207	0.1218695
chr12	23386854	23386913	60	1	1	2	1.30E-04	0.1575501	9	2	2.29264207	0.1575501
chr12	23386913	23488498	101586	1	1	1	3.39E-04	0.1218695	8	1	1.94674965	0.1218695
chr12	23488498	23488557	60	1	1	1	3.39E-04	0.05404976	8	2	1.94674965	0.30102999
chr12	23488557	23685603	197047	1	1	0	3.39E-04	0	8	1	1.94674965	0.30102999
chr12	23685603	23685662	60	1	2	0	0.00221948	0	8	1	1.44210395	0.30102999
chr12	23685662	23807580	121919	2	2	0	0.00493743	0	7	1	1.16581773	0.30102999
chr12	23807580	23807639	60	1	2	1	0.00493743	0.1218695	7	1	1.16581773	0.1218695
chr12	23807639	23854642	47004	1	2	0	0.00493743	0	7	1	1.16581773	0.30102999
chr12	23854642	23904524	49883	1	1	0	8.47E-04	0	7	1	1.62048027	0.30102999
chr12	23904524	24022758	118235	3	1	1	8.47E-04	0.1218695	7	1	1.62048027	0.1218695
chr12	24022758	24022817	60	1	2	1	0.00493743	0.05404976	7	2	1.16581773	0.30102999
chr12	24022817	24102502	79686	2	1	1	8.47E-04	0.1218695	7	1	1.62048027	0.1218695
chr12	24102502	24283358	180857	4	2	1	0.00493743	0.1218695	7	1	1.16581773	0.1218695
chr12	24283358	24389848	106491	4	2	2	0.00493743	0.30102999	7	1	1.16581773	0.05404976
chr12	24389848	24489386	99539	3	2	2	0.00493743	0.1575501	7	2	1.16581773	0.1575501
chr12	24489386	24554915	65530	1	1	2	8.47E-04	0.1575501	7	2	1.62048027	0.1575501
chr12	24554915	24607065	52151	2	1	2	1.30E-04	0.1575501	9	2	2.29264207	0.1575501
chr12	24607065	24607124	60	1	2	3	3.90E-04	0.30102999	10	2	2.0620585	0.08289318
chr12	24607124	24729689	122566	3	1	3	4.80E-05	0.30102999	10	2	2.6588298	0.08289318
chr12	24729689	24861225	131537	1	1	3	1.30E-04	0.30102999	9	2	2.29264207	0.08289318
chr12	24861225	24861284	60	1	1	3	1.30E-04	0.17593012	9	3	2.29264207	0.17593012
chr12	24861284	25027344	166061	3	1	2	1.30E-04	0.30102999	9	1	2.29264207	0.05404976
chr12	25027344	25157644	130301	5	1	2	4.80E-05	0.30102999	10	1	2.6588298	0.05404976
chr12	25157644	25157703	60	1	3	2	7.28E-04	0.30102999	11	1	1.91540774	0.05404976
chr12	25157703	25242667	84965	2	2	2	3.90E-04	0.30102999	10	1	2.0620585	0.05404976
chr12	25242667	25286243	43577	3	2	2	1.52E-04	0.30102999	11	1	2.40668355	0.05404976
chr12	25286243	25348677	62435	2	3	2	7.28E-04	0.30102999	11	1	1.91540774	0.05404976
chr12	25348677	25348736	60	1	3	3	7.28E-04	0.51676182	11	1	1.91540774	0.02438896
chr12	25348736	25485811	137076	3	2	2	1.52E-04	0.30102999	11	1	2.40668355	0.05404976
chr12	25485811	25577112	91302	4	3	2	7.28E-04	0.30102999	11	1	1.91540774	0.05404976
chr12	25577112	25577171	60	1	3	2	7.28E-04	0.1575501	11	2	1.91540774	0.1575501
chr12	25577171	25629086	51916	1	2	2	1.52E-04	0.1575501	11	2	2.40668355	0.1575501
chr12	25629086	25737386	108301	5	2	2	1.52E-04	0.30102999	11	1	2.40668355	0.05404976
chr12	25737386	25737445	60	1	2	3	1.52E-04	0.30102999	11	2	2.40668355	0.08289318
chr12	25737445	25944355	206911	2	2	3	1.52E-04	0.51676182	11	1	2.40668355	0.02438896
chr12	25944355	26069343	124989	1	2	2	1.52E-04	0.61140001	11	0	2.40668355	0
chr12	26069343	26130832	61490	2	4	2	0.00247414	0.61140001	11	0	1.52371709	0
chr12	26130832	26165927	35096	3	4	2	0.00247414	0.30102999	11	1	1.52371709	0.05404976
chr12	26165927	26219894	53968	2	4	3	0.00247414	0.51676182	11	1	1.52371709	0.02438896
chr12	26219894	26219953	60	1	5	3	0.00666883	0.51676182	11	1	1.20557689	0.02438896
chr12	26219953	26333635	113683	2	4	2	0.00247414	0.61140001	11	0	1.52371709	0
chr12	26333635	26333694	60	1	4	3	0.00247414	0.51676182	11	1	1.52371709	0.02438896
chr12	26333694	26385081	51388	2	3	2	7.28E-04	0.61140001	11	0	1.91540774	0
chr12	26385081	26608568	223488	4	4	2	0.00247414	0.61140001	11	0	1.52371709	0
chr12	26608568	26647203	38636	1	3	2	7.28E-04	0.61140001	11	0	1.91540774	0
chr12	26647203	26713281	66079	1	3	1	7.28E-04	0.30102999	11	0	1.91540774	0
chr12	26713281	27113335	400055	11	4	1	0.00247414	0.30102999	11	0	1.52371709	0
chr12	27113335	27156216	42882	5	5	1	0.00666883	0.30102999	11	0	1.20557689	0
chr12	27156216	27458545	302330	5	4	1	0.00247414	0.30102999	11	0	1.52371709	0
chr12	27458545	27486768	28224	2	5	1	0.00666883	0.30102999	11	0	1.20557689	0
chr12	27486768	27523096	36329	1	4	1	0.00247414	0.30102999	11	0	1.52371709	0
chr12	27523096	27523153	58	1	4	1	0.00247414	0.1218695	11	1	1.52371709	0.1218695
chr12	27523153	27655240	132088	4	2	1	1.52E-04	0.1218695	11	1	2.40668355	0.1218695
chr12	27655240	27728643	73404	3	3	1	7.28E-04	0.1218695	11	1	1.91540774	0.1218695
chr12	27728643	27728702	60	1	3	1	2.93E-04	0.1218695	12	1	2.24559516	0.1218695
chr12	27728702	27909069	180368	5	2	1	1.52E-04	0.1218695	11	1	2.40668355	0.1218695
chr12	27909069	27909127	59	1	3	1	7.28E-04	0.1218695	11	1	1.91540774	0.1218695
chr12	27909127	28111479	202353	3	2	1	1.52E-04	0.1218695	11	1	2.40668355	0.1218695
chr12	28111479	28123884	12406	2	2	0	1.52E-04	0	11	1	2.40668355	0.30102999
chr12	28123884	28306461	182578	1	2	0	3.90E-04	0	10	1	2.0620585	0.30102999
chr12	28306461	28306520	60	1	2	0	1.52E-04	0	11	1	2.40668355	0.30102999
chr12	28306520	28457588	151069	3	2	0	3.90E-04	0	10	1	2.0620585	0.30102999
chr12	28457588	28457643	56	1	2	0	1.52E-04	0	11	1	2.40668355	0.30102999
chr12	28457643	28544289	86647	2	2	0	3.90E-04	0	10	1	2.0620585	0.30102999
chr12	28544289	28575393	31105	2	2	0	1.52E-04	0	11	1	2.40668355	0.30102999
chr12	28575393	28600856	25464	2	2	0	5.57E-05	0	12	1	2.77580851	0.30102999
chr12	28600856	28702380	101525	3	2	0	1.52E-04	0	11	1	2.40668355	0.30102999
chr12	28702380	28903466	201087	1	1	0	4.80E-05	0	10	1	2.6588298	0.30102999

chr12	28903466	29329868	426403	3	1	0	1.30E-04	0	9	1	2.29264207	0.30102999
chr12	29329868	29423538	93671	2	1	0	3.39E-04	0	8	1	1.94674965	0.30102999
chr12	29423538	29423589	52	1	1	0	1.30E-04	0	9	1	2.29264207	0.30102999
chr12	29423589	29532012	108424	4	1	0	3.39E-04	0	8	1	1.94674965	0.30102999
chr12	29532012	29575283	43272	1	1	0	0.00204627	0	6	1	1.31360226	0.30102999
chr12	29575283	29813762	238480	6	1	0	0.01091641	0	4	1	0.76005302	0.30102999
chr12	29813762	29886957	73196	3	1	0	0.00478973	0	5	1	1.02643191	0.30102999
chr12	29886957	29914459	27503	1	1	0	0.01091641	0	4	1	0.76005302	0.30102999
chr12	29914459	30013033	98575	2	1	0	0.00478973	0	5	1	1.02643191	0.30102999
chr12	30013033	30013092	60	1	1	0	1.30E-04	0	9	1	2.29264207	0.30102999
chr12	30013092	30218673	205582	1	1	0	3.39E-04	0	8	1	1.94674965	0.30102999
chr12	30218673	30590907	372235	2	1	0	8.47E-04	0	7	0	1.62048027	0
chr12	30590907	30833491	242585	4	2	0	0.00493743	0	7	0	1.16581773	0
chr12	30833491	31157095	323605	7	2	0	0.00221948	0	8	0	1.44210395	0
chr12	31157095	31157154	60	1	2	0	0.00221948	0	8	2	1.44210395	0.61140001
chr12	31157154	31241554	84401	1	2	0	0.00221948	0	8	1	1.44210395	0.30102999
chr12	31241554	31313985	72432	1	2	0	0.00221948	0	8	0	1.44210395	0
chr12	31313985	31356945	42961	2	2	0	0.00221948	0	8	1	1.44210395	0.30102999
chr12	31356945	31357004	60	1	2	0	9.54E-04	0	9	1	1.74076927	0.30102999
chr12	31357004	31408631	51628	1	2	0	0.00221948	0	8	1	1.44210395	0.30102999
chr12	31408631	31477869	69239	3	2	0	0.00221948	0	8	2	1.44210395	0.61140001
chr12	31477869	31542346	64478	1	2	0	0.00493743	0	7	2	1.16581773	0.61140001
chr12	31542346	31579238	36893	2	2	0	0.00221948	0	8	2	1.44210395	0.61140001
chr12	31579238	31739139	159902	6	2	0	9.54E-04	0	9	2	1.74076927	0.61140001
chr12	31739139	31739198	60	1	3	0	0.00378107	0	9	2	1.33111237	0.61140001
chr12	31739198	31819003	79806	1	3	0	0.00378107	0	9	1	1.33111237	0.30102999
chr12	31819003	31933396	114394	4	4	0	0.01077081	0	9	1	1.01542894	0.30102999
chr12	31933396	31933455	60	1	6	0	0.04875589	0	9	1	0.5732208	0.30102999
chr12	31933455	32049529	116075	1	5	0	0.02473314	0	9	1	0.76806864	0.30102999
chr12	32049529	32049588	60	1	5	0	0.01320236	0	10	1	0.97390707	0.30102999
chr12	32049588	32145402	95815	2	4	0	0.01077081	0	9	1	1.01542894	0.30102999
chr12	32145402	32208785	63384	1	3	0	0.00378107	0	9	1	1.33111237	0.30102999
chr12	32208785	32325075	116291	5	3	0	0.00170589	0	10	1	1.61091002	0.30102999
chr12	32325075	32325134	60	1	4	0	0.00530919	0	10	1	1.2568129	0.30102999
chr12	38572677	38956458	383782	5	0	1	0	0.1218695	1	1	0.30102999	0.1218695
chr12	38956458	39045475	89018	2	0	3	0	0.30102999	1	2	0.30102999	0.08289318
chr12	39045475	39114179	68705	3	0	3	0	0.30102999	2	2	0.61140001	0.08289318
chr12	39114179	39114238	60	1	0	3	0	0.30102999	3	2	0.93173516	0.08289318
[truncated: 1,174,985 more chars]
