# Supplementary material for: Identification by array comparative genomic hybridization of a new amplicon on chromosome 17q highly recurrent in BRCA1 mutated triple negative breast cancer
Source: Breast Cancer Res. 2014 Nov 22;16:466. doi: 10.1186/s13058-014-0466-y (PMC4303204; doi:10.1186/s13058-014-0466-y)
Supplement: Supplementary file 2 — Additional file 2: Table S2.: Gains and losses detected in BRCA1 non-mutated triple-negative breast cancer (TNBC) by array comparative genomic hybridization (CGH). (PDF 35 KB) [file 13058_2014_466_MOESM2_ESM.pdf]

Chromosomes	Cytobandes	Max. % Gains	Max. % Losses
chr1	p36.31	4,35	23,81
chr1	p36.22	9,94	34,99
chr1	p36.13 - p36.12	9,52	34,99
chr1	p35.3 - p35.2	5,18	29,40
chr1	p34.3 - p31.3	29,40	23,81
chr1	p31.1	24,22	5,59
chr1	p22.3 - p21.2	28,57	24,22
chr1	q24.2 - q32.3	49,69	8,70
chr2	p24.2 - p24.1	24,22	8,70
chr2	p22.3 - p22.2	34,16	4,35
chr2	p21 - p16.3	29,40	4,35
chr2	p16.1 - p13.3	29,40	4,35
chr2	q13 - q14.3	9,52	28,99
chr2	q21.2 - q22.1	9,94	28,57
chr2	q23.1 - q23.2	3,93	23,81
chr2	q31.1 - q31.2	3,93	24,22
chr2	q32.2	3,93	24,22
chr2	q33.1	0,41	23,81
chr2	q35	4,76	24,22
chr2	q36.2 - q37.3	4,76	34,58
chr3	p26.3	23,81	3,93
chr3	p26.1 - p25.3	24,22	23,81
chr3	p23	0,41	29,81
chr3	p21.31	4,76	37,89
chr3	p21.1 - p14.3	1,40	37,47
chr3	p14.1 - p13	4,76	36,23
chr3	p12.2	4,76	28,99
chr3	q11.2 - q12.1	23,81	23,81
chr3	q13.12	24,22	0,00
chr3	q13.2	15,11	23,81
chr3	q13.31 - q13.33	24,22	23,81
chr3	q21.2 - q21.3	7,87	24,22
chr4	p16.3 - p14	13,04	40,58
chr4	q12	29,40	23,81
chr4	q13.2 - q13.3	28,99	3,52
chr4	q21.1 - q21.21	28,99	11,80
chr4	q27	28,57	2,07
chr4	q28.3	23,81	23,81
chr4	q31.1 - q31.21	23,81	7,87
chr4	q32.1 - q32.3	0,00	34,58
chr4	q34.3	4,76	29,40
chr4	q35.2	4,76	41,41
chr5	p15.2	28,57	4,76
chr5	p14.2	24,22	4,35
chr5	p13.3	24,22	0,83
chr5	q13.1	0,00	40,17
chr5	q14.1 - q15	0,00	40,58
chr5	q21.1 - q21.3	0,00	33,13
chr5	q22.2	4,35	36,65
chr5	q23.1	0,00	29,81
chr5	q31.1 - q32	0,00	40,99
chr5	q33.2 - q34	4,76	35,40
chr6	p12.2 - p11.2	29,81	4,76
chr6	q13 - q14.3	24,22	19,05
chr6	q16.1	24,22	14,29
chr6	q16.3	24,22	10,35
chr6	q22.31 - q23.2	34,16	14,29
chr6	q24.2	24,22	5,59
chr6	q25.1 - q25.2	23,81	28,99
chr7	p22.1	21,33	3,52
chr7	p15.2 - p14.2	23,81	24,22
chr7	p13	10,77	24,22
chr7	q11.22 - q11.23	15,11	24,22
chr7	q35 - q36.1	29,81	6,00

chr8	p12	5,18	34,58
chr8	p21.3 - p21.2	0,00	36,65
chr8	q11.21	35,40	4,35
chr8	q13.1	35,82	0,41
chr8	q21.11	35,82	4,76
chr8	q22.1	37,47	0,00
chr8	q24.3	25,47	5,18
chr9	p24.3 - p24.2	3,52	24,22
chr9	p23	3,11	23,81
chr9	p22.3	6,00	24,22
chr9	p22.1	24,22	19,46
chr9	p21.3 - p21.2	24,22	24,22
chr9	p13.3 - p13.2	24,22	20,29
chr9	q22.31 - q22.33	5,18	24,22
chr9	q31.2	0,00	24,22
chr9	q32 - q34.11	4,76	35,40
chr9	q34.13 - q34.3	4,35	40,17
chr10	p12.33 - p12.1	45,76	0,00
chr10	p11.21	35,40	4,76
chr10	q11.22 - q21.3	19,46	28,57
chr10	q23.1 - q23.2	8,28	29,40
chr10	q24.1 - q26.3	4,35	49,28
chr11	p15.5 - p15.1	8,70	44,51
chr11	p13	19,88	24,22
chr11	p11.2	23,81	15,53
chr11	q12.1	24,22	9,52
chr11	q12.3	23,81	9,94
chr11	q13.4 - q14.1	11,18	28,99
chr11	q14.3	6,00	24,22
chr11	q21	6,83	28,99
chr11	q23.1	29,40	5,59
chr11	q23.3	29,81	2,48
chr11	q24.1 - q24.3	24,22	21,33
chr12	p13.31 - p11.21	39,34	14,29
chr12	q13.13 - q14.1	4,76	36,65
chr12	q21.33	4,76	29,61
chr12	q23.3 - q24.11	4,35	35,40
chr12	q24.13 - q24.21	4,76	34,99
chr12	q24.23 - q24.31	3,93	35,40
chr13	q14.12 - q14.13	4,35	40,99
chr13	q22.2	9,52	29,81
chr13	q33.2 - q33.3	24,22	8,28
chr14	q11.2	9,94	34,99
chr14	q12	5,18	34,99
chr14	q22.1	4,35	34,99
chr14	q23.1	0,00	36,23
chr14	q24.2 - q24.3	4,76	40,58
chr15	q21.2 - q22.31	21,33	35,82
chr15	q24.1 - q24.2	21,74	20,70
chr15	q26.3	29,40	15,11
chr16	q12.1 - q24.3	20,91	34,16
chr17	p13.2 - p13.1	9,52	36,65
chr17	q21.2 - q21.31	8,28	35,40
chr17	q24.1	24,84	0,41
chr17	q24.3 - q25.3	28,36	19,46
chr18	p11.32 - p11.31	34,16	5,18
chr18	p11.22 - p11.21	29,40	3,93
chr18	q12.1	9,94	25,67
chr18	q12.3	0,00	24,84
chr18	q21.31 - q21.32	23,81	20,29
chr19	p13.3 - p13.2	29,40	34,58
chr19	p13.11 - p12	35,40	10,77
chr19	q13.11 - q13.31	16,15	29,40
chr19	q13.33 - q13.43	21,33	39,75

chr20	p13	6,00	28,99
chr20	q12	1,24	28,57
chr20	q13.13 - q13.2	29,81	4,35
chr21	q22.12 - q22.3	28,99	11,80
chr22	q12.3 - q13.33	33,54	47,62
chrX	p22.12	0,00	28,57
chrX	q21.1	5,18	34,58
chrX	q23 - q24	9,94	24,22
chrX	q26.3	4,76	29,40
chrX	q27.3 - q28	9,52	29,40
