# Supplementary material for: Identification by array comparative genomic hybridization of a new amplicon on chromosome 17q highly recurrent in BRCA1 mutated triple negative breast cancer
Source: Breast Cancer Res. 2014 Nov 22;16:466. doi: 10.1186/s13058-014-0466-y (PMC4303204; doi:10.1186/s13058-014-0466-y)
Supplement: Supplementary file 3 — Additional file 3: Table S3.: List of the 145 most important segments. (PDF 370 KB) [file 13058_2014_466_MOESM3_ESM.pdf]

Table 3 : List of the 145 segments used to build the *BRCA1* classifier.

The values are classified from the most important to the less important.

Chromosome n°_start-stop	Variable importance	Gain_Losses
chr17_78260810_81029941	100	gain
chrX_3542001_46323590	99.2277992	losses
chr19_45681759_54221324	94.2084942	gain
chr22_33115310_51178264	90.3474903	losses
chr19_28272497_45588627	89.5752896	gain
chr16_71251249_90111263	84.1698842	gain
chr10_110242525_135404523	81.8532819	losses
chr17_25403446_78198553	81.8532819	losses
chr5_30349974_44873491	80.3088803	losses
chr16_46564557_62705691	78.7644788	gain
chr10_9846848_36912799	76.8339768	gain
chr3_93949_48556219	75.6756757	losses
chr6_61982931_82004749	74.9034749	losses
chr2_42444_82361952	74.1312741	gain
chr19_54263971_59092570	72.2007722	gain
chr4_135347238_179738838	71.8146718	losses
chr1_155296964_156102366	71.042471	gain
chr4_39365720_42175062	67.953668	gain
chr5_84819841_104823961	67.1814672	losses
chr4_64367629_130946954	66.023166	losses
chr1_146564743_155289073	64.0926641	gain
chr11_218365_37442438	61.7760618	gain
chr6_117130571_170890108	61.7760618	gain
chr16_106271_23368052	60.2316602	losses
chr2_220474392_243041364	59.4594595	gain
chr8_8130630_42056282	59.4594595	gain
chr12_125012869_133447358	58.6872587	gain
chr11_55481624_96847330	57.9150579	losses
chr20_53612439_62893189	57.1428571	losses
chr4_59911076_63813565	55.984556	losses
chr18_14316_14358321	54.4401544	gain
chr5_151737_25469388	53.2818533	gain
chr5_26068066_29903010	53.2818533	losses
chr13_85236169_115059020	52.8957529	losses
chr14_20253739_55764313	52.8957529	losses
chr9_71035346_141008915	52.5096525	losses
chr1_156132786_241799145	51.7374517	losses
chr4_71552_18839648	51.7374517	losses
chr10_73348402_109862404	51.3513514	gain
chr15_22318597_88552022	50.1930502	losses
chr14_87514040_107258824	49.4208494	losses
chrX_46363189_88305581	49.4208494	losses
chr3_48603039_75275195	48.2625483	losses
chr7_68194327_76200216	47.1042471	losses
chr8_145516091_146280020	47.1042471	gain
chr1_88079298_104307708	46.7181467	gain
chr1_241913757_249212668	45.5598456	losses

chr11_98487343_134868407	44.7876448	losses
chr12_230421_32325134	44.7876448	losses
chr4_52689101_59258509	44.7876448	losses
chr6_93007777_95148784	44.4015444	losses
chr20_121521_24590067	44.015444	losses
chr7_76476799_153691728	44.015444	gain
chr13_81852809_84832155	43.6293436	losses
chr5_165768313_177259960	43.6293436	losses
chr10_148206_9645787	42.8571429	losses
chr20_29888477_53352073	42.8571429	gain
chr16_23417375_31928113	42.4710425	losses
chr3_117950181_197840339	42.0849421	losses
chr8_221611_6914076	42.0849421	gain
chr13_19296544_81634108	41.6988417	gain
chr10_42889244_46158215	40.9266409	gain
chr3_75936605_86917377	40.9266409	losses
chr22_25481324_28172387	40.5405405	losses
chr6_95417726_103405100	40.5405405	gain
chr19_17623422_23624787	39.7683398	losses
chr8_47681335_142680829	39.7683398	gain
chr11_55375127_55375186	39.3822394	losses
chr21_14861256_14895949	39.3822394	losses
chr3_117516385_117780353	39.3822394	gain
chr4_42414756_47175094	39.3822394	losses
chr6_82363693_82577829	39.3822394	losses
chr7_68065098_68065157	39.3822394	gain
chr13_85075294_85075353	38.6100386	losses
chr3_93580736_117166718	38.6100386	losses
chr7_92532_22527063	38.2239382	gain
chr6_82742906_92045753	37.8378378	losses
chr11_39233962_49532643	37.0656371	gain
chr14_55818972_86933797	37.0656371	losses
chr4_180166540_190896674	37.0656371	losses
chr6_255350_58014532	36.2934363	gain
chr6_104137729_117050796	36.2934363	losses
chr21_15170361_39528526	34.7490347	gain
chr5_49690172_84349709	33.976834	losses
chr5_105623245_159900889	33.976834	losses
chr19_327273_17531973	33.2046332	gain
chr9_271257_39156954	33.2046332	gain
chr1_38154020_87873063	32.8185328	losses
chr22_17280847_25368098	32.8185328	gain
chr2_87776206_89508067	32.8185328	losses
chr1_104852325_105300334	32.4324324	gain
chr11_97854495_97854554	32.4324324	gain
chr16_33385740_33604468	32.4324324	gain
chr2_82510808_83452304	32.4324324	losses
chr2_104356251_104356310	32.4324324	gain
chr2_194682359_194682418	32.4324324	gain
chr21_39618392_48067924	32.4324324	losses
chr4_59598916_59598975	30.5019305	losses
chr7_63374309_67616356	30.5019305	losses

chr8_142840194_142909763	30.1158301	gain
chr12_42945155_124931849	29.7297297	losses
chr4_19370059_32023735	29.3436293	losses
chr17_87009_21199405	29.3436293	losses
chr7_153761547_158909738	28.1853282	losses
chr7_22609575_56786860	27.027027	gain
chr2_83613013_87277610	26.2548263	gain
chr1_759762_1054794	26.2548263	gain
chr12_38572677_42873860	26.2548263	gain
chr16_63494668_63494727	25.4826255	gain
chr2_146048486_146048545	25.4826255	gain
chr4_131400699_131916402	25.4826255	gain
chr5_165212295_165402101	25.4826255	losses
chr1_1110548_38077902	24.7104247	gain
chr5_177383773_180684501	24.3243243	losses
chr22_28278003_33049496	22.7799228	losses
chr8_143008635_143577971	22.3938224	losses
chr18_18539853_77982126	22.3938224	gain
chr8_143692418_145225218	21.6216216	losses
chr2_195799327_220437841	21.2355212	losses
chr10_47604818_53609363	20.8494208	gain
chr10_59028244_73247691	20.4633205	gain
chr4_33080365_39329273	20.0772201	losses
chr2_110895981_110980401	19.6911197	gain
chr15_88628250_102465355	19.3050193	losses
chr2_151792931_193773633	19.3050193	losses
chrX_92377119_113767651	19.3050193	gain
chrX_91504849_91519622	19.3050193	losses
chr11_38868079_38868138	18.5328185	gain
chr22_17096855_17154047	18.5328185	losses
chr10_110057187_110057246	18.1467181	gain
chr10_53709895_58686145	17.7606178	losses
chr4_132962005_135137668	16.6023166	losses
chr1_106497293_145747269	16.2162162	gain
chr2_95529039_104099903	15.4440154	losses
chr5_159960920_164973086	15.4440154	losses
chr2_123973518_145827808	15.0579151	gain
chr21_9832448_9834682	13.1274131	losses
chr2_111399243_123161667	13.1274131	losses
chrX_113852609_152215637	13.1274131	losses
chrX_152356063_152356122	10.4247104	losses
chrX_153630137_154754171	8.49420849	losses
chr2_104514353_110427254	6.56370656	gain
chrX_152485412_153609163	5.40540541	losses
chr16_63841326_70800379	0.77220077	losses
chr2_147008238_151128706	0	gain
