# Supplementary material for: Identification by array comparative genomic hybridization of a new amplicon on chromosome 17q highly recurrent in BRCA1 mutated triple negative breast cancer
Source: Breast Cancer Res. 2014 Nov 22;16:466. doi: 10.1186/s13058-014-0466-y (PMC4303204; doi:10.1186/s13058-014-0466-y)
Supplement: Supplementary file 4 — Additional file 4: Supplementary methods and results. Table S4. classification results. Table S5. list of samples from the triple-negative breast cancer (TCGA) breast cancer used in this study. Table S6. Relative gene expression in mutated and non-mutated TNBC (Taqman low density arrays). Table S7. Relative gene expression in mutated and non-mutated TNBC (Taqman qPCR). Table S8 COLT-cancer database analysis. Table S8. GenomeRNAI database analysis. Figures S1 to S3. (PDF 904 KB) [file 13058_2014_466_MOESM4_ESM.pdf]

Additional file 4 :

Supplementary material and methods.

1. Analysis of The Cancer Genome Atlas Breast Invasive Carcinoma data set.

We analysed the Breast Invasive Carcinoma data set from TCGA (TCGA, Nature 2012). This data sets of 825 samples mutation data for 507 samples, copy number data for 778 samples, expression data for 526 samples and methylation data for 311 samples. We selected TN samples that are ER, PR and HER2 negative (IHC) and of the basal-like subtype (PAM50). HER2+ samples are ER and PR positive or negative and HER2 positive and of the HER2-enriched subtype (PAM50). The luminal samples are HER2 negative (IHC) and of the Luminal A or B subtype (PAM50) (table 5). The data were retrieved through the cBioPortal for Cancer Genomics site (http://www.cbioportal.org/public-portal/) or directly through the TCGA data portal (https://tcga-data.nci.nih.gov/tcga/). In this TCGA cohort, we found 14 samples with mutation in *BRCA1* (10 triple negative BC, 3 Lum A BC, 1 HER2+ BC).

CNV data were retrieved as log2 ratio for the genes in the 17q25.3 region, and genes with a log2 ratio above 0.2 were considered as gain.

For expression data analyses, Z-score were retrieved for the genes in the 17q25.3 region. The Z-score value indicates the number of standard deviations away from the mean of expression in the reference population. This measure determines whether a gene is up- or down-regulated relative to the normal samples or all other tumor samples.

HM27 methylation data are also available. The measure of the level of DNA methylation at each CpG site is scored as beta values ranging from 0 to 1, with values close to 0 indicating low levels of DNA methylation and values close to 1 indicating high levels of DNA methylation. Samples with HM27 beta value >0.5 and *BRCA1* expression downregulation were considered as methylated.

2. Construction of a predictive model

STEP 1: **Reference Segment definition**

The segments are defined per sample using the Agilent algorithm Z-score. Overlapping segments are merged together in larger segments using the *reduce* function available in the R Bioconductor *GenomicRanges* package (http://www.bioconductor.org/packages/release/bioc/html/GenomicRanges.html). A total of 145 segments were defined (Additional file 4, Table S3). These segments are used as a common reference for all samples.

STEP 2: **Value per reference segment per sample**

For each reference segment defined in step 1 and each sample, the mean value is calculated. Ex: if sample 3 has aberrations in segments 4 and 10 (segments found for that sample), that are both contained in reference segment 2, we compute the mean value: (value for segment 4 + value for segment 10)/2. If no segment for that sample is found as being part of the reference segment, we assign 0 as value.

This results in a quite sparse matrix (= matrix with many 0's). Methods like Support Vector Machine (SVM) are robust against sparse data matrix. Moreover SVM allows to build models for high-dimensional data sets.

STEP 3: **Building the model**

A training set was created with 75% of each tumour group (34 samples). The remaining samples (25%) were used for validation (10 samples). SVM as implemented in the R package '*e1071*' (http://cran.r-project.org/web/packages/e1071/index.html) and wrapped in the R package 'caret' (http://caret.r-forge.r-project.org/) has been used for building the model.

The area under the ROC curve (Receiver Operating Characteristic curve) has been chosen as the performance measure. The Leave-One-Out Cross-Validation method has been selected for model validation.

The parameters C and sigma were tuned to get their optimal values (maximizing the area under the ROC curve, or AUC): tested values for C = 0.25,0.5,1 and sigma = 0.001 to 0.01 with a step of 0.001.

Supplementary Results

1. Construction of a predictive model

A predicting model has been built based on 145 variables using SVM. The following parameter values were chosen: C = 0.5 and sigma = 0.004. Those values were selected for 2 reasons: they show a high AUC and a stable AUC (a small change in these parameter values does not degrade the AUC).

With the built model, we reach an AUC = 0.8 both in the validation set and in the test set, which indicates a stability of the model and reduce the risk of overfitting. The most important variables as defined by the *varImp* function available in the '*e1071'* package are given in the table S3.

We tried to reduce the model to the 20 most important variables. This operation resulted in a decrease of the AUC to 0.7. Reducing the model to the 5 most important variables decreased the AUC to 0.6. The density plots of the 5 most important variables are showed in figure S1.

The most important variable is chr17_78260810_81029941, which was also the one previously selected. Using this model, we were able to assign 21/23 BRCA1 mutated samples to the mutated group (sensitivity of 91 % (metrics based on all data, i.e. test and validation data). All the non mutated samples (21/21) were assigned to the right class (specificity of 100%) (Table S4). The positive predictive value is 100 % and the negative predictive value is 91%.

Supplementary Figures and tables.

Table S4 : classification results.

Probability scores for the *BRCA1* mutated and non mutated samples. The positive class is the mutated class. NM : non mutated M: mutated

BRCA1 status	M	NM	Selected Class
NM	0.03756681	0.96243319	NM
NM	0.06168244	0.93831756	NM
NM	0.46391593	0.53608407	NM
NM	0.01411068	0.98588932	NM
NM	0.27414678	0.72585322	NM
NM	0.29803175	0.70196825	NM
NM	0.16609179	0.83390821	NM
NM	0.01055714	0.98944286	NM
NM	0.00808346	0.99191654	NM
NM	0.0265493	0.9734507	NM
NM	0.04387822	0.95612178	NM
NM	0.47724271	0.52275729	NM
NM	0.09769137	0.90230863	NM
NM	0.05690248	0.94309752	NM
NM	0.45139672	0.54860328	NM
NM	0.00714882	0.99285118	NM
NM	0.0121659	0.9878341	NM
NM	0.29761888	0.70238112	NM
NM	0.27021516	0.72978484	NM
NM	0.0354405	0.9645595	NM
NM	0.01342016	0.98657984	NM
M	0.6447504	0.3552496	M
M	0.74249595	0.25750405	M
M	0.60887179	0.39112821	M
M	0.41955305	0.58044695	NM
M	0.11986052	0.88013948	NM
M	0.88748761	0.11251239	M
M	0.88759534	0.11240466	M
M	0.84503455	0.15496545	M
M	0.88723173	0.11276827	M
M	0.6081164	0.3918836	M
M	0.88729109	0.11270891	M
M	0.88771814	0.11228186	M
M	0.85452423	0.14547577	M
M	0.81509475	0.18490525	M
M	0.88729108	0.11270892	M
M	0.75156498	0.24843502	M
M	0.78659429	0.21340571	M
M	0.88751379	0.11248621	M
M	0.75998406	0.24001594	M
M	0.57245218	0.42754782	M
M	0.70365798	0.29634202	M
M	0.62624739	0.37375261	M
M	0.63273671	0.36726329	M

Table S5: list of samples from the TCGA study used as a validation set. Samples in bold are *BRCA1* mutated.

TN (n=77)	Lum A (n=201)		Lum B (n=101)	HER2+ (n=38)
TCGA-A1-A0SK	TCGA-AN-A0XN	TCGA-B6-A0WZ	TCGA-A2-A0SW	TCGA-A2-A0T1
TCGA-A1-A0SP	TCGA-AN-A0XS	TCGA-B6-A0I5	TCGA-AR-A0TY	TCGA-BH-A0EE
TCGA-A2-A04P	TCGA-B6-A0WY	TCGA-B6-A0RV	TCGA-E2-A107	TCGA-A2-A0D1
TCGA-A2-A04Q	TCGA-BH-A0HK	TCGA-B6-A0RO	TCGA-AO-A0JI	TCGA-A2-A04W
TCGA-A2-A04T	TCGA-A7-A0CG	TCGA-B6-A0WT	TCGA-E2-A109	TCGA-AO-A12D
TCGA-A2-A04U	TCGA-A7-A0D9	TCGA-B6-A0IA	TCGA-BH-A0HW	TCGA-AO-A0JE
TCGA-A2-A0CM	TCGA-BH-A0HP	TCGA-B6-A0RI	TCGA-A2-A0CT	TCGA-A2-A0EQ
TCGA-A2-A0D0	TCGA-A2-A0YF	TCGA-A1-A0SE	TCGA-A8-A06N	TCGA-A8-A08X
TCGA-A2-A0ST	TCGA-A8-A091	TCGA-A2-A0EX	TCGA-A8-A07S	TCGA-A8-A0A7
TCGA-A2-A0SX	TCGA-AN-A0FS	TCGA-AO-A0JJ	TCGA-A8-A084	TCGA-AN-A04C
TCGA-A2-A0T0	TCGA-AN-A0XO	TCGA-A1-A0SD	TCGA-A8-A09Z	TCGA-AN-A0FV
TCGA-A2-A0T2	TCGA-AN-A0XT	TCGA-A1-A0SJ	TCGA-AN-A0AK	TCGA-C8-A12L
TCGA-A2-A0YE	TCGA-BH-A0BM	TCGA-A8-A06P	TCGA-AN-A0AM	TCGA-C8-A12P
TCGA-A2-A0YM	TCGA-BH-A0DG	TCGA-A8-A06T	TCGA-AN-A0AS	TCGA-C8-A12Q
TCGA-A2-A1G6	TCGA-A2-A0CU	TCGA-A8-A06Y	TCGA-AN-A0XR	TCGA-C8-A12Z
TCGA-A7-A0CE	TCGA-AR-A0TR	TCGA-A8-A07E	TCGA-AR-A0TT	TCGA-C8-A135
TCGA-A7-A0DA	TCGA-B6-A0X4	TCGA-A8-A07F	TCGA-BH-A0C3	TCGA-C8-A137
TCGA-A7-A26F	TCGA-BH-A0EA	TCGA-A8-A07G	TCGA-E2-A155	TCGA-E2-A14P
TCGA-A7-A26G	TCGA-BH-A18N	TCGA-A8-A07J	TCGA-E2-A15S	TCGA-E2-A1B0
TCGA-A7-A26I	TCGA-BH-A1EU	TCGA-A8-A083	TCGA-A2-A0SV	TCGA-C8-A1HF
TCGA-A8-A07C	TCGA-B6-A0X7	TCGA-A8-A086	TCGA-AO-A03O	TCGA-BH-A18R
TCGA-A8-A07O	TCGA-A2-A04V	TCGA-A8-A08A	TCGA-B6-A0WW	TCGA-BH-A0AW
TCGA-A8-A08R	TCGA-B6-A0RM	TCGA-A8-A08C	TCGA-BH-A18J	TCGA-A2-A0CX
TCGA-A8-A09X	TCGA-BH-A1ET	TCGA-A8-A08O	TCGA-B6-A0IC	TCGA-A8-A07I
TCGA-AN-A04D	TCGA-BH-A1EO	TCGA-A8-A08T	TCGA-B6-A0X5	TCGA-A8-A08B
TCGA-AN-A0AR	TCGA-B6-A0WS	TCGA-A8-A08Z	TCGA-B6-A0WV	TCGA-A8-A09G
TCGA-AN-A0AT	TCGA-B6-A0RP	TCGA-A8-A090	TCGA-B6-A0RL	TCGA-C8-A138
TCGA-AN-A0G0	TCGA-B6-A0IH	TCGA-A8-A093	TCGA-AR-A0U2	TCGA-E2-A152
TCGA-AO-A03U	TCGA-BH-A1ES	TCGA-A8-A09A	TCGA-B6-A0IB	TCGA-B6-A0I9
TCGA-AO-A0J2	TCGA-B6-A0X0	TCGA-A8-A09B	TCGA-AO-A0J7	TCGA-AR-A1AT
TCGA-AO-A0J4	TCGA-B6-A0RQ	TCGA-A8-A09T	TCGA-AO-A0J3	TCGA-BH-A0DZ
TCGA-AO-A0J6	TCGA-BH-A0HO	TCGA-A8-A09V	TCGA-D8-A13Y	TCGA-A2-A04X
TCGA-AO-A0JL	TCGA-BH-A0DQ	TCGA-A8-A0A1	TCGA-A7-A13F	TCGA-B6-A0RH
TCGA-AO-A128	TCGA-A7-A0DB	TCGA-A8-A0A2	TCGA-BH-A0HU	TCGA-A8-A076
TCGA-AO-A129	TCGA-AO-A0J8	TCGA-A8-A0A4	TCGA-A7-A0CJ	TCGA-A8-A07B
TCGA-AO-A12F	TCGA-BH-A0GZ	TCGA-A8-A0A6	TCGA-BH-A0H0	TCGA-AR-A0TX
TCGA-AQ-A04J	TCGA-AO-A0JA	TCGA-A8-A0AD	TCGA-BH-A0BD	TCGA-BH-A0B7
TCGA-AR-A0TS	TCGA-AO-A0JF	TCGA-AN-A03X	TCGA-A2-A0T3	TCGA-E2-A14V
TCGA-AR-A0TU	TCGA-A7-A0CD	TCGA-AN-A046	TCGA-A2-A0T4	**TCGA-C8-A12T**
TCGA-AR-A0U0	TCGA-D8-A145	TCGA-AN-A04A	TCGA-A2-A0YH	
TCGA-AR-A0U1	TCGA-BH-A0DK	TCGA-AN-A0FN	TCGA-A2-A0D4	
TCGA-AR-A1AI	TCGA-BH-A0E2	TCGA-AN-A0XL	TCGA-BH-A0AY	
TCGA-AR-A1AQ	TCGA-A2-A0YI	TCGA-AN-A0XP	TCGA-E2-A10C	
TCGA-AR-A1AR	TCGA-A2-A0YL	TCGA-AN-A0XV	TCGA-E2-A10A	
TCGA-AR-A1AY	TCGA-AO-A0JG	TCGA-AR-A0TW	TCGA-AO-A0JC	
TCGA-AR-A256	TCGA-BH-A0DP	TCGA-AR-A1AK	TCGA-AO-A0JD	
TCGA-B6-A0IE	TCGA-BH-A0E1	TCGA-AR-A1AL	TCGA-A2-A0CW	
TCGA-B6-A0IK	TCGA-A2-A0T5	TCGA-AR-A1AN	TCGA-A2-A0ER	
TCGA-B6-A0IQ	TCGA-A2-A0T6	TCGA-AR-A1AP	TCGA-AO-A12B	
TCGA-B6-A0RE	TCGA-BH-A0HI	TCGA-AR-A1AS	TCGA-A2-A04R	
TCGA-B6-A0RG	TCGA-A2-A0T7	TCGA-AR-A1AU	TCGA-B6-A0IM	
TCGA-B6-A0RN	TCGA-BH-A0BJ	TCGA-AR-A1AW	TCGA-AO-A03P	
TCGA-B6-A0RS	TCGA-BH-A0H7	TCGA-BH-A0AZ	TCGA-A8-A06O	
TCGA-B6-A0RT	TCGA-BH-A0HF	TCGA-BH-A0B0	TCGA-A8-A06Q	
TCGA-B6-A0RU	TCGA-BH-A0EB	TCGA-BH-A0BO	TCGA-A8-A06Z	
TCGA-B6-A0WX	TCGA-BH-A0H6	TCGA-BH-A0BP	TCGA-A8-A079	
TCGA-BH-A0AV	TCGA-A2-A0YD	TCGA-BH-A0BQ	TCGA-A8-A07L	
TCGA-BH-A0B3	TCGA-BH-A0HB	TCGA-BH-A0BR	TCGA-A8-A07W	
TCGA-BH-A0B9	TCGA-BH-A0HX	TCGA-BH-A0BS	TCGA-A8-A07Z	
TCGA-BH-A0BG	TCGA-AO-A12H	TCGA-BH-A0BT	TCGA-A8-A082	

TCGA-BH-A0BL	TCGA-E2-A10E	TCGA-BH-A0C1	TCGA-A8-A085	
TCGA-BH-A0BW	TCGA-A2-A0D3	TCGA-BH-A0DE	TCGA-A8-A08F	
TCGA-BH-A0E0	TCGA-E2-A10F	TCGA-BH-A0DI	TCGA-A8-A08I	
TCGA-BH-A0E6	TCGA-AO-A03V	TCGA-BH-A0DO	TCGA-A8-A095	
TCGA-BH-A0RX	TCGA-A2-A0EW	TCGA-BH-A0DT	TCGA-A8-A096	
TCGA-BH-A18G	TCGA-BH-A0GY	TCGA-BH-A0DX	TCGA-A8-A09C	
TCGA-BH-A18Q	TCGA-A2-A0EV	TCGA-BH-A0E9	TCGA-A8-A09D	
**TCGA-A1-A0SO**	TCGA-BH-A0BC	TCGA-BH-A0EI	TCGA-A8-A09M	
**TCGA-A2-A0D2**	TCGA-A2-A0YC	TCGA-BH-A0H3	TCGA-A8-A09Q	
**TCGA-AN-A0AL**	TCGA-A2-A0EU	TCGA-BH-A0H5	TCGA-A8-A09R	
**TCGA-AN-A0FL**	TCGA-A2-A0ET	TCGA-BH-A0HA	TCGA-A8-A09W	
**TCGA-AN-A0FX**	TCGA-A2-A04Y	TCGA-BH-A0W4	TCGA-A8-A0A9	
**TCGA-AN-A0XU**	TCGA-BH-A0HQ	TCGA-BH-A0W5	TCGA-A8-A0AB	
**TCGA-AO-A124**	TCGA-A2-A0ES	TCGA-BH-A0W7	TCGA-AN-A03Y	
**TCGA-AR-A0U4**	TCGA-BH-A0BA	TCGA-BH-A18H	TCGA-AN-A049	
**TCGA-BH-A0WA**	TCGA-E2-A10B	TCGA-BH-A18I	TCGA-AN-A0FF	
**TCGA-B6-A0X1**	TCGA-BH-A0B1	TCGA-C8-A12N	TCGA-AN-A0FK	
	TCGA-BH-A0DH	TCGA-C8-A133	TCGA-AN-A0FY	
	TCGA-BH-A0H9	TCGA-C8-A1HI	TCGA-AN-A0XW	
	TCGA-AO-A0J9	TCGA-D8-A141	TCGA-AR-A0TV	
	TCGA-AO-A12G	TCGA-E2-A14Q	TCGA-AR-A0TZ	
	TCGA-BH-A0E7	TCGA-E2-A14T	TCGA-AR-A0U3	
	TCGA-AO-A03M	TCGA-E2-A14Z	TCGA-AR-A1AV	
	TCGA-BH-A0BV	TCGA-E2-A153	TCGA-BH-A0AU	
	TCGA-BH-A0B8	TCGA-E2-A154	TCGA-BH-A0B5	
	TCGA-A2-A0CZ	TCGA-E2-A156	TCGA-BH-A0BF	
	TCGA-A2-A0SU	TCGA-E2-A15C	TCGA-BH-A0BZ	
	TCGA-AO-A12E	TCGA-E2-A15D	TCGA-BH-A0W3	
	TCGA-E2-A106	TCGA-E2-A15F	TCGA-BH-A18F	
	TCGA-A2-A0CV	TCGA-E2-A15G	TCGA-C8-A12U	
	TCGA-A2-A0CS	TCGA-E2-A15I	TCGA-C8-A12W	
	TCGA-A2-A0EO	TCGA-E2-A15J	TCGA-C8-A12X	
	TCGA-A2-A0CQ	TCGA-E2-A15O	TCGA-C8-A1HG	
	TCGA-A2-A0EN	TCGA-E2-A15P	TCGA-C8-A1HM	
	TCGA-AO-A12A	TCGA-E2-A15R	TCGA-E2-A14O	
	TCGA-A2-A0CP	TCGA-E2-A1B4	TCGA-E2-A14S	
	TCGA-AO-A126	TCGA-E2-A1BC	TCGA-E2-A15A	
	TCGA-AO-A125	TCGA-E2-A1BD	TCGA-E2-A15K	
	TCGA-A2-A0EM	**TCGA-BH-A0DS**	TCGA-E2-A15L	
	TCGA-A2-A04N	**TCGA-A1-A0SH**	TCGA-E2-A15M	
	TCGA-B6-A0IP	**TCGA-E2-A14Z**	TCGA-E2-A15T	

Table S6 : Relative gene expression in mutated and non mutated TNBC (Taqman Low Density Arrays).

	Mean ± SEM of non mutated N=13	Mean ± SEM of mutated N=15	Difference between means	P value	P value summary
**ARHGDIA**	0.5568 ± 0.02916	0.8428 ± 0.1085	-0.2861 ± 0.1200	0.0247	*
**AZI1**	0.07750 ± 0.01082	0.1292 ± 0.01769	-0.05170 ± 0.02154	0.0238	*
**CSNK1D-**	0.4446 ± 0.03376	0.7431 ± 0.09332	-0.2985 ± 0.1053	0.0087	**
**C17orf56**	0.04343 ± 0.005308	0.08547 ± 0.008940	-0.04205 ± 0.01082	0.0006	***
**C17orf62**	0.03805 ± 0.004920	0.06067 ± 0.006161	-0.02262 ± 0.008055	0.0093	**
**DUS1L**	0.05891 ± 0.006205	0.1036 ± 0.008620	-0.04467 ± 0.01092	0.0004	***
**FLJ90757**	0.02143 ± 0.003124	0.03819 ± 0.003768	-0.01675 ± 0.004987	0.0024	**
**FN3KRP**	0.04102 ± 0.006002	0.08371 ± 0.01178	-0.04269 ± 0.01386	0.0048	**
**GPS1**	0.1413 ± 0.02175	0.2353 ± 0.02842	-0.09400 ± 0.03666	0.0165	*
**HGS**	0.03007 ± 0.004927	0.05330 ± 0.005143	-0.02324 ± 0.007182	0.0033	**
**NPLOC4**	0.1346 ± 0.01224	0.2356 ± 0.02003	-0.1010 ± 0.02438	0.0003	***
**RFNG**	0.08350 ± 0.007018	0.1306 ± 0.009284	-0.04713 ± 0.01193	0.0005	***
**RPTOR**	0.05078 ± 0.004213	0.07140 ± 0.005053	-0.02062 ± 0.006701	0.0049	**
**SIRT7**	0.04444 ± 0.007663	0.07391 ± 0.01073	-0.02947 ± 0.01357	0.0392	*
**SLC25A10**	0.04048 ± 0.005765	0.07594 ± 0.01098	-0.03546 ± 0.01298	0.0111	*
**SLC38A10**	0.1918 ± 0.02096 N	0.3440 ± 0.03219	-0.1523 ± 0.03956	0.0006	***
**THOC4**	0.4631 ± 0.09149	0.8904 ± 0.1241	-0.4274 ± 0.1583	0.012	*

Table S7: Relative gene expression in mutated and non mutated TNBC (Taqman qPCR).

	**Mean ± SEM of non mutated N=13**	**Mean ± SEM of mutated N=15**	**Difference between means**	**P value**	**P value Summary**
**CSNK1D**	0.4250 ± 0.03142	0.6500 ± 0.04857	0.2250 ± 0.05785	**0.0006**	***
**RFNG**	0.1386 ± 0.01086	0.2127 ± 0.01733	0.07409 ± 0.02045	**0.0011**	**
**C17orf56**	0.1160 ± 0.01065	0.1729 ± 0.01583	0.05690 ± 0.01908	**0.0059**	**
**DUS1L**	0.3144 ± 0.03354	0.5067 ± 0.05855	0.1923 ± 0.06748	**0.0081**	**
**SLC25A10**	0.09083 ± 0.01059	0.1822 ± 0.02978	0.09133 ± 0.03161	**0.0074**	**
**HGS**	0.2615 ± 0.02847	0.3944 ± 0.04137	0.1329 ± 0.05022	**0.0132**	*
**FN3KRP**	0.06314 ± 0.008737	0.1130 ± 0.01853	0.04990 ± 0.02049	**0.0215**	*
**SIRT7**	0.08396 ± 0.007473	0.1349 ± 0.02050	0.05094 ± 0.02182	**0.027**	*

Table S8: Identification in the COLT-Cancer database (http://dpsc.ccbr.utoronto.ca/cancer/) of genes of the 17q25.3 region essential for survival of human cancer cell lines (29 breast, 15 ovarian, 6 colon and 28 pancreatic cell lines). The number and percentage of cell lines in which the gene is essential are indicated.

	**Pancreatic**	**Ovarian**	**Breast**	**Colon**
AZI1	14 (50%)	8 (53%)	7 (24%)	3 (50%)
FN3KRP	11 (39%)	3 (20%)	10 (34%)	3 (50%)
**HGS**	5 (17%)	3 (20%)	11 (37%)	
RPTOR	20 (71%)	6 (40%)	25 (86%)	2 (33%)
CSNK1D			1 (3%)	
DUS1L	2 (7%)		1 (3%)	
GPS1	8 (28%)	4 (26%)	7 (24%)	1 (16%)
RFNG			1 (3%)	
THOC4	2 7(96%)	14 (93%)	29 (100%)	6 (100%)

		ARHGDIA	*AZI1*	*C17orf56*	*C17orf62*	*CSNK1D*	*DUS1L*	*FLJ90757*	*FN3KRP*	*GPS1*	HGS	*NPLOC4*	*RFNG*	*RPTOR*	*SIRT7*	*SCL25A10*	*SCL38A10*	*THOC4*
Increased gamma-H2AX phosphorylation and DNA content	DNA damage response		X							X								
Decreased homologous recombination repair frequency										X				X				X
Increased ionizing radiation sensitivity		X																
Synthetic lethal with gemcitabine						X												
Sensitivity to cisplatin		X																
Decreased nuclei in G1, small nuclei in G1	Cell cycle regulation		X						X						X			
Cell division defect					X													
Increased cell death	Cell viability	X									X							
Decreased viability					X		X			X	X			X	X			X
Synthetic lethal interaction with Ras					X													
Decreased cell migration	Migration					X												

Table S9: Phenotypes resulting from the inactivation of genes of the 17q25.3 region in publically available shRNA screens (obtained from the GenomeRNAi database: http://www.genomernai.org).

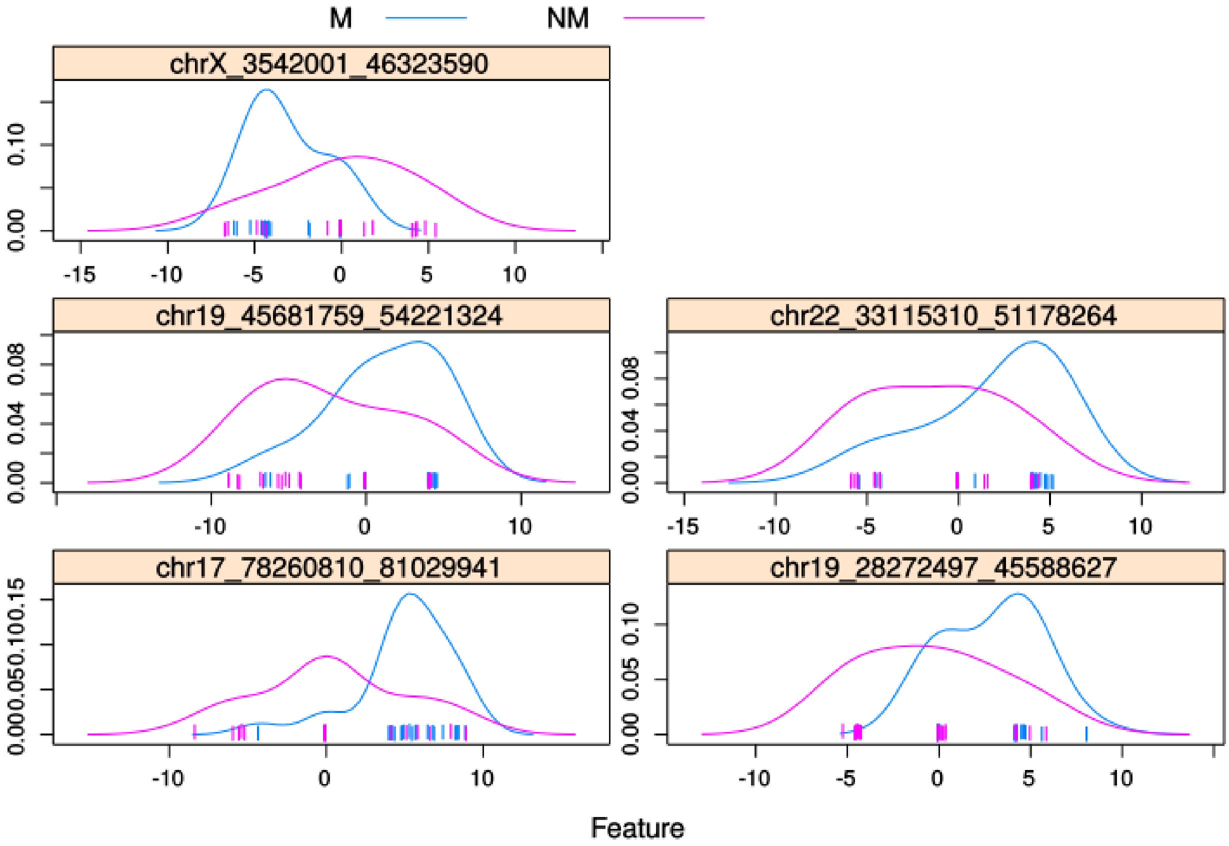

Figure S1: Density plots of the 5 most important variables ((to be read as such: chr.number_start.base_stop.base. 'M' = mutated. 'NM' = non mutated).

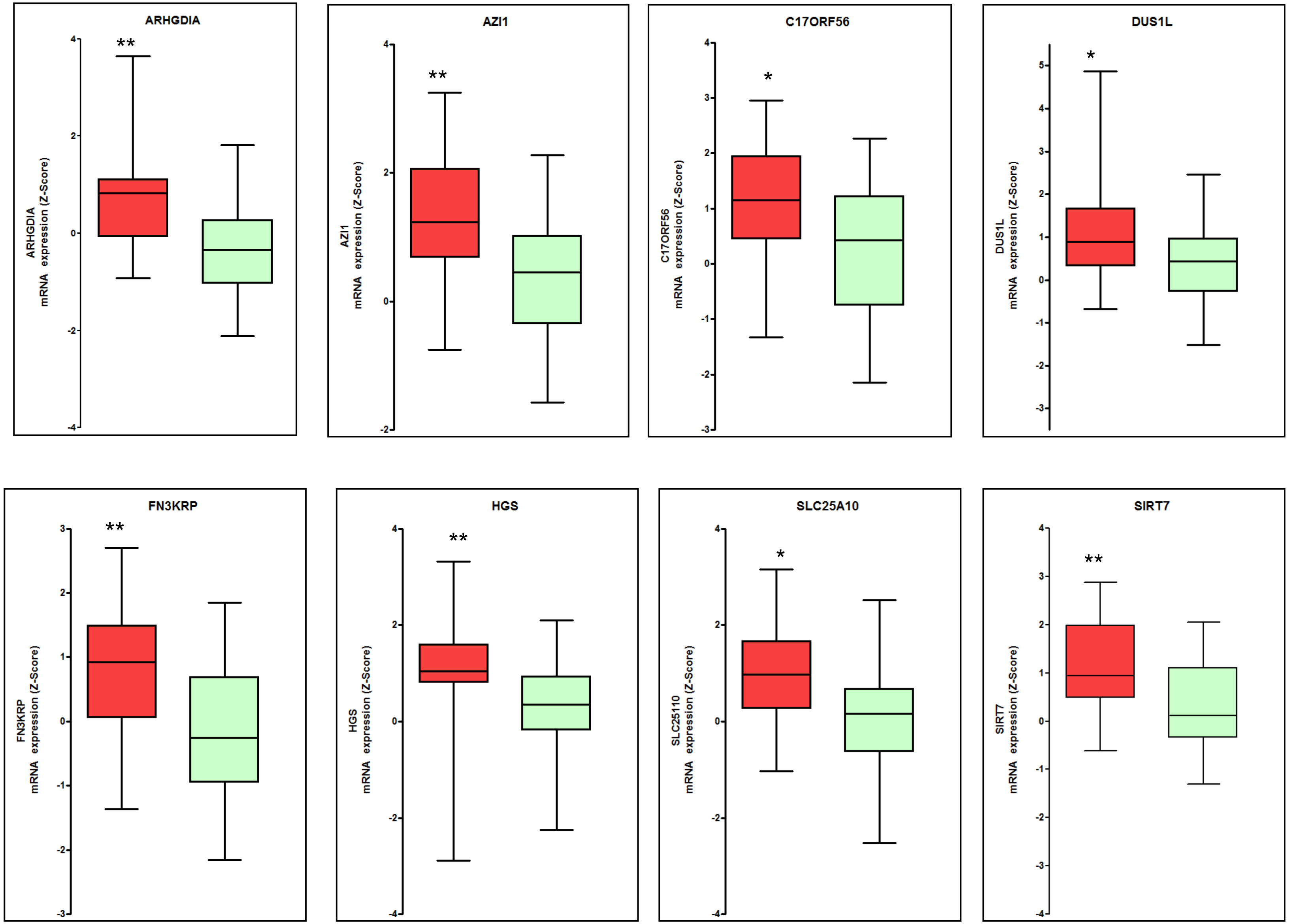

Figure S2: Z-score (retrieved from cBioportal Cancer) for 26 TNBC with a gain in 17q25.3 (red box) and 30 TNBC without a gain in 17q25.3 (green box) (whiskers : minimum and maximum. bar : median). Statistical analysis: Mann-Whitney-Wilcoxon test: p<0.05:*. p<0.01:**. p<0.001:***.

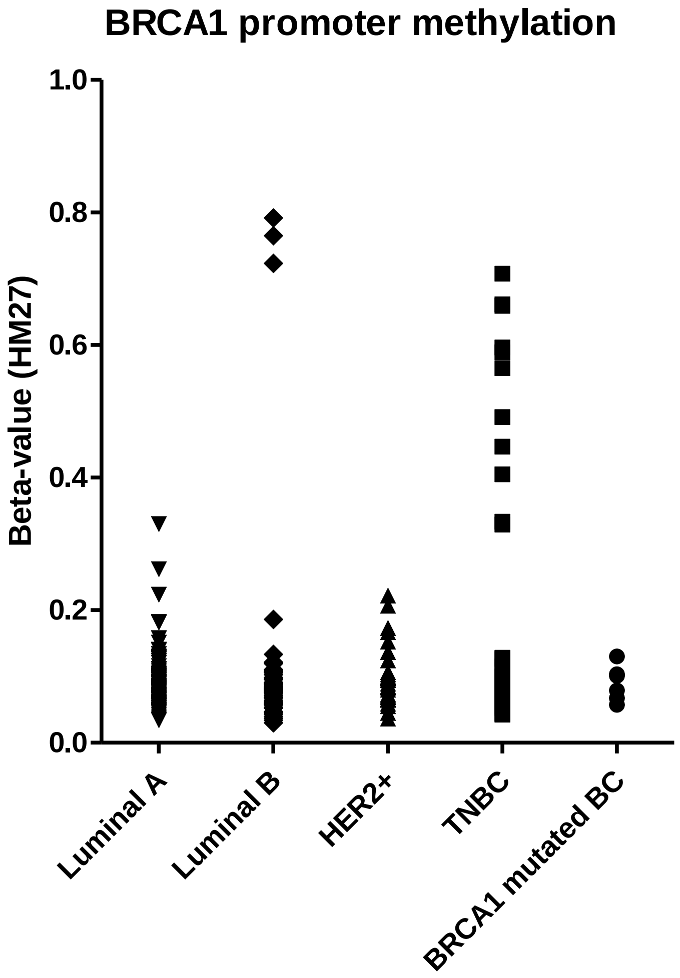

Figure S3: Analysis of the *BRCA1* promoter methylation in the TCGA study. DNA methylation data are presented as beta values, with 0 indicating 0 % DNA methylation and beta values of 1 indicating 100 % DNA methylation.
