# Supplementary figures and images for: Identification by array comparative genomic hybridization of a new amplicon on chromosome 17q highly recurrent in BRCA1 mutated triple negative breast cancer
Source: Breast Cancer Res. 2014 Nov 22;16:466. doi: 10.1186/s13058-014-0466-y (PMC4303204; doi:10.1186/s13058-014-0466-y)

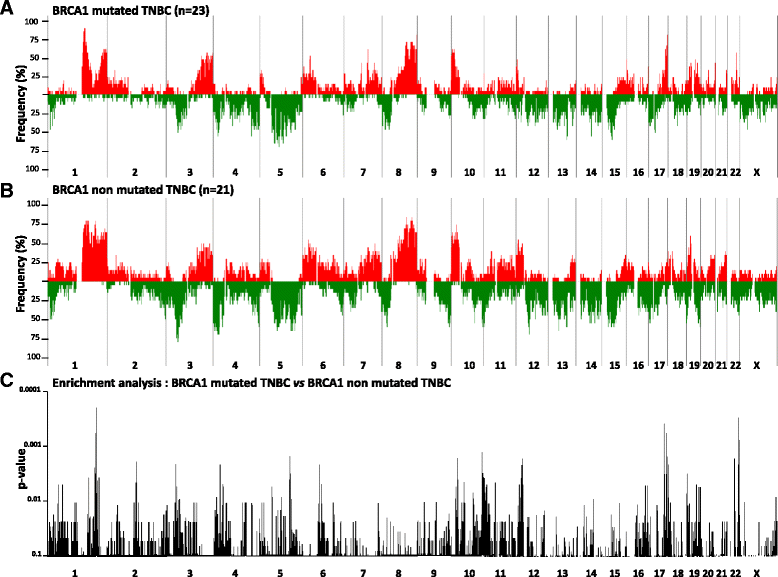

Supplement: Supplementary file 5 — Authors’ original file for figure 1 [file 13058_2014_466_MOESM5_ESM.gif]

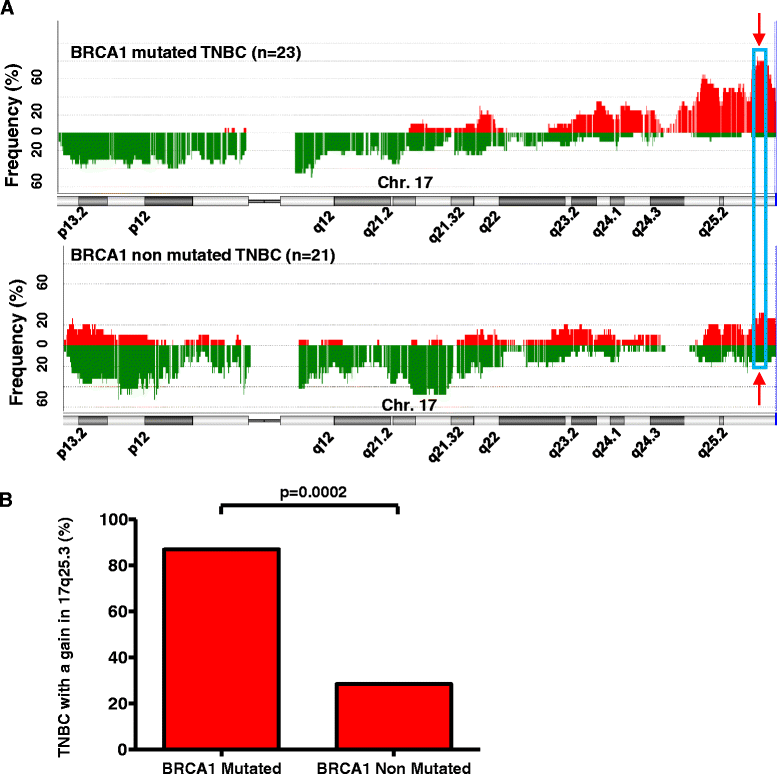

Supplement: Supplementary file 6 — Authors’ original file for figure 2 [file 13058_2014_466_MOESM6_ESM.gif]

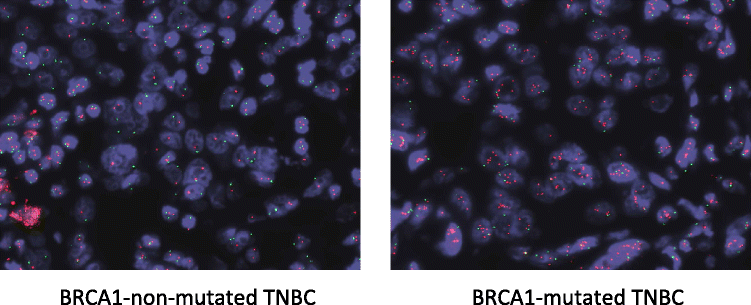

Supplement: Supplementary file 7 — Authors’ original file for figure 3 [file 13058_2014_466_MOESM7_ESM.gif]

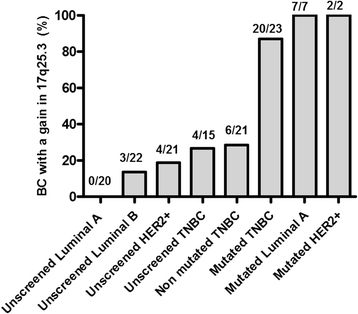

Supplement: Supplementary file 8 — Authors’ original file for figure 4 [file 13058_2014_466_MOESM8_ESM.gif]

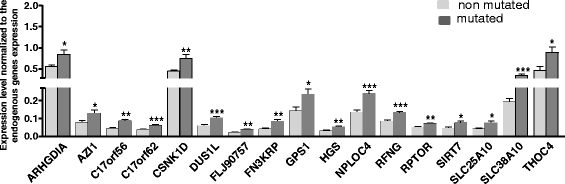

Supplement: Supplementary file 9 — Authors’ original file for figure 5 [file 13058_2014_466_MOESM9_ESM.gif]

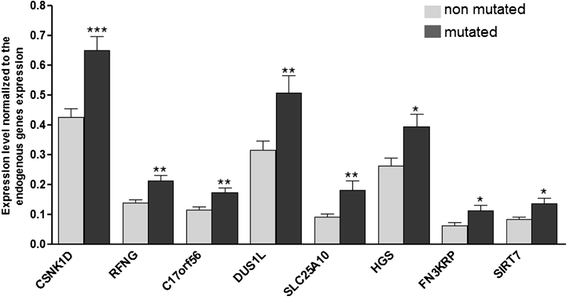

Supplement: Supplementary file 10 — Authors’ original file for figure 6 [file 13058_2014_466_MOESM10_ESM.gif]

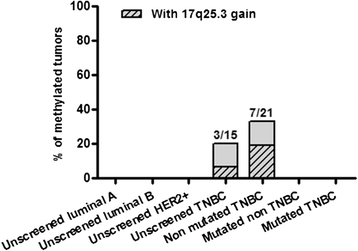

Supplement: Supplementary file 11 — Authors’ original file for figure 7 [file 13058_2014_466_MOESM11_ESM.gif]
